# Accurate identification of circRNA landscape and complexity reveals their pivotal roles in human oligodendroglia differentiation

**DOI:** 10.1186/s13059-022-02621-1

**Published:** 2022-02-07

**Authors:** Yangping Li, Feng Wang, Peng Teng, Li Ku, Li Chen, Yue Feng, Bing Yao

**Affiliations:** 1 Department of Human Genetics, Emory University School of Medicine, Atlanta, GA 30322 USA; 2 Department of Pharmacology and Chemical Biology, Emory University School of Medicine, Atlanta, GA 30322 USA; 3 Department of Biostatistics and Health Data Science, Center for Computational Biology and Bioinformatics, Indiana University School of Medicine, Indianapolis, IN 46202 USA

**Keywords:** CircRNAs, MicroRNAs, Oligodendroglia, OL Differentiation

## Abstract

**Background:**

Circular RNAs (circRNAs), a novel class of poorly conserved non-coding RNAs that regulate gene expression, are highly enriched in the human brain. Despite increasing discoveries of circRNA function in human neurons, the circRNA landscape and function in developing human oligodendroglia, the myelinating cells that govern neuronal conductance, remains unexplored. Meanwhile, improved experimental and computational tools for the accurate identification of circRNAs are needed.

**Results:**

We adopt a published experimental approach for circRNA enrichment and develop CARP (CircRNA identification using A-tailing RNase R approach and Pseudo-reference alignment), a comprehensive 21-module computational framework for accurate circRNA identification and quantification. Using CARP, we identify developmentally programmed human oligodendroglia circRNA landscapes in the HOG oligodendroglioma cell line, distinct from neuronal circRNA landscapes. Numerous circRNAs display oligodendroglia-specific regulation upon differentiation, among which a subclass is regulated independently from their parental mRNAs. We find that circRNA flanking introns often contain *cis*-regulatory elements for RNA editing and are predicted to bind differentiation-regulated splicing factors. In addition, we discover novel oligodendroglia-specific circRNAs that are predicted to sponge microRNAs, which co-operatively promote oligodendroglia development. Furthermore, we identify circRNA clusters derived from differentiation-regulated alternative circularization events within the same gene, each containing a common circular exon, achieving additive sponging effects that promote human oligodendroglia differentiation.

**Conclusions:**

Our results reveal dynamic regulation of human oligodendroglia circRNA landscapes during early differentiation and suggest critical roles of the circRNA-miRNA-mRNA axis in advancing human oligodendroglia development.

**Supplementary Information:**

The online version contains supplementary material available at 10.1186/s13059-022-02621-1.

## Background

Circular RNAs (circRNAs) are a large class of single-stranded, stable, functional RNAs in mammalian cells that have a closed-loop structure [1–5]. CircRNAs are derived from the covalent joining of a downstream 5′ splice donor to an upstream 3′ splice acceptor via a previously underappreciated pre-mRNA splicing mechanism, known as “back-splicing.” Compelling evidence shows that circRNAs play sophisticated biological roles, including regulation of pre-mRNA splicing, miRNA sponging, RNA binding protein (RBP) sequestration, and IRES-mediated cap-independent translation to produce short peptides [6–9]. The molecular mechanisms underlying circRNA biogenesis are attributed to *cis*-regulatory elements, such as the repetitive *Alu* sequence and *trans*-acting RBPs that flank the circular exon in the pre-mRNA. Both mechanisms could help to bring back splicing junction (BSJ) sites into proximity for efficient splicing [10–12]. Recent studies also indicated that RNA adenosine to inosine (A-to-I) editing is located within the *Alu* sequence, interfering with inverted and repeated *Alu* pairs to influence circRNA biogenesis [13, 14].

While circRNAs are widespread in metazoans with generally low levels of expression, many circRNAs are highly enriched in specific tissues, such as the brain, and exhibit cell type-specific expression and function [14]. Specifically, abundant circRNAs are expressed in brain neurons and dynamically regulated during differentiation [14, 15]. Of note, numerous circRNAs are specifically expressed in the human brain [16]. Despite the well-documented neuronal and synaptic circRNAs that display abnormalities in brain diseases [17, 18], a comprehensive and precise understanding of the circRNA landscape and its downstream biological pathways in the human brain is still missing. Moreover, circRNAs were recently found in glia cells isolated from the post-mortem adult brains [19], including oligodendroglia (OL) that are responsible for myelinating neuronal axons to achieve rapid information flow in the brain [20]. Furthermore, alternative splicing is extensive in OLs that govern myelin development [21], and OL defects underlie neurodevelopmental and neurological diseases represented by schizophrenia and multiple sclerosis [22–24]. Therefore, the regulation and function of circRNAs in human OL development warrant rigorous investigation. Nonetheless, due to the difficulty in obtaining human OLs in culture, understanding circRNA biology in human OL development is a prevailing challenge.

Accurate and precise identification of the circRNA landscape relied on RNA-seq data but faced technical challenges. Reads spanning the circRNA specific BSJ sites are the only means for distinguishing circRNAs from their parental transcripts [10, 15, 25–28]. Because BSJ reads only account for a small portion of RNA-seq reads and do not map to the genomic index, the identification and quantification of circRNAs suffer from low power and sensitivity and are thus prone to high false discovery rates [4, 29]. RNase R treatments have been widely used to degrade linear RNA for circRNA enrichment to identify circRNAs of low abundance [11, 30, 31]. However, some transcripts were resistant to RNase R, owing to their lack of single-strand 3′ overhang or possession of secondary structures such as the G-quadruplex (G4) [32]. One recent study employed the addition of poly-A tails in vitro followed by RNase R treatment in optimized buffer conditions by replacing K^+^ to Li^+^ (refer to A-tailing approach hereafter) and achieved the best linear transcripts removal in an experimental setting to date [32].

On the other hand, the comparison of BSJ reads-based computational methods resulted in only a modest overlapping, again suggesting a high false discovery rate of circRNA identification by solely relying on BSJ reads [29]. Although constructing a circRNA pseudo-reference for re-aligning RNA-seq reads achieved more accurate and sensitive circRNA identification [33], pseudo-reference mapping requires a more thorough removal of reads from linear transcripts to reduce the false-positive rate [33]. Furthermore, because circRNAs that harbor the same BSJ can contain multiple exons and even retained introns, BSJ reads-based methods cannot parse circRNA full-length information and internal structural variations, which is critical for circRNA function [34]. One in silico approach used split alignments of the reads pair with BSJ reads to reconstruct circRNA full-length from RNA-seq data despite the challenge for longer circRNAs due to the nature of short reads length from Illumina sequencing [35]. Recently, Nanopore long-read sequencing was performed to find circRNA full-length and alternative splicing events within the circRNA body but was restricted by low read depth [36, 37]. Also, alternative circularization can generate multiple circRNAs within a single gene that share the same BSJ site, showing the complicated diversity of circRNA biology [10, 38]. These “clustered” circRNAs share partial common sequences and may function additively in sponging miRNAs or RBPs, yet their potential coordinating roles are often overlooked. A multi-functional computational framework optimized for A-tailing datasets is needed to identify and quantify circRNAs accurately and cost-effectively.

We developed CARP (CircRNA identification using A-tailing RNase R approach and Pseudo-reference alignment), a comprehensive computational framework for circRNA identification and quantification using A-tailing RNase R RNA-seq data. Using CARP, we systematically interrogated circRNA landscape in an human OL cell line called HOG and identified circRNA dynamic regulation specifically during human OL differentiation. Some circRNAs appeared to be regulated independently of their parental mRNAs during differentiation possibly through flanking intron-associated RBPs or adenosine-to-inosine (A-to-I) RNA editing within *Alu* repetitive elements. Multiple circRNAs regulated upon HOG differentiation could potentially advance OL differentiation via influencing miRNA activities and downstream gene expression.

## Results

### Effective and accurate circRNA identification and quantification by CARP

**Fig. 1:**
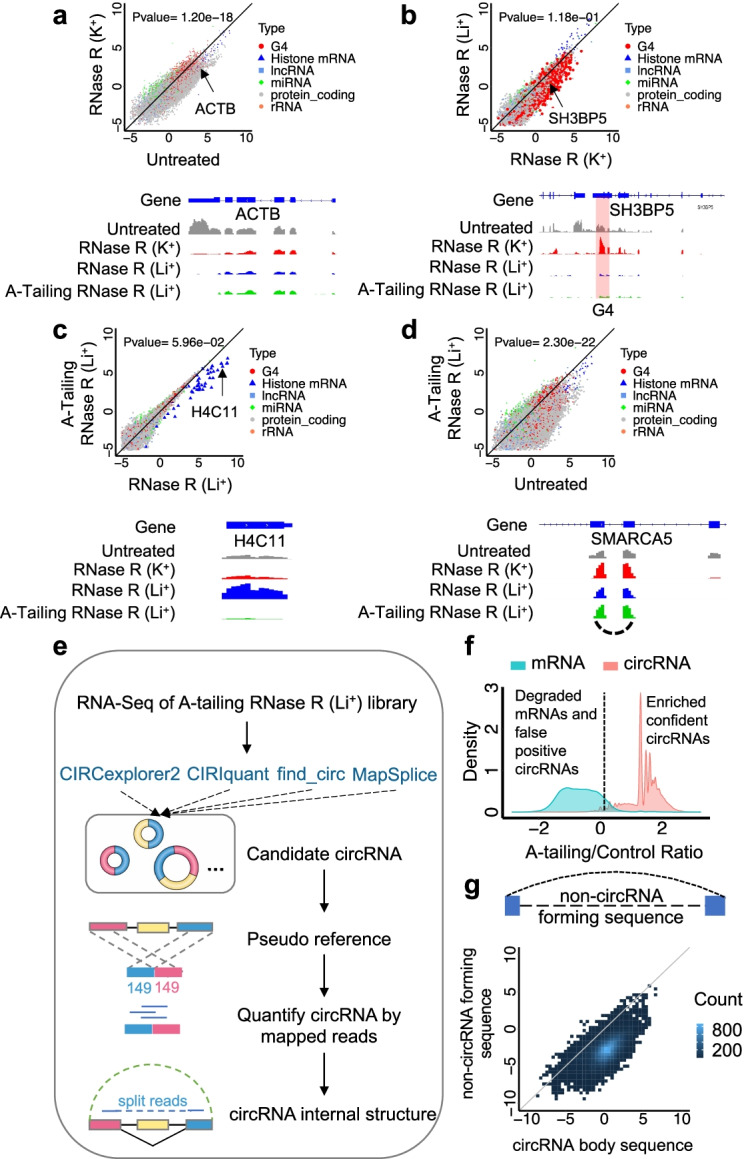
CARP effectively and accurately identifies full-length circRNAs from A-tailing data. **a** Substantial linear mRNAs were degraded by RNase R treatment in K^+^ buffer in HEK293T cells. **b** mRNAs with G-quadruplex (G4) structures were further degraded by RNase R treatment in Li^+^ buffer in HEK293T cell. **c** Linear RNAs with short poly-A tail were resistant to RNase R treatment but could be degraded after adding Poly-A tail in HEK293T cell. **d** A-tailing approach achieved the best linear RNA removal (scatter plot) without affecting circRNA stability (IGV view) in HEK293T cells. **e** Workflow of CARP to identify confident, full-length circRNAs from A-tailing data. **f** Density plot showed confident circRNAs identification by removing false-positive circRNAs sensitive to A-tailing and RNase R treatment in HOG cells. The ratio of RNA levels between the A-tailing treatment and the control was calculated and shown on the *x*-axis. The cutoff that defined resistant vs. sensitive upon A-tailing treatment is shown with the dashed line with an FDR < 0.05. **g** Most of the mapped reads in the A-tailing library were located to a predicted circRNA body sequence other than the non-circRNA forming sequence in HOG cells. Student’s *t*-test (two-tailed and unpaired) was used for gene expression comparison between different libraries

In order to enrich circRNAs in RNA-seq data, we first adopted a recently published method with the addition of poly-A tails in vitro followed by RNase R treatment in Li^+^ buffer (A-tailing approach hereafter) to remove linear RNAs [32]. Total RNA extracted from HEK293T and SH-SY5Y cells was used to test efficiency. The majority of linear mRNAs were degraded in RNase R treatment with traditional K^+^ buffer (Fig. 1a; Additional file 1: Figure S1A). Also, linear RNAs that harbor G4 structures thus are RNase R-resistant in K^+^ buffer were efficiently degraded when switching to Li^+^ buffer (Fig. 1b; Additional file 1: Fig. S1B). Moreover, RNAs that lack 3′ poly-A tails but harbor unique 3′ end structures, such as histone mRNAs, could only be degraded by combined A-tailing and RNase R treatments (Fig. 1c; Additional file 1: Fig. S1C). In contrast, an example circRNA, circSMARCA5 (hsa_circ_0001445) [39], was not affected by A-tailing approach (Fig. 1d; Additional file 1: Fig. S1D). Taken together, A-tailing coupled with RNase R in Li^+^ buffer improved the efficiency for removal of linear RNAs from total RNA samples without affecting circRNA stability (*t*-test, *p*-value = 2.3 × 10^−22^) (Fig. 1d; Additional file 1: Fig. S1D).

We then applied A-tailing to explore the dynamic regulation of human OL circRNA landscapes using the human oligodendroglioma cell line HOG. The human neuroblastoma cell line BE(2)-M17 (referred to as M17 hereafter) was used in parallel experiments for cell-specificity comparisons. Both HOG and M17 cells can be induced to undergo robust differentiation that recapitulates the early morphologic characteristics in OL and neuron development (Additional file 1: Fig. S2A) [40, 41]. In addition, QKI5, an RNA-binding protein enriched in human OLs over neurons (Additional file 1: Fig. S2B) [42], was abundantly expressed in HOG cells but only negligibly expressed in M17 cells (Additional file 1: Fig. S2C). Moreover, global transcriptomic principal component analysis (PCA) demonstrated a close correlation between HOG cells and iPSC-derived OLs (Additional file 1: Fig. S2D-E) [43, 44], further supporting HOG cells as an in vitro model for human OL that provides sufficient materials for reliable detection of low abundance circRNAs.

In addition, to improve computational methods for reliable identification of circRNAs, we developed a computational framework, CARP (CircRNA identification using A-tailing RNase R approach and Pseudo-reference alignment), designed to handle A-tailing datasets. Four well-established algorithms, including CIRCexplorer2, CIRIquant, find_circ, and MapSplice, were first applied for independent putative circRNA identification. However, only a subset of circRNAs was shared by the outputs of these methods (Additional file 1: Fig. S3A), indicating a relative low power using BSJ reads alone and potentially false-positive circRNAs identified by a single software program [29]. Therefore, all circRNAs identified by any one of the four BSJ-based algorithms were pooled, and a pseudo-reference for each candidate circRNA was constructed using sequence ± 149 bp flanking the BSJ sites (Fig. 1e) to overcome these problems. Reads that are directly aligned against the pseudo-reference should be derived explicitly from circRNA BSJ sites.

The stringency of reads that map to the pseudo-reference, such as how many nucleotides surrounding BSJ sites should be included in the sequencing reads, plays critical roles in distinguishing reads from either circRNA or their linear parental genes. To achieve a possible low false discovery rate (FDR) without compromising the actual circRNA reads from A-tailing libraries, CARP can perform a series of optimizations to define the suitable stringency. In addition, CARP also re-aligned these circRNA reads to the genome and transcriptome to eliminate false-positive reads. We determined that reads with 8 bp exact reverse complementary with ± 4 bp flanking the BSJ flanking sequencing achieved FDR < 0.05. Under these criteria, most false-positive reads were removed, and the remaining were considered bona fide circRNA reads (Additional file 1: Fig. S3B).

We further validated and refined the results based on the direct comparison of the circRNA species identified by A-tailing samples to the untreated libraries. A linear reference was constructed using the sequence of the last exon to quantify the linear host transcript of each candidate circRNA because the last exons barely form circRNAs [2, 32]. By calculating the linear mRNA ratio between A-tailing and untreated libraries, CARP determined the RNA pool sensitive or resistant to A-tailing/RNase R treatment using FDR < 0.05 as a cutoff. Compared with 95% of linear RNA, only 4% of circRNAs identified by CARP were significantly sensitive to A-tailing/ RNase R treatment (Fig. 1f; Additional file 1: Fig. S3C). Furthermore, circRNAs that were sensitive to RNase R treatment were subsequently removed, resulting in a substantive true positive circRNA pool for downstream study. Taken together, our data suggest a much-improved circRNA identification both experimentally and computationally in this study.

Compared to untreated libraries, A-tailing allowed the identification of an additional 37,950 de novo circRNAs by CARP in HOG cells (Additional file 1: Fig. S3D). Notably, circRNAs identified by CARP in A-tailing libraries were highly correlated with circRNAs in untreated RNA-seq libraries (Pearson correlation, *R*^2^ = 0.99, *p*-value < 2.2 × 10^−16^), with the majority of circRNAs displaying enrichment after A-tailing (Additional file 1: Fig. S3E). Meanwhile, the circRNA expression levels quantified by CARP using pseudo-references displayed a high correlation with circRNA quantification by CIRCexplorer2 (Pearson correlation, *R*^2^=0.90) and CIRIquant (Pearson correlation, *R*^2^=0.95) using BSJ reads (Additional file 1: Fig. S3F, G). In addition, since A-tailing/RNase R degraded most linear transcripts, the mapped reads spanning discontinuous regions in pre-mRNA, referred to as “split reads,” can be used to determine full-length circRNA sequences (Fig. 1e). The split reads show exact regions in the circRNA host genes that could be included or excluded in circRNAs. CARP supplied accurate circRNA full-length annotation, evidenced by that reads from A-tailing data mapped to predicted circRNA body sequence instead of non-circRNA forming sequence (Fig. 1g). For example, by using split reads, a circRNA from GLRX3 was annotated to contain 3 exons (Additional file 1: Figure S3H, blue color) with one linear exon (Additional file 1: Fig. S3H, red color) excluded from circRNA, and a detailed inspection in IGV confirmed no A-tailing reads in the excluded exon on its host gene (Additional file 1: Fig. S3H, red color). The circGLRX3 full-length constructed by CARP was consistent with the recently published long-read sequencing data (Additional file 1: Fig. S3H, lower box) [36]. These data suggested that CARP demonstrated better sensitivity in circRNA identification with complete length information.

### Identification of human OL progenitor circRNA landscape by CARP

Using CARP, we identified an average of 38,561 confident circRNAs in HOG cells. A similar number of confident circRNA species were identified in M17 cells (Additional file 2). The majority were derived from annotated gene regions, including circRNA derived from exons and introns. Most exon-derived circRNAs bear multiple exons, while a few are from single exon and intron lariats in HOG cells (Additional file 1: Fig. S3I, Additional file 3). The lengths of most circRNAs ranged from 200 to 2000 nt (Additional file 1: Figure S4A). The median exon length of multi-exon circRNAs was comparable to the length of randomly chosen exons that did not form circRNAs, while the exon length of single exon circRNA was much longer (Additional file 1: Fig. S4B) [13]. Both upstream and downstream circRNA flanking introns were much longer than random introns in the human genome (Additional file 1: Fig. S4C). The numbers of exons in various circRNAs ranged from 1 to over 20 (Additional file 1: Figure S4D). Consistent with earlier reports, very few circRNAs contained the first exon or the last exon of the host transcripts due to the lack of splice donor or acceptor sequences to support back-splicing (Additional file 1: Fig. S4E). Using our recently developed algorithm, circMeta [45], we calculated the *Alu* score, which reflects the likelihood of circRNA formation by IR*Alus* formed within or across flanking introns [10]. The confident circRNAs identified by CARP showed a higher *Alu* score compared to false-positive circRNAs and randomly selected intron pairs (Additional file 1: Figure S4F), once again indicating the improvement in *bona fide* circRNA identification.

**Fig. 2:**
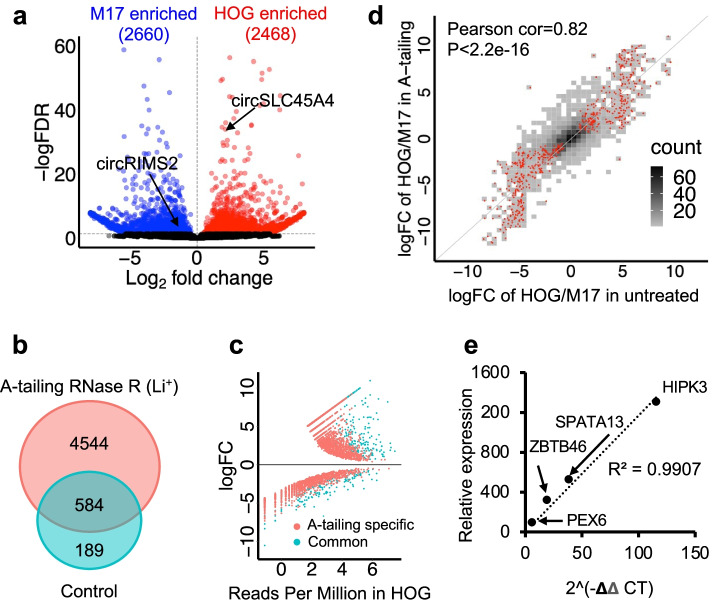
CARP identified a distinct circRNA landscape in M17 and HOG cells more efficiently using A-tailing data. **a** The volcano plot showed significant DE circRNAs in M17 and HOG cells using A-tailing data. Blue and red dots indicate significant M17 and HOG cell-enriched circRNAs (DESeq2, FDR < 0.05). **b** Overlap of DE circRNAs identified by CARP using A-tailing data and control data without A-tailing. **c** A scatter plot showing a log_2_ fold change of DE circRNAs in HOG and M17 cells and their expression (counts per million) in HOG cells. Red dots indicate DE circRNAs that were explicitly identified by A-tailing data. Cyan dots show DE circRNAs identified by both A-tailing data and control data. A-tailing libraries were sensitive in identifying circRNAs with relatively low expression levels (red dots). **d** The density plot showed a high correlation of log_2_ fold change for common circRNAs identified in the A-tailing library and the untreated library. Density colors show circRNA numbers in specific log_2_ fold changes. Red dots represent significantly DE circRNAs identified by A-tailing data (DESeq2, FDR < 0.05). **e** A scatter plot showed a high correlation of circRNA expression quantified by A-tailing data and qPCR for 4 randomly selected circRNAs with different expression levels

In order to identify the OL-specific circRNA landscape, A-tailing samples obtained from HOG and M17 cells were subjected to DE analyses by CARP using integrated DESeq2 [46], which revealed 2468 and 2660 DE circRNAs distinctly enriched in HOG and M17 cells, respectively (Fig. 2a). Among the DE circRNAs, circSLC45A4, a negative regulator of neuronal differentiation [47], was highly expressed in HOG cells. In contrast, the synaptoneurosomal circRIMS2 [14] was found enriched in M17 cells. Of note, only 346 and 427 significant DE circRNAs were identified in HOG and M17 cells using RNA-seq libraries without A-tailing treatment, further indicating improved sensitivity by A-tailing treatment (Additional file 1: Fig. S4G). With the improved sensitivity, CARP detected 6.63 times more de novo DE circRNAs between M17 and HOG cells than traditional untreated RNA-seq (Fig. 2b). The majority of the de novo DE circRNAs are of low abundance (Fig. 2c), demonstrating the ability of CARP in detecting low abundance circRNAs. Importantly, the log_2_ fold changes of each circRNA also showed a high correlation between A-tailing and untreated libraries (Pearson correlation, *R*^2^ = 0.82, *p*-value < 2.2 × 10^−16^), indicating that CARP is accurate for circRNA DE analysis (Fig. 2d). To further validate circRNA expression levels quantified by CARP, the expression of four DE circRNAs representing high, medium and low-level expression in HOG cells were evaluated by qPCR with divergent primers (Additional file 4), which showed a high correlation with our RNA-seq data (Pearson correlation, *R*^2^ = 0.99, Fig. 2e).

### CircRNA biogenesis and sequence composition were regulated upon HOG differentiation

HOG cells were induced to differentiate for 13 days before being subjected to A-tailing and CARP analysis in order to delineate whether and how human OL development regulates circRNA landscapes. In addition to morphological differentiation (Additional file 1: Fig. S2A II), the expression changes of a panel of oligodendroglia differentiation-related genes were evaluated by qPCR in cells that underwent differentiation (Additional file 1: Fig. S5A) [48–55]. M17 cells underwent differentiation for 10 days (Additional file 1: Fig. S2A IV, Figure S5B) were processed in parallel to achieve cell-specificity comparisons.

**Fig. 3:**
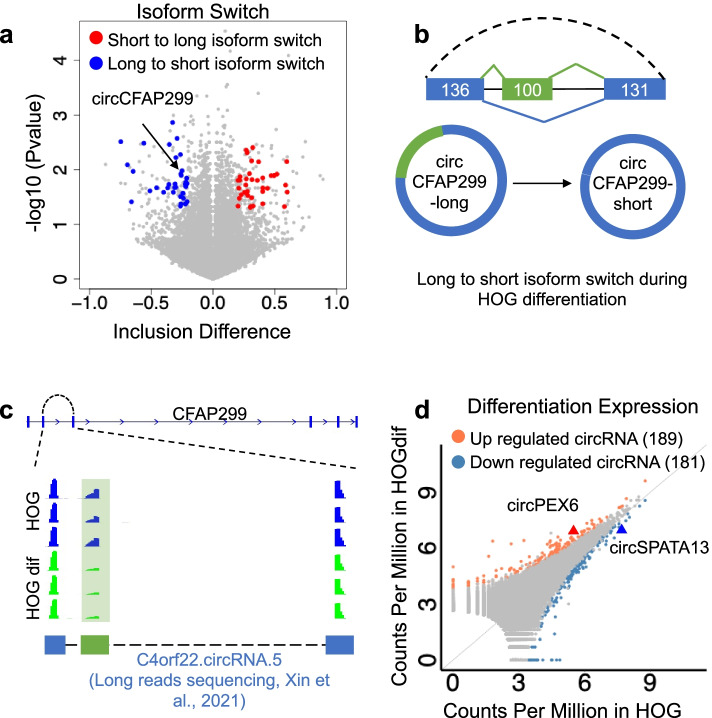
CircRNA internal structure and expression were regulated upon HOG differentiation. **a** CARP identified circRNA isoform switching events upon HOG differentiation. Blue and red dots represent short to long isoform switch and long to short isoform switch, respectively (Student’s *t*-test, two-tailed and unpaired, *P* < 0.05). The “inclusion difference” is the difference of “inclusion level” that is calculated based on junction reads count between undifferentiated and differentiated HOG cells. **b** A 100-bp exon (green) was excluded in circCFAP299 in differentiated HOG cells. **c** IGV view shows that the cassette exon of circCFAP299 was supported by mapped reads. The novel exon in circCFAP299 was also confirmed by a recent study using the Nanopore-based long reads sequencing method. **d** DE analysis of circRNAs in undifferentiated and differentiated HOG cells. Orange and blue dots show upregulated and downregulated circRNAs upon HOG differentiation (DESeq2, FDR < 0.05)

CARP identified 204 circRNA isoforms that carry different sequence compositions but share the same BSJ sites, which underwent significant isoform switching during HOG cell differentiation (*t*-test, *p*-value < 0.05 and inclusion level difference over 0.2, Fig. 3a). To annotate exons excluded or included in the 204 circRNAs isoforms, we compared the sequences in these circRNA isoforms with gene annotation downloaded from UCSC Table Browser and cassette exon information downloaded from HEXEvent [56]. We found that 17.89% of the alternative circular exons are from previously annotated cassette exons, 45.83% are derived from previously annotated constitutive exons, while 36.27% are from novel unannotated exons. For example, two isoforms of circCFAP299 exist due to the alternative inclusion of a 100-nt cassette exon (Fig. 3a, b). The expression level of the short isoform switched from 55% of total circCFAP299 to 76% upon HOG differentiation (Fig. 3c). During differentiation, the isoform switch could cause functional consequences as the unique exon in the long isoform may sponge miRNA and sequester RNA-binding proteins. The cassette exon was circRNA-specific, not annotated in any linear mRNAs produced by the CFAP299 host gene in UCSC genomic browser. The circCFAP299 full-length constructed by CARP, particularly the unique circRNA specific exon, was consistent with the long-read sequencing data (Fig. 3c, lower box) [36].

**Fig. 4:**
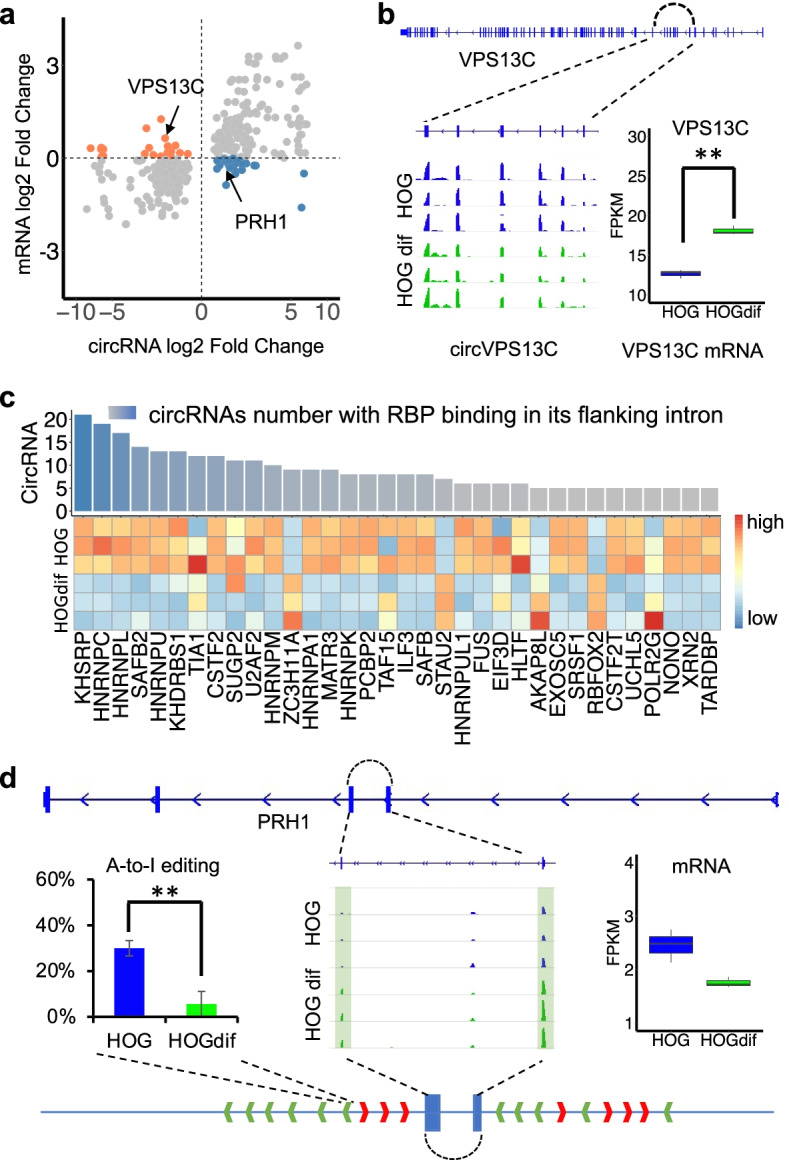
CircRNAs are regulated post-transcriptionally by RBPs and A-to-I editing in their flanking introns upon HOG differentiation. **a** Scatter plot shows the correlation between log_2_ fold change of circRNAs and their host gene expression change upon HOG differentiation. Grey dots are positive correlations between circRNAs and their host genes (group 1), while blue and orange dots stand for inversely correlated circRNAs with their host gene (group 2). **b** IGV view showed circVPS13C was downregulated upon HOG differentiation. Bar plot indicated *VPS13C* mRNA was upregulated upon HOG differentiation. **c** Bar plot indicated the number of circRNAs from group 2 in Fig. 4a that a given RNA-binding protein could bind to their flanking introns. Heatmap showed RPKM levels of those RBPs in parental and differentiated HOG cells. **d** IGV view showed circPRH1 expression was upregulated during HOG differentiation. CircPRH1 full-length sequences are in green. The left bar plot shows that the A-to-I editing in the *Alu* sequence of circPRH1 flanking introns was depleted upon HOG differentiation. The right bar plot shows that *PRH1* mRNA was downregulated upon HOG differentiation. Student’s *t*-test (two-tailed and unpaired) was used for A-to-I editing change. **P < 0.01

In addition to isoform switch, CARP also identified 189 upregulated circRNAs, and 181 downregulated circRNAs in differentiated HOG cells (Fig. 3d, FDR < 0.05 as cutoff) (Additional file 1: Fig. S6A). Few circRNAs were commonly regulated by differentiation of HOG and M17, suggesting cell type-specific roles and regulation of these circRNAs in OLs differentiation (Additional file 1: Fig. S6B). Most significant DE circRNAs during HOG differentiation are positively correlated with developmental regulation of their host genes (Fig. 4a, group 1 shown in grey dots). However, a subclass of differentiation-regulated circRNAs showed distinct changes than the linear RNAs derived from the host genes (Fig. 4a, group 2 shown in orange and blue dots). For example, the gene encoding the vacuolar protein sorting-associated protein 13C (VPS13C), which belongs to the GO term of protein retention in Golgi apparatus, was upregulated in differentiated HOG cells. Conversely, a circRNA derived from the VPS13C locus was downregulated, suggesting circVPS13C could be subjected to post-transcriptional regulation independent of its host gene expression (Fig. 4b).

To elucidate molecular mechanisms that regulate circRNA biogenesis, we first explored the potential roles of RBPs in circRNA biogenesis, focusing on RBP-encoding mRNAs that are significantly regulated during HOG differentiation. CARP was used to systematically survey eCLIP data for 150 available RBPs to search for their potential binding sites within the flanking introns of each selected circRNA (group 2 in Fig. 4a). Among them 34 RBPs were regulated upon HOG differentiation and their binding site were significantly enriched in the flanking introns of the group 2 circRNAs (Fig. 4c, bar plot) [57]. Interestingly, 8 of the 34 RBPs are known splicing factors (23.53%), which showed significant enrichment compared to splicing factors in the RBP database (7.76%) (chi-squared test, *p*-value = 0.02). This is consistent with the reported roles of RBPs in regulating back-splicing [58]. Specifically, a top-ranked RBP, KHSRP, was recently reported to regulate the biogenesis of a large number of circRNAs, including circVPS13C, in HepG2 and K562 cells [59] (Additional file 1: Figure S6C) [59]. Our RNA-seq analysis revealed downregulation of KHSRP during HOG differentiation (Fig. 4c, heatmap) accompanied with reduced circVPS13C, which is consistent with a function of KHSRP decline in attenuating circVPS13C biogenesis.

We next questioned whether A-to-I editing of a *cis*-regulatory element might contribute to circRNA biogenesis upon HOG differentiation. CARP integrated a published algorithm, Software for Accurately Identifying Locations Of RNA-editing (SAILOR) [60–62], and focused on significantly changed A-to-I editing sites within complementary *Alu* sequences in the flanking intron of DE circRNAs upon HOG differentiation based on RNA-seq data from samples without RNase R treatment. As a result, 71 significant A-to-I editing changes occurred within DE circRNA flanking introns during HOG differentiation, suggesting A-to-I editing is robust in the *cis*-regulatory element of DE circRNA (Binomial test, *p*-value < 2.2 × 10^−16^). Among these, circPRH1 was significantly upregulated upon HOG differentiation and inversely correlated with its host gene expression change (Fig. 4d). Interestingly, several inverted and repeated *Alu* pairs (IR*Alus*) were found in the flanking introns of circPRH1 (Fig. 4d, green and red arrows). In addition, CARP found a significant reduction of A-to-I editing within one *Alu* loci during HOG differentiation, which could contribute to the upregulation of circPRH1 (Fig. 4d).

### Identification of circRNAs that may contribute to HOG differentiation via modulating the activity of miRNAs to regulate mRNA targets

**Fig. 5:**
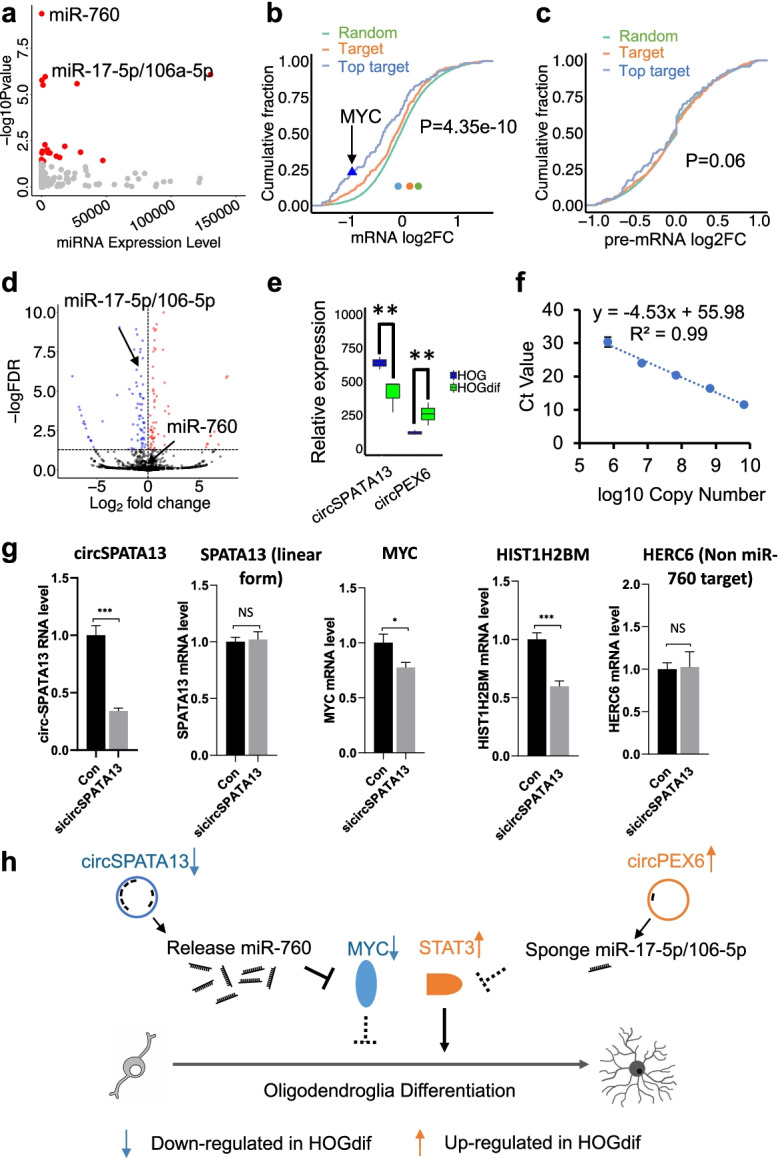
CircSPATA13 regulated oligodendroglioma differentiation via sponging miR-760. **a** Scatter plot showed miRNA expression level (*x*-axis) and significant expression change of their target genes (*y*-axis) during HOG differentiation. An equal number of randomly selected non-miRNA target genes were used as a negative control. **b** Cumulative plot showed miR-760 targets were downregulated in HOG differentiation compared with randomly selected non-miRNA targets. The green line stands for log_2_ fold change of random non-miRNA target genes. The orange line represents log_2_ fold change of target genes according to the TargetScanHuman database. The blue line represents log_2_ fold change of most confident target (top 90% according to context++ score) from TargetScanHuman database. Student’s *t*-test (two-tailed and unpaired) compared top targets versus random targets, and *p*-values indicated. **c** Cumulative plot showed pre-mRNA of miR-760 target genes were not affected in HOG differentiation. **d** Volcano plot showed miRNA expression change upon HOG differentiation. Blue and red dots represent significantly downregulated and upregulated miRNAs during HOG differentiation (DESeq2, FDR < 0.05). Expression of miR-760 was not significantly changed upon HOG differentiation. **e** Bar plot showed circSPATA13 was downregulated while circPEX6 was upregulated in differentiated HOG cells. Cuffdiff was used for gene expression comparison. **P < 0.01. **f** A standard curve was constructed to calculate the copy number of circSPATA13 where the *x*-axis stands for log_2_ copy number of circSPATA13 and the *y*-axis stands for Ct value from qPCR. **g** Expression change of circSPATA13, *MYC*, *HIST1H2BM*, linear *SPATA13*, and *HERC6* upon si-circSPATA13 in HOG cell. The *t*-test (two-tailed and unpaired) was used for gene expression comparison. *n* = 7, ***P < 0.001, *P <0.05, “NS” indicating no significant change. **h** CircRNA-miRNA-mRNA network regulating HOG differentiation. Blue and orange represent down- and upregulated circRNA/miRNA/mRNA upon HOG differentiation**.** Dash lines represent inactivation of upstream regulator promote downstream target or biological process

Because circRNAs are best known as miRNA sponges, to investigate circRNA-miRNA interactions for DE circRNAs during OL differentiation, we hypothesized differentiation-regulated circRNAs in HOG cells may modulate miRNA activity to regulate OL development. We searched for miRNAs whose predicted mRNA targets showed significant expression changes upon HOG differentiation and predicted circRNAs that contain potential binding sites for these miRNAs. Small RNA-seq was also performed in HOG cells with or without differentiation to quantify miRNA abundance using the published algorithm miRge 2.0, which correlated with the expression of their target mRNAs predicted by TargetScan [63, 64]. Using an equal number of random mRNAs without miRNA binding sites as negative controls, CARP identified 45 miRNAs whose target mRNAs were significantly altered during HOG differentiation (Fig. 5a), among which the mRNA targets of miR-760 showed the most significant downregulation during HOG differentiation (*t*-test, *p*-value = 4.35 × 10^−10^) (Fig. 5b). The downregulation of miR-760 target mRNAs likely occurs at post-transcriptional steps, as the pre-mRNA levels of these genes quantified by analyzing intron coverage from RNA-seq data did not show significant changes (*t*-test, *p*-value = 0.06) (Fig. 5c) [65].

Interestingly, miR-760 levels did not change during HOG differentiation (Fig. 5d), raising the question whether a circRNA may sponge and regulate miR-760 activity hence affecting the downstream mRNA targets. Indeed, we identified one circRNA, circSPATA13 (hsa_circ_0004865), which harbors seven predicted miR-760 binding sites and was markedly downregulated during HOG cell differentiation (Fig. 5e, Additional file 1: Fig. S6D). Of note, circSPATA13 is not expressed in M17 cells and the circular exon in human circSPATA13 is poorly conserved in mouse (blastn identity = 66%), suggesting a preferential function of circSPATA13 in human OLs. Each undifferentiated HOG cell was estimated to harbor 2000 copies of circSPATA13 based on a real-time PCR standard curve generated with a known amount of circSPATA13 PCR product (Pearson correlation, *R*^2^ = 0.99, Fig. 5f). Compared with the well-studied functional circCDR1as that efficiently sponges miRNAs when expressed 200–300 copies per HEK293T cell, the amount of circSPATA13 expressed in HOG cells should be sufficient to sponge miR-760 thus may regulate HOG differentiation.

One reported direct target of miR-760 is *MYC*, which suppresses the transition from proliferating OPC to differentiated OLs by binding to the promoter of genes involved in cell cycle regulation and/or chromosome organization [66–71]. The significant reduction of circSPATA13 during HOG cell differentiation is expected to release the sequestered miR-760, which in turn suppresses *MYC*. Indeed, our RNA-seq data revealed downregulation of *MYC* mRNA (Fig. 5b, Additional file 1: Fig. S7A) along with the decline of circSPATA13 (Fig. 5e) in differentiated HOG cells. To directly validate the function of circSPATA13 in modulating the miR-760 pathway, we conducted circSPATA13 knockdown in HOG cells using an siRNA that targets the BSJ sequence of circSPATA13. The level of circSPATA13 was significantly reduced (*P*-value = 2.74 × 10^−6^), without affecting its linear mRNA (Fig. 5g). Importantly, several previously reported or predicted miR-760 targets, including *MYC*, *HIST1H2BM*, *HIST1H3D*, and *HIST3H2A*, were downregulated upon depletion of circSPATA13 whereas the non-miR-760 target *HERC6* was unaffected (Fig. 5g; Additional file 1: Fig. S7E). These data support the model that the developmentally programmed decline of circSPATA13 may turn on the miR-760 pathway independent of altering miR-760 biogenesis to advance human OL differentiation (Fig. 5h).

Reciprocal to the role of miR-760 in suppressing inhibitors of OL differentiation, miR-17-5p and 106a-5p, whose target mRNAs were also significantly altered during HOG cell differentiation (Fig. 5a), share common seed sequences to target STAT3, an important factor known to advance OL development [69, 71]. In contrast to the unaltered miR-760 expression, miR-17-5p/106a-5p levels were significantly downregulated during HOG differentiation (Fig. 5d), accompanied by upregulation of their mRNA targets (Additional file 1: Fig. S7B, C), including the *STAT3* mRNA (Additional file 1: Fig. S7A). Interestingly, miR-17-5p/106a-5p are predicted to be sponged by circPEX6, and each HOG cell is estimated to harbor 369 copies of circPEX6. Upon HOG cell differentiation, circPEX6 was significantly upregulated (Fig. 5e), which is expected to sponge miR-17-5p/106a-5p and accelerate the developmentally programmed decline of miR-17-5p/106a-5p activity (Fig. 5h). Taking together, the reciprocal regulation of the circSPATA13-miR-706 pathway and the circPEX6-miR-17-5p/106a-5p pathway suggest sophisticated functional cooperation of circRNA-miRNA-mRNA networks that drive human OL differentiation, as illustrated in the comprehensive models in Fig. 5h and Additional file 1: Fig. S7D.

### Identification of novel circRNA clusters that may exert additive effects in regulating miRNA activity during HOG differentiation

**Fig. 6:**
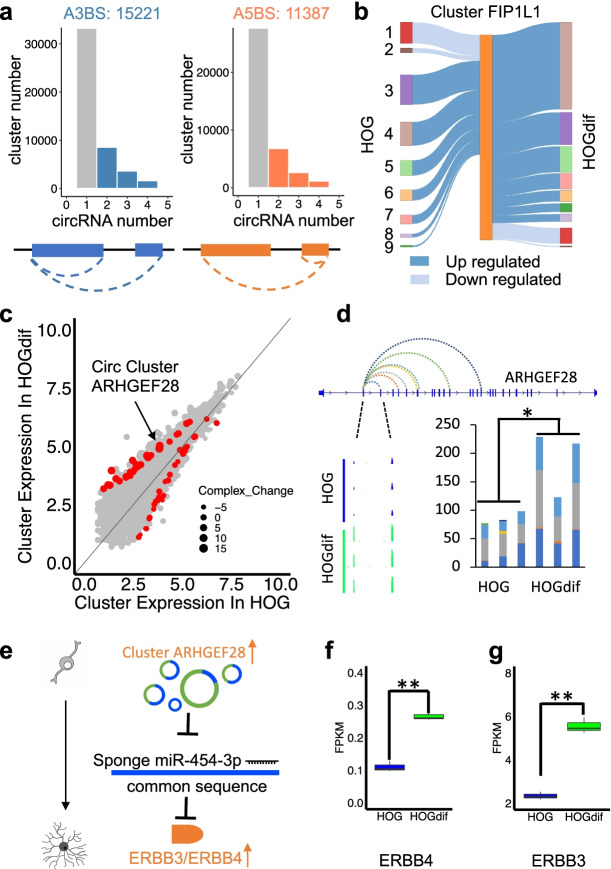
CircRNA alternative circularization generated clustered circRNAs with potential additive functions. **a** Distribution of circRNA numbers in circRNA clusters defined by alternative circularization events. **b** Nine circRNA isoforms in circRNA cluster FIP1L1 identified by A-tailing data display distinct expression patterns during HOG differentiation. **c** Scatter plot shows circRNA cluster complexity and expression changes during HOG differentiation. All dots represent significantly changed circRNA clusters during HOG differentiation. Insignificant circRNA changes are in red. Dot size represents the change of circRNAs number upon HOG differentiation within each cluster. **d** Cumulative bar plot showed the expression change of each circRNA in circRNA cluster ARHGEF28. IGV view showed a common region where circRNA cluster ARHGEF28 accumulated and significantly enriched during HOG differentiation. DESeq2 was used for circRNA cluster expression comparisons. *P < 0.05. **e** Schematic diagram showed that a common sequence of circRNA cluster ARHGEF28 (blue) could potentially regulate OL differentiation by sponging miR-454-3p. **f** Expression of *ERBB4* upon HOG differentiation. **g** Expression of *ERBB3* upon HOG differentiation. Cuffdiff was used for circRNA expression comparison. **P < 0.01

Although many circRNAs were reported to function individually, alternative circularization of circRNAs that share one common BSJ site could form “circRNA clusters,” including alternative 3′ back splicing (A3BS) and alternative 5′ back splicing (A5BS) (Fig. 6a). In HOG cells, CARP found 15,221 and 11,387 clusters containing more than one circRNA with a common 3′ or 5′ BSJ site, respectively (Fig. 6a). For example, one circRNA cluster, FIP1L1, contained nine circRNAs identified by our A-tailing datasets, while untreated RNA-seq only identified one circRNA (circFIP1L1 form #4 in Fig. 6b) (Fig. 6b). Thus, our data provided better sensitivity and additional information compared with previous methods. Interestingly, a clear switch of dominant circRNAs within circRNA cluster FIP1L1 can be detected upon HOG differentiation. Specifically, the circFIP1L1 form #4 level accounted for 19.79% of total circRNAs produced within the circFIP1L1 cluster in HOG cells but elevated to 41.62% in differentiated HOG cells (Fig. 6b). The alternative circularized circRNAs within this cluster appeared to undergo independent regulation, as circFIP1L1 form #1 and 2 were downregulated in contrast to the rest during HOG differentiation, suggesting that a circRNA cluster could provide diverse functions from the same loci.

Importantly, all circRNAs within one cluster contain a common sequence due to the nature of shared 5′ or 3′ back splicing sites, which could act in an additive manner for sponging miRNAs and/or RBPs. The function of clustered circRNAs was often overlooked by previous methods when none of the individual circRNAs were significantly changing, but the common sequence expression shows significant expression change due to additive effect during differentiation. By comparing control and differentiated HOG cells, we identified 533 DE circRNA clusters, 123 of which did not contain significant DE circRNA individually and can be neglected by DE circRNA calling (Fig. 6c, red dots). One of the DE circRNA clusters, ARHGEF28, contains six alternatively circularized circRNAs, none of which showed significant alteration during HOG differentiation. However, the common sequence shared in all the alternative circRNAs derived from this cluster showed significant upregulation (Fig. 6d, e, the sequence in blue). Noticeably, the common sequence was predicted to sequester miR-454-3p, and the overall miR-454-3p target mRNAs were upregulated in differentiated HOG cells post-transcriptionally (Additional file 1: Fig. S8A, B). The top targets of miR-454-3p were subjected to KEGG pathway analysis and found enriched in critical biological pathways involved in OL development, including the mTOR signaling pathway, Wnt signaling pathway, and ErbB signaling pathway (Additional file 1: Fig. S8C). Several miR-454-3p top targets, including *ERBB4* and *ERBB3*, were indeed upregulated during HOG differentiation (Fig. 6f, g), suggesting a potentially novel mechanism by which clustered circRNAs could play additive roles in regulating OL development (Fig. 6e).

## Discussion

This study provided a 21-module computational framework CARP optimized for an A-tailing approach to identify and quantify full-length circRNAs. By applying the A-tailing approach and CARP in the human OL cell line HOG, we identified hundreds of human OL-specific circRNA that regulate OL early differentiation. Furthermore, multiple circRNAs and circRNA clusters were found to form a complicated network with miRNAs and genes in advancing OL differentiation. Thus, to our knowledge, this study stands for the first circRNA profiling in human OL early development.

Current methods for circRNA identification and quantification based on BSJ reads suffer from insufficient power and sensitivity and high FDR owing to the relatively low expression level of many circRNAs [29]. To bypass these hurdles, we adopted a published A-tailing method coupled with RNase R treatment in Li^+^ buffer, and our results suggested the A-tailing method could effectively resolve linear RNAs and enrich circRNAs [32]. We also developed a comprehensive computational framework, CARP, optimized for A-tailing or other RNase R-based experimental data. With a full range of customized and flexible cutoff for a number of bases that match to back-splice junction flanking sites for pseudo-reference alignment to achieve the best balance between FDR and sensitivity from different datasets for circRNA identification, CARP optimized an 8-bp seed sequence matching stringency to pseudo-reference for our dataset and subsequently filtered false-positive reads that could map to the genome or the transcriptome after pseudo-reference mapping. Furthermore, by comparing with a linear reference from the last exon, CARP could further remove A-tailing sensitive circRNAs, which are likely false positives. Without compromising the quality and FDR, CARP reported more circRNAs than most BSJ-based algorithms, including CIRCexplorer2, findcirc, and MapSplice, with higher accuracy.

Since the quantification of circRNA is highly dependent on each individual algorithm, often resulting in difficulties to cross-compare their output results. This issue, however, can be overcome by pseudo-reference-based approach such as CARP by universally merge potential circRNA junctions identified by different approaches. Therefore, CARP offers a streamlined computational approach that maximizes the sensitivity of circRNA detection and standardize the quantified output for better cross-reference comparison. The advantages of using pseudo-reference alignment have also been confirmed by recent publications [33, 72]. Furthermore, CARP provided accurate circRNA quantification and sensitive DE analysis compared with RNase R treatment only, which may be biased because of the uneven efficiency of the RNase R treatment [33].

Importantly, obtaining circRNA full-length information is critical in determining their functions, a task that most BSJ-based circRNA identification software cannot fulfill [4]. Given the effective removal of linear RNAs, CARP could pinpoint circRNA sequence composition using split reads precisely. Despite cell-type specificity, we are still able to identify 12,242 common circRNAs between the two datasets from 44,705 circRNAs identified in HOG cells by CARP and 35,801 circRNA identified in the brain by isoCirc and 10,472 (85.54%) of them displayed identical internal structure between the two datasets, suggest a high level of consistency [36]. Compared with recent efforts that determine circRNA full-length information by long-read sequencing strategy [35, 36], CARP can take advantage of the better coverage and cost-effectiveness of Illumina sequencing to identify low expression circRNAs and quantify circRNA expression more accurately. Meanwhile, the circRNA sequence composition from CARP was critical for downstream circRNA functional investigation and isoform switch events detection. Consequently, CARP provided a framework to predict circRNA-miRNA interplay, considering circRNA expression level, miRNA binding site, miRNA expression level, and their target mRNA expression change. Together, CARP is a highly integrated, multi-functional, and comprehensive framework covering multifaceted circRNA biology.

To date, most efforts were made to study the functions of individual circRNAs solely based on the BSJ [6, 47, 73, 74]. However, our study revealed that many circRNAs that share BSJ sites in the same parental linear RNAs could undergo alternative circularization. Furthermore, these “clustered” circRNAs with various lengths share pieces of common sequences that could be additive in terms of soaking miRNAs or RBPs. Thus, compared with individual circRNA, common and specific functions by independently regulated clustered circRNAs provide more flexible and complex mechanisms in fine-tune gene expression post-transcriptionally. Importantly, CARP was able to identify much more circRNA clusters than untreated RNA-seq data using the A-tailing approach, making the functions of clustered circRNAs more appreciable, which were often overlooked by individual circRNA studies. In addition, it has been increasingly acknowledged that stoichiometry must be considered before proposing a sponging model of relatively low expressing circRNAs [4]. Thus, the additive effects of multiple circRNAs derived from the same cluster could be potentially crucial for the functions of low expressing circRNAs to collectively fine-tune the gene expression.

CircRNAs that are independently regulated in the same cluster demonstrated post-transcriptional regulation of circRNAs, which is also supported by the circRNAs which expression changes were inversely correlated with their host genes. Indeed, some circRNAs have been reported to undergo post-transcriptional regulation in *cis* or *trans*, independent of their host genes [13, 75–77]. Given the universal presence of *cis-*regulatory elements in all cell types, *trans*-factors could well account for circRNA tissue specificity. In addition, our data also suggested that A-to-I editing is a potential regulatory mechanism for *cis-*elements due to its dynamic in OL development. As A-to-I editing is well known to be regulated by ADAR, circRNA biogenesis is coordinated with ADAR activity should be a future challenge.

CircRNAs have been reported as critical regulators in many biological processes, including neurodevelopment and functions, but less conserved among species [14, 47, 78]. Mounting evidence has also demonstrated that dysregulation of circRNAs is involved in human neurological disorders, including Alzheimer’s disease, Parkinson’s disease, and Schizophrenia [17, 18, 75]. Despite the recent circRNA profiling in OLs from the human post-mortem brain, circRNA landscape and functions in the difficult-to-obtaining human early OL development, which is crucial for myelin developmental disorders and lesion repair, is unknown. In this study, we applied CARP to explore circRNA landscape and function in HOG cells and identified dynamic circRNA profiles during human OL development for the first time. A significant overlap of circRNA was found between HOG and OLs in the human post-mortem brain [19], demonstrating HOG as an in vitro system for human early OL development. In addition, much more circRNAs were identified from HOG cells, which represents an early OL progenitor cell stage, either showing better sensitivity of our method in detecting circRNA or a novel biology clue that there are much more circRNA expressed in the early OL stage, which may or may not be retained in mature OL stages.

Among various mechanisms for circRNA to regulate biological processes, the most well-defined was to “sponge” miRNAs and interfere with miRNA silencing activities on target mRNAs [4, 9]. In this study, using a multi-step framework for circRNA functional annotation by CARP, we found a sophisticated network that integrate the function of multiple circRNA-miRNA-mRNA pathways to advance OL differentiation. Numerous repressor and enhancer genes have been shown to impact OL differentiation, represented by MYC and STAT3. MYC-induced repressive histone methylation and premature peripheral nuclear chromatin compaction was previously shown to suppress OPC differentiation [66], whereas STAT3 was thought to advance OPC differentiation and myelin repair [71]. However, the functional co-operation between differentiation repressors and enhancers remains poorly understood. The reciprocal regulation of the circSPATA13-miR-760-MYC pathway and the circPEX6-miR-17-5p/106-5p-STAT3 pathway during early differentiation of human OLs revealed by our studies provide the first example that the developmental regulation of circRNAs may facilitate functional integration of differentiation repressors and enhancers to advance neural development. It should be noted that circRNAs can sponge multiple miRNAs, which could subsequently regulate hundreds of genes to form a complicated network. Moreover, the dynamic regulation of numerous circRNAs and circRNA clusters during OL differentiation identified here argues for the importance to further delineate the sophisticated co-operation of multiple circRNA-miRNA-mRNA axes, which is a prevailing challenge for future studies.

## Conclusions

Our studies provide a robust platform that allowed sensitive and reliable identification of the circRNA landscape by combining the improved experimental condition for circRNA enrichment and our computational algorism CARP. Using this method, we identified the first circRNA landscape in human OLs, which contains hundreds of novel circRNAs undergoing dynamic regulation during early OL differentiation. The precise mapping of full-length circRNA sequences by CARP allows reliable computational prediction of sponged miRNAs and revealed novel circRNA clusters that may achieve additive sponging effects. Importantly, we identified circRNA-miRNA-mRNA pathways that are reciprocally regulated during human OL differentiation to achieve functional cooperation and drive human OL differentiation. Together, our studies established improved methods for circRNA landscape identification, discovered novel circRNA sequence features, and drew direct mechanistic connections between circRNAs and the downstream miRNA-mRNA pathways, which provided important new insights into circRNA biology.

## Methods

### Cell culture and differentiation

BE (2)-M17 human neuroblastoma (M17) cells were propagated in DMEM/F12 medium with 10% FBS (HyClone). For differentiation, M17 cells were incubated with DMEM/F12 medium supplemented with 10% FBS and 20 μm retinoic acid (RA, Sigma, r2625) for 10 days with medium changed every other day. HOG cells were propagated in DMEM medium (Invitrogen) supplemented with low glucose and 10% FBS. HOG cells were plated onto culture dishes coded with PDL (Sigma, P1524-25MG) in the differentiation DMEM medium with low glucose, containing 50 μg/ml transferrin, 0.5 μg/ml insulin, 30 nM triiodothyronine (T3), 30 nM selenium, 16.1 mg/L Putrescine, 0.5 mM IBMX (3-isobutyl-1-methylxanthine), and 0.5 mM cAMP (all from Sigma) for 13 days with medium change every other day to induce differentiation.

### Western blot

M17 and HOG cells were lysed in 1× Laemmli Sample Buffer and heated at 95 °C for 5 min. Equal quantities of protein were separated on SDS-PAGE gels and transferred to PVDF membranes (Immobilon-P, Millipore). PVDF membranes were incubated with 5% milk for 1 h at room temperature and probed with primary antibody at 4 °C overnight. Membranes were rinsed three times in Tris-buffered saline (TBS) with 0.1% Tween (TBST) and then probed with horseradish peroxidase-conjugated secondary antibodies (Promega) for 1 h at room temperature. After rinsing in TBST, membranes were visualized by enhanced chemiluminescence (Pierce Biotechnology, Rockford, IL) and imaged using the Chemidoc MP imaging system (BioRad, Hercules, CA, USA). The following primary antibodies were used: anti-QKI5 (A300-183A-1, Bethyl Laboratories, Inc) and anti-EIF5 (SC-282, Santa Cruz Biotechnology, Inc).

### RNA isolation

Cultured cells were harvested, then centrifuged at 1500*g*, and cell pellets were used for RNA isolation. Cell pellets were homogenized in TRIzol using a hand-held pestle homogenizer and incubated in TRIzol for at least 5 min. Chloroform (1:5 ratio) was added, mixed well, and incubated at room temperature for 15 min. Samples were centrifuged at 12,000*g* for 15 min at 4 °C. The top aqueous layer was transferred to a clean tube, and the RNA was precipitated in 3 M NaAc pH 5.2 (10:1 ratio), 4 μl of glycogen (5 mg/ml), 100% isopropanol (1:1 ratio) overnight at − 80°C. The next day, the samples were centrifuged at 20,000*g* for 20 min at 4 °C. The resulting RNA pellet was washed in 75% ethanol, centrifuged at 7500*g* for 10 min at 4 °C. The washed RNA pellet was dissolved in nuclease-free water, quantified by NanoDrop, and the quality was confirmed by agarose gel electrophoresis.

### A-tailing RNase R treatment

Total RNA from HOG and M17 cells were treated for poly (A) tailing and with RNase R as described in the published method with modifications [32]. In brief, 3 μg of total RNA was subjected to poly (A) tailing in a 50 μL reaction using the poly (A) tailing kit (Thermo Fisher AM1350) following manufacturer’s instructions; 2 μL E-PAP and 40 U RNase inhibitor (Thermo Fisher Scientific N8080119) were also added to the reaction and incubated at 37 °C for 1 h. First, the RNAs were purified by the RNA Clean & Concentrator-25 (Zymo R1018) kit and eluted in 25 μL nuclease-free water. The RNAs were then treated with 5 U RNase R in 30 μL reactions which contained 25 μL of all RNA samples from A-tailing reaction, 3 μL 10× RNase R Buffer (0.2 M Tris–HCl (pH 8.0), 1 mM MgCl_2,_ and 1 M LiCl) and 1 μL RiboLock RNase Inhibitor (40 U/ μL) (Thermo Fisher Scientific EO0381). According to the manufacturer’s instructions, reactions were purified with RNA Clean & Concentrator-25 (Zymo Research R1018), and the RNA was eluted in 30 μL nuclease-free water. Then, the amount of RNAs (in 30 μL nuclease-free water) was used to prepare rRNA depleted RNA-seq library following the KAPA RNA HyperPrep Kit with RiboErase (HMR).

### Library preparation and high-throughput sequencing

For the rRNA-depleted RNA-seq library, sample quality was assessed by Bioanalyzer 2100 Eukaryote Total RNA Pico (Agilent Technologies, CA, USA) and quantified by Qubit RNA HS assay (Thermo Fisher). Ribosomal RNA depletion was performed with Ribo-zero rRNA Removal Kit (Illumina Inc., San Diego, CA) followed by NEBNext® Ultra™ II Nondirectional RNA Library Prep Kit for Illumina® per manufacturer’s recommendation. Library concentration was measured by qPCR, and library quality was evaluated by Tapestation High Sensitivity D1000 ScreenTapes (Agilent Technologies, CA, USA). Equimolar pooling of libraries was performed based on qPCR values. Libraries were sequenced on a HiSeq with a read length configuration of 150 PE, targeting 80M total reads per sample (40 M in each direction).

For small RNA-seq library, total RNA sample quality was assessed by RNA ScreenTape (Agilent Technologies Inc., CA, USA) and quantified by Qubit 2.0 RNA HS assay (Thermo Fisher, MA, USA). According to the manufacturer’s instructions, all library construction occurred according to the QIAseq miRNA Library (Qiagen, Hilden, Germany). The final library quantities were assessed by Qubit 2.0 (Thermo Fisher, MA, USA), and quality was assessed by TapeStation HSD1000 ScreenTape (Agilent Technologies Inc., CA, USA). The final library size was about 180 bp with an insert size of about 20 bp. Illumina® 8-nt single-indices were used. Equimolar pooling of libraries was performed based on QC values and sequenced on an Illumina® HiSeq (Illumina, CA, USA) with a read length configuration of 150 PE for 10M Single-ended reads per sample.

### Linear RNA quantification in HEK293T and SH-SY5Y cell

Paired-end reads from rRNA depleted RNA-seq for untreated, RNase R (K^+^) treated, RNase R (Li^+^) treated, and A-tailing RNase R (Li^+^) treated library were mapped to human genome assembly version (GRCh38/hg38) using TopHat2 version 2.1.1 with default parameter. Bam files were sorted according to genome coordinate by “samtools sort” and converted to bed file by “bedtools bamtobed” with flag “-split.” Bed files were sorted by “sort -k 1,1 -k2,2n” according to the instruction manual for bedtools. The genome coordinate of the last exon of each gene was extracted from gene structure annotation of hg38 and downloaded from the UCSC table using a homemade Perl script. Reads count for each last exon were counted by “bedtools coverage” with flag “-sorted -counts -split” from sorted bed file of each sample. Read counts for the last exon were then normalized to total sequencing reads and exon length as RPKM to stand for linear RNA expression level according to the following equation:

$$\mathrm{RPKM}=\frac{\mathrm{Reads}\ \mathrm{Count}\times \mathrm{1,000,000}\times 1000}{\mathrm{Total}\ \mathrm{Sequencing}\ \mathrm{Reads}\times \mathrm{Exon}\ \mathrm{Length}}$$

### Identification of candidate circRNAs

Candidate circRNAs for each rRNA-depleted RNA-seq sample were first identified individually by CIRCexplorer2, CIRIquant, find_circ, and MapSplice. For CIRCexplorer2, reads were mapped to hg38 by TopHat2 version 2.1.0 with flag “--fusion-search --keep-fasta-order --no-coverage-search --library-type fr-unstranded”. The output bam files were used for “CIRCexplorer2 parse” with flag “--pe -t TopHat-Fusion.” CircRNA were then annotated by “CIRCexplorer2 annotate” with default parameter. CIRIquant was used to identify candidate circRNAs with default parameters with bwa version 0.7.17, HISAT2 version 2.1.0, StringTie version 2.0.3, and Samtools version 1.9. find_circ was used to identify candidate circRNAs following its instruction using Bowtie 2 version 2.3.5.1 and Samtools version 1.9. MapSplice was used for circRNA identification with flag “--bam --fusion-non-canonical --min-fusion-distance 200.” These four software tools and parameters are integrated with CARP now and could be automatically run using “CARP CIRCexplorer2,” “CARP CIRIquant,” “CARP findcirc,” and “CARP MapSplice” with the proper configuration file.

### Confident circRNA identification and quantification

Candidate circRNAs identified by CIRCexplorer2, CIRIquant, find_circ, and MapSplice from all A-tailing samples were pooled together and annotated to their host transcripts. By using “CARP PseudoRef,” a 248-bp “pseudo-reference” was constructed for each circRNA flanking its back splicing junction site (± 149 bp) with 8 bp center sequence as “seed sequence.” Reference for linear isoform quantification was also constructed using the last exon of their host gene (referred hereafter as “the last reference”) by “CARP PseudoRef.” Using “CARP Mapping,” reads from A-tailing and untreated library were mapped to “pseudo reference” and “last reference” by Bowtie 2 using the default parameter. Reads mapped to “pseudo-reference” were compared with seed sequence by “CARP BSJreads” and mapped to genome and transcriptome by Bowtie 2 and TopHat2 using “CARP Remap,” respectively. Reads mapped to genome or transcriptome or did not precisely match the 8 bp “seed sequence” were considered linear isoform derived reads and removed from downstream analysis. Using “CARP ReadsCount,” the remaining reads were used for circRNA identification and quantification, while reads mapped to “last reference” were used for linear RNA quantification. CircRNAs with read counts less than 2 were excluded, and the ratio for read counts in the A-tailing RNase R library and the untreated library was calculated to differentiate RNase R-sensitive or resistant reads. Since linear RNAs, not circRNAs, can be degraded in the A-tailing library, the ratio distribution for linear RNA and circRNA should display a clear difference, as shown in Fig. 1f. We defined a cutoff for A-tailing/Control ratio according to ratio distribution of linear RNAs to ensure a ratio of > 95% linear RNA, which should be sensitive to A-tailing RNase R treatment are lower than this cutoff (Fig. 1f, dash line). CircRNAs which have a ratio higher than this cutoff were considered as confident circRNAs which should be resistant to A-tailing RNase R treatment. Therefore, we controlled the false discovery rate of confident circRNA to 0.05 by using this cutoff.

### CircRNA full-length construction and isoform switch detection

Read pairs that mapped to circRNA “body structure” or pseudo-reference were extracted to determine circRNA internal structure. Mapped reads spanning discontinuous regions in circRNA were regarded as “split reads” and were used to identify candidate junction sites in full-length circRNAs. Junction sites supported by more than two split reads were considered actual splicing sites in circRNA bodies and reported by CARP. The maximum read count for each junction and back splice junction (BSJ) was considered as the total expression level of this specific circRNA isoform, and the following equation calculated the proportion of each junction site:

$$\mathrm{Proportion}\ \mathrm{of}\ \mathrm{junction}\ \mathrm{site}=\frac{\mathrm{Reads}\ \mathrm{count}\ \mathrm{support}\ \mathrm{this}\ \mathrm{junction}\ \mathrm{site}}{\mathrm{Maximum}\ \left(\mathrm{Reads}\ \mathrm{count}\ \mathrm{of}\ \mathrm{each}\ \mathrm{junction}\ \mathrm{site}\ \mathrm{and}\ \mathrm{BSJ}\right)}$$

For downstream functional prediction, the proportion of each junction site of circRNAs was compared, and the dominant isoform of circRNAs was reported. For circRNA alternative splicing analysis, the proportion of each junction site was compared across samples by *t*-test, and *p*-value < 0.05 was considered significant alternative splicing events to cause circRNA isoform switch. CircRNA full-length construction and isoform switch prediction was conducted by “CARP CircAS” and “CARP CircIsoformSwitch.”

### Expression analysis for individual circRNA and circRNA cluster

CircRNA expression levels were normalized before differential expression analysis. Back splicing junction (BSJ) read counts (RC) for each circRNA were first calculated by counting reads mapped directly to a pseudo-reference to normalize circRNA expression in the A-tailing library. The following equation normalized RCs:

$$\mathrm{Normalized}\ \mathrm{RC}=\frac{\mathrm{BSJ}\ \mathrm{RC}\ \mathrm{in}\ \mathrm{A}-\mathrm{tailing}\ \mathrm{Library}}{\mathrm{Total}\ \mathrm{BSJ}\ \mathrm{RC}\ \mathrm{in}\ \mathrm{A}-\mathrm{tailing}\ \mathrm{Library}}\times \frac{\mathrm{Total}\ \mathrm{BSJ}\ \mathrm{RC}\ \mathrm{in}\ \mathrm{Control}\ \mathrm{Library}}{\mathrm{Total}\ \mathrm{Mapped}\ \mathrm{Reads}\ \mathrm{in}\ \mathrm{Control}\ \mathrm{Library}}$$

Differential analyses for individual circRNAs were conducted by a well-defined algorithm DESeq2 using normalized reads count integrated with “CARP DEcirc.” CircRNAs sharing a common 5′ donor site or 3′ acceptor site were defined as one circRNA cluster. Using “CARP CircCluster,” expression of the circRNA cluster was calculated as the total expression of each circRNA in this cluster. DE analysis of circRNA cluster was also conducted by DESeq2, and FDR < 0.05 was considered as significant circRNA cluster differential expression.

### Expression analysis of miRNA, mRNA, and pre-mRNA

Small RNA-seq data were mapped to miRNA database for miRNA quantification by miRge 2.0 to human miRNA database with the following parameter: “-sp human -ad AACTGTAGGCACCATCAAT -ai -gff -trf” [63]. Untreated rRNA depleted RNA-seq data were mapped to hg38 by TopHat2 with default parameter followed by gene expression quantification and differential expression analysis using Cuffdiff [76]. Pre-mRNA expression was quantified using an iRNA-seq package according to reads mapped to the intron sequence with flag “-g hg38 -count intron” [65]. Differential expression analysis for miRNA and pre-mRNA was performed by DESeq2, and FDR < 0.05 was considered a significant expression change.

### CircRNA-miRNA-mRNA network construction

Using “CARP CircNetwork,” miRNA binding sites in circRNA were predicted by targetscan_70 using full-length circRNA sequence [77]. The mRNA targets of these miRNAs were obtained from TargetScanHuman and ranked based on context++ scores [64]. Top targets for a specific miRNA were defined by weighted context++ score ranking higher than 90 percentile. Using “CARP miRTarget,” the log_2_ fold changes upon differentiation between top targets and random non-target genes were compared by *t*-test. Expression changes having a *p*-value < 0.05 were considered significant changes of miRNAs influences on their target mRNAs. To test whether these miRNA targets were regulated at transcription or post-transcription levels, the pre-mRNA log_2_ fold changes of top target and random non-target genes obtained by iRNA-seq package were also compared by *t*-test, and *p*-value > 0.05 showed no significant differences at the transcription level. CircRNA-miRNA-mRNA networks were constructed according to the predicted binding site and positive interplay among the circRNA-miRNA-mRNA axis.

### Absolute circRNA copy number determination

PCR products for the back spliced junction region of circSPATA13 were first obtained using a divergent primer (Additional file 4) and purified by E.Z.N.A.® Gel Extraction Kit. Then, the purified PCR product was serially diluted from 1 ng/μL to 10 pg/μL, and 1 μL aliquots were used for another round of qPCR using divergent primer. Finally, the copy number of templates used for qPCR was calculated by the following equation:

$$\mathrm{Copy}\ \mathrm{number}=\frac{c\times V\times Na}{M}$$

where *c* stands for the concentration of template used for qPCR, *V* represents the volume used for qPCR (1 μL), *M* represents the molecular weight of template calculated by its sequence, and *Na* represents Avogadro’s constant. A standard curve was generated for copy number and Ct value according to the copy number used for qPCR. Total RNA was extracted from 2.8 × 10^6^ HOG cells and quantified by NanoDrop to measure the circSPATA13 copy per HOG cell. An aliquot of 500ng RNA was reverse transcribed into cDNAs for real-time qPCR. The copy number of circSPATA13 per HOG cell was calculated according to the standard curve and Ct value then divided by total cell number. Further, using the copy number and average normalized reads count of circSPATA13 and normalized reads count values of all detected circRNAs in HOG cell RNA-seq datasets as references, we calculated the copy number for each detected circRNA in HOG cells (Additional file 5).

### CircSPATA13 knockdown in HOG

HOG cells were transfected with 200 pmol siRNA target junction site of circSPATA13 (AAGGAGAAGGAGGAGCCCGUG) and a negative control siRNA (Thermo Fisher Scientific, AM4611) for 48 h by using Lipofectamine 2000 (Invitrogen) following the manufacturer’s instructions. The pDsRed2-C1 plasmid was co-transfected into HOG cells to assess transfection efficiency. Expression of circSPATA13 and linear *SPATA13* were quantified by RT qPCR. Expression of miR-760 target include *MYC*, *HIST1H2BM*, *HIST1H3D*, and *HIST3H2A* were also quantified by RT qPCR with non miR-760 target *HERC6* as a negative control. Primer sequences were listed in Additional file 4.

### qPCR for mRNA and circRNA expression

Five hundred nanograms of RNAs from M17 and HOG cells was used to quantify mRNA expression in cells undergoing differentiation for qPCR analysis using SuperScript III (Invitrogen). For neuron differentiation and OL differentiation, markers were quantified using specific primers for M17 differentiation and HOG differentiation (Additional file 4). In addition, we quantified circRNA expression by qPCR analysis using 500 ng RNAs from HOG cells for reverse transcription with SuperScript III (Invitrogen). Randomly selected HOG enriched circRNAs with different expression levels were used for qPCR validation with a divergent primer (Additional file 4). Correlation of Ct value from qPCR and normalized reads count from RNA-seq data were conducted by cor function in R.

### A-to-I editing and RNA binding protein prediction

The dynamic regulation of A-to-I editing during HOG cell differentiation was calculated by CARP integrated Software for Accurately Identifying Locations Of RNA-editing (SAILOR) using untreated RNA-seq data, and significant A-to-I editing loci were obtained by *t*-test with *p*-value < 0.05 using “CARP CircAtoI” [60]. Regulated A-to-I editing events were overlapped with the *Alu* element downloaded from the UCSC table. RNA-binding protein binding site was from the CLIP-seq peaks file in K562 and HepG2 cells [57]. Common CLIP-seq peak regions from 2 replicates were used as confident binding sites and then overlapped with flanking intron of differentially expressed circRNA. Using “CARP CircRBP,” circRNAs with RBP binding sites in both upstream and downstream intron were regulated by specific RNA binding protein.

## Supplementary Information

**Additional file 1.** Figures S1-S8.

**Additional file 2.** Numbers of circRNAs identified by CIRCexplorer2, CIRIquant, find_circ, MapSplice and CARP.

**Additional file 3.** CircRNA list of each class detected by A-tailing RNase R method in HOG.

**Additional file 4.** Primer sequence for circRNAs and mRNAs qPCR validation.

**Additional file 5.** Estimated copy number of circRNAs detected in HOG by A-tailing RNase R method.

**Additional file 6.** Review history.

## Data Availability

All genome-wide sequencing datasets have been deposited to Gene Expression Omnibus (GEO) repository (GSE181729) [79]. Raw image of the Western blot is published in Mendeley Data (https://data.mendeley.com/datasets/btwnt7yb5m/1). CARP is open-source under an MIT license and public available on Github (https://github.com/YaoLabEmory/CARP) [80] and Zenodo (https://zenodo.org/record/5903550#.YfBHifXML0p) [81]. The software has been extensively tested on Linux.
