# Supplementary material for: Accurate identification of circRNA landscape and complexity reveals their pivotal roles in human oligodendroglia differentiation
Source: Genome Biol. 2022 Feb 7;23:48. doi: 10.1186/s13059-022-02621-1 (PMC8819885; doi:10.1186/s13059-022-02621-1)
Supplement: Supplementary file 1 — Additional file 1. Figures S1-S8. [file 13059_2022_2621_MOESM1_ESM.pdf]

Additional file 1

Accurate identification of circRNA landscape and complexity reveals their pivotal roles in human oligodendroglia differentiation

Yangping Li[1†], Feng Wang[1†], Peng Teng[2†], Li Ku[2], Li Chen[3], Yue Feng[2*] and Bing Yao[1*]

Correspondence: yfeng@emory.edu; bing.yao@emory.edu
[†]Yangping Li, Feng Wang and Peng Teng contributed equally to this work.
[1]Department of Human Genetics, Emory University School of Medicine, Atlanta, GA 30322, USA
[2]Department of Pharmacology and Chemical Biology, Emory University School of Medicine, Atlanta, GA 30322, USA
[3]Department of Biostatistics and Health Data Science, Center for Computational Biology and Bioinformatics, Indiana University School of Medicine, Indianapolis, IN 46202, USA

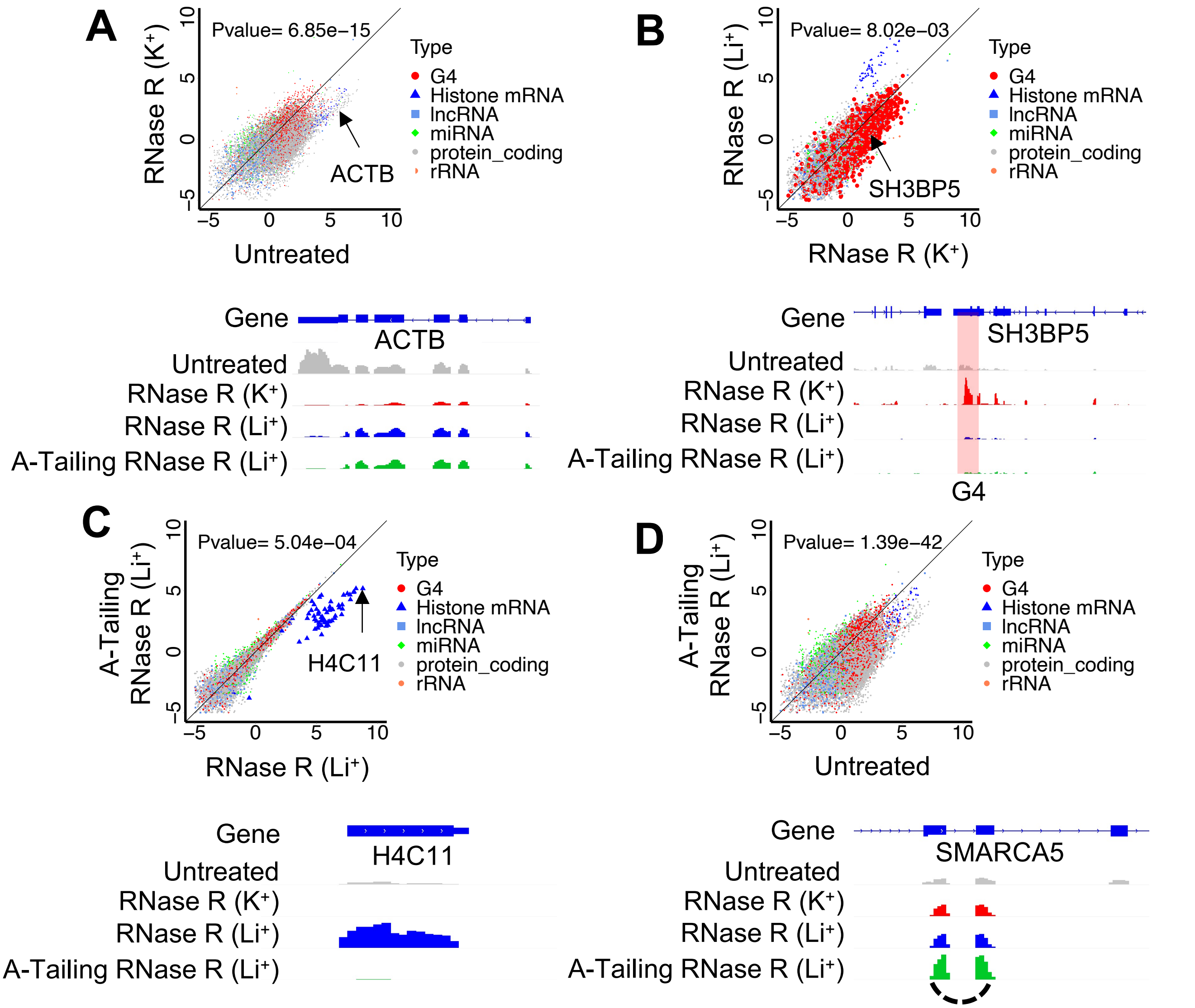

Figure S1 A-tailing RNase R treatment in Li^+ could effectively degrade linear RNAs with circRNAs unaffected in SH-SY5Y cell. **A** Substantial linear mRNAs were degraded by RNase R treatment with K^+ buffer in SH-SY5Y cell. **B** mRNAs with G-quadruplex (G4) structures were effectively degraded by RNase R treatment in Li^+ buffer in SH-SY5Y cell. **C** Linear RNAs with short poly-A tail were resistant to RNase R treatment but could be degraded after adding the Poly-A tail in SHSY-5Y cells. **D** A-tailing approach achieved the best linear RNA removal (scatter plot) without affecting circRNA stability (IGV view) in SH-SY5Y cells.

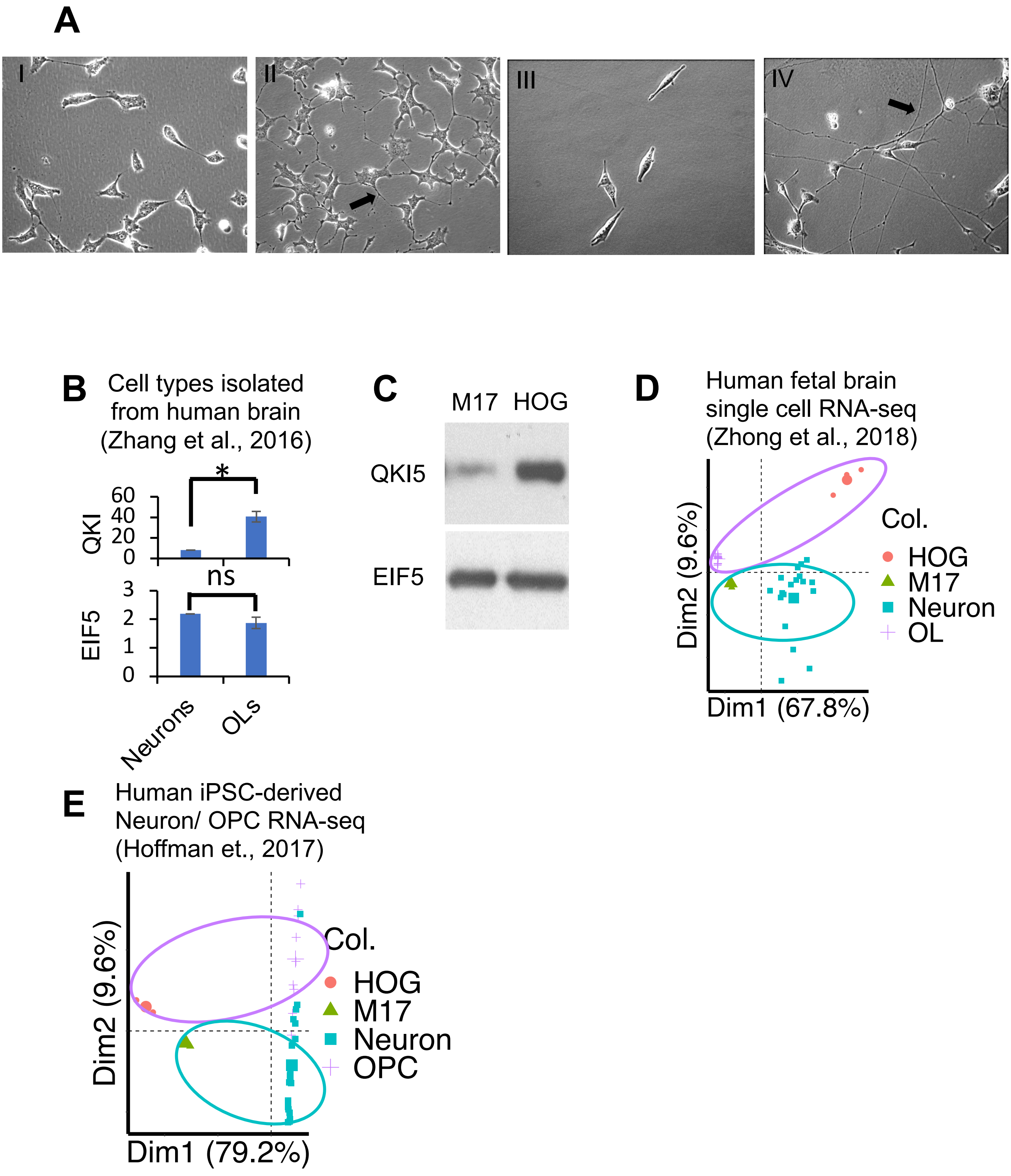
A
I
II
III
IV
B
Cell types isolated from human brain (Zhang et al., 2016)
QKI
60
40
20
0
*
ns
EIF5
3
2
1
0
Neurons
OLs
C
M17
HOG
QKI5
EIF5
D
Human fetal brain single cell RNA-seq (Zhong et al., 2018)
Dim2 (9.6%)
Dim1 (67.8%)
Col.
HOG
M17
Neuron
OL
E
Human iPSC-derived Neuron/ OPC RNA-seq (Hoffman et., 2017)
Dim2 (9.6%)
Dim1 (79.2%)
Col.
HOG
M17
Neuron
OPC

Figure S2 M17 and HOG recapitulate neurons and OLs characteristics. **A** Cell morphology changes upon HOG/M17 differentiation. Prolonged dendrites in differentiated HOG cells and axons in differentiated M17 cells were observed in A(II) and A(IV). **B** Differential expression of *QKI* and *EIF5* were found in human neurons and oligodendroglia *in vivo* (GSE73721). **C** The Western blot of QKI5 and EIF5 in M17 and HOG cells showed similar expression patterns compared to Fig. S2A. **D** Gene expression PCA analysis suggested M17 or HOG cells were closely related to neurons or OLs derived from the human fetal prefrontal cortex, respectively. Neuron and OLs single-cell RNA-seq data were obtained from GSE104276. **E** Gene expression PCA analysis suggested M17 or HOG cells were closely related to iPSC-derived neurons or Oligodendrocyte Progenitor Cells (OPCs), respectively. Neuron and OPCs RNA-seq data were obtained from GSE106589 and GSE130063. * $P < 0.05$.

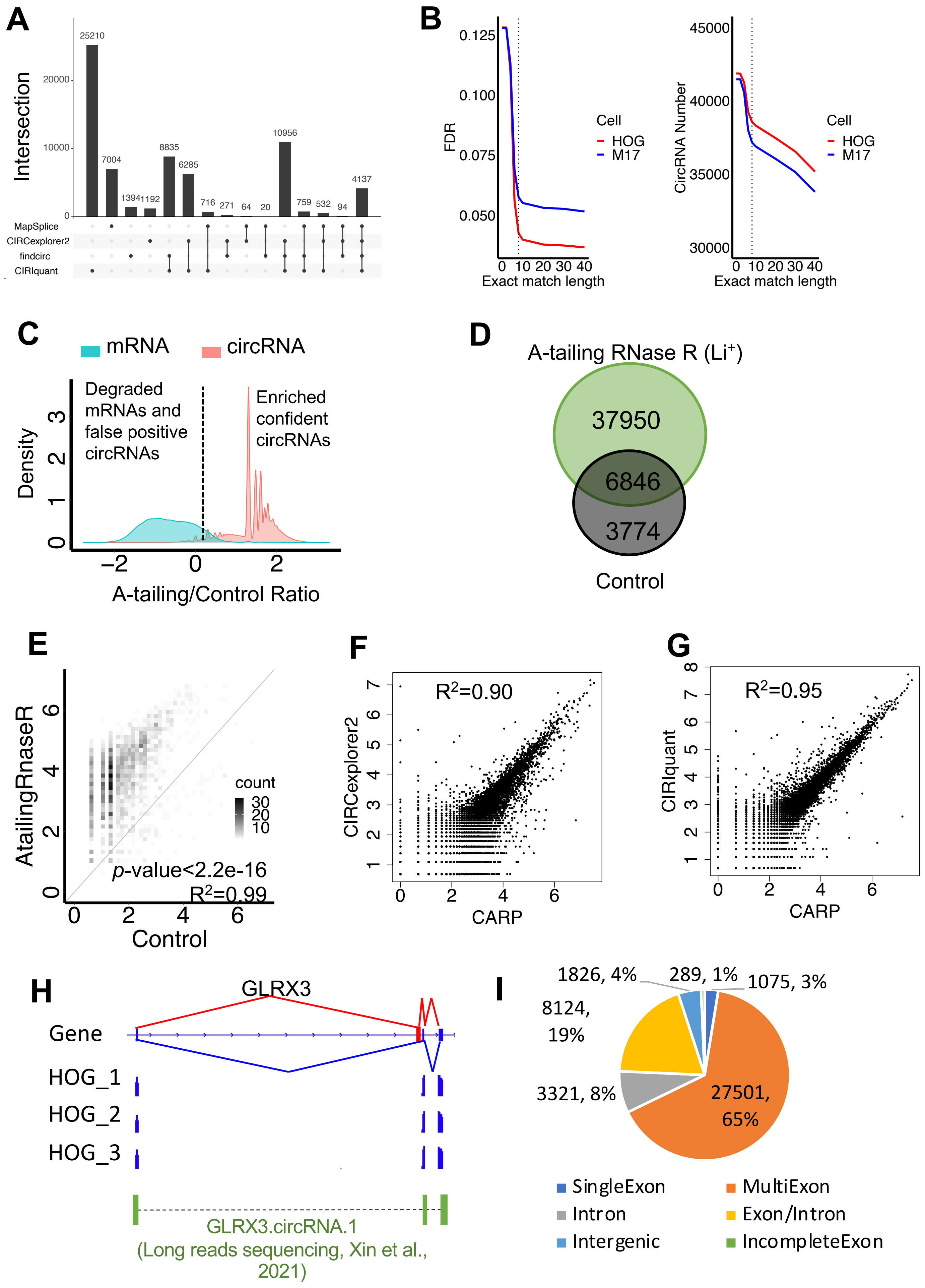

A
Intersection
25210
7004
1394
1192
8835
6285
716
271
64
20
10956
759
532
94
4137
MapSplice
CIRCexplorer2
findcirc
CIRIquant
B
FDR
Exact match length
Cell
HOG
M17
CircRNA Number
C
mRNA
circRNA
Degraded mRNAs and false positive circRNAs
Enriched confident circRNAs
Density
A-tailing/Control Ratio
D
A-tailing RNase R (Li+)
37950
6846
3774
Control
E
AtailingRnaseR
count
p-value<2.2e-16
R2=0.99
Control
F
R2=0.90
CIRCexplorer2
CARP
G
R2=0.95
CIRIquant
CARP
H
GLRX3
Gene
HOG_1
HOG_2
HOG_3
GLRX3.circRNA.1
(Long reads sequencing, Xin et al., 2021)
I
1826, 4%
289, 1%
1075, 3%
8124, 19%
3321, 8%
27501, 65%
SingleExon
MultiExon
Intron
Exon/Intron
Intergenic
IncompleteExon

Figure S3 CARP identified and quantified confident full-length circRNAs. **A** Comparison of circRNAs identified by MapSplice, CIRCexplorer2, find_circ, and CIRIquant using A-tailing data from parental HOG cells. The Student's *t*-test (two-tailed and unpaired) was used for gene expression comparison. **B** A-tailing reads mapping to the pseudo-reference were optimized to eliminate false positives. The association between FDR (y-axis) and the number of base pairs in each read fully matching the BSJ flanking sequence (x-axis) were indicated. Our data shows 8 bp entirely complementary to the BSJ flanking regions in pseudo reference achieved FDR < 0.05 (left, dashed line). The number of circRNAs identified with various stringencies was also indicated (right). **C** Identify confident circRNAs by removing false-positive circRNAs sensitive to A-tailing and RNase R treatment in M17 cells. The ratio of RNA levels between A-tailing treatment and control was calculated and shown on the x-axis. The dashed line with an FDR < 0.05 shows the cutoff defining resistant vs. sensitive A-tailing treatments. **D** Comparison of circRNAs identified by CARP using A-tailing and untreated libraries from HOG cells. **E** Scatter plot showed circRNAs expression quantification was highly correlated in A-tailing and untreated libraries. *P*-value and R^2 for Pearson correlation are shown. **F** Scatter plot showed a high correlation of circRNA quantification between CARP and CIRCexplorer2. R^2 for Pearson correlation was indicated. **G** Scatter plot showed a high correlation of circRNA quantification between CARP and CIRIquant. R^2 for Pearson correlation was indicated. **H** Internal structure of circGLRX3 was different from its host gene. An exon (highlighted in red) was skipped in the circRNA full-length sequence. IGV view confirmed that mapped reads also support an exon skipping event in circGLRX3. The entire length of circGLRX3 was further confirmed by a recent publication using long reads sequencing (highlighted in green). **I** Classification of confident circRNA identified by CARP in HOG. The number and percentage of each class of circRNA were labeled. Corresponding circRNA are listed in Additional file 3.

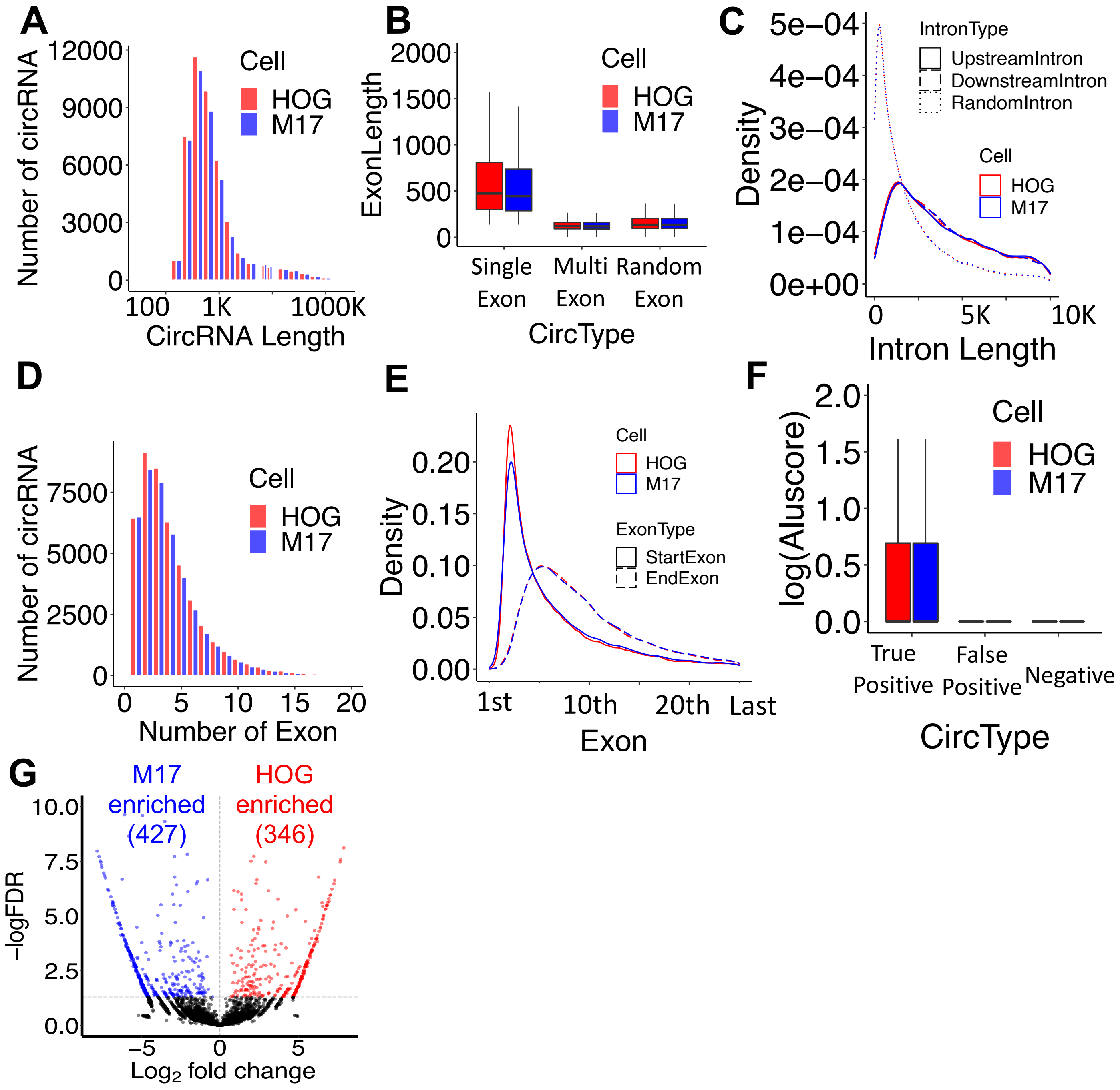

Figure S4 CircRNA showed similar general features but had a distinctive circRNA expression landscape in M17 and HOG cells. **A** Distribution of circRNA length in M17 and HOG cells. **B** Exon length from multi-exon circRNA, single exon circRNA, and random non-circRNA forming exon in M17 and HOG cells. **C** CircRNA flanking intron length distribution in M17 and HOG cells. **D** Distribution of circRNA exon number in M17 and HOG cells. **E** Distribution of start and end exons of multi-exon circRNA in M17 and HOG cells. **F** *Alu* score in the flanking intron of circRNAs in M17 and HOG cells. **G** Volcano plot shows DE circRNAs in M17 and HOG identified by untreated RNA-seq library.

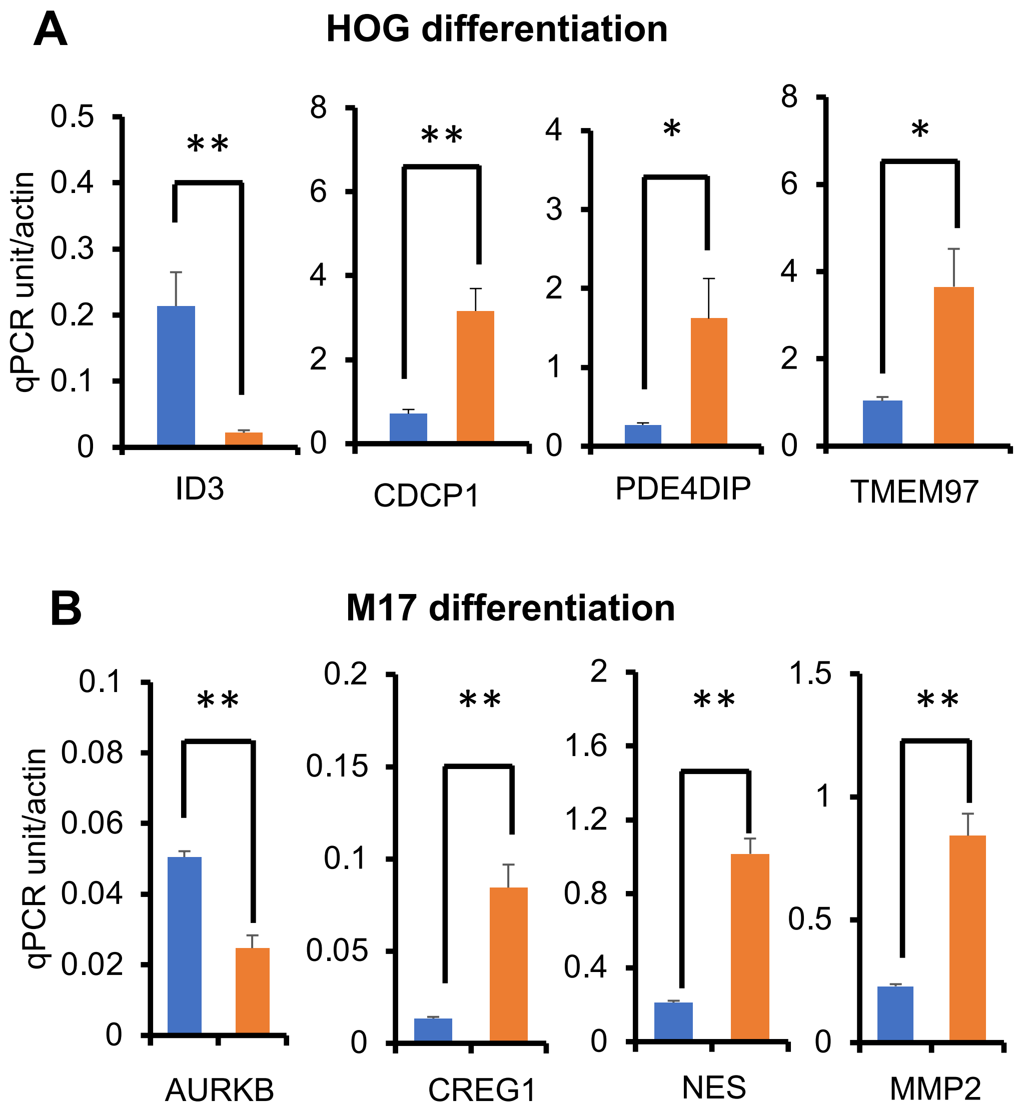

Figure S5 Marker gene expression change suggested M17 and HOG cells could recapitulate neuron and OL differentiation. Expression changes in *ID3*, *CDCP1*, *PDE4DIP*, and *TMEM97* during HOG differentiation were tested by qPCR. **B** Expression changes of *AURKB*, *CREG1*, *NES*, and *MMP2* during M17 differentiation were also tested by qPCR. The Student's *t*-test (two-tailed and unpaired) was used for gene expression comparison (* $P < 0.05$. ** $P < 0.01$).

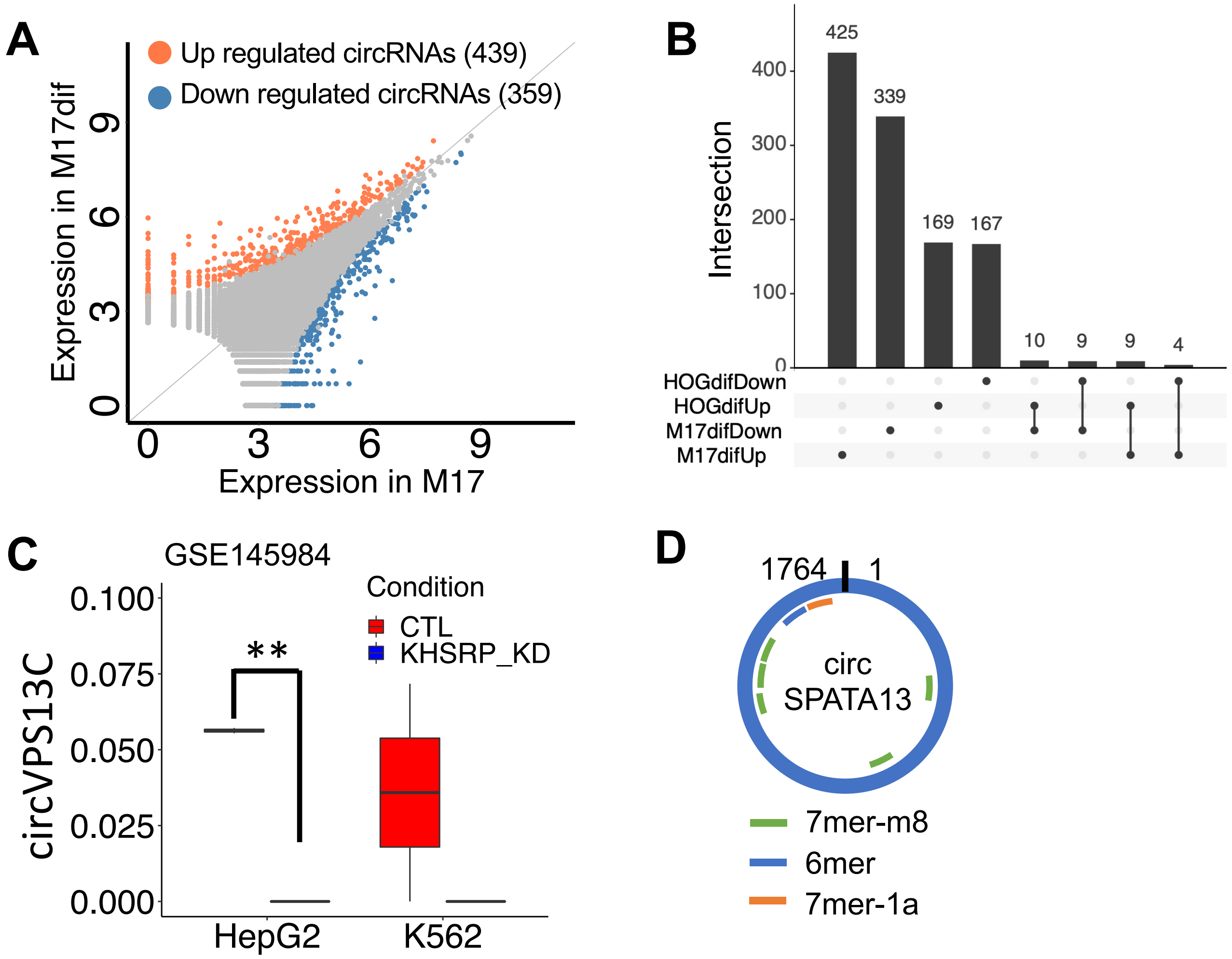

Figure S6 CircRNA expression regulation and their function during HOG differentiation. **A** Scatter plot showed DE circRNAs upon M17 differentiation by A-tailing data (DESeq2, FDR < 0.05). **B** Comparison of up-and downregulated circRNAs during M17 differentiation and HOG differentiation. **C** CircVPS13C was downregulated upon KHSRP knockdown in HepG2 and K562 cells according to published RNA-seq data (GSE145984). **D** CircSPATA13 was predicted to contain 7 conserved miR-760 binding sites. A Chi-squared test was used to compare the overlap in circRNA numbers in differentiated M17 and HOG cells.

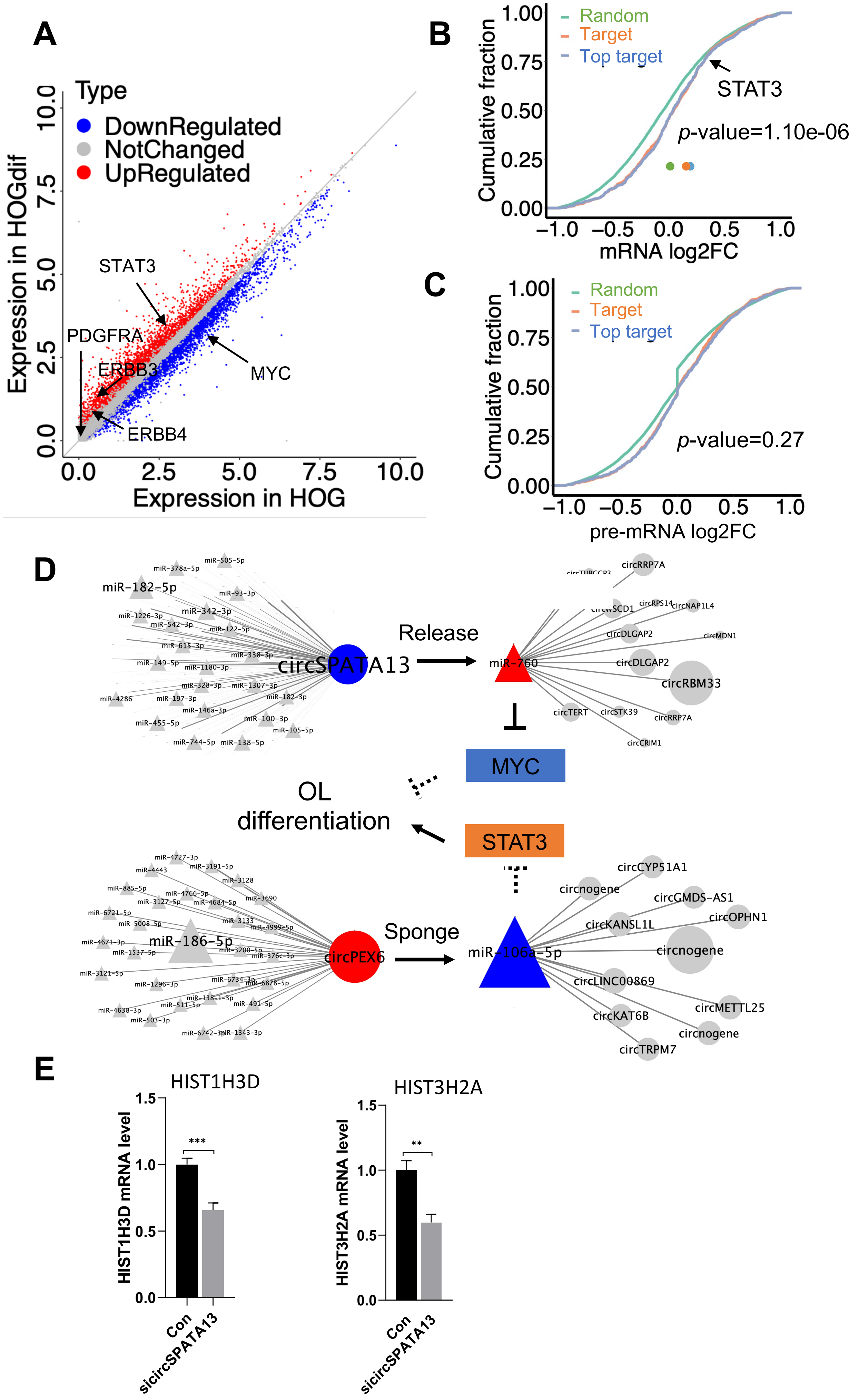
A
Type
DownRegulated
NotChanged
UpRegulated
Expression in HOGdif
Expression in HOG
STAT3
PDGFRA
ERBB3
ERBB4
MYC
B
Random
Target
Top target
Cumulative fraction
STAT3
p-value=1.10e-06
mRNA log2FC
C
Random
Target
Top target
Cumulative fraction
p-value=0.27
pre-mRNA log2FC
D
miR-182-5p
circSPATA13
Release
miR-760
circRBM33
MYC
OL
differentiation
STAT3
Sponge
miR-186-5p
circPEX6
miR-106a-5p
circCYP51A1
circnogene
circGMDS-AS1
circOPHN1
circKANSL1L
circLINC00869
circMETTL25
circKAT6B
circTRPM7
E
HIST1H3D
HIST1H3D mRNA level
***
Con
sicircSPATA13
HIST3H2A
HIST3H2A mRNA level
**
Con
sicircSPATA13

Figure S7 CircRNA functions in regulating mRNA expression via the circRNA-miRNA-mRNA network. **A** Scatter plot showed gene expression changes upon HOG differentiation. Significant up- and downregulated genes are red and blue, respectively (Cuffdiff, FDR < 0.05). **B** Cumulative plot show target genes of miR17-5p/106-5p cluster were significantly upregulated in HOG differentiation compared with randomly selected non-miRNA target genes. *P*-value by Student's *t*-test (two-tailed and unpaired) was indicated. **C** Cumulative plot showed pre-mRNA of miR17-5p/106-5p cluster targets were not affected in HOG differentiation. *P*-value by Student's *t*-test (two-tailed and unpaired) was indicated. **D** CircRNA-miRNA-mRNA network could regulate HOG differentiation by circSPATA13/miR-760/MYC and circPEX6/miR106a-5p/STAT3 pathway. Additional miRNAs that could potentially be regulated by circSPATA13/circPEX6 and additional circRNAs that could potentially regulate miR-760 and miR-106a-5p are shown in grey. **E** Expression change of miR-760 target *HIST1H3D*, *HIST3H2A* upon si-circSPATA13 in HOG cell. *T*-test (two-tailed and unpaired) were used for gene expression comparison. n=7, *** $P < 0.001$, ** $P < 0.01$.

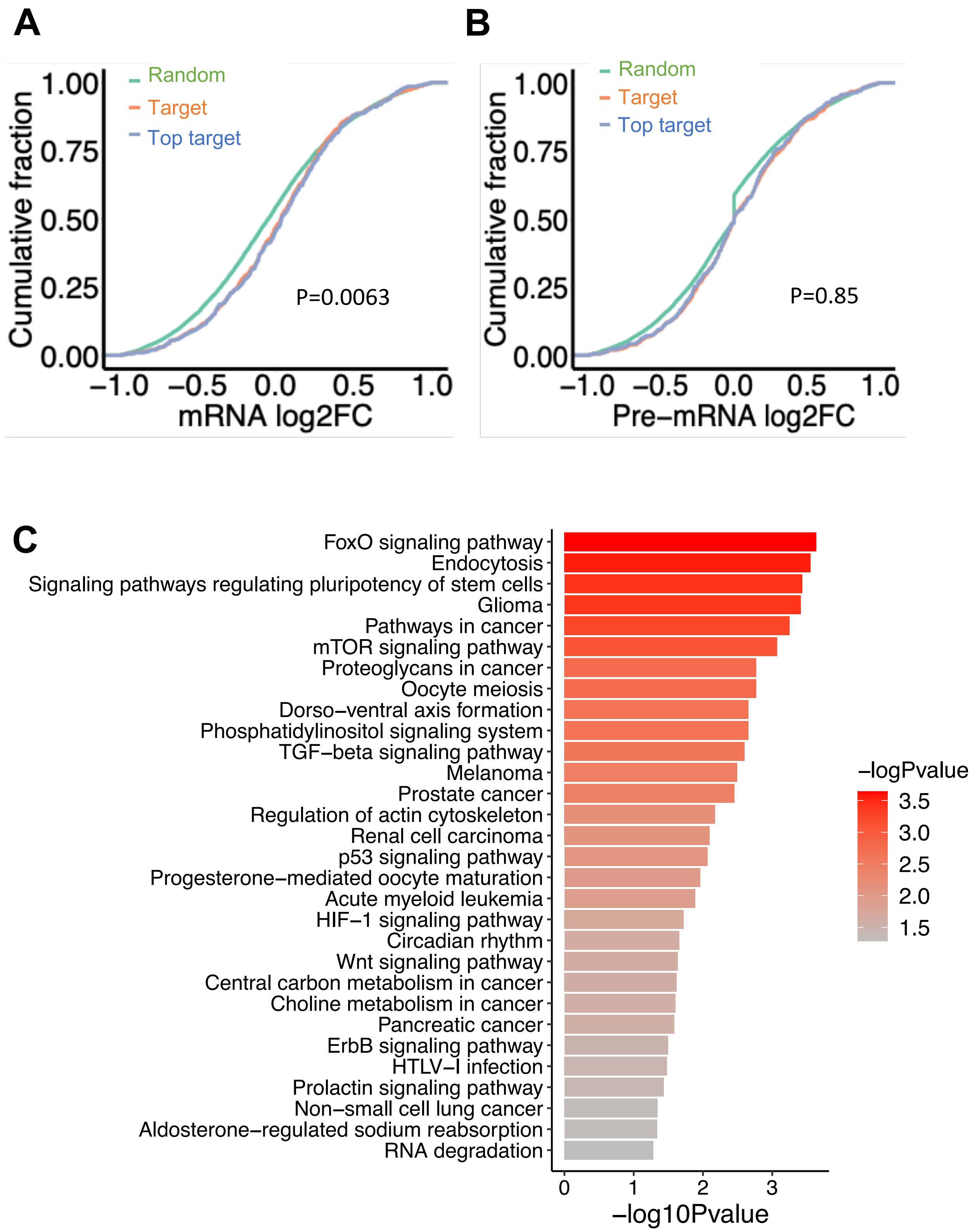

Figure S8 Function of circRNA cluster ARHGEF28 in regulating HOG differentiation. **A** Cumulative plot shows miR-454-3p target genes were significantly upregulated in HOG differentiation. **B** Cumulative plot shows pre-mRNA of miR-454-3p targets were not affected in HOG differentiation. **C** Bar plot shows KEGG analysis for top targets of miR-454-3p (Fisher's exact test, $P < 0.05$).
