# Supplementary material for: Accurate identification of circRNA landscape and complexity reveals their pivotal roles in human oligodendroglia differentiation
Source: Genome Biol. 2022 Feb 7;23:48. doi: 10.1186/s13059-022-02621-1 (PMC8819885; doi:10.1186/s13059-022-02621-1)
Supplement: Supplementary file 2 — Additional file 2. Numbers of circRNAs identified by CIRCexplorer2, CIRIquant, find_circ, MapSplice and CARP. [file 13059_2022_2621_MOESM2_ESM.pdf]

Sample_Name	CIRCexplorer2	CIRIquant	find_circ	MapSplice	BSJ Reads>1	bona fide
M17D0AR_1	20085	52448	24249	11778	40863	38143
M17D0AR_2	12972	33826	18927	6421	31583	30129
M17D0AR_3	23629	60712	27861	13866	45930	43186
M17D10AR_1	14363	36848	18038	7605	30553	28503
M17D10AR_2	16017	40199	19877	8592	31954	29832
M17D10AR_3	14984	37126	18975	7861	31217	29388
HOGD0AR_1	23532	57431	26467	13327	44796	42213
HOGD0AR_2	14966	36231	20715	7029	33976	32887
HOGD0AR_3	21709	52496	24983	11980	42165	40583
HOGD12AR_1	25715	62796	33248	14365	53185	50486
HOGD12AR_2	21697	53341	25480	11829	42324	40420
HOGD12AR_3	20900	50902	28791	10773	46768	45062
M17D0_1	6720	18100	5338	2795		
M17D0_2	6862	19043	6461	2888		
M17D0_3	6903	18495	5676	2767		
M17D10_1	5340	14663	4503	2189		
M17D10_2	3680	10018	5154	1159		
M17D10_3	5682	15248	4792	2338		
HOGD0_2	6595	16913	5283	2571		
HOGD0_3	6685	17130	5407	2512		
HOGD0_4	6346	16556	5230	2357		
HOGD12_2	8477	21442	6655	3327		
HOGD12_3	8342	21066	6737	3323		
HOGD12_4	8456	21580	7309	3376		
