# Supplementary material for: Accurate identification of circRNA landscape and complexity reveals their pivotal roles in human oligodendroglia differentiation
Source: Genome Biol. 2022 Feb 7;23:48. doi: 10.1186/s13059-022-02621-1 (PMC8819885; doi:10.1186/s13059-022-02621-1)
Supplement: Supplementary file 4 — Additional file 4. Primer sequence for circRNAs and mRNAs qPCR validation. [file 13059_2022_2621_MOESM4_ESM.pdf]

Target	Forward Primer	Reverse Primer
ID3	ACTCAGCTTAGCCAGGTGGA	AAGCTCCTTTTGTCGTTGGA
CDCP1	GTTCAAGCTGGAGGACAAGC	CATGGCTCGCTCATTACTCA
PDE4DIP	GGAATTTCGGGAGCTCCTAC	TGGCGCAGTTCTTTTTCTTT
TMEM97	TTCTGTTTTGCGAGCTTGTG	GAAACCACTGGCTTTGGAGA
AURKB	GGGAGAGCTGAAGATTGCTG	GGCGATAGGTCTCGTTGTGT
CREG1	GCACAGACCAACTTCTGCAA	AAGGCCAGGTTTTCATCTCA
NES	AACAGCGACGGAGGTCTCTA	TTCTCTTGTCCCGCAGACTT
MMP2	ATGACAGCTGCACCACTGAG	ATTTGTTGCCCAGGAAAGTG
circHIPK3	GCCAGAGAATATTATGTTGGTGG	GTAGACCAAGACTTGTGAGGC
circSPATA13	CCCCTGGAAGCCACACAT	GTCCTGGCACTCACACCT
circZBTB46	CTGAAGGCCGACGTGCTG	CAGGTGCCGGTAGTGGGA
circPEX6	TGGGCAAGTAGAGATCCTGG	GCTGTAGTGGGGAGAAGACA
HERC6	CACTGGTCAGGTGGTATCTTT	GCAGAAATCAGGCAGCTAATG
SPATA13	TTCCTGAAGACTCGGTTGCT	CATACAAGCTGACCCCACCT
MYC	GGCTCCTGGCAAAAGGTCA	CTGCGTAGTTGTGCTGATGT
HIST1H2BM	GAAAGAAGCGCAAACGCAG	TGATTCCCATAGCCTTGGAAGA
HIST1H3D	CCATTCCAGCGTCTAGTCCG	TCTGAAAACGCAGATCAGTCTTG
HIST3H2A	GGTCGTGGTAAGCAGGGTG	CGCTCCGAATAGTTGCCCTT
