# Supplementary material for: Accurate identification of circRNA landscape and complexity reveals their pivotal roles in human oligodendroglia differentiation
Source: Genome Biol. 2022 Feb 7;23:48. doi: 10.1186/s13059-022-02621-1 (PMC8819885; doi:10.1186/s13059-022-02621-1)
Supplement: Supplementary file 5 — Additional file 5. Estimated copy number of circRNAs detected in HOG by A-tailing RNase R method. [file 13059_2022_2621_MOESM5_ESM.pdf]

CircRNA	Copy Number	Gene
chrX:100914620-100915083	25	XKRX
chr6:31271232-31356326	6668	nogene
chrX:131749305-131794466	1626	FIRRE
chr2:27987992-28045799	13	BRE
chr2:11765532-11767858	122	LPIN1
chr10:101667885-101676436	463	FBXW4
chr2:27987992-28025420	5	BRE
chr14:83646543-83666209	215	nogene
chr18:47865058-47896809	580	SMAD2
chr5:43294055-43297166	86	HMGCS1
chr12:27368261-27380404	2261	ARNTL2
chr7:24623665-24650712	1109	MPP6
chr7:18666212-18666476	84	HDAC9
chr7:155672866-155680908	3240	RBM33
chr12:69251128-69262562	2757	CPSF6
chr5:43295751-43297166	75	HMGCS1
chr4:80335743-80362884	115	C4orf22
chr13:24222818-24224582	2000	SPATA13
chr18:21765771-21779685	2824	MIB1
chr8:127890588-127890998	5153	PVT1
chr1:240123178-240188262	87	FMN2
chr9:20923659-20933103	114	FOCAD
chr1:24514313-24514567	97	RCAN3
chr16:47109482-47132025	1127	NETO2
chr7:155301565-155302417	4	INSIG1
chr7:131375423-131399433	606	MKLN1
chr14:102040235-102040673	587	DYNC1H1
chr3:11358417-11426926	107	ATG7
chr2:206279539-206297373	829	ZDBF2
chr8:125008970-125009343	227	SQLE
chr5:43292473-43297166	138	HMGCS1
chr1:9871186-9877989	32	CTNNBIP1
chr5:179822957-179833782	85	SQSTM1
chr8:1565682-1565894	134	DLGAP2
chr4:80295558-80362884	219	C4orf22
chr13:29453067-29487935	115	nogene
chr9:20923659-20933132	59	FOCAD
chr17:83035034-83048785	416	B3GNTL1
chr15:62007307-62013992	425	VPS13C
chr15:100330888-100334180	518	ADAMTS17
chr9:4286037-4286523	481	GLIS3
chr3:196323743-196324440	130	TM4SF19-TCTEX1D2

chrX:100948917-100949326	0	nogene
chr2:10141853-10142375	318	C2orf48
chr5:73749836-73753202	132	ARHGEF28
chr7:24623662-24668660	381	MPP6
chr6:29887954-29942626	2028	HLA-H
chr3:120750522-120751000	197	GTF2E1
chr22:42519713-42580359	445	RRP7A
chr13:29453067-29492665	93	nogene
chr19:11120091-11120522	361	LDLR
chr19:53521855-53576853	21	nogene
chr1:172551511-172570730	91	SUCO
chr13:24249476-24251862	1366	SPATA13
chr10:101672914-101676436	115	FBXW4
chr6:4891712-4892379	6643	CDYL
chr6:56851396-56900621	128	DST
chr9:95978060-96004701	519	ERCC6L2
chr15:49225204-49239367	173	GALK2
chr17:6080370-6095223	108	WSCD1
chr8:1565682-1633046	104	DLGAP2
chr7:43639448-43640649	826	COA1
chr21:45525816-45538008	141	SLC19A1
chr2:175970763-175995646	389	KIAA1715
chrX:100914620-100949326	8	nogene
chr10:49493116-49506012	116	ERCC6
chr13:23353784-23371165	947	SACS
chr5:1278640-1282624	300	TERT
chr9:93471140-93498886	1425	FAM120A
chr7:24623662-24650712	290	MPP6
chr13:32517856-32527532	1153	N4BP2L2
chr7:158759485-158764853	645	ESYT2
chr7:23185069-23185298	188	NUPL2
chr5:145796441-145826200	576	PRELID2
chr21:45128401-45134832	603	ADARB1
chr1:155438326-155459898	874	ASH1L
chrM:8397-8608	411	nogene
chrX:68192918-68201710	108	OPHN1
chr1:8541213-8557523	328	RERE
chr5:131548046-131604693	65	RAPGEF6
chr1:197642710-197658369	89	DENND1B
chr5:73074741-73077493	248	FCHO2
chr6:10703404-10704844	285	PAK1IP1
chr2:231206197-231216793	6	ARMC9
chr11:65503728-65505019	12	nogene

chr12:46229152-46243314	2173	SLC38A1
chr4:87195323-87195690	343	KLHL8
chr12:11034781-11047188	94	PRH1-PRR4
chr12:27368261-27390239	338	ARNTL2
chr7:30550635-30562128	13	LOC401320
chrX:101041316-101042312	193	TRMT2B
chr12:27368261-27376416	339	ARNTL2
chr10:33254027-33270856	0	NRP1
chr5:180261683-180280608	1152	MAPK9
chrX:68063853-68073299	12	OPHN1
chr22:42516010-42577098	206	RRP7A
chr2:230442936-230450255	236	SP100
chr2:27987992-27995456	0	BRE
chr1:243545509-243573048	239	AKT3
chr1:11030175-11030882	60	MASP2
chr10:50433475-50590247	28	SGMS1
chr7:24623665-24668660	762	MPP6
chr6:42592150-42594304	129	UBR2
chrUn_GL000218v1:90229-90629	116	nogene
chr2:68490189-68545312	123	APLF
chr5:134199085-134206131	405	PPP2CA
chr3:111913318-111920419	162	PHLDB2
chr11:14771936-14789242	303	PDE3B
chr2:11802906-11805156	33	LPIN1
chr21:15014343-15043574	1656	NRIP1
chr12:27368261-27371447	192	ARNTL2
chr1:247156405-247159813	2006	ZNF124
chr14:96846094-96860735	371	VRK1
chr1:247155565-247159813	2019	ZNF124
chr11:62839113-62839867	746	WDR74
chr12:69800208-69801721	401	RAB3IP
chr3:197282678-197282845	0	DLG1
chr5:108348398-108367953	403	FBXL17
chr11:2951258-2979237	389	NAP1L4
chr11:6932093-6955782	200	ZNF215
chr12:77940318-78021862	319	NAV3
chr3:27411641-27424152	375	SLC4A7
chr13:23879267-23886506	211	MIPEP
chr20:63672082-63672549	48	nogene
chr14:99458278-99465813	1519	SETD3
chr7:47994314-47996146	42	SUN3
chr18:9931809-9937066	3004	VAPA
chr5:112981041-112992248	0	DCP2

chr1:149655698-149669310	54	LINC00869
chr4:74174505-74225394	108	MTHFD2L
chr11:46076753-46092223	259	PHF21A
chr9:131506113-131506453	223	POMT1
chr1:178123047-178125501	15	nogene
chr1:155732500-155732748	46	nogene
chr14:96852830-96860735	130	VRK1
chr22:16637039-16638740	528	TPTEP1
chr9:93471140-93476338	987	FAM120A
chr18:8783529-8786091	43	MTCL1
chr7:11061790-11111467	904	PHF14
chr3:11298685-11299416	3	ATG7
chr2:168063499-168129758	168	STK39
chr1:23030468-23044486	149	KDM1A
chr1:19158747-19158910	46	nogene
chr16:1809237-1809833	196	HAGH
chr8:108228266-108241913	297	EIF3E
chr4:112562370-112585725	409	ZGRF1
chr12:1104749-1116033	231	ERC1
chr10:28583398-28596041	84	WAC
chr3:104817472-104855451	242	nogene
chr11:70659827-70661678	299	SHANK2
chr9:14146688-14179780	51	NFIB
chr6:158573419-158625754	188	TMEM181
chr16:8920358-8930397	215	USP7
chrX:100949107-100957528	0	nogene
chrX:68096869-68119332	6	OPHN1
chr7:22308338-22318037	809	RAPGEF5
chr10:45752587-45753237	58	FAM21C
chr9:21476898-21477292	202	MIR31HG
chr12:77940318-78007445	247	NAV3
chr1:45640209-45642499	129	GPBP1L1
chr16:30664214-30666541	650	FBRS
chr4:4086657-4087393	197	nogene
chr4:1311978-1327703	267	MAEA
chr11:9437753-9442197	39	IPO7
chr2:210098085-210154611	20	KANSL1L
chr13:21167987-21168399	251	SKA3
chr6:33776953-33786774	6	LEMD2
chr19:4950610-4950996	254	UHRF1
chr6:83018972-83044659	417	UBE3D
chr14:54981539-55002185	39	WDHD1
chr6:42974002-42975038	369	PEX6

chr7:2213211-2219456	224	MAD1L1
chr18:21765771-21819646	271	MIB1
chr17:82900650-82911789	689	TBCD
chr11:87157340-87158771	49	TMEM135
chr18:69010933-69054133	18	CCDC102B
chr12:50981638-51010766	1	SLC11A2
chr12:82369386-82403130	33	METTL25
chr1:94220257-94231643	307	ARHGAP29
chr1:40189054-40202614	381	RLF
chr19:8539731-8546207	2	nogene
chr7:149494203-149621172	140	ZNF746
chr5:115866669-115870128	279	AP3S1
chrM:8436-14066	424	nogene
chr13:112516439-112527484	243	TUBGCP3
chr11:108319953-108327758	0	ATM
chr9:35295695-35313989	144	UNC13B
chrX:107840669-107854704	427	MID2
chr1:9931890-9934860	664	LZIC
chr6:107503656-107506427	623	SOBP
chr14:104946924-104948904	77	nogene
chr11:61796713-61797143	35	nogene
chrX:21978874-21992712	745	SMS
chr19:31125783-31131933	51	nogene
chr18:9931809-9950568	230	VAPA
chr1:20770929-20773610	110	HP1BP3
chr8:53811163-53829533	171	ATP6V1H
chr6:70589007-70589260	96	nogene
chr12:69251128-69269668	134	CPSF6
chr13:29517143-29517790	140	SLC7A1
chr5:171391304-171392978	360	NPM1
chr11:108715878-108723462	163	DDX10
chr17:82248874-82249602	73	CSNK1D
chr21:46537223-46541895	34	DIP2A
chr6:89751430-89753622	508	MDN1
chr19:11113277-11113762	82	LDLR
chr9:22301599-22347226	1	nogene
chr17:61744431-61780401	152	BRIP1
chr9:96458378-96471094	206	HABP4
chr11:74789625-74817714	638	RNF169
chr14:102830333-102897401	53	TRAF3
chr2:36358903-36396787	378	CRIM1
chr2:168161786-168167407	124	STK39
chr12:27714779-27724186	967	MRPS35

chr11:32587011-32589135	47	EIF3M
chr22:45689711-45718493	412	ATXN10
chr6:43588001-43588486	33	nogene
chr7:75472861-75473093	100	nogene
chr16:11020192-11024921	190	CLEC16A
chr11:68347846-68357847	78	LRP5
chr1:97679094-97721671	0	DPYD
chr1:43671504-43683804	0	KDM4A
chr4:165219933-165263359	765	KLHL2
chr22:33761370-33765953	318	LARGE
chr14:49825866-49831361	34	NEMF
chr7:75394972-75395503	2	nogene
chr6:47503279-47554766	171	CD2AP
chr6:7410385-7411451	121	RIOK1
chr1:229286485-229304000	68	RAB4A
chr8:144190783-144200541	117	MROH1
chr1:45566568-45567020	364	AKR1A1
chr11:77691158-77693611	3	RSF1
chr5:123545416-123545841	35	CSNK1G3
chr5:129104955-129107062	503	ISOC1
chr1:202773117-202777094	33	KDM5B
chr12:19462509-19473355	1151	AEBP2
chr4:139137629-139139497	42	ELF2
chr8:134600435-134610655	98	ZFAT
chr17:83005114-83048785	49	B3GNTL1
chr13:52417991-52427219	125	VPS36
chr10:18553509-18562797	53	nogene
chr1:64812737-64814840	33	RAVER2
chr8:18765448-18804898	175	PSD3
chr10:78035351-78040747	33	RPS24
chr10:5785141-5800705	1183	GDI2
chr18:54877566-54884436	0	RAB27B
chr11:74225204-74239256	150	PPME1
chr14:75109684-75110371	108	NEK9
chr11:123083084-123084713	56	CLMP
chr3:142093060-142101841	116	TFDP2
chr16:30200724-30201293	166	SLX1B-SULT1A4
chr12:19473247-19514784	121	AEBP2
chr20:19997666-19998073	122	nogene
chr2:84441046-84449752	112	SUCLG1
chr10:6157523-6157996	42	nogene
chr17:6087989-6095223	56	WSCD1
chr18:47865058-47870564	170	SMAD2

chr17:7945727-7945986	92	CNTROB
chr19:11110651-11111639	8	LDLR
chr17:1548295-1558559	107	PITPNA
chr5:150445608-150447735	76	RPS14
chr17:20253504-20306082	3	SPECC1
chr19:45398004-45398339	372	PPP1R13L
chr1:245858495-245863886	147	SMYD3
chr11:74118227-74123135	1	C2CD3
chr4:110488540-110520366	0	ENPEP
chr15:41696074-41713496	76	MGA
chr22:36488075-36488630	79	nogene
chr2:173358711-173367831	67	CDCA7
chr3:128262127-128264781	583	EEFSEC
chr11:64228264-64228894	33	nogene
chr17:80922723-80925480	190	RPTOR
chr8:125003175-125005705	0	SQLE
chr18:8718423-8720496	1629	MTCL1
chr7:18762156-18874596	1	HDAC9
chr4:127982938-127989821	0	C4orf29
chr16:31297513-31297954	101	ITGAM
chr5:179705678-179710065	462	CANX
chr20:35899369-35899648	10	PHF20
chr8:116655855-116658980	927	EIF3H
chr20:51366448-51368853	58	nogene
chr11:6932093-6943641	54	ZNF215
chr8:99274197-99275254	117	VPS13B
chrX:68052539-68053810	2	OPHN1
chr12:22493222-22506819	39	C2CD5
chr20:62854015-62860308	188	TCFL5
chr19:51860903-51886912	383	ZNF577
chr10:84417770-84425893	497	CCSER2
chr11:18083534-18089510	93	SAAL1
chr7:93276164-93323732	29	VPS50
chr7:92126252-92131872	80	CYP51A1
chr9:128507215-128509097	29	GLE1
chr11:65435052-65444063	156	nogene
chr12:70278131-70311017	202	CNOT2
chr7:18762156-18835997	0	HDAC9
chr15:50300788-50309798	99	GABPB1
chr15:50648686-50663046	70	TRPM7
chr22:45689711-45729590	33	ATXN10
chr12:409367-418636	30	CCDC77
chr7:2364371-2366448	378	EIF3B

chr3:33684358-33696933	625	CLASP2
chr5:97431743-97439618	1	nogene
chr9:134001083-134002160	1	nogene
chr1:202886489-202886860	32	nogene
chr11:62839113-62839659	500	WDR74
chr4:128515856-128519108	3	LOC100507487
chr10:74842599-74843478	29	KAT6B
chr5:43294055-43298975	5	HMGCS1
chr13:23869291-23886506	189	MIPEP
chr20:29091593-29101550	0	FRG1DP
chr16:29904851-29905959	281	ASPHD1
chr1:223954476-223962273	209	GTF2IP20
chr5:16779544-16783469	278	MYO10
chr5:33881118-33881480	55	ADAMTS12
chr7:22291174-22318037	702	RAPGEF5
chr4:129039040-129082373	136	SCLT1
chr6:43478086-43503706	21	TJAP1
chr3:11358417-11380052	226	ATG7
chr4:53383772-53389873	54	FIP1L1
chr2:235797758-235807331	131	AGAP1
chr12:47104112-47108164	127	PCED1B
chr22:24363260-24369320	0	SPECC1L-ADORA2A
chr3:125848229-125848447	85	nogene
chr4:186706562-186709845	486	FAT1
chr5:16779544-16780608	28	MYO10
chr1:2055440-2059591	82	PRKCZ
chr20:54157168-54171670	216	CYP24A1
chr7:128641202-128648982	191	LINC01000
chrM:10745-10954	91	nogene
chr14:80756891-80778046	53	CEP128
chr6:31648676-31649627	101	BAG6
chr3:151116337-151127984	208	MED12L
chr10:68393137-68394451	317	RUFY2
chr5:38903881-38904503	0	OSMR
chr19:11120091-11123344	38	LDLR
chr2:241403827-241418109	149	FARP2
chr11:65502320-65504595	16	nogene
chr11:46076753-46084302	46	PHF21A
chr3:196391812-196403019	2353	UBXN7
chr2:72731006-72733118	339	EXOC6B
chr15:49235850-49239367	459	GALK2
chr6:2397220-2399037	17	GMDS-AS1
chr6:57507386-57606457	27	PRIM2

chr10:1103601-1105267	65	WDR37
chr2:219174145-219175108	0	CNPPD1
chr19:11120091-11128085	13	LDLR
chrX:24172714-24179770	167	ZFX
chr4:6923372-6967424	400	TBC1D14
chr3:63898398-63913225	51	ATXN7
chr1:93324634-93325895	117	LOC100131564
chr5:179822957-179825226	31	SQSTM1
chr9:21815432-21818202	115	MTAP
chr19:12878381-12878834	16	DNASE2
chr13:112504031-112527484	36	TUBGCP3
chr17:20204332-20205912	1305	SPECC1
chr16:29833732-29834066	341	MVP
chr7:677228-680726	120	PRKAR1B
chr21:15014343-15045323	59	NRIP1
chr7:73686635-73687095	313	WBSCR22
chr2:238433312-238433695	149	ASB1
chr11:93747296-93757465	11	C11orf54
chr3:9750944-9751949	112	OGG1
chr7:155706859-155718443	289	RBM33
chr22:45729424-45740538	793	ATXN10
chr3:143985542-143989837	1156	C3orf58
chr12:51055834-51056502	72	LETMD1
chr4:128466607-128470549	4	LOC100507487
chr2:169606038-169608758	3	PPIG
chr8:69738039-69761875	27	SLCO5A1
chr22:25685169-25704209	0	ADRBK2
chr8:144512391-144512771	123	RECQL4
chr1:94487541-94491247	124	ABCD3
chr8:130152735-130180880	2011	ASAP1
chr12:7403534-7433694	443	CD163L1
chr20:49233305-49233709	48	DDX27
chr1:22078428-22086866	520	CDC42
chr5:109713519-109716264	840	MAN2A1
chr2:134317528-134318739	101	MGAT5
chr10:127125473-127127764	172	DOCK1
chr2:109564365-109565859	55	10-Sep
chr20:35653527-35659014	367	CPNE1
chr3:142736378-142748460	1385	TRPC1
chr9:125207563-125213522	0	RABEPK
chr11:62781893-62782825	58	TAF6L
chr9:33953284-33996333	1	UBAP2
chr17:82763964-82772507	376	TBCD

chr1:203707008-203708104	4	ATP2B4
chr3:172247532-172251541	690	FNDC3B
chr16:2757471-2760499	8	SRRM2
chrX:134473358-134493590	581	HPRT1
chr5:132892163-132893118	96	AFF4
chr19:50800677-50801750	49	nogene
chr1:32945513-32946564	0	RNF19B
chr8:102833055-102836390	43	AZIN1
chr13:30280062-30283791	1440	KATNAL1
chr5:66202824-66208992	32	nogene
chr12:27368261-27370230	193	ARNTL2
chr18:62072672-62095950	208	PIGN
chr18:77095367-77105286	5	MBP
chr4:105424195-105453642	100	PPA2
chr22:28629551-28646308	28	TTC28
chr9:83678440-83686155	319	UBQLN1
chr10:125038996-125122868	62	CTBP2
chr2:120127687-120175004	84	EPB41L5
chrX:119406459-119410362	117	SLC25A43
chr7:24641715-24668660	48	MPP6
chr7:74219743-74220734	6	LAT2
chr1:44874677-44897444	0	EIF2B3
chr8:89943252-89947892	1	NBN
chr7:4768414-4770252	26	nogene
chrX:76147252-76148456	2	nogene
chr19:52618935-52635153	83	ZNF83
chr7:24623665-24641868	327	MPP6
chr11:120474559-120477526	146	ARHGEF12
chr3:122703741-122708268	33	PARP14
chr17:45475099-45475726	294	PLEKHM1
chr3:100281741-100283811	0	TBC1D23
chr3:49510418-49510819	10	DAG1
chr19:45025328-45025737	428	RELB
chrX:135942878-135947139	4148	nogene
chr9:137716625-137752408	26	EHMT1
chrUn_GL000195v1:19564-20186	0	nogene
chr13:21374527-21374880	47	nogene
chr9:96458378-96458541	62	HABP4
chr4:189855885-189864786	35	nogene
chr11:66639677-66640123	26	RBM4
chr16:89098590-89102759	131	ACSF3
chr10:7797046-7802854	1213	ATP5C1
chr1:12578717-12579412	58	DHRS3

chr4:142429172-142462726	3	INPP4B
chr6:170543600-170549113	487	PSMB1
chr18:21366026-21384403	63	GREB1L
chr17:44794366-44807469	0	GJC1
chr3:42619019-42632823	26	NKTR
chr16:29905787-29906127	731	ASPHD1
chr7:4737317-4741023	27	FOXK1
chr15:42827927-42840433	426	TTBK2
chr11:65503728-65503931	8	nogene
chr11:36227084-36227430	385	LDLRAD3
chr6:144487547-144493456	96	UTRN
chr2:205172210-205245822	28	PARD3B
chr5:74634683-74636498	81	ENC1
chr1:240294821-240392562	33	FMN2
chr2:69345903-69356557	0	GFPT1
chr1:233346441-233355011	62	KIAA1804
chr1:236011920-236017273	26	NID1
chr11:17988596-18008076	295	SERGEF
chr17:81270524-81271024	29	SLC38A10
chr10:28583398-28590832	106	WAC
chr3:195923885-195929081	1	nogene
chr21:42850040-42863596	132	WDR4
chr4:53425871-53444103	370	FIP1L1
chr1:228309098-228309559	0	OBSCN
chr1:213167857-213176492	33	RPS6KC1
chr10:93381218-93389154	257	MYOF
chr5:14270824-14336727	129	TRIO
chr7:4976300-4989177	38	RNF216P1
chr6:129502604-129558166	12	nogene
chr15:71469376-71469758	15	nogene
chr5:65452132-65473952	52	ADAMTS6
chr5:73840479-73840760	270	ARHGEF28
chr20:33677104-33680416	0	E2F1
chr22:47686199-47687206	603	LOC284930
chr13:41316845-41331369	41	NAA16
chr11:74203727-74204445	10	PPME1
chr4:71236575-71255399	200	SLC4A4
chr13:59833106-59916249	25	DIAPH3
chrX:68274737-68299096	73	OPHN1
chr2:10791794-10797757	31	PDIA6
chr1:32627507-32634792	91	ZBTB8OS
chr10:32451591-32453436	1	CCDC7
chr19:11116093-11116998	13	LDLR

chr20:35801490-35858381	47	PHF20
chr2:152575135-152581049	270	FMNL2
chr12:66529830-66596927	25	GRIP1
chr12:76059797-76074239	61	NAP1L1
chr10:68959805-68960249	710	DDX21
chr3:57890040-57896932	43	SLMAP
chr11:9471301-9474633	27	ZNF143
chr1:167765582-167776166	362	MPZL1
chr2:99449335-99464985	174	REV1
chr12:56666203-56670364	152	PTGES3
chr2:239990073-240007370	1008	NDUFA10
chr3:27404829-27424152	0	SLC4A7
chr15:49225204-49292537	114	GALK2
chr5:157518822-157537991	58	ADAM19
chr14:96833466-96860735	1108	VRK1
chr1:243293090-243308177	51	SDCCAG8
chr1:236038103-236048989	24	NID1
chr2:20297552-20327378	5	PUM2
chr17:59353214-59353526	74	YPEL2
chr22:25661575-25690283	24	ADRBK2
chr10:101624744-101676436	93	FBXW4
chr22:37615671-37615935	24	nogene
chr7:105285693-105297533	24	SRPK2
chr10:48401611-48404981	174	MAPK8
chr7:24620051-24680520	84	MPP6
chr5:109713519-109755456	163	MAN2A1
chr14:86028368-86041833	36	nogene
chr1:103565434-103575540	0	AMY2B
chr17:82893546-82911789	114	TBCD
chr8:123077110-123084340	425	TBC1D31
chr13:30255446-30283791	398	KATNAL1
chr5:16763480-16764396	36	MYO10
chr19:45035733-45036167	39	nogene
chrX:115633999-115643508	0	PLS3
chr11:94799389-94800311	146	AMOTL1
chr3:52643247-52648442	0	PBRM1
chr1:151315572-151316509	104	PI4KB
chr19:38119305-38130772	25	SIPA1L3
chr1:30992389-30995220	849	PUM1
chr5:25542353-25542790	3	nogene
chr14:64442053-64449622	41	MTHFD1
chr20:63442405-63446837	25	KCNQ2
chr19:29657778-29664207	0	nogene

chr3:100295086-100296275	0	TBC1D23
chr2:202292224-202292404	85	nogene
chr3:104764378-104859959	1	nogene
chr1:12253721-12258103	27	VPS13D
chr14:20403376-20406400	113	TEP1
chrX:68052539-68073299	28	OPHN1
chr21:42862281-42876767	31	WDR4
chr6:156901375-156935576	14	ARID1B
chr20:48941198-48953790	12	ARFGEF2
chr10:76036007-76058783	56	C10orf11
chr4:22401430-22402799	182	ADGRA3
chr4:142145839-142193195	0	INPP4B
chr6:56981710-56982198	1	BEND6
chr15:80120327-80131293	1	ZFAND6
chr1:115651276-115664268	30	VANGL1
chr10:57991695-58015971	36	nogene
chr12:104348352-104387984	3	TXNRD1
chr6:129539175-129540542	0	nogene
chr7:66554584-66578727	2	GS1-124K5.11
chrX:129319081-129326008	13	nogene
chr7:92511579-92513967	35	PEX1
chr2:134254261-134270550	24	MGAT5
chr9:127444028-127445249	0	ZNF79
chr11:62634820-62639731	0	GANAB
chr14:73170796-73173707	5	PSEN1
chr5:38990948-39003622	208	RICTOR
chr4:7868621-7872080	414	AFAP1
chr22:27894554-27914347	394	PITPNB
chr2:112074911-112089718	3	TMEM87B
chr1:37693629-37693919	23	nogene
chr2:30910947-30932194	23	GALNT14
chr13:29445578-29487935	23	nogene
chr14:51243855-51247220	179	TMX1
chr6:151348710-151353752	23	AKAP12
chr14:75639351-75641898	0	FLVCR2
chr17:64571797-64586170	94	SMURF2
chr15:34915037-34927138	95	AQR
chrX:148661907-148662768	315	AFF2
chr5:132699170-132703619	1	KIF3A
chr2:131052282-131055695	48	FAM168B
chr3:107710451-107733023	50	BBX
chr1:240257944-240438210	8	FMN2
chr16:69776188-69799295	15	WWP2

chr5:180238331-180241155	322	MAPK9
chr1:155415743-155459898	101	ASH1L
chr21:28163601-28228478	7	LOC101927973
chr7:116699070-116700284	157	MET
chr11:110272535-110279756	36	RDX
chr20:36781458-36781947	86	nogene
chr13:30227411-30241086	876	KATNAL1
chr14:49669523-49674427	179	POLE2
chr11:64757778-64758704	31	PYGM
chr17:51263273-51268904	579	UTP18
chr5:133990854-133993018	235	VDAC1
chr17:12081355-12095694	180	MAP2K4
chr4:105424195-105456745	425	PPA2
chr7:94549532-94554575	8	CASD1
chr11:9440454-9442197	247	IPO7
chr3:151116337-151122974	57	MED12L
chr13:23361051-23361520	23	nogene
chr6:30888687-30889430	32	DDR1
chr10:68014025-68044563	18	HERC4
chr3:196885150-196886694	281	SENP5
chr5:179847977-179851424	7	C5orf45
chr12:11839139-11841032	24	ETV6
chr13:30280062-30284383	21	KATNAL1
chr15:67231813-67236820	1319	AAGAB
chr14:105226068-105226760	158	BRF1
chr12:116719762-116723223	5	C12orf49
chr12:132786338-132791293	0	GOLGA3
chr1:151117391-151118139	23	GABPB2
chr3:104817472-104829713	8	nogene
chr11:65434478-65444063	49	nogene
chr6:144537581-144551082	0	UTRN
chr1:155378280-155395553	13	ASH1L
chr12:51745902-51751593	27	SCN8A
chr5:73749836-73780745	8	ARHGEF28
chr13:75560752-75594990	1	UCHL3
chr15:41668827-41669958	1551	MGA
chr10:30336802-30341640	31	MTPAP
chr16:23581053-23587319	3	NDUFAB1
chr7:157354505-157367483	5	DNAJB6
chr8:134812609-134813169	37	nogene
chr2:70259359-70261386	24	PCYOX1
chr7:92294888-92362273	33	ANKIB1
chr13:26573714-26642403	24	WASF3

chr14:70988566-71013202	36	PCNX
chr17:37283266-37289528	115	ACACA
chr10:32623718-32625144	0	nogene
chr2:99412730-99435941	13	REV1
chr9:89328659-89328886	0	SECISBP2
chr15:71242648-71256715	9	THSD4
chrX:68046993-68053810	362	OPHN1
chr16:46615945-46618372	28	SHCBP1
chr15:56142777-56179303	20	RFX7
chrX:135545422-135556300	130	DDX26B
chr19:12928341-12928847	841	FARSA
chr11:118554458-118559864	40	IFT46
chr16:87748672-87762040	633	KLHDC4
chr3:149846010-149895560	5	RNF13
chr11:117234266-117246948	21	RNF214
chr17:51020158-51031722	8	SPAG9
chr11:9429673-9433846	107	IPO7
chr3:31576395-31580096	521	STT3B
chr9:96509672-96565483	142	CDC14B
chr13:24484652-24486305	3	PARP4
chr11:107803000-107815965	165	SLC35F2
chr12:7190887-7191700	0	PEX5
chr17:82459991-82472698	48	NARF
chr5:79439009-79457018	325	HOMER1
chr5:154029488-154034967	227	FAM114A2
chr1:240123178-240211235	1	FMN2
chr9:136680936-136681563	40	nogene
chr1:26877990-26878354	31	nogene
chr20:35801490-35847434	47	PHF20
chr19:1147308-1154402	196	SBNO2
chr1:1804418-1825499	137	GNB1
chr3:23208691-23217312	4	UBE2E2
chr15:90439331-90443478	232	IQGAP1
chr20:61997549-62000721	21	TAF4
chr1:19259017-19260012	51	nogene
chr12:71619314-71620315	0	ZFC3H1
chr12:46239678-46243314	877	SLC38A1
chr5:97668550-97670684	0	LINC01340
chr4:153350063-153397333	0	MND1
chr22:37812886-37813609	96	GCAT
chr6:27126314-27127401	0	nogene
chr11:71438403-71440122	0	nogene
chr9:2191265-2192802	34	SMARCA2

chr9:20715321-20740340	232	FOCAD
chr18:23507984-23519174	0	C18orf8
chr3:185687056-185689627	19	IGF2BP2
chr2:175979809-175995646	145	KIAA1715
chr10:32805014-32824604	17	CCDC7
chr1:235780864-235782087	51	LYST
chr16:68287745-68291661	29	SLC7A6
chr4:39737419-39777810	38	UBE2K
chr6:41891793-41916939	0	USP49
chr1:52493610-52509712	230	ZCCHC11
chr6:82224067-82231842	41	IBTK
chr2:40428472-40430304	1280	SLC8A1
chr19:45357269-45361642	51	ERCC2
chr9:88426381-88468605	29	SPIN1
chr5:32135571-32143880	788	GOLPH3
chr16:67036638-67066798	152	CBFB
chr8:67103036-67137603	21	CSPP1
chr7:66127703-66134374	793	CRCP
chr1:167749055-167765749	1	MPZL1
chr2:27656082-27656762	27	nogene
chr3:195156448-195226856	4	XXYLT1
chr10:179993-221356	415	ZMYND11
chr20:49023050-49025481	0	ARFGEF2
chr11:123083084-123097952	5	CLMP
chr3:172112451-172133546	332	FNDC3B
chr2:8943186-8962618	1	MBOAT2
chr11:65435052-65444059	35	nogene
chr22:37687909-37688992	292	NOL12
chr1:20757165-20773610	435	HP1BP3
chr22:37496448-37588689	766	CARD10
chr10:12081471-12094271	91	DHTKD1
chr9:20881870-20916937	23	FOCAD
chr1:171568246-171571421	25	PRRC2C
chr10:26934412-26935627	13	LINC00202-1
chr13:75560752-75569507	324	UCHL3
chr11:11327085-11341004	81	GALNT18
chr21:45525816-45532148	20	SLC19A1
chr1:227193785-227213219	81	CDC42BPA
chr2:37316236-37317179	354	PRKD3
chr20:21365420-21368590	624	XRN2
chr7:67055834-67083539	165	TYW1
chr10:96884402-96907747	1	LCOR
chr5:693277-770117	2	TPPP

chr12:104288930-104327671	0	TXNRD1
chr16:23102318-23108183	213	USP31
chr5:137985256-137988315	3447	FAM13B
chr7:77778337-77780774	23	RSBN1L
chr5:176289653-176290955	50	SIMC1
chr20:17947485-17951595	119	SNX5
chr7:5736015-5736227	23	nogene
chr11:86022366-86031611	730	PICALM
chr1:8541213-8614686	308	RERE
chr20:19990005-19996842	89	RIN2
chr5:133990854-133993015	40	VDAC1
chr5:38523418-38530666	259	LIFR
chr9:91852320-91883383	20	nogene
chr11:17988596-18010139	192	SERGEF
chr8:103401515-103404861	32	SLC25A32
chr17:37179259-37181356	28	ACACA
chr9:137754170-137758011	99	EHMT1
chr10:49915916-49935142	337	PARG
chr12:120557339-120557682	28	RNF10
chr7:140459006-140459936	3	MKRN1
chr8:130152735-130169067	1146	ASAP1
chr14:103003927-103012188	445	CDC42BPB
chr7:39997493-40002031	234	CDK13
chr12:69257731-69262562	1	CPSF6
chr12:11839139-11853561	11	ETV6
chr16:70530033-70530414	64	nogene
chr6:43024984-43026141	161	RRP36
chr10:12117672-12120267	18	DHTKD1
chr4:151454700-151458433	3	FAM160A1
chr6:136064380-136071295	3	nogene
chr12:121293199-121294042	97	nogene
chr8:119795531-119797846	20	TAF2
chr12:32305696-32306122	91	BICD1
chr1:39231181-39258028	52	MACF1
chr17:50982523-50985778	53	SPAG9
chr3:142736378-142762313	149	TRPC1
chr18:24366891-24377535	99	OSBPL1A
chr14:89931540-89932627	101	EFCAB11
chr9:40993252-41011730	122	FRG1HP
chr17:37019004-37031685	54	AATF
chr3:27344271-27356904	0	NEK10
chr7:2364371-2367045	102	EIF3B
chr8:67105904-67116122	231	CSPP1

chr1:155216383-155236469	89	GBAP1
chr11:769087-769661	146	nogene
chr17:3818262-3822052	21	C17orf85
chr4:1238182-1241519	103	CTBP1
chr1:212863724-212885396	59	FLVCR1
chr13:23836239-23886506	227	MIPEP
chr10:103439745-103440850	126	PDCD11
chr2:199368604-199433514	197	SATB2
chr4:53145332-53186486	20	SCFD2
chr17:37248010-37253036	73	ACACA
chr6:83398366-83407901	548	ME1
chr18:36501931-36512543	42	FHOD3
chr9:14602717-14680162	20	ZDHHC21
chr19:7981082-7991831	20	ELAVL1
chr9:112078537-112080165	33	SUSD1
chr21:32694227-32695282	68	SYNJ1
chr8:492458-494597	112	TDRP
chr19:46918487-46937408	18	ARHGAP35
chr2:202952558-202955758	14	CARF
chr7:65726770-65734376	2	LOC441242
chr8:53756554-53801896	0	ATP6V1H
chr4:182874569-182915575	120	DCTD
chr1:246726891-246744125	30	SCCPDH
chr2:44209209-44218537	92	PPM1B
chr20:21326278-21368590	43	XRN2
chr12:22484696-22490218	24	C2CD5
chr3:143985542-143989832	81	C3orf58
chr16:53307680-53319951	24	CHD9
chr12:100097364-100103951	86	UHRF1BP1L
chr8:123231299-123239144	128	ZHX1-C8orf76
chr1:154175307-154175914	65	nogene
chr15:78891483-78892313	0	MORF4L1
chr22:27910963-27914347	156	PITPNB
chr15:51781043-51782828	45	TMOD2
chr7:128677563-128680149	0	FAM71F2
chr1:174275832-174394145	48	RABGAP1L
chr14:63998913-64000719	9	SYNE2
chr13:110622215-110627547	131	CARKD
chr14:67685014-67690421	42	RDH11
chr15:32857014-32902040	2	FMN1
chr19:41248513-41248820	965	AXL
chr8:142343965-142345892	116	TSNARE1
chr3:17508473-17586557	15	TBC1D5

chr12:108658737-108662158	77	CORO1C
chr19:19465339-19465614	49	GATAD2A
chr7:156762133-156763795	0	LMBR1
chr9:100498764-100516771	430	TMEFF1
chr17:28720772-28722882	12	nogene
chr9:88426381-88475513	63	SPIN1
chr11:108235669-108249102	24	ATM
chr5:108959224-109047198	0	FER
chr12:123630168-123630533	21	EIF2B1
chr11:63952237-63952839	20	NAA40
chr4:124641877-124651784	4	nogene
chr1:28459084-28460292	10	PHACTR4
chr15:42260957-42268032	184	TMEM87A
chr7:73465438-73466401	28	BAZ1B
chr1:43359166-43359638	41	CDC20
chr7:105459167-105465414	148	PUS7
chr11:65505186-65505375	1	nogene
chr20:37047052-37068186	27	RBL1
chr22:45689711-45740538	176	ATXN10
chr9:125323797-125326589	0	GAPVD1
chr12:49129499-49186458	647	TUBA1B
chr15:72563393-72570307	9	ARIH1
chr11:63813704-63817959	24	nogene
chr7:24830767-24842413	25	OSBPL3
chr16:47497398-47515601	1349	PHKB
chr8:124508473-124516030	73	TATDN1
chr2:63378385-63571626	20	WDPCP
chr8:93786222-93799758	58	TMEM67
chr2:113444963-113454778	23	CBWD2
chr3:133623090-133624175	15	TOPBP1
chr14:103773612-103797518	40	PPP1R13B
chr18:70086612-70128546	38	RTTN
chr1:226265532-226266332	34	LIN9
chr1:31911695-31919658	1	PTP4A2
chr7:102813276-102822203	48	FBXL13
chr2:219146679-219158362	1	NHEJ1
chr4:139116648-139168042	1	nogene
chr4:128074459-128077962	325	LARP1B
chr18:74781427-74890038	11	ZNF407
chr7:6432836-6436533	300	DAGLB
chr19:14458030-14458375	174	PKN1
chr17:39684596-39686019	52	PGAP3
chr14:105252506-105272894	153	BRF1

chr3:172773902-172783909	81	ECT2
chr9:127523678-127531778	2	FAM129B
chr20:1372075-1375603	78	FKBP1A
chr6:83807293-83817736	0	nogene
chr3:123089167-123092442	113	PDIA5
chr22:46231791-46232239	4	PPARA
chr14:52598271-52600341	6	GPR137C
chr17:62524235-62553755	19	TLK2
chr11:18291441-18292019	120	HPS5
chr11:78469246-78571444	29	NARS2
chr19:55047386-55048763	46	RDH13
chr3:10034468-10036339	0	FANCD2
chr10:101797979-101800400	0	MGEA5
chr9:22301599-22308883	13	nogene
chr3:125848229-125857101	7	nogene
chr5:170878096-170896115	664	RANBP17
chr1:178283563-178300118	64	RASAL2
chr9:3330258-3346764	82	RFX3
chr4:1730886-1735834	19	TACC3
chr1:155370503-155395553	15	ASH1L
chr15:65702304-65703008	2	DENND4A
chr1:61899582-61901459	4	INADL
chr20:13483226-13587370	20	TASP1
chr14:57229733-57247712	37	EXOC5
chr4:1311978-1322503	53	MAEA
chr3:123647223-123649194	3	MYLK
chr22:40519463-40522435	1	nogene
chr14:88839451-88861332	7	TTC8
chr1:225167938-225206179	10	DNAH14
chr20:58667489-58673711	109	STX16
chr2:48474687-48491170	0	PPP1R21
chr16:29808229-29808741	0	MAZ
chrX:65075473-65075912	374	nogene
chr6:30034907-30035983	153	ZNRD1-AS1
chr9:7015852-7049200	5	KDM4C
chr1:47335544-47335858	69	nogene
chr4:6923372-6924111	425	TBC1D14
chr6:10400447-10402610	6	TFAP2A
chr7:128842574-128843577	20	FLNC
chr5:68226289-68227009	118	PIK3R1
chr9:85669484-85712566	522	AGTPBP1
chr20:56373407-56388202	19	AURKA
chr12:2820085-2821792	110	ITFG2

chr17:45275390-45275871	69	nogene
chr3:123124065-123124343	19	PDIA5
chr1:231537212-231542611	295	TSNAX-DISC1
chr4:105446382-105456745	347	PPA2
chr6:45422592-45512407	41	RUNX2
chr3:172251259-172251541	21	FNDC3B
chr1:84179176-84214317	176	PRKACB
chr4:79977620-79984863	46	ANTXR2
chr3:148994141-149009402	37	GYG1
chr9:94923985-94979427	1	C9orf3
chr2:15488893-15511350	361	NBAS
chr4:37902002-37902512	7	TBC1D1
chr1:180397515-180413471	351	ACBD6
chr12:125073875-125103081	0	AACS
chr10:70213462-70218817	31	PPA1
chr3:5170502-5174414	152	ARL8B
chr1:50705785-50801677	0	FAF1
chr9:36351114-36353331	52	RNF38
chr6:148471416-148474222	1	SASH1
chr14:30643315-30653588	4	SCFD1
chr4:70762518-70789592	19	RUFY3
chr17:76312869-76313891	173	PRPSAP1
chr12:57533952-57534452	42	DCTN2
chr20:32917300-32931336	0	nogene
chr10:101790895-101791439	1	MGEA5
chr2:173358711-173359491	233	CDCA7
chr1:21050865-21089213	375	EIF4G3
chr17:80325029-80328477	6	RNF213
chr1:77631849-77641655	90	ZZZ3
chr10:3118781-3120044	48	PFKP
chr5:43292473-43298975	12	HMGCS1
chr1:235465635-235484515	108	B3GALNT2
chr11:107433633-107439183	29	CWF19L2
chr5:10377797-10394785	35	6-Mar
chr1:47368468-47373107	56	CMPK1
chr17:1799856-1801024	42	SMYD4
chr19:41252350-41256611	104	AXL
chr7:66127703-66130842	19	CRCP
chr9:127344234-127345477	61	GARNL3
chr17:36557082-36560871	75	GGNBP2
chr5:102865822-102867393	48	PAM
chr2:99462495-99464985	633	REV1
chr3:39408605-39410999	56	RPSA

chr7:105285008-105297533	151	SRPK2
chr3:14944673-14944903	83	nogene
chr17:39724725-39725853	5	ERBB2
chr1:231370561-231374099	427	EGLN1
chr16:30924009-30927857	19	nogene
chrM:5209-5368	107	nogene
chr10:121913789-121924329	10	ATE1
chr7:2437803-2443746	169	nogene
chr6:57507386-57537625	316	PRIM2
chr7:74356409-74360278	0	CLIP2
chr16:88027482-88038011	1346	BANP
chr3:15212103-15220981	3	CAPN7
chr15:101235081-101235577	1134	CHSY1
chr1:97679094-97740479	36	DPYD
chr7:18644670-18648683	6	HDAC9
chr16:71927613-71928250	0	IST1
chr3:183643479-183651276	24	KLHL24
chr7:156796388-156826744	82	LMBR1
chr12:49097565-49098105	139	LMBR1L
chr5:109781395-109789527	210	MAN2A1
chr19:17152643-17154415	31	MYO9B
chr8:98249255-98287868	21	nogene
chr5:138555021-138555724	146	nogene
chr19:44903046-44903470	43	nogene
chr3:141278341-141287453	40	PXYLP1
chr17:3026007-3027070	51	RAP1GAP2
chr12:49129241-49185990	18	TUBA1B
chr4:1916870-1918623	86	WHSC1
chr10:27142386-27145590	76	YME1L1
chr13:20891829-20892174	19	nogene
chr6:82957311-83038485	28	UBE3D
chr6:72647931-72648904	196	nogene
chr3:155828181-155842619	69	SLC33A1
chr5:94909250-94912976	9	MCTP1
chr22:37751771-37759264	1	TRIOBP
chr1:215575900-215579137	107	KCTD3
chr17:30843912-30844916	20	ATAD5
chr15:25405460-25415842	323	UBE3A
chr7:24620051-24668660	440	MPP6
chrX:131709497-131794466	72	FIRRE
chr16:56939236-56940245	20	HERPUD1
chr18:7754372-7774271	17	PTPRM
chr3:111112029-111126335	217	PVRL3

chr4:139060323-139073567	48	ELF2
chr12:1754257-1754514	15	ADIPOR2
chr5:115866669-115913513	18	AP3S1
chr16:346147-347106	190	AXIN1
chrX:108665506-108681888	0	COL4A5
chr1:240206798-240211235	364	FMN2
chr9:122890311-122890545	0	RC3H2
chr17:59737452-59773885	19	VMP1
chr1:227139575-227254155	0	CDC42BPA
chr10:125880542-125881071	0	DHX32
chr8:1349416-1349649	18	nogene
chr10:12081471-12101181	79	DHTKD1
chr22:41274599-41281082	204	RANGAP1
chr1:173774304-173775842	12	KLHL20
chr11:61414419-61414998	23	nogene
chr11:59658865-59659469	270	PATL1
chr19:16081912-16101409	132	TPM4
chr16:11886470-11887750	18	GSPT1
chr16:30713207-30713711	1	SRCAP
chr5:146160373-146161564	47	LARS
chr11:29037741-29211765	29	nogene
chr2:11221197-11224460	18	ROCK2
chr3:128795359-128813471	33	RAB7A
chr2:197078702-197090032	47	ANKRD44
chr22:45689711-45702847	348	ATXN10
chr7:18629349-18666476	1	HDAC9
chr8:124512347-124513024	21	nogene
chr9:4823547-4860901	29	RCL1
chr17:80730559-80754185	0	RPTOR
chr19:43747632-43747897	0	SMG9
chr12:111932585-111943369	14	TMEM116
chr5:140700818-140702129	0	ZMAT2
chr6:158580939-158589782	596	TMEM181
chr13:29550203-29560349	61	nogene
chr21:37420298-37421866	36	DYRK1A
chr2:227898181-227907252	45	DAW1
chr1:109210795-109211119	18	nogene
chrX:17473060-17477320	34	nogene
chr7:80789305-80810701	1131	SEMA3C
chr10:50460672-50590247	9	SGMS1
chr1:94458606-94480606	54	ABCD3
chr21:37420298-37430400	148	DYRK1A
chrX:118584734-118590302	0	DOCK11

chr15:84637346-84641262	49	SCAND2P
chr2:60768469-60770511	8	PAPOLG
chr14:21357196-21361213	248	SUPT16H
chr1:233054267-233057290	50	PCNXL2
chr7:579255-596304	20	PRKAR1B
chr3:44392854-44396834	1	TCAIM
chr1:1387234-1390865	10	CCNL2
chr21:10338454-10340478	6	nogene
chr16:75603084-75622464	34	ADAT1
chr11:65505301-65505606	2	nogene
chr7:92294888-92371607	28	ANKIB1
chr7:11051611-11062085	20	PHF14
chr12:104019629-104021491	46	GLT8D2
chr3:142743484-142748460	0	TRPC1
chr7:26684735-26690362	1	SKAP2
chr4:77040620-77066405	18	CCNI
chr14:45008912-45027474	18	FAM179B
chr2:26278613-26280115	18	HADHB
chr19:5727267-5739439	38	CATSPERD
chr8:61548070-61556022	23	ASPH
chr17:1636708-1637062	2	SCARF1
chr3:198063717-198072830	21	ANKRD18DP
chr13:45207423-45252970	23	GTF2F2
chr1:100049908-100069685	214	HIAT1
chr19:19482367-19483651	39	nogene
chr3:52943352-52969258	25	SFMBT1
chr10:25466650-25572887	0	GPR158
chr10:1084402-1105267	169	WDR37
chr20:47276313-47287284	19	ZMYND8
chr9:137752330-137762820	7	EHMT1
chr14:54966474-54981696	43	WDHD1
chr4:52626017-52631144	115	USP46
chr8:124504270-124523002	30	TATDN1
chr12:111932585-111938210	0	TMEM116
chr19:44429057-44432366	6	ZNF229
chr21:33750142-33761986	6	ITSN1
chr10:97385165-97388624	23	RRP12
chr8:120453846-120461255	103	MTBP
chr11:107510886-107532406	36	ALKBH8
chr9:125543058-125585727	1	MAPKAP1
chr2:106112645-106129778	23	UXS1
chr3:146121111-146124229	2342	PLOD2
chr6:87215902-87216377	0	ZNF292

chr7:141636959-141641908	21	AGK
chr4:80287336-80301716	5	nogene
chr1:145747994-145771919	2	RNF115
chr3:182884752-182887713	10	ATP11B
chrX:2408744-2425304	78	DHRSX
chr7:22455179-22455879	259	nogene
chr10:67992198-68014186	14	HERC4
chr1:32798170-32798589	21	nogene
chr19:2271384-2273160	0	OAZ1
chr14:54748814-54764540	47	SAMD4A
chr5:78020691-78101025	23	AP3B1
chr1:235470849-235494828	198	B3GALNT2
chr14:20325538-20329373	116	CCNB1IP1
chr1:26446315-26447660	305	DHDDS
chr14:50267752-50303017	25	L2HGDH
chrX:152553158-152554491	0	nogene
chr14:73249983-73255018	15	PAPLN
chr13:32648753-32652007	85	PDS5B
chr20:9337125-9339037	219	PLCB4
chr1:162576776-162581459	72	UAP1
chr2:61178980-61186497	33	AHSA2
chr18:59466738-59480238	28	CCBE1
chr18:12353004-12356831	65	AFG3L2
chr5:140485588-140505869	25	ANKHD1-EIF4EBP3
chr3:32708669-32727870	0	CNOT10
chr20:49067189-49072697	23	CSE1L
chr7:92123119-92131872	117	CYP51A1
chr7:158773340-158793726	1	ESYT2
chr1:59321535-59457076	21	FGGY
chr6:83346172-83407901	93	ME1
chr16:75232426-75233128	24	nogene
chr11:59639047-59643035	24	PATL1
chr19:40783777-40786978	1	RAB4B-EGLN2
chr14:67761365-67762819	10	ZFYVE26
chr5:69269206-69277226	0	CDK7
chr3:37017505-37028932	20	MLH1
chr15:57282444-57292046	20	TCF12
chr6:118511297-118566316	93	CEP85L
chr2:29133654-29145367	61	CLIP4
chr12:1083163-1116033	0	ERC1
chr17:15503097-15506986	8	nogene
chr19:47677603-47678210	33	nogene
chr14:30670255-30675065	298	SCFD1

chr18:58709377-58715967	18	MALT1
chr10:32908367-32910455	143	ITGB1
chr13:23365165-23371165	345	SACS
chr9:96320997-96324169	15	SLC35D2
chr16:18874220-18874831	48	nogene
chr7:93862514-93862717	0	nogene
chr15:49225204-49308777	39	nogene
chr20:35801490-35863400	5	PHF20
chr1:246999347-247000074	57	ZNF695
chr10:17688026-17696869	24	STAM
chr10:72152868-72213331	0	ASCC1
chr4:77056248-77066405	26	CCNI
chr6:158583953-158589782	652	TMEM181
chr12:56603358-56605327	65	BAZ2A
chr20:30388452-30393655	27	FRG1BP
chr8:33453215-33461725	32	FUT10
chr15:87406362-87422036	18	nogene
chr10:26704676-26705394	18	PDSS1
chr16:18782780-18788142	55	RPS15A
chr6:42603587-42606651	284	UBR2
chr5:132883339-132885119	53	AFF4
chr2:72731006-72741469	10	EXOC6B
chr12:26722396-26725765	0	ITPR2
chr6:17771907-17783701	20	KIF13A
chr6:98899267-98917719	0	FBXL4
chr6:100715461-100805880	20	ASCC3
chr12:14436105-14447053	20	ATF7IP
chr10:86838864-86876085	205	BMPR1A
chr1:113908008-113908191	3	DCLRE1B
chr2:72559452-72575668	49	EXOC6B
chr9:41008880-41011730	62	FRG1HP
chr10:863742-885760	182	LARP4B
chr6:149826263-149853160	86	LRP11
chr2:31917925-31943383	211	MEMO1
chr3:52739428-52741499	34	NEK4
chr20:35690412-35698590	0	NFS1
chr6:42888899-42889227	65	nogene
chr15:100819413-100832074	0	nogene
chr9:20582456-20586817	0	nogene
chrX:68096869-68194498	5	OPHN1
chr6:36515337-36521817	2	STK38
chr4:153292981-153296036	6	TRIM2
chr16:3308312-3308836	7	ZNF75A

chr4:139388509-139389132	18	nogene
chrX:24065997-24068108	2	EIF2S3
chr13:29514352-29524253	46	SLC7A1
chr9:134727265-134732127	20	COL5A1
chr1:211787631-211793190	18	LPGAT1
chr8:47407860-47440542	145	SPIDR
chr3:52743351-52766375	19	NEK4
chr11:18564891-18578808	40	UEVLD
chr20:34408650-34413879	18	ITCH
chr13:30283615-30283791	202	KATNAL1
chr11:61902441-61904882	53	RAB3IL1
chr8:47291032-47294030	134	SPIDR
chr18:59466738-59469607	60	CCBE1
chr2:60786946-60787620	17	PAPOLG
chr16:84740308-84745673	569	USP10
chr4:110105496-110198367	0	ELOVL6
chr7:24623662-24641868	163	MPP6
chr3:65974452-65976174	0	nogene
chr20:51349940-51396876	17	nogene
chr7:66427948-66439304	127	nogene
chr1:1223243-1223968	734	SDF4
chr17:4282797-4296814	310	UBE2G1
chr2:96733518-96734526	8	LMAN2L
chr12:6730401-6730820	4	COPS7A
chr5:175492094-175526793	0	SFXN1
chr8:70594505-70598255	3	TRAM1
chr14:34551713-34554912	114	nogene
chr1:94487541-94506642	0	ABCD3
chr11:70337364-70339306	20	PPFIA1
chr7:851383-853618	0	SUN1
chr10:74314666-74398579	98	ADK
chr11:9287701-9295387	6	TMEM41B
chr13:19959957-20036909	28	ZMYM2
chr9:109050282-109050692	185	TMEM245
chr12:6869257-6869773	97	TPI1
chr1:54875092-54876047	4	DHCR24
chr7:135636330-135645018	50	NUP205
chr19:49844315-49844892	9	PTOV1-AS1
chr10:63566337-63599283	1	REEP3
chr5:179560024-179577136	20	RUFY1
chr15:67164894-67170604	114	SMAD3
chr16:74457873-74459789	73	GLG1
chr8:79917977-79919148	30	MRPS28

chr5:95755395-95788858	0	RHOBTB3
chr8:134509618-134521001	102	ZFAT
chr14:74669648-74671483	104	AREL1
chr1:243512323-243573048	5	AKT3
chr16:74353776-74360891	60	LOC283922
chr7:50669721-50756056	38	GRB10
chr7:66155767-66156065	29	nogene
chr6:34237203-34243518	18	HMGA1
chr7:22266963-22318037	172	RAPGEF5
chr6:99503764-99510230	110	USP45
chr10:32451591-32481782	100	CCDC7
chr18:10534465-10550254	0	NAPG
chr3:104817472-104859959	104	nogene
chr1:155438326-155439068	410	ASH1L
chr21:22041219-22096586	14	LOC101927843
chr9:96774787-96776245	35	ZNF510
chr2:32414768-32416163	30	BIRC6
chr15:28265617-28280287	17	HERC2
chr10:13127744-13136897	73	OPTN
chr4:120754552-120785091	0	PRDM5
chr17:18330554-18348440	5	SHMT1
chr3:192727180-192738983	18	FGF12
chr1:225167938-225207220	1	DNAH14
chr16:58722118-58723902	1	GOT2
chr3:4445496-4453049	13	SUMF1
chr21:17594053-17609152	18	BTG3
chr1:44753254-44753699	19	KIF2C
chr1:58714831-58718748	104	nogene
chr2:86065273-86081706	27	POLR1A
chr12:64016949-64043575	17	SRGAP1
chr15:65700543-65701890	3	DENND4A
chr8:41974703-41978777	28	KAT6A
chr6:34821652-34821955	3	UHRF1BP1
chr21:36247516-36248568	358	DOPEY2
chr7:50674435-50703908	17	GRB10
chr9:105721536-105722621	145	TMEM38B
chr16:2075798-2080377	107	TSC2
chr1:35405022-35408159	21	ZMYM4
chr2:75670137-75672016	11	GCFC2
chr22:25375812-25381578	454	LRP5L
chr7:6418963-6419876	21	nogene
chr5:179560024-179569425	20	RUFY1
chr4:142305457-142403054	0	INPP4B

chr2:208345110-208348023	28	PIKFYVE
chr19:50398850-50399484	328	POLD1
chr17:39490556-39509761	0	CDK12
chr14:45246742-45247377	17	MIS18BP1
chr2:238275742-238277955	33	PER2
chr20:63672555-63674093	80	RTEL1
chr16:98144-100509	99	NPRL3
chr21:39224407-39229436	40	BRWD1
chr1:61990167-62051058	23	INADL
chr8:127851933-127890998	17	PVT1
chr19:14177530-14189689	20	ADGRL1
chr2:86541116-86542312	11	CHMP3
chr19:40733159-40733364	2	ITPKC
chr8:118016806-118072777	20	nogene
chr2:202275112-202284481	28	NOP58
chr10:77982153-77984298	45	POLR3A
chr6:130175840-130216616	2	SAMD3
chr7:85036888-85068284	57	SEMA3D
chr8:73688653-73688813	20	STAU2
chr22:37768073-37769375	0	TRIOBP
chr4:176708968-176729746	34	VEGFC
chr20:63775677-63790790	1215	ZBTB46
chr15:80098415-80121820	26	ZFAND6
chr19:13808835-13809237	0	ZSWIM4
chr12:1115865-1190052	28	ERC1
chr3:38183002-38198863	42	OXSR1
chr19:40583397-40583717	91	SHKBP1
chr18:68836748-68874785	7	CCDC102B
chr3:172226870-172251541	100	FNDC3B
chr6:154773988-154795139	366	SCAF8
chr6:47503279-47504758	249	CD2AP
chr17:2701612-2702057	19	CLUH
chr5:131698916-131709272	62	FNIP1
chr4:150435588-150436864	21	LRBA
chr9:132013163-132014467	4	MED27
chr12:77940318-78059115	65	NAV3
chr20:36665045-36682578	31	NDRG3
chr1:10403070-10408140	21	PGD
chr14:88574692-88575940	21	ZC3H14
chr16:53873785-53888951	654	FTO
chr6:80200933-80203212	34	BCKDHB
chr4:113174415-113199109	35	ANK2
chr10:12081471-12084751	31	DHTKD1

chr16:57797704-57798022	9	KIFC3
chr3:126727077-126733222	154	CHCHD6
chr8:123077110-123109620	265	TBC1D31
chr5:153997802-154002969	26	FAM114A2
chr9:36369718-36390616	5	RNF38
chr7:83466481-83490274	7	SEMA3E
chr22:31589446-31602785	0	SFI1
chr17:79077429-79077864	239	ENGASE
chr11:3705199-3712728	0	NUP98
chr2:171925536-171953001	499	HAT1
chrX:53406967-53408091	17	nogene
chr17:6525355-6590630	20	PITPNM3
chr2:189791789-189818180	472	PMS1
chr9:22451063-22467906	2	DMRTA1
chr13:24249476-24257409	49	SPATA13
chr2:29121373-29167540	27	CLIP4
chr5:139600371-139623461	48	UBE2D2
chr9:124971369-124976186	19	SCAI
chr16:11894953-11898035	191	GSPT1
chr16:53314376-53315044	6	CHD9
chr20:49066191-49070297	72	CSE1L
chr4:186639721-186663613	20	FAT1
chr7:155301565-155302846	0	INSIG1
chr6:136694139-136720589	37	MAP3K5
chr15:55223888-55234956	104	RAB27A
chr2:226864603-226914351	339	RHBDD1
chr4:139523353-139529220	1	SETD7
chr4:7004843-7014557	46	TBC1D14
chr21:42850040-42859722	164	WDR4
chr9:3484973-3490345	3	RFX3
chr13:49921477-49928085	0	SPRYD7
chr20:44978442-44997306	38	STK4
chr2:28906459-28914208	194	WDR43
chr2:175111567-175121540	229	ATF2
chr11:78196235-78200808	0	USP35
chr18:21621075-21621672	26	nogene
chr8:109271004-109281172	17	NUDCD1
chr6:18236451-18237516	118	DEK
chr5:171390050-171392978	140	NPM1
chr1:173941264-173943615	12	RC3H1
chr16:29895258-29895860	241	SEZ6L2
chr2:106122969-106129778	96	UXS1
chr10:37729820-37749758	67	nogene

chr1:5967298-5969259	0	NPHP4
chr1:224952669-224974153	1000	DNAH14
chr5:131395562-131417177	2	nogene
chr8:140617537-140618050	34	nogene
chr10:12172764-12173813	19	NUDT5
chr18:54271359-54287411	47	POLI
chr12:98527885-98546447	20	TMPO
chr2:178591138-178600667	139	TTN-AS1
chr1:12290997-12308641	0	VPS13D
chr9:34096715-34098517	14	DCAF12
chr14:54984722-55002185	39	WDHD1
chr19:19640084-19640571	78	GMIP
chr17:42500706-42501304	92	ATP6V0A1
chr7:35010467-35018596	89	DPY19L1
chr3:172760160-172783909	57	ECT2
chr17:64571797-64583544	29	SMURF2
chr3:43299753-43303792	72	SNRK
chr2:148021647-148342336	21	MBD5
chr8:127989161-128010444	169	PVT1
chr15:76665652-76680226	0	SCAPER
chr3:12379703-12392752	0	PPARG
chr11:78465875-78493195	242	NARS2
chr3:27322176-27356904	0	NEK10
chr18:12999420-13017336	0	CEP192
chr7:29923955-29969068	24	SCRN1
chr12:7403534-7406852	1	CD163L1
chr6:44932652-44954607	28	SUPT3H
chr7:27629370-27632445	220	HIBADH
chr13:50927406-50949505	207	RNASEH2B
chr6:157896826-157910025	29	SNX9
chr19:16081912-16086540	121	TPM4
chr17:51263273-51273450	11	UTP18
chr8:42352981-42353203	48	nogene
chr7:74689123-74700634	0	GTF2I
chr15:90466000-90467592	6	IQGAP1
chr1:154170399-154176248	5	TPM3
chr5:126804775-126832801	55	LMNB1
chr14:70988569-70995925	11	PCNX
chr6:121255327-121256285	1	TBC1D32
chr12:50427761-50441643	90	LARP4
chr6:84556018-84602487	2	nogene
chr12:24829822-24836596	57	BCAT1
chr10:26705285-26720359	19	PDSS1

chr7:130201849-130204110	52	TMEM209
chr1:15534236-15567269	1	DNAJC16
chr3:161234726-161238826	27	NMD3
chr2:231206197-231208252	0	ARMC9
chr8:61637946-61644632	2	ASPH
chr3:189207375-189238909	24	TPRG1
chr1:66891153-66905375	81	WDR78
chr15:100973803-100989398	1	LRRK1
chr5:32390274-32400203	0	ZFR
chr15:41696074-41699159	467	MGA
chr10:113898109-113908523	28	NHLRC2
chr18:32111753-32126800	131	RNF138
chr6:85572136-85574378	1	SNX14
chr2:175979809-175994280	36	KIAA1715
chr11:76976288-76998821	19	ACER3
chr12:31442774-31447769	132	DENND5B
chr18:36730645-36747135	27	FHOD3
chr2:10671553-10684612	19	NOL10
chr16:11846500-11847806	372	RSL1D1
chr17:64519320-64526200	3	CEP95
chr1:23030468-23059167	120	KDM1A
chr1:214390987-214398001	0	PTPN14
chrX:71381742-71387461	114	TAF1
chr5:171398051-171398493	20	nogene
chr7:155665174-155678684	16	RBM33
chr6:34606554-34606904	148	C6orf106
chr17:19542392-19548133	64	SLC47A1
chr8:104068511-104148848	57	nogene
chr12:132747831-132755133	41	ANKLE2
chr4:150102477-150102812	186	DCLK2
chr22:19383619-19410778	17	HIRA
chr18:24440495-24445466	0	IMPACT
chr4:41605893-41619440	29	LIMCH1
chr5:170881805-170896115	17	RANBP17
chr2:175947479-175979868	19	KIAA1715
chr17:50723856-50724305	17	nogene
chr8:73673106-73688813	515	STAU2
chr15:61015797-61094611	34	nogene
chr2:233388256-233390483	256	DGKD
chr18:9208656-9221999	86	ANKRD12
chr2:229477139-229547092	17	DNER
chr22:45330602-45332710	16	FAM118A
chr1:153751094-153757763	67	INTS3

chr20:43702489-43702903	38	MYBL2
chr7:5389754-5390115	16	nogene
chr2:10124716-10129154	17	RRM2
chr2:43963587-43975217	85	LRPPRC
chr6:143765260-143765798	31	PHACTR2
chr9:89065528-89077903	2	SHC3
chr7:7877280-7889229	10	UMAD1
chr14:31066125-31072973	172	AP4S1
chr7:129857510-129880971	30	UBE2H
chr17:15977799-15987368	0	ZSWIM7
chr2:148178699-148233395	36	MBD5
chr17:54912665-54915862	59	TOM1L1
chr7:64691247-64691960	19	ZNF107
chr12:40028300-40048210	214	SLC2A13
chr16:533945-536142	179	CAPN15
chr16:48546146-48553669	19	N4BP1
chr15:56960660-56960903	17	nogene
chr5:175940443-175961434	291	THOC3
chr10:97656284-97664984	7	PI4K2A
chr11:64193077-64203622	8	STIP1
chr15:34084208-34088133	17	EMC7
chr9:83343189-83389708	16	FRMD3
chr8:47852672-47855373	51	PRKDC
chr7:44013592-44016523	26	POLR2J4
chr10:69185986-69187756	91	SUPV3L1
chr7:47396799-47415206	16	TNS3
chr5:150942217-150993760	1	ZNF300P1
chr16:85264477-85265058	45	nogene
chr20:2886289-2965202	25	PTPRA
chr10:68142939-68150111	380	MYPN
chr5:138947404-138951884	54	SIL1
chr11:120475339-120477526	24	ARHGEF12
chr6:3410187-3415855	8	SLC22A23
chr18:48757918-48817376	0	CTIF
chr12:48063709-48089293	0	SENP1
chr1:233246426-233252788	25	PCNXL2
chr18:46215810-46216595	70	C18orf25
chr1:240257944-240355908	90	FMN2
chr2:232722553-232724691	51	GIGYF2
chr3:179690240-179708957	86	USP13
chr20:38497352-38509208	40	RALGAPB
chr2:178540106-178542361	13	TTN-AS1
chr2:175111567-175136486	0	ATF2

chr10:37744214-37749758	25	nogene
chr6:56670640-56735289	26	DST
chr4:2655027-2672372	19	FAM193A
chr6:145686121-145721155	0	EPM2A
chr5:163513554-163517674	11	MAT2B
chr12:112039228-112049611	21	NAA25
chr5:97220955-97229457	19	nogene
chr8:98133948-98134659	32	POP1
chr12:40028300-40100089	16	SLC2A13
chr3:196053417-196077122	45	TFRC
chr6:144426286-144429741	16	UTRN
chr9:37126311-37304313	1	ZCCHC7
chr5:42687514-42700002	16	GHR
chr2:186625472-186633374	0	ITGAV
chr7:1687915-1688150	17	nogene
chr1:241506002-241508785	25	FH
chr1:153818790-153819735	92	GATAD2B
chr7:105038125-105041023	162	KMT2E
chr5:10414432-10417404	53	6-Mar
chr12:116236820-116237705	113	MED13L
chr18:21765771-21791557	132	MIB1
chr11:19832483-19892594	28	NAV2
chr2:190514452-190525378	23	NEMP2
chr4:124609508-124651784	0	nogene
chr2:158533179-158625427	23	PKP4
chr2:55581566-55604076	1	PPP4R3B
chr15:43146492-43154831	1	TMEM62
chr10:5773841-5800705	833	GDI2
chr2:11765532-11773745	9	LPIN1
chr20:47262287-47290274	2	ZMYND8
chr10:94030682-94032252	26	PLCE1
chr2:9344531-9374944	28	ASAP2
chr17:47612459-47619164	0	NPEPPS
chr11:77683709-77693611	393	RSF1
chr7:66993216-66993417	4	SBDS
chr7:117099761-117138532	7	ST7
chr1:186893010-186911389	25	PLA2G4A
chrX:2612149-2615969	24	CD99P1
chr4:142305457-142462726	0	INPP4B
chr3:146034531-146035473	4	nogene
chr6:85565331-85574378	48	SNX14
chr6:42663257-42666245	52	UBR2
chr15:42827927-42878684	29	TTBK2

chr2:73419122-73426553	47	ALMS1
chrX:131749305-131785258	191	FIRRE
chr3:20071583-20072459	38	KAT2B
chr12:119803205-119850275	24	CIT
chr14:30580863-30581116	18	G2E3
chr19:18878929-18893575	18	GDF1
chr4:127794076-127798709	24	HSPA4L
chr3:47678197-47693300	1	SMARCC1
chr3:136377659-136423044	54	STAG1
chr19:48981529-48982837	19	GYS1
chr1:30980061-30982640	1	PUM1
chr16:15620450-15623123	2	KIAA0430
chr5:172932435-172935310	197	ERGIC1
chr21:43630321-43644392	74	HSF2BP
chr6:38061592-38082457	9	ZFAND3
chr1:184732836-184737711	5	EDEM3
chr1:28035543-28042650	38	EYA3
chr1:42278347-42323827	33	FOXJ3
chr15:63698727-63716473	16	HERC1
chr6:167864550-167880517	39	MLLT4
chr10:124973644-124974398	2	ZRANB1
chr13:23756544-23886506	34	MIPEP
chr17:2414394-2415954	29	LOC284009
chr5:37125243-37148268	1	C5orf42
chr3:179701007-179708957	77	USP13
chr12:1180539-1190052	49	ERC1
chr16:56328630-56336860	16	GNAO1
chr11:110241595-110242205	19	nogene
chr6:2779262-2784403	56	WRNIP1
chr1:230954332-230961303	257	TTC13
chr8:123023712-123030716	43	DERL1
chr4:150761782-150817257	41	LRBA
chr1:154225083-154228725	4	UBAP2L
chr17:64551583-64571956	23	SMURF2
chr20:34440154-34471515	0	ITCH
chr1:224952669-225023865	14	DNAH14
chr1:151638887-151639119	186	SNX27
chr9:111530874-111534353	2	ZNF483
chr3:121498476-121498670	1	POLQ
chr1:109336239-109345881	6	SORT1
chr5:179594865-179596681	30	RUFY1
chr18:54271359-54274090	85	POLI
chr6:151417278-151436554	0	RMND1

chr18:216485-224179	28	THOC1
chr19:52040487-52047060	31	ZNF432
chr6:56101471-56141983	0	COL21A1
chr12:108652271-108654410	1912	CORO1C
chr7:158773340-158798076	0	ESYT2
chr10:5730486-5731203	1	FAM208B
chr17:75543396-75551404	1	LLGL2
chr10:87878925-87933251	26	PTEN
chr1:30964673-30964910	0	PUM1
chr13:100625534-100670569	0	TMTC4
chr3:142748257-142777763	18	TRPC1
chr3:196368027-196393619	1	UBXN7
chr17:51268836-51280479	38	UTP18
chr5:353729-376716	97	AHRR
chr18:8076454-8143779	106	PTPRM
chr6:109451599-109452615	1	MICAL1
chr5:119525215-119531404	16	HSD17B4
chr20:25418104-25425327	40	GINS1
chr4:150588047-150599131	35	LRBA
chr6:57507386-57601219	16	PRIM2
chr4:66365503-66370916	0	nogene
chr16:29833732-29835798	29	MVP
chr1:162799742-162805492	0	HSD17B7
chr3:160356012-160383504	1	IFT80
chrX:100862170-100863595	0	NOX1
chr19:19383482-19383697	25	nogene
chr5:73832337-73858219	62	ARHGEF28
chr5:157492972-157494795	58	ADAM19
chr2:111833219-111843599	0	ANAPC1
chr12:52073574-52073902	18	ATG101
chr20:56381432-56388202	36	AURKA
chr6:136277864-136282687	27	BCLAF1
chr11:70417012-70419856	18	CTTN
chr2:232014834-232015671	49	DIS3L2
chr5:74745677-74750667	116	GFM2
chr9:92243215-92249941	38	IARS
chr15:90448572-90449643	0	IQGAP1
chr1:10365099-10365648	3	KIF1B
chr17:12054888-12057104	19	MAP2K4
chr11:47625673-47625867	19	MTCH2
chr9:97139852-97211487	18	nogene
chr5:131395562-131415145	0	nogene
chr5:73406444-73406818	18	nogene

chr6:52665273-52667493	16	nogene
chrX:71292453-71293326	27	nogene
chr9:136386699-136388023	26	nogene
chr18:9367487-9388140	78	nogene
chr1:46077514-46080750	6	PIK3R3
chrX:24809897-24888122	37	POLA1
chr4:128992166-129003876	1463	SCLT1
chr17:51031680-51056482	2	SPAG9
chr6:158573419-158589782	662	TMEM181
chr1:25351625-25352974	24	TMEM50A
chr6:99437245-99443662	18	USP45
chrX:101020486-101021315	27	TRMT2B
chr1:97679094-97700302	0	DPYD
chr2:206149019-206153682	4	NDUFS1
chr3:47056820-47062346	30	SETD2
chr10:121551289-121565704	18	FGFR2
chr1:240294821-240334229	27	FMN2
chr10:38159635-38177975	23	LOC100129055
chr12:124372021-124372610	18	NCOR2
chr7:130046627-130046988	28	nogene
chr17:1354210-1355864	19	YWHAE
chr2:108729131-108740681	45	RANBP2
chr11:134194015-134194738	19	NCAPD3
chr12:56213957-56216613	16	RNF41
chr2:203380207-203417081	49	ABI2
chrX:78014661-78020398	77	ATP7A
chr11:686857-688460	0	DEAF1
chr19:14042452-14042789	141	IL27RA
chr15:99671322-99675458	57	MEF2A
chr15:55680053-55680628	24	PRTG
chr15:40709024-40728824	134	RAD51
chr9:125915685-125935607	55	PBX3
chr8:98706466-98707311	1220	STK3
chr5:145703974-145765000	26	PRELID2
chr1:92624175-92636336	58	EVI5
chr6:100715461-100725703	225	ASCC3
chr9:34269256-34286704	8	KIF24
chr17:45096193-45097244	1	NMT1
chr15:42810613-42840433	106	TTBK2
chr4:39922621-39928164	18	PDS5A
chr2:170015280-170040985	1	UBR3
chr6:107098550-107099296	32	BEND3
chr13:24499300-24501834	54	PARP4

chr8:42861744-42887871	97	RNF170
chr1:173941264-173943588	9	RC3H1
chr1:92736397-92736627	0	EVI5
chrX:80289663-80310233	140	CHMP1B2P
chr15:51535662-51547408	32	DMXL2
chr14:96555846-96556413	8	PAPOLA
chr20:37203884-37210165	16	RPN2
chr9:15744272-15848947	21	CCDC171
chr12:31423596-31442925	0	DENND5B
chr13:45151686-45252970	21	GTF2F2
chr5:179617988-179621035	0	HNRNPH1
chr14:24266429-24268619	146	RABGGTA
chr7:44206384-44208198	21	YKT6
chrX:97072940-97075261	16	DIAPH2
chr19:7938026-7941197	24	TIMM44
chr20:32366383-32391167	165	ASXL1
chr10:26764509-26765318	1	ABI1
chr17:35624396-35627726	5	AP2B1
chr16:3685995-3690985	16	TRAP1
chr6:38577599-38580437	20	BTBD9
chr9:137256328-137257054	0	NELFB
chr5:80146089-80147284	0	SERINC5
chr6:1959866-2124731	0	GMDS
chr2:185729328-185757810	1	LOC101927196
chr8:65669872-65708011	18	MTFR1
chr9:98134805-98137688	0	CORO2A
chr9:113284294-113288264	0	PRPF4
chr10:84417770-84438707	197	CCSER2
chr17:82763964-82781721	962	TBCD
chr9:14639895-14662326	78	ZDHHC21
chr11:70419745-70420510	32	CTTN
chr19:8455404-8467584	24	HNRNPM
chr17:59081096-59088407	36	TRIM37
chr16:28487455-28489386	15	CLN3
chr2:37101922-37104132	25	nogene
chr14:35018691-35019074	3	SRP54
chr3:119500694-119517315	122	TIMMDC1
chr6:57128728-57133192	1	ZNF451
chr11:108673466-108679560	146	DDX10
chr4:82844385-82881940	17	SEC31A
chr10:110100624-110112915	17	ADD3
chr5:111344023-111376942	17	CAMK4
chr4:124500895-124556510	11	LOC101927087

chr16:47463881-47547548	4	PHKB
chr16:70526586-70544533	15	SF3B3
chr11:71147119-71224796	24	SHANK2
chr22:39415005-39416877	18	TAB1
chr10:17733834-17776170	7	TMEM236
chr1:1817836-1839238	40	GNB1
chr11:18283794-18285459	27	HPS5
chr1:243415701-243418076	2	SDCCAG8
chr6:80127546-80171390	29	BCKDHB
chr2:201738669-201744429	11	ALS2
chr14:55151694-55154806	15	DLGAP5
chr11:64274907-64281098	15	nogene
chr7:105200912-105203785	16	SRPK2
chr10:74043082-74074903	15	VCL
chr2:85356966-85377474	0	ELMOD3
chr15:90448572-90456315	10	IQGAP1
chrX:63223732-63259817	13	nogene
chr8:61567167-61584029	15	ASPH
chrX:14796159-14797298	1	nogene
chr5:136147831-136154163	373	SMAD5
chr3:143985542-143985846	141	C3orf58
chr9:113198854-113199963	17	FKBP15
chr11:3683199-3695606	0	NUP98
chr2:173363225-173365592	24	CDCA7
chr3:56670072-56673725	0	FAM208A
chr1:108896863-108901945	21	GPSM2
chr18:77016831-77105286	0	MBP
chr1:176027571-176046324	46	RFWD2
chr16:30197269-30203685	0	SLX1B-SULT1A4
chr1:212872677-212885396	0	FLVCR1
chr1:24440171-24445244	2	NIPAL3
chr21:39177434-39181878	0	PSMG1
chr8:140700890-140752316	26	PTK2
chr3:27448650-27452498	312	SLC4A7
chr15:64499292-64500166	2329	ZNF609
chr13:23862301-23886506	126	MIPEP
chr13:45985296-45989097	23	ZC3H13
chr1:99740792-99749001	26	FRRS1
chr15:40751425-40752178	1	RMDN3
chr4:142260491-142270774	1	INPP4B
chr4:103328124-103343235	17	nogene
chr3:67237400-67241915	19	nogene
chr3:172112451-172226947	18	FNDC3B

chr1:167951799-167975015	55	DCAF6
chr12:1083163-1141787	1	ERC1
chr3:192335360-192402696	4	FGF12
chr6:33776922-33786774	0	LEMD2
chr4:1265339-1265907	157	nogene
chr4:378072-378363	173	nogene
chr5:14678680-14693396	17	OTULIN
chr14:22909482-22913845	0	RBM23
chr1:230943805-230954403	17	TTC13
chr1:10095460-10122076	15	UBE4B
chr2:199380363-199381820	15	SATB2
chr3:195294718-195297281	15	ACAP2
chr3:138570317-138572984	278	CEP70
chr6:84444386-84470904	203	LINC01611
chr17:31966146-31976614	79	SUZ12
chr1:155438326-155440593	0	ASH1L
chr5:160074988-160080770	15	PWWP2A
chr8:47595810-47599196	291	SPIDR
chr2:191693202-191693554	14	LOC105747689
chrX:155415574-155419562	125	nogene
chr1:28274824-28279241	84	SESN2
chr14:21371873-21373430	47	SUPT16H
chr8:124320085-124330792	79	TMEM65
chr2:203245845-203246921	39	CYP20A1
chr3:136586801-136591549	0	nogene
chr14:96833466-96853166	19	VRK1
chr3:58806394-58831889	15	C3orf67
chr8:23566107-23568378	34	SLC25A37
chr1:46055794-46062071	2	PIK3R3
chr7:141596561-141611287	15	AGK
chr7:131338840-131399433	17	MKLN1
chr1:110404970-110405270	31	nogene
chr2:155809927-155811964	17	nogene
chr19:5927967-5941707	15	RANBP3
chr22:40353072-40363071	5	ADSL
chr12:123395046-123396432	28	SETD8
chr5:896664-901431	34	TRIP13
chr15:42794643-42817097	32	TTBK2
chr3:196368027-196403019	116	UBXN7
chr1:77154448-77210489	39	PIGK
chr2:159136193-159150556	77	TANC1
chr17:2377856-2381162	0	SGSM2
chr5:75685206-75702810	47	POC5

chr4:106324984-106331883	17	AIMP1
chr14:102159235-102159536	17	nogene
chr10:68522737-68523199	42	nogene
chr2:232747614-232749082	0	GIGYF2
chr7:131397266-131399433	0	MKLN1
chr9:4860124-4860901	181	RCL1
chr5:176943334-176958154	1227	UIMC1
chr7:96277195-96296951	32	SLC25A13
chr7:75052710-75056074	0	WBSCR16
chr7:140739811-140749418	1	BRAF
chr3:33051757-33068970	7	GLB1
chr19:40616888-40619493	45	LTBP4
chr19:48913010-48913564	81	NUCB1
chr20:37065423-37068186	212	RBL1
chrX:154399486-154400626	15	RPL10
chr11:102205892-102227581	15	YAP1
chr4:77031172-77066405	18	CCNI
chrX:108595508-108597568	31	COL4A5
chr14:31156839-31178254	0	HECTD1
chr12:112269703-112274960	26	HECTD4
chr2:58048826-58089723	154	VRK2
chrX:129032349-129034541	2	nogene
chr1:167765582-167803537	0	MPZL1
chr6:131030966-131031817	18	nogene
chr15:67226900-67236820	29	AAGAB
chr4:147939824-147955374	435	ARHGAP10
chr10:35483403-35516623	38	CCNY
chr2:201854873-201880167	21	CDK15
chr2:37013840-37020836	3	HEATR5B
chr19:6222120-6270759	0	MLLT1
chr15:99799809-99800121	1	nogene
chr1:1868780-1869894	21	nogene
chr14:92017681-92033253	18	TRIP11
chr12:11028400-11047188	80	PRH1
chr6:116689319-116692392	507	KPNA5
chr4:74174505-74201370	55	MTHFD2L
chr17:82756164-82781721	141	TBCD
chr10:31820386-31831800	1	ARHGAP12
chr14:31112415-31114340	24	HECTD1
chr10:6213622-6226365	0	PFKFB3
chr12:100204939-100205757	15	ACTR6
chr18:12337340-12371691	15	AFG3L2
chr10:5096409-5196951	0	AKR1C3

chr10:97256317-97266125	26	ARHGAP19
chr12:26922615-26925851	15	ASUN
chr12:22490122-22506819	72	C2CD5
chr20:35630438-35631777	20	CPNE1
chr2:36387971-36396787	129	CRIM1
chr5:138781922-138783372	74	CTNNA1
chr3:41224020-41227352	196	CTNNB1
chr6:18256360-18258405	304	DEK
chr10:12084539-12101181	20	DHTKD1
chr21:46484756-46504489	0	DIP2A
chr14:102019892-102022880	48	DYNC1H1
chr9:137811460-137818138	101	EHMT1
chr12:22643762-22644022	0	ETNK1
chr5:32121494-32143880	68	GOLPH3
chr6:57919783-57926510	192	GUSBP4
chr6:30234357-30254464	0	HCG17
chr5:55896349-55900132	0	IL31RA
chr17:42114218-42115035	20	KAT2A
chr10:32017142-32018562	31	KIF5B
chr18:6961585-6965432	15	LAMA1
chr7:130941412-130945835	0	LINC-PINT
chr6:149836084-149853160	52	LRP11
chr7:24663589-24666089	37	MPP6
chr22:29970975-29991670	5	MTMR3
chr12:54185810-54186391	55	nogene
chr4:142654586-142729497	5	nogene
chr11:29037741-29063536	8	nogene
chr17:30743300-30745851	52	nogene
chr9:125929654-125935607	79	PBX3
chr2:43726271-43738520	0	PLEKHH2
chr7:677228-711527	331	PRKAR1B
chr9:123010353-123020459	0	RABGAP1
chr9:89325426-89328886	20	SECISBP2
chr12:56346145-56346624	0	STAT2
chr3:4351566-4362254	20	SUMF1
chr9:109106507-109108570	496	TMEM245
chr1:95143890-95151419	489	TMEM56
chr17:40402905-40404883	20	TOP2A
chr7:99004189-99005348	20	TRRAP
chr9:83665029-83677961	131	UBQLN1
chr6:34791835-34792294	20	UHRF1BP1
chr2:43225796-43226264	15	ZFP36L2
chr2:11765532-11783922	10	LPIN1

chr1:35831426-35835993	4	AGO4
chr5:126549928-126554393	17	ALDH7A1
chr11:93675570-93679552	17	CEP295
chr12:1371832-1444750	164	ERC1
chr2:210153494-210154611	34	KANSL1L
chr7:131052424-131108803	0	LINC-PINT
chr6:150876089-150887981	33	MTHFD1L
chrX:12799206-12810146	18	PRPS2
chr9:97428459-97432238	17	TDRD7
chr1:153941230-153942113	17	DENND4B
chr4:102714437-102723967	32	MANBA
chr7:158631643-158646563	18	NCAPG2
chr19:12707295-12709114	17	nogene
chr19:43486265-43487123	1	PHLDB3
chr19:5047475-5082504	276	KDM4B
chr11:29476144-29484062	18	nogene
chr1:222724091-222730177	1	BROX
chr10:124681606-124682379	226	FAM53B
chr15:78470531-78471924	48	IREB2
chr15:42950261-42960701	31	UBR1
chrX:65051461-65075912	54	nogene
chr13:32124285-32124875	1	FRY
chr6:167890861-167898467	0	MLLT4
chr15:61015797-61034926	36	nogene
chr15:56142777-56144483	11	RFX7
chr3:141368644-141404031	15	ZBTB38
chr22:38552665-38568289	47	DMC1
chr1:248844482-248845662	7	ZNF672
chr11:12764178-12764434	171	TEAD1
chr5:180316340-180317058	59	GFPT2
chr17:78414878-78419675	31	PGS1
chr8:99038422-99041418	110	VPS13B
chr8:130187235-130236994	7	ASAP1
chr12:54345459-54348051	0	COPZ1
chr2:131052890-131055695	18	FAM168B
chr14:20395449-20396730	18	TEP1
chr17:40406383-40406942	156	TOP2A
chr16:2002753-2002990	17	ZNF598
chr15:92978233-92979283	0	CHD2
chr2:25596085-25628384	1	DTNB
chrX:131731128-131794466	17	FIRRE
chr11:63749990-63753708	1	RTN3
chr2:148178699-148342336	19	MBD5

chr2:29133654-29157402	7	CLIP4
chr21:28163601-28170143	0	LOC101927973
chr5:80848447-80854316	17	MSH3
chr6:43055544-43056445	1023	MRPL2
chr12:131753122-131766308	52	SFSWAP
chr3:192727180-192754017	19	FGF12
chr18:21765771-21804014	53	MIB1
chr1:197642710-197715074	29	DENND1B
chr14:99850123-99851035	41	EML1
chr19:18539370-18539720	864	FKBP8
chr7:18762156-18793452	1	HDAC9
chr6:7602622-7606195	70	SNRNP48
chr20:44972077-44987296	9	STK4
chr2:159136193-159163546	0	TANC1
chr11:67215339-67228163	6	KDM2A
chr5:140676653-140683219	0	HARS
chr4:147906637-147946704	3	ARHGAP10
chr20:35729311-35732135	437	RBM39
chr1:176144004-176176007	1	RFWD2
chr6:42883450-42886470	94	RPL7L1
chr19:50398847-50399484	42	POLD1
chr2:36916077-36925208	4	STRN
chr17:59731420-59773944	24	VMP1
chr11:65393572-65394425	8	FRMD8
chr12:51049033-51058128	78	LETMD1
chr2:159169249-159170805	28	TANC1
chr5:151797226-151799313	67	G3BP1
chr16:4261778-4262701	138	TFAP4
chr12:49371227-49490731	18	SPATS2
chr17:61889785-61891689	3	INTS2
chr1:240294821-240355908	33	FMN2
chr12:98523893-98524353	30	nogene
chr12:7303277-7317157	8	nogene
chr13:25320529-25338731	17	NUPL1
chr3:66236543-66263379	115	SLC25A26
chr7:42023467-42048696	96	GLI3
chr2:230759958-230793331	24	CAB39
chr9:132642003-132652492	3	DDX31
chr1:8866278-8868057	10	ENO1
chr19:16556922-16557126	0	nogene
chr5:181099848-181100673	23	nogene
chr21:44055697-44063785	0	TRAPPC10
chr21:37420298-37425781	67	DYRK1A

chr13:59969943-59974456	21	DIAPH3
chr12:116833791-116836282	15	RNFT2
chr3:63898398-63952483	148	ATXN7
chr6:116689319-116692156	30	KPNA5
chr17:75321658-75332797	1	GRB2
chr10:101939862-101957732	41	C10orf76
chr3:49044389-49077038	9	QRICH1
chr3:66243202-66263379	120	SLC25A26
chr14:88746184-88754671	11	EML5
chr19:45277922-45280734	65	MARK4
chr21:43734068-43743807	17	PDXK
chr5:127523459-127526778	18	PRRC1
chr6:43533906-43553503	46	XPO5
chr18:76849621-76871825	21	ZNF236
chr1:229463542-229475732	17	NUP133
chr20:25632157-25645648	264	ZNF337-AS1
chr18:12999420-13042334	134	CEP192
chr15:92953356-92955512	0	CHD2
chrX:115465389-115465650	0	nogene
chr11:17992930-18008076	15	SERGEF
chr6:144537581-144577288	0	UTRN
chr3:58118871-58121503	27	FLNB
chr1:31005852-31028864	6	PUM1
chr5:16763480-16766198	55	MYO10
chr3:171123595-171128878	1	TNIK
chr14:74549862-74555693	18	LTBP2
chr8:86494828-86507112	26	RMDN1
chr4:9641070-9641431	278	nogene
chr19:14121531-14126237	1	ASF1B
chr17:83048470-83048785	203	B3GNTL1
chr16:29835703-29836958	5	MVP
chr17:67967007-67967236	16	nogene
chr8:52685398-52686156	20	RB1CC1
chr13:23353784-23356007	34	SACS
chr6:75702644-75711416	112	SENP6
chr4:26716212-26720125	16	TBC1D19
chr17:38306532-38320767	1	MRPL45
chr10:11594630-11597717	10	USP6NL
chr9:20694519-20765073	0	FOCAD
chr12:30661140-30666251	26	IPO8
chr5:181185578-181185906	16	nogene
chr17:82905935-82911789	97	TBCD
chr1:8495062-8557523	79	RERE

chr9:36375933-36390616	2	RNF38
chr12:122477840-122478292	60	ZCCHC8
chr13:29530537-29553860	634	SLC7A1
chr15:72695176-72724655	13	BBS4
chr9:137743370-137758011	20	EHMT1
chr2:232922245-232922597	23	nogene
chr4:147653851-147654566	11	PRMT9
chr17:18340042-18348440	4	SHMT1
chr12:51106524-51117747	1	TFCP2
chr4:84708908-84715383	0	WDFY3
chr2:231202784-231203095	0	nogene
chr7:851383-866067	20	SUN1
chr1:155676547-155680456	96	YY1AP1
chr18:12344131-12371691	1	AFG3L2
chr7:2437803-2438545	25	nogene
chr1:26452298-26452844	18	nogene
chr20:3945176-3974400	150	RNF24
chr2:36877890-36894033	28	STRN
chr1:162566011-162590511	42	UAP1
chr17:39423326-39432016	18	MED1
chr4:143415476-143415771	1	GAB1
chr22:23574214-23639569	32	nogene
chr5:134661118-134661586	55	SEC24A
chr8:124504270-124516030	28	TATDN1
chr9:96719508-96721130	63	nogene
chr17:42727634-42730918	46	EZH1
chr9:6980924-6990524	2	KDM4C
chr1:70163378-70164145	17	nogene
chr2:11815087-11820514	6	LPIN1
chr22:44921971-44922363	15	nogene
chr12:1754257-1784073	7	ADIPOR2
chr10:72128081-72133181	10	ASCC1
chr5:71517321-71522949	17	BDP1
chr20:34096656-34098633	187	EIF2S2
chr4:70826123-70833273	1	GRSF1
chr1:100077164-100078548	2	HIAT1
chr4:128074459-128091512	34	LARP1B
chr9:13216773-13250372	1	MPDZ
chr1:236041909-236048989	31	NID1
chr3:138542690-138572984	20	nogene
chr22:26496072-26496889	17	TFIP11
chr1:247860616-247864935	16	TRIM58
chr5:14290715-14336727	379	TRIO

chr7:7877280-7884346	178	UMAD1
chr22:41325563-41325918	36	ZC3H7B
chr9:34680905-34681217	0	nogene
chr1:179120189-179121867	134	ABL2
chrX:63722761-63724711	32	ARHGEF9
chr22:17594528-17600095	1	ATP6V1E1
chr6:84185186-84204096	17	CEP162
chr2:29131257-29135666	60	CLIP4
chr10:472438-520355	0	DIP2C
chr11:103280347-103316620	34	DYNC2H1
chr8:11808793-11831670	2	FDFT1
chr6:109715077-109743772	2	FIG4
chr9:127311635-127325095	43	GARNL3
chr22:19820597-19821375	16	GNB1L
chr1:236178413-236180028	2	GPR137B
chr11:119126934-119128448	3	HINFP
chr1:20770929-20780540	16	HP1BP3
chr21:43613829-43644392	9	HSF2BP
chrX:53647367-53654131	0	HUWE1
chr3:48689537-48691482	24	IP6K2
chr10:63380317-63384661	14	JMJD1C
chr3:44814910-44828300	14	KIF15
chr17:61987006-61995365	4	MED13
chr14:21498297-21503881	0	METTL3
chr12:112071894-112078741	18	NAA25
chr19:12249977-12250368	13	nogene
chr1:155314724-155315284	5	nogene
chr16:72308055-72391215	4	nogene
chr6:150978152-150978936	16	nogene
chrX:152635881-152641295	68	nogene
chr5:126793647-126794160	35	nogene
chr2:174141824-174246815	0	OLA1
chr3:196812218-196820567	11	PAK2
chr5:134197324-134206131	17	PPP2CA
chr5:38996818-39003622	19	RICTOR
chr2:11286538-11287736	60	ROCK2
chr1:169477154-169477757	118	SLC19A2
chr14:61030444-61046167	46	SLC38A6
chr15:58912562-58913696	181	SLTM
chr12:64062916-64080370	1	SRGAP1
chr6:85636966-85641451	36	SYNCRIP
chr15:42794643-42830078	2	TTBK2
chr16:31091087-31091342	1	VKORC1

chr5:113556051-113563492	15	YTHDC2
chr10:31461036-31502509	145	ZEB1
chr11:6941570-6955782	633	ZNF215
chr19:13115625-13116052	0	TRMT1
chr1:155215053-155235311	42	GBAP1
chr3:15003875-15016254	1	NR2C2
chr6:43546570-43573601	15	XPO5
chrX:68234486-68299096	35	OPHN1
chr6:100638600-100662536	14	ASCC3
chr1:174370978-174394145	92	RABGAP1L
chr14:30586717-30593639	26	G2E3
chr1:77803269-77807101	33	FAM73A
chr15:76274411-76292700	8	ETFA
chr3:136443286-136477412	28	STAG1
chr5:148634865-148677235	14	nogene
chr2:71426459-71427414	18	ZNF638
chr3:155893517-155898041	35	GMPS
chr4:153626146-153632838	7	KIAA0922
chr14:103405075-103428440	74	MARK3
chr11:8671785-8680063	0	nogene
chr1:100275598-100287203	17	RTCA
chr15:76471211-76665789	17	SCAPER
chr12:49460769-49489573	16	SPATS2
chr14:89971174-89985210	16	TDP1
chr4:153270334-153276130	28	TRIM2
chr8:86380725-86381629	0	WWP1
chr6:129494067-129558166	23	nogene
chr6:7210803-7211709	1	RREB1
chr17:1067076-1100735	19	ABR
chr6:110736032-110746201	2	CDK19
chr1:6667708-6681037	161	DNAJC11
chr15:89273376-89293063	19	FANCI
chr6:104833041-104843298	253	HACE1
chr1:32031523-32033334	0	KHDRBS1
chr4:122272159-122279981	19	KIAA1109
chr3:138488412-138488649	50	nogene
chr19:33012646-33044364	19	RHPN2
chr19:37425867-37426378	174	ZNF569
chr3:138621406-138622416	41	FAIM
chr4:53425871-53428183	122	FIP1L1
chr3:131468090-131471279	216	MRPL3
chr7:94061455-94064477	62	nogene
chr11:46783273-46790583	25	CKAP5

chr15:80978765-80982182	23	MESDC2
chr6:17661652-17675770	154	NUP153
chr11:2986296-2986727	33	nogene
chr17:66062962-66132763	17	CEP112
chr3:51386207-51387878	17	MANF
chr1:145747994-145788966	0	RNF115
chr10:7200413-7220537	17	SFMBT2
chr7:133035151-133035432	48	nogene
chr8:11808793-11838573	41	FDFT1
chr6:151009818-151015693	78	MTHFD1L
chr15:40597530-40597917	16	nogene
chr1:169836926-169850327	49	C1orf112
chr6:154787860-154805486	17	SCAF8
chrX:131743095-131794466	119	FIRRE
chr5:5436417-5447897	17	ICE1
chr9:85822879-85830576	14	LOC389765
chr12:66891900-66938347	14	nogene
chr10:122400342-122417968	0	PLEKHA1
chr19:29985222-29986417	1473	URI1
chr6:43527633-43567354	20	XPO5
chr6:136070223-136096288	0	nogene
chr14:102194937-102195120	0	WDR20
chr4:73168928-73177533	47	ANKRD17
chr22:19375630-19378066	36	HIRA
chr12:2817222-2818277	53	ITFG2
chr3:125848229-125863113	0	nogene
chr1:43894382-43899727	24	ST3GAL3
chr10:17157440-17168917	52	TRDMT1
chr6:25966334-25969420	6	TRIM38
chr16:21962459-21976243	96	UQCRC2
chr1:180078462-180080661	0	CEP350
chr7:101916114-101948970	1	CUX1
chr14:60798086-60812127	14	MNAT1
chr15:75505674-75527261	18	PTPN9
chr19:43925324-43930000	14	ZNF45
chr10:5959753-5966339	34	IL15RA
chr8:47396375-47407961	561	SPIDR
chr21:42862281-42863596	42	WDR4
chr19:47670442-47757178	1	GLTSCR1
chr7:134137022-134178609	9	LRGUK
chr12:68709237-68715740	17	NUP107
chr8:140360049-140405698	14	TRAPPC9
chr6:35281055-35287845	0	ZNF76

chr3:37273535-37282272	45	GOLGA4
chr5:126804775-126811898	194	LMNB1
chr1:28639313-28639972	39	nogene
chr19:56449556-56462429	28	ZNF667
chr8:130152735-130358143	21	ASAP1
chr16:11020192-11061022	136	CLEC16A
chr6:151014879-151015693	122	MTHFD1L
chr5:2747540-2749787	3	IRX2
chr1:51922927-51923522	16	nogene
chr15:40185164-40185642	30	BUB1B
chr12:19462509-19514784	170	AEBP2
chr12:110018344-110025823	33	ANKRD13A
chr2:39286851-39290334	7	MAP4K3
chr10:26702161-26709768	38	PDSS1
chr1:159918762-159920537	68	TAGLN2
chr3:179740246-179765848	29	USP13
chr7:33146268-33177591	0	BBS9
chr14:22950313-22952683	196	HAUS4
chr5:79640850-79669028	27	PAPD4
chr3:51541497-51590559	576	RAD54L2
chr1:156730699-156731159	52	nogene
chr1:93183359-93193744	78	CCDC18
chr11:93790573-93794035	150	MED17
chr14:75100991-75103997	30	NEK9
chr4:67745826-67802411	0	nogene
chr12:104318912-104325429	0	TXNRD1
chr1:92663419-92704744	139	EVI5
chr1:117402185-117405645	662	MAN1A2
chr10:70206263-70209685	28	PPA1
chr20:33619516-33623296	101	CBFA2T2
chr19:34396300-34396657	6	GPI
chr2:99169549-99171429	716	MITD1
chr18:49232862-49233148	3	nogene
chr17:47618349-47619164	234	NPEPPS
chr9:76067954-76107351	36	PCSK5
chr3:134158120-134195182	158	RYK
chr16:18819501-18828168	16	SMG1
chr18:9195550-9221999	63	ANKRD12
chr10:86875866-86876085	16	BMPR1A
chr5:37153739-37165671	9	C5orf42
chr10:12081471-12089255	6	DHTKD1
chr17:5462318-5463689	16	DHX33
chr5:6737509-6746427	486	PAPD7

chr11:68591575-68600494	5	PPP6R3
chr1:11016843-11020599	0	TARDBP
chr6:155285157-155298585	19	TFB1M
chr21:32383421-32385684	43	URB1
chr21:36391550-36402851	16	CHAF1B
chr20:41512845-41551360	69	CHD6
chr7:74875952-74876373	37	nogene
chr14:73089048-73089455	16	nogene
chr12:53455884-53459403	14	PCBP2
chr12:47212596-47216689	14	PCED1B
chr1:175986942-176027688	7	RFWD2
chr2:231235223-231240041	5	ARMC9
chr9:93471140-93516269	115	FAM120A
chr5:65451474-65473655	16	ADAMTS6
chr2:231206197-231235381	7	ARMC9
chr1:16036235-16058717	0	FAM131C
chr11:72984087-72989097	550	FCHSD2
chr8:41974703-41987563	75	KAT6A
chr19:4101018-4117629	2	MAP2K2
chr14:50475076-50504857	30	MAP4K5
chr6:89677569-89678745	16	MDN1
chr7:33637759-33642918	1	nogene
chr13:114052051-114057474	106	RASA3
chr2:223894233-223895630	14	WDFY1
chr11:76451857-76464080	11	C11orf30
chr12:122483459-122498863	16	ZCCHC8
chr4:83300959-83306317	0	HPSE
chr17:82763964-82814934	106	TBCD
chr5:115230975-115253255	28	PGGT1B
chr10:92974052-93014267	1	EXOC6
chr15:52425829-52428569	16	MYO5A
chr17:2700846-2701139	14	CLUH
chr17:82903404-82911789	122	TBCD
chr19:41378280-41378519	130	TMEM91
chr7:100548821-100555735	27	AGFG2
chr3:28251711-28263380	52	CMC1
chr10:27060344-27066548	0	ANKRD26
chrX:2722626-2726373	25	CD99
chr4:2699679-2700544	87	FAM193A
chr21:28163601-28217347	0	LOC101927973
chr12:115996475-115997230	1	MED13L
chr2:84870224-84870785	446	TRABD2A
chr2:216113015-216148276	14	XRCC5

chr15:100152611-100155320	24	ADAMTS17
chr1:109409927-109415363	18	PSMA5
chr15:38283496-38299547	24	SPRED1
chr22:39029362-39045542	2	APOBEC3D
chr13:24492420-24498229	2	PARP4
chr10:100354426-100354632	1	SCD
chr5:88270525-88287622	2	TMEM161B-AS1
chr2:11765532-11805156	0	LPIN1
chr5:64325185-64330406	89	RNF180
chr4:146292928-146294095	0	SLC10A7
chr5:19838758-19839242	6	CDH18
chr1:25448807-25456831	9	TMEM57
chr14:96833466-96847344	106	VRK1
chr1:108148278-108161293	206	SLC25A24
chr7:67055834-67117618	24	TYW1
chr10:35516522-35530243	269	CCNY
chr14:76154353-76195972	41	GPATCH2L
chr1:9972017-9981170	60	NMNAT1
chr4:39759544-39759926	17	nogene
chrX:72605270-72619305	16	PHKA1
chr14:34603347-34609742	21	SNX6
chr16:1397177-1403344	0	UNKL
chr2:232747614-232761436	297	GIGYF2
chr17:81270917-81284895	82	SLC38A10
chr6:43565659-43573601	98	XPO5
chr6:36524340-36525642	0	STK38
chr7:77765494-77773314	46	RSBN1L
chr19:4405911-4409759	147	CHAF1A
chr12:116230532-116237705	840	MED13L
chr7:69899285-69899498	16	AUTS2
chr7:13900737-13911307	1	ETV1
chr1:45509624-45509866	72	nogene
chr8:55950458-55953984	107	LYN
chr2:206279539-206318135	2	ZDBF2
chr2:109564365-109585838	35	10-Sep
chr12:45948436-45964188	0	SCAF11
chr3:47720665-47772936	34	SMARCC1
chr14:76166662-76180849	157	GPATCH2L
chr19:11411252-11417059	1	RGL3
chr8:73527690-73709162	14	STAU2
chr20:64022756-64026058	92	PRPF6
chr10:68962081-68963469	0	DDX21
chr10:121898840-121926527	19	ATE1

chr7:6591645-6591867	26	C7orf26
chrX:1386681-1389727	30	SLC25A6
chr14:96846094-96876120	23	VRK1
chr16:70264957-70265662	35	AARS
chr11:2964679-2979237	7	NAP1L4
chr11:77358897-77379994	1	PAK1
chr2:64551442-64553409	24	AFTPH
chr4:165305607-165310750	16	KLHL2
chr4:127930682-127942158	16	MFSD8
chr12:14794970-14797003	16	WBP11
chr22:41808874-41810291	827	CCDC134
chr10:30322390-30341640	23	MTPAP
chr4:80287336-80362884	71	C4orf22
chr16:15047441-15048099	1	NTAN1
chr16:47463881-47515601	53	PHKB
chr4:1918140-1918623	114	WHSC1
chr11:102205892-102209564	39	YAP1
chr8:22206853-22207516	0	BMP1
chr4:77055961-77056323	38	CCNI
chr18:49331863-49379758	364	DYM
chr17:44424348-44474903	10	GPATCH8
chr9:33068823-33071892	16	SMU1
chr11:126380829-126409411	25	ST3GAL4
chr21:31182420-31203012	86	TIAM1
chr2:200856681-200858089	32	CLK1
chr11:73843246-73860009	0	MRPL48
chr17:39709812-39715939	1	ERBB2
chr18:47841791-47896809	9	SMAD2
chr19:32599703-32602126	0	ANKRD27
chr11:67608551-67608722	0	NDUFV1
chr14:39074414-39085906	5	SEC23A
chr8:140347756-140360193	118	TRAPPC9
chr1:33280123-33281811	14	ZNF362
chr17:82756164-82783714	32	TBCD
chr4:2894012-2894731	14	ADD1
chr9:85618982-85633374	275	AGTPBP1
chr2:215312497-215319731	2	ATIC
chr1:50655441-50705891	16	FAF1
chrX:54151244-54159616	0	FAM120C
chr9:128508875-128515639	69	GLE1
chr16:19871815-19872846	14	GPRC5B
chr17:46066536-46067667	24	KANSL1
chr19:53202551-53204142	0	nogene

chr2:240686437-240688825	25	nogene
chr3:132603228-132631479	29	NPHP3-ACAD11
chr3:183856431-183868060	24	PARL
chr11:85981128-85982003	6	PICALM
chr14:50640749-50645014	100	SAV1
chr19:44228445-44229816	19	ZNF227
chr3:97475351-97484059	0	EPHA6
chr14:51554589-51570410	4	FRMD6
chr12:77966228-78021862	3	NAV3
chr2:218673624-218676278	15	STK36
chr3:129827802-129828508	14	TMCC1
chr6:87213901-87218731	15	ZNF292
chr16:88033108-88038011	171	BANP
chr2:28926467-28935607	20	WDR43
chr11:68347846-68348243	94	LRP5
chr6:30627459-30642247	4	ATAT1
chr10:103347237-103348999	73	PCGF6
chr18:49491625-49491833	68	RPL17
chr20:41092471-41101353	140	TOP1
chr6:107503656-107587175	15	SOBP
chr7:131429032-131443702	3	MKLN1
chr2:230440475-230450255	3	SP100
chr20:13806444-13807091	14	nogene
chr8:140789473-140879637	27	PTK2
chr1:62577261-62586624	53	DOCK7
chr5:1611668-1612072	26	nogene
chr2:235883344-235968623	18	AGAP1
chr7:92294888-92327900	368	ANKIB1
chr5:78034360-78101025	18	AP3B1
chr6:129611532-129629522	2	ARHGAP18
chr3:11331339-11426926	9	ATG7
chr1:160313989-160333679	18	COPA
chr16:3770569-3770986	18	CREBBP
chr1:244657845-244692020	18	DESI2
chr6:30659471-30660242	1	DHX16
chr14:101991526-101994849	18	DYNC1H1
chr7:107179878-107196151	0	HBP1
chr3:183650295-183651276	12	KLHL24
chr4:102664684-102723967	18	MANBA
chr3:61621969-61623140	24	nogene
chr16:72193245-72308188	18	nogene
chr15:85109052-85126374	18	PDE8A
chr3:121467518-121485184	18	POLQ

chr1:32668738-32672901	0	RBBP4
chr1:173946475-173964187	18	RC3H1
chr2:86251956-86282242	14	REEP1
chr20:36927181-36941111	18	SAMHD1
chr2:229829192-229860531	18	TRIP12
chr8:102299451-102300151	18	UBR5
chr7:99525812-99526418	18	ZKSCAN5
chr2:135345549-135350215	3	ZRANB3
chr2:168074981-168129758	59	STK39
chr14:37285317-37308519	117	MIPOL1
chr9:4823547-4841357	20	RCL1
chr11:3038049-3042256	15	CARS
chr12:120183568-120184887	0	GCN1
chr3:155114993-155118811	5	MME
chr2:11286538-11288449	261	ROCK2
chr2:9287003-9297445	16	ASAP2
chr9:135850136-135850461	218	CAMSAP1
chr5:50399106-50405852	19	EMB
chr4:48684672-48710698	125	FRYL
chr19:4475274-4488876	0	HDGFRP2
chr4:169424552-169433665	14	NEK1
chr12:71657548-71660427	14	nogene
chr1:51745535-51765981	48	OSBPL9
chr17:8262919-8263636	33	PFAS
chr2:28783906-28788809	156	PPP1CB
chr13:41907585-41950013	37	VWA8
chr19:51839892-51886912	19	ZNF577
chr4:76130655-76132719	3	NUP54
chr19:15397149-15410594	15	AKAP8L
chrX:15845378-15852524	0	AP1S2
chr21:25954589-25982477	29	APP
chr3:57575547-57577387	14	ARF4
chr6:36213879-36218010	105	BRPF3
chr2:44704282-44715353	366	CAMKMT
chr1:92736397-92745802	42	EVI5
chr22:32479132-32493281	15	FBXO7
chr1:37587310-37589082	206	GNL2
chr4:44707751-44722242	15	GNPDA2
chr4:142402937-142468055	4	INPP4B
chr3:122463941-122496570	15	KPNA1
chr12:52237508-52243137	15	KRT7
chr1:235693349-235697272	15	LYST
chr13:23745852-23746124	15	nogene

chr17:18661913-18663227	0	nogene
chr10:63448450-63448857	23	nogene
chr20:63672082-63672579	14	nogene
chr7:135571104-135573825	15	NUP205
chr10:3144291-3147737	15	PITRM1
chr17:76287191-76287897	15	QRICH2
chr17:783082-786716	15	RNMTL1
chr17:82884144-82911789	168	TBCD
chr17:82805874-82809782	30	TBCD
chr3:194604286-194628509	0	TMEM44
chr14:90676522-90786328	0	TTC7B
chr17:42612074-42613073	21	TUBG1
chr17:31885979-31887313	15	UTP6
chr20:58438944-58441083	1153	VAPB
chr3:51490154-51492513	15	nogene
chr16:22313619-22315208	46	POLR3E
chr3:23589733-23595250	39	UBE2E2
chr13:32522181-32527532	16	N4BP2L2
chr3:33812071-33828969	5	PDCD6IP
chr1:48385308-48413151	16	SPATA6
chrX:129496749-129515994	0	SMARCA1
chr4:80335743-80420323	0	nogene
chr2:219541151-219541615	0	CHPF
chr19:17314022-17315995	0	DDA1
chr6:53071056-53076543	881	FBXO9
chr2:238182064-238184113	10	ILKAP
chr10:93387796-93389154	2	MYOF
chr4:98583518-98586087	27	nogene
chr16:67912855-67914144	18	nogene
chr19:19482813-19483551	7	nogene
chr3:145983607-145990927	15	nogene
chr4:2954423-2956811	73	NOP14
chr2:46145092-46151229	1	PRKCE
chr5:87331347-87353235	113	RASA1
chr14:90943077-90947550	29	RPS6KA5
chr11:63748374-63750198	0	RTN3
chr6:44932652-45365301	24	SUPT3H
chr2:28941460-28947161	16	WDR43
chr2:61493893-61495613	23	XPO1
chr7:148846496-148847305	95	EZH2
chr5:43675510-43702736	2	NNT
chr16:72096821-72097009	17	DHX38
chr10:49500537-49506012	166	ERCC6

chr9:20907149-20916937	89	FOCAD
chr22:20446442-20471515	6	KLHL22
chr10:102057052-102057230	17	nogene
chr11:74203727-74222369	9	PPME1
chr21:37108391-37132766	34	TTC3
chr10:122392302-122424959	39	PLEKHA1
chr16:58532231-58534395	0	CNOT1
chr15:44484223-44499813	21	CTDSPL2
chr4:430343-437511	39	nogene
chr16:69260049-69270285	0	SNTB2
chr5:151786571-151803996	30	G3BP1
chr20:9217389-9365514	16	PLCB4
chr1:176081151-176149074	14	RFWD2
chr12:122377388-122380558	164	CLIP1
chr12:122920187-122921043	0	ABCB9
chr19:19042710-19044074	0	ARMC6
chr10:103049706-103056964	6	CNNM2
chr2:210129030-210154611	6	KANSL1L
chr4:4299926-4303091	16	ZBTB49
chr9:127658374-127661205	16	STXBP1
chr6:154441291-154442290	21	CNKSR3
chr4:150798080-150817257	243	LRBA
chr5:113101738-113104355	18	MCC
chr3:183840569-183844326	2	PARL
chr3:112563384-112573956	2	SLC35A5
chr3:27376715-27424152	18	SLC4A7
chr2:159248371-159283093	1	WDSUB1
chr17:1258559-1259559	10	nogene
chr7:66795510-66797506	14	RABGEF1
chrX:68335242-68336551	0	nogene
chr3:4669653-4676801	0	ITPR1
chr1:112917177-112918044	24	SLC16A1
chr16:3791980-3793626	77	CREBBP
chr6:136866626-136870003	1	PEX7
chr12:100098351-100103951	28	UHRF1BP1L
chr6:89335301-89342455	0	UBE2J1
chr18:23504370-23509279	0	C18orf8
chr2:69070646-69077488	7	ANTXR1
chr10:989997-990473	18	nogene
chr18:32111753-32113860	2789	RNF138
chr2:109585125-109585838	99	10-Sep
chr2:230720455-230760115	29	CAB39
chr10:67932596-67941105	14	HERC4

chr1:155923626-155929840	14	KIAA0907
chr3:132603228-132619545	1	NPHP3-ACAD11
chr8:18011236-18011827	30	PCM1
chr16:70526586-70529199	41	SF3B3
chr1:28580559-28581229	619	SNHG12
chr2:201749711-201754671	1	ALS2
chr10:42791626-42793991	4	BMS1
chr2:202952558-202954134	0	CARF
chr7:134137022-134191751	13	LRGUK
chr5:95490907-95495017	13	TTC37
chr3:10234458-10239039	4	IRAK2
chr12:45331294-45357424	98	ANO6
chrX:68048415-68053810	4	OPHN1
chr4:128992166-129082373	29	SCLT1
chr14:35007282-35013902	0	SRP54
chr7:100355142-100355804	293	STAG3L5P-PVRIG2P-PILRB
chr10:27116059-27116345	2	YME1L1
chr14:81185530-81216514	15	GTF2A1
chr18:12999420-13030608	326	CEP192
chr7:2213211-2222754	97	MAD1L1
chr2:10600852-10607311	112	NOL10
chr17:82763964-82768566	55	TBCD
chr4:98881040-98891332	15	EIF4E
chr19:9337795-9339319	70	ZNF559
chr17:29489663-29491865	13	TAOK1
chr15:99993047-99997589	16	ADAMTS17
chr11:108330213-108332037	4	ATM
chr19:1969507-1969959	23	CSNK1G2
chr2:182962158-182967361	6	NCKAP1
chr19:53521855-53576819	3	nogene
chr2:69996685-70085624	2	PCBP1-AS1
chr15:64859893-64865228	6	PLEKHO2
chr3:40461406-40462248	145	RPL14
chr7:98190727-98194572	11	LMTK2
chr7:92343023-92362273	16	ANKIB1
chr6:130955104-130956499	77	EPB41L2
chr12:104895273-104928694	6	SLC41A2
chr19:12810070-12810404	32	RNASEH2A
chr11:29106242-29211765	10	nogene
chrX:64348659-64350070	4	MTMR8
chr13:29324612-29359473	59	MTUS2
chr2:44201185-44218537	47	PPM1B
chr8:73582769-73739868	4	STAU2

chr17:37246825-37253036	31	ACACA
chr2:36358903-36442735	149	CRIM1
chr6:57532410-57606457	38	PRIM2
chr9:33886880-33900271	48	UBE2R2
chr2:69849651-69871832	0	GMCL1
chr16:69840125-69842120	0	WWP2
chr7:22960255-22991139	947	FAM126A
chr12:7432415-7433694	62	CD163L1
chr4:904636-913668	17	GAK
chr10:102258928-102260116	36	GBF1
chr19:18934302-18939049	9	HOMER3
chr10:124774674-124790338	140	METTL10
chr3:37047518-37049017	28	MLH1
chr9:122270863-122280598	28	MRRF
chr11:19868924-19892594	15	NAV2
chr15:90937253-90937581	15	nogene
chr19:42247908-42248424	15	nogene
chr16:11933516-11937668	77	nogene
chr14:31093701-31094760	15	nogene
chr11:48069699-48070069	15	nogene
chr17:8256523-8256963	49	PFAS
chr3:48549863-48550234	20	PFKFB4
chr22:21486799-21488381	47	PI4KAP2
chr6:105368902-105377519	2	PREP
chr7:105508114-105508520	15	PUS7
chr7:66771882-66810847	78	RABGEF1
chr20:37056145-37068186	15	RBL1
chr5:94643272-94654752	34	SLF1
chr11:18506856-18509665	2	TSG101
chr17:67920014-67922990	27	BPTF
chr15:67231229-67236820	120	AAGAB
chr8:13098398-13100770	13	DLC1
chr1:225144521-225185425	15	DNAH14
chr10:21918778-21929141	21	DNAJC1
chr5:171910571-171914405	61	FBXW11
chr10:1005817-1009580	25	GTPBP4
chr10:68025545-68044563	438	HERC4
chr3:37503177-37542585	15	ITGA9
chr21:28212239-28228478	68	LOC101927973
chr4:150893051-150897818	0	LRBA
chr2:74207703-74211866	13	MTHFD2
chr11:62768351-62768619	15	nogene
chr14:61713935-61715603	13	nogene

chr11:68519500-68551186	50	PPP6R3
chr7:66768830-66783841	0	RABGEF1
chr9:127912267-127912680	15	ST6GALNAC4
chr4:106095481-106212835	1	TBCK
chr15:66532246-66546705	26	ZWILCH
chr17:4064360-4067242	15	ZZEF1
chr18:12351084-12356831	18	AFG3L2
chr14:21415573-21429335	23	CHD8
chr1:219981377-219983398	1	EPRS
chrX:70341798-70387297	95	KIF4A
chr6:141704581-141705067	35	nogene
chr17:1344858-1345238	14	nogene
chr1:46066053-46080750	1	PIK3R3
chr17:50139604-50140092	13	PPP1R9B
chr1:43538197-43578920	41	PTPRF
chr3:47097954-47106120	259	SETD2
chr1:172600063-172602787	40	SUCO
chr18:62348112-62349937	15	TNFRSF11A
chr7:25142240-25142814	20	C7orf31
chr3:67746770-67768433	1	SUCLG2-AS1
chr3:156694019-156696025	265	TIPARP
chr2:113934278-113942359	3	ACTR3
chr14:58304943-58318805	16	ARID4A
chr8:123328189-123336532	15	ATAD2
chr6:26462796-26463525	14	BTN2A1
chr11:46769962-46770982	15	CKAP5
chr8:17234715-17237373	0	CNOT7
chr16:58184259-58196844	1	CSNK2A2
chr16:28723163-28735582	29	EIF3C
chr5:65988634-66014725	540	ERBB2IP
chr6:98899267-98927822	30	FBXL4
chr7:55982209-55983868	91	GBAS
chr12:109855618-109858741	15	GLTP
chr15:72350517-72353178	0	HEXA
chr10:132705192-132710456	15	INPP5A
chr3:45485691-45500579	3	LARS2
chr7:24661840-24668660	15	MPP6
chr13:29767098-29796911	1	nogene
chr15:82958974-82959308	27	nogene
chr5:151946638-152055452	18	nogene
chr11:29381241-29384112	15	nogene
chr13:32732100-32746100	45	PDS5B
chr7:66768830-66775393	36	RABGEF1

chr3:8912311-8941804	15	RAD18
chr4:2490336-2500738	296	RNF4
chr6:88890494-88929267	0	RNGTT
chr12:123327425-123331375	18	SBNO1
chr14:30628208-30643405	16	SCFD1
chr19:20934948-20937361	13	ZNF85
chr22:23268407-23273774	0	BCR
chr8:67095292-67095732	102	CSPP1
chr21:46529091-46534687	18	DIP2A
chr13:49187121-49188633	19	FNDC3A
chr6:73111307-73169854	15	KCNQ5
chr5:140925895-140929325	7	PCDHAC1
chr17:64562770-64583544	16	SMURF2
chr1:11013715-11025208	17	TARDBP
chr20:13569506-13630152	56	TASP1
chr14:102424978-102428382	15	TECPR2
chr2:97158106-97167767	53	ANKRD36
chr6:83934594-83960029	1	CYB5R4
chr7:157339720-157367483	5	DNAJB6
chr8:42964310-42982696	18	HOOK3
chr3:121532989-121541479	35	POLQ
chr6:123252411-123267751	21	TRDN
chr3:122449573-122467429	32	KPNA1
chr2:88749980-88751082	0	RPIA
chr17:29285991-29287716	46	NUFIP2
chr9:125337017-125337591	413	GAPVD1
chr6:104744159-104750472	94	HACE1
chr7:66712166-66734928	0	RABGEF1
chr16:29798747-29798950	4	KIF22
chr3:15563357-15568586	13	HACL1
chr20:50940864-50945923	21	DPM1
chr12:116230432-116237705	351	MED13L
chr2:241325992-241336098	39	2-Sep
chr9:77247258-77252352	6	VPS13A
chr1:100424221-100462881	15	CDC14A
chr3:71041327-71053773	109	FOXP1
chr1:43669099-43671875	1	KDM4A
chrX:24073600-24074959	15	nogene
chr1:204552180-204552423	42	nogene
chr6:34341861-34370492	57	NUDT3
chr1:233090060-233161363	15	PCNXL2
chr2:20307977-20333063	77	PUM2
chr19:11147817-11148113	105	SPC24

chr8:140023936-140252929	15	TRAPPC9
chr20:17952586-17955475	43	SNX5
chr4:159238808-159241368	7	nogene
chr12:16721-17859	13	LOC100288778
chr9:133412298-133412921	607	REXO4
chr1:145771710-145788966	17	RNF115
chr4:169155479-169156679	192	SH3RF1
chr10:120880825-120890009	26	WDR11
chr5:168360614-168371533	0	WWC1
chr19:45419097-45421393	40	ERCC1
chr5:141983379-141984933	0	RNF14
chr22:29358725-29367270	13	AP1B1
chr10:94245945-94246621	73	PLCE1
chr8:43097523-43103830	13	POMK
chr3:142748257-142748460	0	TRPC1
chr7:148846469-148847305	804	EZH2
chr4:145845955-145849561	172	ZNF827
chr17:76097795-76098018	0	EXOC7
chr9:4823547-4827033	141	RCL1
chr17:37284837-37289528	4	ACACA
chr7:127807483-127844424	15	SND1
chr1:70292387-70324957	13	ANKRD13C
chr6:33320010-33320591	17	DAXX
chr15:65490188-65507355	17	DPP8
chr6:167003722-167013567	17	FGFR1OP
chrX:152345580-152364596	3	GABRA3
chr11:33286412-33286952	43	HIPK3
chr1:63478763-63510181	13	ITGB3BP
chr8:23875895-23922600	17	nogene
chr10:100361465-100361621	2	nogene
chr17:81558146-81558664	17	nogene
chr2:120141978-120170783	17	nogene
chr16:2981377-2982882	2	nogene
chr20:58693736-58703071	17	NPEPL1
chr10:34450316-34493725	13	PARD3
chr20:37044085-37055656	17	RBL1
chr3:27420699-27452498	3	SLC4A7
chr3:67508806-67529186	13	SUCLG2
chr19:3013668-3015760	19	TLE2
chr22:40277076-40301333	0	TNRC6B
chr15:82404611-82415675	13	UBE2Q2P2
chr4:84739009-84740416	17	WDFY3
chr4:176096462-176139974	17	WDR17

chr10:1056347-1072293	18	WDR37
chr20:18297984-18298464	7	ZNF133
chr11:126364530-126381942	32	nogene
chr17:81567416-81572088	0	NPLOC4
chr17:4189381-4197577	1	ANKFY1
chr14:67352922-67353959	0	ATP6V1D
chr9:120545713-120568388	16	CDK5RAP2
chr16:80684537-80685129	281	CDYL2
chr8:11444030-11444808	32	FAM167A
chr6:5368549-5431172	14	FARS2
chr19:43900867-43901775	19	LOC100505715
chr1:85036709-85041177	0	MCOLN3
chr16:4650364-4657363	50	MGRN1
chr16:4651962-4652824	1	MGRN1
chr5:172125656-172126326	13	nogene
chr2:10644319-10668723	321	NOL10
chr1:19890384-19907660	0	OTUD3
chr13:24441845-24453654	1	PARP4
chr3:136260478-136283947	2	PCCB
chr5:134697898-134703932	63	SEC24A
chr5:128138596-128152805	48	SLC12A2
chr5:179823857-179825226	108	SQSTM1
chr11:75879878-75912039	86	UVRAG
chr6:31795272-31795545	50	VARS
chrX:118387339-118410959	20	WDR44
chr9:122865348-122883379	33	RC3H2
chrX:101035612-101041371	4	TRMT2B
chr8:61525976-61556022	6	ASPH
chr15:40608846-40619011	30	CASC5
chr16:67066681-67082339	94	CBFB
chrX:147921932-147936613	1	FMR1
chr20:18487887-18488353	0	nogene
chr11:59787036-59795482	2	STX3
chr5:60642811-60647533	47	DEPDC1B
chr1:54871349-54876047	5	DHCR24
chr5:94868332-94912976	6	MCTP1
chr11:93790573-93801972	37	MED17
chr8:103404775-103407784	287	SLC25A32
chr12:15956935-15982436	3	DERA
chr13:100330560-100340259	5	PCCA
chr5:43122038-43161931	18	ZNF131
chr2:108873450-108876442	30	CCDC138
chr17:81588943-81597316	37	NPLOC4

chr4:128997873-129003876	56	SCLT1
chr15:64974601-64983593	134	SPG21
chr14:72993142-72993357	12	ZFYVE1
chr3:57847196-57858159	29	SLMAP
chr6:77271339-77273307	668	nogene
chr9:93629969-93649212	1	PHF2
chr11:111752158-111753577	15	PPP2R1B
chr2:9527785-9536828	11	ADAM17
chr17:42480666-42495716	2	ATP6V0A1
chr20:41533049-41551360	138	CHD6
chr1:200648438-200649226	0	DDX59
chr5:137692284-137709856	3	KLHL3
chr10:96944112-96952196	116	LCOR
chr11:47295495-47296055	2	MADD
chr5:25177590-25180826	20	nogene
chr17:47596352-47601747	0	NPEPPS
chr11:68583042-68596218	48	PPP6R3
chr19:49583206-49583952	0	PRRG2
chr6:138907494-138915976	17	REPS1
chr1:179347599-179348942	7	SOAT1
chr13:41865735-41912168	17	VWA8
chr13:111233204-111244294	14	ARHGEF7
chr6:156829226-156901525	50	ARID1B
chr6:38568436-38598121	15	BTBD9
chr9:128836801-128838367	0	CCBL1
chr5:123364495-123399284	15	CEP120
chr17:8238030-8238629	108	CTC1
chr9:19276157-19276479	23	DENND4C
chr19:32432715-32455040	2	DPY19L3
chr7:2360709-2372795	15	EIF3B
chr7:100819556-100820296	115	EPHB4
chr14:31113103-31114340	46	HECTD1
chr6:33664869-33665976	15	ITPR3
chr20:25467388-25470095	12	NINL
chr19:48980032-48980627	13	nogene
chr10:37729820-37749733	41	nogene
chr1:51814034-51834170	46	NRD1
chr3:170106831-170122744	0	PHC3
chr20:50579702-50579926	0	PTPN1
chr20:2986764-3007420	16	PTPRA
chr12:120546404-120546601	15	RNF10
chr1:213125765-213176492	15	RPS6KC1
chr4:109491326-109494856	303	SEC24B

chr5:134157706-134174022	386	SKP1
chr9:121348014-121356156	14	STOM
chr1:95138274-95173889	29	TMEM56
chr4:119245336-119248882	4	USP53
chr3:183786124-183786301	15	YEATS2
chr12:133041099-133048848	0	ZNF84
chr4:145162639-145171720	57	OTUD4
chr22:26717252-26718231	15	MIATNB
chr3:196801926-196805383	8	PAK2
chr17:60288522-60345608	5	USP32
chr7:148786550-148790441	14	CUL1
chr22:39403830-39404132	13	nogene
chr4:38020590-38027879	21	TBC1D1
chr3:183715143-183728851	69	YEATS2
chr3:127793784-127794251	24	nogene
chr11:33141666-33161369	27	CSTF3
chr9:137206231-137216017	2	NDOR1
chr4:151352899-151353391	2	nogene
chr14:39177411-39177916	35	PNN
chr20:37061102-37062270	73	RBL1
chr2:61495454-61499894	36	XPO1
chr5:66053405-66054951	94	ERBB2IP
chr2:69806384-69812709	3	ANXA4
chr2:222928861-222934687	1	ACSL3
chr12:27368261-27376387	36	ARNTL2
chr1:235480053-235496308	193	B3GALNT2
chr2:171925536-171966949	20	HAT1
chr1:117460488-117502970	174	MAN1A2
chr6:112912876-112937686	9	nogene
chr11:11432620-11448936	35	GALNT18
chr19:7441652-7447168	0	ARHGEF18
chr1:93239309-93239896	15	CCDC18
chr16:58576462-58583182	30	CNOT1
chr2:171448680-171458075	83	DCAF17
chr15:76287845-76297348	0	ETFA
chr15:89268398-89276891	6	FANCI
chr12:100591735-100622382	78	GAS2L3
chr17:30484213-30492753	25	GOSR1
chr17:46066536-46094701	374	KANSL1
chr13:23756544-23809924	25	MIPEP
chr6:27951606-27958148	0	nogene
chr16:223343-223705	18	nogene
chr17:68115705-68115938	527	nogene

chr5:79668891-79682061	10	PAPD4
chr10:34269656-34284245	460	PARD3
chr11:581491-592674	38	PHRF1
chr1:151303540-151306363	1	PI4KB
chr2:158533179-158603104	273	PKP4
chr10:119898426-119898959	1	SEC23IP
chr10:13333816-13345028	67	SEPHS1
chr16:22471627-22471852	8	SMG1P1
chr16:28979154-28982545	0	SPNS1
chr14:21364826-21373430	0	SUPT16H
chr5:1255286-1282624	0	TERT
chr14:102791205-102903429	16	TRAF3
chr11:35725923-35726163	42	TRIM44
chr12:122249870-122251099	15	VPS33A
chr2:223884647-223895630	12	WDFY1
chr20:47262287-47276795	118	ZMYND8
chr5:14293011-14316743	17	TRIO
chr11:65503853-65505019	2	nogene
chr13:50927406-50943394	20	RNASEH2B
chr2:237973091-237994809	56	UBE2F
chr15:78157547-78169077	16	IDH3A
chr8:73582769-73617451	1	STAU2
chr2:23810315-23823569	13	ATAD2B
chr7:2229983-2230727	254	MAD1L1
chr5:109713519-109729513	750	MAN2A1
chr9:126392065-126421948	82	MVB12B
chr2:32197343-32209561	2	SLC30A6
chr2:177420268-177462018	23	AGPS
chrX:1285777-1290509	83	CSF2RA
chr13:59774188-59839448	2	DIAPH3
chr11:77379240-77379994	192	PAK1
chr12:89425160-89472275	26	POC1B
chr18:59466738-59471769	17	CCBE1
chr3:15563357-15573242	0	HACL1
chr10:45946836-45962994	55	PARGP1
chr4:145123214-145125101	14	ABCE1
chr19:1510149-1511160	11	ADAMTSL5
chr1:43197552-43199503	13	CFAP57
chr7:2239417-2239676	13	FTSJ2
chr11:58549785-58551232	3	LPXN
chr15:71888003-71893778	30	MYO9A
chr16:71669274-71676649	14	PHLPP2
chr1:15717892-15721388	185	PLEKHM2

chr1:62439798-62443319	14	USP1
chr1:94177611-94190083	14	ARHGAP29
chr17:63751939-63752799	27	CCDC47
chr8:60800387-60808272	38	CHD7
chr9:137564433-137565154	98	DPH7
chr14:76166662-76195972	147	GPATCH2L
chr5:179615545-179617911	14	HNRNPH1
chr11:65864496-65865145	14	MUS81
chr7:65403175-65403644	14	nogene
chr1:243270091-243286397	43	SDCCAG8
chr9:33956857-33963791	14	UBAP2
chr9:130862762-130863035	21	ABL1
chr5:177536357-177539042	39	FAM193B
chr11:47486749-47489024	4	CELF1
chr19:48985460-48986035	0	GYS1
chr5:43675510-43677806	64	NNT
chr3:3156218-3174261	42	CRBN
chr17:5460938-5462546	193	DHX33
chr12:50625975-50660319	4	DIP2B
chrX:24065997-24071727	15	EIF2S3
chr17:82763964-82893632	23	TBCD
chr9:33935838-33941862	4	UBAP2
chr22:42514105-42574818	94	RRP7A
chr17:39490556-39501439	7	CDK12
chr4:151454700-151482648	12	FAM160A1
chr3:45417481-45458886	16	LARS2
chr19:11727432-11727986	19	nogene
chr2:9410807-9411533	13	nogene
chr16:69718122-69718534	115	NQO1
chr3:196818056-196820567	8	PAK2
chr12:25995036-26003450	0	RASSF8
chr2:202206736-202220106	13	SUMO1
chr17:78700330-78709732	0	CYTH1
chr15:90803520-90804359	13	BLM
chr1:20757165-20771073	12	HP1BP3
chr22:33761370-33769743	27	LARGE
chr11:63850874-63851367	19	nogene
chr7:66771882-66783841	169	RABGEF1
chr5:141984802-141987975	0	RNF14
chr20:63928334-63931734	38	DNAJC5
chr12:65308528-65328603	32	MSRB3
chr4:147857552-147881932	410	ARHGAP10
chr5:140438460-140441142	0	ANKHD1-EIF4EBP3

chr18:26315228-26357794	47	TAF4B
chr22:40868998-40886515	16	XPNPEP3
chr4:88475840-88479507	37	HERC5
chr22:46433392-46439411	160	CELSR1
chr16:29610169-29610757	14	nogene
chr5:168557422-168568998	12	PANK3
chr4:39199477-39203722	14	WDR19
chr10:179993-249088	14	ZMYND11
chr9:129503322-129503672	4	LINC00963
chr17:82763964-82800996	74	TBCD
chr16:53154446-53157541	16	CHD9
chr15:51451644-51455228	37	DMXL2
chr3:183205875-183207823	205	MCF2L2
chr21:43630321-43633421	47	HSF2BP
chr2:86159535-86162079	86	IMMT
chr3:37294978-37302332	20	GOLGA4
chr7:27629370-27649623	1	HIBADH
chr7:5715052-5716766	14	RNF216
chr7:761752-770618	13	DNAAF5
chr4:75655065-75655870	0	G3BP2
chr16:11883014-11885274	2	GSPT1
chr3:195523691-195524896	13	PPP1R2
chr2:202464808-202552888	15	BMPR2
chr6:108323824-108347039	0	LACE1
chr17:12054888-12113360	152	MAP2K4
chr6:3154519-3225432	29	nogene
chr7:5480864-5485820	26	nogene
chr1:16164470-16168253	6	nogene
chr1:10403070-10404279	82	PGD
chr1:99993536-99999415	63	SLC35A3
chr3:12489783-12496554	55	TSEN2
chr3:9810620-9817759	1	TTLL3
chr3:51440969-51443903	105	VPRBP
chr14:102325984-102335838	21	ZNF839
chr12:6655637-6656798	16	ING4
chr10:124777061-124777777	15	nogene
chr6:75844896-75862723	0	MYO6
chr2:28901986-28906581	52	WDR43
chr4:168890921-168903906	10	PALLD
chr3:56566949-56571302	17	CCDC66
chr8:141185138-141190417	10	DENND3
chr5:50399106-50411383	668	EMB
chr5:119473907-119479021	0	HSD17B4

chr9:6805598-6814745	1	KDM4C
chr1:113127852-113129039	17	nogene
chr12:110538143-110551968	30	PPTC7
chr17:32182756-32183272	21	RHOT1
chr6:10935057-10956242	49	SYCP2L
chr19:34451980-34467014	71	UBA2
chr3:188484591-188490641	29	LPP
chr1:213071005-213104569	5	RPS6KC1
chr11:128986002-128988125	0	ARHGAP32
chrX:63665885-63706449	3	ARHGEF9
chr11:33583337-33598947	17	KIAA1549L
chr11:47634671-47639051	1	MTCH2
chr10:68488466-68493570	39	SLC25A16
chr2:28906459-28917995	84	WDR43
chr7:5216562-5217187	4	WIPI2
chr4:87046165-87047594	610	AFF1
chr6:52267864-52269226	123	MCM3
chr10:68206769-68210547	58	MYPN
chr15:73122524-73136027	30	NEO1
chr2:55646258-55647453	2	PNPT1
chr2:213340162-213364145	15	SPAG16
chr17:54985613-55007597	0	STXBP4
chr1:42194889-42323827	16	FOXJ3
chr19:3425105-3452666	47	NFIC
chr10:68722454-68747275	14	CCAR1
chr3:113399905-113427339	14	CFAP44
chr7:846239-846436	25	nogene
chrX:123686547-123697751	39	THOC2
chr17:59808795-59817773	15	VMP1
chr10:115215696-115315736	0	ATRNL1
chr7:133317283-133375002	10	EXOC4
chr16:16007815-16016621	57	ABCC1
chr20:34031521-34078553	0	RALY
chr12:28198942-28259442	1	CCDC91
chr6:157148623-157150853	19	ARID1B
chr2:23884764-23888399	18	ATAD2B
chr2:159448241-159453801	0	BAZ2B
chr19:48231659-48241063	24	CARD8
chr6:109140420-109146937	52	CEP57L1
chr5:50399106-50403454	54	EMB
chr9:20715321-20765073	29	FOCAD
chr2:69342151-69363670	0	GFPT1
chr17:30484213-30510909	1	GOSR1

chr10:91460426-91462184	10	HECTD2
chr2:27470927-27472362	17	IFT172
chr2:39299742-39315388	0	MAP4K3
chr19:12079094-12079397	17	nogene
chrX:71554512-71557672	17	OGT
chr20:20524405-20536784	0	RALGAPA2
chr6:151445307-151445825	15	RMND1
chr3:47678197-47686170	52	SMARCC1
chr7:2255000-2278305	0	SNX8
chr20:49135832-49166285	34	STAU1
chr12:51103669-51116420	0	TFCP2
chr12:98527885-98537572	63	TMPO
chr20:3121383-3123406	0	UBOX5
chr1:246435944-246440124	39	nogene
chr15:68189202-68193645	0	nogene
chrX:68192918-68213972	0	OPHN1
chr11:77626561-77626801	38	nogene
chr18:31897785-31913522	1	TRAPPC8
chr13:26612960-26676724	19	WASF3
chr6:47283937-47286595	181	TNFRSF21
chr22:43926939-43940125	0	PNPLA3
chr2:231270981-231296253	5	ARMC9
chr7:140776911-140787584	52	BRAF
chr11:93725650-93727637	0	CEP295
chr17:63578469-63589237	5	DCAF7
chr2:47834578-47834871	23	FBXO11
chr1:37574342-37576556	27	GNL2
chr5:1494699-1501603	15	LPCAT1
chr13:29281703-29359473	30	MTUS2
chr10:72708282-72741845	5	nogene
chr16:15698070-15698562	14	nogene
chr8:94743307-94743727	21	nogene
chr12:128808787-128815070	259	SLC15A4
chr13:29514352-29553860	106	SLC7A1
chr9:127435089-127436003	15	ZNF79
chr11:46808030-46809874	62	CKAP5
chr14:23061083-23064488	15	ACIN1
chr22:25672295-25695214	3	ADRBK2
chr22:25594298-25644665	14	ADRBK2
chr1:65190709-65224870	16	AK4
chr1:243637610-243695716	10	AKT3
chr17:4209823-4235890	26	ANKFY1
chr5:14741826-14751239	19	ANKH

chr9:97994630-98015517	26	ANP32B
chr5:73832337-73840760	72	ARHGEF28
chr2:9356046-9368519	77	ASAP2
chr2:175111567-175118369	8	ATF2
chr18:31630958-31666372	9	B4GALT6
chr2:28127586-28129380	0	BRE
chr9:135850136-135866536	153	CAMSAP1
chr22:37510211-37518108	14	CARD10
chr1:100484291-100504937	16	CDC14A
chr10:14825478-14828272	0	CDNF
chr3:138498030-138537347	14	CEP70
chr3:138525489-138537347	81	CEP70
chr1:16628467-16633346	13	CROCCP2
chr11:107416208-107418287	52	CWF19L2
chr1:100196213-100206636	4	DBT
chr2:232087486-232087721	38	DIS3L2
chr1:224952673-225007544	16	DNAH14
chr7:157409794-157416255	0	DNAJB6
chr7:34973513-35018596	14	DPY19L1
chr5:31448546-31451640	97	DROSHA
chr6:52423686-52454287	19	EFHC1
chr9:137743370-137754291	14	EHMT1
chr6:53269086-53276256	39	ELOVL5
chr16:70465125-70468968	14	FUK
chr3:158681994-158691648	30	GFM1
chrX:84321841-84361612	20	HDX
chr11:43798319-43816391	16	HSD17B12
chr5:133064979-133070496	156	HSPA4
chr1:116613764-116616457	14	IGSF3
chr19:7141676-7163199	16	INSR
chr3:4516475-4521094	14	ITPR1
chr6:18207397-18217885	17	KDM1B
chr7:152220522-152222682	15	KMT2C
chr2:185729328-185788943	2	LOC101927196
chr3:37121491-37149038	15	LRRFIP2
chr7:1898199-1936897	38	MAD1L1
chr3:183309715-183311772	16	MCF2L2
chr6:109446695-109447229	16	MICAL1
chr2:96360142-96367373	14	NCAPH
chr15:55869578-55874008	14	NEDD4
chr9:14146688-14180866	4	NFIB
chr19:19427695-19427992	16	nogene
chr14:74735983-74736325	57	nogene

chr5:85821331-85827796	14	nogene
chr17:62474354-62476831	18	nogene
chr6:17646066-17649300	50	NUP153
chr2:174123594-174142000	26	OLA1
chr2:205440369-205440672	149	PARD3B
chr3:130703722-130708492	14	PIK3R4
chr1:52404680-52412624	16	PRPF38A
chr9:131430089-131432794	267	PRRC2B
chr18:8076454-8247919	14	PTPRM
chr12:69784719-69801721	3	RAB3IP
chr1:32672449-32672901	0	RBBP4
chr20:37020658-37022826	16	RBL1
chr17:75651185-75666620	8	RECQL5
chr1:246726891-246767329	16	SCCPDH
chr3:185590113-185613408	7	SENP2
chr14:99412950-99465813	21	SETD3
chr11:66058829-66059045	16	SF3B2
chr18:12420324-12433000	16	SLMO1
chr16:18815174-18834438	16	SMG1
chr1:179335505-179337896	2	SOAT1
chr2:230357804-230359132	11	SP140L
chr1:1561331-1564916	186	SSU72
chr6:147260614-147278204	27	STXBP5
chr19:45844029-45854507	16	SYMPK
chr2:9910735-9919820	14	TAF1B
chr20:13559007-13580981	117	TASP1
chr11:12764178-12795367	16	TEAD1
chr4:38986335-38998909	4	TMEM156
chr6:11565665-11567535	14	TMEM170B
chr19:16679983-16680581	10	TMEM38A
chr8:91008742-91021404	2	TMEM55A
chr7:128997388-129015135	14	TNPO3
chr8:140347756-140405698	25	TRAPPC9
chr17:59079753-59088407	2	TRIM37
chr8:58434394-58439770	0	UBXN2B
chr3:179701007-179721589	0	USP13
chr2:58048826-58139832	16	VRK2
chr6:169582835-169627708	14	WDR27
chr7:5199575-5214704	16	WIPI2
chr6:38061592-38116739	8	ZFAND3
chr6:57124254-57142095	16	ZNF451
chr15:64499292-64623052	6	ZNF609
chr15:51563380-51576181	18	DMXL2

chr16:68121986-68126610	1839	NFATC3
chr3:8935870-8941804	323	RAD18
chr13:23353784-23365277	14	SACS
chr2:28926467-28929710	14	WDR43
chr19:44007010-44010397	14	ZNF230
chr19:32615657-32625966	43	ANKRD27
chr17:66129745-66183307	8	CEP112
chr17:6589778-6590630	14	KIAA0753
chr6:89751430-89762530	264	MDN1
chr7:6634970-6636235	29	nogene
chr14:35721687-35742565	14	RALGAPA1
chr12:120782654-120791557	35	SPPL3
chr2:219601715-219601966	14	STK11IP
chr9:77381975-77407607	131	VPS13A
chr9:100470634-100516771	21	TMEFF1
chr11:73707419-73730823	82	RAB6A
chr19:11175901-11176817	0	KANK2
chr14:57209566-57229881	18	EXOC5
chr22:16611657-16614178	81	TPTEP1
chr11:47486749-47500932	17	CELF1
chr11:67215339-67215949	3	KDM2A
chr3:11358417-11477268	3	ATG7
chr16:47463898-47515601	89	PHKB
chr7:128579998-128592017	76	nogene
chr10:103091563-103094455	4	NT5C2
chr8:108229028-108240098	34	nogene
chr10:102872447-102874661	26	AS3MT
chr2:144141881-144145739	4	GTDC1
chr1:202909009-202911203	0	KLHL12
chr3:131462689-131471279	15	MRPL3
chr8:53769617-53772167	23	ATP6V1H
chr19:47750177-47752607	0	GLTSCR2
chr8:124516610-124517185	25	nogene
chr5:72848384-72855923	0	TNPO1
chr9:79612655-79654075	142	TLE4
chr21:36208714-36212097	14	DOPEY2
chr3:120002045-120093593	3	GSK3B
chr17:46170854-46172232	523	KANSL1
chr13:30227411-30255615	61	KATNAL1
chr1:32154587-32158361	16	KPNA6
chr11:96091891-96093517	47	MAML2
chr17:36509061-36511455	236	MYO19
chr6:75886003-75886994	20	MYO6

chr2:183001952-183003325	82	NCKAP1
chr19:35763790-35764373	18	nogene
chr11:3691346-3695606	7	NUP98
chr1:228213440-228215829	0	OBSCN
chr8:100706650-100706997	227	PABPC1
chr7:105142007-105146658	0	SRPK2
chr3:11807832-11829864	6	TAMM41
chr2:74046277-74048411	104	TET3
chrX:47887998-47895940	5	ZNF81
chr20:49074784-49091022	59	CSE1L
chr19:5616131-5616486	25	SAFB2
chr11:65504819-65505019	5	nogene
chr11:62533660-62536567	14	AHNAK
chr1:113901235-113901885	14	AP4B1
chr2:27221217-27226962	14	CAD
chr4:184631036-184638468	0	CASP3
chr3:48174283-48183030	14	CDC25A
chr9:123757702-123769563	54	DENND1A
chr7:103319755-103322632	14	DNAJC2
chr2:232556415-232567214	12	EIF4E2
chr5:176486354-176489027	0	FAF2
chr8:42906172-42964474	14	HOOK3
chr1:63478763-63508570	114	ITGB3BP
chr12:48659904-48660619	157	KANSL2
chr8:17331149-17349081	14	MTMR7
chr12:78021746-78059115	91	NAV3
chr10:12368607-12369147	18	nogene
chr11:65436537-65444063	126	nogene
chr2:183151507-183182680	48	NUP35
chr1:113724758-113726574	40	PHTF1
chr3:196069454-196077122	18	TFRC
chr17:68429995-68434626	0	WIPI1
chr4:343781-344430	30	ZNF141
chr15:44328684-44380976	91	CASC4
chr3:121468304-121473487	17	POLQ
chr19:41220858-41221107	20	AXL
chr19:32948265-32953801	25	CEP89
chr7:8003907-8024814	40	GLCCI1
chr17:80048582-80050283	46	CCDC40
chr5:112234633-112266330	18	EPB41L4A
chr18:36834319-36959435	21	KIAA1328
chr7:65726770-65732055	2	LOC441242
chrX:10523091-10567603	1	MID1

chr16:68157868-68174514	26	NFATC3
chr7:92489292-92489911	41	PEX1
chr19:9566335-9569082	47	ZNF121
chr5:14741826-14758598	89	ANKH
chr6:41840892-41891875	2	USP49
chr19:29971192-30005708	28	URI1
chr14:30633946-30638247	30	SCFD1
chr14:50741581-50744365	3	NIN
chr10:75882655-75883525	13	C10orf11
chr8:123019194-123030716	93	DERL1
chr8:108228266-108240075	0	EIF3E
chr14:49709158-49743805	1	KLHDC1
chr2:33257442-33280158	14	LTBP1
chr19:45021082-45021669	14	nogene
chr1:219970120-219970531	14	nogene
chr17:64644163-64644470	14	nogene
chr5:115539664-115542901	11	nogene
chr5:132591223-132595810	21	RAD50
chr15:66502611-66503164	14	RPL4
chr7:98915722-98917679	35	TRRAP
chr9:128325142-128325881	18	COQ4
chr1:43538197-43553941	86	PTPRF
chr11:70354300-70356254	280	PPFIA1
chr8:123333877-123336532	297	ATAD2
chr3:197847408-197854445	1	LRCH3
chr6:75840584-75841378	77	MYO6
chr1:156755536-156767495	0	nogene
chr8:140252776-140360193	0	TRAPPC9
chr4:73090666-73097272	0	ANKRD17
chr12:48083590-48101516	7	SENP1
chr21:26021839-26112146	0	APP
chrX:63643979-63678572	1	ARHGEF9
chr15:59104693-59107670	0	CCNB2
chr3:170280226-170281968	0	PRKCI
chr4:176711498-176740765	28	VEGFC
chr15:84646183-84653699	0	WDR73
chr6:56535121-56536940	0	DST
chr1:241896958-241909063	0	nogene
chr1:6616756-6620337	2	PHF13
chr6:79042842-79063910	2	PHIP
chr10:97743093-97744915	11	ZFYVE27
chr12:131753122-131756644	17	SFSWAP
chr16:53235184-53247503	3	CHD9

chr11:73707419-73718890	685	RAB6A
chr19:1270866-1272051	2	CIRBP
chr11:73000989-73015885	77	FCHSD2
chr10:87059149-87062835	1	GLUD1
chr3:188490421-188524787	20	LPP
chr17:50979745-50987237	7	SPAG9
chr15:43021274-43025429	97	UBR1
chr1:40514514-40515918	4	EXO5
chr16:1724556-1729578	38	MAPK8IP3
chr8:144160969-144200541	2	MROH1
chr5:6616726-6625669	26	NSUN2
chr8:39090046-39091346	10	ADAM9
chr14:34775918-34783232	15	BAZ1A
chr1:9755113-9756510	15	CLSTN1
chr11:108673466-108723462	21	DDX10
chr13:107598840-107789110	15	FAM155A-IT1
chr5:74780362-74841679	20	FAM169A
chr11:62626859-62627353	15	GANAB
chr10:133398434-133399619	6	MTG1
chr6:111469195-111474476	15	nogene
chr3:196257787-196270541	6	PCYT1A
chr16:71658232-71658815	16	PHLPP2
chr12:122508217-122508447	15	RSRC2
chr15:76504858-76665789	1	SCAPER
chr17:77402058-77402703	335	9-Sep
chr11:62824165-62825539	0	STX5
chr21:32694227-32702047	20	SYNJ1
chr13:75326196-75362607	1	TBC1D4
chr11:11903262-11920494	15	USP47
chr8:134564986-134610655	0	ZFAT
chr6:57124733-57134870	39	ZNF451
chr19:37150834-37170054	15	ZNF585A
chr10:27060344-27061242	3	ANKRD26
chr3:37281957-37302332	61	GOLGA4
chr15:65016435-65023794	0	MTFMT
chr10:28595732-28596041	19	WAC
chr1:176135009-176163891	18	RFWD2
chr15:72771782-72775097	172	ADPGK
chr13:31269213-31286819	31	B3GLCT
chr8:42048377-42049707	332	KAT6A
chr3:197090911-197119530	396	DLG1
chr1:43538197-43569778	28	PTPRF
chr2:61522610-61533903	1658	XPO1

chr5:40852173-40854096	0	CARD6
chr5:128134164-128141981	0	SLC12A2
chr17:42211983-42217464	0	STAT5B
chr15:50449399-50459162	74	USP8
chr6:36593088-36593371	16	nogene
chr7:100355142-100356883	0	STAG3L5P-PVRIG2P-PILRB
chr16:11846500-11846843	92	RSL1D1
chr4:80583092-80648987	0	C4orf22
chr6:5368549-5404701	29	FARS2
chr17:47663088-47668410	248	KPNB1
chr6:75879819-75886994	1	MYO6
chr9:109050282-109064567	150	TMEM245
chr18:24123833-24125550	7	TTC39C
chr16:1685438-1698843	0	HN1L
chr2:32488587-32493667	96	BIRC6
chr10:130079565-130079901	0	LINC00959
chr4:148152468-148154901	11	NR3C2
chr1:112581991-112584126	14	ST7L
chr14:51568349-51570410	4	FRMD6
chr1:233090060-233095863	182	PCNXL2
chr14:21211809-21263336	5	HNRNPC
chr8:43064100-43077364	40	FNTA
chr22:23791755-23793688	41	SMARCB1
chr10:68142939-68161752	38	MYPN
chr4:139071865-139073567	29	ELF2
chr4:165310552-165310750	55	KLHL2
chr8:140311247-140360193	0	TRAPPC9
chr22:32478980-32479275	296	FBXO7
chr4:47875126-47910994	28	NFXL1
chr13:24475471-24478276	1189	PARP4
chr8:43158574-43172386	18	HGSNAT
chr6:44113886-44116629	1902	MRPL14
chr16:24540611-24553557	14	RBBP6
chr4:113232160-113242209	0	ANK2
chr14:92096092-92096838	31	ATXN3
chr5:119105179-119121139	18	DMXL1
chr16:2234170-2235001	0	E4F1
chr12:1027747-1116033	16	ERC1
chr5:179617513-179617911	0	HNRNPH1
chr10:37735131-37739714	40	nogene
chr18:51058124-51059916	7	SMAD4
chr3:196073929-196075360	113	TFRC
chr15:70690453-70699660	0	UACA

chr15:74655732-74675142	24	EDC3
chr11:85996825-86007583	11	PICALM
chr6:100644030-100647451	20	ASCC3
chr21:29327280-29329693	8	BACH1
chr2:214728917-214769312	14	BARD1
chr4:184398914-184429070	1	IRF2
chr7:152247901-152252715	14	KMT2C
chr10:17700190-17705741	14	STAM
chr7:22492564-22495480	58	STEAP1B
chr3:67495797-67520634	5	SUCLG2
chr6:167890861-167902386	2	MLLT4
chr11:66816052-66822674	16	C11orf80
chr14:80895717-80906081	248	CEP128
chr3:197104902-197119530	864	DLG1
chr1:153635129-153635523	13	nogene
chr19:40623603-40624082	78	LTBP4
chr11:78528841-78571444	75	NARS2
chr9:105534492-105539351	37	FSD1L
chr19:15170085-15170825	5	NOTCH3
chr19:44118187-44118574	0	ZNF225
chr7:66551524-66576932	1	GS1-124K5.11
chr5:73832337-73852692	110	ARHGEF28
chr13:23858859-23870195	68	MIPEP
chr22:37806220-37806579	13	nogene
chr10:27158548-27160138	1	MASTL
chr2:203138161-203138748	2	NBEAL1
chr2:27599582-27601442	230	ZNF512
chr6:73730343-73736508	1	CD109
chr1:147252622-147268878	15	CHD1L
chr2:9428764-9443661	0	CPSF3
chr10:12084539-12091684	0	DHTKD1
chr15:49319603-49328130	0	GALK2
chr1:186451178-186470395	29	LOC102724919
chr5:171394451-171394902	27	nogene
chr11:66657135-66661335	0	nogene
chr12:68648698-68654252	10	RAP1B
chr9:96392397-96398314	47	ZNF367
chr12:11121009-11124187	67	nogene
chr6:34967238-34970166	0	ANKS1A
chr10:72203426-72213331	2	ASCC1
chr2:32478634-32500109	28	BIRC6
chr18:79728906-79736521	73	CTDP1
chr11:126272968-126273454	12	FOXRED1

chr2:37049652-37053607	0	HEATR5B
chr1:117405548-117420649	6	MAN1A2
chr18:58165787-58252054	14	NEDD4L
chr2:185950643-185952902	4	nogene
chr3:52112698-52138302	0	POC1A
chr19:55236642-55247109	0	PPP6R1
chr10:16752538-16754989	121	RSU1
chrX:135994785-135998968	20	SLC9A6
chr1:162599270-162601514	16	UAP1
chr10:86453219-86460496	4	WAPL
chr11:70419745-70425401	1	CTTN
chr20:35801490-35871829	4	PHF20
chrX:123888618-123892773	25	XIAP
chr13:46356431-46359631	0	KIAA0226L
chr6:44932652-45014891	14	SUPT3H
chr11:120447873-120475507	16	ARHGEF12
chr1:179393902-179395202	16	AXDND1
chr10:68749135-68749685	5	CCAR1
chr4:99908710-99923185	1	DNAJB14
chr11:72984087-73015885	36	FCHSD2
chr6:35597247-35642843	10	FKBP5
chr10:87049400-87051870	1	GLUD1
chr8:28970042-28980156	14	HMBOX1
chr2:85027888-85049648	29	KCMF1
chr2:31883385-31943383	31	MEMO1
chr5:151795236-151796312	16	nogene
chr1:96769702-96785254	16	PTBP2
chr19:7267344-7267896	2	INSR
chr5:141638794-141640445	5	RELL2
chr21:26089942-26112146	25	APP
chr6:110667433-110746201	63	CDK19
chr11:103215720-103223086	4	DYNC2H1
chr5:142135729-142140629	2	NDFIP1
chr10:15128349-15130312	29	NMT2
chr9:100052416-100060172	41	ERP44
chr5:43294690-43298134	0	HMGCS1
chr19:1510149-1510917	75	ADAMTSL5
chr1:35913841-35914274	13	AGO1
chr21:25954589-26000182	6	APP
chr14:102959630-102964650	17	CDC42BPB
chr9:120467859-120471878	13	CDK5RAP2
chr4:1225359-1241519	1010	CTBP1
chr10:127018709-127061776	24	DOCK1

chr8:116726015-116737287	42	EIF3H
chr20:34440154-34457474	19	ITCH
chr17:16149450-16171995	16	NCOR1
chr17:18704618-18705164	61	nogene
chr10:103427328-103434975	13	PDCD11
chr21:34094862-34125480	17	SLC5A3
chr18:51046919-51067187	49	SMAD4
chr12:64042789-64043575	14	SRGAP1
chr4:6923372-7001251	64	TBC1D14
chr14:67806544-67809289	3	ZFYVE26
chr11:74132843-74139828	46	C2CD3
chr2:235750353-235807331	129	AGAP1
chr2:222630112-222648795	20	FARSB
chr10:21713771-21727928	116	MLLT10
chr2:119317465-119340361	13	C2orf76
chr9:137743391-137758011	13	EHMT1
chr9:2804256-2812362	39	KIAA0020
chr13:20731840-20732121	17	N6AMT2
chr2:70051202-70085903	13	PCBP1-AS1
chr3:50093719-50105709	23	RBM5
chr3:184913518-184920198	13	VPS8
chr12:111792060-111792782	0	ALDH2
chr11:100941783-100949956	13	ARHGAP42
chr8:1876570-1885707	77	ARHGEF10
chr3:93995873-94039988	13	ARL13B
chr12:111509548-111513539	13	ATXN2
chr13:36248605-36256167	0	CCDC169-SOHLH2
chr7:6430479-6436533	25	DAGLB
chrX:2291501-2425304	0	DHRSX
chr7:103321931-103322794	0	DNAJC2
chr5:126914929-126918227	77	3-Mar
chr12:65306862-65369026	6	MSRB3
chr20:32930397-32931336	42	nogene
chr2:97490434-97491145	13	nogene
chr11:73909398-73919032	8	PAAF1
chr3:52678499-52682233	9	PBRM1
chr3:196257787-196283538	10	PCYT1A
chr1:20648314-20649353	24	PINK1-AS
chr1:151139424-151140090	2	SEMA6C
chr7:99051356-99052446	0	SMURF1
chr17:58999380-59017424	32	TRIM37
chr9:6477621-6500709	0	UHRF2
chr3:179719939-179730709	72	USP13

chr16:3977481-3983440	24	ADCY9
chr16:56401732-56414344	65	AMFR
chrX:77557450-77593849	117	ATRX
chr6:105115685-105116868	19	BVES
chr17:82127691-82134194	28	CCDC57
chr1:243191017-243225321	13	CEP170
chr15:78593091-78593628	30	CHRNA5
chr17:7448578-7448788	7	CHRNB1
chr16:58585337-58599511	48	CNOT1
chr2:210556718-210582709	13	CPS1
chr7:6432788-6435020	0	DAGLB
chr2:227893794-227898281	4	DAW1
chr3:98881539-98892606	12	DCBLD2
chrX:154764898-154770879	21	DKC1
chr12:63644402-63647365	161	DPY19L2
chr16:22225653-22225975	218	EEF2K
chr4:139137629-139168042	0	ELF2
chr20:46380252-46394532	13	ELMO2
chr5:176486354-176496663	5	FAF2
chr10:5726884-5735546	14	FAM208B
chr11:72981277-72989097	0	FCHSD2
chr2:53728711-53750941	10	GPR75-ASB3
chr10:67992198-67992682	7	HERC4
chr2:38573209-38591648	1	HNRNPLL
chr9:6849506-6893232	36	KDM4C
chrX:45010960-45020730	36	KDM6A
chr10:96907264-96949295	31	LCOR
chr9:22723308-22768403	2	LINC01239
chr3:139398860-139423114	14	LOC100507291
chr4:150798080-150849575	36	LRBA
chr2:39299742-39333574	13	MAP4K3
chr7:24623665-24649764	2	MPP6
chr13:41316845-41323190	13	NAA16
chr3:27162700-27174833	0	NEK10
chr16:47109482-47134410	117	NETO2
chr11:102350345-102378157	0	nogene
chr17:15503097-15516659	1	nogene
chr19:19133738-19134358	21	nogene
chr4:4086657-4087339	40	nogene
chr6:129502604-129540542	0	nogene
chr3:38198721-38236961	6	OXSR1
chr19:39168229-39169757	13	PAK4
chr7:11051611-11111467	19	PHF14

chr6:79015081-79026164	42	PHIP
chr2:189794848-189795951	13	PMS1
chr18:9570156-9577191	20	PPP4R1
chr14:61443110-61457679	3	PRKCH
chr8:18867673-18872733	50	PSD3
chr12:121416158-121417911	76	RNF34
chr1:92320598-92324375	33	RPAP2
chr19:5641593-5641946	142	SAFB
chr19:5604582-5616486	14	SAFB2
chr12:123323679-123330509	0	SBNO1
chr21:33559229-33569087	7	SON
chr3:136443286-136464988	16	STAG1
chr17:31993241-31995762	25	SUZ12
chr15:78044899-78054187	81	TBC1D2B
chr5:1279290-1282624	14	TERT
chr9:100308499-100321771	13	TEX10
chr21:31164961-31203012	55	TIAM1
chr1:179846106-179852811	13	TOR1AIP2
chr7:23506137-23507535	0	TRA2A
chr5:14270824-14316743	51	TRIO
chr3:3137259-3140648	118	TRNT1
chr17:3580456-3592383	0	TRPV1
chr3:41835863-41935940	14	ULK4
chr2:85619219-85630946	16	USP39
chr6:169582835-169602321	13	WDR27
chr3:101652495-101654821	5	ZBTB11
chr3:141368620-141405887	24	ZBTB38
chr11:110136662-110153058	21	ZC3H12C
chr1:52477707-52489035	2	ZCCHC11
chr10:97744728-97753182	1	ZFYVE27
chr8:130358016-130361771	3239	ASAP1
chr13:59911734-59916249	16	DIAPH3
chr1:28010946-28017239	34	EYA3
chr9:113198854-113206578	13	FKBP15
chr3:52912537-52913617	3	SFMBT1
chr20:49264715-49266266	48	ZNFX1
chr16:56470002-56470791	6	OGFOD1
chr16:58574608-58575129	61	CNOT1
chr6:13639562-13657276	24	RANBP9
chr2:73557219-73559142	83	ALMS1
chr7:2362644-2363760	16	EIF3B
chr3:32441839-32454542	61	CMTM7
chr22:32406661-32408833	0	RTCB

chr2:121582156-121582823	19	nogene
chr4:2662837-2672372	48	FAM193A
chr1:47368468-47376872	1	CMPK1
chr5:179709872-179724783	20	CANX
chr7:23345939-23361741	30	IGF2BP3
chr14:31562067-31787873	0	NUBPL
chr7:72938589-72939968	0	POM121
chr12:125102678-125114557	3	AACS
chr16:70264957-70268379	14	AARS
chr10:68786135-68786692	29	CCAR1
chr2:101257790-101262691	4	CNOT11
chr10:5794884-5800705	1184	GDI2
chr14:76177987-76180849	13	GPATCH2L
chr14:21211405-21234229	14	HNRNPC
chr6:73077321-73133641	13	KCNQ5
chr7:134137022-134158159	0	LRGUK
chr9:137646335-137651037	13	nogene
chr13:26593607-26613538	0	nogene
chr15:91915992-91916458	17	SLCO3A1
chr10:17771308-17776170	0	TMEM236
chr14:52511239-52544371	75	TXNDC16
chr9:83682950-83686155	144	UBQLN1
chr1:165890203-165891322	357	UCK2
chr19:47086383-47090189	13	ZC3H4
chr1:244477540-244518698	0	C1orf101
chr17:16139007-16143696	50	NCOR1
chr3:149846010-149895570	1	RNF13
chr14:90644047-90658387	3	TTC7B
chr3:187038741-187051346	30	ST6GAL1
chr7:67098540-67101430	29	TYW1
chr12:111679150-111683307	58	BRAP
chr2:69854822-69864975	1	GMCL1
chr3:188609160-188609844	94	LPP
chr18:58165787-58333892	1	NEDD4L
chr8:124490432-124490892	46	nogene
chr9:41025763-41027092	5	PGM5P2
chr5:170892386-170924550	86	RANBP17
chr1:2395783-2399514	2	RER1
chr18:163307-204692	1	USP14
chr18:196636-199316	110	USP14
chr3:195301574-195302174	76	ACAP2
chr7:107248395-107298346	0	COG5
chr15:94356136-94385525	0	MCTP2

chr7:28101618-28105070	3	nogene
chr2:128097428-128104014	36	UGGT1
chrX:63343205-63345571	1	nogene
chr3:17372074-17376613	1	TBC1D5
chr2:27987992-28051648	0	BRE
chr15:75407054-75414311	1	SIN3A
chr21:39236594-39250889	15	BRWD1
chr17:30792439-30804108	9	CRLF3
chr12:132795851-132813419	15	GOLGA3
chr22:35419883-35423341	15	MCM5
chr9:69220886-69221496	15	TJP2
chr11:120431770-120437382	2	ARHGEF12
chr5:154871729-154872651	33	CNOT8
chr22:50372019-50406875	34	PPP6R2
chr11:119273867-119285566	26	CBL
chr2:169544720-169557297	108	FASTKD1
chr6:169672254-169689308	27	WDR27
chr11:66485172-66488007	0	DPP3
chr6:82210813-82211572	12	IBTK
chr12:111509548-111555919	16	ATXN2
chr7:1936686-1980541	34	MAD1L1
chr17:7563065-7570933	1	SENP3-EIF4A1
chr7:43595900-43626586	0	STK17A
chr19:5604582-5604936	616	SAFB2
chr12:124863594-124913774	14	SCARB1
chr3:196071395-196077122	23	TFRC
chr5:150010110-150018697	12	HMGXB3
chr10:13172375-13175681	71	MCM10
chr10:4830674-4908420	38	AKR1E2
chr1:243563719-243573048	1	AKT3
chr8:130214555-130401970	29	ASAP1
chr14:35832977-35834923	0	BRMS1L
chr11:66759042-66788269	1	C11orf80
chr12:4517060-4525583	16	C12orf4
chr1:186406082-186419077	5	C1orf27
chrX:101101057-101120784	27	CENPI
chr4:186633683-186639783	13	FAT1
chr11:72849754-72868026	0	FCHSD2
chr5:43292473-43297673	0	HMGCS1
chr4:150761782-150808398	101	LRBA
chr20:51516783-51523596	13	NFATC2
chrX:71300222-71300708	23	nogene
chr3:122536167-122540852	2	PARP9

chr9:41039610-41053899	7	PGM5P2
chr10:97656284-97656974	8	PI4K2A
chr4:105446382-105453642	13	PPA2
chr7:66775226-66795592	0	RABGEF1
chr20:37040152-37047190	32	RBL1
chr20:49945381-49949355	115	RNF114
chr3:49691129-49704756	0	RNF123
chr22:41866830-41871035	4	SREBF2
chr2:135313488-135315530	14	ZRANB3
chr18:14262074-14265668	0	nogene
chr8:123333877-123339446	24	ATAD2
chr14:21426127-21429335	74	CHD8
chr17:39439164-39443628	52	MED1
chr11:108301647-108304852	87	ATM
chr4:40934455-40945070	86	APBB2
chr2:206127796-206130242	387	NDUFS1
chr9:33351559-33352719	90	NFX1
chr20:34029768-34031604	372	RALY
chr12:109102838-109103611	74	UNG
chr2:227491546-227494933	0	AGFG1
chr7:4740837-4741023	18	FOXK1
chr15:71999850-72046634	29	MYO9A
chr12:14612878-14619916	3	PLBD1-AS1
chr6:129611532-129618852	28	ARHGAP18
chr17:61058503-61084564	0	BCAS3
chr9:94923985-94928531	34	C9orf3
chr5:115236389-115253255	73	PGGT1B
chr5:136153591-136154163	79	SMAD5
chr10:84380097-84477664	24	CCSER2
chr21:45175729-45204736	19	ADARB1
chr15:89801110-89806809	1	ANPEP
chr10:72196810-72213331	79	ASCC1
chr9:114235598-114243560	1	COL27A1
chr14:39313339-39327022	38	CTAGE5
chr3:186786501-186787264	53	EIF4A2
chr9:20881870-20933132	2	FOCAD
chr7:138894341-138912460	42	KIAA1549
chr6:13639562-13658832	21	RANBP9
chr17:2186662-2188515	30	SMG6
chr6:42632551-42637194	19	UBR2
chr21:43669883-43673955	26	RRP1B
chr2:69505568-69643274	13	AAK1
chr2:203355153-203402734	13	ABI2

chr3:154187684-154217958	13	ARHGEF26
chr11:46667772-46669532	0	ATG13
chr1:235458602-235470960	6	B3GALNT2
chr14:105252506-105286376	25	BRF1
chr21:41942146-41943752	13	C2CD2
chr2:29167475-29197036	1	CLIP4
chr1:41001028-41003176	13	CTPS1
chr7:66573384-66576929	94	GS1-124K5.11
chr10:67932596-67936235	17	HERC4
chr12:122597730-122624688	13	KNTC1
chr13:48077152-48090418	21	MED4
chr4:153350063-153409015	13	MND1
chr1:28480450-28480604	13	PHACTR4
chr9:95476033-95485874	13	PTCH1
chr19:32990513-32996220	2	RHPN2
chr18:70086612-70114599	0	RTTN
chr3:170359698-170384765	13	SKIL
chr20:447888-448074	0	TBC1D20
chr16:67820838-67821225	13	TSNAXIP1
chr2:128183674-128187614	38	UGGT1
chr3:179730188-179730709	13	USP13
chr20:49264715-49271750	0	ZNFX1
chr2:23875828-23888399	28	ATAD2B
chr18:13008455-13030608	13	CEP192
chr6:43206006-43206510	46	CUL9
chr2:232014834-232087721	18	DIS3L2
chr17:49716164-49717714	0	FAM117A
chr15:49281986-49292537	0	GALK2
chr16:74451054-74459789	13	GLG1
chr6:24456497-24472636	13	GPLD1
chr10:17599289-17604047	13	HACD1
chr15:63745726-63747858	13	HERC1
chr5:133091192-133092789	348	HSPA4
chr7:140113490-140120529	0	KDM7A
chr1:32036909-32045390	0	KHDRBS1
chr18:36959315-37067545	13	KIAA1328
chr11:28077006-28094800	16	KIF18A
chr19:11110651-11113762	0	LDLR
chr3:37094791-37129149	0	LRRFIP2
chr22:20551435-20568631	13	MED15
chr21:36338778-36344707	414	MORC3
chr17:17175292-17177412	2	MPRIP
chr12:27714779-27737608	100	MRPS35

chr2:203175146-203188589	18	NBEAL1
chr5:137757866-137759020	27	nogene
chr18:8731496-8731754	31	nogene
chr5:60962740-60962980	13	nogene
chr19:1005754-1006098	20	nogene
chr3:23954536-23956125	7	NR1D2
chr14:67692437-67693052	13	RDH11
chr4:75969295-75971458	13	SDAD1
chr11:62853800-62855466	0	SNHG1
chrX:71421308-71424238	64	TAF1
chr8:123109316-123120188	13	TBC1D31
chr15:64406329-64409828	13	TRIP4
chr10:86470991-86473973	61	WAPL
chr19:18573292-18573748	100	UBA52
chr10:125110989-125111882	2	CTBP2
chr5:176350612-176355749	29	KIAA1191
chr14:74672830-74676752	1	AREL1
chr3:136980465-136995556	45	IL20RB
chrX:80288905-80310233	160	CHMP1B2P
chr9:128185546-128187934	52	CIZ1
chr1:85029105-85045362	1	MCOLN3
chr4:2596083-2596329	16	nogene
chr7:44425899-44427703	96	NUDCD3
chr1:151264831-151267339	0	PSMD4
chr1:21001198-21050994	42	EIF4G3
chr2:203366876-203394846	0	ABI2
chr4:142260491-142305537	4	INPP4B
chr15:63690540-63694894	1	HERC1
chr22:29636750-29639212	110	NF2
chr14:96833466-96876120	20	VRK1
chr2:230169046-230170761	0	SP110
chr2:215312497-215349249	0	ATIC
chr4:22375729-22392690	14	ADGRA3
chr14:76171842-76180849	74	GPATCH2L
chr6:4999808-5002245	14	RPP40
chr12:56169528-56170208	17	SMARCC2
chr2:58048826-58123233	15	VRK2
chr17:37243370-37246976	2	ACACA
chr5:75653141-75666993	0	ANKDD1B
chrX:65075473-65113813	368	nogene
chr19:37738902-37740194	24	ZNF573
chr11:992502-994245	19	AP2A2
chr1:156976982-156986173	49	ARHGEF11

chr2:214767481-214781509	159	BARD1
chr16:69136695-69143389	98	CIRH1A
chr4:77742140-77756951	2	CNOT6L
chr10:484780-486530	81	DIP2C
chr1:44308309-44352925	0	ERI3
chr10:92504793-92508205	14	IDE
chr9:85822879-85840849	2	LOC389765
chr18:58696365-58700591	206	MALT1
chr12:62493914-62501698	3	MON2
chr4:139334173-139341069	13	NAA15
chr2:182952404-182953331	68	NCKAP1
chr12:32729737-32730134	13	nogene
chr12:67318683-67349398	45	nogene
chr11:69316833-69341678	26	nogene
chr7:9708000-9719071	20	nogene
chr3:179198749-179230376	13	PIK3CA
chr7:108479183-108491467	1	PNPLA8
chr20:34017409-34031604	4	RALY
chr5:171213618-171242820	16	RANBP17
chr3:78714396-78717883	53	ROBO1
chr17:80730559-80791509	4	RPTOR
chr4:109530288-109533685	15	SEC24B
chr10:7276891-7285954	698	SFMBT2
chr17:19542392-19551468	68	SLC47A1
chr9:2493074-2539552	13	VLDLR-AS1
chr1:12400180-12403973	13	VPS13D
chr3:142355400-142365366	13	XRN1
chr13:19835476-19837821	13	ZMYM5
chr20:47262287-47283648	40	ZMYND8
chr5:119101934-119116336	42	DMXL1
chr10:68142939-68148467	7	MYPN
chr6:42603587-42617507	2	UBR2
chr17:29467144-29482288	13	TAOK1
chr1:154227281-154228725	3	UBAP2L
chr19:4475274-4475583	25	HDGFRP2
chr12:7403534-7436602	26	CD163L1
chr5:647804-655584	23	CEP72
chr4:103547853-103552602	6	nogene
chr12:27368261-27403541	63	ARNTL2
chr8:53743576-53756656	0	ATP6V1H
chr8:90017123-90021056	0	DECR1
chr22:31758545-31768863	62	DEPDC5
chr22:31447425-31450360	56	EIF4ENIF1

chr4:165238777-165263359	16	KLHL2
chr7:152309965-152330742	14	KMT2C
chr9:137255906-137272222	0	NELFB
chr5:137757866-137758314	0	nogene
chr1:117112094-117120901	0	nogene
chr13:24494572-24498229	0	PARP4
chr11:14771936-14819209	19	PDE3B
chr7:77893607-77901920	0	PHTF2
chr10:73444724-73454489	0	PPP3CB
chr1:213117316-213129889	96	RPS6KC1
chr3:196899665-196900412	31	SENP5
chr5:33460901-33463825	54	TARS
chr20:18297984-18306393	25	ZNF133
chr16:71745143-71746692	19	AP1G1
chr11:108248932-108257606	1	ATM
chr15:22903705-22912275	14	CYFIP1
chr1:197583151-197611976	31	DENND1B
chr11:33591236-33618662	13	KIAA1549L
chr6:80326536-80353897	14	nogene
chr3:37010833-37011224	13	nogene
chr5:134847571-134848380	13	nogene
chr19:3965540-3965774	15	nogene
chr19:43181361-43259688	5	nogene
chr19:35762018-35762356	38	PROSER3
chr16:24553512-24556447	0	RBBP6
chr1:176116623-176184692	17	RFWD2
chr18:2771532-2775924	14	SMCHD1
chr17:63340100-63355390	7	TANC2
chr17:64193558-64195095	3	TEX2
chr2:61481184-61485962	13	XPO1
chr2:71355718-71368528	0	ZNF638
chr16:67036638-67082339	603	CBFB
chr1:24919239-24929852	15	RUNX3
chr4:142402937-142462726	7	INPP4B
chr6:157110471-157167185	3	ARID1B
chr10:32469624-32474023	0	CCDC7
chr3:96972606-96987993	9	EPHA6
chr9:13216773-13224583	2	MPDZ
chr5:80767932-80792844	134	MSH3
chr1:236850343-236863554	14	MTR
chr18:58341037-58343103	11	NEDD4L
chr7:96156839-96157550	13	nogene
chr13:28669590-28669897	13	nogene

chr11:77332729-77349287	1	PAK1
chr13:41907585-41954024	1	VWA8
chr2:71396140-71418639	356	ZNF638
chr19:32714249-32714692	18	nogene
chr17:37283266-37284970	4	ACACA
chr18:9182381-9211784	2	ANKRD12
chr12:63644402-63663845	78	DPY19L2
chr9:86033281-86040868	26	GOLM1
chr1:172817552-172851482	2	nogene
chr9:123169901-123173842	15	STRBP
chr4:87183202-87195690	39	KLHL8
chr20:36244696-36244926	15	AAR2
chr22:29349217-29351266	0	AP1B1
chr17:60460662-60461915	0	APPBP2
chr2:43912431-43925961	144	LRPPRC
chr15:41291117-41291523	6	nogene
chr9:128600079-128605477	16	SPTAN1
chr8:98703086-98707311	0	STK3
chr6:57379900-57402268	7	PRIM2
chr12:109980951-109983694	11	GIT2
chr5:154790324-154795319	9	LARP1
chr12:56562416-56571855	282	RBMS2
chr19:41419134-41423169	1	BCKDHA
chr15:99287256-99334129	1	LRRC28
chr7:2437803-2439330	7	nogene
chr20:35801490-35842744	5	PHF20
chr8:140283888-140311374	0	TRAPPC9
chr17:56844018-56845809	54	DGKE
chr16:89977638-89978878	1	AFG3L1P
chr8:1880047-1903451	13	ARHGEF10
chr6:100715461-100800504	13	ASCC3
chr2:151841667-151842088	15	CACNB4
chr12:7430205-7433694	2	CD163L1
chr1:160313989-160325652	1	COPA
chr7:87163494-87181341	13	DMTF1
chr2:201016990-201022932	0	FAM126B
chr2:69356495-69363670	19	GFPT1
chr5:154790324-154800042	36	LARP1
chr21:36333645-36345031	15	MORC3
chr4:118636225-118636517	13	nogene
chr14:73287109-73297285	2	NUMB
chr8:127855154-127939676	13	PVT1
chr1:11013715-11020599	13	TARDBP

chr12:27368261-27377564	151	ARNTL2
chr6:35619095-35637158	26	FKBP5
chr10:68910306-68919981	2	DDX50
chr9:130086419-130092381	2	GPR107
chr2:84839148-84842007	28	TRABD2A
chr15:45040407-45043421	25	SORD
chr11:10028451-10042981	8	SBF2
chr17:82756164-82768566	67	TBCD
chr2:121757239-121763084	103	TSN
chr20:24968891-24973737	3	APMAP
chr6:141829735-141927055	8	nogene
chr14:73083493-73088161	19	RBM25
chr7:38795464-38796864	1	VPS41
chr5:95895627-95919545	14	ELL2
chr7:32489248-32490766	2	LSM5
chr6:43546570-43558591	14	XPO5
chrX:132281094-132283032	19	nogene
chr19:19104912-19106385	7	SLC25A42
chr9:71866798-71875083	107	ABHD17B
chr2:231216637-231240041	4	ARMC9
chr1:1468383-1529331	193	ATAD3C
chr11:66521269-66523882	2	BBS1
chr22:41125863-41131633	6	EP300
chr11:65503120-65504595	3	nogene
chr14:101901718-101906529	45	PPP2R5C
chr6:7176654-7182082	58	RREB1
chr18:63978298-63985245	0	SERPINB8
chr4:94276338-94283303	14	SMARCAD1
chr1:245915529-245929937	58	SMYD3
chr12:77940318-77941133	84	NAV3
chr3:15707923-15797144	13	ANKRD28
chr1:94203929-94205683	15	ARHGAP29
chr6:85636966-85637356	19	SYNCRIP
chr3:196065442-196077122	13	TFRC
chr2:65246523-65251099	4	ACTR2
chr4:40821870-40830577	1	APBB2
chr20:35477870-35478101	15	CEP250
chr1:147252622-147259918	260	CHD1L
chr1:117881822-117886653	18	GDAP2
chr20:43620856-43642624	15	IFT52
chr3:49121432-49121860	0	LAMB2
chr2:43946112-43948518	13	LRPPRC
chr10:21670448-21673919	1	MLLT10

chr4:80287336-80376917	31	nogene
chr14:90285081-90285532	15	nogene
chr22:37856355-37857077	23	nogene
chr12:54165373-54172433	13	nogene
chr7:2442546-2443746	6	nogene
chr12:19269769-19287556	15	PLEKHA5
chr5:75547009-75552591	15	POLK
chr1:8655972-8656441	15	RERE
chr1:40227412-40231658	15	RLF
chr5:40728344-40747019	40	TTC33
chr1:229632268-229654388	15	URB2
chr4:75770438-75774796	0	USO1
chr12:122232325-122232968	3	VPS33A
chr2:43712224-43738520	1	PLEKHH2
chr7:4768392-4770257	20	nogene
chr9:92258853-92265553	14	IARS
chr19:10446217-10450941	36	PDE4A
chr2:9318523-9335179	64	ASAP2
chr1:233353806-233355011	15	KIAA1804
chr9:22466115-22467906	0	nogene
chr19:8241428-8243083	15	nogene
chr22:40953554-40967884	19	RBX1
chr1:64009304-64050716	56	ROR1
chr11:93162705-93170221	21	SLC36A4
chr21:31152635-31154426	15	TIAM1
chr15:29761138-29773357	49	TJP1
chr18:6080880-6171942	0	L3MBTL4
chr17:59737452-59738947	0	VMP1
chr20:63775677-63805464	13	ZBTB46
chr20:34031521-34033239	20	RALY
chr22:38751392-38752506	1	SUN2
chr13:21413651-21425678	31	ZDHHC20
chr21:34859473-34887096	7	RUNX1
chr1:240355815-240438210	17	FMN2
chr5:37213558-37224592	0	C5orf42
chr20:33573973-33611335	0	CBFA2T2
chr18:46118180-46123364	5	HAUS1
chr13:29453067-29485306	13	MTUS2-AS1
chr19:52864032-52879590	13	nogene
chr17:15658801-15683157	3	nogene
chr1:5947103-5986232	13	NPHP4
chr18:62830034-62838909	40	PHLPP1
chr20:35721739-35739017	0	RBM39

chr3:195326884-195345317	144	ACAP2
chr2:203245845-203266681	2	CYP20A1
chr9:123757702-123923415	13	DENND1A
chr1:172851214-172933643	0	nogene
chr5:41762110-41794749	0	OXCT1
chr2:232703423-232761436	56	GIGYF2
chr15:56827523-56917694	3	nogene
chr15:84680712-84687774	62	SEC11A
chr18:42020637-42049605	0	PIK3C3
chr18:31638643-31645479	0	B4GALT6
chr14:69091673-69122360	1	DCAF5
chr2:36441257-36464655	91	CRIM1
chr3:134188836-134195182	135	RYK
chr4:1694773-1696351	4	SLBP
chr1:243221723-243225321	278	CEP170
chr1:20757165-20767664	3	HP1BP3
chr10:87767672-87776427	278	ATAD1
chr1:156324971-156334918	50	CCT3
chr4:110059602-110198367	0	ELOVL6
chr22:32479132-32487828	34	FBXO7
chr8:17989858-18014840	14	PCM1
chr17:1364858-1369741	65	YWHAE
chr16:66727687-66729099	25	DYNC1LI2
chr1:233161270-233208689	0	PCNXL2
chr9:105364554-105366429	0	SLC44A1
chr19:34490375-34495771	62	WTIP
chr12:7423845-7433694	0	CD163L1
chr14:72714422-72723728	0	DPF3
chr20:31862570-31869597	0	DUSP15
chr11:29119510-29211765	0	nogene
chr3:125576188-125582978	13	OSBPL11
chr1:92833544-92834913	0	RPL5
chr15:74852068-74854649	0	SCAMP2
chr3:53229279-53230621	0	TKT
chr5:109037421-109100519	87	FER
chr5:179797664-179801969	5	MGAT4B
chr1:10095460-10105744	130	UBE4B
chr8:134583831-134610655	5	ZFAT
chr13:19993062-20006586	28	ZMYM2
chr4:145514437-145515013	45	SMAD1
chr8:25296512-25300657	1	DOCK5
chr11:107806716-107815965	52	SLC35F2
chr3:33392562-33425741	21	UBP1

chr7:6751866-6752780	153	nogene
chr11:58609829-58616815	13	ZFP91-CNTF
chr7:42148225-42148468	390	GLI3
chr12:31423596-31426424	10	DENND5B
chr9:20819795-20823115	479	FOCAD
chr3:70987993-71053773	13	FOXP1
chr13:44959380-44982154	13	NUFIP1
chr1:201712816-201718755	0	NAV1
chr15:50439008-50441493	1	USP8
chr8:39013964-39025884	0	ADAM9
chr12:350620-352324	45	KDM5A
chr17:31200421-31214585	15	NF1
chr1:115659640-115664268	89	VANGL1
chr1:23055068-23069151	55	KDM1A
chr11:65894013-65897006	815	FOSL1
chrX:103491213-103497613	4	nogene
chr5:14405847-14406672	33	TRIO
chr5:134741062-134741523	59	CAMLG
chr3:136433555-136477412	6	STAG1
chr17:59764970-59817773	31	VMP1
chr10:124021255-124046724	19	CHST15
chr20:32366383-32371874	265	ASXL1
chr11:119675158-119678765	14	PVRL1
chr1:243637610-243843282	13	AKT3
chr5:78141142-78216237	13	AP3B1
chr5:69258042-69262304	80	CDK7
chr1:47372954-47375293	0	CMPK1
chr5:112985834-113001674	158	DCP2
chr7:13931501-13977480	13	ETV1
chr10:102360183-102360395	33	GBF1
chr2:37000585-37020836	0	HEATR5B
chr18:6093354-6312056	0	L3MBTL4
chr6:83768480-83768971	0	nogene
chr4:189855885-189895643	13	nogene
chr5:168510580-168516950	15	RARS
chr6:89379223-89387590	7	RRAGD
chr2:113717752-113735858	0	SLC35F5
chr8:98749275-98774819	58	STK3
chr4:165085585-165100892	21	TMEM192
chr4:1900625-1951203	13	WHSC1
chr11:102186017-102209564	43	YAP1
chr2:70181162-70181997	0	C2orf42
chr16:70312608-70316104	0	DDX19B

chr17:8569719-8577338	21	MYH10
chr7:103103050-103128792	0	NAPEPLD
chr6:17665238-17675770	27	NUP153
chr3:101803644-101807126	0	NXPE3
chr15:43763076-43769646	8	PDIA3
chr7:90747680-90955817	0	CDK14
chr1:217610320-217614202	52	GPATCH2
chr19:57619411-57621493	10	ZNF134
chr12:110381751-110382960	11	ANAPC7
chr1:150492410-150496908	103	TARS2
chr16:1625972-1632365	151	CRAMP1L
chr17:82252433-82265796	40	CSNK1D
chr3:128637798-128638105	5	RPN1
chr4:38035587-38049898	39	TBC1D1
chr9:86305191-86310017	238	ZCCHC6
chr19:47681975-47695474	60	GLTSCR1
chrX:102816991-102839972	26	LINC00630
chr7:4877604-4878203	16	RADIL
chr12:130798524-130808704	0	STX2
chr4:77048448-77058635	0	CCNI
chr10:125038996-125074843	15	CTBP2
chr20:6355479-6356702	20	nogene
chr1:10417997-10418484	25	nogene
chr5:179580946-179589647	46	RUFY1
chr3:195979743-195982310	13	SDHAP1
chr3:47675474-47678311	65	SMARCC1
chr3:49335019-49335596	69	USP4
chr8:134600435-134608879	0	ZFAT
chr9:5924657-5954095	4	KIAA2026
chr11:62638982-62639731	429	GANAB
chr7:6139091-6146248	41	USP42
chr9:71862550-71875083	107	ABHD17B
chr7:40062825-40088331	21	CDK13
chr10:35016191-35031619	18	CUL2
chr12:53016472-53022627	459	EIF4B
chr4:140527465-140543586	0	ELMOD2
chr5:154026398-154034967	74	FAM114A2
chr11:28058925-28062516	0	KIF18A
chr1:117460488-117522937	27	MAN1A2
chr3:47977864-47998879	36	MAP4
chr12:109872855-109873141	1	nogene
chr17:38319328-38319816	16	nogene
chr6:143772257-143774215	28	PHACTR2

chr9:112250928-112297916	275	PTBP3
chr1:19193432-19197265	47	UBR4
chr15:32523028-32524600	13	WHAMMP1
chr22:38493709-38501280	3	DDX17
chr2:178535731-178542361	415	TTN-AS1
chr5:10618332-10619378	25	ANKRD33B
chr2:110666262-110669553	49	BUB1
chr13:23332487-23332913	0	nogene
chr8:140846259-140879637	499	PTK2
chr5:134160986-134174022	0	SKP1
chr3:142777631-142785040	61	TRPC1
chr6:20781144-20874430	15	CDKAL1
chr16:47463898-47547548	5	PHKB
chr19:7560647-7563773	1	PNPLA6
chr11:87067693-87091395	67	TMEM135
chr20:21339043-21344208	109	XRN2
chr16:16124788-16126167	14	ABCC1
chr5:65260599-65334085	7	ADAMTS6
chr6:135431207-135457713	14	AHI1
chr1:156976982-156979286	14	ARHGEF11
chr17:46352819-46358414	14	ARL17B
chr3:9800184-9804013	0	ARPC4
chr3:11315343-11348035	0	ATG7
chr5:37179360-37181005	16	C5orf42
chrX:18650409-18650592	14	CDKL5
chr1:160325542-160335322	3	COPA
chr20:35632310-35632923	14	CPNE1
chr19:49707517-49708839	14	CPT1C
chr2:232329812-232330776	14	DIS3L2
chr10:127008731-127018835	12	DOCK1
chr2:25628170-25639094	35	DTNB
chr2:62979187-62996766	87	EHBP1
chr3:186784961-186787882	14	EIF4A2
chr14:88714938-88754671	14	EML5
chr9:95978060-95979866	95	ERCC6L2
chr4:105726578-105825800	14	GSTCD
chr4:142402937-142405324	1	INPP4B
chr16:31361835-31362753	1	ITGAX
chr6:36470051-36475062	7	KCTD20
chr9:6880011-6893232	308	KDM4C
chr17:28618790-28621085	14	KIAA0100
chr3:122442037-122467429	0	KPNA1
chr18:21849195-21858646	14	MIB1

chr13:41328723-41336756	14	NAA16
chr1:201817087-201817285	15	NAV1
chr2:24564294-24584560	49	NCOA1
chr12:124362125-124374463	14	NCOR2
chr2:77831032-77986697	14	nogene
chr6:129535931-129558166	1	nogene
chr13:98551633-98551973	14	nogene
chr2:27798868-27799329	14	nogene
chr5:113260799-113263865	14	nogene
chr1:201527883-201528402	0	nogene
chr18:32424784-32465975	14	nogene
chr19:43909227-43910495	14	nogene
chr6:85663673-85666147	14	nogene
chr19:57708699-57709315	14	nogene
chr6:17661652-17669028	25	NUP153
chrX:68111853-68119332	11	OPHN1
chr8:65747651-65782843	18	PDE7A
chr2:86070017-86102508	14	POLR1A
chr2:112551774-112559574	14	POLR1B
chr2:48474682-48491170	70	PPP1R21
chr14:35096557-35099384	3	PPP2R3C
chr6:57379900-57537625	96	PRIM2
chr9:131430093-131447206	0	PRRC2B
chr1:96777584-96785254	14	PTBP2
chr12:120222862-120223833	14	PXN
chr5:233476-236599	14	SDHA
chr2:113731583-113735858	1	SLC35F5
chr17:64561499-64591149	14	SMURF2
chr9:112817704-112838697	14	SNX30
chr9:128566737-128568897	14	SPTAN1
chr9:100320264-100340369	14	TEX10
chr2:112494142-112503181	24	TTL
chr15:90250619-90253367	14	TTLL13P
chr3:41819422-41883952	14	ULK4
chr2:61190517-61203263	14	USP34
chr10:1103598-1105267	158	WDR37
chr1:180836521-180863874	14	XPR1
chr1:35381258-35389082	109	ZMYM4
chr18:76849525-76881512	173	ZNF236
chr17:49311311-49312845	165	ZNF652
chr19:36761642-36772829	14	ZNF850
chr13:23353784-23368487	45	SACS
chr19:48959867-48960303	30	nogene

chr2:108788851-108816105	13	CCDC138
chr14:80743074-80761613	73	CEP128
chr7:92126252-92127631	43	CYP51A1
chr9:19060087-19096769	13	HAUS6
chr8:30101897-30104486	172	LEPROTL1
chr15:41727179-41729349	16	MGA
chr2:218735460-218736101	13	nogene
chr2:77939830-77951533	25	nogene
chr19:29657660-29664207	0	nogene
chr6:17675228-17675770	13	NUP153
chr3:141540532-141559995	0	RASA2
chr3:67528131-67529186	2	SUCLG2
chr19:45831383-45831588	39	SYMPK
chr6:33267681-33269557	3	VPS52
chr1:42278347-42311110	9	FOXJ3
chr5:154011240-154034967	50	FAM114A2
chr7:6139091-6144196	58	USP42
chr6:75695098-75695523	19	nogene
chr2:84431507-84449752	41	SUCLG1
chr20:31743121-31744257	17	nogene
chr13:24459043-24460136	20	PARP4
chr11:9403289-9414411	13	IPO7
chr2:69542522-69556978	0	AAK1
chr1:94177611-94184288	8	ARHGAP29
chr1:11080763-11081238	0	EXOSC10
chr15:32888171-32902040	7	FMN1
chr2:227328677-227340380	3	MFF
chr8:140743229-140746860	2	PTK2
chr1:178283563-178390206	14	RASAL2
chr12:50192451-50206068	0	LIMA1
chr22:42797467-42826999	0	ARFGAP3
chr3:37281957-37282272	0	GOLGA4
chr19:41272081-41274055	0	HNRNPUL1
chr9:110941420-111036201	53	LPAR1
chr17:39419716-39423821	0	MED1
chrX:68096869-68299096	2	OPHN1
chr15:55223888-55389433	10	RAB27A
chr12:128814774-128815070	285	SLC15A4
chr15:51893814-51902036	0	TMOD3
chr17:4064360-4064829	83	ZZEF1
chrX:70402565-70404034	3	KIF4A
chr19:55140882-55141302	4	TNNT1
chr16:1453833-1456206	0	CLCN7

chr19:8371055-8371522	37	ANGPTL4
chr17:59644275-59651316	2	CLTC
chr19:46891726-46891999	28	nogene
chr14:96527429-96532649	23	PAPOLA
chr4:184659339-184672460	1	PRIMPOL
chr20:35035122-35057571	13	TRPC4AP
chr10:45639815-45663842	33	ZFAND4
chr8:60794985-60808272	23	CHD7
chr11:68505144-68519651	89	PPP6R3
chr17:80263662-80273404	1	RNF213
chr3:126022785-126026479	1	SLC41A3
chr3:47037665-47057490	9	SETD2
chr16:48347506-48348290	310	LONP2
chr17:31248983-31265339	48	NF1
chr8:47785112-47794501	28	PRKDC
chr7:67049948-67067403	13	TYW1
chr15:90439331-90440615	0	IQGAP1
chr6:145671238-145721155	0	nogene
chr12:28255580-28391411	56	CCDC91
chr9:76067954-76096102	6	PCSK5
chr2:208315192-208320359	9	PIKFYVE
chrX:102829887-102865152	13	LINC00630
chr19:29971192-29986417	842	URI1
chr9:128716746-128718625	5	PKN3
chr16:89531903-89532636	33	SPG7
chr17:61847100-61859907	23	BRIP1
chr11:95799231-95818904	13	CEP57
chr5:250991-251468	128	SDHA
chr2:9849372-9868429	13	TAF1B
chr8:33372106-33389798	14	FUT10
chrX:148383074-148383725	0	nogene
chr5:36225541-36227565	6	NADK2
chr10:101521638-101526199	4	BTRC
chr4:80295558-80335879	3	C4orf22
chr2:203245845-203280113	1	CYP20A1
chr9:137752330-137818138	0	EHMT1
chr10:119107947-119117667	6	FAM45A
chr16:2756333-2756606	21	SRRM2
chr2:121757239-121758806	21	TSN
chr17:43097243-43104956	0	BRCA1
chr9:135862466-135883078	24	CAMSAP1
chr7:94544410-94554575	1	CASD1
chr6:98875414-98934879	0	FBXL4

chr20:56378437-56379098	25	nogene
chr21:14968296-15043574	19	NRIP1
chr1:227747691-227748233	55	SNAP47
chr17:58703195-58709990	4	RAD51C
chr12:66145840-66161172	43	TMBIM4
chr4:139393590-139404490	56	nogene
chr2:3559447-3559729	29	RNASEH1-AS1
chr13:30630834-30631474	33	USPL1
chr5:37173754-37175986	0	C5orf42
chr1:240177920-240188262	197	FMN2
chr1:117870446-117920424	0	GDAP2
chr12:121452392-121453344	18	KDM2B
chr9:136013198-136016429	61	NACC2
chr2:182967215-182981376	0	NCKAP1
chr5:170892386-170968377	0	RANBP17
chr11:62827154-62827631	5	STX5
chr2:10419733-10420135	251	HPCAL1
chr4:185247293-185264610	42	SNX25
chr1:1482137-1486668	35	ATAD3B
chr16:57123413-57134826	45	CPNE2
chr5:50399231-50411383	26	EMB
chr3:149846010-149872154	156	RNF13
chr20:44511288-44514040	48	SERINC3
chr2:69816100-69819338	4	ANXA4
chr11:78568631-78571444	20	NARS2
chr16:11999296-12027444	45	SNX29
chr10:32471063-32518505	1	CCDC7
chr8:67149782-67164508	15	CSPP1
chr12:53016472-53019006	94	EIF4B
chr8:67161810-67164508	21	CSPP1
chrX:72462003-72464731	170	HDAC8
chr19:53438210-53475572	21	nogene
chr4:75515432-75516979	16	THAP6
chr6:49244965-49250609	2	nogene
chr22:38067703-38070891	1	PICK1
chr16:28641339-28752592	3	NPIPB9
chr3:105740493-105776542	14	CBLB
chr4:121804621-121810099	32	EXOSC9
chr1:240257944-240334229	47	FMN2
chr2:69861277-69871832	0	GMCL1
chr1:247999599-248000062	1	nogene
chr4:87438276-87451735	0	NUDT9
chr19:2762505-2769091	63	SGTA

chr2:40139400-40177855	56	SLC8A1
chr2:170055459-170061443	59	UBR3
chr19:4929221-4932956	0	UHRF1
chr2:182967215-182983382	4	NCKAP1
chr1:155783985-155795151	27	GON4L
chr15:100132006-100134621	39	ADAMTS17
chr10:102023476-102029745	32	C10orf76
chr20:37040152-37055656	61	RBL1
chr5:128985132-128990464	15	SLC27A6
chr10:88893870-88905660	28	STAMBPL1
chr5:126559239-126568356	0	ALDH7A1
chr2:175111567-175151158	0	ATF2
chr16:58586544-58599511	3	CNOT1
chr5:34793272-34796027	25	RAI14
chr8:70594505-70600082	1	TRAM1
chr7:100023418-100030375	0	ZKSCAN1
chr2:27598069-27608039	0	ZNF512
chr7:6731172-6731562	13	nogene
chr1:114749820-114750207	0	CSDE1
chr19:39879554-39894495	0	FCGBP
chr7:50669721-50703908	45	GRB10
chr17:5365121-5368468	25	RABEP1
chr9:33948373-33956146	166	UBAP2
chr14:52872798-52893427	3	FERMT2
chr7:24830767-24849176	0	OSBPL3
chr2:173358711-173366432	14	CDCA7
chr1:155378280-155415923	21	ASH1L
chr1:8876953-8877810	3	nogene
chr4:169002070-169007756	11	CBR4
chr19:14100825-14107409	1	PRKACA
chr5:135369405-135370142	86	H2AFY
chr10:13172375-13172765	152	MCM10
chr17:38361710-38366417	5	SOCS7
chr1:153762727-153765063	18	INTS3
chr5:146271482-146271674	17	RBM27
chr20:48953555-48963898	190	ARFGEF2
chr11:74084880-74098255	0	C2CD3
chr5:37108292-37125409	4	C5orf42
chr3:132483374-132484672	4	DNAJC13
chr8:129846803-129871496	26	FAM49B
chr7:128677563-128683255	3	FAM71F2
chr7:4754458-4761288	13	FOXK1
chr6:2115770-2124731	52	GMDS

chr1:23333517-23341017	20	HNRNPR
chr5:55960401-55968396	13	IL6ST
chr1:153668452-153669878	33	ILF2
chr15:41095600-41096353	47	INO80
chr6:108355653-108402054	1	LACE1
chr1:219193081-219218712	121	LYPLAL1
chr2:39315309-39326277	1	MAP4K3
chr1:169287302-169310080	5	NME7
chr1:72749165-72898883	15	nogene
chr12:71657525-71660427	54	nogene
chr7:156676385-156679306	0	nogene
chr7:69037742-69040699	1	nogene
chr4:139391882-139393722	13	nogene
chr6:141829735-141910011	32	nogene
chr6:141831499-141910011	6	nogene
chr5:93544794-93563463	13	NR2F1-AS1
chr7:24861612-24872069	32	OSBPL3
chr19:33511027-33512776	39	PEPD
chr11:85974707-85983973	182	PICALM
chr3:138688874-138694907	78	PIK3CB
chr1:161323613-161356840	25	SDHC
chr15:43043214-43048491	6	UBR1
chr3:196391812-196393619	218	UBXN7
chr19:57778750-57780146	25	ZNF586
chr12:132945620-132948270	15	ZNF605
chr12:123645454-123652735	80	GTF2H3
chr6:36496710-36499990	37	STK38
chrX:24062415-24068108	1	EIF2S3
chrX:53595186-53607699	6	HUWE1
chr11:64151238-64152393	69	MACROD1
chr15:71967983-72010447	0	MYO9A
chr8:123077110-123120188	28	TBC1D31
chr2:135748266-135755691	12	UBXN4
chr12:2820085-2840995	0	ITFG2
chr12:110396786-110398007	19	nogene
chr5:168559031-168564065	39	PANK3
chr2:43742740-43745963	14	PLEKHH2
chr20:34072065-34078553	383	RALY
chr7:127704838-127721400	137	SND1
chr3:136398748-136502779	0	STAG1
chr6:42573733-42594304	33	UBR2
chr1:23030468-23053839	19	KDM1A
chr14:102701277-102714617	0	RCOR1

chr3:191357087-191370036	18	CCDC50
chr19:34208928-34215661	11	LSM14A
chr18:68753292-68784285	9	nogene
chr13:23365165-23375269	51	SACS
chr2:161179613-161224746	6	TANK
chr22:22303223-22310401	0	BMS1P20
chr10:84463932-84477664	38	CCSER2
chr4:102689573-102726683	37	MANBA
chr9:14102421-14116346	0	NFIB
chr17:32173830-32194107	74	RHOT1
chr7:17839806-17873616	0	SNX13
chr3:126607650-126647296	0	TXNRD3NB
chr5:112754872-112780903	0	APC
chr14:99257470-99257839	29	BCL11B
chr11:66816052-66828364	60	C11orf80
chr5:123532568-123557564	33	CSNK1G3
chr7:133475327-133480138	0	EXOC4
chr16:53873785-53934109	68	FTO
chr15:63706779-63716473	45	HERC1
chr3:183643479-183672484	14	KLHL24
chr1:233969171-233971323	13	nogene
chr17:82394330-82398319	16	OGFOD3
chr8:106679209-106684359	156	OXR1
chr6:78988208-78990985	18	PHIP
chr11:66668614-66669291	20	RBM4B
chr1:150960562-150962158	45	SETDB1
chr5:136147831-136163391	97	SMAD5
chr7:56072996-56074736	1	SUMF2
chr4:38971031-38993998	0	TMEM156
chr6:83018972-83038485	14	UBE3D
chr8:134637460-134657737	0	ZFAT
chr4:145823421-145892415	0	ZNF827
chr22:41808874-41813822	677	CCDC134
chr18:2666156-2674145	63	SMCHD1
chr1:70184784-70189273	6	LRRC40
chr1:113938315-113938590	0	nogene
chr1:147648245-147655248	0	ACP6
chr15:89835461-89837722	24	AP3S2
chr10:72196810-72203524	37	ASCC1
chr3:11360580-11364734	1	ATG7
chr2:28025225-28051648	3	BRE
chr1:227004993-227017050	61	CDC42BPA
chr2:29160332-29167540	0	CLIP4

chr1:16486425-16486661	1	CROCCP3
chr16:67610823-67612121	275	CTCF
chr14:102040235-102042748	18	DYNC1H1
chr4:1654977-1668905	35	FAM53A
chr12:32581959-32596876	13	FGD4
chr14:21263310-21263582	0	HNRNPC
chrX:70146632-70150322	5	IGBP1
chr3:20125904-20140364	27	KAT2B
chr7:156724111-156826744	85	LMBR1
chrX:136231543-136240486	0	MAP7D3
chr5:10403406-10407202	13	6-Mar
chr1:220579353-220581118	0	MARK1
chr10:101793912-101795480	13	MGEA5
chr11:63946724-63947003	0	NAA40
chr13:107523563-107635379	0	nogene
chr5:179538973-179540832	54	nogene
chr13:29511188-29511584	13	nogene
chr10:103422987-103427391	13	PDCD11
chr12:42374637-42398994	30	PPHLN1
chr1:202562777-202569197	13	PPP1R12B
chr7:5048032-5057779	95	RBAK
chr3:150622384-150624899	25	SELT
chr7:80798091-80828745	1	SEMA3C
chr2:32174062-32184338	148	SLC30A6
chr7:128081359-128084847	13	SND1
chr7:17868406-17907340	40	SNX13
chr8:125008952-125009343	2	SQLE
chr6:121205074-121242339	13	TBC1D32
chr4:48228476-48228659	0	TEC
chr2:192179667-192184483	13	TMEFF2
chr16:46668929-46674660	0	VPS35
chr10:86458988-86473973	29	WAPL
chr18:56779430-56816144	4	WDR7
chr9:37126311-37126942	409	ZCCHC7
chr1:33294936-33295305	34	ZNF362
chr10:68468122-68470163	1081	DNA2
chr11:85974707-86003451	28	PICALM
chr11:85981128-85996929	25	PICALM
chr5:14286870-14316743	20	TRIO
chr1:225144396-225185425	3	DNAH14
chr2:85555483-85559075	24	GGCX
chr8:97815327-97819238	17	LAPTM4B
chr4:40106899-40118024	18	N4BP2

chrX:129565771-129569399	4	OCRL
chr4:6999084-7004924	4	TBC1D14
chr20:30388452-30398045	13	FRG1BP
chr21:25975069-25976028	2	APP
chr9:2804321-2812362	5	KIAA0020
chr16:72284571-72308188	8	LINC01572
chr4:7786193-7800653	17	AFAP1
chr12:31689006-31702007	8	AMN1
chr15:60355918-60382441	36	ANXA2
chr11:108301647-108310315	0	ATM
chr17:61776400-61793729	36	BRIP1
chr9:15874531-15920422	2	CCDC171
chr7:5901656-5912954	30	CCZ1
chr3:33619602-33632371	25	CLASP2
chr2:36387971-36442735	59	CRIM1
chr8:67103036-67116122	34	CSPP1
chr5:50382570-50411383	45	EMB
chr1:1825396-1879902	14	GNB1
chr2:9961045-9986282	28	GRHL1
chr10:1005817-1007128	50	GTPBP4
chr4:142402937-142431385	7	INPP4B
chr2:200504747-200507091	0	KCTD18
chr7:107978046-107980811	0	LAMB1
chr7:130945719-130967517	1	LINC-PINT
chr16:74674959-74675764	14	MLKL
chr4:169463242-169508852	0	NEK1
chr6:29889112-29927834	2	nogene
chr6:53263028-53263213	0	nogene
chr7:99375714-99377209	20	nogene
chr16:58717142-58717949	20	nogene
chr16:47547432-47550205	0	PHKB
chrX:21971896-21972571	13	SMS
chr3:17508473-17518995	0	TBC1D5
chr1:231529254-231542611	11	TSNAX-DISC1
chrX:10090770-10098579	13	WWC3
chr11:102114143-102186131	2	YAP1
chr8:103430611-103441618	17	DCAF13
chr12:50462581-50474167	58	LARP4
chr10:37729820-37734572	34	nogene
chr11:67608395-67609635	4	NDUFV1
chr20:34072065-34073866	32	RALY
chr3:15735409-15751820	37	ANKRD28
chr16:74348866-74350738	12	LOC283922

chr10:15275854-15284105	72	FAM171A1
chr21:33241885-33252830	19	IFNAR2
chr7:6015145-6018045	119	AIMP2
chr16:81022596-81026638	16	CENPN
chr12:31423596-31447769	6	DENND5B
chr7:2366316-2371849	27	EIF3B
chr2:54910953-54928751	0	EML6
chr12:56086530-56088868	4	ERBB3
chr15:76283756-76287945	9	ETFA
chr21:32453311-32495170	2	EVA1C
chr11:65882852-65885663	16	FIBP
chr6:159991183-160010785	47	IGF2R
chr1:20713453-20715639	2	KIF17
chr7:2149151-2222754	21	MAD1L1
chr19:13025020-13025552	195	NFIX
chr14:68115818-68116112	4	nogene
chr5:98884345-98885153	14	nogene
chr2:185800079-185952902	1	nogene
chr7:43780120-43780886	14	nogene
chr6:159594102-159595204	5	nogene
chrX:103428627-103450660	2	nogene
chr15:55314038-55348725	0	nogene
chr5:6739731-6746427	14	PAPD7
chr3:146088585-146121248	14	PLOD2
chr22:50372019-50394135	558	PPP6R2
chr8:140818276-140846678	98	PTK2
chr9:125207563-125220700	32	RABEPK
chr11:4107434-4126833	14	RRM1
chr10:67891401-67909442	14	SIRT1
chr1:246544520-246556721	15	TFB2M
chr15:23023946-23037152	2	TUBGCP5
chrX:118394054-118404444	68	WDR44
chr15:43827964-43835150	14	WDR76
chr7:130022318-130029029	14	ZC3HC1
chr6:85640220-85641451	19	SYNCRIP
chr17:62010549-62035608	1	MED13
chr16:3536121-3536994	2	CLUAP1
chr16:57172223-57173869	1	FAM192A
chr5:42688889-42700002	49	GHR
chr1:156743651-156745373	38	HDGF
chr18:50918109-50940386	11	ME2
chr2:196912881-196920150	85	PGAP1
chr5:134535857-134552209	32	JADE2

chr1:88770351-88771879	104	PKN2
chr16:4760491-4766076	13	ZNF500
chr1:169309969-169324500	25	NME7
chr2:55585050-55588956	2	PPP4R3B
chr17:44797562-44807469	10	GJC1
chr18:42043732-42049605	47	PIK3C3
chr19:6230569-6270759	1011	MLLT1
chr17:59028414-59041898	0	TRIM37
chr13:60008543-60010669	51	DIAPH3
chr19:10574416-10575029	54	AP1M2
chr15:59458549-59460206	31	FAM81A
chr13:29514352-29517790	13	SLC7A1
chr14:31156839-31172122	29	HECTD1
chr15:59030803-59031702	72	RNF111
chr1:21748137-21753119	31	USP48
chr7:66771882-66797506	24	RABGEF1
chr18:13012972-13019206	51	CEP192
chr10:32038162-32040457	13	KIF5B
chr12:119673963-119676670	3	PRKAB1
chr3:119503953-119517315	2	TIMMDC1
chr18:688573-706578	1	ENOSF1
chr6:154441291-154450258	54	CNKSR3
chr8:130179263-130188183	39	ASAP1
chr13:49167243-49175541	23	FNDC3A
chr6:42822795-42830172	31	GLTSCR1L
chr1:233227225-233236980	43	PCNXL2
chr5:122019259-122022407	175	SRFBP1
chr15:41668827-41699159	17	MGA
chr1:220157269-220159421	1	RAB3GAP2
chr6:43527633-43531575	31	XPO5
chr6:129634041-129642018	2	ARHGAP18
chr19:34327420-34328151	0	KIAA0355
chr3:119947267-120093593	10	GSK3B
chr12:69784719-69795344	19	RAB3IP
chr10:114970367-114974385	48	TRUB1
chr2:199943764-199948472	0	TYW5
chr8:99431536-99481802	9	VPS13B
chr5:65242103-65334085	7	ADAMTS6
chr20:35724569-35725155	294	RBM39
chr1:151088189-151118139	0	GABPB2
chr11:107275460-107311232	1	nogene
chr1:11989164-11997421	1	MFN2
chr19:24105939-24106643	20	ZNF254

chr6:141900322-141927055	36	nogene
chr17:7321864-7322866	42	NEURL4
chr2:208285725-208288818	125	PIKFYVE
chr11:73707419-73716362	96	RAB6A
chr9:35228014-35259050	27	UNC13B
chr6:31669508-31669810	2	CSNK2B
chr22:29094257-29098953	17	KREMEN1
chr15:42827927-42872758	20	TTBK2
chr6:158448995-158452268	56	TULP4
chr19:6262227-6270759	136	MLLT1
chr6:43002451-43003099	23	nogene
chr14:23301880-23302159	7	PPP1R3E
chr1:150417595-150446401	33	RPRD2
chr6:31148404-31148728	65	CCHCR1
chr15:74671454-74671774	43	EDC3
chr1:10271501-10278128	0	KIF1B
chr14:75109684-75118935	17	NEK9
chr17:15487887-15488334	0	nogene
chr3:125560378-125582978	17	OSBPL11
chr8:31147052-31150455	2	WRN
chr10:13659322-13663509	6	FRMD4A
chr11:118509135-118512025	2	KMT2A
chr15:52405286-52410476	24	MYO5A
chr2:202284344-202287724	26	NOP58
chr9:127890636-127891043	3	ST6GALNAC6
chr10:13073867-13098602	0	CCDC3
chr10:100243697-100246935	14	CWF19L1
chr16:22225653-22230664	16	EEF2K
chr12:26954144-26957743	15	FGFR1OP2
chr7:887369-893971	0	GET4
chr19:47356206-47357442	14	nogene
chr1:174637374-174752354	0	RABGAP1L
chr10:16752538-16782084	202	RSU1
chr19:45641766-45642197	1	EML2-AS1
chr2:169537226-169544835	0	FASTKD1
chr8:98212404-98222600	0	NIPAL2
chr15:32617749-32618655	16	nogene
chr5:134302589-134321903	16	CDKL3
chr12:110127359-110135437	16	IFT81
chr1:202001375-202001767	20	RNPEP
chr6:42644213-42648170	4	UBR2
chr1:174272413-174371072	24	RABGAP1L
chr7:23505745-23513082	28	TRA2A

chr10:11997671-12004727	49	UPF2
chr1:35945691-35973511	55	AGO3
chr21:14825522-14857708	3	nogene
chr9:136348696-136349763	34	GPSM1
chr22:46878652-46894846	57	TBC1D22A
chr8:140549113-140551436	6	AGO2
chr13:25326915-25338731	20	NUPL1
chr20:25323324-25339351	14	ABHD12
chr3:57242855-57267792	27	APPL1
chr14:34862043-34862322	480	BAZ1A
chrX:49242015-49243462	14	CCDC22
chr1:160323430-160332557	312	COPA
chr12:96018762-96019240	14	LTA4H
chr7:114942274-114979781	4	MDFIC
chr22:40355428-40355905	19	nogene
chr5:131504625-131548190	47	RAPGEF6
chr8:140300468-140360193	13	TRAPPC9
chr20:63752685-63790790	1	ZBTB46
chr5:43161248-43161931	200	ZNF131
chr2:29121373-29181697	90	CLIP4
chr22:31089698-31090180	27	SMTN
chr6:107039748-107040230	1	C6orf203
chr5:116317516-116318300	47	nogene
chr2:169603641-169614724	193	PPIG
chr3:9948066-9948469	13	PRRT3-AS1
chr10:63190893-63194375	3	JMJD1C
chr18:46890579-46903635	502	PIAS2
chr12:111509548-111510582	171	ATXN2
chr3:197115926-197142768	16	DLG1
chr12:125073875-125086443	4	AACS
chr16:89418283-89427069	75	ANKRD11
chr2:218217462-218239484	14	ARPC2
chr10:310030-341329	3	DIP2C
chr4:142082030-142124760	18	INPP4B
chr6:112900344-112937686	17	nogene
chr20:44916755-44919277	19	PABPC1L
chr3:49076708-49077038	11	QRICH1
chr5:65680493-65685472	28	SGTB
chr5:55652528-55664863	25	SLC38A9
chr14:77448493-77454102	3	VIPAS39
chr8:134564986-134590355	2	ZFAT
chr3:43574808-43580472	93	ANO10
chr8:37765525-37766355	297	PROSC

chr2:109564365-109593119	291	10-Sep
chr9:111574733-111586165	181	PTGR1
chr16:3129247-3129653	0	ZNF213-AS1
chr14:24235736-24237562	0	GMPR2
chr11:3705199-3706627	17	NUP98
chr12:46229152-46254936	528	SLC38A1
chr8:123226499-123239144	3	C8orf76
chr1:200760838-200815644	25	CAMSAP2
chr2:10784933-10790833	20	PDIA6
chr3:44455120-44458364	18	ZNF445
chr8:58623708-58635546	1	NSMAF
chr15:41069569-41079904	12	INO80
chr7:66681005-66681505	28	nogene
chr10:103345023-103348999	33	PCGF6
chr7:99396599-99408573	89	nogene
chr18:31638643-31666372	187	B4GALT6
chr17:67166477-67167796	14	HELZ
chrX:71294349-71297461	15	NONO
chr11:86007541-86026367	6	PICALM
chr2:135748266-135761911	113	UBXN4
chr14:75717860-75735289	5	TTLL5
chr1:36004340-36009594	1	AGO3
chr4:121833230-121835283	1	BBS7
chr5:148960916-148970284	0	nogene
chr1:246193674-246193963	0	nogene
chr5:37331684-37341242	61	NUP155
chr4:88051990-88068061	7	PKD2
chr7:5286655-5287985	2	SLC29A4
chr1:19755633-19787117	12	TMCO4
chr19:19592505-19599365	8	PBX4
chr9:91717306-91722580	15	nogene
chr20:20018495-20019023	18	nogene
chr1:240206798-240234450	15	FMN2
chr1:173402258-173442097	0	nogene
chr2:161318821-161371322	0	PSMD14
chr5:34813573-34821850	14	RAI14
chr9:122865348-122897576	1	RC3H2
chr10:119926035-119933787	0	SEC23IP
chr17:50998443-51021365	0	SPAG9
chr20:47262287-47276754	0	ZMYND8
chr7:152199278-152207428	17	KMT2C
chr20:62124616-62131506	26	LSM14B
chr4:1892165-1918623	31	WHSC1

chr3:37075023-37094908	0	LRRFIP2
chr3:193662821-193667280	0	OPA1
chr2:96194957-96195549	45	STARD7
chr3:141512162-141529802	7	RASA2
chr6:30723428-30723674	96	TUBB
chr8:67175295-67190759	2	CSPP1
chr19:6010135-6016271	12	RFX2
chr5:179585865-179586300	20	RUFY1
chr17:20204332-20232405	4	SPECC1
chr2:101002701-101006071	16	RPL31
chrX:110171419-110173453	29	TMEM164
chr17:82248874-82265796	75	CSNK1D
chr5:55272086-55281515	0	DHX29
chr20:38972558-38994880	0	DHX35
chr17:44804015-44807469	0	GJC1
chr9:3879426-3937189	0	GLIS3
chrX:16754944-16757309	0	SYAP1
chr5:80146089-80158962	0	SERINC5
chrX:91414903-91446693	4	nogene
chr12:119822819-119825368	21	CIT
chr1:45615012-45616393	32	NASP
chr1:155957712-155958396	1	ARHGEF2
chr1:8866278-8867250	64	ENO1
chr2:197493323-197495376	65	HSPD1
chr18:36195256-36203189	78	MOCOS
chr11:78489912-78493195	4	NARS2
chr7:108479183-108497577	1	PNPLA8
chr8:134501828-134521001	0	ZFAT
chr2:168138087-168182090	21	STK39
chr3:30644746-30650460	16	TGFBR2
chr16:3810602-3813121	4	CREBBP
chr15:63542054-63563008	25	USP3
chr1:1655368-1719455	14	CDK11B
chr10:100937401-100944128	50	SLF2
chr9:92268173-92285839	24	IARS
chr1:224294266-224303857	30	NVL
chr2:233434379-233437479	39	DGKD
chr19:11123173-11123344	0	LDLR
chr17:82956694-82965746	20	B3GNTL1
chr5:171183170-171205612	25	RANBP17
chr9:112142319-112143623	1	SUSD1
chr1:203833790-203840374	0	ZC3H11A
chr4:150735257-150808398	40	LRBA

chr14:24080234-24084745	1	nogene
chr5:171391304-171400925	123	NPM1
chr4:182894488-182915575	3	DCTD
chr2:231214830-231216793	6	ARMC9
chr1:35474804-35479212	8	KIAA0319L
chr1:152226425-152227051	2	nogene
chr5:134692601-134697246	5	SEC24A
chr2:229858771-229880128	156	TRIP12
chr9:33941648-33953474	23	UBAP2
chr6:43546570-43548460	166	XPO5
chr20:37066723-37068186	82	RBL1
chr12:55789074-55803728	55	SARNP
chr21:39274373-39278813	17	BRWD1
chr13:23862301-23881787	31	MIPEP
chr22:46097925-46109908	15	MIRLET7BHG
chr4:17822982-17831116	27	NCAPG
chr6:35855224-35855844	26	nogene
chr12:104288930-104339273	14	TXNRD1
chr16:28152658-28156527	47	XPO6
chr2:9350807-9358889	94	ASAP2
chr11:66821642-66828943	0	C11orf80
chr5:37138719-37164327	13	C5orf42
chr3:105734008-105776542	17	CBLB
chr2:108788851-108794721	37	CCDC138
chr1:224952669-224968874	137	DNAH14
chr8:25242194-25243757	50	DOCK5
chr8:11808793-11826215	6	FDFT1
chr4:53425871-53453133	41	FIP1L1
chr16:1557934-1602591	13	IFT140
chr3:160521778-160528039	7	KPNA4
chr5:1477403-1501603	13	LPCAT1
chr15:72045723-72046634	41	MYO9A
chr2:190670325-190687317	1	NAB1
chr12:6887922-6888158	13	nogene
chr4:103328124-103371506	1	nogene
chr19:4410318-4410475	13	nogene
chr11:47822086-47837623	0	NUP160
chr13:24499300-24503777	388	PARP4
chr9:26928099-26928408	13	PLAA
chr18:9577063-9588235	13	PPP4R1
chr8:140818276-140890769	1454	PTK2
chr11:66365300-66366564	2	SLC29A2
chr10:110600438-110601130	13	SMC3

chr4:6994183-7001251	189	TBC1D14
chr12:44031636-44211387	13	TMEM117
chr20:33770083-33781387	13	ZNF341
chr11:63651935-63659252	1	ATL3
chr6:110887504-110893662	81	AMD1
chr4:147854675-147881932	113	ARHGAP10
chr10:115461940-115519324	18	ATRNL1
chr3:197756425-197768587	23	FYTTD1
chr10:17229419-17229871	1	nogene
chr7:66993216-66994341	6	SBDS
chr13:21157921-21172681	54	SKA3
chr1:23816674-23820593	12	HMGCL
chr20:34782120-34792563	42	NCOA6
chr12:101714089-101723847	17	CHPT1
chr7:158630496-158646563	17	NCAPG2
chr14:50466582-50482416	6	MAP4K5
chr15:35410834-35454820	17	DPH6
chr5:138319218-138329647	94	CDC25C
chr12:31402497-31409384	0	DENND5B
chr7:75553452-75554526	0	HIP1
chr16:47109482-47129364	119	NETO2
chr6:57379900-57382168	58	PRIM2
chr8:140275657-140300614	25	TRAPPC9
chr2:147896300-147899898	614	ACVR2A
chr12:1702885-1781076	0	ADIPOR2
chr15:89790459-89791093	0	ANPEP
chr3:63898398-63983021	0	ATXN7
chr10:35016191-35028887	3	CUL2
chr11:108688935-108693600	41	DDX10
chr17:63792411-63806654	15	DDX42
chr8:123786494-123789745	63	FAM91A1
chr2:241407536-241436538	28	FARP2
chr2:232832856-232839971	3	GIGYF2
chr15:41055246-41070547	0	INO80
chr12:51049033-51056502	62	LETMD1
chr3:152414940-152455577	0	MBNL1
chr9:36669309-36674937	53	MELK
chr8:143575422-143575702	0	NAPRT
chr20:36656359-36671397	0	NDRG3
chr4:173164977-173168113	0	nogene
chr10:102157018-102161925	0	NOLC1
chr4:87434980-87438372	0	NUDT9
chr10:34516978-34517159	0	PARD3

chr3:111913318-111913702	0	PHLDB2
chr4:154539950-154545923	0	PLRG1
chr14:73147794-73174376	0	PSEN1
chr3:50105076-50109688	0	RBM5
chr6:2954591-2959342	2	SERPINB6
chr12:53381658-53409561	4	SP1
chr8:47279861-47294030	449	SPIDR
chr6:45321795-45365301	23	SUPT3H
chr21:33455726-33467122	26	TMEM50B
chr5:151060317-151065131	34	TNIP1
chr14:102870184-102876525	106	TRAF3
chr6:30021666-30035983	49	ZNRD1-AS1
chr6:131145284-131219808	15	AKAP7
chr2:214752446-214769312	41	BARD1
chr16:14648387-14649973	0	BFAR
chr7:77292951-77294938	77	CCDC146
chrX:1282677-1288888	0	CSF2RA
chr18:61577027-61592623	0	nogene
chr2:18644169-18688328	0	nogene
chr11:19762228-19762631	33	nogene
chr5:177244194-177248324	30	NSD1
chr6:13639562-13641307	0	RANBP9
chr17:75957458-75960375	0	ACOX1
chr11:72707174-72714321	27	ARAP1
chr4:186663298-186709845	32	FAT1
chr17:46082440-46120049	20	KANSL1
chr9:593608-594073	0	nogene
chr16:89895037-89907322	20	TCF25
chr16:69669976-69677335	34	NFAT5
chr3:47498285-47504448	3	ELP6
chr21:43004325-43028874	116	PKNOX1
chr12:32598496-32625779	24	FGD4
chr7:78976487-79007206	0	MAGI2
chr11:130109428-130122513	98	APLP2
chr10:31861394-31861658	5	ARHGAP12
chrX:63697124-63724711	11	ARHGEF9
chr12:53531743-53534659	1	ATF7
chr1:62803718-62841547	19	ATG4C
chr6:109145218-109146937	144	CEP57L1
chr19:2185854-2194577	26	DOT1L
chr8:108214606-108241913	60	EIF3E
chr12:53027781-53028188	317	EIF4B
chr10:93676652-93687449	63	FRA10AC1

chr10:13663452-13670528	0	FRMD4A
chr10:102351374-102351816	20	GBF1
chr10:72715110-72742273	3	MCU
chr2:134253094-134254644	217	MGAT5
chr16:29833732-29836958	12	MVP
chr17:3804532-3805018	25	nogene
chr1:36284512-36284924	0	nogene
chr1:25988230-25990863	13	PAFAH2
chr14:58286639-58291707	27	PSMA3-AS1
chr7:66944975-66948694	5	TMEM248
chr8:132727797-132757294	3	TMEM71
chr20:35347163-35394196	1	UQCC1
chr2:71368381-71370005	2	ZNF638
chr2:17726388-17745951	13	SMC6
chr9:136522849-136523979	12	NOTCH1
chr6:138207041-138210041	53	ARFGEF3
chr18:42049530-42067513	1	PIK3C3
chr2:171022871-171046362	0	TLK1
chr10:68142939-68158627	125	MYPN
chr1:1022200-1022462	17	AGRN
chr7:73449541-73459718	0	BAZ1B
chr2:46356150-46361090	0	EPAS1
chr9:20907149-20933132	16	FOCAD
chr8:41611902-41615048	17	GPAT4
chr8:43178073-43182260	0	HGSNAT
chr6:149842982-149853160	2	LRP11
chr14:71105234-71105440	58	PCNX
chr9:131430089-131465078	1	PRRC2B
chr8:127855154-127890998	184	PVT1
chr5:141973582-141987975	2	RNF14
chr12:111933885-111943369	54	TMEM116
chr14:39158284-39159550	13	TRAPPC6B
chr15:43024828-43025429	24	UBR1
chr8:102327741-102345545	0	UBR5
chr16:74938149-74951543	16	WDR59
chr20:47276313-47290274	2	ZMYND8
chr9:111527267-111530963	15	ZNF483
chr1:235465635-235494828	249	B3GALNT2
chr10:126996747-126999435	49	DOCK1
chr8:100527844-100529860	2	ANKRD46
chr3:172285925-172310881	7	FNDC3B
chr2:239176412-239236664	18	HDAC4
chr13:24517746-24518043	26	nogene

chr20:35721739-35742113	5	RBM39
chrX:108614910-108626349	0	COL4A5
chr21:25741572-25752234	19	GABPA
chr5:109767534-109774965	28	MAN2A1
chr2:39196951-39229588	4	nogene
chr2:43943686-43982434	21	LRPPRC
chr10:73030270-73047101	48	P4HA1
chr6:20546345-20548705	78	CDKAL1
chr7:128997388-129001234	59	TNPO3
chr7:66286510-66286709	189	TPST1
chr19:13868177-13868567	1	nogene
chr15:100132006-100199423	25	ADAMTS17
chr14:92126121-92143294	118	CPSF2
chr16:1390631-1403344	21	UNKL
chr10:1072115-1096246	45	WDR37
chr16:11779165-11782388	76	ZC3H7A
chr5:65424525-65473952	19	ADAMTS6
chrX:108575909-108578383	1	COL4A5
chr10:77493797-77495103	14	nogene
chr13:31260945-31286819	1	B3GLCT
chr14:55649775-55659703	1	KTN1
chr2:206126538-206130242	0	NDUFS1
chr5:94628810-94630743	53	SLF1
chr5:160010499-160036803	65	TTC1
chr10:7243557-7276989	23	SFMBT2
chr1:31059203-31059577	200	PUM1
chr17:8255026-8258199	15	PFAS
chr3:122437169-122496570	13	KPNA1
chr5:150400006-150400164	70	nogene
chr17:1842802-1872526	27	RPA1
chr20:17953995-17955475	1	SNX5
chr3:122449573-122454001	26	KPNA1
chr7:92225719-92226682	3	KRIT1
chr11:108135141-108148858	19	ACAT1
chr21:45128401-45144701	13	ADARB1
chr18:79126266-79193263	10	ATP9B
chrX:77654097-77664778	12	ATRX
chr15:92953356-92956649	35	CHD2
chr4:1225359-1228343	33	CTBP1
chr1:168002481-168004793	32	DCAF6
chr3:56666259-56673725	28	FAM208A
chrX:154712228-154716329	26	GAB3
chr22:37618447-37621696	8	GGA1

chr7:23345939-23351586	35	IGF2BP3
chr8:55966714-55969793	107	LYN
chr7:24623665-24680520	121	MPP6
chr2:42640172-42659862	64	MTA3
chr2:206141940-206152510	0	NDUFS1
chr2:230295436-230394574	16	nogene
chrX:71536177-71538072	9	OGT
chr16:2581294-2586893	3	PDPK1
chr10:13291830-13295606	2	PHYH
chr4:88065373-88068061	0	PKD2
chr19:43655418-43656640	13	PLAUR
chr11:66624213-66625678	5	RBM14
chr2:127989563-127993308	7	SAP130
chr12:121743628-121752223	98	TMEM120B
chr6:125993756-126013101	3	TRMT11
chr2:128097428-128109746	41	UGGT1
chr5:55467868-55475450	1	PPAP2A
chr2:119877322-119885882	67	PTPN4
chr8:119793365-119802025	68	TAF2
chr12:19493799-19500221	28	AEBP2
chr10:80280172-80284038	31	MAT1A
chr5:131415070-131417615	2	nogene
chr11:3735190-3753408	43	NUP98
chr16:68025414-68038149	43	DUS2
chr5:73864816-73873246	8	ARHGEF28
chr10:12209966-12217467	13	CDC123
chr15:64300318-64300723	13	CSNK1G1
chr10:21805979-21882439	2	DNAJC1
chr2:30459551-30479911	12	LCLAT1
chr2:151416560-151423042	13	RIF1
chr3:17372074-17384015	16	TBC1D5
chr5:160010499-160043169	29	TTC1
chr13:113190871-113200516	12	PCID2
chr2:61294268-61294528	25	nogene
chrX:96912327-96918617	28	DIAPH2
chr10:32038162-32048551	4	KIF5B
chr17:32171042-32183272	36	RHOT1
chr1:197652234-197715074	51	DENND1B
chr5:69355648-69355953	7	AK6
chr3:136255855-136262065	18	PCCB
chr2:203366876-203395780	3	ABI2
chr5:142885297-142932125	99	ARHGAP26
chr7:90374301-90378211	17	GTPBP10

chr13:77176496-77180318	13	MYCBP2
chr1:72341546-72380160	1	nogene
chr13:41907585-41912168	41	VWA8
chr11:62882907-62885608	93	SLC3A2
chr12:82369386-82389922	2	METTL25
chr12:100102765-100108548	19	UHRF1BP1L
chr6:135394775-135428759	24	AHI1
chr22:29502378-29506777	4	nogene
chr20:49066191-49075520	12	CSE1L
chr11:61323978-61331691	0	DDB1
chr6:25482256-25500235	21	LRRC16A
chr16:69653235-69659899	23	NFAT5
chr10:26720217-26724127	19	PDSS1
chr14:69116365-69149503	19	DCAF5
chr8:42944401-42945326	24	nogene
chr18:26078887-26079612	18	nogene
chr11:86306244-86326676	0	C11orf73
chr3:15232518-15247457	2	CAPN7
chr2:26792130-26793897	17	CENPA
chr3:113395749-113407041	4	CFAP44
chr10:68090183-68093258	0	nogene
chr5:4587902-4593368	13	nogene
chr20:35863012-35871829	31	PHF20
chr2:46086207-46159752	0	PRKCE
chr6:110101587-110127629	9	WASF1
chr7:66278952-66286709	50	TPST1
chr12:28198942-28391411	4	CCDC91
chr6:53273219-53291963	20	ELOVL5
chr19:14402596-14404562	15	ADGRE5
chr19:51965512-51966214	3	ZNF350
chr7:3434176-3440256	12	nogene
chr8:93998702-94020460	3	nogene
chr1:215586494-215595471	31	KCTD3
chr16:88018427-88027650	10	BANP
chr13:35040932-35070852	16	NBEA
chr10:49960135-49969883	15	nogene
chr4:185264437-185267155	9	SNX25
chr22:40117054-40156182	43	TNRC6B
chr2:25628170-25628384	21	DTNB
chr11:2969802-2972243	96	NAP1L4
chr17:63073942-63099357	2	TANC2
chr9:83677726-83677961	28	UBQLN1
chr10:7243557-7285954	357	SFMBT2

chr15:100152611-100199423	48	ADAMTS17
chr17:76063975-76067330	16	SRP68
chr7:886055-887519	13	GET4
chr9:92285721-92289426	13	IARS
chr19:40255157-40265351	235	AKT2
chr12:53027781-53037622	0	EIF4B
chr4:83470202-83482244	5	FAM175A
chr1:27578550-27578784	0	nogene
chr7:96191108-96208977	15	SLC25A13
chrX:147924918-147945616	27	FMR1
chr13:30251930-30255615	119	KATNAL1
chr1:23050386-23059234	1	KDM1A
chr10:78035351-78037304	12	RPS24
chr15:24975357-24977029	14	SNRPN
chr21:15014343-15043570	18	NRIP1
chr17:78400676-78401106	39	PGS1
chr9:19298055-19300331	4	DENND4C
chr18:192841-204692	16	USP14
chr21:43004325-43018230	17	PKNOX1
chr19:57813293-57850561	12	ZNF552
chr20:43702489-43713106	18	MYBL2
chr19:45263112-45271708	10	MARK4
chr13:30240459-30255615	34	KATNAL1
chr13:114246946-114272481	1	CDC16
chr1:53772328-53789196	23	NDC1
chr13:29222511-29224162	140	nogene
chr18:21791373-21804014	93	MIB1
chr19:32605834-32607832	49	ANKRD27
chr2:98600528-98604191	1	COA5
chr10:5766240-5800705	1	GDI2
chr11:34685230-34715234	0	nogene
chr10:119904083-119909130	11	SEC23IP
chr8:99442400-99481802	6	VPS13B
chr10:12217360-12238485	43	CDC123
chr10:67908045-67909442	13	SIRT1
chr1:179117331-179118764	29	ABL2
chr1:62803718-62821209	27	ATG4C
chr6:43202715-43205115	0	CUL9
chr18:36353568-36372744	20	FHOD3
chr5:157754853-157755192	0	LSM11
chr16:13105265-13109349	1	nogene
chr17:8508553-8509949	11	MYH10
chr10:14867082-14867909	10	HSPA14

chr17:3816115-3822052	13	C17orf85
chr15:51895475-51895854	12	nogene
chr1:41113282-41117010	141	SCMH1
chr5:72855773-72865729	64	TNPO1
chr4:74174505-74180866	2	MTHFD2L
chr17:44805253-44807469	14	GJC1
chr5:80725452-80744615	89	MSH3
chr7:98962301-98971945	23	TRRAP
chr7:99390892-99391253	40	ARPC1B
chr12:51240342-51241116	2	DAZAP2
chr12:12487027-12521463	1	DUSP16
chr5:93824147-93881688	12	FAM172A
chr16:29910977-29912049	28	KCTD13
chrX:136236243-136241277	2	MAP7D3
chr16:83906986-83912367	39	MLYCD
chr6:150955994-150965037	61	MTHFD1L
chr10:68201828-68206903	15	MYPN
chr1:176162868-176184692	102	RFWD2
chr5:95763507-95783963	30	RHOBTB3
chr7:17868406-17893434	29	SNX13
chr3:4410864-4453049	2	SUMF1
chr1:179865435-179865854	1	TOR1AIP2
chr2:106145189-106166083	153	UXS1
chr7:38862544-38869253	11	VPS41
chr6:37929958-38082457	8	ZFAND3
chr17:59731420-59773885	120	VMP1
chr4:99081364-99082116	0	ADH5
chr1:180041137-180048705	1	CEP350
chr12:49097565-49100454	2	LMBR1L
chr1:36170970-36173478	86	MAP7D1
chr1:206728709-206735210	0	MAPKAPK2
chr1:247156405-247159867	28	nogene
chr7:129671428-129690546	36	NRF1
chr18:32111753-32111919	0	RNF138
chr1:15937162-15938006	32	SPEN
chr6:37279303-37287072	53	TBC1D22B
chr17:653290-710613	0	VPS53
chr14:74829212-74835908	52	YLPM1
chr20:25634048-25674007	2	ZNF337-AS1
chr10:76944772-76970067	45	KCNMA1
chr10:74600378-74670269	2	ADK
chr10:32565557-32623817	1	CCDC7
chr9:110972072-111011690	613	LPAR1

chr4:75918760-75940165	0	NAAA
chr16:70539455-70539871	65	nogene
chr2:118103196-118108377	23	INSIG2
chr2:203380207-203427269	38	ABI2
chr19:726137-736078	112	PALM
chr9:172080-175784	7	CBWD1
chr2:196669832-196695159	15	CCDC150
chr17:59079753-59104394	15	TRIM37
chr11:128484822-128490576	200	ETS1
chr3:52918483-52928341	89	SFMBT1
chr1:29060421-29097935	35	EPB41
chr17:64591083-64606640	393	SMURF2
chr2:134936840-134942674	0	CCNT2
chr3:56669699-56671692	12	FAM208A
chr5:103003032-103009866	59	PAM
chr2:231235223-231262398	2	ARMC9
chr7:92294888-92345066	15	ANKIB1
chr14:64303475-64330921	15	ESR2
chr6:149572284-149582603	89	GINM1
chr2:10373605-10396920	1	HPCAL1
chr17:59070822-59091340	1	TRIM37
chr17:69259705-69261748	0	ABCA5
chr16:16033108-16036603	0	ABCC1
chr7:65068455-65069757	0	CCT6P3
chr10:126970701-126990603	817	DOCK1
chr12:43452273-43454052	11	ADAMTS20
chr7:102963532-103029418	16	FBXL13
chr1:117911993-117918736	93	GDAP2
chr11:75983386-76016980	10	UVRAG
chr19:47081512-47082295	38	ZC3H4
chr1:92088697-92129776	16	BTBD8
chr10:132645867-132697919	20	INPP5A
chr7:100328835-100332576	29	PMS2P1
chr19:43497117-43497876	76	PHLDB3
chr2:201145377-201149835	40	CFLAR
chr2:46956838-46978907	13	TTC7A
chr1:176043185-176046324	45	RFWD2
chr2:241343751-241348191	38	2-Sep
chr3:15685225-15690220	7	ANKRD28
chr13:31215050-31229294	305	B3GLCT
chr1:169798856-169827199	13	C1orf112
chr18:45865614-45867748	13	EPG5
chr17:67215898-67218822	13	HELZ

chr15:70900921-70936822	13	LRRC49
chr8:17713213-17755961	13	MTUS1
chr1:10008706-10009118	13	nogene
chr3:184318543-184318794	13	nogene
chr7:5391577-5392220	65	nogene
chr20:32511346-32527913	0	NOL4L
chr1:46055794-46077613	13	PIK3R3
chr1:30992389-31007099	46	PUM1
chr7:80810610-80828745	92	SEMA3C
chr5:175492094-175492267	3	SFXN1
chr17:2379035-2381162	13	SGSM2
chr20:13559007-13587370	131	TASP1
chr9:99128854-99149301	12	TGFBR1
chr2:43279764-43293213	7	THADA
chr17:59064354-59070947	13	TRIM37
chr8:15730665-15748465	13	TUSC3
chr7:157163809-157170450	48	UBE3C
chr20:56381432-56386533	271	AURKA
chr2:111831285-111850910	16	ANAPC1
chr5:171899003-171914405	0	FBXW11
chr1:240257944-240392562	118	FMN2
chr19:15272814-15273133	54	BRD4
chr4:189952161-189960839	43	FRG1
chr14:65455620-65629606	14	FUT8
chr6:128218921-128242602	27	PTPRK
chr7:6585015-6585260	3	ZDHHC4
chr11:17168676-17169806	19	PIK3C2A
chr16:3749626-3770986	29	CREBBP
chr10:45900346-45916171	15	PARGP1
chr3:12489783-12506835	13	TSEN2
chr2:201723042-201724459	4	ALS2
chr18:31931328-31936291	0	TRAPPC8
chr10:12529821-12553356	29	CAMK1D
chr12:32701414-32711015	2	DNM1L
chr19:45626704-45630046	25	EML2
chr6:84540984-84541709	6	nogene
chr2:32098795-32128479	11	SPAST
chr15:100132006-100133315	56	ADAMTS17
chr20:25413789-25418195	73	GINS1
chr7:102465816-102466270	207	LRWD1
chr8:70159504-70162854	108	NCOA2
chr2:240005209-240022340	53	NDUFA10
chr2:183151507-183161195	81	NUP35

chr13:100268688-100340259	0	PCCA
chr17:2236491-2244719	1	SMG6
chr1:47269634-47272241	2	STIL
chr15:71141448-71215399	0	THSD4
chr3:185921103-185923984	165	TRA2B
chr15:42810613-42872758	50	TTBK2
chr2:61278164-61284957	23	USP34
chr19:43924237-43931365	12	ZNF45
chr3:51681341-51684634	19	TEX264
chr16:19073920-19075860	55	COQ7
chr3:113341743-113344712	4	CFAP44
chr4:110488540-110491164	16	ENPEP
chrX:131749305-131772563	19	FIRRE
chr2:171053760-171061156	3	TLK1
chr9:111914604-111915836	125	UGCG
chr14:102194937-102209862	211	WDR20
chr3:49423678-49423959	4	nogene
chr10:4830674-4873307	11	AKR1E2
chrX:77656559-77664778	13	ATRX
chr7:140776911-140794467	87	BRAF
chr15:44337762-44338317	5	CASC4
chr5:112985834-112992770	5	DCP2
chr10:472438-486530	150	DIP2C
chr19:45277922-45278615	47	MARK4
chr1:176111068-176111403	24	nogene
chr5:34051881-34064715	32	nogene
chr10:103139405-103181328	75	NT5C2
chr4:87438276-87454455	63	NUDT9
chr7:75474703-75511114	1	POM121C
chr15:59084128-59085785	17	RNF111
chr22:31528689-31531129	1	SFI1
chr1:165743166-165754274	2	TMCO1
chr11:36494975-36501537	16	TRAF6
chr6:42615070-42632904	54	UBR2
chr13:52416016-52442445	13	VPS36
chr4:10097710-10099139	33	WDR1
chr13:24498115-24503777	392	PARP4
chrX:119410189-119417640	10	SLC25A43
chr1:153748688-153757763	2	INTS3
chr12:120154969-120155719	3002	GCN1
chr5:36136308-36143406	41	LMBRD2
chr3:141945430-141946776	19	nogene
chr2:168012633-168075231	164	STK39

chr21:36896859-36962170	0	HLCS
chr2:171540807-171541793	32	CYBRD1
chr18:32111753-32118352	32	RNF138
chr5:138517560-138518867	25	ETF1
chr6:12129758-12135892	21	HIVEP1
chr5:38903881-38924595	3	OSMR
chr9:127339644-127342334	94	GARNL3
chr17:82943821-83048785	1	B3GNTL1
chr17:54912665-54939020	28	TOM1L1
chr2:25628170-25652661	26	DTNB
chr15:39860819-39862419	13	nogene
chr1:58713055-58715641	15	nogene
chr9:96827079-96845016	74	ZNF782
chr2:69505568-69507578	8	AAK1
chr6:144537581-144539443	54	UTRN
chr12:100204939-100207786	30	ACTR6
chr17:9022310-9023391	14	NTN1
chr22:41125863-41135906	1	EP300
chr12:124911698-124912072	2	nogene
chr3:72832368-72844423	185	SHQ1
chr7:770470-785897	50	DNAAF5
chr12:123176687-123198334	3	MPHOSPH9
chr2:152542738-152561035	52	FMNL2
chr4:79977620-80036032	0	ANTXR2
chr4:4434769-4471706	19	STX18
chr2:159373044-159374753	5	BAZ2B
chr4:48684672-48780173	13	FRYL
chr4:870710-884086	13	GAK
chr21:36930250-36938994	1	HLCS
chr4:101080531-101109078	13	PPP3CA
chr12:109216617-109216920	13	ACACB
chr20:49063201-49070297	13	CSE1L
chr16:27330065-27342259	0	IL4R
chr11:8983874-8988282	0	NRIP3
chr19:44865119-44874478	1	PVRL2
chr1:109326991-109345878	1	SORT1
chr7:93257393-93259632	17	VPS50
chr13:95161188-95166367	49	ABCC4
chr19:32628721-32643631	0	ANKRD27
chr3:11340644-11358612	0	ATG7
chr19:15272518-15273133	0	BRD4
chr11:74078114-74078717	26	C2CD3
chr3:45089053-45091726	0	CDCP1

chr2:207567445-207577655	0	CREB1
chr20:37765196-37803048	5	CTNNBL1
chr6:52423945-52454287	13	EFHC1
chr4:2596083-2626577	0	FAM193A
chr14:52864400-52864853	0	FERMT2
chr7:55984846-55997449	13	GBAS
chr1:32326938-32327677	75	HDAC1
chr5:119493817-119529980	0	HSD17B4
chr6:108323824-108366332	0	LACE1
chr6:149695073-149702266	58	LATS1
chr16:25132401-25140247	0	LCMT1
chr6:89677569-89688808	19	MDN1
chr12:124466172-124495368	0	NCOR2
chr19:45187330-45187740	14	nogene
chr11:74988586-75005625	0	nogene
chr8:51845660-51861246	19	PCMTD1
chr15:43765449-43766910	4	PDIA3
chr3:170106831-170113519	0	PHC3
chr22:31701655-31712365	0	PRR14L
chr8:140800458-140830526	110	PTK2
chr12:56210296-56216613	16	RNF41
chr6:88904714-88941180	29	RNGTT
chr3:47097954-47114004	0	SETD2
chr17:64571797-64591149	15	SMURF2
chr15:75598278-75621056	2	SNUPN
chr1:15911100-15916279	61	SPEN
chr7:117119560-117138532	2	ST7
chr2:170996652-171014948	0	TLK1
chr9:109033306-109050692	28	TMEM245
chr14:102836809-102903429	0	TRAF3
chr15:42810613-42878684	0	TTBK2
chrX:75293471-75303520	0	UPRT
chr17:623532-661895	42	VPS53
chr2:63259306-63439871	13	WDPCP
chr10:1072115-1086357	24	WDR37
chr14:72981788-72993357	0	ZFYVE1
chr14:72977926-72978969	0	ZFYVE1
chr19:51877281-51880877	91	ZNF577
chr1:45330515-45334511	13	MUTYH
chr9:93497470-93498886	18	FAM120A
chr5:75354502-75356448	2	HMGCR
chr2:39278406-39286964	16	MAP4K3
chr10:4830674-4837581	3	AKR1E2

chr22:31738386-31738911	110	PRR14L
chr2:119962615-119967972	5	PTPN4
chr2:45976428-45984680	82	PRKCE
chr2:178331551-178333041	0	OSBPL6
chr19:34208928-34219877	12	LSM14A
chr1:167765582-167773368	124	MPZL1
chr1:219193129-219218712	48	LYPLAL1
chr5:181239066-181242345	28	GNB2L1
chr12:50427761-50437838	19	LARP4
chr4:177353307-177360677	150	NEIL3
chr11:65503728-65504595	30	nogene
chr2:218715900-218716514	28	nogene
chr2:183178806-183182680	165	nogene
chr22:46792519-46797620	27	TBC1D22A
chr6:13316570-13321095	2	TBC1D7
chr10:102488149-102489138	5	ACTR1A
chr5:73840479-73858219	20	ARHGEF28
chr12:122771753-122792121	0	CCDC62
chr16:57167092-57173869	36	FAM192A
chr12:31293804-31298224	70	FAM60A
chr3:15258830-15262289	0	SH3BP5
chr20:4883641-4902558	0	SLC23A2
chr12:827041-830160	121	WNK1
chr1:15649719-15651895	17	DDI2
chr15:76292430-76295737	142	ETFA
chr4:158891445-158895865	14	FNIP2
chr11:77332729-77343931	4	PAK1
chr3:32357372-32370026	31	CMTM8
chr1:235455558-235484515	61	B3GALNT2
chr11:93723040-93724375	0	CEP295
chr1:197583151-197645743	24	DENND1B
chr10:21882281-21920963	115	DNAJC1
chr14:31173155-31178254	0	HECTD1
chr1:39047035-39047987	1	nogene
chr12:56666203-56671847	0	PTGES3
chr1:172551511-172579267	61	SUCO
chr4:75448466-75455855	5	nogene
chr1:155265859-155266895	234	CLK2
chr1:155915872-155917672	43	KIAA0907
chr13:52457143-52457568	18	nogene
chr20:13483226-13610919	16	TASP1
chr2:206866696-206881891	4	nogene
chr12:105189990-105195501	19	APPL2

chr6:14131519-14135909	37	CD83
chr17:45030091-45034999	3	DCAKD
chr13:30208514-30241086	3	KATNAL1
chr7:6469595-6474284	91	KDELR2
chr14:39179090-39179462	366	PNN
chr3:47083719-47088247	0	SETD2
chr4:80335692-80362884	3	C4orf22
chr15:66484989-66487292	6	MAP2K1
chr2:182751657-182762801	16	DNAJC10
chr16:3194833-3195245	0	nogene
chr15:71411686-71476935	0	THSD4
chr6:75272463-75272955	6	nogene
chr5:178211889-178231523	16	PHYKPL
chr13:24477700-24493733	183	PARP4
chr5:134158454-134174022	208	SKP1
chr5:143037195-143057747	38	ARHGAP26
chr10:37803832-37838111	9	ZNF248
chr14:29915175-29918288	1	nogene
chr13:45149769-45207505	32	GTF2F2
chr1:2135261-2150978	38	PRKCZ
chr5:139278326-139279129	272	SNHG4
chr15:55327530-55334007	7	PIGB
chr16:89895037-89904205	4	TCF25
chr20:49255807-49260577	1	ZNFX1
chr16:4259010-4259243	29	nogene
chr20:47246007-47254424	1	ZMYND8
chr6:85530191-85542043	23	SNX14
chr7:24744561-24749793	174	DFNA5
chr3:186105304-186105942	70	ETV5
chr16:58716013-58719255	79	GOT2
chr9:98060781-98060997	2	NANS
chr9:14179726-14307520	1	NFIB
chr10:34359146-34360259	15	PARD3
chr5:34922216-34923234	79	BRIX1
chr5:154294815-154329738	4	GALNT10
chr8:47729996-47730981	28	nogene
chr3:47423932-47428670	16	SCAP
chr11:47411925-47412467	13	SLC39A13
chr15:43432193-43438416	4	TP53BP1
chr8:61682438-61684188	2	ASPH
chr11:9283370-9299701	20	TMEM41B
chr9:2720343-2777994	0	nogene
chr8:140668268-140686694	0	PTK2

chr12:49496832-49500205	195	SPATS2
chr2:43485233-43508780	18	THADA
chr12:112090725-112093136	43	NAA25
chr11:36036102-36038184	18	LDLRAD3
chr7:156724111-156728720	17	LMBR1
chr6:100625191-100631213	21	ASCC3
chr2:20326260-20333063	56	PUM2
chr16:27533290-27545523	11	GTF3C1
chr16:50078740-50079018	20	HEATR3
chr1:212020121-212021212	4	INTS7
chr11:94435831-94447438	8	MRE11A
chr1:246186742-246207459	63	nogene
chr1:46269529-46269888	29	nogene
chr9:128459566-128461067	16	ODF2
chr6:147205970-147239270	72	STXBP5
chr1:172924598-172933643	5	nogene
chr20:35721739-35732135	87	RBM39
chr4:145033041-145064692	1	ANAPC10
chr1:175004613-175008706	55	CACYBP
chr20:63424176-63446837	27	KCNQ2
chr20:37207272-37210165	70	RPN2
chr15:63529013-63563008	161	USP3
chr12:10882216-10883096	5	PRH1
chr14:65455620-65458207	10	FUT8
chr16:70463168-70465176	73	FUK
chr1:205616477-205627874	29	nogene
chr8:100259106-100275161	23	RNF19A
chr2:201760880-201761818	314	ALS2
chr11:118581245-118592856	0	ARCN1
chr1:94177611-94178167	1	ARHGAP29
chr2:231206197-231259102	0	ARMC9
chr2:175080659-175121540	99	ATF2
chr22:41117186-41127748	0	EP300
chr12:56562416-56564227	1	RBMS2
chr9:2181570-2186228	14	SMARCA2
chr6:158083428-158084174	3	SYNJ2
chr10:101939862-102040125	1	C10orf76
chr12:96323947-96334865	369	CDK17
chr1:20893516-20904971	5	EIF4G3
chr9:122313226-122322745	64	MRRF
chr18:68897218-68897682	51	CCDC102B
chr7:66712166-66712957	15	RABGEF1
chr6:75817500-75832947	35	MYO6

chr17:27304779-27311616	9	WSB1
chr1:155415743-155439068	9	ASH1L
chr16:304303-314683	120	AXIN1
chr9:16552529-16583085	0	BNC2
chr1:150620889-150627592	0	ENSA
chr6:36470051-36484824	1	KCTD20
chr3:42630545-42632823	0	NKTR
chr11:65504325-65504595	0	nogene
chr12:65886621-65887306	0	nogene
chr17:78079394-78083166	1	TNRC6C
chr8:73805650-73825249	0	UBE2W
chr4:84708908-84721572	4	WDFY3
chr2:32290482-32307190	0	YIPF4
chr12:53033805-53037622	20	EIF4B
chr14:102870184-102871968	18	TRAF3
chr3:197130526-197140264	18	DLG1
chr3:134183001-134195182	51	RYK
chr10:12087534-12120267	1	DHTKD1
chr4:150588047-150683717	27	LRBA
chr10:13172375-13183100	25	MCM10
chr4:67728539-67730170	2	nogene
chr14:60114699-60125823	28	PCNXL4
chr2:223998114-224001922	128	SERPINE2
chr8:134509618-134532972	2	ZFAT
chr12:49460769-49500205	26	SPATS2
chr17:37568790-37585430	4	SYNRG
chr8:690897-692612	42	ERICH1
chr12:104991711-104996026	312	C12orf45
chr14:91188909-91199905	0	C14orf159
chr1:226295841-226301205	18	LIN9
chr19:12813082-12813565	103	RNASEH2A
chr20:38040434-38066256	154	RPRD1B
chr2:128113083-128129179	3	UGGT1
chr6:73440208-73440445	72	MB21D1
chr9:105520622-105520916	26	nogene
chr9:107302034-107325004	62	RAD23B
chr12:54182503-54183959	1	SMUG1
chr3:128897631-128904505	1	ACAD9
chr2:233449981-233452060	216	DGKD
chr8:43064100-43083180	0	FNTA
chr8:15623079-15673836	34	TUSC3
chr12:118079345-118082429	0	VSIG10
chr19:36058779-36068010	1	WDR62

chr9:37819287-37842286	15	DCAF10
chr18:21765771-21798228	25	MIB1
chr1:171523220-171527844	125	PRRC2C
chr6:57050773-57055797	83	KIAA1586
chr15:44379689-44380976	25	CASC4
chr7:2002064-2004848	38	MAD1L1
chr3:170136418-170136665	3	PHC3
chr15:79845157-79846856	46	nogene
chr11:12138389-12162419	39	MICAL2
chr2:159001079-159097834	12	TANC1
chr2:45546316-45562756	28	SRBD1
chr12:120177446-120190400	0	GCN1
chr9:100320264-100321771	25	TEX10
chr4:128466607-128470216	0	LOC100507487
chr8:144160969-144192401	0	MROH1
chr19:17159394-17168064	2	MYO9B
chr14:50792711-50822077	23	NIN
chr2:189791789-189844080	3	PMS1
chr17:29480367-29482288	0	TAOK1
chr21:41226265-41246063	20	BACE2
chr1:45654542-45659142	2	GPBP1L1
chr7:105285790-105297533	13	SRPK2
chr4:1900625-1904378	410	WHSC1
chr3:130930415-130941699	219	ATP2C1
chr10:43154896-43160595	28	CSGALNACT2
chr2:43918024-43918398	17	LRPPRC
chr17:31169890-31206371	28	NF1
chr17:7665713-7666178	13	nogene
chrX:140783174-140784659	4	nogene
chr22:38474232-38474623	2	KDELR3
chr12:79285786-79299551	2	SYT1
chr14:64439095-64441453	49	MTHFD1
chr19:43186341-43203973	7	PSG5
chr17:16164978-16171995	7	NCOR1
chr4:82866807-82872086	16	SEC31A
chr16:23424748-23445961	0	COG7
chr19:13808835-13809220	16	ZSWIM4
chr18:7754372-7755920	0	nogene
chr19:36081432-36084744	0	WDR62
chr7:74187610-74189918	1	EIF4H
chr6:163478779-163535125	2	QKI
chr3:8921538-8941804	13	RAD18
chr18:79410402-79433738	26	NFATC1

chr5:135172499-135174172	13	C5orf66
chr1:93674613-93674941	279	BCAR3
chr9:539515-594080	1	KANK1
chr1:223804173-223806947	185	TP53BP2
chr1:85321412-85324883	45	DDAH1
chr3:57890040-57909150	3	SLMAP
chr2:202755537-202759353	33	FAM117B
chr11:68780639-68793402	2	CPT1A
chr7:98937155-98937820	2	TRRAP
chr14:75100991-75107487	4	NEK9
chr5:160252882-160259771	20	CCNJL
chr1:42265114-42278672	300	FOXJ3
chr19:5071009-5082504	1	KDM4B
chr19:48127276-48128020	17	LIG1
chr16:12042896-12129758	5	SNX29
chr6:24416396-24418578	171	MRS2
chr9:120536371-120539164	16	CDK5RAP2
chr11:72867864-72921950	7	FCHSD2
chr10:92637183-92639900	24	KIF11
chr2:232701710-232702153	18	nogene
chr16:68859883-68867220	16	TANGO6
chr6:82957311-83044659	64	UBE3D
chr10:45625950-45639963	43	ZFAND4
chr3:44929305-44945292	4	ZDHHC3
chr17:82171700-82179189	14	CCDC57
chr7:22960255-22964531	51	FAM126A
chr13:31147958-31150182	14	HSPH1
chr11:14867505-14869630	23	PDE3B
chr2:227531090-227536993	73	AGFG1
chr11:108227594-108229323	164	ATM
chr15:74619196-74620225	26	CLK3
chr5:180549930-180553471	158	CNOT6
chr20:17969800-17970130	27	MGME1
chr7:158644288-158646563	243	NCAPG2
chr19:3568768-3569352	17	nogene
chr16:3067983-3081818	41	IL32
chr4:147946616-147966839	198	ARHGAP10
chr13:26337566-26353880	469	CDK8
chr7:6425987-6435020	78	DAGLB
chr9:93527154-93543471	1	FAM120A
chr7:130120748-130122202	125	KLHDC10
chr14:50199690-50204409	11	SOS2
chr1:120160383-120163370	0	SEC22B

chr2:32087491-32116212	0	SPAST
chrX:123888618-123900693	4	XIAP
chr3:183736717-183762279	9	YEATS2
chr1:45421724-45457871	4	TESK2
chr11:31456259-31473786	7	IMMP1L
chr12:108813128-108813482	13	nogene
chr11:33043749-33054725	23	TCP11L1
chr2:26230191-26234355	10	HADHA
chr2:63196121-63201291	1	nogene
chr3:15735409-15737233	11	ANKRD28
chr12:123340987-123350441	14	SBNO1
chr12:116985205-116988869	287	FBXW8
chr10:80468546-80489314	26	TSPAN14
chr4:41013582-41014398	227	APBB2
chr10:42787167-42790511	122	BMS1
chr3:113399905-113416627	8	CFAP44
chr21:46496987-46511614	60	DIP2A
chr20:18427066-18443464	0	DZANK1
chr9:93470328-93498886	38	FAM120A
chr9:127332273-127339751	20	GARNL3
chr9:96458378-96484659	1	HABP4
chr12:26695605-26716242	3	ITPR2
chr22:33761370-33830209	4	LARGE
chr2:43912431-43948518	0	LRPPRC
chr14:75103841-75124223	15	NEK9
chr3:170097171-170128836	5	nogene
chr16:10747142-10752678	6	NUBP1
chr17:59697561-59697978	0	PTRH2
chr2:32193888-32209561	0	SLC30A6
chr1:24646666-24649029	20	SRRM1
chr14:21211809-21234229	26	HNRNPC
chr7:156724111-156763795	14	LMBR1
chr18:79096475-79154555	94	ATP9B
chr1:235470849-235496308	40	B3GALNT2
chr3:32733422-32737490	85	CNOT10
chr8:73947289-73947932	20	nogene
chr20:44582584-44589866	16	PKIG
chr9:92256679-92269983	300	IARS
chr10:68393137-68396881	16	RUFY2
chr11:125602302-125604247	42	STT3A
chr1:113829591-113834439	5	PTPN22
chr14:30581075-30589484	1	G2E3
chrX:85067126-85074391	165	APOOL

chr1:243293090-243344331	13	SDCCAG8
chr1:224398096-224399034	8	WDR26
chr3:141913651-141922063	27	ATP1B3
chr4:77058506-77066405	13	CCNI
chr1:100995109-101021765	19	DPH5
chr17:79078328-79079154	21	nogene
chr4:84678167-84679242	23	WDFY3
chr2:9502172-9510131	3	ADAM17
chr22:32478980-32487828	33	FBXO7
chr16:70563875-70565524	18	SF3B3
chr4:147939824-147966839	193	ARHGAP10
chr5:138319218-138328529	102	CDC25C
chr5:131315975-131316144	16	CDC42SE2
chr6:130885095-130890466	1	EPB41L2
chr16:57153810-57173869	5	FAM192A
chr7:65891227-65899620	20	nogene
chr4:80301282-80420400	3	nogene
chr2:135162554-135176443	38	RAB3GAP1
chr20:37044085-37047190	24	RBL1
chr2:223878528-223894339	1	WDFY1
chr12:76841962-76846679	13	ZDHHC17
chr21:45120793-45134832	19	ADARB1
chr1:212016885-212021212	2	INTS7
chr16:70226769-70231158	0	nogene
chr6:37279303-37291357	31	TBC1D22B
chr14:101994985-101995300	4	DYNC1H1
chr14:96552479-96556413	0	PAPOLA
chr19:18863437-18864251	45	UPF1
chr3:45089053-45093657	20	CDCP1
chr6:127314815-127331030	33	ECHDC1
chr8:47595810-47612965	12	SPIDR
chr15:49292326-49292537	23	GALK2
chr3:119863418-119905852	146	GSK3B
chr15:22534067-22534243	30	HERC2P2
chr3:141509567-141540609	73	RASA2
chr4:25154997-25159107	168	SEPSECS
chr17:4282797-4307123	321	UBE2G1
chr2:32401162-32406557	3	BIRC6
chr16:68287322-68287871	33	SLC7A6
chr10:91939951-91942568	39	BTAF1
chr5:138191473-138202155	3	CDC23
chr14:102999564-103012188	50	CDC42BPB
chr3:45095346-45110841	0	CDCP1

chr14:80497468-80530886	123	CEP128
chr19:14409893-14410215	14	DDX39A
chr1:15536474-15536814	25	DNAJC16
chr1:206597099-206600308	0	EIF2D
chr19:55688869-55689950	0	EPN1
chr7:8003907-8071131	24	GLCCI1
chr9:92249857-92251885	0	IARS
chr12:30649136-30653092	0	IPO8
chr6:18161326-18166378	14	KDM1B
chr13:23805949-23841488	79	MIPEP
chr9:22301599-22303231	5	nogene
chrX:68192918-68299096	23	OPHN1
chr7:24820174-24842413	0	OSBPL3
chr2:241325992-241343093	41	2-Sep
chr17:19542392-19549677	47	SLC47A1
chr6:85557975-85574378	257	SNX14
chr6:35888814-35891013	0	SRPK1
chr15:43385790-43386330	14	TUBGCP4
chr11:47797778-47798463	31	NUP160
chr10:112527086-112668998	6	VTI1A
chrX:150793690-150795486	18	CD99L2
chr7:130203787-130204110	27	TMEM209
chr3:123223558-123259589	149	SEC22A
chr18:45928868-45952643	26	EPG5
chr1:32036909-32039573	70	KHDRBS1
chr14:88626837-88644511	18	EML5
chr10:68025545-68044558	84	HERC4
chr5:145796441-145823134	26	PRELID2
chr3:47610065-47638780	23	SMARCC1
chr20:36607714-36609730	156	TGIF2-C20orf24
chr8:91018389-91021404	20	TMEM55A
chr2:61498672-61526521	15	XPO1
chr17:4051970-4058155	12	ZZEF1
chr3:10046579-10049505	102	FANCD2
chr7:128574719-128592017	4	nogene
chr1:1637761-1704655	151	CDK11B
chr5:173090314-173091401	9	CREBRF
chr20:49849578-49864844	2	SLC9A8
chr12:98677424-98686873	8	APAF1
chr1:243552728-243843282	1	AKT3
chr15:34003031-34005851	18	AVEN
chr18:35974895-35977600	0	C18orf21
chr9:36197550-36204179	0	CLTA

chr11:44114184-44130138	4	EXT2
chr2:156549607-156557582	12	GPD2
chr18:23515855-23516423	18	C18orf8
chr10:7363877-7381949	2	SFMBT2
chr2:43571706-43575027	5	THADA
chr3:179719939-179721589	56	USP13
chr5:77449759-77463226	10	WDR41
chr11:34957383-34984728	58	PDHX
chr19:40736985-40737769	34	ITPKC
chr3:142777631-142792967	23	TRPC1
chr7:2244562-2250002	35	NUDT1
chr1:92702140-92704744	29	EVI5
chr8:73552011-73688813	2	STAU2
chr2:61284874-61293550	56	USP34
chr5:179705678-179709059	184	CANX
chr7:44700140-44701615	35	OGDH
chr6:52469332-52479787	31	EFHC1
chr6:37279303-37284464	12	TBC1D22B
chr4:40890363-40934699	1	APBB2
chr3:57583897-57584464	148	ARF4
chr11:74113779-74123135	0	C2CD3
chr6:17507168-17513954	143	CAP2
chr6:24475120-24479959	31	GPLD1
chr11:68403482-68416527	12	LRP5
chr11:129266866-129269715	12	nogene
chr1:113705889-113712113	1	PHTF1
chr1:64777622-64789514	102	RAVER2
chr3:141993537-142093127	1	TFDP2
chrX:123696020-123703505	12	THOC2
chr9:83677726-83686155	211	UBQLN1
chr8:38347496-38348215	23	WHSC1L1
chr3:195306510-195333123	27	ACAP2
chr2:38343267-38343512	8	ATL2
chr10:102023476-102040125	26	C10orf76
chr9:120536371-120568388	4	CDK5RAP2
chr2:85368685-85369830	4	ELMOD3
chr7:102854776-102884312	50	FBXL13
chr10:27165063-27165539	65	MASTL
chr6:89749222-89750532	27	MDN1
chr22:37678064-37678848	12	nogene
chr1:5927648-5986327	56	NPHP4
chr13:28266714-28270866	19	PAN3
chr12:110545879-110551968	31	PPTC7

chr3:149852515-149921227	3	RNF13
chr5:74750578-74751493	28	GFM2
chr22:38499399-38501280	14	DDX17
chr19:41245144-41245508	18	nogene
chr17:5308693-5332152	45	RABEP1
chr11:108315822-108317521	17	ATM
chr8:123021446-123030716	54	DERL1
chr11:32588593-32589135	13	EIF3M
chr11:44107682-44171742	33	EXT2
chr3:172295362-172310881	59	FNDC3B
chr17:82571723-82587272	16	FOXK2
chr1:117918596-117920424	40	GDAP2
chr10:7778837-7780307	13	KIN
chr19:49866527-49867021	29	nogene
chr14:35307993-35310895	2	PSMA6
chr11:18086865-18089510	16	SAAL1
chr2:241342991-241343897	28	2-Sep
chr1:100896569-100921841	0	SLC30A7
chr4:183663846-183668117	17	TRAPPC11
chr18:74781427-74920692	1	ZNF407
chr20:49639677-49643650	2	B4GALT5
chr14:80878745-80906081	0	CEP128
chr9:89314719-89315297	13	CKS2
chr13:42155290-42174144	0	DGKH
chr19:47362475-47362693	331	DHX34
chr10:119051198-119056853	15	EIF3A
chr7:148817876-148819687	18	EZH2
chr5:137985256-138019146	3	FAM13B
chr5:108897658-108959347	3	FER
chr5:109781395-109804277	37	MAN2A1
chr7:131375423-131397376	0	MKLN1
chr6:75822781-75832947	19	MYO6
chr4:80287336-80301329	3	nogene
chr10:37745216-37745548	1	nogene
chr7:44484967-44485622	1	NUDCD3
chr11:47791929-47801930	0	NUP160
chr3:111112029-111147484	135	PVRL3
chr5:69373829-69374711	20	RAD17
chr19:19198106-19199234	11	RFXANK
chr10:100937401-100950753	71	SLF2
chr18:47851273-47870564	0	SMAD2
chr8:76704042-76778439	0	ZFHX4
chr12:106842588-106860412	20	RIC8B

chr19:14479411-14480778	12	GIPC1
chr2:171028338-171046362	193	TLK1
chr17:37330172-37339850	7	ACACA
chr15:61094355-61112119	15	nogene
chr17:45513867-45517282	120	LRRC37A4P
chr6:15651312-15660521	1	DTNBP1
chr1:88770351-88786213	220	PKN2
chr10:102023476-102025248	11	C10orf76
chr4:2630934-2639859	18	FAM193A
chr1:204525483-204532246	14	MDM4
chr20:58667489-58671297	37	STX16
chr4:17909093-17972885	13	LCORL
chr5:65170616-65226219	12	ADAMTS6
chr10:4830674-4842504	71	AKR1E2
chr13:111217678-111233293	18	ARHGEF7
chr15:72566562-72572165	28	ARIH1
chr21:29305203-29329693	115	BACH1
chr6:34606554-34646798	164	C6orf106
chr11:128758114-128772985	39	FLI1
chr18:47130243-47136449	20	HDHD2
chr1:146943290-146946607	6	NBPF12
chrX:126243345-126302446	1	nogene
chr4:13425109-13428299	29	nogene
chr9:97138642-97139954	61	nogene
chr6:124996574-125058106	1	RNF217
chr19:11723232-11725327	1	ZNF823
chr2:27987992-28129380	7	BRE
chr1:16130765-16131348	19	nogene
chr1:155925631-155927156	56	KIAA0907
chr5:134942480-134960728	1	nogene
chr10:11936544-11952249	15	UPF2
chr6:150882761-150887981	2	MTHFD1L
chr2:219213589-219214186	0	ABCB6
chr2:222933165-222934687	0	ACSL3
chr19:40458154-40458545	48	BLVRB
chr10:32451591-32474023	310	CCDC7
chr6:117519815-117545597	4	DCBLD1
chr6:35312301-35312772	17	DEF6
chr20:18441592-18443464	0	DZANK1
chr11:128484822-128486146	0	ETS1
chr18:21366026-21403994	116	GREB1L
chr17:44082584-44083854	1	HDAC5
chr6:29888466-29943026	2859	HLA-H

chr3:129288853-129290804	0	HMCES
chrX:53645310-53680186	57	HUWE1
chr2:202785917-202819899	12	ICA1L
chrX:51895052-51897271	14	MAGED1
chr2:74159090-74172752	16	MOB1A
chr18:36215515-36220217	26	MOCOS
chr18:36203112-36213482	2	MOCOS
chr9:98075569-98078347	61	NANS
chr5:142131807-142144676	42	NDFIP1
chr7:93786566-93797279	16	nogene
chr1:36405818-36410593	0	nogene
chr21:45728675-45738571	0	nogene
chr10:67975124-67975444	18	nogene
chr8:144402866-144405295	2	nogene
chr12:87566994-87715112	0	nogene
chr4:39895496-39896758	0	nogene
chr19:29645261-29664207	0	nogene
chr6:85471236-85471425	0	NT5E
chr1:212329134-212345933	1	PPP2R5A
chr19:799412-806556	20	PTBP1
chr14:67823541-67865139	55	RAD51B
chr3:49972058-50054395	0	RBM6
chr17:32173830-32192299	13	RHOT1
chr7:105551569-105555227	2	RINT1
chr5:179593477-179594963	7	RUFY1
chr1:179302676-179307216	1	SOAT1
chr6:42612170-42617507	32	UBR2
chr11:102203987-102206074	7	YAP1
chr8:47712661-47713641	24	SPIDR
chr11:43815436-43840064	7	HSD17B12
chr7:111214884-111216267	12	nogene
chr4:61934839-61948276	4	ADGRL3
chr2:215332381-215333457	1	ATIC
chr7:33604864-33642918	1	BBS9
chr4:3086938-3099394	6	HTT
chr15:78465250-78466489	40	IREB2
chr1:202755270-202764145	4	KDM5B
chr7:98137314-98159425	24	LMTK2
chr4:150870524-150872755	6	LRBA
chr5:151853271-151857893	1	nogene
chr6:33302906-33303205	7	nogene
chr12:123012612-123014042	60	PITPNM2
chr3:156677656-156696025	80	TIPARP

chr19:45521321-45522818	93	VASP
chr6:18236451-18258405	109	DEK
chr6:90560075-90571807	18	MAP3K7
chr22:28296197-28297852	785	TTC28
chr5:141928150-141930274	12	KIAA0141
chr4:23592788-23634627	12	nogene
chr4:159238808-159304473	12	RAPGEF2
chr4:122927636-122947488	12	SPATA5
chr12:106245903-106246469	23	nogene
chr5:69585593-69586356	37	GTF2H2B
chr17:59731420-59735473	10	VMP1
chr1:24797741-24827116	43	CLIC4
chr1:6231901-6232119	42	ICMT
chr16:27708704-27708920	2	KIAA0556
chr4:56403039-56406701	34	PPAT
chr1:145747994-145751510	1	RNF115
chr12:111470107-111470742	32	ATXN2
chr12:78049992-78059115	202	NAV3
chr1:235419472-235430804	4	TBCE
chr7:152263015-152265209	145	KMT2C
chr1:25989447-25990863	1	PAFAH2
chr13:19741564-19759418	37	PSPC1
chr12:95251939-95266632	17	VEZT
chr1:92563641-92607727	52	EVI5
chr5:108946129-108959347	94	FER
chr6:87621353-87622013	14	ORC3
chr7:98899421-98903517	1	TRRAP
chr5:65988634-65994864	1429	ERBB2IP
chr17:83084937-83085323	12	METRNL
chr6:159775169-159777456	2	ACAT2
chr1:203710876-203722689	0	ATP2B4
chr19:38838396-38840559	0	HNRNPL
chr3:47706408-47714490	27	SMARCC1
chr13:24286213-24290884	15	SPATA13
chr17:82889667-82900731	18	TBCD
chr19:34443843-34445121	40	UBA2
chr9:36344831-36353331	3	RNF38
chr9:19093170-19096769	20	HAUS6
chr1:203720548-203722689	0	ATP2B4
chr4:42503449-42543986	17	ATP8A1
chr2:170852713-170854022	19	GAD1
chr14:92803060-92806963	0	GOLGA5
chr8:42957093-42964474	371	HOOK3

chr4:142145839-142160561	2	INPP4B
chr10:7759889-7766103	24	KIN
chr10:72475099-72508269	16	MICU1
chr21:36333645-36349408	2	MORC3
chr1:43588730-43592601	17	PTPRF
chr1:176116623-176149074	36	RFWD2
chr12:120554717-120565529	1	RNF10
chr1:77736055-77739417	0	USP33
chr1:85775673-85786461	1	COL24A1
chr2:190659157-190687317	5	NAB1
chr9:105721536-105748190	37	TMEM38B
chr4:1900625-1935262	367	WHSC1
chr7:139053450-139055295	3	ZC3HAV1
chr17:60679452-60747279	30	BCAS3
chr4:151482523-151588926	8	FAM160A1
chr1:226265532-226287797	11	LIN9
chr6:43055896-43056445	19	MRPL2
chr18:12370848-12371691	21	AFG3L2
chr15:85639373-85669830	0	AKAP13
chr2:111872629-111880849	27	ANAPC1
chr19:16506700-16509988	0	C19orf44
chr20:3916926-3918796	12	PANK2
chr2:70214348-70216994	2	TIA1
chr3:9810620-9813345	9	TTLL3
chr2:169891164-169896735	12	UBR3
chr17:42728824-42730918	3	EZH1
chr10:68973544-68974743	40	DDX21
chr17:75660954-75661708	3	RECQL5
chr1:225123526-225185425	3	DNAH14
chr2:118096418-118109900	17	INSIG2
chr7:66638252-66639228	1	KCTD7
chr8:58631495-58635546	1	NSMAF
chr2:171006146-171007063	2	TLK1
chr10:74089195-74094461	31	VCL
chr11:129040927-129164427	38	ARHGAP32
chr12:68696818-68702784	9	NUP107
chr3:141368620-141404015	1	ZBTB38
chr2:202275112-202282472	12	NOP58
chr5:177191883-177239865	12	NSD1
chr9:128122361-128123134	12	PTGES2
chr5:140537337-140539083	1	ANKHD1-EIF4EBP3
chr13:40942951-40943925	0	ELF1
chr9:100264821-100284603	0	INVS

chr10:88309386-88314641	12	RNLS
chr4:76163224-76166301	0	SCARB2
chr20:62859363-62860308	0	TCFL5
chr5:14297071-14336727	20	TRIO
chr2:99321954-99327653	7	TXNDC9
chrX:23730526-23736016	1	ACOT9
chr1:97515725-97573970	1	DPYD
chr17:47590716-47592545	10	NPEPPS
chr3:149846010-149902162	19	RNF13
chr5:179567460-179577136	17	RUFY1
chr16:28121669-28132403	5	XPO6
chr4:142108092-142129023	1	INPP4B
chr16:68266592-68297476	20	SLC7A6
chr12:113267842-113269845	28	TPCN1
chr16:28101457-28101945	95	XPO6
chr6:116399197-116399662	15	DSE
chr15:90439331-90441684	7	IQGAP1
chr1:70306223-70315566	0	ANKRD13C
chr9:127788692-127789137	0	CDK9
chr19:19465339-19474167	0	GATAD2A
chr10:842931-885760	19	LARP4B
chr18:14265934-14266153	0	nogene
chr2:27583657-27608039	0	ZNF512
chr11:72984087-73083740	23	FCHSD2
chr18:42037691-42049605	6	PIK3C3
chrX:155298784-155299145	27	CLIC2
chr16:13934191-13935743	82	ERCC4
chr6:44248629-44253598	1	HSP90AB1
chr4:142082030-142112682	2	INPP4B
chr19:57843848-57844240	26	nogene
chr6:73638411-73641924	3	SLC17A5
chr3:98756368-98773983	16	ST3GAL6
chr20:18628126-18744271	1	DTD1
chr9:135850136-135883078	171	CAMSAP1
chr5:123545416-123557564	631	CSNK1G3
chr4:24554724-24570847	18	DHX15
chr8:140800458-140819020	29	PTK2
chr17:64212573-64214242	102	TEX2
chr11:61366117-61367998	7	TMEM138
chr16:58574608-58583182	78	CNOT1
chr3:10087133-10088950	18	FANCD2
chr5:14673641-14693094	70	OTULIN
chr2:65090982-65104806	7	RAB1A

chr20:13623445-13630152	71	TASP1
chr4:1900625-1918623	262	WHSC1
chr22:28694031-28695873	83	CHEK2
chr15:65702304-65706224	28	DENND4A
chr9:19350701-19352693	12	DENND4C
chr9:83663874-83669327	1	UBQLN1
chr19:12623699-12624710	27	ZNF791
chr4:83467453-83472288	0	FAM175A
chr12:68733451-68735344	17	NUP107
chr19:5653114-5657347	12	SAFB
chr2:63856305-63857936	373	UGP2
chr5:80455691-80474830	5	ZFYVE16
chr17:42838092-42839012	5	PSME3
chr8:30612298-30635123	1	GTF2E2
chr5:115831321-115837764	2	ATG12
chr12:50453459-50467120	21	LARP4
chr1:179063926-179066859	0	FAM20B
chr2:32501712-32505205	12	BIRC6
chr1:20744743-20745656	12	HP1BP3
chr6:17632649-17640064	16	NUP153
chr20:37203884-37213865	99	RPN2
chr21:37108391-37138714	2	TTC3
chr5:149509878-149513209	57	CSNK1A1
chr4:47744401-47786925	12	CORIN
chr12:31398174-31402643	1	DENND5B
chr10:37833024-37838111	1	ZNF248
chr11:64320660-64321874	5	PRDX5
chr11:119271734-119274953	35	CBL
chr7:137912990-137916012	20	CREB3L2
chr3:155893517-155925366	0	GMPS
chr14:44945029-44945928	10	KLHL28
chr4:80295558-80420400	0	nogene
chr11:65326387-65326786	19	nogene
chr2:33563477-33567204	0	nogene
chr9:122957010-122990213	0	RABGAP1
chr5:814760-825251	0	ZDHHC11
chr14:24209889-24211829	14	CHMP4A
chr22:29571938-29572179	1	nogene
chr4:22389087-22413814	32	ADGRA3
chr1:15767375-15768630	18	FBLIM1
chr7:64814917-64817335	37	ZNF138
chr6:36894503-36914693	7	C6orf89
chr7:92350949-92371607	17	ANKIB1

chr22:45692995-45718493	25	ATXN10
chr10:35031299-35033273	6	CUL2
chr3:197085579-197116083	47	DLG1
chr8:61680967-61684188	4922	ASPH
chr6:43012925-43013389	3	MEA1
chr18:21838364-21844253	9	MIB1
chr6:107921796-107924932	15	SEC63
chr3:9365015-9371536	15	THUMPD3
chr17:19920035-19963022	23	AKAP10
chr7:157339971-157367483	2	DNAJB6
chr2:47408400-47429941	10	MSH2
chr10:32726733-32729457	3	CCDC7
chr1:158072178-158072661	33	nogene
chr15:28245880-28263169	15	HERC2
chr11:118551785-118559864	35	IFT46
chr1:41070594-41161432	21	SCMH1
chr9:137586966-137588718	20	ZMYND19
chr8:102838743-102843747	85	AZIN1
chr2:10654480-10668723	24	NOL10
chr7:208897-248430	50	FAM20C
chr5:75354502-75355522	5	HMGCR
chr9:22820737-22821707	33	LINC01239
chr4:185247293-185264498	12	SNX25
chr12:51073822-51074082	56	CSRNP2
chr6:53269086-53291963	147	ELOVL5
chr15:49588015-49611268	21	FAM227B
chr6:52266074-52273916	0	MCM3
chr6:129539175-129558166	3	nogene
chr13:40602879-40603334	0	nogene
chr6:63646526-63646795	19	PHF3
chr4:1108342-1111821	158	RNF212
chr20:44503814-44506996	14	SERINC3
chr10:100937401-100956537	0	SLF2
chr4:26717932-26748526	21	TBC1D19
chr13:113635976-113640375	0	TFDP1
chr6:158501677-158504178	18	TULP4
chr4:110506636-110520366	12	ENPEP
chr5:21491320-21501826	63	GUSBP1
chr11:65434478-65435145	20	nogene
chr7:135635580-135638683	12	NUP205
chr11:581491-587464	60	PHRF1
chr21:43004325-43013238	31	PKNOX1
chr16:70556178-70561784	14	SF3B3

chr13:60444682-60483846	12	TDRD3
chr18:76849525-76871825	276	ZNF236
chr14:35651804-35659196	52	RALGAPA1
chr19:48999318-48999833	16	RUVBL2
chr4:53383772-53414722	2	FIP1L1
chr1:117402185-117502970	1	MAN1A2
chr5:10381799-10394785	13	6-Mar
chr10:93363960-93369776	10	MYOF
chr20:35727252-35727606	20	nogene
chr8:42444645-42465917	0	SLC20A2
chr7:157216866-157257044	0	UBE3C
chr2:27238430-27239798	28	CAD
chr7:23611170-23611553	725	CCDC126
chr1:32657426-32662337	13	RBBP4
chr13:59833106-59861536	3	DIAPH3
chr1:225185290-225207220	18	DNAH14
chr12:112229697-112233085	7	HECTD4
chr1:43569589-43592601	27	PTPRF
chr20:36660336-36671397	39	NDRG3
chr1:197539963-197583253	4	DENND1B
chr6:160046497-160048543	94	IGF2R
chr1:45334390-45334967	7	MUTYH
chr1:213104453-213176492	0	RPS6KC1
chr10:68401619-68404844	1	RUFY2
chr3:100713642-100728858	0	TFG
chr7:19055252-19056165	17	nogene
chr16:57248536-57250507	3	ARL2BP
chr17:50964549-50964840	12	nogene
chr12:76450850-76487618	43	OSBPL8
chr19:34464025-34467014	15	UBA2
chr3:130967144-130969396	31	ATP2C1
chr10:96858291-96907747	47	LCOR
chrX:154399337-154399941	15	RPL10
chr19:17197791-17200829	2	MYO9B
chr12:122557383-122562699	25	KNTC1
chr12:50179828-50180440	16	nogene
chr8:10826151-10834775	82	PINX1
chr7:66799322-66805396	23	RABGEF1
chr17:56847921-56862251	2	DGKE
chr8:73688653-73739868	103	STAU2
chr11:61334619-61345946	18	TKFC
chr10:102361021-102363798	12	GBF1
chr9:95907077-95928864	2	ERCC6L2

chr8:43064100-43072307	26	FNTA
chr11:68505144-68537891	78	PPP6R3
chr6:45003652-45365301	51	SUPT3H
chr10:13197622-13198807	23	MCM10
chr3:122437169-122452064	10	KPNA1
chr14:103654563-103657776	17	KLC1
chr18:12718516-12720683	38	PSMG2
chr15:51869216-51902036	19	TMOD3
chr19:56576712-56578278	9	ZNF470
chr3:197115926-197119530	366	DLG1
chr9:69732138-69750950	40	PTAR1
chr19:10143765-10143987	19	DNMT1
chr1:44757555-44759348	17	KIF2C
chr17:82889667-82903478	19	TBCD
chr15:63774693-63775649	47	HERC1
chr16:48258617-48262872	25	LONP2
chr6:52267864-52281674	13	MCM3
chr9:22301599-22467906	1	nogene
chr10:110964124-110985765	275	SHOC2
chr16:8929368-8930397	173	USP7
chr7:73700377-73700931	3	nogene
chr18:46890579-46891054	98	PIAS2
chr18:79193182-79277196	0	ATP9B
chr10:132749516-132777782	0	INPP5A
chr4:123028185-123090705	16	SPATA5
chr12:78864997-79047362	0	SYT1
chr4:1900625-1901251	10	WHSC1
chr14:103662115-103670283	6	KLC1
chr16:53919557-53934109	0	FTO
chr15:41053928-41056706	0	INO80
chr3:51490154-51492488	0	nogene
chr14:105352377-105355177	0	PACS2
chr1:113705889-113706722	0	PHTF1
chr7:66991136-66994282	9	SBDS
chr1:93582300-93592393	14	BCAR3
chr11:130260855-130261929	500	ZBTB44
chr5:14270824-14280436	167	TRIO
chr8:53795646-53817530	4	ATP6V1H
chr10:111000414-111004794	1	SHOC2
chr16:4724749-4727177	14	ANKS3
chr12:124362125-124363799	46	NCOR2
chr10:27526827-27533994	40	RAB18
chr7:1009949-1010300	15	C7orf50

chr19:879936-881714	18	MED16
chr1:955922-957273	61	NOC2L
chr11:126203344-126205781	36	RPUSD4
chr1:1564604-1564916	97	SSU72
chr19:52282110-52283413	429	ZNF766
chr2:200997444-201011472	0	FAM126B
chr5:138561579-138567561	19	HSPA9
chr1:25983945-25984528	0	PAFAH2
chr13:24446680-24447186	1	PARP4
chr19:29980158-30012531	26	URI1
chr19:58260850-58263676	43	ZNF544
chr5:139614696-139623461	11	UBE2D2
chr12:118019071-118022359	27	RFC5
chrX:155116056-155120168	34	BRCC3
chr20:26018831-26019924	24	LOC100134868
chr15:40571603-40571900	13	RPUSD2
chr17:58999380-59001714	8	TRIM37
chr12:89603042-89604346	13	ATP2B1
chr11:68793314-68796933	48	CPT1A
chr7:6737075-6737936	26	PMS2CL
chr18:54277702-54287411	152	POLI
chr19:38141183-38142710	264	SIPA1L3
chr7:92294888-92333720	27	ANKIB1
chr1:156785469-156785847	26	nogene
chr17:69255542-69256283	4	ABCA5
chr12:68715626-68731719	21	NUP107
chr5:14270824-14297263	19	TRIO
chr1:150817919-150823345	6	ARNT
chr10:68906710-68910382	42	DDX50
chr8:61548070-61567318	5	ASPH
chr6:84194883-84200905	13	CEP162
chr10:68430435-68437241	14	DNA2
chr1:51402434-51406108	3	EPS15
chr12:1289851-1408247	94	ERC1
chr4:83463493-83469151	23	FAM175A
chr4:41406138-41412198	1	nogene
chr12:116230532-116240462	15	nogene
chr21:41847089-41860372	1	PRDM15
chr1:52854464-52867777	13	ZYG11A
chr16:30664214-30667622	29	FBRS
chr6:26426241-26428785	13	BTN2A3P
chr2:8888017-8958642	56	MBOAT2
chr17:54938923-54947312	23	TOM1L1

chr8:142343965-142354763	1	TSNARE1
chr14:90644047-90680535	14	TTC7B
chr7:33367766-33388144	71	BBS9
chr7:55982209-55984878	23	GBAS
chr22:46310827-46311172	16	nogene
chr10:15833629-15843272	38	FAM188A
chr21:44032090-44052476	31	TRAPPC10
chr13:75326196-75349302	4	TBC1D4
chr20:43613849-43620925	26	IFT52
chr16:47311239-47459175	26	ITFG1
chr1:85025938-85045362	18	MCOLN3
chrX:65075843-65113813	8	nogene
chr10:122393180-122424959	1	PLEKHA1
chr9:6843508-6893232	0	KDM4C
chr18:23763404-23784157	0	LAMA3
chr2:72968447-73058596	0	SFXN5
chr6:161022009-161034449	175	MAP3K4
chr11:77619605-77625818	499	CLNS1A
chr11:65894013-65894497	4	FOSL1
chr17:281767-321415	56	RPH3AL
chr6:129629352-129634105	27	ARHGAP18
chr3:197814907-197817302	8	LRCH3
chr4:1918140-1935262	20	WHSC1
chr12:328834-334422	0	KDM5A
chr3:101398860-101417790	0	SENP7
chr17:82571723-82584188	19	FOXK2
chr17:80994580-80995758	29	CHMP6
chr17:47328268-47345098	14	EFCAB13
chr5:177619077-177632738	67	LOC202181
chr3:57657679-57672438	35	DENND6A
chr2:157857464-157860179	4	nogene
chr1:93648040-93650141	12	nogene
chr20:17955364-17957037	125	SNX5
chr10:69155811-69158211	28	VPS26A
chr7:66795510-66810847	2	RABGEF1
chr12:111525191-111555919	20	ATXN2
chr11:104950924-105000495	51	CASP4
chr9:92268173-92269983	298	IARS
chr1:235434203-235437474	9	TBCE
chr12:121502582-121503065	17	nogene
chr10:18556308-18558216	83	nogene
chr2:241325992-241343897	3	2-Sep
chr15:65722847-65732818	1	DENND4A

chr6:130022226-130094367	12	L3MBTL3
chr20:20022455-20026919	4	NAA20
chr2:218463305-218466176	42	USP37
chr7:6135840-6145656	12	USP42
chr16:11894953-11896785	121	GSPT1
chr8:98526741-98548161	57	STK3
chr5:14286870-14336727	74	TRIO
chr13:23805949-23886506	388	MIPEP
chr20:33669374-33672422	1	NECAB3
chr3:51416786-51418876	8	VPRBP
chr4:20709682-20727397	1	PACRGL
chr8:81670938-81681584	68	IMPA1
chr5:72887069-72888303	17	TNPO1
chr19:8455404-8471427	24	HNRNPM
chr14:54995602-55002185	18	WDHD1
chr15:89113724-89155534	14	ABHD2
chr2:65239851-65265175	14	ACTR2
chr2:9517900-9543285	18	ADAM17
chr12:98706484-98708704	0	APAF1
chr12:111485270-111486824	81	ATXN2
chr2:74526717-74528935	0	AUP1
chr3:113249721-113250833	14	BOC
chr11:86306244-86306482	30	C11orf73
chr6:31835410-31837435	2	C6orf48
chrX:15692962-15764775	0	CA5BP1
chr3:45112082-45112445	3	CDCP1
chr5:116091078-116092700	21	COMMD10
chr2:203289764-203296563	26	CYP20A1
chr2:135930481-135933990	1	DARS
chr8:141175199-141180854	33	DENND3
chr3:154280578-154295339	0	DHX36
chr10:77853353-77856892	25	DLG5
chr21:36237261-36238701	0	DOPEY2
chr15:55385599-55399929	0	DYX1C1-CCPG1
chr7:36281114-36297187	35	EEPD1
chrX:24057420-24057754	72	EIF2S3
chr7:2376949-2379511	12	EIF3B
chr1:28993329-28997319	17	EPB41
chr1:44247938-44339322	23	ERI3
chr4:158697558-158703591	1	ETFDH
chr10:92893348-92895020	6	EXOC6
chr12:117004435-117010450	11	FBXW8
chr8:11808793-11809850	30	FDFT1

chr7:65960951-65976202	1	GUSB
chr14:61732417-61734285	0	HIF1A
chr1:212006638-212017023	13	INTS7
chr7:130094894-130116666	0	KLHDC10
chr1:225412453-225419798	11	LBR
chr7:130945719-131052555	4	LINC-PINT
chr13:23837551-23886506	63	MIPEP
chr10:21614830-21651768	2	MLLT10
chr6:30619492-30619989	74	MRPS18B
chr12:76053090-76053909	107	NAP1L1
chr16:15694164-15696860	4	NDE1
chr17:35139234-35140066	33	NLE1
chr16:58150393-58150686	0	nogene
chr2:203152168-203152375	0	nogene
chr3:129857249-129880594	0	nogene
chr1:151300529-151301325	0	nogene
chr22:35388704-35389537	1	nogene
chr3:189343453-189385040	0	nogene
chr7:66742726-66743178	34	nogene
chr16:70156473-70156870	0	nogene
chrX:2345569-2345915	0	nogene
chr11:102192824-102204122	0	nogene
chr17:46692902-46728934	1	NSF
chr21:46381693-46385983	75	PCNT
chr4:88038250-88065879	2	PKD2
chr4:105396248-105434161	25	PPA2
chr5:134200984-134206131	0	PPP2CA
chr1:3186124-3186474	3	PRDM16
chr6:31623731-31628239	0	PRRC2A
chr1:31918969-31919364	0	PTP4A2
chr8:127969415-127989291	14	PVT1
chr8:60569954-60584815	0	RAB2A
chr2:226864603-226908878	15	RHBDD1
chr19:32990513-32991969	14	RHPN2
chr7:105542407-105555227	0	RINT1
chr12:120539367-120546601	7	RNF10
chr17:30984616-30988106	30	RNF135
chr6:45470323-45492114	0	RUNX2
chr6:130184102-130216616	0	SAMD3
chr1:243293090-243426558	13	SDCCAG8
chr13:29522316-29553860	57	SLC7A1
chr4:143526280-143528714	0	SMARCA5
chr17:31940285-31988497	13	SUZ12

chr10:58388189-58390860	24	TFAM
chr7:98933240-98937820	0	TRRAP
chr16:2065518-2080377	18	TSC2
chr1:154260891-154266568	10	UBAP2L
chr3:196362293-196372042	2	UBXN7
chr19:36712385-36712867	64	ZNF567
chr12:104861290-104928694	8	SLC41A2
chr9:19356971-19358160	32	DENND4C
chr8:143638645-143638987	13	nogene
chr20:33550640-33556738	8	nogene
chr13:32648753-32678929	26	PDS5B
chr2:42261120-42264731	14	EML4
chr20:39001729-39006356	34	DHX35
chr2:11782200-11783922	5	LPIN1
chr2:20254862-20256170	59	PUM2
chr8:53743576-53769743	5	ATP6V1H
chr2:75518055-75522487	200	EVA1A
chr15:63622814-63624327	6	HERC1
chr19:34215123-34215661	33	LSM14A
chr12:57069233-57072912	32	NEMP1
chr12:116712234-116714201	8	nogene
chr6:136064380-136111101	0	nogene
chr14:23301878-23302159	23	PPP1R3E
chr11:68601862-68603492	119	PPP6R3
chr9:131455075-131459356	98	PRRC2B
chr18:12801969-12859254	4	PTPN2
chr1:243304712-243316893	53	SDCCAG8
chr7:84110469-84194668	0	SEMA3A
chr10:27122840-27126786	23	YME1L1
chr6:87601783-87622013	2	ORC3
chr14:91481575-91490802	62	PPP4R3A
chr11:9286454-9295387	3	TMEM41B
chr20:13569506-13587370	104	TASP1
chr2:113927344-113942359	4	ACTR3
chr2:37282541-37293271	17	PRKD3
chr14:24210347-24210768	52	CHMP4A
chr15:90713166-90715042	121	CRTC3-AS1
chr9:136862905-136863871	2	EDF1
chr18:45928868-45934966	2	EPG5
chr12:109959846-109961685	3	GIT2
chr5:57246299-57249576	26	GPBP1
chr15:67646230-67703408	64	MAP2K5
chr19:311844-313643	56	MIER2

chr5:14270824-14287063	113	TRIO
chr3:183070998-183071357	143	MCCC1
chr10:68072882-68073686	17	HERC4
chr2:172568740-172593048	14	PDK1
chr8:81815324-81829516	7	SNX16
chr1:5873251-5880239	8	NPHP4
chr10:3112221-3116846	20	PFKP
chr8:99431536-99467634	4	VPS13B
chr9:37495168-37498224	8	POLR1E
chr3:47736033-47745993	68	SMARCC1
chr2:173954872-173956232	139	SP3
chr2:53919150-53927483	4	PSME4
chr11:9961956-10002689	14	SBF2
chr1:75749418-75750546	33	ACADM
chr11:118583178-118590506	1	ARCN1
chr6:106248149-106308491	40	ATG5
chr16:87975047-87981127	74	BANP
chr7:45063917-45064646	5	CCM2
chr9:95901992-95916434	0	ERCC6L2
chr10:63176296-63177856	4	JMJD1C
chr13:32648753-32659280	0	PDS5B
chr3:141509567-141516431	1	RASA2
chr15:57231151-57234107	106	TCF12
chr22:26540788-26544675	1	TPST2
chr8:15659506-15673836	3	TUSC3
chr4:20467751-20491899	31	SLIT2
chr18:31645354-31666372	2	B4GALT6
chr2:36396613-36517542	19	CRIM1
chr1:44874677-44880008	19	EIF2B3
chr6:31271598-31356442	169	HLA-C
chr20:16378804-16381747	19	KIF16B
chr1:172797452-172851482	5	nogene
chr11:120406117-120437382	15	ARHGEF12
chr12:122777490-122777683	16	CCDC62
chr10:68142939-68166666	13	MYPN
chr17:16143605-16153395	4	NCOR1
chrX:71554512-71555385	1	OGT
chr5:88220562-88245792	210	TMEM161B
chr10:68740628-68742569	7	CCAR1
chr1:28459084-28466768	41	PHACTR4
chr2:46001403-46010517	20	PRKCE
chr17:59764970-59773944	17	VMP1
chr9:94923985-94956015	38	C9orf3

chr8:60794985-60801593	193	CHD7
chr2:73946718-73957240	0	DGUOK
chr11:86250295-86256512	98	EED
chr5:93776158-93824267	0	FAM172A
chr16:58709416-58718300	0	GOT2
chr1:23814189-23820593	91	HMGCL
chr3:131162407-131170887	12	NEK11
chr4:47875126-47894302	16	NFXL1
chr17:40394625-40395031	26	nogene
chr19:16080914-16101409	80	nogene
chrX:24745417-24752353	25	POLA1
chr9:113286231-113288264	20	PRPF4
chr20:2975214-2988474	0	PTPRA
chr18:7755322-7774271	3	PTPRM
chr1:51269955-51271473	14	RNF11
chr16:58677325-58678894	2	SLC38A7
chr12:49494740-49500205	39	SPATS2
chr10:122132608-122143706	2	TACC2
chr13:75356146-75362607	5	TBC1D4
chr9:69225303-69226175	1	TJP2
chr12:26996741-26999676	45	TM7SF3
chr16:74942737-74956610	0	WDR59
chr19:33174138-33174979	36	nogene
chr6:18249650-18258405	21	DEK
chr3:150562541-150568292	14	EIF2A
chr6:130926604-130956499	17	EPB41L2
chr21:39180284-39181878	1	PSMG1
chr20:13528432-13580981	13	TASP1
chr11:6400988-6403822	6	APBB1
chr1:20980333-21002808	30	EIF4G3
chr22:43989110-43990406	17	SAMM50
chr7:127807483-127929329	15	SND1
chr12:62470700-62501698	24	MON2
chr10:74394140-74398579	170	ADK
chr11:118768222-118786518	21	DDX6
chr3:33014055-33046232	8	GLB1
chr5:109817272-109823837	188	MAN2A1
chr17:31252937-31265339	65	NF1
chr8:125151170-125195272	3	NSMCE2
chr10:7188623-7285954	1	SFMBT2
chr2:195680031-195683906	1	SLC39A10
chr20:13551886-13587370	13	TASP1
chr20:58434601-58441083	25	VAPB

chr10:75219062-75220970	33	VDAC2
chr6:73223698-73225421	7	nogene
chr12:121942533-121949061	58	WDR66
chr3:101671129-101672213	129	ZBTB11
chr16:58574608-58581515	1	CNOT1
chr11:9420410-9420698	24	IPO7
chr8:66592439-66602523	65	MYBL1
chr15:83151214-83164075	151	HDGFRP3
chrX:21978878-21992712	1	SMS
chr11:12675408-12764434	21	TEAD1
chr10:836402-864270	26	LARP4B
chr10:72562894-72566794	93	MICU1
chr11:61437624-61438113	1368	SDHAF2
chr16:71789278-71789482	1	AP1G1
chrX:1282677-1290509	6	CSF2RA
chr6:104833041-104849246	136	HACE1
chr19:5039835-5071059	7	KDM4B
chr5:37349171-37358151	23	NUP155
chr16:14586317-14604226	38	PARN
chr7:29966326-29969068	2	SCRN1
chr17:37553114-37554059	25	SYNRG
chr2:229836847-229880128	12	TRIP12
chr17:60265411-60288682	1	USP32
chr16:69840125-69871931	7	WWP2
chr19:11722953-11725327	27	ZNF823
chr7:106605178-106615771	21	nogene
chr22:28724976-28725367	93	CHEK2
chr21:21280577-21292241	11	NCAM2
chr2:213296039-213317356	1	SPAG16
chr8:123084161-123097441	17	TBC1D31
chr1:30964673-30974802	12	PUM1
chr21:15762890-15805258	26	USP25
chr12:99806403-99825389	53	ANKS1B
chr10:73725960-73728587	38	BMS1P4
chr16:70466131-70471352	5	FUK
chr1:28683582-28691709	8	GMEB1
chr13:113344862-113355481	15	GRTP1
chr12:112075677-112078741	212	NAA25
chr12:73430428-73477374	2	nogene
chr12:67349209-67349398	14	nogene
chr6:57318436-57326045	17	PRIM2
chr7:105465312-105470848	12	PUS7
chr7:105508114-105508544	26	PUS7

chr13:26337566-26385342	17	CDK8
chr16:53235184-53238586	24	CHD9
chr3:119863418-120093593	8	GSK3B
chr3:104817472-104831805	1	nogene
chr8:70583163-70594590	1	TRAM1
chr1:21924381-21924983	41	nogene
chr2:130358866-130359646	7	PTPN18
chr7:105472131-105506273	15	PUS7
chr4:87441828-87451735	18	NUDT9
chr12:30673989-30674753	15	IPO8
chrX:148651998-148662768	120	AFF2
chr18:12358669-12371691	41	AFG3L2
chr10:84371013-84373815	48	CCSER2
chr3:32437954-32442013	43	CMTM7
chr15:64204440-64204949	13	CSNK1G1
chr10:68936935-68948168	3	DDX50
chr17:47402131-47414919	157	EFCAB13
chr5:163473178-163478800	1	HMMR
chr6:89738325-89743714	25	MDN1
chr9:122270863-122291840	36	MRRF
chr1:2171250-2171460	1	nogene
chr16:22429803-22430774	0	nogene
chr5:41840450-41853554	20	OXCT1
chr18:7774148-7839401	0	PTPRM
chr15:65877331-65877955	14	RAB11A
chr3:47427156-47435137	23	SCAP
chr1:1223243-1228946	25	SDF4
chrX:119629317-119653040	10	6-Sep
chr1:185180390-185231708	0	SWT1
chr22:39426702-39428183	0	TAB1
chr8:142300485-142331831	0	TSNARE1
chr3:138209816-138223733	96	ARMC8
chr1:24814093-24827116	58	CLIC4
chr3:121872536-121874121	18	EAF2
chr3:81577924-81594023	37	GBE1
chr10:69382486-69386418	1	HK1
chr8:97815327-97825153	117	LAPTM4B
chr21:32270406-32284155	2	MIS18A
chr4:184659339-184661903	8	PRIMPOL
chr5:159194715-159203656	433	RNF145
chr12:76845708-76849470	10	ZDHHC17
chr7:73182076-73183298	13	GTF2IP1
chr9:124301935-124327445	81	NEK6

chr4:20280268-20280877	28	nogene
chr14:50153069-50158646	2	SOS2
chr6:42615070-42617507	54	UBR2
chr3:172760155-172784803	1	ECT2
chr11:64802683-64802884	47	MAP4K2
chr15:96282831-96290783	39	NR2F2-AS1
chr3:196241947-196248323	14	PCYT1A
chr17:44875933-44880644	13	EFTUD2
chr11:94821529-94831541	119	AMOTL1
chr1:243136142-243156455	15	CEP170
chr14:96833466-96856586	78	VRK1
chr20:38972558-38988917	2	DHX35
chr8:26581968-26583983	12	DPYSL2
chr11:118481714-118482495	12	KMT2A
chr11:29059388-29063536	1	nogene
chr12:111661979-111662290	27	nogene
chr7:67009582-67025022	3	TYW1
chr17:5448808-5450934	1	DHX33
chr12:1027747-1141787	1	ERC1
chr7:74689123-74691111	1	GTF2I
chr12:2822793-2836956	16	ITFG2
chr4:139703454-139704196	23	MGST2
chr2:203068392-203084569	5	NBEAL1
chr4:183153690-183154609	24	nogene
chr5:132217196-132218644	5	P4HA2
chr11:32589546-32596047	243	EIF3M
chr10:3158039-3160330	30	PITRM1
chr18:47841517-47896809	2	SMAD2
chr2:24135118-24147086	75	FAM228B
chr1:63477414-63490218	16	ITGB3BP
chr14:89980539-89985210	27	TDP1
chr16:21315883-21385932	0	CRYM-AS1
chr16:47641034-47669417	7	PHKB
chr7:67067284-67083539	74	TYW1
chr20:43702489-43705358	227	MYBL2
chr4:2625261-2626577	63	FAM193A
chr15:43334944-43335826	31	ADAL
chr3:155893517-155906263	61	GMPS
chrX:136820680-136848582	11	nogene
chr5:139614585-139614966	99	UBE2D2
chr11:768729-769573	115	nogene
chr17:42498923-42501304	142	ATP6V0A1
chr5:15615982-15634464	33	FBXL7

chr2:190900563-190924593	261	GLS
chr7:35667433-35673278	204	HERPUD2
chr4:128831732-128857454	2	JADE1
chr7:131387119-131399433	405	MKLN1
chr7:4797055-4797677	19	nogene
chr1:211260107-211289396	4	RCOR3
chr5:179567460-179569425	21	RUFY1
chr16:70565064-70569141	14	SF3B3
chr14:63864299-63909227	16	SYNE2
chrX:154769166-154770879	10	DKC1
chr12:87527757-87548850	3	nogene
chr15:67235978-67236820	60	AAGAB
chr1:4711899-4712699	47	AJAP1
chr10:32565557-32584304	9	CCDC7
chr5:69171269-69174409	23	CCNB1
chr4:168220654-168237795	8	DDX60
chr7:100812746-100813208	950	EPHB4
chr3:183276871-183295477	23	MCF2L2
chr10:103326533-103348999	16	PCGF6
chrX:150727214-150750843	19	MTMR1
chr12:121548876-121549638	3	KDM2B
chr22:24347085-24369320	4	SPECC1L-ADORA2A
chr6:89377670-89387590	17	RRAGD
chr4:80420213-80583183	0	C4orf22
chr5:127956803-127966928	11	nogene
chr7:42917132-42917835	2	PSMA2
chr7:131437784-131445903	23	MKLN1
chr15:74489588-74491582	17	nogene
chr5:180567087-180568003	32	CNOT6
chr13:107664846-107693167	17	nogene
chr7:131429032-131445903	72	MKLN1
chr10:102488149-102490613	1	ACTR1A
chr6:129599215-129618852	2	ARHGAP18
chr1:180964515-181034074	0	MR1
chr7:96170044-96193183	26	SLC25A13
chr1:186406082-186425297	3	C1orf27
chr15:44337762-44380976	18	CASC4
chr16:53605480-53611051	18	RPGRIP1L
chr19:38700599-38701121	23	ACTN4
chr1:180976117-180993425	3	STX6
chr19:3758524-3758977	20	nogene
chr17:19924407-19931978	25	AKAP10
chr2:157760879-157766196	0	ACVR1

chr10:4830674-4835809	33	AKR1E2
chr9:98745558-98771046	15	ANKS6
chr3:57242855-57248351	54	APPL1
chr1:155395458-155439068	12	ASH1L
chr1:1482137-1518990	824	ATAD3B
chr17:8206490-8207901	16	AURKB
chr6:26385014-26388294	10	BTN2A2
chr14:22987519-22999156	0	C14orf93
chr5:134321790-134350427	0	CDKL3
chr11:77622499-77625818	106	CLNS1A
chr15:90619740-90629532	3	CRTC3
chr11:61325610-61330074	41	DDB1
chr10:99894945-99896397	2	DNMBP
chr19:32973183-32973562	14	FAAP24
chr1:59346246-59378837	24	FGGY
chr1:28690103-28704329	3	GMEB1
chr16:50078740-50086351	17	HEATR3
chr11:33286412-33287511	4939	HIPK3
chr1:202755270-202758510	0	KDM5B
chr17:6589778-6635171	21	KIAA0753
chr9:5986135-5988545	2	KIAA2026
chr14:55612018-55619312	121	KTN1
chr1:200368664-200374254	0	LINC00862
chr6:118826146-118829250	15	MCM9
chr17:2464207-2473664	37	METTL16
chr22:26717252-26738240	2	MIATNB
chr4:153394261-153397333	302	MND1
chr2:42718987-42723035	46	MTA3
chr9:98076917-98078347	72	NANS
chr14:92104372-92104729	16	nogene
chr7:55555946-55556534	19	nogene
chr1:152248894-152253510	25	nogene
chr5:150364956-150365889	24	nogene
chr15:96334075-96337535	149	NR2F2
chr11:3731390-3753408	190	NUP98
chr7:44645326-44647759	348	OGDH
chr11:73909398-73916660	0	PAAF1
chr16:2577424-2583585	6	PDPK1
chr16:47580294-47610920	0	PHKB
chr3:171677565-171692442	0	PLD1
chr3:12312379-12392752	20	PPARG
chr2:20253821-20256170	21	PUM2
chr2:226838072-226867318	0	RHBDD1

chr1:213129526-213176492	25	RPS6KC1
chrX:19541924-19546050	20	SH3KBP1
chr3:47706408-47729094	100	SMARCC1
chr4:122935713-122979386	70	SPATA5
chr10:11931640-11936712	0	UPF2
chr17:653290-661895	28	VPS53
chr2:63484603-63571626	34	WDPCP
chr19:9341101-9343784	13	ZNF559
chr17:81270917-81277133	79	SLC38A10
chrX:72490928-72495268	35	HDAC8
chr9:131430089-131439061	17	PRRC2B
chr8:140370963-140405698	31	TRAPPC9
chr19:33798670-33811552	7	KCTD15
chr3:104522668-104556044	27	nogene
chr1:31028795-31059577	31	PUM1
chr6:135442581-135457713	8	AHI1
chrX:80717572-80730020	6	BRWD3
chr19:11111513-11116212	21	LDLR
chr17:31169890-31182665	23	NF1
chr2:95834358-95834565	24	nogene
chr1:44866320-44866664	20	nogene
chr14:73147794-73217244	17	PSEN1
chr4:86764592-86767976	100	PTPN13
chr4:53145332-53145582	50	SCFD2
chrX:53405011-53405391	31	SMC1A
chr17:64561499-64571956	103	SMURF2
chr14:20395449-20395949	39	TEP1
chr7:73459535-73466401	0	BAZ1B
chr21:37375050-37420384	0	DYRK1A
chr12:110485077-110486717	23	FAM216A
chr2:232735154-232749082	10	GIGYF2
chr4:87159457-87195690	0	KLHL8
chr1:93110229-93110626	13	MTF2
chr13:24477700-24490828	0	PARP4
chr10:91819269-91836998	1	TNKS2
chr4:1892165-1935262	30	WHSC1
chr2:214745066-214781509	36	BARD1
chr17:49716164-49732720	0	FAM117A
chr17:30481142-30484770	354	GOSR1
chr3:119863418-119876508	28	GSK3B
chr3:28575069-28576496	0	LINC00693
chr12:56712785-56714439	1	NACA
chr10:121959323-121963328	14	NSMCE4A

chr6:136825213-136846181	1	PEX7
chr14:73147794-73165413	0	PSEN1
chr1:176027571-176085890	2	RFWD2
chr2:218748835-218751806	15	TTLL4
chr17:51263273-51280479	5	UTP18
chr6:87210450-87233527	0	ZNF292
chr13:27253843-27254219	110	RPL21
chr1:203816925-203818689	63	ZC3H11A
chr3:183977516-183978651	21	ABCC5
chr20:49090741-49094963	56	CSE1L
chr2:15553425-15558634	63	NBAS
chr1:161102047-161102375	139	PFDN2
chr8:73805650-73830472	223	UBE2W
chr11:46769962-46788773	4	CKAP5
chr12:124956690-124957135	38	DHX37
chr2:241403827-241436538	7	FARP2
chr2:121730892-121732204	2	NIFK
chr1:77154448-77212347	13	PIGK
chr8:140789473-140846678	41	PTK2
chr17:42329409-42329776	13	STAT3
chr6:111570084-111576470	13	TRAF3IP2-AS1
chr15:50706387-50707157	98	nogene
chr17:37440504-37442652	36	TADA2A
chr20:49072285-49091022	0	CSE1L
chr7:157339837-157367483	0	DNAJB6
chr22:42619702-42628183	4	nogene
chr9:125213369-125220700	81	RABEPK
chr16:2557702-2577500	0	PDPK1
chr16:11020192-11126146	135	CLEC16A
chr9:123169901-123184298	82	STRBP
chr9:95897848-95921315	5	ERCC6L2
chr4:109491326-109500054	1	SEC24B
chr2:72988257-73001578	29	SFXN5
chr15:74917773-74929232	1	COX5A
chr22:38620211-38623279	26	FAM227A
chr18:36658074-36693423	83	FHOD3
chr3:52737585-52739634	68	NEK4
chr12:50312228-50312840	0	nogene
chr12:68690630-68700853	33	NUP107
chr15:44626330-44629388	31	SPG11
chr15:43455891-43470066	50	TP53BP1
chr9:19063007-19076704	14	HAUS6
chr10:3132379-3135838	20	PFKP

chr8:67093542-67095732	130	CSPP1
chr7:155763808-155766655	32	RBM33
chr4:42001615-42049479	11	SLC30A9
chr2:187496880-187503770	5	TFPI
chr14:95140658-95141748	38	DICER1
chr13:114040999-114073837	170	RASA3
chr16:15882948-15884205	1	FOPNL
chr8:43069554-43072307	86	FNTA
chr19:37425867-37444964	49	ZNF569
chr2:158533179-158634289	4	PKP4
chr1:16484822-16631109	95	CROCCP3
chr2:171046173-171050174	57	TLK1
chr11:66825398-66825936	2	nogene
chr19:32617588-32622619	43	ANKRD27
chr4:165085585-165103096	13	TMEM192
chr17:60679452-60709325	115	BCAS3
chr17:81667728-81672155	4	CCDC137
chr9:96562692-96565512	0	CDC14B
chr1:179043714-179054634	0	FAM20B
chr15:49235850-49292537	180	GALK2
chr12:46571255-46577284	0	LOC100288798
chr20:46068501-46079485	0	NCOA5
chr6:138473305-138496371	178	NHSL1
chr5:179535164-179540832	0	nogene
chr5:178143626-178144108	0	RMND5B
chr6:107881148-107883146	58	SEC63
chr4:188104836-188105587	31	TRIML2
chr4:39874288-39877153	20	PDS5A
chr22:42890946-42891182	81	PACSIN2
chr3:138755753-138759359	7	PIK3CB
chr8:47900372-47930787	24	PRKDC
chr11:62569396-62570171	19	nogene
chr6:7895105-7904723	3	TXNDC5
chr4:37631384-37635123	58	RELL1
chr17:80236927-80237594	6	SLC26A11
chr21:15864267-15874526	26	USP25
chr18:62539680-62544873	1	ZCCHC2
chr1:243613670-243843282	46	AKT3
chr15:50489800-50490525	1	USP8
chr4:150928515-150929065	16	LRBA
chr16:47128324-47134410	19	NETO2
chr3:61622462-61623555	56	nogene
chr5:66153462-66159334	28	SREK1

chr3:75741275-75741736	38	ZNF717
chr19:36917578-36937242	29	ZNF568
chr15:76274411-76286481	37	ETFA
chr4:103048892-103057971	4	SLC9B2
chr10:31373017-31387798	152	nogene
chr22:29042494-29046883	1	ZNRF3
chr12:1777853-1784073	78	ADIPOR2
chr20:33379460-33382179	11	CDK5RAP1
chr5:14304460-14336727	312	TRIO
chrX:10924729-10925298	17	nogene
chr16:2365680-2379581	0	ABCA17P
chr8:39054480-39082721	0	ADAM9
chr22:40360401-40363071	0	ADSL
chr10:84438281-84477664	18	CCSER2
chr11:68807466-68812576	32	CPT1A
chr12:93849969-93859442	0	CRADD
chr9:95897848-95928864	72	ERCC6L2
chrX:154361978-154362332	21	FLNA
chr10:1000676-1007128	20	GTPBP4
chr9:96298415-96302311	0	HSD17B3
chr15:60442415-60445791	2	ICE2
chr19:45264683-45266281	5	MARK4
chr10:72551178-72566794	47	MICU1
chr19:17145396-17154066	0	MYO9B
chr2:191678989-191685855	0	NABP1
chr16:69626402-69655799	4	NFAT5
chr5:149739473-149740125	0	nogene
chr8:103246293-103246684	9	nogene
chr1:224275338-224289733	0	NVL
chr5:41762110-41807438	101	OXCT1
chr3:196782625-196803164	15	PAK2
chr3:111913318-111932397	2	PHLDB2
chr12:106842588-106851594	28	RIC8B
chr6:154808069-154810208	19	SCAF8
chr5:78415519-78418903	0	SCAMP1
chr2:213310058-213317356	26	SPAG16
chr1:47287550-47304996	3	STIL
chr17:63028003-63200957	0	TANC2
chr1:235419472-235434280	32	TBCE
chr17:40395448-40399131	31	TOP2A
chr6:123331878-123366182	0	TRDN
chr5:14297071-14316743	25	TRIO
chr15:50631416-50663046	54	TRPM7

chr7:98929988-98930206	0	TRRAP
chr15:77052384-77056255	13	TSPAN3
chr4:102801453-102826636	0	UBE2D3
chr2:85619219-85625691	48	USP39
chr6:148964409-149074112	0	UST
chr3:142397328-142405076	16	XRN1
chr1:32627507-32634072	6	ZBTB8OS
chrX:24179182-24179770	31	ZFX
chr1:245858495-245929937	52	SMYD3
chr11:86487336-86508867	13	ME3
chr17:2172657-2188515	6	SMG6
chr11:35176574-35190065	11	CD44
chr9:96360153-96368305	4	SLC35D2
chr1:72749165-72874338	4	nogene
chr12:104120399-104123423	89	NFYB
chr8:47927193-47930787	64	PRKDC
chr7:105202431-105203785	1	SRPK2
chr22:29520007-29525946	1	THOC5
chrX:110020351-110144876	14	TMEM164
chr12:132668810-132672322	55	POLE
chr1:70270855-70276844	3	ANKRD13C
chr15:29053844-29054835	86	APBA2
chr2:214745066-214769312	80	BARD1
chr9:21324075-21329419	16	nogene
chr19:32371381-32371734	23	nogene
chrX:134413490-134417302	19	PHF6
chr17:1842802-1846641	2	RPA1
chr22:40429605-40435554	12	MKL1
chr15:65481514-65490299	1	DPP8
chr15:63696123-63716473	119	HERC1
chr10:75547030-75547662	27	nogene
chr7:127373341-127377329	39	ZNF800
chrX:131332206-131369052	33	nogene
chr8:48043032-48049978	83	UBE2V2
chr7:129203383-129206587	36	SMO
chr1:155415743-155415923	24	ASH1L
chr1:94458606-94475737	14	ABCD3
chr20:49092045-49094963	136	CSE1L
chr10:126970701-126998249	1	DOCK1
chr5:108020924-108348530	58	FBXL17
chr17:39297126-39343241	19	FBXL20
chr4:106193608-106212835	61	TBCK
chr9:83665029-83678599	7	UBQLN1

chr19:17156908-17163122	35	MYO9B
chr3:155893517-155903960	0	GMPS
chr6:83346172-83352139	6	ME1
chr7:30455136-30457042	3	NOD1
chr4:82854902-82861708	13	SEC31A
chr17:44435064-44464544	7	GPATCH8
chr21:42686191-42733426	15	PDE9A
chr3:172226870-172247776	6	FNDC3B
chr16:70128783-70129122	2	PDPR
chr1:246591511-246591941	50	CNST
chr15:28245880-28248736	59	HERC2
chr6:37650108-37652340	0	MDGA1
chr4:40978403-40986415	7	nogene
chr4:39837855-39842056	29	PDS5A
chr4:6372522-6389952	0	PPP2R2C
chr20:56473300-56484189	23	RTFDC1
chr15:45486610-45490881	1	SLC30A4
chr20:10556230-10560820	141	SLX4IP
chr1:151638887-151693483	26	SNX27
chr17:51005211-51007326	0	SPAG9
chr17:82870223-82903478	27	TBCD
chr20:43973366-44006792	31	TOX2
chr8:42398711-42399703	0	VDAC3
chr5:154790324-154803745	8	LARP1
chr1:50787999-50801677	137	FAF1
chr3:40461465-40462104	104	nogene
chr6:84305115-84313884	1	nogene
chrX:155468285-155509901	0	nogene
chr8:43122106-43122898	0	POMK
chr7:22976209-22991139	854	FAM126A
chr16:11730644-11736233	39	TXNDC11
chr1:16137852-16138430	98	EPHA2
chr2:61498672-61499894	41	XPO1
chr6:118479870-118483858	28	CEP85L
chr1:174219124-174221164	25	RABGAP1L
chr5:150396281-150396842	3	TCOF1
chr1:24169416-24180854	21	IFNLR1
chr10:125038996-125111093	265	CTBP2
chr1:35460304-35466695	6	KIAA0319L
chr4:25345547-25351689	20	ZCCHC4
chr20:47246007-47249439	13	ZMYND8
chr10:1093451-1105267	64	WDR37
chr16:71667177-71681905	26	PHLPP2

chr16:71676446-71690709	17	PHLPP2
chr17:15677175-15677721	63	TRIM16
chr2:178591117-178600667	17	TTN-AS1
chr13:41811224-41819386	153	VWA8
chr3:23900807-23901049	14	NKIRAS1
chr10:109891721-109915099	1	XPNPEP1
chr2:61339350-61350693	1	USP34
chr11:130120705-130122513	29	APLP2
chr1:1637407-1704344	137	CDK11B
chr7:39999360-40063100	12	CDK13
chr5:138781922-138904441	2	CTNNA1
chr6:117525361-117537225	12	DCBLD1
chr15:49625112-49634794	18	DTWD1
chr22:45330602-45340561	16	FAM118A
chr6:5431040-5467069	12	FARS2
chr5:171868609-171878129	0	FBXW11
chr15:65558679-65564342	12	HACD3
chrX:118741016-118761289	2	IL13RA1
chr14:103662115-103662927	101	KLC1
chr22:33761370-33771008	12	LARGE
chr3:197865422-197871462	11	LRCH3
chr17:45273502-45274593	27	MAP3K14
chr9:122280442-122322745	32	MRRF
chr12:65326825-65369026	48	MSRB3
chr5:6775558-6777509	6	nogene
chr19:57861351-57863260	12	nogene
chr5:178224641-178230099	12	PHYKPL
chr14:39180502-39183830	4	PNN
chr8:140846259-140921132	13	PTK2
chr6:130287819-130313093	3	SAMD3
chr13:98474820-98519473	40	STK24
chr18:31846715-31852663	43	TRAPPC8
chr6:25963048-25964954	12	TRIM38
chr19:34430575-34433412	20	UBA2
chr10:73575511-73576361	26	USP54
chr11:75851882-75912039	6	UVRAG
chr18:12999420-13008631	108	CEP192
chr15:22943172-22947291	12	CYFIP1
chr10:96949007-96952196	19	LCOR
chr15:75407054-75422823	23	SIN3A
chr12:6933839-6934578	1	ATN1
chr17:40260534-40262641	28	WIPF2
chr21:45116932-45128573	17	ADARB1

chr18:9195550-9216900	0	ANKRD12
chr4:40881283-40893411	7	APBB2
chr16:67452237-67453715	0	ATP6V0D1
chr7:90917600-90984241	0	CDK14
chr17:63578469-63585328	4	DCAF7
chr1:153937487-153937863	46	DENND4B
chr12:31413435-31433248	5	DENND5B
chr1:68494429-68494695	7	DEPDC1
chr2:85356966-85357252	2	ELMOD3
chr10:119107967-119132609	0	FAM45A
chr14:45118027-45130790	109	FKBP3
chr1:240123178-240158307	0	FMN2
chr7:42076751-42223295	1	GLI3
chr4:157136468-157153010	48	GLRB
chr17:30481142-30510909	92	GOSR1
chr2:144208602-144211579	97	GTDC1
chr4:142654586-142725901	1	INPP4B
chr1:156550260-156552095	0	IQGAP3
chr11:108479300-108491167	12	KDELC2
chr10:124488421-124498128	55	LHPP
chr8:89724628-89724966	0	LOC101929709
chr2:43912431-43934878	52	LRPPRC
chr2:39290291-39309519	0	MAP4K3
chr18:2552694-2555038	0	METTL4
chr14:37285317-37369624	188	MIPOL1
chr7:131387119-131388972	0	MKLN1
chr9:14146688-14155893	28	NFIB
chr3:106741560-106771161	17	nogene
chr1:235267826-235268638	0	nogene
chr11:65503116-65503931	2	nogene
chr17:81606690-81610258	0	NPLOC4
chr1:151227319-151232703	20	PIP5K1A
chr10:78002196-78007866	57	POLR3A
chr14:50915335-50917105	5	PYGL
chr1:178123047-178300118	4	RASAL2
chr3:49967469-49975392	226	RBM6
chr1:202001375-202003461	29	RNPEP
chr1:243270091-243308177	14	SDCCAG8
chr4:109527321-109531522	0	SEC24B
chr17:64593439-64606640	2	SMURF2
chr9:4622407-4635399	1	SPATA6L
chr3:136602078-136604473	0	STAG1
chr9:123158331-123184298	19	STRBP

chr4:106260436-106308989	17	TBCK
chr10:68651845-68652594	0	TET1
chr8:140275657-140360193	1	TRAPPC9
chr1:1255233-1267992	0	UBE2J2
chr14:88563650-88575940	0	ZC3H14
chr19:57722315-57723340	0	ZNF671
chr10:124971964-124974398	0	ZRANB1
chr8:143689203-143690123	14	ZNF707
chr2:202530793-202552888	12	BMPR2
chr12:122572936-122574380	1	KNTC1
chr17:76564508-76564833	14	nogene
chr2:10373605-10374580	1	nogene
chr3:125288102-125331238	74	ZNF148
chr10:112890965-112906213	2	nogene
chr4:88043232-88043457	13	PKD2
chr18:54271359-54284065	3	POLI
chr8:127939507-128010444	35	PVT1
chr5:61490785-61494410	5	ZSWIM6
chr7:73183114-73184719	7	GTF2IP1
chr5:140482579-140487060	13	ANKHD1-EIF4EBP3
chr9:36198978-36204179	2	CLTA
chr11:70405264-70407591	132	CTTN
chr15:52593577-52601165	2	FAM214A
chr10:7258090-7285954	19	SFMBT2
chr9:38544753-38545193	53	nogene
chr6:157036834-157133207	1	ARID1B
chr17:15545784-15555364	92	TVP23C-CDRT4
chr11:61326778-61330074	13	DDB1
chr14:61720381-61721823	68	HIF1A
chr10:28535561-28590832	19	WAC
chr3:72792915-72817384	14	SHQ1
chr15:76857808-76883876	136	SCAPER
chr15:51862810-51869373	16	TMOD3
chr13:24490667-24501834	12	PARP4
chr1:220006105-220007338	25	EPRS
chr17:12095574-12113360	116	MAP2K4
chr7:6735304-6741852	19	PMS2CL
chrX:131749305-131768696	28	FIRRE
chr3:136422409-136423044	1	STAG1
chr1:112996840-112999109	28	nogene
chr5:65170616-65197151	330	ADAMTS6
chr8:99878139-99887618	20	COX6C
chr6:150877633-150887981	40	MTHFD1L

chr7:66742130-66743522	40	nogene
chr3:51541588-51590559	20	RAD54L2
chr4:37902002-37929982	27	TBC1D1
chr2:65239851-65251099	0	ACTR2
chr10:100990435-100991010	13	C10orf2
chr11:34079627-34086413	10	CAPRIN1
chr1:92725597-92736627	100	EVI5
chr18:57554259-57554952	21	FECH
chr6:35619095-35684485	1	FKBP5
chr9:96949554-96962963	9	HIATL2
chr12:50427764-50430570	32	LARP4
chr8:120488158-120502609	0	MTBP
chr12:77994802-78007445	5	NAV3
chr3:104630288-104630579	9	nogene
chr4:39848850-39849652	3	PDS5A
chr8:47783741-47798397	23	PRKDC
chr9:107300140-107318879	62	RAD23B
chrX:20188496-20204103	0	RPS6KA3
chr16:68266592-68275249	476	SLC7A6
chr4:98105952-98134459	62	STPG2
chr13:60439687-60460540	97	TDRD3
chr11:75961443-76016980	2	UVRAG
chr3:184982565-184999861	30	VPS8
chr7:158911549-158918869	55	WDR60
chr7:30362376-30366295	0	ZNRF2
chr1:77631834-77641655	31	ZZZ3
chr3:57633264-57634769	0	DENND6A
chr9:96551795-96565483	128	CDC14B
chr6:30670409-30671274	19	DHX16
chr7:32620403-32679130	25	DPY19L1P1
chr1:235791698-235793612	19	LYST
chr8:6477593-6499929	16	MCPH1
chr7:22291174-22340886	12	RAPGEF5
chr18:51054780-51067187	56	SMAD4
chrX:130236213-130247005	32	ZNF280C
chr1:112591524-112600848	2	ST7L
chr8:25243673-25278665	9	DOCK5
chr3:43574808-43577261	13	ANO10
chr13:28197184-28261458	1	PAN3
chr7:17814833-17830047	3	SNX13
chr17:16001914-16006568	98	TTC19
chr19:34678736-34683238	27	ZNF302
chr4:105233896-105237351	4	TET2

chr5:74813839-74841679	598	FAM169A
chr5:65451474-65452918	129	ADAMTS6
chr9:85642826-85712566	30	AGTPBP1
chr4:47535901-47546862	0	ATP10D
chr5:135074416-135174172	15	C5orf66
chr6:30649557-30651466	10	C6orf136
chr18:68836748-68846421	4	CCDC102B
chr1:156317165-156334918	1	CCT3
chr3:113409105-113416627	0	CFAP44
chr16:57119194-57123473	1	CPNE2
chr10:94938436-94942020	0	CYP2C9
chr9:123667025-123769563	8	DENND1A
chr19:10173089-10177367	23	DNMT1
chr7:32655986-32679130	19	DPY19L1P1
chr5:31464235-31526290	0	DROSHA
chr16:68038005-68056424	14	DUS2
chr6:170388286-170391121	2	FAM120B
chrX:154512313-154512936	23	FAM3A
chr7:102822039-102854860	11	FBXL13
chr10:121564501-121565704	13	FGFR2
chrX:154441622-154441794	0	GDI1
chr9:130104419-130110426	12	GPR107
chrX:307359-307880	2	GTPBP6
chr6:82181878-82204958	2	IBTK
chr4:142108092-142124760	27	INPP4B
chr17:27550567-27558187	15	KSR1
chr20:14002287-14009973	24	MACROD2
chr1:113646485-113659265	18	MAGI3
chr2:171337452-171339554	183	METTL8
chr9:126392065-126395697	37	MVB12B
chr12:78127166-78146392	0	NAV3
chr4:177353307-177353728	339	NEIL3
chr1:169298555-169324500	38	NME7
chr12:69459516-69462525	31	nogene
chr2:218249362-218253965	2	nogene
chr18:12375056-12375577	21	nogene
chr21:45755416-45760441	1	nogene
chr8:127969415-128010444	20	nogene
chr17:61353587-61354502	0	nogene
chr5:97429848-97439618	0	nogene
chr16:14608280-14617650	1	PARN
chr10:94258781-94259150	0	PLCE1
chr17:28350437-28351800	35	POLDIP2

chr3:121496807-121498670	172	POLQ
chr10:102137849-102138980	0	PPRC1
chr10:87931045-87933251	3	PTEN
chr19:40780018-40786978	1	RAB4B-EGLN2
chr9:127195090-127199066	17	RALGPS1
chr13:114037750-114073837	0	RASA3
chr20:35653797-35659014	29	RBM12
chr20:35721739-35725155	436	RBM39
chr15:59030803-59058550	25	RNF111
chr2:7014453-7030215	3	RNF144A
chr5:139026800-139127853	0	SIL1
chrX:119410189-119410362	3	SLC25A43
chr3:27437387-27448797	107	SLC4A7
chrX:21966982-21967316	0	SMS
chr12:108818248-108818558	5	SSH1
chr12:15894046-15903143	0	STRAP
chr13:37012191-37024442	0	SUPT20H
chr8:123105287-123109620	8	TBC1D31
chr4:165100627-165103096	11	TMEM192
chr2:3387619-3424663	3	TRAPPC12
chr10:73536268-73539593	24	USP54
chr1:45087880-45088237	42	ZSWIM5
chr7:107230614-107258383	9	COG5
chr4:44293403-44369094	18	nogene
chr14:54681934-54702580	69	SAMD4A
chr5:43381800-43382052	4	CCL28
chr10:127061667-127110354	39	DOCK1
chr10:70419913-70421966	33	EIF4EBP2
chr6:130885095-130908863	21	EPB41L2
chr2:24135118-24139450	90	FAM228B
chrX:152284667-152364596	4	GABRA3
chr5:62530708-62561257	15	IPO11
chr15:90481959-90482281	12	IQGAP1
chr3:124809021-124812920	12	ITGB5
chr6:17836877-17837583	3	KIF13A
chr19:18564868-18565190	64	KXD1
chr4:128074459-128098330	46	LARP1B
chr11:68386315-68390052	25	LRP5
chr2:15327749-15330765	40	NBAS
chr8:95041569-95048558	12	NDUFAF6
chr11:65502320-65503931	11	nogene
chr3:15303560-15304233	12	nogene
chr11:46428750-46429066	53	nogene

chr7:65313569-65317566	13	nogene
chr7:135577795-135593192	131	NUP205
chr7:5997325-6002636	16	PMS2
chrX:24809897-24843677	61	POLA1
chr8:140800458-140864399	55	PTK2
chr6:43026036-43029190	16	RRP36
chr20:18510821-18512282	46	SEC23B
chr15:49027364-49028682	8	SECISBP2L
chr4:146488065-146510049	19	SLC10A7
chrX:46620976-46653713	3	SLC9A7
chr18:26032398-26057742	16	SS18
chr8:98547968-98596169	3	STK3
chr2:36893897-36925208	12	STRN
chr1:235438768-235442911	18	TBCE
chr11:69078481-69079020	12	TPCN2
chr12:72562164-72575542	16	TRHDE
chr1:62447340-62448666	1	USP1
chr3:49305714-49311654	29	USP4
chr6:144451369-144459354	24	UTRN
chr19:45517662-45518094	94	VASP
chr1:35104294-35110447	39	ZMYM1
chr2:127869453-127871359	6	AMMECR1L
chr9:32984630-32989895	1	APTX
chr10:125827557-125831607	4	BCCIP
chr14:80862756-80907493	1	CEP128
chr14:53072596-53103856	3	DDHD1
chr1:28027788-28048426	1	EYA3
chr3:192727180-192728554	16	FGF12
chrX:131743095-131749458	42	FIRRE
chr4:165218024-165228913	5	KLHL2
chr9:36580072-36589652	16	MELK
chr14:37285317-37422949	27	MIPOL1
chr3:138707158-138712304	1	PIK3CB
chr2:55640626-55644720	57	PNPT1
chr7:128310779-128315020	17	RBM28
chr4:6923372-7014557	18	TBC1D14
chr20:50127974-50143621	1	TMEM189-UBE2V1
chr7:5320317-5325248	1	TNRC18
chr17:4296716-4303745	38	UBE2G1
chr12:42199135-42210619	15	YAF2
chr10:124962286-124966781	1	ZRANB1
chr7:92294888-92352642	73	ANKIB1
chr12:62349220-62355475	26	USP15

chr12:31697360-31709425	4	AMN1
chr3:141903619-141922063	5	ATP1B3
chr2:70175672-70181997	11	C2orf42
chr17:59660390-59661643	1	CLTC
chr11:108046269-108054955	24	CUL5
chr2:171925536-171979363	37	HAT1
chr15:72345445-72348134	0	HEXA
chr18:46946056-46946923	10	KATNAL2
chr21:28162191-28170143	0	LOC101927973
chr7:67356030-67362278	0	nogene
chr11:66216118-66217617	0	PACS1
chr18:54271359-54292038	13	POLI
chr7:860127-872562	0	SUN1
chr21:28577218-28626676	35	nogene
chr2:10122982-10127220	17	RRM2
chr7:32639273-32679130	13	DPY19L1P1
chr11:93117646-93166016	1	SLC36A4
chr2:36396613-36431221	18	CRIM1
chr3:52687501-52690704	21	GNL3
chr17:8253858-8254301	33	PFAS
chr19:47150201-47169923	4	SAE1
chr9:123757702-123879021	342	DENND1A
chr7:17845594-17868490	39	SNX13
chr2:61873001-61876234	19	CCT4
chr11:111754498-111755450	16	PPP2R1B
chr16:88004294-88006265	10	BANP
chr12:30661140-30663654	26	IPO8
chr3:15411244-15415942	36	METTL6
chr16:50216074-50217663	9	PAPD5
chr17:58694930-58709990	4	RAD51C
chr2:30942200-30945870	14	GALNT14
chr15:58912562-58916999	1863	SLTM
chr2:187484123-187503770	23	TFPI
chr13:95986332-95996135	21	UGGT2
chr15:61908991-61912004	17	VPS13C
chr16:31122021-31122604	43	nogene
chr1:26942659-26943065	554	NUDC
chr10:92326834-92340803	1	5-Mar
chr17:47668185-47670832	1	KPNB1
chr3:33836043-33838327	7	PDCD6IP
chr11:8965903-8968041	89	TMEM9B-AS1
chr1:37889556-37891454	1	INPP5B
chr16:21617864-21618074	12	METTL9

chr15:64203081-64300723	1	CSNK1G1
chr4:47855058-47875293	8	NFXL1
chr20:2986764-2988474	131	PTPRA
chr3:196072002-196077122	36	TFRC
chr5:33576053-33596060	6	ADAMTS12
chr1:156951572-156955799	14	ARHGEF11
chr14:80895717-80916562	13	CEP128
chr11:93729869-93730148	7	CEP295
chr10:127012294-127061776	16	DOCK1
chr10:80420465-80427496	1	FAM213A
chr19:4354948-4355173	34	MPND
chr3:27256295-27287743	1	NEK10
chr9:133976429-133978130	16	nogene
chr19:37729903-37730337	47	nogene
chr10:34450316-34517159	38	PARD3
chr11:85981128-85983973	24	PICALM
chr18:9577063-9595102	32	PPP4R1
chr5:168493893-168502105	46	RARS
chr18:36030807-36033837	1	RPRD1A
chr20:49864727-49884066	2	SLC9A8
chr7:127807483-127888012	47	SND1
chr4:133075343-133096883	2	LOC101927359
chr14:64442053-64448317	47	MTHFD1
chr3:52204692-52206751	52	ALAS1
chr1:236168705-236183906	166	GPR137B
chr2:233388256-233398449	57	DGKD
chr21:15011169-15014466	63	NRIP1
chr11:77675035-77693611	37	RSF1
chr14:66965190-67058786	73	GPHN
chr14:21211405-21213117	3	HNRNPC
chr2:55643157-55645432	16	PNPT1
chr17:61904459-61919513	13	INTS2
chr11:17336959-17337534	4	nogene
chr1:1403036-1403338	16	nogene
chr2:36396613-36479694	201	CRIM1
chr17:59570517-59573235	5	DHX40
chr6:45491940-45512407	51	RUNX2
chr6:12015525-12020418	16	HIVEP1
chr3:136452032-136502779	0	STAG1
chr17:1354210-1362008	162	YWHAE
chr17:17214984-17219209	21	FLCN
chr20:417525-417930	5	RBCK1
chr1:32037834-32039573	8	KHDRBS1

chr22:21799011-21807846	118	MAPK1
chr7:102397619-102407498	40	LOC100630923
chr17:58216087-58216736	25	MKS1
chr3:47037665-47062346	8	SETD2
chr3:129867009-129880559	31	TMCC1
chr2:37003541-37007304	23	HEATR5B
chr21:42850040-42876767	90	WDR4
chr7:11028680-11111467	1	PHF14
chr10:68394327-68401737	20	RUFY2
chr7:66944975-66945261	9	TMEM248
chr6:36072872-36102649	27	MAPK14
chr8:61618977-61638363	25	ASPH
chr5:148548667-148550262	325	HTR4
chr9:122280442-122313386	33	MRRF
chr19:726137-734194	25	PALM
chr4:88038250-88052158	16	PKD2
chr6:147353322-147364166	10	STXBP5
chr7:1936686-2002121	80	MAD1L1
chr6:35867733-35868082	19	nogene
chr20:21326489-21330539	54	XRN2
chr19:34194477-34209051	32	LSM14A
chr9:133019737-133020418	122	nogene
chr9:35663321-35664492	0	ARHGEF39
chr19:15271321-15273133	15	BRD4
chr11:46801199-46809874	21	CKAP5
chr2:36441257-36442735	948	CRIM1
chr9:125341176-125346941	77	GAPVD1
chr6:17825767-17837583	0	KIF13A
chr4:128178430-128179512	0	LARP1B
chr2:159743060-159752571	0	7-Mar
chr12:120356901-120359053	0	MSI1
chr5:41749526-41794078	0	OXCT1
chr11:74203727-74239256	2	PPME1
chr22:40792524-40799083	0	SLC25A17
chr13:95983803-95999307	115	UGGT2
chr2:216141185-216162048	49	XRCC5
chr11:120406117-120424415	13	ARHGEF12
chr1:225231072-225240822	1	DNAH14
chr12:131981488-131982478	16	EP400
chr1:89686469-89712935	25	LRRC8C
chr8:47803305-47819510	1	PRKDC
chr2:208271510-208277708	2	PIKFYVE
chr1:160895644-160895878	153	nogene

chr5:93958776-93964523	8	FAM172A
chr2:99429839-99435941	77	REV1
chr13:41865735-41869189	2	VWA8
chr3:170113359-170122744	76	PHC3
chr3:47686048-47693300	50	SMARCC1
chr20:25632157-25674007	3	ZNF337-AS1
chr4:55470716-55510065	42	CLOCK
chr6:53514203-53522527	2	GCLC
chr16:5079747-5081056	18	ALG1
chr10:97656284-97662968	16	PI4K2A
chr10:116906626-116921516	3	SHTN1
chr16:28146093-28156527	63	XPO6
chr20:49269941-49271750	46	ZNFX1
chr16:47497398-47503090	18	PHKB
chr8:97668194-97668649	35	nogene
chr11:103255414-103286386	14	DYNC2H1
chr19:10368054-10368418	9	TYK2
chr15:78513083-78515107	3	HYKK
chr15:63553714-63563008	184	USP3
chr6:18215006-18217885	77	KDM1B
chr3:64010877-64022523	13	PSMD6
chr22:24119366-24134415	2	CABIN1
chr3:3140509-3144710	43	TRNT1
chr2:214780559-214792445	12	BARD1
chr7:66795510-66799414	74	RABGEF1
chr4:38986335-38993998	53	TMEM156
chr10:89751345-89762835	47	KIF20B
chr12:71769799-71782658	13	RAB21
chr4:153270337-153276130	11	TRIM2
chr2:219792376-219940777	1	nogene
chr20:44305803-44305973	50	nogene
chr10:7370280-7381949	18	SFMBT2
chr2:111850775-111858401	23	ANAPC1
chr11:65897995-65898726	12	nogene
chr15:75876186-75883424	65	UBE2Q2
chr21:33718796-33722651	18	ITSN1
chr7:5528575-5529202	12	nogene
chr4:7006631-7014557	47	TBC1D14
chr22:44913821-44920490	18	PHF21B
chr20:38066080-38066256	1	RPRD1B
chr14:30670255-30700258	23	SCFD1
chr4:83588209-83594960	1	GPAT3
chr9:92640709-92652639	20	IPPK

chr14:46920011-47035304	20	MDGA2
chr3:196069454-196071498	6	TFRC
chr10:72128081-72152988	15	ASCC1
chr19:49599271-49599938	2	PRR12
chr7:74674094-74674345	15	nogene
chr22:50409074-50409471	33	nogene
chr8:47933019-47954446	16	PRKDC
chr6:38256408-38288461	8	BTBD9
chr3:138077498-138084253	3	DZIP1L
chr8:125059221-125063651	1	KIAA0196
chr5:150374616-150375554	24	TCOF1
chr21:36247516-36251284	41	DOPEY2
chr10:63380317-63409988	25	JMJD1C
chr2:36369107-36464655	1	CRIM1
chr20:63928334-63931022	519	DNAJC5
chr16:1809237-1823037	20	HAGH
chr19:57455652-57456182	16	nogene
chr9:81590804-81593274	20	TLE1
chr8:94867275-94876147	24	INTS8
chr2:99442316-99464985	33	REV1
chr5:110742046-110756759	8	SLC25A46
chr5:78509783-78510398	147	LHFPL2
chr6:149676259-149680457	15	LATS1
chr1:36004340-36014048	33	AGO3
chr13:102816427-102822159	17	BIVM
chr14:68904654-68912242	18	ACTN1
chr5:65242103-65273447	102	ADAMTS6
chr20:21326278-21357792	24	XRN2
chr20:6473538-6477363	1	CASC20
chr5:57230845-57231321	55	GPBP1
chr17:30607551-30610942	40	LRRC37BP1
chr1:5927648-5961949	2	NPHP4
chr14:29632868-29634541	2	PRKD1
chr1:168239130-168243069	1	SFT2D2
chr8:70583163-70600082	2	TRAM1
chr3:47994423-47998879	18	MAP4
chr17:80289658-80291827	3	RNF213
chr1:114461053-114464388	3	TRIM33
chr4:83045625-83049984	1	COPS4
chr16:4332214-4335393	30	GLIS2
chr6:151034492-151037117	16	MTHFD1L
chr7:33357854-33388144	57	BBS9
chr7:6037423-6039014	12	EIF2AK1

chr9:33060464-33073806	12	SMU1
chr8:99096311-99193057	23	VPS13B
chr10:91425280-91462184	174	HECTD2
chr20:35366556-35384133	344	UQCC1
chr17:4235771-4242448	31	ANKFY1
chr5:55968275-55969855	13	IL6ST
chr1:40189054-40222710	25	RLF
chr6:31410029-31505098	62	nogene
chr15:34932317-34942080	18	AQR
chr11:12208022-12259897	1	MICAL2
chrX:109659353-109683375	2	ACSL4
chr17:1436789-1437155	40	CRK
chr9:105464235-105484502	17	FSD1L
chr1:100043072-100061949	16	HIAT1
chr7:67049948-67083539	1	TYW1
chr14:58215204-58223856	2	ACTR10
chr1:51402434-51408332	104	EPS15
chr6:117566853-117575352	4	GOPC
chr4:142305457-142405324	12	INPP4B
chr15:49027364-49037769	26	SECISBP2L
chr14:50148284-50150230	13	SOS2
chr12:104326346-104334332	15	TXNRD1
chr8:102834188-102836541	12	AZIN1
chr12:123639263-123652735	80	GTF2H3
chrX:19407187-19426343	18	MAP3K15
chr18:20990616-20990870	2	nogene
chr5:73749836-73795391	11	ARHGEF28
chr3:197974376-197980504	12	LMLN
chr1:156743123-156743420	28	nogene
chr20:47262287-47287284	1	ZMYND8
chr5:14290715-14304592	342	TRIO
chr7:2364371-2364529	13	EIF3B
chr1:20969473-21050994	37	EIF4G3
chr19:33021570-33044364	2	RHPN2
chr10:91819269-91822362	1	TNKS2
chr12:110395100-110396452	5	ANAPC7
chr8:99013759-99275254	13	VPS13B
chr7:35638349-35670328	2	HERPUD2
chr5:138018301-138019146	9	FAM13B
chr11:47835650-47837623	35	NUP160
chr14:49831298-49834449	3	NEMF
chr5:33588598-33596060	3	ADAMTS12
chr2:121601567-121601922	32	nogene

chr9:125055875-125066058	14	SCAI
chr16:2317787-2319580	0	ABCA3
chr2:203402575-203417081	35	ABI2
chr17:1083519-1100735	10	ABR
chr17:19931804-19936430	2	AKAP10
chr11:46493607-46548500	7	AMBRA1
chr17:4183397-4185046	13	ANKFY1
chr4:73118687-73125312	85	ANKRD17
chr18:14183651-14191917	0	ANKRD20A5P
chr15:50968262-50984145	0	AP4E1
chr15:34860041-34867609	20	AQR
chr4:147909731-147955374	21	ARHGAP10
chr6:129583987-129605959	0	ARHGAP18
chr12:101396398-101405981	168	ARL1
chr7:99385701-99388261	2	ARPC1B
chr2:25759481-25771540	5	ASXL2
chr8:123359576-123361646	0	ATAD2
chr12:110327552-110328017	0	ATP2A2
chr19:1791749-1792135	17	ATP8B3
chr20:56386256-56388202	62	AURKA
chr16:88006089-88038011	20	BANP
chr22:17692355-17702386	0	BCL2L13
chr2:32501712-32508259	0	BIRC6
chr2:32543240-32575366	7	BIRC6
chr11:63817804-63819002	2	C11orf84
chr4:128008919-128021238	4	C4orf29
chr9:26861065-26887513	68	CAAP1
chr1:64663694-64664679	36	CACHD1
chr1:15493891-15495452	18	CASP9
chr4:77048448-77066405	17	CCNI
chr5:138201106-138202155	0	CDC23
chr17:47137151-47143982	8	CDC27
chr17:40293455-40294503	16	CDC6
chr16:22349278-22349849	221	CDR2
chr6:4881364-4892379	30	CDYL
chr1:26239761-26259802	26	CEP85
chr19:32881843-32899998	0	CEP89
chr21:36402757-36411604	35	CHAF1B
chr16:69150536-69154457	4	CIRH1A
chr16:1453833-1465338	0	CLCN7
chr17:31852811-31853030	0	COPRS
chr16:1632211-1641187	64	CRAMP1L
chr12:56275995-56282991	51	CS

chr20:49074784-49078622	6	CSE1L
chr1:112412193-112416264	13	CTTNBP2NL
chr10:35032434-35039082	62	CUL2
chr10:35013698-35028887	23	CUL2
chr2:224495831-224500487	3	CUL3
chr2:224495831-224514772	26	CUL3
chr6:117503766-117521576	0	DCBLD1
chr11:108688935-108706837	5	DDX10
chr12:31084582-31085126	3	DDX11
chr10:68911067-68914204	86	DDX50
chr6:35312213-35312772	0	DEF6
chr1:197617658-197647114	0	DENND1B
chr11:9150080-9170777	19	DENND5A
chr12:15956935-15962947	10	DERA
chr7:111935539-111945798	28	DOCK4
chr2:26429627-26432006	2	DRC1
chr2:25576837-25596240	43	DTNB
chr11:103303092-103312033	32	DYNC2H1
chr1:46683299-46691937	34	EFCAB14
chr6:52429968-52438591	1	EFHC1
chr2:62706990-62831158	0	EHBP1
chr1:20941490-20973099	0	EIF4G3
chr22:41135812-41140257	81	EP300
chr5:65966735-65994864	1	ERBB2IP
chr18:21536041-21540700	27	ESCO1
chr10:97437190-97443311	0	EXOSC1
chr10:97436416-97438703	15	EXOSC1
chr1:28027788-28042650	18	EYA3
chr2:131067090-131082657	0	FAM168B
chr5:74834425-74841679	801	FAM169A
chr14:45148836-45159280	20	FANCM
chr2:241403827-241411130	18	FARP2
chr6:98875414-98905670	5	FBXL4
chr14:55350380-55355222	14	FBXO34
chr5:148394713-148402513	21	FBXO38
chr3:196577452-196577809	1	FBXO45
chr4:174259698-174263100	4	FBXO8
chr5:108832769-108832943	0	FER
chr6:109727108-109743772	13	FIG4
chr1:240177920-240211235	144	FMN2
chr17:82718905-82720363	0	FN3KRP
chr13:49138746-49167303	19	FNDC3A
chr2:48346200-48346751	118	FOXN2

chr4:876529-877809	1	GAK
chr20:25417103-25441776	19	GINS1
chr9:3932359-3937189	4	GLIS3
chr1:28681085-28683740	2	GMEB1
chr2:53693883-53716743	13	GPR75-ASB3
chr19:42232056-42236941	25	GSK3A
chr9:133042086-133043926	79	GTF3C5
chr10:68014025-68038169	19	HERC4
chr1:113940381-113941459	48	HIPK1
chr5:133061815-133070496	0	HSPA4
chr5:5466333-5476079	14	ICE1
chr11:77921339-77922471	36	INTS4
chr11:77891318-77891840	20	INTS4
chr16:19730429-19733827	0	IQCK
chr12:26681873-26695650	24	ITPR2
chr13:30280062-30284882	92	KATNAL1
chr19:5119117-5119852	0	KDM4B
chr19:34327420-34330347	46	KIAA0355
chr6:17775014-17779099	21	KIF13A
chr7:130116444-130129576	3	KLHDC10
chr16:87748672-87762150	0	KLHDC4
chr11:118497935-118499420	10	KMT2A
chr22:33713547-33761558	2	LARGE
chr12:50453459-50454417	13	LARP4
chr14:92709671-92714451	17	LGMN
chr12:70219392-70225390	0	LINC01481
chr7:131052424-131053465	0	LINC-PINT
chr4:32146052-32158081	0	LOC102723828
chrX:19398225-19400663	2	MAP3K15
chr3:47998637-48065951	12	MAP4
chr2:39307942-39333574	14	MAP4K3
chr15:52045829-52050137	51	MAPK6
chr5:10415472-10417404	0	6-Mar
chr12:57498156-57498623	89	MARS
chr5:69424600-69433093	0	MARVELD2
chr10:72868702-72871580	3	MCU
chr9:36597221-36607673	30	MELK
chr5:179798512-179799121	14	MGAT4B
chr22:46080338-46109353	0	MIRLET7BHG
chr8:54147381-54148266	2	MRPL15
chr15:52428397-52433285	0	MYO5A
chr10:93396141-93431516	0	MYOF
chr13:35056009-35070852	0	NBEA

chr17:16177331-16194639	0	NCOR1
chr17:16164978-16194639	1	NCOR1
chr16:15664735-15667439	43	NDE1
chr16:23581053-23585423	12	NDUFAB1
chr2:206149779-206152510	0	NDUFS1
chr2:206127796-206133105	4	NDUFS1
chr3:131027929-131109921	0	NEK11
chr3:42621428-42632823	3	NKTR
chr1:169230717-169287408	286	NME7
chr6:42992933-43001374	0	nogene
chr5:179844364-179847400	0	nogene
chr1:247155565-247159867	8	nogene
chr19:33194286-33205759	19	nogene
chr9:111911025-111911611	33	nogene
chr11:65436537-65441442	0	nogene
chr12:11121009-11133367	26	nogene
chr6:142820032-142870339	0	nogene
chr15:67600644-67661494	31	nogene
chr11:65898455-65898727	40	nogene
chr19:11648357-11651885	0	nogene
chr17:13865078-13898732	1	nogene
chr16:15554687-15554998	0	nogene
chr19:11648357-11650417	18	nogene
chr10:100361532-100361724	3	nogene
chr12:87412676-87415076	0	nogene
chr7:128838314-128845194	0	nogene
chr1:8743202-8743493	15	nogene
chr4:48291800-48295899	0	nogene
chr17:76419610-76419965	0	nogene
chr6:126112865-126115855	5	nogene
chr5:151824990-151889465	21	nogene
chr17:46966533-46970309	18	nogene
chr1:58713055-58718748	46	nogene
chr9:79193521-79201407	0	nogene
chr3:113719462-113719743	0	nogene
chr4:35983011-36012831	0	nogene
chr5:177235820-177239865	86	NSD1
chr1:232955556-232956443	15	NTPCR
chr14:31562067-31673574	15	NUBPL
chr7:44404439-44427703	6	NUDCD3
chr16:56446625-56447989	1	NUDT21
chr1:229489954-229498306	40	NUP133
chr11:47783072-47785063	0	NUP160

chr2:183151507-183188763	18	NUP35
chr6:149742389-149743715	8	NUP43
chr15:41356038-41375828	114	NUSAP1
chr11:74279387-74298361	0	P4HA3
chr13:28174271-28177935	401	PAN3
chr13:28174271-28197346	36	PAN3
chr16:14586317-14627336	21	PARN
chr13:100257594-100309908	17	PCCA
chr14:70988566-71023232	5	PCNX
chr1:119734633-119737266	19	PHGDH
chr16:71672261-71676649	20	PHLPP2
chr7:77840220-77922778	1	PHTF2
chr18:42037691-42058051	0	PIK3C3
chr1:151239954-151242567	25	PIP5K1A
chr20:45902266-45902604	0	PLTP
chr1:151427822-151442205	15	POGZ
chr19:1090907-1091907	14	POLR2E
chr4:105420329-105424322	0	PPA2
chr4:56403039-56406618	15	PPAT
chr6:149533457-149541586	27	PPIL4
chr2:48498492-48511468	10	PPP1R21
chr4:101093775-101099722	0	PPP3CA
chr11:129955511-129961082	7	PRDM10
chr4:120798266-120821317	0	PRDM5
chr6:57526954-57537625	4	PRIM2
chr8:47953619-47957431	132	PRKDC
chr17:18865468-18867334	37	PRPSAP2
chr1:151017811-151025673	4	PRUNE
chr10:16484336-16486617	33	PTER
chr12:56671748-56673065	127	PTGES3
chr8:140700890-140702707	85	PTK2
chr9:122957010-122996151	15	RABGAP1
chr1:174219124-174252590	0	RABGAP1L
chr7:66805139-66810847	0	RABGEF1
chr7:66795510-66805396	19	RABGEF1
chr9:107321982-107325004	33	RAD23B
chr1:178283563-178420620	2	RASAL2
chr18:22982217-22984990	0	RBBP8
chr7:155763808-155774647	19	RBM33
chr3:50108069-50109688	0	RBM5
chr12:15111343-15348437	10	RERG
chr6:111349217-111357125	4	REV3L
chr1:176149005-176184692	76	RFWD2

chr12:106825725-106860412	13	RIC8B
chr8:86486483-86507112	27	RMDN1
chr16:2262277-2264331	49	RNPS1
chr1:27897004-27907282	0	RPA2
chr8:73291042-73292797	21	RPL7
chr1:150457287-150457570	17	RPRD2
chr16:57216106-57221415	0	RSPRY1
chr6:106583327-106602922	33	RTN4IP1
chr3:128097299-128104924	70	RUVBL1
chr10:70153837-70161931	11	SAR1A
chr11:9895942-9963882	0	SBF2
chr14:30705822-30734858	17	SCFD1
chrX:18304971-18334095	15	SCML2
chrX:18246576-18257030	0	SCML2
chr7:29923955-29926632	20	SCRN1
chr3:185614563-185624082	0	SENP2
chrX:119640691-119675668	0	6-Sep
chr16:70532478-70541834	63	SF3B3
chr16:28871779-28873579	2	SH2B1
chrX:19683822-19695741	90	SH3KBP1
chr22:40777039-40794580	2	SLC25A17
chr2:26764682-26775215	0	SLC35F6
chr3:126050897-126068246	0	SLC41A3
chr18:47848474-47865133	0	SMAD2
chr18:2688393-2688747	15	SMCHD1
chr1:245858495-245915640	23	SMYD3
chr19:45686533-45688566	14	SNRPD2
chr5:122801868-122808355	0	SNX2
chr4:185247293-185346650	13	SNX25
chr7:2264297-2278305	20	SNX8
chr6:107633513-107635469	18	SOBP
chr15:44567423-44572820	3	SPG11
chr18:12493071-12546904	29	SPIRE1
chr2:54616206-54623596	0	SPTBN1
chr2:45546731-45553730	366	SRBD1
chr1:70221614-70228555	24	SRSF11
chr14:69770466-69771544	0	SRSF5
chr2:85861180-85863485	2	ST3GAL5
chr3:136477288-136521417	0	STAG1
chr8:73595165-73688813	1	STAU2
chr20:44995089-45001353	103	STK4
chr1:172551511-172591071	31	SUCO
chr9:33503826-33510711	21	SUGT1P1

chr3:4449265-4453049	18	SUMF1
chr6:45003652-45020626	0	SUPT3H
chr1:185202653-185222036	0	SWT1
chr4:1727707-1728787	4	TACC3
chr17:37437737-37440662	5	TADA2A
chr1:234463836-234472811	0	TARBP1
chr20:13559007-13630152	13	TASP1
chr10:94441895-94500327	34	TBC1D12
chr6:121106022-121131752	2	TBC1D32
chr6:121126377-121170527	2	TBC1D32
chr8:55792697-55813118	14	TGS1
chr2:241632916-241634079	198	THAP4
chr2:241601895-241634079	0	THAP4
chr22:29517028-29519120	1	THOC5
chr9:100498764-100561520	12	TMEFF1
chr9:109073355-109093591	18	TMEM245
chr15:43148754-43160794	15	TMEM62
chr22:40273424-40285770	56	TNRC6B
chr9:98091908-98095029	18	TRIM14
chr7:138504289-138519321	0	TRIM24
chr5:904147-908461	3	TRIP13
chr15:64414084-64425631	4	TRIP4
chr22:46343261-46346544	16	TRMU
chr20:35035122-35055031	17	TRPC4AP
chr2:32630522-32650233	6	TTC27
chr22:28296197-28306643	55	TTC28
chr18:24123833-24130417	3	TTC39C
chr7:67024899-67050066	0	TYW1
chr7:67098540-67117618	185	TYW1
chr1:162576776-162600903	132	UAP1
chr19:34458768-34467014	15	UBA2
chr6:42615070-42617338	0	UBR2
chr2:169926691-169933008	0	UBR3
chr1:161153997-161157693	0	UFC1
chr13:27069110-27090158	40	USP12
chr2:233549100-233551519	37	USP40
chr2:63949036-63972244	0	VPS54
chr3:184824544-184839752	0	VPS8
chr13:51719197-51739175	13	WDFY2
chr2:159248371-159256375	2	WDSUB1
chr16:69783031-69799295	0	WWP2
chr19:43553706-43554645	0	XRCC1
chr17:1361091-1362008	730	YWHAE

chr4:112562370-112589874	46	ZGRF1
chr20:47246007-47262428	0	ZMYND8
chr19:51877281-51886912	95	ZNF577
chr4:145886032-145903215	0	ZNF827
chr20:49260462-49263483	3	ZNFX1
chr2:113916883-113934386	2	ACTR3
chr15:100199317-100331054	27	ADAMTS17
chr15:72518066-72555907	113	ARIH1
chr8:123357561-123361646	1	ATAD2
chr2:28025225-28129380	3	BRE
chr17:63760913-63766194	60	CCDC47
chr9:120529977-120568388	35	CDK5RAP2
chr8:60741258-60743097	127	CHD7
chr17:30785918-30792572	16	CRLF3
chr10:43163865-43176032	7	CSGALNACT2
chr15:65741714-65752628	1	DENND4A
chr3:124857231-124859446	25	ITGB5
chr13:30227411-30241681	1	KATNAL1
chr2:61113769-61118116	115	KIAA1841
chr6:83194530-83195739	19	nogene
chr1:246186742-246195464	54	nogene
chr9:93629969-93636525	71	PHF2
chr3:52122378-52138302	43	POC1A
chr7:100111727-100112253	9	TAF6
chr2:159194256-159199053	14	TANC1
chr2:170015280-170055584	2	UBR3
chr9:133806080-133834340	14	VAV2
chr8:144829042-144837507	18	ZNF7
chr13:29553760-29560349	87	SLC7A1
chr2:121757239-121761524	475	TSN
chr11:93702459-93706897	24	CEP295
chr4:150735257-150872755	36	LRBA
chr16:19703041-19704172	15	nogene
chr17:2603507-2603835	14	nogene
chr16:11733981-11736233	2	TXNDC11
chr9:132658670-132662695	34	DDX31
chr6:161165576-161166417	23	AGPAT4
chr11:128758114-128782023	23	FLI1
chr10:3163724-3167042	24	PITRM1
chr1:46309100-46310316	40	UQCRH
chr9:85974966-85978381	33	NAA35
chr16:66813563-66823600	27	NAE1
chr2:85043563-85046278	45	KCMF1

chr14:102968252-102970261	42	CDC42BPB
chr22:41140139-41141222	33	EP300
chr11:78465875-78571444	8	NARS2
chr4:122170614-122175914	14	KIAA1109
chr6:53076485-53081098	12	FBXO9
chr3:155893517-155911279	203	GMPS
chrX:63314996-63323240	7	nogene
chr2:120278617-120293309	18	RALB
chr6:144537581-144542870	63	UTRN
chr8:67287954-67302466	63	ARFGEF1
chr8:90042727-90044995	15	DECR1
chr8:120453846-120463761	1	MTBP
chr13:24459043-24493733	37	PARP4
chr15:43146492-43160794	10	TMEM62
chr6:145671238-145686296	4	EPM2A
chr2:38313150-38343512	0	ATL2
chr12:22482556-22493337	0	C2CD5
chr22:19048439-19089490	3	DGCR2
chr10:99894945-99898760	15	DNMBP
chr17:82091707-82092454	0	FASN
chr2:175964371-175979868	36	KIAA1715
chr7:131387119-131411383	58	MKLN1
chr14:50738139-50741728	0	NIN
chr11:29044444-29106315	0	nogene
chr5:132920343-132920876	33	nogene
chr8:125182102-125195272	214	NSMCE2
chr20:38488402-38497516	1	RALGAPB
chr8:47728932-47729465	1	SPIDR
chr17:82756164-82772507	216	TBCD
chr10:104018159-104018908	4	SLK
chr15:49225204-49328130	21	nogene
chr13:100309832-100368574	3	PCCA
chr1:222715683-222722514	4	BROX
chr4:82454721-82457038	132	ENOPH1
chr2:47818778-47819078	41	FBXO11
chr10:63217206-63219983	24	JMJD1C
chr1:77734346-77736158	3	USP33
chr10:68737844-68747566	21	CCAR1
chr19:45357269-45357699	40	ERCC2
chr17:44424348-44436545	31	GPATCH8
chr2:20307977-20318645	41	PUM2
chr2:61178980-61185618	40	AHSA2
chr15:85655416-85669830	20	AKAP13

chr15:50719939-50749746	3	SPPL2A
chr4:153550072-153558368	39	KIAA0922
chr17:82563353-82571870	180	FOXK2
chr19:43880009-43884051	35	ZNF404
chr21:32453311-32467848	25	EVA1C
chr5:177626435-177632738	53	LOC202181
chr22:32478980-32485209	174	FBXO7
chr12:64423004-64431823	4	XPOT
chr1:162566011-162597858	69	UAP1
chr14:68890138-68893747	25	ACTN1
chr11:65855934-65856242	19	CFL1
chr9:112405623-112409021	19	HSDL2
chr1:226265532-226268090	3	LIN9
chr16:18841564-18845651	140	SMG1
chr3:52412810-52414587	96	PHF7
chr11:112078214-112080558	49	C11orf57
chr3:32532985-32541206	3	DYNC1LI1
chr2:15394226-15427794	26	NBAS
chr12:102257214-102423180	3	nogene
chr9:133437748-133438366	42	ADAMTS13
chr17:12908896-12929046	17	ARHGAP44
chr1:62803718-62810586	25	ATG4C
chr5:69292187-69311204	1	CCDC125
chr1:97515725-97595166	36	DPYD
chr15:49292326-49308777	7	GALK2
chr15:49201161-49239367	6	GALK2
chr3:33068229-33068970	124	GLB1
chr16:27537783-27545523	3	GTF3C1
chr14:50265357-50303017	2	L2HGDH
chr16:72284571-72349206	5	LINC01572
chr11:94429910-94447438	100	MRE11A
chr5:16684969-16685320	1	nogene
chr10:49832802-49857453	4	PARG
chr19:43186341-43198275	2	PSG5
chr6:42885973-42886470	24	RPL7L1
chr11:66365935-66369532	38	SLC29A2
chr4:122935713-122938249	5	SPATA5
chr11:119070233-119073951	9	VPS11
chr9:19356971-19360489	20	DENND4C
chr4:74199793-74201370	9	MTHFD2L
chr3:154217868-154240579	45	ARHGEF26
chr7:33146241-33183405	43	BBS9
chr12:50430494-50437838	11	LARP4

chr20:34297589-34297878	15	nogene
chr12:128799258-128801009	19	SLC15A4
chr2:134866541-134869041	6	CCNT2-AS1
chr16:27749583-27751924	20	KIAA0556
chr4:26748410-26764737	17	TBC1D19
chr16:67036638-67082308	420	CBFB
chr9:77794462-77815770	41	GNAQ
chr10:67954594-68014186	46	HERC4
chr12:98548946-98549335	15	nogene
chr12:112946742-112950975	11	OAS3
chr1:112576985-112600848	36	ST7L
chr13:98519242-98519473	54	STK24
chr1:40540585-40541710	13	ZNF684
chr16:70314901-70317588	12	DDX19B
chr17:82900650-82909307	54	TBCD
chr17:66732687-66738855	2	PRKCA
chr11:73363833-73364134	0	ARHGEF17
chr9:96471010-96484633	0	HABP4
chr2:26212569-26215175	0	HADHA
chr2:240782589-240789312	0	KIF1A
chr12:121416158-121420334	15	RNF34
chr1:186341027-186344090	23	TPR
chr1:52471951-52475535	0	ZCCHC11
chr7:55171174-55174820	17	EGFR
chr3:136989440-136995556	21	IL20RB
chr5:131510313-131521521	1	RAPGEF6
chr5:134697125-134708888	11	SEC24A
chr21:32457596-32467848	2	EVA1C
chr19:39457674-39459960	17	SUPT5H
chr8:124515745-124523002	6	TATDN1
chr16:58670112-58672243	16	SLC38A7
chr2:46950362-46978907	7	TTC7A
chr11:66748378-66788269	12	C11orf80
chr6:15374116-15410365	6	JARID2
chr16:25164597-25170805	13	LCMT1
chr17:59070822-59084089	54	TRIM37
chr11:33551039-33552241	172	KIAA1549L
chr19:34633158-34669864	20	nogene
chr8:98123335-98130226	32	POP1
chr6:57193841-57207713	1	RAB23
chr3:49368427-49373342	14	RHOA
chr11:65434478-65437657	15	nogene
chr10:179993-210048	34	ZMYND11

chr3:119905754-119947351	12	GSK3B
chr17:12054888-12095694	509	MAP2K4
chr17:17792932-17803849	1	RAI1
chr18:22968805-22984990	23	RBBP8
chr18:70145611-70150733	19	RTTN
chr9:14639895-14680162	394	ZDHHC21
chr19:9566335-9568175	55	ZNF121
chr20:62854015-62859526	48	TCFL5
chr2:120019076-120042110	65	EPB41L5
chr12:122530021-122539754	19	KNTC1
chr6:88769773-88853764	80	RNGTT
chr1:40267785-40286029	4	ZMPSTE24
chr6:35637013-35646366	15	FKBP5
chr15:67208561-67236820	0	AAGAB
chr12:70319297-70330469	7	CNOT2
chr20:49084025-49089746	0	CSE1L
chr11:33337074-33341686	15	HIPK3
chr13:75621762-75804541	13	LMO7
chrX:120162946-120240355	0	NKAPP1
chr22:43039779-43046544	0	nogene
chrX:123686547-123703505	15	THOC2
chr12:29356381-29361685	16	ERGIC2
chr5:162093827-162104026	27	GABRG2
chr3:144237757-144304804	21	nogene
chr5:55322326-55328811	52	SKIV2L2
chr17:78803384-78803978	57	USP36
chr15:64930821-64934234	1	ANKDD1A
chr17:18872582-18882683	1	PRPSAP2
chr10:68959805-68967203	31	DDX21
chr10:34696317-34728116	17	PARD3
chr3:47706408-47736126	26	SMARCC1
chr3:52730324-52741499	16	NEK4
chr11:66651739-66657259	16	nogene
chr1:108896863-108904254	17	GPSM2
chr22:19253958-19254227	20	CLTCL1
chr2:190900563-190905167	43	GLS
chr13:52632421-52637446	17	HNRNPA1L2
chr14:21230318-21234229	1171	HNRNPC
chr15:69423158-69426457	28	KIF23
chr18:58342905-58357252	17	NEDD4L
chr11:102362895-102368548	318	BIRC2
chr18:6974902-6980637	10	LAMA1
chr15:30745021-30750556	51	LOC100288637

chr13:114040999-114057474	67	RASA3
chr11:73707419-73718718	567	RAB6A
chr11:108838445-108852209	11	DDX10
chr20:34089515-34093731	1	EIF2S2
chr19:4110508-4117629	15	MAP2K2
chr11:28290205-28296931	1	METTL15
chr2:24554388-24584560	7	NCOA1
chr1:176162868-176163891	4	RFWD2
chr16:53619024-53622356	39	RPGRIP1L
chr9:15199860-15211397	11	TTC39B
chr19:34431860-34434968	16	UBA2
chr9:77238001-77252352	6	VPS13A
chr9:19360243-19361963	6	DENND4C
chr16:931641-954666	2	LMF1
chr8:120413990-120432123	40	MRPL13
chr3:101579789-101585511	8	PCNP
chr1:26729650-26732792	638	ARID1A
chr22:35936290-35938897	14	RBFOX2
chr9:133806080-133861432	4	VAV2
chr13:95723241-95725252	47	DNAJC3
chr1:99668622-99671404	20	nogene
chr18:12506476-12635096	19	SPIRE1
chr9:33953284-33963791	853	UBAP2
chr1:155269428-155270977	1	CLK2
chr5:75411010-75417033	3	COL4A3BP
chr2:36396613-36464655	245	CRIM1
chr9:96949554-96972948	90	HIATL2
chr7:1486624-1489682	9	INTS1
chr16:206003-220747	1	LUC7L
chr12:82369386-82434724	1	METTL25
chr8:70213902-70296800	6	NCOA2
chr8:140700890-140706205	392	PTK2
chr18:12830942-12859254	1	PTPN2
chr2:11235701-11235962	1	ROCK2
chr7:35781208-35794022	4	SEPT7-AS1
chr8:123077110-123097441	97	TBC1D31
chr18:23309679-23373763	25	TMEM241
chr1:54040755-54047372	14	TMEM59
chr1:165680486-165695397	9	ALDH9A1
chr9:20715321-20885230	2	FOCAD
chr6:125855019-125855240	3	NCOA7
chr6:57182031-57190666	5	BAG2
chrX:129908059-129911921	45	UTP14A

chr9:96515648-96565483	28	CDC14B
chr22:33564847-33577964	104	LARGE
chr10:27180952-27181581	21	MASTL
chr6:7187433-7189322	32	RREB1
chr7:43595900-43608400	16	STK17A
chr4:13179195-13182068	4	nogene
chr19:53555844-53571730	3	ZNF331
chr7:100102674-100102960	30	AP4M1
chr1:235454155-235470960	0	B3GALNT2
chr1:108930070-108932501	0	CLCC1
chr12:132037681-132038096	39	EP400
chr1:153663214-153665336	15	ILF2
chr15:100922774-100924729	2	LRRK1
chr10:26966678-26969653	0	nogene
chr14:90610741-90786328	0	TTC7B
chr3:38362936-38365736	0	XYLB
chr17:18330554-18340813	16	SHMT1
chr1:114463103-114464388	429	TRIM33
chr9:2115821-2123937	13	SMARCA2
chr6:144482208-144485519	12	UTRN
chr3:121467518-121468431	2	POLQ
chr22:25375812-25376008	33	LRP5L
chr1:145825756-145827005	25	POLR3C
chr1:39370029-39372596	28	MACF1
chr16:74660931-74661451	182	RFWD3
chr7:85055716-85065552	15	SEMA3D
chr9:77794462-77824880	14	GNAQ
chr12:32293993-32306122	4	BICD1
chr1:225117683-225123614	9	DNAH14
chr1:32030297-32037043	23	KHDRBS1
chr2:237748363-237760205	30	LRRFIP1
chr3:104764378-104842744	1	nogene
chrX:68096869-68193952	4	OPHN1
chr10:73072028-73074915	10	P4HA1
chr10:94474667-94500327	21	TBC1D12
chr2:61488164-61490776	19	XPO1
chr6:4042486-4044023	19	PRPF4B
chr1:70296129-70315566	28	ANKRD13C
chr2:55232807-55234962	19	RPS27A
chr2:68141693-68147567	11	WDR92
chr3:43565652-43580472	14	ANO10
chr4:150302624-150350159	3	LRBA
chr7:134137022-134183853	17	LRGUK

chr20:38065802-38066256	172	RPRD1B
chr1:28491649-28493091	9	PHACTR4
chr3:125277725-125331238	120	ZNF148
chr3:33687059-33689932	13	CLASP2
chr7:66130743-66134374	102	CRCP
chr20:37777344-37779335	13	CTNNBL1
chr7:886055-893971	18	GET4
chr8:66804374-66822459	3	SGK3
chr2:32514989-32518946	27	BIRC6
chr7:6042932-6050045	36	EIF2AK1
chr2:15475686-15478289	2	NBAS
chr9:22301599-22337312	5	nogene
chr3:10028624-10036339	21	FANCD2
chr3:183775914-183790980	25	YEATS2
chr7:152814549-152825122	5	ACTR3B
chr5:77098654-77118521	17	ZBED3-AS1
chr5:57246299-57251141	52	GPBP1
chr10:119051198-119059397	4	EIF3A
chr5:41850029-41862750	35	OXCT1
chr11:47582087-47582468	28	NDUFS3
chr12:42374862-42398994	160	PPHLN1
chr11:65502320-65505019	14	nogene
chr12:22469708-22474891	1	C2CD5
chr4:152829624-152872564	41	ARFIP1
chr8:130179263-130187285	23	ASAP1
chr7:72830691-72831798	30	SBDSP1
chr9:128739930-128742745	12	ZER1
chr2:15461200-15478289	152	NBAS
chr1:193138084-193142066	52	CDC73
chr8:67131950-67137603	151	CSPP1
chr6:100848147-100848707	25	ASCC3
chr10:65585664-65615761	17	LINC01515
chr17:16137310-16146548	34	NCOR1
chr17:68522755-68524958	2	PRKAR1A
chr14:30670255-30694869	2	SCFD1
chr1:32553458-32557965	14	ZBTB8A
chr17:44154555-44155411	142	C17orf53
chr5:31448546-31495372	16	DROSHA
chr7:105073618-105081797	50	KMT2E
chr7:4076997-4079584	16	SDK1
chr12:102039867-102061346	2	CCDC53
chr1:222594235-222603276	42	nogene
chr15:89792159-89793126	8	ANPEP

chr2:168012633-168129758	12	STK39
chr16:47451395-47459175	41	ITFG1
chr4:127930682-127933093	16	MFSD8
chr7:6432788-6436533	46	DAGLB
chr12:31442774-31452476	15	DENND5B
chr14:76154743-76195972	2	GPATCH2L
chr6:57379900-57507454	2	PRIM2
chr11:76360955-76365972	85	PRKRIR
chr3:12935447-12941865	51	IQSEC1
chr3:132434537-132447939	9	DNAJC13
chr5:73901183-73911575	1	ARHGEF28
chr5:5447840-5457741	23	ICE1
chr12:122570875-122574380	3	KNTC1
chr12:57932009-57932421	3	nogene
chr4:76134174-76144473	359	NUP54
chr14:73071626-73077536	31	RBM25
chr20:33385649-33392242	15	CDK5RAP1
chr11:78465875-78469313	15	NARS2
chr16:21315310-21317126	33	nogene
chr9:132271709-132281574	1	SETX
chr8:47912409-47915418	21	PRKDC
chr4:169585348-169599197	12	NEK1
chr3:52737585-52752336	12	NEK4
chr12:42355160-42387535	11	PPHLN1
chr2:53887258-53893799	15	PSME4
chr13:29522316-29524253	12	SLC7A1
chr13:21400372-21413772	12	ZDHHC20
chr3:155903862-155911279	23	GMPS
chr11:66365935-66366564	15	SLC29A2
chr2:207976650-207977586	368	PLEKHM3
chrX:71303261-71304300	1	ITGB1BP2
chr9:22820737-22821598	24	LINC01239
chr2:224556294-224558241	2	nogene
chr12:31423596-31424687	8	DENND5B
chr6:151331792-151332131	17	nogene
chr7:848417-866067	16	SUN1
chr17:50353629-50356773	12	XYLT2
chr15:49588015-49590007	14	FAM227B
chr5:146260744-146263631	18	RBM27
chr22:42808766-42817857	11	ARFGAP3
chr7:105472131-105502564	16	PUS7
chr16:87730551-87755292	4	KLHDC4
chr3:48927461-48980496	60	ARIH2

chr15:85075861-85083644	3	PDE8A
chr10:31373121-31461237	2	ZEB1
chr10:27167101-27171083	8	MASTL
chr10:50343491-50344345	35	SGMS1
chr11:992502-1000598	17	AP2A2
chr3:184964467-184983094	2	VPS8
chr3:123110943-123124343	12	PDIA5
chr5:128112813-128161800	17	SLC12A2
chr2:111831285-111838512	18	ANAPC1
chr8:103040797-103042407	24	ATP6V1C1
chr19:19492305-19492712	226	GATAD2A
chr15:64112572-64123546	57	SNX1
chr5:177209635-177212195	57	NSD1
chr22:31580271-31611840	3	SFI1
chr3:197814907-197820430	135	LRCH3
chr3:197051576-197069260	50	DLG1
chr9:127311635-127320778	34	GARNL3
chr15:63712774-63716473	38	HERC1
chr7:36330981-36334161	2	KIAA0895
chr15:55924645-55966546	8	NEDD4
chr19:44686055-44687108	2	nogene
chr5:168488601-168494650	1469	RARS
chr10:75168540-75176216	3	SAMD8
chr6:107503656-107533610	90	SOBP
chr1:32094783-32100562	30	TMEM39B
chr3:179708772-179721589	2	USP13
chr11:76985642-76998821	6	ACER3
chr5:179708977-179710065	1	CANX
chr14:30911776-30956242	43	STRN3
chr12:122030699-122044053	55	BCL7A
chr19:17567405-17568713	11	COLGALT1
chr16:68038005-68054617	8	DUS2
chr1:92605306-92625934	27	EVI5
chr5:108341926-108348530	136	FBXL17
chr16:56434330-56447989	15	NUDT21
chr17:29684562-29703062	160	SSH2
chr5:71495249-71504751	4	BDP1
chr1:19770541-19787117	6	TMCO4
chr14:102983555-102986580	4	CDC42BPB
chr16:70514334-70519731	4	COG4
chr7:66395878-66402545	2	LINC00174
chr2:15488893-15539356	43	NBAS
chr1:5952699-5969259	2	NPHP4

chr8:65734793-65745470	2	PDE7A
chr3:170149085-170178938	9	PHC3
chr2:159149141-159150556	53	TANC1
chr5:157841488-157841730	58	nogene
chr7:3433042-3440256	15	nogene
chr12:113269756-113284771	1	TPCN1
chr6:30710662-30711130	15	nogene
chr1:244418759-244420296	18	ADSS
chr11:72867864-72889945	86	FCHSD2
chr5:108883395-108897848	15	FER
chr16:4822874-4832912	1	GLYR1
chr10:69392124-69395105	0	HK1
chr3:44801449-44812289	16	KIF15
chr2:46612227-46615185	36	PIGF
chr14:49663314-49674427	223	POLE2
chr20:13559007-13610919	12	TASP1
chr6:133980081-133984479	0	TBPL1
chr7:103025062-103029418	30	FBXL13
chr19:12928341-12928932	30	FARSA
chr7:156756392-156826744	180	LMBR1
chr7:22960255-22976305	2	FAM126A
chr5:108768092-108835807	6	FER
chr15:34896896-34897705	4	AQR
chr12:22484696-22513379	15	C2CD5
chr7:90726566-90726812	182	CDK14
chr20:33360350-33374212	1	CDK5RAP1
chr8:128009589-128010444	324	nogene
chr9:124971369-124999990	8	SCAI
chr4:109513746-109520484	17	SEC24B
chr7:2263229-2278305	60	SNX8
chr1:48355669-48359770	74	SPATA6
chr6:56699652-56704369	41	DST
chr6:90561621-90571807	16	MAP3K7
chr18:21765771-21773728	12	MIB1
chr11:126181494-126183087	54	nogene
chr2:183133565-183159652	36	NUP35
chr12:50437734-50454417	2	LARP4
chr1:233198938-233208689	247	PCNXL2
chr17:81270917-81276151	3	SLC38A10
chr15:29761138-29800702	33	TJP1
chr5:43122043-43139309	5	ZNF131
chr5:160035139-160043169	94	TTC1
chr6:170388286-170395579	93	FAM120B

chr15:89102499-89116521	2	ABHD2
chr2:9448197-9459618	15	CPSF3
chr7:919967-935505	34	ADAP1
chr7:44024915-44025559	2	nogene
chr15:90981747-90981981	2	PRC1
chr12:120557281-120557682	83	RNF10
chr10:73819531-73824084	3	CAMK2G
chr22:46384542-46386585	5	CELSR1
chr18:46815311-46821072	32	PIAS2
chr9:135866455-135883078	237	CAMSAP1
chr2:127271335-127293718	3	ERCC3
chr17:62524235-62536337	36	TLK2
chr2:74167013-74172752	7	MOB1A
chr19:5700788-5705992	9	LONP1
chr10:13172375-13186280	32	MCM10
chr1:95143890-95173889	282	TMEM56
chr16:69368375-69372355	29	TERF2
chr18:37066889-37084232	46	KIAA1328
chr2:171435082-171449957	14	DCAF17
chr18:58341037-58351045	11	NEDD4L
chr5:37302778-37303414	5	NUP155
chr9:41007439-41033022	3	nogene
chr3:129965700-129972108	48	nogene
chr12:27368261-27385560	30	ARNTL2
chr16:15483936-15515428	14	C16orf45
chr16:85633913-85634132	1198	GSE1
chr15:63747723-63756748	13	HERC1
chr11:108176245-108177090	198	NPAT
chr1:243270104-243308177	19	SDCCAG8
chrX:1435021-1439144	31	ASMTL
chr8:67045811-67047859	3	COPS5
chr1:243613670-243695716	19	AKT3
chr5:126559239-126570859	19	ALDH7A1
chr2:120027589-120042110	36	EPB41L5
chr2:26364301-26383347	39	EPT1
chr6:154860737-154860998	77	nogene
chr6:53285909-53286315	13	nogene
chr6:73635381-73638499	2	SLC17A5
chr5:73773854-73795391	76	ARHGEF28
chr20:51669996-51671257	5	ATP9A
chr2:117995445-118008658	14	CCDC93
chr1:207757013-207767195	8	CD46
chr11:9160712-9193681	36	DENND5A

chr12:101897862-101901433	36	DRAM1
chr21:37420298-37472880	265	DYRK1A
chr6:57924416-57930291	26	GUSBP4
chr12:82369448-82403130	1	METTL25
chr14:51489340-51491534	15	nogene
chrX:56014844-56024501	4	nogene
chr12:79817393-79822190	17	PPP1R12A
chr8:42861744-42865489	6	RNF170
chr7:127701162-127707647	4	SND1
chr17:9674890-9680366	5	USP43
chr5:73840479-73852692	15	ARHGEF28
chrX:30665510-30677452	9	GK
chr2:105296355-105298705	60	TGFBRAP1
chr2:203380207-203402734	78	ABI2
chr9:96522505-96565512	3	CDC14B
chr9:36246030-36246482	32	GNE
chr3:37273535-37289291	12	GOLGA4
chr7:131387119-131445903	14	MKLN1
chr1:241896958-241920227	1	nogene
chr3:110461309-110502152	1	nogene
chr5:37314197-37328420	1	NUP155
chr7:105505954-105508544	12	PUS7
chr1:179347599-179350431	9	SOAT1
chr8:124508473-124522200	19	TATDN1
chr1:168291159-168300483	4	TBX19
chr6:75260823-75267748	6	TMEM30A
chr7:23342063-23351586	256	IGF2BP3
chr5:36953617-36962274	72	NIPBL
chr7:92117043-92131872	10	CYP51A1
chr1:212872677-212889257	12	FLVCR1
chr20:36656359-36666392	9	NDRG3
chr9:124301935-124339665	11	NEK6
chr11:74634582-74640841	16	POLD3
chr12:68654111-68657217	13	RAP1B
chr2:3545700-3552308	7	RNASEH1
chr16:2253303-2254063	29	RNPS1
chr11:4101992-4112062	41	RRM1
chr11:77740730-77764689	87	RSF1
chr1:112581991-112600848	13	ST7L
chr17:31200421-31219118	5	NF1
chr19:45698546-45698900	4	QPCTL
chr9:111533738-111534353	96	ZNF483
chr15:92924320-92927330	17	CHD2

chr9:23762005-23765099	19	ELAVL2
chr16:74480240-74483124	14	GLG1
chr10:13170921-13172765	2	MCM10
chr12:76059797-76060279	46	NAP1L1
chr6:33697622-33710851	2	nogene
chr13:28628543-28636986	2	nogene
chr11:75413519-75414320	11	nogene
chr10:68487143-68506718	43	SLC25A16
chr8:102261914-102270183	26	UBR5
chr3:183752072-183762279	11	YEATS2
chr1:246435944-246441299	18	nogene
chr6:42615070-42635546	4	UBR2
chr6:99558496-99561681	20	CCNC
chr6:169582835-169662424	52	WDR27
chrX:152345580-152458262	11	GABRA3
chr1:205723925-205729621	56	NUCKS1
chr6:18255730-18258405	40	DEK
chr2:203208636-203209322	15	NBEAL1
chr3:134175608-134183071	12	RYK
chr20:46063359-46079453	4	NCOA5
chr7:158739022-158748280	20	ESYT2
chr6:169705132-169721111	3	PHF10
chr10:37833024-37856337	31	ZNF248
chr10:15821655-15843272	114	FAM188A
chr1:52396045-52397863	28	ORC1
chr13:30240459-30241086	8	KATNAL1
chr17:4049707-4051043	3	ZZEF1
chrX:119633359-119637195	2	6-Sep
chr11:74132843-74161556	28	C2CD3
chr3:179413411-179419505	33	GNB4
chr1:235985378-235991058	16	NID1
chr2:61280243-61281242	5	USP34
chr2:227531090-227552117	14	AGFG1
chr18:45907957-45913828	16	EPG5
chr16:81854443-81870935	2	PLCG2
chr16:30961266-30963554	5	SETD1A
chr7:66079177-66080133	6	nogene
chr8:134608728-134610655	31	ZFAT
chr19:47725811-47726224	126	EHD2
chr10:114830851-114848737	59	FAM160B1
chr13:24490667-24498229	26	PARP4
chr11:34931403-34960518	3	PDHX
chr1:935771-939412	26	SAMD11

chr5:38510463-38530666	25	LIFR
chr1:44757555-44758140	256	KIF2C
chr4:128074459-128082305	75	LARP1B
chr17:51201591-51203925	2	MBTD1
chr10:118009075-118040565	1	RAB11FIP2
chr2:85349383-85351021	9	RETSAT
chr3:47706408-47745993	3	SMARCC1
chr10:92474840-92475994	12	IDE
chr17:28349082-28355876	2	POLDIP2
chr6:147260614-147291172	14	STXBP5
chr12:7432415-7436602	2	CD163L1
chrX:154030297-154032557	14	MECP2
chrX:68192918-68206673	14	OPHN1
chr3:196806578-196818156	16	PAK2
chr7:140041643-140058034	6	PARP12
chr6:2835855-2840594	1	SERPINB1
chr18:56923921-56962529	7	WDR7
chr9:123557569-123879021	6	DENND1A
chr5:677749-678064	59	TPPP
chr1:94455732-94475737	16	ABCD3
chr14:93257194-93263993	13	BTBD7
chr2:174915057-174952202	325	CHN1
chr12:48923781-48925111	3	FKBP11
chr3:136464880-136477412	16	STAG1
chr8:125102050-125127067	18	NSMCE2
chr10:119155061-119160969	6	SFXN4
chr4:147965023-147966839	63	ARHGAP10
chr3:172760155-172783909	17	ECT2
chr2:43918024-43925961	126	LRPPRC
chr4:189895531-189937333	9	nogene
chr3:197817175-197820430	27	LRCH3
chr7:158679959-158689953	33	NCAPG2
chr4:47744401-47807047	8	CORIN
chr12:110536923-110551968	43	PPTC7
chr8:103514017-103697296	12	RIMS2
chr14:52744851-52759754	2	STYX
chr22:41135812-41141222	13	EP300
chr12:112450317-112456063	4	PTPN11
chr22:33761370-33767719	32	LARGE
chr19:37246075-37246491	14	nogene
chr4:39898388-39902460	122	PDS5A
chr7:105462620-105495253	14	PUS7
chr15:40389505-40392023	2	KNSTRN

chr7:155737529-155745602	6	RBM33
chr5:168500590-168516950	12	RARS
chr1:224267003-224268133	24	NVL
chr9:85575314-85596449	8	AGTPBP1
chr3:52686768-52689206	18	GNL3
chrX:77870807-77879613	19	MAGT1
chr3:196067517-196077122	12	TFRC
chr7:23415247-23418824	37	IGF2BP3
chr1:158077990-158078507	14	KIRREL
chr13:20051432-20067390	20	ZMYM2
chr7:6187049-6190531	62	CYTH3
chr16:69136695-69150904	15	CIRH1A
chr15:24979535-24982437	3	nogene
chr4:2956669-2957740	40	NOP14
chr15:40641378-40650819	31	nogene
chr1:109336239-109345878	21	SORT1
chr12:56741434-56744123	12	PRIM1
chrX:19950752-19955566	16	CXorf23
chr19:48904346-48905885	2	NUCB1
chr1:42278347-42278672	19	FOXJ3
chr21:28162191-28163717	24	LOC101927973
chr17:56848695-56856665	118	DGKE
chr3:158658921-158666386	7	GFM1
chr7:26196400-26197732	9	HNRNPA2B1
chr3:170259968-170270561	4	PRKCI
chr17:39490556-39511630	11	CDK12
chr15:101217998-101235577	2	CHSY1
chr12:53016472-53028188	103	EIF4B
chr2:239236592-239354414	12	HDAC4
chr2:210104103-210154611	32	KANSL1L
chr11:12162078-12162419	2	MICAL2
chr19:6230594-6270759	18	MLLT1
chr5:16761463-16762637	12	MYO10
chr12:87648516-87715112	3	nogene
chr13:25312882-25315467	3	NUPL1
chr11:74285808-74289130	2	P4HA3
chr6:111172694-111177665	31	SLC16A10
chr15:42751973-42753247	2	TTBK2
chr18:12358669-12358943	0	AFG3L2
chr10:31831738-31861658	1	ARHGAP12
chr19:7462151-7464690	34	ARHGEF18
chr15:72686753-72731726	0	BBS4
chr12:4517060-4518880	63	C12orf4

chr11:74095227-74109152	0	C2CD3
chr15:40628069-40629371	3	CASC5
chr2:196656632-196669876	0	CCDC150
chr10:32565557-32635158	4	CCDC7
chr5:123545416-123575963	56	CSNK1G3
chr10:124989501-125039155	0	CTBP2
chr22:31758545-31765060	13	DEPDC5
chr1:62653724-62663130	13	DOCK7
chr18:49257009-49379758	25	DYM
chr11:103303092-103307831	0	DYNC2H1
chr3:5199591-5203149	3	EDEM1
chr17:79077429-79081073	27	ENGASE
chr5:112259228-112307490	4	EPB41L4A
chr9:95907077-95956013	0	ERCC6L2
chr1:92563641-92625934	0	EVI5
chr11:47734024-47736740	3	FNBP4
chr14:65561336-65561766	168	FUT8
chr9:86077411-86079341	24	GOLM1
chr5:141627892-141630125	2	HDAC3
chr10:67988662-68038169	0	HERC4
chr3:124769012-124848558	0	ITGB5
chr17:6628116-6635171	17	KIAA0753
chr2:61081451-61088468	4	KIAA1841
chr16:87726764-87762040	5	KLHDC4
chr18:6093354-6264038	0	L3MBTL4
chr15:42545076-42548456	0	LRRC57
chr18:58741864-58745791	0	MALT1
chr4:139351190-139354098	13	NAA15
chrX:152635881-152639794	116	nogene
chr15:68068340-68068497	15	nogene
chr3:17622647-17733460	31	nogene
chr5:171024490-171034980	10	nogene
chr11:84902079-84903098	0	nogene
chr5:93553760-93563460	1	NR2F1-AS1
chr1:52383419-52393803	0	ORC1
chr13:100301459-100307260	0	PCCA
chr14:70976941-70988699	0	PCNX
chr1:28407409-28459258	2	PHACTR4
chrX:37386598-37426000	161	PRRG1
chr18:12718516-12724619	0	PSMG2
chr6:57193841-57210445	469	RAB23
chr11:73707419-73720899	556	RAB6A
chr1:174219124-174231355	10	RABGAP1L

chr1:178808044-178821704	0	RALGPS2
chr17:1878992-1883944	4	RPA1
chr10:16752538-16764510	36	RSU1
chr14:30670255-30673997	4	SCFD1
chr12:51684173-51699791	0	SCN8A
chr17:80223179-80237594	0	SLC26A11
chr5:35628459-35649425	0	SPEF2
chr17:49611279-49613605	0	SPOP
chr5:33453288-33459861	4	TARS
chr10:94507266-94511654	9	TBC1D12
chr15:51766372-51808544	0	TMOD2
chr9:35295695-35295930	0	UNC13B
chr2:61220309-61223296	17	USP34
chr10:1084402-1086357	16	WDR37
chr20:21349388-21365704	18	XRN2
chr9:37260454-37305714	0	ZCCHC7
chr9:37126311-37127263	56	ZCCHC7
chr8:144777457-144778525	0	ZNF34
chr19:57819567-57839149	0	ZNF587B
chr15:72760406-72775097	176	ADPGK
chr2:235908737-235930923	34	AGAP1
chr7:99333324-99338285	13	ARPC1A
chr1:161846448-161863312	73	ATF6
chr2:233263129-233265143	2	ATG16L1
chr18:31630958-31638760	2	B4GALT6
chr12:24842081-24849949	16	BCAT1
chr21:39228499-39229436	64	BRWD1
chr14:93293857-93296157	239	BTBD7
chr5:37153739-37162566	6	C5orf42
chr11:95799231-95813589	29	CEP57
chr8:25407982-25408940	149	DOCK5
chr15:69210315-69261329	19	GLCE
chr3:185689354-185689627	23	IGF2BP2
chr19:49139667-49143004	1	PPFIA3
chr7:66775226-66805396	6	RABGEF1
chr1:168231513-168243069	37	SFT2D2
chr11:70659827-70698763	11	SHANK2
chr10:68487143-68503695	12	SLC25A16
chr9:123169901-123179227	11	STRBP
chr12:6333068-6334244	24	TNFRSF1A
chr5:14304460-14330900	16	TRIO
chr2:61220309-61232532	2	USP34
chr2:159248371-159259843	4	WDSUB1

chr1:35358924-35361789	378	ZMYM4
chr19:40336109-40336407	17	C19orf47
chr6:34646600-34649830	14	C6orf106
chr11:103303092-103399872	9	DYNC2H1
chr4:22364240-22402799	12	ADGRA3
chr8:67251298-67259926	9	ARFGEF1
chr2:224556294-224557856	21	CUL3
chr1:156785464-156785857	16	nogene
chr12:131764455-131778330	3	SFSWAP
chrX:155506897-155507134	2	TMLHE
chr5:65451474-65460338	274	ADAMTS6
chr11:103231259-103245374	1	DYNC2H1
chr3:44784844-44794426	70	KIF15
chr9:97645610-97653908	2	NCBP1
chr3:47736033-47772936	159	SMARCC1
chr17:37221723-37226452	14	ACACA
chr1:75739979-75750546	19	ACADM
chr12:48939654-48941188	562	ARF3
chrX:63655493-63678572	10	ARHGEF9
chr8:130168991-130187285	1	ASAP1
chr12:14423908-14438267	13	ATF7IP
chr18:57731626-57731829	54	ATP8B1
chr7:73469516-73470483	40	BAZ1B
chr12:101714089-101720254	27	CHPT1
chr3:33612000-33644903	3	CLASP2
chr5:180553385-180571432	3	CNOT6
chr17:17254858-17260474	3	COPS3
chr1:44308309-44319744	102	ERI3
chr9:5810010-5813035	2	ERMP1
chr14:35053277-35053451	38	FAM177A1
chr19:39883304-39896143	6	FCGBP
chrX:131783886-131785258	79	FIRRE
chr2:48359046-48366125	15	FOXN2
chrX:152189729-152208144	16	GABRA3
chr10:14848608-14855943	16	HSPA14
chr19:7184315-7184637	35	INSR
chr2:30459551-30568176	39	LCLAT1
chr12:12147365-12151038	6	LRP6
chr11:28211061-28296931	12	METTL15
chr12:78116771-78119945	3	NAV3
chr13:35040932-35045401	24	NBEA
chr4:102525511-102537956	327	NFKB1
chrX:100948917-100957528	1	nogene

chr1:178123055-178125501	136	nogene
chr17:38777686-38786920	42	PIP4K2B
chr17:60623520-60656841	11	PPM1D
chr3:128795359-128798069	15	RAB7A
chr2:151414822-151416901	2	RIF1
chr4:109462900-109481781	16	SEC24B
chr19:40582350-40583717	35	SHKBP1
chr7:17821508-17840000	4	SNX13
chr17:60345480-60377292	12	USP32
chr17:59764970-59773885	77	VMP1
chr12:132933034-132945797	21	ZNF605
chr1:114733731-114738072	47	CSDE1
chr19:1555052-1556075	19	nogene
chr2:63933672-63984019	16	VPS54
chr22:42831548-42835493	14	ARFGAP3
chr17:48835322-48841887	13	CALCOCO2
chr2:61873001-61883548	38	CCT4
chr9:17330631-17342444	4	CNTLN
chr7:156966269-156967092	135	NOM1
chr11:3731390-3735324	33	NUP98
chr14:102662322-102681978	12	RCOR1
chr15:75859877-75883424	23	UBE2Q2
chr15:44322964-44332078	34	CASC4
chr11:46754887-46760784	19	CKAP5
chr14:88744022-88754671	13	EML5
chr13:30208576-30283791	97	KATNAL1
chr4:150914194-150916738	16	LRBA
chr6:43774340-43778918	42	VEGFA
chr5:138418952-138419232	2	KDM3B
chr3:184936245-184971752	115	VPS8
chr7:99385701-99391253	1	ARPC1B
chr7:32548846-32554596	2	AVL9
chr19:17276494-17276909	340	BABAM1
chr10:86875866-86892229	39	BMPR1A
chr19:13913167-13913638	25	CC2D1A
chr7:6416626-6416921	20	DAGLB
chr20:18412645-18415449	26	DZANK1
chr12:6974338-6975378	16	EMG1
chr19:14046153-14046618	50	IL27RA
chr17:57257465-57262192	1	MSI2
chr19:3558631-3559091	1	nogene
chr17:18865468-18882683	39	PRPSAP2
chr6:87541917-87569590	14	RARS2

chr20:37198396-37210165	3	RPN2
chr9:89455887-89479825	14	SEMA4D
chr15:70682757-70684446	1	UACA
chr7:157220688-157223351	101	UBE3C
chr10:133395712-133402773	264	MTG1
chr6:147260614-147267167	10	STXBP5
chr6:57190392-57210445	20	RAB23
chr5:132883339-132888160	1	AFF4
chr7:92221901-92222978	8	KRIT1
chr11:47628952-47631087	2	MTCH2
chr6:44932652-45106006	5	SUPT3H
chr2:201016990-201045593	18	FAM126B
chr20:49072285-49072697	18	CSE1L
chr1:61694696-61709437	7	TM2D1
chr6:13579450-13600949	10	SIRT5
chr7:30323641-30366295	24	ZNRF2
chr12:70278131-70319364	41	CNOT2
chr18:50918109-50932360	31	ME2
chr12:50701619-50702005	26	nogene
chr7:24804314-24820238	1	OSBPL3
chr8:47849153-47849503	2	PRKDC
chr4:1904215-1935262	9	WHSC1
chr12:95485878-95504161	40	METAP2
chr20:63922061-63922857	2	nogene
chr4:105424195-105438036	3	PPA2
chr9:134401631-134409119	4	RXRA
chr16:15879803-15884205	45	FOPNL
chr15:72007825-72027793	158	MYO9A
chr2:152144887-152148300	10	STAM2
chr3:197119409-197142768	34	DLG1
chr15:90217438-90221765	43	SEMA4B
chr10:27093348-27098326	7	ANKRD26
chr9:83335515-83389708	18	FRMD3
chr22:47686199-47738576	2	LOC284930
chr5:142345415-142353583	6	LOC101926941
chr11:68793314-68812576	5	CPT1A
chr5:31406852-31410887	4	DROSHA
chr6:104771192-104772074	6	HACE1
chr16:25132401-25134290	1	LCMT1
chr1:45824432-45829581	1	MAST2
chr11:47625673-47631711	3	MTCH2
chr11:126182836-126183087	58	nogene
chr1:26942656-26943065	18	NUDC

chr2:218582970-218587695	17	RQCD1
chr22:40270121-40273600	11	TNRC6B
chr5:32390274-32397338	58	ZFR
chr9:77247258-77260224	19	VPS13A
chr2:69505568-69509460	13	AAK1
chr10:73378910-73380201	18	ANXA7
chr12:11722599-11752579	18	ETV6
chr15:40709024-40731344	58	RAD51
chr5:132927120-132937193	11	AFF4
chr6:24456497-24467274	1	GPLD1
chr4:159186641-159210583	24	nogene
chr20:35714184-35732135	12	RBM39
chr4:183693588-183701808	1	TRAPPC11
chr5:40728344-40771863	2	TTC33
chr15:63553714-63574403	21	USP3
chr7:6412810-6416921	128	DAGLB
chr1:180413365-180495525	12	ACBD6
chr11:103116569-103121496	16	DYNC2H1
chr7:6466070-6469754	38	KDELR2
chr16:74938149-74956610	16	WDR59
chr6:532468-576882	30	EXOC2
chr17:58955648-58972925	6	PPM1E
chr12:120199117-120199485	3	RPLP0
chr17:60345480-60380399	18	USP32
chr1:155239615-155240077	45	GBA
chr17:16158759-16171995	13	NCOR1
chr2:232014834-232030080	290	DIS3L2
chr1:227193785-227317246	7	CDC42BPA
chr17:4242255-4242448	27	ANKFY1
chr8:94534838-94538346	2	KIAA1429
chr4:6372522-6381094	4	PPP2R2C
chr4:51874270-51899378	23	DCUN1D4
chr2:32439507-32453942	33	BIRC6
chr11:11998695-12002437	2	DKK3
chr1:38998170-39001106	39	AKIRIN1
chr14:76176622-76195972	55	GPATCH2L
chr5:80678926-80752256	7	MSH3
chr12:26982772-26990627	5	TM7SF3
chr12:26995236-27003390	62	TM7SF3
chr10:102473569-102474012	21	TMEM180
chr3:183724418-183728851	5	YEATS2
chr9:69046384-69053260	18	FXN
chr19:6039979-6047504	2	RFX2

chr19:57861375-57863260	2	nogene
chr1:244989113-245017326	10	EFCAB2
chr19:49488836-49489355	29	nogene
chr8:98693136-98707311	18	STK3
chr4:2881897-2894731	8	ADD1
chr10:68465666-68468306	26	DNA2
chr7:30323641-30362456	2	ZNRF2
chr8:130060578-130061069	25	ASAP1
chr17:43099774-43115779	31	BRCA1
chr1:222990624-222992110	16	DISP1
chr16:47428803-47459175	52	ITFG1
chr1:226277774-226287797	12	LIN9
chr17:50742047-50743243	12	nogene
chr14:103702073-103704256	13	nogene
chr22:29606237-29606764	18	nogene
chr19:39417554-39418105	3	PLEKHG2
chr1:150949121-150949525	11	SETDB1
chr3:4410864-4420146	62	SUMF1
chr10:12081471-12120267	118	DHTKD1
chr7:2369471-2371849	25	EIF3B
chr22:40349831-40354327	41	ADSL
chr7:65964322-65970366	3	GUSB
chr15:28141431-28141846	14	HERC2
chr8:71962922-71973659	13	MSC-AS1
chr11:47422573-47422973	148	PSMC3
chr17:1091664-1100735	7	ABR
chr17:37149863-37162050	14	ACACA
chr1:35816881-35832185	0	AGO4
chr1:246885492-246888517	7	AHCTF1
chr20:58519503-58523806	2	APCDD1L-AS1
chr15:34915037-34932434	0	AQR
chr6:129611532-129642018	0	ARHGAP18
chr2:231206197-231296253	3	ARMC9
chr2:241651009-241659187	12	ATG4B
chr12:69589484-69592159	202	CCT2
chr17:63813227-63813454	0	DDX42
chr14:74885585-74894409	0	DLST
chr1:97573759-97595166	3	DPYD
chr17:47402131-47429961	3	EFCAB13
chr10:15796099-15847943	49	FAM188A
chr5:171891466-171997051	1	FBXW11
chr16:2896351-2899231	2	FLYWCH2
chr3:172352802-172353083	0	FNDC3B

chr6:41772634-41778176	1	FRS3
chr3:158654546-158682157	0	GFM1
chr7:18654762-18666476	0	HDAC9
chr14:31168297-31172122	21	HECTD1
chr15:28280067-28321464	2	HERC2
chr6:82181878-82196401	0	IBTK
chr20:34438473-34442303	12	ITCH
chr7:23123776-23144025	24	KLHL7
chr5:146160373-146172774	16	LARS
chr4:150588047-150817257	0	LRBA
chrX:77857356-77870925	0	MAGT1
chr17:51218929-51225209	26	MBTD1
chr3:179592643-179602797	16	MRPL47
chr10:68109604-68122340	18	MYPN
chrX:63259667-63315853	2	nogene
chr1:247920424-247923492	0	nogene
chr6:83017299-83037549	0	nogene
chr3:42087103-42087400	48	nogene
chr22:23923865-23924497	0	nogene
chr7:84793809-84889860	2	nogene
chr19:7908369-7909048	23	nogene
chr15:49318110-49328130	0	nogene
chr5:179676400-179677544	0	nogene
chr19:36154703-36154939	0	nogene
chr3:178908521-178921910	2	nogene
chr17:47601607-47605552	2	NPEPPS
chr22:30741160-30753153	0	OSBP2
chr1:149965776-149967521	0	OTUD7B
chrX:111123076-111152447	0	PAK3
chr11:74346731-74368575	0	PGM2L1
chr16:47497398-47547548	394	PHKB
chr4:184652022-184666064	0	PRIMPOL
chr8:140789473-140890769	202	PTK2
chr9:107306378-107318879	9	RAD23B
chr3:141529707-141577106	0	RASA2
chr1:8508626-8614686	42	RERE
chr4:82880798-82881940	0	SEC31A
chr15:43793083-43793689	0	SERF2-C15ORF63
chrX:19683822-19746441	0	SH3KBP1
chr22:23791755-23825415	0	SMARCB1
chr17:64598381-64626595	0	SMURF2
chr2:45579874-45585774	0	SRBD1
chr8:123077110-123093742	34	TBC1D31

chr14:90680471-90786328	0	TTC7B
chr1:52423867-52435465	0	ZCCHC11
chr19:52076043-52077084	35	ZNF841
chr7:2363631-2364529	309	EIF3B
chr17:9491828-9557528	16	STX8
chr20:31792734-31794548	68	TPX2
chr12:96311051-96334865	92	CDK17
chr22:38494629-38495046	7	DDX17
chr8:42950387-42997637	16	HOOK3
chr4:6804522-6824684	18	KIAA0232
chr10:119160438-119160705	17	nogene
chr13:95757643-95760798	16	DNAJC3
chr20:4932454-4970919	32	SLC23A2
chr6:85528261-85542043	12	SNX14
chr19:1218416-1223172	4	STK11
chr15:63532646-63574403	25	USP3
chr4:84896910-84912319	23	WDFY3
chr3:64018598-64022523	14	PSMD6
chr1:214464629-214464957	54	PTPN14
chr9:33060464-33068934	2	SMU1
chr14:69115667-69122360	14	DCAF5
chr7:32632542-32639365	20	DPY19L1P1
chr17:68209653-68210465	13	nogene
chr15:56156515-56162240	24	nogene
chr10:1072115-1080476	287	WDR37
chr2:32595033-32611582	25	BIRC6
chr16:20802795-20803670	3	ERI2
chr9:33592912-33594308	13	nogene
chr4:1184266-1184627	14	nogene
chr2:97265915-97268003	19	nogene
chr1:233177801-233208689	167	PCNXL2
chrX:19456912-19464406	104	MAP3K15
chr1:179347599-179351462	17	SOAT1
chr2:241581011-241606473	1	THAP4
chr8:53993667-54010492	278	TCEA1
chr7:103194976-103199965	6	nogene
chr21:34834409-34859578	119	RUNX1
chr1:224952669-224967583	27	DNAH14
chr7:6469595-6482589	25	KDELR2
chr21:36338771-36344707	597	MORC3
chr20:48951322-48953790	46	ARFGEF2
chr10:74977315-74989112	13	KAT6B
chr3:160521778-160531557	25	KPNA4

chr20:61997549-62014707	49	TAF4
chrX:77557450-77595785	28	ATRX
chr2:155698966-155704210	6	nogene
chr6:20479845-20481425	1	E2F3
chr18:36353568-36355645	13	FHOD3
chr19:8455404-8463686	136	HNRNPM
chr3:38252392-38252789	18	OXSR1
chr16:89098590-89114487	30	ACSF3
chr10:27935009-27944962	20	ARMC4
chr10:121836717-121870038	31	ATE1
chr12:111658735-111681835	2	BRAP
chr9:19286768-19305527	29	DENND4C
chrX:147932424-147932763	31	FMR1
chr15:78470531-78478397	2	IREB2
chrX:10566887-10567603	64	MID1
chr9:122291748-122322745	2	MRRF
chr14:31703116-31704138	54	nogene
chr2:158603069-158634289	110	PKP4
chr2:168063499-168075231	628	STK39
chr3:100294844-100296275	2	TBC1D23
chr19:37583970-37586745	36	ZNF571
chr4:88419301-88420428	21	nogene
chr17:41902489-41903901	8	nogene
chr20:36684412-36688784	40	NDRG3
chr2:120278617-120289757	53	RALB
chr1:171783739-171794527	11	METTL13
chr13:24441845-24452593	17	PARP4
chr17:38395308-38395998	6	SOCS7
chr5:33750336-33751548	5	ADAMTS12
chr7:92319329-92352642	17	ANKIB1
chr5:173163724-173164296	19	BNIP1
chr5:69300011-69311204	16	CCDC125
chr10:127106230-127127764	24	DOCK1
chr19:11245562-11245878	5	DOCK6
chr1:155214835-155235311	1	GBA
chr7:102399547-102407498	49	LOC100630923
chr21:14853273-14857708	102	nogene
chr17:41828789-41832477	34	NT5C3B
chr10:94258799-94259150	3	PLCE1
chr12:89491935-89497342	29	POC1B
chr16:11841694-11847806	51	RSL1D1
chr6:145934906-145935441	5	SHPRH
chr3:155828181-155834041	17	SLC33A1

chr9:88415606-88426591	21	SPIN1
chr12:123586746-123590449	602	TMED2
chr2:46974972-46978907	67	TTC7A
chr1:154225083-154246375	3	UBAP2L
chr5:157751389-157755192	12	LSM11
chr12:131764175-131778330	1	SFSWAP
chr7:55982209-55997449	12	GBAS
chr19:10446217-10449150	23	PDE4A
chr17:783082-788132	17	RNMTL1
chr4:39075964-39103511	1	KLHL5
chr19:36761642-36762675	4	ZNF850
chr2:23798137-23834078	63	ATAD2B
chr22:19375630-19410778	3	HIRA
chr20:17943109-17954117	15	SNX5
chr4:169580841-169602678	26	NEK1
chr21:42883605-42886959	3	nogene
chr6:142166227-142170421	72	VTA1
chr12:57535055-57535845	10	DCTN2
chr18:21383509-21403994	20	GREB1L
chr5:177191883-177212195	30	NSD1
chr8:47785112-47798397	7	PRKDC
chr14:75745094-75752955	13	TTLL5
chr20:35347163-35384133	40	UQCC1
chr3:49324701-49327816	31	USP4
chr17:49732279-49732720	6	FAM117A
chr18:58341037-58357252	4	NEDD4L
chr2:171082780-171117857	10	TLK1
chr3:183756527-183762279	68	YEATS2
chr3:52688108-52689206	25	GNL3
chr10:5773841-5786050	30	GDI2
chr2:61347869-61350693	27	USP34
chr9:33943419-33948587	14	UBAP2
chr21:39215236-39218660	33	BRWD1
chr15:77467094-77471814	80	HMG20A
chr10:45862985-45900426	150	PARGP1
chr7:67305986-67309715	32	STAG3L4
chr1:97691716-97740479	3	DPYD
chr1:39287285-39287562	2	MACF1
chr1:31915890-31919658	55	PTP4A2
chr8:140743229-140761262	1	PTK2
chr2:55254040-55262427	1	MTIF2
chr20:38524777-38532859	13	RALGAPB
chr22:50703859-50715753	14	SHANK3

chr12:99772921-99782097	51	ANKS1B
chr5:111344023-111394782	16	CAMK4
chr10:15132816-15141557	3	NMT2
chr11:74965033-74969699	185	SPCS2
chr4:5849391-5861210	3	CRMP1
chr7:123689041-123692867	3	WASL
chr15:90439331-90456315	6	IQGAP1
chr7:65752163-65756574	223	CCT6P1
chr11:43798319-43840064	19	HSD17B12
chr20:35913248-35917662	3	PHF20
chr5:179716104-179720560	4	CANX
chr7:104188250-104204234	56	ORC5
chr20:33679754-33680416	46	E2F1
chr17:65743067-65750724	5	CEP112
chr1:77801330-77875111	4	FAM73A
chr16:48252130-48262872	11	LONP2
chr6:112912876-112921171	8	nogene
chr7:100351251-100355240	2	STAG3L5P-PVRIG2P-PILRB
chr19:1430221-1432690	12	DAZAP1
chr16:24952288-24970580	13	ARHGAP17
chr5:151786571-151804446	15	G3BP1
chr20:25413789-25425327	156	GINS1
chr1:223954476-223962708	34	GTF2IP20
chr7:8218304-8235940	4	ICA1
chr4:82930942-82946474	13	LIN54
chr6:150944485-150972058	3	MTHFD1L
chr2:63968956-63984019	11	VPS54
chr20:19990005-19992299	34	RIN2
chr22:41459940-41460487	6	PHF5A
chr17:2700854-2701139	58	CLUH
chr16:67626554-67629533	26	CTCF
chr18:45907957-45912456	2	EPG5
chr22:23683724-23695517	17	GUSBP11
chr12:62498918-62501698	32	MON2
chr12:85410132-85424204	10	nogene
chr15:30582893-30641617	19	nogene
chr8:125357218-125357818	68	NSMCE2
chr14:22909482-22911403	689	RBM23
chr14:67685014-67692593	26	RDH11
chr8:123077110-123105464	277	TBC1D31
chr10:45624582-45627105	8	ZFAND4
chr12:122352725-122355312	4	CLIP1
chr3:81288078-81325698	12	nogene

chr3:52916149-52928341	1	SFMBT1
chr8:73527690-73552319	17	STAU2
chr9:109057190-109064567	75	TMEM245
chr19:1271328-1274440	9	CIRBP
chr4:36582656-36584796	24	nogene
chr12:45945248-45951727	4	SCAF11
chr3:72792915-72844423	23	SHQ1
chr6:110879986-110890356	29	AMD1
chr3:197980325-197996282	1	LMLN
chr3:49967469-49999513	23	RBM6
chr14:89924512-89932627	30	EFCAB11
chr6:32971594-32972927	17	BRD2
chr17:61712449-61712722	12	nogene
chr11:77991659-77992462	13	nogene
chr9:111570090-111586165	91	PTGR1
chr5:151060317-151063747	30	TNIP1
chr13:29530537-29536202	2186	SLC7A1
chr15:90713142-90715042	34	nogene
chr2:135932782-135961498	17	DARS
chr15:76283756-76292700	79	ETFA
chr20:51689063-51697482	33	ATP9A
chr12:96323947-96395917	13	CDK17
chr5:116091078-116134178	39	COMMD10
chr2:37019808-37020836	12	HEATR5B
chr6:118826146-118856545	14	MCM9
chr14:37266937-37270525	13	MIPOL1
chr12:110485077-110490171	16	FAM216A
chr7:2068666-2069338	13	MAD1L1
chr22:40953554-40955446	12	RBX1
chr20:48941198-48963898	5	ARFGEF2
chr12:79592610-79621207	12	PAWR
chr1:243645892-243843282	23	AKT3
chr12:103806321-103815121	27	NT5DC3
chr7:66797373-66805396	455	RABGEF1
chr15:68209636-68218650	28	CLN6
chr5:157803649-157817547	20	CLINT1
chrX:49115725-49117810	12	GPKOW
chr12:76805316-76822531	14	ZDHHC17
chr2:169872235-169878574	84	UBR3
chr19:47357865-47362693	18	DHX34
chr19:10143765-10146524	14	DNMT1
chr8:67299208-67301380	37	ARFGEF1
chr8:65622262-65627433	15	ARMC1

chr21:33421479-33432871	29	IFNGR2
chr5:138372673-138379708	60	KDM3B
chr10:21681331-21713950	4	MLLT10
chr17:9162812-9182969	8	NTN1
chr13:19751270-19752316	21	PSPC1
chr1:43553491-43553941	43	PTPRF
chr18:12951854-12971251	27	SEH1L
chr15:75394679-75396496	15	SIN3A
chr12:46239678-46239893	7	SLC38A1
chr19:36089037-36089306	13	WDR62
chr11:74921213-74933872	2	XRRA1
chr1:213129526-213167973	1	RPS6KC1
chr19:9652952-9653878	16	ZNF562
chr10:119853049-119859884	17	MCMBP
chr7:33351218-33388144	30	BBS9
chr17:66139485-66183307	3	CEP112
chr4:118331715-118338314	455	PRSS12
chr12:100204939-100205744	11	ACTR6
chr16:69155870-69167185	217	CIRH1A
chr1:167974829-168004793	2	DCAF6
chr14:103448918-103457212	11	MARK3
chrX:21851508-21853503	136	MBTPS2
chr3:15411244-15414162	11	METTL6
chr8:97661071-97706750	14	MTDH
chr7:158671513-158675656	14	NCAPG2
chr13:72835242-72931267	8	PIBF1
chr9:111578795-111586165	130	PTGR1
chr19:5941620-5951596	3	RANBP3
chr3:158122102-158146775	26	RSRC1
chr18:2694526-2698041	16	SMCHD1
chr17:48118703-48119186	8	SNX11
chr8:73582769-73709162	2	STAU2
chr7:47344754-47346356	98	TNS3
chr14:90644047-90786328	16	TTC7B
chr4:148023262-148047051	60	ARHGAP10
chr11:36610080-36648155	17	C11orf74
chr4:67518155-67519502	19	CENPC
chr3:52693407-52694103	29	GNL3
chr4:87163478-87195690	15	KLHL8
chr18:9588088-9588853	67	PPP4R1
chr17:63604533-63606440	12	TACO1
chr2:271865-277372	1	ACP1
chr9:78241695-78243636	7	CEP78

chr1:26268482-26269614	67	CEP85
chr7:100687297-100687616	3	GIGYF1
chr2:197523623-197540437	2	MOB4
chr10:31373121-31387266	50	nogene
chr16:14552020-14593378	3	PARN
chr19:2213538-2214596	43	DOT1L
chr8:42950387-42974194	38	HOOK3
chr3:108554375-108560841	23	KIAA1524
chr4:3253206-3253482	13	MSANTD1
chr4:13460698-13479526	166	RAB28
chr1:63651668-63654466	13	PGM1
chr1:1669663-1734835	29	SLC35E2B
chr13:28662409-28672432	48	POMP
chr2:101281858-101294082	1	RNF149
chr21:43882503-43904019	3	AGPAT3
chr22:38495795-38499499	7	DDX17
chr14:61720381-61734285	2	HIF1A
chr7:79454885-79458999	2	MAGI2-AS3
chr10:49832802-49861663	3	PARG
chr19:418922-425231	7	SHC2
chr1:15872815-15876678	68	SPEN
chr16:72950468-72950965	32	ZFHX3
chr19:13927892-13928188	33	CC2D1A
chr18:74877196-74920692	25	ZNF407
chr7:102877466-102884312	233	FBXL13
chr1:39763091-39764817	8	BMP8B
chr12:14540763-14553412	23	PLBD1
chr2:85555483-85556260	14	GGCX
chr2:37236095-37237867	46	NDUFAF7
chr1:19890384-19907876	11	OTUD3
chr19:13923332-13923811	49	CC2D1A
chr5:168559031-168566266	36	PANK3
chr12:98595432-98595661	16	SLC25A3
chrX:23713134-23730564	18	ACOT9
chr10:110100624-110122292	11	ADD3
chr2:27058399-27059404	24	AGBL5
chr11:46433473-46508370	17	AMBRA1
chr15:68787412-68787919	1	ANP32A
chr5:142885297-142913293	24	ARHGAP26
chr12:26922615-26928905	16	ASUN
chr3:11331339-11348035	16	ATG7
chr2:32491424-32493667	35	BIRC6
chr2:27236283-27236830	12	CAD

chr12:121268637-121274585	9	CAMKK2
chr6:89854816-89857199	32	CASP8AP2
chr5:96722638-96730860	21	CAST
chr20:33619516-33640531	2	CBFA2T2
chr2:36369107-36442735	43	CRIM1
chr12:113178976-113181058	1	DDX54
chr9:123452275-123457897	13	DENND1A
chr2:48346200-48362707	47	FOXN2
chr2:232787149-232796221	107	GIGYF2
chr3:149063430-149071657	19	HLTF
chr15:41016087-41027736	11	INO80
chr13:51383328-51395483	1	INTS6
chr1:32030297-32038619	48	KHDRBS1
chr7:105062163-105081797	84	KMT2E
chr7:74105874-74106243	2	LIMK1
chr16:48252130-48258740	16	LONP2
chr15:67563282-67586913	1	MAP2K5
chr14:21500494-21501903	61	METTL3
chr2:227328677-227330846	9	MFF
chr17:4547957-4550353	1	MYBBP1A
chr2:203056426-203057453	8	NBEAL1
chr3:38183002-38230430	13	OXSR1
chr15:85067013-85083644	146	PDE8A
chr4:39902346-39922748	2	PDS5A
chr6:136825213-136870003	2	PEX7
chr17:78414878-78419725	43	PGS1
chrX:15455847-15459740	18	PIR
chr2:53895006-53901559	11	PSME4
chr11:66639699-66644140	15	RBM4
chr17:1842802-1844686	167	RPA1
chr7:80789305-80805758	120	SEMA3C
chr10:100979847-100981229	6	SEMA4G
chr2:200419249-200419496	54	SPATS2L
chr11:64193077-64197974	35	STIP1
chr2:168129540-168182090	12	STK39
chr18:26274662-26275053	1	TAF4B
chr10:45982522-45988847	74	TIMM23
chr8:140275657-140291078	52	TRAPPC9
chr1:1256044-1257310	21	UBE2J2
chr10:11525385-11597717	15	USP6NL
chr16:31090774-31091342	596	VKORC1
chr8:99502835-99511512	65	VPS13B
chr14:96846094-96856586	32	VRK1

chr1:52974768-52988136	18	SCP2
chr11:108097635-108098529	19	CUL5
chr20:34489265-34492597	92	ITCH
chr19:6453980-6454717	6	SLC25A23
chr8:10207832-10319989	2	MSRA
chr4:70762518-70764576	31	RUFY3
chr8:47396375-47440542	33	SPIDR
chr9:131643238-131650949	92	RAPGEF1
chr1:77873000-77875111	35	FAM73A
chr20:21214534-21232830	31	KIZ
chr2:214752446-214792445	35	BARD1
chr6:149512154-149517450	4	PPIL4
chr7:55163732-55165437	9	EGFR
chr16:13242389-13244460	9	nogene
chr14:34575755-34609742	76	SNX6
chr11:43403997-43407577	25	TTC17
chr22:40907586-40914324	11	XPNPEP3
chr6:10697323-10702639	23	PAK1IP1
chr11:67393495-67393790	12	RAD9A
chr16:30716065-30716479	15	SRCAP
chr1:180824752-180825344	270	XPR1
chr2:111822421-111825855	19	ANAPC1
chr2:174846879-174878128	12	CHN1
chr5:131677702-131699002	33	FNIP1
chr10:124484138-124498128	4	LHPP
chr16:910930-954666	72	LMF1
chr4:150798080-150808398	64	LRBA
chr1:222651976-222652332	33	MIA3
chr1:5978269-5986327	21	NPHP4
chr1:43553491-43569778	105	PTPRF
chr15:44606024-44608605	2	SPG11
chr1:9355742-9356585	10	SPSB1
chr9:98099930-98100164	12	TRIM14
chr7:33349067-33388144	53	BBS9
chr10:49530719-49532978	1	ERCC6-PGBD3
chr5:133064979-133074126	64	HSPA4
chr4:3086938-3116263	41	HTT
chr20:35899369-35917662	2	PHF20
chr8:132811045-132817545	1	PHF20L1
chr8:140686631-140717709	24	PTK2
chr4:42018110-42023384	105	SLC30A9
chr6:35874232-35891013	43	SRPK1
chr6:24652982-24654530	4	TDP2

chr10:38012297-38017386	16	ZNF33A
chr17:49298105-49312042	1	ZNF652
chr2:43397970-43430302	45	THADA
chr12:103979830-103980962	9	TDG
chr5:65926581-65994864	29	ERBB2IP
chr1:50490587-50535457	33	FAF1
chr6:38078039-38084991	8	nogene
chr14:102084726-102085425	19	nogene
chr10:69185986-69191766	82	SUPV3L1
chr19:9656546-9660874	138	ZNF562
chr4:159304341-159314768	24	RAPGEF2
chr7:32833007-32834919	45	nogene
chr1:23055068-23057565	6	KDM1A
chr11:76458182-76464080	12	C11orf30
chr10:74394140-74600493	2	ADK
chr9:33528726-33536945	26	ANKRD18B
chr11:32588593-32589641	40	EIF3M
chr1:155853275-155853806	1107	GON4L
chr11:123081862-123082661	13	nogene
chr9:97185441-97194840	29	nogene
chr15:71991102-72010447	25	MYO9A
chr16:70258032-70259186	12	AARS
chr17:37111530-37113265	3	ACACA
chr12:109249983-109254334	0	ACACB
chr8:39077227-39091346	12	ADAM9
chr5:65409518-65452918	0	ADAMTS6
chr17:78206910-78207257	0	AFMID
chr6:87675859-87681763	16	AKIRIN2
chr1:243545509-243695716	10	AKT3
chr5:115852978-115870128	23	AP3S1
chr21:25997359-26053348	0	APP
chr14:74683295-74701774	17	AREL1
chr4:147955315-147966839	27	ARHGAP10
chr10:31826303-31861658	12	ARHGAP12
chr13:111205288-111217880	9	ARHGEF7
chr6:157036834-157167185	2	ARID1B
chr3:48979481-48982979	0	ARIH2
chr2:9279316-9297445	260	ASAP2
chr12:14446987-14457295	26	ATF7IP
chr11:108319953-108321420	13	ATM
chr4:47535508-47546862	4	ATP10D
chr3:194438855-194448009	2	ATP13A3
chr18:79126266-79253541	11	ATP9B

chr16:309972-347106	17	AXIN1
chr16:289439-293718	5	AXIN1
chr17:60679452-60689761	13	BCAS3
chr11:102350843-102368548	21	BIRC2
chr2:32467524-32467739	0	BIRC6
chr11:76513385-76528466	148	C11orf30
chr9:94923985-94955279	0	C9orf3
chr9:26861065-26886188	0	CAAP1
chr17:40161752-40162901	7	CASC3
chr6:110210706-110219869	0	CDC40
chr17:40300827-40301608	0	CDC6
chr2:37216983-37222563	0	CEBPZ
chr9:92379762-92380913	0	CENPP
chr1:111159379-111161296	13	CEPT1
chr3:113395749-113416627	17	CFAP44
chr19:4405911-4418076	8	CHAF1A
chr11:46750273-46751534	4	CKAP5
chr2:121503166-121530325	200	CLASP1
chr16:58520938-58525359	0	CNOT1
chr19:54142928-54144332	2	CNOT3
chr11:36276136-36280837	1	COMMD9
chr2:36476888-36479694	40	CRIM1
chr20:49088008-49091022	11	CSE1L
chr15:64203081-64207594	32	CSNK1G1
chr15:64251511-64300723	16	CSNK1G1
chr1:84563256-84570720	2	CTBS
chr16:67616744-67621591	20	CTCF
chr11:70417012-70431280	0	CTTN
chr11:70422939-70431280	27	CTTN
chr8:123022683-123030716	12	DERL1
chr22:19064847-19089490	0	DGCR2
chr22:19056985-19089490	0	DGCR2
chr7:157358546-157410001	0	DNAJB6
chr19:10775702-10777216	2	DNM2
chr14:72714422-72771893	11	DPF3
chr15:65463760-65480399	11	DPP8
chr7:34966893-34989941	3	DPY19L1
chr1:97234851-97248050	0	DPYD
chr9:137815946-137818138	14	EHMT1
chr22:37855564-37863345	0	EIF3L
chr6:53275089-53295707	11	ELOVL5
chr14:88754511-88754671	14	EML5
chr17:19309884-19329647	55	EPN2

chr12:1289851-1351501	0	ERC1
chr7:158749648-158798076	2	ESYT2
chr7:148832633-148847305	139	EZH2
chr7:22983943-22991139	65	FAM126A
chr5:10140030-10227647	0	FAM173B
chr3:10032831-10036339	43	FANCD2
chr7:102813276-102931933	0	FBXL13
chr12:48923781-48926223	0	FKBP11
chr1:99740792-99742273	0	FRRS1
chr9:105484380-105539351	4	FSD1L
chr5:154323781-154329738	0	GALNT10
chr1:117902616-117918736	0	GDAP2
chr5:103096540-103097781	60	GIN1
chr3:142171418-142177576	0	GK5
chr6:1959866-2117556	21	GMDS
chr2:144208602-144248697	1	GTDC1
chr22:23683724-23714317	0	GUSBP11
chr1:108650210-108659962	11	HENMT1
chr8:28963810-28980156	20	HMBOX1
chr1:20749722-20749975	36	HP1BP3
chr10:119781634-119811955	0	INPP5F
chr6:33688238-33688431	0	ITPR3
chr21:33750142-33766012	6	ITSN1
chr6:73223698-73224348	0	KHDC1L
chr7:152251938-152273867	4	KMT2C
chr4:128074459-128091144	9	LARP1B
chr19:11100222-11107514	4	LDLR
chr19:54458313-54458521	27	LENG8
chr4:150806270-150817257	8	LRBA
chr3:197847408-197865471	1	LRCH3
chr7:134191654-134221918	11	LRGUK
chr8:91102218-91133305	6	LRRC69
chr4:102722870-102723967	0	MANBA
chr15:67600684-67658614	13	MAP2K5
chr2:101864929-101870415	0	MAP4K4
chr5:139307238-139308327	46	MATR3
chr6:167870382-167880517	14	MLLT4
chrX:106980890-106985250	120	MORC4
chr12:123218375-123223127	13	MPHOSPH9
chr1:167772274-167776166	145	MPZL1
chr17:40126182-40126406	26	MSL1
chrX:150727214-150737448	34	MTMR1
chr8:66572481-66573506	28	MYBL1

chr20:23390208-23403072	0	NAPB
chr2:15488893-15558634	3	NBAS
chr8:89980733-89982855	1	NBN
chr12:124426621-124430787	13	NCOR2
chr11:67608551-67609635	64	NDUFV1
chr2:69415184-69423717	0	NFU1
chr3:23900807-23911450	57	NKIRAS1
chr1:45689163-45689588	16	nogene
chr12:109106724-109107359	0	nogene
chr12:85342471-85424204	10	nogene
chr2:39179186-39229588	7	nogene
chr7:2254251-2254601	0	nogene
chr4:84879405-84898597	11	nogene
chr5:146237918-146238273	15	nogene
chr22:28514756-28545577	12	nogene
chr5:172244959-172245498	0	nogene
chr3:67094831-67096603	12	nogene
chr12:1506066-1512004	0	nogene
chr7:66889134-66889521	2	nogene
chr6:141882660-141910011	0	nogene
chr13:26592428-26593722	0	nogene
chr8:125147080-125195272	28	nogene
chr14:34647791-34648139	19	nogene
chr1:26261039-26261205	17	nogene
chr12:102324786-102330442	0	nogene
chr1:222594235-222599969	0	nogene
chr22:42149278-42153200	0	nogene
chr14:55354500-55355222	25	nogene
chr1:151202704-151203301	12	nogene
chr6:2283498-2283763	3	nogene
chr10:34306571-34306996	20	nogene
chr22:17705180-17705392	11	nogene
chr17:66327827-66328092	0	nogene
chr22:20993105-20993319	0	nogene
chr6:34606629-34646792	0	nogene
chr16:72308055-72369679	0	nogene
chr10:123361318-123389292	0	nogene
chr1:200364201-200374246	0	nogene
chrX:23700354-23700851	0	nogene
chr8:124492205-124492560	14	nogene
chr12:6936921-6937167	0	nogene
chr9:130204977-130205447	0	nogene
chr1:5961793-5986327	252	NPHP4

chr7:33021271-33024108	0	NT5C3A
chr7:135592986-135594729	18	NUP205
chr1:154046068-154061674	0	NUP210L
chr17:75231339-75231979	0	NUP85
chr16:46693092-46696085	0	ORC6
chr22:42890946-42893613	2	PACSIN2
chr5:103019789-103028238	0	PAM
chr5:79645122-79669028	19	PAPD4
chr14:96520054-96521072	102	PAPOLA
chr11:12473744-12477949	25	PARVA
chr3:101579841-101585511	2	PCNP
chr2:10784933-10793202	0	PDIA6
chr10:95238567-95247366	0	PDLIM1
chr15:77133004-77133750	45	PEAK1
chr20:35842572-35858381	40	PHF20
chr17:28559575-28560191	0	PIGS
chr12:57594024-57601636	4	PIP4K2C
chr3:146104278-146124229	0	PLOD2
chr12:89459637-89472275	178	POC1B
chr14:103784794-103797518	17	PPP1R13B
chr2:37274420-37293271	0	PRKD3
chr16:68321425-68329174	14	PRMT7
chr17:18911102-18918387	0	PRPSAP2
chr1:171587647-171588505	0	PRRC2C
chr12:121899633-121903107	4	PSMD9
chr8:140818276-140925749	87	PTK2
chr3:61748877-61769416	14	PTPRG
chr4:13425109-13460828	14	RAB28
chr9:123065347-123076762	3	RABGAP1
chr7:66783671-66799414	12	RABGEF1
chr5:171221757-171242820	26	RANBP17
chr5:131548046-131638060	2	RAPGEF6
chr6:87530783-87541994	0	RARS2
chr1:32668224-32680614	7	RBBP4
chr11:114402827-114405799	0	RBM7
chr17:32173830-32199031	12	RHOT1
chr5:38971876-38978650	6	RICTOR
chr15:59030803-59076215	10	RNF111
chr3:196471639-196475312	11	RNF168
chr7:5739275-5741815	144	RNF216
chr6:125045210-125058106	71	RNF217
chr12:56210296-56210568	16	RNF41
chr6:88844356-88891915	49	RNGTT

chr17:281767-327579	36	RPH3AL
chr3:134173115-134195182	2	RYK
chr2:199368604-199381820	62	SATB2
chr3:47435007-47443064	0	SCAP
chr1:41142877-41143112	14	SCMH1
chr7:80798091-80810701	27	SEMA3C
chr10:50340386-50344345	25	SGMS1
chr9:105273496-105361330	0	SLC44A1
chr3:27411641-27434104	5	SLC4A7
chr1:44008352-44008623	24	SLC6A9
chr13:29517572-29517790	15	SLC7A1
chr7:2269558-2278305	10	SNX8
chr1:109350928-109355469	11	SORT1
chr4:122979231-123057288	2	SPATA5
chr17:20204332-20232414	0	SPECC1
chr8:125003175-125009343	0	SQLE
chr4:56474053-56474391	19	SRP72
chr2:168063499-168140758	2	STK39
chr1:36361220-36362656	137	STK40
chr4:98109192-98134459	25	STPG2
chr16:31034012-31039651	0	STX4
chr10:102592581-102599544	18	SUFU
chr9:112124256-112157613	4	SUSD1
chr17:37437737-37444768	34	TADA2A
chr2:9882705-9919820	11	TAF1B
chr16:69022827-69040421	12	TANGO6
chr1:234420701-234430301	0	TARBP1
chr20:13616856-13630152	11	TASP1
chr3:10270130-10271015	6	TATDN2
chr3:100297922-100302237	1	TBC1D23
chr8:123120054-123126687	0	TBC1D31
chr3:196062581-196077122	0	TFRC
chr16:66517135-66517877	3	TK2
chr15:79321977-79411640	0	TMED3
chr4:38986335-39025726	0	TMEM156
chr15:51900154-51902036	28	TMOD3
chr1:223789007-223796590	0	TP53BP2
chr20:63869295-63875875	11	TPD52L2
chr19:58548987-58549240	0	TRIM28
chr10:114959725-114974385	15	TRUB1
chr3:150408179-150411308	0	TSC22D2
chr5:40746797-40747019	0	TTC33
chr18:24053313-24083081	0	TTC39C

chr19:34433347-34438766	11	UBA2
chr1:10072027-10072214	0	UBE4B
chr5:176943334-176951577	292	UIMC1
chr10:11956319-11964125	0	UPF2
chr20:35374183-35384133	37	UQCC1
chr2:61378362-61406128	0	USP34
chr1:21680807-21695221	17	USP48
chr17:8161607-8162369	0	VAMP2
chrX:155889457-155900587	36	VAMP7
chr17:59735337-59738947	0	VMP1
chr8:99501686-99521010	18	VPS13B
chr13:41575739-41605276	0	VWA8
chr4:84879405-84932363	15	WDFY3
chr2:19935470-19953978	0	WDR35
chr2:28912589-28929710	0	WDR43
chr16:69786995-69799295	26	WWP2
chr8:21966856-21976521	0	XPO7
chr11:74848114-74863021	0	XRRA1
chr11:102205892-102223752	13	YAP1
chr3:114380216-114500391	14	ZBTB20
chr19:47089966-47094608	0	ZC3H4
chr6:157628348-157653627	15	ZDHHC14
chr19:36360434-36362238	0	ZFP14
chr11:65085114-65087433	0	ZFPL1
chr7:6697338-6697811	95	ZNF12
chr18:76878008-76899222	0	ZNF236
chr9:111527267-111534353	145	ZNF483
chr19:9355656-9364948	13	ZNF559-ZNF177
chr2:71396140-71406262	9	ZNF638
chr19:36762304-36762675	10	ZNF850
chr12:133120287-133122024	0	ZNF891
chr3:5172149-5172740	16	ARL8B
chr7:152220522-152238826	242	KMT2C
chr6:10931439-10956242	4	SYCP2L
chr21:29351633-29411624	19	BACH1
chr7:102822039-102832974	1	FBXL13
chr12:116985205-117010450	32	FBXW8
chr6:33655765-33659549	1	ITPR3
chr16:27213584-27215008	63	KDM8
chr5:141494806-141505481	2	PCDHGA6
chr11:17959469-18010139	70	SERGEF
chr14:52997669-53043755	13	nogene
chr6:99545111-99562948	2	CCNC

chr8:27294079-27298559	7	TRIM35
chr7:2239417-2239707	7	FTSJ2
chr16:18782780-18783102	2	RPS15A
chr1:100439931-100468094	11	CDC14A
chr4:139342825-139344339	102	NAA15
chr6:132463992-132472375	11	STX7
chr5:126552020-126570859	11	ALDH7A1
chr10:12081471-12087729	18	DHTKD1
chr22:31458474-31463133	11	EIF4ENIF1
chr12:112239051-112240027	10	HECTD4
chr17:8520877-8521285	11	MYH10
chr3:171708754-171726076	2	PLD1
chr11:107803000-107806876	67	SLC35F2
chr8:123126055-123126687	11	TBC1D31
chr21:44037791-44052476	1	TRAPPC10
chr17:59031895-59047819	5	TRIM37
chr6:42644213-42650386	1	UBR2
chr3:196391812-196407393	126	UBXN7
chr4:84894824-84932363	1	WDFY3
chr1:156976982-156980486	15	ARHGEF11
chr17:4982756-4986286	20	CAMTA2
chr16:3504719-3508468	2	CLUAP1
chr22:46626750-46637960	69	GRAMD4
chr7:66382236-66390250	4	LINC00174
chr6:167870382-167898467	17	MLLT4
chr16:30580732-30580974	16	nogene
chr3:136316940-136329984	5	PCCB
chr8:15623079-15662296	18	TUSC3
chr8:42401787-42403461	141	VDAC3
chr2:61490641-61493053	20	XPO1
chr18:21383509-21384403	15	GREB1L
chr17:69273958-69284072	68	ABCA5
chr2:38309378-38319019	78	ATL2
chr21:39224407-39225197	7	BRWD1
chr16:58599235-58601867	1	CNOT1
chr15:66306896-66308844	12	DIS3L
chr17:39708320-39710481	163	ERBB2
chr7:55982209-55994988	8	GBAS
chr1:93894613-93901669	262	GCLM
chr4:44717112-44722242	20	GNPDA2
chr4:88649839-88658491	32	HERC3
chr18:6982496-7002385	1	LAMA1
chr3:16286323-16303592	29	nogene

chr2:200933875-200937966	4	ORC2
chr7:77768660-77773314	3	RSBN1L
chr12:813641-830160	118	WNK1
chr17:4066446-4086655	12	ZZEF1
chr16:66725827-66729099	63	DYNC1LI2
chr1:155795049-155827028	4	GON4L
chr11:78469246-78493195	112	NARS2
chr5:14487463-14488260	17	TRIO
chr12:104318912-104327671	19	TXNRD1
chr2:45476992-45553730	23	SRBD1
chr16:71678754-71681905	64	PHLPP2
chr2:32597750-32607643	2	BIRC6
chr5:69561033-69562042	23	nogene
chr3:144237757-144270349	35	nogene
chr12:124912962-124913190	710	nogene
chr10:119577079-119580010	2	TIAL1
chr19:52574076-52576021	7	ZNF701
chrX:68052539-68064177	20	OPHN1
chr20:33623114-33625017	29	CBFA2T2
chr1:59321535-59346398	118	FGGY
chr16:72249162-72349206	1	LINC01572
chr2:95150196-95159373	12	nogene
chr16:47463881-47596531	1	PHKB
chr20:49849578-49855581	33	SLC9A8
chr6:85530191-85536924	1	SNX14
chrX:9501779-9654322	1	TBL1X
chr2:43578512-43590954	93	THADA
chr16:84744632-84745673	3	USP10
chr6:24412221-24418578	4	MRS2
chr18:37066867-37084232	12	nogene
chr2:183128286-183130545	176	NUP35
chr12:128799258-128815070	38	SLC15A4
chr6:24502522-24522925	37	ALDH5A1
chr17:50990449-50993935	10	SPAG9
chr11:77938650-77981576	33	INTS4
chr20:50902016-50904849	3	ADNP
chr9:109093474-109108570	32	TMEM245
chr14:92007639-92011072	26	TRIP11
chr5:83195769-83204921	7	XRCC4
chr3:142355400-142370620	2	XRN1
chr2:175093060-175121540	43	ATF2
chr9:95978060-96012357	21	ERCC6L2
chr8:33388962-33389798	26	FUT10

chr15:34382796-34383053	34	GOLGA8A
chr5:119506817-119515046	57	HSD17B4
chr1:117402185-117442325	732	MAN1A2
chr8:97661071-97689103	3	MTDH
chr4:168890921-168916027	27	PALLD
chr20:64010199-64011503	3	PRPF6
chr1:10117458-10135186	2	UBE4B
chr21:39200218-39202545	11	BRWD1
chr11:119284968-119285566	173	CBL
chr9:95107065-95111637	12	FANCC
chr10:13666096-13670528	11	FRMD4A
chr16:50068779-50086351	11	HEATR3
chr5:5441111-5447897	2	ICE1
chr10:132690391-132710456	105	INPP5A
chr13:101567698-101583356	11	ITGBL1
chr21:36333645-36338921	19	MORC3
chr17:36512657-36515990	1	MYO19
chr12:112075677-112093136	29	NAA25
chr17:58696692-58720812	2	RAD51C
chr17:20096630-20110562	14	SPECC1
chr9:121989194-121989770	58	TTLL11
chr8:99134631-99193057	37	VPS13B
chr1:247138715-247159813	122	ZNF124
chr1:155438326-155462599	39	ASH1L
chr8:61618977-61653660	189	ASPH
chr5:179705678-179708380	18	CANX
chr22:37495516-37497178	29	CARD10
chrX:131749305-131756709	18	FIRRE
chr5:126469676-126477787	3	GRAMD3
chr2:31917925-31920910	60	MEMO1
chr7:128577952-128638897	12	nogene
chr17:78400713-78401106	12	nogene
chr17:66513335-66514466	5	nogene
chr3:134207471-134222539	19	RYK
chr2:101050400-101059539	12	TBC1D8
chr12:82965023-82986046	15	TMTC2
chr10:68338502-68341680	11	HNRNPH3
chr4:40144631-40154267	16	N4BP2
chr12:11047019-11133367	15	PRH1
chr12:6749847-6750766	107	MLF2
chr20:3854557-3857809	8	MAVS
chr20:35938696-35941047	2	PHF20
chr14:77720710-77723277	14	SNW1

chr1:35415465-35415714	4	ZMYM4
chr17:17866295-17907531	14	TOM1L2
chr1:179337836-179342943	28	SOAT1
chr20:48953555-48969331	1	ARFGEF2
chr10:27542752-27578713	3	nogene
chr22:41137652-41141222	15	EP300
chr14:23322180-23324289	7	PABPN1
chr7:579255-711527	12	PRKAR1B
chr3:31876432-31879830	141	OSBPL10
chr14:68115818-68118479	33	nogene
chrX:155506897-155524632	24	TMLHE
chr6:57152220-57161152	70	ZNF451
chr7:141601204-141621801	15	AGK
chr5:82164398-82178587	55	ATG10
chr11:116762552-116765446	2	BUD13
chr21:41912331-41922085	12	C2CD2
chr1:26255170-26255865	91	CEP85
chr18:47112976-47136449	5	HDHD2
chr2:43912431-43918398	122	LRPPRC
chr17:1479430-1479705	9	MYO1C
chr2:240005209-240014860	63	NDUFA10
chr17:47590716-47601747	13	NPEPPS
chr8:58623219-58643073	16	NSMAF
chr3:138694785-138699095	30	PIK3CB
chr6:149533457-149535738	5	PPIL4
chr8:47877723-47886147	11	PRKDC
chr3:8898893-8902520	5	RAD18
chr3:50106766-50109688	45	RBM5
chr3:196885150-196903610	15	SENP5
chr16:88671276-88672122	1	SNAI3-AS1
chr7:17890362-17907340	38	SNX13
chr16:87981035-87984259	32	BANP
chr8:67118247-67137603	25	CSPP1
chr3:170171372-170178938	186	PHC3
chr5:40716396-40730343	2	TTC33
chr7:98312096-98317356	17	BAIAP2L1
chr5:146164309-146172774	18	LARS
chr2:189689526-189696369	12	ANKAR
chr4:40934455-40945073	39	APBB2
chr13:110687720-110705571	2	CARS2
chr8:117818434-117837201	42	EXT1
chr1:155312254-155312584	1	FDPS
chr5:177619077-177627614	38	LOC202181

chr2:171809605-171855949	4	SLC25A12
chr5:37177620-37181005	2	C5orf42
chr3:141903619-141907274	2	ATP1B3
chr22:45738730-45740538	3	ATXN10
chr20:32795408-32797299	19	DNMT3B
chr3:56669699-56673725	188	FAM208A
chr5:138555482-138555787	45	nogene
chr6:17669292-17675033	23	NUP153
chr2:135129994-135130721	20	RAB3GAP1
chr1:176116623-176163891	29	RFWD2
chrX:47243395-47245482	8	USP11
chr4:3316069-3318051	53	RGS12
chr20:13483226-13537049	12	TASP1
chr8:118580714-118585992	20	SAMD12
chr2:135090997-135093693	29	RAB3GAP1
chr22:45700281-45740538	476	ATXN10
chr21:29326058-29351813	3	BACH1
chr6:35619095-35672759	3	FKBP5
chr13:19751270-19759418	4	PSPC1
chr3:41931843-41938197	8	ULK4
chr2:190900563-190910321	52	GLS
chr5:149501014-149513209	24	CSNK1A1
chr14:64442053-64453861	20	MTHFD1
chr4:143543508-143543972	2002	SMARCA5
chr13:30227411-30284882	11	KATNAL1
chr3:144263866-144270349	2	nogene
chr6:167864550-167891029	48	MLLT4
chr2:111800796-111803839	21	ANAPC1
chr19:10772478-10786706	29	DNM2
chr7:148846469-148850525	28	EZH2
chr3:71493425-71501988	25	FOXP1
chr6:1675210-1675608	15	nogene
chr5:151946638-151947795	35	nogene
chr8:31142625-31150455	12	WRN
chr10:1010419-1012662	18	GTPBP4
chr2:3421880-3424663	43	TRAPPC12
chr4:128091000-128091512	31	LARP1B
chr20:58418210-58441083	11	VAPB
chr22:24113565-24134415	43	CABIN1
chr1:64666056-64675983	25	CACHD1
chr10:126970701-126987617	39	DOCK1
chr1:23053760-23057565	3	KDM1A
chr2:135129994-135135932	14	RAB3GAP1

chr6:108854224-108876350	16	ARMC2
chr16:57164000-57172874	15	FAM192A
chr1:153761569-153765063	39	INTS3
chr1:150927703-150941428	14	SETDB1
chr18:662145-671451	24	TYMS
chr17:76738281-76754204	1	MFSD11
chr7:106092948-106099282	17	SYPL1
chrX:46853475-46854141	2	RP2
chr5:179765325-179769089	17	MAML1
chr16:74644361-74649202	2	RFWD3
chr12:123334052-123341088	7	SBNO1
chr19:40255157-40257054	18	AKT2
chr1:155721516-155726019	195	DAP3
chr21:32748498-32755353	33	PAXBP1
chr3:126629378-126647296	10	TXNRD3
chr1:16294782-16316568	7	FBXO42
chr16:4831978-4832912	6	GLYR1
chr6:30730021-30731100	16	FLOT1
chr16:81011929-81014391	2	CENPN
chr17:36986616-36990857	105	AATF
chr10:32543299-32584304	85	CCDC7
chr11:77624962-77629899	349	CLNS1A
chr2:15374607-15427794	19	NBAS
chr2:9568364-9568726	1	nogene
chr11:73716250-73720899	5	RAB6A
chr15:89594414-89595611	36	TICRR
chr8:63186146-63187745	55	YTHDF3
chr9:125484442-125506417	30	MAPKAP1
chr6:44250422-44251116	31	nogene
chr13:100268688-100273346	36	PCCA
chr6:110667433-110773937	3	CDK19
chr1:44241914-44248038	11	ERI3-IT1
chr16:89752137-89767237	3	FANCA
chr4:39300020-39303151	64	RFC1
chr7:67055834-67098718	33	TYW1
chr1:162597791-162601514	17	UAP1
chr3:33400188-33425741	55	UBP1
chr1:220182256-220182931	45	RAB3GAP2
chr5:138781922-138812302	16	CTNNA1
chr7:90352815-90355230	19	GTPBP10
chr20:44978442-44987296	52	STK4
chr7:92294888-92307656	124	ANKIB1
chr22:20554935-20566817	16	MED15

chr3:47729024-47772936	187	SMARCC1
chr4:25314045-25315400	52	ZCCHC4
chr11:74963594-74969699	11	SPCS2
chr8:130358016-130362366	34	ASAP1
chr10:35483403-35553185	13	CCNY
chr10:43154896-43158931	6	CSGALNACT2
chr4:15630665-15644708	19	FBXL5
chr14:65455620-65478414	2	FUT8
chr2:172558707-172566933	34	PDK1
chr14:39095897-39096139	7	SEC23A
chr20:31544946-31561736	3	HM13
chr7:129379640-129389734	4	AHCYL2
chr20:32704022-32706760	6	COMMD7
chr9:20715321-20758191	40	FOCAD
chr10:68038092-68044563	13	HERC4
chr19:5039835-5041251	89	KDM4B
chr20:5952010-5955251	2	MCM8
chr6:42883450-42884750	45	RPL7L1
chr7:24623665-24663721	20	MPP6
chrX:57842245-57850693	10	nogene
chr1:168231513-168239160	25	SFT2D2
chr4:3186596-3199939	3	HTT
chr4:165218024-165238899	23	KLHL2
chr1:151658234-151693483	15	SNX27
chr3:133608534-133617326	3	TOPBP1
chr21:37108391-37123028	11	TTC3
chr2:200920221-200937966	16	ORC2
chr5:122799691-122808355	38	SNX2
chr2:74205704-74208721	16	MTHFD2
chr4:505717-508970	10	PIGG
chr6:154787860-154810208	15	SCAF8
chr3:12528887-12532773	3	TSEN2
chr9:88462495-88462749	3	SPIN1
chr12:32708152-32713371	16	DNM1L
chr18:79110354-79154555	12	ATP9B
chr2:53693883-53750941	20	GPR75-ASB3
chr4:13336892-13381594	5	RAB28
chr3:185590113-185600855	17	SENP2
chr14:99457555-99465813	33	SETD3
chr22:49804203-49824331	33	BRD1
chr12:82369386-82456820	37	METTL25
chr1:171512031-171522259	9	PRRC2C
chr5:65451474-65471142	14	ADAMTS6

chr11:120457720-120474635	16	ARHGEF12
chr7:34966893-34973605	32	DPY19L1
chr16:57172775-57173869	53	FAM192A
chr14:31113103-31119866	2	HECTD1
chr2:55661955-55673079	16	PNPT1
chr14:50640749-50649389	3	SAV1
chr19:19303347-19303847	18	SUGP1
chr5:65242103-65473952	16	ADAMTS6
chr7:73459535-73470483	28	BAZ1B
chr15:92924320-92929091	154	CHD2
chr5:134790469-134807941	14	DDX46
chr17:43498861-43500103	41	DHX8
chr15:76287845-76292700	102	ETFA
chr1:20765376-20779911	3	HP1BP3
chrX:115122526-115130199	50	LRCH2
chr10:101798841-101807901	4	MGEA5
chr7:66528672-66529097	30	nogene
chr3:170359698-170361429	28	SKIL
chr10:91849511-91851336	20	TNKS2
chr8:99156548-99275254	31	VPS13B
chr12:133191488-133192003	79	ZNF268
chr7:64543706-64544432	205	ZNF680
chr7:65757982-65760556	27	CCT6P1
chr3:126642031-126647296	75	TXNRD3
chr1:144955438-144960398	19	SRGAP2B
chr6:42573733-42573993	13	UBR2
chr5:21491320-21495182	160	GUSBP1
chr3:197149742-197161738	2	DLG1
chr16:66730111-66732505	36	DYNC1LI2
chr6:70486166-70502791	2	FAM135A
chr1:1815755-1825499	334	GNB1
chr13:23798723-23886506	171	MIPEP
chr5:88196375-88203075	2	TMEM161B
chr2:61492034-61497007	49	XPO1
chr8:140800458-140803650	21	PTK2
chr18:12814202-12836891	84	PTPN2
chr4:53383772-53428183	48	FIP1L1
chr10:27935009-27936880	45	ARMC4
chr10:21612347-21617207	5	MLLT10
chr5:80678926-80744615	28	MSH3
chr14:34585421-34586036	2	nogene
chr12:8749861-8754093	16	RIMKLB
chr15:84538531-84546277	7	UBE2Q2P1

chr19:37089038-37091121	21	ZNF420
chr11:63658760-63659252	173	ATL3
chr10:110502720-110510596	16	DUSP5
chr8:140735250-140764290	8	PTK2
chr8:116655855-116726172	3	EIF3H
chr6:35587033-35642843	3	FKBP5
chr7:86925527-86947922	7	KIAA1324L
chr6:169582835-169634525	1	WDR27
chr6:110887504-110888983	29	AMD1
chr1:84179176-84235364	16	PRKACB
chr15:41375711-41377304	61	NUSAP1
chr14:77557040-77576915	35	SPTLC2
chr3:184181695-184182868	13	AP2M1
chr5:135343259-135370142	23	H2AFY
chr1:45602254-45605016	12	NASP
chr1:8508626-8557523	43	RERE
chr1:32786361-32791254	2	YARS
chr5:43122038-43139309	105	ZNF131
chr1:243270104-243286397	26	SDCCAG8
chr9:100303631-100321771	11	TEX10
chr1:155358610-155359386	19	nogene
chr3:57841298-57864857	5	SLMAP
chr2:9523248-9536828	12	ADAM17
chr5:126603569-126604183	57	PHAX
chr6:124996574-125045444	2	RNF217
chr11:65504132-65504595	4	nogene
chr1:11947975-11950520	6	PLOD1
chr2:69806384-69808189	4	ANXA4
chr5:633768-644425	11	CEP72
chr14:37369516-37422949	28	MIPOL1
chr20:36911217-36912552	16	SAMHD1
chr10:27134038-27136385	18	YME1L1
chr7:148729961-148737743	20	CUL1
chr11:47625673-47634734	6	MTCH2
chr20:47309658-47310204	30	ZMYND8
chr12:22484696-22506819	40	C2CD5
chr5:157791702-157791995	5	CLINT1
chr10:68442916-68445083	14	DNA2
chr13:30240459-30283791	254	KATNAL1
chr4:55411613-55424643	3	TMEM165
chr9:125467971-125484583	7	MAPKAP1
chr11:34957383-34970286	6	PDHX
chr17:5338018-5350629	2	RABEP1

chr19:37643264-37647899	4	ZFP30
chr2:215312497-215325329	1	ATIC
chr7:40045835-40088331	4	CDK13
chr20:9437001-9444243	3	PLCB4
chr16:72088986-72090786	234	TXNL4B
chr12:68715626-68725796	102	NUP107
chr1:225038693-225043983	45	DNAH14
chr3:196487398-196488683	51	RNF168
chr18:9577063-9595153	38	PPP4R1
chr20:45075017-45077961	4	STK4
chr10:3138363-3138903	13	PITRM1
chr4:183810105-183829990	2	nogene
chr3:150622384-150627914	13	SELT
chr4:145110967-145121391	36	ABCE1
chr3:53874210-53876080	63	ACTR8
chr14:32117139-32152528	55	ARHGAP5
chr8:61644599-61684188	67	ASPH
chr10:32471063-32481782	15	CCDC7
chr11:117335604-117344277	28	CEP164
chr11:70405264-70429199	14	CTTN
chr13:59833106-59839448	18	DIAPH3
chr8:28129501-28162078	6	ELP3
chr5:153994918-154034967	21	FAM114A2
chr2:222599927-222624479	24	FARSB
chr6:42822017-42830172	2	GLTSCR1L
chr19:5016256-5033031	8	KDM4B
chr7:92213194-92226682	17	KRIT1
chr9:110941420-110973558	274	LPAR1
chr19:12230459-12234908	14	nogene
chr6:87621353-87636486	12	ORC3
chr5:50778068-50797233	24	PARP8
chr1:214369456-214372839	7	PTPN14
chr6:3283851-3298218	23	SLC22A23
chr2:213310058-213340270	56	SPAG16
chr3:30644746-30674246	3	TGFBR2
chr20:35044504-35057571	19	TRPC4AP
chr14:102193462-102209862	14	WDR20
chr4:55411613-55417985	156	TMEM165
chr22:28629551-28633974	83	TTC28
chr12:63597808-63663845	18	DPY19L2
chr20:34477771-34479789	36	ITCH
chr7:130173631-130181719	6	TMEM209
chr10:92934143-92940824	18	EXOC6

chr5:62550366-62601848	17	IPO11
chr1:185300436-185305643	11	IVNS1ABP
chr9:97139852-97146151	16	nogene
chrX:155415532-155419562	6	nogene
chr2:183128286-183133623	197	NUP35
chr8:100709132-100718280	27	PABPC1
chr7:1898199-2002121	7	MAD1L1
chrX:77633206-77636056	16	ATRX
chr4:2596083-2646832	8	FAM193A
chr17:47664158-47665158	7	KPNB1
chr17:75208526-75209985	7	NUP85
chr11:18514677-18519603	41	TSG101
chr1:233227225-233259344	12	PCNXL2
chr8:140830471-140879637	184	PTK2
chr10:99969114-99972134	75	DNMBP
chr16:11994028-11995160	11	nogene
chr1:23785674-23786423	25	PITHD1
chr16:70541668-70548442	28	SF3B3
chrX:131755597-131777744	77	FIRRE
chr17:82568053-82571870	6	FOXK2
chr22:40832568-40848369	48	ST13
chr3:119905754-119916174	15	GSK3B
chr12:48063709-48101516	28	SENP1
chr1:180397515-180495525	85	ACBD6
chr1:32036909-32038619	80	KHDRBS1
chr4:150735257-150817257	36	LRBA
chr2:197523623-197548415	12	MOB4
chr21:22032384-22043087	6	nogene
chr3:196361843-196403019	16	UBXN7
chr12:110292019-110296737	34	ATP2A2
chr12:50427761-50435624	41	LARP4
chr5:138191473-138192657	26	CDC23
chr10:7774830-7780307	7	KIN
chr6:100798712-100848707	27	ASCC3
chr17:82805874-82814934	48	TBCD
chr22:46390391-46391816	6	CELSR1
chr4:41142986-41145230	21	APBB2
chr21:25954589-25955755	76	APP
chr8:130159863-130188183	6	ASAP1
chr12:53669147-53669948	23	ATP5G2
chr16:75293909-75305182	19	CFDP1
chr7:6421726-6436533	34	DAGLB
chr7:6028614-6029032	32	EIF2AK1

chr1:42265114-42278784	107	FOXJ3
chr9:128533512-128533951	12	GLE1
chr5:62449926-62451933	23	IPO11
chr5:176347577-176350737	3	KIAA1191
chr1:28890322-28891801	8	nogene
chr4:67798638-67802419	6	nogene
chr11:74850929-74874895	7	nogene
chr10:124942456-124962408	94	nogene
chr1:15792836-15796943	21	nogene
chr18:24332941-24341658	6	OSBPL1A
chr13:24441845-24443730	34	PARP4
chr3:196247366-196248323	63	PCYT1A
chr9:125158240-125160906	2	PPP6C
chr2:203489583-203495353	64	RAPH1
chr9:112078537-112098662	12	SUSD1
chrX:71368053-71375286	21	TAF1
chr2:61278164-61281242	98	USP34
chr2:63981645-63984019	29	VPS54
chr2:71348752-71350271	35	ZNF638
chr5:118928536-118944649	152	DTWD2
chr12:110025741-110030758	23	ANKRD13A
chr4:40890363-40945073	8	APBB2
chr17:67945408-67975958	87	BPTF
chr1:85350414-85351579	8	DDAH1
chr22:38537591-38538612	6	DMC1
chr2:85368685-85371209	74	ELMOD3
chr7:139596716-139620563	10	HIPK2
chr4:129724509-129727033	2	LOC101927282
chr5:10415487-10417404	394	6-Mar
chr10:94520458-94520754	17	nogene
chr19:43872628-43884051	15	nogene
chr10:99907300-99907490	15	nogene
chr10:34377966-34384254	11	PARD3
chr6:3737078-3742297	21	PXDC1
chr17:64551583-64562966	24	SMURF2
chr9:37126311-37305714	61	ZCCHC7
chr13:77251150-77288376	3	MYCBP2
chr1:179986168-179992221	21	CEP350
chr22:31758545-31778168	10	DEPDC5
chr17:8204849-8207840	18	AURKB
chr10:68963290-68967203	142	DDX21
chr12:124952397-124954211	113	DHX37
chr8:77002693-77007102	5	nogene

chr15:55327530-55341802	2	PIGB
chr12:113320330-113321348	35	SLC8B1
chr8:102261914-102264558	35	UBR5
chr6:57379900-57606457	1	PRIM2
chr2:39272282-39280356	2	MAP4K3
chr5:108867766-108898132	5	FER
chr14:31162592-31169468	3	HECTD1
chr15:68145815-68153695	15	PIAS1
chr3:61748877-62003497	13	PTPRG
chr3:184936245-184966713	18	VPS8
chr2:63433744-63439871	188	WDPCP
chr9:37302187-37305714	33	ZCCHC7
chr7:99385701-99394119	2	ARPC1B
chr15:74926765-74929232	132	COX5A
chr17:8444259-8460160	33	NDEL1
chr3:61989624-62015932	2	PTPRG
chr18:756556-756835	218	YES1
chr15:64180347-64216713	15	CSNK1G1
chr17:28921687-28927063	189	PHF12
chr6:138785226-138788439	3	CCDC28A
chr19:44076321-44085803	21	ZNF284
chr3:198072755-198077288	8	ANKRD18DP
chr2:101257790-101265047	68	CNOT11
chr19:12679456-12680325	51	DHPS
chr6:629834-689212	2	EXOC2
chr3:183017265-183022554	7	MCCC1
chr7:24620051-24650712	166	MPP6
chr3:179690240-179707076	20	USP13
chr10:103091563-103095980	15	NT5C2
chr2:69329296-69329798	3	GFPT1
chr11:118583178-118584644	18	ARCN1
chr1:20892618-20904971	23	EIF4G3
chr17:7936817-7937127	15	CNTROB
chr4:107682014-107694006	51	PAPSS1
chr5:133959493-133960020	30	C5orf15
chr1:212329134-212349242	13	PPP2R5A
chr1:40406735-40409835	32	SMAP2
chr9:112262434-112276013	30	PTBP3
chr6:57926425-57930291	14	GUSBP4
chrX:70290437-70290805	26	KIF4A
chr6:49431728-49443881	4	nogene
chr10:94474667-94511654	20	TBC1D12
chr10:94497054-94511654	5	TBC1D12

chr10:12081471-12117755	2	DHTKD1
chr15:89307475-89314707	30	FANCI
chr5:108832769-108835807	2	FER
chr15:60455002-60468322	2	ICE2
chr11:108185231-108190500	45	NPAT
chr1:51823663-51827869	11	NRD1
chr11:117239111-117239800	75	RNF214
chr14:54984722-55000992	40	WDHD1
chr3:183747671-183762279	7	YEATS2
chr1:155675009-155679512	413	YY1AP1
chr19:21033455-21034184	135	ZNF430
chr5:60918264-60928959	11	ERCC8
chr10:45888164-45939447	16	PARGP1
chr7:158741522-158743678	2	ESYT2
chr19:42335034-42335385	47	MEGF8
chr5:80767932-80778836	6	MSH3
chr2:61490641-61498913	4	XPO1
chr9:17309057-17342444	9	CNTLN
chr17:82178473-82179189	3	CCDC57
chr3:53304176-53319473	8	DCP1A
chr1:182842544-182843434	26	DHX9
chr3:136945582-136946295	24	NCK1
chr12:100282942-100291660	31	SCYL2
chr1:109573736-109574037	19	GNAI3
chr10:27169943-27171083	54	MASTL
chr2:213310058-213364145	96	SPAG16
chr4:130787-131505	36	ZNF718
chr18:26170674-26171200	4	nogene
chr11:108186481-108190500	68	NPAT
chr17:75242335-75243570	136	GGA3
chr2:39286851-39288280	27	MAP4K3
chr21:37087246-37088886	33	TTC3
chrX:129919174-129921587	14	UTP14A
chr9:91883257-91887113	39	nogene
chr1:225097117-225123614	4	DNAH14
chr12:110387738-110396452	13	ANAPC7
chr14:93293857-93294937	60	BTBD7
chr10:102029637-102033339	2	C10orf76
chr12:50671185-50680763	5	DIP2B
chr15:77365162-77388802	17	nogene
chr20:1452492-1458274	28	NSFL1C
chr3:73047185-73061060	1	PPP4R2
chr2:226864603-226867688	36	RHBDD1

chr14:52757318-52759754	18	STYX
chr17:18290556-18292852	3	TOP3A
chr6:99488199-99510230	13	USP45
chr20:25639728-25645648	48	ZNF337-AS1
chr10:86838864-86900126	12	BMPR1A
chr18:76868684-76881512	28	ZNF236
chr19:45613540-45614700	43	EML2
chr10:5903019-5909294	6	FBXO18
chr22:21669912-21675115	12	PPIL2
chr1:77858937-77866391	122	FAM73A
chr2:108752997-108753563	31	RANBP2
chr17:35833906-35838553	3	TAF15
chr17:41981854-41982401	4	DNAJC7
chr21:45184922-45204736	14	ADARB1
chr1:243693666-243843282	43	AKT3
chr3:138221925-138223733	9	ARMC8
chr10:32451591-32463049	7	CCDC7
chr6:83909008-83924592	45	CYB5R4
chr1:77803269-77813867	16	FAM73A
chr15:69421646-69423329	34	KIF23
chr19:55674122-55674621	12	nogene
chr3:56570090-56570499	12	nogene
chr3:8840195-8841539	3	nogene
chr8:47933019-47935058	58	PRKDC
chr17:76287191-76290077	23	QRICH2
chr3:49962575-49975392	25	RBM6
chr2:40428472-40430301	205	SLC8A1
chr20:31757406-31771682	12	TPX2
chr5:172234121-172234358	38	UBTD2
chr11:9494645-9516362	2	ZNF143
chr22:41125863-41140257	70	EP300
chr2:108839184-108856970	83	CCDC138
chr2:241403827-241413421	84	FARP2
chr6:15246380-15410365	11	JARID2
chr7:4989062-4996798	60	RNF216P1
chr5:134697125-134698057	58	SEC24A
chr9:134139819-134148343	26	WDR5
chr17:1079329-1100735	28	ABR
chr12:122932191-122935431	9	ABCB9
chr6:30627459-30643011	3	ATAT1
chr3:191380158-191391897	2	CCDC50
chr12:70294070-70311017	20	CNOT2
chr4:151415723-151482648	13	FAM160A1

chr10:130145210-130166980	30	GLRX3
chr1:1793244-1817875	27	GNB1
chr16:11891061-11898035	10	GSPT1
chr10:68025545-68038169	33	HERC4
chr12:109451590-109460805	14	KCTD10
chrX:101163970-101164368	2	nogene
chrX:107150855-107153503	14	NUP62CL
chr1:7977651-7985135	3	PARK7
chr20:38488402-38499633	3	RALGAPB
chr15:90959899-90961774	37	RCCD1
chr20:19956614-19960811	21	RIN2
chr8:213120-213710	31	RPL23AP53
chr3:52926030-52928341	113	SFMBT1
chr1:24662659-24670315	19	SRRM1
chr3:197763465-197768587	27	FYTTD1
chr2:177434326-177445626	6	AGPS
chr6:104811310-104843298	5	HACE1
chrX:150727214-150732730	25	MTMR1
chr5:177950308-177957662	5	nogene
chr4:112808902-112818264	83	ANK2
chr12:22506710-22544179	12	C2CD5
chr17:17254858-17264981	11	COPS3
chr13:30274693-30283791	25	KATNAL1
chr14:70748828-70750081	92	MAP3K9
chrX:27301038-27336493	3	nogene
chr17:5462232-5462826	13	nogene
chr2:45476992-45562756	43	SRBD1
chr22:28617897-28629830	2	TTC28
chr19:52001812-52002281	18	ZNF615
chr14:59456437-59479351	4	L3HYPDH
chr7:18585280-18594029	67	HDAC9
chrX:106985095-106986200	18	MORC4
chr9:128464436-128465460	13	nogene
chr10:45939360-45962994	321	PARGP1
chr3:11331339-11380052	7	ATG7
chr1:59321535-59340069	17	FGGY
chr14:39055142-39076093	4	SEC23A
chr19:47209158-47210724	26	SAE1
chr3:126050942-126068246	4	SLC41A3
chr10:86875866-86900126	68	BMPR1A
chr17:28122602-28172618	92	NLK
chr16:29969479-29969965	4	nogene
chr18:6263946-6312056	166	L3MBTL4

chr17:47326225-47345098	35	EFCAB13
chr1:231364326-231367636	15	EGLN1
chr11:72867864-72902638	18	FCHSD2
chr15:83151214-83158041	6	HDGFRP3
chr19:38845616-38846090	3	HNRNPL
chr2:87052813-87055800	16	LOC285074
chr9:36369718-36376127	6	RNF38
chr10:132244878-132245955	56	STK32C
chr7:130022318-130023723	3	ZC3HC1
chr12:121494578-121549638	2	KDM2B
chr2:128186682-128187614	5	UGGT1
chr15:78544189-78545764	13	PSMA4
chr7:152309965-152358675	19	KMT2C
chr11:94795010-94831541	5	AMOTL1
chr12:32710928-32718763	16	DNM1L
chr3:56668396-56673725	32	FAM208A
chr1:109573736-109579361	15	GNAI3
chr4:168894578-168898714	13	PALLD
chr16:72617182-72617553	26	nogene
chr11:113840597-113854335	54	USP28
chr3:43565652-43577261	13	ANO10
chr21:33579349-33586181	2	DONSON
chr20:37201551-37210165	3	RPN2
chr2:9428764-9433960	70	CPSF3
chr5:40746797-40771863	4	TTC33
chr1:18942362-18943433	12	nogene
chr5:94868332-94924021	10	MCTP1
chr7:33351218-33367862	12	BBS9
chr12:124426621-124437996	17	NCOR2
chr8:47279861-47330895	5	SPIDR
chr6:10961304-10963843	14	SYCP2L
chr9:111914604-111931357	24	UGCG
chr1:44002312-44002985	2	SLC6A9
chr10:27114520-27121448	28	YME1L1
chr3:101417592-101459052	27	SENP7
chr5:179580946-179593645	83	RUFY1
chr2:177482058-177513908	14	AGPS
chr7:92350949-92362273	11	ANKIB1
chr3:107710451-107728960	15	BBX
chr18:23506969-23509279	4	C18orf8
chr19:3623687-3624162	468	CACTIN
chr2:134946100-134947899	2	CCNT2
chr2:207726841-207740706	1	CCNYL1

chr9:120437294-120448126	19	CDK5RAP2
chr1:239826349-239827378	6	CHRM3
chr5:119110150-119121139	2	DMXL1
chr8:94726330-94739790	17	DPY19L4
chr22:31462931-31463133	34	EIF4ENIF1
chr7:55978125-55984878	19	GBAS
chr3:158646164-158653467	14	GFM1
chr1:21847353-21849031	16	HSPG2
chr16:19732828-19733827	23	IQCK
chr8:144168294-144200541	9	MROH1
chr5:141410978-141411458	14	nogene
chr17:29280088-29281063	19	nogene
chr22:47860464-47862122	54	nogene
chr7:128577952-128580094	3	nogene
chr17:80266712-80266985	2	nogene
chr1:5922059-5933329	11	NPHP4
chr3:16294855-16303592	19	OXNAD1
chr1:148961836-148962640	16	PDE4DIP
chr1:28490950-28493091	35	PHACTR4
chr17:18865468-18889877	46	PRPSAP2
chr17:1875660-1883944	9	RPA1
chr16:89510489-89513037	26	SPG7
chr17:35834565-35838553	28	TAF15
chr1:230945388-230961303	60	TTC13
chr1:10149183-10161286	2	UBE4B
chr6:41856625-41891875	13	USP49
chr18:56758864-56781656	116	WDR7
chr7:99388038-99388261	19	ARPC1B
chr17:47402131-47409691	20	EFCAB13
chr8:55792697-55796152	2	TGS1
chr3:195342470-195392147	8	ACAP2
chr5:78156257-78165672	34	AP3B1
chr22:45700281-45702847	25	ATXN10
chr1:227139575-227317246	3	CDC42BPA
chr6:20781144-20846178	12	CDKAL1
chr11:68759568-68761687	54	CPT1A
chr4:182891402-182915575	427	DCTD
chr13:113474123-113484056	41	DCUN1D2
chr6:109765012-109777060	13	FIG4
chr5:131698916-131719417	8	FNIP1
chr12:112216291-112217195	20	HECTD4
chr11:119123567-119127125	27	HINFP
chr17:61954935-61956481	11	MED13

chr10:68121437-68122340	47	MYPN
chr12:6521797-6523346	45	NCAPD2
chr5:36982164-36986301	18	NIPBL
chr2:55598415-55604076	24	PPP4R3B
chr20:38057531-38066256	402	RPRD1B
chr12:123309309-123309856	13	SBNO1
chr11:85974707-85981228	16	PICALM
chr15:40698756-40709116	8	RAD51
chr7:18722951-18835997	6	HDAC9
chr11:122776218-122777210	164	UBASH3B
chr5:108348398-108378726	2	FBXL17
chr3:48772954-48794049	30	PRKAR2A
chr13:26665027-26671989	2	WASF3
chr2:197007805-197009031	18	ANKRD44
chr1:93212100-93217869	15	CCDC18
chr1:151236557-151236763	13	PIP5K1A
chr9:136391941-136392777	12	SNAPC4
chrX:150793690-150816078	99	CD99L2
chr5:10386993-10391731	12	6-Mar
chr15:44410792-44411135	26	nogene
chr6:17637152-17640064	12	NUP153
chr13:24249476-24253220	12	SPATA13
chr17:82870223-82911789	21	TBCD
chr3:32138586-32140366	6	GPD1L
chr8:140605531-140605914	12	nogene
chr17:1534098-1558559	76	PITPNA
chr7:6002452-6002636	16	PMS2
chr6:138943512-138944622	277	REPS1
chr5:677749-679441	14	TPPP
chr5:69355648-69366595	55	AK6
chr7:35879822-35885879	32	7-Sep
chr1:42227882-42278672	17	FOXJ3
chr4:17623600-17625845	6	MED28
chr10:104008176-104018908	79	SLK
chr1:179118586-179131481	1	ABL2
chr5:179152141-179158879	12	ADAMTS2
chr3:122759223-122768882	12	HSPBAP1
chr16:11983565-12027444	3	SNX29
chr2:169878524-169906164	1	UBR3
chr12:1027747-1290012	21	ERC1
chr11:3768580-3768744	35	NUP98
chr2:48498492-48514967	5	PPP1R21
chr14:90019315-90033214	12	TDP1

chr5:65170616-65215487	57	ADAMTS6
chr2:32377587-32401623	24	BIRC6
chr18:13099475-13103588	11	CEP192
chr2:62135830-62141360	3	COMMD1
chr7:158735502-158748280	3	ESYT2
chr17:82720271-82720841	7	FN3KRP
chr13:23874845-23881787	16	MIPEP
chr18:54273925-54287411	2	POLI
chr3:121449314-121468431	23	POLQ
chr6:88844356-88853764	170	RNGTT
chr16:70213660-70222480	46	SMG1P7
chr4:7006631-7010781	3	TBC1D14
chr16:24750725-24777358	24	TNRC6A
chr12:132932712-132948270	4	ZNF605
chr7:149549192-149558839	6	ZNF767P
chr4:145885677-145892415	12	ZNF827
chr15:72217408-72219110	18	PKM
chr7:66797373-66827578	14	RABGEF1
chr18:8076454-8114827	83	PTPRM
chr13:21400372-21425678	50	ZDHHC20
chr7:23140768-23152209	11	KLHL7
chr5:171183166-171205612	41	RANBP17
chr16:8905186-8917156	13	USP7
chr20:21340720-21346550	137	XRN2
chr11:46508190-46512813	41	AMBRA1
chr3:11306942-11315493	6	ATG7
chr3:33626987-33663515	17	CLASP2
chr7:80188950-80212869	18	GNAI1
chr4:129724509-129749720	10	LOC101927282
chr12:78006418-78007445	4	NAV3
chr9:128803249-128804119	35	TBC1D13
chr21:31245487-31252189	6	TIAM1
chr7:130181622-130192823	35	TMEM209
chr17:623532-627316	21	VPS53
chr19:58285716-58295581	18	ZNF8
chr3:155910691-155925366	41	GMPS
chrX:150814861-150831293	3	CD99L2
chr15:50643339-50663046	75	TRPM7
chr17:59737452-59808876	21	VMP1
chr1:230663140-230671640	115	COG2
chr7:32675931-32679130	23	DPY19L1P1
chr20:46393092-46394532	6	ELMO2
chr22:41129889-41131633	16	EP300

chr15:77252366-77365225	25	PEAK1
chr13:23353784-23358481	12	SACS
chr18:2795948-2796521	12	SMCHD1
chr1:179302676-179323495	41	SOAT1
chr1:206392723-206421274	19	SRGAP2
chr8:123231299-123231757	95	ZHX1-C8orf76
chr10:110228188-110280085	12	MXI1
chr16:68121986-68174514	4	NFATC3
chr5:131548046-131562047	12	RAPGEF6
chr7:5739275-5741644	46	RNF216
chr1:40406735-40417096	7	SMAP2
chr12:31447537-31460381	9	DENND5B
chr12:110013124-110029635	13	ANKRD13A
chr16:71769622-71774592	13	AP1G1
chr7:107583881-107613432	1	BCAP29
chr1:227193785-227199652	37	CDC42BPA
chr13:60008543-60016145	26	DIAPH3
chr22:38562286-38568289	48	DMC1
chr1:42227882-42323827	64	FOXJ3
chr12:109988962-109991760	3	GIT2
chr7:50669721-50705323	26	GRB10
chr10:89758705-89762835	1	KIF20B
chr2:241089187-241094432	14	MTERF4
chr14:55060436-55060888	16	nogene
chr3:194604286-194611020	24	TMEM44
chr13:46020448-46045513	19	ZC3H13
chr3:195365481-195412916	14	nogene
chr2:189785345-189818180	4	PMS1
chr8:56963962-56980197	26	IMPAD1
chr15:41055246-41058781	15	INO80
chr5:168488601-168492847	92	RARS
chr3:197830769-197839397	157	LRCH3
chr7:1957628-2014642	6	MAD1L1
chr1:155395458-155459898	86	ASH1L
chr10:87756788-87776427	12	ATAD1
chr10:126970701-127061776	16	DOCK1
chr17:60479147-60500487	9	APPBP2
chr5:75379336-75403058	2	COL4A3BP
chr3:121460049-121468431	17	POLQ
chr5:151117126-151119390	2	ANXA6
chr10:24666984-24670397	3	ARHGAP21
chr10:84425730-84477664	21	CCSER2
chr1:225185290-225206179	12	DNAH14

chr17:6589778-6600458	32	KIAA0753
chr4:128162193-128179512	13	LARP1B
chr20:35696376-35698590	2	NFS1
chr16:30069847-30070211	1	nogene
chr12:106363869-106369683	29	POLR3B
chr1:178776681-178811404	3	RALGPS2
chr2:3545700-3556904	19	RNASEH1
chr13:60444682-60494575	2	TDRD3
chr12:56433380-56434231	17	TIMELESS
chr6:42659655-42666245	17	UBR2
chr22:40881769-40882177	76	XPNPEP3
chr22:29748417-29754863	1	ZMAT5
chr9:96827079-96852005	11	ZNF782
chr10:28535561-28583505	17	WAC
chr18:79193182-79214038	17	ATP9B
chr2:171435082-171453213	3	DCAF17
chr3:197104902-197149796	23	DLG1
chr10:92510049-92537550	3	IDE
chr17:61982197-61985090	4	MED13
chr10:73051052-73053590	11	P4HA1
chr5:102895850-102901413	2	PAM
chr15:34252169-34255392	1	SLC12A6
chr10:100923974-100926019	139	SLF2
chr2:43485233-43527988	63	THADA
chr1:243572925-243843282	18	AKT3
chrX:77557450-77558846	43	ATRX
chr15:90441505-90443478	150	IQGAP1
chr16:70532478-70544533	99	SF3B3
chr17:62552301-62553755	60	TLK2
chr9:19276157-19305527	5	DENND4C
chr22:29121356-29125416	58	KREMEN1
chr5:150541569-150549787	33	NDST1
chr18:56624287-56626457	8	TXNL1
chr7:156992943-156994181	23	nogene
chr3:196814450-196820567	37	PAK2
chr7:65754526-65756574	9	CCT6P1
chr7:139631164-139631725	2	HIPK2
chr13:32658238-32659280	2	PDS5B
chr12:6678126-6688226	4	ZNF384
chr5:157518822-157520031	87	ADAM19
chr12:54241743-54257692	5	CBX5
chr1:114736757-114749904	1	CSDE1
chr2:38837830-38847073	2	DHX57

chr12:122216487-122224748	2	DIABLO
chr12:32713208-32722633	54	DNM1L
chr11:103280347-103323990	30	DYNC2H1
chr3:186787126-186787584	110	EIF4A2
chr1:44308309-44339322	204	ERI3
chr5:137985256-138021197	25	FAM13B
chr15:50300788-50301368	50	GABPB1
chr4:150467672-150491035	61	LRBA
chr17:45513184-45515525	13	LRRC37A4P
chr2:206841106-206881891	11	nogene
chr13:44949721-44959574	14	NUFIP1
chr5:168566012-168568998	25	PANK3
chr3:123089167-123116298	1	PDIA5
chr9:127166068-127168772	1	RALGPS1
chr5:170878096-170924550	43	RANBP17
chr7:151470570-151491014	308	RHEB
chr16:57204503-57217035	29	RSPRY1
chr4:122979231-123028395	2	SPATA5
chr7:38789800-38821265	8	VPS41
chr8:86374029-86381629	42	WWP1
chr18:12698978-12701113	16	CEP76
chr17:12081355-12113360	123	MAP2K4
chr3:61621969-61623555	33	nogene
chr2:203193794-203197391	58	NBEAL1
chr22:38498084-38501280	69	DDX17
chr8:140879470-140890769	1106	PTK2
chr7:2149151-2219456	23	MAD1L1
chr6:44138479-44141098	3	TMEM63B
chr16:11698124-11721670	3	TXNDC11
chr3:122442037-122454001	30	KPNA1
chr8:125359174-125359428	28	nogene
chr16:88608933-88611536	20	ZC3H18
chr10:49832802-49843632	43	PARG
chr10:12249580-12250469	60	CDC123
chr1:246186742-246186942	345	nogene
chr17:60294682-60345608	17	USP32
chr7:130039463-130041473	48	ZC3HC1
chr4:176711498-176729746	94	VEGFC
chr1:228291897-228304566	7	OBSCN
chr2:74377431-74378245	15	DCTN1
chrX:97072940-97114965	13	DIAPH2
chr3:180968050-180970358	40	FXR1
chr11:9414254-9420698	5	IPO7

chr7:74096621-74097189	1	LIMK1
chr16:69653235-69655799	19	NFAT5
chr2:215325240-215338907	27	ATIC
chr2:43794456-43810524	11	DYNC2LI1
chr17:47664158-47674782	61	KPNB1
chr18:36198689-36205276	13	MOCOS
chr8:120453846-120456670	1	MTBP
chr9:35780266-35786775	11	nogene
chr19:52923368-52923675	11	nogene
chr8:47840015-47854214	33	PRKDC
chr6:88801563-88853764	65	RNGTT
chr21:36053582-36058924	11	SETD4
chr4:151147921-151149561	40	SH3D19
chr7:129205609-129208851	11	SMO
chr9:6420911-6460791	30	UHRF2
chr13:27075190-27090158	24	USP12
chr16:47311239-47375940	19	ITFG1
chr6:31648676-31651776	4	BAG6
chr18:8624937-8638416	34	RAB12
chr5:73773854-73780745	35	ARHGEF28
chr20:33848466-33852203	14	CHMP4B
chr1:234425672-234427766	11	TARBP1
chr1:182874853-182880608	1	DHX9
chr15:67585889-67630927	11	MAP2K5
chr16:30079458-30080069	17	nogene
chr15:51931030-51934354	1	nogene
chr2:10788696-10790833	58	PDIA6
chr6:121223235-121255410	20	TBC1D32
chr1:235450239-235458786	1	B3GALNT2
chr18:1269669-1361221	2	nogene
chr20:44503814-44512994	1	SERINC3
chr9:127050042-127069356	12	RALGPS1
chr6:38577599-38598121	126	BTBD9
chr20:37765196-37779335	17	CTNNBL1
chr1:225079206-225123614	2	DNAH14
chr2:151416560-151428922	18	RIF1
chr4:147633764-147634558	4	TMEM184C
chr19:46838053-46839578	1	AP2S1
chr19:4475274-4498915	1	HDGFRP2
chr7:102813276-102854860	25	FBXL13
chr3:4303154-4304602	18	nogene
chr2:72718102-72733118	1229	EXOC6B
chr15:50300788-50314670	12	GABPB1

chr8:103400464-103407784	2	SLC25A32
chr12:19343322-19348519	26	PLEKHA5
chr19:14563981-14564858	4	TECR
chr20:35653527-35658982	36	CPNE1
chr20:18543018-18554390	73	SEC23B
chr4:183193598-183209025	92	WWC2
chr17:63578469-63579943	51	DCAF7
chr19:13814514-13814865	50	nogene
chr16:47463898-47589102	9	PHKB
chr11:68569747-68596218	14	PPP6R3
chr20:37055552-37065473	3	RBL1
chr13:21301418-21301618	2	MIPEPP3
chr7:157207397-157225539	2	UBE3C
chr4:23105501-23186098	3	nogene
chr15:101010449-101010837	10	LRRK1
chr16:68266592-68273904	13	SLC7A6
chr19:52929075-52929414	15	ZNF321P
chr17:36513428-36515990	79	MYO19
chr5:31451532-31472232	14	DROSHA
chr8:89587890-89596512	24	nogene
chr22:28514756-28517279	116	nogene
chr20:49855437-49864844	5	SLC9A8
chr13:95757643-95763953	61	DNAJC3
chr3:28263290-28298350	41	CMC1
chr5:112234633-112240810	21	EPB41L4A
chr4:3154292-3160392	3	HTT
chr1:172924570-172933643	8	nogene
chr4:128077787-128082305	19	LARP1B
chr1:32948221-32949774	284	RNF19B
chr6:26422932-26458718	2	BTN2A3P
chr6:169759837-169768171	11	ERMARD
chr4:147906637-147913139	32	ARHGAP10
chr2:9297299-9327911	14	ASAP2
chr4:47512503-47515670	1	ATP10D
chr12:32713208-32718763	13	DNM1L
chr4:38932234-38935790	5	FAM114A1
chr16:48252130-48270274	11	LONP2
chr12:68824362-68828931	9	MDM2
chr13:23858859-23886506	106	MIPEP
chr1:235990885-236048989	1	NID1
chr9:2667150-2668061	49	nogene
chr17:82748742-82749656	1	nogene
chrX:155415574-155421708	1	nogene

chrX:65060697-65113813	7	nogene
chr1:229477596-229487613	1	NUP133
chr12:53454725-53471807	2	PCBP2
chr1:150332683-150333199	19	PRPF3
chr13:52417991-52426066	13	VPS36
chr13:20821703-20855765	11	XPO4
chr1:33280123-33280457	3	ZNF362
chr1:19183810-19192540	2	UBR4
chr22:38495795-38501280	82	DDX17
chr18:36133237-36145034	2	ELP2
chr6:130022226-130071127	1	L3MBTL3
chr10:34374873-34384254	38	PARD3
chr12:3821872-3863957	7	PARP11
chr20:41133486-41135811	6	PLCG1-AS1
chr8:100259106-100288267	21	RNF19A
chr14:30628208-30639864	18	SCFD1
chr5:77459061-77464809	12	WDR41
chr12:119822819-119850275	46	CIT
chr2:45546731-45562756	435	SRBD1
chr10:12133240-12149961	5	SEC61A2
chrX:108607274-108607578	45	nogene
chr5:139278326-139280058	70	SNHG4
chr16:58716013-58723902	12	GOT2
chr10:96544075-96578320	12	TM9SF3
chr1:1256044-1267992	82	UBE2J2
chr20:21365420-21382057	19	XRN2
chr10:11979056-12004727	8	UPF2
chr12:111418118-111418877	276	SH2B3
chr9:32985970-32989895	3	APTX
chr2:231235223-231296253	1	ARMC9
chr21:39218151-39225197	26	BRWD1
chr17:1436619-1437155	49	CRK
chr11:86250295-86252240	21	EED
chr9:125337017-125337568	14	GAPVD1
chr6:24454014-24467274	1	GPLD1
chr1:100049908-100061949	42	HIAT1
chr9:111378579-111385442	48	KIAA0368
chr18:36195256-36213482	1	MOCOS
chr3:17622647-17672705	6	nogene
chr1:51781163-51782643	7	OSBPL9
chr13:24477700-24478276	941	PARP4
chr1:233135012-233179175	90	PCNXL2
chr12:42387455-42398994	36	PPHLN1

chr14:67690211-67693052	20	RDH11
chr4:48369848-48383784	147	SLAIN2
chr14:34597549-34609742	14	SNX6
chr5:113532878-113542503	36	YTHDC2
chr18:14077333-14085076	6	ZNF519
chr6:53071056-53082618	10	FBXO9
chr4:150302624-150325898	2	LRBA
chr18:79410402-79411501	141	NFATC1
chr19:16081019-16101409	2	nogene
chr14:77296163-77304800	2	POMT2
chr8:103766226-103766537	9	RIMS2
chr16:2262277-2264760	59	RNPS1
chr3:196362293-196393619	18	UBXN7
chr4:139336847-139341069	79	NAA15
chr14:52536718-52544371	5	TXNDC16
chr2:64572945-64585545	65	AFTPH
chr17:29648143-29672129	35	SSH2
chr1:154172907-154176248	33	TPM3
chr5:126561082-126593404	3	ALDH7A1
chr9:86016538-86018821	11	NAA35
chr4:1892165-1904378	53	WHSC1
chr1:92088697-92108001	11	BTBD8
chr7:66210876-66211229	17	nogene
chr14:93186259-93188997	11	TMEM251
chr2:240552913-240555055	114	ANKMY1
chr1:201847278-201848594	71	IPO9
chr6:100512708-100540387	2	ASCC3
chr8:43059091-43069659	3	FNTA
chr14:20346862-20354247	3	PARP2
chr2:181057692-181063041	35	UBE2E3
chr10:103433947-103434975	1	PDCD11
chr8:140717597-140746860	4	PTK2
chr19:49719456-49719935	14	nogene
chr6:83315309-83407901	8	ME1
chr3:161241045-161247330	10	NMD3
chr6:135394775-135411544	6	AHI1
chr4:150870524-150893149	8	LRBA
chr3:52204692-52208247	2	ALAS1
chr10:119526031-119527424	23	RGS10
chr15:74923647-74929232	109	COX5A
chr12:109071611-109073532	14	USP30
chr12:45421070-45421273	11	ANO6
chr19:39820476-39821292	16	nogene

chr12:56568974-56571855	109	RBMS2
chr3:196058283-196065600	31	TFRC
chr7:99491076-99494631	11	ZNF394
chr7:23185069-23196766	8	NUPL2
chr15:32623491-32628802	3	ARHGAP11A
chr12:45811417-45821487	28	ARID2
chr22:17808843-17823060	20	MICAL3
chr12:6917037-6918162	1	ENO2
chr7:55978125-55997449	1	GBAS
chr1:224268033-224303857	1	NVL
chr6:128064757-128067792	23	PTPRK
chr12:813641-827262	16	WNK1
chr1:147658959-147659775	8	ACP6
chr19:11205809-11206108	2	nogene
chr7:832504-843520	33	SUN1
chr4:183683974-183685403	6	TRAPPC11
chr12:56666203-56673065	53	PTGES3
chr14:49799474-49800696	6	NEMF
chr17:27303366-27304911	13	WSB1
chr12:71642435-71644318	27	ZFC3H1
chr22:27873737-27897892	37	PITPNB
chr5:131521389-131548190	160	RAPGEF6
chr5:138424074-138425582	45	KDM3B
chr1:45602254-45606591	31	NASP
chr8:115619131-115623758	4	TRPS1
chr7:6017813-6018045	7	AIMP2
chr1:212044659-212047417	74	DTL
chr6:36072872-36075962	17	MAPK14
chr12:111672660-111683307	16	BRAP
chr5:134817495-134819004	3	DDX46
chr4:98099429-98134459	2	STPG2
chr21:15777903-15811210	13	USP25
chr22:37870175-37878171	29	EIF3L
chr22:33761370-33847741	78	LARGE
chr1:236806143-236816543	163	MTR
chr9:33293679-33295427	29	NFX1
chr17:46963696-46965039	6	nogene
chr20:20531686-20546832	6	RALGAPA2
chr7:6578565-6585260	20	ZDHHC4
chr9:146101-172172	27	CBWD1
chr1:93217737-93226449	8	CCDC18
chr3:98881539-98881767	437	DCBLD2
chr17:30780591-30784892	13	nogene

chr10:119909040-119918511	2	SEC23IP
chr2:109553086-109593119	6	10-Sep
chr10:88901655-88905660	14	STAMBPL1
chr10:69185986-69189435	21	SUPV3L1
chr7:141159525-141212704	2	TMEM178B
chr21:15762890-15766141	290	USP25
chr10:109907690-109915099	11	XPNPEP1
chr1:62577261-62578966	26	DOCK7
chr18:2577745-2579029	15	NDC80
chrX:74592342-74596000	13	RLIM
chr1:41113282-41151684	37	SCMH1
chr4:42001615-42039053	15	SLC30A9
chr9:98069458-98078347	40	NANS
chr21:46388741-46391376	27	PCNT
chr1:6333476-6349866	2	ACOT7
chr11:129123445-129124894	25	ARHGAP32
chr7:65757982-65759271	47	CCT6P1
chr9:128169051-128180799	103	CIZ1
chr14:54430260-54436437	16	CNIH1
chr11:65998441-65999675	35	EIF1AD
chr5:138513567-138517700	3	ETF1
chr4:53379072-53414722	2	FIP1L1
chr1:10342049-10348733	8	KIF1B
chr19:325634-334542	5	MIER2
chr2:47790926-47796063	24	MSH6
chr9:14102421-14125766	20	NFIB
chr7:2737560-2738181	14	nogene
chr10:27143009-27143446	49	nogene
chr4:94618003-94640450	16	PDLIM5
chr11:48142918-48146963	13	PTPRJ
chr5:171199669-171213738	5	RANBP17
chr1:235152705-235155112	94	RBM34
chr7:29923955-29955360	15	SCRN1
chr5:134682372-134688299	16	SEC24A
chr20:62660411-62661175	2	SLCO4A1
chr1:150492410-150497747	29	TARS2
chr12:123679188-123687035	21	TCTN2
chr6:41891793-41909522	2	USP49
chr7:93252652-93272733	7	VPS50
chr1:108982608-109011718	2	WDR47
chr7:112895568-112915447	17	C7orf60
chr18:13666478-13671973	36	FAM210A
chr15:28260776-28263169	57	HERC2

chr12:110292019-110323072	18	ATP2A2
chr10:12235047-12238485	101	CDC123
chr3:56671599-56673725	4	FAM208A
chr9:124538091-124540187	26	NR6A1
chr1:229460610-229465519	11	NUP133
chr14:70962225-70978648	24	PCNX
chr18:62138242-62148338	42	PIGN
chr19:43655438-43656640	27	PLAUR
chr1:214314761-214318983	14	SMYD2
chr17:37467453-37471137	6	TADA2A
chr1:77858937-77875111	4	FAM73A
chr4:105370836-105438036	16	PPA2
chr1:235246419-235260752	69	ARID4B
chr16:3810602-3854850	12	CREBBP
chr7:39781234-39789544	2	LINC00265
chr22:42299996-42301247	16	nogene
chr2:97490456-97491153	34	nogene
chr2:53897869-53901559	2	PSME4
chr5:748403-837480	11	ZDHHC11
chr4:119460182-119462979	47	LOC645513
chr12:116096668-116237705	42	MED13L
chr16:70280364-70282049	14	nogene
chr1:180984676-181005463	78	STX6
chr19:52001812-52003900	2	ZNF615
chr2:101288972-101295181	86	RNF149
chr7:102397619-102399648	18	LOC100630923
chr4:70942822-70970024	4	MOB1B
chr4:13425109-13426354	11	nogene
chr11:107223209-107225846	1	nogene
chrX:75112885-75114834	19	ABCB7
chr5:179705678-179724783	18	CANX
chr20:35595476-35595754	18	FER1L4
chr1:230178217-230203290	42	GALNT2
chr1:109579203-109582565	10	GNAI3
chr4:82354048-82359639	11	HNRNPD
chr7:8218304-8236005	126	ICA1
chr16:20813189-20816212	5	LOC81691
chr7:1898199-1898390	12	MAD1L1
chr3:179592643-179601790	95	MRPL47
chr3:25764065-25778688	19	NGLY1
chr19:21062605-21063734	15	nogene
chr3:132575771-132605205	37	NPHP3-ACAD11
chr7:154960892-154962458	4	PAXIP1

chr9:111570090-111597432	6	PTGR1
chr8:60572045-60591969	156	RAB2A
chr7:66771882-66775393	663	RABGEF1
chr1:151185505-151185950	9	VPS72
chr7:5213109-5214704	16	WIPI2
chr3:57575547-57584007	3	ARF4
chr3:52760794-52768604	4	NEK4
chr14:102193462-102195120	387	WDR20
chr1:31337174-31348974	207	ZCCHC17
chr16:69608384-69626528	3	NFAT5
chr13:95115921-95170628	16	ABCC4
chr2:203366876-203427269	13	ABI2
chr21:45116932-45134832	26	ADARB1
chr4:147939824-148023413	13	ARHGAP10
chr1:1489203-1490671	34	ATAD3B
chr3:11340644-11348035	2	ATG7
chrX:78012878-78020398	32	ATP7A
chr9:91296020-91325404	2	AUH
chr11:74132843-74133557	89	C2CD3
chrX:73577406-73584572	84	CHIC1
chr20:49063201-49067280	19	CSE1L
chr5:149525044-149529719	11	CSNK1A1
chr12:123617757-123618837	29	DDX55
chr10:12081471-12113064	29	DHTKD1
chr10:422823-486530	3	DIP2C
chr14:73653169-73671597	23	DNAL1
chr22:31463680-31468302	2	EIF4ENIF1
chr2:72465159-72575668	2	EXOC6B
chr16:647544-648099	2	FAM195A
chr4:15612265-15644708	12	FBXL5
chr2:48328560-48346751	71	FOXN2
chr15:50300788-50304133	10	GABPB1
chr1:45634095-45660377	2	GPBP1L1
chr14:67110139-67122377	13	GPHN
chr7:18829160-18874596	2	HDAC9
chr12:112207873-112208630	5	HECTD4
chr1:20744743-20780540	37	HP1BP3
chr7:2567115-2573488	79	IQCE
chr13:30254104-30283791	2	KATNAL1
chr19:5032865-5041251	29	KDM4B
chr9:111410979-111416352	18	KIAA0368
chr7:152205105-152207428	9	KMT2C
chr1:29216004-29216685	2	MECR

chr2:202382857-202386960	25	nogene
chr11:65435066-65437657	2	nogene
chr19:4356452-4356735	9	nogene
chr22:27873737-27914347	54	PITPNB
chr12:19345841-19354002	8	PLEKHA5
chr16:482524-489000	9	RAB11FIP3
chr3:186804276-186804724	15	RFC4
chr6:130154824-130184623	5	SAMD3
chr3:47425484-47435137	25	SCAP
chr1:53014889-53039046	12	SCP2
chr5:134692601-134693933	5	SEC24A
chr16:70556178-70560591	5	SF3B3
chr6:46655842-46671228	11	SLC25A27
chr17:81275968-81284895	53	SLC38A10
chr9:100498764-100509134	21	TMEFF1
chr21:44076551-44080127	3	TRAPPC10
chr14:90780737-90786328	23	TTC7B
chr9:34220907-34242108	21	UBAP1
chr1:12318071-12321964	18	VPS13D
chr4:84896910-84932363	54	WDFY3
chr2:68131276-68134799	4	WDR92
chr1:155674684-155675096	12	YY1AP1
chr3:141368620-141386937	113	ZBTB38
chr12:122477840-122480311	19	ZCCHC8
chr14:73892026-73909430	46	ZNF410
chr9:32480218-32481497	4	DDX58
chr22:23659864-23683867	68	GUSBP11
chr12:121453119-121455536	14	KDM2B
chr1:154225083-154237136	42	UBAP2L
chr15:90593635-90607478	11	CRTC3
chr1:17022607-17024074	2	SDHB
chr10:127031649-127037816	50	DOCK1
chr9:107306378-107311737	9	RAD23B
chr1:8556474-8614686	9	RERE
chr5:179966805-179970506	8	RNF130
chr12:66529830-66541950	28	GRIP1
chr2:65251026-65265175	4	ACTR2
chr22:38521607-38568289	122	DMC1
chr20:63928334-63929525	2	DNAJC5
chr10:73174686-73193593	45	FAM149B1
chr17:51041500-51079704	11	SPAG9
chr8:42906172-42959314	18	HOOK3
chr3:51463113-51483836	27	VPRBP

chr15:58886974-58887540	76	SLTM
chr5:34169026-34182867	45	nogene
chr2:196912881-196923125	76	PGAP1
chr11:13403167-13445181	19	BTBD10
chr5:43675510-43704400	190	NNT
chr10:35148367-35179276	26	CREM
chr16:66821449-66823600	3	NAE1
chr16:23642954-23665728	2	DCTN5
chr3:47818021-47829134	2	DHX30
chr5:80678926-80761678	12	MSH3
chr17:42345558-42348517	34	STAT3
chr2:169872235-169906164	2	UBR3
chr15:59195460-59214720	21	MYO1E
chr3:196814450-196818156	89	PAK2
chr17:36525227-36528202	31	MYO19
chr11:32097143-32098528	19	RCN1
chr1:70221614-70239520	3	SRSF11
chr9:20758089-20770238	12	FOCAD
chr19:3660965-3662001	167	PIP5K1C
chr12:12179809-12187119	33	LRP6
chr18:9117837-9122681	3	NDUFV2
chr5:751132-842674	5	nogene
chr12:100095666-100103951	17	UHRF1BP1L
chr10:101803734-101804019	6	MGEA5
chr15:50441348-50449485	3	USP8
chr20:32366383-32376182	15	ASXL1
chr11:678693-681089	21	DEAF1
chr9:137574207-137577603	18	DPH7
chr7:34989883-35018596	15	DPY19L1
chr3:47425484-47428670	274	SCAP
chr20:49151581-49174269	61	STAU1
chr3:41789660-41800293	2	ULK4
chr17:59185053-59197789	17	PRR11
chr15:44392127-44451387	34	nogene
chr12:49991997-49992655	274	RACGAP1
chr10:103174857-103181328	5	NT5C2
chr2:175937343-175964589	21	KIAA1715
chr5:73904221-73911575	23	ARHGEF28
chr2:121525844-121530325	9	CLASP1
chr7:8055432-8071131	2	GLCCI1
chr9:124908376-124912026	197	GOLGA1
chr3:183650295-183672484	10	KLHL24
chrX:154031395-154032557	2	MECP2

chr9:36581643-36607673	16	MELK
chr1:198220998-198264235	3	NEK7
chr6:36158647-36196170	12	nogene
chr21:42686191-42699011	10	PDE9A
chr7:103308951-103310475	77	PMPCB
chr9:132296886-132311856	26	SETX
chr1:214401684-214414726	21	PTPN14
chr2:134936840-134947899	23	CCNT2
chr19:52076043-52089002	115	ZNF841
chr6:35410986-35411217	17	PPARD
chr18:163307-192900	39	USP14
chr2:189728659-189744788	43	ANKAR
chr7:5324213-5325248	36	TNRC18
chr19:53423061-53427219	3	nogene
chr7:38817816-38830328	3	VPS41
chr16:1816892-1823037	41	HAGH
chr7:27603137-27649633	27	HIBADH
chr3:136452032-136477412	5	STAG1
chr12:2866392-2868754	6	FOXM1
chr14:88694306-88702632	13	EML5
chr6:143707981-143712183	6	PHACTR2
chr18:9117837-9126907	24	NDUFV2
chr16:29807109-29807681	11	nogene
chr14:31771815-31787873	3	NUBPL
chr7:6111124-6115523	261	USP42
chr4:99034229-99048876	18	METAP1
chr6:141900322-141910011	8	nogene
chr2:118142795-118278471	13	nogene
chr3:170284460-170293508	9	PRKCI
chr18:57731626-57731832	30	ATP8B1
chr2:32377587-32416163	8	BIRC6
chr2:201129728-201140439	11	CFLAR
chr2:197494156-197495376	18	HSPD1
chr11:77374327-77392541	37	PAK1
chr14:29624151-29638904	15	PRKD1
chr8:140890542-140890769	32	PTK2
chr2:150486693-150487455	61	RND3
chr17:78818666-78821061	15	USP36
chr4:87163557-87195690	12	KLHL8
chr21:15799756-15818846	12	USP25
chr13:41865735-41887361	16	VWA8
chr4:39224883-39240334	13	WDR19
chr1:179043714-179050365	13	FAM20B

chr4:146442782-146510049	21	SLC10A7
chr20:3907925-3912634	143	PANK2
chr1:10403070-10413251	14	PGD
chr22:38605253-38623279	13	FAM227A
chr22:30963263-30964106	13	nogene
chr20:44921593-44924256	5	PABPC1L
chr2:53936086-53937540	21	PSME4
chr20:18532663-18542402	16	SEC23B
chr19:16679983-16682508	31	TMEM38A
chr2:23880638-23885829	15	ATAD2B
chr10:125824766-125825185	20	nogene
chr4:109506327-109513856	7	SEC24B
chr1:246876036-246877302	197	AHCTF1
chr11:61410937-61429290	15	CPSF7
chr2:3421880-3443891	5	TRAPPC12
chr18:12794383-12859254	24	PTPN2
chr16:11020192-11051641	53	CLEC16A
chr7:128835325-128855314	29	FLNC
chr11:18291441-18292976	452	HPS5
chr13:77217839-77233263	5	MYCBP2
chr4:118282830-118298938	14	PRSS12
chr7:26844029-26854890	42	SKAP2
chr2:26279993-26284204	17	HADHB
chr19:1241660-1242686	38	nogene
chrX:65051461-65113813	5	nogene
chr16:50214220-50217663	15	PAPD5
chr17:78400734-78401057	24	PGS1
chr2:55647346-55662026	16	PNPT1
chrX:84333758-84361612	11	HDX
chr18:9117837-9119590	7	NDUFV2
chr4:147864845-147881932	48	ARHGAP10
chr4:5856142-5861210	7	CRMP1
chr6:632940-637861	27	EXOC2
chr10:68038092-68067217	10	HERC4
chr2:61178980-61186754	36	nogene
chr20:34096656-34105545	28	EIF2S2
chr3:113788712-113789840	17	ATP6V1A
chr10:92648211-92650517	12	KIF11
chr1:220761657-220770556	35	2-Mar
chr8:127983903-128010444	18	PVT1
chr8:98767242-98774819	17	STK3
chr1:64602805-64641969	11	CACHD1
chr20:34065043-34072330	11	RALY

chr20:6446722-6473655	11	CASC20
chr2:200853902-200855086	5	CLK1
chr17:14033551-14034416	30	COX10-AS1
chr16:58186754-58196844	14	CSNK2A2
chr8:116657214-116658980	4	EIF3H
chr2:20774809-20790398	47	LDAH
chr4:82970326-82979006	39	LIN54
chr4:118620413-118628226	78	LOC729218
chr2:127335869-127339050	2	MAP3K2
chr19:12851933-12858739	11	MAST1
chr4:169576927-169599197	5	NEK1
chrX:84321841-84351815	5	nogene
chr6:129494181-129558166	8	nogene
chr1:110381576-110382205	23	nogene
chr3:197884867-197885322	16	nogene
chr13:24477700-24486305	26	PARP4
chr6:149789953-149796500	8	PCMT1
chr3:138663905-138665203	6	PIK3CB
chr6:43024984-43025329	12	RRP36
chr5:139121034-139127853	126	SIL1
chr7:129202044-129203589	11	SMO
chr7:127976996-127991056	14	SND1
chr1:48395266-48413151	35	SPATA6
chr5:179833031-179833782	11	SQSTM1
chr1:36358238-36361340	4	STK40
chr6:34867224-34867616	19	UHRF1BP1
chrX:155895622-155900587	2	VAMP7
chr2:9344531-9344800	14	ASAP2
chr13:49196876-49198574	11	FNDC3A
chr7:131463216-131478677	8	MKLN1
chr2:233449958-233452060	24	DGKD
chr17:42837648-42839380	57	PSME3
chr22:45692995-45702847	13	ATXN10
chr14:103398950-103405267	346	MARK3
chr11:66486539-66488007	58	DPP3
chr11:857693-861423	33	nogene
chr12:31992377-31996935	2	KIAA1551
chr1:120741807-120793496	50	nogene
chr1:151025514-151028944	4	PRUNE
chr10:7769215-7780307	17	KIN
chr2:241654545-241668685	5	ATG4B
chr5:6623213-6625669	43	NSUN2
chrX:2211488-2212828	15	nogene

chr13:28197184-28239696	14	PAN3
chr2:241324215-241336098	12	2-Sep
chr3:47867245-47872100	19	MAP4
chrX:131755597-131756709	3	FIRRE
chr1:173733712-173734286	30	KLHL20
chr5:134674614-134676125	38	SEC24A
chr10:22326430-22328698	14	BMI1
chr6:31889227-31896989	3	EHMT2
chr10:32919884-32935558	18	ITGB1
chr19:5016256-5041251	39	KDM4B
chr9:34269256-34306441	3	KIF24
chr9:27455132-27455748	15	MOB3B
chr14:67269700-67303599	122	MPP5
chr8:140830471-140864399	605	PTK2
chr2:73985979-74080591	12	TET3
chr12:2909528-2922402	31	TULP3
chr2:238025341-238035940	20	UBE2F
chr9:133806080-133812216	124	VAV2
chr9:86317218-86322475	15	ZCCHC6
chr13:24477700-24501834	34	PARP4
chr17:59015609-59017424	19	TRIM37
chr8:125007390-125009343	4	SQLE
chr22:41893116-41894937	8	SREBF2
chr12:123609938-123616603	14	DDX55
chr2:191360630-191364276	14	MYO1B
chr7:36425701-36443862	2	ANLN
chr17:81667728-81672802	7	CCDC137
chr14:34533443-34536275	3	EAPP
chr1:231270757-231276196	3	GNPAT
chr6:79042842-79060818	669	PHIP
chr10:7367648-7381949	46	SFMBT2
chr10:94474667-94497172	3	TBC1D12
chr13:60439687-60494575	5	TDRD3
chr9:14639895-14674385	59	ZDHHC21
chr12:9680922-9694969	8	CLEC2D
chr6:83934594-83936376	2	CYB5R4
chr7:92448751-92454685	4	GATAD1
chrX:46984883-46998277	11	JADE3
chr10:93347616-93349969	28	MYOF
chr11:20080063-20083179	8	NAV2
chr17:319419-321415	74	RPH3AL
chr14:54737023-54776540	2	SAMD4A
chr22:40777039-40799083	17	SLC25A17

chr1:77725621-77736158	2	USP33
chr8:42966472-42968214	19	HOOK3
chr3:47670657-47676782	54	SMARCC1
chr10:79118914-79216274	3	ZMIZ1
chr2:9535833-9543285	42	ADAM17
chr2:30942200-30955977	13	GALNT14
chr5:6737509-6739852	26	PAPD7
chr3:170291853-170293508	99	PRKCI
chr15:50637421-50639548	52	TRPM7
chr21:15777903-15805258	19	USP25
chr7:5216562-5218014	3	WIPI2
chr18:79126266-79207012	53	ATP9B
chr14:100262303-100274758	17	YY1
chr5:65170616-65188220	20	ADAMTS6
chr8:123380528-123381493	52	ATAD2
chr14:102999564-103008555	2	CDC42BPB
chr1:212863724-212864519	11	FLVCR1
chr16:50078740-50084651	4	HEATR3
chr10:92508727-92537550	1	IDE
chr15:94440175-94458246	2	MCTP2
chr3:179362357-179367592	1	MFN1
chr4:159186641-159241368	11	nogene
chr16:69314617-69315069	26	nogene
chr16:19706591-19712772	11	nogene
chr10:45862985-45964871	11	PARGP1
chr2:88729277-88738076	35	RPIA
chr13:26612960-26613538	1	WASF3
chr2:205158721-205193320	28	PARD3B
chr18:9524593-9525851	413	RALBP1
chr11:94830049-94831541	10	AMOTL1
chr3:98849460-98881767	571	DCBLD2
chr5:60887439-60904873	12	ERCC8
chr1:45642426-45655319	6	GPBP1L1
chr8:74244528-74245175	37	JPH1
chr13:79520809-79539775	9	NDFIP2
chr18:9117837-9124983	45	NDUFV2
chr5:10213490-10227647	188	FAM173B
chr11:29383255-29384112	425	nogene
chr15:43469857-43477759	14	TP53BP1
chr17:81084831-81086580	17	BAIAP2
chr12:111552279-111555919	2936	ATXN2
chr13:31274508-31286819	8	B3GLCT
chr10:68442916-68446413	21	DNA2

chr1:16294782-16315435	26	FBXO42
chr13:30630834-30647057	12	USPL1
chr8:140818276-140864399	480	PTK2
chr1:227193785-227254155	48	CDC42BPA
chr12:42355160-42393689	4	PPHLN1
chr8:123344883-123349444	4	ATAD2
chr1:85350414-85361854	2	DDAH1
chr1:112610840-112616895	43	ST7L
chr6:44932652-45020632	2	SUPT3H
chr4:48556977-48557712	21	FRYL
chr15:55223888-55238491	7	RAB27A
chr4:22389087-22438420	1	ADGRA3
chr7:151117094-151118632	78	AGAP3
chr5:115864566-115870128	2	AP3S1
chr14:103571622-103587364	7	APOPT1
chr19:43625077-43627790	2	CADM4
chr12:28305648-28391411	186	CCDC91
chr10:12215739-12237266	11	CDC123
chr17:66167500-66183307	1	CEP112
chr12:119775785-119776842	27	CIT
chr12:38829378-38848662	34	CPNE8
chr5:138886211-138904441	15	CTNNA1
chr9:123583168-123879021	16	DENND1A
chr13:98897487-98904706	10	DOCK9
chr1:92624175-92639237	1	EVI5
chr15:65551675-65564342	17	HACD3
chr9:19070218-19096769	74	HAUS6
chr7:18585280-18666476	33	HDAC9
chr8:42964310-42986795	1	HOOK3
chr12:12179809-12203400	34	LRP6
chr1:84952242-84965708	73	MCOLN2
chr14:70584816-70597777	29	MED6
chr2:86206105-86207327	33	MRPL35
chr20:62195801-62199257	26	MTG2
chr11:66652276-66657259	24	nogene
chrX:126243345-126250294	86	nogene
chr22:25509182-25510949	1	nogene
chr5:85805306-85856126	1	nogene
chrX:131743095-131785258	33	nogene
chr5:41840450-41862750	53	OXCT1
chr3:53185926-53186340	1	PRKCD
chr12:120221622-120224377	15	PXN
chr5:168500590-168518062	24	RARS

chr14:69453240-69455866	7	SLC39A9
chr8:125003175-125007487	45	SQLE
chr22:46912073-46997709	10	TBC1D22A
chr17:7465710-7467327	18	ZBTB4
chr19:9652952-9653881	32	ZNF562
chr19:37771563-37773751	3	ZNF573
chr5:173944966-173953272	30	CPEB4
chr6:36974060-36974899	28	nogene
chr12:8120907-8138253	2	nogene
chr15:64970113-64983593	2	SPG21
chr1:24649970-24651612	9	SRRM1
chr3:195878252-195888606	109	TNK2
chr3:39069642-39084741	33	WDR48
chr2:61522610-61526521	439	XPO1
chr16:23442476-23445961	32	COG7
chr2:43794456-43805246	15	DYNC2LI1
chr16:89305205-89317075	46	ANKRD11
chr7:43595900-43596113	23	STK17A
chr3:67528131-67609596	11	SUCLG2
chr13:41761127-41787543	10	VWA8
chr5:37437921-37516590	11	WDR70
chr18:21656860-21664230	27	ABHD3
chr7:131375423-131429145	38	MKLN1
chr12:104126113-104128517	3	NFYB
chr20:4899554-4902558	11	SLC23A2
chr16:50749575-50754424	11	CYLD
chr8:108214606-108229195	56	EIF3E
chr11:44107682-44114301	31	EXT2
chr17:75321658-75326020	2	GRB2
chr6:82225476-82234255	2	IBTK
chr16:25161101-25175034	12	LCMT1
chrX:131364748-131369052	354	nogene
chr9:131174086-131178410	14	NUP214
chr6:13632369-13644729	298	RANBP9
chr19:45022052-45025737	29	RELB
chr4:84702352-84721572	3	WDFY3
chr5:38527154-38530666	12	LIFR
chr1:94498601-94499614	21	ABCD3
chr11:62581105-62581701	12	TUT1
chr3:47408490-47408754	12	PTPN23
chr6:107039748-107044332	12	C6orf203
chr5:149505446-149550181	15	CSNK1A1
chr12:108541329-108541648	24	nogene

chr19:40363701-40364080	3	nogene
chr9:131430089-131455166	29	PRRC2B
chr17:21006895-21011309	5	USP22
chr3:47113875-47126663	17	SETD2
chr1:12299201-12308641	93	VPS13D
chr16:58174450-58186856	25	CSNK2A2
chr5:172274432-172274795	16	nogene
chr6:7176307-7176773	37	RREB1
chr15:50449399-50462322	13	USP8
chr6:157036834-157084905	94	ARID1B
chr21:43420029-43420233	14	SIK1
chr9:36603989-36643083	2	MELK
chr7:8003907-8022569	9	GLCCI1
chr9:96541825-96565483	20	CDC14B
chr18:79096475-79207012	17	ATP9B
chr1:75803730-75810807	2	MSH4
chr13:51719197-51751415	2	WDFY2
chr22:31455851-31463133	26	EIF4ENIF1
chr12:69258998-69262562	24	CPSF6
chr10:119041993-119044142	38	EIF3A
chr2:135529637-135530132	18	nogene
chr16:88598177-88609051	18	ZC3H18
chr16:11763477-11775013	13	ZC3H7A
chr1:155521099-155521618	178	ASH1L
chr8:38114191-38135766	2	ASH2L
chr17:61015750-61040892	3	BCAS3
chr15:73702254-73703086	3	CD276
chr19:32915336-32926273	58	CEP89
chr3:33626987-33644903	23	CLASP2
chr17:43489448-43493886	11	DHX8
chr2:54816786-54829477	10	EML6
chr6:53878992-53913969	11	LRRC1
chr17:61960866-61962971	15	MED13
chr17:45081643-45086652	84	NMT1
chr16:71684475-71714801	4	PHLPP2
chr11:63590099-63598163	12	PLA2G16
chr7:66775226-66797506	2	RABGEF1
chr3:128081229-128087808	10	RUVBL1
chr2:200329430-200440784	2	SPATS2L
chr7:100868494-100868866	4	TRIP6
chr10:69483690-69495689	2	TSPAN15
chr19:17153966-17157038	27	MYO9B
chr11:68803999-68812576	63	CPT1A

chr5:108224120-108348530	16	FBXL17
chr17:47618349-47619784	19	NPEPPS
chr7:6580554-6583431	15	ZDHHC4
chr15:65497863-65507355	11	DPP8
chr18:36213365-36220217	8	MOCOS
chr17:78079394-78087093	16	TNRC6C
chr7:129657342-129727365	9	NRF1
chr9:20715321-20823115	31	FOCAD
chr1:93903764-93904588	17	GCLM
chr18:70047970-70065922	4	RTTN
chr2:99206633-99216114	6	nogene
chr3:63912587-63952483	20	ATXN7
chr12:123616510-123618837	14	DDX55
chr13:110622215-110624279	70	CARKD
chr15:36691684-36709961	41	C15orf41
chr19:5653114-5654456	55	SAFB
chr9:15177696-15187035	13	TTC39B
chr1:235480053-235494828	649	B3GALNT2
chr17:63805070-63809659	7	DDX42
chr12:64108931-64115893	31	SRGAP1
chrX:109668100-109669173	17	ACSL4
chr6:38577599-38600008	8	BTBD9
chr21:46314715-46315547	20	C21orf58
chr11:74138719-74139828	7	C2CD3
chr5:131695404-131699002	12	FNIP1
chr1:99747293-99749001	19	FRRS1
chr19:325634-327989	17	MIER2
chr15:25019026-25020761	6	nogene
chr7:140054661-140058034	17	PARP12
chr14:70988566-70995925	179	PCNX
chr11:68551120-68564432	100	PPP6R3
chr7:66797373-66799414	3	RABGEF1
chr10:94441895-94474783	301	TBC1D12
chr10:49945031-49973142	13	TIMM23B
chr15:42775134-42840433	6	TTBK2
chr5:176970741-176982623	12	UIMC1
chr8:130236921-130361771	11	ASAP1
chr22:45692995-45740538	17	ATXN10
chr7:16682779-16697061	31	BZW2
chr2:201133028-201149835	18	CFLAR
chr13:42953947-42970670	6	EPSTI1
chr7:4754458-4757187	18	FOXK1
chr1:230243305-230265367	11	GALNT2

chr10:32021021-32021287	34	KIF5B
chr3:17586352-17672705	2	nogene
chr2:202290322-202295837	69	NOP58
chr19:49864438-49865126	11	PNKP
chr19:52215778-52216663	3	PPP2R1A
chr6:42989610-43006606	51	PPP2R5D
chr3:61748877-61828817	30	PTPRG
chr4:13376544-13381594	2	RAB28
chr6:36524340-36540204	20	STK38
chr13:47948939-47954557	15	SUCLA2
chr14:64055943-64065650	16	SYNE2
chr10:94493364-94511654	19	TBC1D12
chr22:42169846-42215341	4	TCF20
chr5:176943334-176963843	2	UIMC1
chr8:100923954-100925039	114	YWHAZ
chr8:43158574-43173743	11	HGSNAT
chr15:85113872-85121014	49	PDE8A
chr14:58252118-58257998	5	PSMA3
chr19:1816224-1816569	14	REXO1
chr17:48910807-48922937	9	UBE2Z
chr4:142108092-142145996	2	INPP4B
chr17:45437823-45450763	2	PLEKHM1
chr3:50099981-50105709	25	RBM5
chr7:99597935-99610601	16	nogene
chr12:31433154-31442925	20	DENND5B
chr17:39297126-39303639	12	FBXL20
chr4:169537808-169556095	76	NEK1
chr6:110997182-111015856	36	RPF2
chr3:81642780-81670953	12	GBE1
chr3:194433771-194437573	16	ATP13A3
chr5:32143748-32170768	28	GOLPH3
chr5:62437273-62476753	11	IPO11
chr3:51590366-51590559	28	RAD54L2
chr12:51662763-51663093	27	SCN8A
chr7:67067284-67183236	14	TYW1
chr19:14695009-14706773	14	ZNF333
chr22:25509182-25510908	34	nogene
chr5:78165609-78216237	15	AP3B1
chr17:83035766-83048785	26	B3GNTL1
chr21:41226265-41257326	74	BACE2
chr4:142193086-142209026	12	INPP4B
chr14:54737023-54737287	32	SAMD4A
chr3:16286341-16303592	72	OXNAD1

chr7:4983670-4996798	10	RNF216P1
chr4:15636493-15644708	16	FBXL5
chr9:65682765-65720092	18	CBWD5
chr10:68394074-68401737	30	RUFY2
chr17:81845870-81859387	236	P4HB
chr3:11340644-11342279	13	ATG7
chr10:102006539-102040125	2	C10orf76
chr1:28690103-28693045	14	GMEB1
chr5:79396822-79457018	9	HOMER1
chr3:188758631-188760282	25	LPP
chrX:75604917-75624888	3	nogene
chr6:143687678-143712183	15	PHACTR2
chr8:10831664-10834775	7	PINX1
chr3:183717650-183722136	11	YEATS2
chr1:150927703-150930118	25	SETDB1
chr12:26415302-26443650	5	ITPR2
chr7:117099761-117170976	14	ST7
chr17:36986616-37031685	2	AATF
chr17:61776400-61784424	2	BRIP1
chr10:68044403-68073686	2	HERC4
chr12:104381785-104387984	24	nogene
chr2:138559021-138564993	15	SPOPL
chr12:109516764-109526416	2	UBE3B
chr6:82957311-83024038	3	UBE3D
chr15:34890214-34897705	14	AQR
chr10:115461974-115469329	11	ATRNL1
chr11:68363746-68365702	13	LRP5
chr3:131462748-131471279	52	MRPL3
chr19:55341793-55343997	2	SUV420H2
chr10:121790168-121870038	20	ATE1
chr11:86250295-86264263	11	EED
chr7:90372154-90378211	40	GTPBP10
chr11:43750910-43840064	2	HSD17B12
chr16:1747028-1748720	34	MAPK8IP3
chr14:60115718-60125823	275	PCNXL4
chr17:32446944-32464578	5	PSMD11
chr14:102701277-102721084	3	RCOR1
chr3:134175608-134178073	13	RYK
chr1:9553625-9573412	27	SLC25A33
chr7:140355667-140358785	303	SLC37A3
chr16:69235747-69260260	12	SNTB2
chr11:68185780-68190152	60	SUV420H1
chr22:39374315-39376197	13	SYNGR1

chr8:53993667-54000050	26	TCEA1
chr3:196368027-196372042	35	UBXN7
chr16:21968627-21976243	98	UQCRC2
chr10:11597630-11601980	76	USP6NL
chr10:1072115-1105267	8	WDR37
chr20:13528432-13630152	4	TASP1
chr7:139715931-139717015	971	HIPK2
chr10:63380317-63404965	48	JMJD1C
chr11:45979759-45979966	3	PHF21A
chr10:127052680-127061776	38	DOCK1
chr3:32128075-32146734	19	GPD1L
chr9:27044741-27048274	2	IFT74
chr16:21617864-21625115	2	METTL9
chr20:62009051-62014707	17	TAF4
chr1:33013206-33014594	45	AK2
chr22:36493632-36496208	4	FOXRED2
chr19:42236605-42240142	12	GSK3A
chr1:20749722-20773610	233	HP1BP3
chr18:6237963-6264038	41	L3MBTL4
chr19:48802714-48803275	13	nogene
chr7:44624316-44647759	32	OGDH
chr11:61437624-61438362	35	SDHAF2
chr1:109342013-109345881	43	SORT1
chr3:129827802-129880559	21	TMCC1
chr12:104331533-104334332	3	TXNRD1
chr1:246735974-246760227	2	SCCPDH
chr7:32812756-32823221	10	nogene
chr1:180075021-180084178	11	CEP350
chr5:50750150-50788589	11	PARP8
chr10:119577079-119580538	11	TIAL1
chr3:49305714-49325845	11	USP4
chr10:35188199-35207051	6	CREM
chr16:4821379-4832912	5	GLYR1
chr12:32598496-32601423	3	FGD4
chr9:110941420-110942168	81	LPAR1
chr3:196806578-196807914	456	PAK2
chr21:34859473-34880713	3	RUNX1
chrX:65366637-65499989	11	ZC3H12B
chr20:48952704-48963898	47	ARFGEF2
chr6:32120770-32121348	33	ATF6B
chr21:39232176-39250889	23	BRWD1
chr4:80301282-80362884	65	C4orf22
chr19:7605225-7608264	1	CAMSAP3

chr1:91501653-91515876	1	CDC7
chr16:58559803-58560362	101	CNOT1
chr8:67074242-67086110	18	CSPP1
chr3:182947237-182964049	13	DCUN1D1
chr1:21236745-21238244	12	ECE1
chr6:104843222-104852371	14	HACE1
chr7:105038125-105066807	12	KMT2E
chr5:113122683-113260948	1	MCC
chr5:113101738-113102316	1	MCC
chr10:13188880-13192568	11	MCM10
chr22:39403827-39404132	31	nogene
chr8:58601284-58603386	23	NSMAF
chr1:174272413-174305127	63	RABGAP1L
chr4:109449416-109463644	11	SEC24B
chrX:119410189-119419344	25	SLC25A43
chr1:10130499-10130813	12	UBE4B
chr14:93219211-93220411	27	UBR7
chr12:121966954-121975685	1	WDR66
chr19:51581390-51581882	1	ZNF175
chr9:106972004-106974273	4	ZNF462
chr15:68141945-68153695	74	PIAS1
chr1:151224247-151234496	10	PIP5K1A
chr16:68274690-68287871	78	SLC7A6
chr17:1354210-1365058	14	YWHAE
chr1:243304712-243330692	26	SDCCAG8
chr1:213072869-213104569	24	RPS6KC1
chr9:36233919-36249397	30	GNE
chr14:80862756-80900029	83	CEP128
chr12:26711172-26725765	47	ITPR2
chr12:8713810-8754093	33	RIMKLB
chr11:61437624-61438120	8	SDHAF2
chr17:37411268-37444768	25	TADA2A
chr10:11509594-11527567	135	USP6NL
chr1:225412453-225415332	28	LBR
chr3:197015364-197016369	30	MFI2
chr1:154234590-154235291	33	UBAP2L
chr2:32377587-32395593	283	BIRC6
chr10:102351255-102353654	4	GBF1
chr9:33351559-33370014	3	NFX1
chr14:102208602-102209862	7	WDR20
chrX:49113550-49113938	17	GPKOW
chr9:111383210-111391824	9	KIAA0368
chr4:94573350-94586444	53	PDLIM5

chr10:21571974-21595440	23	MLLT10
chr16:2756333-2757945	52	SRRM2
chr2:119920068-119934958	32	PTPN4
chr17:73202999-73203793	48	COG1
chr1:155821473-155822476	1	GON4L
chr5:160035139-160049662	21	TTC1
chrX:155895622-155919880	16	VAMP7
chr22:45700281-45718493	65	ATXN10
chr7:2213211-2225550	24	MAD1L1
chr5:138951170-138951884	14	SIL1
chr8:67299208-67302466	166	ARFGEF1
chr21:39269898-39270433	13	BRWD1
chr1:109661164-109690564	8	GSTM4
chr10:127031649-127043164	34	DOCK1
chr1:29035825-29039426	73	EPB41
chr3:186079816-186105942	25	ETV5
chr3:155922186-155925366	7	GMPS
chr15:28245880-28257261	24	HERC2
chr7:24804314-24834736	6	OSBPL3
chr15:100327375-100331054	13	ADAMTS17
chr2:233434379-233438379	33	DGKD
chr20:5958527-5972037	32	MCM8
chr8:70213902-70216764	479	NCOA2
chr10:18642475-18648645	4	NSUN6
chr20:63775677-63775962	59	ZBTB46
chr10:99955213-99972134	11	DNMBP
chr21:34102834-34142707	13	nogene
chr1:61683445-61723786	3	nogene
chr1:40260838-40268535	5	ZMPSTE24
chr9:85962012-85978381	2	NAA35
chr2:121605700-121606180	505	CLASP1
chrX:53561755-53562244	29	HUWE1
chr15:40386361-40393593	1	KNSTRN
chr11:47308590-47315327	1	MADD
chr8:140275657-140311374	2	TRAPPC9
chr1:155668626-155670464	1	YY1AP1
chr19:1032391-1032696	129	CNN2
chr17:5460938-5463689	55	DHX33
chr3:168309304-168314176	17	EGFEM1P
chr10:69000423-69011015	9	KIAA1279
chr4:3086938-3107423	1517	HTT
chr5:37114959-37120340	20	C5orf42
chr10:96544075-96565426	65	TM9SF3

chr15:42817031-42840433	41	TTBK2
chr3:138681966-138694907	6	PIK3CB
chr20:25209848-25218614	11	ENTPD6
chr1:236859832-236863554	89	MTR
chr9:33582565-33583338	10	nogene
chr8:124320085-124327421	20	TMEM65
chr3:183761506-183762279	38	YEATS2
chr19:10795371-10798572	39	DNM2
chr17:40053297-40053657	7	MED24
chr9:132342689-132349435	24	SETX
chr5:138559863-138567722	39	HSPA9
chr16:5027134-5028185	11	NAGPA
chr5:143041815-143057747	2	ARHGAP26
chr1:21247220-21258839	6	ECE1
chr20:25413789-25441776	31	GINS1
chr7:6414549-6414916	10	nogene
chr19:55098783-55103568	403	PPP1R12C
chr15:34882501-34886661	20	AQR
chr7:13906429-13911307	18	ETV1
chr22:29636750-29642285	370	NF2
chr9:100457757-100471231	20	nogene
chr13:95970111-95999307	132	UGGT2
chr7:65964322-65967907	227	GUSB
chr16:23102318-23108781	3	USP31
chr12:130821688-130827267	6	STX2
chr3:129260400-129260597	37	COPG1
chr10:124706635-124706887	82	FAM53B
chr1:35470862-35479212	42	KIAA0319L
chr22:28296197-28360201	28	TTC28
chr10:125807412-125816525	14	UROS
chr11:75851882-75888903	19	UVRAG
chr9:35228014-35237826	11	UNC13B
chr12:122577671-122582985	15	KNTC1
chr22:25455711-25459515	28	CRYBB2P1
chr7:754588-763974	30	DNAAF5
chr4:152329671-152337936	15	FBXW7
chr11:64685645-64685947	11	NRXN2
chr7:24804314-24809951	17	OSBPL3
chr17:2617196-2638320	15	PAFAH1B1
chr6:111363852-111368028	11	REV3L
chr7:5711760-5730817	123	RNF216
chr1:235465635-235496308	19	B3GALNT2
chr19:10168329-10177367	41	DNMT1

chr1:170016461-170024596	13	KIFAP3
chr3:179706933-179730709	16	USP13
chr15:40645655-40650819	13	CASC5
chr5:131504625-131562047	12	RAPGEF6
chr1:40695174-40695470	29	nogene
chr1:85350414-85367779	63	DDAH1
chr4:25255064-25263849	14	PI4K2B
chr7:44050224-44052941	5	DBNL
chr7:141636959-141649333	429	AGK
chr22:20933778-20934244	1314	CRKL
chr8:100712361-100718280	15	PABPC1
chr17:18865468-18877870	31	PRPSAP2
chr5:173108623-173112379	19	CREBRF
chr2:73946718-73950732	11	DGUOK
chr10:15254720-15284105	17	FAM171A1
chr14:30581075-30593639	92	G2E3
chr5:138557401-138567722	43	HSPA9
chr16:66816580-66823600	12	NAE1
chr13:24490667-24493733	33	PARP4
chr6:169664165-169689308	15	WDR27
chr17:30797310-30804108	11	CRLF3
chr15:65466677-65467223	23	DPP8
chr13:29550203-29554673	73	nogene
chr6:34821652-34834861	26	UHRF1BP1
chr7:139083779-139089759	36	ZC3HAV1
chr16:16086823-16090588	41	ABCC1
chr10:121902390-121924329	87	ATE1
chr6:52464894-52479787	3	EFHC1
chr3:183650295-183665039	7	KLHL24
chr14:71587570-71589370	31	SIPA1L1
chr10:67954594-67966802	151	HERC4
chrX:85046445-85055925	17	APOOL
chr12:121296637-121494665	25	CAMKK2
chr21:46504360-46529183	11	DIP2A
chr2:85027888-85046278	16	KCMF1
chr10:76949141-76970067	2	KCNMA1
chr1:12001400-12002103	167	MFN2
chr8:70121301-70124865	14	NCOA2
chr1:5947103-5961949	99	NPHP4
chr11:74230244-74239256	9	PPME1
chr20:37061102-37065473	93	RBL1
chr6:130288226-130313093	63	SAMD3
chr1:246735974-246740301	21	SCCPDH

chr4:82866807-82867316	38	SEC31A
chr6:75647730-75659407	48	SENP6
chr17:31884423-31887313	9	UTP6
chr11:76004004-76016980	8	UVRAG
chr21:42852254-42876767	11	WDR4
chr19:56407025-56414440	80	ZNF583
chr2:28901986-28929710	3	WDR43
chr1:197672036-197715074	8	DENND1B
chr4:176727777-176729746	142	VEGFC
chr11:86250295-86266216	12	EED
chr9:136049635-136050580	24	NACC2
chr16:14644273-14648592	14	BFAR
chr11:128758114-128768272	35	FLI1
chr5:179705678-179707190	151	CANX
chr9:128422867-128424614	5	CERCAM
chr7:6434793-6436533	12	DAGLB
chrX:135545422-135549805	51	DDX26B
chr11:78493062-78571444	11	NARS2
chr4:2938442-2938625	25	nogene
chr6:130311290-130334845	71	nogene
chr16:69119668-69119968	3	nogene
chr4:76124648-76132719	4	NUP54
chr15:43753823-43761531	3	PDIA3
chr11:86000642-86001158	15	PICALM
chr17:5361207-5363016	3	RABEP1
chr21:44052279-44077784	4	TRAPPC10
chr16:84758715-84775225	17	USP10
chr12:24832722-24836596	58	BCAT1
chr15:64965319-64983593	3	SPG21
chr11:33061529-33072653	16	TCP11L1
chr7:127694827-127721400	16	SND1
chr1:173838189-173840973	21	DARS2
chr9:26923177-26935206	3	PLAA
chr20:36578740-36578966	80	TGIF2
chr7:139137846-139148377	2	TTC26
chr9:74984906-75017448	4	CARNMT1
chr12:8039818-8040165	16	FOXJ2
chr9:96465336-96471094	7	HABP4
chr10:12397268-12403369	3	nogene
chr11:17145667-17155629	12	PIK3C2A
chr20:35716739-35721877	4	RBM39
chr4:151132330-151135132	28	SH3D19
chr19:4365481-4366994	16	SH3GL1

chr14:29632868-29638904	10	PRKD1
chr5:77048935-77055596	17	AGGF1
chr6:83918004-83924592	14	CYB5R4
chr19:33798670-33807007	5	KCTD15
chr6:157592987-157595824	27	ZDHHC14
chr9:131638634-131650949	19	RAPGEF1
chr18:45939599-45952643	18	EPG5
chrX:68111853-68194498	2	OPHN1
chr4:147857552-147879338	16	ARHGAP10
chr1:224411426-224424654	3	WDR26
chr4:148477372-148544584	11	nogene
chr3:27424036-27452498	15	SLC4A7
chr2:15504144-15539356	71	NBAS
chr1:121280749-121284995	3	SRGAP2C
chr13:24484652-24503777	247	PARP4
chr2:214752446-214781509	12	BARD1
chr1:51405904-51408332	47	EPS15
chr1:77858937-77861322	70	FAM73A
chr17:12107789-12113360	3	MAP2K4
chr20:5958527-5963359	60	MCM8
chr1:169323116-169324500	137	NME7
chr7:101311275-101311725	15	nogene
chr2:159001079-159136298	3	TANC1
chr21:43644288-43656737	11	HSF2BP
chr17:67922839-67924589	12	BPTF
chr5:10223958-10227647	13	FAM173B
chr7:152273704-152315338	10	KMT2C
chr1:246186742-246193963	324	nogene
chr6:18212487-18213781	30	KDM1B
chr10:126970701-127018835	14	DOCK1
chr9:124938576-124940155	4	GOLGA1
chr5:95755395-95763620	307	RHOBTB3
chr22:31095302-31097338	24	SMTN
chr19:45852311-45854507	6	SYMPK
chr6:31647590-31649627	13	BAG6
chr12:50427761-50430570	169	LARP4
chr16:25161101-25170805	28	LCMT1
chr4:183828177-183829990	27	nogene
chr11:108186481-108189330	12	NPAT
chr1:77154448-77169395	16	PIGK
chr20:35724569-35732135	15	RBM39
chr1:168231513-168242987	5	SFT2D2
chr9:71750042-71805209	13	TMEM2

chr5:172230897-172234358	11	UBTD2
chr22:30946340-30958694	14	MORC2
chr6:110638648-110670541	3	CDK19
chr10:72834358-72871580	3	MCU
chr2:231206197-231240041	62	ARMC9
chr15:83041727-83056545	23	BTBD1
chr15:49225204-49319805	17	GALK2
chr5:160010499-160051183	17	TTC1
chr5:37721114-37727045	27	WDR70
chr5:154294815-154298079	56	GALNT10
chr9:111428041-111433372	12	KIAA0368
chr18:1269669-1276077	6	LINC00470
chr4:152872451-152888307	21	ARFIP1
chr1:74710097-74719372	18	CRYZ
chr22:38302860-38303248	18	CSNK1E
chr22:38493709-38498573	4	DDX17
chr5:119240418-119244576	18	DMXL1
chr1:225264196-225290082	7	DNAH14
chr5:72989426-72990864	28	FCHO2
chr3:52690591-52691629	23	GNL3
chr16:48252130-48277479	10	LONP2
chr8:97686667-97713769	5	MTDH
chr17:36503096-36505404	4	MYO19
chr19:45639978-45640475	14	nogene
chr4:9565858-9567271	36	nogene
chr3:16269125-16286448	11	OXNAD1
chr10:103438014-103438808	151	PDCD11
chr3:128795359-128807671	220	RAB7A
chr8:52668020-52676571	24	RB1CC1
chr17:80707840-80710103	17	RPTOR
chr10:70868344-70871986	28	SGPL1
chr15:75400729-75422823	14	SIN3A
chr1:156014960-156018368	18	SSR2
chr17:59737452-59817773	34	VMP1
chr12:30728647-30751133	59	CAPRIN2
chr10:130145210-130160997	43	GLRX3
chr13:29522316-29532982	16	SLC7A1
chr6:43524470-43525214	34	XPO5
chr17:47664158-47668410	116	KPNB1
chr20:2116665-2117415	64	STK35
chr2:196656632-196677361	10	CCDC150
chr13:23858859-23881787	26	MIPEP
chr1:31918969-31919658	24	PTP4A2

chr10:68487143-68516843	37	SLC25A16
chr10:103314185-103333952	17	PCGF6
chr2:230358963-230443099	41	SP140L
chr17:41827425-41828952	16	NT5C3B
chr15:100281228-100331054	48	ADAMTS17
chr1:92177810-92180416	13	KIAA1107
chr8:17098457-17120401	26	MICU3
chr12:102205023-102315490	7	nogene
chr1:119986959-120051779	13	NOTCH2
chr12:112446275-112450512	2	PTPN11
chr18:12814202-12831041	33	PTPN2
chr20:49855437-49878063	5	SLC9A8
chr19:19296988-19306076	10	SUGP1
chr8:86368938-86381629	39	WWP1
chr1:33276099-33281811	13	ZNF362
chr6:108928087-108936999	2	ARMC2
chr2:46535812-46537576	2	nogene
chr4:82870324-82881940	50	SEC31A
chr3:194448456-194450276	2	ATP13A3
chr11:17099859-17112666	4	PIK3C2A
chr1:1482544-1520306	906	ATAD3B
chr7:11040671-11111467	11	PHF14
chr17:50982523-50987237	25	SPAG9
chr19:58088687-58090386	13	ZSCAN18
chr7:5989799-5992057	2	PMS2
chr3:125323308-125331238	11	ZNF148
chr7:5287808-5291821	11	SLC29A4
chr19:43732857-43733733	3	SMG9
chr5:14363731-14381252	3	TRIO
chr2:127850589-127853105	31	POLR2D
chr10:87709195-87715843	18	PAPSS2
chr12:123004393-123005548	28	PITPNM2
chr2:10659171-10668723	26	NOL10
chr7:141611194-141621801	19	AGK
chr5:34175444-34182867	31	nogene
chr17:76558373-76558600	25	nogene
chr2:168138087-168140758	24	STK39
chr17:56895342-56899180	4	TRIM25
chr12:12635776-12635974	39	CREBL2
chr5:133091192-133099652	14	HSPA4
chr15:78488646-78497311	8	IREB2
chr9:136908069-136916615	5	TRAF2
chr1:44978314-44981177	4	EIF2B3

chr18:21791373-21844253	3	MIB1
chr18:26052623-26087577	23	SS18
chr4:103343091-103453812	32	nogene
chr5:176322272-176324757	26	SIMC1
chr14:52482229-52491005	11	TXNDC16
chr2:65261246-65265175	8	ACTR2
chr7:27748517-27758133	3	TAX1BP1
chr1:206729971-206731919	4	MAPKAPK2
chr10:94441895-94511654	21	TBC1D12
chr17:51263273-51288203	7	UTP18
chr6:136694139-136698682	172	MAP3K5
chr14:30633946-30653588	36	SCFD1
chr3:138263738-138274544	12	ARMC8
chr19:18596621-18597049	28	CRLF1
chrX:134473358-134498684	379	HPRT1
chr12:19473247-19512465	3	AEBP2
chr11:9403289-9410086	11	IPO7
chr21:45116932-45144701	10	nogene
chr16:89418283-89422305	268	ANKRD11
chr11:34122458-34124400	16	NAT10
chr10:101584601-101586156	15	POLL
chr19:39420625-39422288	5	PLEKHG2
chr9:5787060-5787593	38	ERMP1
chr6:53498606-53522527	23	GCLC
chr2:226838072-226914351	21	RHBDD1
chr9:91852320-91887113	7	nogene
chr3:47701277-47729094	5	SMARCC1
chr10:1084402-1105241	13	WDR37
chr17:66062962-66070001	4	CEP112
chr1:51403418-51406108	13	EPS15
chr15:40387158-40392023	13	KNSTRN
chr18:21765771-21782763	4	MIB1
chr3:115334016-115370176	5	nogene
chr13:72893783-72931267	15	PIBF1
chr6:128240034-128322310	2	PTPRK
chr3:136366942-136377752	6	STAG1
chr20:13528432-13559114	31	TASP1
chr7:5312502-5313863	29	TNRC18
chr2:178535731-178548554	12	TTN-AS1
chr1:150091936-150110627	11	VPS45
chr20:3200000-3203416	10	DDRGK1
chr1:51394380-51409696	2	EPS15
chr19:41268222-41294686	23	HNRNPUL1

chr19:19915279-19933302	12	ZNF93
chr10:7796120-7802854	25	ATP5C1
chrX:16667513-16702941	2	CTPS2
chr6:5431040-5433056	4	FARS2
chr16:13060267-13063698	17	nogene
chr8:96680442-96682076	29	nogene
chr12:122727738-122728748	14	nogene
chr3:16269125-16271722	19	OXNAD1
chr12:49489464-49490731	18	SPATS2
chr9:83669184-83686155	18	UBQLN1
chr20:32366364-32369123	4	ASXL1
chr2:29133654-29167540	2	CLIP4
chr11:34076235-34076642	9	CAPRIN1
chr20:48969146-48976199	7	ARFGEF2
chr7:6594434-6602140	12	C7orf26
chr22:41135812-41146816	3	EP300
chr5:180330699-180338600	5	GFPT2
chr7:25154019-25156368	3	nogene
chr17:1658252-1658763	5	PRPF8
chr7:22219865-22267012	14	RAPGEF5
chr2:110672660-110674365	10	BUB1
chr20:38941958-38948000	41	FAM83D
chr15:68175636-68176654	27	PIAS1
chr11:125572457-125575408	14	EI24
chr8:73582769-73688813	4	STAU2
chr17:47137151-47138891	11	CDC27
chr7:157223253-157231327	15	UBE3C
chrX:46625262-46625978	20	nogene
chr2:86121478-86127991	67	PTCD3
chr6:136601021-136622981	11	MAP3K5
chr16:58599235-58599511	30	CNOT1
chr1:236168705-236205250	7	GPR137B
chr20:32791600-32792770	27	DNMT3B
chr3:196242121-196242561	30	PCYT1A
chr16:48252130-48252365	18	LONP2
chr7:99333324-99354121	6	ARPC1A
chr14:72945883-72947415	4	DCAF4
chr6:149738642-149742570	52	NUP43
chr7:99386684-99388261	38	ARPC1B
chr6:16585779-16657901	10	ATXN1
chr10:28119650-28131691	3	MPP7
chr13:20058574-20067390	4	ZMYM2
chr15:65480221-65481615	25	DPP8

chrX:27301038-27317930	2	nogene
chr3:52930328-52932308	35	SFMBT1
chr18:42027442-42049605	104	PIK3C3
chr11:18000496-18008076	10	SERGEF
chr5:138386021-138400012	20	KDM3B
chr9:36594627-36633200	17	MELK
chr12:6520985-6522037	6	NCAPD2
chr7:4049347-4079584	12	SDK1
chr1:99999260-100017815	12	SLC35A3
chr19:12710620-12711613	37	TNPO2
chrX:16693140-16702941	18	CTPS2
chr8:108234997-108241913	93	EIF3E
chr1:16305802-16316727	2	FBXO42
chr1:224304735-224305166	17	NVL
chr3:149872028-149912083	2	RNF13
chr18:32111753-32129327	17	RNF138
chr11:62585093-62585593	33	nogene
chr12:48088800-48098124	54	SENP1
chr1:25351625-25356853	11	TMEM50A
chr1:151161735-151162100	23	LYSMD1
chr20:56382984-56388202	10	AURKA
chr15:49235850-49319805	11	GALK2
chr4:4240320-4246343	7	TMEM128
chr3:53290790-53292827	13	DCP1A
chr4:124457180-124556510	4	LOC101927087
chr1:155904310-155904804	99	RIT1
chr1:150230570-150231926	118	ANP32E
chr17:56814868-56825321	2	C17orf67
chr2:61203139-61214694	15	USP34
chr5:94909250-94924021	29	MCTP1
chrX:110020351-110067396	6	TMEM164
chr2:37139630-37141701	4	EIF2AK2
chr6:57124733-57128840	36	ZNF451
chr14:75030542-75046589	10	MLH3
chr2:170817048-170818510	12	GAD1
chr1:168235100-168243069	18	SFT2D2
chr1:155867964-155868791	3	SYT11
chrX:115946385-115951383	16	DANT2
chr8:97699753-97723035	14	MTDH
chr2:172562219-172565073	18	PDK1
chr2:70179531-70181997	112	C2orf42
chr9:129120512-129123138	7	PPP2R4
chrX:68063853-68113239	3	OPHN1

chr14:77462159-77465667	2	AHSA1
chr7:99333324-99348959	17	ARPC1A
chr21:36247516-36260732	15	DOPEY2
chr16:30666990-30667296	2	FBRS
chr10:32037253-32037607	18	KIF5B
chr13:75800683-75823873	2	LMO7
chr2:31917925-31950717	2	MEMO1
chr1:172797452-172933643	34	nogene
chr5:115221823-115253255	5	PGGT1B
chrX:24821451-24855481	6	POLA1
chr9:109057190-109060452	5	TMEM245
chr6:107946434-107946810	11	nogene
chrX:85873051-85894284	9	CHM
chr10:1008957-1012662	14	GTPBP4
chr17:64171099-64188415	144	TEX2
chr10:31461036-31514708	15	ZEB1
chr1:153760310-153765063	11	INTS3
chr7:11061790-11062085	46	PHF14
chr3:185598411-185613408	65	SENP2
chr18:49272177-49379758	16	DYM
chr12:51053777-51056502	15	LETMD1
chr17:81596115-81608822	26	NPLOC4
chr14:102870184-102903429	24	TRAF3
chr19:11495949-11498339	15	ZNF653
chr9:20881870-20885230	11	FOCAD
chr3:121509560-121544906	11	POLQ
chr15:100983527-100989398	5	LRRK1
chr1:151224244-151232703	5	PIP5K1A
chr3:12301520-12312453	9	PPARG
chr2:113916883-113942359	65	ACTR3
chr11:86000642-86031611	29	PICALM
chr12:13211468-13214673	14	EMP1
chr22:46997633-47037198	9	TBC1D22A
chr6:158605266-158608750	91	TMEM181
chr11:9998265-10002689	17	SBF2
chr1:244418759-244424387	5	ADSS
chr10:103049706-103076270	2	CNNM2
chrX:71397252-71398737	21	TAF1
chr10:100536410-100546581	3	HIF1AN
chr1:193203794-193236356	23	CDC73
chr22:33761370-33761558	36	LARGE
chr6:30627459-30640422	2	ATAT1
chr1:156333546-156334918	161	CCT3

chr13:21045684-21046230	64	LATS2
chr1:39385433-39388658	6	MACF1
chr16:69083484-69085542	9	TANGO6
chr13:60444682-60467379	19	TDRD3
chr1:32091674-32100562	4	TMEM39B
chr2:78199604-78203818	36	nogene
chr7:149450998-149456037	13	ZNF777
chr7:143383155-143383322	5	ZYX
chr1:51750144-51765981	6	OSBPL9
chr16:1586129-1588024	3	IFT140
chr3:132639491-132644896	16	ACAD11
chr2:218245419-218249921	6	ARPC2
chr2:9356046-9358889	38	ASAP2
chr15:92996956-92998621	15	CHD2
chr2:29152684-29167540	3	CLIP4
chr1:245002120-245022296	24	EFCAB2
chr1:50567076-50584811	12	FAF1
chr7:35638349-35673278	16	HERPUD2
chr5:176352621-176355749	12	KIAA1191
chr11:68032151-68033283	18	NDUFS8
chr2:10788696-10789889	50	PDIA6
chr2:55667861-55673079	13	PNPT1
chr14:50150007-50153173	13	SOS2
chr18:31890734-31909760	32	TRAPPC8
chr3:183797922-183798989	8	YEATS2
chr1:155725381-155729366	7	DAP3
chr6:3076763-3077935	273	RIPK1
chr2:236036560-236049281	42	AGAP1
chr11:93724253-93725831	39	CEP295
chr16:48553538-48562444	43	N4BP1
chr16:27703895-27708920	7	KIAA0556
chr1:149660853-149669310	6	LINC00869
chr15:25019026-25022964	6	nogene
chr5:614469-655584	26	nogene
chr8:140789473-140864399	82	PTK2
chr6:145888019-145894977	144	SHPRH
chr9:96351102-96360221	6	SLC35D2
chr5:160010499-160010858	16	TTC1
chr3:184924861-184930568	7	VPS8
chr4:39224883-39228690	6	WDR19
chr14:35122951-35127611	4	KIAA0391
chr10:91996368-92008275	105	BTAF1
chr2:85368685-85371562	81	ELMOD3

chr5:179888102-179894614	14	TBC1D9B
chr8:98526741-98529652	110	STK3
chr20:34290331-34295585	14	AHCY
chr5:112766325-112801383	15	APC
chr7:122436359-122451475	1	CADPS2
chr16:67066681-67082308	34	CBFB
chr1:240253750-240258032	1	FMN2
chr15:78160091-78162367	10	IDH3A
chr15:44615362-44620403	45	SPG11
chr18:62348167-62360049	15	TNFRSF11A
chr7:67067284-67072389	36	TYW1
chr8:98706466-98722818	20	STK3
chr20:37765196-37768044	62	CTNNBL1
chr22:25663629-25674528	45	ADRBK2
chr8:125003175-125011632	9	SQLE
chr19:52728729-52730005	37	ZNF611
chr19:13955911-13956522	35	DCAF15
chr19:18938352-18939049	33	HOMER3
chr17:56901418-56904488	70	TRIM25
chr6:151361485-151363195	2	nogene
chr4:25188077-25188463	2	nogene
chr8:30786210-30799755	41	PPP2CB
chr11:74775145-74810330	16	RNF169
chr2:31883385-31932135	105	MEMO1
chr4:88061905-88068061	9	PKD2
chr3:47661293-47678311	95	SMARCC1
chr17:76902554-76906187	18	MGAT5B
chr6:167022408-167024894	27	FGFR1OP
chr9:540507-594080	1	KANK1
chr1:44753732-44762658	11	KIF2C
chr8:60533307-60533558	1	nogene
chr15:85097947-85117839	14	PDE8A
chr15:43146492-43169677	10	TMEM62
chr1:12362719-12416827	1	VPS13D
chr12:14423908-14425473	18	ATF7IP
chr11:122712171-122714877	15	nogene
chr1:77641383-77641655	31	ZZZ3
chr22:38299894-38300952	14	CSNK1E
chr6:128322038-128397688	14	PTPRK
chr1:179986168-180003287	13	CEP350
chr6:31741162-31744581	21	MSH5
chr19:3192469-3198897	40	NCLN
chr5:177135086-177204292	15	NSD1

chr3:183833491-183844326	13	PARL
chr17:60269449-60271481	63	USP32
chr1:243545509-243615161	3	AKT3
chr4:147906637-147955374	16	ARHGAP10
chr4:147808065-147822957	3	ARHGAP10
chr19:15268904-15273133	12	BRD4
chr17:44154552-44155411	11	C17orf53
chr22:28694031-28719485	13	CHEK2
chr11:68061952-68081457	2	CHKA
chrX:134473358-134514051	11	HPRT1
chr20:5958527-5973196	24	MCM8
chr17:31155982-31169997	285	NF1
chr16:69695135-69695379	284	NFAT5
chr9:22270998-22301707	2	nogene
chr3:52122378-52125181	7	POC1A
chr8:100274952-100288267	16	RNF19A
chr1:165896189-165905969	18	UCK2
chr14:64490360-64495559	2	ZBTB25
chr17:49796749-49798558	14	KAT7
chr7:65726770-65734350	7	LOC441242
chr15:40183713-40185642	47	BUB1B
chr1:193212064-193236356	11	CDC73
chr12:131838194-131845449	12	MMP17
chr7:72881449-72891110	8	POM121
chr6:43533906-43551453	41	XPO5
chr11:74921213-74937068	1	XRRA1
chr17:19920035-19936430	12	AKAP10
chr2:9279316-9327911	29	ASAP2
chr17:82171700-82178605	3	CCDC57
chr10:382646-390863	29	DIP2C
chr5:13914519-13931244	15	DNAH5
chr12:101908185-101914232	19	DRAM1
chr10:863742-864270	157	LARP4B
chr5:180248972-180280608	55	MAPK9
chr12:112068879-112081134	3	NAA25
chr13:24474531-24478276	24	PARP4
chr6:143765260-143777383	24	PHACTR2
chr18:42015475-42029441	24	PIK3C3
chr1:229286485-229313760	16	RAB4A
chr13:37040404-37051583	84	SUPT20H
chr22:42826939-42835493	29	ARFGAP3
chr11:61806663-61811075	14	FADS1
chr7:102878330-102884312	17	FBXL13

chr7:66554584-66576932	94	GS1-124K5.11
chr6:57919783-57930291	17	GUSBP4
chr16:67939885-67940478	59	LCAT
chr4:150806270-150831976	6	LRBA
chr13:32521372-32527532	29	N4BP2L2
chr17:1045081-1045650	16	nogene
chr15:40698756-40706294	14	RAD51
chr17:319419-327579	38	RPH3AL
chr1:213104453-213129889	3	RPS6KC1
chr3:47037665-47067118	127	SETD2
chr7:17821508-17845694	10	SNX13
chr15:71660529-71660734	5	THSD4
chr4:147633764-147634581	3	TMEM184C
chr17:4282797-4296809	20	UBE2G1
chr18:9593767-9595102	40	PPP4R1
chr1:43824730-43838311	15	ST3GAL3
chr3:142748257-142781029	5	TRPC1
chr11:47750915-47751290	30	FNBP4
chr17:39202316-39203109	13	RPL19
chr7:141601204-141633980	12	AGK
chr10:110124016-110130486	26	ADD3
chr3:15685225-15686309	6	ANKRD28
chr14:32117142-32152528	17	ARHGAP5
chr8:102834188-102838916	16	AZIN1
chr1:111147641-111161296	4	CEPT1
chr12:108657303-108701323	43	CORO1C
chr11:107326923-107336713	13	CWF19L2
chr7:32716717-32726470	2	DPY19L1P1
chr5:179617988-179618323	12	HNRNPH1
chr5:79439009-79447145	10	HOMER1
chr5:62550366-62591672	20	IPO11
chr15:79456097-79458445	20	KIAA1024
chr5:36211843-36227565	96	NADK2
chr9:130222649-130226504	25	NCS1
chr3:170293382-170297393	11	PRKCI
chr3:170293382-170299110	27	PRKCI
chr2:37289355-37290999	19	PRKD3
chr12:112502143-112504796	13	PTPN11
chr20:37056145-37065473	51	RBL1
chrX:136872267-136875585	11	RBMX
chr9:122879754-122883379	10	RC3H2
chr8:42459895-42472654	54	SLC20A2
chr15:58913498-58916999	6	SLTM

chr16:23079945-23082557	34	USP31
chr2:9845219-9854421	23	TAF1B
chr17:1534098-1538952	5	PITPNA
chr7:5740972-5741815	20	RNF216
chr10:121790168-121924329	21	ATE1
chr2:32478634-32482582	35	BIRC6
chr2:174915057-174944943	15	CHN1
chr6:53078798-53082618	3	FBXO9
chrX:19425530-19464406	15	MAP3K15
chr2:134253094-134270550	41	MGAT5
chr21:34125340-34142707	6	MRPS6
chr8:70170201-70174859	3	NCOA2
chr9:91852320-91886770	40	nogene
chr7:72890626-72891110	210	POM121
chr13:29517572-29536202	5	SLC7A1
chr4:122935713-123028395	3	SPATA5
chr12:49489464-49500205	4	SPATS2
chr7:157163809-157187021	25	UBE3C
chr1:165890203-165903279	2	UCK2
chr2:85027888-85035155	19	KCMF1
chr10:73474918-73479517	28	PPP3CB
chr1:28613246-28622165	50	TAF12
chr6:159736257-159748369	14	WTAP
chr4:185258847-185267155	56	SNX25
chr7:111487237-111492514	6	IMMP2L
chr5:168488601-168497348	286	RARS
chr5:171183321-171221840	14	RANBP17
chr19:12145257-12146224	41	ZNF625
chr17:63760913-63764847	12	CCDC47
chr2:218217462-218249921	12	ARPC2
chr4:89020459-89029649	20	FAM13A
chr16:46909683-46909927	13	GPT2
chr19:45258988-45271708	15	MARK4
chr7:158759485-158799072	110	ESYT2
chr14:69203916-69210201	25	EXD2
chr9:127517825-127531778	3	FAM129B
chr7:5603495-5604030	21	FSCN1
chr15:67600684-67646469	17	MAP2K5
chr1:176043185-176136547	24	RFWD2
chr2:190510360-190519022	2	NEMP2
chr8:140735250-140752316	182	PTK2
chr20:35655156-35659014	14	RBM12
chr11:72887469-72889945	89	FCHSD2

chr1:113738109-113758733	6	PHTF1
chr4:122927633-122938249	7	SPATA5
chr9:122285168-122286166	40	MRRF
chr19:49135778-49136122	11	PPFIA3
chr16:53498390-53500329	47	AKTIP
chr1:93567278-93576129	8	BCAR3
chr11:68799217-68812576	12	CPT1A
chr3:197104902-197142768	12	DLG1
chr5:119097978-119105258	23	DMXL1
chrX:130665642-130679748	2	ENOX2
chr2:58198593-58232112	576	FANCL
chr6:5431040-5445504	25	FARS2
chr18:32379211-32393035	13	GAREM
chr1:1804418-1806538	77	GNB1
chr7:139715931-139755259	3	HIPK2
chr2:27485017-27485503	19	IFT172
chr8:97805352-97819238	23	LAPTM4B
chr19:11123173-11131795	4	LDLR
chr3:197858833-197875775	18	LRCH3
chr8:95031994-95052230	4	NDUFAF6
chr10:15128349-15135418	15	NMT2
chr7:75385082-75395503	5	nogene
chr11:17863565-18010139	27	nogene
chr19:36678044-36680479	5	nogene
chr7:83134249-83135656	16	PCLO
chr1:233014664-233057290	5	PCNXL2
chr3:121449314-121487509	59	POLQ
chr8:38266140-38267960	4	PPAPDC1B
chr3:48756378-48807684	13	PRKAR2A
chr5:98779579-98780088	33	RGMB
chr2:151441904-151443709	10	RIF1
chr16:18782780-18789118	5	RPS15A
chr22:42574679-42577962	33	RRP7BP
chr1:161323613-161340655	17	SDHC
chr10:50433475-50466932	108	SGMS1
chr19:38106536-38119882	30	SIPA1L3
chr19:46782382-46784559	17	SLC1A5
chr15:64974601-64976555	4	SPG21
chr14:77512156-77518167	6	SPTLC2
chr1:29154695-29154910	1212	SRSF4
chr1:95138274-95151419	6	TMEM56
chr16:375132-376812	15	TMEM8A
chr15:42829937-42840433	19	TTBK2

chr6:30032988-30035983	14	ZNRD1-AS1
chrX:155506897-155514265	11	TMLHE
chr7:129671428-129727365	7	NRF1
chr7:140334678-140337349	5	SLC37A3
chr20:47246007-47246517	4	ZMYND8
chr17:43201685-43203786	2	NBR1
chr10:16695022-16734064	6	RSU1
chr16:2323522-2329854	10	ABCA3
chr4:51860542-51891851	33	DCUN1D4
chr3:184317320-184317816	6	EIF4G1
chr5:66021321-66028343	19	ERBB2IP
chr10:94507266-94522453	25	TBC1D12
chr14:39313339-39348977	13	CTAGE5
chr3:155906159-155916192	11	GMPS
chr1:247443971-247444821	26	NLRP3
chr14:103778744-103807869	13	PPP1R13B
chr15:72518066-72544964	53	ARIH1
chr5:111374849-111394782	48	CAMK4
chr16:53810139-53888951	12	FTO
chr6:167870385-167891029	3	MLLT4
chr8:73955910-73959818	50	TCEB1
chr15:42983896-42990120	4	UBR1
chr6:169632946-169643786	10	WDR27
chr2:113927344-113934386	11	ACTR3
chr3:43549719-43561402	12	ANO10
chr5:73892051-73901284	3	ARHGEF28
chr12:121268637-121270945	4	CAMKK2
chr17:46050532-46094701	53	KANSL1
chr12:4657747-4685325	25	NDUFA9
chr1:44859580-44859866	23	nogene
chr17:75208526-75218306	12	NUP85
chr12:79820773-79822190	8	PPP1R12A
chr8:94404076-94411315	3	RAD54B
chr19:13993524-13993895	13	RFX1
chr6:33435151-33435613	3	SYNGAP1
chr22:43059383-43064324	5	TTLL1
chr19:36092688-36094164	5	WDR62
chr12:15665755-15681302	7	EPS8
chr5:5441111-5444326	24	ICE1
chr19:40616888-40617225	25	LTBP4
chr1:11121245-11122126	30	MTOR
chr5:131395562-131408207	39	nogene
chr7:44014605-44016523	20	POLR2J4

chr8:142330900-142354763	3	TSNARE1
chr11:110136662-110137414	212	ZC3H12C
chr1:43588730-43589000	2	PTPRF
chr7:92569822-92581603	16	FAM133B
chr2:144317478-144320702	3	nogene
chr17:68306942-68401450	5	ARSG
chr7:2256873-2278305	4	SNX8
chr1:32631826-32634072	14	ZBTB8OS
chr5:126561082-126577211	9	ALDH7A1
chr3:71015548-71053773	23	FOXP1
chr2:43973606-43982434	132	LRPPRC
chrX:65075605-65113813	35	nogene
chr11:17878207-18008076	53	SERGEF
chr1:108154982-108185954	26	SLC25A24
chr6:30061514-30062333	11	ZNRD1
chr3:33412721-33425741	34	UBP1
chr11:46808030-46821268	70	CKAP5
chr8:129871374-129904585	70	FAM49B
chr4:108594669-108598695	6	nogene
chr1:224250211-224268133	67	NVL
chr4:109462900-109473186	4	SEC24B
chr7:93334116-93341575	23	VPS50
chr12:111525191-111552977	34	ATXN2
chr5:55327715-55341771	12	SKIV2L2
chr6:149771161-149796500	28	PCMT1
chr20:17947485-17957037	459	SNX5
chr1:243563719-243695716	20	AKT3
chr1:35745450-35746992	26	CLSPN
chr8:141150833-141151837	3	DENND3
chr16:48258617-48277479	27	LONP2
chr13:23874845-23886506	33	MIPEP
chr11:75404060-75404843	12	nogene
chr10:92893348-92899644	447	EXOC6
chr11:76959076-76998821	32	ACER3
chr8:39221609-39257343	10	ADAM32
chr1:26239761-26244318	56	CEP85
chr15:40009613-40019200	42	EIF2AK4
chr6:98880552-98934879	12	FBXL4
chr8:42354424-42371662	57	nogene
chr14:22906194-22909595	12	RBM23
chr7:66286510-66356871	40	TPST1
chr3:41705056-41717861	28	ULK4
chr7:86925527-86945046	8	KIAA1324L

chr2:241157806-241169867	4	PPP1R7
chr8:22474953-22475624	145	PPP3CC
chr19:5039835-5047669	76	KDM4B
chr2:240523884-240555055	12	ANKMY1
chr15:40386361-40389929	5	KNSTRN
chr16:71676446-71679535	54	PHLPP2
chr16:24927683-24939597	26	ARHGAP17
chr16:16052723-16056295	39	ABCC1
chr5:140438460-140449305	40	ANKHD1-EIF4EBP3
chr15:83030135-83056545	12	BTBD1
chr11:34090178-34092056	46	CAPRIN1
chr2:72134760-72135419	14	CYP26B1
chr10:21904521-21929141	106	DNAJC1
chr7:65974294-65976202	100	GUSB
chr1:201854594-201857194	14	IPO9
chr20:34442207-34462221	7	ITCH
chr18:2937691-2938037	5	LPIN2
chr2:43963587-43982434	14	LRPPRC
chr6:36052698-36059347	56	MAPK14
chr12:87648520-87715112	11	nogene
chr12:6556949-6557642	12	NOP2
chr17:7499038-7499491	603	POLR2A
chr2:28752037-28799433	58	PPP1CB
chr5:170918712-171028989	13	RANBP17
chr22:35759887-35810004	13	RBFOX2
chr2:226837547-226914351	17	RHBDD1
chr6:107912715-107924932	43	SEC63
chr11:57491223-57491862	28	SLC43A1
chr4:67663138-67665292	5	UBA6
chr15:61990999-61991802	42	VPS13C
chr6:169602218-169652008	19	WDR27
chr19:36823730-36827849	8	ZNF790-AS1
chr10:68737844-68742569	287	CCAR1
chr6:143844485-143846224	12	LTV1
chr4:39837855-39849652	12	PDS5A
chr1:54865302-54876047	16	DHCR24
chr17:18865468-18872649	18	PRPSAP2
chr1:180984676-180993425	801	STX6
chr1:93154655-93160226	12	TMED5
chr2:232014834-232024330	29	DIS3L2
chr2:135943377-135961498	3	DARS
chr7:104136780-104165282	45	ORC5
chr5:111669418-111669635	6	STARD4-AS1

chr18:21564202-21568094	57	ESCO1
chr5:109767534-109789527	28	MAN2A1
chr7:135622776-135630470	6	NUP205
chr19:45022052-45028992	56	RELB
chr17:80922723-80940601	16	RPTOR
chr8:97713661-97719189	23	MTDH
chrX:1593443-1595532	25	AKAP17A
chr15:72518066-72561549	100	ARIH1
chr21:39276172-39278813	12	BRWD1
chr3:197115926-197149796	5	DLG1
chr5:131677702-131709272	107	FNIP1
chr12:132813306-132822311	4	GOLGA3
chr14:50446078-50482416	15	MAP4K5
chr17:62029540-62033984	2	MED13
chr7:131375423-131388972	21	MKLN1
chr17:2665371-2670331	13	PAFAH1B1
chr2:108751263-108751994	2	RANBP2
chr12:116749840-116779348	21	RNFT2
chr1:234418083-234427766	10	TARBP1
chr17:15677175-15683157	6	TRIM16
chr6:144793833-144821018	11	UTRN
chr17:1050049-1102027	17	ABR
chr6:52500285-52500764	3	nogene
chr8:119806282-119831721	6	TAF2
chr3:105734008-105751618	10	CBLB
chr4:105424195-105449403	38	PPA2
chr12:110382842-110388623	15	ANAPC7
chr3:136604308-136630981	7	STAG1
chr8:42355515-42361365	13	POLB
chr5:65451474-65473952	1309	ADAMTS6
chr16:75620265-75620820	18	ADAT1
chr1:35966954-35973511	108	AGO3
chr3:141903619-141916020	29	ATP1B3
chr12:56642457-56643958	17	ATP5B
chr8:53811163-53817530	19	ATP6V1H
chr16:297055-298251	20	AXIN1
chr1:235450239-235494828	7	B3GALNT2
chr2:32439507-32443588	15	BIRC6
chr19:55294016-55294397	17	BRSK1
chr9:26842274-26887513	17	CAAP1
chr7:122379367-122393582	6	CADPS2
chr18:68823365-68874785	17	CCDC102B
chr3:56563683-56571302	25	CCDC66

chr3:48167845-48177988	61	CDC25A
chr3:48174283-48177988	49	CDC25A
chr1:227030407-227035607	6	CDC42BPA
chr2:121448952-121457757	15	CLASP1
chr2:29121373-29174445	2	CLIP4
chr3:32733422-32734976	57	CNOT10
chr2:62000700-62000982	112	COMMD1
chr1:160311867-160313167	12	COPA
chr2:36396613-36396787	58	CRIM1
chr20:49058452-49063344	13	CSE1L
chr9:123609435-123879021	5	DENND1A
chr20:62905886-62914406	45	DIDO1
chr10:72343365-72345127	4	DNAJB12
chrX:24062415-24071727	13	EIF2S3
chr1:51461090-51465326	56	EPS15
chr8:28713456-28718207	42	EXTL3
chr17:42706006-42708083	19	EZH1
chr10:124806830-124831463	19	FAM175B
chr5:108867766-108897848	30	FER
chr1:240294821-240438210	15	FMN2
chr9:20862577-20885230	29	FOCAD
chr2:48328560-48359147	17	FOXN2
chr10:130166506-130171636	32	GLRX3
chr9:124898548-124912026	16	GOLGA1
chr16:11875760-11876175	4	GSPT1
chr4:105717592-105729499	26	GSTCD
chr12:112306063-112319742	49	HECTD4
chr10:94590637-94596956	11	HELLS
chr15:63655741-63658718	5	HERC1
chr20:3218516-3218994	34	ITPA
chr10:92637183-92650517	12	KIF11
chr1:225417983-225424089	21	LBR
chr16:72284571-72291413	21	LINC01572
chr4:150735257-150849575	4	LRBA
chr1:235773841-235777308	11	LYST
chr6:52276267-52277198	26	MCM3
chr18:50924097-50932360	34	ME2
chr8:17341362-17349081	21	MTMR7
chr13:77257670-77264002	2	MYCBP2
chr15:71991102-72027793	18	MYO9A
chr1:53800692-53809746	2	NDC1
chr8:95035453-95047127	12	NDUFAF6
chr16:68157868-68183366	28	NFATC3

chr12:32319836-32320393	10	nogene
chr16:89587716-89588100	79	nogene
chr17:42241262-42241552	12	nogene
chr5:160302049-160302544	14	nogene
chr1:5927648-5952836	49	NPHP4
chr7:44618823-44624565	12	OGDH
chr2:200935698-200937966	14	ORC2
chr18:24280841-24312106	13	OSBPL1A
chr3:38183002-38236961	14	OXSR1
chr5:50750150-50778650	12	PARP8
chr13:100257594-100340259	3	PCCA
chr1:233160282-233179175	2	PCNXL2
chr2:44338350-44346390	16	PREPL
chr3:48765003-48794049	16	PRKAR2A
chr2:37274420-37279929	2	PRKD3
chr8:47836337-47839246	31	PRKDC
chr17:59197543-59197789	50	PRR11
chr2:231075510-231087181	106	PSMD1
chr8:60584207-60591969	85	RAB2A
chr2:108769259-108773046	20	RANBP2
chr16:74651919-74661451	57	RFWD3
chr8:86486483-86488639	36	RMDN1
chr15:59030803-59067083	14	RNF111
chr3:134175608-134191974	30	RYK
chr19:5653114-5653420	34	SAFB
chr11:10028451-10031170	3	SBF2
chr6:75621531-75623960	18	SENP6
chr6:75634706-75663518	71	SENP6
chr6:158128107-158144420	11	SERAC1
chr16:70541668-70544533	29	SF3B3
chr5:128131066-128135808	21	SLC12A2
chr2:40139400-40164984	29	SLC8A1
chr20:10449355-10458231	5	SLX4IP
chr16:18839697-18842454	12	SMG1
chr17:64561499-64562966	117	SMURF2
chr7:100355142-100359537	28	STAG3L5P-PVRIG2P-PILRB
chr10:17693221-17696869	2	STAM
chr10:132222640-132228128	13	STK32C
chr1:36358238-36358822	16	STK40
chr1:36358238-36362656	33	STK40
chr3:31576395-31600459	19	STT3B
chr4:4611493-4649542	23	STX18-AS1
chr4:55403235-55417985	24	TMEM165

chr6:158573419-158605347	18	TMEM181
chr7:66240324-66286709	476	TPST1
chr14:75882684-75902224	53	TTLL5
chr17:15553684-15555364	25	TVP23C-CDRT4
chr7:93252652-93259632	29	VPS50
chr5:37697654-37727045	2	WDR70
chr1:40267785-40272035	72	ZMPSTE24
chrX:47982948-47983411	15	ZNF182
chr7:149166157-149166930	37	ZNF398
chr20:32366383-32369883	17	ASXL1
chr10:84417770-84432098	14	CCSER2
chr20:34072065-34076815	12	RALY
chr1:111691333-111709264	24	RAP1A
chr6:36083030-36091043	11	nogene
chr7:102070338-102115273	15	CUX1
chr4:75656923-75662049	10	G3BP2
chr16:4650364-4652824	19	MGRN1
chr2:199323802-199328910	14	SATB2
chr18:76378854-76380303	75	ZNF516
chr1:179118586-179120274	33	ABL2
chr7:140781575-140783157	3	BRAF
chr13:23805949-23870195	30	MIPEP
chr2:26069573-26069800	51	nogene
chr10:45892431-45900426	10	PARGP1
chr8:127932052-127939676	10	PVT1
chr10:67887416-67906937	3	SIRT1
chr4:26683674-26720125	12	TBC1D19
chr1:52465069-52472102	3	ZCCHC11
chr1:23071224-23072197	103	KDM1A
chr3:186797534-186804724	19	RFC4
chr17:59586152-59588053	14	DHX40
chr5:50399858-50411383	18	EMB
chr1:220114313-220140289	14	IARS2
chr1:37980585-37984779	4	SF3A3
chr6:169632946-169638660	21	WDR27
chr4:73120161-73125312	43	ANKRD17
chr1:160237134-160244034	3	DCAF8
chr7:7873286-7884346	4	nogene
chr7:140781575-140794467	15	BRAF
chr18:45876235-45879214	2	EPG5
chr22:26457858-26458577	76	HPS4
chr13:20983223-20983806	5	LATS2
chr10:26705285-26709768	20	PDSS1

chrX:361404-362332	148	PPP2R3B
chr1:31907660-31919658	17	PTP4A2
chr5:72848384-72865729	4	TNPO1
chr11:107308282-107336713	3	CWF19L2
chr9:83679774-83683066	58	UBQLN1
chr2:96797011-96797647	4	CNNM4
chr8:140605523-140605914	30	nogene
chr8:135542653-135582073	18	KHDRBS3
chr6:13639562-13644729	171	RANBP9
chr15:75876186-75879188	3	UBE2Q2
chr5:31448546-31472232	26	DROSHA
chr6:18159882-18166378	136	KDM1B
chr2:37989540-37997514	2	RMDN2
chr2:54813231-54820462	16	EML6
chrX:14850504-14859334	85	FANCB
chr6:36515337-36540204	13	STK38
chr8:95031994-95048558	30	NDUFAF6
chr14:54966474-54987387	19	WDHD1
chr13:30230510-30283791	14	KATNAL1
chr17:5331948-5332152	43	RABEP1
chr1:229486370-229498306	17	NUP133
chr17:30796159-30804108	3	CRLF3
chr20:47623910-47633636	31	NCOA3
chr17:67853939-67854762	54	BPTF
chr11:117161144-117163892	19	PAFAH1B2
chr7:32874706-32875157	24	KBTBD2
chr5:177041125-177050936	112	ZNF346
chr16:16014490-16016621	8	ABCC1
chr19:40705095-40705447	28	ADCK4
chr1:31737428-31740541	2	ADGRB2
chr22:49775590-49777813	14	BRD1
chr7:65755779-65756574	71	CCT6P1
chr15:49137149-49139653	30	COPS2
chr22:38493709-38494802	10	DDX17
chr6:167022408-167044576	23	FGFR1OP
chr13:49168612-49178654	54	FNDC3A
chr20:25417103-25425327	21	GINS1
chr7:50732271-50762014	6	GRB10
chr3:160300882-160307781	14	IFT80
chr4:127663380-127687867	13	INTU
chr7:156734176-156763795	13	LMBR1
chr13:35040932-35058863	2	NBEA
chr1:25318062-25319218	11	nogene

chr15:96334075-96342998	11	NR2F2
chr17:2672654-2676606	10	PAFAH1B1
chr10:49841949-49865381	24	PARG
chr15:43761423-43763206	39	PDIA3
chr11:68600340-68603492	31	PPP6R3
chr7:155672866-155700944	4	RBM33
chr1:32825312-32830067	42	S100PBP
chr10:100507424-100509102	2	SEC31B
chr3:196930812-196932023	20	SENP5
chr10:116901764-116915484	4	SHTN1
chr13:24222818-24251862	29	SPATA13
chr4:39215840-39218105	2	WDR19
chr14:88574692-88578140	120	ZC3H14
chr6:87215902-87245644	14	ZNF292
chr20:25634048-25634827	42	ZNF337-AS1
chr2:111872629-111878971	42	ANAPC1
chr1:23323555-23333631	19	HNRNPR
chr19:48149762-48150210	10	LIG1
chr12:112472943-112478015	26	PTPN11
chr21:29321220-29321514	12	BACH1
chr2:175964371-175995646	8	KIAA1715
chrX:24809897-24930549	3	POLA1
chr12:120565071-120566980	13	RNF10
chr15:42794643-42872758	12	TTBK2
chr5:34182679-34182867	173	nogene
chr15:85113872-85115487	100	PDE8A
chr1:52023490-52028630	24	TXNDC12
chr16:72522552-72570515	16	LINC01572
chr12:64455839-64474390	11	TBK1
chr6:43497880-43503706	62	TJAP1
chr5:37301436-37309267	5	NUP155
chr4:147909731-147946704	12	ARHGAP10
chr8:47293866-47407961	17	SPIDR
chr14:58219693-58223856	3	ACTR10
chr18:12366964-12367382	5	AFG3L2
chr2:235585724-235750488	26	AGAP1
chr1:243545509-243843282	46	AKT3
chr17:4206320-4235890	29	ANKFY1
chr1:93571669-93584121	7	BCAR3
chr16:15581216-15583238	30	C16orf45
chr12:405510-418636	30	CCDC77
chr20:49058452-49070297	2	CSE1L
chr11:70405264-70417123	6	CTTN

chr10:101667885-101673673	13	FBXW4
chr15:83151214-83192308	8	HDGFRP3
chr5:56856599-56865977	12	MAP3K1
chr3:183379296-183389779	147	MCF2L2
chr8:125102013-125102487	3	NSMCE2
chr3:52658198-52679726	8	PBRM1
chr17:78392475-78399537	30	PGS1
chr6:143772257-143777383	2	PHACTR2
chr22:27853536-27873815	16	PITPNB
chr6:3727550-3739161	12	PXDC1
chr3:47037665-47046621	179	SETD2
chr6:157901897-157906212	7	SNX9
chr2:230442936-230449150	5	SP100
chr7:105293570-105297533	48	SRPK2
chr20:32155102-32158514	6	TM9SF4
chr4:183683974-183693147	3	TRAPPC11
chr6:126011252-126013101	16	TRMT11
chr15:77046788-77056255	33	TSPAN3
chr18:12308686-12311053	2	TUBB6
chr6:159736257-159743792	12	WTAP
chr1:32633644-32634110	19	ZBTB8OS
chr3:194433771-194450276	7	ATP13A3
chr9:130076411-130086476	2	GPR107
chr17:39564759-39566892	21	nogene
chr6:63646526-63680161	17	PHF3
chr1:227747691-227759485	25	SNAP47
chr19:19296988-19297344	142	SUGP1
chr17:82082696-82082913	14	FASN
chr2:11784885-11792006	19	LPIN1
chr17:29482196-29498521	2	TAOK1
chr16:27528597-27545523	9	GTF3C1
chr8:129862228-129904585	18	FAM49B
chr7:75052710-75057616	13	WBSCR16
chrX:135545422-135552146	71	DDX26B
chr17:66096250-66132763	21	CEP112
chr8:109271004-109293524	11	NUDCD1
chr9:129128984-129131639	28	PPP2R4
chr19:38917723-38918813	85	SARS2
chr13:35040932-35098405	2	NBEA
chr16:70528872-70529199	2	SF3B3
chr4:2642211-2642827	29	nogene
chr18:62838783-62860601	2	PHLPP1
chr17:37571790-37585430	2	SYNRG

chr2:68490189-68538227	6	APLF
chr22:49794033-49824331	11	BRD1
chr14:44995745-45012075	31	FAM179B
chr6:89771560-89776695	2	MDN1
chr19:40292005-40295341	2	nogene
chr10:45939360-45965855	7	PARGP1
chr12:89466769-89472275	137	POC1B
chr12:45945248-45964188	90	SCAF11
chr16:89883350-89885966	38	TCF25
chr9:79652592-79654075	21	TLE4
chr6:43555835-43567354	96	XPO5
chr5:74796029-74839050	11	FAM169A
chr19:6749933-6750633	19	TRIP10
chr14:50635213-50649389	15	nogene
chr4:139517884-139523435	10	SETD7
chr19:1116947-1117322	3	SBNO2
chr19:45842249-45844200	3	SYMPK
chr19:45702903-45703294	19	QPCTL
chr15:42794643-42840433	38	TTBK2
chr14:34792774-34795765	7	BAZ1A
chr9:35705749-35706111	28	TLN1
chr2:113916883-113931396	25	ACTR3
chr6:135358131-135394896	15	AHI1
chr14:104775651-104792722	2	AKT1
chr12:45378052-45390498	15	ANO6
chr3:194448456-194454392	170	ATP13A3
chr21:39218151-39229436	26	BRWD1
chr1:244552321-244561145	7	C1orf101
chr1:186390710-186391791	14	C1orf27
chr10:77841880-77843706	13	DLG5
chr1:225038693-225097239	2	DNAH14
chr10:99907994-99909146	34	DNMBP
chr15:65490188-65500779	14	DPP8
chr6:52422917-52490350	3	EFHC1
chr21:32453311-32457720	4	EVA1C
chr11:47731373-47734129	11	FNBP4
chr22:35283961-35288407	4	HMGXB4
chr4:3186596-3189093	92	HTT
chr9:100272863-100284603	2	INVS
chr17:5013652-5014837	38	KIF1C
chr1:173755922-173757159	6	KLHL20
chr6:108323824-108402054	2	LACE1
chr6:149683078-149695221	11	LATS1

chr9:36594627-36607673	38	MELK
chr18:21815615-21819646	5	MIB1
chr2:42570436-42644244	10	MTA3
chr4:74174505-74181791	27	MTHFD2L
chr13:77224450-77233263	18	MYCBP2
chr9:131450549-131451118	14	nogene
chr12:102175482-102182627	13	PARPBP
chr13:32687133-32696902	11	PDS5B
chr1:28473553-28476291	57	PHACTR4
chr11:85996825-86003451	435	PICALM
chr11:17094260-17097264	3	PIK3C2A
chr14:49653989-49655843	15	POLE2
chr6:43583006-43583651	3	POLH
chr8:47849153-47855373	2	PRKDC
chr8:140846598-140890769	91	PTK2
chr20:18543018-18551175	91	SEC23B
chr7:80818298-80828745	62	SEMA3C
chr4:139529030-139533366	33	SETD7
chr17:35362738-35363826	17	SLFN11
chr4:20491760-20539584	30	SLIT2
chr17:2282646-2283735	14	SMG6
chr22:41866830-41868792	17	SREBF2
chr7:117098612-117138532	2	ST7
chr20:49135832-49154071	9	STAU1
chr17:82884144-82900731	161	TBCD
chr2:223881941-223884749	12	WDFY1
chr22:21700975-21710908	20	YPEL1
chr20:62854015-62857635	23	TCFL5
chr1:235612123-235612503	7	nogene
chr22:24338385-24347176	9	SPECC1L-ADORA2A
chr6:170323078-170348323	3	FAM120B
chr11:94445809-94461036	17	MRE11A
chr5:65992709-65994864	36	ERBB2IP
chr13:19705661-19709599	33	PSPC1
chr5:109781395-109823837	82	MAN2A1
chr14:58291634-58293926	32	PSMA3-AS1
chr3:3137259-3144710	118	TRNT1
chr22:42831548-42847632	20	ARFGAP3
chr13:98883812-98885092	6	DOCK9
chr1:92563641-92636336	25	EVI5
chr7:5603256-5605384	2	FSCN1
chr7:65964322-65976202	31	GUSB
chr1:27668225-27669346	10	IFI6

chr6:18159882-18162924	14	KDM1B
chr7:152330600-152358675	97	KMT2C
chr2:134250049-134270550	7	MGAT5
chr2:42640172-42682589	12	MTA3
chr13:41369089-41372830	10	NAA16
chr17:59912683-59914703	9	RPS6KB1
chr17:18330554-18353817	28	SHMT1
chr16:68274690-68275249	232	SLC7A6
chr3:51684412-51684884	13	TEX264
chr8:139885878-139910300	27	TRAPPC9
chr2:61485767-61490776	139	XPO1
chr4:38054198-38103157	69	TBC1D1
chr17:30481142-30492753	148	GOSR1
chr3:12516610-12519197	13	TSEN2
chr2:239139683-239163923	7	HDAC4
chr19:50216184-50217771	3	MYH14
chr5:96729152-96737947	11	CAST
chr17:61919399-61925099	201	INTS2
chr12:116096668-116111512	115	MED13L
chr9:100441905-100442356	203	MSANTD3-TMEFF1
chr16:68181474-68191775	30	NFATC3
chr15:75414204-75422823	17	SIN3A
chr5:122795265-122808355	37	SNX2
chr17:63237813-63267873	5	TANC2
chr1:162576776-162655374	11	UAP1
chr17:49311311-49312042	89	ZNF652
chr9:85669484-85681335	34	AGTPBP1
chr7:92294888-92319512	69	ANKIB1
chr1:235252729-235260752	15	ARID4B
chrX:101140665-101148161	8	CENPI
chr10:68963290-68969121	24	DDX21
chr2:43794456-43801709	31	DYNC2LI1
chr5:95906522-95932713	50	ELL2
chr3:56660730-56671692	10	FAM208A
chr2:222639579-222648795	11	FARSB
chr4:102714437-102734571	3	MANBA
chr3:127710575-127781895	12	MGLL
chr12:6983001-6988369	57	nogene
chr5:170916464-170953702	12	RANBP17
chr22:39313750-39314106	16	RPL3
chr17:80619626-80643810	4	RPTOR
chr3:43332168-43343478	11	SNRK
chr20:17947485-17961326	11	SNX5

chr11:9429673-9431003	732	IPO7
chr1:26296918-26298062	6	UBXN11
chr3:33663444-33696933	45	CLASP2
chr10:14932853-14935564	10	DCLRE1C
chr2:69354488-69374113	27	GFPT1
chr15:22516875-22539247	78	HERC2P2
chr15:78476187-78483434	15	IREB2
chr14:59456437-59461173	3	nogene
chr18:12794383-12862744	6	PTPN2
chr3:119503531-119517315	165	TIMMDC1
chr3:41931843-41954807	17	ULK4
chr3:44929305-44959460	11	ZDHHC3
chr12:31392266-31409384	4	DENND5B
chr13:25307805-25315467	23	NUPL1
chr2:54050957-54057360	13	nogene
chr10:27067156-27086609	10	ANKRD26
chr11:83206057-83211430	33	ANKRD42
chr7:2544908-2547478	2	BRAT1
chr11:126018329-126019765	10	CDON
chr12:108652271-108657423	14	CORO1C
chr3:197090911-197105005	2	DLG1
chr12:32708152-32718763	59	DNM1L
chr4:152332595-152337936	5	FBXW7
chr5:180316340-180336577	19	GFPT2
chr12:112219385-112228811	13	HECTD4
chr1:220102128-220102777	11	IARS2
chr17:46050532-46082542	18	KANSL1
chr10:77403861-77447869	10	KCNMA1
chr3:120334861-120335953	20	LRRC58
chr1:54205017-54205410	13	MRPL37
chr8:66572481-66576375	10	MYBL1
chr2:15275483-15277101	18	NBAS
chr9:33293652-33295427	6	NFX1
chr19:49331616-49332320	15	nogene
chr3:195248036-195248401	90	nogene
chr19:48911148-48913564	9	NUCB1
chr11:85981128-86007583	10	PICALM
chr17:28349082-28351800	3	POLDIP2
chr11:70349922-70356254	19	PPFIA1
chr14:22926132-22927660	437	PRMT5
chr9:112250928-112262599	28	PTBP3
chr1:100906851-100918127	12	SLC30A7
chr6:35857260-35872728	23	SRPK1

chr3:129827802-129857405	4	TMCC1
chr22:40117054-40251200	2	TNRC6B
chr2:47005921-47011435	16	TTC7A
chr11:74906238-74927488	11	XRRA1
chr16:72811576-72812038	65	ZFHX3
chr5:178860996-178867071	2	ZNF354B
chr9:114001995-114016784	3	ZNF618
chr9:111574733-111597432	6	PTGR1
chr4:67530814-67544195	5	CENPC
chr17:19909180-19924517	29	AKAP10
chr17:76328716-76332435	12	PRPSAP1
chr9:20881870-20933103	18	FOCAD
chr15:66481754-66485191	36	MAP2K1
chr22:20554935-20555148	53	MED15
chr18:50941168-50941468	13	nogene
chr18:7838335-7839401	16	nogene
chr1:224411426-224413335	4	WDR26
chrX:23730526-23733217	13	ACOT9
chr12:69253050-69262562	14	CPSF6
chr14:102043874-102044698	4	DYNC1H1
chr5:137985256-138018514	4	FAM13B
chr1:42227882-42278784	12	FOXJ3
chr6:141798082-141831645	8	nogene
chr22:23814100-23814695	6	nogene
chr11:117152440-117163892	588	PAFAH1B2
chr13:29523265-29536202	18	SLC7A1
chr13:98474820-98482321	36	STK24
chrX:71284396-71285097	10	NONO
chrX:103449273-103461354	2	nogene
chr15:76728594-76733384	15	SCAPER
chr11:46758922-46760784	4	CKAP5
chr13:59861406-59924870	18	DIAPH3
chr12:63626468-63647365	3	DPY19L2
chr18:36142280-36160015	23	ELP2
chr9:95897848-95907271	2	ERCC6L2
chr14:52780678-52784664	12	GNPNAT1
chr20:34768463-34792563	18	NCOA6
chr3:52743351-52752336	19	NEK4
chr3:23954536-23968023	15	NR1D2
chr5:168557422-168564065	16	PANK3
chr15:85064369-85067204	47	PDE8A
chr9:37489314-37493703	4	POLR1E
chr4:105370836-105399164	11	PPA2

chrX:107639294-107642490	9	PRPS1
chr3:61989624-62003497	76	PTPRG
chr5:168493893-168497348	5	RARS
chr12:123315372-123317356	2	SBNO1
chr1:225987895-225988388	17	SDE2
chr3:72792915-72842402	16	SHQ1
chr9:100320264-100327962	5	TEX10
chr15:29766265-29772166	47	TJP1
chr3:129827802-129832826	42	TMCC1
chr18:31870371-31871120	36	TRAPPC8
chr6:148941278-148964563	16	UST
chr12:68816811-68824651	127	MDM2
chr11:76513385-76523291	3	C11orf30
chr1:227139575-227147559	87	CDC42BPA
chr18:21560858-21566206	13	ESCO1
chr1:91343429-91353299	3	HFM1
chr11:20402918-20464546	4	PRMT3
chr8:96295097-96309622	4	PTDSS1
chr4:122927633-122979386	46	SPATA5
chr15:25393745-25411971	17	UBE3A
chr17:60343956-60344238	14	nogene
chr12:42374862-42393689	18	PPHLN1
chr10:31817787-31831800	10	ARHGAP12
chr11:129164318-129267361	11	ARHGAP32
chr8:61637946-61684188	33	ASPH
chr18:68836748-68897682	11	CCDC102B
chr9:120477349-120518645	11	CDK5RAP2
chr16:11886470-11898035	11	GSPT1
chr5:43294055-43297673	11	HMGCS1
chr2:86151296-86159671	4	IMMT
chr17:47664158-47678413	21	KPNB1
chr12:132147280-132148895	11	NOC4L
chr7:73545516-73545868	5	nogene
chr12:11121009-11124323	12	nogene
chr19:32709184-32711905	19	NUDT19
chr1:28473553-28480604	67	PHACTR4
chr8:22474953-22498112	11	PPP3CC
chr5:145764930-145823134	31	PRELID2
chr3:47610065-47622341	8	SMARCC1
chr4:185247293-185288082	328	SNX25
chr1:150497529-150499293	8	TARS2
chr14:90676522-90730196	13	TTC7B
chr19:34443843-34467014	11	UBA2

chr9:83661776-83669327	72	UBQLN1
chr2:135772419-135776351	10	UBXN4
chr19:29971192-30012531	6	URI1
chr4:143212317-143214943	11	USP38
chr7:130040950-130049144	15	ZC3HC1
chr1:112653597-112654671	25	CAPZA1
chr9:128508875-128509097	27	GLE1
chr3:195381008-195392147	74	ACAP2
chr5:65260599-65273447	206	ADAMTS6
chr16:30067233-30070029	27	ALDOA
chr12:111655565-111660675	13	BRAP
chr6:42934474-42938207	4	CNPY3
chr4:83272086-83279114	24	COQ2
chr9:123676719-123879021	10	DENND1A
chr3:32528445-32533097	2	DYNC1LI1
chr1:28035543-28058094	256	EYA3
chr12:50437734-50441643	71	LARP4
chr10:21670448-21682257	34	MLLT10
chr3:184161301-184161848	14	nogene
chr14:31561405-31562215	3	NUBPL
chr9:131174054-131189131	2	NUP214
chr2:55558774-55568363	6	PPP4R3B
chr7:105462620-105468463	124	PUS7
chr2:218580560-218584721	11	RQCD1
chr12:108543027-108549214	11	SART3
chr22:30406341-30407603	4	SEC14L2
chr7:99045697-99047882	3	SMURF1
chr17:20245925-20253586	3	SPECC1
chr1:162576776-162599355	36	UAP1
chr19:32615657-32622619	12	ANKRD27
chr2:215325240-215326138	14	ATIC
chr4:173195594-173248440	3	GALNT7
chr1:45633488-45642499	41	GPBP1L1
chr1:155926675-155929840	19	KIAA0907
chr9:33328580-33347117	21	NFX1
chr9:111914604-111917806	5	UGCG
chr16:53308685-53318340	3	CHD9
chr14:39345903-39348977	14	CTAGE5
chr8:108239957-108241913	3	EIF3E
chr3:131109802-131133956	7	NEK11
chr15:73570149-73573795	33	NPTN
chr7:75507366-75511114	16	PMS2P3
chr11:20426765-20464546	2	PRMT3

chr4:13342238-13381594	2	RAB28
chr1:183512836-183517820	11	SMG7
chr10:96544075-96551411	10	TM9SF3
chr6:32126116-32127750	16	ATF6B
chr2:108846737-108856970	36	CCDC138
chr2:232811243-232812491	7	GIGYF2
chr11:68590659-68603492	14	PPP6R3
chr9:105348357-105361330	3	SLC44A1
chr17:42345558-42348539	35	STAT3
chr21:37108391-37126143	54	TTC3
chr8:6455142-6499929	19	MCPH1
chr12:19462509-19484053	24	AEBP2
chr17:6659924-6661413	5	ALOX15P1
chr1:184477464-184507682	37	C1orf21
chr12:75312847-75326527	13	CAPS2
chr9:152033-172172	32	CBWD1
chr7:133274981-133289116	20	EXOC4
chr4:41661419-41671594	134	LIMCH1
chrX:64348659-64359527	2	MTMR8
chr11:17356650-17357001	13	NCR3LG1
chr1:155562555-155562821	35	nogene
chr10:13849756-13849937	18	nogene
chr2:95832385-95834565	2	nogene
chr12:106376358-106380139	15	POLR3B
chr14:50150007-50150230	49	SOS2
chr6:121170404-121242339	41	TBC1D32
chr8:38114191-38119363	24	ASH2L
chr1:32030297-32045390	27	KHDRBS1
chr18:45899403-45904117	5	EPG5
chr5:93958776-94053227	11	FAM172A
chr13:51365702-51395483	12	INTS6
chr20:35842572-35871829	9	PHF20
chrX:115633999-115645099	2	PLS3
chr5:134666822-134671886	30	SEC24A
chr10:126996747-127000307	70	DOCK1
chr1:235830225-235833667	36	LYST
chr3:36996618-37007063	13	MLH1
chr10:68142939-68145526	13	MYPN
chr7:155706859-155707380	21	RBM33
chr16:67046151-67066798	11	CBFB
chr20:49074784-49089746	17	CSE1L
chr2:223884647-223899070	12	WDFY1
chr1:179131310-179133374	366	ABL2

chr9:110194874-110198574	25	nogene
chr3:136713457-136720173	12	nogene
chr9:124321458-124327445	23	NEK6
chr17:66496200-66514466	35	PRKCA
chr19:53148680-53153793	26	ZNF347
chr21:33340998-33345360	17	IFNAR1
chr6:131160058-131169273	45	AKAP7
chr9:91325317-91356155	6	AUH
chr1:201852103-201858994	15	IPO9
chr14:102343820-102344389	9	nogene
chrX:68046993-68064177	11	OPHN1
chr7:106097689-106099282	19	SYPL1
chr14:22947170-22955176	47	HAUS4
chrX:101126708-101148161	77	CENPI
chr11:94485927-94490965	30	MRE11A
chr19:10931067-10931864	18	nogene
chr16:74923925-74942826	14	WDR59
chr4:7793680-7800653	10	AFAP1
chr10:95637092-95643206	38	ALDH18A1
chr7:6599828-6602140	11	C7orf26
chr7:23604085-23611553	36	CCDC126
chr16:1459106-1465338	27	CLCN7
chr10:5959753-5960567	57	IL15RA
chr11:67215339-67219403	9	KDM2A
chr19:34194477-34196763	123	LSM14A
chr22:28515626-28519320	11	nogene
chr3:195923811-195929081	19	nogene
chr4:145159502-145171720	165	OTUD4
chr7:140041643-140047007	26	PARP12
chr3:52576540-52579199	40	PBRM1
chr15:77252366-77284030	10	PEAK1
chr1:151027232-151028944	29	PRUNE
chr20:35713018-35725155	96	RBM39
chr8:86484871-86507112	19	RMDN1
chr20:13528432-13569586	67	TASP1
chr3:129880308-129880562	16	TMCC1
chr15:56704120-56707306	3	ZNF280D
chr20:5176647-5178956	15	CDS2
chr11:65504325-65505019	11	nogene
chr2:29120029-29174525	17	nogene
chr1:234418083-234472811	10	TARBP1
chr4:38014508-38021718	21	TBC1D1
chr11:85280533-85285365	5	DLG2

chr7:22960255-22977421	26	FAM126A
chr1:2393207-2403589	2	nogene
chr7:44624316-44666851	3	OGDH
chr15:38299372-38339895	5	SPRED1
chr3:69039550-69044595	15	TMF1
chr7:67009582-67018143	3	TYW1
chr21:42850040-42852324	17	WDR4
chr2:218656149-218656463	95	ZNF142
chr7:149266334-149278527	3	ZNF783
chr5:82252561-82254325	11	ATG10
chr18:69946733-69947433	8	CD226
chrX:129557324-129559001	7	OCRL
chr9:128492425-128492805	36	ODF2
chr11:47420263-47420727	13	PSMC3
chr1:203847183-203847687	25	ZC3H11A
chr12:106999550-107000082	14	CRY1
chr15:50643339-50648885	29	TRPM7
chr14:88574692-88609803	6	ZC3H14
chr1:245927930-245929937	12	SMYD3
chr6:116698703-116716318	11	KPNA5
chr18:22990936-22996462	8	RBBP8
chr11:46433473-46435037	35	AMBRA1
chr12:48933576-48941188	5	ARF3
chr8:130358016-130401970	138	ASAP1
chr10:12760947-12769799	15	CAMK1D
chr4:182891288-182915575	24	DCTD
chr5:108883395-108946222	5	FER
chr12:42097441-42119171	18	GXYLT1
chr6:129491900-129492483	27	LAMA2
chr5:5292566-5293674	6	nogene
chr10:34251601-34269899	12	PARD3
chr1:214451804-214464957	30	PTPN14
chr1:30964673-30981405	11	PUM1
chr6:125045210-125076858	4	RNF217
chr10:68355352-68364113	23	RUFY2
chr4:82680706-82705413	16	SCD5
chr4:82854902-82857130	2	SEC31A
chr5:1057470-1065478	28	SLC12A7
chr16:66513730-66517877	21	TK2
chr6:126011252-126021280	13	TRMT11
chr17:68432215-68432835	23	nogene
chr8:23447679-23449997	12	ENTPD4
chr3:155893517-155916192	24	GMPS

chr17:64522701-64526200	5	CEP95
chr17:44805253-44805577	28	nogene
chr1:202428854-202434768	10	PPP1R12B
chr17:64583460-64586170	102	SMURF2
chr20:44972077-44995257	13	STK4
chr4:176711498-176731524	13	VEGFC
chr2:29133654-29157347	29	CLIP4
chr2:190900563-190931637	41	GLS
chr11:32927156-32935435	12	QSER1
chr10:70152262-70161931	71	SAR1A
chr10:110596397-110598290	537	SMC3
chr17:31982998-31988497	307	SUZ12
chr17:59012327-59041898	19	TRIM37
chr9:137180180-137180384	17	ANAPC2
chr10:92606264-92607237	17	KIF11
chr3:184327215-184331606	21	EIF4G1
chr4:112253207-112260862	26	AP1AR
chr11:120473049-120477526	123	ARHGEF12
chr14:80831142-80838278	6	CEP128
chr4:55475962-55482828	16	CLOCK
chr11:61356529-61357828	19	CYB561A3
chr10:126970701-127012374	17	DOCK1
chr4:139125163-139139497	131	ELF2
chr4:38931450-38935790	5	FAM114A1
chr9:112405623-112418958	2	HSDL2
chr15:90466000-90466436	15	IQGAP1
chr12:116237467-116237705	39	MED13L
chr10:101792838-101807901	3	MGEA5
chr6:31411071-31505871	37	MICA
chr9:126340507-126395697	91	MVB12B
chr16:68121986-68123121	974	NFATC3
chr4:10085992-10087508	11	nogene
chr7:129657345-129690546	7	NRF1
chr16:15044333-15048099	6	NTAN1
chr17:5410702-5416682	10	NUP88
chr7:44624316-44647534	28	OGDH
chr8:100717772-100718280	20	PABPC1
chr4:39910243-39925933	15	PDS5A
chr19:42117249-42122576	15	POU2F2
chr18:8076454-8088851	172	PTPRM
chr8:47279901-47294030	12	SPIDR
chr8:47273336-47440542	3	SPIDR
chr21:32368402-32375483	26	URB1

chr7:75061206-75070769	2	WBSCR16
chr11:110136662-110159490	187	ZC3H12C
chr20:25632157-25634827	308	ZNF337-AS1
chr11:108258985-108267342	92	ATM
chr2:25623517-25628384	13	DTNB
chr14:70988566-71009724	4	PCNX
chr1:229539455-229549434	28	ABCB10
chr17:43521368-43522226	40	DHX8
chr1:206595717-206612357	14	EIF2D
chr6:108569561-108664889	30	FOXO3
chr14:92806735-92809519	10	GOLGA5
chr6:25515674-25520337	14	LRRC16A
chr3:185437446-185451395	7	MAP3K13
chr5:139871720-139880974	6	NRG2
chr11:3744508-3753408	15	NUP98
chr17:8253858-8258199	28	PFAS
chr15:64418540-64425631	38	TRIP4
chr1:39853969-39857417	147	TRIT1
chr6:158312050-158314268	49	TULP4
chr1:52293452-52303925	2	ZFYVE9
chr11:36633283-36648155	13	C11orf74
chr6:135438374-135457713	23	AHI1
chr12:22523425-22527892	9	C2CD5
chr10:68963290-68970350	15	DDX21
chr10:72340788-72341170	318	DNAJB12
chr16:68025414-68049550	62	DUS2
chr5:145587324-145591128	33	nogene
chr2:237682624-237683495	18	nogene
chr17:76443581-76443967	12	nogene
chr7:44674410-44676149	67	OGDH
chr3:133612388-133624175	7	TOPBP1
chr19:55404060-55404478	5	UBE2S
chr19:4941527-4941931	136	UHRF1
chr9:94278757-94293069	67	ZNF169
chr5:151062126-151065131	21	TNIP1
chr14:29712937-29725674	16	PRKD1
chr9:107302034-107311737	13	RAD23B
chr16:71756018-71764726	19	AP1G1
chr2:61482956-61490776	104	XPO1
chr9:91694319-91722580	11	nogene
chr9:132896788-132897183	10	TSC1
chr5:84060299-84066606	24	EDIL3
chr12:101780151-101790057	11	GNPTAB

chr13:46368041-46368815	11	KIAA0226L
chr11:12204249-12208139	11	MICAL2
chr9:128968505-128973249	12	NUP188
chr7:77920278-77922778	10	PHTF2
chr9:94064733-94095454	20	PTPDC1
chr1:173978487-173993135	11	RC3H1
chr17:32173830-32183272	49	RHOT1
chr11:224077-233215	17	SIRT3
chr5:128131066-128141981	65	SLC12A2
chr7:127976651-127991056	6	SND1
chr16:69384579-69385486	14	TERF2
chr19:12702822-12703801	11	TNPO2
chr9:37126311-37302231	12	ZCCHC7
chr9:37126311-37219098	11	ZCCHC7
chr9:106972004-107003426	12	ZNF462
chr10:119836895-119849576	3	MCMBP
chr7:103300177-103304490	2	PMPCB
chr8:66572481-66602523	6	MYBL1
chr5:38903881-38919062	3	OSMR
chr4:121833230-121839696	11	BBS7
chr5:123573392-123575963	13	CSNK1G3
chr17:51217333-51225183	6	MBTD1
chr5:112766325-112780903	10	APC
chr6:118894026-118895339	2	nogene
chr2:222927016-222934687	14	ACSL3
chr16:74632522-74637970	42	RFWD3
chr5:153997802-154034967	93	FAM114A2
chr6:73440208-73445747	11	MB21D1
chr4:106171094-106236808	9	TBCK
chr7:92343023-92371607	24	ANKIB1
chr3:32534510-32545965	3	DYNC1LI1
chr8:47927193-47927890	26	PRKDC
chr11:93736140-93736751	4	TAF1D
chr3:97895958-97900485	12	CRYBG3
chr2:23887831-23895970	12	ATAD2B
chr11:119232447-119232729	5	CBL
chr10:127052680-127127764	6	DOCK1
chr19:11513055-11514221	351	ECSIT
chr10:73174686-73210438	25	FAM149B1
chr6:35619095-35646850	57	FKBP5
chr10:836402-845055	49	LARP4B
chr12:12124564-12130893	4	LRP6
chr16:72349045-72391215	18	nogene

chr1:28011305-28011711	16	nogene
chr1:233054267-233095863	7	PCNXL2
chr8:132836539-132844318	9	PHF20L1
chr12:68650399-68654252	108	RAP1B
chrX:72275052-72276234	2	RPS4X
chr19:38099961-38101226	10	SIPA1L3
chr4:102304316-102315830	215	SLC39A8
chr17:2282646-2292977	3	SMG6
chr11:64194188-64197974	37	STIP1
chr1:224404429-224424654	2	WDR26
chr14:50321482-50322719	13	ATP5S
chr8:67103036-67118821	18	CSPP1
chr4:6967303-7004924	8	TBC1D14
chr17:75812111-75813878	32	UNK
chr14:30902535-30913657	12	STRN3
chr10:150096-180128	5	ZMYND11
chr19:38130497-38130772	55	SIPA1L3
chr22:40835559-40850880	10	ST13
chr2:61495454-61498913	62	XPO1
chr1:165679441-165695397	18	ALDH9A1
chr19:4430548-4433539	13	CHAF1A
chr11:9193493-9207632	24	DENND5A
chr19:45854174-45854507	68	SYMPK
chr16:14593300-14604226	87	PARN
chr10:119072889-119073577	13	EIF3A
chr9:12610904-12631607	6	nogene
chr4:189855885-189937333	5	nogene
chr9:98018288-98026451	20	nogene
chr19:3656404-3662001	10	PIP5K1C
chr5:69174876-69175537	565	CCNB1
chr4:142402937-142433830	6	INPP4B
chr18:77066297-77105286	16	MBP
chr1:198277960-198297240	31	NEK7
chr1:229466633-229487613	26	NUP133
chr1:64777622-64814840	34	RAVER2
chr20:35716739-35725155	348	RBM39
chr6:35872562-35891013	4	SRPK1
chr9:99915177-99928843	86	STX17
chr1:228394736-228395252	27	TRIM11
chr19:29985222-30005708	18	URI1
chr12:1754257-1772961	31	ADIPOR2
chr3:15028585-15030452	23	NR2C2
chr7:74356409-74357477	65	CLIP2

chr16:48252224-48277479	3	LONP2
chr11:108070095-108078240	5	CUL5
chr16:84099052-84102107	16	MBTPS1
chr21:43004325-43031617	13	PKNOX1
chr11:32587011-32595013	4	EIF3M
chr17:82248874-82255577	3	CSNK1D
chr9:33026474-33026990	33	DNAJA1
chr22:32478980-32493281	48	FBXO7
chr2:30525586-30533314	275	LCLAT1
chr17:8257806-8258199	17	PFAS
chr20:35858301-35863400	21	PHF20
chr16:11721576-11736233	79	TXNDC11
chr20:41267462-41269083	57	ZHX3
chr3:50252099-50253184	94	GNAI2
chr2:36509982-36522313	38	CRIM1
chrX:44961283-45020730	12	KDM6A
chr1:243683196-243843282	23	AKT3
chr12:110029477-110030758	3	ANKRD13A
chrX:2618739-2626642	21	CD99P1
chr5:65528451-65529199	60	CENPK
chr16:4405490-4413404	23	CORO7
chr11:61410937-61411937	13	CPSF7
chr15:76283756-76295737	53	ETFA
chr6:108319616-108324048	10	LACE1
chr19:2076810-2078679	10	MOB3A
chr5:145438071-145473315	20	nogene
chr20:62256056-62263691	108	OSBPL2
chr10:87864453-87933251	56	PTEN
chr8:140789473-140803650	220	PTK2
chr6:106602873-106622969	7	RTN4IP1
chr17:2372488-2372926	14	SGSM2
chr2:128164729-128173939	7	UGGT1
chr6:99464603-99510230	16	USP45
chr16:88627621-88628854	14	ZC3H18
chr8:134509618-134590355	6	ZFAT
chr17:4051970-4064829	15	ZZEF1
chr11:46433473-46443598	21	AMBRA1
chr2:97576378-97580568	11	ANKRD36B
chr15:72544819-72582187	2	ARIH1
chr15:40602914-40615378	2	CASC5
chr10:32451591-32462703	31	CCDC7
chr9:33271151-33278225	71	CHMP5
chr11:68799217-68807638	2	CPT1A

chr10:73156274-73156655	7	ECD
chr7:2374527-2377075	92	EIF3B
chr4:5729306-5756362	2	EVC
chr12:116949617-116964854	15	FBXW8
chr10:13663452-13666325	3	FRMD4A
chr9:124885359-124888396	2	GOLGA1
chr5:135345967-135370142	10	H2AFY
chr4:82355303-82359639	2	HNRNPD
chr2:197523623-197550386	11	MOB4
chr1:236824118-236826896	27	MTR
chr14_GL000194v1_random:92152-92446	4	nogene
chr19:51689771-51690145	10	nogene
chr7:74563821-74564062	13	nogene
chr2:241140520-241143074	41	PASK
chr15:85113376-85126374	3	PDE8A
chr9:131436619-131455166	2	PRRC2B
chr12:69755383-69756663	157	RAB3IP
chr5:179562546-179569425	3	RUFY1
chr6:130312977-130341122	16	SAMD3
chr1:246726891-246740301	90	SCCPDH
chr2:168012633-168065381	68	STK39
chr6:158573419-158607343	2	TMEM181
chr17:59075646-59081219	14	TRIM37
chr14:90644047-90655110	10	TTC7B
chr1:83904078-83933766	2	TTLL7
chr17:21015751-21028674	24	USP22
chr6:135318518-135323324	21	AHI1
chr17:81053667-81086580	13	BAIAP2
chr19:49139667-49140088	13	PPFIA3
chr2:10046149-10048595	4	KLF11
chr5:33453288-33456227	7	TARS
chrX:70341798-70376210	8	KIF4A
chr2:231206197-231262398	14	ARMC9
chr13:23805949-23809924	20	MIPEP
chr9:100126382-100131954	5	INVS
chr5:177164850-177165266	6	nogene
chr8:95252187-95263774	14	C8orf37
chr21:41854565-41860372	49	PRDM15
chr4:82824554-82827632	7	SEC31A
chr6:159682473-159688242	92	SOD2
chr20:37777344-37803048	11	CTNNBL1
chr16:53810139-53826491	32	FTO
chr4:127669035-127687867	101	INTU

chr11:9428539-9431003	113	IPO7
chr4:128121825-128162317	12	LARP1B
chr6:89743149-89750532	23	MDN1
chrX:150699194-150718700	19	MTMR1
chr8:77578034-77582405	4	nogene
chr12:49441729-49461037	4	SPATS2
chr12:104311289-104325429	2	TXNRD1
chr1:21001198-21002808	171	EIF4G3
chr2:235750353-235799522	8	AGAP1
chr1:3682332-3707791	12	TP73
chr1:155346382-155347904	27	ASH1L
chr17:63435085-63437560	2	CYB561
chr5:133070373-133074126	13	HSPA4
chr7:129657342-129671543	16	NRF1
chr13:24441845-24449817	24	PARP4
chr11:596922-598502	17	PHRF1
chr17:37576340-37585430	25	SYNRG
chr17:15545784-15553829	80	TVP23C-CDRT4
chr16:74915869-74918008	11	WDR59
chr6:43546570-43551453	14	XPO5
chr3:104764378-104855451	15	nogene
chr5:223481-228333	3	SDHA
chr17:20204332-20205936	34	SPECC1
chr5:64788950-64804386	57	CWC27
chr17:82084512-82084953	5	FASN
chr15:41707727-41713496	23	MGA
chr2:203380207-203395780	6	ABI2
chr3:195357690-195392147	11	ACAP2
chr3:57575547-57584464	16	ARF4
chr4:152337801-152350124	15	FBXW7
chr1:92247061-92264638	3	GLMN
chr14:103654563-103654825	24	KLC1
chr9:22466115-22468710	16	nogene
chr14:71045132-71076419	18	PCNX
chr7:6387211-6405755	11	RAC1
chr10:7220410-7227937	17	SFMBT2
chr17:37576340-37579434	3	SYNRG
chr1:243199059-243211964	76	CEP170
chr19:32432715-32439910	13	DPY19L3
chrX:155033402-155051801	3	FUNDC2
chr13:30240459-30241681	5	KATNAL1
chr2:159745769-159748903	16	7-Mar
chr3:136326802-136329984	14	PCCB

chr1:151427822-151430841	27	POGZ
chr11:82838386-82860117	4	PRCP
chr3:12599690-12604289	23	RAF1
chrX:20162963-20167747	33	RPS6KA3
chr4:75977645-75982037	17	SDAD1
chr16:29879863-29885749	6	SEZ6L2
chr2:109347673-109347949	34	SH3RF3
chr15:57063749-57091891	5	TCF12
chr17:18292644-18294785	34	TOP3A
chr3:51470928-51496780	11	VPRBP
chr17:15700207-15708247	30	ZNF286A
chr5:177041125-177044533	62	ZNF346
chr7:99493039-99494631	212	ZNF394
chr1:39853969-39862935	16	TRIT1
chr2:229512782-229547092	14	DNER
chr1:155921374-155923687	14	KIAA0907
chr11:36081652-36098461	52	LDLRAD3
chr7:2200261-2222754	11	MAD1L1
chr14:86028368-86070967	5	LOC101928767
chr7:149266334-149267222	13	ZNF783
chr2:55558774-55573777	15	PPP4R3B
chr11:102350843-102363716	20	BIRC2
chr2:72718102-72731245	26	EXOC6B
chr10:15816834-15834616	18	FAM188A
chr17:39424626-39432016	17	MED1
chr16:14586317-14617650	4	PARN
chr10:17603559-17604047	114	HACD1
chr17:64521401-64522895	5	CEP95
chr11:118556905-118559864	13	IFT46
chr13:30255446-30284882	13	KATNAL1
chr2:39331916-39337581	347	MAP4K3
chr4:118724590-118728053	3	nogene
chr16:66598177-66598840	39	nogene
chr18:24332941-24366966	6	OSBPL1A
chr1:151440927-151442205	8	POGZ
chr7:42926535-42927459	20	PSMA2
chr10:27117575-27121448	23	YME1L1
chr12:69592058-69593613	3	CCT2
chr4:4073032-4087393	14	nogene
chr11:107308282-107311232	3	nogene
chr1:151262160-151264028	29	PSMD4
chr2:9517900-9527954	15	ADAM17
chr2:201749711-201757759	3	ALS2

chr7:139146983-139148377	3	TTC26
chr5:65224319-65300131	4	ADAMTS6
chr5:149505446-149525171	4	CSNK1A1
chr15:41069569-41072058	38	INO80
chr5:102990271-103009866	9	PAM
chr13:30227411-30240565	12	KATNAL1
chr8:17558294-17562110	13	SLC7A2
chr1:145747994-145784596	6	RNF115
chr9:83659779-83669327	18	UBQLN1
chr22:21770502-21770906	16	nogene
chr16:89098590-89133262	11	ACSF3
chr2:32543240-32549481	17	BIRC6
chr1:151683355-151693483	18	SNX27
chr10:31461036-31495838	14	ZEB1
chr18:47868322-47896809	20	SMAD2
chr18:63350887-63362868	31	KDSR
chr1:93305387-93325895	26	LOC100131564
chr10:93313019-93316813	16	MYOF
chr12:104927972-104928694	142	SLC41A2
chr3:9365015-9366985	60	THUMPD3
chr1:244477540-244499079	6	C1orf101
chrX:41660438-41671530	31	CASK
chr2:120285873-120289757	12	RALB
chr12:26990449-27003390	40	TM7SF3
chr11:77355667-77379994	7	PAK1
chr3:57847196-57864716	17	SLMAP
chr18:9522159-9522509	7	RALBP1
chr20:44051305-44054526	12	TOX2
chr1:243613670-243664883	16	AKT3
chr8:61618977-61644632	50	ASPH
chr7:39987598-39997664	14	CDK13
chr9:120487293-120491477	16	CDK5RAP2
chr2:37032628-37041292	8	HEATR5B
chr17:8553954-8576831	11	MYH10
chr7:66805139-66805396	36	RABGEF1
chr4:106171094-106212835	103	TBCK
chr8:130159863-130169067	41	ASAP1
chr15:40165052-40170681	2	BUB1B
chr13:31941307-31946181	14	EEF1DP3
chr20:34093674-34098633	25	EIF2S2
chr1:193096647-193101204	9	GLRX2
chr6:149623102-149638560	105	KATNA1
chr10:99919709-99920185	13	nogene

chr11:3691346-3700839	4	NUP98
chr11:74775145-74817714	31	RNF169
chr6:144789193-144793991	2	UTRN
chr11:44124788-44130138	10	EXT2
chr12:56746044-56751195	14	PRIM1
chr20:49279103-49280570	14	ZFAS1
chr6:87214997-87218731	10	ZNF292
chr5:69566966-69572550	4	GTF2H2B
chr7:45797477-45805289	19	nogene
chr9:136461176-136464562	10	SEC16A
chr4:888846-904779	10	GAK
chr19:41268222-41279176	41	HNRNPUL1
chr8:135542653-135557587	13	KHDRBS3
chr1:213071005-213129889	5	RPS6KC1
chr2:241601895-241603079	35	THAP4
chr16:2328452-2329854	76	ABCA3
chr16:16007815-16009901	23	ABCC1
chr1:35747906-35748604	60	CLSPN
chr2:207555627-207570321	9	CREB1
chr18:49329572-49379758	21	DYM
chr9:137728348-137754291	6	EHMT1
chr3:47498285-47511226	15	ELP6
chr14:88754511-88766867	32	EML5
chr9:93476255-93498886	113	FAM120A
chr12:116928022-116964854	49	FBXW8
chr2:190895151-190902026	2	GLS
chr4:6804522-6842204	18	KIAA0232
chr14:20996208-20996711	38	METTL17
chr5:65792453-65797693	3	NLN
chr5:155014042-155014637	6	nogene
chr1:220761655-220784071	19	nogene
chr19:57876305-57885415	33	nogene
chr17:77293431-77293827	14	nogene
chr8:47929091-47934090	8	PRKDC
chr4:42001615-42078325	19	SLC30A9
chr1:24666814-24670315	59	SRRM1
chr1:32810590-32811057	103	YARS
chr8:28963810-28970519	15	HMBOX1
chrX:96948934-96965207	11	DIAPH2
chr12:22659013-22684556	11	ETNK1
chr17:17171717-17180088	25	MPRIP
chr2:15488893-15534641	30	NBAS
chr7:128997388-129018157	12	TNPO3

chr8:108229069-108241913	11	EIF3E
chr2:111233627-111239996	92	nogene
chr2:202952558-202961426	2	CARF
chr1:155725381-155727738	24	DAP3
chr3:192335360-192360538	10	FGF12
chr1:117414712-117442325	39	MAN1A2
chr1:99668622-99675498	3	nogene
chr7:135596188-135596636	11	nogene
chr3:27433915-27452498	42	SLC4A7
chr17:29666866-29703062	15	SSH2
chr6:44136348-44139759	7	TMEM63B
chr3:78938600-78938927	42	ROBO1
chr6:169582835-169613656	18	WDR27
chr12:88106674-88107098	26	CEP290
chr6:33676767-33677089	12	ITPR3
chr3:33619602-33644903	150	CLASP2
chr15:67164894-67166853	31	SMAD3
chr2:111344060-111480061	33	nogene
chr3:56663540-56673725	21	FAM208A
chr20:17952586-17957037	3	SNX5
chr4:107644801-107701285	20	PAPSS1
chr19:47040443-47042904	62	NPAS1
chr5:171199669-171221840	23	RANBP17
chr7:134221778-134249076	18	LRGUK
chr7:30456720-30457042	13	NOD1
chr22:38355889-38356270	6	nogene
chr8:124567646-124568914	14	nogene
chr12:108701123-108718954	31	CORO1C
chr10:67988662-68014186	17	HERC4
chr11:9408485-9417148	13	IPO7
chr7:131429032-131478677	10	MKLN1
chr2:9237832-9239953	2	nogene
chr17:66773986-66788979	10	PRKCA
chr2:151440026-151443709	5	RIF1
chr22:50459254-50464917	34	SBF1
chr1:246556572-246557534	45	TFB2M
chr14:91999239-92000108	3	TRIP11
chr6:150944485-150965037	11	MTHFD1L
chr22:38298785-38314169	3	CSNK1E
chr19:49357251-49360081	3	TEAD2
chr2:30955617-30966302	17	GALNT14
chr16:4811169-4812248	92	GLYR1
chr19:48633443-48633655	9	DBP

chr17:31163185-31182665	20	NF1
chr16:57123413-57127903	6	CPNE2
chr11:66803994-66828364	9	C11orf80
chr16:16052723-16071729	2	ABCC1
chr20:34882793-34883615	2	ACSS2
chr11:78113885-78121174	45	ALG8
chr12:110013124-110030758	10	ANKRD13A
chr2:23823257-23823569	58	ATAD2B
chr6:84185186-84186623	46	CEP162
chr16:68038005-68061113	10	DUS2
chr14:101983018-101988852	10	DYNC1H1
chrX:152320808-152364596	2	GABRA3
chr12:101780151-101796762	10	GNPTAB
chr11:18402839-18403811	18	LDHA
chr10:13191298-13195269	19	MCM10
chr10:13172375-13189080	19	MCM10
chr4:139695196-139704196	27	MGST2
chr15:52425829-52433285	48	MYO5A
chr4:103536345-103552602	20	nogene
chr7:5432243-5434126	2	nogene
chr5:32724697-32739030	10	NPR3
chr14:90312393-90318113	2	NRDE2
chr1:149959683-149964349	45	OTUD7B
chr1:149964221-149977576	12	OTUD7B
chr15:72206727-72208891	15	PKM
chr7:43986600-43988247	18	POLR2J4
chr9:122989296-122990213	28	RABGAP1
chr7:66783674-66805396	2	RABGEF1
chr1:150951364-150962719	19	SETDB1
chr16:70532478-70535420	28	SF3B3
chr3:47701277-47714490	2	SMARCC1
chr16:70219911-70222480	40	SMG1P7
chr7:38207446-38219660	32	STARD3NL
chr7:90225366-90232484	12	STEAP2
chr10:69196991-69199197	17	SUPV3L1
chr15:50637421-50663046	9	TRPM7
chr14:90744791-90786328	28	TTC7B
chr5:37605063-37703087	2	WDR70
chr1:52281660-52303925	11	ZFYVE9
chr6:29890263-29944616	65	HLA-H
chr19:1356323-1358464	5	MUM1
chr6:73723250-73736508	173	CD109
chr7:21020867-21023309	26	LINC01162

chr3:142743484-142762313	4	TRPC1
chr15:100609706-100612427	84	ASB7
chrX:2719660-2726373	15	CD99
chr12:31392266-31392696	48	DENND5B
chr13:41252644-41260915	52	MTRF1
chr1:147286297-147287733	219	CHD1L
chr15:44484223-44490999	27	CTDSPL2
chr15:65463760-65481615	5	DPP8
chr2:27642428-27647943	10	GPN1
chr16:1743331-1748720	17	MAPK8IP3
chr14:23475170-23477560	6	NGDN
chr12:87707646-87715112	13	nogene
chr2:44490785-44558013	3	nogene
chr3:56674436-56674937	21	nogene
chr16:2757471-2896771	3	SRRM2
chr16:88598177-88599948	3	ZC3H18
chr2:45976428-45980381	30	PRKCE
chr2:113729400-113735858	29	SLC35F5
chr11:86007541-86031611	327	PICALM
chr3:101656103-101672213	8	ZBTB11
chr8:61618977-61684188	55	ASPH
chr1:193141849-193152444	42	CDC73
chr10:122393180-122417968	14	PLEKHA1
chr10:122393180-122413045	7	PLEKHA1
chr3:140963126-140974005	16	SLC25A36
chr10:86472207-86473973	37	WAPL
chr14:96943516-96945382	41	nogene
chr12:26980565-27003390	13	TM7SF3
chr17:46050532-46067667	19	KANSL1
chr11:68569747-68576043	17	PPP6R3
chr2:175080659-175118369	5	ATF2
chr2:32524887-32549481	13	BIRC6
chr15:76274411-76292513	2	ETFA
chr20:34096656-34103565	13	EIF2S2
chr17:44174462-44174859	7	nogene
chr15:50941441-50950169	10	AP4E1
chr18:79110354-79126375	21	ATP9B
chr6:18236451-18249839	5	DEK
chr21:46484756-46511614	18	DIP2A
chr15:49575010-49590007	3	FAM227B
chr19:14479411-14492912	2	GIPC1
chr9:86040738-86052591	38	GOLM1
chr1:26884124-26886133	4	GPN2

chr8:33488377-33489139	8	MAK16
chr13:48079843-48083428	3	MED4
chr17:77176197-77176681	12	nogene
chr16:29793402-29794613	12	nogene
chr1:229495494-229496047	2	NUP133
chr3:138663905-138684803	13	PIK3CB
chr7:5986758-5992057	19	PMS2
chr7:75474703-75486247	12	POM121C
chr15:75517258-75527261	20	PTPN9
chr7:66712166-66712613	15	RABGEF1
chr3:11809516-11810897	11	TAMM41
chr2:43498832-43508780	10	THADA
chr5:113539073-113542503	20	YTHDC2
chr16:50287911-50288350	27	ADCY7
chr1:235480053-235489268	10	B3GALNT2
chr5:171868609-171900100	35	FBXW11
chr7:39781769-39789544	12	LINC00265
chr5:80778719-80792844	9	MSH3
chr3:123707753-123708895	5	MYLK
chr19:17172335-17172963	18	MYO9B
chr12:85685057-85713416	19	nogene
chr12:68715626-68732739	10	NUP107
chr5:149820432-149826885	18	PPARGC1B
chr1:32668224-32669563	31	RBBP4
chr3:27400763-27424152	17	SLC4A7
chr15:66535932-66540210	5	ZWILCH
chr8:100287500-100288267	20	RNF19A
chr6:15593058-15627475	4	DTNBP1
chr16:89895037-89900794	113	TCF25
chr1:36258665-36259484	14	THRAP3
chr13:21381433-21391854	16	ZDHHC20
chr5:140438460-140445975	80	ANKHD1-EIF4EBP3
chr5:78156257-78216237	12	AP3B1
chr17:40163480-40164166	14	CASC3
chr5:138566625-138569049	12	HSPA9
chr5:177269601-177288925	2	NSD1
chr1:52438219-52461769	2	ZCCHC11
chr2:231206199-231235381	3	ARMC9
chr18:36681435-36709391	20	FHOD3
chr10:96544075-96559736	14	TM9SF3
chr6:37929958-38116739	5	ZFAND3
chr6:149603267-149604782	9	KATNA1
chrX:112804946-112815877	13	AMOT

chr3:43598531-43605863	62	ANO10
chr2:24135118-24161613	19	FAM228B
chr18:62348167-62354534	18	TNFRSF11A
chr2:68513079-68545312	3	APLF
chr4:147854675-147866816	12	ARHGAP10
chr15:72695176-72716850	51	BBS4
chr7:74338447-74339004	52	CLIP2
chr5:10386993-10394785	56	6-Mar
chr16:71667177-71672322	20	PHLPP2
chr16:68880547-68902504	2	TANGO6
chr21:42873550-42876767	32	WDR4
chr10:42816598-42817494	16	BMS1
chr15:89273376-89276891	4	FANCI
chr3:121790072-121828972	11	IQCB1
chr4:17961902-17972885	294	LCORL
chr3:188882976-188883465	11	nogene
chr1:16620920-16624877	10	nogene
chr6:106640348-106643090	11	QRSL1
chr7:151470570-151498180	11	RHEB
chr1:47272075-47282459	11	STIL
chr9:106972004-106984409	49	ZNF462
chr17:16143605-16146548	20	NCOR1
chr16:14606483-14617650	4	PARN
chr11:9961956-9989595	15	SBF2
chr8:141175199-141178196	5	DENND3
chr1:162566011-162566348	138	UAP1
chr10:77794007-77796594	13	DLG5
chr8:43021954-43022920	15	nogene
chr8:118379559-118403227	55	SAMD12
chr20:21354788-21357792	3	XRN2
chr4:22389087-22392690	10	ADGRA3
chr11:108838445-108841476	16	DDX10
chr10:68437010-68470163	31	DNA2
chr1:169319158-169324500	24	NME7
chr12:121804723-121806105	3	SETD1B
chr1:154225083-154236611	9	UBAP2L
chr3:81577924-81586190	40	GBE1
chr16:48277337-48303305	264	LONP2
chr3:12574791-12576741	38	MKRN2
chr4:131409-218778	92	ZNF718
chr12:116945363-116964854	13	FBXW8
chr17:31993864-31994721	24	SUZ12
chr19:12526919-12528696	9	ZNF564

chr1:16201781-16202574	35	ARHGEF19
chr12:64648536-64649552	8	nogene
chr19:39979816-39980717	10	PSMC4
chr17:50984922-50987237	2	SPAG9
chr12:7396094-7406852	3	CD163L1
chr12:110134947-110135437	3	IFT81
chr1:12005710-12009726	3	MFN2
chr15:72774871-72780322	13	ADPGK
chr16:2319580-2329854	11	ABCA3
chr15:60360940-60386086	17	ANXA2
chr3:141512162-141540609	92	RASA2
chr3:195306510-195345317	14	ACAP2
chrX:41346228-41346622	28	DDX3X
chr22:38636450-38639724	3	FAM227A
chr9:85596361-85633374	79	AGTPBP1
chr20:34291418-34294156	46	AHCY
chr12:99244341-99246864	3	ANKS1B
chr5:78110206-78129307	20	AP3B1
chr8:130358016-130402893	67	ASAP1
chr1:161819632-161821161	18	ATF6
chr17:60683981-60709325	2	BCAS3
chr12:31398174-31409384	23	DENND5B
chr18:49363160-49379758	73	DYM
chr5:94050730-94053227	7	FAM172A
chr1:236178413-236183906	100	GPR137B
chr10:1000676-1005907	9	GTPBP4
chr14:61720378-61721823	18	HIF1A
chr2:38591529-38597354	16	HNRNPLL
chr8:94827262-94832174	14	INTS8
chr3:37083635-37096660	16	LRRFIP2
chr6:136694139-136705133	4	MAP3K5
chr17:29103598-29107189	12	MYO18A
chr17:63028003-63099357	3	nogene
chr15:69462011-69462315	15	nogene
chr3:183833491-183868060	11	PARL
chr7:92517275-92519078	36	PEX1
chr3:195519017-195524896	5	PPP1R2
chr12:11047019-11124187	14	PRH1
chr12:112446275-112482205	4	PTPN11
chr2:135602499-135622732	10	R3HDM1
chr7:66771882-66805396	20	RABGEF1
chr9:4823547-4849550	40	RCL1
chr19:33011678-33012724	23	RHPN2

chr19:58392975-58393487	724	RPS5
chr5:139121034-139133471	7	SIL1
chr6:85533598-85542043	11	SNX14
chr1:204112914-204114605	53	SOX13
chr6:159784665-159785496	11	TCP1
chr21:31164961-31195305	7	TIAM1
chr21:44094062-44098534	24	TRAPPC10
chr2:229802251-229804227	23	TRIP12
chr2:28850327-28865119	69	TRMT61B
chr10:1084402-1096246	263	WDR37
chr1:77639448-77641655	39	ZZZ3
chr10:73192555-73193593	4	FAM149B1
chr3:70987993-71015653	13	FOXP1
chr5:87331347-87349364	25	RASA1
chr1:75732643-75733628	17	ACADM
chr1:36008689-36009594	4	AGO3
chr6:106279660-106308491	77	ATG5
chr20:51674152-51676208	11	ATP9A
chr16:53255599-53268126	24	CHD9
chr16:58534146-58534395	10	CNOT1
chr10:5709529-5717750	47	FAM208B
chr12:110128949-110147048	13	IFT81
chr1:11228667-11232528	10	MTOR
chr16:29841595-29842112	10	MVP
chr4:107644801-107660072	21	PAPSS1
chr20:35721739-35734257	5	RBM39
chr2:45488239-45562756	18	SRBD1
chr8:9704662-9710220	18	TNKS
chr5:65647054-65658501	11	TRAPPC13
chr10:5956378-5960567	4	IL15RA
chr19:18838616-18839120	17	nogene
chr1:52886955-52892975	13	ZYG11A
chr1:147648245-147659775	38	ACP6
chr10:18666574-18668677	12	ARL5B
chr1:179990506-180006567	16	CEP350
chr2:9428764-9436361	10	CPSF3
chr4:1951071-1961151	3	WHSC1
chr5:72861807-72887222	16	TNPO1
chr1:155709772-155729366	3	DAP3
chr14:101983018-101983609	5	DYNC1H1
chr2:190900563-190923983	19	GLS
chr3:132688649-132694965	10	NPHP3-ACAD11
chr2:112059976-112067067	12	TMEM87B

chr11:674535-691598	14	DEAF1
chr20:3026686-3027841	12	PTPRA
chr17:82763964-82783714	29	TBCD
chr15:85064369-85083644	14	PDE8A
chr10:35137781-35179276	3	CREM
chr7:23317638-23361741	2	IGF2BP3
chr8:6455142-6480876	12	MCPH1
chr18:21765771-21799974	26	MIB1
chr4:139336847-139344339	7	NAA15
chr2:63571374-63571626	59	nogene
chr6:17665238-17669546	96	NUP153
chr11:74342460-74343416	3	PGM2L1
chr7:67295617-67297303	19	PMS2P4
chr8:103885297-103886223	118	RIMS2
chr17:54930072-54939020	11	TOM1L1
chr4:4306137-4315970	25	ZBTB49
chr13:42159265-42160136	33	DGKH
chr6:84556018-84567813	4	nogene
chr1:41113282-41161432	25	SCMH1
chr11:119273867-119278713	13	CBL
chr6:47503279-47504054	21	CD2AP
chr7:107527236-107558115	18	COG5
chr3:71041327-71047095	21	FOXP1
chrX:102713781-102721091	6	GPRASP2
chr5:32255692-32276742	5	MTMR12
chr7:38010920-38013581	14	nogene
chr2:45840379-45843063	6	PRKCE
chr6:42066311-42077239	9	TAF8
chr16:50068779-50079018	7	HEATR3
chr1:233346441-233362293	95	KIAA1804
chr6:136669282-136698751	10	MAP3K5
chr19:3425105-3449139	17	NFIC
chrX:119931935-119936682	11	NKAP
chr5:33023074-33023327	7	nogene
chr10:100380982-100381448	10	OLMALINC
chr11:20407910-20426865	10	PRMT3
chr8:127795893-127890998	12	PVT1
chr10:125812213-125816525	6	UROS
chr2:27615167-27617571	17	ZNF512
chr8:39136656-39151548	4	ADAM32
chr2:175117989-175136486	26	ATF2
chr3:48180720-48184695	4	CDC25A
chr3:32764454-32769962	14	CNOT10

chr2:55643157-55644720	45	PNPT1
chr2:101054107-101059539	14	TBC1D8
chr9:100320264-100349372	41	TEX10
chr2:199940088-199948472	4	TYW5
chr21:33581301-33586181	7	DONSON
chr1:9935491-9936627	4	LZIC
chr11:59608483-59610589	11	OSBP
chr6:7885960-7904723	4	TXNDC5
chr16:16033108-16056295	15	ABCC1
chr1:197583151-197715074	12	DENND1B
chr8:120453846-120470937	16	MTBP
chr15:64112572-64118854	25	SNX1
chr1:155408137-155415923	7	ASH1L
chr16:22248754-22251322	89	EEF2K
chr3:100339086-100341161	7	NIT2
chr11:45913818-45916303	11	PEX16
chr3:72824423-72844423	26	SHQ1
chr7:21477078-21482123	55	SP4
chr6:158429735-158461729	13	TULP4
chr6:34646600-34646798	10	C6orf106
chr4:2625261-2646832	13	FAM193A
chr2:239990073-240011696	95	NDUFA10
chr18:2775733-2784621	7	SMCHD1
chr9:100310298-100330169	5	TEX10
chr15:49235850-49308777	39	GALK2
chr2:239990073-240014860	247	NDUFA10
chr5:25180728-25191033	5	nogene
chr9:35231119-35259050	14	UNC13B
chr9:96827079-96853069	5	ZNF782
chr4:87089983-87094969	12	AFF1
chr1:155354472-155357749	12	ASH1L
chr21:36237261-36239955	6	DOPEY2
chr6:125993756-126008472	12	TRMT11
chr16:3810602-3851009	6	CREBBP
chr4:13336892-13342312	5	nogene
chr10:35501500-35530243	52	CCNY
chr20:34412514-34413879	13	ITCH
chr14:31306731-31308020	21	HEATR5A
chr10:74240927-74243033	6	nogene
chr3:179752284-179765848	4	USP13
chr5:163512001-163517674	12	MAT2B
chr15:100885266-100898185	11	ALDH1A3
chr19:49497193-49498046	151	RPS11

chr13:29522316-29536202	167	SLC7A1
chr9:96458378-96465778	32	HABP4
chr19:5039835-5082504	9	KDM4B
chr1:84179176-84202805	18	PRKACB
chr8:127882061-127890998	33	PVT1
chr20:56474677-56477124	11	RTFDC1
chr2:32392038-32406557	2	BIRC6
chr12:77998336-78021862	2	NAV3
chr16:31473681-31473977	2	TGFB1I1
chr2:161367477-161371322	25	PSMD14
chr18:9182381-9221999	183	ANKRD12
chr15:41243666-41262883	5	CHP1
chrX:131777622-131794466	12	FIRRE
chr14:73892026-73896534	20	ZNF410
chr1:113724758-113758733	32	PHTF1
chr10:74242737-74314745	52	ADK
chrX:80733455-80736088	99	BRWD3
chr3:197075836-197119530	30	DLG1
chr6:43600987-43605319	12	POLH
chr12:55800561-55803728	9	SARNP
chr15:42651015-42663917	18	STARD9
chr1:205268949-205269884	26	TMCC2
chr15:51887588-51893945	11	TMOD3
chr5:122799691-122803613	10	SNX2
chr8:47279861-47599196	16	SPIDR
chr7:130039463-130049144	122	ZC3HC1
chr6:43524470-43526747	24	XPO5
chr4:25151959-25159107	144	SEPSECS
chr11:74859157-74863021	6	XRRA1
chr3:72781304-72793036	23	SHQ1
chr8:140585118-140585311	53	AGO2
chr4:42543916-42556040	2	ATP8A1
chr6:16485971-16753347	10	ATXN1
chr22:17688985-17696210	13	BCL2L13
chr5:71545038-71549606	12	BDP1
chr15:36644277-36657905	63	C15orf41
chr5:179840871-179853437	11	C5orf45
chr13:114242120-114247004	16	CDC16
chr17:17261599-17264981	13	COPS3
chr11:108070095-108073497	6	CUL5
chr11:118754704-118758902	16	DDX6
chr22:37874369-37878171	2	EIF3L
chr4:38905521-38915073	2	FAM114A1

chr18:41516419-41525526	16	KC6
chr4:99041042-99045310	16	METAP1
chr18:21798083-21804014	10	MIB1
chr3:9662266-9671170	17	MTMR14
chr3:47897357-47897743	2	nogene
chr9:106009281-106019200	2	nogene
chr12:57395748-57398198	17	nogene
chr19:52588531-52592228	82	nogene
chr8:32595827-32616885	29	NRG1
chr10:49932099-49934163	6	PARG
chr1:233177801-233208610	29	PCNXL2
chr11:14789105-14819209	7	PDE3B
chr8:140890542-140925749	20	PTK2
chr1:40189054-40195764	48	RLF
chr16:57204503-57231319	2	RSPRY1
chr1:232513856-232515608	5	SIPA1L2
chr10:68487143-68488629	6	SLC25A16
chr14:102376649-102465289	2	TECPR2
chr4:84774819-84794979	2	WDFY3
chr4:182891348-182915575	214	DCTD
chr19:32899856-32918339	4	CEP89
chr14:103673013-103673431	13	KLC1
chr3:101816794-101821750	37	NXPE3
chr17:4066446-4072756	94	ZZEF1
chr9:94923985-94967801	12	C9orf3
chr9:137790847-137818138	21	EHMT1
chr16:89589896-89591260	11	CPNE7
chr7:133317283-133356573	20	EXOC4
chr7:22571774-22572013	5	LOC100506178
chr5:116399481-116412760	15	nogene
chr15:22976427-22976961	31	nogene
chr6:135300499-135323324	46	AHI1
chr10:121898840-121913893	17	ATE1
chr4:77055961-77066405	76	CCNI
chrX:338603-361590	42	PPP2R3B
chr1:39340501-39340953	16	nogene
chr1:197607072-197658369	5	DENND1B
chr1:51440346-51448135	50	EPS15
chr7:65979398-65980409	80	GUSB
chr10:103314185-103345132	2	PCGF6
chr18:42043732-42067513	3	PIK3C3
chr5:78089392-78165672	182	AP3B1
chr17:17261965-17264981	21	COPS3

chr10:408925-415888	6	DIP2C
chr3:44780884-44800437	3	KIF15
chr6:116692054-116726622	92	KPNA5
chr2:37974039-37975314	10	RMDN2
chr4:176727777-176740765	39	VEGFC
chr9:146101-164037	37	CBWD1
chr5:40746797-40777586	3	TTC33
chr1:15534236-15536814	89	DNAJC16
chr4:15625251-15644657	11	FBXL5
chr14:30628208-30638247	24	SCFD1
chr2:222613810-222634541	10	FARSB
chr5:157359004-157361598	53	CYFIP2
chr7:105470687-105495253	9	PUS7
chr3:196722414-196731092	15	PIGX
chr18:31846715-31849739	15	TRAPPC8
chr22:46098010-46098469	3	MIRLET7BHG
chr6:104795578-104843298	2	HACE1
chr2:174078967-174082064	14	OLA1
chr4:108655563-108657647	18	OSTC
chr14:70962225-71013202	2	PCNX
chr10:27169943-27181581	9	MASTL
chr17:36986616-37021014	30	AATF
chr9:85692688-85712566	20	AGTPBP1
chr3:138274448-138290639	4	ARMC8
chr11:74113779-74139828	4	C2CD3
chr12:48833276-48834497	42	DDX23
chr1:97450058-97515941	3	DPYD
chr11:32587011-32589641	25	EIF3M
chr2:241403827-241431774	3	FARP2
chr3:33065462-33072713	46	GLB1
chr3:37075023-37096660	31	LRRFIP2
chr5:34163117-34182867	24	nogene
chr5:171213618-171265847	4	RANBP17
chr18:47845339-47896809	7	SMAD2
chr18:12506476-12535601	43	SPIRE1
chr12:103976917-103980962	20	TDG
chr16:69361403-69368482	12	TERF2
chr1:151888272-151889373	14	THEM4
chr16:88627919-88628854	4	ZC3H18
chr14:100084686-100097658	26	EVL
chr22:25661575-25695214	5	ADRBK2
chr5:148394713-148425701	17	FBXO38
chr12:25225613-25245395	11	KRAS

chr7:2720603-2721550	4	nogene
chr14:73355625-73410068	18	NUMB
chr17:62573214-62586226	8	TLK2
chr4:147906637-147966839	2	ARHGAP10
chr4:51874270-51891851	19	DCUN1D4
chr2:206445306-206481308	19	ADAM23
chr2:27213176-27216620	10	ATRAID
chr14:92093251-92096838	19	ATXN3
chr1:154212319-154213946	5	C1orf43
chr18:13037236-13040956	52	CEP192
chr12:119832770-119850275	10	CIT
chr4:1225359-1238337	32	CTBP1
chr5:137948954-137949184	20	FAM13B
chr2:222630112-222634541	18	FARSB
chr5:108883395-109047198	2	FER
chr19:19465344-19465614	10	GATAD2A
chr2:196271192-196308085	16	HECW2
chr1:23030468-23050520	24	KDM1A
chr18:21778102-21779685	109	MIB1
chr6:97254556-97269992	12	MMS22L
chr17:31155982-31206371	24	NF1
chr19:16635806-16636479	14	nogene
chr17:78392475-78398351	3	PGS1
chr7:151484736-151498180	56	RHEB
chr10:75150513-75168658	81	SAMD8
chr2:127989563-127996491	29	SAP130
chr10:7220410-7276989	10	SFMBT2
chr7:17868406-17875790	667	SNX13
chr17:20245925-20306082	27	SPECC1
chr2:45413113-45419894	21	SRBD1
chr9:112098469-112112868	3	SUSD1
chr4:6996324-7004924	21	TBC1D14
chr15:43428015-43441583	7	TP53BP1
chr15:42872610-42878684	93	TTBK2
chr9:15210087-15226012	14	TTC39B
chr13:27089882-27116596	41	USP12
chr15:50432337-50439177	11	USP8
chr6:142166227-142189534	35	VTA1
chr1:247308474-247310456	87	ZNF496
chr5:157518822-157530883	14	ADAM19
chr8:6730710-6732650	14	AGPAT5
chr11:46547112-46548500	5	AMBRA1
chr10:87784469-87792755	11	ATAD1

chr7:16681300-16697061	21	BZW2
chr3:33606590-33663515	4	CLASP2
chr22:19963565-19964299	13	COMT
chr12:56282419-56283857	6	CS
chr12:116964696-116988869	48	FBXW8
chr3:10226370-10239039	4	IRAK2
chr1:32163234-32167296	33	KPNA6
chrX:150736594-150737448	128	MTMR1
chr4:17814852-17818088	20	NCAPG
chr12:55789074-55796076	19	SARNP
chr3:27383152-27391808	10	SLC4A7
chr18:2722518-2729409	2	SMCHD1
chr1:31269157-31292006	7	SNRNP40
chr15:44595258-44596943	11	SPG11
chr9:109050282-109060452	14	TMEM245
chr10:80507227-80509471	11	TSPAN14
chr1:32187953-32190249	31	TXLNA
chr11:75861745-75912039	3	UVRAG
chr10:1072115-1078003	20	WDR37
chr3:48450036-48454418	5	ATRIP
chr10:73718014-73722577	4	BMS1P4
chr21:41901621-41909523	8	C2CD2
chr3:138505294-138529462	4	CEP70
chr2:47822217-47823360	191	FBXO11
chr17:62010549-62063301	4	MED13
chr16:47086225-47132025	15	NETO2
chr1:169287302-169324500	5	NME7
chr10:103178796-103179036	7	nogene
chr8:94934036-94934467	7	nogene
chr18:56096074-56097195	4	nogene
chr4:76130655-76144473	45	NUP54
chr1:202431479-202449171	7	PPP1R12B
chr2:53899880-53901559	9	PSME4
chr5:128131066-128161800	32	SLC12A2
chr17:64591083-64598490	28	SMURF2
chr12:53381658-53411435	26	SP1
chr15:42634855-42638812	40	STARD9
chr13:52657531-52659249	11	SUGT1
chr15:23008698-23010133	25	TUBGCP5
chr17:2635382-2638320	6	PAFAH1B1
chr8:140664916-140674404	84	PTK2
chr17:18340042-18340813	14	SHMT1
chr15:76225848-76295737	16	ETFA

chr20:21339043-21346550	19	XRN2
chr10:74394140-74525426	2	ADK
chr11:72110087-72111496	11	ANAPC15
chr8:102839649-102843747	10	AZIN1
chr14:34826012-34862322	48	BAZ1A
chr2:73239642-73244715	13	CCT7
chr15:73702771-73703997	638	CD276
chr22:46384542-46389499	108	CELSR1
chr12:38760846-38776301	48	CPNE8
chr2:171468887-171469456	26	DCAF17
chr11:44107682-44130138	79	EXT2
chr1:93894613-93897898	125	GCLM
chr14:31156839-31157303	14	HECTD1
chr5:138372673-138377825	12	KDM3B
chr18:62221036-62232427	13	KIAA1468
chr18:8714138-8720496	228	MTCL1
chr21:21280577-21374013	2	NCAM2
chr12:68715626-68727389	14	NUP107
chr5:41749526-41805681	12	OXCT1
chr12:53454749-53471807	4	PCBP2
chr17:45450617-45454272	2	PLEKHM1
chr3:158122102-158225733	6	RSRC1
chr6:107901369-107924932	52	SEC63
chr14:30902535-30911162	97	STRN3
chr17:82763964-82870380	12	TBCD
chr17:998778-999584	20	TIMM22
chr2:32630522-32678922	34	TTC27
chr21:32378444-32385684	14	URB1
chr6:144521979-144523188	43	UTRN
chr1:52477707-52481923	24	ZCCHC11
chr3:185687056-185698347	10	IGF2BP2
chrX:21868466-21869678	4	MBTPS2
chr17:60347657-60347842	29	nogene
chr14:58318541-58330169	42	ARID4A
chr8:140846259-140925749	145	PTK2
chr12:82430892-82456820	19	METTL25
chr9:31942990-31973112	19	nogene
chr9:83659779-83686155	77	UBQLN1
chr20:1452492-1455132	17	NSFL1C
chr18:36681435-36693423	65	FHOD3
chr8:42398711-42403461	31	VDAC3
chr6:156829226-156829421	18	ARID1B
chr11:107454449-107455776	10	CWF19L2

chr12:124964393-124965812	13	DHX37
chr6:89793761-89803554	10	MDN1
chr11:61436760-61438113	17	SDHAF2
chr15:44567423-44569505	10	SPG11
chr8:67266005-67277457	8	ARFGEF1
chr9:96533926-96541892	3	CDC14B
chr21:36362182-36369876	6	MORC3
chr21:46381693-46391376	12	PCNT
chr10:122392302-122400388	10	PLEKHA1
chr12:19253939-19261021	75	PLEKHA5
chr18:9775277-9815222	40	RAB31
chr17:15612700-15614033	11	CDRT1
chr2:171448680-171476950	29	DCAF17
chr16:67120111-67132525	2	C16orf70
chr14:61995875-61996542	3	SYT16
chr3:112109542-112113145	9	C3orf52
chr15:50912077-50930971	16	AP4E1
chr16:11894953-11896782	3	GSPT1
chr2:9509978-9543285	28	ADAM17
chr18:12356693-12358943	10	AFG3L2
chr12:132005076-132006877	35	EP400
chr1:212872677-212895354	10	FLVCR1
chr12:68820324-68828931	11	MDM2
chr11:19976746-19984247	13	NAV2
chr20:31545951-31546495	29	nogene
chr5:37325900-37328420	84	NUP155
chr1:233208517-233218184	12	PCNXL2
chr8:98706466-98722969	89	STK3
chr12:130812956-130827267	6	STX2
chr11:43448001-43451265	17	TTC17
chr3:179681877-179707076	28	USP13
chr4:176727777-176731524	16	VEGFC
chr8:99384207-99442635	19	VPS13B
chr17:68444492-68452992	28	WIPI1
chr1:78013050-78013619	4	DNAJB4
chrX:53614533-53615835	226	HUWE1
chr11:29381072-29384112	26	nogene
chr10:87894024-87895777	5	PTEN
chr3:196323743-196327591	40	TM4SF19-TCTEX1D2
chr13:111280350-111280505	4	ARHGEF7
chr11:65920342-65920662	4	DRAP1
chr11:20402918-20426865	35	PRMT3
chr1:174272413-174394145	69	RABGAP1L

chr3:47046486-47067118	42	SETD2
chr4:20704465-20713539	6	PACRGL
chr3:27448650-27458991	5	SLC4A7
chr1:75728400-75734871	2	ACADM
chr22:25663629-25687667	2	ADRBK2
chr1:225085709-225097239	12	DNAH14
chr6:136637324-136642569	2	MAP3K5
chrX:115629197-115640503	10	PLS3
chr2:3338016-3356363	5	TSSC1
chr16:89305205-89317078	29	ANKRD11
chr4:17804403-17805225	3	DCAF16
chr8:73673106-73709162	131	STAU2
chr18:46815311-46829867	4	PIAS2
chr2:190927305-190954818	6	GLS
chr3:155897926-155911279	13	GMPS
chr2:66464108-66512294	4	MEIS1
chr7:66770322-66775393	13	RABGEF1
chr4:4315635-4315970	23	ZBTB49
chr4:145152540-145155996	7	OTUD4
chr13:20731840-20737805	35	N6AMT2
chrX:119410281-119426354	4	nogene
chr11:31452717-31463305	107	IMMP1L
chr19:11178344-11178720	87	KANK2
chr2:235744697-235750488	140	AGAP1
chr3:195971322-195979866	11	SDHAP1
chr15:50458999-50462322	24	USP8
chr12:28225794-28259442	188	CCDC91
chr7:139237004-139266403	17	UBN2
chr9:94292340-94293069	17	ZNF169
chr18:9583116-9595238	18	PPP4R1
chr10:49827401-49832908	23	PARG
chr19:39417554-39419003	21	PLEKHG2
chr7:105168007-105169265	20	SRPK2
chr17:4189381-4209947	4	ANKFY1
chr16:21315883-21317126	8	CRYM-AS1
chr5:75685206-75692500	33	POC5
chr4:70762518-70768661	77	RUFY3
chr19:9652952-9660874	101	ZNF562
chr8:130214555-130361771	11	ASAP1
chr1:6549570-6550615	9	NOL9
chr15:78289618-78290556	14	WDR61
chr15:62020478-62044255	63	VPS13C
chr12:333486-334422	10	KDM5A

chr18:57731626-57761939	3	ATP8B1
chr2:186501272-186502587	11	ZC3H15
chr12:122216487-122218397	24	DIABLO
chr12:112060269-112061388	13	NAA25
chr13:95970111-95996135	57	UGGT2
chr3:15720914-15751820	16	ANKRD28
chr12:51073788-51074082	8	CSRNP2
chr1:198262574-198297240	4	NEK7
chr1:235612123-235612506	141	nogene
chrX:65075473-65107023	212	nogene
chr3:183717650-183728851	53	YEATS2
chr15:65178933-65180204	259	CLPX
chr22:28703504-28734727	23	CHEK2
chr4:17961902-17963049	14	LCORL
chr8:47800792-47807326	16	PRKDC
chr7:47396799-47400914	18	TNS3
chr2:42256500-42264731	15	EML4
chr1:117442230-117502970	6	MAN1A2
chr17:47826636-47828670	6	MRPL10
chr2:155809927-155811890	5	nogene
chr6:120978891-121016795	4	nogene
chr1:32224033-32224475	15	EIF3I
chr6:70699274-70732511	16	SMAP1
chr6:87677817-87681763	17	AKIRIN2
chr12:99772921-99806700	17	ANKS1B
chr22:28699837-28725367	50	CHEK2
chr9:132612086-132632091	16	DDX31
chr1:197672036-197674169	14	DENND1B
chr6:167022408-167033971	172	FGFR1OP
chr3:197756425-197763638	37	FYTTD1
chr1:155813633-155814449	12	GON4L
chr21:34102834-34125480	305	MRPS6
chr11:34070937-34071336	4	nogene
chr5:34048649-34049185	20	nogene
chr13:24441845-24455212	28	PARP4
chr22:35809779-35938897	13	RBFOX2
chr4:76995780-76996539	15	11-Sep
chr2:161179613-161204793	92	TANK
chr18:24080584-24083081	62	TTC39C
chr9:33944364-33953474	8	UBAP2
chr10:87784469-87814612	33	ATAD1
chr17:3026007-3032449	15	RAP1GAP2
chr2:119236469-119237165	27	nogene

chr6:3410187-3438555	10	SLC22A23
chr21:37087246-37096643	11	TTC3
chrX:16818573-16818877	7	TXLNG
chr6:135039572-135042118	5	HBS1L
chr2:58123100-58135199	2	VRK2
chr1:244411286-244424387	6	ADSS
chr11:68574108-68583129	4	PPP6R3
chr7:17873527-17875790	46	SNX13
chr12:77940318-77968702	84	NAV3
chr19:15368234-15377014	3	AKAP8
chr18:58514992-58529238	4	ALPK2
chr15:50948019-50950169	7	AP4E1
chr1:35743140-35746992	24	CLSPN
chr9:123652012-123879021	8	DENND1A
chr14:66916002-66924292	15	GPHN
chr14:49709158-49725769	8	KLHDC1
chr7:1957628-2069338	5	MAD1L1
chr6:136694139-136697387	21	MAP3K5
chr5:34175444-34179097	43	nogene
chr8:106679209-106692877	43	OXR1
chr10:34331116-34341816	5	PARD3
chr7:103353920-103355793	3	PSMC2
chr1:8495062-8508675	12	RERE
chr7:96277195-96309691	79	SLC25A13
chr12:53406584-53409561	18	SP1
chr18:58709377-58723251	4	MALT1
chr20:49646964-49656702	3	B4GALT5
chr9:120771347-120778545	11	FBXW2
chr17:31258343-31265339	78	NF1
chr22:30243739-30246797	30	nogene
chr7:105146492-105203785	17	SRPK2
chr13:113510237-113534240	6	TMCO3
chr12:350620-354234	59	KDM5A
chr7:98967040-98971945	9	TRRAP
chr17:66123264-66132763	4	CEP112
chr16:50786854-50793664	51	CYLD
chr1:44284834-44352925	4	ERI3
chr13:21161789-21172681	11	SKA3
chr3:11807832-11809682	5	TAMM41
chr5:65260599-65300131	59	ADAMTS6
chr4:121677893-121686372	3	ANXA5
chr11:34082824-34089456	9	CAPRIN1
chr6:7181123-7189322	94	RREB1

chr20:4883641-4899712	27	SLC23A2
chr14:73953434-73955928	13	COQ6
chr7:66130743-66145500	42	CRCP
chr7:6434761-6436533	15	DAGLB
chr10:93670769-93687449	6	FRA10AC1
chr12:112258495-112259265	3	HECTD4
chr20:26018831-26019202	13	LOC100134868
chr2:42656199-42682589	2	MTA3
chr22:46978546-46978934	3	nogene
chr13:78635109-78644997	32	RNF219
chr7:17875479-17875790	89	SNX13
chr9:112078537-112102285	35	SUSD1
chr17:82756164-82814934	29	TBCD
chr5:88220589-88240916	3	TMEM161B
chr1:154172907-154173201	116	TPM3
chr3:33411580-33425741	108	UBP1
chr3:49286097-49286325	6	USP4
chr8:99274197-99328914	20	VPS13B
chr2:174581309-174585611	10	WIPF1
chr13:59911734-59924870	13	DIAPH3
chr9:97139852-97144740	130	nogene
chr2:200439121-200440784	59	SPATS2L
chr10:91830922-91836998	3	TNKS2
chr20:23389945-23403072	174	NAPB
chr7:157382248-157385611	4	DNAJB6
chr5:178222354-178225429	15	PHYKPL
chr1:27841013-27843330	25	PPP1R8
chr3:179740246-179745217	16	USP13
chr14:77451186-77454102	3	VIPAS39
chr11:130109428-130110661	391	APLP2
chr18:79126266-79307234	13	ATP9B
chr12:111658735-111679340	30	BRAP
chr5:37238856-37247745	64	C5orf42
chr1:155269487-155270977	16	CLK2
chr19:54142928-54146657	11	CNOT3
chr1:197617658-197715074	12	DENND1B
chr1:184732836-184749592	33	EDEM3
chr15:82214716-82230971	5	EFTUD1
chr12:11869423-11869969	83	ETV6
chr6:629834-637861	309	EXOC2
chr5:453369-454051	16	EXOC3
chr1:1804418-1815862	4	GNB1
chrX:70374150-70387297	2	KIF4A

chr1:93339356-93340510	7	LOC100131564
chr12:112068879-112074764	15	NAA25
chr2:190659157-190673152	2000	NAB1
chr4:119412351-119420155	15	nogene
chr12:108545888-108546309	10	nogene
chr2:95833905-95834224	18	nogene
chrX:85091377-85091770	12	nogene
chr2:10644319-10671690	24	NOL10
chr17:46692902-46713986	5	NSF
chr10:103139405-103174982	13	NT5C2
chr12:89459637-89467685	42	POC1B
chr2:108771700-108773046	231	RANBP2
chr1:185087551-185093276	63	RNF2
chr10:119902798-119909130	2	SEC23IP
chr11:17988596-17995909	18	SERGEF
chr1:100906851-100921841	188	SLC30A7
chr13:60439687-60467379	301	TDRD3
chr13:113520615-113539507	16	TMCO3
chr8:140283888-140287734	15	TRAPPC9
chr18:76875491-76881512	3	ZNF236
chr2:173364794-173367831	43	CDCA7
chr11:34089394-34092056	21	CAPRIN1
chr1:117414712-117466427	26	MAN1A2
chr19:50795740-50797396	12	nogene
chr12:95031348-95057878	7	NR2C1
chr11:73359833-73361161	14	ARHGEF17
chr4:39913610-39925933	558	PDS5A
chr4:40890363-40935139	89	APBB2
chr3:5170502-5172740	9	ARL8B
chr1:161802051-161821161	15	ATF6
chr19:41230966-41231298	36	AXL
chr15:73699585-73704472	23	CD276
chr3:98876666-98881767	6	DCBLD2
chr8:119838258-119850516	26	DSCC1
chr16:68025414-68056424	7	DUS2
chr16:23529501-23529897	19	EARS2
chr3:172773902-172786574	28	ECT2
chr4:2657802-2683115	3	FAM193A
chr2:232747614-232756334	18	GIGYF2
chr1:45630481-45640403	12	GPBP1L1
chr7:152235816-152238826	23	KMT2C
chr2:15461200-15461791	3	NBAS
chr6:107038005-107038768	12	nogene

chr17:58001488-58005041	5	nogene
chr12:9564159-9571573	11	nogene
chr1:36170963-36173480	12	nogene
chr22:50410558-50411377	7	nogene
chr3:121439991-121468431	14	POLQ
chr14:94256459-94259369	33	PPP4R4
chr2:160300650-160318227	19	RBMS1
chr11:74789625-74810330	108	RNF169
chr15:41529469-41529979	12	RPAP1
chr3:47435007-47466447	28	SCAP
chr7:12214808-12218521	28	TMEM106B
chr19:34430575-34434968	793	UBA2
chr3:124735092-124744203	17	UMPS
chr8:17284472-17286427	10	VPS37A
chr13:26573714-26613058	68	WASF3
chr16:57164000-57173869	675	FAM192A
chr7:23347599-23351586	3	IGF2BP3
chrX:70404714-70407075	23	KIF4A
chr4:76130655-76136412	45	NUP54
chr7:157220688-157225539	3	UBE3C
chr19:12145257-12147802	5	ZNF625-ZNF20
chr2:152542738-152581049	32	FMNL2
chr11:64193077-64194620	40	STIP1
chr8:93786222-93816515	12	TMEM67
chr16:50333573-50340086	7	BRD7
chr3:122833316-122860202	7	DIRC2
chr3:184324877-184328990	3	EIF4G1
chr3:122461223-122467429	7	KPNA1
chr22:38361398-38380990	12	LOC400927
chr8:100225172-100233537	5	SPAG1
chr4:53414614-53453133	7	FIP1L1
chr3:32757013-32757274	37	nogene
chr11:122064179-122089048	11	nogene
chr20:34065043-34078553	40	RALY
chr16:53933984-53937294	16	FTO
chr5:34686871-34688248	11	RAI14
chr14:35721687-35725153	10	RALGAPA1
chr1:54036609-54047372	11	TMEM59
chr16:27701588-27708920	31	KIAA0556
chr12:53018797-53022627	19	EIF4B
chr12:122232129-122232968	6	VPS33A
chr8:61633682-61644632	12	ASPH
chr22:17655661-17689142	15	BCL2L13

chr7:2360709-2364529	138	EIF3B
chr20:16526091-16528440	329	KIF16B
chr2:113951478-113955706	17	ACTR3
chr11:67180078-67181892	42	KDM2A
chr18:24070750-24071023	10	nogene
chr17:81613317-81629805	58	NPLOC4
chr13:25307805-25321093	23	NUPL1
chr16:14580873-14586361	2	PARN
chr18:79933968-79935598	29	PQLC1
chr17:80339200-80340356	10	RNF213
chr21:33460412-33468926	11	TMEM50B
chr6:43572505-43573601	11	XPO5
chr5:168399487-168406327	10	WWC1
chr8:130179263-130358143	10	ASAP1
chr14:65455620-65561766	11	FUT8
chr2:239156651-239176563	18	HDAC4
chr11:2570627-2572986	13	KCNQ1
chr18:24178012-24179835	6	OSBPL1A
chr10:70209204-70218817	53	PPA1
chr19:45025328-45028992	85	RELB
chr1:236168705-236180028	54	GPR137B
chr3:52470858-52473829	7	NISCH
chr10:97390422-97393760	38	RRP12
chr21:33559229-33559775	17	SON
chr15:89877305-89889140	19	AP3S2
chr5:25177590-25191033	4	nogene
chr9:97610345-97611699	17	TSTD2
chr2:229407231-229477253	10	DNER
chr21:36754179-36767285	12	HLCS
chr11:108317372-108321420	9	ATM
chr1:22078428-22091501	18	CDC42
chr1:15768527-15774796	12	FBLIM1
chr3:160366042-160384646	10	IFT80
chr9:6719070-6720997	19	KDM4C
chr18:51046919-51049324	45	SMAD4
chr9:2028986-2039900	31	SMARCA2
chr10:115461940-115469329	93	ATRNL1
chr21:15762890-15791664	4	USP25
chr7:42145437-42148468	19	GLI3
chr1:205103856-205105168	14	RBBP5
chr8:124515745-124516030	5	TATDN1
chr9:95240648-95249369	53	FANCC
chr15:22909193-22912275	6	CYFIP1

chr5:33561026-33596060	2	ADAMTS12
chr12:1754257-1778025	3	ADIPOR2
chr1:27558304-27558883	14	AHDC1
chr8:130214555-130358143	45	ASAP1
chr2:38315283-38319019	8	ATL2
chr6:80127546-80273221	3	BCKDHB
chr12:28305648-28307749	863	CCDC91
chr2:61872081-61872588	31	CCT4
chr17:56844018-56848065	76	DGKE
chr20:35547411-35548865	17	ERGIC3
chr6:592468-610178	15	EXOC2
chr17:44804587-44807469	6	GJC1
chr14:59475868-59479351	4	L3HYPDH
chr4:119457970-119462979	19	LOC645513
chr2:39292772-39309519	19	MAP4K3
chr3:52760794-52763624	24	NEK4
chr4:103548742-103593429	35	nogene
chr11:3702462-3702892	5	NUP98
chr3:111112029-111122238	46	PVRL3
chr15:67829178-67833257	7	SKOR1
chr17:29451454-29482288	15	TAOK1
chr7:66240324-66248646	92	TPST1
chr14:75707017-75720703	33	TTLL5
chr7:157223253-157248580	8	UBE3C
chr1:77711746-77714783	33	USP33
chr8:123427923-123437334	7	WDYHV1
chr2:61499712-61502310	16	XPO1
chr3:101652495-101652938	27	ZBTB11
chr10:124942456-124974398	11	ZRANB1
chr13:28174271-28239696	14	PAN3
chr7:134148237-134174636	13	LRGUK
chr12:104861290-104895345	12	SLC41A2
chr16:70276485-70282784	19	AARS
chr10:125038996-125088319	28	CTBP2
chr5:181241491-181242345	160	GNB2L1
chr13:48373404-48381443	11	RB1
chr3:123209198-123225297	10	SEC22A
chr9:120873978-120874756	5	PHF19
chr7:75510930-75511289	4	PMS2P3
chr19:32915336-32918339	107	CEP89
chr17:30785918-30804108	4	CRLF3
chr2:99369391-99371730	11	EIF5B
chr13:49168612-49175541	4	FNDC3A

chr16:47593499-47610920	4	PHKB
chr6:89342323-89343756	30	UBE2J1
chr20:51639342-51690819	13	ATP9A
chr5:173080584-173086626	7	CREBRF
chr7:6432836-6435020	17	DAGLB
chr9:93471140-93497599	94	FAM120A
chr2:9995878-9999029	7	GRHL1
chr10:92483254-92508205	50	IDE
chr15:41085368-41092182	2	INO80
chr11:77941179-77961138	2	INTS4
chr12:87377107-87415076	2	nogene
chr15:68141945-68173892	117	PIAS1
chr1:145839889-145841071	20	POLR3C
chr1:32825312-32828081	2	S100PBP
chr16:68273817-68275249	30	SLC7A6
chr12:110626361-110632559	8	TCTN1
chr2:32777881-32787149	50	TTC27
chr7:149198332-149213814	2	ZNF282
chr5:179719667-179720560	25	CANX
chr11:122776218-122779695	12	UBASH3B
chr1:26442730-26447660	10	DHDDS
chr7:78343777-78369213	10	MAGI2
chr4:139116648-139139497	78	nogene
chr9:123169901-123236969	4	STRBP
chr9:83678440-83683066	63	UBQLN1
chr8:123436452-123448224	10	WDYHV1
chr22:28710005-28719485	7	CHEK2
chr4:5719247-5733435	17	EVC
chr11:11372514-11379264	11	GALNT18
chr12:46261295-46261851	20	nogene
chr2:189753915-189754345	4	OSGEPL1
chr4:39869393-39890364	17	PDS5A
chr12:112477650-112478015	70	PTPN11
chr7:129014978-129041717	13	TNPO3
chr17:4032125-4033002	10	ZZEF1
chr6:135453340-135455926	21	AHI1
chr5:140438502-140449305	4	ANKHD1-EIF4EBP3
chr10:68747373-68749685	16	CCAR1
chr1:77858937-77864028	97	FAM73A
chr3:23310451-23310727	2	nogene
chr7:66775226-66799414	65	RABGEF1
chr12:123334052-123348133	12	SBNO1
chr1:179342113-179343635	28	SOAT1

chr15:93004616-93014909	14	CHD2
chr8:94824892-94849532	13	INTS8
chr4:4237826-4246343	141	TMEM128
chr10:104008176-104018289	14	SLK
chr19:18742909-18749875	19	CRTC1
chr12:125102678-125107268	57	AACS
chrX:75098941-75114834	25	ABCB7
chr4:87108158-87115299	12	AFF1
chr5:77046346-77048987	30	AGGF1
chr1:243552728-243573048	17	AKT3
chr9:98777404-98782573	11	ANKS6
chr2:223775900-223777869	26	AP1S3
chr5:78089392-78101025	3	AP3B1
chr21:25954589-25997416	16	APP
chr20:32362355-32369123	3	ASXL1
chr10:68747160-68757377	3	CCAR1
chrX:2714421-2720424	4	CD99
chr5:138198198-138201448	15	CDC23
chr4:55999301-55999645	12	CEP135
chr3:138505294-138532570	3	CEP70
chr1:239718795-239827378	14	CHRM3
chr1:47374908-47375293	8	CMPK1
chr11:61415665-61429290	14	CPSF7
chr20:49066191-49067280	39	CSE1L
chr10:68973544-68981581	23	DDX21
chr11:118786051-118786518	191	DDX6
chr22:38537591-38568289	81	DMC1
chr13:95709226-95725252	38	DNAJC3
chr6:83096730-83097115	3	DOPEY1
chr15:76274411-76295737	188	ETFA
chr1:11070899-11076948	103	EXOSC10
chr22:45332424-45332710	13	FAM118A
chr2:153900935-153944639	31	GALNT13
chr5:154911726-154913038	43	GEMIN5
chr3:155919232-155925366	54	GMPS
chr2:26279993-26285571	7	HADHB
chr10:69392124-69398828	3	HK1
chr11:9423005-9425262	26	IPO7
chr8:41974703-41981954	3	KAT6A
chr8:125049005-125057666	7	KIAA0196
chr7:138905021-138912460	3	KIAA1549
chrX:65523559-65532630	41	LAS1L
chr3:65609743-65622088	29	MAGI1

chr12:111865249-111871180	58	MAPKAPK5
chr15:66119085-66128411	24	MEGF11
chrX:107916001-107917649	3	MID2
chr13:23841334-23886506	19	MIPEP
chr6:31743903-31744581	17	MSH5
chr15:71991102-71994585	4	MYO9A
chr11:20062306-20083179	13	NAV2
chr6:125874888-125878370	6	NCOA7
chr5:142131807-142140629	9	NDFIP1
chr7:66551519-66578727	2	nogene
chr10:27566831-27578713	89	nogene
chr11:65505662-65506054	4	nogene
chr2:241566725-241567620	3	nogene
chr7:66848488-66880877	4	nogene
chr19:11648357-11654411	29	nogene
chr3:132626690-132631479	4	NPHP3-ACAD11
chr2:174123179-174142000	181	OLA1
chr13:28256291-28281379	24	PAN3
chr14:71026116-71036157	55	PCNX
chr6:169712385-169721111	11	PHF10
chr7:102566996-102569924	35	POLR2J3
chr19:55245119-55245678	45	PPP6R1
chr7:1569123-1569360	33	PSMG3
chr13:19730238-19782791	2	PSPC1
chr2:119920068-119926666	20	PTPN4
chr3:61981707-62003497	3	PTPRG
chr1:174637374-174811960	10	RABGAP1L
chr5:170892386-170896115	56	RANBP17
chr4:53145332-53147370	10	SCFD2
chr1:41142877-41151684	11	SCMH1
chr18:12963159-12971251	2	SEH1L
chr3:196899665-196903610	78	SENP5
chr20:44509890-44512994	25	SERINC3
chr2:171809605-171815202	14	SLC25A12
chr17:48112030-48113401	265	SNX11
chr7:17868406-17891635	2	SNX13
chr13:49927918-49931134	3	SPRYD7
chr2:45546316-45553730	10	SRBD1
chr1:54251615-54257186	29	SSBP3
chr1:43831654-43838311	11	ST3GAL3
chr19:19007346-19008428	38	SUGP2
chr1:235427139-235434280	3	TBCE
chr6:84738496-84762748	10	TBX18

chr1:246548544-246556721	11	TFB2M
chr19:41344746-41348455	18	TGFB1
chr2:43505621-43527988	12	THADA
chr2:70224553-70236175	58	TIA1
chr17:44014356-44015155	3	TMEM101
chr12:88148287-88176319	51	TMTC3
chr17:42610107-42659552	23	TUBG1
chr15:43377846-43386330	10	TUBGCP4
chr15:42976716-43025429	4	UBR1
chr11:18570213-18578808	4	UEVLD
chr15:50462279-50471795	26	USP8
chr3:51440969-51441897	12	VPRBP
chr15:62033440-62044255	151	VPS13C
chr2:63404047-63439871	26	WDPCP
chr16:74885652-74886396	6	WDR59
chr6:38082391-38116739	46	ZFAND3
chr18:76371466-76380303	45	ZNF516
chr16:16124788-16125911	79	ABCC1
chrX:23713134-23722753	21	ACOT9
chr20:41423491-41426153	13	CHD6
chr12:108652271-108701323	12	CORO1C
chr11:33141666-33141986	21	CSTF3
chr1:245017241-245083688	19	EFCAB2
chr3:68998010-69004482	16	EOGT
chr11:22674849-22685789	5	GAS2
chr5:74745677-74751493	5	GFM2
chr15:28245880-28246897	14	HERC2
chr4:3180514-3189093	10	HTT
chrX:53614533-53617446	11	HUWE1
chr1:37866458-37874088	38	INPP5B
chr18:21765771-21849388	7	MIB1
chr1:32821224-32821536	10	nogene
chr4:41004759-41005200	13	nogene
chr7:66882892-66886455	9	nogene
chr3:195897236-195897424	2	nogene
chr3:136326802-136328857	11	PCCB
chr2:196890827-196898369	11	PGAP1
chr22:20810966-20813506	2	PI4KA
chr11:20402918-20408032	2	PRMT3
chr3:185598411-185626393	2	SENP2
chr4:146503848-146510049	13	SLC10A7
chr1:108143542-108161293	21	SLC25A24
chr6:121303616-121310847	27	TBC1D32

chr3:129880308-129884971	8	TMCC1
chr3:12742453-12748792	2	TMEM40
chr21:37187048-37196036	33	TTC3
chr1:162499954-162503848	2	UHMK1
chr18:74877196-74890038	71	ZNF407
chr8:67076481-67095732	9	CSPP1
chr21:43592224-43633421	10	HSF2BP
chr15:76574157-76665789	29	SCAPER
chr2:28850327-28852499	3	TRMT61B
chr19:9652952-9656653	12	ZNF562
chr9:85585462-85633374	34	AGTPBP1
chr17:56848695-56858665	13	DGKE
chr11:108267170-108271406	11	ATM
chr17:31973145-31988497	11	SUZ12
chr9:41025763-41031512	47	PGM5P2
chr16:19706591-19711440	52	KNOP1
chr6:35637013-35646850	30	FKBP5
chr16:70261043-70262524	213	AARS
chr3:104764378-104817613	19	nogene
chr3:100555208-100568967	15	TMEM45A
chr14:21401956-21403663	117	CHD8
chr13:98457167-98466561	42	STK24
chr8:53769617-53801896	15	ATP6V1H
chr17:8506317-8509949	25	MYH10
chr6:79060477-79060818	13	PHIP
chr4:101193558-101196116	12	PPP3CA
chr3:29868857-29899755	56	RBMS3
chr6:13579450-13584225	189	SIRT5
chr4:138214584-138236451	23	SLC7A11
chr14:77552095-77562489	6	SPTLC2
chr20:62852722-62860308	12	TCFL5
chr20:50127974-50130947	10	TMEM189-UBE2V1
chr14:93214928-93215281	2	UBR7
chr11:113852500-113854335	40	USP28
chr17:37151300-37155780	11	ACACA
chr8:53743576-53772167	9	ATP6V1H
chr6:83096924-83100886	45	DOPEY1
chr11:103243692-103259977	13	DYNC2H1
chr11:94485923-94490965	50	MRE11A
chr13:77081426-77087633	11	MYCBP2
chr17:3726583-3726839	12	nogene
chr5:127523459-127539143	14	PRRC1
chr14:67685014-67693052	10	RDH11

chr2:37929261-38004216	2	RMDN2
chr9:136465961-136468512	2	SEC16A
chr6:79654494-79673799	9	SH3BGRL2
chr1:245764040-245929937	11	SMYD3
chr15:74466035-74479278	2	UBL7-AS1
chr15:63529013-63537156	216	USP3
chr3:141368644-141386937	14	ZBTB38
chr2:144424795-144430026	4	ZEB2
chr14:103405075-103405267	20	MARK3
chr3:114061841-114062119	24	nogene
chr8:53769617-53817530	3	ATP6V1H
chr16:4407500-4408251	5	CORO7-PAM16
chr16:70226769-70227260	3	nogene
chr2:65112005-65112257	3	nogene
chr3:51541497-51552063	108	nogene
chr12:5182976-5276889	12	nogene
chr16:16247394-16256158	12	NOMO3
chr11:68519500-68548204	34	PPP6R3
chr16:70526586-70532620	11	SF3B3
chr5:33448540-33453412	24	TARS
chr1:151774412-151783049	12	TDRKH
chr1:117084017-117086522	11	TTF2
chr1:52263772-52303925	5	ZFYVE9
chr1:28271673-28275015	7	SESN2
chr15:75605149-75621056	15	SNUPN
chr15:72518066-72572165	43	ARIH1
chr11:66748378-66828364	8	C11orf80
chr1:92662718-92704744	38	EVI5
chr3:56623643-56628614	9	FAM208A
chr17:35101200-35106481	3	RAD51D
chr6:88906364-88941180	17	RNGTT
chr17:62560015-62565137	10	TLK2
chr8:140360049-140426641	5	TRAPPC9
chr10:37831231-37838111	5	ZNF248
chr17:41912419-41913900	6	ACLY
chr4:22435310-22473843	10	ADGRA3
chr15:92945820-92967513	13	CHD2
chr11:46780193-46788773	5	CKAP5
chr3:32357372-32367988	10	CMTM8
chr17:73199864-73201900	10	COG1
chr3:186064416-186066072	4	ETV5
chr14:65721774-65724323	33	FUT8
chr19:34377502-34377881	19	GPI

chr8:94867139-94876489	46	INTS8
chr14:50735515-50744365	5	NIN
chr10:34799340-34799743	10	nogene
chr11:32927156-32928123	18	QSER1
chr19:47143493-47169923	51	SAE1
chr9:100498764-100550160	24	TMEFF1
chr7:66941847-66945261	116	TMEM248
chr22:43039779-43046573	32	TTLL1
chr8:19364071-19373660	10	SH2D4A
chr1:235335241-235342883	3	GGPS1
chr1:236564497-236569124	13	HEATR1
chr4:150467672-150599131	4	LRBA
chr1:62829039-62841547	19	ATG4C
chr2:171448680-171469030	55	DCAF17
chr1:92732509-92736627	108	EVI5
chrX:72462003-72495268	34	HDAC8
chr15:59084128-59089759	5	RNF111
chr12:123348028-123350441	21	SBNO1
chr16:70526586-70538460	3	SF3B3
chr5:121974195-121975387	14	SRFBP1
chr17:4216982-4235890	20	ANKFY1
chr1:26432890-26460144	5	DHDDS
chr3:51260155-51280204	4	DOCK3
chr11:9408485-9414411	51	IPO7
chr2:206160881-206161132	24	nogene
chr12:107742220-107746424	7	PRDM4
chr14:90676522-90695578	5	TTC7B
chr7:16698047-16704669	17	BZW2
chr9:33060464-33071892	10	SMU1
chr17:61776400-61801474	26	BRIP1
chr6:43527633-43534007	13	XPO5
chr12:28304625-28307749	76	CCDC91
chr6:104824813-104843298	19	HACE1
chr5:618989-624579	95	CEP72
chr12:52178502-52179217	4	nogene
chr16:70010750-70014167	29	PDXDC2P
chr5:37238856-37239869	17	C5orf42
chr10:84417770-84464016	16	CCSER2
chr21:36391550-36394650	20	CHAF1B
chr1:85816787-85849406	36	COL24A1
chrX:130042880-130043126	2	nogene
chr11:18336376-18337175	12	nogene
chr3:38183002-38242778	2	OXSR1

chr3:50105076-50110770	2	RBM5
chr19:45011935-45012276	2	RELB
chr2:127954985-127955344	7	SAP130
chr20:34477771-34481206	31	ITCH
chr4:15625251-15630791	9	FBXL5
chr11:67291475-67292180	4	ANKRD13D
chr1:246621428-246634587	89	CNST
chr9:20907149-20933103	56	FOCAD
chr15:63693963-63716473	10	HERC1
chr11:18281949-18285459	14	HPS5
chr3:197826877-197832317	11	LRCH3
chr10:121959323-121961517	13	NSMCE4A
chr1:109336239-109369589	21	SORT1
chr10:119578725-119588248	34	TIAL1
chr7:157220688-157248580	41	UBE3C
chr9:113969116-113988580	36	ZNF618
chr4:2625261-2631169	50	FAM193A
chr6:130184102-130184623	53	SAMD3
chr14:67656415-67662535	12	VTI1B
chr2:222900673-222909150	23	ACSL3
chr10:125074742-125111093	16	CTBP2
chr3:121493477-121498670	5	POLQ
chr16:70538322-70539207	4	SF3B3
chr16:48355337-48362430	11	SIAH1
chr17:81913773-81915681	12	SIRT7
chr1:116019581-116020885	5	SLC22A15
chr12:104288930-104289542	14	TXNRD1
chr19:45522167-45522818	37	VASP
chr21:25741572-25758204	16	GABPA
chr14:55652849-55659703	12	KTN1
chr16:69653235-69653428	10	NFAT5
chr9:120518426-120539164	28	CDK5RAP2
chr11:687910-691598	3	DEAF1
chr1:44247938-44319744	4	ERI3
chr12:79832331-79872938	3	PPP1R12A
chr18:9583116-9595153	362	PPP4R1
chr6:45003652-45020632	3	SUPT3H
chr17:31993864-31995762	27	SUZ12
chr6:82210813-82212793	403	IBTK
chr1:42265114-42311110	167	FOXJ3
chr20:5958527-5968025	38	MCM8
chr17:50989676-50990668	10	SPAG9
chr7:100111727-100112917	5	TAF6

chr15:71348151-71411823	12	THSD4
chr3:51470928-51492488	5	VPRBP
chr9:33953284-33960878	9	UBAP2
chr15:28143872-28146344	15	HERC2
chr15:89299799-89301442	13	FANCI
chrX:65106918-65113813	15	nogene
chrX:155545095-155548224	14	TMLHE
chr1:6192929-6197756	70	RPL22
chr19:41292244-41292511	27	HNRNPUL1
chr6:17507168-17507726	9	CAP2
chr1:156317165-156335888	13	CCT3
chr13:50433412-50528064	3	DLEU1
chr5:131647089-131651999	9	FNIP1
chr4:39841947-39844801	69	PDS5A
chr19:18342858-18343415	13	PGPEP1
chr11:68567013-68603492	16	PPP6R3
chr18:22992747-22996462	51	RBBP8
chr18:20954782-20955245	6	ROCK1
chr6:75702644-75703072	157	SENP6
chr17:18294702-18306966	24	TOP3A
chr15:50631416-50648885	46	TRPM7
chr1:180803387-180811488	25	XPR1
chr9:110136126-110138539	156	AKAP2
chr7:158797941-158799072	13	ESYT2
chr10:34374873-34401917	9	PARD3
chrX:345515-347693	116	PPP2R3B
chr6:111387764-111416472	10	REV3L
chr11:77941179-77981576	9	INTS4
chr12:111883580-111883768	11	MAPKAPK5
chr2:189805651-189818180	45	PMS1
chr6:123316456-123382147	27	TRDN
chr11:76958978-76998821	37	ACER3
chr11:94429910-94437235	3	MRE11A
chrX:68192918-68210282	16	OPHN1
chr14:50635213-50645014	29	SAV1
chr16:68859883-68880630	154	TANGO6
chr15:42811687-42840433	4	TTBK2
chr19:36712370-36712867	20	ZNF567
chr22:21769194-21788808	15	MAPK1
chr15:84538531-84555070	78	UBE2Q2P1
chr6:133980081-133984671	33	TBPL1
chr12:99655066-99825389	3	ANKS1B
chr3:43576691-43580472	13	ANO10

chr10:131970637-131973943	18	BNIP3
chr12:69259427-69262562	3	CPSF6
chr4:107944969-107947537	13	CYP2U1
chr5:112985834-112992248	33	DCP2
chr5:112985834-113008042	3	DCP2
chr15:65752378-65761438	97	DENND4A
chr9:36233919-36246482	2	GNE
chr2:206046503-206063244	2	INO80D
chr1:110222963-110226178	46	KCNC4
chr4:122192228-122199450	3	KIAA1109
chr7:138867974-138869761	11	KIAA1549
chr19:33061715-33062256	55	nogene
chr1:168249491-168251000	12	nogene
chr1:51750144-51772182	2	OSBPL9
chr3:167704841-167725473	25	PDCD10
chr19:11415946-11417059	6	RGL3
chr1:52978216-52980543	3	SCP2
chr12:50094436-50096972	33	SMARCD1
chr7:17890362-17897446	226	SNX13
chr9:33886880-33912098	67	UBE2R2
chr2:63856305-63862928	71	UGP2
chr5:69294792-69303929	3	CCDC125
chr10:96907264-96952196	69	LCOR
chr15:75394679-75400156	19	SIN3A
chr9:111914604-111933035	3	UGCG
chr6:169632946-169652008	113	WDR27
chr7:64541729-64544432	40	ZNF680
chr10:74022453-74022731	17	nogene
chr2:202282350-202287724	55	NOP58
chr2:85647929-85649203	9	USP39
chr16:11003073-11024921	12	CLEC16A
chr20:37198396-37213865	45	RPN2
chr12:110322991-110328017	12	ATP2A2
chr1:179986168-179997175	32	CEP350
chr2:65331986-65334773	5	SPRED2
chr3:33684358-33689932	146	CLASP2
chr10:842931-864270	2	LARP4B
chr8:144135635-144136000	17	nogene
chr22:42019661-42030823	2	WBP2NL
chr8:96879797-96880005	8	CPQ
chr12:62689698-62720289	47	PPM1H
chr8:47783741-47794501	17	PRKDC
chr4:128952768-128999794	3	SCLT1

chr5:128112813-128114681	3	SLC12A2
chr6:116645713-116660846	57	ZUFSP
chr10:127061667-127127764	72	DOCK1
chr11:62723586-62724426	72	HNRNPUL2-BSCL2
chr8:73542901-73552319	3	STAU2
chr6:82220589-82231842	11	IBTK
chr16:89426766-89431326	11	ANKRD11
chr2:23798137-23823569	16	ATAD2B
chr16:15695907-15696860	23	NDE1
chr14:96520054-96525391	62	PAPOLA
chr1:117456084-117466427	23	MAN1A2
chr10:101975211-102014024	3	C10orf76
chr11:93683558-93687865	20	CEP295
chr16:58580632-58581515	8	CNOT1
chr17:56848695-56862251	35	DGKE
chr6:83096730-83100886	13	DOPEY1
chr4:139060323-139062057	7	ELF2
chr12:22643762-22661205	8	ETNK1
chr2:169540050-169557297	9	FASTKD1
chr12:55789074-55800900	24	SARNP
chr11:66365381-66366564	38	SLC29A2
chr8:38819521-38820635	15	TACC1
chr3:44629064-44632599	8	ZNF197
chr19:11058257-11060187	4	SMARCA4
chr5:55343330-55366875	13	SKIV2L2
chr4:105386566-105438036	30	PPA2
chr1:27906927-27907282	14	RPA2
chr2:32429145-32453942	3	BIRC6
chr7:132838398-132885745	9	CHCHD3
chr1:236249884-236253505	3	ERO1B
chr2:24510968-24584560	9	NCOA1
chr7:5729431-5741644	12	RNF216
chr3:47658557-47678311	9	SMARCC1
chr1:204112914-204117707	75	SOX13
chr15:43017094-43025429	12	UBR1
chr4:41142986-41143142	11	APBB2
chr1:225554905-225567414	32	ENAH
chr1:155753203-155760641	4	GON4L
chr6:161048615-161049979	64	MAP3K4
chr3:9653620-9662375	3	MTMR14
chr3:58077045-58081776	97	FLNB
chr1:11073933-11076948	35	EXOSC10
chr3:9750944-9754885	30	OGG1

chrX:134377571-134378106	9	PHF6
chr5:77034417-77036720	45	AGGF1
chr2:152549020-152581049	3	FMNL2
chr20:63882718-63931022	3	TPD52L2
chr5:77436260-77440997	3	WDR41
chr9:71750042-71750385	105	TMEM2
chr1:225828724-225831959	7	EPHX1
chr1:51402434-51465326	13	EPS15
chr6:77088401-77151974	7	nogene
chr17:5297344-5308822	3	RABEP1
chr2:86770022-86771712	20	RMND5A
chr19:16081912-16089120	35	TPM4
chrX:118404337-118410959	37	WDR44
chr1:75732643-75750546	5	ACADM
chr4:129726921-129771562	5	LOC101927282
chr11:67281975-67282117	2	nogene
chr16:23621361-23626397	14	PALB2
chr2:85356966-85387225	6	ELMOD3
chr1:21861756-21862115	19	HSPG2
chr9:21837907-21854870	11	MTAP
chr12:102112107-102118583	27	NUP37
chr10:122392302-122406673	9	PLEKHA1
chr6:75633580-75634811	18	SENP6
chr20:21326278-21334185	19	XRN2
chr17:59257121-59272836	4	GDPD1
chr1:155926675-155927156	106	KIAA0907
chrX:24809897-24826601	21	POLA1
chr9:122877470-122897576	43	RC3H2
chr2:96192368-96195549	6	STARD7
chr16:879569-911079	42	LMF1
chr2:121397139-121410969	3	CLASP1
chr21:33904974-33905710	3	nogene
chr3:47427156-47428670	10	SCAP
chr1:179368799-179395202	5	AXDND1
chr14:46957373-47035304	37	MDGA2
chr9:120820838-120826907	16	PSMD5
chr6:45431862-45438051	31	RUNX2
chr1:243695590-243843282	27	AKT3
chr21:26050999-26053348	153	APP
chr10:31843460-31854206	8	ARHGAP12
chr20:51669996-51690819	16	ATP9A
chr12:111658735-111659345	47	BRAP
chr8:94885066-94885558	26	CCNE2

chr11:47472190-47489024	21	CELF1
chr22:28719394-28725367	8	CHEK2
chr2:207596913-207597483	5	CREB1
chr10:68934198-68936079	13	DDX50
chr2:171725617-171729853	13	DYNC1I2
chr3:5195208-5211216	7	EDEM1
chr14:99914178-99914697	14	EML1
chr10:49528416-49532978	3	ERCC6-PGBD3
chr2:201016990-201024039	81	FAM126B
chr5:108946129-109100519	2	FER
chr9:20819795-20874807	45	FOCAD
chr17:82582740-82584188	4	FOXK2
chr3:155893517-155931880	10	GMPS
chr19:41268222-41281275	90	HNRNPUL1
chr10:76969973-77019099	5	KCNMA1
chr17:47663088-47670832	17	KPNB1
chr10:70339995-70340702	11	LRRC20
chr20:5958527-5985000	28	MCM8
chr17:62010549-62011233	24	MED13
chr17:47456683-47491192	19	MRPL45P2
chr13:35040932-35048684	23	NBEA
chr3:23910810-23911450	69	NKIRAS1
chr16:4466152-4469465	131	NMRAL1
chr7:134145511-134145991	11	nogene
chr9:111005464-111011690	84	nogene
chr8:128009589-128070361	35	nogene
chr5:140281448-140300582	11	PFDN1
chr6:169710235-169721111	39	PHF10
chr7:11057951-11111467	2	PHF14
chr7:6735304-6737936	42	PMS2CL
chr11:65294152-65295990	13	POLA2
chr4:120754552-120811449	95	PRDM5
chr3:170259968-170280403	8	PRKCI
chrX:18320331-18334095	11	SCML2
chr5:134692601-134708888	2	SEC24A
chr2:241030283-241030571	11	SNED1
chr6:36506582-36525642	11	STK38
chr3:67495797-67498295	46	SUCLG2
chr3:17508473-17623913	13	TBC1D5
chr3:119500694-119513719	142	TIMMDC1
chr16:66517135-66549009	10	TK2
chr3:142736378-142792967	17	TRPC1
chr7:139279317-139284574	5	UBN2

chr17:78806155-78838750	4	USP36
chr1:21747066-21757783	31	USP48
chr9:133925842-133939219	159	VAV2
chr8:99192875-99275254	13	VPS13B
chr12:132932712-132933155	10	ZNF605
chr2:202297378-202297906	76	NOP58
chr6:47503279-47533755	34	CD2AP
chr4:105686691-105689280	12	INTS12
chr2:176296859-176330660	83	MTX2
chr1:32944018-32945628	39	RNF19B
chr13:75566694-75569507	9	UCHL3
chr2:61370320-61395233	27	USP34
chr14:31364188-31387375	12	HEATR5A
chr3:183663457-183672484	6	KLHL24
chr3:47086194-47098081	24	SETD2
chr4:169120818-169122266	3	SH3RF1
chr10:113086046-113089540	10	TCF7L2
chr8:127890588-128010444	11	nogene
chr12:123313619-123315660	53	SBNO1
chr15:44413335-44427772	6	CASC4
chr8:81713917-81718224	42	ZFAND1
chr14:35307993-35314455	15	PSMA6
chr11:3719411-3720825	16	NUP98
chr20:44620298-44622930	3	ADA
chr2:240523884-240525849	4	ANKMY1
chr8:102843550-102858150	15	AZIN1
chr8:129871374-129879471	3	FAM49B
chrX:107526581-107533550	27	FRMPD3
chr19:18147442-18147624	52	MAST3
chr22:29560852-29561170	23	NIPSNAP1
chr11:47815478-47819458	14	NUP160
chr17:45120172-45121372	12	PLCD3
chr7:87693907-87741579	12	RUNDC3B
chr4:138214584-138223324	4	SLC7A11
chr12:51116314-51117747	29	TFCP2
chr15:61972624-61981478	10	VPS13C
chr8:53970391-54010492	16	TCEA1
chr1:28683582-28697084	40	GMEB1
chr10:12400015-12403369	85	nogene
chr12:132729678-132730270	11	ANKLE2
chr12:56274776-56282991	9	CS
chr3:56638705-56641752	12	FAM208A
chr4:150896393-150929065	97	LRBA

chr7:7588497-7596461	11	MIOS
chr7:33635176-33642918	14	nogene
chr11:65436537-65444059	29	nogene
chr12:19320561-19322667	3	PLEKHA5
chr10:13605630-13611683	11	PRPF18
chr1:32825312-32853166	34	S100PBP
chr10:94500220-94511654	32	TBC1D12
chr11:122776218-122783222	2	UBASH3B
chr1:32779381-32782539	11	YARS
chr11:66061685-66061998	13	SF3B2
chr7:2069193-2200557	10	MAD1L1
chr16:29806676-29807422	12	nogene
chr18:58709377-58735329	13	MALT1
chr2:151440026-151446575	2	RIF1
chr3:81670837-81705613	89	GBE1
chr12:132151257-132151820	11	NOC4L
chr20:32366383-32428422	4	ASXL1
chr10:91996368-91997751	46	BTAF1
chr7:40078004-40078851	21	CDK13
chr19:12135774-12147834	7	nogene
chr4:3314790-3343053	36	RGS12
chr8:73292238-73292797	15	RPL7
chr16:30733279-30734615	15	SRCAP
chr4:6994183-7014557	42	TBC1D14
chr1:35370028-35370627	17	ZMYM4
chr16:11896557-11898035	34	GSPT1
chr19:4859589-4860024	19	PLIN3
chr5:138417482-138425582	4	KDM3B
chr3:42184684-42189124	20	TRAK1
chr5:132886309-132893118	13	AFF4
chr12:132735405-132755133	2	ANKLE2
chr11:129062279-129164427	9	ARHGAP32
chr2:215312497-215344871	10	ATIC
chr22:49797804-49799119	9	BRD1
chr9:135881632-135888273	29	CAMSAP1
chr1:180062219-180065272	10	CEP350
chr3:33592394-33644903	13	CLASP2
chr7:43639448-43647634	53	COA1
chr5:94050730-94074769	21	FAM172A
chr2:241434157-241435030	4	FARP2
chr17:67145742-67151224	17	HELZ
chr6:29889327-29943543	1260	HLA-H
chr10:72859176-72868863	13	MCU

chr6:89722954-89723619	38	MDN1
chrX:64328771-64359527	17	MTMR8
chr11:67125121-67125522	20	nogene
chr5:141593748-141594268	3	nogene
chr7:23185069-23187146	632	NUPL2
chr1:150460059-150460317	9	RPRD2
chr3:47042560-47067118	4	SETD2
chr2:109329223-109347949	56	SH3RF3
chr15:42638699-42652592	6	STARD9
chr12:63802090-63802405	2	TMEM5
chr12:22478312-22493337	37	C2CD5
chr16:70314901-70316104	14	DDX19B
chr12:50461134-50474167	14	LARP4
chr22:31258290-31260077	14	LIMK2
chr2:20278582-20327378	41	PUM2
chr6:105115685-105133588	17	BVES
chr11:63895158-63895633	51	MARK2
chr10:68959805-68963469	15	DDX21
chr2:47408400-47445657	39	MSH2
chr12:53308051-53320692	16	AAAS
chr2:3497670-3500993	14	ADI1
chr17:82183773-82188419	36	CCDC57
chrX:16689449-16702941	12	CTPS2
chr8:131949968-131955905	26	EFR3A
chr4:140525419-140543586	6	ELMOD2
chr4:2657802-2660054	18	FAM193A
chr2:222599927-222624775	18	FARSB
chr18:46118180-46120060	18	HAUS1
chr16:1602369-1602591	6	IFT140
chr11:31456259-31488241	16	IMMP1L
chr7:152194008-152199459	10	KMT2C
chr16:4681549-4683932	3	MGRN1
chr13:23870012-23886506	52	MIPEP
chr20:23381212-23403072	3	NAPB
chr1:247333134-247334426	3	nogene
chr4:2949933-2951245	20	NOP14
chr10:34450316-34470263	20	PARD3
chr4:105396248-105438036	63	PPA2
chr8:22474953-22513432	16	PPP3CC
chr4:82827368-82829058	30	SEC31A
chr1:151658234-151668635	102	SNX27
chr3:67400730-67409034	29	SUCLG2
chr12:103982798-103985728	10	TDG

chr16:69366806-69372355	13	TERF2
chr6:52516005-52516737	23	TRAM2
chr1:193069033-193077050	10	TROVE2
chr15:43380083-43383504	8	TUBGCP4
chr1:46309896-46310316	23	UQCRH
chr18:56816030-56880165	13	WDR7
chr19:52373110-52406643	14	ZNF880
chr10:92143028-92145112	81	CPEB3
chr15:41356038-41377304	124	NUSAP1
chr1:35913841-35915542	10	AGO1
chr10:31808648-31814361	3	ARHGAP12
chr7:157339862-157367483	3	DNAJB6
chr1:92693801-92697985	12	EVI5
chr12:122568260-122577029	30	KNTC1
chr18:57611636-57615975	46	NARS
chr3:31830039-31879830	5	OSBPL10
chr7:22492564-22494886	10	STEAP1B
chr17:35817715-35822833	8	TAF15
chr4:84708908-84717016	21	WDFY3
chr2:236120191-236124199	29	AGAP1
chr15:72544819-72572165	21	ARIH1
chr19:32901244-32915517	17	CEP89
chr2:29121373-29192257	3	CLIP4
chr3:197059888-197069260	4	DLG1
chr16:1816892-1820014	3	HAGH
chr19:6936343-6936717	4	nogene
chr12:8920984-8921750	11	PHC1
chr1:112576985-112591603	13	ST7L
chr17:43104121-43115779	10	BRCA1
chr10:27093348-27093799	9	ANKRD26
chr22:17864898-17896981	4	MICAL3
chr1:226152334-226159358	12	ACBD3
chr15:85664562-85693451	15	AKAP13
chr6:157084661-157084905	12	ARID1B
chr2:25799384-25806337	168	ASXL2
chr3:142465096-142470183	4	ATR
chr2:32377587-32406557	223	BIRC6
chr10:59804419-59814755	12	CCDC6
chr3:182947237-182965753	26	DCUN1D1
chrX:131768548-131785258	39	FIRRE
chr17:82563353-82568201	839	FOXK2
chr7:111487237-111521449	83	IMMP2L
chr19:10499394-10500080	20	KEAP1

chr17:43810806-43811205	3	MPP3
chr9:122280442-122291840	237	MRRF
chr17:1480526-1480885	82	MYO1C
chr5:85746189-85821466	3	nogene
chrX:56002824-56014961	14	nogene
chr10:78007701-78010540	11	POLR3A
chr8:140803542-140890769	3	PTK2
chr3:50060955-50062108	33	RBM6
chr1:113777209-113777808	39	RSBN1
chr20:44503814-44504891	10	SERINC3
chr10:70844472-70859499	14	SGPL1
chr17:18340042-18347656	11	SHMT1
chr3:125497340-125504744	27	SNX4
chr6:149369908-149379518	3	TAB2
chr3:17508473-17562142	5	TBC1D5
chr3:142736378-142793960	7	TRPC1
chr7:67017852-67018143	14	TYW1
chr12:122263571-122264199	8	VPS33A
chr4:201080-211885	5	ZNF718
chr10:91951402-91953873	35	BTAF1
chr19:40333850-40336407	15	C19orf47
chr1:88857205-88860286	9	GTF2B
chr13:20842894-20868697	12	XPO4
chr13:31229184-31286819	14	B3GLCT
chr18:59438110-59439816	26	CCBE1
chr12:1289851-1371977	106	ERC1
chr6:35637013-35680422	7	FKBP5
chrX:154695916-154700059	36	GAB3
chr19:19492305-19496219	10	GATAD2A
chr10:74838682-74843478	166	KAT6B
chr6:89670918-89673462	11	MDN1
chr10:124408541-124408965	26	OAT
chr20:38531166-38541192	12	RALGAPB
chrX:46651109-46653705	55	SLC9A7
chr7:67024899-67067403	5	TYW1
chr1:193028084-193029657	39	UCHL5
chr19:57861375-57862570	15	nogene
chr15:90766909-90769586	32	BLM
chr19:4422565-4433539	12	CHAF1A
chr6:42934474-42935670	179	CNPY3
chr1:173830659-173853905	13	DARS2
chr5:73051348-73068779	23	FCHO2
chr12:123194385-123198334	13	MPHOSPH9

chr7:596145-607452	12	PRKAR1B
chr2:68137298-68141795	38	WDR92
chr8:140585118-140605914	208	AGO2
chr5:78216054-78267595	7	AP3B1
chr6:80200933-80273221	4	BCKDHB
chr2:58163433-58165874	5	FANCL
chrX:72462003-72474729	9	HDAC8
chr15:40386361-40392023	9	KNSTRN
chr7:130984052-130988888	3	LINC-PINT
chr14:70742350-70742591	18	MAP3K9
chr10:46014443-46016694	54	NCOA4
chr3:72708843-72716617	23	nogene
chr3:151795738-151803925	68	nogene
chr3:127003329-127007913	11	PLXNA1
chr18:9512990-9513289	15	RALBP1
chr16:53439015-53451831	4	RBL2
chr6:107911345-107924932	8	SEC63
chr10:10786644-10792525	10	SFTA1P
chr17:64578491-64606640	7	SMURF2
chr16:68867078-68880630	48	TANGO6
chr16:69370482-69372355	332	TERF2
chr1:185124943-185137796	12	TRMT1L
chr15:43007078-43025429	15	UBR1
chr8:17265906-17268956	81	VPS37A
chr10:86451966-86461287	24	WAPL
chr4:84849901-84879494	12	WDFY3
chr13:26349071-26353880	7	CDK8
chrX:44524001-44527365	325	FUNDC1
chr5:135343259-135346057	5	H2AFY
chr9:38544746-38545185	7	nogene
chr5:143054438-143057747	53	ARHGAP26
chr11:47484388-47489024	31	CELF1
chr7:139715931-139726108	18	HIPK2
chr3:56565637-56567053	41	CCDC66
chr19:10772478-10777216	45	DNM2
chr1:20893516-20942330	29	EIF4G3
chr1:240329075-240330749	21	FMN2
chr12:42393569-42398994	46	PPHLN1
chr4:4648976-4653757	7	STX18-AS1
chr5:72861807-72875737	13	TNPO1
chr4:75806485-75812375	11	USO1
chr12:22474750-22484888	9	C2CD5
chr18:49272177-49286616	12	DYM

chr1:219972068-219978719	17	EPRS
chr18:26476514-26501250	9	KCTD1
chr7:67297216-67299424	3	PMS2P4
chr11:70343668-70348420	8	PPFIA1
chr9:100240059-100253136	4	INVS
chr14:45006007-45025872	16	FAM179B
chrX:50042315-50048103	11	CLCN5
chr14:52860340-52864853	3	FERMT2
chr12:2880260-2880674	13	nogene
chr3:27418485-27452498	28	SLC4A7
chr7:152783186-152801731	280	ACTR3B
chr2:29121373-29145367	23	CLIP4
chr11:103219914-103223086	49	DYNC2H1
chr5:154376276-154380631	6	GALNT10
chr1:219193081-219241308	64	LYPLAL1
chr17:76738281-76776541	17	MFSD11
chr20:43686851-43700044	29	MYBL2
chr4:88061905-88065879	49	PKD2
chr11:77725544-77747128	19	RSF1
chr20:4902441-4932716	9	SLC23A2
chr7:105638352-105643121	28	ATXN7L1
chr22:27894554-27897892	30	PITPNB
chr7:66423378-66423796	47	nogene
chr2:172564502-172596023	11	PDK1
chr5:50399106-50428227	3	EMB
chr2:196656632-196701180	10	CCDC150
chr12:26628032-26655852	32	ITPR2
chr6:89803327-89803554	11	MDN1
chr9:36580072-36583712	16	MELK
chr16:69119754-69119913	36	nogene
chr3:27381867-27382359	14	nogene
chr20:35714184-35725155	1043	RBM39
chr2:226906792-226914351	383	RHBDD1
chr1:23694659-23697445	2	RPL11
chr9:128345154-128345549	3	SLC27A4
chr3:41681507-41717861	4	ULK4
chr10:12209966-12238485	9	CDC123
chr7:32974616-32988652	6	FKBP9
chr19:726137-727694	4	PALM
chr16:66528993-66549009	4	TK2
chr2:203366876-203402734	10	ABI2
chr18:50895808-50940386	10	ME2
chr3:146079115-146091901	9	PLOD2

chr5:177537872-177539147	20	FAM193B
chr3:47729024-47745993	19	SMARCC1
chr14:51698141-51702588	24	FRMD6
chrX:118741016-118766976	10	IL13RA1
chr6:43498974-43503706	3	TJAP1
chr12:26996741-27003390	3	TM7SF3
chr11:120476660-120477526	56	ARHGEF12
chr14:55181212-55183753	34	DLGAP5
chr7:42023467-42026412	28	GLI3
chr11:62824165-62825117	10	STX5
chr1:35816881-35835993	3	AGO4
chr5:126546323-126552137	11	ALDH7A1
chr1:20969473-20980448	30	EIF4G3
chr7:2794927-2814389	5	GNA12
chr1:100067976-100068586	3	HIAT1
chrX:149490313-149501037	9	IDS
chr7:152202933-152207428	9	KMT2C
chr3:25751097-25778688	15	NGLY1
chr7:154975695-154998784	9	PAXIP1
chr8:42344952-42352568	43	POLB
chr1:112576985-112616844	13	ST7L
chr15:42958012-42970607	40	UBR1
chr10:130159994-130180321	18	GLRX3
chr1:94455732-94468007	17	ABCD3
chr15:77364073-77365225	5	nogene
chr3:194459470-194462236	211	ATP13A3
chr5:112259228-112266330	24	EPB41L4A
chr1:236594011-236596976	24	HEATR1
chr10:132690391-132726905	19	INPP5A
chr1:246540937-246556721	10	TFB2M
chr15:50631416-50634556	6	TRPM7
chr14:88726540-88754671	80	EML5
chr19:50061799-50067800	5	FLJ26850
chr1:213071005-213117410	43	RPS6KC1
chr7:100075134-100075630	9	ZNF3
chr1:109340723-109345878	26	SORT1
chr1:243637610-243664883	7	AKT3
chr5:179841906-179851424	5	C5orf45
chr15:90598449-90600441	4	nogene
chr15:34927034-34934635	3	AQR
chr1:150823193-150826617	5	ARNT
chr3:194427074-194427252	31	ATP13A3
chr17:61743012-61808757	11	BRIP1

chr21:32603160-32610005	5	C21orf59
chr22:31784813-31798656	10	DEPDC5
chr19:32432715-32437339	11	DPY19L3
chr1:97549559-97595166	16	DPYD
chr8:116726015-116755797	10	EIF3H
chr20:46380252-46389220	10	ELMO2
chr22:41125863-41127748	31	EP300
chr1:240333886-240355908	5	FMN2
chr10:119430374-119455246	10	GRK5
chrX:72462003-72489041	26	HDAC8
chr10:68025545-68067217	35	HERC4
chr1:153665219-153668557	174	ILF2
chr7:66638252-66643611	6	KCTD7
chr2:20739970-20790398	11	LDAH
chr2:43960540-43975217	17	LRPPRC
chr5:109713495-109729513	12	MAN2A1
chrX:136219397-136222486	4	MAP7D3
chr5:80775693-80792844	282	MSH3
chr8:96243918-96258700	17	MTERF3
chr13:77205255-77243951	17	MYCBP2
chr12:78159202-78168866	6	NAV3
chr18:79433578-79467582	10	NFATC1
chr9:97135545-97139954	32	nogene
chr20:37307182-37307537	50	nogene
chr7:106371655-106372442	11	nogene
chr10:89335016-89335534	4	nogene
chr9:2690950-2782846	20	nogene
chr19:51839892-51843315	2	nogene
chr5:179831569-179831878	62	nogene
chr22:32268287-32273507	34	nogene
chrX:71555953-71557672	51	OGT
chr4:39902346-39925933	27	PDS5A
chr3:48782985-48794049	8	PRKAR2A
chr9:133410984-133412921	10	REXO4
chr11:71982806-71990717	27	RNF121
chr1:185087551-185091739	9	RNF2
chr20:36904156-36905503	10	SAMHD1
chr6:154808069-154824378	10	SCAF8
chr2:109567814-109585838	42	10-Sep
chr1:206205428-206206037	8	SRGAP2
chr12:112130510-112135066	42	TRAFD1
chr11:11873799-11880380	12	USP47
chr17:4081375-4086655	33	ZZEF1

chr5:65242103-65291470	40	ADAMTS6
chr18:23561359-23572180	15	NPC1
chr13:32325075-32326613	38	BRCA2
chr17:39490556-39492890	54	CDK12
chr6:4935514-4937737	10	CDYL
chr9:137743370-137762820	14	EHMT1
chr10:97437190-97438703	12	EXOSC1
chr16:56758537-56805632	5	NUP93
chr3:58431592-58431984	6	PDHB
chr4:102799406-102826636	17	UBE2D3
chr7:157183877-157201807	12	UBE3C
chr6:31784213-31785733	11	VARS
chr4:36210389-36214480	54	ARAP2
chr10:121902390-121922411	16	ATE1
chr19:47352753-47362693	77	DHX34
chr2:27350313-27350632	315	GTF3C2
chr7:98244570-98246165	4	TECPR1
chr2:135335399-135345635	16	ZRANB3
chr2:231206197-231226798	6	ARMC9
chr16:70361417-70365131	3	DDX19A
chr7:6581606-6582251	12	ZDHHC4
chr15:72007825-72046634	13	MYO9A
chr14:103669510-103673431	23	KLC1
chr7:103025062-103047020	11	FBXL13
chr22:29137341-29142100	12	KREMEN1
chr10:119857346-119859884	20	MCMBP
chr1:45614095-45616393	48	NASP
chr10:34470084-34517159	37	PARD3
chr11:34957383-34980503	12	PDHX
chr4:13376544-13425227	12	RAB28
chrX:107088435-107088839	348	RBM41
chr3:195688532-195689438	188	SDHAP2
chr7:96184276-96208977	4	SLC25A13
chr3:67609454-67637839	11	SUCLG2
chr4:147879231-147881932	82	ARHGAP10
chr5:74840073-74841679	61	FAM169A
chr15:71978170-72010447	12	MYO9A
chrX:17687741-17692468	55	NHS
chr10:47501279-47501514	13	nogene
chrX:68096869-68113239	14	OPHN1
chr3:171642839-171677694	3	PLD1
chr19:47150201-47153040	4	SAE1
chr9:77238001-77260224	3	VPS13A

chr12:76805316-76809857	6	ZDHHC17
chr7:99333324-99344515	19	ARPC1A
chr15:34003031-34003209	4	AVEN
chr14:88657375-88665489	12	EML5
chr19:43506722-43508060	16	ETHE1
chr9:93527154-93532329	14	FAM120A
chr1:16305802-16315435	63	FBXO42
chr7:5603256-5604030	18	FSCN1
chr14:66965190-67023675	12	GPHN
chr14:55648024-55651927	17	KTN1
chr10:124462502-124462962	17	nogene
chr3:196061801-196062273	24	nogene
chr2:10787280-10793202	4	PDIA6
chr3:170136418-170145521	34	PHC3
chr18:9588088-9595102	47	PPP4R1
chr3:45703437-45723543	3	SACM1L
chr14:54736675-54737287	17	SAMD4A
chr2:200532973-200536142	18	SGOL2
chr7:56068481-56073111	23	SUMF2
chr19:49342437-49360081	39	TEAD2
chr6:44134560-44141098	17	TMEM63B
chr15:42827927-42830078	11	TTBK2
chr1:28742402-28743986	124	YTHDF2
chr8:80499493-80500382	47	ZBTB10
chr22:28724976-28734727	13	CHEK2
chr7:5528003-5528406	10	ACTB
chr15:60355918-60364523	33	ANXA2
chr11:43326506-43335994	139	API5
chr8:123356388-123361646	75	ATAD2
chr3:113778740-113786383	28	ATP6V1A
chr10:118729294-118730410	298	CACUL1
chr9:97347313-97350555	28	CCDC180
chr16:69155870-69168998	40	CIRH1A
chr3:41224020-41236709	4	CTNNB1
chr5:112262493-112280323	16	EPB41L4A
chr9:95897848-95941549	12	ERCC6L2
chr1:42265114-42323827	103	FOXJ3
chr1:1806474-1825499	30	GNB1
chr21:33781460-33782133	11	ITSN1
chr4:165228806-165263359	11	KLHL2
chrX:150445453-150471490	11	MAMLD1
chr21:36333645-36341546	4	MORC3
chr1:156132377-156132934	9	nogene

chr5:37061586-37061936	14	nogene
chr11:65503853-65504595	8	nogene
chr7:135576968-135578915	9	NUP205
chr10:91240483-91271697	2	PCGF5
chr10:27532506-27533994	118	RAB18
chr7:85036888-85042285	9	SEMA3D
chr7:140334633-140337349	41	SLC37A3
chr17:81260237-81284895	12	SLC38A10
chr19:11033289-11060187	9	SMARCA4
chr17:20245925-20257607	9	SPECC1
chr10:14896845-14897517	35	SUV39H2
chr2:171053760-171082852	20	TLK1
chr11:87067693-87071615	16	TMEM135
chr7:139273296-139274074	6	UBN2
chr3:108191520-108214021	12	IFT57
chr16:14079289-14140760	21	MKL2
chr19:13912322-13913638	18	CC2D1A
chr7:6416626-6421804	3	DAGLB
chr8:131949968-131959663	9	EFR3A
chr8:100715461-100718280	13	PABPC1
chr16:47463881-47503090	4	PHKB
chr17:20253504-20260294	43	SPECC1
chr8:25298943-25300657	32	DOCK5
chr11:77978995-77991299	12	INTS4
chr16:48256609-48277479	130	LONP2
chr7:24649611-24650712	33	MPP6
chr12:111702161-111709684	2	ACAD10
chr5:135052453-135075388	17	C5orf66
chr14:45008912-45032376	6	FAM179B
chr9:85815878-85823080	19	LOC389765
chr4:102714437-102736435	13	MANBA
chr9:81654639-81655118	2	nogene
chr14:30715923-30722559	16	SCFD1
chr11:66063627-66068333	9	SF3B2
chr10:94441895-94493447	3	TBC1D12
chr3:12489783-12492217	12	TSEN2
chr17:81588943-81608822	7	NPLOC4
chr2:25767582-25806337	10	ASXL2
chr22:28719394-28734727	19	CHEK2
chr12:108652271-108662158	13	CORO1C
chr7:156826604-156836885	224	LMBR1
chr6:89781398-89790401	6	MDN1
chr16:56818663-56823846	18	NUP93

chr19:46375603-46376574	18	PPP5C
chr19:10328641-10330526	18	RAVER1
chr13:46020448-46045516	10	ZC3H13
chr13:100257594-100273346	174	PCCA
chr19:7458511-7459994	5	ARHGEF18
chr10:68459103-68470163	401	DNA2
chr9:96918642-96942752	87	nogene
chr7:128982247-128997535	5	TNPO3
chr9:95966561-95978215	23	ERCC6L2
chr2:171976156-171979363	5	HAT1
chr7:919967-926644	16	ADAP1
chr7:140794307-140808995	92	BRAF
chr3:128637798-128644983	32	RPN1
chr2:23819746-23823569	14	ATAD2B
chr5:69294792-69311204	93	CCDC125
chr12:121416158-121420778	15	RNF34
chr17:82903404-82909307	18	TBCD
chr16:47497398-47610920	47	PHKB
chr1:26447558-26460144	24	DHDDS
chr15:63764095-63775649	15	HERC1
chr1:83947123-83952387	3	TTLL7
chr10:121836717-121841263	6	ATE1
chr8:102834188-102836390	47	AZIN1
chrX:1288518-1288888	31	CSF2RA
chr18:48757918-48761689	23	CTIF
chr3:129458598-129464781	10	IFT122
chr6:111262256-111263946	166	KIAA1919
chr21:36338774-36344707	313	MORC3
chr10:68121437-68135512	3	MYPN
chr2:152085567-152086011	31	nogene
chr14:22833885-22834553	16	nogene
chr15:25266430-25278884	12	nogene
chr17:42380685-42381464	2	nogene
chr1:6533279-6549698	21	NOL9
chr7:82822494-82841509	16	PCLO
chr8:140818276-140830526	109	PTK2
chr1:31910037-31911826	106	PTP4A2
chr1:52978216-52988136	11	SCP2
chr3:123209198-123259589	27	SEC22A
chr17:38361710-38368050	135	SOCS7
chr17:78092932-78093763	2	TNRC6C
chr19:43988458-43991934	11	ZNF155
chr7:149074385-149080717	11	ZNF786

chr2:31917925-31932135	837	MEMO1
chr5:172382035-172406352	12	SH3PXD2B
chr1:59321535-59378837	80	FGGY
chr2:36578596-36591011	196	FEZ2
chr5:80174953-80178064	10	SERINC5
chr16:50354424-50354922	2	BRD7
chr11:95812931-95813589	215	CEP57
chr7:111808820-111834686	13	DOCK4
chr12:122584277-122588816	37	KNTC1
chr14:54702061-54751537	12	SAMD4A
chr11:70798442-70820682	21	SHANK2
chr5:128148753-128161800	2	SLC12A2
chr7:857827-872562	2	SUN1
chr14:102376649-102408619	13	TECPR2
chr1:154170399-154173201	38	TPM3
chr12:49325500-49329454	13	TROAP
chr2:25799384-25856839	2	ASXL2
chr10:32048463-32050523	13	KIF5B
chrX:77688817-77717243	10	ATRX
chr4:76144148-76144473	153	NUP54
chr1:51745535-51761971	4	OSBPL9
chr11:20452129-20464546	21	PRMT3
chr7:129205202-129206587	939	SMO
chr3:12496517-12519197	4	TSEN2
chr16:11774235-11782388	25	ZC3H7A
chr2:69530007-69532162	14	AAK1
chr3:43549719-43580472	8	ANO10
chr19:17271596-17275825	13	BABAM1
chr5:96722638-96729725	20	CAST
chr2:55308808-55309254	21	CCDC88A
chr19:12928341-12928936	12	FARSA
chr10:67966682-68025676	15	HERC4
chr4:127921523-127939997	61	MFSD8
chr15:48165932-48168839	15	MYEF2
chr11:17356650-17379665	10	NCR3LG1
chr1:28476106-28480604	17	PHACTR4
chr20:62139780-62140944	15	PSMA7
chr1:24514313-24533254	5	RCAN3
chr5:115130591-115137616	41	TRIM36
chr7:67024899-67055887	17	TYW1
chr1:179112308-179117516	19	ABL2
chrX:109674401-109678415	4	ACSL4
chr2:113913171-113942359	16	ACTR3

chrX:85046445-85074391	5	APOOL
chr11:47166267-47168251	38	ARFGAP2
chr2:9279316-9323250	19	ASAP2
chr3:194425341-194431892	10	ATP13A3
chr15:34003031-34023497	35	AVEN
chr14:60149352-60153178	27	DHRS7
chr9:129978464-129979374	5	FNBP1
chr1:155756957-155760641	6	GON4L
chr11:33286412-33328633	60	HIPK3
chr19:18175039-18175697	195	IFI30
chr2:210129030-210155404	3	KANSL1L
chr12:350620-356537	30	KDM5A
chr15:69417382-69422435	37	KIF23
chr7:152229922-152238826	139	KMT2C
chr12:122601535-122624688	9	KNTC1
chr1:117442230-117466427	77	MAN1A2
chr1:117402185-117420649	2575	MAN1A2
chr3:47867245-47877523	11	MAP4
chr22:40519463-40552367	7	MKL1
chr8:65669872-65704929	16	MTFR1
chr3:172645622-172648114	297	NCEH1
chr1:236032400-236038253	18	NID1
chr20:63910450-63910767	37	nogene
chr19:31228004-31232280	7	nogene
chr2:130066454-130067902	4	nogene
chrX:65352872-65368998	17	nogene
chr5:172259407-172259810	16	nogene
chr17:81610209-81629805	7	NPLOC4
chr5:43543003-43547913	12	PAIP1
chr7:140034234-140047007	13	PARP12
chr3:196716857-196728136	10	PIGX
chr5:149836262-149845914	6	PPARGC1B
chr14:58285590-58291707	11	PSMA3-AS1
chr17:67342185-67344780	6	PSMD12
chr4:26386352-26424743	21	RBPJ
chr7:832504-843232	12	SUN1
chr3:119503531-119513719	7	TIMMDC1
chr5:88220589-88228528	38	TMEM161B
chr5:14405847-14420021	277	TRIO
chr15:42817031-42830078	10	TTBK2
chr1:230943805-230961303	185	TTC13
chr7:139258487-139266403	11	UBN2
chr1:77734346-77739417	16	USP33

chr10:73504849-73505426	17	USP54
chr2:144424795-144517419	13	ZEB2
chr13:77243058-77264002	11	MYCBP2
chr3:57871635-57896932	14	SLMAP
chr5:65291328-65300131	27	ADAMTS6
chr9:19423861-19424841	6	ACER2
chr3:198019252-198024788	6	LMLN
chr1:17580552-17588479	141	ARHGEF10L
chr17:47585499-47590881	7	NPEPPS
chr12:98597855-98598703	13	SLC25A3
chr6:135318518-135346796	30	AHI1
chr1:70292387-70315566	527	ANKRD13C
chr15:40628069-40650819	23	CASC5
chr15:65729071-65738875	19	DENND4A
chr8:116726015-116734354	10	EIF3H
chr1:20941490-20942330	7	EIF4G3
chr2:31891991-31932135	151	MEMO1
chr6:43671839-43675617	4	MRPS18A
chr16:48546146-48562444	20	N4BP1
chr14:74119855-74120164	10	nogene
chr4:730629-744688	10	PCGF3
chr22:46235132-46235509	4	PPARA
chr18:8624937-8635622	14	RAB12
chr3:134183001-134191974	11	RYK
chr19:38119305-38119882	81	SIPA1L3
chr11:62881892-62888218	5	SLC3A2
chr7:127698874-127721400	5	SND1
chr6:35888023-35891013	30	SRPK1
chr20:49135832-49144649	3	STAU1
chr12:6869257-6870136	19	TPI1
chr5:14290715-14330900	11	TRIO
chr1:32184524-32188124	39	TXLNA
chr9:33986759-34017189	62	UBAP2
chr3:114350273-114351878	77	ZBTB20
chr19:9658008-9660874	7	ZNF562
chr20:24969525-24973737	14	APMAP
chr15:55376948-55389433	3	DYX1C1-CCPG1
chr10:119107947-119132609	13	FAM45A
chr9:96458378-96484633	42	HABP4
chr15:20461191-20466370	34	HERC2P3
chr2:131125752-131130760	20	PLEKHB2
chr13:19741564-19751467	10	PSPC1
chr6:138941334-138947913	64	REPS1

chr15:41475524-41477515	12	RTF1
chr2:168129540-168140758	125	STK39
chr17:42613646-42663503	18	TUBG1
chr16:74915869-74916259	17	WDR59
chr20:41202056-41205066	16	ZHX3
chr16:89100661-89102759	27	ACSF3
chr13:48253807-48261321	12	ITM2B
chr8:22196679-22197420	3	BMP1
chrX:91562992-91661744	3	nogene
chr1:173751763-173757159	34	KLHL20
chr11:118581245-118584644	46	ARCN1
chr22:46386401-46389499	27	CELSR1
chr11:68780639-68785010	5	CPT1A
chr19:45093083-45093527	3	PPP1R37
chr8:42436988-42437577	20	SLC20A2
chr13:32362522-32363533	17	BRCA2
chr1:247860616-247876037	14	TRIM58
chr17:46082440-46094701	204	KANSL1
chr5:108883395-108959347	36	FER
chr20:3281640-3287790	10	C20orf194
chr14:50456515-50486194	10	MAP4K5
chr11:119124029-119125460	10	nogene
chr19:43155171-43182918	10	nogene
chr3:146102754-146124229	216	PLOD2
chr5:149835300-149845914	10	PPARGC1B
chr9:124971369-124971844	3	SCAI
chr20:13587249-13630152	11	TASP1
chr2:43428099-43485325	5	THADA
chr5:168453967-168460742	16	WWC1
chr3:98768429-98773983	95	ST3GAL6
chr12:10709907-10710788	13	YBX3
chr5:126582850-126593404	4	ALDH7A1
chr4:1228198-1241519	223	CTBP1
chr2:69348170-69363670	27	GFPT1
chrX:77829030-77830895	15	MAGT1
chr9:13247634-13250372	18	MPDZ
chr1:9726908-9727772	10	PIK3CD
chr9:26928099-26935206	3	PLAA
chr14:22926132-22926814	214	PRMT5
chr16:74300114-74304394	3	PSMD7
chr1:156265780-156274686	15	SMG5
chr2:128170287-128171284	15	UGGT1
chr17:51217333-51225209	103	MBTD1

chr1:70292387-70300908	37	ANKRD13C
chr17:8161472-8162369	7	VAMP2
chr10:114830851-114836246	24	FAM160B1
chr15:50300788-50303123	11	GABPB1
chr16:15691143-15696860	10	NDE1
chr5:87332506-87346724	10	RASA1
chr1:28571175-28571900	25	TRNAU1AP
chr3:104630284-104630579	27	nogene
chrX:139757807-139774953	18	ATP11C
chr17:61074919-61084564	89	BCAS3
chr15:76287845-76295737	6	ETFA
chr2:239156651-239163923	53	HDAC4
chr1:220136808-220139139	9	IARS2
chrX:70329404-70333689	21	KIF4A
chrX:129540743-129588263	6	OCRL
chr2:147972738-147975975	468	ORC4
chr2:196870940-196873758	4	PGAP1
chr2:20297552-20333063	20	PUM2
chr17:82756164-82797802	10	TBCD
chr3:51463113-51496780	10	VPRBP
chr13:52416016-52427219	2	VPS36
chr12:51073822-51076647	31	CSRNP2
chr4:41598919-41619440	13	LIMCH1
chr10:4830674-4889923	6	AKR1E2
chr11:108267170-108268609	31	ATM
chr15:81127589-81143820	10	C15orf26
chr12:22482556-22506819	19	C2CD5
chr22:46308149-46308943	14	GTSE1
chr2:37056439-37057480	9	HEATR5B
chr9:125238140-125239533	10	HSPA5
chr1:117402185-117466427	153	MAN1A2
chr2:171325840-171331867	32	METTL8
chr4:103547853-103593429	20	nogene
chr12:6882195-6883919	46	nogene
chrX:27158904-27176384	27	nogene
chr15:96191351-96201643	4	NR2F2-AS1
chr10:45888164-45962994	28	PARGP1
chr7:154983218-154998784	90	PAXIP1
chr11:68544837-68571104	4	PPP6R3
chr9:69723325-69741858	9	PTAR1
chr10:70152262-70161069	14	SAR1A
chr3:112563384-112570670	3	SLC35A5
chr1:47295764-47301748	140	STIL

chr9:6420911-6465846	14	UHRF2
chr14:55010308-55013596	6	WDHD1
chr1:100995109-101025466	16	DPH5
chr7:4989062-5005228	12	RNF216P1
chr10:121790168-121841263	3	ATE1
chr7:16665436-16674588	16	BZW2
chr16:53303719-53306397	24	CHD9
chr14:45025782-45028329	21	FAM179B
chr11:93788000-93790793	15	MED17
chr6:88844356-88904955	25	RNGTT
chr2:61325374-61333971	6	USP34
chr9:86308428-86310017	8	ZCCHC6
chr15:44484223-44486700	46	CTDSPL2
chr5:618989-644425	11	CEP72
chr15:63526919-63537156	6	USP3
chr1:1825396-1853297	6	GNB1
chr19:17101659-17102557	320	MYO9B
chr6:35619095-35680422	34	FKBP5
chr14:57274243-57280422	10	AP5M1
chr6:129583987-129618852	17	ARHGAP18
chr2:175111567-175114868	4	ATF2
chr2:61162841-61164540	161	C2orf74
chr3:15230441-15233973	13	CAPN7
chr20:5173522-5175279	13	CDS2
chr1:114725220-114738072	12	CSDE1
chr2:224535527-224567111	13	CUL3
chr5:151790322-151794249	6	G3BP1
chr6:2263600-2273417	27	GMDS-AS1
chr7:23140768-23144025	24	KLHL7
chr14:19064582-19066347	39	LINC01296
chr17:51201591-51203228	3	MBTD1
chr8:47969797-47971468	54	MCM4
chr13:23798723-23841488	7	MIPEP
chr2:177232391-177234271	34	NFE2L2
chr20:61963440-61964030	28	nogene
chr11:62522629-62526511	56	nogene
chr7:24816609-24834736	8	OSBPL3
chr14:29599026-29626556	19	PRKD1
chr14:29624151-29636494	61	PRKD1
chr4:109473003-109481781	9	SEC24B
chr1:151638887-151666011	9	SNX27
chr12:2994737-2994992	43	TEAD4
chr10:119576610-119588248	7	TIAL1

chr6:43562246-43573601	18	XPO5
chr10:34347964-34360259	16	PARD3
chr12:42374862-42387535	16	PPHLN1
chr1:173992754-173993135	159	RC3H1
chr8:9016321-9020464	19	ERI1
chr9:78254835-78264316	36	CEP78
chr4:39081962-39086727	9	KLHL5
chr5:71501561-71504751	10	BDP1
chr3:154284582-154295339	11	DHX36
chr7:770470-775162	344	DNAAF5
chr5:108954728-109047198	15	FER
chr8:94836523-94842488	11	INTS8
chr1:10271501-10276399	10	KIF1B
chr10:79804484-79808832	11	NUTM2B-AS1
chr1:30974650-30982640	5	PUM1
chr7:140776911-140783157	35	BRAF
chr1:155438326-155477958	8	ASH1L
chr22:37496448-37497178	39	CARD10
chr13:60093627-60132989	49	DIAPH3
chr5:151786602-151800869	9	G3BP1
chr15:41005592-41027736	10	INO80
chrX:70341798-70353807	210	KIF4A
chr17:5214049-5215293	9	LOC100130950
chr3:100339086-100348881	6	NIT2
chrX:102897573-102905737	33	nogene
chr11:108192117-108197420	11	NPAT
chr1:51745535-51772723	13	OSBPL9
chr20:18142207-18150942	23	PET117
chr8:128009589-128070272	25	PVT1
chr6:3076763-3083313	31	RIPK1
chr7:45751528-45759174	26	SEPT7P2
chr2:171833962-171855949	7	SLC25A12
chr14:34593044-34609742	28	SNX6
chr20:37393894-37396311	14	SRC
chr1:77729859-77736158	12	USP33
chr4:84702352-84709347	4	WDFY3
chr1:153639145-153640498	10	nogene
chr11:60897845-60903861	10	PRPF19
chr1:64049690-64050716	39	ROR1
chr17:37330172-37330425	29	ACACA
chr1:92665938-92704744	99	EVI5
chr19:14039507-14039910	99	IL27RA
chr6:73425463-73440445	14	MB21D1

chr6:132372607-132374777	17	MOXD1
chr17:44801060-44801884	16	nogene
chr7:111386973-111390694	5	nogene
chr9:6475390-6477808	20	UHRF2
chr10:102699372-102705489	4	ARL3
chr14:96832430-96860735	6	VRK1
chr19:32625873-32628849	39	ANKRD27
chrX:108595508-108603061	10	COL4A5
chr7:122379367-122416164	7	CADPS2
chr7:2229983-2230764	90	MAD1L1
chr11:977094-981299	5	AP2A2
chr7:24679130-24680520	46	MPP6
chr2:206132944-206142831	4	NDUFS1
chr1:35385441-35390098	6	ZMYM4
chr4:112253207-112266716	4	AP1AR
chr14:54408744-54415930	13	CDKN3
chr7:137901353-137916012	20	CREB3L2
chr3:81642780-81705613	91	GBE1
chr9:4117767-4118881	30	GLIS3
chr10:72715110-72715902	26	MCU
chr14:45235813-45237721	14	MIS18BP1
chr22:41264663-41281082	15	RANGAP1
chr8:99134631-99148010	16	VPS13B
chr2:171468887-171469452	26	DCAF17
chr19:50394562-50394930	12	nogene
chr1:16630889-16633346	18	CROCCP2
chr1:119146952-119150746	14	LOC101929147
chr12:121743628-121761738	23	TMEM120B
chr4:5729306-5741814	11	EVC
chr3:189132398-189147647	17	nogene
chr9:96533926-96534542	5	CDC14B
chr6:42934128-42934881	10	nogene
chr10:68430435-68430660	29	DNA2
chr18:37084124-37173081	15	KIAA1328
chr21:46279992-46280575	48	MCM3AP
chr7:2244562-2245277	32	NUDT1
chr13:49451552-49467960	9	SETDB2
chr19:37770004-37773751	16	ZNF573
chr22:24302194-24313466	186	SPECC1L-ADORA2A
chr5:65242103-65300131	49	ADAMTS6
chr2:97146485-97154741	15	ANKRD36
chr14:57244164-57247712	32	EXOC5
chr16:87415275-87418901	16	ZCCHC14

chr19:6382553-6383011	13	nogene
chr8:6724869-6747828	256	AGPAT5
chr2:108791674-108805008	21	CCDC138
chr6:44403808-44408632	73	CDC5L
chr16:53254437-53274302	52	CHD9
chr9:67188784-67201219	11	CNTNAP3P2
chr11:68796855-68812576	4	CPT1A
chr19:3118923-3119359	20	GNA11
chr1:41628748-41628947	10	HIVEP3
chr11:77955941-77961138	84	INTS4
chr6:36465600-36470257	11	KCTD20
chr7:21020867-21033558	10	LINC01162
chr4:169576927-169590809	19	NEK1
chr19:3449013-3452666	16	NFIC
chr5:41794002-41801070	19	OXCT1
chr4:120903260-120907557	11	PRDM5
chr14:73198030-73211942	14	PSEN1
chr8:140864311-140890769	1568	PTK2
chr8:140761164-140890769	7	PTK2
chr12:113304320-113307844	9	SLC8B1
chr19:11059752-11060187	11	SMARCA4
chr11:68189916-68190152	28	SUV420H1
chr7:27748517-27769834	62	TAX1BP1
chr1:154241512-154246375	38	UBAP2L
chr12:27046157-27066226	9	nogene
chr5:73074741-73082825	5	FCHO2
chr2:69348170-69350183	14	GFPT1
chr2:203402575-203427269	96	ABI2
chr1:110009033-110014857	4	AHCYL1
chr11:76451857-76460085	3	C11orf30
chr3:119905754-119923483	55	GSK3B
chr8:30700080-30708141	11	GSR
chr5:178055663-178055981	23	nogene
chr2:24823955-24824639	46	nogene
chr2:65246523-65265175	112	ACTR2
chr7:50703820-50710990	16	GRB10
chr2:74874265-74886393	9	HK2
chr14:59487673-59498908	14	JKAMP
chr21:46275185-46280575	15	MCM3AP
chr8:103910133-103961133	3	RIMS2
chr10:124484138-124488575	10	LHPP
chr5:139121034-139128052	8	SIL1
chr10:124942456-124943307	156	ZRANB1

chr1:66940027-66963160	73	MIER1
chr4:173384650-173403950	4	nogene
chr11:85974707-86031611	39	PICALM
chr7:80789305-80828745	23	SEMA3C
chr7:77360823-77375101	2	GSAP
chr8:47465024-47465483	5	nogene
chr12:102148229-102165883	42	PARPBP
chr17:39989738-39990197	3	PSMD3
chr7:105481051-105495253	51	PUS7
chr12:108817037-108818558	24	SSH1
chr12:120713273-120717042	2	UNC119B
chr4:169463242-169479534	23	NEK1
chr7:904125-926644	29	ADAP1
chr5:150006472-150018697	9	HMGXB3
chr12:87527757-87583786	34	nogene
chr5:154790324-154811640	16	LARP1
chr18:62348167-62349937	105	TNFRSF11A
chr10:31820386-31854206	7	ARHGAP12
chr2:105339582-105345389	17	C2orf49
chr12:28305648-28412938	10	CCDC91
chr5:75343852-75345658	13	HMGCR
chr7:23342063-23361741	32	IGF2BP3
chr1:187269395-187329060	38	LINC01036
chr1:58671869-58685251	4	MYSM1
chr17:47590716-47613725	6	NPEPPS
chr3:132681913-132692818	12	NPHP3-ACAD11
chr3:171733443-171738082	11	PLD1
chr12:79845301-79872938	11	PPP1R12A
chr1:150334934-150340477	9	PRPF3
chr9:112262434-112330474	12	PTBP3
chr5:69391830-69393500	26	RAD17
chr12:116740344-116741094	16	RNFT2
chr16:1962930-1963148	9	RPS2
chrX:18247768-18257030	13	SCML2
chr1:11018732-11020599	6	TARDBP
chr8:140300468-140311374	12	TRAPPC9
chr9:135933056-135933515	19	UBAC1
chr9:111914604-111926496	28	UGCG
chr14:96833466-96837817	39	VRK1
chr16:879569-881505	24	LMF1
chr3:188484591-188524787	14	LPP
chr3:142347233-142376594	6	XRN1
chr10:24629995-24670397	9	ARHGAP21

chr2:176296859-176329426	6	MTX2
chr5:40764513-40777586	63	PRKAA1
chr17:30868332-30869646	10	ATAD5
chr5:113534304-113539181	100	YTHDC2
chr22:17864898-17872023	15	MICAL3
chr10:86443274-86446449	21	WAPL
chr1:176081151-176136547	208	RFWD2
chr22:46891265-46929635	3	TBC1D22A
chr9:69064937-69072830	13	FXN
chr13:45136732-45207505	3	GTF2F2
chr2:96601642-96602862	7	KANSL3
chr2:84850010-84853410	12	nogene
chr5:178885613-178931326	51	nogene
chr13:24484652-24493733	234	PARP4
chr10:1080010-1080476	45	WDR37
chr2:202796889-202829016	11	ICA1L
chr20:50561362-50568478	13	PTPN1
chr14:66681106-66824566	9	GPHN
chr9:110941420-111011690	128	LPAR1
chr14:70748828-70761182	4	MAP3K9
chr1:6531967-6533441	9	NOL9
chr20:47282101-47287284	3	ZMYND8
chr1:52863980-52867777	3	ZYG11A
chr6:125292840-125300659	3	HDDC2
chr3:154187684-154194718	23	ARHGEF26
chrX:17477145-17477320	8	nogene
chr15:70919049-70984257	11	LRRC49
chr15:55348331-55348725	8	nogene
chr8:43023113-43023547	71	nogene
chr21:43734068-43755764	4	PDXK
chr14:99461191-99465813	12	SETD3
chr10:50460672-50466932	17	SGMS1
chr20:62857394-62859526	398	TCFL5
chr19:56146246-56147208	14	ZNF444
chr5:115866669-115902992	23	AP3S1
chr9:26884809-26887513	17	CAAP1
chr19:6362445-6366363	15	CLPP
chr4:5835914-5843161	4	CRMP1
chr8:17064083-17090584	8	MICU3
chr15:41356038-41371684	29	NUSAP1
chr9:109931926-109943381	3	PALM2
chr1:8614560-8656441	12	RERE
chr4:129043394-129082373	12	SCLT1

chr2:39006411-39010703	12	SOS1
chr1:235419416-235434280	4	TBCE
chr12:109101901-109103611	53	UNG
chrX:75450801-75478985	7	ZDHHC15
chr13:20026611-20036909	24	ZMYM2
chr20:47294665-47298947	10	ZMYND8
chr6:38592575-38598121	20	BTBD9
chr1:94458606-94491247	9	ABCD3
chr1:36009474-36027298	5	AGO3
chr8:140275657-140287734	18	TRAPPC9
chrX:96881578-96918617	10	DIAPH2
chr1:171523220-171524965	25	PRRC2C
chr1:70296129-70324957	12	ANKRD13C
chr7:148766560-148767749	34	CUL1
chr1:25553921-25557267	20	LDLRAP1
chr7:67083429-67098718	25	TYW1
chr2:48346200-48359147	12	FOXN2
chr9:94773001-94801002	98	C9orf3
chr12:24878529-24894475	24	BCAT1
chr5:32121494-32126636	10	GOLPH3
chr9:93476255-93516269	19	FAM120A
chr12:70329422-70330469	4	CNOT2
chr4:42001615-42023384	61	SLC30A9
chr1:152332582-152341210	9	FLG-AS1
chr14:50180571-50204409	36	SOS2
chr10:50095147-50110270	4	FAM21A
chr13:30227411-30283687	4	KATNAL1
chr12:14456560-14457295	24	ATF7IP
chr8:102833055-102834747	28	AZIN1
chr1:28027788-28058094	7	EYA3
chr9:710803-713464	9	KANK1
chr13:113309642-113309862	5	LAMP1
chr3:15032378-15034809	19	NR2C2
chr3:29868857-29936196	7	RBMS3
chr2:9237832-9297445	55	ASAP2
chr10:48401611-48410168	184	MAPK8
chr8:103697085-103697296	57	RIMS2
chr9:36581643-36589652	30	MELK
chr1:62618705-62625401	13	DOCK7
chr8:42906172-42974194	4	HOOK3
chr3:50065030-50070552	10	RBM6
chr11:83206057-83236509	52	ANKRD42
chr17:18865468-18911251	60	PRPSAP2

chr5:168488601-168502105	15	RARS
chr6:135300499-135394896	27	AHI1
chr19:57925622-57928047	11	ZNF418
chr11:30330885-30332993	3	ARL14EP
chr11:47750915-47765362	3	FNBP4
chr13:73813151-73846373	3	KLF12
chr4:110418240-110419105	3	nogene
chr19:55098783-55112795	41	PPP1R12C
chr3:45703437-45732151	3	SACM1L
chr10:15248638-15284105	19	FAM171A1
chr7:77292951-77294929	6	CCDC146
chr7:30836657-30840848	13	FAM188B
chr8:56963962-56966352	4	IMPAD1
chr3:101801234-101807126	31	NXPE3
chr19:3638883-3648708	20	PIP5K1C
chr2:247537-253115	4	SH3YL1
chr19:9532844-9535279	34	ZNF426
chr2:61068848-61070500	26	KIAA1841
chr12:22517985-22544179	12	C2CD5
chr18:79695224-79704917	32	CTDP1
chr2:203355153-203367044	13	ABI2
chr11:94795010-94800311	63	AMOTL1
chr10:73387688-73400857	21	ANXA7
chr7:107362032-107412632	13	COG5
chr15:80978930-80982182	15	MESDC2
chr10:97476682-97478389	34	MMS19
chr17:16034764-16040494	24	NCOR1
chr6:111343924-111357125	4	REV3L
chr14:39048651-39067296	13	SEC23A
chr4:143527867-143530526	33	SMARCA5
chr19:9417827-9420218	5	ZNF266
chr4:145123020-145125101	29	ABCE1
chr10:24591886-24595039	2	ARHGAP21
chr3:11355420-11380052	21	ATG7
chr1:235484321-235494828	14	B3GALNT2
chr7:82335134-82403855	10	CACNA2D1
chr17:64519320-64522895	46	CEP95
chr11:125629231-125633351	18	CHEK1
chr4:73064777-73065413	2	COX18
chr4:15007304-15033211	15	CPEB2
chr11:68803999-68807638	132	CPT1A
chr8:67164390-67179926	9	CSPP1
chr5:157285338-157287108	9	CYFIP2

chr1:15638306-15649823	2	DDI2
chr10:110502720-110507154	54	DUSP5
chr20:34526267-34534795	8	DYNLRB1
chr10:73141145-73146361	2	ECD
chr3:55950424-56007321	13	ERC2
chr12:53276619-53277960	3	ESPL1
chr4:53379072-53399839	2	FIP1L1
chr9:20694519-20720534	2	FOCAD
chr4:48590658-48605833	2	FRYL
chr3:197756425-197778464	13	FYTTD1
chr9:125297428-125299106	20	GAPVD1
chr16:4811169-4817697	18	GLYR1
chr10:67990903-68044563	7	HERC4
chr5:133064979-133067557	12	HSPA4
chr1:158084421-158088454	16	KIRREL
chr20:62345817-62347028	19	LAMA5
chr16:20821761-20828537	19	LOC81691
chr9:125506309-125559809	2	MAPKAP1
chr2:96351830-96354388	6	NCAPH
chr2:45697940-45701491	6	nogene
chr4:22727390-22727772	12	nogene
chr3:184363973-184364275	11	nogene
chr14:49853750-49862805	58	nogene
chr9:121057893-121067564	9	nogene
chr7:99113580-99113835	18	nogene
chr1:51819799-51834170	6	NRD1
chr11:3731390-3744649	15	NUP98
chr11:117152440-117161261	19	PAFAH1B2
chr2:205172210-205193320	92	PARD3B
chr11:86011029-86031611	143	PICALM
chr4:56990747-56999781	2	POLR2B
chr22:50372019-50389111	14	PPP6R2
chr8:47799209-47807326	2	PRKDC
chr20:38488402-38493132	33	RALGAPB
chr1:2395783-2403589	29	RER1
chr16:74649131-74652122	13	RFWD3
chrX:84116228-84135210	2	RPS6KA6
chr10:97388251-97388624	16	RRP12
chr2:199348700-199381820	37	SATB2
chr1:41046406-41048890	9	SCMH1
chr19:46776974-46784559	2	SLC1A5
chr2:32114637-32128479	12	SPAST
chr2:168161786-168182090	102	STK39

chr7:99903217-99917885	10	TRIM4
chr14:90676522-90689712	10	TTC7B
chr13:41811224-41868477	7	VWA8
chr16:28166507-28169909	53	XPO6
chr14:54957033-54957635	6	WDHD1
chr10:102001970-102014024	12	C10orf76
chr11:68519500-68537891	110	PPP6R3
chr6:36524340-36540207	45	STK38
chr1:92605306-92704744	9	EVI5
chr11:82849048-82860117	9	PRCP
chr6:158573419-158608750	48	TMEM181
chr5:160010499-160023692	8	TTC1
chr12:110342227-110342448	9	ATP2A2
chr18:79303603-79307234	4	ATP9B
chr2:48451007-48465642	11	PPP1R21
chr3:125313307-125323444	34	ZNF148
chr16:70255727-70268379	7	AARS
chr10:122951953-122953032	4	C10orf88
chr2:113444963-113457204	41	CBWD2
chr1:168043024-168068463	12	DCAF6
chr1:15638306-15651895	50	DDI2
chr22:31403028-31411081	12	DRG1
chr2:148761781-148765146	10	EPC2
chr6:98899267-98934879	17	FBXL4
chr3:49123131-49123373	13	LAMB2
chrX:3826484-3829392	12	LOC389906
chr12:123210055-123223127	10	MPHOSPH9
chr14:105445417-105454310	15	MTA1
chr8:71961930-71973659	15	nogene
chr10:900269-900729	10	nogene
chr13:24484652-24501834	33	PARP4
chr2:200876918-200887685	52	PPIL3
chr3:114065236-114080057	8	QTRTD1
chr3:57847196-57862086	21	SLMAP
chr10:94493364-94500327	42	TBC1D12
chr17:59012327-59017424	25	TRIM37
chr7:98983263-98988966	10	TRRAP
chr7:120806548-120840109	15	TSPAN12
chr15:99156147-99161867	14	TTC23
chr1:21721035-21721764	14	USP48
chr10:120858642-120867169	10	WDR11
chr3:141368620-141413639	10	ZBTB38
chr20:25685566-25686466	24	ZNF337

chr12:53276619-53277227	25	ESPL1
chr12:94400849-94412591	10	CEP83
chr13:98213219-98213413	34	FARP1
chr10:69364782-69369624	14	HK1
chr18:63338799-63362868	10	KDSR
chr11:71916073-71923475	4	LOC100133315
chr22:28514756-28583669	10	nogene
chr7:9708000-9716909	6	nogene
chr17:45475099-45478147	8	PLEKHM1
chr5:69123198-69128132	9	SLC30A5
chr8:140830471-140846678	12	PTK2
chr4:86686709-86701801	14	PTPN13
chr7:74239937-74249118	26	RFC2
chr1:244411286-244437768	3	ADSS
chr17:19651546-19657862	20	ALDH3A2
chr17:18021122-18028659	14	ATPAF2
chr3:63912587-63913225	537	ATXN7
chr11:116757145-116758407	31	BUD13
chr2:36378356-36442735	51	CRIM1
chr8:141151618-141155970	14	DENND3
chr6:52437561-52438591	26	EFHC1
chr10:5709529-5712918	27	FAM208B
chrX:147921932-147932763	3	FMR1
chr3:172251259-172310881	21	FNDC3B
chr9:4286037-4286435	51	GLIS3
chr3:155906159-155911279	14	GMPS
chr9:36246030-36249397	24	GNE
chr10:884446-890829	112	LARP4B
chr10:96907264-96944245	14	LCOR
chr4:41666560-41692384	6	LIMCH1
chr7:156734176-156826744	16	LMBR1
chr11:68357649-68390052	19	LRP5
chr7:1936686-2069338	4	MAD1L1
chr12:111649179-111649771	24	nogene
chr22:40367398-40368678	21	nogene
chr3:132715084-132719830	4	NPHP3-ACAD11
chr3:136260478-136262065	81	PCCB
chr2:172558707-172570824	13	PDK1
chr2:189785345-189805754	18	PMS1
chr7:105472131-105482440	14	PUS7
chr14:73076318-73077536	12	RBM25
chr14:54737023-54751537	23	SAMD4A
chr18:47896520-47896809	13	SMAD2

chr7:105203627-105268869	143	SRPK2
chr3:136417884-136423044	27	STAG1
chr16:89892192-89896083	42	TCF25
chr6:42268153-42269848	14	TRERF1
chr1:114463103-114463556	41	TRIM33
chr19:34433347-34467014	12	UBA2
chr2:106129673-106166083	16	UXS1
chr8:99170038-99193057	24	VPS13B
chr10:86460398-86461287	6	WAPL
chr11:74906238-74907273	10	XRRA1
chr2:32290482-32292348	3	YIPF4
chr19:2084506-2084641	34	nogene
chr1:225530553-225567414	7	ENAH
chr15:67664596-67748601	13	MAP2K5
chr20:31782248-31797515	35	TPX2
chr8:42350006-42361365	39	POLB
chr5:618989-655584	28	CEP72
chr9:93470328-93476338	51	FAM120A
chr11:45862059-45872233	29	CRY2
chr10:68913161-68919981	13	DDX50
chr5:112259890-112307490	4	EPB41L4A
chr10:74267564-74398579	13	ADK
chr10:74242737-74398579	64	ADK
chr10:12088985-12094271	13	DHTKD1
chr3:138621406-138629156	11	FAIM
chr12:95134738-95141539	3	FGD6
chr6:17771907-17779099	39	KIF13A
chr4:102714437-102730736	12	MANBA
chr14:54566198-54567846	8	nogene
chr17:47613668-47619164	4	NPEPPS
chr1:229458160-229463676	9	NUP133
chr6:143470960-143474856	63	PEX3
chr2:169606038-169614724	41	PPIG
chr16:89544647-89548113	7	SPG7
chr4:98105952-98109305	9	STPG2
chr14:39154210-39159550	24	TRAPPC6B
chr17:4112608-4114470	4	ZZEF1
chr13:26324771-26353880	63	CDK8
chr2:197459931-197462731	24	COQ10B
chr7:128856921-128857096	12	FLNC
chr2:154140336-154259138	3	GALNT13
chr7:50703820-50705323	31	GRB10
chr6:77214625-77273307	27	nogene

chr4:48851805-48857365	3	OCIAD1
chr1:93156299-93160226	7	TMED5
chr5:73857655-73858219	25	ARHGEF28
chr11:66521269-66526807	6	BBS1
chr1:114719578-114720717	99	CSDE1
chr9:113198854-113211592	11	FKBP15
chr1:240121572-240123345	78	FMN2
chr14:31168297-31169468	40	HECTD1
chrX:70341798-70365889	10	KIF4A
chr7:131387119-131429145	14	MKLN1
chr3:15016151-15030452	19	NR2C2
chr7:44674410-44675268	159	OGDH
chrX:41183997-41186642	10	USP9X
chr9:111583471-111597432	9	PTGR1
chr8:140861223-140890769	20	PTK2
chr20:49023050-49032166	2	ARFGEF2
chr15:100609706-100630042	14	ASB7
chr3:11298685-11309061	5	ATG7
chr3:107710451-107716849	219	BBX
chr1:222724091-222725555	14	BROX
chr2:61876094-61879011	16	CCT4
chr1:227026054-227035607	13	CDC42BPA
chr14:80831142-80840768	27	CEP128
chr12:6587383-6588422	41	CHD4
chr7:6430479-6432959	5	DAGLB
chr14:53058476-53093444	2	DDHD1
chr9:137834348-137835088	25	EHMT1
chr14:57246710-57247712	26	EXOC5
chr18:13666478-13682105	92	FAM210A
chr17:6447462-6447758	53	FAM64A
chr10:121538591-121565704	6	FGFR2
chr9:20715321-20781929	17	FOCAD
chr9:83290602-83299186	13	FRMD3
chr2:69337897-69345999	30	GFPT1
chr17:36554819-36567776	140	GGNBP2
chr1:235341707-235342883	20	GGPS1
chr4:2984512-3029409	14	GRK4
chr2:238188130-238189972	18	ILKAP
chr8:28835291-28846809	2	INTS9
chr2:9412268-9418732	16	ITGB1BP1
chr3:124796387-124809156	5	ITGB5
chr10:89718709-89726521	11	KIF20B
chrX:102839800-102905737	24	LINC00630

chr4:150870524-150897818	11	LRBA
chr1:117460488-117493262	15	MAN1A2
chr2:31891991-31956598	3	MEMO1
chr17:42540949-42541206	3	NAGLU
chr5:150520867-150521767	204	NDST1
chr19:3433517-3435207	3	NFIC
chr1:30948145-30948506	13	nogene
chr5:85805306-85827796	10	nogene
chr3:196019879-196027260	12	nogene
chr3:168057080-168057613	10	nogene
chr19:17934761-17935924	9	nogene
chr21:29372434-29411624	2	nogene
chr19:11114013-11114607	19	nogene
chr16:27233000-27235299	23	NSMCE1
chrX:68197185-68206673	4	OPHN1
chr10:117318871-117341102	50	PDZD8
chr14:91481575-91485730	116	PPP4R3A
chr9:112252677-112297916	26	PTBP3
chr9:109420423-109438234	10	PTPN3
chr2:1639005-1639301	11	PXDN
chr2:108740488-108753563	9	RANBP2
chr5:168493893-168516950	12	RARS
chr15:64748418-64751638	28	RBPMS2
chr6:3076763-3081116	19	RIPK1
chr1:41070594-41075451	675	SCMH1
chr12:100282942-100298175	514	SCYL2
chr14:39093186-39096139	10	SEC23A
chrX:119629317-119629508	6	6-Sep
chr6:111172694-111219042	5	SLC16A10
chr4:102304316-102315742	16	SLC39A8
chr11:4055525-4074679	17	STIM1
chr17:82870223-82900731	42	TBCD
chr6:125993756-126021280	15	TRMT11
chr9:33948373-33963791	32	UBAP2
chr10:11967340-12014184	30	UPF2
chr7:6153755-6155195	11	USP42
chr4:1900625-1942565	18	WHSC1
chr9:128740771-128742745	39	ZER1
chr17:32369294-32373453	4	ZNF207
chr18:76937155-76960855	8	ZNF236
chr22:20400631-20401372	40	ZNF74
chr6:135346674-135394896	19	AHI1
chr1:167966628-167993440	16	DCAF6

chr16:89758576-89762022	10	FANCA
chr2:85043563-85049648	50	KCMF1
chr15:41810882-41813101	52	MAPKBP1
chr12:77966228-78007445	10	NAV3
chr11:14771936-14804050	9	PDE3B
chr18:42027442-42040741	7	PIK3C3
chr8:140700890-140746860	10	PTK2
chr4:122979231-123090705	19	SPATA5
chr10:94441895-94444539	14	TBC1D12
chr8:80051526-80064593	55	TPD52
chr5:32414968-32417792	52	ZFR
chr18:6093354-6138296	5	L3MBTL4
chr1:201997318-202000015	6	RNPEP
chr7:2200261-2219456	14	MAD1L1
chr19:36449398-36450001	72	ZNF566
chr15:51896418-51902036	11	TMOD3
chr15:50465046-50471795	51	USP8
chr9:86343074-86346480	18	ZCCHC6
chr17:46050532-46169605	16	KANSL1
chr17:47169916-47172064	46	CDC27
chr6:20739518-20781265	11	CDKAL1
chr8:144309454-144314054	3	HSF1
chr17:18865468-18865952	62	PRPSAP2
chr1:31423692-31426823	3	SERINC2
chr7:5370374-5371364	32	TNRC18
chr2:216141185-216148276	17	XRCC5
chr8:123436452-123441497	8	WDYHV1
chr5:77046346-77048272	77	AGGF1
chr21:43980988-43982619	15	AGPAT3
chr17:19931804-19963022	12	AKAP10
chr12:48939034-48941188	19	ARF3
chr6:100715461-100767345	19	ASCC3
chr2:214767481-214797117	137	BARD1
chr1:19356634-19357563	19	CAPZB
chr11:46816197-46821268	25	CKAP5
chr17:7939512-7940242	26	CNTROB
chr12:31451939-31499671	18	DENND5B
chr14:99850852-99865646	56	EML1
chr5:138510564-138512954	65	ETF1
chr5:137942874-137943216	231	FAM13B
chr7:66479008-66588580	28	GS1-124K5.11
chr1:100049908-100068586	11	HIAT1
chr5:138568924-138571141	189	HSPA9

chrX:46462747-46463308	4	KRBOX4
chr6:167870382-167902386	14	MLLT4
chr16:68190767-68191775	39	NFATC3
chrX:91414903-91453186	208	nogene
chr9:133976432-133978130	13	nogene
chr1:151262160-151267339	6	PSMD4
chr7:29936555-29969068	5	SCRN1
chr1:179335505-179343635	27	SOAT1
chr6:158573419-158631389	15	TMEM181
chr16:24776932-24778468	15	TNRC6A
chr16:24750725-24758599	76	TNRC6A
chr5:113532878-113535798	64	YTHDC2
chr1:200807375-200832845	12	CAMSAP2
chr12:70335437-70342307	3	CNOT2
chr3:97843194-97864647	15	CRYBG3
chr16:56935234-56942237	9	HERPUD1
chrX:107840669-107905626	10	MID2
chr3:52737585-52741499	225	NEK4
chr3:196810589-196818156	13	PAK2
chr17:78414951-78424192	5	PGS1
chr17:28921687-28924302	55	PHF12
chr12:10847044-10883096	15	PRH1-PRR4
chr1:32944018-32949774	4	RNF19B
chr16:70255727-70261157	3	AARS
chr21:45120793-45128573	3	ADARB1
chr11:94850113-94859715	15	AMOTL1
chr8:61679276-61684188	12	ASPH
chr18:79126266-79214038	37	ATP9B
chr15:55432496-55450367	11	DYX1C1-CCPG1
chr8:29009071-29018913	3	HMBOX1
chr1:20744743-20749882	15	HP1BP3
chr8:70637814-70641050	18	LACTB2
chr13:32517856-32522270	9	N4BP2L2
chr17:16079963-16101805	13	NCOR1
chr11:64371521-64371829	10	nogene
chr7:44645326-44666851	58	OGDH
chr5:168559031-168568998	39	PANK3
chr2:172558707-172565073	13	PDK1
chr19:5941620-5957973	26	RANBP3
chr10:68487143-68493570	233	SLC25A16
chr1:48451551-48453131	6	SPATA6
chr17:76067216-76070444	15	SRP68
chr1:25340473-25352974	4	TMEM50A

chr14:100468020-100484124	11	WDR25
chr2:68131276-68137433	3	WDR92
chr12:76797433-76822531	12	ZDHHC17
chr10:27208245-27208445	4	ACBD5
chr1:246876036-246888517	23	AHCTF1
chr12:28305648-28362515	6	CCDC91
chr6:47533601-47554766	140	CD2AP
chr4:139043073-139045389	67	NOCT
chr3:47395086-47396217	15	PTPN23
chr1:30964673-30968492	10	PUM1
chr9:122853951-122855878	7	RC3H2
chr5:154026398-154029580	6	FAM114A2
chr1:77803269-77815231	14	FAM73A
chr4:105691975-105695668	29	INTS12
chr6:57207627-57210445	256	RAB23
chr1:193203794-193212477	20	CDC73
chr5:43298008-43298975	7	HMGCS1
chr2:61115978-61118116	101	KIAA1841
chr3:197884861-197885357	42	nogene
chr14:73147794-73148094	136	PSEN1
chr3:125489407-125504744	9	SNX4
chr3:196368027-196369511	14	UBXN7
chr3:179701007-179730709	10	USP13
chr20:50902016-50918781	11	ADNP
chr6:35619095-35646366	27	FKBP5
chr5:1032151-1036384	27	NKD2
chr19:34450264-34464131	58	UBA2
chr8:43059091-43064215	303	FNTA
chr5:170919440-171028989	11	RANBP17
chr17:82756164-82870380	11	TBCD
chr2:63378385-63439871	24	WDPCP
chr2:111802427-111805893	11	ANAPC1
chr13:100301459-100340259	35	PCCA
chr2:229768615-229771632	12	TRIP12
chr13:111217678-111244294	18	ARHGEF7
chr4:98379016-98404602	4	RAP1GDS1
chr8:141253988-141254629	1357	SLC45A4
chr4:107682014-107687177	11	PAPSS1
chrX:55729293-55757331	3	RRAGB
chr2:54982514-54987698	1608	RTN4
chr12:49460769-49484669	8	SPATS2
chr5:14280321-14304592	16	TRIO
chr4:147946616-148023413	4	ARHGAP10

chr4:56017647-56019555	34	CEP135
chr2:227893794-227905035	37	DAW1
chr13:32194142-32202527	4	FRY
chr19:45065514-45066153	47	nogene
chr3:128060101-128060661	37	SEC61A1
chr2:147896300-147899567	105	ACVR2A
chr2:32509737-32518946	8	BIRC6
chr10:131973033-131973943	10	BNIP3
chr17:67891843-67894165	62	BPTF
chr16:57521067-57526127	21	CCDC102A
chr22:17143097-17149745	27	CECR5
chr4:56017647-56024614	9	CEP135
chr18:79695224-79697988	70	CTDP1
chr4:168236251-168237795	15	DDX60
chr12:31433154-31447769	37	DENND5B
chr5:181237608-181238239	11	GNB2L1
chr10:67932596-67955130	4	HERC4
chr7:78343777-78346043	28	MAGI2
chrX:40659226-40659607	11	MED14
chr21:36338773-36344707	9	MORC3
chr13:41252644-41260875	9	MTRF1
chr10:68174065-68175461	53	MYPN
chr2:206127796-206142069	24	NDUFS1
chr10:73068845-73074915	36	P4HA1
chr2:70259359-70277874	8	PCYOX1
chr17:1521578-1558559	3	PITPNA
chr16:18834891-18845651	14	SMG1
chr17:63713405-63728413	3	STRADA
chr20:13528432-13610919	13	TASP1
chr7:5394041-5394595	6	TNRC18
chr21:32378444-32384464	59	URB1
chr14:100353686-100361921	11	WARS
chr12:93476953-93487660	16	MRPL42
chr5:115130591-115141374	15	TRIM36
chr14:22947170-22952683	9	HAUS4
chr20:35672754-35681981	5	NFS1
chr3:94084106-94095154	4	NSUN3
chr20:35713018-35714389	4	RBM39
chr4:4457190-4471706	61	STX18
chr20:36829053-36839441	90	SOGA1
chr4:145845955-145892415	4	ZNF827
chr3:50068689-50070552	17	RBM6
chr19:1149368-1154402	16	SBNO2

chr20:33392141-33397084	13	CDK5RAP1
chr11:20407910-20464546	21	PRMT3
chr6:89627582-89628341	13	ANKRD6
chr16:4700969-4724831	13	ANKS3
chr14:32090501-32117287	9	ARHGAP5
chr15:72563393-72572165	49	ARIH1
chr17:3814321-3816270	18	C17orf85
chr12:122327946-122328426	4	CLIP1
chr3:182940358-182965753	8	DCUN1D1
chr9:123557569-123652123	28	DENND1A
chr22:31876156-31879752	10	DEPDC5
chr18:36141136-36160015	12	ELP2
chr1:50596120-50744775	4	FAF1
chr2:144141881-144211579	10	GTDC1
chr4:128176871-128179512	9	LARP1B
chr5:96979137-96986670	12	LNPEP
chr5:72283504-72314158	13	MRPS27
chr15:72032493-72046634	25	MYO9A
chr12:77940318-77998476	33	NAV3
chr12:6837086-6837401	11	P3H3
chr2:205300529-205301701	35	PARD3B
chr19:3653289-3667353	10	PIP5K1C
chr1:212329134-212333598	16	PPP2R5A
chr9:107318751-107325004	7	RAD23B
chr6:138907494-138917627	9	REPS1
chr5:159181959-159203656	12	RNF145
chrX:18256847-18265802	9	SCML2
chr2:173918592-173956232	18	SP3
chr7:27796115-27800090	10	TAX1BP1
chr14:88852970-88861332	14	TTC8
chr13:99238426-99244624	154	UBAC2
chr11:103311877-103358359	16	DYNC2H1
chr11:86250295-86257596	32	EED
chr16:27213584-27215989	3	KDM8
chr4:107682014-107701285	536	PAPSS1
chr11:78106806-78109581	4	ALG8
chr11:977094-988689	11	AP2A2
chr1:214629045-214638001	12	CENPF
chr17:37625894-37628672	10	DDX52
chr15:65512294-65515791	13	DPP8
chr4:143861141-143979952	119	LOC101927636
chrX:19395080-19398359	2	MAP3K15
chr6:90556499-90571807	16	MAP3K7

chr19:45271471-45271708	5	MARK4
chr10:5699524-5699737	2	nogene
chr10:49832802-49865381	52	PARG
chr9:131430089-131459356	17	PRRC2B
chr17:5361207-5368468	13	RABEP1
chr1:174272413-174278779	11	RABGAP1L
chr1:2395783-2400935	15	RER1
chr5:40827699-40834606	15	RPL37
chr8:119756005-119791459	7	TAF2
chr22:20052464-20056013	2	TANGO2
chr18:662145-669173	17	TYMS
chr9:35236468-35243364	62	UNC13B
chr16:88598177-88611536	12	ZC3H18
chr6:38116571-38142767	2	ZFAND3
chr14:102338815-102339223	15	ZNF839
chr20:33379460-33379691	12	CDK5RAP1
chr6:1959866-1960966	41	GMDS
chr1:173756975-173775842	14	KLHL20
chr15:72019038-72046634	14	MYO9A
chr9:121182929-121193419	16	RAB14
chr1:24531217-24533254	23	RCAN3
chr17:81270917-81272627	17	SLC38A10
chr5:81976192-81987678	4	ATG10
chr10:68430435-68432510	5	DNA2
chr11:47536527-47537526	4	nogene
chr10:73692042-73713332	12	AGAP5
chr20:34519419-34534795	12	DYNLRB1
chr2:69328270-69345999	13	GFPT1
chr2:177440964-177445626	3	AGPS
chr5:37243012-37247745	40	C5orf42
chr7:77274481-77280653	21	CCDC146
chr15:42559338-42563857	13	HAUS2
chr6:25482256-25495215	3	LRRC16A
chr17:5346789-5350629	30	RABEP1
chr1:45457563-45457871	4	TESK2
chr20:31770342-31778984	17	TPX2
chr6:33263483-33264900	3	VPS52
chr21:43613829-43633421	17	HSF2BP
chr6:52516005-52535846	11	TRAM2
chr11:1000431-1003804	29	AP2A2
chr10:115120184-115171292	71	ATRNL1
chrX:101120401-101148161	5	CENPI
chr10:366274-384146	58	DIP2C

chr1:53233541-53235635	17	MAGOH
chr6:87656902-87658018	9	ORC3
chr5:69118498-69128132	11	SLC30A5
chr17:39281388-39285573	7	FBXL20
chr16:70261043-70268379	33	AARS
chr1:167966628-168004793	26	DCAF6
chr1:44874677-44881739	3	EIF2B3
chr6:88890494-88891915	61	RNGTT
chr1:246548544-246557534	3	TFB2M
chr16:56407963-56409588	3	AMFR
chr1:197642710-197674169	33	DENND1B
chr20:50940864-50955285	56	DPM1
chr10:73152292-73160551	16	ECD
chr21:28986138-28986934	20	LTN1
chr2:135862606-135865163	130	MCM6
chr10:110287994-110288793	14	nogene
chr6:154860737-154863898	20	nogene
chr1:88740987-88741288	78	PKN2
chr12:10973654-11047188	35	PRH1
chrX:110020351-110109146	18	TMEM164
chr3:179761111-179765848	3	USP13
chr18:56923921-56939393	91	WDR7
chr14:102325984-102339223	28	ZNF839
chr4:36107564-36121326	4	ARAP2
chr12:31413435-31447769	7	DENND5B
chrX:19392001-19400663	58	MAP3K15
chr8:42344952-42350065	5	POLB
chr3:69043749-69048562	11	TMF1
chr16:46668929-46676692	6	VPS35
chr10:112460523-112464657	30	VTI1A
chr16:89988851-89992828	18	AFG3L1P
chr10:77249564-77251256	15	KCNMA1
chr2:216302512-216302666	17	nogene
chr7:11013746-11022979	8	PHF14
chr7:151675419-151676412	4	PRKAG2
chr7:85121740-85153739	9	SEMA3D
chr2:230466300-230474447	4	SP100
chr17:37048653-37049308	10	nogene
chr18:57731413-57732023	25	nogene
chr3:50778674-51090384	12	DOCK3
chr7:1498706-1499360	12	INTS1
chr6:33406195-33406875	7	KIFC1
chr11:85981744-85990399	87	PICALM

chr15:75409835-75413045	12	SIN3A
chr15:63570432-63574403	21	USP3
chr2:222887829-222909150	15	ACSL3
chr8:39007885-39026810	8	ADAM9
chr21:45118563-45134832	18	ADARB1
chr22:25667738-25674528	13	ADRBK2
chr12:19493799-19518113	11	AEBP2
chr6:135318518-135394896	54	AHI1
chr19:32617588-32625966	39	ANKRD27
chr17:68368547-68370524	10	ARSG
chr8:130159863-130180880	52	ASAP1
chrX:77574249-77595785	11	ATRX
chr1:200807375-200815644	13	CAMSAP2
chr11:34071725-34079765	13	CAPRIN1
chr12:102025973-102061346	15	CCDC53
chr10:32462682-32474023	16	CCDC7
chr3:45110472-45118621	27	CDCP1
chr3:45110472-45112445	33	CDCP1
chr22:46408995-46410561	4	CELSR1
chr5:123416009-123418515	5	CEP120
chr4:169630164-169636088	3	CLCN3
chr16:3504719-3512478	38	CLUAP1
chr12:108701123-108701323	56	CORO1C
chr9:108990725-108999256	11	CTNNAL1
chr11:108094390-108095691	43	CUL5
chr10:100243697-100253539	5	CWF19L1
chr14:69062383-69075411	3	DCAF5
chr1:114587386-114595873	14	DENND2C
chr9:19286768-19300331	15	DENND4C
chr10:12100177-12101181	14	DHTKD1
chr1:28208341-28214996	4	DNAJC8
chr19:3981338-3982424	10	EEF2
chr9:137710966-137717182	7	EHMT1
chr2:27366758-27367201	3	EIF2B4
chr3:96987329-96987993	28	EPHA6
chr6:70426438-70428419	12	FAM135A
chr1:179063926-179064496	29	FAM20B
chr2:58221941-58232112	1291	FANCL
chr14:75622078-75641898	11	FLVCR2
chr3:71112535-71299880	9	FOXP1
chr3:180957818-180968254	11	FXR1
chr3:37289234-37296219	9	GOLGA4
chr3:148994141-148996904	16	GYG1

chr8:42906172-42930172	137	HOOK3
chr16:27346466-27352696	16	IL4R
chr8:94851552-94866191	5	INTS8
chr12:26595464-26602706	8	ITPR2
chr14:58482512-58487166	13	KIAA0586
chr10:69005731-69011015	10	KIAA1279
chr8:94495730-94499506	11	KIAA1429
chr6:116716218-116724375	27	KPNA5
chr4:41473004-41494606	8	LIMCH1
chr7:2002064-2014642	10	MAD1L1
chr14:50456515-50464133	14	MAP4K5
chr16:1743334-1748720	5	MAPK8IP3
chr5:94909250-94931991	7	MCTP1
chr6:83315309-83352139	7	ME1
chr13:23798723-23870195	11	MIPEP
chr13:23798723-23809924	41	MIPEP
chr9:20360741-20365744	18	MLLT3
chr2:197535529-197540437	12	MOB4
chr3:179592643-179598771	67	MRPL47
chr2:190659157-190670459	74	NAB1
chr7:140702865-140704963	188	NDUFB2
chr3:27256295-27293652	9	NEK10
chr12:103766734-103775630	27	nogene
chr1:156743256-156743444	21	nogene
chr17:45140564-45140752	15	nogene
chr1:155243879-155244335	33	nogene
chr7:6428268-6428596	16	nogene
chr6:42941509-42943970	18	nogene
chr11:29044444-29063536	19	nogene
chr2:10602775-10607311	18	NOL10
chr13:24490667-24503777	35	PARP4
chr8:51857492-51861246	12	PCMTD1
chr1:233236844-233252788	18	PCNXL2
chr16:71663898-71679535	4	PHLPP2
chr2:208333314-208348023	3	PIKFYVE
chr7:30045084-30049382	13	PLEKHA8
chr6:43587271-43587652	7	POLH
chr10:70204872-70218817	7	PPA1
chr9:112250928-112268195	14	PTBP3
chr8:140879470-140925749	124	PTK2
chr15:75523120-75527261	46	PTPN9
chr6:163455278-163478896	4	QKI
chr16:461403-489000	4	RAB11FIP3

chr1:174275832-174305127	8	RABGAP1L
chr5:132559283-132580066	5	RAD50
chr19:11415936-11417059	105	RGL3
chr8:53946664-53954310	10	RGS20
chr7:5711760-5739352	10	RNF216
chrX:84145477-84164387	15	RPS6KA6
chr10:68394074-68394451	81	RUFY2
chr11:9812531-9829496	3	SBF2
chr19:2765185-2769091	13	SGTA
chr19:430683-440932	86	SHC2
chr4:1694773-1703700	14	SLBP
chr13:29517330-29517572	13	SLC7A1
chr2:230357804-230361697	188	SP140L
chr3:136443286-136502779	13	STAG1
chr19:19316617-19320462	3	SUGP1
chr12:88160113-88176319	19	TMTC3
chr8:9580158-9580383	21	TNKS
chr3:133616813-133620347	9	TOPBP1
chr15:43441525-43470066	4	TP53BP1
chr19:13112704-13113011	3	TRMT1
chrX:101018970-101020588	53	TRMT2B
chr12:104309758-104327671	10	TXNRD1
chr1:162566011-162576981	9	UAP1
chr2:180982017-180984093	51	UBE2E3
chr15:43047160-43048491	16	UBR1
chr17:60181323-60185651	30	USP32
chr2:218558497-218562813	21	USP37
chr17:31865365-31875413	6	UTP6
chr6:144700086-144757989	9	UTRN
chr5:133990854-133991154	26	VDAC1
chr1:27414832-27428933	5	WASF2
chr2:61493893-61497007	25	XPO1
chr2:61498672-61533903	6	XPO1
chr20:21326278-21328670	39	XRN2
chr7:64814917-64820323	14	ZNF138
chr18:76849525-76851939	718	ZNF236
chr19:37080344-37091121	17	ZNF420
chr19:51880322-51886912	12	ZNF577
chr2:71355718-71380565	27	ZNF638
chr10:124942456-124966781	42	ZRANB1
chr3:47113875-47116754	12	SETD2
chr18:36026899-36033837	468	RPRD1A
chr16:15068168-15073080	25	RRN3

chr3:196371895-196403019	57	UBXN7
chr5:168568645-168568998	107	PANK3
chr3:130996042-130999659	11	ATP2C1
chr7:107281299-107298346	81	COG5
chr5:116303529-116318300	8	nogene
chr6:100798712-100805880	188	ASCC3
chr5:65528451-65542848	36	CENPK
chrX:53557381-53559060	17	HUWE1
chr5:138372673-138381590	18	KDM3B
chr17:47656859-47658660	25	KPNB1
chr6:52267864-52273916	32	MCM3
chr19:19564932-19569584	45	PBX4
chr1:233198938-233227371	21	PCNXL2
chr1:244411286-244417752	7	ADSS
chr2:73534823-73573424	4	ALMS1
chr12:110025741-110029635	10	ANKRD13A
chrX:63674037-63697304	4	ARHGEF9
chr9:91296020-91356155	33	AUH
chrX:154768301-154770879	18	DKC1
chr1:109582436-109586882	15	GNAI3
chr5:133070373-133076898	11	HSPA4
chrX:70341798-70343982	14	KIF4A
chr19:11106564-11110771	3	LDLR
chr10:101800241-101807901	6	MGEA5
chr21:45749380-45755452	4	nogene
chr4:39879727-39890364	19	PDS5A
chr6:128005083-128089992	4	PTPRK
chr1:174304985-174394145	14	RABGAP1L
chr2:203489583-203491319	20	RAPH1
chr10:63610186-63619800	57	REEP3
chr2:32098795-32116212	86	SPAST
chr19:39453355-39453521	4	SUPT5H
chr3:17586352-17623913	8	TBC1D5
chr3:44361364-44367708	3	TCAIM
chr8:15650696-15673836	237	TUSC3
chr2:180982017-181063041	12	UBE2E3
chr6:89335301-89343756	12	UBE2J1
chr10:11929864-11952249	4	UPF2
chr13:41811224-41833531	78	VWA8
chr22:41338312-41339191	69	ZC3H7B
chr11:6941570-6943641	11	ZNF215
chr12:82965023-82966997	12	TMTC2
chr17:81511425-81512058	15	nogene

chr13:23358334-23371165	10	SACS
chr1:236824118-236838599	11	MTR
chrX:91414903-91418871	321	PABPC5-AS1
chr2:203380207-203391143	4	ABI2
chr19:6934986-6937648	5	ADGRE1
chr19:48182454-48182767	13	C19orf68
chr7:65756468-65758317	6	CCT6P1
chr14:24210347-24211829	70	CHMP4A
chr20:49066191-49072697	15	CSE1L
chr3:41224020-41239354	14	CTNNB1
chr15:65696365-65697383	3	DENND4A
chr15:65669778-65670188	7	DENND4A
chr15:65737706-65738875	18	DENND4A
chr22:46308149-46314013	4	GTSE1
chr21:33718796-33735204	95	ITSN1
chr9:6843548-6893232	4	KDM4C
chr17:62680453-62680777	6	MRC2
chr12:78159202-78181045	3	NAV3
chr19:10371558-10372100	9	nogene
chr2:131684708-131688303	13	nogene
chr11:77374327-77379994	99	PAK1
chr16:71714511-71714801	89	PHLPP2
chr1:109411876-109415363	13	PSMA5
chr20:34072065-34080072	14	RALY
chr11:77672041-77693611	11	RSF1
chr6:158146781-158158364	9	SERAC1
chr1:179341027-179348942	4	SOAT1
chr15:25405460-25411971	1138	UBE3A
chr7:157223253-157225539	144	UBE3C
chr1:21701497-21703618	43	USP48
chr1:151178000-151184493	21	VPS72
chr18:56731382-56781656	18	WDR7
chr1:52860730-52867777	17	ZYG11A
chr11:3771747-3773739	9	NUP98
chr14:58290698-58291707	9	PSMA3-AS1
chr2:32441328-32445668	5	BIRC6
chr1:111565307-111566042	6	nogene
chr19:35258944-35262692	3	LSR
chr10:68965376-68967203	8	DDX21
chr2:25607235-25639094	6	DTNB
chr1:21073462-21111383	3	EIF4G3
chr14:31127784-31129448	13	HECTD1
chr5:177280564-177288925	18	NSD1

chr10:127982273-128049666	3	PTPRE
chr14:34567685-34568013	75	SNX6
chr19:34438644-34445121	46	UBA2
chr3:41789660-41835971	14	ULK4
chr11:103280347-103358359	26	DYNC2H1
chr3:194658956-194660133	14	LSG1
chr21:39269898-39274472	24	BRWD1
chr1:42773562-42775522	14	C1orf50
chr10:124405435-124412200	13	OAT
chr1:233090060-233139855	10	PCNXL2
chr17:3720876-3722221	13	nogene
chr12:31701862-31709425	3	AMN1
chr7:92343023-92352642	27	ANKIB1
chr11:76496214-76528466	52	C11orf30
chr6:89846561-89857199	134	CASP8AP2
chr1:15518109-15518395	16	CASP9
chr16:3740398-3751806	11	CREBBP
chr8:141155848-141168525	9	DENND3
chr7:105877180-105912638	3	nogene
chr16:8804766-8813106	12	PMM2
chr6:75634706-75670720	18	SENP6
chr4:42035274-42049479	11	SLC30A9
chr2:213310058-213375119	55	SPAG16
chr12:120782654-120784593	23	SPPL3
chr8:98663929-98707311	3	STK3
chr20:45366874-45410255	3	SYS1
chr10:67888881-67891554	3	SIRT1
chr14:89980539-89993483	11	TDP1
chr18:9927095-9937066	13	VAPA
chr7:32543140-32559464	31	AVL9
chr21:36281482-36292233	11	DOPEY2
chr8:42950387-42968214	7	HOOK3
chr15:55850541-55852543	5	NEDD4
chr3:61885966-61895136	5	nogene
chr17:28348018-28355876	19	POLDIP2
chr4:3422904-3428169	7	RGS12
chr15:42810613-42817097	75	TTBK2
chr5:57246299-57247215	33	GPBP1
chr6:135002733-135042126	14	HBS1L
chr16:50086214-50102435	18	HEATR3
chr3:131132744-131133956	14	NEK11
chr3:196779419-196782833	12	PAK2
chr15:90979157-90980389	29	PRC1

chr5:131455800-131462088	3	RAPGEF6
chr4:39081102-39086727	11	KLHL5
chr11:43327788-43335994	27	API5
chr11:13413529-13445181	247	BTBD10
chr4:113531215-113548729	17	CAMK2D
chr17:40291057-40291668	7	CDC6
chr10:80425865-80427496	13	FAM213A
chr5:134535857-134573762	10	JADE2
chr16:27211145-27215008	15	KDM8
chr7:105040838-105081797	5	KMT2E
chr6:116698703-116705160	256	KPNA5
chr12:50427761-50461347	21	LARP4
chr3:37047518-37051710	29	MLH1
chr5:41794002-41807438	471	OXCT1
chr10:73030270-73053590	10	P4HA1
chr16:482524-496859	34	RAB11FIP3
chr2:17738220-17745951	5	SMC6
chr20:17956932-17961326	131	SNX5
chr11:102186017-102206074	92	YAP1
chr19:9656546-9659467	26	ZNF562
chr5:112985834-113004077	137	DCP2
chr9:125326415-125330218	5	GAPVD1
chr20:5958527-5978017	13	MCM8
chr12:102099105-102112232	10	NUP37
chr4:120777187-120811449	4	PRDM5
chr6:52276267-52279599	4	MCM3
chr12:109938379-109939247	18	GIT2
chr15:75601137-75621056	4	SNUPN
chr14:34807450-34862322	19	BAZ1A
chr18:13008455-13019206	4	CEP192
chr12:42355160-42398994	99	PPHLN1
chr1:10433841-10442441	13	APITD1-CORT
chr11:95812931-95828027	11	CEP57
chr12:101714089-101716812	97	CHPT1
chr16:4454810-4455780	11	DNAJA3
chr18:49257009-49282175	79	DYM
chr10:15833629-15847943	560	FAM188A
chr12:32598496-32611283	190	FGD4
chr9:125295457-125299106	15	GAPVD1
chr7:8022482-8071131	13	GLCCI1
chr8:41598291-41599304	150	GPAT4
chr16:1683508-1685587	4	HN1L
chr14:103405075-103408139	9	MARK3

chr17:62029540-62052705	19	MED13
chr17:28132619-28172618	5	NLK
chr15:39860819-39874509	3	nogene
chr5:145703974-145704295	34	nogene
chr10:34269656-34269899	10	PARD3
chr13:28662409-28668574	9	POMP
chr9:112232098-112252788	14	PTBP3
chr1:41113282-41143112	143	SCMH1
chr14:61037058-61052135	9	SLC38A6
chr1:109342013-109345878	65	SORT1
chr6:42055530-42057513	5	TAF8
chr1:54040755-54043525	32	TMEM59
chr19:34430575-34438766	492	UBA2
chr11:75879878-75888903	6	UVRAG
chr15:29766265-29800702	38	TJP1
chr14:22950313-22955176	6	HAUS4
chr9:112403994-112418958	38	HSDL2
chr16:71915625-71917134	32	IST1
chr11:107308282-107311572	4	nogene
chr1:51772069-51772723	4	OSBPL9
chr3:15212103-15218540	12	CAPN7
chr1:201857095-201858994	15	IPO9
chr16:67865101-67868599	7	NUTF2
chr3:149895472-149921227	72	RNF13
chr3:197085579-197119530	128	DLG1
chr3:108605334-108616657	14	DZIP3
chr20:47276313-47283648	10	ZMYND8
chr16:70366084-70366861	15	DDX19A
chr20:48953555-48969277	12	ARFGEF2
chr10:101889411-101957732	15	C10orf76
chr8:67074242-67076581	43	CSPP1
chr19:1417499-1432690	32	DAZAP1
chr22:40552105-40594735	173	MKL1
chr10:21688490-21727928	12	MLLT10
chr6:97267871-97273062	3	MMS22L
chr12:98604589-98605290	28	nogene
chr15:77283882-77365225	11	nogene
chr14:49663314-49669598	11	POLE2
chr7:80765154-80805758	11	SEMA3C
chr12:46229152-46239893	26	SLC38A1
chr9:33504537-33509336	28	SUGT1P1
chr1:180834873-180863874	17	XPR1
chr8:134565332-134590355	10	ZFAT

chr20:48953555-48976199	7	ARFGEF2
chr8:55941854-55953984	32	LYN
chr17:31939337-31939618	13	nogene
chr2:147972738-147982081	257	ORC4
chr5:94654629-94663908	5	SLF1
chr9:34241184-34242108	12	UBAP1
chr2:9344531-9358889	37	ASAP2
chr1:32386778-32387181	10	BSDC1
chr1:6806982-6825210	141	CAMTA1
chr2:207724799-207742342	58	CCNYL1
chr15:74918794-74929232	3	COX5A
chr22:31814991-31815212	4	DEPDC5
chr19:47716557-47717016	14	EHD2
chr6:167022408-167040339	35	FGFR1OP
chr11:128768117-128772985	6	FLI1
chr1:212863724-212888594	10	FLVCR1
chr16:70468848-70471352	11	FUK
chr2:190900563-190927482	5	GLS
chr19:33101494-33106899	4	GPATCH1
chr14:66681106-66688386	31	GPHN
chr16:11883014-11898035	6	GSPT1
chr11:28211061-28212331	7	METTL15
chr12:27719807-27724186	20	MRPS35
chr13:29242296-29281865	6	MTUS2
chr11:2949226-2979237	5	NAP1L4
chr17:60623520-60633977	209	PPM1D
chr1:43553491-43578920	45	PTPRF
chr11:114443855-114450166	9	REXO2
chr5:38990948-39021136	173	RICTOR
chr17:59910561-59914703	63	RPS6KB1
chr1:246735974-246744125	25	SCCPDH
chr11:107803000-107805515	5	SLC35F2
chr4:26620612-26720125	5	TBC1D19
chr19:51948592-51949235	33	ZNF350-AS1
chr1:229458160-229465519	9	NUP133
chr15:56373440-56412436	13	TEX9
chr2:151416560-151420379	12	RIF1
chr17:35594007-35608387	50	AP2B1
chr1:114463103-114477087	24	TRIM33
chr12:19493799-19493986	12	AEBP2
chr19:46918487-46922356	10	ARHGAP35
chr5:73749836-73776696	5	ARHGEF28
chr8:103040797-103055936	8	ATP6V1C1

chr20:51725818-51729978	100	ATP9A
chr7:105620199-105624267	4	ATXN7L1
chr10:35529972-35553185	11	CCNY
chr10:84425730-84464016	11	CCSER2
chr5:138198198-138202155	10	CDC23
chr7:39997493-40047877	42	CDK13
chr1:41008793-41010254	31	CTPS1
chr22:31845017-31846967	15	DEPDC5
chr17:56848695-56856625	192	DGKE
chr19:10135735-10136287	10	DNMT1
chr8:108203400-108229195	11	EIF3E
chr6:53273219-53276256	70	ELOVL5
chr3:37273535-37302332	38	GOLGA4
chr7:66551519-66576932	7	GS1-124K5.11
chr4:108009758-108028069	4	HADH
chr4:184418166-184419568	8	IRF2
chr3:183338799-183389779	102	MCF2L2
chr4:70950699-70959040	34	MOB1B
chr8:72052538-72062365	15	MSC-AS1
chr19:40209533-40210147	8	nogene
chr7:129258580-129338536	14	nogene
chr19:27493583-27494240	14	nogene
chr3:31639987-31647091	3	nogene
chrX:126243345-126287274	3	nogene
chr2:189791789-189805754	156	PMS1
chr10:102143044-102145090	3	PPRC1
chr1:171512031-171527844	3	PRRC2C
chr1:109409927-109421926	11	PSMA5
chr6:88928984-88941180	54	RNGTT
chr20:38040434-38059520	55	RPRD1B
chr11:4107434-4112062	19	RRM1
chr7:29940681-29955360	10	SCRN1
chr3:52954316-52969258	26	SFMBT1
chr17:29451454-29467216	28	TAOK1
chr16:89895989-89900794	10	TCF25
chr3:129827802-129880562	5	TMCC1
chr7:66352504-66360109	12	TPST1
chr7:98890334-98897866	17	TRRAP
chr14:75863666-75902224	84	TTLL5
chr3:179690240-179730709	4	USP13
chr8:143465716-143475585	13	ZC3H3
chr13:19959957-20006586	3	ZMYM2
chr19:9535187-9536356	15	ZNF426

chr5:78955294-78969192	35	ARSB
chr14:77464597-77468508	33	AHSA1
chr19:41256451-41257629	9	AXL
chr17:81053667-81057967	25	BAIAP2
chr18:12999420-13019206	1067	CEP192
chr1:197652234-197674169	58	DENND1B
chr20:34089515-34098633	8	EIF2S2
chr6:24777221-24781621	14	GMNN
chr19:33113766-33114419	87	GPATCH1
chr17:6620787-6635171	4	KIAA0753
chr2:97671330-97675928	4	LINC01125
chr4:70958873-70975286	10	MOB1B
chr7:156676385-156680574	9	nogene
chr4:143865970-143982026	24	nogene
chrX:1378321-1378812	9	nogene
chr19:43925141-43926500	9	nogene
chr7:129630077-129727365	10	NRF1
chr11:821957-824436	10	PNPLA2
chr3:184301536-184306834	10	PSMD2
chr2:86740926-86766024	6	RMND5A
chr19:47143493-47153040	6	SAE1
chr19:5609994-5613527	14	SAFB2
chr9:105714365-105722621	9	TMEM38B
chr6:42592150-42606651	36	UBR2
chrX:152457249-152468299	24	nogene
chrX:78122834-78123374	17	PGK1
chr3:58256561-58267345	16	ABHD6
chr1:246863923-246867811	63	AHCTF1
chr1:243664771-243843282	15	AKT3
chr20:29091593-29100460	53	FRG1DP
chr4:150798080-150848998	17	LRBA
chr9:42978850-42986862	9	nogene
chr5:37351189-37358151	8	NUP155
chr14:71036064-71052012	9	PCNX
chr17:7499038-7503791	4	POLR2A
chr11:65649560-65650051	10	SIPA1
chr17:51020158-51021365	10	SPAG9
chr12:111513356-111555919	33	ATXN2
chrX:102834627-102865152	18	LINC00630
chr13:45408926-45411480	10	SLC25A30
chrX:24161714-24179770	8	ZFX
chr19:45355664-45357699	55	ERCC2
chr1:77801330-77859056	10	FAM73A

chr16:67685456-67685802	128	GFOD2
chr11:77938650-77961138	31	INTS4
chr2:127763061-127771004	83	WDR33
chr19:36145805-36146312	12	CAPNS1
chr1:181002605-181005463	64	STX6
chr14:64065431-64065650	18	SYNE2
chr8:52668020-52686967	29	RB1CC1
chr1:243293090-243330692	19	SDCCAG8
chr2:235883344-235908906	34	AGAP1
chr1:1478643-1482614	5	ATAD3B
chr14:67340439-67347453	9	ATP6V1D
chr10:118685982-118695230	4	CACUL1
chr1:230659463-230664587	16	COG2
chr9:83780381-83806560	4	GKAP1
chr8:94849461-94859632	13	INTS8
chr19:5047475-5047669	36	KDM4B
chr14:49709158-49723952	7	KLHDC1
chr4:150735257-150897818	94	LRBA
chr1:30721287-30721722	12	MATN1-AS1
chr2:131125752-131140275	16	PLEKHB2
chr3:141260122-141287453	11	PXYLP1
chr14:35625360-35635598	16	RALGAPA1
chr12:56433025-56434231	30	TIMELESS
chr1:230928936-230961303	9	TTC13
chr8:48034865-48049978	10	UBE2V2
chrX:68511848-68522917	50	YIPF6
chr5:801099-821895	12	ZDHHC11
chr20:47276313-47276795	17	ZMYND8
chr1:35892556-35895269	4	AGO1
chr11:120469287-120477526	13	ARHGEF12
chr11:108267170-108267342	57	ATM
chr22:17655661-17696210	9	BCL2L13
chr22:41808874-41810000	143	CCDC134
chr14:24211414-24211829	46	CHMP4A
chr10:14922980-14935564	10	DCLRE1C
chr1:28038838-28058094	9	EYA3
chr4:3182353-3189093	4	HTT
chr2:238185180-238194870	41	ILKAP
chr2:148922136-148942760	12	KIF5C
chr17:64786566-64797409	11	PLEKHM1P
chr3:50105076-50105709	19	RBM5
chr3:149895472-149912083	5	RNF13
chr17:55031167-55047154	90	STXBP4

chr10:94474667-94477829	11	TBC1D12
chr5:160035139-160051183	77	TTC1
chr18:163307-166819	10	USP14
chr21:15762890-15778027	8	USP25
chr11:102186017-102206086	24	YAP1
chr15:90067109-90068595	32	ZNF710
chr11:34970138-34984728	80	PDHX
chr12:56604216-56605327	38	BAZ2A
chr5:614469-624579	21	CEP72
chr1:3865811-3872572	10	DFFB
chr1:224952669-224955098	3	DNAH14
chr5:171845505-171847749	11	nogene
chr8:17064083-17085318	9	MICU3
chr19:37089038-37096115	13	ZNF420
chr7:104183942-104204234	8	ORC5
chr19:36449230-36450001	17	ZNF566
chr1:52854464-52857749	7	ZYG11A
chr2:65239851-65255694	29	ACTR2
chr14:70946914-70962331	8	PCNX
chr17:81275968-81277133	11	SLC38A10
chr15:85664562-85669830	20	AKAP13
chr14:34794748-34795765	10	BAZ1A
chr17:81700466-81701717	9	HGS
chr13:29024456-29034125	29	MTUS2
chr4:103548742-103569402	5	nogene
chr20:320504-322565	4	NRSN2-AS1
chr1:43604189-43604880	12	PTPRF
chr1:154588550-154597267	7	ADAR
chr15:89865494-89889140	9	AP3S2
chr14:45006007-45012075	5	FAM179B
chr1:153663214-153664474	8	ILF2
chr7:86938125-86965024	11	KIAA1324L
chr8:127806368-127807862	9	nogene
chr4:148259977-148436862	3	NR3C2
chr21:15011169-15043574	10	NRIP1
chr7:129005015-129018157	24	TNPO3
chr5:32414968-32420103	13	ZFR
chr10:32469384-32474023	16	CCDC7
chr14:88726540-88746283	33	EML5
chr21:33534578-33539356	77	GART
chr9:539515-540667	13	KANK1
chr2:177505505-177523805	24	AGPS
chr11:72709869-72711499	74	ARAP1

chr20:48972325-48973284	11	ARFGEF2
chr7:2364371-2369682	8	EIF3B
chr22:41530686-41540795	74	POLR3H
chr2:11249660-11288449	18	ROCK2
chr15:76764960-76767088	13	SCAPER
chr4:75967276-75982037	14	SDAD1
chr5:65704278-65713064	3	SGTB
chr2:27677294-27677924	32	SLC4A1AP
chr1:112555867-112616895	3	ST7L
chr17:37437737-37442652	10	TADA2A
chr12:104309758-104311412	15	TXNRD1
chr15:75868950-75883424	11	UBE2Q2
chr3:9797658-9801756	9	ARPC4
chr3:151185329-151193666	24	MED12L
chr1:11090553-11091597	25	EXOSC10
chr5:37024584-37027412	5	NIPBL
chr20:38531166-38532859	62	RALGAPB
chr7:151477332-151491014	26	RHEB
chr17:653290-655953	18	VPS53
chr1:32631826-32634110	99	ZBTB8OS
chr15:59677910-59682514	5	BNIP2
chr12:68824362-68824651	11	MDM2
chr10:5801206-5801463	20	nogene
chr11:119013742-119014300	15	nogene
chr12:28204092-28226199	7	nogene
chr13:24452405-24456478	5	PARP4
chr16:68249289-68252653	11	PLA2G15
chr11:74634582-74636275	5	POLD3
chrX:37406208-37426000	31	PRRG1
chr12:50996816-51010766	5	SLC11A2
chr14:60982507-61030523	5	SLC38A6
chr5:27472382-27477801	4	LINC01021
chr19:9658224-9693820	47	ZNF812
chr6:17539268-17551604	9	CAP2
chr7:886055-891066	9	GET4
chr15:28292887-28299516	36	HERC2
chr13:49719774-49746993	4	KPNA3
chr13:24484652-24498229	30	PARP4
chr2:43428099-43527988	10	THADA
chr5:151452342-151479489	27	SLC36A1
chr19:12658062-12658510	13	MAN2B1
chr2:197535529-197550386	4	MOB4
chr17:42614843-42666484	19	TUBG1

chr3:191380158-191396033	9	CCDC50
chr16:66808520-66810772	11	NAE1
chr17:68039239-68039966	11	nogene
chr2:219714256-219715412	13	nogene
chrX:136847804-136848582	17	nogene
chr17:37440504-37465541	28	TADA2A
chr4:16238770-16323036	16	TAPT1-AS1
chr10:71977690-71978149	5	nogene
chr10:79201583-79216274	17	ZMIZ1
chr17:60679452-60808076	18	BCAS3
chr9:95880868-95882340	11	ERCC6L2
chr18:12535475-12635096	17	SPIRE1
chr19:32643122-32643631	40	ANKRD27
chr22:31462931-31463967	9	EIF4ENIF1
chr3:12379703-12381491	9	PPARG
chr7:129839206-129880971	5	UBE2H
chr12:102025973-102046119	40	CCDC53
chr2:175114689-175121540	3	ATF2
chr9:16435554-16437524	16	BNC2
chr10:68747160-68747566	56	CCAR1
chr17:30792439-30793672	19	CRLF3
chr10:35060873-35071339	301	CUL2
chr17:59573739-59575471	12	DHX40
chr10:127037718-127061776	6	DOCK1
chr3:51089242-51160702	14	DOCK3
chr1:50535368-50596216	62	FAF1
chr1:212863724-212895354	9	FLVCR1
chr14:31127784-31133675	137	HECTD1
chr8:42906172-42943445	218	HOOK3
chr1:200581200-200592240	11	KIF14
chr6:116689319-116705160	100	KPNA5
chr11:68423488-68426187	33	LRP5
chr9:36630298-36651877	4	MELK
chr2:31994209-31994411	3	nogene
chr17:28917284-28921808	25	PHF12
chr7:10990702-11062085	5	PHF14
chr20:35913248-35914197	20	PHF20
chr10:22567850-22609717	10	PIP4K2A
chr1:153626319-153632240	11	S100A13
chrX:119410189-119426354	184	SLC25A43
chr6:85517755-85533800	5	SNX14
chr11:65034846-65035663	5	SNX15
chr8:47284027-47300097	4	SPIDR

chr1:112591524-112616895	17	ST7L
chr3:125277725-125313656	39	ZNF148
chr17:37149863-37181356	11	ACACA
chr20:3540221-3549338	9	ATRN
chr13:59879228-59924870	4	DIAPH3
chr3:9973627-9977446	23	EMC3
chr9:20948270-20953065	8	FOCAD
chr2:241262710-241268541	14	HDLBP
chr4:142260491-142314762	7	INPP4B
chr11:77955941-77981576	68	INTS4
chr1:89712708-89713273	6	LRRC8C
chr9:131430093-131432794	23	PRRC2B
chr15:32643585-32644359	3	SCG5
chr20:17952586-17954223	5	SNX5
chr18:12463350-12464958	70	SPIRE1
chrX:68513326-68522917	3	YIPF6
chr15:72544819-72561549	9	ARIH1
chr11:13413529-13421838	360	BTBD10
chr6:47526627-47554766	70	CD2AP
chr1:23750844-23752518	41	TCEB3
chr16:696772-697419	132	FBXL16
chr15:40383227-40392023	27	KNSTRN
chr9:131508910-131521472	5	POMT1
chrX:23721880-23736016	57	ACOT9
chr16:28829400-28831072	10	ATXN2L
chr14:73736997-73737297	17	ELMSAN1
chr6:5771290-5775316	50	FARS2
chr12:68828770-68836749	41	MDM2
chr10:21713771-21735235	18	MLLT10
chr1:224281122-224303857	11	NVL
chr3:30623198-30650460	20	TGFBR2
chr1:52877681-52892975	106	ZYG11A
chr15:41356038-41365589	36	NUSAP1
chr1:1485015-1520306	17	ATAD3B
chr15:93000511-93014909	36	CHD2
chr9:128522667-128523846	7	GLE1
chr9:6814630-6893232	10	KDM4C
chr6:167870385-167880517	25	MLLT4
chr4:39745751-39777810	10	UBE2K
chr16:70258979-70261157	11	AARS
chr5:115831693-115837764	28	ATG12
chr17:75042180-75042659	13	ATP5H
chr16:56509956-56514680	11	BBS2

chr10:76036007-76047303	11	C10orf11
chr16:72020713-72021311	7	DHODH
chr1:44339044-44352925	17	ERI3
chr5:151786602-151795575	18	G3BP1
chr16:4332214-4333519	250	GLIS2
chr1:153664042-153668557	16	ILF2
chr17:46170854-46177272	3	KANSL1
chr3:44794216-44794426	10	KIF15
chr2:30459551-30476635	10	LCLAT1
chr22:29970975-29979052	75	MTMR3
chr11:134184636-134185526	25	NCAPD3
chr1:32322539-32323027	13	nogene
chr3:15013588-15014174	21	NR2C2
chr18:22975152-22984990	29	RBBP8
chr18:22989220-23001729	10	RBBP8
chr12:51684173-51688849	12	SCN8A
chr5:134674614-134682482	33	SEC24A
chr13:29535818-29553860	27	SLC7A1
chr1:151638887-151683445	30	SNX27
chr10:94441895-94451609	12	TBC1D12
chr5:176943334-176970866	12	UIMC1
chr2:63484603-63492940	63	WDPCP
chr12:19473247-19500221	193	AEBP2
chr12:26911177-26917733	4	ASUN
chr10:7796120-7800091	9	ATP5C1
chr7:32544693-32554596	31	AVL9
chr17:43099774-43104956	42	BRCA1
chr2:73239642-73240543	9	CCT7
chr2:29121373-29152828	7	CLIP4
chr11:70405264-70409960	18	CTTN
chr11:107348936-107353736	10	CWF19L2
chr7:770470-780144	21	DNAAF5
chr1:225231072-225266769	5	DNAH14
chr10:110506934-110510596	19	DUSP5
chr1:44319627-44339322	29	ERI3
chr16:1822299-1823037	10	HAGH
chr7:11038759-11062085	5	PHF14
chr4:70762518-70778438	12	RUFY3
chr13:29553760-29554673	13	SLC7A1
chr8:70583163-70587176	4	TRAM1
chr1:19183810-19185286	56	UBR4
chr13:95985238-95996135	34	UGGT2
chr3:49324701-49335596	9	USP4

chr12:62500782-62508480	16	MON2
chr5:179709872-179716294	99	CANX
chr21:36344578-36349408	8	MORC3
chr1:44701178-44707389	15	nogene
chr17:81588943-81600427	26	NPLOC4
chr14:75717860-75720703	37	TTLL5
chr15:59202325-59214720	5	MYO1E
chr5:126568258-126577211	9	ALDH7A1
chr1:224952669-225007544	513	DNAH14
chr8:127982359-127989291	20	PVT1
chr19:34431860-34438766	218	UBA2
chr3:128902552-128904505	4	ACAD9
chr11:129093620-129124894	29	ARHGAP32
chr3:48922747-48980496	13	ARIH2
chr12:110340658-110342448	56	ATP2A2
chr1:222725449-222730177	12	BROX
chr1:108939635-108944057	13	CLCC1
chr7:50755886-50762014	11	GRB10
chr20:16404812-16429982	10	KIF16B
chr9:130186716-130187780	10	nogene
chr5:14219408-14222930	14	nogene
chr18:20991175-20992937	16	ROCK1
chr17:29676819-29695523	9	SSH2
chr17:28358915-28360847	13	TMEM199
chr11:108131906-108135242	29	ACAT1
chr14:103752999-103797518	4	PPP1R13B
chr3:185617479-185624082	15	SENP2
chr14:68921005-68925672	12	ACTN1
chr3:192727180-192745356	19	FGF12
chr12:120151144-120151391	21	GCN1
chr11:68567013-68571104	3	PPP6R3
chr2:9845219-9876018	19	TAF1B
chr22:18126353-18127034	3	TUBA8
chr7:106251069-106254504	10	NAMPT
chr4:78826036-78851056	140	BMP2K
chr12:122216487-122224644	11	DIABLO
chr4:53383772-53391139	35	FIP1L1
chr2:9475986-9488392	8	IAH1
chr4:165228806-165238899	5	KLHL2
chr14:50466582-50476306	34	MAP4K5
chr17:36507162-36507398	16	MYO19
chr8:93922015-94020460	18	PDP1
chr21:44855209-44861271	611	PTTG1IP

chr2:85647929-85648853	4	USP39
chr19:13817215-13819492	4	ZSWIM4
chr15:58621470-58643978	24	ADAM10
chr6:88890494-88941180	59	RNGTT
chrX:71388236-71389665	16	TAF1
chr16:46677314-46679156	13	VPS35
chr2:202464808-202520201	6	BMPR2
chr12:118019071-118020985	13	RFC5
chr5:134782944-134785586	9	DDX46
chr3:120770727-120776664	14	GTF2E1
chr10:58216144-58237814	48	IPMK
chr2:28850327-28861308	57	TRMT61B
chrX:131768548-131794466	13	FIRRE
chr3:47435007-47443091	176	SCAP
chr2:48328560-48362707	23	FOXN2
chr16:16121974-16125911	21	ABCC1
chr18:12363781-12371691	51	AFG3L2
chr8:140580904-140585311	11	AGO2
chr9:85618982-85657643	12	AGTPBP1
chr6:109641517-109662663	11	AK9
chr5:112766325-112767390	40	APC
chr7:51190849-51193589	10	COBL
chr1:155717005-155726019	86	DAP3
chr10:419064-472549	8	DIP2C
chr11:85598656-85626753	20	DLG2
chr12:12519861-12521463	100	DUSP16
chr2:43776781-43794643	9	DYNC2LI1
chr17:19281954-19283714	26	EPN2
chr2:241373083-241373290	13	FARP2
chr4:173224309-173248440	9	GALNT7
chr1:153667554-153668557	51	ILF2
chr4:128121825-128179512	5	LARP1B
chr7:156762133-156796492	3	LMBR1
chr1:39444661-39448763	10	MACF1
chr10:27158548-27159758	9	MASTL
chr12:62543096-62553174	5	MON2
chr12:78127166-78140334	21	NAV3
chr2:183001952-183023916	26	NCKAP1
chr7:128579998-128615970	11	nogene
chr8:124456518-124457437	28	nogene
chr22:17595276-17595631	30	nogene
chr20:62276203-62276962	13	nogene
chr2:10600852-10668723	10	NOL10

chr1:5927648-5933329	17	NPHP4
chr3:38216095-38236961	12	OXSR1
chr14:105348492-105355177	52	PACS2
chr1:113726417-113738799	14	PHTF1
chr10:122392302-122413045	38	PLEKHA1
chr10:11851996-11852215	17	PROSER2
chr6:138943512-138947913	11	REPS1
chr5:78388836-78459362	27	SCAMP1
chr8:47394977-47407961	21	SPIDR
chr17:29676819-29684684	16	SSH2
chr9:81610219-81620557	10	TLE1
chr3:189215291-189238909	17	TPRG1
chr8:140368017-140405698	8	TRAPPC9
chr8:102360070-102391161	6	UBR5
chr5:37437921-37605238	10	WDR70
chrX:75421863-75431520	5	ZDHHC15
chr7:112286872-112296311	7	ZNF277
chr17:4021128-4022828	5	ZZEF1
chr18:12356693-12371691	105	AFG3L2
chr8:53814661-53829533	3	ATP6V1H
chrX:77988241-78003236	19	ATP7A
chr2:61872081-61880394	5	CCT4
chr21:37472683-37480826	4	DYRK1A
chr12:112258495-112264212	11	HECTD4
chr12:109447066-109451813	7	KCTD10
chr22:21788256-21805945	27	MAPK1
chrX:150614588-150649901	10	MTM1
chr2:45677831-45701491	5	nogene
chr15:79411399-79413557	10	nogene
chr5:41739389-41807438	15	OXCT1
chr16:14606483-14627336	18	PARN
chr6:63694580-63700466	49	PHF3
chr11:65649756-65650051	35	SIPA1
chr15:50747494-50749746	28	SPPL2A
chr14:77576766-77579109	67	SPTLC2
chr4:56483138-56484864	4	SRP72
chr9:15210087-15214249	8	TTC39B
chrX:118404337-118407026	9	WDR44
chrX:68518769-68521497	5	YIPF6
chr7:100019230-100024307	10	ZKSCAN1
chr2:159224231-159225779	4	TANC1
chr11:94568729-94577492	19	PIWIL4
chr14:69116365-69122360	275	DCAF5

chr12:1289851-1316187	5	ERC1
chr2:27607844-27608039	65	ZNF512
chr6:170317369-170323259	56	FAM120B
chr11:28058925-28059161	12	KIF18A
chrX:118392631-118395344	15	WDR44
chr4:102689573-102714561	10	MANBA
chr10:419064-486530	8	DIP2C
chr13:32319076-32326613	21	BRCA2
chr1:70296129-70313790	7	ANKRD13C
chr4:73085248-73092300	64	ANKRD17
chr17:60500398-60509375	7	APPBP2
chr1:155360300-155395553	12	ASH1L
chr1:77801330-77843407	55	FAM73A
chr7:77376847-77404615	12	GSAP
chr9:94438400-94441099	11	HIATL1
chr1:32160614-32167296	3	KPNA6
chr2:8882510-8958642	9	MBOAT2
chr16:68121986-68158068	26	NFATC3
chr1:235985378-235993872	12	NID1
chr5:171028871-171034980	10	nogene
chr4:103328124-103437842	10	nogene
chr11:65502289-65504595	8	nogene
chr3:146110284-146124229	37	PLOD2
chr5:40771718-40777586	11	PRKAA1
chr3:184305767-184306834	20	PSMD2
chr18:12825809-12859254	24	PTPN2
chr19:33002246-33003000	72	RHPN2
chr7:23505745-23507535	227	TRA2A
chr17:21006895-21021226	9	USP22
chr11:113799145-113799415	20	USP28
chr7:26725427-26739964	9	SKAP2
chr7:105565276-105565648	4	RINT1
chr17:14074322-14077056	49	COX10
chr2:190244886-190252307	43	HIBCH
chr6:3076763-3089657	13	RIPK1
chr17:82127691-82128597	21	CCDC57
chrUn_KI270467v1:2263-2842	39	nogene
chr3:51229511-51237590	15	DOCK3
chrX:134473358-134475364	8	HPRT1
chr20:58343717-58359735	17	RAB22A
chrX:97072940-97141794	13	DIAPH2
chr3:197081050-197105005	5	DLG1
chr8:140347756-140365686	10	nogene

chr2:20307977-20312423	8	PUM2
chr3:19950805-19951061	34	RAB5A
chr17:59880893-59891041	17	TUBD1
chr11:62632564-62639731	9	GANAB
chr8:116747331-116747881	33	nogene
chr16:22308147-22309510	7	POLR3E
chr10:63380317-63414899	76	JMJD1C
chrX:77520787-77523401	25	ATRX
chr19:47718508-47736533	5	EHD2
chr11:22674849-22726433	19	GAS2
chr12:120173652-120174169	6	GCN1
chr3:47062162-47067118	19	SETD2
chr22:25354595-25381578	4	LRP5L
chr19:2271781-2273160	5	OAZ1
chr8:47440322-47440542	65	SPIDR
chr10:100990435-100991054	6	C10orf2
chr1:20893516-20895501	26	EIF4G3
chr5:78508061-78510398	6	LHFPL2
chr22:17145023-17149745	34	CECR5
chr8:47777685-47785317	5	PRKDC
chrX:136876502-136878086	15	RBMX
chr3:71437213-71493555	3	FOXP1
chr2:43586400-43592416	11	THADA
chr2:178339671-178374027	5	OSBPL6
chr21:15799756-15811210	11	USP25
chr1:184706642-184711877	16	EDEM3
chr14:45120886-45130800	7	FKBP3
chr2:10396834-10423088	4	HPCAL1
chr14:50247040-50303017	6	L2HGDH
chr12:110503805-110506693	11	RAD9B
chr6:10898014-10907684	11	SYCP2L
chr3:125277725-125288228	9	ZNF148
chr2:175092542-175121540	15	ATF2
chr13:77015408-77021861	3	FBXL3
chr5:181239486-181241639	3	GNB2L1
chr7:93303459-93311272	41	VPS50
chr3:37335052-37347296	9	GOLGA4
chr14:103405075-103411765	10	MARK3
chr16:66805776-66810772	9	NAE1
chr17:2292551-2292977	4	SMG6
chr5:77463094-77464809	13	WDR41
chr1:63629424-63654466	12	PGM1
chr5:65260599-65473952	13	ADAMTS6

chr7:102757853-102761411	5	FAM185A
chr10:995928-1007128	13	GTPBP4
chr17:49805362-49811574	16	KAT7
chr1:58714831-58715641	16	nogene
chr8:17589467-17589765	23	PDGFRL
chrX:53411994-53412253	13	SMC1A
chr15:44733249-44733404	13	nogene
chr1:35972023-35973511	33	AGO3
chr19:57790972-57791802	10	nogene
chr1:151423396-151442205	4	POGZ
chr4:38035587-38054338	20	TBC1D1
chr17:82582740-82587272	77	FOXK2
chr8:47969797-47976866	31	MCM4
chr20:62256056-62272259	20	OSBPL2
chr7:22219865-22318037	14	RAPGEF5
chr7:45063917-45069961	9	CCM2
chr10:11481769-11527567	14	USP6NL
chr1:94961316-94963170	7	LOC729970
chr7:131375423-131445903	26	MKLN1
chr14:49832050-49834449	13	NEMF
chr20:2947981-2975241	7	PTPRA
chr1:47287550-47300152	35	STIL
chr6:72880876-72897142	82	nogene
chr17:40289407-40291668	4	CDC6
chr6:96523145-96528632	10	UFL1
chr2:172568740-172596023	68	PDK1
chr3:32703867-32717237	10	CNOT10
chr21:36263545-36270157	12	DOPEY2
chr7:128626294-128631869	6	nogene
chr2:54974694-54987698	16	RTN4
chr5:55343330-55351029	32	SKIV2L2
chrX:123885630-123892773	14	XIAP
chr1:161778243-161802272	57	ATF6
chr4:105719059-105729499	46	GSTCD
chr19:6742966-6743836	13	TRIP10
chr16:68053563-68056424	11	DUS2
chr2:110649233-110661841	18	BUB1
chr16:72368652-72391215	24	LINC01572
chr8:89937025-89946295	42	NBN
chr1:232479626-232493660	4	SIPA1L2
chr2:36396613-36442735	1118	CRIM1
chr9:95101614-95111637	50	FANCC
chr3:10213206-10213548	13	IRAK2

chr13:41761127-41833531	17	VWA8
chr3:196361843-196368155	9	UBXN7
chr3:38221577-38236961	10	OXSR1
chr13:77205255-77212160	9	MYCBP2
chr5:145472609-145473315	9	nogene
chr14:71009633-71023232	9	PCNX
chr17:28917284-28927063	20	PHF12
chr1:25340473-25343073	9	TMEM50A
chr5:618989-620261	300	CEP72
chr17:64571797-64606640	57	SMURF2
chr17:37179259-37193415	37	ACACA
chr10:63264650-63455089	12	JMJD1C
chr15:43814500-43815074	20	MFAP1
chr13:95177706-95188542	18	ABCC4
chr2:127873827-127874272	9	AMMECR1L
chr12:132734384-132735512	71	ANKLE2
chr4:147965023-148023413	10	ARHGAP10
chr5:179853196-179853437	5	C5orf45
chr1:6820180-6825210	43	CAMTA1
chr7:26202970-26206510	3	CBX3
chr20:63926541-63931022	20	DNAJC5
chr19:18537600-18538433	123	FKBP8
chr10:102362474-102363798	27	GBF1
chr11:18281949-18288013	4	HPS5
chr12:122568260-122574380	9	KNTC1
chr12:122582704-122584990	11	KNTC1
chr9:124301935-124313985	123	NEK6
chr12:19283279-19283745	16	PLEKHA5
chr1:166840954-166849526	3	POGK
chr2:55581566-55588956	14	PPP4R3B
chr17:80263573-80273404	38	RNF213
chr21:43686803-43688240	23	RRP1B
chr15:38299372-38324809	55	SPRED1
chr12:111965594-111991889	6	TMEM116
chr2:178591138-178608266	11	TTN-AS1
chr19:21309245-21310627	4	ZNF708
chr1:175986942-175989479	71	RFWD2
chr5:160010499-160049662	4	TTC1
chr12:133083102-133083561	29	ZNF140
chr4:189864592-189895643	12	nogene
chr7:22960255-22984045	37	FAM126A
chr19:38162252-38164906	75	SIPA1L3
chr1:15638306-15646984	5	DDI2

chr4:2657802-2690970	29	FAM193A
chr11:18400836-18403811	19	LDHA
chr3:111945267-111962312	9	PHLDB2
chr8:123359220-123361646	354	ATAD2
chr2:241654545-241655343	5	ATG4B
chr13:102807145-102822159	26	BIVM
chr3:191357087-191361159	86	CCDC50
chr12:32718642-32722633	23	DNM1L
chr10:5903019-5927541	10	FBXO18
chr9:130593642-130595588	8	FUBP3
chr2:238173533-238185287	40	ILKAP
chr13:46723220-46733998	63	LRCH1
chr16:48548006-48562444	18	N4BP1
chr4:87441828-87454455	5	NUDT9
chr5:102948377-102950820	6	PAM
chr8:102212775-102232304	18	RRM2B
chr4:128970377-129003876	6	SCLT1
chr6:157867546-157875176	72	SNX9
chr19:41341882-41342247	50	TGFB1
chr12:56430880-56432524	41	TIMELESS
chr17:15488180-15503232	21	TVP23C
chr20:50096671-50098981	8	UBE2V1
chr6:144531051-144557311	4	UTRN
chr17:29676819-29703062	31	SSH2
chr4:39755656-39777810	8	UBE2K
chr2:28901986-28914208	30	WDR43
chr7:32548846-32552295	9	AVL9
chrX:136241159-136244795	25	MAP7D3
chr3:57657679-57663716	9	DENND6A
chr19:12207331-12209431	9	LOC100289333
chr6:53505391-53509250	14	GCLC
chr7:99434789-99435268	5	PTCD1
chr12:98527885-98547671	5	TMPO
chr10:68722454-68749685	15	CCAR1
chr7:131443480-131445903	10	MKLN1
chr14:77673559-77675794	10	nogene
chr20:62286582-62289330	74	OSBPL2
chr18:9195550-9211784	4	ANKRD12
chr17:42480666-42494473	4	ATP6V0A1
chr3:5195208-5208263	16	EDEM1
chr10:97437190-97441259	4	EXOSC1
chrX:2345070-2345915	39	nogene
chr1:224296500-224303857	10	NVL

chr11:77353535-77359017	14	PAK1
chr10:78002196-78004888	4	POLR3A
chr14:103778744-103798868	16	PPP1R13B
chr1:176027571-176136547	15	RFWD2
chr16:70526586-70530917	70	SF3B3
chr10:17660463-17673078	12	STAM
chr3:183736717-183754365	14	YEATS2
chr1:203828298-203840374	3	ZC3H11A
chr10:121836717-121924329	19	ATE1
chr1:225185290-225192911	9	DNAH14
chr2:222599927-222643005	14	FARSB
chr13:77217839-77243951	31	MYCBP2
chr14:35625360-35627951	10	RALGAPA1
chr15:44651490-44660616	10	SPG11
chr19:4408902-4409759	47	CHAF1A
chr2:224528946-224557856	23	CUL3
chr9:127243078-127313559	4	GARNL3
chr16:47497398-47580358	8	PHKB
chr4:78826036-78833687	58	BMP2K
chr1:215573785-215579999	10	KCTD3
chr20:32366383-32371876	21	ASXL1
chr2:32607454-32611582	38	BIRC6
chr22:49787389-49804360	25	BRD1
chr9:120477349-120491477	3	CDK5RAP2
chr3:138525489-138572932	3	CEP70
chr19:852208-861956	12	CFD
chr1:53313572-53326992	3	LRP8
chr4:39841947-39849652	3	PDS5A
chr17:64821766-64822393	43	PLEKHM1P
chr8:47794289-47798397	3	PRKDC
chr1:27105884-27109777	29	SLC9A1
chr14:34586229-34597645	3	SNX6
chr8:73603725-73688813	3	STAU2
chr2:63404047-63404657	21	WDPCP
chr8:123371669-123380677	26	ATAD2
chr6:161034258-161049979	134	MAP3K4
chr1:85164525-85178272	7	SYDE2
chr19:47409098-47409547	11	MEIS3
chr4:183183402-183193708	35	WWC2
chr18:79143801-79176907	7	ATP9B
chr10:91994534-91997751	24	BTAF1
chr2:36396613-36522313	28	CRIM1
chr8:103430611-103435790	47	DCAF13

chr7:152263015-152273867	14	KMT2C
chr2:63604695-63606028	36	MDH1
chr6:75817500-75822851	9	MYO6
chr5:115221823-115241606	37	PGGT1B
chrX:134377571-134393952	12	PHF6
chr3:146206525-146222092	9	PLSCR4
chr9:70350113-70350471	6	SMC5
chr4:185247293-185310816	38	SNX25
chr12:49486249-49490731	25	SPATS2
chr17:82884144-82906053	12	TBCD
chr17:62052536-62063301	9	MED13
chr3:43299753-43343478	10	SNRK
chr5:69254601-69262304	10	CDK7
chr7:152800530-152801731	32	ACTR3B
chr22:45718412-45740538	33	ATXN10
chrX:130071039-130072417	11	ELF4
chr2:28929578-28947161	13	WDR43
chr14:80504911-80530886	25	CEP128
chr1:156782281-156787174	16	PRCC
chr9:15497596-15498177	13	nogene
chr4:39320382-39323417	17	RFC1
chr17:623532-628231	7	VPS53
chr3:155829687-155842619	36	SLC33A1
chr5:133980728-133993018	14	VDAC1
chr17:4952794-4953845	42	ENO3
chr5:138006989-138021197	144	FAM13B
chr17:47663088-47674782	36	KPNB1
chr6:89753511-89762530	8	MDN1
chr11:65635528-65636307	33	PCNXL3
chr19:34454259-34464131	20	UBA2
chr8:99481598-99521010	11	VPS13B
chr12:71626259-71629037	96	ZFC3H1
chr8:130118160-130126089	40	ASAP1
chr19:10154292-10155056	40	DNMT1
chr1:108914337-108929975	5	GPSM2
chr4:128121825-128122188	14	LARP1B
chr11:3691346-3693375	10	NUP98
chr7:130173631-130175638	10	TMEM209
chr2:201140356-201164084	10	CFLAR
chr1:85350414-85358847	334	DDAH1
chr7:65979385-65980409	14	GUSB
chr18:7032065-7046367	6	LAMA1
chr5:32263112-32276742	8	MTMR12

chr7:99054789-99061837	18	SMURF1
chr4:67720421-67730170	23	UBA6-AS1
chr6:43562246-43567354	91	XPO5
chr9:109087172-109108570	31	TMEM245
chr17:1050049-1100735	42	ABR
chr3:195306510-195308837	19	ACAP2
chr1:244415980-244424387	8	ADSS
chr11:62535944-62536567	163	AHNAK
chr4:73113791-73125312	6	ANKRD17
chr19:32643122-32646615	6	ANKRD27
chr3:43561219-43574864	14	ANO10
chr5:112775628-112801383	37	APC
chr17:60460662-60462061	116	APPBP2
chr14:74692040-74692328	16	AREL1
chr6:156826684-156829421	18	ARID1B
chr12:110326389-110328017	14	ATP2A2
chr10:73719159-73721504	13	BMS1P4
chr7:140794307-140834872	10	BRAF
chr11:66796280-66828364	18	C11orf80
chr13:110683050-110705571	10	CARS2
chr11:83278725-83280260	17	CCDC90B
chr12:28305648-28328191	5	CCDC91
chr6:31148404-31154799	9	CCHCR1
chr7:90726566-90747775	41	CDK14
chr7:90726566-90790652	98	CDK14
chr18:13104983-13117643	10	CEP192
chr16:69150536-69150904	82	CIRH1A
chr6:154422505-154422983	12	CNKSR3
chr20:49076979-49091022	10	CSE1L
chr20:49067189-49070297	14	CSE1L
chr8:67172415-67177726	11	CSPP1
chr3:47840876-47843255	27	DHX30
chr1:209830107-209831043	33	DIEXF
chr3:197161639-197194589	149	DLG1
chr10:77824383-77830873	10	DLG5
chr1:225080378-225097239	4	DNAH14
chr10:99898085-99900066	29	DNMBP
chr15:35410834-35484507	11	DPH6
chr8:94766611-94770571	9	DPY19L4
chr19:3979454-3979807	18	EEF2
chr11:10803029-10806047	25	EIF4G2
chr2:99394266-99394883	4	EIF5B
chr1:16148377-16149047	10	EPHA2

chr12:1408148-1444750	9	ERC1
chr10:92893348-92920050	36	EXOC6
chr2:72718102-72741469	85	EXOC6B
chr10:15212471-15214601	33	FAM171A1
chr4:2657802-2672372	54	FAM193A
chr1:224115713-224134212	14	FBXO28
chr5:108897658-108898132	12	FER
chr6:109715077-109735298	10	FIG4
chr1:240206798-240294883	83	FMN2
chr17:59257121-59267174	24	GDPD1
chr19:3110148-3119359	9	GNA11
chr9:77922160-77931856	38	GNAQ
chr1:1825396-1839238	17	GNB1
chr19:33093358-33094269	38	GPATCH1
chr9:37424844-37432138	14	GRHPR
chr4:108009758-108014588	12	HADH
chr6:82231717-82240843	20	IBTK
chr5:5436417-5454638	6	ICE1
chr8:42325969-42329214	106	IKBKB
chr16:19730429-19735450	6	IQCK
chr20:34440154-34449480	18	ITCH
chr11:67215339-67231960	6	KDM2A
chr9:111433232-111444494	30	KIAA0368
chr16:27749583-27750306	28	KIAA0556
chr18:36946248-36959435	33	KIAA1328
chr7:138908990-138912460	15	KIAA1549
chr5:109817272-109866994	10	MAN2A1
chr15:67646230-67664645	35	MAP2K5
chr10:92333469-92340803	12	5-Mar
chr6:73428708-73440445	4	MB21D1
chr1:84937754-84952530	20	MCOLN2
chr17:39423326-39424738	21	MED1
chr15:99598429-99602109	5	MEF2A
chr7:131373709-131399433	27	MKLN1
chr6:151009818-151037117	89	MTHFD1L
chr1:11124497-11133197	4	MTOR
chr15:72007825-72032588	11	MYO9A
chr13:41316845-41320824	8	NAA16
chr12:112078187-112087801	9	NAA25
chr16:66805776-66806026	32	NAE1
chr11:2969802-2979237	184	NAP1L4
chr20:36680815-36688784	16	NDRG3
chr6:41079028-41084192	23	NFYA

chr1:9972017-9975775	4	NMNAT1
chr17:35468136-35468822	25	nogene
chr7:66847121-66848666	18	nogene
chr6:83809633-83841697	7	nogene
chr8:144174844-144175564	7	nogene
chr6:126521536-126571600	4	nogene
chr1:222827427-222835215	10	nogene
chr7:23509726-23510355	10	nogene
chr1:63478764-63494287	13	nogene
chr4:13336892-13361633	6	nogene
chr9:2720343-2782846	20	nogene
chr3:67041864-67042057	4	nogene
chr16:89515789-89516473	10	nogene
chr7:32722722-32726470	19	nogene
chr5:10681378-10682305	14	nogene
chr6:52665273-52666878	32	nogene
chr14:73282358-73297285	14	NUMB
chr5:14673641-14681624	27	OTULIN
chr3:38221577-38230430	78	OXSR1
chr17:2676504-2680320	5	PAFAH1B1
chr10:49832802-49869555	91	PARG
chr13:24441845-24447186	51	PARP4
chr1:233025145-233057290	16	PCNXL2
chr6:42968289-42969987	7	PEX6
chr3:48543579-48550234	8	PFKFB4
chr3:138663905-138699095	14	PIK3CB
chr12:11047019-11129363	16	PRH1
chr8:140746759-140761262	10	PTK2
chr8:140752231-140764290	13	PTK2
chr7:155678607-155680908	63	RBM33
chr9:4823530-4827033	18	RCL1
chr7:151477332-151498180	14	RHEB
chr15:41438320-41457876	9	RTF1
chr15:41452900-41457876	4	RTF1
chr2:199308759-199328910	4	SATB2
chr1:243304712-243344331	61	SDCCAG8
chr4:25144773-25159107	13	SEPSECS
chr2:109564365-109567976	29	10-Sep
chr9:132278069-132281574	13	SETX
chr16:46603538-46608389	5	SHCBP1
chr11:26678711-26686544	10	SLC5A12
chr3:47693240-47701402	10	SMARCC1
chr1:246327200-246335474	34	SMYD3

chr2:45546731-45585774	10	SRBD1
chr1:29148079-29154910	23	SRSF4
chr11:126406095-126409411	12	ST3GAL4
chr1:112597970-112616895	30	ST7L
chr6:159783940-159788143	27	TCP1
chr9:79652589-79654075	9	TLE4
chr3:129832773-129880559	26	TMCC1
chr20:41112781-41114155	6	TOP1
chr18:31890734-31917667	12	TRAPPC8
chr6:80027884-80035417	6	TTK
chr3:23499607-23532701	12	UBE2E2
chr2:169905113-169933008	11	UBR3
chr16:21957234-21962885	5	UQCRC2
chr19:30012284-30012531	5	URI1
chr1:77729859-77739417	9	USP33
chr6:144482208-144488834	23	UTRN
chr3:184924861-184971752	10	VPS8
chr6:110100525-110108681	4	WASF1
chr13:26612960-26671989	19	WASF3
chr1:108991253-108995837	4	WDR47
chr16:74948518-74965822	12	WDR59
chr19:47081512-47094608	6	ZC3H4
chr1:31310043-31339048	21	ZCCHC17
chr10:45662579-45663842	45	ZFAND4
chr1:35093913-35110447	15	ZMYM1
chr17:32362913-32368014	51	ZNF207
chr9:106938915-106984409	3	ZNF462
chr7:30323641-30324302	17	ZNRF2
chr1:52856997-52864157	25	ZYG11A
chr3:56566593-56567053	324	CCDC66
chr1:197553021-197772932	11	DENND1B
chr22:47686199-47704747	64	LOC284930
chr11:116896252-116927380	11	SIK3
chr19:12714820-12715739	200	TNPO2
chr11:77619605-77629899	371	CLNS1A
chr6:56670640-56704369	35	DST
chr3:96866824-96987993	5	EPHA6
chr6:5368549-5369182	51	FARS2
chr8:144357139-144357438	10	FBXL6
chr1:59321535-59332032	13	FGGY
chr14:35126734-35127611	36	KIAA0391
chr6:89781398-89781592	9	MDN1
chr11:93788000-93794035	53	MED17

chr15:78887268-78894230	21	MORF4L1
chr1:145405097-145589039	11	NBPF20
chr2:53919150-53921104	5	PSME4
chr9:112268048-112276013	18	PTBP3
chr6:75621531-75647801	18	SENP6
chr12:40028300-40092933	20	SLC2A13
chrX:101018970-101019403	45	TRMT2B
chr21:37144524-37147603	10	TTC3
chr4:82447919-82451245	11	ENOPH1
chr19:11169876-11170248	3	KANK2
chr1:27789561-27801815	21	STX12
chr3:160356012-160381718	74	IFT80
chr2:196292564-196308085	5	HECW2
chr10:27012881-27014711	19	ANKRD26
chr8:38128290-38128951	20	ASH2L
chr5:37114959-37125409	27	C5orf42
chr22:37496448-37510421	4	CARD10
chr11:119296917-119297481	8	CBL
chr7:121266226-121271430	7	CPED1
chr1:26446315-26460144	35	DHDDS
chr2:25576837-25628384	9	DTNB
chr1:21050865-21111383	252	EIF4G3
chr12:11752449-11853561	11	ETV6
chr12:110485077-110487943	170	FAM216A
chr3:172251259-172262083	148	FNDC3B
chr7:42148225-42223295	17	GLI3
chr22:23659864-23686995	55	GUSBP11
chr9:112438430-112441770	8	HSDL2
chr11:77928341-77981576	3	INTS4
chrX:19407187-19464406	4	MAP3K15
chr17:29165941-29167021	10	MYO18A
chr12:78006418-78021862	18	NAV3
chr2:18644169-18665115	6	nogene
chr5:135074416-135075388	10	nogene
chr1:207815928-207817414	141	nogene
chr5:15579089-15580812	3	nogene
chr16:14552020-14584791	21	PARN
chr11:111754498-111759951	5	PPP2R1B
chr2:99421498-99435941	6	REV1
chr16:66528993-66541953	13	TK2
chr3:69029814-69035115	9	TMF1
chr7:23507419-23516528	9	TRA2A
chr14:54966474-54967394	52	WDHD1

chr4:1892165-1893909	13	WHSC1
chr9:14672829-14680162	29	ZDHHC21
chr18:76849525-76855201	173	ZNF236
chr2:100566502-100569721	24	PDCL3
chr17:60207020-60209543	6	USP32
chr3:184936245-184957521	9	VPS8
chr8:70583163-70598255	20	TRAM1
chr14:71658332-71672622	10	SIPA1L1
chr7:36410513-36411166	25	ANLN
chr14:102963060-102964650	7	CDC42BPB
chr15:44331987-44380976	20	CASC4
chr9:111440371-111444494	20	KIAA0368
chr6:167914167-167925091	10	MLLT4
chr7:103303841-103304490	106	PMPCB
chr4:105437949-105456745	9	PPA2
chr6:10907541-10912927	10	SYCP2L
chr6:121317494-121321794	5	TBC1D32
chr10:125038996-125040877	4	CTBP2
chr10:100174207-100179247	10	ERLIN1
chr1:89843637-89846658	18	LRRC8D
chr11:134206598-134210454	9	NCAPD3
chr5:168561392-168566266	8	PANK3
chr11:94583447-94589232	21	PIWIL4
chr1:151166933-151167414	181	TNFAIP8L2-SCNM1
chr19:51573209-51581882	27	ZNF175
chr18:21761441-21779685	29	MIB1
chr15:20669467-20683500	3	NBEAP1
chr3:136398748-136477412	9	STAG1
chr19:51860903-51880877	73	ZNF577
chr9:71874613-71875083	16	ABHD17B
chr1:36036176-36043548	8	AGO3
chr3:43549719-43565727	45	ANO10
chr4:41100638-41143142	18	APBB2
chr4:152829624-152888307	14	ARFIP1
chr5:142879373-142913293	12	ARHGAP26
chr8:130179263-130180880	21	ASAP1
chr2:215325240-215333457	9	ATIC
chr3:130992950-131001344	9	ATP2C1
chr7:73462921-73470483	9	BAZ1B
chr2:32478634-32493667	42	BIRC6
chr9:16727796-16738485	17	BNC2
chr21:39278742-39298582	16	BRWD1
chr7:7238254-7243750	9	C1GALT1

chr20:3340503-3359593	9	C20orf194
chr9:94759648-94801002	10	C9orf3
chr9:114636989-114638726	24	C9orf91
chr19:10391262-10391706	7	CDC37
chr21:36397414-36402851	10	CHAF1B
chr9:121161855-121162271	13	CNTRL
chr19:18759550-18760228	12	CRTC1
chr15:64259200-64300723	6	CSNK1G1
chr11:118759921-118763306	21	DDX6
chr10:68450027-68470163	10	DNA2
chr14:74004604-74015930	9	ENTPD5
chr2:26364301-26367220	20	EPT1
chr10:87151349-87158155	20	FAM35A
chr1:77801330-77815231	358	FAM73A
chr16:89758576-89767237	6	FANCA
chr5:108341926-108367953	29	FBXL17
chr4:53379224-53399839	5	FIP1L1
chr3:172247532-172298787	13	FNDC3B
chr14:30597419-30602131	13	G2E3
chr1:168104476-168104894	48	GPR161
chr22:23705428-23709867	94	GUSBP11
chr2:26269952-26277160	9	HADHB
chr2:26263379-26277160	182	HADHB
chr5:150036635-150041969	4	HMGXB3
chr7:23342063-23346062	40	IGF2BP3
chr6:149597062-149598350	10	KATNA1
chr15:34145397-34148750	12	KATNBL1
chr1:23050386-23057565	28	KDM1A
chr12:362962-376724	7	KDM5A
chr10:32022139-32023036	14	KIF5B
chr17:47663088-47665158	21	KPNB1
chr12:50430494-50454417	12	LARP4
chr1:200368664-200374246	8	LINC00862
chr4:150282449-150286034	9	LRBA
chr2:33020908-33021206	14	LTBP1
chr1:117493146-117502970	81	MAN1A2
chr2:227328677-227329783	10	MFF
chr6:97151770-97186690	4	MMS22L
chr2:74165217-74172752	36	MOB1A
chr10:133395712-133419592	8	MTG1
chr22:29970975-29998857	29	MTMR3
chr2:176296859-176326901	9	MTX2
chr12:112087682-112093136	4	NAA25

chr1:236011920-236048989	8	NID1
chr5:34179012-34182867	289	nogene
chr2:183316754-183334209	16	nogene
chr6:43598542-43599089	18	nogene
chrX:121090398-121151724	4	nogene
chr8:128009589-128074291	4	nogene
chrX:131364748-131389148	36	nogene
chr5:73447242-73448206	20	nogene
chr12:68692486-68692844	19	nogene
chr7:19020901-19056165	28	nogene
chr3:185417467-185418306	24	nogene
chrX:131364748-131399704	45	nogene
chr11:14296054-14356769	23	nogene
chr2:111208487-111208936	9	nogene
chr21:15014343-15015225	9	NRIP1
chr10:79691621-79692056	57	NUTM2B-AS1
chr5:41749526-41807438	27	OXCT1
chr20:3907925-3918796	19	PANK2
chr12:53461014-53471807	4	PCBP2
chr3:167695595-167725473	8	PDCD10
chrX:134413490-134425330	3	PHF6
chr3:142664201-142678113	38	PLS1
chr14:49663314-49666413	16	POLE2
chr22:46176752-46198591	36	PPARA
chr14:103778744-103797518	865	PPP1R13B
chr14:73147794-73148106	67	PSEN1
chr12:112472943-112482205	10	PTPN11
chr18:12825809-12836891	10	PTPN2
chr2:119877322-119915242	15	PTPN4
chr20:2964271-2965202	357	PTPRA
chr2:20318536-20333063	21	PUM2
chr9:136223762-136226874	112	QSOX2
chr11:66271796-66272460	7	RAB1B
chr1:220189702-220191284	3	RAB3GAP2
chr12:15217428-15348437	15	RERG
chr1:176135009-176184692	8	RFWD2
chr9:3330258-3395596	18	RFX3
chr11:71982760-71990717	81	RNF121
chr6:88769773-88904955	15	RNGTT
chr17:59935192-59940943	45	RPS6KB1
chr3:158122102-158146771	4	RSRC1
chr10:12160929-12162289	11	SEC61A2
chr3:185619298-185624082	6	SENP2

chr13:29532823-29536202	40	SLC7A1
chr3:47710682-47772936	13	SMARCC1
chr16:69235747-69245815	11	SNTB2
chr7:17814833-17850964	15	SNX13
chr1:179335505-179341310	34	SOAT1
chr1:109336239-109342158	4	SORT1
chr8:100220278-100233537	87	SPAG1
chr17:50970706-50987237	26	SPAG9
chr9:92067965-92080963	17	SPTLC1
chr2:152132113-152150229	9	STAM2
chr10:94441895-94458595	6	TBC1D12
chr22:47034455-47037198	11	TBC1D22A
chr8:93772588-93782460	11	TMEM67
chr6:30196515-30204765	4	TRIM26
chr17:59051213-59057054	55	TRIM37
chr1:247860616-247868063	163	TRIM58
chr5:14286870-14304592	43	TRIO
chr15:43398040-43401850	6	TUBGCP4
chr5:134388324-134390363	9	UBE2B
chr2:128164729-128171284	164	UGGT1
chr1:162503753-162512823	9	UHMK1
chr7:7786787-7801743	49	UMAD1
chr9:35228014-35243364	170	UNC13B
chr16:21961327-21976243	14	UQCRC2
chr1:7773441-7778169	9	VAMP3
chr8:99096311-99111279	20	VPS13B
chr14:55000502-55002185	4	WDHD1
chr9:134139819-134142719	11	WDR5
chr12:1631297-1646297	24	WNT5B
chr8:31096767-31100955	9	WRN
chr6:43565659-43567354	51	XPO5
chr5:83104909-83204921	21	XRCC4
chr10:27119293-27126786	7	YME1L1
chr1:32633644-32634792	134	ZBTB8OS
chr6:38061592-38061775	25	ZFAND3
chr16:88485938-88489153	10	ZFPM1
chr7:149128822-149129542	5	ZNF398
chr2:71396140-71400180	19	ZNF638
chr12:50999173-51010766	18	SLC11A2
chr22:45689711-45693078	5	ATXN10
chrX:68511848-68513405	9	YIPF6
chr2:120127687-120175000	59	EPB41L5
chr19:7440343-7447168	12	ARHGEF18

chr2:232136471-232163632	10	DIS3L2
chr4:25847293-25847864	9	SEL1L3
chr10:11747348-11755608	171	ECHDC3
chr12:100594874-100612141	6	GAS2L3
chr16:74300114-74302292	97	PSMD7
chr3:141501948-141540609	11	RASA2
chr5:121974195-121994670	18	SRFBP1
chr1:43920403-43920928	80	ST3GAL3
chr17:40395448-40396465	30	TOP2A
chr2:128113083-128145967	8	UGGT1
chr4:1892165-1939778	28	WHSC1
chr6:43560177-43573601	30	XPO5
chr8:78675786-78698513	16	ZC2HC1A
chr5:112821895-112828006	3	APC
chr17:73235475-73236995	13	C17orf80
chr4:168998201-169007756	4	CBR4
chr16:75391379-75395209	15	CFDP1
chr20:50935029-50955285	4	DPM1
chr6:5404541-5445504	10	FARS2
chr2:169555123-169563350	23	FASTKD1
chr17:44424348-44464544	6	GPATCH8
chr12:350620-366104	36	KDM5A
chr3:185437446-185437630	10	MAP3K13
chr18:54187713-54205157	20	MBD2
chr14:103390239-103390731	19	nogene
chr1:15691314-15691854	10	nogene
chr4:94640275-94657547	4	PDLIM5
chr19:7542560-7551437	6	PNPLA6
chr7:155672866-155707068	4	RBM33
chr7:5729431-5741815	11	RNF216
chr11:9807999-9809002	31	SBF2
chr5:69100806-69114496	6	SLC30A5
chr5:122801868-122803613	4	SNX2
chr3:113493205-113506605	11	SPICE1
chr1:47301560-47310362	15	STIL
chr20:7996025-8010315	31	TMX4
chr18:31832083-31839457	24	TRAPPC8
chr9:33944364-33956146	89	UBAP2
chr13:29767098-29777263	246	UBL3
chr12:95294271-95296258	31	VEZT
chr2:71363152-71380565	12	ZNF638
chr5:32397218-32404097	11	ZFR
chr1:179126376-179131481	9	ABL2

chr20:45848449-45855292	7	ACOT8
chr1:212997154-213013418	3	ANGEL2
chr5:71522288-71532427	7	BDP1
chr16:50354424-50361932	11	BRD7
chr3:143985542-144048812	13	C3orf58
chr9:114624349-114628250	13	C9orf91
chr22:46707842-46721015	11	CERK
chr1:155725381-155726019	52	DAP3
chr12:31447537-31452476	11	DENND5B
chr1:50490587-50596216	17	FAF1
chr11:20364236-20383258	16	HTATIP2
chr5:146144266-146144709	3	LARS
chr10:71828980-71831247	6	nogene
chr15:67604824-67605108	13	nogene
chr7:104161071-104168525	49	ORC5
chr4:2156787-2179465	17	POLN
chr4:25333182-25333988	159	ZCCHC4
chr11:9471301-9479546	3	ZNF143
chr21:45486860-45488444	6	COL18A1
chr16:11123741-11123946	12	CLEC16A
chr17:82183773-82193830	8	CCDC57
chr17:47143882-47172064	6	CDC27
chr3:33683265-33696933	5	CLASP2
chr8:119838258-119847080	53	DSCC1
chr5:41927012-41929917	64	FBXO4
chr14:50475076-50486194	85	MAP4K5
chr2:134317528-134362408	5	MGAT5
chr13:98504914-98519473	41	STK24
chr6:158573419-158585425	6	TMEM181
chr6:37929958-38061775	4	ZFAND3
chr6:100766564-100805880	19	ASCC3
chr10:102033025-102040125	7	C10orf76
chr1:44284834-44319744	99	ERI3
chr1:160279800-160283639	12	PEX19
chr1:10256246-10268263	8	KIF1B
chr6:32118955-32120902	11	ATF6B
chr1:70296129-70306290	48	ANKRD13C
chr15:89871474-89889140	191	AP3S2
chr17:8206490-8207840	91	AURKB
chr14:80862756-80906081	71	CEP128
chr12:108701123-108717762	11	CORO1C
chr1:161123838-161124526	12	DEDD
chr18:49281996-49379758	17	DYM

chr11:103303092-103316620	109	DYNC2H1
chr6:52423686-52465115	5	EFHC1
chr2:24060813-24063782	8	FKBP1B
chr8:43059091-43085396	23	FNTA
chr20:25418104-25441776	47	GINS1
chr3:129458598-129469417	11	IFT122
chr7:2014501-2069338	16	MAD1L1
chr14:47061248-47097123	11	MDGA2
chrX:40713777-40715220	18	MED14
chr19:42335930-42351761	4	MEGF8
chr18:36203112-36220217	4	MOCOS
chr2:42682400-42697834	19	MTA3
chr17:31155982-31219118	11	NF1
chr1:5961793-5986232	12	NPHP4
chr17:75208526-75215823	11	NUP85
chr1:224231225-224268133	16	NVL
chr10:73454411-73479517	13	PPP3CB
chr9:123070349-123076762	10	RABGAP1
chr5:131548046-131592466	26	RAPGEF6
chr19:38130497-38142710	19	SIPA1L3
chr5:134157706-134161130	8	SKP1
chr6:44227262-44232428	5	SLC29A1
chr18:51054780-51059916	13	SMAD4
chr4:185339378-185342116	18	SNX25
chr17:38361710-38377842	144	SOCS7
chr3:136398748-136422613	3	STAG1
chr15:50609580-50611321	5	TRPM7
chr5:61472680-61473037	147	ZSWIM6
chr11:83198478-83211430	11	ANKRD42
chr3:48979481-48980496	319	ARIH2
chr2:218228737-218239484	4	ARPC2
chr3:11298685-11313420	4	ATG7
chr2:214752446-214797117	55	BARD1
chr7:33383665-33388144	6	BBS9
chr8:85281229-85281639	10	CA13
chr5:180573987-180576684	18	CNOT6
chr15:44490783-44496458	11	CTDSPL2
chr16:20860047-20862643	9	DCUN1D3
chr2:25274940-25276166	5	DNMT3A
chr1:28035543-28048426	112	EYA3
chr3:138619710-138629156	9	FAIM
chr14:65455620-65464473	16	FUT8
chr5:151799888-151800869	18	G3BP1

chr5:52861446-52865817	9	ITGA1
chr1:35470862-35474906	5	KIAA0319L
chr4:165219933-165238899	228	KLHL2
chr20:32833716-32836841	7	MAPRE1
chr1:46562648-46572167	10	MKNK1
chr11:47638698-47639051	26	MTCH2
chrX:64331556-64337393	7	MTMR8
chr12:56155418-56159730	126	MYL6B
chr19:37001330-37001683	7	nogene
chr2:236264772-236268343	12	nogene
chr14:96931330-96945382	5	nogene
chr3:52637868-52638468	10	nogene
chr11:6497449-6498939	5	nogene
chr10:27151187-27151637	10	nogene
chr17:81610209-81622278	12	NPLOC4
chr19:46383878-46384794	3	PPP5C
chr8:140702569-140706205	16	PTK2
chr6:43024984-43029190	9	RRP36
chr5:172350362-172373815	12	SH3PXD2B
chr1:9553625-9567361	4	SLC25A33
chr3:155829687-155834041	26	SLC33A1
chr9:96320997-96322080	29	SLC35D2
chr19:43747441-43750747	10	SMG9
chr15:44633504-44657263	3	SPG11
chr2:85861180-85870258	10	ST3GAL5
chr17:64188167-64195095	3	TEX2
chr3:196075158-196077122	26	TFRC
chr13:100642210-100670569	10	TMTC4
chr17:18292644-18306966	21	TOP3A
chr6:158282262-158314268	16	TULP4
chr7:67067284-67098718	42	TYW1
chr3:41681507-41705305	10	ULK4
chr2:218457091-218463366	3	USP37
chr12:121934044-121934356	19	WDR66
chr6:43567168-43573601	3	XPO5
chr19:57988206-57999896	8	ZNF606
chr19:36094030-36099617	10	WDR62
chrX:107029126-107030315	12	nogene
chr8:89587890-89596481	5	nogene
chr11:85996825-86001158	35	PICALM
chr1:8495062-8541318	52	RERE
chr10:31852516-31861658	11	ARHGAP12
chr8:38114191-38116725	4	ASH2L

chr17:67959540-67975958	4	BPTF
chr22:28710005-28734727	32	CHEK2
chr12:92826165-92853025	10	EEA1
chr20:34089515-34090602	33	EIF2S2
chr3:33358966-33364719	20	FBXL2
chr4:53414633-53428183	8	FIP1L1
chr15:90486953-90487582	3	IQGAP1
chr10:32908367-32935558	25	ITGB1
chr2:30525586-30568176	24	LCLAT1
chr22:40552105-40586112	10	MKL1
chr15:65023671-65027040	12	MTFMT
chr11:9261128-9261546	6	nogene
chr9:111591219-111591479	17	nogene
chr6:126112865-126130858	8	nogene
chr22:31648100-31650778	7	nogene
chr1:233135012-233208689	53	PCNXL2
chr20:18142207-18142919	36	PET117
chr20:35842572-35847434	41	PHF20
chr20:34585436-34616139	3	PIGU
chr3:121529644-121544906	6	POLQ
chr11:68544837-68558679	3	PPP6R3
chr1:31055312-31059577	39	PUM1
chr3:128806371-128813471	29	RAB7A
chr5:223481-226047	9	SDHA
chr7:2275111-2278305	40	SNX8
chr17:50984922-50989872	4	SPAG9
chr17:20017986-20110562	30	SPECC1
chr8:123077110-123101007	93	TBC1D31
chr3:119500694-119504021	178	TIMMDC1
chrX:118387339-118398470	12	WDR44
chr1:26760855-26763285	51	ARID1A
chr7:6421726-6430607	8	DAGLB
chr22:35292991-35293743	25	HMGXB4
chr10:68175322-68195532	55	MYPN
chr7:78828294-78902612	11	nogene
chr1:233217898-233252788	5	PCNXL2
chr6:3737078-3738148	11	PXDC1
chr3:108057476-108071182	5	CD47
chr2:233434379-233441995	8	DGKD
chr10:5768212-5773941	24	GDI2
chr1:94203929-94231643	5	ARHGAP29
chr3:27256295-27284961	9	NEK10
chr7:26684735-26727006	30	SKAP2

chr3:121772556-121781874	4	IQCB1
chr16:81312727-81313120	6	nogene
chr3:72841044-72842402	26	SHQ1
chr2:97833364-97844261	12	TMEM131
chr2:170073427-170080684	4	UBR3
chr12:95105006-95141539	13	FGD6
chr18:46122466-46124893	17	HAUS1
chr2:111344060-111367575	9	MIR4435-2HG
chr2:61492034-61495613	19	XPO1
chr20:35800093-35812719	6	nogene
chr1:90005132-90007750	23	ZNF326
chr3:195333027-195345317	4	ACAP2
chr12:32710928-32722633	17	DNM1L
chr1:23053760-23059167	5	KDM1A
chr17:39466521-39467073	10	nogene
chr3:45703437-45709647	18	SACM1L
chr5:172211966-172234358	16	UBTD2
chr1:150966161-150969091	15	CERS2
chrX:152846281-152858916	17	NSDHL
chr4:184685408-184685684	11	PRIMPOL
chr6:129638393-129642018	14	ARHGAP18
chr7:101916114-102195603	8	CUX1
chr12:132120419-132121980	24	EP400NL
chr9:21859302-21862135	11	MTAP
chr5:37333462-37341242	8	NUP155
chr2:10791794-10793202	129	PDIA6
chr8:132777795-132798860	14	PHF20L1
chr1:186893010-186894211	4	PLA2G4A
chr3:100368043-100375152	6	TOMM70A
chr2:63313247-63382094	28	WDPCP
chr16:68093355-68126610	16	NFATC3
chr1:120741807-120763709	97	nogene
chr11:108299713-108304852	45	ATM
chr9:85669484-85692813	93	AGTPBP1
chr6:89595915-89606105	5	ANKRD6
chr8:61633682-61653660	91	ASPH
chr6:80167677-80203212	9	BCKDHB
chr15:59116689-59117368	9	CCNB2
chr11:47482694-47483532	21	CELF1
chr20:35458303-35462553	5	CEP250
chr1:111147641-111174963	27	CEPT1
chr7:107324439-107372760	5	COG5
chr11:88300529-88312554	10	CTSC

chr11:107329917-107353736	18	CWF19L2
chr7:87884978-87897339	11	DBF4
chr15:65454262-65456371	10	DPP8
chr6:131882344-131886724	4	ENPP1
chr19:41391732-41392980	15	EXOSC5
chr20:58895611-58905480	33	GNAS
chr10:13191298-13192568	147	MCM10
chr22:36316516-36318325	6	MYH9
chr12:77994802-78021862	5	NAV3
chr19:5710355-5710782	31	nogene
chr10:31355146-31387266	15	nogene
chr9:110784812-110785728	18	nogene
chr8:125102013-125182256	8	NSMCE2
chr2:219565241-219568324	4	OBSL1
chr8:89909566-89921171	10	OSGIN2
chr3:136260478-136317064	21	PCCB
chr15:64156161-64156724	16	PPIB
chr19:45138513-45142458	9	PPP1R37
chr11:82849048-82853278	11	PRCP
chr10:98231511-98236164	5	R3HCC1L
chr5:87331347-87341321	25	RASA1
chr3:149846010-149921227	348	RNF13
chr9:105335562-105358433	9	SLC44A1
chr13:29532823-29553860	115	SLC7A1
chr16:68273794-68275249	13	SLC7A6
chr16:67255020-67258447	5	SLC9A5
chr3:47662333-47678311	4	SMARCC1
chr1:47295764-47304996	24	STIL
chr11:64199939-64200293	11	STIP1
chr6:85618817-85619417	102	SYNCRIP
chrX:71423116-71424238	32	TAF1
chr17:63193990-63200957	53	TANC2
chr15:101701085-101703937	7	TARSL2
chr7:143875988-143876622	18	TCAF1
chr2:171011372-171028405	13	TLK1
chr4:55403235-55411839	14	TMEM165
chr7:98967040-98967698	21	TRRAP
chr10:114951093-114974385	14	TRUB1
chr7:67055834-67067403	52	TYW1
chr7:157201720-157225539	23	UBE3C
chr7:139237004-139259370	11	UBN2
chr11:113840597-113841768	5	USP28
chr7:93303459-93334197	18	VPS50

chr13:26612960-26642403	28	WASF3
chr17:27303366-27309272	28	WSB1
chr4:183173557-183193708	5	WWC2
chr17:83002672-83048785	5	B3GNTL1
chr11:68567013-68576043	74	PPP6R3
chr9:109080838-109108570	101	TMEM245
chr10:27134830-27136385	27	YME1L1
chr1:198262574-198293039	4	NEK7
chr1:8495062-8614686	52	RERE
chr10:13322834-13345028	9	SEPHS1
chr2:61331275-61350693	4	USP34
chr3:196321303-196322714	8	nogene
chr10:87925512-87952259	58	PTEN
chr12:50461134-50467120	19	LARP4
chr11:122794701-122799034	3	UBASH3B
chr10:31839636-31854206	8	ARHGAP12
chr7:56054368-56056414	12	CCT6A
chr6:82223439-82231842	32	IBTK
chr7:5982823-5992057	12	PMS2
chr6:43497880-43503508	18	TJAP1
chr10:101797979-101810314	3	MGEA5
chr17:35624396-35650789	7	AP2B1
chr19:47746966-47752607	5	GLTSCR2
chr5:135388921-135393187	5	H2AFY
chr7:74255596-74255853	8	nogene
chr20:38488402-38509208	4	RALGAPB
chr21:42859661-42863596	39	WDR4
chr10:68906710-68919981	18	DDX50
chr2:15473221-15478289	5	NBAS
chr9:93439402-93441373	11	nogene
chr20:62256056-62264718	4	OSBPL2
chr8:47897160-47930012	11	PRKDC
chr14:68291883-68411527	4	RAD51B
chr17:7574545-7577694	11	SENP3-EIF4A1
chr3:57841298-57864716	9	SLMAP
chr7:127807483-127904819	58	SND1
chr1:1564486-1564916	13	SSU72
chr3:12496517-12503784	30	TSEN2
chr3:51310231-51315128	6	DOCK3
chr12:50430494-50441643	71	LARP4
chr5:151927173-151947795	9	nogene
chr8:72052538-72076627	59	MSC-AS1
chr9:33351559-33354892	50	NFX1

chr1:100912111-100921841	5	SLC30A7
chr4:152411302-152411872	15	FBXW7
chr12:111888487-111893585	4	MAPKAPK5
chr17:18911102-18928957	26	PRPSAP2
chr17:783082-783327	6	RNMTL1
chr19:19024618-19031082	33	SUGP2
chr17:82884144-82903478	156	TBCD
chr1:32161946-32167296	17	KPNA6
chr3:9424466-9435906	21	SETD5
chr20:32145090-32146855	12	TM9SF4
chr11:78112649-78121174	45	ALG8
chr12:32216246-32306122	6	BICD1
chr15:41243666-41270618	14	CHP1
chr4:77756861-77776392	12	CNOT6L
chr7:134148237-134183853	4	LRGUK
chr11:73825707-73844976	30	MRPL48
chr9:126340507-126392195	9	MVB12B
chr22:25509182-25510862	7	nogene
chr3:38190730-38198863	10	OXSR1
chr3:196801926-196803164	57	PAK2
chr17:66468590-66496283	8	PRKCA
chr2:238347454-238356080	17	TRAF3IP1
chr6:41871563-41916939	36	USP49
chr18:56731382-56758953	47	WDR7
chr9:37126311-37327834	35	ZCCHC7
chr3:171188701-171194635	90	TNIK
chr18:13029662-13042334	12	CEP192
chr7:55978125-55981567	9	GBAS
chr16:69669976-69684970	17	NFAT5
chr6:52452687-52454287	6	EFHC1
chr6:31892406-31892910	136	EHMT2
chr6:149578821-149582603	8	GINM1
chr15:50465046-50477499	6	USP8
chr13:32535768-32537027	64	N4BP2L2
chrX:19929784-19935898	8	CXorf23
chr19:19655527-19656183	5	ATP13A1
chr17:30266884-30286902	15	BLMH
chr20:49058452-49066510	9	CSE1L
chr5:135360496-135370142	27	H2AFY
chr3:23954536-23959815	18	NR1D2
chr15:68141945-68164804	85	PIAS1
chrX:338603-362332	13	PPP2R3B
chr1:151024610-151028944	5	PRUNE

chr5:176289653-176296320	17	SIMC1
chr3:67518246-67529186	19	SUCLG2
chr2:112064161-112067067	11	TMEM87B
chr10:84371013-84425893	4	CCSER2
chrX:52955153-52956755	17	FAM156A
chr10:5726884-5731203	13	FAM208B
chr1:16294782-16316571	25	FBXO42
chr15:48717017-48729540	9	nogene
chr10:111017221-111053120	12	nogene
chr6:3076763-3085408	72	RIPK1
chr14:35107303-35110629	19	PPP2R3C
chr2:31891991-31943383	10	MEMO1
chr20:25020009-25030958	5	ACSS1
chr5:140506834-140513479	9	ANKHD1-EIF4EBP3
chr10:72715110-72736571	7	MCU
chrX:18948743-18954412	10	PHKA2
chr9:122877470-122883379	43	RC3H2
chr16:88027482-88065332	7	BANP
chr4:77742140-77776392	27	CNOT6L
chr1:108943474-108944057	16	CLCC1
chr2:239134244-239163923	9	HDAC4
chr16:2566355-2586893	9	PDPK1
chr9:134139819-134140811	55	WDR5
chr15:51463378-51464876	15	DMXL2
chr6:31742876-31744581	16	MSH5
chr4:23120319-23186098	12	nogene
chr7:17821508-17875790	13	SNX13
chr10:1105125-1125024	5	WDR37
chr19:10151397-10162748	4	DNMT1
chr14:69203916-69209803	12	EXD2
chr7:102822039-102884312	12	FBXL13
chr3:145983607-146034688	9	nogene
chr16:625431-626121	12	RAB40C
chr17:80891719-80893865	117	RPTOR
chr11:47161190-47161436	16	C11orf49
chr2:29121373-29133816	11	CLIP4
chr19:12954139-12954526	61	GADD45GIP1
chr2:85542564-85543034	29	MAT2A
chr8:37877108-37877551	53	RAB11FIP1
chr12:122261260-122264199	16	VPS33A
chr14:73024025-73024942	9	ZFYVE1
chr1:28459084-28466535	25	PHACTR4
chr11:61437624-61446113	21	SDHAF2

chr5:134703758-134718173	11	SEC24A
chr3:197149742-197194589	52	DLG1
chr2:27677294-27682359	25	SLC4A1AP
chr20:52088391-52098587	73	ZFP64
chr16:16007815-16014628	7	ABCC1
chr10:115120184-115165645	46	ATRNL1
chr15:72544819-72555907	24	ARIH1
chr15:89113724-89116521	299	ABHD2
chr16:71746587-71750332	9	AP1G1
chr20:33395012-33397084	17	CDK5RAP1
chr20:5175182-5178956	10	CDS2
chr16:53231418-53243016	16	CHD9
chr5:138824529-138827718	71	CTNNA1
chr11:107348936-107418287	10	CWF19L2
chr15:65756139-65756472	4	DENND4A
chr6:158637127-158641360	7	DYNLT1
chr17:7850071-7851394	13	KDM6B
chr2:37236095-37248426	12	NDUFAF7
chr9:33624555-33624763	9	nogene
chr1:155675616-155675970	20	nogene
chr13:28664508-28672432	10	POMP
chrX:18246576-18247882	14	SCML2
chr3:27403138-27452498	10	SLC4A7
chr17:50990449-50998617	6	SPAG9
chr2:161367477-161395203	123	PSMD14
chr16:14644273-14655210	11	BFAR
chr19:49614532-49616219	32	PRR12
chr3:72792915-72824551	8	SHQ1
chr12:120716627-120717042	16	UNC119B
chr13:31229184-31261082	16	B3GLCT
chr12:1371832-1408247	14	ERC1
chr17:39264174-39265453	16	FBXL20
chr20:31527483-31528050	50	HM13
chr8:54044816-54052756	4	LYPLA1
chr5:151458787-151479489	51	SLC36A1
chr9:33996222-34017189	121	UBAP2
chr8:99013759-99038566	10	VPS13B
chr2:32510525-32513154	37	BIRC6
chr15:69255793-69261329	9	GLCE
chr3:161224929-161235212	45	NMD3
chrX:17477145-17482724	12	nogene
chr11:65503116-65504595	20	nogene
chrX:45844850-45845520	10	nogene

chr19:16640919-16641651	13	nogene
chr3:12312379-12381491	8	PPARG
chr8:127939507-127989291	10	PVT1
chr12:106851449-106860412	27	RIC8B
chr11:60919685-60920988	46	TMEM109
chr16:2317282-2317764	24	ABCA3
chr22:31447425-31449531	9	EIF4ENIF1
chr6:73003907-73042062	4	KCNQ5
chr12:131725404-131728428	16	SFSWAP
chr7:105073618-105077433	72	KMT2E
chr18:9211583-9221999	4	ANKRD12
chr10:32919884-32930044	4	ITGB1
chr17:27303366-27310174	7	WSB1
chr11:66639699-66640123	330	RBM4
chr8:139730657-139732202	9	TRAPPC9
chr13:100330560-100368574	12	PCCA
chr6:130312977-130334845	7	SAMD3
chr5:140440118-140445975	174	ANKHD1-EIF4EBP3
chr4:51860542-51877854	32	DCUN1D4
chr10:119065398-119073937	17	EIF3A
chr7:152309965-152315338	564	KMT2C
chr7:105073618-105078963	16	KMT2E
chr1:171783739-171790616	7	METTL13
chr4:145150512-145171720	12	OTUD4
chr1:213077695-213117410	145	RPS6KC1
chr8:100220278-100225339	8	SPAG1
chr13:19993062-19993919	20	ZMYM2
chr6:116645713-116658924	6	ZUFSP
chr8:131940498-131955905	18	EFR3A
chr4:2657802-2663288	19	FAM193A
chr3:172247532-172285984	18	FNDC3B
chr3:155914418-155916192	8	GMPS
chr10:119930328-119933787	8	SEC23IP
chr6:145864387-145894977	15	SHPRH
chr3:136464880-136502779	33	STAG1
chr1:185174371-185175113	4	SWT1
chr6:158081108-158081510	11	SYNJ2
chr15:91000489-91003131	10	VPS33B
chr20:49279103-49280964	17	ZFAS1
chr8:140793353-140803650	4	PTK2
chr4:56378147-56384341	10	AASDH
chr3:15707923-15737233	7	ANKRD28
chr17:35657598-35682824	8	AP2B1

chr8:102838743-102839823	11	AZIN1
chr12:32216246-32216459	8	BICD1
chr17:44154555-44157941	13	C17orf53
chr5:138319218-138326054	16	CDC25C
chr1:243169627-243225321	3	CEP170
chr12:31479588-31495919	7	DENND5B
chr5:55267137-55272175	25	DHX29
chr13:98920953-98923371	3	DOCK9
chr10:132196860-132197101	24	DPYSL4
chr6:56670640-56843238	8	DST
chr1:20879322-20904971	8	EIF4G3
chr6:79924951-79926381	9	ELOVL4
chr18:45948502-45952643	39	EPG5
chr1:44339044-44339322	16	ERI3
chr1:50535368-50567231	11	FAF1
chr7:22964414-22991139	68	FAM126A
chr19:38948566-38950336	10	FBXO17
chr4:893376-904779	5	GAK
chr1:1815755-1853297	10	GNB1
chr18:46122466-46123364	14	HAUS1
chr2:38577460-38591648	17	HNRNPLL
chr1:153759525-153765063	17	INTS3
chr8:94827262-94829026	13	INTS8
chr13:30208576-30210442	9	KATNAL1
chr1:183108275-183110654	4	LAMC1
chr10:829384-845055	4	LARP4B
chr10:884446-885760	43	LARP4B
chr7:128642216-128648982	31	LINC01000
chr8:55950458-55969793	36	LYN
chr5:139318907-139319501	15	MATR3
chr10:101798841-101810314	5	MGEA5
chr21:36338767-36344707	7	MORC3
chr12:65308528-65369026	24	MSRB3
chr9:21859302-21864433	9	MTAP
chr8:120459214-120470937	18	MTBP
chr15:71875790-71888116	17	MYO9A
chr5:178885613-178887787	11	nogene
chr4:110418220-110419105	3	nogene
chr13:29513931-29514149	10	nogene
chrX:148522107-148546313	10	nogene
chr19:11648357-11652053	19	nogene
chrX:129116609-129122193	10	nogene
chr7:73550467-73550767	9	nogene

chr19:49490482-49491871	6	nogene
chr8:58623219-58635546	94	NSMAF
chr11:77340645-77349287	3	PAK1
chr15:68164730-68176654	12	PIAS1
chr12:42384939-42398994	89	PPHLN1
chr17:60633852-60648082	72	PPM1D
chr8:140761164-140800576	54	PTK2
chr17:2991296-3020595	28	RAP1GAP2
chr7:155700772-155718443	25	RBM33
chr7:155763811-155766655	10	RBM33
chr14:102714422-102721377	29	RCOR1
chr16:2262277-2263287	35	RNPS1
chr3:158122102-158123991	1559	RSRC1
chr6:130175840-130184623	16	SAMD3
chr6:70732377-70798737	8	SMAP1
chr1:245915319-245915640	9	SMYD3
chr12:49460769-49490731	141	SPATS2
chr8:47284027-47294030	532	SPIDR
chr9:88418777-88426591	4	SPIN1
chr14:35013345-35019074	6	SRP54
chr8:73595165-73709162	4	STAU2
chr11:59787036-59797396	14	STX3
chr11:61366044-61367998	406	TMEM138
chr7:66950951-66953369	67	TMEM248
chr18:670691-671451	24	TYMS
chr13:27089882-27105944	23	USP12
chr6:41871563-41891875	25	USP49
chrX:41123470-41123724	18	USP9X
chr8:99134631-99156743	33	VPS13B
chr15:28751487-28754897	4	WHAMMP2
chr9:37302187-37349452	21	ZCCHC7
chr2:20297552-20318645	4	PUM2
chr16:2317282-2319840	9	ABCA3
chr13:95161188-95170628	74	ABCC4
chr7:152800530-152825122	13	ACTR3B
chr2:157737270-157738570	5	ACVR1
chr5:65260599-65291470	208	ADAMTS6
chr12:111792060-111798242	9	ALDH2
chr10:27067156-27079161	9	ANKRD26
chr15:34900621-34904505	8	AQR
chr15:89903215-89910819	4	ARPIN
chr8:130236921-130358143	21	ASAP1
chr1:155395458-155415923	14	ASH1L

chr2:233256101-233265143	20	ATG16L1
chr13:32346826-32357929	8	BRCA2
chr22:49797804-49804360	23	BRD1
chr6:89846561-89855544	7	CASP8AP2
chr9:96562692-96565483	13	CDC14B
chr1:193130173-193147965	6	CDC73
chr9:120453455-120458622	9	CDK5RAP2
chr1:180003173-180006567	150	CEP350
chr8:67056518-67059445	9	COPS5
chr16:67610823-67621591	18	CTCF
chr7:101916114-101916507	13	CUX1
chr1:160237134-160240370	8	DCAF8
chr22:38494629-38501280	117	DDX17
chr9:123557569-123565648	13	DENND1A
chr15:65715477-65732818	3	DENND4A
chr3:154288865-154295339	4	DHX36
chr13:59861406-59916249	5	DIAPH3
chr10:68459103-68465812	12	DNA2
chr10:127031649-127061776	12	DOCK1
chr6:56900420-56900621	14	DST
chr6:15615266-15637804	106	DTNBP1
chr19:5787380-5787588	8	DUS3L
chr18:49331863-49391645	4	DYM
chr1:245002120-245083688	9	EFCAB2
chr6:52438303-52438591	7	EFHC1
chr22:36523208-36526131	18	EIF3D
chr2:46360637-46361090	15	EPAS1
chr19:45413676-45416897	7	ERCC1
chr12:56133587-56134428	17	ESYT1
chr22:29286922-29287134	12	EWSR1
chr11:73419873-73468492	15	FAM168A
chr12:31282715-31298224	3	FAM60A
chr9:120787768-120788278	11	FBXW2
chr12:120173652-120176217	3	GCN1
chr14:76166662-76176690	14	GPATCH2L
chr9:130075635-130092381	9	GPR107
chr9:112403994-112405722	26	HSDL2
chr5:138559863-138561789	59	HSPA9
chr7:23342063-23347734	8	IGF2BP3
chr15:41073427-41079904	20	INO80
chr15:41079700-41087682	6	INO80
chr5:62550366-62561257	92	IPO11
chr20:34489265-34507908	9	ITCH

chr6:17869045-17873437	3	KIF13A
chr1:10320042-10376676	29	KIF1B
chr13:113306484-113310867	23	LAMP1
chr6:79489083-79493750	9	LCA5
chr2:96733518-96738067	14	LMAN2L
chr15:30716219-30721271	7	LOC100288637
chr12:57498156-57504299	8	MARS
chr6:83223759-83253738	6	ME1
chr9:36597221-36633200	13	MELK
chr18:21815615-21847125	24	MIB1
chr1:66946149-66959743	11	MIER1
chr9:20353524-20365744	19	MLLT3
chr18:36220054-36260175	6	MOCOS
chr6:150877633-150885734	29	MTHFD1L
chr8:66572481-66593194	13	MYBL1
chr13:77205255-77217957	11	MYCBP2
chr2:191360630-191370292	16	MYO1B
chr8:95031994-95036494	10	NDUFAF6
chr5:53646232-53658624	33	NDUFS4
chr4:169602007-169602678	21	NEK1
chr6:138496218-138572061	8	NHSL1
chr1:169230717-169237687	50	NME7
chr1:169230717-169310080	10	NME7
chrX:71291830-71294340	4	nogene
chr5:77098654-77100391	29	nogene
chr19:46607865-46608140	6	nogene
chr7:66684351-66724454	12	nogene
chr21:10325780-10340478	3	nogene
chr7:66848488-66880864	3	nogene
chr14:21460507-21460723	67	nogene
chr4:97315664-97323287	6	nogene
chr2:24564294-24593721	57	nogene
chr1:246143821-246193963	26	nogene
chr1:155671353-155681140	14	nogene
chr7:2721272-2722138	11	nogene
chr5:143310096-143314168	8	NR3C1
chr10:79804484-79814716	8	NUTM2B-AS1
chr10:124408541-124412200	121	OAT
chr19:2271384-2271954	8	OAZ1
chr3:38183002-38190839	3	OXSR1
chr4:56448417-56448823	9	PAICS
chr3:52641953-52648442	33	PBRM1
chr2:10790718-10793202	21	PDIA6

chr2:196912881-196922186	48	PGAP1
chr20:35858301-35899648	10	PHF20
chr11:46084165-46092223	10	PHF21A
chr1:113710253-113712113	9	PHTF1
chr10:13280975-13288541	3	PHYH
chr11:85981744-85983973	31	PICALM
chr1:167332469-167376155	9	POU2F1
chr4:105424195-105446502	16	PPA2
chr17:60623520-60623749	11	PPM1D
chr1:202416786-202428929	31	PPP1R12B
chr18:9583116-9593874	5	PPP4R1
chr12:110551788-110551968	16	PPTC7
chr4:147653851-147661038	5	PRMT9
chr9:112275843-112288682	11	PTBP3
chr10:87864453-87952259	14	PTEN
chr8:140800458-140890769	71	PTK2
chr2:65090982-65098066	16	RAB1A
chr5:34762196-34796027	20	RAI14
chr18:70086612-70092804	28	RTTN
chr18:70109497-70128546	7	RTTN
chr19:47143493-47150375	4	SAE1
chr1:52974768-52980543	4	SCP2
chr3:47097954-47103423	79	SETD2
chr4:42022837-42049479	10	SLC30A9
chr10:100937401-100947847	6	SLF2
chr16:18834891-18838689	4	SMG1
chr12:49496832-49514613	11	SPATS2
chr1:15916127-15922349	4	SPEN
chr2:45546731-45574723	11	SRBD1
chr17:29648143-29650800	48	SSH2
chr20:44997168-45001353	3	STK4
chr14:52756550-52759754	24	STYX
chr21:32693171-32695282	14	SYNJ1
chr1:28617952-28622165	29	TAF12
chr17:82781588-82783714	98	TBCD
chr15:89584285-89585942	6	TICRR
chr3:129670329-129671264	7	TMCC1
chr8:55750963-55763980	3	TMEM68
chr1:223798214-223800047	12	TP53BP2
chr2:229840821-229860531	9	TRIP12
chr10:69483690-69485215	12	TSPAN15
chr9:33956078-33973237	16	UBAP2
chr6:42662183-42666245	4	UBR2

chr8:102346235-102361626	17	UBR5
chr9:6477621-6482099	36	UHRF2
chr10:11997671-12014184	449	UPF2
chr7:6111124-6146248	3	USP42
chrX:129919174-129919489	3	UTP14A
chr6:144473719-144474759	5	UTRN
chr8:99520898-99556653	9	VPS13B
chr8:99431536-99442635	10	VPS13B
chr14:54984722-54987387	25	WDHD1
chr1:224400949-224404570	7	WDR26
chrX:118396969-118404444	12	WDR44
chr4:1893729-1904378	12	WHSC1
chr2:61498864-61533903	3	XPO1
chr2:173169804-173191177	3	ZAK
chr16:88586599-88599948	4	ZC3H18
chr1:35388909-35390098	36	ZMYM4
chr7:100077302-100079656	8	ZNF3
chr20:33766850-33770292	9	ZNF341
chr19:11724193-11725327	13	ZNF823
chr5:61472680-61494410	54	ZSWIM6
chr15:71916369-71938927	7	MYO9A
chr20:9419806-9437152	5	PLCB4
chr1:151423396-151430841	14	POGZ
chr1:234457666-234472811	38	TARBP1
chr11:46811006-46821268	43	CKAP5
chr14:45095220-45097005	59	PRPF39
chr11:102162455-102206074	5	YAP1
chr12:1702885-1754514	78	ADIPOR2
chr2:23810315-23834078	3	ATAD2B
chr21:39215236-39225197	9	BRWD1
chr7:16681300-16689906	69	BZW2
chr20:3294138-3294629	25	C20orf194
chrX:108621802-108677633	14	COL4A5
chr16:68056363-68066636	11	DUS2
chr5:96912636-96915769	17	ERAP2
chr1:50539591-50596216	41	FAF1
chr11:110435833-110457047	3	FDX1
chr9:96465336-96465778	8	HABP4
chr2:190287585-190296953	3	HIBCH
chr19:38845616-38847434	11	HNRNPL
chr16:27340185-27345020	3	IL4R
chr2:206056197-206063244	8	INO80D
chr14:50462664-50486194	14	MAP4K5

chr2:159715680-159729175	20	7-Mar
chr17:62029540-62063301	17	MED13
chr10:68109673-68122340	9	MYPN
chr6:126522079-126637735	3	nogene
chr14:63391816-63396716	3	PPP2R5E
chr12:925449-930144	10	RAD52
chr12:124807760-124810289	12	SCARB1
chr14:102889559-102903429	23	TRAF3
chr17:623532-632841	10	VPS53
chr20:47224316-47229806	6	ZMYND8
chr16:75617141-75622464	5	ADAT1
chr7:32632542-32679130	50	DPY19L1P1
chr14:75622078-75624752	6	FLVCR2
chr2:53714383-53716743	142	GPR75-ASB3
chr7:139061035-139074030	17	ZC3HAV1
chr9:71870082-71875083	18	ABHD17B
chr2:64551442-64573068	4	AFTPH
chr8:6724869-6755174	9	AGPAT5
chr5:126561082-126570859	10	ALDH7A1
chr10:72196810-72210831	6	ASCC1
chr8:123333877-123337821	64	ATAD2
chr20:51617489-51619043	8	ATP9A
chr7:16674411-16682845	10	BZW2
chr7:16665436-16689906	35	BZW2
chr5:37245478-37247745	11	C5orf42
chr7:23611170-23622227	5	CCDC126
chr12:7367235-7379298	11	CD163L1
chr15:92924320-92939718	14	CHD2
chr7:43633278-43647634	10	COA1
chr7:87391588-87393067	19	CROT
chrX:119542504-119545504	12	CXorf56
chr1:85350414-85496281	7	DDAH1
chr11:9203659-9207632	107	DENND5A
chr17:43498861-43504825	14	DHX8
chr5:31483553-31486562	4	DROSHA
chr11:103222029-103223086	30	DYNC2H1
chr1:21001198-21089213	10	EIF4G3
chr8:690897-716007	5	ERICH1
chr5:137952627-138019146	3	FAM13B
chr5:138006989-138018514	15	FAM13B
chr5:74838964-74841679	96	FAM169A
chr11:72887469-72921950	5	FCHSD2
chr5:108946129-109047198	14	FER

chr22:36501240-36504766	9	FOXRED2
chr10:102358038-102359435	36	GBF1
chr12:109983372-109983694	6	GIT2
chr9:83753257-83768970	9	GKAP1
chr16:74459681-74463479	116	GLG1
chr8:43007846-43013400	25	HOOK3
chr2:238182064-238188257	8	ILKAP
chr12:26681873-26686632	8	ITPR2
chr10:77376009-77404023	17	KCNMA1
chr4:6842066-6858506	9	KIAA0232
chr9:5968018-5988545	9	KIAA2026
chr20:32316217-32316874	131	KIF3B
chr12:50430494-50440549	15	LARP4
chr5:126818921-126832801	10	LMNB1
chr5:142353225-142353583	8	LOC101926941
chr3:197870159-197875775	25	LRCH3
chr2:43943686-43948518	13	LRPPRC
chr16:199061-220747	6	LUC7L
chr6:89781398-89787957	8	MDN1
chr13:23836239-23841488	320	MIPEP
chr1:236835546-236863554	4	MTR
chr15:71999850-72010447	18	MYO9A
chr7:158692841-158693497	8	NCAPG2
chr16:69613898-69626528	12	NFAT5
chr19:39490497-39491703	11	nogene
chr11:77825902-77826149	23	nogene
chr7:100469149-100470401	8	nogene
chr15:45476078-45476552	10	nogene
chr8:133408173-133408387	30	nogene
chr13:24244838-24245617	3	nogene
chr9:97185441-97196594	12	nogene
chr3:15013588-15016254	9	NR2C2
chr11:117159933-117163892	93	PAFAH1B2
chr15:43769517-43770580	6	PDIA3
chr6:42966241-42966447	10	PEX6
chr20:35801490-35812719	26	PHF20
chr4:105386566-105399164	13	PPA2
chr14:29724469-29725674	14	PRKD1
chr8:47918276-47930012	9	PRKDC
chr6:31622726-31623909	9	PRRC2A
chr17:42838730-42839380	162	PSME3
chr4:86693586-86717117	10	PTPN13
chr1:29282675-29284869	17	PTPRU

chr20:34031521-34072330	13	RALY
chr1:235148403-235155112	11	RBM34
chr11:14294470-14295855	27	RRAS2
chr14:30715923-30734858	194	SCFD1
chr2:179172119-179229524	19	SESTD1
chr17:59088290-59104394	12	TRIM37
chr14:90695499-90786328	10	TTC7B
chr2:112485916-112520425	10	TTL
chr6:42676055-42679832	164	UBR2
chr6:144748245-144774364	6	UTRN
chr17:59764970-59811786	11	VMP1
chr2:63962057-63984019	20	VPS54
chr7:75057528-75058769	4	WBSCR16
chr4:84695969-84709347	10	WDFY3
chr2:127724332-127726777	9	WDR33
chr2:216190224-216194986	32	XRCC5
chr3:183715143-183724531	12	YEATS2
chr3:183715143-183718592	15	YEATS2
chr19:3651825-3667353	23	PIP5K1C
chr1:10256246-10261970	12	KIF1B
chr2:182952404-182957596	10	NCKAP1
chr16:53307680-53308854	29	CHD9
chr20:16427103-16429982	12	KIF16B
chr3:41896821-41911659	9	ULK4
chr17:57879610-57885879	3	CUEDC1
chr16:15873490-15884205	18	FOPNL
chr6:31412324-31507300	152	MICA
chr22:29988479-29991670	14	MTMR3
chr12:112446275-112457379	11	PTPN11
chr8:53984362-54000050	11	TCEA1
chr2:227491546-227536993	27	AGFG1
chr15:99808146-99877148	5	nogene
chr12:27714779-27735556	12	MRPS35
chr8:55782747-55796152	5	TGS1
chrX:80707426-80719656	172	BRWD3
chr4:51863436-51877854	695	DCUN1D4
chr8:140539319-140541358	9	AGO2
chr17:30803900-30804108	19	CRLF3
chr11:9160712-9170777	14	DENND5A
chr12:112486474-112489175	17	PTPN11
chr9:123179006-123184298	28	STRBP
chr17:48916075-48922937	20	UBE2Z
chr3:11347876-11380052	17	ATG7

chr22:32479132-32485209	21	FBXO7
chr5:33546058-33549383	10	ADAMTS12
chr2:202825694-202829016	10	ICA1L
chr7:66551524-66578727	9	nogene
chr1:28569908-28570342	9	nogene
chr10:46003933-46004526	7	nogene
chr17:76385654-76385962	10	SPHK1
chr2:45573206-45585774	11	SRBD1
chr9:113330983-113336440	10	WDR31
chr4:145885677-145903215	19	ZNF827
chr11:130126699-130135715	37	APLP2
chr2:197459931-197470171	11	COQ10B
chr12:108678271-108701323	140	CORO1C
chr11:43830975-43840064	21	HSD17B12
chr1:94732257-94743993	13	LINC01057
chr7:149018991-149021147	162	PDIA4
chr1:30941150-30942123	11	PUM1
chr19:651610-652884	49	RNF126
chr2:202513718-202520201	8	BMPR2
chr7:129657342-129690546	14	NRF1
chr5:40767465-40771863	3	PRKAA1
chr3:57831382-57864716	10	SLMAP
chr20:58418210-58439025	8	VAPB
chr10:74589281-74670269	21	ADK
chr15:72760406-72780322	61	ADPGK
chr12:50780139-50814439	4	ATF1
chrX:77616612-77620532	10	ATRX
chr12:111513356-111520076	37	ATXN2
chr12:32305663-32306122	7	BICD1
chr7:140749286-140754233	5	BRAF
chr1:230663140-230679052	20	COG2
chr17:63787033-63792562	4	DDX42
chr14:95132514-95141748	9	DICER1
chr3:132499125-132500913	15	DNAJC13
chrX:118654601-118654961	28	DOCK11
chr3:172769006-172786574	61	ECT2
chr3:47498285-47501851	11	ELP6
chr2:241407536-241434321	25	FARP2
chr5:151786602-151803996	12	G3BP1
chr9:124923094-124938866	24	GOLGA1
chr7:26196806-26197732	13	HNRNPA2B1
chr19:8468773-8474244	4	HNRNPM
chr1:169972512-169992255	8	KIFAP3

chr4:165219933-165299656	9	KLHL2
chr4:102664684-102674070	8	MANBA
chr1:53789132-53797144	25	NDC1
chr19:3425105-3435207	26	NFIC
chr3:104764378-104818695	3	nogene
chr10:91807141-91807445	24	nogene
chr18:14049774-14057515	14	nogene
chr1:113074816-113078735	10	nogene
chr11:83163678-83164401	14	PCF11
chr16:70010750-70018097	124	PDXDC2P
chrX:78122834-78125425	10	PGK1
chr8:140793353-140864399	8	PTK2
chr1:75790151-75790517	40	RABGGTB
chr6:34421829-34424668	8	RPS10
chr5:235143-236599	14	SDHA
chr10:7227854-7285954	15	SFMBT2
chr8:66813852-66831311	5	SGK3
chr14:50129960-50153173	3	SOS2
chr1:48395266-48403882	20	SPATA6
chr6:43178882-43179066	9	SRF
chr3:98756368-98757451	3	ST3GAL6
chr3:136357719-136363467	12	STAG1
chr6:33432160-33432806	26	SYNGAP1
chr17:29517452-29522519	18	TAOK1
chr6:133980081-133987168	9	TBPL1
chr11:12764178-12902113	8	TEAD1
chr2:170015280-170061443	14	UBR3
chr2:61339350-61339681	6	USP34
chr1:21747066-21751615	13	USP48
chr15:50432337-50459162	8	USP8
chr16:74923925-74951543	28	WDR59
chr6:2768690-2770361	10	WRNIP1
chr3:183710168-183728851	17	YEATS2
chr11:130260855-130288893	14	ZBTB44
chr3:179337168-179337431	9	nogene
chr20:25314924-25320318	13	ABHD12
chr2:232087486-232088040	26	DIS3L2
chr16:18841564-18850467	12	SMG1
chr2:9350807-9368519	192	ASAP2
chr12:26924354-26928905	4	ASUN
chr1:93588976-93592393	4	BCAR3
chr9:135850136-135881794	13	CAMSAP1
chr11:77616205-77629899	4	CLNS1A

chr6:37458611-37463008	47	CMTR1
chr14:22947600-22952683	4	HAUS4
chr15:41055246-41056706	36	INO80
chr17:16101249-16101805	52	NCOR1
chr15:90759501-90759701	8	nogene
chr2:44502688-44505730	16	nogene
chr1:151224247-151236763	23	PIP5K1A
chr7:99618524-99621574	51	ZSCAN25
chr10:68754713-68757377	10	CCAR1
chr3:132626690-132644896	20	ACAD11
chr1:37700435-37703347	27	CDCA8
chr16:3850296-3851009	840	CREBBP
chr1:20840855-20886371	10	EIF4G3
chr1:1804418-1817875	219	GNB1
chr21:33414887-33432871	24	IFNGR2
chr2:95834069-95834898	9	nogene
chr16:74660931-74664745	10	RFWD3
chr17:32171042-32176213	146	RHOT1
chr2:27668842-27677924	20	SLC4A1AP
chr21:15833347-15842540	85	USP25
chr5:113579585-113584479	7	YTHDC2
chr5:34175821-34182867	40	nogene
chr7:118185646-118188405	32	LSM8
chr3:128629950-128632157	10	RPN1
chr17:15545784-15547148	48	TVP23C-CDRT4
chr20:25020009-25023641	4	ACSS1
chr2:113931300-113942359	4	ACTR3
chr5:140509636-140510181	67	ANKHD1-EIF4EBP3
chr8:144580845-144581277	20	ARHGAP39
chr1:93567278-93567851	1098	BCAR3
chr5:179705678-179716294	27	CANX
chr11:47482694-47489024	24	CELF1
chr11:107326005-107336713	11	CWF19L2
chr4:53383772-53444103	19	FIP1L1
chr12:100591735-100612141	29	GAS2L3
chr16:67683379-67685802	11	GFOD2
chr2:196789203-196791460	24	GTF3C3
chr12:112175735-112176702	9	HECTD4
chr10:27173117-27181581	25	MASTL
chr5:139317052-139319501	50	MATR3
chr17:2438108-2464350	11	METTL16
chr16:47122656-47132025	18	NETO2
chr14:39654844-39655808	15	nogene

chr6:82057938-82059679	10	nogene
chr22:28547673-28550151	10	nogene
chr5:120672015-120673393	3	nogene
chr12:121228532-121229099	7	P2RX4
chr21:43004325-43021431	28	PKNOX1
chr2:86765026-86766024	45	RMND5A
chr8:42861744-42889414	4	RNF170
chrX:55729293-55731586	37	RRAGB
chr8:73673106-73709177	6	STAU2
chr17:63314387-63319090	19	TANC2
chr18:31897785-31909760	21	TRAPPC8
chr2:3338016-3354633	6	TSSC1
chr17:21004201-21004990	7	USP22
chr2:218234351-218239484	5	ARPC2
chr19:334399-336173	24	MIER2
chr1:805798-810170	48	nogene
chr3:196812218-196814568	48	PAK2
chr9:135482682-135484082	9	PPP1R26
chr2:30459551-30533314	11	LCLAT1
chr6:77211980-77273307	28	nogene
chr7:128335542-128336042	5	RBM28
chr4:41260646-41264161	8	UCHL1
chr2:40428474-40430304	10	SLC8A1
chr11:33328509-33341686	18	HIPK3
chr6:77151200-77151974	27	nogene
chr5:65291328-65334458	19	ADAMTS6
chr8:33372643-33389798	15	FUT10
chr4:41666560-41676462	30	LIMCH1
chrX:123633952-123639027	33	THOC2
chr19:19645873-19646347	49	ATP13A1
chr10:12081471-12097996	9	DHTKD1
chr1:92042822-92045660	29	EPHX4
chr19:45263112-45266281	50	MARK4
chr7:151564077-151570225	19	PRKAG2
chr5:146223402-146237432	30	RBM27
chr19:6026162-6040241	15	RFX2
chr1:41113282-41152741	6	SCMH1
chr12:116096668-116118129	3	MED13L
chr3:11807832-11817337	13	TAMM41
chr4:119596522-119607297	13	PDE5A
chr12:2920762-2922402	27	TULP3
chr12:44143524-44211387	48	TMEM117
chr10:102029637-102040125	17	C10orf76

chr2:232130618-232136719	33	DIS3L2
chr21:33432176-33432871	52	IFNGR2
chr2:238182064-238194870	98	ILKAP
chr19:35729158-35730462	12	KMT2B
chr12:68302619-68303913	19	MDM1
chr17:61984170-61987128	12	MED13
chr5:65704278-65708558	17	SGTB
chr17:18305111-18306966	32	TOP3A
chr7:76617965-76618390	15	nogene
chr2:36464533-36479694	42	CRIM1
chr1:23333517-23341020	11	HNRNPR
chr2:86024014-86024892	10	nogene
chr6:43029883-43030596	8	nogene
chr20:38057537-38066256	13	RPRD1B
chr1:168183901-168191500	10	TIPRL
chr15:89790459-89793126	13	ANPEP
chr2:61872081-61883548	23	CCT4
chr8:86528535-86532580	62	CPNE3
chr4:5663106-5665649	125	EVC2
chr5:108798123-108835807	15	FER
chr6:89678598-89680751	8	MDN1
chrX:40712913-40715220	21	MED14
chr7:66427948-66430017	11	nogene
chr1:19904266-19904987	19	OTUD3
chr2:74046277-74080591	15	TET3
chr16:21971524-21976243	19	UQCRC2
chr12:820496-827262	4	WNK1
chr14:23033367-23033674	268	PSMB5
chr10:123160406-123162828	3	BUB3
chr10:27039964-27043567	27	ANKRD26
chr2:131038852-131043583	15	ARHGEF4
chr1:155357315-155357749	19	ASH1L
chr6:97254556-97268002	16	MMS22L
chr17:47590716-47605552	25	NPEPPS
chr2:70051202-70085624	75	PCBP1-AS1
chr17:80837921-80857900	4	RPTOR
chr17:69273942-69284072	29	ABCA5
chr19:38152839-38164906	13	SIPA1L3
chr18:21536041-21564317	5	ESCO1
chr12:2873976-2874525	92	FOXM1
chr2:24739431-24742186	3	NCOA1
chr1:26941456-26941818	50	NUDC
chr1:88807312-88822003	15	PKN2

chr12:77966228-77968702	14	NAV3
chr5:14687520-14693094	11	OTULIN
chr10:3158913-3160330	54	PITRM1
chr3:134175608-134195182	339	RYK
chr15:43446386-43447485	9	TP53BP1
chr11:110136662-110163379	8	ZC3H12C
chr3:183866624-183868060	9	PARL
chr1:46678468-46691937	7	EFCAB14
chr9:122984484-122986419	10	RABGAP1
chr14:53091784-53103856	89	DDHD1
chr7:42040037-42048696	29	GLI3
chr15:40009613-40026089	6	EIF2AK4
chr12:6766685-6770848	9	nogene
chr6:73479731-73482620	23	MTO1
chr4:84765809-84766372	10	WDFY3
chr3:50065030-50066502	425	RBM6
chr14:60811986-60879835	20	MNAT1
chr2:173761578-173811253	11	nogene
chr20:46610445-46613725	5	SLC13A3
chr11:61321594-61323094	11	DDB1
chr5:180549930-180571432	9	CNOT6
chr15:65463760-65474288	12	DPP8
chr1:155906094-155906516	56	nogene
chr10:72741721-72742273	80	nogene
chr9:109087172-109093591	13	TMEM245
chr1:97691716-97721671	9	DPYD
chr2:32488587-32500109	33	BIRC6
chr2:28237201-28244862	11	BRE
chr5:137952627-137953465	3	FAM13B
chr8:47900372-47930012	9	PRKDC
chr3:47745907-47772936	19	SMARCC1
chr17:21019083-21028674	3	USP22
chr4:173795520-173856466	4	nogene
chr8:38295795-38299590	4	WHSC1L1
chr11:88309162-88312554	8	CTSC
chr15:64213889-64216713	10	CSNK1G1
chr11:73843246-73844976	36	MRPL48
chr10:68165701-68175461	24	MYPN
chr15:101701085-101702385	4	TARSL2
chr1:243199059-243225321	130	CEP170
chr5:138006989-138019146	30	FAM13B
chr1:240188206-240211235	100	FMN2
chr19:27797753-27798176	12	LOC101927151

chr5:37342548-37350265	24	NUP155
chr3:196806578-196812851	6	PAK2
chr1:27838698-27843330	20	PPP1R8
chr14:81485671-81490465	5	SEL1L
chr17:80222654-80227960	6	SLC26A11
chr6:99482752-99510230	7	USP45
chr19:39889478-39902837	11	FCGBP
chr6:85526125-85542043	7	SNX14
chr4:22401391-22402799	14	ADGRA3
chr22:38949111-38992149	9	APOBEC3B
chr2:207555627-207577655	38	CREB1
chr15:76285636-76292700	13	ETFA
chr5:108867766-108883518	45	FER
chr8:30607056-30635123	7	GTF2E2
chr3:149074214-149076047	10	HLTF
chr17:44933922-44934430	8	KIF18B
chr2:186081329-186086370	11	LINC01473
chr6:34341861-34343032	7	NUDT3
chr16:14580873-14593378	10	PARN
chr16:14997752-15030056	7	PDXDC1
chr20:62139074-62140944	690	PSMA7
chr12:929763-930144	45	RAD52
chr11:117279907-117280259	63	RNF214
chr2:45413113-45488331	9	SRBD1
chr2:9919040-9919820	6	TAF1B
chr21:37108391-37147603	13	TTC3
chr7:139251955-139266403	23	UBN2
chr17:1361091-1365058	32	YWHAE
chr1:52813535-52816629	8	ZYG11B
chr5:112992153-113004077	6	DCP2
chr14:60793043-60879835	10	MNAT1
chr12:106842588-106843947	203	RIC8B
chr14:50180571-50188700	7	SOS2
chr20:49151581-49154071	9	STAU1
chrX:123686547-123706949	9	THOC2
chr5:159272022-159275246	11	UBLCP1
chr5:80767932-80813741	19	MSH3
chr2:172558707-172562291	12	PDK1
chr5:132927120-132937740	6	AFF4
chr2:111802427-111803839	33	ANAPC1
chr19:46002982-46008301	3	CCDC61
chr12:28225794-28307749	19	CCDC91
chr6:47607926-47612536	38	CD2AP

chr1:227142944-227147559	4	CDC42BPA
chr1:160305432-160305773	8	COPA
chr8:96834972-96880005	25	CPQ
chr1:112412193-112449172	3	CTTNBP2NL
chr3:197194424-197194589	104	DLG1
chr19:2185854-2193783	10	DOT1L
chr14:102002536-102002965	30	DYNC1H1
chr3:172362632-172381093	18	FNDC3B
chr16:74496440-74508925	6	GLG1
chr16:1809753-1823037	5	HAGH
chr3:195685817-195689438	11	LINC00969
chr1:187254375-187329060	51	LINC01036
chr13:46711790-46733998	14	LRCH1
chr2:134250049-134254644	8	MGAT5
chr14:75095371-75105996	5	NEK9
chr19:57778749-57790398	8	nogene
chr8:16495792-16514838	21	nogene
chr8:100712361-100713181	53	PABPC1
chr3:52411028-52414587	20	PHF7
chr20:32215268-32216721	10	POFUT1
chrX:24821451-24888122	17	POLA1
chr6:35421819-35424779	4	PPARD
chr7:103361956-103364307	4	PSMC2
chr1:17422202-17425684	3	RCC2
chr1:176043185-176085890	23	RFWD2
chr9:91756070-91775818	11	ROR2
chr12:45931505-45934505	9	SCAF11
chr7:2269558-2275229	5	SNX8
chr1:48411463-48453131	9	SPATA6
chr18:12463350-12479871	8	SPIRE1
chr3:136500222-136502779	4	STAG1
chr18:192841-199316	13	USP14
chr11:61132419-61138835	4	VPS37C
chr14:96833466-96945382	8	VRK1
chr3:167554649-167560228	3	WDR49
chr12:46239678-46256769	4	SLC38A1
chr17:43104121-43104956	11	BRCA1
chr5:65521474-65542848	9	CENPK
chr19:5787378-5787588	3	DUS3L
chr1:28987430-28987905	16	EPB41
chr19:35729296-35729945	9	KMT2B
chr6:69737941-69752356	3	LMBRD1
chr2:46907809-46909177	32	MCFD2

chr14:50806736-50822077	10	NIN
chr6:141824165-141910011	18	nogene
chrX:78113743-78114160	3	PGK1
chr20:58271362-58272875	152	PPP4R1L
chr8:140793353-140890769	9	PTK2
chr1:176149005-176163891	81	RFWD2
chr3:47714414-47772936	24	SMARCC1
chr22:40312504-40316012	3	TNRC6B
chr10:11942664-11952249	11	UPF2
chr14:102325984-102326887	27	ZNF839
chr9:33528726-33529173	14	ANKRD18B
chr4:150193137-150224558	6	DCLK2
chr1:87072933-87092669	40	HS2ST1
chr11:94445809-94447438	405	MRE11A
chr10:49932099-49935142	15	PARG
chr9:132927200-132928952	5	TSC1
chr11:76888497-76926667	15	ACER3
chr1:202950929-202954307	11	ADIPOR1
chr7:141611194-141633980	9	AGK
chr6:32169469-32169965	7	AGPAT1
chr12:111781917-111785346	9	ALDH2
chr17:4194977-4197577	10	ANKFY1
chr17:4216982-4249248	6	ANKFY1
chr5:112775628-112792529	11	APC
chr20:48984728-48985613	9	ARFGEF2
chr16:24968350-24970580	75	ARHGAP17
chr17:68401359-68420506	12	ARSG
chr8:53756554-53772167	12	ATP6V1H
chr16:87975047-88006265	13	BANP
chr2:214767481-214792445	26	BARD1
chr2:32439507-32441462	34	BIRC6
chr17:67928354-67940656	29	BPTF
chr17:43051062-43063951	11	BRCA1
chr11:76513385-76516312	50	C11orf30
chr1:42773562-42775479	58	C1orf50
chr19:3620296-3620706	7	CACTIN
chr11:34079627-34083041	15	CAPRIN1
chr20:33606955-33629914	3	CBFA2T2
chr10:32471063-32474023	346	CCDC7
chr2:61873001-61880394	17	CCT4
chr10:124038514-124044919	17	CHST15
chr16:69155870-69163178	15	CIRH1A
chr11:46790075-46790583	43	CKAP5

chr3:33606590-33608626	5	CLASP2
chr3:33619602-33632374	37	CLASP2
chr6:99377385-99383824	13	COQ3
chr10:92143028-92181019	13	CPEB3
chr11:61411768-61429290	10	CPSF7
chr10:68941059-68943257	4	DDX50
chr17:56848695-56849232	9	DGKE
chr5:55274613-55281515	9	DHX29
chr1:225152004-225153826	9	DNAH14
chr1:21272698-21279332	6	ECE1
chr9:137728348-137758011	20	EHMT1
chr12:53037408-53038411	54	EIF4B
chr2:54928312-54928751	43	EML6
chr2:120127687-120146289	65	EPB41L5
chr1:44313168-44339322	10	ERI3
chr18:21539919-21566206	9	ESCO1
chr2:72465159-72480750	17	EXOC6B
chr3:56633043-56633966	10	FAM208A
chr10:50104041-50113997	5	FAM21A
chr10:45728861-45746647	18	FAM21C
chr16:87342876-87347250	109	FBXO31
chr5:73006444-73017308	18	FCHO2
chr17:41818088-41818527	43	FKBP10
chr1:240257944-240294883	13	FMN2
chr3:81699972-81705613	19	GBE1
chr17:36581343-36581538	11	GGNBP2
chr10:87053341-87062835	10	GLUD1
chr16:58708078-58709567	27	GOT2
chr10:67954594-67988835	13	HERC4
chr9:94415157-94441099	14	HIATL1
chr5:79439009-79451121	14	HOMER1
chr2:197490196-197495376	11	HSPD1
chr2:202785917-202811789	11	ICA1L
chr9:108922841-108929921	4	IKBKAP
chr15:41081019-41085583	17	INO80
chr12:30676497-30684457	9	IPO8
chr16:19733697-19735450	19	IQCK
chr16:47218867-47459175	8	ITFG1
chr5:138398177-138400012	27	KDM3B
chr12:122569680-122574380	16	KNTC1
chr14:50265357-50294246	18	L2HGDH
chrX:102839800-102865152	6	LINC00630
chr7:156796388-156836885	17	LMBR1

chr6:29063929-29065172	12	LOC100129636
chrX:3817544-3818500	99	LOC389906
chr16:55545734-55551102	11	LPCAT2
chr18:2951054-2960849	37	LPIN2
chr6:53878992-53904462	9	LRRC1
chr19:34194477-34215661	49	LSM14A
chr17:61984170-61985090	257	MED13
chr12:131838194-131841800	13	MMP17
chr10:93408786-93426158	13	MYOF
chr2:177231888-177234271	113	NFE2L2
chr10:94352309-94355093	9	NOC3L
chr4:34993201-35005121	8	nogene
chr11:28227994-28230985	13	nogene
chr1:72376422-72391566	8	nogene
chr5:171398707-171398915	12	nogene
chr3:106741560-106750905	8	nogene
chr4:163986497-163988709	5	nogene
chr11:122704781-122705172	3	nogene
chr4:26338840-26339362	8	nogene
chr3:15030274-15034809	3	NR2C2
chr10:18609844-18616293	34	NSUN6
chr8:109243150-109245481	12	NUDCD1
chr6:87601783-87606021	16	ORC3
chr22:42909426-42912157	15	PACSIN2
chr14:96542242-96565214	14	PAPOLA
chr13:24459043-24475596	12	PARP4
chr16:70130422-70132300	30	PDPR
chr4:39837855-39845880	9	PDS5A
chr3:170136418-170178938	94	PHC3
chr1:6619802-6620337	13	PHF13
chr16:47497398-47562495	12	PHKB
chr2:208276711-208288818	8	PIKFYVE
chr4:120799660-120821345	3	PRDM5
chr9:69732138-69741858	5	PTAR1
chr8:140761164-140803650	47	PTK2
chr8:140830471-140925749	18	PTK2
chr18:12825809-12831041	10	PTPN2
chr5:160422301-160427873	11	PTTG1
chr16:461403-471389	4	RAB11FIP3
chr1:51933317-51937412	4	RAB3B
chr8:52676368-52684013	8	RB1CC1
chr9:5732387-5738538	15	RIC1
chr17:1842802-1853189	583	RPA1

chr1:150446225-150446401	7	RPRD2
chrX:72273800-72276234	14	RPS4X
chr6:89372436-89387590	29	RRAGD
chr19:10224011-10224947	5	S1PR2
chr10:75150513-75164740	31	SAMD8
chr20:18527495-18542402	30	SEC23B
chr7:45744996-45759174	11	SEPT7P2
chr12:128814774-128815652	14	SLC15A4
chr6:87477361-87488102	10	SLC35A1
chr4:103066326-103067592	14	SLC9B2
chr17:76558373-76559043	48	SNHG16
chr20:17955364-17961326	20	SNX5
chr2:230461261-230474447	6	SP100
chr16:30728965-30734008	35	SRCAP
chr14:30950862-30956242	20	STRN3
chr9:112080073-112098662	17	SUSD1
chr8:119803877-119819506	4	TAF2
chr14:102376649-102414793	11	TECPR2
chr11:9286454-9299701	34	TMEM41B
chr7:128967279-129001234	8	TNPO3
chr7:5324213-5333049	20	TNRC18
chr8:140360049-140365686	10	TRAPPC9
chr17:59047682-59057054	10	TRIM37
chr3:3137259-3148187	8	TRNT1
chr17:21017941-21021226	51	USP22
chr2:61325374-61339681	27	USP34
chrX:41144521-41151057	10	USP9X
chr17:623532-631628	35	VPS53
chrX:118396969-118398470	7	WDR44
chr7:158901738-158906091	24	WDR60
chr17:27303366-27311616	21	WSB1
chr2:61481184-61484105	9	XPO1
chr16:28132333-28146203	3	XPO6
chr19:34929883-34933723	10	ZNF30
chr11:113741693-113744040	11	ZW10
chr1:147275353-147285487	82	CHD1L
chr3:197104902-197130671	5	DLG1
chr7:23123776-23125172	4	KLHL7
chr2:39331916-39356339	40	MAP4K3
chr6:138441982-138447193	10	NHSL1
chr17:42227528-42232137	5	STAT5B
chr7:140739811-140754233	16	BRAF
chr3:38499680-38506968	6	EXOG

chr6:31623731-31626162	20	PRRC2A
chr9:132346260-132349435	6	SETX
chr16:18828030-18834438	11	SMG1
chr16:3979115-3983440	98	ADCY9
chr10:72161537-72213331	10	ASCC1
chr10:121841081-121924329	9	ATE1
chr11:74118227-74133557	8	C2CD3
chr19:17559310-17560465	26	COLGALT1
chr5:134781132-134782086	46	DDX46
chr12:31451939-31460381	14	DENND5B
chr4:2596083-2631169	11	FAM193A
chr5:108798123-108798389	160	FER
chr5:74758848-74763766	9	GFM2
chr12:112203635-112204623	24	HECTD4
chr15:99671322-99690428	11	MEF2A
chr11:2976023-2979237	21	NAP1L4
chr10:92012328-92012686	10	nogene
chr16:2557702-2566453	204	PDPK1
chr16:14997752-15004333	12	PDXDC1
chr17:45120324-45121372	5	PLCD3
chr1:174220971-174305127	10	RABGAP1L
chr11:110263959-110264874	209	RDX
chr1:154965542-154966517	8	SHC1
chr15:50586391-50592626	36	TRPM7
chr1:22489879-22491533	135	ZBTB40
chr12:125114476-125118765	4	AACS
chr1:243613670-243637742	142	AKT3
chr5:126577078-126593404	26	ALDH7A1
chr19:32644324-32646615	9	ANKRD27
chr6:138333969-138335188	10	ARFGEF3
chr11:129093620-129164427	83	ARHGAP32
chr8:130214555-130236994	126	ASAP1
chr12:53531743-53537552	4	ATF7
chr5:71548681-71549606	15	BDP1
chr17:61715950-61744591	5	BRIP1
chr15:40196544-40202694	6	BUB1B
chr11:93747296-93759858	51	C11orf54
chr5:37170040-37175986	8	C5orf42
chr20:33606955-33611335	11	CBFA2T2
chr6:47595860-47606277	11	CD2AP
chrX:101101057-101109998	285	CENPI
chr20:35479230-35480145	20	CEP250
chr15:92953356-92967513	8	CHD2

chr16:58582792-58583182	60	CNOT1
chr9:17298189-17342444	36	CNTLN
chr16:58174450-58196844	4	CSNK2A2
chr1:113906995-113908191	8	DCLRE1B
chr11:118758773-118760044	24	DDX6
chr9:33029884-33030667	13	DNAJA1
chr17:82064254-82064822	4	DUS1L
chr10:73160433-73163950	6	ECD
chr5:50402285-50428227	4	EMB
chr5:464289-465845	10	EXOC3
chr6:70475409-70502791	10	FAM135A
chr10:73177845-73193593	9	FAM149B1
chr1:27732602-27734183	12	FAM76A
chr2:169530586-169544835	41	FASTKD1
chr15:75913202-75917394	10	FBXO22
chr16:70463168-70463774	106	FUK
chr5:151790322-151795575	33	G3BP1
chr5:162142163-162153372	12	GABRG2
chr17:36554819-36560871	17	GGNBP2
chr6:104744159-104785319	28	HACE1
chr2:37037854-37041292	9	HEATR5B
chr14:31133237-31133675	94	HECTD1
chr20:43635925-43642624	6	IFT52
chr6:149623102-149632916	11	KATNA1
chr5:141933258-141934586	11	KIAA0141
chr20:16494290-16515664	123	KIF16B
chr7:130116444-130122202	26	KLHDC10
chr18:6301902-6312056	351	L3MBTL4
chr6:108355653-108366332	15	LACE1
chr16:72349066-72391215	6	LINC01572
chr16:72522552-72555326	27	LINC01572
chr1:93324634-93340510	17	LOC100131564
chr9:85822879-85829606	27	LOC389765
chr1:113093205-113094755	9	LRIG2
chr5:56864733-56865977	37	MAP3K1
chr2:159729008-159762993	13	7-Mar
chr12:68816811-68828931	20	MDM2
chr15:99598429-99633173	6	MEF2A
chr9:36597221-36643083	122	MELK
chr9:130219724-130223159	8	NCS1
chr2:206132944-206142069	13	NDUFS1
chr10:15112795-15119513	8	NMT2
chr6:129556551-129558166	16	nogene

chr6:77072298-77151974	7	nogene
chr11:65435052-65437657	46	nogene
chr17:4888123-4888792	18	nogene
chr1:31045409-31045843	13	nogene
chr17:82403936-82406482	20	OGFOD3
chr1:233090060-233179175	4	PCNXL2
chr16:71676446-71714801	8	PHLPP2
chr20:34634614-34645334	12	PIGU
chr11:68505144-68545024	11	PPP6R3
chr3:48782985-48807684	14	PRKAR2A
chr2:20318536-20327378	3	PUM2
chr5:160089505-160094065	21	PWWP2A
chr2:1687631-1693134	9	PXDN
chr11:32966299-32973549	11	QSER1
chr10:118038971-118040565	19	RAB11FIP2
chr15:55223888-55270284	11	RAB27A
chr3:141572608-141577106	3	RASA2
chr16:74626342-74636577	11	RFWD3
chr2:226864603-226867318	164	RHBDD1
chr5:179966805-179980200	24	RNF130
chr11:10519033-10534205	10	RNF141
chr7:5715052-5725438	18	RNF216
chr1:213061381-213077816	9	RPS6KC1
chr5:78388836-78421960	47	SCAMP1
chr1:41046406-41075451	9	SCMH1
chr16:70565064-70567536	40	SF3B3
chr15:75422646-75430408	10	SIN3A
chr5:55343330-55358698	8	SKIV2L2
chr2:171826797-171844508	8	SLC25A12
chr1:27113825-27114286	9	SLC9A1
chr12:49460769-49461037	11	SPATS2
chr9:128612246-128613346	20	SPTAN1
chr4:56478378-56483270	53	SRP72
chr1:27789561-27793632	18	STX12
chr9:112149243-112157613	53	SUSD1
chr4:26613168-26640187	48	TBC1D19
chr15:66349059-66349437	33	TIPIN
chr13:113510237-113520733	41	TMCO3
chrX:110046356-110109146	8	TMEM164
chr2:229774096-229777479	5	TRIP12
chr7:67183125-67195337	18	TYW1
chr9:34234215-34242108	12	UBAP1
chr3:196362293-196369511	33	UBXN7

chr1:165890203-165896332	110	UCK2
chr9:77214328-77227485	74	VPS13A
chr16:46674313-46677398	341	VPS35
chr10:1077906-1105267	7	WDR37
chr12:64418045-64419094	9	XPOT
chr16:88586599-88611536	9	ZC3H18
chr8:134583831-134590355	15	ZFAT
chr1:35381258-35390098	18	ZMYM4
chr10:91994534-92008275	122	BTAF1
chr20:35655201-35658982	35	CPNE1
chr12:53018797-53019006	20	EIF4B
chr4:40978403-41014398	5	APBB2
chr9:92634385-92635308	5	IPPK
chr5:41731633-41739491	14	OXCT1
chr11:2347545-2347796	10	nogene
chr17:30785918-30796337	14	CRLF3
chr2:61010655-61011905	12	PUS10
chr1:176135009-176176007	38	RFWD2
chr2:203391045-203402734	25	ABI2
chr5:65291328-65334085	36	ADAMTS6
chr5:65931179-65994864	7	ERBB2IP
chr1:41186120-41194399	45	SCMH1
chr16:2937384-2938162	7	FLYWCH1
chr5:65329377-65473952	53	ADAMTS6
chr19:37759046-37771696	4	ZNF573
chr16:66469601-66469865	4	BEAN1
chr10:86912239-86919469	8	BMPR1A
chr10:101990550-102040125	4	C10orf76
chr9:2807813-2812362	4	KIAA0020
chr7:86938125-86947922	17	KIAA1324L
chr20:25496662-25498346	4	NINL
chr13:28174271-28220378	63	PAN3
chr8:140789473-140830526	127	PTK2
chr2:135675331-135680324	130	R3HDM1
chr1:176162868-176176007	87	RFWD2
chr7:92319329-92327900	26	ANKIB1
chr15:40602914-40611511	70	CASC5
chr15:39713443-39741900	15	FSIP1
chr3:155910691-155914570	36	GMPS
chr1:233353806-233362293	18	KIAA1804
chr12:12130782-12147556	8	LRP6
chr9:137039970-137040436	3	NPDC1
chr7:154946359-154948090	9	PAXIP1-AS2

chr2:135638738-135639122	3	R3HDM1
chr4:106295093-106308989	78	TBCK
chr14:93214928-93218735	3	UBR7
chr1:935771-939460	19	SAMD11
chr2:135090997-135115381	11	RAB3GAP1
chr8:103910320-103961133	8	RIMS2
chr21:39236594-39238573	15	BRWD1
chr5:179707131-179710065	32	CANX
chr6:43056080-43056445	4	MRPL2
chr16:1314019-1320517	33	UBE2I
chr7:93308823-93334197	14	VPS50
chr7:55492281-55497690	10	VOPP1
chr12:132741418-132755133	5	ANKLE2
chr11:118583808-118584644	32	ARCN1
chr1:155395458-155404350	10	ASH1L
chr6:83924469-83936376	7	CYB5R4
chr1:20969473-20981227	123	EIF4G3
chr5:112275325-112307490	17	EPB41L4A
chrX:63509943-63517082	52	LINC01278
chr3:197830769-197832317	4	LRCH3
chr17:62033786-62035608	80	MED13
chr9:36589535-36607673	17	MELK
chr2:219792376-219794170	42	nogene
chr13:28174271-28256539	11	PAN3
chr2:60781884-60783209	5	PAPOLG
chr7:10990702-11022979	30	PHF14
chr14:73147794-73186920	26	PSEN1
chr9:136823524-136832370	8	RABL6
chr2:37974039-38004216	16	RMDN2
chr3:47662333-47686170	7	SMARCC1
chr22:29511105-29519120	7	THOC5
chr12:101333300-101334504	31	UTP20
chr16:28125688-28132403	16	XPO6
chr1:155676547-155679512	249	YY1AP1
chr9:20819795-20867012	11	FOCAD
chr18:41481881-41501276	8	KC6
chr5:69285336-69303929	7	CCDC125
chr3:47179427-47179683	13	nogene
chr1:28553639-28571900	4	TRNAU1AP
chr3:81577924-81581275	14	GBE1
chr19:32625873-32643631	11	ANKRD27
chr11:78119181-78127436	50	ALG8
chr5:108782662-108798389	14	FER

chr10:101798841-101800400	29	MGEA5
chr15:70656929-70664814	14	UACA
chr11:93747301-93757465	6	C11orf54
chr10:68787926-68788328	21	CCAR1
chr10:68913161-68914204	39	DDX50
chr1:50787999-50857997	38	FAF1
chr5:150010110-150012353	8	HMGXB3
chr12:42373392-42398994	13	PPHLN1
chr3:195289120-195320813	5	ACAP2
chr2:222918045-222924595	23	ACSL3
chr5:140472238-140487060	7	ANKHD1
chr1:70300763-70315566	51	ANKRD13C
chr8:130159863-130187285	38	ASAP1
chr3:11340644-11426926	5	ATG7
chr21:41914597-41922085	70	C2CD2
chr9:71918424-71947112	15	C9orf85
chr1:223759269-223772239	30	CAPN2
chr10:35516522-35553185	49	CCNY
chr22:28694031-28696987	7	CHEK2
chr5:157791702-157794972	6	CLINT1
chr12:122352725-122380558	33	CLIP1
chr15:90619740-90625993	12	CRTC3
chr15:65752378-65756472	11	DENND4A
chr5:141580743-141583615	42	DIAPH1
chr19:2189731-2194577	47	DOT1L
chr16:67192760-67197646	8	E2F4
chr5:148394713-148417204	11	FBXO38
chr5:109037421-109047198	61	FER
chr9:19086733-19089559	20	HAUS6
chr15:72350517-72356617	8	HEXA
chr4:3154292-3157199	14	HTT
chr6:82214230-82214829	4	IBTK
chr7:128641237-128648982	17	LINC01000
chr3:183323234-183389779	12	MCF2L2
chr11:28296796-28362036	12	METTL15
chr14:21503172-21508235	9	METTL3
chr18:36195256-36220217	31	MOCOS
chr11:78559538-78571444	114	NARS2
chr5:36953617-36976402	124	NIPBL
chr5:65777426-65792655	14	NLN
chr19:47478500-47479152	8	nogene
chr7:32812756-32833164	10	nogene
chr19:36078358-36078607	6	nogene

chr5:93553760-93563463	63	NR2F1-AS1
chr10:18585948-18596327	9	NSUN6
chr7:92517275-92518255	29	PEX1
chr8:47927193-47930012	61	PRKDC
chr2:119900717-119934958	7	PTPN4
chr1:31005852-31007099	30	PUM1
chr1:174250474-174394145	9	RABGAP1L
chr12:925449-933076	19	RAD52
chr12:916343-916820	6	RAD52
chr20:34072065-34077245	228	RALY
chr20:37065423-37067297	16	RBL1
chr9:3395471-3420870	8	RFX3
chr15:76753807-76775117	15	SCAPER
chr14:30705822-30722559	16	SCFD1
chr14:30633946-30643405	34	SCFD1
chr4:75964134-75982037	50	SDAD1
chr10:119909040-119915889	12	SEC23IP
chr12:48071666-48074793	32	SENP1
chr3:47720665-47745993	119	SMARCC1
chr2:17731740-17745951	6	SMC6
chr15:64118116-64123546	96	SNX1
chr7:105203627-105206529	4	SRPK2
chr1:28605371-28622165	40	TAF12
chr18:26315228-26335231	62	TAF4B
chr14:20384984-20386195	4	TEP1
chrX:155524455-155545277	10	TMLHE
chr8:142330900-142345892	59	TSNARE1
chr17:60265411-60294801	40	USP32
chr4:84829003-84897010	4	WDFY3
chr10:1080010-1096246	21	WDR37
chr18:56717963-56781656	14	WDR7
chr9:86310705-86319670	4	ZCCHC6
chr9:37126311-37147445	23	ZCCHC7
chr20:25634728-25688835	8	ZNF337-AS1
chr5:37348504-37358151	19	NUP155
chr5:65567512-65572286	153	PPWD1
chr1:150334934-150335241	26	PRPF3
chr1:155709772-155717128	4	DAP3
chr1:59339957-59346398	79	FGGY
chr2:61284874-61295290	34	USP34
chr7:8003907-8060248	109	GLCCI1
chr17:29482196-29491865	12	TAOK1
chr11:46811006-46816404	6	CKAP5

chr17:60377093-60380399	18	nogene
chr20:32366383-32369123	2827	ASXL1
chr1:169978084-170031975	30	KIFAP3
chr2:31942720-31943383	12	MEMO1
chr11:3719411-3753408	9	NUP98
chr20:48974765-48976199	39	ARFGEF2
chr7:99388038-99390012	49	ARPC1B
chr1:93567278-93571841	14	BCAR3
chr7:6591645-6602140	9	C7orf26
chr1:64629354-64641969	8	CACHD1
chr3:14654425-14658929	6	CCDC174
chr17:66027500-66030023	14	CEP112
chr1:47368468-47375293	104	CMPK1
chr4:83056925-83068522	7	COPS4
chr14:69122216-69149503	76	DCAF5
chr9:123667025-123879021	26	DENND1A
chr1:225152004-225185425	65	DNAH14
chr1:15534236-15548428	39	DNAJC16
chr6:56578813-56600221	8	DST
chr7:6037423-6039035	13	EIF2AK1
chr10:119072889-119073937	130	EIF3A
chr1:21001198-21111383	27	EIF4G3
chr17:76094413-76101863	12	EXOC7
chrX:131793993-131794466	10	FIRRE
chr14:65561336-65629606	28	FUT8
chr1:28683582-28704329	6	GMEB1
chr16:58722149-58723902	47	GOT2
chrX:84321841-84344457	11	HDX
chr5:74715509-74716673	67	HEXB
chr1:20757165-20779911	18	HP1BP3
chr21:33246717-33248854	8	IFNAR2
chr12:30649136-30666251	7	IPO8
chr20:34470047-34481206	190	ITCH
chr8:125049005-125050665	21	KIAA0196
chrX:70296997-70302398	25	KIF4A
chr4:87170078-87176868	4	KLHL8
chr1:187321809-187329060	5	LINC01036
chr11:68403482-68406813	7	LRP5
chr12:68828770-68839801	12	MDM2
chr18:21765771-21768752	25	MIB1
chr7:24620051-24641868	15	MPP6
chr1:167772274-167788019	63	MPZL1
chr2:42707902-42709096	8	MTA3

chr8:70123883-70124865	8	NCOA2
chr16:68157868-68167015	24	NFATC3
chr19:3381711-3382243	59	NFIC
chr1:40747533-40753246	10	NFYC
chr1:169287302-169298763	13	NME7
chr7:135504930-135505293	9	nogene
chr2:131483963-131486181	10	nogene
chr22:28514756-28519320	93	nogene
chr12:11121009-11129363	9	nogene
chr7:5528571-5529201	9	nogene
chr1:51748368-51765981	12	OSBPL9
chr3:52668497-52679726	4	PBRM1
chr15:85123060-85126374	9	PDE8A
chr3:123089167-123124343	7	PDIA5
chr2:189791789-189818829	10	PMS1
chr9:122957010-122979040	4	RABGAP1
chr1:176135009-176149074	37	RFWD2
chr15:76567269-76574284	12	SCAPER
chr18:12951854-12955609	9	SEH1L
chr1:100911062-100921841	24	SLC30A7
chr15:75609557-75621056	6	SNUPN
chr8:47279269-47440542	9	SPIDR
chr5:179822957-179824323	19	SQSTM1
chr12:118400658-118414470	8	SUDS3
chr1:85190056-85190752	29	SYDE2
chr7:116249019-116252476	9	TES
chr9:69236027-69237136	8	TJP2
chr6:158580939-158585425	5	TMEM181
chr9:120923704-120926688	7	TRAF1
chr9:132921362-132935095	6	TSC1
chr9:135945027-135947905	5	UBAC1
chr2:23958300-23984831	49	UBXN2A
chr2:28912589-28914208	13	WDR43
chr16:28166507-28181031	7	XPO6
chr16:87417459-87418901	6	ZCCHC14
chr10:87151349-87152879	10	FAM35A
chr20:50903888-50928825	8	ADNP
chr6:100589633-100607088	4	ASCC3
chrX:77663381-77664778	43	ATRX
chr20:5700744-5773043	11	C20orf196
chr1:100424221-100442996	42	CDC14A
chr9:120408346-120409316	4	CDK5RAP2
chr2:211619176-211630594	23	ERBB4

chr10:68337198-68339555	8	HNRNPH3
chr3:185672540-185675913	9	IGF2BP2
chr10:124496960-124517271	18	LHPP
chr12:8390269-8397120	4	LINC00937
chrX:3817544-3818703	43	LOC389906
chr11:122116132-122180398	13	MIR100HG
chr4:139349461-139354098	10	NAA15
chr3:196270414-196283538	83	PCYT1A
chr4:120811369-120821345	57	PRDM5
chr15:78544189-78546698	11	PSMA4
chr14:30649527-30675065	20	SCFD1
chr22:31580271-31602785	12	SFI1
chr3:27437387-27452498	12	SLC4A7
chr16:68859883-68909402	9	TANGO6
chr2:241601895-241606473	107	THAP4
chr7:23505745-23522432	12	TRA2A
chr2:84832054-84842007	8	TRABD2A
chr19:34450264-34467014	53	UBA2
chr11:11880176-11897693	8	USP47
chr4:201080-212781	16	ZNF718
chr11:123594081-123594838	4	GRAMD1B
chr21:31958842-31983609	3	HUNK
chrX:21878041-21878692	15	MBTPS2
chr6:87603383-87606021	3	ORC3
chr3:138742557-138755895	3	PIK3CB
chr2:119932423-119952129	3	PTPN4
chr1:147255812-147268878	5	CHD1L
chr5:138810037-138827718	39	CTNNA1
chr10:100256261-100262063	18	CWF19L1
chr12:50692948-50697175	5	DIP2B
chrX:24064200-24071727	27	EIF2S3
chr12:11839139-11869969	9	ETV6
chr9:111417882-111433372	8	KIAA0368
chr8:94537102-94543942	8	KIAA1429
chr12:51053811-51056502	6	LETMD1
chr3:151156160-151165982	8	MED12L
chr1:178737107-178741609	7	nogene
chrX:81324575-81328708	4	nogene
chr2:40160764-40177855	68	SLC8A1
chr10:31355143-31461237	12	ZEB1
chr9:65682765-65693473	210	CBWD5
chr1:108657450-108659962	24	HENMT1
chr17:72841757-72849804	29	SLC39A11

chr2:241456746-241475987	10	FARP2
chr6:24538935-24539680	5	nogene
chr11:75961443-75983513	18	UVRAG
chr5:154865191-154872651	58	CNOT8
chr4:88906378-88938241	11	FAM13A
chr10:6213622-6217191	11	PFKFB3
chr2:199348700-199433514	33	SATB2
chr8:99442400-99521010	6	VPS13B
chr16:16009775-16016621	10	ABCC1
chr8:130152735-130187285	30	ASAP1
chr19:41248513-41248948	11	AXL
chr2:44372715-44505730	3	CAMKMT
chr4:109659010-109664394	91	CCDC109B
chr1:37695909-37703347	9	CDCA8
chr19:32899856-32901412	73	CEP89
chr22:28703504-28725367	3	CHEK2
chr4:55453053-55456300	40	CLOCK
chr2:171448680-171458477	10	DCAF17
chr4:51863436-51891851	637	DCUN1D4
chr1:225079206-225097239	46	DNAH14
chr3:172786492-172807924	21	ECT2
chr10:15248638-15254879	7	FAM171A1
chr7:208897-209384	9	FAM20C
chr1:77783237-77843407	17	FAM73A
chr12:116945363-116949706	37	FBXW8
chr9:20885108-20916937	11	FOCAD
chr12:110455585-110457634	72	GPN3
chr19:35019190-35019533	9	GRAMD1A
chr7:65974294-65980409	11	GUSB
chr10:68014025-68073686	30	HERC4
chr12:68302619-68302872	25	MDM1
chr13:23836239-23870195	33	MIPEP
chr8:120459214-120488332	14	MTBP
chr8:97686667-97689103	25	MTDH
chr10:31373017-31387266	413	nogene
chr14:35002261-35002527	21	nogene
chr14:45096423-45096758	5	nogene
chrX:65366637-65398704	3	nogene
chr17:81604547-81608822	75	NPLOC4
chr10:13108961-13109288	6	OPTN
chr6:79015081-79060818	6	PHIP
chr1:3186124-3244137	6	PRDM16
chr6:57532410-57537625	146	PRIM2

chr6:128304667-128322310	10	PTPRK
chr14:30702295-30715977	9	SCFD1
chr4:128992166-129044051	9	SCLT1
chr6:75622750-75634811	15	SENP6
chr7:94598774-94629841	17	SGCE
chr3:47720665-47736126	8	SMARCC1
chr16:18849216-18850467	40	SMG1
chr19:16647095-16654125	14	SMIM7
chr16:69245601-69270285	43	SNTB2
chr2:181921947-181928278	35	SSFA2
chr17:31940285-31976614	59	SUZ12
chr17:4282797-4289408	18	UBE2G1
chr1:7777159-7778169	930	VAMP3
chr19:52314152-52315962	8	ZNF480
chrX:63665885-63678572	18	ARHGEF9
chr7:22961234-22991139	12	FAM126A
chr3:58145920-58150227	4	FLNB
chr8:43059091-43077364	33	FNTA
chr1:230236013-230249271	4	GALNT2
chr14:22947170-22950410	23	HAUS4
chr8:135542653-135607037	4	KHDRBS3
chr22:36285356-36285657	4	MYH9
chr13:41316845-41336756	18	NAA16
chr5:154823198-154824092	10	nogene
chr2:45840379-45860198	26	nogene
chr19:56378604-56379370	4	nogene
chr17:81909055-81909513	4	PCYT2
chr14:77296163-77306441	16	POMT2
chr1:51933317-51977117	4	RAB3B
chr12:121416158-121423517	9	RNF34
chr6:42603587-42612291	128	UBR2
chr1:21748137-21751615	26	USP48
chrX:118398386-118404444	10	WDR44
chr5:69252417-69262304	42	CDK7
chr16:66622061-66623502	5	CMTM4
chr1:92736397-92738040	6	EVI5
chr18:13681604-13682105	352	FAM210A
chr3:172112451-172251541	18	FNDC3B
chr17:81693653-81694853	178	HGS
chr20:34470047-34492597	109	ITCH
chr15:30658765-30750556	11	LOC100288637
chr5:80725452-80761678	34	MSH3
chr1:51823663-51834170	103	NRD1

chr7:6002452-6004058	12	PMS2
chr8:140874462-140890769	12	PTK2
chr7:105459167-105470848	10	PUS7
chr14:39074414-39087008	7	SEC23A
chr6:158583953-158608750	15	TMEM181
chr3:126621741-126647296	53	TXNRD3
chr2:71355718-71370005	50	ZNF638
chr10:73571420-73576361	47	USP54
chr14:51689690-51715499	20	FRMD6
chr4:150735257-150848998	7	LRBA
chr12:69251128-69253154	21	CPSF6
chr18:21536041-21568094	77	ESCO1
chr6:1726415-1742586	29	GMDS
chr2:239176412-239352918	13	HDAC4
chr3:124841382-124873531	11	ITGB5
chr1:36170970-36171581	10	MAP7D1
chr11:73844806-73860009	4	MRPL48
chr11:64197270-64197974	40	STIP1
chr6:158028755-158043399	8	SYNJ2
chr2:61259710-61266167	9	USP34
chr2:62222211-62225414	24	B3GNT2
chr4:83581561-83590292	9	GPAT3
chr11:19832483-19880127	39	NAV2
chr22:29654656-29665064	7	NF2
chr4:139043073-139048147	9	NOCT
chr7:24816609-24849176	61	OSBPL3
chr15:82404611-82408367	20	UBE2Q2P2
chrX:155898111-155919880	9	VAMP7
chr1:6222395-6223195	8	nogene
chr4:82878729-82880922	36	SEC31A
chr5:31464235-31472232	24	DROSHA
chr1:77801330-77864028	12	FAM73A
chr15:70980100-70984257	27	LRRC49
chr2:190670325-190673152	10	NAB1
chr9:137265876-137267346	17	NELFB
chrX:131344812-131364926	30	nogene
chr22:41636112-41637791	21	XRCC6
chr15:90947795-90948794	11	UNC45A
chr21:39258486-39274472	11	BRWD1
chr3:49368427-49375591	17	RHOA
chr5:140438502-140445975	10	ANKHD1-EIF4EBP3
chr12:110012004-110019328	18	ANKRD13A
chrX:23856301-23874457	4	APOO

chr11:72709869-72714321	29	ARAP1
chr10:24629995-24635103	37	ARHGAP21
chr6:100766564-100767345	37	ASCC3
chr14:34800223-34802988	11	BAZ1A
chr22:49794033-49804360	5	BRD1
chr11:66828265-66828943	15	C11orf80
chr6:17462995-17507726	19	CAP2
chr11:47483452-47484523	38	CELF1
chr5:98868494-98872555	24	CHD1
chr7:101916114-102115273	15	CUX1
chr10:68936935-68941194	17	DDX50
chrX:2243022-2291603	5	DHRSX
chr10:440870-486530	16	DIP2C
chr8:108449822-108450492	19	EMC2
chr12:1110191-1116033	27	ERC1
chr16:53810139-53934109	8	FTO
chr4:75655065-75662049	27	G3BP2
chr9:127291167-127325095	6	GARNL3
chr10:130145210-130175089	5	GLRX3
chr14:76171842-76176690	21	GPATCH2L
chr7:18644670-18666476	10	HDAC9
chr6:15246380-15374252	11	JARID2
chr6:72647931-72677214	9	KCNQ5-IT1
chr12:50454313-50467120	10	LARP4
chr12:12148941-12151038	8	LRP6
chr2:159743060-159745937	7	7-Mar
chr5:69419370-69424636	53	MARVELD2
chr2:15461200-15558634	5	NBAS
chr12:6509716-6511252	87	NCAPD2
chr4:177351379-177353728	15	NEIL3
chr16:69656188-69656546	10	nogene
chr17:74989909-74991127	5	nogene
chr1:152248894-152251758	32	nogene
chr4:23103586-23186098	17	nogene
chr9:111009147-111011690	30	nogene
chr1:229450524-229465519	7	NUP133
chr1:228213440-228215098	9	OBSCN
chr17:78414951-78419725	4	PGS1
chr14:49664625-49674427	78	POLE2
chr10:70209204-70209685	8	PPA1
chr8:140761164-140864399	10	PTK2
chr18:36026899-36040865	9	RPRD1A
chr6:75621531-75634811	158	SENP6

chr9:132296886-132300803	16	SETX
chr10:100915998-100918441	18	SLF2
chr3:47670657-47678311	11	SMARCC1
chr3:47738028-47772936	35	SMARCC1
chr15:44569397-44572820	9	SPG11
chr9:88426381-88448989	11	SPIN1
chr17:63713405-63714108	8	STRADA
chr10:69198371-69203043	10	SUPV3L1
chr14:69658512-69658713	8	SUSD6
chr8:119801793-119831721	55	TAF2
chr16:68859883-68863061	106	TANGO6
chr10:94474667-94522453	12	TBC1D12
chr3:63834062-63839773	17	THOC7
chr7:130173631-130178527	36	TMEM209
chr7:5345561-5352094	24	TNRC18
chr5:14293011-14336727	29	TRIO
chr2:229829192-229836984	38	TRIP12
chr15:70682757-70699660	58	UACA
chr1:162576776-162601514	14	UAP1
chr9:33960825-33973237	246	UBAP2
chr1:154234590-154246375	6	UBAP2L
chr6:34821652-34823348	15	UHRF1BP1
chr3:41715236-41717861	50	ULK4
chr18:196636-204692	122	USP14
chr10:73505016-73505307	4	USP54
chr2:106122969-106145370	19	UXS1
chr8:99134631-99170163	6	VPS13B
chr1:12415086-12416827	10	VPS13D
chr14:63598755-63599973	79	WDR89
chr1:32806481-32811057	9	YARS
chr6:87210450-87218731	347	ZNF292
chr2:48328516-48346751	42	FOXN2
chr5:79447052-79457018	12	HOMER1
chr20:32825924-32839856	15	MAPRE1
chr5:87372117-87374916	12	RASA1
chr16:29554415-29560215	4	SMG1P2
chr19:34431860-34467014	53	UBA2
chr12:118095532-118095814	14	VSIG10
chr17:58003650-58005041	63	nogene
chr1:246551212-246557534	10	TFB2M
chr20:33619516-33628435	81	CBFA2T2
chr1:235341707-235401587	15	GGPS1
chr1:35841164-35851053	8	AGO4

chr20:51617489-51629072	10	ATP9A
chr15:44328684-44338317	24	CASC4
chr3:123931101-123949132	21	CCDC14
chr12:122777490-122781330	74	CCDC62
chr21:37472683-37496258	20	DYRK1A
chr10:31355143-31387266	23	nogene
chr9:131159382-131164144	15	NUP214
chr11:34947505-34984728	19	PDHX
chr16:2566355-2583585	14	PDPK1
chr5:133980728-133980956	24	VDAC1
chr7:5199575-5222672	8	WIPI2
chr6:36894503-36902434	34	C6orf89
chr1:40064226-40064559	69	CAP1
chr17:81206743-81207239	7	CEP131
chr8:42861744-42874006	25	RNF170
chr19:37643264-37654879	7	ZFP30
chr14:54981539-55000992	15	WDHD1
chr8:23042040-23043243	125	TNFRSF10B
chr11:72887469-72902638	38	FCHSD2
chr4:82970326-82984876	4	LIN54
chr7:1957628-2002121	28	MAD1L1
chr19:45258988-45263367	13	MARK4
chr19:38081255-38088851	11	SIPA1L3
chr12:98537474-98547671	13	TMPO
chr5:176975395-176982623	192	UIMC1
chr6:30650993-30651466	438	C6orf136
chr1:225002782-225023865	9	DNAH14
chr14:22947600-22955176	15	HAUS4
chr1:170031885-170055436	12	KIFAP3
chr3:51260155-51271007	12	DOCK3
chr13:77126317-77129230	48	MYCBP2
chr1:235435744-235437474	7	TBCE
chr18:6973056-6980637	8	LAMA1
chr8:140221458-140252929	27	TRAPPC9
chr1:244418759-244432595	3	ADSS
chr12:19493799-19514784	4	AEBP2
chr2:227519947-227524915	26	AGFG1
chr3:105520066-105534845	4	ALCAM
chr10:97246271-97263629	27	ARHGAP19
chr11:129062279-129124894	8	ARHGAP32
chr15:72561482-72572165	9	ARIH1
chr8:61618977-61646878	33	ASPH
chr12:123733925-123737271	17	ATP6V0A2

chr1:235465635-235470960	17	B3GALNT2
chr1:235470849-235484515	50	B3GALNT2
chr16:75242469-75243090	10	BCAR1
chr6:80343663-80354354	26	BCKDHB
chr17:67891843-67893725	4	BPTF
chr5:134741062-134744052	573	CAMLG
chr16:2439352-2443800	18	CCNF
chr10:84380097-84438707	9	CCSER2
chr14:80478253-80530886	6	CEP128
chr2:201129728-201133134	25	CFLAR
chr8:96879797-96902096	5	CPQ
chr5:140194445-140194652	271	CYSTM1
chr9:123671290-123879021	4	DENND1A
chr3:197085579-197091026	7	DLG1
chr19:10180349-10182077	13	DNMT1
chr2:25274940-25275543	7	DNMT3A
chr5:31429474-31451640	14	DROSHA
chr20:46393092-46394503	41	ELMO2
chr2:26364301-26375148	115	EPT1
chr18:21560858-21568094	21	ESCO1
chr12:22643762-22673660	334	ETNK1
chr1:27988534-28004419	21	EYA3
chr10:15816834-15847943	634	FAM188A
chr4:158825915-158835476	10	FNIP2
chr6:1930102-1952129	6	GMDS
chr2:53728711-53765585	9	GPR75-ASB3
chr6:134986065-134993875	15	HBS1L
chr7:27629370-27649633	237	HIBADH
chr1:20744743-20773610	11	HP1BP3
chr4:3099273-3107423	21	HTT
chr4:6824184-6842204	33	KIAA0232
chr7:152238706-152250966	13	KMT2C
chr4:82946257-82970469	10	LIN54
chr9:127467739-127473931	19	LRSAM1
chr2:39272282-39288280	8	MAP4K3
chr14:50442731-50448832	6	MAP4K5
chr18:50895808-50908196	9	ME2
chr8:97686667-97706750	7	MTDH
chr10:68201828-68210547	8	MYPN
chr3:161238112-161247330	8	NMD3
chr10:15104936-15112963	5	NMT2
chr7:44481291-44485622	33	nogene
chr7:2720115-2721550	6	nogene

chr7:66740156-66745009	17	nogene
chr1:151202999-151203275	11	nogene
chr6:138645754-138646807	9	nogene
chr3:129838897-129880562	5	nogene
chr9:128472912-128473741	13	ODF2
chr8:100709132-100712789	7	PABPC1
chr15:77283882-77286504	6	PEAK1
chr16:47487459-47515601	4	PHKB
chr12:19253939-19265850	34	PLEKHA5
chr22:41583958-41584603	78	PMM1
chr7:75474703-75475188	131	POM121C
chr8:98123335-98128540	15	POP1
chr12:42374862-42375074	7	PPHLN1
chr18:9583116-9584828	10	PPP4R1
chr18:9588088-9595153	47	PPP4R1
chr8:140789473-140819020	138	PTK2
chr8:140800458-140879637	28	PTK2
chr12:121780871-121781192	36	RHOF
chr13:23353784-23375269	9	SACS
chr16:46617633-46618372	27	SHCBP1
chr11:124647364-124648175	23	SIAE
chr10:68493131-68493570	5	SLC25A16
chr1:100913662-100974902	9	SLC30A7
chr1:99993536-100015420	21	SLC35A3
chr3:125453809-125460860	6	SNX4
chr17:28591697-28593066	42	SPAG5
chr17:20245925-20260294	170	SPECC1
chr3:113488944-113499582	95	SPICE1
chr8:47279269-47294030	8	SPIDR
chr3:136443286-136473637	9	STAG1
chr8:73673106-73739866	8	STAU2
chr1:47287550-47295848	20	STIL
chr3:67495797-67529186	18	SUCLG2
chr17:82756164-82800996	31	TBCD
chr17:62552301-62565137	106	TLK2
chr2:97772296-97776018	4	TMEM131
chr9:109080838-109087342	39	TMEM245
chr2:112074911-112086104	11	TMEM87B
chr15:42950261-42970607	11	UBR1
chr1:19192187-19192540	31	UBR4
chr3:41705056-41754488	10	ULK4
chr17:60288522-60301704	23	USP32
chr2:63313247-63439871	20	WDPCP

chr22:40868998-40926463	7	XPNPEP3
chr2:61482379-61490776	86	XPO1
chr14:88575839-88578140	17	ZC3H14
chr6:157592987-157595723	40	ZDHHC14
chr10:42631928-42632439	4	ZNF33B
chr19:58260850-58263271	16	ZNF544
chr19:29817136-29817541	4	CCNE1
chr6:87555407-87569590	17	RARS2
chr3:149520830-149527969	5	WWTR1
chr2:232735154-232761436	115	GIGYF2
chr1:247159005-247159813	742	ZNF124
chr8:140864311-140879637	36	PTK2
chrX:153702006-153704094	11	BCAP31
chr1:32228498-32229208	25	EIF3I
chr5:131670462-131679175	6	FNIP1
chr11:20364236-20367281	7	HTATIP2
chr6:161080880-161084617	12	MAP3K4
chr10:48325896-48410168	9	MAPK8
chr12:64062916-64115893	13	SRGAP1
chr12:128800853-128815070	8	SLC15A4
chr10:86912239-86917326	27	BMPR1A
chr9:107300140-107311737	602	RAD23B
chr2:201145377-201160942	10	CFLAR
chr19:10163325-10177367	48	DNMT1
chr17:44110728-44117704	23	HDAC5
chr12:112075677-112081134	16	NAA25
chr5:50750150-50763242	108	PARP8
chr5:150695505-150699271	11	RBM22
chr6:43026036-43027477	5	RRP36
chr7:6135840-6140984	9	USP42
chr1:167966628-167975015	295	DCAF6
chr5:146159406-146164471	8	LARS
chr17:78190969-78192785	7	AFMID
chr12:110016387-110025823	7	ANKRD13A
chr10:91942421-91956657	8	BTAF1
chr2:201145377-201164084	9	CFLAR
chr15:82219651-82241403	8	EFTUD1
chr5:108186116-108348530	211	FBXL17
chr9:105615277-105620061	5	FKTN
chr6:34132977-34133859	33	GRM4
chr10:67966682-68038169	15	HERC4
chr3:122752590-122777906	21	HSPBAP1
chr17:61921724-61925099	117	INTS2

chr20:34413741-34442303	17	ITCH
chr1:23030468-23040434	28	KDM1A
chr3:183057310-183092545	8	MCCC1
chr21:46264116-46272829	4	MCM3AP
chr5:36207169-36227565	13	NADK2
chr7:66407289-66439304	19	nogene
chr7:85012781-85022613	8	SEMA3D
chr3:14443623-14447816	136	SLC6A6
chr7:117129792-117138532	6	ST7
chr12:98544229-98546447	11	TMPO
chr5:72889785-72893703	6	TNPO1
chr21:44077692-44080127	58	TRAPPC10
chr6:89333085-89343756	10	UBE2J1
chr8:86442618-86452679	4	WWP1
chr7:105268805-105297533	7	SRPK2
chr3:128246835-128264781	14	EEFSEC
chr2:71401955-71418639	13	ZNF638
chr2:127755383-127771004	10	WDR33
chr4:185247293-185323800	4	SNX25
chr17:56847921-56856625	14	DGKE
chr9:137754170-137782397	11	EHMT1
chr15:49235850-49283718	61	GALK2
chr22:23714186-23715194	20	GUSBP11
chrX:70290437-70302398	52	KIF4A
chr12:107699372-107710510	15	PWP1
chr6:24502522-24528166	5	ALDH5A1
chr19:41220635-41222056	11	AXL
chr10:86912239-86921695	10	BMPR1A
chr7:82335134-82410611	5	CACNA2D1
chr8:25298943-25310532	20	DOCK5
chr21:33579349-33583666	10	DONSON
chr5:137952627-137954376	159	FAM13B
chr6:82225476-82231842	35	IBTK
chrX:153789711-153790851	32	IDH3G
chr12:30665219-30666251	5	IPO8
chr7:149233014-149233369	8	nogene
chr17:45538321-45538589	14	nogene
chr1:173980808-173993135	8	RC3H1
chr3:47661293-47686170	8	SMARCC1
chr17:64578491-64580991	15	SMURF2
chr15:44569397-44570658	18	SPG11
chr7:138504289-138515359	17	TRIM24
chr10:86890061-86892229	37	BMPR1A

chr17:66096250-66183307	14	CEP112
chr1:168038370-168068463	12	DCAF6
chrX:131783886-131794466	12	FIRRE
chr10:13659322-13675044	6	FRMD4A
chr12:132720677-132721217	56	PGAM5
chr13:114052051-114073837	57	RASA3
chr6:35857260-35870959	14	SRPK1
chr6:25963048-25963282	5	TRIM38
chr10:37775343-37776575	12	ZNF248
chr1:120530295-120557127	8	nogene
chr6:33398028-33398387	30	KIFC1
chr19:879936-891149	10	MED16
chr11:70330172-70335694	38	PPFIA1
chr12:121335532-121347005	15	ANAPC5
chr8:130214555-130290138	5	ASAP1
chr22:24095930-24134415	25	CABIN1
chr10:68430435-68470163	8	DNA2
chr4:53383772-53399839	68	FIP1L1
chr1:117881822-117920424	12	GDAP2
chr8:43146947-43161507	14	HGSNAT
chr4:3086942-3107423	5	HTT
chr15:41085368-41087682	142	INO80
chr1:236850343-236853088	33	MTR
chr11:119009380-119009891	25	nogene
chr1:28473553-28474151	68	PHACTR4
chr3:142669389-142671122	6	PLS1
chr12:132680606-132681279	13	POLE
chr4:6347845-6381094	16	PPP2R2C
chr17:59193491-59197789	20	PRR11
chr22:41268096-41281082	123	RANGAP1
chr11:18502485-18519603	13	TSG101
chr17:19924407-19963022	14	AKAP10
chrX:46669606-46682535	41	SLC9A7
chr9:37126311-37165935	14	ZCCHC7
chr10:87925512-87933251	55	PTEN
chr11:108673466-108693600	45	DDX10
chr3:125019261-125029488	5	HEG1
chr11:34108210-34118503	8	NAT10
chr12:51975264-51976575	10	ACVR1B
chr1:197583151-197617759	27	DENND1B
chr17:19936285-19968461	9	AKAP10
chr2:9279316-9350895	8	ASAP2
chr7:33604864-33606553	27	BBS9

chr17:66175043-66183307	90	CEP112
chr20:35511362-35512066	45	CEP250
chr1:26432890-26438284	8	DHDDS
chr1:50582617-50596216	17	FAF1
chr1:15767375-15774796	5	FBLIM1
chr8:43059091-43080441	9	FNTA
chr5:151790322-151797428	9	G3BP1
chr6:125277101-125300659	9	HDDC2
chr18:6213148-6244588	10	L3MBTL4
chr1:117493146-117522937	12	MAN1A2
chr14:75038339-75042477	9	MLH3
chr3:27344271-27352919	5	NEK10
chr11:74994508-75005625	15	NEU3
chr20:25476042-25477089	9	NINL
chr15:22845145-22853268	8	NIPA2
chr11:3768580-3776021	65	NUP98
chr13:24469023-24475596	76	PARP4
chr16:71658232-71664099	15	PHLPP2
chr7:100325690-100329019	82	PMS2P1
chr11:71960749-71962289	9	RNF121
chr4:82874610-82875822	48	SEC31A
chr6:132783932-132797121	12	SLC18B1
chr18:2769693-2770108	8	SMCHD1
chr14:75732337-75735289	122	TTLL5
chr1:12299201-12311925	27	VPS13D
chr9:112142319-112157613	26	SUSD1
chr10:32037253-32040457	10	KIF5B
chr6:108319616-108332999	9	nogene
chr1:197172159-197191899	16	ZBTB41
chr17:42818220-42820841	11	BECN1
chr20:33636639-33640531	15	CBFA2T2
chr10:92111075-92145112	4	CPEB3
chr17:44729904-44731015	4	DBF4B
chr9:137754170-137818138	4	EHMT1
chr5:50402285-50405852	11	EMB
chr15:99218587-99221864	4	TTC23
chr6:109475398-109476930	19	ZBTB24
chr10:73739926-73740266	13	nogene
chr22:29825086-29832342	10	ASCC2
chr17:76328716-76348581	5	PRPSAP1
chr20:33619516-33629914	25	CBFA2T2
chr1:32030297-32031640	9	KHDRBS1
chr2:44549554-44558013	9	nogene

chr5:116298246-116318300	25	nogene
chr20:44479984-44486706	19	TTPAL
chr8:98706466-98749390	81	STK3
chr6:104843222-104849246	12	HACE1
chr1:51465260-51481314	9	EPS15
chr7:66808885-66810847	106	RABGEF1
chr2:203489583-203497395	7	RAPH1
chr18:14077328-14085076	23	ZNF519
chr19:32615657-32626827	10	ANKRD27
chr1:233177801-233252788	18	PCNXL2
chr15:78289618-78290771	8	WDR61
chr8:130079901-130092143	137	ASAP1
chr5:163009789-163012288	9	nogene
chrX:48925846-48926050	14	OTUD5
chr8:99274197-99391704	7	VPS13B
chr8:102834188-102839823	34	AZIN1
chr10:68740628-68747566	21	CCAR1
chr8:119850381-119853215	17	DSCC1
chr19:11100222-11102786	5	LDLR
chr2:74156417-74172752	14	MOB1A
chr11:29381090-29384112	8	nogene
chr2:200925835-200937966	16	ORC2
chr2:208300936-208302353	14	PIKFYVE
chr14:73071626-73088161	15	RBM25
chr4:2490336-2497121	169	RNF4
chr15:25340084-25360527	7	UBE3A
chr5:77118319-77118521	13	ZBED3-AS1
chr17:18206814-18208375	55	ALKBH5
chr6:17539268-17543143	34	CAP2
chr3:45091172-45093657	5	CDCP1
chr5:108184941-108186247	13	FBXL17
chr16:81854443-81859163	17	PLCG2
chr13:52433661-52434882	5	VPS36
chr12:45860800-45893721	15	ARID2
chr11:85981128-86031611	34	PICALM
chr1:229539455-229542371	17	ABCB10
chr1:244409484-244437768	6	ADSS
chr12:31689006-31709425	31	AMN1
chr12:45421070-45423062	13	ANO6
chr10:31820386-31861658	20	ARHGAP12
chr16:18797924-18798834	171	ARL6IP1
chrX:1421657-1428121	5	ASMTL
chr5:81987558-82178587	8	ATG10

chr7:140776911-140808995	31	BRAF
chr7:16685923-16697061	12	BZW2
chr6:4867355-4892379	14	CDYL
chr18:12999420-13001582	104	CEP192
chr1:26255170-26259802	47	CEP85
chr20:41533049-41533570	9	CHD6
chr5:116087496-116134178	12	COMMD10
chr20:49088008-49089746	52	CSE1L
chr8:103430611-103437307	5	DCAF13
chr15:74640465-74675142	8	EDC3
chr7:2357660-2360902	8	EIF3B
chr8:93706044-93718788	68	FAM92A1
chr5:73006444-73041315	5	FCHO2
chr4:53383772-53391498	25	FIP1L1
chr4:173247979-173248440	97	GALNT7
chr16:81362498-81365478	6	GAN
chr7:139715931-139742038	32	HIPK2
chr3:160356012-160366152	9	IFT80
chr20:21214534-21215648	12	KIZ
chr3:197832196-197839397	36	LRCH3
chr21:25588834-25601467	5	MRPL39
chr17:42538338-42541206	11	NAGLU
chr9:33294419-33295427	8	NFX1
chr5:65780178-65792655	12	NLN
chr1:238471652-238472160	25	nogene
chr22:20710214-20710540	45	nogene
chr6:84532449-84541724	18	nogene
chr19:36310198-36312249	10	nogene
chr11:3778872-3782145	12	NUP98
chrX:68046993-68073299	25	OPHN1
chr10:45862985-45896244	11	PARGP1
chr4:30920152-30950215	27	PCDH7
chr3:48535511-48536463	14	PFKFB4
chr3:138683677-138694907	9	PIK3CB
chr22:27853512-27860241	10	PITPNB
chr5:160093100-160094065	36	PWWP2A
chr3:49056861-49077038	14	QRICH1
chr1:174250474-174278779	10	RABGAP1L
chr19:39354005-39357089	8	SAMD4B
chr12:124807760-124817707	6	SCARB1
chr14:30719324-30734858	5	SCFD1
chr11:18611476-18612488	16	SPTY2D1
chr20:13569506-13617123	5	TASP1

chr10:94441895-94522453	18	TBC1D12
chr10:113040024-113089540	26	TCF7L2
chr9:33953284-33956146	115	UBAP2
chr10:11997671-12001825	7	UPF2
chr8:99511103-99521010	9	VPS13B
chr17:27304779-27309272	30	WSB1
chr6:43527633-43528928	28	XPO5
chr1:32592930-32595223	13	ZBTB8A
chr6:38061592-38062525	15	ZFAND3
chrX:47846104-47895940	560	ZNF81
chr1:52856997-52867777	12	ZYG11A
chr15:52141139-52154076	8	GNB5
chr1:13773077-13782831	19	PRDM2
chr7:87836192-87860640	11	SLC25A40
chr14:22947170-22948013	15	HAUS4
chr19:32622421-32626827	14	ANKRD27
chr10:73713203-73722577	8	BMS1P4
chr7:16689796-16697061	31	BZW2
chr22:46687106-46721015	4	CERK
chr5:98879551-98881375	79	CHD1
chr1:15638306-15643650	123	DDI2
chr17:56844018-56849232	11	DGKE
chr3:197282678-197297235	21	DLG1
chr2:17761395-17765073	5	GEN1
chr10:87057690-87068157	13	GLUD1
chr7:99030594-99033039	8	LOC101927550
chr19:17720935-17725189	6	MAP1S
chr10:94350112-94352403	43	NOC3L
chr11:68519500-68554257	10	PPP6R3
chr20:18524934-18526531	5	SEC23B
chr12:130806981-130813031	14	STX2
chr6:123224092-123267751	19	TRDN
chr1:10095460-10119613	10	UBE4B
chr5:176943334-176982623	8	UIMC1
chr2:61378362-61395233	12	USP34
chr1:27260955-27287861	13	WDTC1
chr6:150955994-150972058	74	MTHFD1L
chr4:1265339-1270295	62	nogene
chr8:119783380-119791459	27	TAF2
chrX:101101057-101130073	11	CENPI
chr1:117402185-117414831	1417	MAN1A2
chr2:24643965-24658766	12	NCOA1
chr7:66479008-66490107	12	nogene

chr1:233217898-233227371	232	PCNXL2
chr22:31725537-31738911	63	PRR14L
chr1:154963518-154970199	16	SHC1
chr18:36576450-36612095	15	FHOD3
chr18:21791373-21799974	11	MIB1
chr17:31155982-31182665	106	NF1
chr16:46668929-46672472	8	VPS35
chr17:41887598-41892447	4	ACLY
chr17:4206320-4209947	43	ANKFY1
chr4:73135116-73177533	4	ANKRD17
chr19:32631401-32644479	9	ANKRD27
chr11:959436-972255	6	AP2A2
chr6:156829226-156835950	14	ARID1B
chr2:175080659-175111654	8	ATF2
chr1:93667095-93674941	212	BCAR3
chr19:3620294-3620706	5	CACTIN
chr10:118685982-118730410	16	CACUL1
chr7:128748572-128759852	9	CALU
chr5:179708380-179708946	15	CANX
chr6:17539268-17541148	18	CAP2
chr2:207714341-207742342	10	CCNYL1
chr11:65079352-65079787	14	CDCA5
chr3:113358744-113366309	19	CFAP44
chr2:201133028-201140439	10	CFLAR
chr5:123557494-123575963	8	CSNK1G3
chr4:1212912-1241519	5	CTBP1
chr1:155602072-155726019	14	DAP3
chr3:57633264-57641747	29	DENND6A
chr14:74889094-74894409	9	DLST
chr10:68437010-68445083	7	DNA2
chr6:43225646-43226395	9	DNPH1
chr1:62577261-62625401	14	DOCK7
chr18:49257009-49286616	6	DYM
chr11:32587011-32601822	7	EIF3M
chr7:158773340-158799072	154	ESYT2
chr12:22659013-22673660	168	ETNK1
chrX:54465450-54465852	37	FGD1
chr4:53383772-53453133	9	FIP1L1
chrX:131777622-131784039	14	FIRRE
chr2:31078933-31125253	8	GALNT14
chr3:81642780-81753280	8	GBE1
chr5:55960401-55969855	5	IL6ST
chr14:103673013-103677523	8	KLC1

chr12:50435487-50454417	12	LARP4
chr6:84444386-84556154	8	LINC01611
chr9:110941420-111009201	6	LPAR1
chr6:89751430-89756390	9	MDN1
chr14:67301388-67303599	114	MPP5
chr8:97699753-97713769	9	MTDH
chr12:124398118-124422555	12	NCOR2
chr7:66890250-66894018	18	nogene
chr2:94196054-94203287	48	nogene
chr9:96895895-96942752	12	nogene
chr6:43479877-43480609	24	nogene
chr18:57763778-57780956	9	nogene
chr2:44490785-44549728	14	nogene
chr19:46971313-46971594	31	nogene
chr2:189752848-189754064	8	OSGEPL1
chr5:41799993-41803163	13	OXCT1
chr1:233177801-233218184	4	PCNXL2
chr7:99403397-99404953	37	PDAP1
chr2:10797080-10797757	10	PDIA6
chr2:172570725-172596023	46	PDK1
chr20:9419806-9423952	14	PLCB4
chr22:43939335-43940125	14	PNPLA3
chr8:140735250-140744767	6	PTK2
chr18:8085670-8088851	5	PTPRM
chr7:66797373-66805400	7	RABGEF1
chr9:107302034-107306647	17	RAD23B
chr8:94458267-94467555	15	RAD54B
chrX:16857593-16869220	8	RBBP7
chr2:60891682-60901083	7	REL
chr16:74649131-74661451	4	RFWD3
chr12:120565071-120571291	10	RNF10
chr5:179591624-179594963	6	RUFY1
chr6:148468544-148487715	70	SASH1
chr1:243415701-243426558	129	SDCCAG8
chr4:25127263-25159107	75	SEPSECS
chr11:70798442-70807171	6	SHANK2
chr3:72841044-72844423	63	SHQ1
chr12:46239678-46254936	479	SLC38A1
chr3:27418485-27424152	15	SLC4A7
chr7:17845594-17875790	6	SNX13
chr7:38214356-38219660	12	STARD3NL
chr1:85178145-85190752	7	SYDE2
chr12:103984748-103985728	4	TDG

chr7:98228013-98229166	16	TECPR1
chr2:70214348-70230854	8	TIA1
chr2:171081639-171117857	10	TLK1
chr22:28163091-28163599	52	TTC28
chr21:37087246-37098160	10	TTC3
chr1:32192310-32193300	37	TXLNA
chr13:29767617-29777263	15	UBL3
chr2:169896506-169906164	9	UBR3
chr19:36065957-36068010	6	WDR62
chr4:1930625-1939778	7	WHSC1
chr16:88598177-88598712	6	ZC3H18
chr19:9364861-9371752	29	ZNF177
chr1:236803430-236816543	8	MTR
chr2:174877839-174878128	11	CHN1
chr9:85956359-85962180	29	NAA35
chr17:5346789-5354490	4	RABEP1
chr4:7838523-7843350	21	AFAP1
chr3:112548532-112558417	21	ATG3
chr7:25151595-25161260	10	C7orf31
chr6:110746125-110779547	9	CDK19
chr1:16625774-16635168	18	CROCCP2
chr9:137728348-137762820	78	EHMT1
chr12:53033805-53038411	23	EIF4B
chr1:240329075-240392562	19	FMN2
chrX:72567888-72568884	20	HDAC8
chr1:40738835-40753246	21	NFYC
chr4:2488370-2488905	8	nogene
chr8:118239714-118241270	59	nogene
chr2:208298640-208302353	34	PIKFYVE
chr11:111747953-111755450	6	PPP2R1B
chrX:20193486-20204103	7	RPS6KA3
chr11:62885176-62885608	25	SLC3A2
chr9:111922848-111931357	70	UGCG
chr2:218546918-218560883	10	USP37
chr19:2180712-2194577	31	DOT1L
chr5:127526617-127547921	39	PRRC1
chr8:103652204-103697296	19	RIMS2
chr7:5721032-5730817	7	RNF216
chr16:68181474-68183366	5	NFATC3
chr2:201707868-201710038	13	ALS2
chr11:46780193-46790583	5	CKAP5
chr1:75787496-75791571	13	RABGGTB
chr17:5388801-5399650	91	NUP88

chr1:58681784-58685251	28	MYSM1
chrX:63697124-63706449	19	ARHGEF9
chr16:14661891-14665071	6	BFAR
chr2:119311621-119340361	6	C2orf76
chr3:33124407-33132700	8	CRTAP
chr2:75518055-75526199	52	EVA1A
chr5:141625207-141630125	12	HDAC3
chr7:110886592-111561923	11	IMMP2L
chr10:69005045-69011015	8	KIAA1279
chr5:78592144-78632368	8	LHFPL2
chr15:67580753-67600749	82	MAP2K5
chr14:64448216-64449622	32	MTHFD1
chr12:56224833-56226419	23	NABP2
chr5:36953617-36958231	18	NIPBL
chr9:83574088-83575055	20	nogene
chr17:7408011-7415671	11	nogene
chr4:2949933-2953645	9	NOP14
chr11:3762901-3776021	14	NUP98
chr14:63415139-63453885	8	PPP2R5E
chr2:101281858-101295181	77	RNF149
chr4:109491326-109506512	19	SEC24B
chr11:230451-233534	7	SIRT3
chr16:18815174-18819654	9	SMG1
chr5:122998207-123002004	4	SNX24
chr17:63351249-63355390	4	TANC2
chr1:234450427-234472811	59	TARBP1
chr10:94500220-94522453	28	TBC1D12
chr17:54912665-54947312	26	TOM1L1
chr19:34430575-34464131	23	UBA2
chrX:41128999-41134837	5	USP9X
chr8:99556449-99577633	10	VPS13B
chr10:1124217-1125024	50	WDR37
chr3:47169168-47174263	13	KIF9-AS1
chr9:131135939-131147584	6	NUP214
chr5:87331347-87346724	12	RASA1
chr12:121417860-121420778	21	RNF34
chr20:56473300-56477124	18	RTFDC1
chr22:28629551-28631128	20	TTC28
chr4:168220654-168225676	4	DDX60
chr12:123647962-123651156	6	GTF2H3
chr10:13195040-13198807	5	MCM10
chr3:196245384-196245731	32	nogene
chr4:82821036-82829058	4	SEC31A

chr20:63521283-63521589	24	PPDPF
chr15:50574851-50575789	65	TRPM7
chr17:18024623-18028659	25	ATPAF2
chr2:36387971-36479694	10	CRIM1
chr18:6171827-6312056	13	L3MBTL4
chr4:184672172-184682336	13	PRIMPOL
chr1:150817919-150826617	35	ARNT
chr3:11313303-11333093	9	ATG7
chr10:115120184-115129535	260	ATRNL1
chr17:43067607-43074521	49	BRCA1
chr6:20649292-20781265	12	CDKAL1
chr1:20980333-21050994	32	EIF4G3
chr5:151786602-151791062	39	G3BP1
chr9:19063007-19063580	25	HAUS6
chr15:69421646-69422435	62	KIF23
chr17:12081355-12129287	11	MAP2K4
chr16:29809539-29809764	93	MAZ
chr13:29480149-29492719	31	MTUS2
chr20:36687491-36688784	70	NDRG3
chr15:55951375-55966546	28	NEDD4
chr12:73444851-73477374	21	nogene
chr19:19786608-19789821	12	nogene
chr3:167695595-167720273	19	PDCD10
chr20:20653529-20680801	7	RALGAPA2
chr6:13652658-13696896	24	RANBP9
chr17:59119318-59131367	10	SKA2
chr4:42001615-42020515	9	SLC30A9
chr3:63826823-63839773	6	THOC7
chr15:42775134-42817097	7	TTBK2
chr13:45516142-45519094	21	COG3
chr7:107548110-107558115	12	COG5
chr10:68422224-68422890	26	DNA2
chr1:198232552-198264235	25	NEK7
chr20:34031521-34033318	4	RALY
chr18:23006362-23022270	16	RBBP8
chr8:118375781-118379700	167	SAMD12
chr12:48074323-48074793	6	SENP1
chr8:66835762-66836074	5	SGK3
chr3:98756368-98772916	26	ST3GAL6
chr13:41230052-41230334	10	nogene
chr15:50583088-50592626	35	TRPM7
chr19:1031070-1032696	54	CNN2
chr13:30254104-30255615	80	KATNAL1

chr19:41866111-41867454	15	nogene
chr3:185492224-185493651	7	nogene
chr5:177244194-177246796	33	NSD1
chr9:127925106-127925979	5	PIP5KL1
chr4:2470986-2500738	6	RNF4
chr3:25643693-25645455	4	TOP2B
chr15:90486953-90491545	9	IQGAP1
chr17:13892724-13898732	9	nogene
chr13:24477700-24503777	16	PARP4
chr1:94498601-94506642	14	ABCD3
chr20:49637340-49647078	8	B4GALT5
chr11:116757145-116760952	8	BUD13
chr7:6425987-6436533	24	DAGLB
chr21:33534578-33535320	24	GART
chr22:29978441-29991670	59	MTMR3
chr16:15677800-15687511	8	NDE1
chr3:52490231-52490678	5	NISCH
chr5:65758566-65763108	9	NLN
chr1:169298555-169303195	14	NME7
chr4:128037203-128037435	8	nogene
chr5:10213490-10224061	330	nogene
chr12:68690630-68702784	9	NUP107
chr20:3910576-3918796	21	PANK2
chr4:107653490-107701285	8	PAPSS1
chr10:34450316-34696419	16	PARD3
chr11:64927801-64928425	8	PPP2R5B
chr18:22936753-22949713	54	RBBP8
chr7:66991136-66994341	15	SBDS
chr7:29940681-29944179	10	SCRN1
chr12:111418118-111420488	8	SH2B3
chr15:43377846-43383504	11	TUBGCP4
chr2:170040881-170061443	4	UBR3
chr1:21701497-21706868	7	USP48
chr10:45651965-45663842	8	ZFAND4
chr1:35093913-35113181	8	ZMYM1
chr14:102331621-102339223	9	ZNF839
chr3:138570317-138571356	409	CEP70
chr1:117896832-117920424	21	GDAP2
chr18:49286433-49379758	7	DYM
chr10:31843460-31861658	11	ARHGAP12
chr7:102158559-102178657	71	CUX1
chr2:190510360-190525378	17	NEMP2
chr9:112268048-112297916	557	PTBP3

chr6:47574063-47582065	32	CD2AP
chr20:2492698-2500742	9	ZNF343
chr12:110473077-110486717	18	FAM216A
chr9:98780188-98782573	47	ANKS6
chr10:7798989-7802854	9	ATP5C1
chr5:138512763-138513706	4	ETF1
chr11:12236176-12239585	19	MICAL2
chr2:203041764-203057453	12	NBEAL1
chr15:25059154-25062088	9	nogene
chr5:37302778-37305210	5	NUP155
chr16:56466151-56468018	13	OGFOD1
chr15:50739679-50748870	5	SPPL2A
chr12:130795994-130801488	5	STX2
chr1:162566011-162568386	23	UAP1
chr19:43543390-43546977	4	XRCC1
chr16:3506330-3512478	26	CLUAP1
chr12:53018797-53028188	6	EIF4B
chr14:54702061-54702580	401	SAMD4A
chr17:64583460-64591149	9	SMURF2
chr4:102799406-102802638	10	UBE2D3
chr10:93500039-93507056	19	CEP55
chr6:17647806-17649300	15	NUP153
chr16:47311239-47313836	10	ITFG1
chr2:235968461-235975448	24	AGAP1
chr5:126575419-126593404	49	ALDH7A1
chr5:112754872-112767390	46	APC
chr19:16509149-16509988	8	C19orf44
chr5:154029488-154034946	11	FAM114A2
chr18:76441244-76443211	89	ZNF516
chr1:12455997-12460396	15	VPS13D
chr15:90486953-90492711	42	IQGAP1
chr18:6213148-6312056	34	L3MBTL4
chr1:45654542-45655319	31	GPBP1L1
chr2:26263379-26285571	11	HADHB
chr15:66349059-66351600	34	TIPIN
chr2:178540106-178600667	11	TTN-AS1
chr4:2875895-2884666	70	ADD1
chr22:25685169-25695214	11	ADRBK2
chr1:165679441-165683110	188	ALDH9A1
chr14:55380572-55382191	13	ATG14
chrX:77688817-77698629	9	ATRX
chr2:32597750-32611582	7	BIRC6
chr7:16674411-16681404	9	BZW2

chr11:76458182-76472840	19	C11orf30
chr1:207757013-207759724	23	CD46
chr11:61329362-61330074	76	DDB1
chr11:108688935-108723462	15	DDX10
chr11:103303092-103323990	202	DYNC2H1
chr9:137716625-137717182	40	EHMT1
chr22:31468174-31471917	10	EIF4ENIF1
chr5:50402285-50411383	241	EMB
chr4:88990972-89009078	19	FAM13A
chr12:112306063-112313147	24	HECTD4
chr6:34240736-34243518	116	HMGA1
chr21:33810974-33819323	9	ITSN1
chr1:32154587-32159531	21	KPNA6
chr3:45474242-45500579	9	LARS2
chr3:197830769-197866219	13	LRCH3
chr14:50462664-50482416	8	MAP4K5
chr5:113063983-113085310	16	MCC
chr2:190655976-190673152	96	NAB1
chr2:203056426-203068475	5	NBEAL1
chr17:31155982-31163376	92	NF1
chr16:57017073-57021007	5	NLRC5
chr12:10506539-10506769	7	nogene
chr7:128591849-128609579	20	nogene
chr1:5947103-5986327	41	NPHP4
chr2:178284926-178328378	9	OSBPL6
chr11:77355667-77359017	17	PAK1
chr13:24459043-24478276	6	PARP4
chr2:70046502-70051305	24	PCBP1-AS1
chr11:34957383-34992379	53	PDHX
chr7:30045084-30062742	11	PLEKHA8
chr22:46198341-46198591	12	PPARA
chr12:27650009-27667320	18	PPFIBP1
chr14:61529074-61530595	5	PRKCH
chr1:43569589-43578920	8	PTPRF
chr16:22429795-22435812	18	RRN3P3
chr2:127989563-127989866	30	SAP130
chr10:73746629-73747004	29	SEC24C
chr13:49480218-49483563	11	SETDB2
chr8:91289135-91295703	6	SLC26A7
chr4:185339378-185346650	26	SNX25
chr1:172551511-172557794	293	SUCO
chr6:121279120-121310847	7	TBC1D32
chr12:64484302-64486017	33	TBK1

chr10:45975453-45988847	426	TIMM23
chr14:21488589-21489403	9	TOX4
chr17:56908467-56908877	12	TRIM25
chr14:90578105-90618045	12	TTC7B
chr9:77238001-77238386	21	VPS13A
chr8:99096311-99275254	10	VPS13B
chr8:99111097-99121445	10	VPS13B
chr12:122238586-122239945	9	VPS33A
chr2:63968956-63981887	15	VPS54
chr20:63789820-63805464	6	ZBTB46
chr8:143688661-143690123	27	ZNF707
chr7:45736786-45759174	39	SEPT7P2
chr1:220114313-220139139	10	IARS2
chr19:17733192-17734416	7	MAP1S
chr3:8935870-8948570	50	RAD18
chr9:132887368-132888437	5	C9orf9
chr9:129506176-129506420	8	nogene
chr12:19462509-19493986	36	AEBP2
chr11:77624962-77625818	838	CLNS1A
chr15:65729071-65756472	8	DENND4A
chr1:225167938-225185425	71	DNAH14
chr2:25313912-25314161	6	DNMT3A
chr12:53019909-53022627	81	EIF4B
chr12:1289851-1444750	39	ERC1
chr6:629834-647666	10	EXOC2
chr14:53156242-53189936	10	LOC101927620
chr16:48256609-48303305	67	LONP2
chr10:68175322-68189126	26	MYPN
chr9:124684600-124684986	13	nogene
chr2:155759630-155814067	7	nogene
chr6:34288739-34295685	6	NUDT3
chr12:123484179-123499536	5	RILPL1
chr6:145893214-145894977	18	SHPRH
chr5:151458787-151467925	11	SLC36A1
chr17:37440504-37462121	18	TADA2A
chr4:26748410-26769852	16	TBC1D19
chr5:14222781-14280436	10	TRIO
chr7:98965695-98971945	13	TRRAP
chr1:162588692-162590511	9	UAP1
chr15:75854385-75883424	55	UBE2Q2
chr2:23958300-23983033	10	UBXN2A
chr3:51466802-51483836	7	VPRBP
chr18:56756582-56758953	173	WDR7

chr3:141368620-141381487	11	ZBTB38
chr10:31495775-31502509	26	ZEB1
chr1:77631849-77633405	15	ZZZ3
chr2:37199703-37201726	15	CEBPZOS
chr16:56385918-56409588	6	AMFR
chr5:78141142-78165672	5	AP3B1
chr3:94003658-94003908	141	ARL13B
chr10:114843740-114848737	26	FAM160B1
chr1:93896617-93904588	21	GCLM
chr16:74474545-74477533	9	GLG1
chr3:155897926-155906263	17	GMPS
chr5:179768849-179771243	50	MAML1
chr1:167772274-167788137	98	MPZL1
chr6:130311290-130341122	8	nogene
chr16:23603586-23608012	8	PALB2
chr22:31556941-31561392	12	SFI1
chr19:38141183-38164906	9	SIPA1L3
chr2:85637368-85641118	8	USP39
chr1:48305786-48359770	17	SPATA6
chr9:120447894-120471878	6	CDK5RAP2
chr1:225167938-225231151	35	DNAH14
chr4:36187450-36214480	9	ARAP2
chr20:49646964-49647892	12	B4GALT5
chr17:30266884-30272899	29	BLMH
chr8:96784863-96785330	11	CPQ
chr3:142181460-142187779	10	GK5
chr1:150661813-150663763	10	GOLPH3L
chr11:18407116-18446333	36	LDHA
chr20:37301207-37302425	18	MANBAL
chr5:43609142-43628387	21	NNT
chr11:70366524-70372534	12	nogene
chr2:97267654-97267971	31	nogene
chr7:24815058-24816688	11	OSBPL3
chr6:37374619-37381354	12	RNF8
chr13:29514352-29536202	48	SLC7A1
chr1:35097316-35110447	12	ZMYM1
chr10:31820386-31852597	4	ARHGAP12
chr10:100806429-100809236	4	PAX2
chr2:24554388-24564430	42	nogene
chr5:14286870-14297263	14	TRIO
chr4:102525511-102567135	32	NFKB1
chr3:141403926-141405887	12	ZBTB38
chr3:52911002-52913617	4	SFMBT1

chr11:72726619-72732597	11	ARAP1
chr10:73704985-73721504	17	BMS1P4
chr16:3504719-3515591	12	CLUAP1
chrX:130783546-130809261	8	ENOX2
chr2:26364301-26373629	32	EPT1
chr7:22976209-22984045	437	FAM126A
chr12:32564136-32701562	24	FGD4
chr7:112455762-112457038	9	IFRD1
chr15:41081019-41087682	53	INO80
chr12:2820085-2836956	4	ITFG2
chr8:89724628-89725641	7	LOC101929709
chr2:63597398-63599292	9	MDH1
chr5:154950820-154960049	176	MRPL22
chr12:65306862-65328603	20	MSRB3
chr16:69982349-69986509	9	PDXDC2P
chr12:26990449-26999676	48	TM7SF3
chr7:66941847-66951135	16	TMEM248
chr6:42573733-42592229	4	UBR2
chr8:99431536-99556653	4	VPS13B
chr15:80098415-80122800	90	ZFAND6
chr1:243512323-243843282	11	AKT3
chr20:20022455-20022988	23	NAA20
chr17:8450779-8454887	8	NDEL1
chr14:75087017-75088641	35	NEK9
chr6:141772872-141831645	18	nogene
chr2:37316236-37317899	36	PRKD3
chr12:64062916-64097375	8	SRGAP1
chr10:69185986-69186550	14	SUPV3L1
chr9:6475390-6482099	21	UHRF2
chr4:1904215-1918623	33	WHSC1
chr17:81979183-81985607	60	ASPSCR1
chr17:2330514-2332359	19	TSR1
chr19:38723936-38724565	37	ACTN4
chr8:130167535-130187285	21	ASAP1
chr10:101847403-101849892	15	C10orf76
chr10:101975211-101995355	25	C10orf76
chr20:33628349-33629914	13	CBFA2T2
chr22:17044872-17047426	381	CECR7
chr20:35508942-35510054	14	CEP250
chr1:108943474-108950448	18	CLCC1
chr4:140409836-140413087	5	CLGN
chr11:14469335-14477015	15	COPB1
chr12:108652271-108678394	9	CORO1C

chr1:168065589-168068463	12	DCAF6
chr1:168019544-168023047	12	DCAF6
chr8:131940498-131950090	11	EFR3A
chr7:158759485-158773396	16	ESYT2
chr1:77873003-77875111	32	FAM73A
chr1:240123178-240178068	9	FMN2
chr5:151790322-151791062	368	G3BP1
chr1:1806474-1839238	14	GNB1
chr15:52133377-52141272	6	GNB5
chr7:74705163-74716950	49	GTF2I
chr4:105693298-105695668	10	INTS12
chr9:111428041-111442424	20	KIAA0368
chr2:61092463-61106662	8	KIAA1841
chr14:74091231-74101238	106	LIN52
chr16:954356-954666	209	LMF1
chr12:68835828-68839801	5	MDM2
chr2:15534542-15539356	36	NBAS
chr15:61015797-61041377	4	nogene
chr3:129867009-129880562	11	nogene
chr6:44253209-44253683	10	nogene
chr1:229458160-229460769	9	NUP133
chr11:3768580-3771928	17	NUP98
chr5:139363758-139364743	67	PAIP2
chr10:13294427-13295606	8	PHYH
chr22:27853536-27860241	34	PITPNB
chr3:121532989-121544906	9	POLQ
chr7:42917513-42917835	9	PSMA2
chr11:73716250-73730823	21	RAB6A
chr6:42880860-42886470	61	RPL7L1
chr13:23365165-23368487	70	SACS
chr14:39045162-39063413	9	SEC23A
chr10:13328350-13345028	14	SEPHS1
chr13:29550203-29553860	30	SLC7A1
chr19:49090290-49098704	6	SNRNP70
chr7:105203627-105297533	21	SRPK2
chr7:832504-842130	13	SUN1
chr4:26717932-26753890	6	TBC1D19
chr9:100308499-100310379	27	TEX10
chr15:99156147-99175155	5	TTC23
chr12:104311289-104327671	47	TXNRD1
chr17:4289229-4296814	12	UBE2G1
chr3:184936245-184999861	9	VPS8
chr3:44629064-44631221	43	ZNF197

chr16:75608223-75608988	4	ADAT1
chr2:68490189-68567387	12	APLF
chr8:130136950-130187285	4	ASAP1
chr11:63651935-63652575	4	ATL3
chrX:139802235-139826823	4	ATP11C
chr6:99375939-99383824	28	COQ3
chr1:197595207-197715074	5	DENND1B
chr10:99894945-99900066	13	DNMBP
chr14:73729639-73730179	8	ELMSAN1
chr1:113091317-113096365	8	LRIG2
chrX:150744360-150751240	4	MTMR1
chr1:32231743-32232184	16	MTMR9LP
chr2:53603390-53606080	4	nogene
chr6:57050571-57050975	24	nogene
chr1:207815928-207819146	4	nogene
chr1:233250738-233263163	10	PCNXL2
chr11:70324401-70326818	5	PPFIA1
chr14:103752999-103754244	41	PPP1R13B
chr2:135120818-135135932	4	RAB3GAP1
chr5:131592382-131624334	6	RAPGEF6
chr9:4841201-4860901	14	RCL1
chr19:47150201-47155213	16	SAE1
chr1:161323613-161328497	4	SDHC
chr7:35863551-35873775	14	7-Sep
chr20:5106187-5109445	53	TMEM230
chr15:99241364-99245510	10	TTC23
chr16:3311752-3313175	5	ZNF75A
chr3:9750944-9751957	23	OGG1
chr14:23061083-23061622	37	ACIN1
chr15:85664562-85684873	4	AKAP13
chr12:14456560-14466590	11	ATF7IP
chr22:24035443-24038461	32	CABIN1
chr16:53247292-53255779	7	CHD9
chr2:36499218-36522313	45	CRIM1
chr5:138904348-138925407	6	CTNNA1
chr12:100591735-100601753	20	GAS2L3
chr5:75343852-75351602	4	HMGCR
chr3:185692698-185698347	10	IGF2BP2
chr1:187324092-187329060	14	LINC01036
chr4:124522106-124556510	7	LOC101927087
chr4:150896393-150916738	13	LRBA
chr5:33023074-33027826	8	nogene
chr9:2652943-2668061	8	nogene

chr12:11053032-11121180	11	nogene
chr11:17926737-18010139	6	nogene
chr14:103752999-103757751	13	PPP1R13B
chr12:118019071-118025828	11	RFC5
chr2:171826797-171855949	8	SLC25A12
chr20:17952586-17954117	114	SNX5
chr17:55031167-55081183	35	STXBP4
chr17:31975481-31988497	16	SUZ12
chr4:38014508-38027879	6	TBC1D1
chr8:140397619-140426641	78	TRAPPC9
chr18:178932-192900	11	USP14
chr1:247156831-247159813	8	ZNF124
chr8:104093470-104148848	24	nogene
chr3:160375811-160384646	8	IFT80
chr4:99028866-99048876	27	METAP1
chr8:47595810-47713641	5	SPIDR
chr3:196362293-196362687	81	UBXN7
chr16:14984049-14986841	31	nogene
chr2:68490189-68513680	42	APLF
chr12:31451939-31452476	16	DENND5B
chr1:167765582-167788137	73	MPZL1
chr14:62950132-62950559	13	KCNH5
chr8:140846259-140912467	8	PTK2
chr17:2394042-2395454	470	MNT
chr12:71769799-71774022	138	RAB21
chr17:17884633-17893810	11	TOM1L2
chr7:158748193-158767774	6	ESYT2
chr1:8465924-8541318	12	RERE
chr3:149902071-149921227	48	RNF13
chr10:110964124-110965061	19	SHOC2
chr15:63529013-63574403	74	USP3
chr19:23662113-23663158	8	ZNF675
chr3:195342470-195345317	47	ACAP2
chr19:49781756-49782724	49	AP2A1
chr20:48951322-48952884	16	ARFGEF2
chr6:110626775-110632161	5	CDK19
chr6:82214226-82223620	8	IBTK
chrX:103879163-103882115	6	nogene
chr3:47052738-47052999	6	nogene
chr3:170280226-170284596	9	PRKCI
chr22:49787389-49794294	20	BRD1
chr20:44978442-45001353	5	STK4
chr7:67067284-67117618	24	TYW1

chr1:193232992-193236356	37	CDC73
chr12:96294998-96297721	6	CDK17
chr18:6237963-6244588	40	L3MBTL4
chr10:27173120-27181581	47	MASTL
chr19:16079987-16080498	10	nogene
chr7:105160506-105268869	19	SRPK2
chr1:26897300-26897725	18	GPATCH3
chr1:113119232-113119523	13	LRIG2
chr8:94530962-94538346	12	KIAA1429
chr6:136070223-136111101	8	nogene
chr11:129123445-129164427	135	ARHGAP32
chr19:48221729-48241063	15	CARD8
chr7:65066252-65067050	25	CCT6P3
chr3:138570317-138571353	5	CEP70
chr16:53255599-53274302	48	CHD9
chr11:7665323-7672891	5	CYB5R2
chr21:33426883-33432871	40	IFNGR2
chr17:46169530-46172232	32	KANSL1
chr1:235677090-235697272	17	LYST
chr9:4841204-4860901	20	nogene
chr20:13559007-13569586	25	TASP1
chr8:102271133-102272772	20	UBR5
chr20:52097372-52098587	29	ZFP64
chr4:128992166-129039096	29	SCLT1
chr5:160035139-160036803	16	TTC1
chr1:223798214-223798677	16	TP53BP2
chrX:120532421-120534586	4	CUL4B
chr7:94618757-94629841	78	SGCE
chr2:24563903-24584560	15	NCOA1
chr4:61934839-61998265	15	ADGRL3
chr15:92967324-92967513	34	CHD2
chr17:34983001-34986131	48	LIG3
chrX:23721880-23733217	37	ACOT9
chr2:32543240-32545860	11	BIRC6
chr17:769868-778744	10	GLOD4
chr11:75851882-75880040	23	UVRAG
chr4:145870244-145892415	12	ZNF827
chr12:109249983-109259108	7	ACACB
chr9:74996446-74999870	9	CARNMT1
chr11:125643791-125656335	8	CHEK1
chr22:28699837-28734727	38	CHEK2
chr20:38983698-38994880	87	DHX35
chr1:245002120-245017326	143	EFCAB2

chr4:98887078-98891332	8	EIF4E
chr14:57235710-57244359	18	EXOC5
chr3:56660730-56673725	196	FAM208A
chr16:53879843-53934109	20	FTO
chr5:151790322-151799313	24	G3BP1
chr4:157120555-157153010	4	GLRB
chr19:38843841-38847434	15	HNRNPL
chr2:238173533-238194870	18	ILKAP
chr16:27618424-27631162	9	KIAA0556
chr4:39075964-39086727	137	KLHL5
chr7:152263015-152315338	76	KMT2C
chr14:50265357-50284033	11	L2HGDH
chr3:197613224-197624197	10	LOC220729
chr6:52267864-52277688	16	MCM3
chr10:72708282-72715902	41	MCU
chr18:50924097-50940386	7	ME2
chr11:93793727-93801972	4	MED17
chr2:99194750-99195756	24	MRPL30
chr5:72290949-72297702	8	MRPS27
chr12:76053090-76056161	13	NAP1L1
chr16:68157868-68191775	17	NFATC3
chr15:34143889-34144680	33	nogene
chr10:5712393-5714207	5	nogene
chr3:23956036-23959815	43	NR1D2
chr18:9583116-9595102	406	PPP4R1
chr11:45181760-45183123	6	PRDM11
chr17:66732687-66742760	55	PRKCA
chr17:1842802-1877314	24	RPA1
chr10:116901764-116921516	14	SHTN1
chr1:108181940-108185954	28	SLC25A24
chr15:64980863-64983593	84	SPG21
chr17:9505037-9557528	5	STX8
chr11:87067693-87075786	39	TMEM135
chr15:51869216-51889145	8	TMOD3
chr19:49196439-49196874	5	TRPM4
chr3:132670197-132672177	10	UBA5
chr18:163307-180339	59	USP14
chr15:63553714-63559970	32	USP3
chr8:99134234-99156743	13	VPS13B
chr10:27111985-27116345	8	YME1L1
chr6:110210706-110213160	12	CDC40
chrX:96939265-97247839	4	DIAPH2
chr2:229388264-229394497	30	DNER

chr9:127507866-127508538	5	FAM129B
chr3:190604127-190609181	17	IL1RAP
chr10:122392302-122417968	19	PLEKHA1
chr18:31931328-31942893	4	TRAPPC8
chr4:48377895-48383784	19	SLAIN2
chr3:53240250-53242242	24	TKT
chr16:89418283-89431326	68	ANKRD11
chr14:67345767-67347453	16	ATP6V1D
chr11:124975095-124977600	8	CCDC15
chr7:24641715-24650712	61	MPP6
chr2:95834350-95835127	4	nogene
chr16:69651902-69652215	48	nogene
chr10:102157018-102158214	63	NOLC1
chr5:139880855-139887511	26	NRG2
chr17:81908567-81909602	4	PCYT2
chr11:34931403-34970286	26	PDHX
chr3:48772954-48807684	9	PRKAR2A
chr9:111578795-111597432	17	PTGR1
chr1:224418259-224424654	23	WDR26
chr20:18159083-18162945	7	CSRP2BP
chr10:97229146-97266125	12	ARHGAP19
chr3:93995873-94003908	201	ARL13B
chr8:65613243-65627433	8	ARMC1
chr16:1625972-1637907	30	CRAMP1L
chr5:42514079-42565944	10	GHR
chr16:68174373-68183366	15	NFATC3
chr2:101009391-101009838	21	nogene
chr10:79349938-79350522	10	nogene
chr20:35716739-35732135	37	RBM39
chr15:56142777-56243287	19	RFX7
chr10:68363589-68364113	18	RUFY2
chr6:169582835-169652008	10	WDR27
chr2:27598066-27608039	14	ZNF512
chr7:66086584-66089695	11	ASL
chr11:46541944-46548500	9	AMBRA1
chr9:37842088-37861235	14	DCAF10
chr20:25306832-25317078	21	ABHD12
chr1:70292387-70306290	132	ANKRD13C
chr10:73383174-73383690	13	ANXA7
chr4:36228581-36229645	43	ARAP2
chr12:56644780-56645353	5	ATP5B
chr8:53795646-53801896	50	ATP6V1H
chr3:107728764-107733023	11	BBX

chr2:32377587-32380290	20	BIRC6
chr11:116759073-116765446	8	BUD13
chr11:36610080-36636117	10	C11orf74
chr5:37120215-37122488	9	C5orf42
chr3:122362967-122365466	20	CCDC58
chr7:39987598-40002031	261	CDK13
chr2:121347037-121348718	6	CLASP1
chr16:11120614-11126146	69	CLEC16A
chr7:107248395-107258383	12	COG5
chr19:18742909-18753585	9	CRTC1
chr20:49074784-49077064	17	CSE1L
chr9:108948185-108955827	6	CTNNAL1
chr19:1417499-1422396	32	DAZAP1
chr19:1417499-1430362	11	DAZAP1
chr11:674535-688460	10	DEAF1
chr21:37420298-37480826	13	DYRK1A
chr14:88736363-88754671	14	EML5
chr1:51461090-51481314	52	EPS15
chr5:137942874-137949184	56	FAM13B
chr4:151566137-151578076	5	FAM160A1
chr15:48596282-48613092	37	FBN1
chr17:17214984-17217182	24	FLCN
chr1:240329075-240334229	87	FMN2
chr16:53825863-53826491	19	FTO
chr9:124938576-124938866	9	GOLGA1
chr9:86033281-86046572	36	GOLM1
chr15:65554886-65558731	7	HACD3
chr1:32324478-32327677	19	HDAC1
chr15:60453602-60466713	29	ICE2
chr8:56978049-56980197	12	IMPAD1
chr12:26711172-26716242	21	ITPR2
chr10:63380317-63380482	11	JMJD1C
chr2:210104103-210155404	7	KANSL1L
chr13:30251930-30283791	26	KATNAL1
chr1:32030297-32039573	6	KHDRBS1
chr1:170016461-170055436	5	KIFAP3
chr14:103669510-103677523	5	KLC1
chr1:235686948-235697272	26	LYST
chr5:179765325-179771243	7	MAML1
chr14:21503172-21503577	10	METTL3
chr15:41748636-41750615	6	MGA
chr2:134254261-134254644	9	MGAT5
chr8:96243918-96246454	6	MTERF3

chr9:126340507-126421948	35	MVB12B
chr8:66599049-66602523	6	MYBL1
chr11:78478437-78493195	10	NARS2
chr19:13073046-13075671	55	NFIX
chr5:36953617-36986301	4	NIPBL
chr21:33550219-33551289	12	nogene
chr8:42517197-42523925	15	nogene
chr3:57679486-57681895	8	nogene
chr2:155906359-155914227	17	nogene
chr15:96285325-96290783	5	NR2F2-AS1
chr13:100368471-100449305	14	PCCA
chr16:70120460-70120719	18	PDPR
chr7:10990702-11013906	5	PHF14
chr9:120869181-120874756	10	PHF19
chr2:86098610-86100172	25	POLR1A
chr22:46175988-46198591	8	PPARA
chr14:103746372-103797518	23	PPP1R13B
chr5:145817893-145820018	20	PRELID2
chr8:47930671-47935058	12	PRKDC
chr3:62157066-62168163	13	PTPRG
chr17:5361207-5378232	4	RABEP1
chr3:51630271-51630931	30	RAD54L2
chr16:67727326-67738035	16	RANBP10
chr8:103644043-103697296	25	RIMS2
chr8:42861744-42870112	25	RNF170
chr17:77142645-77143659	5	SEC14L1
chr6:75647730-75670720	4	SENP6
chr7:100856867-100859161	16	SLC12A9
chr5:55697845-55704434	10	SLC38A9
chr15:75609557-75609994	11	SNUPN
chr2:36877890-36886826	23	STRN
chr8:53979024-54010492	40	TCEA1
chr20:62852722-62854157	8	TCFL5
chr15:56383951-56412436	16	TEX9
chrX:123665641-123686714	46	THOC2
chr7:66941847-66953369	12	TMEM248
chr2:3401776-3424663	11	TRAPPC12
chr22:28094079-28108403	13	TTC28
chr2:178571299-178600667	20	TTN-AS1
chr16:21962459-21972122	5	UQCRC2
chr1:62441487-62442299	52	USP1
chr16:84762988-84775225	12	USP10
chr3:51470928-51483836	8	VPRBP

chr1:12400180-12416827	26	VPS13D
chr2:63933672-63944655	9	VPS54
chrX:65488645-65499989	12	ZC3H12B
chr10:45639815-45653059	16	ZFAND4
chr17:49316825-49322508	5	ZNF652
chr19:11903572-11925273	24	ZNF700
chr19:21062605-21098910	21	ZNF714
chr19:53423061-53449638	7	ZNF761
chr15:66535932-66546705	56	ZWILCH
chr16:16016495-16036603	8	ABCC1
chr10:74525255-74600493	11	ADK
chr10:48555462-48583152	15	ARHGAP22
chr17:43049120-43057135	12	BRCA1
chr1:100494817-100499262	10	CDC14A
chr7:39984070-40002031	21	CDK13
chr16:67616744-67629533	8	CTCF
chr5:134763903-134767060	20	DDX46
chr22:20091434-20094795	8	DGCR8
chr10:126970701-127026424	16	DOCK1
chr17:39268826-39281463	7	FBXL20
chr6:78897828-78903110	15	IRAK1BP1
chr1:187254972-187329060	5	LINC01036
chr1:70173464-70189273	11	LRRC40
chr1:113585386-113594560	5	MAGI3
chr20:32825924-32836841	15	MAPRE1
chr10:119838534-119842595	5	MCMBP
chr6:89690672-89694183	13	MDN1
chr2:207620640-207621917	4	METTL21A
chr12:120353298-120364755	5	MSI1
chr8:97699753-97719189	8	MTDH
chr7:44481291-44485284	52	NUDCD3
chr13:28256291-28270866	15	PAN3
chr1:233135012-233139855	9	PCNXL2
chr21:43004325-43010224	46	PKNOX1
chr2:169603641-169607148	7	PPIG
chr2:119932423-119946574	16	PTPN4
chr2:135150368-135153876	4	RAB3GAP1
chr14:35595633-35605709	6	RALGAPA1
chr14:24440782-24442452	15	SDR39U1
chr4:151127617-151128356	9	SH3D19
chr17:72648802-72649268	9	SLC39A11
chr14:34581560-34609742	17	SNX6
chr17:78086502-78087093	42	TNRC6C

chr12:49269464-49269976	10	TUBA1C
chr1:32187953-32188124	10	TXLNA
chr20:35366556-35394196	32	UQCC1
chr22:41342528-41343576	89	ZC3H7B
chr19:12046367-12046760	5	ZNF878
chr11:116784854-116787643	25	ZPR1
chrX:77663381-77676298	29	ATRX
chr8:86531154-86537646	9	CPNE3
chr13:49997073-50045232	13	DLEU2
chr19:10805915-10820089	8	DNM2
chr11:19832483-19984247	9	NAV2
chr4:4648976-4649542	323	STX18-AS1
chr13:113495501-113503588	9	TMCO3
chr18:68697225-68701790	36	TMX3
chr15:70682757-70687820	11	UACA
chr14:77464597-77465667	79	AHSA1
chr8:53795646-53829533	35	ATP6V1H
chr7:8003907-8055549	9	GLCCI1
chr1:113590483-113594560	9	MAGI3
chr1:5947103-5952836	30	NPHP4
chr15:63528628-63537156	8	USP3
chr2:85644947-85648853	14	USP39
chr13:95034604-95062859	6	ABCC4
chr2:9535833-9536828	165	ADAM17
chr12:1702885-1772961	14	ADIPOR2
chr2:236036560-236040841	15	AGAP1
chr5:140507784-140513479	8	ANKHD1-EIF4EBP3
chr1:156951572-156955802	9	ARHGEF11
chr6:157084661-157133207	7	ARID1B
chr10:102685815-102705489	6	ARL3
chr8:130358016-130361788	28	ASAP1
chr21:29321220-29329693	299	BACH1
chr3:107710451-107755678	16	BBX
chr11:47137315-47137729	19	C11orf49
chr3:56592969-56594028	413	CCDC66
chr12:69589484-69593613	175	CCT2
chr17:65689128-65750724	17	CEP112
chr19:4430548-4432207	31	CHAF1A
chr16:58574608-58576582	10	CNOT1
chr7:92123733-92131872	11	CYP51A1
chr1:15626668-15638434	9	DDI2
chr8:123019194-123025050	11	DERL1
chr16:58403097-58405110	17	GINS3

chr1:37582222-37587495	13	GNL2
chr9:124899328-124900547	15	GOLGA1
chr8:30700080-30712088	11	GSR
chr22:46316031-46326654	14	GTSE1
chr1:20765376-20773610	51	HP1BP3
chr3:129463559-129469417	20	IFT122
chr17:49038167-49040091	10	IGF2BP1
chr7:23404993-23418824	10	IGF2BP3
chr1:23050386-23059167	134	KDM1A
chr1:35460304-35470960	14	KIAA0319L
chr14:58498782-58508709	5	KIAA0586
chr6:149701778-149702266	15	LATS1
chr4:102664684-102714561	4	MANBA
chr12:116111427-116237705	12	MED13L
chr15:99645560-99675458	16	MEF2A
chr11:12226170-12227131	10	MICAL2
chr5:80775693-80813741	9	MSH3
chr11:47627079-47635571	18	MTCH2
chr13:29222511-29281865	4	MTUS2
chr6:134875094-134879469	4	nogene
chr6:77259666-77273307	52	nogene
chrUn_GL000218v1:74510-75077	4	nogene
chr22:28515626-28528728	8	nogene
chr14:31154569-31155107	15	nogene
chr3:132618633-132631479	30	NPHP3-ACAD11
chr3:132713125-132716909	25	NPHP3-ACAD11
chr5:37323991-37328420	13	NUP155
chr3:183833491-183833825	41	PARL
chr10:26704676-26709768	50	PDSS1
chr11:17131915-17145942	42	PIK3C2A
chr3:138681966-138684803	83	PIK3CB
chr3:138681966-138699095	33	PIK3CB
chr11:70349922-70354452	5	PPFIA1
chr1:84179176-84197824	11	PRKACB
chr4:118313200-118318556	65	PRSS12
chr12:112446275-112456063	139	PTPN11
chr8:37870419-37877551	12	RAB11FIP1
chr1:174304985-174371072	15	RABGAP1L
chr9:127034430-127069356	16	RALGPS1
chr22:35809779-35810004	9	RBFOX2
chr7:151467144-151491014	14	RHEB
chr14:90920205-90947550	17	RPS6KA5
chr12:108545138-108549214	67	SART3

chr16:30704063-30704315	163	SRCAP
chr2:152123765-152150229	13	STAM2
chr6:158017203-158033680	7	SYNJ2
chr12:51116314-51118772	23	TFCP2
chr12:51101934-51104203	10	TFCP2
chr7:130170399-130181719	15	TMEM209
chr12:104311289-104321316	8	TXNRD1
chr9:111922848-111926496	54	UGCG
chr7:38789800-38796864	109	VPS41
chr13:26577354-26613058	15	WASF3
chr22:42019310-42020096	5	WBP2NL
chr13:20064450-20083032	14	ZMYM2
chr19:44144556-44150505	8	ZNF234
chr19:14705053-14706773	6	ZNF333
chr11:111836164-111857737	6	ALG9
chr3:37335052-37355187	9	GOLGA4
chr12:122584277-122584990	26	KNTC1
chr12:50454313-50461347	71	LARP4
chr2:95100473-95104730	9	MRPS5
chr12:124483595-124486568	4	NCOR2
chr5:37292878-37299568	47	NUP155
chr8:9706810-9710220	15	TNKS
chr16:84733434-84745673	98	USP10
chr15:78593091-78601387	10	CHRNA5
chr15:64203081-64204949	13	CSNK1G1
chr5:50759642-50763242	49	PARP8
chr1:37582222-37583933	9	GNL2
chr7:23123776-23152209	17	KLHL7
chr5:160010499-160019031	9	TTC1
chr19:11214274-11217391	33	DOCK6
chr4:70827851-70833273	9	GRSF1
chr6:7176654-7189322	356	RREB1
chr14:30702295-30722559	12	SCFD1
chr1:108154982-108161293	39	SLC25A24
chr1:6318491-6349866	8	ACOT7
chr14:103587273-103590429	24	APOPT1
chr1:155347655-155349596	14	ASH1L
chr21:39206107-39229436	24	BRWD1
chr19:1990003-1997463	7	BTBD2
chr20:41512845-41514952	97	CHD6
chr1:92693801-92704744	174	EVI5
chr1:117906505-117918736	39	GDAP2
chr15:41069569-41070547	99	INO80

chr10:122880670-122888723	7	LOC399815
chr3:66405197-66417266	7	LRIG1
chr13:77278757-77296674	8	MYCBP2
chr15:20669467-20671268	45	NBEAP1
chr7:128579998-128609579	15	nogene
chr10:100779497-100781365	17	PAX2
chr9:109050282-109057322	14	TMEM245
chr12:104325336-104327671	92	TXNRD1
chr2:106122969-106166083	50	UXS1
chr15:43333310-43335826	7	ADAL
chr10:102025155-102033339	27	C10orf76
chr19:46608196-46608981	16	CALM3
chr5:65188015-65215487	27	ADAMTS6
chr2:227491546-227524915	40	AGFG1
chr6:106544570-106544933	4	AIM1
chr3:184244600-184245812	5	ALG3
chr16:89305205-89313589	52	ANKRD11
chr21:25891721-25911962	11	APP
chr2:130930951-130946635	12	ARHGEF4
chr6:157084661-157110561	138	ARID1B
chr3:138284430-138290639	6	ARMC8
chr8:130167535-130180880	6	ASAP1
chr20:32366383-32429431	5	ASXL1
chr2:25767582-25771540	7	ASXL2
chr6:106248149-106293106	6	ATG5
chrX:78009101-78020398	9	ATP7A
chr6:80129160-80203212	15	BCKDHB
chr6:56992377-57015353	4	BEND6
chr7:16665436-16682845	8	BZW2
chr19:16509498-16509988	27	C19orf44
chr15:40650300-40652105	9	CASC5
chr1:93210801-93214966	11	CCDC18
chr10:32543299-32623817	42	CCDC7
chr5:618989-647916	11	CEP72
chr22:28710005-28725367	6	CHEK2
chr11:14457783-14458687	7	COPB1
chr4:83261717-83279114	8	COQ2
chr10:68967017-68969121	9	DDX21
chr9:19316416-19316839	8	DENND4C
chr13:42119094-42129632	5	DGKH
chr10:12081471-12091684	65	DHTKD1
chr16:4450399-4455780	6	DNAJA3
chr7:111977131-112004131	7	DOCK4

chr21:36291619-36292233	15	DOPEY2
chr3:32534510-32541206	23	DYNC1LI1
chr11:103399662-103456356	8	DYNC2H1
chr18:45910520-45912456	26	EPG5
chr1:51394380-51448135	13	EPS15
chr11:128781957-128809204	8	FLI1
chr1:240165688-240178068	8	FMN2
chr2:152626524-152629905	9	FMNL2
chr13:49168612-49188633	4	FNDC3A
chr13:49131136-49145935	5	FNDC3A
chr10:130169432-130175089	13	GLRX3
chr2:190895151-190905167	8	GLS
chr12:132780797-132782493	8	GOLGA3
chr8:41609415-41611992	14	GPAT4
chr18:46124821-46128837	8	HAUS1
chrX:53561755-53562929	31	HUWE1
chr6:159991183-160027314	9	IGF2R
chr6:33690916-33691719	7	ITPR3
chr5:138372673-138393372	11	KDM3B
chr12:122577671-122580670	74	KNTC1
chr7:156756392-156763795	94	LMBR1
chr1:173460377-173462454	9	LOC101928673
chr17:82027673-82028311	5	LRRC45
chr14:45231140-45237721	5	MIS18BP1
chr19:6270578-6270759	4	MLLT1
chr11:94459407-94471759	9	MRE11A
chr19:10258221-10258685	36	MRPL4
chr19:13073046-13081855	15	NFIX
chr5:36953617-36972041	16	NIPBL
chr1:169303144-169324500	13	NME7
chr9:127400435-127400778	10	nogene
chr4:34980486-35005121	226	nogene
chr6:10537030-10537425	15	nogene
chr15:79845248-79846856	10	nogene
chr12:102257214-102315490	46	nogene
chr4:13179195-13181146	10	nogene
chr20:34023799-34024010	4	nogene
chr9:128982548-128983550	17	NUP188
chr22:45178237-45178900	99	NUP50
chr4:76124648-76144473	75	NUP54
chrX:68048415-68073299	12	OPHN1
chr18:24196124-24239382	17	OSBPL1A
chr8:100709132-100713181	259	PABPC1

chr14:20344931-20352347	20	PARP2
chr5:50759642-50778650	10	PARP8
chr8:17989858-17993619	12	PCM1
chr1:233014664-233025399	7	PCNXL2
chr3:170145422-170178938	74	PHC3
chr6:43582315-43587489	6	POLH
chr5:75534827-75552591	9	POLK
chr2:55577314-55588956	5	PPP4R3B
chr11:68558565-68596218	8	PPP6R3
chr4:86686709-86693674	16	PTPN13
chr17:76287191-76304941	47	QRICH2
chr9:131650128-131650949	4	RAPGEF1
chr1:32668224-32672901	112	RBBP4
chr11:66643449-66644140	12	RBM14-RBM4
chr13:79370944-79371930	5	RBM26
chr1:2395783-2397220	9	RER1
chr1:40190771-40202614	65	RLF
chr1:32942251-32949774	5	RNF19B
chr1:213077695-213129889	15	RPS6KC1
chr11:77722054-77725699	12	RSF1
chr3:134188836-134202874	12	RYK
chr8:118580714-118580893	16	SAMD12
chr8:30069641-30070059	13	SARAF
chr5:134697125-134705437	38	SEC24A
chr20:44506826-44514040	9	SERINC3
chr12:46229152-46229639	29	SLC38A1
chr3:27383152-27424152	16	SLC4A7
chrX:71061027-71062949	9	SNX12
chr6:85557975-85572374	16	SNX14
chr1:98691074-98701903	62	SNX7
chr7:2271849-2278305	19	SNX8
chr5:172055587-172057473	13	STK10
chr1:108782421-108794907	5	STXBP3
chr1:108796233-108798237	8	STXBP3
chr6:45003652-45106006	26	SUPT3H
chr6:10935057-10958875	4	SYCP2L
chr10:94497054-94500327	9	TBC1D12
chr20:31757406-31760179	5	TPX2
chr2:201407402-201420706	4	TRAK2
chr7:99908581-99909660	28	TRIM4
chr19:34451980-34464131	103	UBA2
chr2:23958300-23977075	104	UBXN2A
chr2:23982895-23984831	46	UBXN2A

chr12:101311718-101312276	4	UTP20
chr12:57945627-57953689	16	XRCC6BP1
chr7:55911615-55923699	6	ZNF713
chr11:86556552-86559823	141	ME3
chr10:11481769-11485911	84	USP6NL
chr8:63175330-63187745	38	YTHDF3
chr1:53800692-53832591	16	NDC1
chr18:13001456-13019206	24	CEP192
chr9:134690911-134701333	36	COL5A1
chr1:197583151-197658369	18	DENND1B
chr4:53399729-53428183	99	FIP1L1
chr2:239236592-239352918	70	HDAC4
chr1:39957086-39958825	21	MFSD2A
chr20:3910576-3912634	28	PANK2
chr17:18347495-18348440	4	SHMT1
chr2:15415545-15427794	19	NBAS
chr7:151781151-151786541	33	PRKAG2
chr16:2933715-2933979	23	FLYWCH1
chr10:73817022-73848082	9	CAMK2G
chr1:179986168-180006567	37	CEP350
chr12:31413435-31426424	13	DENND5B
chr10:92531747-92537550	107	IDE
chr12:50435487-50453672	4	LARP4
chr14:70592879-70597777	16	MED6
chr10:68145474-68161752	12	MYPN
chr8:123378624-123378898	16	nogene
chr6:3006467-3016985	11	NQO2
chr11:85996825-86031611	174	PICALM
chr6:149512154-149541586	18	PPIL4
chr2:20307977-20327378	99	PUM2
chr5:171158333-171170203	8	RANBP17
chr2:86740926-86773435	21	RMND5A
chr17:81280608-81284895	123	SLC38A10
chr8:47279861-47407961	9	SPIDR
chr3:143032783-143038960	6	U2SURP
chr13:19851354-19852190	47	ZMYM5
chr19:19878480-19880146	37	ZNF253
chr12:53018797-53020026	7	EIF4B
chr12:125102678-125118765	29	AACS
chr10:43158714-43176032	15	CSGALNACT2
chr20:63928339-63931022	19	DNAJC5
chr7:65970281-65976202	13	GUSB
chr2:61112521-61118116	20	KIAA1841

chr10:27169943-27173259	47	MASTL
chr4:120710308-120811449	6	PRDM5
chr5:176313690-176337146	9	SIMC1
chr1:167772274-167773368	11	MPZL1
chr20:5189066-5190757	5	CDS2
chr10:14928015-14935564	21	DCLRE1C
chr1:39163035-39163309	14	nogene
chr10:112888734-112906213	23	nogene
chr7:92457551-92492286	9	nogene
chr8:100225172-100231288	21	SPAG1
chr2:168138087-168167407	24	STK39
chr11:755878-760253	36	TALDO1
chr9:129807187-129809032	9	TOR1B
chr2:3387619-3401893	70	TRAPPC12
chr8:99575657-99585517	38	VPS13B
chr15:65485098-65490299	19	DPP8
chr1:235240312-235260752	18	ARID4B
chr1:227213135-227317246	5	CDC42BPA
chr11:9414254-9417148	8	IPO7
chr7:6387211-6392041	59	RAC1
chr10:11979056-12014184	128	UPF2
chr19:32352828-32360618	40	ZNF507
chr16:3738558-3745354	9	CREBBP
chr2:20774809-20801465	33	LDAH
chr9:125669817-125672643	86	MAPKAP1
chr8:47700402-47702015	119	SPIDR
chr14:102376649-102376940	10	TECPR2
chr2:222916318-222919202	7	ACSL3
chr7:151116792-151123886	45	AGAP3
chr10:4830674-4847660	27	AKR1E2
chr11:108267170-108279608	6	ATM
chr18:79126266-79176907	31	ATP9B
chr7:16685904-16697061	47	BZW2
chr19:10904950-10909207	57	CARM1
chr12:96297626-96298983	9	CDK17
chr17:15596513-15598653	10	CDRT1
chr18:74505848-74508928	5	CNDP2
chr1:100230732-100240884	4	DBT
chr11:61311783-61314486	9	DDB1
chr11:684897-688460	8	DEAF1
chr1:197595207-197772932	9	DENND1B
chr15:66306823-66308844	35	DIS3L
chr10:68437010-68446413	10	DNA2

chr20:32788853-32792770	70	DNMT3B
chr10:126970701-126996883	9	DOCK1
chr6:83132175-83142046	8	DOPEY1
chr10:119073440-119073937	30	EIF3A
chr3:71690009-71710484	8	EIF4E3
chr12:1027398-1028572	5	ERC1
chr7:158788003-158799072	691	ESYT2
chr16:81351582-81377702	35	GAN
chr16:74503530-74508925	11	GLG1
chr1:28691584-28704329	7	GMEB1
chr1:155777621-155784089	46	GON4L
chr17:7313028-7314597	9	GPS2
chr13:27427091-27430621	5	GTF3A
chr14:31122913-31133675	4	HECTD1
chr12:112178930-112179397	12	HECTD4
chr10:68033964-68073686	10	HERC4
chrX:118766529-118776511	23	IL13RA1
chrX:53217142-53218398	16	KDM5C
chr19:34330188-34339992	11	KIAA0355
chr2:23639138-23642850	12	KLHL29
chr16:25054818-25140247	6	LCMT1
chr6:69737941-69790472	12	LMBRD1
chr8:132622610-132661627	8	LRRC6
chr5:109729341-109755456	10	MAN2A1
chr14:75032067-75038412	6	MLH3
chr11:94429910-94461036	25	MRE11A
chr11:94429910-94456338	24	MRE11A
chr12:124426621-124449867	12	NCOR2
chr4:177353307-177358258	11	NEIL3
chr1:77916054-77918273	8	NEXN
chr20:35680736-35681981	9	NFS1
chr1:160017610-160021071	12	nogene
chr1:112877729-112885053	7	nogene
chr15:51244629-51281307	10	nogene
chr11:96130348-96136347	9	nogene
chr6:157206248-157206842	6	nogene
chr7:44392296-44404583	32	NUDCD3
chr16:56748233-56758655	17	NUP93
chr7:44693824-44694576	20	OGDH
chr7:24870731-24872069	8	OSBPL3
chr13:24455016-24460136	9	PARP4
chr19:19564932-19570833	6	PBX4
chr6:78997413-79019159	5	PHIP

chr3:111903965-111920419	10	PHLDB2
chr3:138733360-138759359	5	PIK3CB
chr1:151231670-151232703	31	PIP5K1A
chr2:158533179-158621421	25	PKP4
chr19:4859589-4861411	13	PLIN3
chr1:202427039-202449171	18	PPP1R12B
chrX:345515-361590	5	PPP2R3B
chr2:119809836-119885882	6	PTPN4
chr7:105491539-105506273	19	PUS7
chr3:49967332-49975392	10	RBM6
chr6:111405469-111472140	7	REV3L
chr12:106783996-106825820	15	RIC8B
chr19:47143493-47180303	14	SAE1
chr14:30628208-30653588	38	SCFD1
chr7:140355667-140369682	54	SLC37A3
chr3:27394517-27403384	11	SLC4A7
chr13:29535818-29536202	24	SLC7A1
chr16:18845428-18850467	15	SMG1
chr17:64583460-64606640	43	SMURF2
chr14:50182462-50188700	4	SOS2
chr6:42066311-42068607	10	TAF8
chr22:46974289-46997709	30	TBC1D22A
chr8:93786222-93791319	74	TMEM67
chr21:37108391-37140673	7	TTC3
chr7:157201720-157231327	76	UBE3C
chr11:18546879-18566482	6	UEVLD
chr4:75804133-75805303	25	USO1
chr21:15808808-15831629	8	USP25
chr1:77711746-77715868	61	USP33
chr6:99476142-99510230	21	USP45
chr16:8923214-8930397	10	USP7
chr6:43549488-43567354	51	XPO5
chr16:31884497-31885256	11	ZNF267
chr11:108297286-108304852	30	ATM
chr6:35637013-35642843	25	FKBP5
chr10:92531747-92559215	11	IDE
chr20:3896863-3912634	9	PANK2
chr3:67495797-67609596	10	SUCLG2
chr3:17562018-17623913	6	TBC1D5
chr6:43553372-43567354	23	XPO5
chr16:70269617-70282784	4	AARS
chr7:17309935-17335786	13	AHR
chr16:71745143-71745614	28	AP1G1

chr10:87770951-87776427	8	ATAD1
chr5:37114959-37122488	15	C5orf42
chr1:112653597-112659779	376	CAPZA1
chr17:39490556-39526316	7	CDK12
chr7:90726566-90863269	18	CDK14
chr13:26262549-26353880	6	CDK8
chr2:101257790-101266879	6	CNOT11
chr14:101975711-101980550	5	DYNC1H1
chr11:47752915-47754664	117	FNBP4
chr16:74456648-74459789	26	GLG1
chr10:68032777-68044563	12	HERC4
chr6:53896497-53904462	11	LRRC1
chr3:15414020-15415942	14	METTL6
chr10:93310019-93310643	13	MYOF
chr14:71413947-71467247	20	nogene
chr1:229460610-229463676	16	NUP133
chr11:3768580-3782145	12	NUP98
chr4:48832619-48848446	17	OCIAD1
chr2:200931338-200937966	10	ORC2
chr11:74604691-74620089	46	POLD3
chr9:131436619-131447804	6	PRRC2B
chr10:16484336-16484816	7	PTER
chr8:94399413-94411315	12	RAD54B
chr3:196475230-196488683	12	RNF168
chr3:50175095-50175416	26	SEMA3F
chr12:131778064-131778330	12	SFSWAP
chr17:39651751-39653750	9	STARD3
chr3:196058283-196060247	19	TFRC
chr2:229836847-229840927	10	TRIP12
chr2:28861117-28865119	15	TRMT61B
chr15:42783418-42840433	12	TTBK2
chr6:169582835-169689308	8	WDR27
chr17:1364858-1365058	35	YWHAE
chr21:29061495-29062415	14	CCT8
chr17:39423326-39427790	30	MED1
chr12:5159273-5171889	16	nogene
chr6:169658275-169662424	9	WDR27
chr17:4112608-4117166	8	ZZEF1
chr14:61726705-61727655	15	HIF1A
chr11:120406117-120409450	56	ARHGEF12
chr12:6581043-6581814	10	CHD4
chr1:77783237-77815231	18	FAM73A
chr13:23864140-23886506	10	MIPEP

chr15:78886140-78887349	25	MORF4L1
chr10:125088190-125122868	15	nogene
chr7:105160506-105169265	23	SRPK2
chr2:112501205-112503181	94	TTL
chr15:50747494-50748870	85	SPPL2A
chr2:219501475-219502441	7	GMPPA
chr7:128629393-128631869	9	nogene
chr6:144490070-144493456	51	UTRN
chr15:100254135-100331054	15	ADAMTS17
chr3:138263738-138290639	10	ARMC8
chr3:32708669-32717237	11	CNOT10
chr13:110429884-110501784	15	COL4A2
chr4:2625261-2639859	45	FAM193A
chr1:212863724-212872818	43	FLVCR1
chr2:152549020-152561035	5	FMNL2
chr4:48602019-48609823	8	FRYL
chr9:83768817-83788678	26	GKAP1
chr9:4117767-4125941	28	GLIS3
chr14:61721508-61721823	76	HIF1A
chr1:61762857-61766473	10	INADL
chr9:7011697-7049200	11	KDM4C
chr7:103128482-103128792	19	NAPEPLD
chr14:75120509-75124223	10	NEK9
chr16:57059061-57059445	10	NLRC5
chr1:169230717-169298763	12	NME7
chr3:130918899-130919319	8	nogene
chr10:121960357-121974081	4	NSMCE4A
chr18:24225041-24239382	38	OSBPL1A
chr14:71019008-71036157	8	PCNX
chr4:39898388-39925933	10	PDS5A
chr3:170145422-170149244	307	PHC3
chr1:151239954-151240259	6	PIP5K1A
chr3:170291853-170299110	45	PRKCI
chr2:152662602-152669270	25	PRPF40A
chr14:73147794-73160138	31	PSEN1
chr1:31910037-31919658	21	PTP4A2
chr4:13332384-13381594	9	RAB28
chr1:64777622-64781571	14	RAVER2
chr16:70538322-70544533	9	SF3B3
chr8:98706466-98728621	20	STK3
chr17:31947616-31976614	12	SUZ12
chrX:123696020-123712908	21	THOC2
chr12:26979783-26990627	23	TM7SF3

chr9:109033306-109064567	9	TMEM245
chr20:35366556-35382025	14	UQCC1
chr4:1348089-1366431	11	UVSSA
chr1:12299201-12304728	14	VPS13D
chr14:100337061-100343387	10	WARS
chr20:47290186-47294779	10	ZMYND8
chr7:134137022-134163540	51	LRGUK
chr14:45154696-45159280	24	FANCM
chr8:23225804-23226123	5	LOC389641
chr1:40260838-40272035	14	ZMPSTE24
chr5:150541569-150545486	82	NDST1
chr16:56484760-56501025	11	BBS2
chrX:1288518-1290509	10	CSF2RA
chr6:73519336-73520056	49	EEF1A1
chr1:178776681-178785607	31	RALGPS2
chr18:49363160-49430427	8	DYM
chr1:45659027-45661225	19	GPBP1L1
chr5:65758566-65758826	19	NLN
chr10:13280975-13295606	33	PHYH
chr3:134202729-134222539	9	RYK
chr3:37379041-37416724	6	C3orf35
chr10:35032434-35049765	9	CUL2
chr12:121532805-121534590	17	KDM2B
chr9:34286616-34297114	16	KIF24
chr17:47664158-47670832	31	KPNB1
chr2:8908620-8958642	153	MBOAT2
chr1:53803927-53806517	9	NDC1
chr5:131504625-131521521	11	RAPGEF6
chr1:179337836-179341310	55	SOAT1
chr5:88202961-88240916	9	TMEM161B
chr20:35842572-35863400	7	PHF20
chr7:43881658-43887816	6	URGCP
chr9:19423861-19435084	25	ACER2
chr2:157760879-157780600	4	ACVR1
chr7:91973710-91980958	7	AKAP9
chr12:105203702-105211317	9	APPL2
chr8:130290000-130361771	17	ASAP1
chr11:46788680-46790583	16	CKAP5
chr1:230663140-230675124	107	COG2
chr20:34093674-34105545	4	EIF2S2
chr10:124681606-124706887	48	FAM53B
chr7:65960951-65967907	4	GUSB
chr7:43396813-43407731	4	HECW1

chr12:110128949-110132636	21	IFT81
chr1:226265532-226301205	23	LIN9
chr18:36248921-36260175	4	MOCOS
chr8:144179454-144180523	4	MROH1
chr1:236803427-236816543	33	MTR
chr20:58999342-58999809	48	nogene
chr11:66795041-66795396	9	nogene
chr17:47054117-47072559	10	nogene
chr20:58693736-58701158	4	NPEPL1
chr11:65287715-65295990	24	POLA2
chr13:50927406-50934999	16	RNASEH2B
chr3:185600764-185613408	15	SENP2
chr3:27379248-27403384	29	SLC4A7
chr1:28567293-28571900	8	TRNAU1AP
chr8:102342472-102361626	106	UBR5
chr17:21017941-21028674	34	USP22
chr10:1080010-1086357	20	WDR37
chr20:21354788-21368590	49	XRN2
chr1:203816925-203840374	9	ZC3H11A
chr15:80120327-80122800	340	ZFAND6
chr18:45899403-45910742	11	EPG5
chr22:31701655-31704726	11	PRR14L
chr3:57831382-57849816	7	SLMAP
chr7:66950951-66955851	13	TMEM248
chr1:92605306-92607727	80	EVI5
chr2:135709432-135710231	85	R3HDM1
chr9:137175499-137175707	17	ANAPC2
chr10:27012881-27017792	8	ANKRD26
chr11:90210535-90218184	35	CHORDC1
chr6:142820032-142837047	10	HIVEP2
chr20:36660336-36666392	8	NDRG3
chr17:51166826-51170049	9	NME1-NME2
chr6:126173395-126174911	10	nogene
chr4:523458-533981	8	PIGG
chr12:68748075-68752190	5	SLC35E3
chr1:108752256-108760085	21	STXBP3
chr17:31966146-31988497	8	SUZ12
chr5:907129-911996	8	TRIP13
chr17:41378955-41379731	30	KRT34
chr1:247864704-247868063	21	TRIM58
chr5:65224319-65242206	14	ADAMTS6
chr11:103280347-103307831	14	DYNC2H1
chr11:47722975-47723316	25	FNBP4

chr19:33462994-33493337	6	PEPD
chr2:37279745-37290999	9	PRKD3
chr6:128322038-128322310	32	PTPRK
chr7:77765494-77778814	16	RSBN1L
chr15:90217438-90219891	35	SEMA4B
chr6:43546570-43553503	8	XPO5
chr13:20019546-20036909	10	ZMYM2
chr11:108235669-108245026	23	ATM
chr5:109755328-109774965	17	MAN2A1
chr20:35069295-35078174	48	TRPC4AP
chr15:39763820-39777910	8	FSIP1
chr1:156009267-156009650	10	SSR2
chr1:155668626-155680456	9	YY1AP1
chr5:123575728-123588153	7	CSNK1G3
chr5:83104909-83258677	7	XRCC4
chr1:26257596-26259802	225	CEP85
chr1:53594107-53600278	14	GLIS1
chr10:87062655-87068157	6	GLUD1
chr15:90452774-90456315	5	IQGAP1
chr18:6080880-6093528	8	L3MBTL4
chr4:109527321-109533685	13	SEC24B
chr8:15650696-15662296	6	TUSC3
chr5:113524980-113526785	17	YTHDC2
chr1:32627507-32634110	24	ZBTB8OS
chr3:136464641-136464988	6	STAG1
chr13:98409337-98412034	10	FARP1
chr1:145405097-145420756	21	NBPF20
chr7:135584831-135587992	12	NUP205
chr8:140830471-140890769	430	PTK2
chr2:135129994-135153876	9	RAB3GAP1
chr17:62520772-62524331	49	TLK2
chr10:96544075-96576829	153	TM9SF3
chr20:34280521-34281165	12	AHCY
chr11:46493607-46508370	274	AMBRA1
chr12:31689006-31719356	11	AMN1
chr8:38114191-38121149	60	ASH2L
chr15:44331987-44338317	40	CASC4
chr16:23392379-23417121	15	COG7
chr13:113236822-113239551	7	CUL4A
chr1:85350414-85361500	12	DDAH1
chr10:12118748-12120267	95	DHTKD1
chr18:49331863-49333853	98	DYM
chr15:74671454-74675142	310	EDC3

chr1:77801330-77866391	9	FAM73A
chr2:241407536-241413421	69	FARP2
chr2:169555123-169557297	103	FASTKD1
chr11:77938650-77941251	636	INTS4
chr18:62282261-62287467	21	KIAA1468
chr4:103436934-103552602	6	LOC101929448
chr4:47875126-47885999	11	NFXL1
chr7:94061455-94061864	10	nogene
chr9:97139852-97144760	18	nogene
chr11:62625031-62625539	7	nogene
chr11:70354300-70362488	46	PPFIA1
chr5:103146526-103159328	9	PPIP5K2
chr11:76355617-76365972	8	PRKRIR
chr12:123498543-123499536	37	RILPL1
chr5:78475503-78483051	9	SCAMP1
chr2:109574580-109593119	29	10-Sep
chr3:47067069-47098081	7	SETD2
chr7:96208837-96234917	10	SLC25A13
chr11:93148652-93168171	8	SLC36A4
chr3:67400730-67409031	9	SUCLG2
chr17:37538323-37540543	5	SYNRG
chr8:140397619-140405698	257	TRAPPC9
chr9:33960825-33989126	10	UBAP2
chr3:179754731-179765848	9	USP13
chr17:42787277-42788797	14	WNK4
chr16:89733481-89737905	29	ZNF276
chr19:12524795-12528696	11	ZNF564
chr6:18159882-18171479	47	KDM1B
chr13:95115921-95188542	17	ABCC4
chr3:197130526-197142768	12	DLG1
chr10:34331116-34360259	37	PARD3
chr17:82870223-82893632	43	TBCD
chr7:122113117-122133741	125	AASS
chr3:58256561-58274815	7	ABHD6
chr2:203411284-203427269	7	ABI2
chr17:37328838-37330425	9	ACACA
chr2:9509978-9527954	29	ADAM17
chr15:100325073-100331054	8	ADAMTS17
chr21:45180329-45185091	18	ADARB1
chr21:45118563-45128573	5	ADARB1
chr1:31741623-31742217	11	ADGRB2
chr20:50902016-50904001	8	ADNP
chr22:25678815-25695214	30	ADRBK2

chr12:19473247-19493986	20	AEBP2
chr12:19462509-19500221	274	AEBP2
chr2:27057302-27059404	21	AGBL5
chr1:35901947-35902337	23	AGO1
chr8:140572811-140605914	27	AGO2
chr1:36039789-36040441	29	AGO3
chr8:6724869-6732650	39	AGPAT5
chr1:27552891-27603494	10	AHDC1
chr6:135453340-135457713	14	AHI1
chr9:4718418-4722625	12	AK3
chr17:19920035-19931978	5	AKAP10
chr10:4830674-4847299	14	AKR1E2
chr1:243563719-243843282	28	AKT3
chr10:95613741-95621251	9	ALDH18A1
chr5:126568258-126570859	43	ALDH7A1
chr11:78109441-78121174	21	ALG8
chr11:111836164-111853736	7	ALG9
chr16:89418283-89440210	7	ANKRD11
chr19:32631401-32640385	27	ANKRD27
chr3:43549719-43555469	5	ANO10
chr3:43576691-43577261	7	ANO10
chr4:79977620-80018976	9	ANTXR2
chr10:73398180-73400857	8	ANXA7
chr2:200615970-200627449	8	AOX1
chr5:115866669-115895158	43	AP3S1
chr11:43326506-43328893	9	API5
chr2:68490189-68515736	20	APLF
chr14:74683295-74692328	7	AREL1
chr22:42817141-42817857	10	ARFGAP3
chr10:31839636-31852597	11	ARHGAP12
chr11:120465236-120467308	7	ARHGEF12
chr5:73911274-73923164	6	ARHGEF28
chr5:73794401-73832459	4	ARHGEF28
chr6:156829226-156935576	28	ARID1B
chr3:93995877-94003908	11	ARL13B
chr5:78964415-78985558	17	ARSB
chr8:130057953-130061069	24	ASAP1
chr8:130134295-130169067	16	ASAP1
chr8:123344883-123361646	9	ATAD2
chr6:30627459-30628430	8	ATAT1
chr3:11331339-11364734	6	ATG7
chr3:194433771-194454392	30	ATP13A3
chr22:45700281-45729590	63	ATXN10

chr12:111513356-111518427	9	ATXN2
chr17:8204849-8205390	10	AURKB
chr18:31630958-31658089	7	B4GALT6
chr21:29326058-29329693	134	BACH1
chr2:214745066-214797117	38	BARD1
chr7:33604864-33637881	5	BBS9
chr7:33146241-33155702	84	BBS9
chr2:32575155-32594060	8	BIRC6
chr2:32439507-32445668	5	BIRC6
chr17:67928354-67932019	17	BPTF
chrX:80791853-80793772	145	BRWD3
chr7:16694833-16697061	33	BZW2
chr11:76526461-76528466	54	C11orf30
chr11:76463820-76472840	219	C11orf30
chr16:9102983-9103259	13	C16orf72
chr11:74074252-74078717	7	C2CD3
chr2:47151257-47153016	8	C2orf61
chr5:37221323-37224740	7	C5orf42
chr5:37213558-37224740	15	C5orf42
chr6:24714317-24716324	10	C6orf62
chr7:81959274-81965665	4	CACNA2D1
chr7:82064303-82117173	5	CACNA2D1
chr6:17421554-17463073	5	CAP2
chr9:75016267-75017448	89	CARNMT1
chr11:119232447-119232695	86	CBL
chr1:93256334-93264901	16	CCDC18
chr10:32472480-32518505	10	CCDC7
chr1:207757013-207770362	21	CD46
chr1:227160542-227199652	8	CDC42BPA
chr5:131385542-131391108	8	CDC42SE2
chr3:45089053-45091538	59	CDCP1
chr1:1636896-1703940	33	CDK11B
chr2:201854873-201890927	6	CDK15
chr9:120539040-120568388	14	CDK5RAP2
chr18:12999420-13042350	8	CEP192
chr5:98885577-98889238	7	CHD1
chr22:28699837-28719485	7	CHEK2
chr2:86507478-86510479	12	CHMP3
chr14:24211410-24211829	6	CHMP4A
chr9:33270622-33278225	9	CHMP5
chr10:124038514-124046724	58	CHST15
chr3:33581820-33604209	14	CLASP2
chr3:33606590-33612071	11	CLASP2

chr3:33632291-33663515	5	CLASP2
chr2:55206269-55209831	5	CLHC1
chr15:68211262-68218650	14	CLN6
chr12:70329422-70332846	13	CNOT2
chr5:180529274-180550117	16	CNOT6
chr1:246631893-246634587	10	CNST
chr9:121080305-121090405	7	CNTRL
chr16:70514334-70517740	7	COG4
chr5:75400204-75411103	36	COL4A3BP
chr20:35655201-35659014	103	CPNE1
chr8:86528535-86537646	33	CPNE3
chr14:92134010-92138347	12	CPSF2
chr11:61415665-61416519	19	CPSF7
chr3:97912166-97915736	9	CRYBG3
chr5:149511793-149525171	7	CSNK1A1
chr5:123545416-123553147	35	CSNK1G3
chr8:67111971-67118821	23	CSPP1
chr8:67154023-67159137	18	CSPP1
chr5:138886211-138887642	8	CTNNA1
chr2:224535527-224557856	632	CUL3
chr10:100256261-100260319	16	CWF19L1
chr2:203278572-203292326	11	CYP20A1
chr7:6421726-6426114	47	DAGLB
chr17:45033934-45035002	5	DCAKD
chr11:118756259-118758902	8	DDX6
chr2:73932597-73950732	9	DGUOK
chr10:12084539-12087729	5	DHTKD1
chr2:38802714-38806693	13	DHX57
chrX:154764898-154767382	14	DKC1
chr5:119110150-119116336	23	DMXL1
chr1:225185290-225240822	26	DNAH14
chr7:157358546-157385611	39	DNAJB6
chr10:21904521-21920963	118	DNAJC1
chr10:21918778-21920963	11	DNAJC1
chr10:126970701-127000307	565	DOCK1
chr3:51208773-51229609	10	DOCK3
chr13:98845923-98856031	5	DOCK9
chr20:50942030-50955285	41	DPM1
chr7:32620403-32639365	18	DPY19L1P1
chr1:161751654-161752464	10	DUSP12
chr18:49163684-49379758	8	DYM
chr2:171690146-171692894	7	DYNC1I2
chr11:103316544-103323990	18	DYNC2H1

chr20:18433651-18449069	12	DZANK1
chr1:245002120-245059458	26	EFCAB2
chr6:52423686-52479787	8	EFHC1
chr9:137775108-137782397	33	EHMT1
chr9:137743370-137744090	5	EHMT1
chr11:10803210-10804050	7	EIF4G2
chr1:20969473-21002808	29	EIF4G3
chr1:20892618-20895501	9	EIF4G3
chr1:20980333-20981227	54	EIF4G3
chr8:28137697-28162078	8	ELP3
chr2:42295124-42304551	16	EML4
chr1:8862886-8868057	11	ENO1
chr18:697239-706578	23	ENOSF1
chr2:120073177-120078581	131	EPB41L5
chr1:51421785-51448135	17	EPS15
chr17:39708320-39712448	10	ERBB2
chr5:65992709-66014725	90	ERBB2IP
chr12:1263033-1290012	69	ERC1
chr12:1027747-1444750	4	ERC1
chr11:128480190-128490576	37	ETS1
chr6:610097-637861	6	EXOC2
chr6:158783519-158789371	21	EZR
chr5:14600964-14602332	11	FAM105A
chr5:74796029-74841679	24	FAM169A
chr4:83470202-83472288	7	FAM175A
chr14:35046504-35053451	23	FAM177A1
chr10:15837203-15847943	50	FAM188A
chr15:89247628-89260843	76	FANCI
chr15:89245606-89260843	7	FANCI
chr2:58221941-58229874	12	FANCL
chr2:58159649-58165874	70	FANCL
chr12:132507251-132508350	95	FBRSL1
chr5:171910571-171997051	11	FBXW11
chr12:116928022-116949706	47	FBXW8
chrX:131749305-131784039	11	FIRRE
chr6:35637013-35672759	11	FKBP5
chr8:43059091-43072307	11	FNTA
chr6:41565744-41578081	14	FOXP4
chr22:36493632-36493972	4	FOXRED2
chr15:49235850-49328130	25	GALK2
chr10:5796762-5800705	95	GDI2
chr2:69337897-69338565	91	GFPT1
chr7:2794927-2795143	71	GNA12

chr1:109573736-109582565	9	GNAI3
chr9:77794462-77797648	423	GNAQ
chr2:156496043-156513496	23	GPD2
chr16:46897647-46900790	55	GPT2
chr12:66515618-66596927	10	GRIP1
chr13:113344862-113363910	6	GRTP1
chr7:66576754-66578727	14	GS1-124K5.11
chr7:66573384-66576932	201	GS1-124K5.11
chr7:74705163-74718941	118	GTF2I
chr2:27341947-27343578	4	GTF3C2
chr7:18585280-18634742	7	HDAC9
chr12:112319224-112319742	74	HECTD4
chr10:67990903-68014186	33	HERC4
chr9:94444963-94454070	8	HIATL1
chr10:69376933-69380095	76	HK1
chr8:42925556-42950455	9	HOOK3
chr8:42925556-42959314	15	HOOK3
chr21:43592224-43644392	10	HSF2BP
chr21:43592224-43613947	18	HSF2BP
chr2:197493323-197498850	5	HSPD1
chr5:148636988-148677235	15	HTR4
chrX:53600117-53604834	33	HUWE1
chrX:53645310-53654131	144	HUWE1
chr6:82214226-82214829	36	IBTK
chr15:41069569-41087682	7	INO80
chr5:62437273-62451933	11	IPO11
chr11:9420410-9423876	8	IPO7
chr12:26550246-26602706	9	ITPR2
chr21:33856735-33867331	6	ITSN1
chr13:30208576-30255615	9	KATNAL1
chr19:33798667-33807007	11	KCTD15
chr11:67207509-67231960	9	KDM2A
chr12:121509566-121510039	21	KDM2B
chr18:36834202-36959435	5	KIAA1328
chr8:94506499-94512089	17	KIAA1429
chr1:233346441-233376527	12	KIAA1804
chr1:10258492-10268263	8	KIF1B
chr12:8959985-8995288	8	KLRG1
chr3:122442037-122464041	9	KPNA1
chr17:47678045-47678413	29	KPNB1
chr6:108759417-108760403	13	LINC00222
chr2:186081329-186099830	7	LINC01473
chr3:66412870-66417266	6	LRIG1

chr11:46889410-46890494	5	LRP4
chr8:91189521-91200792	8	LRRC69
chr2:237708543-237720822	15	LRRFIP1
chr16:206003-208188	14	LUC7L
chr1:219193081-219211752	13	LYPLAL1
chr1:9931890-9935627	170	LZIC
chr7:1898199-2014642	43	MAD1L1
chr7:1898199-1957719	6	MAD1L1
chr5:109817272-109830980	12	MAN2A1
chr19:4117418-4117629	5	MAP2K2
chr15:67630887-67664645	60	MAP2K5
chr2:227328677-227332588	11	MFF
chr11:12204249-12216319	15	MICAL2
chr13:23836239-23881787	8	MIPEP
chr7:131411305-131429145	20	MKLN1
chr4:153350063-153358622	9	MND1
chr17:43816037-43824005	29	MPP3
chr5:80725452-80741548	19	MSH3
chr14:64412471-64424931	14	MTHFD1
chr14:64417887-64419925	34	MTHFD1
chr14:64431531-64441453	15	MTHFD1
chrX:150699194-150750843	15	MTMR1
chr15:30974313-30976955	29	MTMR10
chr8:66595582-66602523	14	MYBL1
chr17:8518630-8535931	9	MYH10
chr15:71848844-71888116	5	MYO9A
chr2:15374607-15402301	11	NBAS
chr1:144439828-144450870	9	NBPF15
chr19:3192469-3196277	9	NCLN
chr17:16071408-16073569	32	NCOR1
chr1:53825797-53832591	9	NDC1
chr4:169580841-169599197	14	NEK1
chr3:131029612-131080588	6	NEK11
chr17:31200421-31206371	9	NF1
chr16:28955977-28959100	24	NFATC2IP
chr2:177230813-177234271	12	NFE2L2
chr9:33318730-33319127	6	NFX1
chr10:94356534-94358215	16	NOC3L
chr4:139043073-139043343	16	NOCT
chr12:50153876-50163308	8	nogene
chr4:165077031-165077551	18	nogene
chr7:156986371-156986609	5	nogene
chr4:103536345-103569402	25	nogene

chr7:66898178-66900897	11	nogene
chr6:149734100-149734547	8	nogene
chr20:19908398-19908959	8	nogene
chr13:107466014-107467250	7	nogene
chr4:128037170-128037460	5	nogene
chr19:27472554-27521041	10	nogene
chr4:23078613-23083788	14	nogene
chr10:245900-263658	10	nogene
chrX:152457249-152458262	110	nogene
chr7:145293-148714	9	nogene
chr9:34680905-34703038	6	nogene
chr9:96895895-96903108	5	nogene
chr13:50027362-50045232	10	nogene
chr17:28722030-28722393	12	nogene
chr7:128574719-128580094	36	nogene
chrX:130071101-130071727	4	nogene
chr2:231497094-231503204	4	nogene
chr17:517939-518459	14	nogene
chr13:28701789-28717655	11	nogene
chr20:45836398-45838988	12	nogene
chr9:128308833-128309271	9	nogene
chr5:37533137-37534043	9	nogene
chr1:9893008-9894243	6	nogene
chr6:140804234-140816230	6	nogene
chrX:3822373-3826729	9	nogene
chr3:8840195-8852948	16	nogene
chrM:3789-4053	97	nogene
chrX:152834650-152835121	7	nogene
chr7:149585129-149608783	10	nogene
chr1:113024967-113025415	8	nogene
chr10:119575690-119580538	14	nogene
chr22:37977159-37977328	4	nogene
chr2:111233627-111248241	12	nogene
chr18:45611490-45611797	8	nogene
chr6:141772872-141910011	4	nogene
chr14:96943516-96945386	5	nogene
chr1:20775723-20776005	31	nogene
chrX:119539037-119539709	36	nogene
chr7:128631679-128638897	9	nogene
chr22:37612696-37613446	34	nogene
chr11:118582360-118582804	5	nogene
chr5:172254401-172255817	5	nogene
chr1:26716113-26716720	7	nogene

chrX:11055935-11110981	90	nogene
chr8:128009589-128048609	18	nogene
chr17:81596115-81600427	27	NPLOC4
chr6:3006467-3012674	26	NQO2
chr7:129657342-129677758	4	NRF1
chr5:6622015-6625669	67	NSUN2
chr5:37301436-37305210	23	NUP155
chr10:13110273-13122487	15	OPTN
chr2:200942684-200958133	11	ORC2
chr1:51750144-51761971	11	OSBPL9
chr5:14678680-14681607	49	OTULIN
chr5:14678680-14681624	8	OTULIN
chr10:73046924-73074915	9	P4HA1
chr17:81855141-81855586	242	P4HB
chr13:28197184-28220378	57	PAN3
chr13:24459043-24469110	51	PARP4
chr22:44093927-44119140	7	PARVB
chr15:43768488-43769646	5	PDIA3
chr4:94523723-94586444	10	PDLIM5
chr16:2557702-2561522	8	PDPK1
chr6:107324555-107334332	15	PDSS2
chr5:115230975-115238009	15	PGGT1B
chr17:78392475-78404089	32	PGS1
chr7:11040671-11051780	15	PHF14
chr6:79015081-79019159	14	PHIP
chr16:71702597-71714801	51	PHLPP2
chr1:113724758-113738799	16	PHTF1
chr11:86007541-86014963	6	PICALM
chr11:85990249-86031611	35	PICALM
chr4:505717-506866	7	PIGG
chr10:22591628-22609717	38	PIP4K2A
chr10:22567850-22591781	21	PIP4K2A
chr7:30074070-30078778	19	PLEKHA8
chr2:43745865-43765726	18	PLEKHH2
chr2:55640626-55643425	5	PNPT1
chrX:24821451-24843677	23	POLA1
chr11:65267476-65275998	11	POLA2
chr6:43597695-43601091	105	POLH
chr6:43587271-43597865	7	POLH
chr6:43582315-43583141	25	POLH
chr7:102539605-102569924	7	POLR2J3
chr12:42351884-42375074	8	PPHLN1
chr2:169603641-169608758	42	PPIG

chr5:134197775-134206131	27	PPP2CA
chr11:111752158-111755450	6	PPP2R1B
chrX:361404-361590	10	PPP2R3B
chr8:47935008-47936517	7	PRKDC
chr1:150334934-150338326	6	PRPF3
chr15:55624341-55627128	4	PRTG
chr9:120820838-120821464	10	PSMD5
chr20:1118752-1135306	9	PSMF1
chr10:87864453-87961118	7	PTEN
chr8:140846259-140864399	1239	PTK2
chr8:140735250-140743330	6	PTK2
chr8:140789473-140921132	9	PTK2
chr18:7926567-7955414	14	PTPRM
chr1:31005852-31007102	514	PUM1
chr7:105459167-105495253	16	PUS7
chr7:105481051-105502564	25	PUS7
chr9:136224006-136226874	79	QSOX2
chr12:57298089-57310463	8	R3HDM2
chr17:5335183-5338138	6	RABEP1
chr9:122957010-122986419	54	RABGAP1
chr7:66712166-66713468	7	RABGEF1
chr2:113628549-113633060	8	RABL2A
chr14:67823541-67887204	74	RAD51B
chr14:35595633-35635598	27	RALGAPA1
chr5:170916464-171028989	8	RANBP17
chr5:87331347-87338091	112	RASA1
chr13:114052051-114057441	8	RASA3
chr20:37044085-37044250	9	RBL1
chr16:53439015-53447106	69	RBL2
chr5:146254942-146258593	246	RBM27
chr7:155763811-155774647	10	RBM33
chr3:50068689-50075330	9	RBM6
chr1:8465924-8557523	5	RERE
chr2:99418827-99435941	12	REV1
chr16:74632522-74636577	76	RFWD3
chr2:151422949-151438746	7	RIF1
chr8:100264033-100288267	6	RNF19A
chr18:21039471-21044186	19	ROCK1
chr14:90943077-91001159	40	RPS6KA5
chr1:213077695-213088030	10	RPS6KC1
chr16:15063195-15073080	5	RRN3
chr6:43024984-43027477	35	RRP36
chr11:77725544-77764689	45	RSF1

chr11:77740730-77747128	26	RSF1
chr11:77685104-77693611	47	RSF1
chr15:41471171-41477515	29	RTF1
chr9:35558196-35559272	12	RUSC2
chr12:55794358-55800900	14	SARNP
chr12:123334052-123350441	5	SBNO1
chr9:124999890-125003570	8	SCAI
chr7:29926451-29936721	9	SCRN1
chr3:195688532-195699207	76	SDHAP2
chr14:39064912-39076093	4	SEC23A
chr14:39055142-39067296	6	SEC23A
chr5:134674614-134679728	9	SEC24A
chr4:109462900-109463644	75	SEC24B
chr4:82878729-82881940	166	SEC31A
chr6:107904628-107924932	52	SEC63
chr2:241342991-241348191	63	2-Sep
chr6:2833873-2840594	8	SERPINB1
chr3:47056820-47057490	11	SETD2
chr16:70560468-70564050	41	SF3B3
chr4:107895309-107903386	6	SGMS2
chr11:116863667-116873636	12	SIK3
chr19:38109685-38110384	11	SIPA1L3
chr4:1696201-1700070	5	SLBP
chr6:44227259-44232428	5	SLC29A1
chr2:32171286-32184338	13	SLC30A6
chr9:96336716-96352109	39	SLC35D2
chr11:62870744-62871669	11	SLC3A2
chr16:68266592-68291661	12	SLC7A6
chr4:103028746-103047226	4	SLC9B2
chr6:70754979-70798737	14	SMAP1
chr3:47706408-47720735	7	SMARCC1
chr3:47638724-47678311	27	SMARCC1
chr19:43744771-43750747	8	SMG9
chr17:64583460-64598490	5	SMURF2
chr15:24962113-24968082	32	SNRPN
chr5:122795265-122803613	30	SNX2
chr16:21338920-21352138	12	SNX29P1
chr1:179341027-179343635	13	SOAT1
chr20:36804734-36805957	43	SOGA1
chr2:39056701-39058804	4	SOS1
chr4:123028185-123028395	8	SPATA5
chr2:200412310-200419496	13	SPATS2L
chr15:64976474-64983593	25	SPG21

chr3:113499438-113506605	32	SPICE1
chr18:12506476-12546904	5	SPIRE1
chr9:128630320-128632955	16	SPTAN1
chr7:105293533-105297533	82	SRPK2
chr18:26018018-26078160	4	SS18
chr1:84655831-84671308	31	SSX2IP
chr3:136568764-136591549	17	STAG1
chr12:130808630-130827267	11	STX2
chr9:127661101-127666296	6	STXBP1
chr3:67495797-67508903	105	SUCLG2
chr1:172555868-172557794	89	SUCO
chr1:85164525-85164757	6	SYDE2
chr14:63961524-63976727	5	SYNE2
chr17:37458523-37465541	8	TADA2A
chrX:71397252-71408151	9	TAF1
chr3:11807832-11839314	9	TAMM41
chr2:159065895-159097834	34	TANC1
chr20:13528432-13587370	15	TASP1
chr10:94441895-94497172	7	TBC1D12
chr4:26613168-26620688	7	TBC1D19
chr3:17508473-17576549	4	TBC1D5
chr17:82756164-82893632	9	TBCD
chr17:47698594-47699697	9	TBKBP1
chrX:9684042-9697429	24	TBL1X
chr20:62854015-62857638	122	TCFL5
chr19:41348294-41348455	9	TGFB1
chr2:43291695-43293213	7	THADA
chrX:123686547-123712908	15	THOC2
chr2:70227734-70236175	8	TIA1
chr10:49952125-49955060	28	TIMM23B
chr15:66349059-66352207	6	TIPIN
chr6:43498922-43503706	4	TJAP1
chr15:29761138-29762438	10	TJP1
chr9:79709622-79721888	8	TLE4
chr17:62520772-62536337	5	TLK2
chr13:99529466-99537863	10	TM9SF2
chr9:109057190-109073438	21	TMEM245
chr9:105705596-105722621	135	TMEM38B
chr7:129014978-129018157	1329	TNPO3
chr7:128978982-128980031	28	TNPO3
chr17:54947260-54950126	6	TOM1L1
chr1:223810430-223814353	9	TP53BP2
chr7:66240324-66241270	438	TPST1

chr22:16602063-16614178	4	TPTEP1
chr14:102816738-102830472	19	TRAF3
chr18:31897785-31917667	18	TRAPPC8
chr1:228396946-228402161	4	TRIM11
chr7:138504289-138525357	9	TRIM24
chr20:35044504-35049994	19	TRPC4AP
chr7:98925111-98927366	15	TRRAP
chr3:12516610-12532773	14	TSEN2
chr15:42810613-42830078	37	TTBK2
chr18:24064139-24114647	5	TTC39C
chr18:24064139-24083081	414	TTC39C
chr17:59863663-59878334	9	TUBD1
chr15:43398037-43401850	40	TUBGCP4
chr15:23026115-23037152	10	TUBGCP5
chr12:104311289-104315896	36	TXNRD1
chr9:33948373-33953474	175	UBAP2
chr9:33944364-33948587	25	UBAP2
chr17:4289229-4303745	8	UBE2G1
chr4:39737419-39755739	272	UBE2K
chr15:43056343-43059192	8	UBR1
chr1:19176591-19187540	4	UBR4
chr8:102342472-102347092	35	UBR5
chr8:102329089-102346395	49	UBR5
chr9:6420911-6434173	441	UHRF2
chr13:27069110-27075388	10	USP12
chr13:27069089-27090158	4	USP12
chr17:21015751-21021226	5	USP22
chr15:63532646-63563008	299	USP3
chr16:23102318-23120246	16	USP31
chr17:60236137-60271481	8	USP32
chr2:218509978-218530040	20	USP37
chr2:85619219-85636130	7	USP39
chr3:49324701-49325845	39	USP4
chr10:11518534-11597717	23	USP6NL
chr6:144421877-144429741	23	UTRN
chr16:70731494-70744579	16	VAC14
chr8:99170038-99275254	16	VPS13B
chr8:99142973-99156743	6	VPS13B
chr16:46680670-46683606	29	VPS35
chr3:184924861-185024389	4	VPS8
chr14:102194937-102197847	166	WDR20
chr14:102194937-102198514	5	WDR20
chr1:224398096-224401069	5	WDR26

chr10:1079555-1080476	8	WDR37
chr12:121931745-121942975	8	WDR66
chr18:56918071-56939393	12	WDR7
chr17:68428828-68434626	10	WIPI1
chr6:43568710-43573601	15	XPO5
chr16:28146093-28169909	12	XPO6
chr1:32779381-32781145	13	YARS
chr4:4313040-4315970	55	ZBTB49
chr19:47084344-47085414	10	ZC3H4
chr16:11763477-11765685	21	ZC3H7A
chr7:6578500-6585260	11	ZDHHC4
chr15:80098415-80131293	21	ZFAND6
chr16:72957426-72960194	10	ZFHX3
chr20:63708552-63709164	5	ZGPAT
chr7:99512452-99520304	5	ZKSCAN5
chr5:43122038-43179950	7	ZNF131
chr10:42631928-42636972	8	ZNF33B
chr9:106923353-106935621	8	ZNF462
chr19:32356615-32360618	48	ZNF507
chr19:37155864-37170054	4	ZNF585A
chr15:66528851-66533013	17	ZWILCH
chr17:4021128-4025118	31	ZZEF1
chr6:130311290-130313093	167	SAMD3
chr2:32377587-32401385	4	BIRC6
chr14:39291018-39295029	14	CTAGE5
chr14:76171842-76178042	6	GPATCH2L
chr2:171325840-171339554	6	METTL8
chr1:146968447-146972960	67	NBPF12
chr16:31700661-31706999	9	nogene
chrX:132239619-132283032	4	RAP2C-AS1
chr9:36052264-36065624	17	RECK
chr16:30769185-30769600	4	RNF40
chr19:45821383-45827918	4	SYMPK
chr14:102870184-102897401	19	TRAF3
chr5:14304460-14316743	10	TRIO
chr4:87114366-87115299	10	AFF1
chr8:140572811-140585311	252	AGO2
chr5:73554321-73561288	13	ANKRA2
chr19:32615657-32643631	14	ANKRD27
chr15:60360940-60382441	34	ANXA2
chr7:100103408-100104154	35	AP4M1
chr8:123325892-123336532	21	ATAD2
chr10:32451591-32485144	8	CCDC7

chr1:1649498-1712400	6	CDK11B
chr14:80743074-80778046	153	CEP128
chr9:128185546-128185776	9	CIZ1
chr15:22872824-22873729	33	CYFIP1
chr14:33925617-33927033	6	EGLN3
chr7:100818519-100820296	7	EPHB4
chr12:56099647-56100245	9	ERBB3
chr1:42227882-42311110	39	FOXJ3
chr8:11748918-11757083	11	GATA4
chr9:124898548-124900547	15	GOLGA1
chr4:108009758-108027760	20	HADH
chr1:155921374-155925843	47	KIAA0907
chr1:46580529-46594280	16	MKNK1
chr2:151278827-151282954	8	NMI
chr7:32833007-32838666	77	nogene
chr14:24170833-24171379	24	nogene
chr18:47091173-47097404	19	nogene
chr12:27426131-27426317	5	nogene
chr5:177280564-177283928	25	NSD1
chr10:34493285-34517159	8	PARD3
chr5:31995575-32010482	9	PDZD2
chr15:77252366-77286504	53	PEAK1
chr3:170097171-170122744	4	PHC3
chr1:88804390-88813733	8	PKN2
chr18:54271359-54284013	46	POLI
chr9:131459163-131459356	10	PRRC2B
chr1:226885537-226889049	8	PSEN2
chr9:112250928-112252788	74	PTBP3
chr8:103766226-103809321	4	RIMS2
chr1:92333390-92345914	43	RPAP2
chr3:47425484-47443091	8	SCAP
chr7:85121740-85224441	8	SEMA3D
chr4:151143909-151149561	7	SH3D19
chrX:46620976-46653705	17	SLC9A7
chr1:227758994-227759485	12	SNAP47
chr16:2756333-2896771	26	SRRM2
chr1:235437321-235441882	6	TBCE
chr3:177049996-177065022	5	TBL1XR1
chrX:123665641-123671761	21	THOC2
chr15:42244049-42268032	6	TMEM87A
chr11:35725923-35735445	13	TRIM44
chr11:62735381-62738490	23	TTC9C
chr4:39733358-39745810	10	UBE2K

chr7:139266332-139272440	37	UBN2
chr13:96013306-96031971	11	UGGT2
chr2:61284874-61296925	11	USP34
chr18:9936109-9945058	5	VAPA
chr8:99575657-99577633	36	VPS13B
chr2:127768188-127771004	50	WDR33
chrX:54232808-54239099	10	WNK3
chr7:130026157-130049144	8	ZC3HC1
chr17:30449552-30451846	10	CPD
chr1:197642710-197652315	10	DENND1B
chr16:58709416-58716813	9	GOT2
chr3:197983942-197996282	10	LMLN
chr17:64561499-64586170	24	SMURF2
chrX:67882125-67921219	5	nogene
chr7:66551519-66554837	5	GS1-124K5.11
chr12:122579904-122582985	13	KNTC1
chr1:11989164-11992690	32	MFN2
chr11:106009582-106011067	17	MSANTD4
chr2:200886719-200887124	7	nogene
chr14:85978969-86041833	8	nogene
chr3:17586352-17625224	10	nogene
chr11:73677720-73679720	13	RAB6A
chr6:88769773-88891915	7	RNGTT
chr5:69104630-69114496	45	SLC30A5
chr12:63983946-63990072	16	SRGAP1
chr16:3679718-3690985	5	TRAP1
chr1:19176591-19177743	7	UBR4
chr11:18570620-18578808	16	UEVLD
chr14:31380466-31383771	17	HEATR5A
chr6:75835900-75844977	11	MYO6
chr9:100239998-100272982	5	nogene
chr2:135661269-135680324	8	R3HDM1
chr6:145919347-145933178	17	SHPRH
chr17:51014231-51021365	34	SPAG9
chr17:82884144-82909307	10	TBCD
chr19:4944131-4944450	39	UHRF1
chr3:51470928-51492513	11	VPRBP
chr6:85517755-85530275	87	SNX14
chr15:72555270-72561549	10	ARIH1
chr20:37732878-37737484	10	CTNNBL1
chr1:224236505-224268133	46	NVL
chr1:161041323-161043360	10	USF1
chr2:134336216-134338420	11	MGAT5

chr4:55417071-55417985	12	TMEM165
chr2:74003109-74048411	12	TET3
chr6:24502522-24533687	6	ALDH5A1
chr12:31479588-31499671	27	DENND5B
chrX:131692437-131794466	20	FIRRE
chr8:101199194-101201743	19	ZNF706
chr2:73419122-73432291	38	ALMS1
chr10:92081319-92091944	33	CPEB3
chr2:43957384-43975217	6	LRPPRC
chr1:84937754-84965708	12	MCOLN2
chrX:68081700-68119332	8	OPHN1
chr15:43768488-43770580	11	PDIA3
chr3:128884652-128904505	6	ACAD9
chr5:65188015-65197151	35	ADAMTS6
chr16:89977677-89978878	7	AFG3L1P
chr1:36039789-36043548	12	AGO3
chr1:246913231-246918377	107	AHCTF1
chr15:85521427-85543955	25	AKAP13
chr19:49458485-49459848	13	ALDH16A1
chr5:126570781-126593404	6	ALDH7A1
chr10:27092405-27098326	4	ANKRD26
chr5:10618332-10638168	8	ANKRD33B
chr5:112780789-112801383	18	APC
chr21:25975069-25997416	7	APP
chr4:147857552-147866816	12	ARHGAP10
chr6:129629352-129642018	8	ARHGAP18
chr13:111153904-111244294	11	ARHGEF7
chr3:48979481-48983292	8	ARIH2
chr8:130358016-130427822	25	ASAP1
chr8:61676139-61684188	16	ASPH
chr11:108227775-108235834	11	ATM
chrX:77557450-77574358	6	ATRX
chr16:88018427-88038011	147	BANP
chr15:90769132-90769586	25	BLM
chr10:73727292-73728587	14	BMS1P4
chr17:67945408-67948306	30	BPTF
chr11:93747296-93754037	4	C11orf54
chr11:74012577-74013525	5	C2CD3
chr5:37114959-37121784	14	C5orf42
chr7:82060427-82117173	18	CACNA2D1
chr17:63751939-63754518	12	CCDC47
chr10:59832524-59852702	49	CCDC6
chr16:2439352-2439826	5	CCNF

chr7:90747680-90863269	13	CDK14
chr17:15605222-15615809	10	CDRT1
chr1:243186258-243225321	16	CEP170
chr16:53228982-53243016	36	CHD9
chr12:119850173-119857698	8	CIT
chr2:121450912-121458975	12	CLASP1
chr16:58560211-58560362	13	CNOT1
chr3:32703867-32704972	7	CNOT10
chr12:69258589-69262562	7	CPSF6
chr9:129098012-129098650	8	CRAT
chr16:3736035-3745354	8	CREBBP
chr10:125110989-125133612	8	CTBP2
chr11:57794009-57796992	9	CTNND1
chr10:35013698-35031619	52	CUL2
chr1:167987494-168004793	13	DCAF6
chr3:182963880-182965753	9	DCUN1D1
chr22:19063201-19089490	21	DGCR2
chrX:2266739-2291603	11	DHRSX
chr20:36496704-36500611	19	DLGAP4
chr3:51137442-51146630	13	DOCK3
chr15:65500605-65512564	4	DPP8
chr1:212042992-212047417	9	DTL
chr14:102040235-102044698	12	DYNC1H1
chr8:85202146-85209409	9	E2F5
chr2:62747394-62771392	30	EHBP1
chr8:108234997-108240075	12	EIF3E
chr8:116706337-116726172	9	EIF3H
chr3:186784696-186784935	7	EIF4A2
chr14:103336676-103339803	41	EIF5
chr4:139125163-139168042	14	ELF2
chr1:205616477-205623891	145	ELK4
chr8:28129501-28137891	12	ELP3
chr12:1289851-1319288	13	ERC1
chr12:22643762-22671355	14	ETNK1
chr6:592468-619543	29	EXOC2
chr6:576756-599225	10	EXOC2
chr7:22983943-22998161	26	FAM126A
chr10:50090775-50093917	7	FAM21A
chrX:14843659-14845286	8	FANCB
chr5:15927889-15928501	5	FBXL7
chr2:105373558-105386540	8	FHL2
chr13:49138746-49145935	68	FNDC3A
chr10:93684055-93687449	30	FRA10AC1

chr14:51689690-51708233	12	FRMD6
chr1:77955254-77956700	29	FUBP1
chr5:151799211-151800869	8	G3BP1
chr3:81577924-81705613	8	GBE1
chr3:158665336-158691648	11	GFM1
chr2:69363544-69374113	53	GFPT1
chr1:1815755-1839238	926	GNB1
chr12:132807176-132822311	65	GOLGA3
chr9:86033281-86036507	11	GOLM1
chr17:30495425-30520021	12	GOSR1
chr9:130079629-130092381	17	GPR107
chr1:108901789-108904254	13	GPSM2
chr7:66573384-66578727	14	GS1-124K5.11
chr5:21491320-21502203	4	GUSBP1
chr2:171965337-171966949	15	HAT1
chrX:72351732-72464731	10	HDAC8
chr8:42964310-42968214	20	HOOK3
chr2:24312211-24315224	9	ITSN2
chr17:46094559-46172232	5	KANSL1
chr18:41501119-41525526	6	KC6
chr17:6594971-6600458	18	KIAA0753
chr2:61068848-61118116	7	KIAA1841
chr13:49705620-49706372	18	KPNA3
chr6:149676259-149678745	30	LATS1
chr10:65571424-65615761	17	LINC01515
chr22:47686199-47686938	6	LOC284930
chr1:211778917-211793190	47	LPGAT1
chr4:150828179-150831976	8	LRBA
chr7:2222574-2230727	14	MAD1L1
chr7:1898199-1980541	17	MAD1L1
chr3:185437446-185466963	9	MAP3K13
chr9:125583405-125585727	7	MAPKAP1
chr19:18121684-18124774	10	MAST3
chr18:54188873-54205157	10	MBD2
chr21:46273387-46277717	20	MCM3AP
chr17:2438108-2473664	14	METTL16
chr8:17077782-17090584	8	MICU3
chr10:87508335-87521169	41	MINPP1
chr13:23836239-23864189	21	MIPEP
chr14:37240432-37247907	15	MIPOL1
chr14:37308055-37369624	16	MIPOL1
chr17:43808955-43818110	4	MPP3
chr2:42570436-42609584	30	MTA3

chr8:97713661-97723035	82	MTDH
chrX:150638942-150649901	41	MTM1
chr6:73466208-73466606	167	MTO1
chr1:11212311-11216234	5	MTOR
chr18:3253232-3255962	9	MYL12A
chr3:123793676-123876617	14	MYLK
chr15:71999850-72027793	25	MYO9A
chr12:76059797-76068994	6	NAP1L1
chr2:96364480-96367373	10	NCAPH
chr9:97653797-97656085	48	NCBP1
chr3:172635915-172648114	34	NCEH1
chr5:150541569-150551855	7	NDST1
chr2:206141940-206149925	5	NDUFS1
chr12:96909751-96912817	5	NEDD1
chr14:75117194-75121174	15	NEK9
chr12:104120399-104135532	6	NFYB
chr5:36953617-36961583	5	NIPBL
chr5:65780178-65788484	7	NLN
chr3:37275818-37276517	13	nogene
chr3:40309870-40312169	21	nogene
chr3:51541588-51552063	9	nogene
chr6:30723619-30723775	10	nogene
chr2:44174761-44180397	16	nogene
chr10:5699524-5714207	28	nogene
chr1:7777158-7778165	9	nogene
chr1:187326920-187443726	6	nogene
chr22:41474879-41475610	6	nogene
chr1:120533815-120535809	25	nogene
chr3:196714672-196714862	8	nogene
chr19:21062605-21077216	4	nogene
chr10:31355143-31358333	12	nogene
chr12:10216278-10216634	4	nogene
chr8:123378624-123378901	17	nogene
chr11:61374864-61375417	14	nogene
chr10:38066360-38066854	13	nogene
chr7:66520594-66523179	5	nogene
chr4:2952274-2956811	24	NOP14
chr1:119996996-120029987	25	NOTCH2
chr11:108172198-108177090	30	NPAT
chr17:41828789-41830890	30	NT5C3B
chr6:17688395-17688618	17	NUP153
chr15:41356038-41358258	23	NUSAP1
chr1:224233200-224268133	18	NVL

chr1:58506059-58536741	241	OMA1
chr11:69667894-69673321	10	ORAOV1
chr5:38903881-38917622	8	OSMR
chr16:14552020-14604226	7	PARN
chr13:24469893-24478276	6	PARP4
chr21:46363479-46367139	4	PCNT
chr14:70976941-70995925	27	PCNX
chr1:233258027-233259344	7	PCNXL2
chr14:60114699-60116060	11	PCNXL4
chr4:156850220-156861489	42	PDGFC
chr11:34947505-34970286	125	PDHX
chr18:62895010-62920114	7	PHLPP1
chr8:10820192-10834775	94	PINX1
chr11:94574998-94583569	11	PIWIL4
chr11:94583447-94608686	7	PIWIL4
chr4:88051990-88065879	6	PKD2
chr1:15719733-15721388	36	PLEKHM2
chr2:207828222-207861262	6	PLEKHM3
chr15:75043440-75048721	10	PPCDC
chr2:44194197-44209327	13	PPM1B
chr2:48474682-48480016	10	PPP1R21
chrX:338603-341931	54	PPP2R3B
chr14:91468894-91470995	7	PPP4R3A
chr12:11047019-11121180	23	PRH1
chr12:56746780-56751195	29	PRIM1
chrX:3626418-3641971	16	PRKX
chr8:140700890-140717709	51	PTK2
chr9:94085250-94088263	17	PTPDC1
chr20:2923206-2988474	9	PTPRA
chr5:170896049-170924550	9	RANBP17
chr17:40330856-40331396	11	RARA
chr5:168500590-168502105	65	RARS
chr1:64768655-64789514	19	RAVER2
chr5:146228945-146237432	5	RBM27
chr11:110263959-110279756	10	RDX
chr16:74644361-74652122	30	RFWD3
chr12:122508217-122511188	6	RSRC2
chr3:128035378-128087808	9	RUVBL1
chr20:36916721-36919519	6	SAMHD1
chr15:76574157-76683061	14	SCAPER
chr4:82878729-82881937	69	SEC31A
chr3:47105996-47116754	47	SETD2
chr9:132295871-132311856	10	SETX

chr3:52930328-52969258	14	SFMBT1
chr1:86722539-86724405	5	SH3GLB1
chr10:110951607-110965061	21	SHOC2
chr10:116951908-116960230	11	SHTN1
chr1:115983548-115992243	7	SLC22A15
chr14:69441831-69442266	29	SLC39A9
chrX:136012957-136016758	26	SLC9A6
chrX:46620976-46648797	12	SLC9A7
chr18:12420324-12421629	37	SLMO1
chr9:33060464-33062177	7	SMU1
chr17:64578491-64586170	28	SMURF2
chr17:64571797-64598490	26	SMURF2
chr15:45068180-45069052	16	SORD
chr2:32087491-32089605	5	SPAST
chr1:48385308-48403882	34	SPATA6
chr12:64062916-64087026	26	SRGAP1
chr4:56478378-56484864	12	SRP72
chr20:62162751-62163622	20	SS18L1
chr12:108817037-108852678	6	SSH1
chr1:112591524-112611003	17	ST7L
chr3:136472412-136502779	9	STAG1
chr9:123173676-123184298	7	STRBP
chr14:21366438-21373430	7	SUPT16H
chr17:31940285-31973231	127	SUZ12
chr22:46974289-47037198	69	TBC1D22A
chr6:121279120-121283910	11	TBC1D32
chr17:82763964-82809782	7	TBCD
chr22:42209650-42215341	50	TCF20
chr11:33065829-33068859	4	TCP11L1
chrX:123625911-123627968	14	THOC2
chr14:58410842-58411971	5	TIMM9
chr13:113495501-113520733	12	TMCO3
chr12:111933885-111991889	10	TMEM116
chr1:95136692-95151419	9	TMEM56
chr15:43148754-43169677	6	TMEM62
chr8:93763841-93785378	8	TMEM67
chrX:155506897-155537118	37	TMLHE
chr5:14330777-14336727	212	TRIO
chr3:142736378-142785040	20	TRPC1
chr9:122031722-122040479	7	TTLL11
chr19:34433347-34464131	9	UBA2
chr19:34430575-34467014	21	UBA2
chr1:154251040-154251653	89	UBAP2L

chr1:1256991-1257310	227	UBE2J2
chr3:33409235-33425741	80	UBP1
chr6:42637010-42642481	10	UBR2
chr3:196361843-196362687	28	UBXN7
chr11:18544622-18558330	11	UEVLD
chr13:95832926-95859675	8	UGGT2
chr5:176955958-176982623	8	UIMC1
chr3:124737567-124744203	48	UMPS
chr15:63532646-63559970	17	USP3
chr3:49302383-49305888	21	USP4
chr10:74097203-74101097	23	VCL
chr3:51440969-51483836	10	VPRBP
chr7:38789800-38830328	8	VPS41
chr1:224393827-224399034	10	WDR26
chr12:121942533-121942975	5	WDR66
chr6:2779262-2783561	36	WRNIP1
chr5:113553766-113563492	50	YTHDC2
chr1:155675009-155680456	14	YY1AP1
chr1:52445786-52461769	10	ZCCHC11
chr18:62539680-62576652	14	ZCCHC2
chr20:25632157-25634170	30	ZNF337-AS1
chr19:36472910-36473458	9	ZNF566
chr1:90916639-90918154	52	ZNF644
chr19:58089864-58090386	10	ZSCAN18
chr2:218667911-218668679	4	RNF25
chr19:57863364-57863759	49	nogene
chr5:109729341-109789527	10	MAN2A1
chr12:124913039-124913267	26	nogene
chr16:71658232-71679535	73	PHLPP2
chr5:170919440-170968377	7	RANBP17
chr1:92320598-92345914	21	RPAP2
chr4:75967276-75971458	7	SDAD1
chr20:44509890-44514040	26	SERINC3
chr5:110638197-110642371	6	TMEM232
chr5:154924468-154936022	5	GEMIN5
chr5:179841906-179853437	16	C5orf45
chr16:14644273-14644609	85	BFAR
chr8:38247712-38252287	10	DDHD2
chr7:80798091-80805758	39	SEMA3C
chr20:25634048-25645648	105	ZNF337-AS1
chr12:31451939-31495919	16	DENND5B
chr3:142365046-142370620	7	XRN1
chr8:125102050-125102487	18	NSMCE2

chr2:37289355-37293271	8	PRKD3
chr1:179112308-179121867	8	ABL2
chr9:97994630-97998678	87	ANP32B
chr13:106557047-106559657	7	ARGLU1
chr22:17499409-17500735	4	CECR2
chr15:92948951-92967513	4	CHD2
chr22:38494020-38501280	16	DDX17
chr18:49329572-49333853	51	DYM
chr8:38424508-38426245	25	FGFR1
chr3:125027204-125029488	29	HEG1
chr11:119131534-119133219	96	HINFP
chr19:47416651-47417350	535	MEIS3
chr19:3205938-3206425	7	NCLN
chr10:73249187-73249967	24	nogene
chr3:28251711-28253765	4	nogene
chr14:96520054-96528006	50	PAPOLA
chr12:107743196-107746424	14	PRDM4
chr10:87875404-87933251	7	PTEN
chr15:75490207-75527261	30	PTPN9
chr2:86688231-86701949	6	RNF103-CHMP3
chr19:45686533-45687727	19	SNRPD2
chr1:112591524-112598086	7	ST7L
chr13:75349169-75359858	17	TBC1D4
chr2:218546220-218562813	14	USP37
chr2:218476839-218482234	9	USP37
chr19:57776542-57780146	32	ZNF586
chr2:111862388-111878971	15	ANAPC1
chr5:79619611-79623489	19	PAPD4
chr5:65291328-65473952	16	ADAMTS6
chr1:155415743-155477958	7	ASH1L
chr1:235494680-235496308	63	B3GALNT2
chr9:137790847-137800984	46	EHMT1
chr5:154033790-154034967	585	FAM114A2
chr3:9418732-9418882	8	nogene
chr16:50211322-50217663	11	PAPD5
chr13:79368729-79371930	10	RBM26
chr5:146233449-146237432	21	RBM27
chr22:40844838-40850880	20	ST13
chr8:39054480-39083073	5	ADAM9
chr4:73177379-73235080	43	ANKRD17
chr8:90017123-90036940	10	DECR1
chr3:197085579-197105005	7	DLG1
chr4:2662837-2663288	17	FAM193A

chr2:69358328-69363670	29	GFPT1
chr8:41609415-41612960	8	GPAT4
chr17:44435064-44474903	6	GPATCH8
chr19:41276158-41281275	6	HNRNPUL1
chr8:94534838-94543942	55	KIAA1429
chr1:10275427-10282533	8	KIF1B
chr15:67585889-67600749	9	MAP2K5
chr22:21788256-21799128	11	MAPK1
chr6:43617842-43618265	20	nogene
chr15:56827523-56850186	16	nogene
chr9:128469182-128473741	10	ODF2
chr1:7962762-7970963	12	PARK7
chr4:39841947-39845880	128	PDS5A
chr20:35869437-35871829	34	PHF20
chr1:119721169-119727102	9	PHGDH
chr8:140846598-140879637	12	PTK2
chr2:20278582-20283486	10	PUM2
chr1:243330539-243344331	10	SDCCAG8
chr1:100007033-100017815	9	SLC35A3
chr8:141215758-141221765	7	SLC45A4
chr3:27404829-27421779	10	SLC4A7
chr18:47851273-47896809	10	SMAD2
chr17:31940285-31966196	63	SUZ12
chr22:46891265-46894846	5	TBC1D22A
chr5:72877227-72883232	35	TNPO1
chr2:9587413-9591515	37	YWHAQ
chr1:32226170-32226530	76	EIF3I
chr16:16086823-16102717	23	ABCC1
chr5:65273339-65300131	9	ADAMTS6
chr19:32625873-32626827	11	ANKRD27
chr14:57274243-57274889	11	AP5M1
chr4:40890363-40893411	114	APBB2
chr11:108327644-108332037	57	ATM
chr16:28887188-28887722	9	ATP2A1
chr15:83041727-83050178	23	BTBD1
chr17:39490556-39494694	8	CDK12
chr2:36476888-36522313	11	CRIM1
chr20:505117-508660	11	CSNK2A1
chr18:48663751-48670744	45	CTIF
chr17:4149890-4154860	7	CYB5D2
chr15:65478879-65480399	15	DPP8
chr1:50567076-50596216	231	FAF1
chr16:50083936-50086351	11	HEATR3

chr1:113544664-113622994	6	MAGI3
chr1:93114687-93115618	19	MTF2
chr19:47500632-47503502	6	NAPA
chr2:15551492-15558634	322	NBAS
chr2:182994838-183023916	5	NCKAP1
chr2:239990073-240022340	103	NDUFA10
chr15:73116539-73136027	11	NEO1
chr17:28168447-28185265	13	NLK
chr1:246437147-246440124	52	nogene
chr17:31327648-31358632	15	nogene
chr3:195245088-195245270	18	nogene
chr1:179113081-179113668	7	nogene
chr10:119662357-119662730	4	nogene
chr10:91240483-91251440	9	PCGF5
chr3:196722414-196728136	5	PIGX
chr8:47929091-47935058	11	PRKDC
chr15:78544868-78545764	77	PSMA4
chr5:170878096-170881896	30	RANBP17
chr6:33294687-33295045	6	RGL2
chr5:95763507-95788858	10	RHOBTB3
chr3:149872028-149921227	28	RNF13
chr7:5725323-5739352	8	RNF216
chr6:106619201-106622969	15	RTN4IP1
chr5:179575240-179577136	5	RUFY1
chr10:68393137-68401737	50	RUFY2
chrX:129488936-129499841	8	SMARCA1
chr22:40840625-40850880	50	ST13
chr1:166862202-166863921	45	TADA1
chr6:121160009-121170527	14	TBC1D32
chr6:133982567-133984671	21	TBPL1
chr11:8621343-8621644	8	TRIM66
chr11:102114143-102162571	11	YAP1
chr4:182914922-182915575	182	DCTD
chr15:59668891-59680308	5	BNIP2
chr7:152783186-152825122	35	ACTR3B
chr15:58665096-58682314	7	ADAM10
chr17:19939712-19963022	7	AKAP10
chr12:99772921-99825389	34	ANKS1B
chr2:9318523-9323250	9	ASAP2
chr2:9344531-9350895	20	ASAP2
chr8:61637946-61646878	8	ASPH
chr8:61642887-61684188	7	ASPH
chr3:11358417-11362928	12	ATG7

chr22:45736967-45807022	15	ATXN10
chr2:110666262-110674365	16	BUB1
chr11:76496214-76523291	11	C11orf30
chr4:80321340-80362884	30	C4orf22
chr15:44413335-44414615	30	CASC4
chr16:2439352-2445622	11	CCNF
chr5:138319218-138331686	7	CDC25C
chr10:60780140-60785787	9	CDK1
chrX:101101057-101102411	10	CENPI
chr18:13087016-13096307	8	CEP192
chr16:53267293-53268126	23	CHD9
chr22:28694031-28699937	13	CHEK2
chr3:32483931-32491886	14	CMTM6
chr6:154422505-154442290	5	CNKSR3
chr12:51067672-51067969	14	CSRNP2
chr12:31423596-31433248	13	DENND5B
chr3:197081050-197085756	26	DLG1
chr1:65364634-65366196	324	DNAJC6
chr22:31420255-31423410	8	DRG1
chr6:56699652-56735289	80	DST
chr11:86249470-86252240	10	EED
chr8:131940498-131959663	16	EFR3A
chr15:82151464-82152423	13	EFTUD1
chr15:39972907-39976844	8	EIF2AK4
chr22:31454143-31463133	10	EIF4ENIF1
chr19:41389764-41392980	7	EXOSC5
chr9:93471140-93532329	8	FAM120A
chr2:222613810-222624775	12	FARSB
chr2:47832963-47834711	7	FBXO11
chr8:431340-435707	89	FBXO25
chrX:147940575-147945616	47	FMR1
chr9:20819795-20885230	40	FOCAD
chr9:20778680-20789608	21	FOCAD
chrX:107526581-107530511	9	FRMPD3
chr6:29621100-29624024	8	GABBR1
chr2:75665928-75672016	14	GCFC2
chr12:120177446-120178950	9	GCN1
chr17:65053501-65053728	17	GNA13
chr1:109573736-109586882	12	GNAI3
chr5:181238098-181241639	18	GNB2L1
chr6:117566853-117579064	11	GOPC
chr16:27494762-27495492	8	GTF3C1
chr10:67966682-68014186	123	HERC4

chr11:33338756-33341686	36	HIPK3
chr5:133067416-133070496	7	HSPA4
chr1:43451427-43452319	21	HYI
chr8:94824892-94829026	8	INTS8
chr11:9433569-9442197	20	IPO7
chr16:71920738-71923380	11	IST1
chr8:94491577-94491909	81	KIAA1429
chr16:48256609-48262872	12	LONP2
chr17:50736959-50741731	80	LUC7L3
chr2:8943186-8958642	13	MBOAT2
chr2:31917925-31956598	27	MEMO1
chr2:111944959-111947567	29	MERTK
chr5:80678926-80728965	13	MSH3
chr6:31741162-31747432	26	MSH5
chr6:75873206-75880120	11	MYO6
chr17:16137310-16139186	18	NCOR1
chr9:122148111-122152408	45	NDUFA8
chr16:28958716-28959100	6	NFATC2IP
chr22:37587762-37588446	7	nogene
chr2:98462383-98462581	7	nogene
chr9:2720343-2783594	9	nogene
chr5:134775752-134776142	5	nogene
chr5:138360870-138362040	5	nogene
chr12:87707646-87721292	5	nogene
chr14:34647791-34648300	6	nogene
chr17:13892724-13933185	7	nogene
chr19:58420326-58421474	7	nogene
chr9:593608-594080	11	nogene
chr22:37881672-37882024	5	nogene
chr3:136331500-136331826	9	nogene
chr4:128291799-128303035	8	nogene
chr1:27243474-27243877	15	nogene
chr4:2953510-2956811	13	NOP14
chr8:125102050-125195272	13	NSMCE2
chr7:33015669-33035988	8	NT5C3A
chr2:200925835-200933974	19	ORC2
chr3:125578959-125582978	7	OSBPL11
chr13:24477700-24484748	80	PARP4
chr1:233198938-233218184	21	PCNXL2
chr10:103405984-103406790	7	PDCD11
chr1:148977936-148981397	13	PDE4DIP
chr16:2577424-2586893	27	PDPK1
chr12:7190361-7203551	7	PEX5

chr11:581491-582081	7	PHRF1
chr11:68519500-68545024	17	PPP6R3
chr11:68519500-68558679	21	PPP6R3
chr7:105457761-105468463	6	PUS7
chr10:98186466-98209899	6	R3HCC1L
chr2:135120818-135127662	9	RAB3GAP1
chr3:158122102-158132263	15	RSRC1
chr12:123345257-123350441	37	SBNO1
chr6:154808069-154818592	15	SCAF8
chr7:101127244-101128368	16	SERPINE1
chr3:52920536-52928341	10	SFMBT1
chr3:52911002-52916214	9	SFMBT1
chr10:7248547-7285954	29	SFMBT2
chr15:75409835-75422823	5	SIN3A
chr12:40018845-40048210	9	SLC2A13
chr1:100912111-100918127	7	SLC30A7
chr9:33068823-33073806	16	SMU1
chr5:139276120-139279129	10	SNHG4
chr4:185310634-185346650	8	SNX25
chr2:45599448-45605441	8	SRBD1
chr1:47260288-47260539	7	STIL
chr4:26666332-26688407	8	TBC1D19
chr5:179891378-179894614	5	TBC1D9B
chr5:150367843-150369602	19	TCOF1
chr3:100732507-100736716	12	TFG
chr10:49952125-49958480	129	TIMM23B
chr8:70583163-70583793	26	TRAM1
chr21:44063537-44076628	7	TRAPPC10
chr3:142748257-142762313	11	TRPC1
chr9:33948373-33948587	85	UBAP2
chr9:111924776-111931357	8	UGCG
chr2:128127360-128134961	6	UGGT1
chr1:151184316-151185950	5	VPS72
chrX:118442243-118444494	23	WDR44
chr2:61520390-61533903	7	XPO1
chr5:133006758-133022906	18	ZCCHC10
chr6:157632833-157653627	15	ZDHHC14
chr10:31373017-31502509	61	ZEB1
chr20:41238687-41269083	25	ZHX3
chr1:35381258-35381758	47	ZMYM4
chr1:247156999-247159813	484	ZNF124
chr3:125313307-125331238	1027	ZNF148
chr18:76849525-76875664	76	ZNF236

chr2:71363152-71370005	87	ZNF638
chrX:47842649-47895940	5	ZNF81
chr14:102325984-102331846	11	ZNF839
chr1:28987430-28997319	14	EPB41
chr8:119788789-119797846	14	TAF2
chr2:203416907-203427269	10	ABI2
chr2:9526110-9536828	5	ADAM17
chr17:81889993-81890688	10	ALYREF
chr17:61776400-61808757	13	BRIP1
chr2:61876094-61880394	24	CCT4
chr5:138781922-138827718	39	CTNNA1
chr7:6424751-6426114	10	DAGLB
chr10:68959805-68962157	13	DDX21
chr3:108669680-108677598	9	DZIP3
chr6:158776407-158789371	19	EZR
chr2:241403827-241434321	11	FARP2
chr6:109715077-109716568	35	FIG4
chr1:240392510-240438210	11	FMN2
chr16:81351582-81365478	14	GAN
chr1:153811730-153813452	67	GATAD2B
chr3:142170324-142177576	8	GK5
chr10:130166506-130169490	14	GLRX3
chr1:155820605-155827028	51	GON4L
chr11:77978995-77981576	5	INTS4
chr12:122546175-122547969	52	KNTC1
chr16:20911182-20915714	6	LYRM1
chr7:2013083-2014642	13	MAD1L1
chr18:21791373-21819646	13	MIB1
chr6:24405167-24418578	29	MRS2
chr14:64431531-64439172	33	MTHFD1
chr4:17814852-17831116	4	NCAPG
chr17:16158759-16165161	20	NCOR1
chr6:85494748-85495026	16	nogene
chr4:23120319-23122436	14	nogene
chrX:131777622-131785258	46	nogene
chr10:11834281-11834450	12	nogene
chrX:63722761-63743446	6	nogene
chr10:87709195-87727489	8	PAPSS2
chr17:1548295-1553149	39	PITPNA
chr12:42374637-42375074	10	PPHLN1
chr8:140743229-140752316	7	PTK2
chr20:2923206-2965202	41	PTPRA
chr13:79377378-79378907	10	RBM26

chr7:105563732-105565648	20	RINT1
chr18:61816033-61816598	11	RNF152
chr15:76795279-76862533	12	SCAPER
chr16:30707182-30707712	11	SRCAP
chr13:47988540-47988981	19	SUCLA2
chr2:101027382-101033758	5	TBC1D8
chr10:95682650-95684624	14	TCTN3
chr14:89966146-89993483	8	TDP1
chr7:5356915-5359569	10	TNRC18
chr18:24019859-24083081	9	TTC39C
chr13:51703595-51739175	14	WDFY2
chr2:28906459-28929710	10	WDR43
chr6:43555835-43573601	7	XPO5
chr9:92855810-92856318	15	ZNF484
chr2:135313488-135345635	30	ZRANB3
chr11:93721311-93722050	14	CEP295
chr3:71690009-71699708	13	EIF4E3
chr2:219212386-219215082	8	ABCB6
chr7:36410513-36420314	8	ANLN
chr12:1236768-1290012	7	ERC1
chr6:617710-637861	25	EXOC2
chr16:67544298-67544830	7	FAM65A
chr16:53879843-53888951	77	FTO
chr4:173292107-173304118	9	GALNT7
chr22:38715906-38717000	14	GTPBP1
chr2:43905691-43918398	11	LRPPRC
chr6:136592172-136622981	8	MAP3K5
chr17:75611658-75611865	4	MYO15B
chr10:125074742-125122868	6	nogene
chr15:62428902-62429322	12	nogene
chr11:20127830-20130385	5	nogene
chr15:85075861-85098036	8	PDE8A
chr9:36352741-36353331	162	RNF38
chr22:24567983-24572061	7	SNRPD3
chr16:89544647-89550609	6	SPG7
chr16:84758715-84768358	7	USP10
chr8:99142973-99193057	7	VPS13B
chr4:42616032-42627109	7	ATP8A1
chr19:45421177-45421393	33	ERCC1
chr15:72345445-72356617	9	HEXA
chr2:173692683-173698482	7	nogene
chr7:17814833-17840000	11	SNX13
chr2:43960540-43963706	11	LRPPRC

chr18:36576450-36602768	17	FHOD3
chr6:13639562-13652681	4	RANBP9
chrX:75098941-75114831	9	ABCB7
chr16:3988993-3993510	42	ADCY9
chr5:77034417-77039719	33	AGGF1
chr6:131145284-131169273	145	AKAP7
chr5:140440118-140449305	239	ANKHD1-EIF4EBP3
chr7:36406180-36411166	20	ANLN
chr5:78089392-78129307	97	AP3B1
chr4:41013582-41033304	41	APBB2
chr11:129093620-129127305	21	ARHGAP32
chr8:123361191-123361646	5	ATAD2
chr3:11331339-11342279	18	ATG7
chr12:89609936-89617039	7	ATP2B1
chr10:102023476-102033339	24	C10orf76
chr11:66821642-66828364	65	C11orf80
chr4:128017362-128028853	27	C4orf29
chr20:33360350-33372697	6	CDK5RAP1
chr16:53247292-53254605	7	CHD9
chr4:55475962-55510065	31	CLOCK
chr7:135393917-135398226	11	CNOT4
chr10:35044565-35054539	8	CUL2
chrX:19950752-19955506	9	CXorf23
chrX:19929784-19936629	4	CXorf23
chr4:182877510-182894605	4	DCTD
chr4:51863436-51886638	141	DCUN1D4
chr15:65722847-65741784	9	DENND4A
chr1:26457790-26460144	18	DHDDS
chr7:754588-775162	13	DNAAF5
chr15:65487689-65500779	7	DPP8
chr15:65507242-65512564	23	DPP8
chr3:121844452-121872788	23	EAF2
chr1:21272698-21290156	107	ECE1
chr12:53016472-53020026	50	EIF4B
chr1:44284834-44339322	94	ERI3
chr4:5565257-5584850	20	EVC2
chr9:93543221-93550691	9	FAM120A
chr5:137985256-138011149	13	FAM13B
chr10:15796099-15834616	5	FAM188A
chr1:179043714-179054638	115	FAM20B
chr1:77783237-77807101	7	FAM73A
chr16:89791402-89792547	17	FANCA
chr14:45148836-45170746	7	FANCM

chr17:75931228-75938232	8	FBF1
chr12:132508152-132527988	42	FBRSL1
chr5:73074741-73078312	8	FCHO2
chrX:131755597-131785258	20	FIRRE
chr3:172251259-172298787	9	FNDC3B
chr14:65629491-65669480	11	FUT8
chr2:232700584-232761436	8	GIGYF2
chr10:94588228-94594854	25	HELLS
chr10:67939587-67941105	108	HERC4
chr1:87072933-87097935	9	HS2ST1
chr2:186638276-186641588	5	ITGAV
chr17:6628116-6628741	7	KIAA0753
chr1:10271501-10275503	30	KIF1B
chr7:98137314-98154876	29	LMTK2
chr2:242114638-242138888	9	LOC728323
chr3:197835673-197839397	12	LRCH3
chr11:68416327-68426187	8	LRP5
chr12:12158828-12165295	16	LRP6
chr5:109804153-109823837	6	MAN2A1
chr3:47870812-47877523	6	MAP4
chr3:50640365-50642332	73	MAPKAPK3
chr2:85541082-85543034	10	MAT2A
chr2:8877029-8908716	16	MBOAT2
chr17:61965005-61968258	16	MED13
chr9:36583626-36589652	8	MELK
chr18:21768622-21773728	7	MIB1
chr1:11121094-11122126	8	MTOR
chr3:172645622-172648034	8	NCEH1
chr2:182952404-182967361	11	NCKAP1
chr20:34727258-34736758	150	NCOA6
chr17:16064869-16068121	12	NCOR1
chr18:79448781-79451816	61	NFATC1
chr4:102533844-102567135	25	NFKB1
chr15:77361314-77388802	60	nogene
chr6:126111218-126174911	8	nogene
chrX:65366637-65368998	46	nogene
chr2:95148884-95159373	8	nogene
chr10:45059386-45092468	9	nogene
chr7:152652489-152652750	6	nogene
chr14:74298569-74299794	7	nogene
chr5:69504176-69514109	6	OCLN
chr10:34359146-34384254	39	PARD3
chr13:24469023-24478276	29	PARP4

chr5:58988492-58993465	47	PDE4D
chr11:34947505-34960518	10	PDHX
chr1:31630549-31635522	9	PEF1
chr7:11013746-11028818	17	PHF14
chr11:85981128-85990399	26	PICALM
chr18:62082672-62095950	9	PIGN
chr3:138698906-138714719	5	PIK3CB
chr1:151242122-151242567	23	PIP5K1A
chr1:88784638-88786213	33	PKN2
chr2:43712224-43741043	24	PLEKHH2
chr7:43965866-43973323	9	POLR2J4
chr7:124863349-124863640	11	POT1
chr2:28776850-28799433	7	PPP1CB
chr5:134199085-134202021	9	PPP2CA
chr8:26360168-26363890	5	PPP2R2A
chr4:101093775-101109078	26	PPP3CA
chr12:107739382-107746424	6	PRDM4
chr8:140686631-140706205	14	PTK2
chr11:112230230-112230595	4	PTS
chr1:30981311-30995220	9	PUM1
chr2:120278614-120289757	13	RALB
chr3:141501948-141529802	5	RASA2
chr20:37035241-37055656	13	RBL1
chr4:3316069-3343053	39	RGS12
chr2:3575888-3576460	6	RPS7
chr18:70121555-70128546	29	RTTN
chr10:70153837-70161069	5	SAR1A
chr11:10188348-10193987	11	SBF2
chr3:195668518-195673987	6	SDHAP2
chr12:48071666-48096427	12	SENP1
chr3:185614563-185617611	5	SENP2
chr3:47086194-47106120	28	SETD2
chr9:132300629-132311856	8	SETX
chr11:111616242-111620402	7	SIK2
chr5:55340009-55366875	10	SKIV2L2
chr12:40028300-40085700	25	SLC2A13
chr4:102304316-102324998	12	SLC39A8
chr15:64974601-64981025	11	SPG21
chr6:36496710-36507602	5	STK38
chr12:15889927-15895496	11	STRAP
chr14:30935162-30956242	73	STRN3
chr14:30918965-30956242	5	STRN3
chr6:149342684-149370099	17	TAB2

chr3:129880308-129880559	119	TMCC1
chr21:33455726-33468926	16	TMEM50B
chr10:133297951-133302980	7	TUBGCP2
chr15:70687539-70699660	20	UACA
chr1:162499954-162518190	7	UHMK1
chr10:11964008-12014184	9	UPF2
chr17:21006895-21012935	16	USP22
chr15:63532646-63558188	18	USP3
chr8:99467413-99481802	19	VPS13B
chr4:84702352-84717016	7	WDFY3
chr1:32631826-32634792	83	ZBTB8OS
chrX:65497131-65499989	8	ZC3H12B
chr1:247157083-247159813	13	ZNF124
chr19:53074139-53075183	27	ZNF160
chr7:30355727-30366295	70	ZNRF2
chr10:84417770-84477664	148	CCSER2
chrX:101145063-101148161	8	CENPI
chr5:149507026-149513209	11	CSNK1A1
chr3:179378340-179392104	8	MFN1
chr6:75855138-75858993	5	MYO6
chr1:151017811-151027327	5	PRUNE
chr12:50004234-50006636	12	RACGAP1
chr3:155833467-155842619	69	SLC33A1
chr1:168291159-168308877	5	TBX19
chr8:91018389-91024366	31	TMEM55A
chr16:89224718-89225631	7	ZNF778
chr16:70269783-70270195	28	AARS
chr7:16624982-16636455	54	ANKMY2
chr1:227112311-227112913	18	CDC42BPA
chr13:60008543-60042820	20	DIAPH3
chr1:50655441-50744775	40	FAF1
chr7:4740837-4747179	7	FOXK1
chr7:130116444-130127451	10	KLHDC10
chr17:64751806-64764548	6	LOC146880
chr3:47998637-47998879	18	MAP4
chr5:163456928-163457610	7	NUDCD2
chr9:2096656-2123937	5	SMARCA2
chr6:42612170-42632904	15	UBR2
chr5:83104909-83111203	24	XRCC4
chr10:31461036-31461237	82	ZEB1
chr7:105038125-105081797	9	KMT2E
chr9:36657240-36674937	6	MELK
chr18:36198689-36200324	8	MOCOS

chr14:20344931-20346913	31	PARP2
chr12:72618890-72621751	9	TRHDE
chr19:29980158-29986417	89	URI1
chr21:31700994-31706357	19	SCAF4
chr12:111729805-111734068	10	ACAD10
chr5:65260599-65273443	8	ADAMTS6
chr4:40890363-40945070	25	APBB2
chr11:120440128-120445464	36	ARHGEF12
chr11:34079627-34089456	16	CAPRIN1
chr3:138570317-138572914	10	CEP70
chr16:53235184-53254605	6	CHD9
chr3:32703867-32708820	20	CNOT10
chr6:170388286-170404601	8	FAM120B
chr5:135369405-135389126	7	H2AFY
chr14:31144110-31172122	8	HECTD1
chr17:31349119-31374129	8	NF1
chr19:3003977-3004406	9	nogene
chr10:17597445-17597992	16	nogene
chr1:32674937-32679078	9	nogene
chr9:109426949-109438234	20	PTPN3
chr7:66771882-66795592	36	RABGEF1
chrX:20186295-20204103	9	RPS6KA3
chr1:243316754-243378863	9	SDCCAG8
chr2:229807707-229811045	8	TRIP12
chr19:29985222-30012531	16	URI1
chr6:144531051-144542870	9	UTRN
chr2:202884197-202896219	9	WDR12
chr17:36988518-36990857	10	AATF
chr1:155408141-155415923	36	ASH1L
chr3:129267936-129275292	5	COPG1
chr6:149632758-149638560	121	KATNA1
chr1:153682497-153685084	10	NPR1
chr11:3775881-3782145	12	NUP98
chr8:73738283-73739868	11	STAU2
chr12:2994737-3022017	30	TEAD4
chr17:41907598-41908988	4	ACLY
chr2:157799426-157818559	39	ACVR1
chr12:1744521-1754514	13	ADIPOR2
chr16:56385918-56414344	62	AMFR
chr7:36399078-36415884	12	ANLN
chr10:121898840-121924329	28	ATE1
chr2:105342847-105343223	19	C2orf49
chr18:23234607-23237245	23	CABLES1

chr2:27237378-27239798	10	CAD
chr4:113500462-113517657	20	CAMK2D
chr5:179707131-179709059	131	CANX
chr11:119273867-119276134	11	CBL
chr21:29060540-29071988	4	CCT8
chr1:227160542-227317246	10	CDC42BPA
chrX:18575353-18588143	8	CDKL5
chr19:54142928-54153840	7	CNOT3
chr12:56282419-56286645	13	CS
chr11:107348936-107392895	7	CWF19L2
chr11:103066268-103089318	8	DCUN1D5
chr20:49233305-49236510	15	DDX27
chr11:118758773-118763306	19	DDX6
chr9:123557569-123609481	7	DENND1A
chr18:49257009-49272303	8	DYM
chr3:32544875-32545965	16	DYNC1LI1
chr12:1289851-1290012	116	ERC1
chr12:56133416-56134428	25	ESYT1
chr11:72849754-72889945	8	FCHSD2
chr16:4822874-4843602	5	GLYR1
chr1:45642426-45659142	4	GPBP1L1
chr1:41628748-41646243	10	HIVEP3
chr3:160277305-160282613	7	IFT80
chr13:30227411-30280223	11	KATNAL1
chr5:138375092-138381590	17	KDM3B
chr3:183671033-183672484	5	KLHL24
chr4:87170078-87195690	20	KLHL8
chr17:19093464-19219475	10	LOC388436
chr2:8939820-8958642	52	MBOAT2
chr9:20448122-20456786	20	MLLT3
chr21:25597301-25599866	13	MRPL39
chr2:42682400-42682589	6	MTA3
chr2:191329934-191364276	4	MYO1B
chr16:15672689-15677949	12	NDE1
chr13:32444544-32445017	4	nogene
chr17:30733942-30734223	8	nogene
chr7:106489906-106491130	15	nogene
chr12:79666512-79668408	9	nogene
chr20:3784967-3785142	32	nogene
chr1:187254375-187255868	4	nogene
chr21:34102834-34103519	8	nogene
chr8:78933742-78943071	8	nogene
chr1:1819474-1820143	7	nogene

chr5:179957412-179957909	7	nogene
chr5:6604137-6605408	140	NSUN2
chr14:31599288-31673574	5	NUBPL
chr5:37301436-37318085	4	NUP155
chr10:73014223-73047101	13	P4HA1
chr10:34399329-34450448	7	PARD3
chr4:39904039-39925933	23	PDS5A
chr18:46775489-46821072	46	PIAS2
chr3:138683677-138688974	16	PIK3CB
chr19:14450317-14452023	28	PKN1
chr5:90487999-90497706	10	POLR3G
chr7:105481051-105482440	21	PUS7
chr2:135089650-135093693	20	RAB3GAP1
chr5:168488601-168495436	45	RARS
chr15:64748418-64750381	8	RBPMS2
chr4:76996424-77020670	10	11-Sep
chr1:86715723-86722673	10	SH3GLB1
chr11:57488915-57494171	5	SLC43A1
chr7:17834051-17850964	5	SNX13
chr14:50129960-50130762	8	SOS2
chr1:23971849-23978817	4	SRSF10
chr3:136604308-136604473	33	STAG1
chr4:38089931-38118192	33	TBC1D1
chr10:94510090-94522453	21	TBC1D12
chr7:5361593-5362825	8	TNRC18
chr4:188101055-188105587	27	TRIML2
chr15:42950261-42966286	202	UBR1
chr8:102329089-102345545	24	UBR5
chr17:60265411-60345608	6	USP32
chr17:78812859-78820012	20	USP36
chr2:218495759-218498157	5	USP37
chr7:93252652-93311272	6	VPS50
chr3:184824544-184826231	8	VPS8
chrX:152963838-152970541	4	ZNF185
chr7:100075134-100079656	12	ZNF3
chr6:57182031-57184225	15	BAG2
chr3:122740257-122741113	5	HSPBAP1
chr17:49041377-49042377	75	IGF2BP1
chr11:70330172-70339306	18	PPFIA1
chr6:107929414-107931314	30	SEC63
chr4:39224883-39232272	8	WDR19
chr19:5648988-5649499	7	SAFB
chr8:125102050-125151277	8	NSMCE2

chr1:97679094-97699547	58	DPYD
chr7:30598795-30603572	17	GARS
chr8:22414779-22416280	7	SLC39A14
chr7:6697338-6705223	35	ZNF12
chr5:65224319-65226219	309	ADAMTS6
chr14:39308448-39327022	13	CTAGE5
chr1:225002782-225097239	16	DNAH14
chr1:108885274-108904254	15	GPSM2
chr12:12244261-12244655	732	LRP6
chr6:89745272-89750532	7	MDN1
chr4:169585348-169590809	15	NEK1
chr9:137449922-137452435	30	NSMF
chr1:113704080-113706722	7	PHTF1
chr9:122892908-122897576	10	RC3H2
chr12:128799258-128809473	14	SLC15A4
chr2:213310058-213350645	124	SPAG16
chr6:135300499-135358187	7	AHI1
chr19:56413958-56414440	10	ZNF583
chr8:97661071-97691188	152	MTDH
chrX:65075605-65107023	9	nogene
chr19:17277326-17277763	13	nogene
chr16:8811637-8813106	29	PMM2
chr9:127034430-127052939	18	RALGPS1
chr5:78128029-78165672	46	AP3B1
chr6:44392666-44393573	7	CDC5L
chr4:83049165-83049984	7	COPS4
chr3:197081050-197136678	7	DLG1
chr3:172251259-172307501	10	FNDC3B
chr18:35654658-35692320	7	GALNT1
chr9:77922160-77935889	19	GNAQ
chrX:57091049-57096235	4	nogene
chrX:129116609-129169451	18	nogene
chr10:73016845-73047101	7	P4HA1
chr12:3814036-3863957	4	PARP11
chr7:72802399-72815481	4	TYW1B
chr16:16071641-16079478	7	ABCC1
chr7:73465438-73470483	25	BAZ1B
chr3:56572336-56594028	7	CCDC66
chr10:124816575-124831463	8	FAM175B
chr1:240206798-240215851	6	FMN2
chr9:125307412-125307880	9	GAPVD1
chr2:69864899-69871832	8	GMCL1
chr6:30289361-30296237	10	HCG18

chr14:31116235-31121505	49	HECTD1
chr4:82978882-82984876	23	LIN54
chr19:57784189-57784640	7	nogene
chr2:26045523-26046043	7	nogene
chr17:5354358-5365238	7	RABEP1
chr3:101398860-101459052	23	SENP7
chr5:139021170-139026992	13	SIL1
chr8:73688653-73738349	5	STAU2
chr8:124515745-124522200	70	TATDN1
chr17:82889667-82911789	34	TBCD
chr1:229643532-229654388	11	URB2
chr1:168063620-168068463	50	DCAF6
chr2:174223032-174246815	12	OLA1
chr1:52509595-52526373	11	ZCCHC11
chr10:863742-890829	8	LARP4B
chr15:80970461-80982182	10	MESDC2
chr19:10913876-10916777	112	CARM1
chr12:38693656-38730358	8	CPNE8
chr17:45237280-45239065	32	FMNL1
chr10:74969659-74989112	20	KAT6B
chr21:36007318-36007834	11	LINC01436
chr1:113671733-113673465	103	MAGI3
chr10:13188880-13201534	8	MCM10
chr3:9445047-9445740	19	SETD5
chr8:17265906-17286427	19	VPS37A
chr17:60454301-60460787	12	APPBP2
chr17:60460662-60500487	18	APPBP2
chr8:22605735-22607325	15	CCAR2
chr17:59651202-59656027	10	CLTC
chr12:108652271-108657699	13	CORO1C
chr10:43154896-43176032	14	CSGALNACT2
chr5:13911385-13931244	12	DNAH5
chr1:97515725-97549744	93	DPYD
chr9:37780657-37784063	9	EXOSC3
chr1:50738862-50801677	8	FAF1
chr2:15402167-15427794	8	NBAS
chr17:1351364-1351837	5	nogene
chrX:57091049-57095538	24	nogene
chr2:151465853-151475219	9	nogene
chr3:23259618-23310727	7	nogene
chr8:51860844-51861246	98	PCMTD1
chr12:106363869-106380139	12	POLR3B
chr1:171522176-171523522	58	PRRC2C

chr5:170924016-170924550	9	RANBP17
chr1:32657426-32672901	9	RBBP4
chr3:49972058-49999513	25	RBM6
chr10:78035351-78035720	12	RPS24
chr9:2181570-2192802	13	SMARCA2
chr13:24288998-24290884	17	SPATA13
chr17:20231757-20232405	10	SPECC1
chr14:35007282-35008831	34	SRP54
chr7:47297081-47305003	5	TNS3
chr4:39737419-39774933	36	UBE2K
chr2:61241559-61246477	7	USP34
chr12:64409961-64419094	16	XPOT
chr6:36899425-36902434	6	C6orf89
chr5:148394713-148410765	8	FBXO38
chr12:109481636-109483981	28	UBE3B
chr5:157509300-157513505	11	ADAM19
chr11:129040927-129066868	24	ARHGAP32
chr8:61644599-61653660	10	ASPH
chr7:16665436-16704669	7	BZW2
chr4:15502428-15502923	7	CC2D2A
chr3:33687059-33696933	11	CLASP2
chr12:12500518-12521463	12	DUSP16
chr6:135039572-135050647	25	HBS1L
chr4:74199793-74225394	8	MTHFD2L
chr13:29439982-29492719	13	MTUS2
chr20:43681783-43687072	6	MYBL2
chr12:78116771-78118297	10	NAV3
chr10:110593072-110598290	9	SMC3
chr18:2732264-2740821	10	SMCHD1
chr4:105259618-105272918	6	TET2
chr12:49328926-49329454	8	TROAP
chr1:19186539-19187540	18	UBR4
chr1:77736055-77741748	14	USP33
chrX:131389016-131399704	136	nogene
chr3:50059648-50062108	15	RBM6
chr1:19344357-19357563	9	CAPZB
chr7:740818-741465	24	DNAAF5
chr22:40552105-40552367	9	MKL1
chr8:47918276-47927890	8	PRKDC
chr9:6465743-6477808	10	UHRF2
chr3:44629073-44631221	10	ZNF197
chr14:103491776-103500200	11	MARK3
chr5:134697125-134703932	34	SEC24A

chr18:21366026-21395561	26	GREB1L
chr3:125313307-125315053	12	ZNF148
chr22:19191303-19199841	7	CLTCL1
chr16:11904500-11905587	9	nogene
chr20:41512845-41533570	20	CHD6
chr2:36441257-36479694	21	CRIM1
chr7:6425987-6432959	67	DAGLB
chr10:68459103-68471885	5	DNA2
chr1:44319627-44352925	17	ERI3
chr5:128519272-128530693	5	FBN2
chr22:32487744-32495530	5	FBXO7
chr5:131716564-131744690	26	FNIP1
chr18:32269690-32270383	9	GAREM
chr13:30230510-30241086	21	KATNAL1
chr9:14120439-14179780	5	NFIB
chr2:130156908-130157114	43	nogene
chr6:17674904-17675770	35	NUP153
chr11:68537658-68537891	7	PPP6R3
chr5:65576878-65583219	11	PPWD1
chr1:92333390-92380873	660	RPAP2
chr12:100282942-100312653	9	SCYL2
chr3:47056820-47067118	85	SETD2
chr12:123390629-123396432	20	SETD8
chr11:107804717-107806876	11	SLC35F2
chr3:27397683-27403384	20	SLC4A7
chr2:32114637-32116212	29	SPAST
chr3:67518246-67609596	7	SUCLG2
chr22:46974289-46978934	43	TBC1D22A
chr15:75854385-75873568	35	UBE2Q2
chr7:157220688-157231327	78	UBE3C
chr6:144781921-144803147	102	UTRN
chr9:14609849-14640012	5	ZDHHC21
chr10:31387123-31461237	19	ZEB1
chr13:20002849-20006586	25	ZMYM2
chr21:29066715-29070337	11	CCT8
chr22:24570590-24570913	10	nogene
chr1:5952699-5986327	18	NPHP4
chr9:114466266-114478771	23	DFNB31
chr10:35137781-35152380	8	CREM
chr17:35605704-35608387	24	AP2B1
chr5:177531281-177539147	6	FAM193B
chr11:61313498-61316567	13	DDB1
chr1:117460488-117466427	70	MAN1A2

chr6:56526360-56536940	9	DST
chr8:47836337-47837419	5	PRKDC
chr14:54774933-54776540	7	SAMD4A
chr16:53844154-53879987	12	FTO
chr22:25027944-25029540	8	KIAA1671
chr15:67600684-67664645	7	MAP2K5
chr5:43612907-43628387	6	NNT
chr8:128009589-128080430	8	nogene
chr9:131455075-131465078	6	PRRC2B
chr8:140830471-140921132	7	PTK2
chr1:41113282-41186250	6	SCMH1
chr1:246327200-246355094	28	SMYD3
chr20:61997549-61999108	8	TAF4
chr21:43903956-43904452	9	AGPAT3
chr8:61651049-61684188	57	ASPH
chr5:133064979-133076898	80	HSPA4
chr19:10489191-10489853	7	KEAP1
chr4:74174505-74175403	19	MTHFD2L
chr4:1900625-1939778	245	WHSC1
chr12:100097364-100098541	11	UHRF1BP1L
chr3:132525609-132531097	8	DNAJC13
chr17:1875660-1880691	9	RPA1
chr12:56581183-56582152	12	RBMS2
chr8:42459895-42465917	38	SLC20A2
chr22:17688985-17702386	10	BCL2L13
chr7:102097363-102115273	23	CUX1
chr14:22906194-22913845	13	RBM23
chr2:222916318-222924595	17	ACSL3
chr15:43335695-43349045	26	ADAL
chr10:408925-423033	16	DIP2C
chr7:158793649-158798076	5	ESYT2
chr12:120142506-120142941	19	GCN1
chr14:73077368-73088161	23	RBM25
chr7:854919-857957	19	SUN1
chr12:49661965-49668554	24	FMNL3
chr16:66727687-66732505	6	DYNC1LI2
chr14:60796216-60818847	24	MNAT1
chr11:1286001-1295794	69	TOLLIP
chr2:9376907-9388546	14	ASAP2
chr3:3167633-3175269	7	CRBN
chr18:79096475-79176907	42	ATP9B
chr22:49787389-49799119	24	BRD1
chr5:173080584-173091401	15	CREBRF

chr2:65344435-65344896	7	SPRED2
chr8:102323310-102347092	7	UBR5
chr19:32622421-32625966	38	ANKRD27
chr17:60494465-60500487	15	APPBP2
chr16:53254437-53255779	7	CHD9
chr3:33644756-33696933	6	CLASP2
chr5:112259228-112280323	13	EPB41L4A
chr9:523892-540667	12	KANK1
chr12:122584277-122586757	6	KNTC1
chr3:14944650-14944903	7	nogene
chr2:208304170-208305013	5	PIKFYVE
chr16:53442657-53447106	14	RBL2
chr8:100268784-100275161	15	RNF19A
chr1:204112914-204123804	13	SOX13
chr17:54985613-54990957	27	STXBP4
chr15:65497863-65500779	27	DPP8
chr17:28682726-28683816	16	SUPT6H
chr3:183840569-183868060	45	PARL
chr15:43756648-43763206	67	PDIA3
chrX:63665885-63724711	26	ARHGEF9
chr16:23642954-23661281	28	DCTN5
chr5:31421271-31424471	517	DROSHA
chr3:37281957-37296219	4	GOLGA4
chr20:4795814-4801062	13	RASSF2
chr15:44648865-44652266	15	SPG11
chr1:83911164-83947282	19	TTLL7
chr10:12028744-12029524	5	UPF2
chr4:84860411-84932363	14	WDFY3
chr14:74289482-74290499	10	ABCD4
chr9:32429418-32431843	10	ACO1
chr4:61934839-62044549	8	ADGRL3
chr6:135358131-135411544	8	AHI1
chr19:40241937-40242687	36	AKT2
chr12:110029477-110036328	7	ANKRD13A
chr12:45891779-45893721	30	ARID2
chr3:48927461-48927813	29	ARIH2
chr8:61567167-61619019	8	ASPH
chr2:175093060-175118369	7	ATF2
chr11:108227594-108267342	28	ATM
chr1:111456085-111459636	37	ATP5F1
chr13:31215050-31261082	26	B3GLCT
chr16:88004294-88038011	33	BANP
chr5:34023908-34035758	8	C1QTNF3

chr5:37238856-37243119	7	C5orf42
chr2:151841902-151860820	11	CACNB4
chr5:179708238-179709059	178	CANX
chr19:13920556-13923811	29	CC2D1A
chr6:47576523-47609304	11	CD2AP
chr4:184710071-184725062	8	CENPU
chr7:102097363-102111774	10	CUX1
chr10:100243697-100250332	14	CWF19L1
chr9:123557569-123557964	7	DENND1A
chr5:60638749-60647533	75	DEPDC1B
chr2:229447315-229477253	5	DNER
chr10:126970701-127127764	15	DOCK1
chrX:24064200-24068108	39	EIF2S3
chr18:45880074-45912456	9	EPG5
chr7:158759485-158767774	28	ESYT2
chr1:92605306-92636336	48	EVI5
chr10:15821655-15847943	227	FAM188A
chr4:2596083-2639859	36	FAM193A
chr5:171899003-171900100	10	FBXW11
chr4:122876320-122892296	16	FGF2
chr9:113198854-113203035	20	FKBP15
chr3:197755669-197756557	7	FYTTD1
chr7:50669721-50674658	14	GRB10
chr8:30607056-30614715	27	GTF2E2
chr15:83157414-83164075	8	HDGFRP3
chr14:31380466-31387375	20	HEATR5A
chr8:28980070-29018913	10	HMBOX1
chr20:43613849-43624045	42	IFT52
chr7:111487237-111561923	117	IMMP2L
chr1:61990167-62038049	12	INADL
chr13:51421111-51430383	12	INTS6
chr8:94828974-94841591	11	INTS8
chr16:47311239-47313823	59	ITFG1
chr2:24293687-24295804	26	ITSN2
chr19:10491576-10492262	67	KEAP1
chr1:32030297-32033334	310	KHDRBS1
chr15:64365973-64376905	8	KIAA0101
chr18:37066889-37067545	33	KIAA1328
chr6:57052685-57059377	8	KIAA1586
chr12:122587710-122588816	6	KNTC1
chr14:50283870-50303017	14	L2HGDH
chr13:27581296-27581803	36	LNX2
chr16:74360632-74360891	21	LOC283922

chr1:53255116-53258471	5	LRP8
chr11:65605760-65608448	14	MAP3K11
chr6:167870385-167889326	9	MLLT4
chr14:64412471-64418024	7	MTHFD1
chr3:9669431-9671170	15	MTMR14
chr22:36341369-36349255	125	MYH9
chr11:34108210-34127599	9	NAT10
chr17:16034764-16048988	12	NCOR1
chr19:52588531-52599170	10	nogene
chr18:57731413-57735592	29	nogene
chr8:123452562-123453459	14	nogene
chr3:48749712-48750170	9	nogene
chr17:45054541-45055894	10	nogene
chr12:5160306-5198646	18	nogene
chr14:26729558-26731156	6	nogene
chr7:66882892-66890411	6	nogene
chr9:104750954-104759440	12	nogene
chr9:99844648-99847615	9	NR4A3
chr1:51800555-51814089	6	NRD1
chr10:18596207-18616293	12	NSUN6
chr3:31733256-31748120	7	OSBPL10
chr17:78414878-78424192	7	PGS1
chr3:170122590-170126007	25	PHC3
chr6:43587271-43601091	39	POLH
chr1:212342187-212349242	18	PPP2R5A
chr11:68515027-68537891	10	PPP6R3
chr1:156791696-156797341	9	PRCC
chr8:127890588-127939676	16	PVT1
chr7:39686630-39696859	42	RALA
chr3:50099981-50105142	14	RBM5
chr21:34521498-34523710	7	RCAN1
chr18:13731475-13731934	14	RNMT
chr1:92834778-92837633	33	RPL5
chr1:92833388-92837633	9	RPL5
chr3:158122102-158203245	8	RSRC1
chr1:109230877-109231786	75	SARS
chr7:72830750-72831798	14	SBDSP1
chr7:4049347-4051830	11	SDK1
chr14:99458278-99463578	9	SETD3
chrX:119406459-119426354	46	SLC25A43
chr11:93148652-93166016	5	SLC36A4
chr12:46197719-46198743	25	SLC38A1
chr3:27379248-27424152	6	SLC4A7

chr10:100915998-100926019	9	SLF2
chrX:53394777-53396617	8	SMC1A
chr16:29553085-29554992	15	SMG1P2
chr6:85557975-85567577	33	SNX14
chr18:26032398-26078160	12	SS18
chr6:36515337-36540207	8	STK38
chr11:59790518-59795482	6	STX3
chr13:47988540-47997023	7	SUCLA2
chr18:26265169-26282060	7	TAF4B
chr3:100295086-100298045	13	TBC1D23
chr14:39151745-39159550	16	TRAPPC6B
chr14:90730074-90744922	5	TTC7B
chr2:178535731-178571449	7	TTN-AS1
chr6:3155624-3177642	119	TUBB2A
chr15:43400043-43401850	31	TUBGCP4
chr6:158448995-158461729	114	TULP4
chr1:162566011-162600903	15	UAP1
chr1:154227281-154237136	8	UBAP2L
chr8:17280374-17286427	18	VPS37A
chr5:37697654-37703087	19	WDR70
chr4:1892165-1901251	8	WHSC1
chr4:1900625-1930770	149	WHSC1
chr21:39390697-39393280	8	WRB
chr1:180787752-180811488	19	XPR1
chr2:216113015-216162048	8	XRCC5
chr3:142425221-142426841	56	XRN1
chr7:130026157-130039547	13	ZC3HC1
chr14:73931502-73932112	46	ZNF410
chr19:32382466-32386721	6	ZNF507
chr17:15978043-15987368	13	ZSWIM7
chr20:33366858-33372697	12	CDK5RAP1
chr14:21412912-21415907	6	CHD8
chr12:38829378-38874511	34	CPNE8
chr2:232130618-232163632	12	DIS3L2
chr15:89299799-89303915	16	FANCI
chr16:53844154-53845086	8	FTO
chr16:48296014-48303305	19	LONP2
chr9:110972072-111005539	12	LPAR1
chr12:68820324-68824651	121	MDM2
chr12:65306862-65308655	19	MSRB3
chr7:10990702-11028818	13	PHF14
chr6:35410213-35411217	7	PPARD
chr12:27633361-27635115	46	PPFIBP1

chr20:2964271-2975241	13	PTPRA
chr6:130385172-130387592	25	TMEM200A
chr9:109057190-109108570	6	TMEM245
chr15:50637421-50648885	8	TRPM7
chr19:37165594-37170054	11	ZNF585A
chr7:64535818-64544432	19	ZNF680
chr18:21656860-21659343	5	ABHD3
chr19:48230437-48241063	24	CARD8
chr11:9152357-9170777	8	DENND5A
chr1:1804418-1839238	158	GNB1
chr7:1898199-2069338	25	MAD1L1
chr17:47603914-47613725	10	NPEPPS
chr7:6002452-6006031	18	PMS2
chr2:28776850-28783978	8	PPP1CB
chr3:19970547-19978403	19	RAB5A
chr19:5645336-5649975	7	SAFB
chr15:76841733-76862533	47	SCAPER
chr10:86467278-86473973	63	WAPL
chr22:40914238-40924482	5	XPNPEP3
chr22:21769194-21807846	7	MAPK1
chr9:94789894-94801002	25	C9orf3
chr13:60093627-60112186	26	DIAPH3
chrX:64328771-64337393	56	MTMR8
chr11:29037741-29044660	15	nogene
chr15:67830190-67833257	13	SKOR1
chr4:148063147-148072179	7	ARHGAP10
chr2:25799384-25845563	37	ASXL2
chr22:25457258-25459515	38	CRYBB2P1
chr4:82358658-82359639	5	HNRNPD
chr1:150440922-150446401	30	RPRD2
chr1:186398312-186406268	12	C1orf27
chr7:66553988-66554837	8	GS1-124K5.11
chr9:35707920-35708340	16	TLN1
chr12:104288930-104315896	17	TXNRD1
chr19:12110304-12111372	13	ZNF788
chr2:235968461-235968957	16	AGAP1
chr7:65726770-65727108	18	LOC441242
chr1:28702437-28704329	18	GMEB1
chr1:155370503-155370983	84	ASH1L
chr9:96719508-96721517	10	nogene
chr10:263203-263658	35	nogene
chr3:104817472-104842744	8	nogene
chr3:16271658-16272321	8	OXNAD1

chr2:65344718-65432084	14	SPRED2
chr8:98746709-98749390	10	STK3
chr10:327005-349454	28	DIP2C
chr11:66748378-66759096	24	C11orf80
chr19:1270927-1274440	13	CIRBP
chr3:50106766-50108304	21	RBM5
chr12:96313320-96334865	8	CDK17
chr1:22863036-22865212	12	EPHB2
chr5:142873399-142879445	7	ARHGAP26
chr11:33105566-33106064	71	CSTF3
chr17:61919399-61926662	7	INTS2
chr10:93366391-93373085	7	MYOF
chr2:40428474-40430301	7	nogene
chr13:24455016-24493733	12	PARP4
chrX:15425905-15459740	28	PIR
chr10:7367648-7370375	27	SFMBT2
chr5:132927120-132934941	45	AFF4
chr15:85662387-85682212	8	AKAP13
chr17:1072613-1100735	10	ABR
chr11:108133819-108135242	17	ACAT1
chr20:25021388-25023641	9	ACSS1
chr2:113939958-113942359	87	ACTR3
chr12:19500096-19514784	7	AEBP2
chr2:235744697-235807331	9	AGAP1
chr2:177440964-177462018	130	AGPS
chr9:85575314-85579096	32	AGTPBP1
chr17:19939712-19958571	12	AKAP10
chr4:145081659-145096111	6	ANAPC10
chr9:137175501-137175707	17	ANAPC2
chr9:97994630-98012472	9	ANP32B
chr15:50948019-50958794	42	AP4E1
chr21:25975069-26000182	71	APP
chr17:60464020-60494617	7	APPBP2
chr22:42810812-42817857	5	ARFGAP3
chr11:46688172-46696156	8	ARHGAP1
chr14:32146262-32152528	9	ARHGAP5
chr1:155352705-155354630	12	ASH1L
chrX:139814877-139826823	150	ATP11C
chr22:17612811-17619526	8	ATP6V1E1
chr20:56381432-56384324	359	AURKA
chr13:102807145-102834552	25	BIVM
chr15:59671182-59672739	10	BNIP2
chr21:39274373-39277351	11	BRWD1

chr11:76451857-76472840	41	C11orf30
chr19:16500791-16501551	8	C19orf44
chr1:154212319-154214574	14	C1orf43
chr1:64676894-64679756	11	CACHD1
chr12:28198942-28307749	11	CCDC91
chr10:12230947-12238485	105	CDC123
chr1:227119803-227129231	8	CDC42BPA
chr7:39987598-40047877	14	CDK13
chr3:113427186-113433669	5	CFAP44
chr12:70278131-70294294	8	CNOT2
chr7:135410523-135422353	11	CNOT4
chr12:108657303-108662158	24	CORO1C
chr17:30384988-30390159	6	CPD
chr16:3791980-3810779	12	CREBBP
chr2:36358903-36464655	5	CRIM1
chr8:67115913-67118821	7	CSPP1
chr16:67610823-67616878	11	CTCF
chr15:44458990-44459200	20	CTDSPL2
chr10:100245798-100246935	7	CWF19L1
chrX:19965043-19970298	11	CXorf23
chr15:22925981-22932340	10	CYFIP1
chr7:6421726-6424835	10	DAGLB
chr6:117540667-117543211	9	DCBLD1
chr11:679687-681089	72	DEAF1
chr12:31426292-31447769	18	DENND5B
chr5:60686961-60687838	29	DEPDC1B
chrX:2266739-2425304	12	DHRSX
chr12:124966792-124967218	6	DHX37
chr13:59774188-59810923	27	DIAPH3
chr1:231795241-231818517	11	DISC1
chr11:112033403-112051349	5	DLAT
chr9:33036573-33037115	30	DNAJA1
chr3:51146548-51160702	14	DOCK3
chr7:35117747-35132369	13	DPY19L2P1
chr2:26924886-26928323	26	DPYSL5
chr3:32541036-32545965	63	DYNC1LI1
chr20:35123889-35126375	19	EDEM2
chr14:89931540-89954001	8	EFCAB11
chr14:33925617-33931215	39	EGLN3
chr19:47718508-47726224	45	EHD2
chr20:34093674-34103565	5	EIF2S2
chr1:20980333-21089213	11	EIF4G3
chr14:88712270-88715195	10	EML5

chr22:41135812-41137790	11	EP300
chr4:65601640-65602304	7	EPHA5
chr1:51363865-51366029	15	EPS15
chr12:1189858-1290012	30	ERC1
chr9:95897848-95956013	30	ERCC6L2
chr10:49524032-49524777	12	ERCC6-PGBD3
chr10:100164003-100179247	6	ERLIN1
chr1:50705785-50744775	12	FAF1
chr4:38931450-38932374	6	FAM114A1
chr9:93471140-93501120	6	FAM120A
chr9:93471262-93498886	5	FAM120A
chr9:93561108-93562304	8	FAM120A
chrX:134781820-134789266	6	FAM122B
chr5:137941943-137943216	19	FAM13B
chr5:138018301-138021197	27	FAM13B
chr18:13671861-13682105	15	FAM210A
chr15:52584744-52593736	36	FAM214A
chr15:30908117-30914091	24	FAN1
chr5:108768092-108798389	102	FER
chr6:167022408-167026615	13	FGFR1OP
chr14:75633628-75641898	8	FLVCR2
chr13:40559508-40560860	9	FOXO1
chr16:53873785-53879987	29	FTO
chr2:75694240-75696315	4	GCFC2
chr2:190895151-190910321	23	GLS
chr3:168036835-168041474	26	GOLIM4
chr2:156476097-156513496	10	GPD2
chr2:9961045-9965381	7	GRHL1
chr13:45151686-45207505	63	GTF2F2
chr7:74589850-74591017	11	GTF2IRD1
chr2:37000585-37002572	9	HEATR5B
chr10:67992198-68025676	7	HERC4
chr2:190287585-190310796	14	HIBCH
chr14:61720381-61727655	25	HIF1A
chr15:77477554-77479321	9	HMG20A
chr10:68341173-68341851	13	HNRNPH3
chr8:42973288-42986795	65	HOOK3
chr2:238182064-238185287	408	ILKAP
chr11:77955941-77991299	10	INTS4
chr8:94827262-94827793	17	INTS8
chr8:28835291-28837776	8	INTS9
chr10:63380317-63455089	103	JMJD1C
chr1:35478965-35479212	52	KIAA0319L

chr5:62363177-62366481	6	KIF2A
chr16:87755193-87762040	8	KLHDC4
chr4:165219933-165228913	40	KLHL2
chr3:122442037-122452064	11	KPNA1
chr3:122457725-122467429	15	KPNA1
chr8:70637814-70644243	38	LACTB2
chr8:97805352-97825153	331	LAPTM4B
chr2:30562145-30568176	29	LCLAT1
chr5:126804775-126826107	52	LMNB1
chr17:5209749-5223484	5	LOC100130950
chr7:102397619-102404683	11	LOC100630923
chr3:197866111-197871462	57	LRCH3
chr11:68347846-68390052	20	LRP5
chr1:3780637-3785203	6	LRRC47
chr1:219193129-219241308	14	LYPLAL1
chr12:8943404-8943910	11	M6PR
chr3:65429519-65442849	9	MAGI1
chr1:113580541-113594560	12	MAGI3
chr11:95985530-95991723	32	MAML2
chr4:102714437-102726683	167	MANBA
chr15:67550033-67600749	25	MAP2K5
chr15:67664596-67703408	77	MAP2K5
chr2:159714556-159729175	7	7-Mar
chr2:85541632-85543034	20	MAT2A
chr5:113082859-113085310	15	MCC
chr1:84947032-84965708	32	MCOLN2
chr6:89722954-89725396	54	MDN1
chr18:50918109-50924212	10	ME2
chr12:82386802-82403130	15	METTL25
chr4:127930682-127938838	13	MFSD8
chr9:21476898-21481152	23	MIR31HG
chr18:36266748-36269675	5	MOCOS
chr18:36195256-36260175	10	MOCOS
chr12:62580296-62588156	7	MON2
chr21:25597301-25603935	19	MRPL39
chr1:37835078-37838756	14	MTF1
chr12:109590770-109595181	8	MVK
chr18:3253232-3254050	12	MYL12A
chr15:52416144-52433285	8	MYO5A
chr19:17156908-17159484	8	MYO9B
chr9:85959792-85996577	51	NAA35
chr11:71490844-71491903	10	NADSYN1
chr20:36665045-36671397	35	NDRG3

chr16:47122656-47134410	11	NETO2
chr20:25517752-25526598	13	NINL
chr17:28163542-28172618	110	NLK
chr10:15128194-15130312	19	NMT2
chr1:120419849-120427847	104	nogene
chr1:44834464-44836058	5	nogene
chr17:38505285-38505652	15	nogene
chr6:117662905-117664862	8	nogene
chr19:16078608-16101409	12	nogene
chr17:78004169-78009697	23	nogene
chr11:47800144-47800523	8	nogene
chr3:67237400-67241819	15	nogene
chr3:153164765-153178789	16	nogene
chr3:67218045-67218290	13	nogene
chr12:21233699-21234299	12	nogene
chr5:179538993-179540832	19	nogene
chr16:3640714-3643036	15	nogene
chr9:97188464-97200565	7	nogene
chr2:177214730-177215110	10	nogene
chr16:70226769-70246610	32	nogene
chr1:5961793-5969259	28	NPHP4
chr5:143399655-143400852	544	NR3C1
chr5:177135086-177192019	31	NSD1
chr6:17661652-17665385	15	NUP153
chr17:5388801-5404246	9	NUP88
chr11:3768580-3773739	11	NUP98
chrX:68096869-68206673	9	OPHN1
chr7:104161071-104188381	15	ORC5
chr7:24815058-24834736	18	OSBPL3
chr8:100709132-100717888	7	PABPC1
chr8:100704296-100705673	128	PABPC1
chr16:23621361-23623130	105	PALB2
chr13:28256291-28266876	6	PAN3
chr10:49841949-49869555	69	PARG
chr14:71073544-71089342	6	PCNX
chr14:71073544-71076419	7	PCNX
chr1:233208517-233252788	13	PCNXL2
chr11:34931403-34984728	28	PDHX
chr11:34957383-34978182	134	PDHX
chr2:216065311-216066518	10	PECR
chr1:10411417-10413251	36	PGD
chr6:143765260-143807133	6	PHACTR2
chr3:170122590-170129552	17	PHC3

chr7:11028680-11062085	16	PHF14
chr20:35858301-35871829	150	PHF20
chr16:71690518-71702731	7	PHLPP2
chr1:151224247-151242567	10	PIP5K1A
chr22:27853524-27860241	5	PITPNB
chr8:42355515-42362698	13	POLB
chr6:43587271-43610723	14	POLH
chr1:145974824-145975436	9	POLR3GL
chr11:68505144-68548204	6	PPP6R3
chr8:47794289-47807326	9	PRKDC
chr1:171568246-171575128	121	PRRC2C
chr2:86121478-86137140	6	PTCD3
chr8:96306443-96331095	318	PTDSS1
chr8:140864311-140921132	9	PTK2
chr3:19975600-19976169	21	RAB5A
chr7:66775226-66783841	55	RABGEF1
chr9:107300140-107306647	144	RAD23B
chr5:131521389-131562047	10	RAPGEF6
chr8:52683548-52686967	39	RB1CC1
chr5:150696790-150698631	7	RBM22
chr1:173946475-173947582	14	RC3H1
chr1:176144004-176163891	9	RFWD2
chr1:176081151-176116681	14	RFWD2
chr19:11397243-11397597	17	RGL3
chr2:3545700-3550472	5	RNASEH1
chr15:59052304-59058550	15	RNF111
chr3:196486532-196487578	8	RNF168
chr8:100259106-100264785	26	RNF19A
chr12:121402741-121417911	26	RNF34
chr17:1842802-1883944	12	RPA1
chr2:10126874-10127220	6	RRM2
chr16:11846500-11850418	23	RSL1D1
chr5:179591624-179593645	45	RUFY1
chr11:61437624-61446231	16	SDHAF2
chr12:48096342-48101516	31	SENP1
chr1:67420008-67425223	48	SERBP1
chr14:99458278-99466055	7	SETD3
chr19:4177078-4179286	9	SIRT6
chr7:100856867-100857176	70	SLC12A9
chr8:42439449-42444762	7	SLC20A2
chr19:10631056-10632156	11	SLC44A2
chr18:47841850-47896809	7	SMAD2
chrX:53403565-53403893	92	SMC1A

chr17:1786809-1787604	16	SMYD4
chr11:62853551-62855466	7	SNHG1
chr1:31267870-31292006	38	SNRNP40
chr15:64118116-64118854	74	SNX1
chr14:34586229-34609742	106	SNX6
chr17:51020158-51041651	9	SPAG9
chr22:24365475-24369320	20	SPECC1L-ADORA2A
chr1:112600793-112616895	6	ST7L
chr3:136472412-136477412	11	STAG1
chr12:56348528-56349636	36	STAT2
chr17:42322281-42323625	12	STAT3
chr20:49135832-49174269	23	STAU1
chr20:49165996-49174269	7	STAU1
chr1:172555868-172570730	23	SUCO
chr3:11809516-11829864	24	TAMM41
chr2:159169249-159172272	8	TANC1
chr17:29475669-29482288	20	TAOK1
chr1:246544520-246557534	11	TFB2M
chr3:129860939-129880559	12	TMCC1
chr9:109050282-109108570	14	TMEM245
chr9:109050282-109073438	33	TMEM245
chr14:56585000-56636598	19	TMEM260
chr11:1288623-1295794	9	TOLLIP
chr1:223810430-223818337	15	TP53BP2
chr21:44052279-44055893	8	TRAPPC10
chr5:115130591-115134147	13	TRIM36
chr18:24064139-24130417	8	TTC39C
chr15:23030884-23037152	8	TUBGCP5
chr17:18789352-18804266	6	TVP23B
chr1:154251040-154257434	11	UBAP2L
chr15:25354352-25360527	25	UBE3A
chr6:42603587-42615178	6	UBR2
chr9:111929499-111931357	16	UGCG
chr9:111914604-111929678	9	UGCG
chr2:61325374-61350693	9	USP34
chr10:75219062-75222402	12	VDAC2
chr9:77337254-77371149	7	VPS13A
chr15:78289618-78295725	12	WDR61
chr18:56717963-56731597	15	WDR7
chr15:43836160-43839728	6	WDR76
chr2:159271701-159283093	7	WDSUB1
chr3:183747671-183758965	4	YEATS2
chr10:27119293-27121448	10	YME1L1

chr1:32592930-32593754	47	ZBTB8A
chr18:62539680-62550460	465	ZCCHC2
chr11:57692610-57696873	18	ZDHHC5
chr16:89733301-89734038	5	ZNF276
chr12:132933034-132948270	18	ZNF605
chr9:113969116-114016784	25	ZNF618
chr1:52885832-52892975	16	ZYG11A
chr2:177513818-177523805	8	AGPS
chr1:246618257-246634587	18	CNST
chr15:90638445-90641199	19	CRTC3
chr13:49997073-50044768	10	DLEU2
chr1:1839189-1879902	10	GNB1
chr14:66709356-66776521	10	GPHN
chr6:110960413-110962505	10	GTF3C6
chr9:111425417-111433372	8	KIAA0368
chr15:59256283-59272449	7	MYO1E
chr6:75835900-75841378	57	MYO6
chr5:150532944-150535885	30	NDST1
chr9:96913237-96913633	14	nogene
chr16:89393613-89394390	19	nogene
chr5:10213490-10227663	8	nogene
chr20:3907925-3917050	6	PANK2
chr8:103961064-104014615	6	RIMS2
chr8:47279861-47440542	15	SPIDR
chr5:14270824-14330900	9	TRIO
chr10:112527086-112538330	54	VTI1A
chr2:216113015-216117794	27	XRCC5
chr7:66127703-66145500	74	CRCP
chr15:65551675-65558731	38	HACD3
chr12:124419956-124430787	141	NCOR2
chr5:143295459-143314168	54	NR3C1
chr3:160420739-160428942	10	SMC4
chr14:102193462-102197847	20	WDR20
chr2:65246523-65255694	17	ACTR2
chrX:147928321-147945616	8	FMR1
chr8:39021642-39023325	31	ADAM9
chr8:61661805-61684188	28	ASPH
chr12:1497699-1502581	10	nogene
chr12:121894738-121903107	31	PSMD9
chr2:127993186-127996491	74	SAP130
chr3:100279648-100281847	10	TBC1D23
chr20:63789820-63790790	147	ZBTB46
chr19:47264602-47264946	137	CCDC9

chr18:49118743-49163787	6	DYM
chr14:104157754-104167048	7	KIF26A
chr3:156852767-156888397	9	LEKR1
chr19:5711420-5712002	7	LONP1
chr1:243293090-243378863	9	SDCCAG8
chrX:118441367-118443687	17	WDR44
chr16:30362278-30362777	6	nogene
chr4:128888678-128946152	9	SCLT1
chr2:215336034-215349249	6	ATIC
chr7:39987598-40063100	12	CDK13
chr16:58585337-58587413	7	CNOT1
chr16:23416966-23418827	8	COG7
chr2:62943801-62949162	12	EHBP1
chr17:44084554-44087696	6	HDAC5
chr12:50435487-50437838	6	LARP4
chr2:15178987-15218968	15	NBAS
chr4:169477123-169479534	11	NEK1
chr6:41933549-41933881	18	nogene
chr10:68296265-68306906	8	PBLD
chr6:42966043-42966447	9	PEX6
chr13:29530537-29532982	25	SLC7A1
chr15:44592330-44596943	61	SPG11
chr2:202892616-202897415	6	WDR12
chr2:233434379-233436441	30	DGKD
chr1:184734530-184749592	12	EDEM3
chr18:688573-691276	8	ENOSF1
chr22:41158411-41162779	14	EP300
chr2:69354488-69363670	13	GFPT1
chr1:39439273-39439500	12	MACF1
chr2:39331916-39378123	10	MAP4K3
chr7:66771882-66799414	51	RABGEF1
chr6:88769773-88844521	36	RNGTT
chr2:210015972-210017559	7	RPE
chr1:243316754-243344331	10	SDCCAG8
chr1:168231513-168231919	8	SFT2D2
chr16:66513730-66549009	10	TK2
chr5:72888077-72889957	8	TNPO1
chr10:68142939-68175461	48	MYPN
chr6:141772872-141798155	10	nogene
chr11:238997-244761	21	PSMD13
chr6:3737078-3739161	153	PXDC1
chr7:72831601-72831798	14	SBDSP1
chr10:110589604-110593223	16	SMC3

chr14:30947089-30956242	48	STRN3
chr17:78004169-78031842	62	TNRC6C
chr18:47889020-47896809	9	SMAD2
chr19:32408216-32411372	36	DPY19L3
chr15:57091788-57095163	121	TCF12
chr4:67745826-67790511	7	nogene
chr5:140189063-140194652	62	CYSTM1
chr7:44674410-44681848	24	OGDH
chr11:62605112-62607099	5	EML3
chr1:98393054-98395390	10	nogene
chr3:183862696-183868060	10	PARL
chr19:40237968-40240110	38	AKT2
chr3:195708133-195708841	7	LINC00969
chr3:29696097-29762989	7	RBMS3
chr19:11397243-11400297	7	RGL3
chr4:84713158-84721572	8	WDFY3
chr7:99338180-99348959	8	ARPC1A
chr2:214780559-214781509	9	BARD1
chr1:168043024-168045227	16	DCAF6
chr15:74655732-74656068	71	EDC3
chr8:17950614-17953186	9	PCM1
chr12:64080285-64097375	6	SRGAP1
chrX:96937232-96945591	10	DIAPH2
chr16:879569-954666	7	LMF1
chr13:49451552-49461162	13	SETDB2
chr2:229836847-229860531	8	TRIP12
chr15:50471632-50477499	28	USP8
chr5:126554286-126556015	7	ALDH7A1
chr1:32224033-32226530	26	EIF3I
chr6:45422592-45492114	7	RUNX2
chr3:52912537-52916214	100	SFMBT1
chr15:42966152-42970607	7	UBR1
chr8:130124012-130128090	5	ASAP1
chr11:35204511-35208206	7	CD44
chr4:55475962-55489465	20	CLOCK
chr5:10381799-10391731	10	6-Mar
chr12:78127166-78159286	12	NAV3
chr8:140710652-140711019	10	nogene
chr1:109281288-109281618	8	PSRC1
chr17:80922723-80923673	57	RPTOR
chr4:42018110-42049479	34	SLC30A9
chr2:219473503-219473903	7	SPEG
chr15:43135511-43160794	59	TMEM62

chr16:3675323-3689137	16	TRAP1
chr14:51022834-51025360	8	TRIM9
chr6:34821652-34836348	5	UHRF1BP1
chr17:51041500-51056482	241	SPAG9
chr7:105160506-105203785	21	SRPK2
chr22:23683724-23686995	16	GUSBP11
chr1:168183901-168198981	9	TIPRL
chr9:13205035-13250372	8	MPDZ
chr3:161248034-161248237	8	nogene
chr21:39206107-39212707	96	BRWD1
chr15:51916014-51934354	6	nogene
chr1:46587108-46587570	9	nogene
chr18:47896520-47930446	9	SMAD2
chr20:35381917-35394196	5	UQCC1
chr1:28430288-28430896	15	nogene
chr15:84680712-84691644	409	SEC11A
chr20:47227202-47236516	13	ZMYND8
chr3:170291853-170297393	87	PRKCI
chr1:179110281-179112398	30	ABL2
chr16:68121986-68183366	7	NFATC3
chr2:112485916-112501341	11	TTL
chr15:28220451-28222215	19	HERC2
chr16:58558472-58560362	10	CNOT1
chr4:109527321-109538596	10	SEC24B
chr19:7444203-7444454	9	ARHGEF18
chr3:170136418-170149244	16	PHC3
chr9:112098469-112102285	5	SUSD1
chr1:240123178-240137802	11	FMN2
chr20:28583018-28591519	16	FRG1CP
chr1:44410583-44412722	6	RNF220
chrX:49038009-49040568	13	TFE3
chr12:32599401-32599642	6	nogene
chr10:18585948-18616293	20	NSUN6
chr8:140843946-140890769	7	PTK2
chr1:52860730-52892975	16	ZYG11A
chr2:8770669-8776892	93	KIDINS220
chr3:194644572-194646243	7	LSG1
chr1:233208517-233236980	71	PCNXL2
chr15:90980241-90984140	10	PRC1
chr7:138576372-138579532	10	TRIM24
chr1:109345750-109369589	12	SORT1
chr6:70426438-70452571	81	FAM135A
chr5:1814338-1816054	29	NDUFS6

chr6:37312917-37317206	25	TBC1D22B
chr8:123372636-123380677	7	ATAD2
chr1:45824432-45959477	10	MAST2
chr17:17866295-17869473	13	TOM1L2
chr2:111863674-111863895	89	ANAPC1
chrX:18506934-18510854	23	CDKL5
chr8:528894-531576	10	nogene
chr15:42511832-42515354	11	SNAP23
chr1:236168705-236178636	27	GPR137B
chr7:5057294-5057779	7	RBAK
chr20:2492698-2494044	14	ZNF343
chr5:50274987-50411383	8	EMB
chr15:34488962-34490796	32	nogene
chr14:103338326-103339803	5	EIF5
chr4:13376544-13428299	9	RAB28
chr12:28255580-28307749	209	CCDC91
chr6:610097-619543	28	EXOC2
chr6:5404541-5431172	114	FARS2
chr7:156762133-156826744	11	LMBR1
chr5:109842327-109847790	12	MAN2A1
chr9:126340507-126386658	26	MVB12B
chr8:144196197-144196451	20	nogene
chr15:75407054-75413045	14	SIN3A
chr6:85565331-85572374	16	SNX14
chr6:43498977-43503706	9	TJAP1
chr2:32678452-32702920	9	TTC27
chr1:212329134-212342280	12	PPP2R5A
chr15:40744046-40745259	463	RMDN3
chr3:136366942-136423044	6	STAG1
chr7:105040838-105066807	11	KMT2E
chr5:139600371-139614966	236	UBE2D2
chr8:130076347-130092143	43	ASAP1
chr7:140739811-140808995	12	BRAF
chr9:94955176-94967801	9	C9orf3
chr9:100016321-100022226	9	ERP44
chr9:100239998-100272986	5	nogene
chr16:742249-742543	80	nogene
chr15:76621763-76665789	12	SCAPER
chr14:30947089-30950944	11	STRN3
chr19:46733038-46738888	7	STRN4
chr1:230943805-230958299	7	TTC13
chr3:125277725-125315053	7	ZNF148
chr2:202290322-202297906	9	NOP58

chr2:235930764-235968623	101	AGAP1
chr17:43057051-43063951	18	BRCA1
chr9:135850136-135862608	94	CAMSAP1
chr20:33611093-33611335	11	CBFA2T2
chr10:84438511-84477664	172	CCSER2
chr9:96522505-96565483	347	CDC14B
chr1:3833861-3835092	13	CEP104
chr17:59647397-59651316	8	CLTC
chr17:17260300-17264981	7	COPS3
chr16:3738558-3740549	8	CREBBP
chr6:43196068-43196862	12	CUL9
chr9:123630375-123879021	10	DENND1A
chr1:197642710-197672156	8	DENND1B
chr8:141150833-141168525	8	DENND3
chr9:19288595-19300331	26	DENND4C
chr14:88681889-88688359	11	EML5
chr10:92909426-92920050	5	EXOC6
chr17:30484213-30520021	5	GOSR1
chr11:123594081-123596037	14	GRAMD1B
chr15:41020899-41027736	55	INO80
chr9:7011697-7015929	17	KDM4C
chr17:44933922-44936358	21	KIF18B
chr13:46692535-46728984	10	LRCH1
chr8:88167668-88186598	11	MMP16
chr16:47109482-47122912	5	NETO2
chr10:89792838-89794672	20	nogene
chr3:32282590-32292155	10	nogene
chrX:53097040-53097447	7	nogene
chr7:32812756-32838666	18	nogene
chr4:435769-437511	29	nogene
chr19:17516172-17517850	12	PGLS
chr4:184659339-184678394	34	PRIMPOL
chr1:84179176-84185182	34	PRKACB
chr9:122945331-122986419	7	RABGAP1
chr1:174275832-174371072	60	RABGAP1L
chr5:38990948-38996882	7	RICTOR
chr11:74810183-74817714	139	RNF169
chr9:125018798-125056007	8	SCAI
chr4:82874610-82881940	64	SEC31A
chr11:71109940-71119032	7	SHANK2
chr13:21161789-21168399	7	SKA3
chr17:4938338-4938975	11	SLC25A11
chr10:68483419-68493570	19	SLC25A16

chr2:27668842-27675692	12	SLC4A1AP
chr7:129205609-129206587	16	SMO
chr1:15911100-15922349	5	SPEN
chr7:43595900-43619723	13	STK17A
chr13:52397231-52402681	12	THSD1
chr3:100372605-100386978	9	TOMM70A
chr3:185921103-185922126	316	TRA2B
chr11:18514677-18516164	8	TSG101
chr8:102346235-102347092	28	UBR5
chr8:27294079-27294310	58	TRIM35
chr1:51814034-51814813	11	NRD1
chrX:124022530-124042645	6	STAG2
chr18:46084503-46095131	5	ATP5A1
chr2:173358711-173363462	8	CDCA7
chr18:76927036-76960855	15	ZNF236
chr1:154967670-154969448	5	SHC1
chr6:16657775-16754091	7	ATXN1
chr2:190901946-190910321	7	GLS
chr18:36195256-36205276	26	MOCOS
chr12:96909751-96920125	19	NEDD1
chrX:24814978-24843677	9	POLA1
chr2:46576086-46576656	14	RHOQ
chr4:139517884-139533366	16	SETD7
chr6:42616001-42632904	7	UBR2
chr13:50027206-50045232	7	DLEU2
chr5:69226337-69228387	9	MRPS36
chr6:126112865-126174911	11	nogene
chr2:227519947-227536993	17	AGFG1
chr5:140504820-140505869	19	ANKHD1-EIF4EBP3
chr17:60460662-60479271	11	APPBP2
chr8:61633682-61684188	24	ASPH
chr3:112537734-112550262	27	ATG3
chr1:147259836-147267518	7	CHD1L
chr16:66617230-66623502	7	CMTM4
chr8:67103036-67132080	8	CSPP1
chr15:44496379-44497138	23	CTDSPL2
chr1:77801330-77807101	45	FAM73A
chr5:148394713-148404822	10	FBXO38
chr5:41927012-41934308	10	FBXO4
chr9:120771347-120776226	5	FBXW2
chr2:9475986-9484550	15	IAH1
chr1:200614317-200615609	61	KIF14
chr9:34286616-34290389	45	KIF24

chr17:43377670-43381660	8	LINC00910
chr5:126804775-126805696	19	LMNB1
chr4:150806270-150849575	12	LRBA
chr7:1936686-2014642	15	MAD1L1
chr16:21612644-21625115	98	METTL9
chr10:97460694-97467583	8	MMS19
chr1:144439828-144456805	10	NBPF15
chrX:91616342-91662446	7	nogene
chr18:35322387-35327063	26	nogene
chr9:33676487-33676667	10	nogene
chr20:51900121-51913814	20	nogene
chr3:13386274-13391307	7	NUP210
chr6:117693041-117694180	73	NUS1
chr10:13127744-13133581	11	OPTN
chr16:14552020-14555709	14	PARN
chr14:71045132-71057724	39	PCNX
chr2:229155817-229202012	10	PID1
chr14:101894506-101909690	7	PPP2R5C
chr1:13778417-13782831	9	PRDM2
chr3:49044389-49057890	10	QRICH1
chr6:13641198-13644729	8	RANBP9
chr20:35716739-35739017	8	RBM39
chr1:175986942-176046324	25	RFWD2
chr16:74644361-74661451	61	RFWD3
chr1:26560725-26561663	8	RPS6KA1
chr11:68180131-68190152	11	SUV420H1
chr8:119801793-119806401	7	TAF2
chr4:38089931-38103157	635	TBC1D1
chr22:46878652-46912188	20	TBC1D22A
chr10:119581921-119588248	7	TIAL1
chr21:31182420-31195305	20	TIAM1
chr9:105721536-105774026	43	TMEM38B
chr2:218737578-218740584	13	TTLL4
chr8:102258984-102269108	7	UBR5
chr7:93252652-93297243	12	VPS50
chr22:40881769-40886515	25	XPNPEP3
chr20:47282101-47347926	25	ZMYND8
chr19:51880322-51880877	7	ZNF577
chr19:23373741-23374764	10	ZNF91
chr1:1839189-1853297	32	GNB1
chr1:5904616-5986327	10	NPHP4
chr19:51896471-51900294	10	ZNF649
chr6:100848147-100849096	26	ASCC3

chr2:53897869-53900017	8	PSME4
chr2:61228644-61232532	5	USP34
chr2:72718102-72721164	12	EXOC6B
chr7:23507419-23513082	73	TRA2A
chr2:214745066-214752555	19	BARD1
chr5:34918363-34923234	52	BRIX1
chr11:76496214-76516312	16	C11orf30
chr3:113010642-113017602	18	C3orf17
chr11:34089394-34090678	10	CAPRIN1
chr2:29133654-29143945	12	CLIP4
chr3:47569151-47572874	9	CSPG5
chr14:39276933-39295029	5	CTAGE5
chr5:119105179-119116336	7	DMXL1
chr7:32620403-32656044	8	DPY19L1P1
chr6:20479845-20482949	12	E2F3
chr9:129923843-129929695	11	FNBP1
chr3:119876412-120093593	10	GSK3B
chr5:138561579-138567722	154	HSPA9
chr3:45400244-45458886	7	LARS2
chr6:136694139-136698751	5	MAP3K5
chr14:75039910-75042477	26	MLH3
chr12:5358269-5367934	20	nogene
chr1:145422795-145423431	9	nogene
chr3:8742458-8753224	13	OXTR
chr10:34372497-34384254	35	PARD3
chr10:34516978-34545288	5	PARD3
chr12:53465931-53471807	7	PCBP2
chr1:75787496-75789356	12	RABGGTB
chr7:155700772-155707068	12	RBM33
chr11:4106045-4109703	6	RRM1
chr3:47661293-47676782	6	SMARCC1
chr17:64562770-64571956	26	SMURF2
chr8:47279861-47291137	16	SPIDR
chr3:177046128-177065022	5	TBL1XR1
chr22:28629551-28629830	13	TTC28
chr22:40868998-40870139	19	XPNPEP3
chr17:12054888-12081530	297	MAP2K4
chr9:85618982-85621285	5	AGTPBP1
chr6:17421554-17426690	38	CAP2
chr16:66938036-66939358	93	CES2
chr1:20825099-20829272	9	EIF4G3
chr1:21002712-21111383	33	EIF4G3
chr5:171891466-171914405	6	FBXW11

chr9:108911009-108919349	6	IKBKAP
chr9:88418777-88475513	26	nogene
chr4:107693770-107701285	21	PAPSS1
chr22:20824325-20838731	20	PI4KA
chr1:174637374-174702256	7	RABGAP1L
chr1:100906851-100913806	17	SLC30A7
chr11:72757766-72759381	129	STARD10
chr19:1625575-1632405	16	TCF3
chr9:136898712-136900520	9	TRAF2
chr10:45625950-45627105	13	ZFAND4
chr1:15626668-15643650	13	DDI2
chr12:31495809-31499671	19	DENND5B
chr15:96269847-96290783	9	NR2F2-AS1
chr8:38788703-38820635	18	TACC1
chr12:104311289-104339273	11	TXNRD1
chr1:246647138-246660334	5	CNST
chr11:118489791-118493230	6	KMT2A
chr1:6227856-6228374	12	nogene
chr19:32655116-32655634	10	nogene
chr16:28169749-28177332	14	XPO6
chr1:93210801-93216746	62	CCDC18
chr11:87355505-87362476	9	nogene
chr5:128519272-128527967	7	FBN2
chr3:197756425-197770244	43	FYTTD1
chr7:156763107-156826744	5	LMBR1
chr6:73479731-73480805	5	MTO1
chr1:43898234-43899727	19	ST3GAL3
chr4:78826036-78845049	9	BMP2K
chr1:93183359-93192106	5	CCDC18
chr12:122777490-122798200	13	CCDC62
chr6:118523801-118566316	11	CEP85L
chr3:38501354-38506968	20	EXOG
chr15:41069569-41074565	14	INO80
chr9:100104497-100126549	11	INVS
chr2:85035015-85035847	18	KCMF1
chr1:66940027-66959743	9	MIER1
chr3:125576188-125580040	10	OSBPL11
chr1:151024610-151025673	126	PRUNE
chr15:90219792-90221765	20	SEMA4B
chr9:96539077-96565483	17	CDC14B
chr16:53228982-53231784	23	CHD9
chr6:36515337-36525642	8	STK38
chr12:110018344-110029635	6	ANKRD13A

chr17:5354358-5368468	7	RABEP1
chr12:19462509-19518113	43	AEBP2
chr10:91992118-92008275	19	BTAF1
chr3:47610065-47610327	12	SMARCC1
chr16:16141172-16142942	26	ABCC1
chr16:89112091-89120913	6	ACSF3
chr1:244432544-244437768	6	ADSS
chr2:9344531-9368519	14	ASAP2
chr17:61776400-61780401	210	BRIP1
chr11:66756352-66762450	51	C11orf80
chr7:90716339-90726812	8	CDK14
chr10:100253420-100262063	13	CWF19L1
chr5:113001083-113001674	47	DCP2
chrX:2211488-2221229	10	DHRSX
chr6:15615266-15627475	23	DTNBP1
chr7:107575187-107576592	6	DUS4L
chr1:245017241-245059458	11	EFCAB2
chr8:117818434-117822597	24	EXT1
chr1:27997319-28004419	13	EYA3
chr5:108867766-108872212	60	FER
chr10:13737843-13740577	7	FRMD4A
chr2:24246128-24254431	9	ITSN2
chr7:1980452-2069338	9	MAD1L1
chr2:8943186-8974435	25	MBOAT2
chr4:99028866-99057818	7	METAP1
chr14:75030542-75042477	8	MLH3
chr8:97686667-97691188	189	MTDH
chrX:64354776-64356338	6	MTMR8
chr13:35040932-35110978	11	NBEA
chr12:124362125-124372610	6	NCOR2
chrUn_KI270742v1:1325-34982	14	nogene
chr5:25567671-25572560	10	nogene
chr1:28329576-28329955	12	nogene
chr6:77271339-77316704	7	nogene
chr3:151803705-151803925	9	nogene
chr2:173657935-173693837	7	nogene
chr1:233217898-233236980	129	PCNXL2
chr7:11028680-11051780	6	PHF14
chr12:110536923-110546078	62	PPTC7
chr4:184659339-184666064	11	PRIMPOL
chr15:75517258-75524298	20	PTPN9
chr18:8624937-8633327	6	RAB12
chr6:138941334-138945697	8	REPS1

chr21:31700994-31705467	25	SCAF4
chr17:50979745-50995533	7	SPAG9
chr20:62852722-62857638	6	TCFL5
chr7:47368364-47369621	8	TNS3
chr8:142300485-142318634	6	TSNARE1
chr17:78802323-78803978	15	USP36
chr12:56477659-56478262	10	GLS2
chr11:68569747-68583129	5	PPP6R3
chr1:43792101-43838311	9	ST3GAL3
chr3:197081050-197119530	298	DLG1
chr9:111433232-111442424	16	KIAA0368
chr9:116618323-116620443	24	ASTN2
chr21:37420298-37456308	110	DYRK1A
chr16:72950468-72960194	19	ZFHX3
chr14:74691058-74692328	12	AREL1
chr7:7234302-7243750	9	C1GALT1
chr16:21612644-21618074	8	METTL9
chr9:37424844-37426654	501	GRHPR
chr20:38988812-38994880	24	DHX35
chr1:20941490-20950111	9	EIF4G3
chr18:6237963-6312056	75	L3MBTL4
chr4:82970326-82981475	9	LIN54
chr16:48299661-48303305	7	LONP2
chr6:35672591-35680422	8	nogene
chr8:143640578-143641432	10	nogene
chr7:77778337-77778814	78	RSBN1L
chr4:138214584-138242061	8	SLC7A11
chr15:43002554-43025429	24	UBR1
chr10:110124016-110132400	9	ADD3
chr1:155352705-155360409	18	ASH1L
chr3:11358417-11364734	86	ATG7
chr7:39987598-39988258	207	CDK13
chr11:46783273-46788773	58	CKAP5
chr2:29121373-29135666	122	CLIP4
chrX:16667513-16683226	5	CTPS2
chr7:102227366-102227669	208	CUX1
chr1:63478763-63490218	14	ITGB3BP
chr10:92637183-92645642	17	KIF11
chr18:50895808-50921187	11	ME2
chr22:29978441-29998857	28	MTMR3
chr10:32623718-32635158	9	nogene
chrX:121090398-121164157	14	nogene
chr9:96895895-96898127	13	nogene

chr15:24998325-25000606	6	nogene
chr7:29923955-29969013	5	nogene
chr10:49841949-49861663	20	PARG
chr8:51860860-51861246	18	PCMTD1
chr6:42972882-42975038	213	PEX6
chr11:86014869-86031611	9	PICALM
chr17:1534098-1535518	10	PITPNA
chr2:135645378-135680324	18	R3HDM1
chr6:154787860-154792976	78	SCAF8
chr1:86715723-86719635	95	SH3GLB1
chr19:16081912-16093753	40	TPM4
chr5:14293011-14304592	32	TRIO
chr14:90730074-90786328	35	TTC7B
chr2:112485916-112494375	18	TTL
chr8:102360070-102361626	1053	UBR5
chr8:99431536-99521010	21	VPS13B
chr16:74948518-74956610	16	WDR59
chr18:62539680-62565096	9	ZCCHC2
chr20:51712965-51729978	105	ATP9A
chr19:5047475-5071059	12	KDM4B
chr9:6420911-6421142	13	UHRF2
chr12:122489385-122492789	50	ZCCHC8
chr12:49004963-49013110	10	PRKAG1
chr2:38818876-38819144	7	DHX57
chr1:15770408-15774796	7	FBLIM1
chr17:43047642-43057135	21	BRCA1
chr5:27472382-27485029	7	nogene
chr13:100307191-100340259	9	PCCA
chr1:243545509-243563848	19	AKT3
chr10:125038996-125133612	26	CTBP2
chr3:197136541-197149796	28	DLG1
chr1:8872654-8874334	53	nogene
chr13:26609328-26613058	17	WASF3
chr7:99476402-99479787	6	ZNF789
chr7:148729961-148730283	15	CUL1
chr8:25366869-25369641	20	DOCK5
chr6:592468-637861	9	EXOC2
chr5:163471184-163471463	23	HMMR
chr6:13632369-13658832	40	RANBP9
chr6:135318518-135428759	10	AHI1
chr20:51674152-51690819	121	ATP9A
chr10:21651672-21670704	10	MLLT10
chr10:70217811-70218817	14	PPA1

chr12:112502143-112506422	6	PTPN11
chr6:166328275-166330247	10	SFT2D1
chr2:97813970-97818721	23	TMEM131
chr10:74070669-74074903	18	VCL
chr8:103420263-103421082	30	DCAF13
chr16:57435786-57436253	20	nogene
chr10:11509594-11597717	10	USP6NL
chr3:196927795-196932023	18	SENP5
chr7:66941847-66948694	47	TMEM248
chr3:184849070-184870805	14	VPS8
chr1:20767583-20773610	7	HP1BP3
chr16:12061527-12078915	7	SNX29
chr5:73078179-73082825	43	FCHO2
chr18:21768622-21779685	10	MIB1
chr11:67181319-67207688	12	KDM2A
chr20:34575103-34588607	5	PIGU
chr1:197642710-197772932	28	DENND1B
chr9:128523279-128523846	14	GLE1
chr16:11774235-11779363	20	ZC3H7A
chr12:56282419-56282991	42	CS
chr5:151786571-151800869	40	G3BP1
chr15:59080935-59081284	9	RNF111
chr7:7877280-7899036	5	UMAD1
chr1:93876267-93877862	24	DNTTIP2
chr8:98104488-98108751	19	HRSP12
chr1:53923903-53940097	18	HSPB11
chr12:2822793-2824252	53	ITFG2
chr17:16186553-16194639	27	NCOR1
chr11:64927801-64930589	11	PPP2R5B
chr9:125543058-125559809	29	MAPKAP1
chr1:94458606-94468007	11	ABCD3
chr1:179118586-179121867	36	ABL2
chr1:180314691-180413471	11	ACBD6
chr1:180397515-180430262	86	ACBD6
chr2:235797758-235799522	30	AGAP1
chr19:32631401-32642145	18	ANKRD27
chr19:46845992-46846824	10	AP2S1
chr4:36210389-36229645	13	ARAP2
chr4:147906637-147913178	10	ARHGAP10
chr17:12908896-12919831	28	ARHGAP44
chr11:120440128-120447085	8	ARHGEF12
chr2:218238663-218239484	29	ARPC2
chr20:51674152-51697482	9	ATP9A

chr22:45793544-45807022	95	ATXN10
chr17:83014453-83048785	14	B3GNTL1
chr2:214780559-214797117	19	BARD1
chr20:50876485-50877587	12	BCAS4
chr11:102350345-102368548	6	BIRC2
chr2:32377587-32392150	29	BIRC6
chr1:32383829-32386895	5	BSDC1
chr11:46987207-47052518	6	C11orf49
chr10:118701305-118730410	27	CACUL1
chr6:17540972-17551604	7	CAP2
chr1:112647209-112659779	6	CAPZA1
chr15:44328684-44332078	16	CASC4
chrX:41626603-41671530	8	CASK
chr2:113460672-113482228	11	CBWD2
chr2:108839184-108850755	12	CCDC138
chr2:61876094-61883548	17	CCT4
chr13:26400452-26401624	117	CDK8
chr19:4433069-4433539	9	CHAF1A
chr2:121515664-121530325	36	CLASP1
chr4:77773080-77776392	476	CNOT6L
chr9:121161855-121166180	60	CNTRL
chr16:23413447-23418827	18	COG7
chr6:99375939-99380341	36	COQ3
chr5:138886211-138925407	8	CTNNA1
chr11:108094390-108098529	8	CUL5
chr1:173830659-173833499	11	DARS2
chr4:182891402-182894605	17	DCTD
chr5:150753478-150756487	10	DCTN4
chr4:51863436-51914078	6	DCUN1D4
chr11:82914722-82931849	23	DDIAS
chr11:11998695-12008608	8	DKK3
chr16:46964610-46967646	6	DNAJA2
chr7:103316829-103319678	11	DNAJC2
chr5:31431575-31451640	19	DROSHA
chr6:15615266-15652140	7	DTNBP1
chr16:2246094-2246986	23	ECI1
chr8:131940498-131946633	350	EFR3A
chr2:62707163-62831158	7	EHBP1
chr2:62987930-62996766	13	EHBP1
chr1:44881611-44897444	8	EIF2B3
chr3:184140079-184140730	10	EIF2B5
chr3:186784563-186786051	25	EIF4A2
chr1:20857402-20862332	6	EIF4G3

chrX:130069299-130072417	14	ELF4
chr7:37948839-37950392	42	EPDR1
chr7:133356329-133375002	7	EXOC4
chr10:92893348-92909631	54	EXOC6
chr8:117830229-117837201	8	EXT1
chr9:93527154-93550691	10	FAM120A
chr10:119107947-119123569	11	FAM45A
chrX:14843659-14859334	34	FANCB
chr1:224130471-224134212	9	FBXO28
chr20:6096901-6097631	12	FERMT1
chr12:95208842-95211267	7	FGD6
chr2:152560882-152581049	7	FMNL2
chr5:131698916-131744690	8	FNIP1
chr9:20715321-20720534	94	FOCAD
chr9:20715321-20789608	43	FOCAD
chr16:70468848-70469323	8	FUK
chr5:151786571-151791062	104	G3BP1
chr1:230236013-230243427	16	GALNT2
chr6:53520777-53522527	28	GCLC
chr10:5785851-5800705	196	GDI2
chr7:80199224-80203832	10	GNAI1
chr22:23095246-23096418	18	GNAZ
chr3:37251394-37282272	14	GOLGA4
chr1:155795049-155814449	24	GON4L
chr9:130075635-130083602	7	GPR107
chr1:168104476-168114549	13	GPR161
chr8:30700080-30703240	18	GSR
chr6:57919783-57924591	21	GUSBP4
chr10:67988662-68073686	7	HERC4
chr22:35262322-35293743	31	HMGXB4
chr8:42925556-42964474	13	HOOK3
chr15:98707561-98708107	90	IGF1R
chr1:156530104-156531247	5	IQGAP3
chr20:34424479-34438631	9	ITCH
chr12:26550246-26580155	7	ITPR2
chr13:30208514-30283791	12	KATNAL1
chr11:108477606-108491167	11	KDELC2
chr13:46368041-46387879	7	KIAA0226L
chr9:111391755-111394305	10	KIAA0368
chr9:112574056-112575251	390	KIAA1958
chr1:10267379-10268263	30	KIF1B
chr2:207123773-207124404	8	KLF7
chr1:6599046-6599452	28	KLHL21

chr3:122437169-122442116	33	KPNA1
chr7:92213194-92214777	13	KRIT1
chr15:51959898-51960733	6	LEO1
chr7:156724111-156734257	36	LMBR1
chr8:89722014-89725641	15	LOC101929709
chr4:150798080-150831976	43	LRBA
chr1:235800319-235801097	10	LYST
chr3:185437446-185443636	20	MAP3K13
chr14:70800666-70801080	17	MAP3K9
chr12:111865249-111880527	8	MAPKAPK5
chr5:10415487-10426522	9	6-Mar
chr2:159743060-159748903	13	7-Mar
chr18:54155237-54166166	21	MBD2
chr1:84947032-84958702	8	MCOLN2
chr11:86446313-86448255	14	ME3
chr1:29216004-29216812	11	MECR
chr17:61992539-62011233	7	MED13
chr12:116205052-116237705	15	MED13L
chr10:124760385-124774782	12	METTL10
chr14:21503172-21503881	876	METTL3
chr17:76767385-76776541	28	MFSD11
chr18:21819494-21847125	10	MIB1
chr11:122155550-122202909	15	MIR100HG
chr5:80787564-80792844	26	MSH3
chr11:47627079-47631087	16	MTCH2
chr15:79889092-79889354	10	MTHFS
chr1:236835546-236853088	7	MTR
chr2:176296859-176337577	37	MTX2
chr5:53603451-53604895	9	NDUFS4
chr12:8095600-8096312	5	NECAP1
chr1:16447869-16452015	26	NECAP2
chr12:96909751-96917737	7	NEDD1
chr15:73176402-73178427	19	NEO1
chr5:43655839-43677806	13	NNT
chr12:5160306-5171889	40	nogene
chrX:152574135-152574980	15	nogene
chr1:240092041-240092400	9	nogene
chrX:135942878-135960073	9	nogene
chr11:28916880-28922625	9	nogene
chr5:150367868-150368846	9	nogene
chr1:47368486-47373075	6	nogene
chrX:74277947-74278679	10	nogene
chr12:121291123-121291811	10	nogene

chr16:29807306-29807824	15	nogene
chr12:87648516-87651983	7	nogene
chr12:5377776-5378105	5	nogene
chr3:161284716-161285241	10	nogene
chr19:653711-654297	5	nogene
chr4:80335743-80376917	6	nogene
chr2:171016000-171016251	40	nogene
chr11:29230935-29384112	8	nogene
chr4:148435103-148436862	213	NR3C2
chr5:177248180-177260168	6	NSD1
chr7:33026816-33035988	7	NT5C3A
chr9:131174054-131178410	56	NUP214
chr16:56837607-56839604	38	NUP93
chr11:3727074-3753408	5	NUP98
chr1:58527260-58536741	48	OMA1
chrX:68081700-68206673	7	OPHN1
chr22:30741160-30743835	11	OSBP2
chr11:77332729-77353599	15	PAK1
chr4:39872985-39890364	24	PDS5A
chr13:32753331-32760720	7	PDS5B
chr3:170117225-170122744	13	PHC3
chr20:35847349-35863400	32	PHF20
chrX:53987077-53987944	34	PHF8
chr10:13291830-13298245	7	PHYH
chr10:13283689-13295606	13	PHYH
chr11:86012280-86031611	50	PICALM
chr11:17105168-17112666	16	PIK3C2A
chr3:179203543-179204588	11	PIK3CA
chr6:99412550-99416428	18	PNISR
chr22:43926934-43928889	13	PNPLA3
chr2:55683784-55687705	12	PNPT1
chr10:70204872-70209685	32	PPA1
chr10:119172381-119174592	11	PRDX3
chr1:171579353-171584526	10	PRRC2C
chr14:73147794-73173707	275	PSEN1
chr9:15486005-15490124	7	PSIP1
chr2:53866084-53887467	9	PSME4
chr9:112262434-112297916	585	PTBP3
chr8:140864311-140912467	5	PTK2
chr18:8113485-8143779	11	PTPRM
chr7:105481051-105506273	32	PUS7
chr9:121190553-121193419	9	RAB14
chr4:13376544-13460828	11	RAB28

chr17:5338018-5346925	13	RABEP1
chr9:122957010-123020459	24	RABGAP1
chr14:67885868-67887204	13	RAD51B
chr17:58696692-58709990	9	RAD51C
chr1:64802975-64814840	14	RAVER2
chr16:74636345-74637970	101	RFWD3
chr10:119515508-119527424	45	RGS10
chr9:5656582-5666072	9	RIC1
chr2:86740926-86753558	232	RMND5A
chr1:145771710-145794196	7	RNF115
chr9:35546429-35548535	350	RUSC2
chr3:128081253-128087808	26	RUVBL1
chr6:130175840-130222813	7	SAMD3
chr2:199323802-199381820	16	SATB2
chr5:78415519-78421960	190	SCAMP1
chr14:30670255-30708065	13	SCFD1
chr4:82856951-82857764	11	SEC31A
chr2:179172119-179191866	17	SESTD1
chr10:7283903-7285954	18	SFMBT2
chr10:116944923-116948997	13	SHTN1
chr5:176313690-176324757	23	SIMC1
chr1:2302977-2304585	36	SKI
chr7:5287805-5291821	8	SLC29A4
chr4:42018110-42039053	15	SLC30A9
chr2:113723103-113735858	8	SLC35F5
chr4:102304316-102315739	5	SLC39A8
chr6:70732377-70773425	49	SMAP1
chr9:70277309-70286271	7	SMC5
chr15:75617407-75621056	56	SNUPN
chr1:179302676-179335657	8	SOAT1
chr18:12506476-12512531	13	SPIRE1
chr16:30728965-30729572	447	SRCAP
chr16:30720712-30722286	9	SRCAP
chr12:63983946-63997128	12	SRGAP1
chr12:108792285-108792829	27	SSH1
chr8:98526741-98596169	5	STK3
chr12:130806981-130808704	10	STX2
chr17:37561194-37571987	9	SYNRG
chr6:121223235-121242339	7	TBC1D32
chr15:56919891-57091891	21	TCF12
chr2:74003109-74080591	5	TET3
chr1:246540937-246551302	9	TFB2M
chr3:100713642-100720058	12	TFG

chrX:123665641-123712908	7	THOC2
chr15:29737263-29762438	9	TJP1
chr12:26990449-26996862	7	TM7SF3
chr7:130201849-130202663	10	TMEM209
chr19:12714820-12715710	7	TNPO2
chr7:128989960-128993914	18	TNPO3
chr16:3675323-3690985	8	TRAP1
chr6:123331878-123382147	6	TRDN
chrX:101020486-101038051	6	TRMT2B
chr5:160064931-160065766	8	TTC1
chr1:154249238-154251653	20	UBAP2L
chr15:82404611-82414804	19	UBE2Q2P2
chr9:33900173-33912098	6	UBE2R2
chr8:102327741-102346395	59	UBR5
chr3:196369420-196403019	5	UBXN7
chr17:31865365-31878781	20	UTP6
chr10:75219062-75219356	31	VDAC2
chr2:159282671-159283093	13	WDSUB1
chr8:86411537-86411874	8	WWP1
chr9:37126311-37206494	21	ZCCHC7
chr3:125313307-125331233	9	ZNF148
chr3:44455120-44455696	17	ZNF445
chr18:76370527-76380303	24	ZNF516
chr19:37707913-37711692	23	ZNF607
chr19:40081818-40084798	11	ZNF780A
chr4:145823421-145849561	108	ZNF827
chr19:52373110-52374427	60	ZNF880
chr19:23374637-23376330	8	ZNF91
chr22:41117186-41117821	20	EP300
chr11:46493607-46512813	73	AMBRA1
chr3:56139508-56149132	11	ERC2
chr18:21791373-21812468	6	MIB1
chr18:1269669-1278633	14	nogene
chr21:44079563-44080127	17	TRAPPC10
chr1:155670319-155771217	20	YY1AP1
chr5:136153591-136163391	15	SMAD5
chr4:38089931-38125131	19	TBC1D1
chr13:95986332-95999307	5	UGGT2
chr18:79126266-79154555	108	ATP9B
chr14:105226068-105241414	24	BRF1
chr9:74996446-75017448	74	CARNMT1
chrX:16698235-16702941	13	CTPS2
chr14:72714422-72731934	42	DPF3

chr7:6044561-6050045	19	EIF2AK1
chr12:1027747-1371977	7	ERC1
chr8:129871374-129903350	19	FAM49B
chr16:89752137-89762022	9	FANCA
chr4:173224113-173248440	7	GALNT7
chr12:112269703-112273795	20	HECTD4
chr11:67180078-67207688	9	KDM2A
chr12:122544158-122547969	21	KNTC1
chr10:72508154-72566794	7	MICU1
chr1:61332511-61359274	7	NFIA
chr14:102743477-102743710	14	nogene
chr11:67873205-67874489	10	nogene
chr1:51827795-51834170	74	NRD1
chr10:13123994-13136897	7	OPTN
chr18:24280841-24377535	16	OSBPL1A
chr1:233090060-233112966	11	PCNXL2
chr15:85113872-85117839	9	PDE8A
chr12:79828319-79872938	9	PPP1R12A
chr1:31028795-31059439	5	PUM1
chr2:135602499-135605016	158	R3HDM1
chr15:55223888-55230486	9	RAB27A
chr4:37631384-37638504	1589	RELL1
chr12:118019071-118028030	29	RFC5
chr19:11033289-11035132	9	SMARCA4
chr16:30733279-30734008	46	SRCAP
chr1:47280240-47282459	205	STIL
chr6:149378017-149379518	8	TAB2
chr3:51674270-51684634	14	TEX264
chr9:71694508-71698204	9	TMEM2
chr2:112502911-112503181	31	TTL
chr8:48043032-48043626	37	UBE2V2
chr13:95832926-95837202	128	UGGT2
chr15:40902615-40903831	64	VPS18
chr4:176149806-176151967	52	WDR17
chr8:76704042-76708048	14	ZFHX4
chr14:49802452-49806133	7	NEMF
chr20:13483226-13630152	7	TASP1
chr1:46077514-46131956	10	PIK3R3
chr4:7855465-7872080	26	AFAP1
chr16:934189-954666	31	LMF1
chrX:150445453-150462846	10	MAMLD1
chr11:63669077-63669316	8	nogene
chr2:237973091-237973776	14	UBE2F

chr19:57876915-57889008	11	ZNF814
chr10:125110989-125122868	384	CTBP2
chr15:76841733-76883876	61	SCAPER
chr19:12457430-12466850	6	ZNF709
chrX:102825992-102865152	9	LINC00630
chr12:123309309-123315660	9	SBNO1
chr17:60889694-60924500	7	BCAS3
chr5:6761219-6764872	17	nogene
chr6:87601783-87612248	9	ORC3
chr19:5650977-5654456	19	SAFB
chr14:31306731-31309182	23	HEATR5A
chr19:32937630-32966466	12	CEP89
chr2:36464533-36522313	11	CRIM1
chr6:131884930-131886724	13	ENPP1
chr9:92256679-92260234	37	IARS
chr16:31120185-31128139	10	KAT8
chr6:150955994-151037117	13	MTHFD1L
chr2:15394226-15417712	17	NBAS
chr19:52585239-52592228	9	nogene
chr5:50788522-50797233	12	PARP8
chr9:126962224-126977745	14	RALGPS1
chr17:80707840-80709114	8	RPTOR
chr22:31580271-31589577	6	SFI1
chr7:832504-833194	8	SUN1
chr22:25661575-25674528	19	ADRBK2
chr15:34930257-34942080	6	AQR
chr16:88004294-88018667	8	BANP
chr7:73539881-73543644	9	BCL7B
chr4:113500462-113531299	10	CAMK2D
chr11:10800730-10802209	14	EIF4G2
chr12:5169153-5171889	8	nogene
chr12:131939172-131941983	13	PUS1
chr2:135090997-135113270	9	RAB3GAP1
chr2:226839408-226914351	32	RHBDD1
chr1:47260288-47269866	6	STIL
chr6:3157406-3226669	48	TUBB2A
chr7:157216866-157225539	8	UBE3C
chr6:41871563-41907287	10	USP49
chr11:66759042-66762450	70	C11orf80
chrX:85978766-86027557	80	CHM
chr1:21089137-21123555	14	EIF4G3
chr10:97436416-97437750	139	EXOSC1
chr15:43753823-43756766	55	PDIA3

chr8:139988725-140024079	9	TRAPPC9
chr11:77622499-77629899	46	CLNS1A
chr12:11028400-11121180	9	nogene
chr2:241246751-241268541	8	HDLBP
chr1:235480053-235484515	7	B3GALNT2
chr17:63756239-63756570	8	CCDC47
chr8:74314860-74315620	6	JPH1
chr10:113876520-113884380	86	NHLRC2
chr5:143295459-143300763	21	NR3C1
chr12:122935271-122940995	10	ABCB9
chr2:108659604-108673029	17	LIMS1
chr10:92504793-92537550	9	IDE
chr12:50427761-50454417	6	LARP4
chr11:78465875-78478683	24	NARS2
chr16:70762539-70763025	6	VAC14
chr20:56382984-56386533	10	AURKA
chr20:49639677-49640665	77	B4GALT5
chr5:139317052-139319033	21	MATR3
chr3:196361843-196372042	9	UBXN7
chr3:195381008-195411009	8	ACAP2
chr1:6281101-6294980	19	ACOT7
chr5:140440118-140441142	13	ANKHD1-EIF4EBP3
chr11:129062279-129066868	78	ARHGAP32
chr1:62816574-62841547	9	ATG4C
chrX:80699956-80704846	33	BRWD3
chr2:61873001-61879011	10	CCT4
chr4:55959681-55965859	7	CEP135
chr19:32915336-32915517	12	CEP89
chr1:9740657-9741256	50	CLSTN1
chr12:70278131-70285586	8	CNOT2
chr8:141151618-141155989	16	DENND3
chr12:1104749-1141787	101	ERC1
chr2:131071854-131082657	45	FAM168B
chr9:95135345-95172147	8	FANCC
chr2:241403827-241407615	20	FARP2
chr2:36581289-36591011	37	FEZ2
chr10:102362474-102365599	14	GBF1
chr1:170539209-170544845	7	GORAB
chr16:46906841-46909927	11	GPT2
chr6:104730302-104750472	8	HACE1
chr1:6231901-6233643	74	ICMT
chr7:128400094-128401116	11	IMPDH1
chr12:122544158-122546674	9	KNTC1

chr2:191693202-191693416	9	LOC105747689
chr4:150735257-150896456	19	LRBA
chr2:31883385-31920910	27	MEMO1
chr13:23798723-23837756	12	MIPEP
chr1:11247624-11248094	12	MTOR
chr4:17814852-17815358	342	NCAPG
chr17:31223443-31227606	19	NF1
chr6:141900322-141927052	8	nogene
chr9:37160064-37165935	9	nogene
chr3:17562018-17733460	6	nogene
chr5:85805306-85821466	65	nogene
chr2:219917762-219940777	8	nogene
chr3:48548531-48550234	19	PFKFB4
chr10:22607926-22609717	51	PIP4K2A
chr2:158533179-158577383	24	PKP4
chr12:42374955-42398994	8	PPHLN1
chr10:87875404-87952259	8	PTEN
chr6:128078812-128089992	16	PTPRK
chr6:127280230-127287007	19	RNF146
chr7:5641153-5652510	123	RNF216
chr19:47143493-47155213	57	SAE1
chr4:128936654-128943188	7	SCLT1
chr6:2836107-2840594	6	SERPINB1
chrX:136022585-136033493	40	SLC9A6
chrX:129504733-129511983	11	SMARCA1
chr18:2738396-2739520	9	SMCHD1
chr16:30298376-30301358	7	SMG1P5
chr4:185247293-185267155	877	SNX25
chr9:100320264-100330169	23	TEX10
chr1:10149183-10151561	6	UBE4B
chr12:101308184-101319635	6	UTP20
chr2:63433744-63439855	30	WDPCP
chr6:43570501-43570994	70	XPO5
chr3:141368620-141427821	8	ZBTB38
chr15:78493908-78497311	9	IREB2
chr1:113614600-113622994	8	MAGI3
chr5:112818965-112828972	16	APC
chr11:129123445-129127305	68	ARHGAP32
chr21:39264459-39298582	8	BRWD1
chr21:39247700-39258672	24	BRWD1
chr8:86515461-86531229	14	CPNE3
chr3:51159243-51160702	19	DOCK3
chr7:22977340-22991139	7	FAM126A

chr6:149572284-149580723	11	GINM1
chr1:88859881-88860286	10	GTF2B
chr10:67954594-68044563	9	HERC4
chr4:165263196-165297725	7	KLHL2
chr16:48252130-48256741	9	LONP2
chr7:131375423-131411383	19	MKLN1
chr2:42640172-42656302	7	MTA3
chr1:220212886-220213979	6	RAB3GAP2
chr3:186791724-186804724	26	RFC4
chr20:38057531-38059520	18	RPRD1B
chr9:109080838-109093591	16	TMEM245
chr6:110887504-110890356	590	AMD1
chr14:52652238-52666495	11	ERO1A
chr3:153164765-153247531	7	nogene
chr3:178908521-178909706	15	nogene
chr1:152226425-152251758	29	nogene
chr15:56115288-56115696	13	nogene
chr6:78997413-79026164	7	PHIP
chr3:136102006-136106322	8	PPP2R3A
chr12:120562944-120563943	25	RNF10
chr3:149846010-149912083	78	RNF13
chr2:237973091-237973747	40	UBE2F
chr2:61229547-61236389	6	USP34
chr6:43549488-43555964	79	XPO5
chr5:175492094-175512196	20	SFXN1
chr3:167653260-167658326	13	WDR49
chr10:79118914-79162133	21	ZMIZ1
chr9:123757702-123792630	47	DENND1A
chr17:18299558-18306966	9	TOP3A
chr10:73396518-73400857	362	ANXA7
chr15:65756139-65761438	28	DENND4A
chr5:154927384-154932250	9	GEMIN5
chr15:64823777-64824354	36	PIF1
chr9:137467316-137484736	5	PNPLA7
chr8:47783741-47785317	12	PRKDC
chr13:24284134-24290884	8	SPATA13
chr1:31337174-31339048	178	ZCCHC17
chr1:224365878-224371423	28	CNIH4
chr10:15135273-15141557	9	NMT2
chr17:2163759-2172859	8	SMG6
chr22:31810520-31815212	40	DEPDC5
chr9:93515669-93516269	34	FAM120A
chr17:46094559-46169605	17	KANSL1

chr7:66088806-66089695	17	ASL
chr2:202464808-202467689	467	BMPR2
chr2:25576837-25607321	47	DTNB
chr1:8862886-8864092	12	ENO1
chr6:130858130-130865634	7	EPB41L2
chr16:89791402-89796018	7	FANCA
chr9:20881870-20929596	10	FOCAD
chr2:38880178-38881578	11	MORN2
chr18:63280886-63281214	17	nogene
chr12:42210354-42210680	342	nogene
chr2:119926597-119934958	57	PTPN4
chr17:31887235-31889403	7	UTP6
chr14:69769510-69770042	24	nogene
chr4:4434769-4438509	7	STX18
chr17:43095845-43124115	21	BRCA1
chr6:38277564-38288461	10	BTBD9
chr1:15630324-15643650	8	DDI2
chr20:28590556-28591519	7	FRG1CP
chr1:77949154-77962930	12	FUBP1
chr19:19457006-19465614	15	GATAD2A
chr19:45550128-45552285	10	nogene
chr12:8930434-8930927	11	PHC1
chr12:106814695-106825820	13	RIC8B
chr5:39002534-39030572	17	RICTOR
chr14:89984515-89993483	14	TDP1
chr12:123603847-123606159	12	DDX55
chr2:177461892-177468524	19	AGPS
chr22:23273080-23273774	26	BCR
chr3:138542690-138572932	5	CEP70
chr2:241407536-241418109	20	FARP2
chr21:33765874-33767828	14	ITSN1
chr3:183086692-183092545	40	MCCC1
chr1:213077695-213104569	5	RPS6KC1
chr2:179172119-179237433	11	SESTD1
chr1:186318446-186318828	18	TPR
chr11:43379232-43389821	21	TTC17
chr20:49260462-49266266	9	ZNFX1
chr21:41237512-41257326	52	BACE2
chr19:36472910-36476616	7	ZNF566
chr16:2319580-2319840	31	ABCA3
chr13:95161188-95188542	52	ABCC4
chr13:95206531-95210781	29	ABCC4
chr3:53874210-53878467	7	ACTR8

chr4:22389087-22402799	52	ADGRA3
chr12:110388511-110396452	20	ANAPC7
chr12:110382842-110396452	26	ANAPC7
chr17:4206320-4217118	18	ANKFY1
chr18:9204475-9221999	51	ANKRD12
chr5:112754872-112801383	6	APC
chr2:68490189-68526242	99	APLF
chr21:25897572-25911962	42	APP
chr4:152863605-152888307	14	ARFIP1
chr5:142901934-142913293	8	ARHGAP26
chr8:123325892-123337821	7	ATAD2
chr12:50814009-50814439	51	ATF1
chr2:215325986-215333457	6	ATIC
chr11:108330213-108335961	16	ATM
chr1:93571669-93576129	40	BCAR3
chr16:31111299-31112027	6	BCKDK
chr17:43045661-43063951	16	BRCA1
chrX:80744031-80745729	39	BRWD3
chr19:57961390-57966632	7	C19orf18
chr8:90978578-90985841	5	C8orf88
chr22:24095930-24113748	7	CABIN1
chr11:34086063-34092056	10	CAPRIN1
chr1:112653597-112667145	85	CAPZA1
chr6:89853165-89857199	17	CASP8AP2
chr19:11349653-11350199	7	CCDC159
chr3:191357087-191380932	8	CCDC50
chr10:32685969-32729457	11	CCDC7
chr6:110217701-110219869	19	CDC40
chr2:174875787-174878128	54	CHN1
chr19:1270927-1272051	40	CIRBP
chr7:107527236-107548332	25	COG5
chr16:3850296-3854850	10	CREBBP
chr2:36378356-36396787	242	CRIM1
chr2:36369107-36396787	303	CRIM1
chr5:157294782-157304366	8	CYFIP2
chr8:30177125-30180630	9	DCTN6
chr3:57663635-57672438	18	DENND6A
chr12:32698220-32701562	9	DNM1L
chr2:25235706-25237005	11	DNMT3A
chrX:118542724-118543593	7	DOCK11
chr14:101991526-101995300	26	DYNC1H1
chr11:103303092-103358359	85	DYNC2H1
chr11:103253284-103307831	7	DYNC2H1

chr16:22256747-22258695	9	EEF2K
chr9:95997245-96004701	7	ERCC6L2
chr1:44247938-44352925	8	ERI3
chr5:138006989-138011149	11	FAM13B
chr4:151566137-151588926	5	FAM160A1
chr7:102772408-102787469	24	FAM185A
chr10:124696157-124706887	33	FAM53B
chr5:108798123-108799985	11	FER
chr20:6094938-6097631	9	FERMT1
chrX:154354824-154357623	9	FLNA
chr9:105506398-105539351	11	FSD1L
chrX:74274341-74286170	5	FTX
chrX:74274341-74281848	8	FTX
chr3:142198801-142215692	9	GK5
chr7:42148225-42172686	25	GLI3
chr3:179413411-179426242	20	GNB4
chr3:37294978-37296219	6	GOLGA4
chr4:70831538-70833273	17	GRSF1
chr22:23709129-23709867	10	GUSBP11
chr9:19076601-19096769	32	HAUS6
chr12:112229697-112231715	29	HECTD4
chr10:67966682-68073686	11	HERC4
chr2:190244886-190261234	14	HIBCH
chr5:43294690-43298975	5	HMGCS1
chr1:20757165-20780540	45	HP1BP3
chr2:197503038-197503330	35	HSPE1
chr8:42290155-42293568	10	IKBKB
chr17:61911519-61925099	16	INTS2
chr17:46082440-46162525	18	KANSL1
chr13:30227411-30231472	78	KATNAL1
chr11:67231565-67231960	6	KDM2A
chr18:63355203-63362868	7	KDSR
chr16:29799627-29800048	32	KIF22
chr7:152249875-152250966	7	KMT2C
chr7:105062163-105066807	33	KMT2E
chr6:108319616-108347039	7	LACE1
chr5:146168127-146172774	12	LARS
chr17:38878085-38898519	7	LASP1
chr2:30552492-30568176	61	LCLAT1
chr9:110972072-111036201	26	LPAR1
chr16:192926-199238	9	LUC7L
chr15:67563282-67600749	8	MAP2K5
chr22:21769194-21805945	15	MAPK1

chr6:36072872-36080086	31	MAPK14
chr1:156475107-156477202	33	MEF2D
chr17:2438108-2467876	8	METTL16
chr17:43808955-43811205	20	MPP3
chr11:66435972-66438259	9	MRPL11
chr5:154950820-154967237	238	MRPL22
chr21:25592811-25601467	8	MRPL39
chr13:40749137-40759106	9	MRPS31
chr3:9689963-9702064	32	MTMR14
chr19:17197791-17198308	6	MYO9B
chr2:15424314-15511350	6	NBAS
chr2:105816429-105855289	8	NCK2
chr12:124419956-124449867	19	NCOR2
chr20:36687417-36688784	16	NDRG3
chr9:124312508-124327445	8	NEK6
chr17:31325819-31326252	12	NF1
chr9:33311105-33319127	13	NFX1
chr1:236024069-236038253	31	NID1
chr5:43700118-43704400	15	NNT
chr8:128009567-128010444	8	nogene
chr3:183015418-183022557	5	nogene
chr3:61622462-61623140	8	nogene
chr11:87355505-87372868	7	nogene
chr2:241322542-241323215	7	nogene
chrX:63314996-63345571	12	nogene
chr12:122877801-122879505	10	nogene
chr11:3395712-3397406	9	nogene
chr5:66191155-66203030	11	nogene
chr6:52429968-52430374	19	nogene
chr21:43729851-43730220	17	nogene
chr9:88418849-88462631	5	nogene
chr19:54204328-54205846	8	nogene
chr4:128017357-128021238	9	nogene
chr2:25709302-25722165	5	nogene
chr9:97139852-97148100	55	nogene
chr15:96269847-96271798	5	NR2F2-AS1
chr1:229463542-229465519	16	NUP133
chr13:25307805-25325068	11	NUPL1
chr5:14673641-14681607	168	OTULIN
chr3:8767265-8768591	11	OXTR
chr7:140046883-140058034	49	PARP12
chr11:34931403-34978182	16	PDHX
chr11:34947505-34978182	24	PDHX

chr7:149014903-149019197	9	PDIA4
chr13:32732100-32735330	55	PDS5B
chr15:77284957-77286504	16	PEAK1
chr8:132777795-132804740	8	PHF20L1
chrX:54022258-54022843	6	PHF8
chr11:85981744-85996929	77	PICALM
chr12:106405856-106410960	5	POLR3B
chr20:18480037-18481810	8	POLR3F
chr9:131436619-131439061	99	PRRC2B
chr2:53874338-53885775	36	PSME4
chr9:112275843-112297916	69	PTBP3
chr1:31915894-31919658	1204	PTP4A2
chr4:86672364-86693674	25	PTPN13
chr20:2947981-2965202	145	PTPRA
chr7:66783674-66799414	53	RABGEF1
chr7:6387211-6400188	7	RAC1
chr14:67823541-67825577	14	RAD51B
chr22:41245114-41246672	7	RANGAP1
chr17:2991296-3008145	9	RAP1GAP2
chr3:141509567-141559995	6	RASA2
chr12:64684786-64694894	17	RASSF3
chr14:73077368-73083551	14	RBM25
chr5:146218984-146237432	6	RBM27
chr7:155665174-155680908	167	RBM33
chr7:74239937-74246763	12	RFC2
chr9:5656582-5720750	5	RIC1
chr5:38971876-38982036	7	RICTOR
chr12:120557281-120560886	29	RNF10
chr18:32042178-32045732	5	RNF125
chr2:210015972-210021064	46	RPE
chr16:53686432-53692364	6	RPGRIP1L
chr14:90978305-91001159	42	RPS6KA5
chr7:77749423-77750064	9	RSBN1L
chr7:77765494-77778446	15	RSBN1L
chr3:134155135-134195182	8	RYK
chr2:199380363-199433514	41	SATB2
chr7:66993216-66994282	38	SBDS
chr4:128992166-128999794	8	SCLT1
chr12:51684173-51689096	26	SCN8A
chr5:254392-256455	8	SDHA
chr20:18524934-18530803	5	SEC23B
chr4:82871943-82875822	230	SEC31A
chr7:85055716-85068284	79	SEMA3D

chr3:72812670-72817384	12	SHQ1
chr8:103355210-103407784	7	SLC25A32
chr12:104927972-104931791	33	SLC41A2
chrX:129515686-129518447	12	SMARCA1
chr1:151658234-151662270	12	SNX27
chr3:113488944-113506605	275	SPICE1
chr12:64091275-64115893	32	SRGAP1
chr16:2757471-2757945	13	SRRM2
chr1:112576985-112582472	7	ST7L
chr8:73673106-73739868	396	STAU2
chr20:44975323-44987296	16	STK4
chr11:68166981-68190152	26	SUV420H1
chr11:9713465-9732710	30	SWAP70
chr17:37440504-37444768	26	TADA2A
chr8:119791323-119802025	10	TAF2
chr12:64485454-64490119	5	TBK1
chr6:84738496-84748087	24	TBX18
chr9:100303631-100310379	52	TEX10
chr1:246544520-246551302	7	TFB2M
chr3:142054114-142101841	49	TFDP2
chr13:52397231-52398194	19	THSD1
chr3:9383198-9384825	30	THUMPD3
chr3:156677656-156678614	17	TIPARP
chr6:10749201-10755232	30	TMEM14B
chr7:130173631-130175609	7	TMEM209
chr9:109073355-109108570	17	TMEM245
chr1:25340473-25356853	14	TMEM50A
chr15:42233212-42268032	8	TMEM87A
chr12:82857009-82857580	185	TMTC2
chr5:72887069-72889957	37	TNPO1
chr7:5345561-5359569	5	TNRC18
chr22:40273424-40280143	10	TNRC6B
chr7:23506137-23521840	8	TRA2A
chr15:50574636-50575789	8	TRPM7
chr22:28629551-28656539	7	TTC28
chr1:162576776-162597858	57	UAP1
chr9:33953284-33973237	136	UBAP2
chr15:43043214-43056442	6	UBR1
chr6:42603587-42617338	6	UBR2
chr1:62439798-62442299	12	USP1
chr3:179764001-179765848	86	USP13
chr2:61256383-61266167	9	USP34
chr6:144537581-144557311	12	UTRN

chr8:99641810-99661491	16	VPS13B
chr8:99096311-99121445	47	VPS13B
chr15:61929500-61931259	13	VPS13C
chr10:28589735-28590832	7	WAC
chr5:77449759-77489572	27	WDR41
chr1:108982608-108986680	21	WDR47
chr16:28175897-28181031	11	XPO6
chr20:44901530-44906096	7	YWHAB
chr14:73893796-73904110	7	ZNF410
chr19:9614020-9618179	12	ZNF561
chr17:39908957-39909924	8	GSDMB
chr10:31831738-31852597	13	ARHGAP12
chr4:56011411-56011985	57	CEP135
chr19:1271328-1272051	556	CIRBP
chr7:66130126-66134374	11	CRCP
chr11:65266581-65268729	32	POLA2
chr12:27650009-27656730	21	PPFIBP1
chr12:56670274-56673065	106	PTGES3
chr19:49854969-49858114	6	PTOV1
chr3:50113382-50114251	79	RBM5
chr1:179900125-179908673	17	TOR1AIP1
chr8:23027132-23029721	7	TNFRSF10B
chr6:157110471-157148951	9	ARID1B
chr17:61743012-61793729	10	BRIP1
chr5:134781132-134785586	15	DDX46
chr8:108214606-108217460	10	EIF3E
chr6:136388392-136389517	27	MAP7
chr20:44918905-44921731	5	PABPC1L
chr15:90976675-90981981	10	PRC1
chr4:17501718-17504475	10	QDPR
chr18:70092109-70092804	5	RTTN
chr11:107803000-107811794	12	SLC35F2
chr8:102257631-102267007	7	UBR5
chr2:202882710-202883741	17	WDR12
chr9:85677435-85712566	65	AGTPBP1
chr11:78106806-78121174	11	ALG8
chr2:111847137-111850910	36	ANAPC1
chr15:32633932-32636408	20	ARHGAP11A
chr4:55489373-55510065	11	CLOCK
chr5:149505446-149513209	56	CSNK1A1
chr4:182877510-182915575	92	DCTD
chr4:24570653-24570847	5	DHX15
chr19:10162666-10166685	7	DNMT1

chr20:18593730-18596241	24	DTD1
chr15:74640465-74656068	8	EDC3
chr2:46346872-46361090	10	EPAS1
chr11:128768117-128782023	18	FLI1
chr10:14854124-14855943	11	HSPA14
chr13:30255446-30257867	8	KATNAL1
chr5:146168127-146171990	29	LARS
chr1:3781150-3781601	12	LRRC47
chr8:66601697-66602523	134	MYBL1
chr16:3476221-3479600	33	NAA60
chr9:97647491-97654944	14	NCBP1
chr17:16034764-16039654	18	NCOR1
chr12:102257214-102282298	47	nogene
chr3:136709391-136715592	11	nogene
chrX:71297938-71298468	8	NONO
chr3:183862752-183868060	30	PARL
chr17:67532801-67578257	8	PITPNC1
chr1:88760221-88786213	7	PKN2
chr5:127526617-127539143	64	PRRC1
chr1:151024610-151027327	37	PRUNE
chr9:122855183-122855878	13	RC3H2
chr1:150440922-150444377	5	RPRD2
chr3:9453738-9464672	45	SETD5
chr3:27379248-27391808	16	SLC4A7
chr2:102657563-102665350	11	SLC9A2
chr3:47675474-47686170	34	SMARCC1
chr7:17850346-17875790	23	SNX13
chr1:48355669-48403882	13	SPATA6
chr1:47287550-47301748	16	STIL
chr3:133608534-133612552	8	TOPBP1
chr6:43555835-43568737	11	XPO5
chr6:170546102-170549113	16	PSMB1
chr10:94511582-94522453	30	TBC1D12
chr2:96797011-96817502	7	CNNM4
chr1:222686929-222703217	68	AIDA
chr11:83273646-83278829	52	CCDC90B
chr3:52374187-52374885	6	nogene
chr10:75150513-75151106	104	SAMD8
chr15:56689040-56693215	29	ZNF280D
chr12:47679492-47690639	7	RPAP3
chr12:110030644-110036328	9	ANKRD13A
chr3:43598531-43600581	10	ANO10
chrX:23856301-23868688	7	APOO

chr19:12745376-12745759	7	ASNA1
chr19:32915336-32926984	14	CEP89
chr1:173838189-173845291	18	DARS2
chr15:66294987-66314117	6	DIS3L
chr8:131940498-131970643	50	EFR3A
chr4:3103823-3121432	12	HTT
chr5:126878184-126918227	8	3-Mar
chr14:64417887-64424931	29	MTHFD1
chr12:50004234-50016719	8	RACGAP1
chr2:199323802-199349173	6	SATB2
chr12:64080285-64115893	8	SRGAP1
chr9:79627373-79654075	57	TLE4
chr7:157216866-157231327	26	UBE3C
chr10:1103534-1105267	9	WDR37
chr11:117159933-117161261	49	PAFAH1B2
chr16:2056195-2058873	54	TSC2
chr8:144748682-144749080	8	nogene
chr3:104817472-104818695	8	nogene
chr16:70267658-70268379	8	AARS
chr11:108307896-108317521	8	ATM
chr11:108227594-108235834	151	ATM
chr1:235454155-235484515	13	B3GALNT2
chr7:16682779-16689906	11	BZW2
chr5:179841906-179844227	26	C5orf45
chr10:118691264-118695230	11	CACUL1
chr11:107390073-107392895	191	CWF19L2
chr7:80199224-80212869	9	GNAI1
chr16:74348866-74360891	6	LOC283922
chr13:25265818-25267941	29	MTMR6
chr12:76067370-76074239	94	NAP1L1
chr1:198232552-198297240	6	NEK7
chr18:42038780-42049605	12	PIK3C3
chr19:43652224-43655573	8	PLAUR
chr1:202422619-202449171	8	PPP1R12B
chr14:67823541-67835196	151	RAD51B
chr1:44411980-44412722	259	RNF220
chr3:47701277-47706530	7	SMARCC1
chr6:85543604-85558060	10	SNX14
chr1:230933778-230961303	6	TTC13
chr15:42988818-43025429	7	UBR1
chr5:178860127-178860974	15	ZNF354B
chr6:28271853-28272787	20	ZSCAN26
chr1:30974650-30981405	11	PUM1

chr1:241881915-241885507	19	EXO1
chr14:54479785-54483767	5	GMFB
chr13:30227411-30283791	673	KATNAL1
chr16:56470486-56470791	5	OGFOD1
chr11:66363137-66368671	5	SLC29A2
chr1:151638887-151668635	89	SNX27
chr19:2936523-2939407	30	ZNF77
chr17:4085669-4086655	85	ZZEF1
chr12:109222506-109223904	7	ACACB
chr5:180261683-180269409	18	MAPK9
chr18:12356693-12360051	62	AFG3L2
chr5:343892-376716	7	AHRR
chr10:27907662-27944962	21	ARMC4
chr7:18722951-18793452	11	HDAC9
chr19:18134578-18134982	16	MAST3
chr5:150539227-150540264	37	NDST1
chr3:50347575-50350022	12	NPRL2
chr12:45931505-45951727	10	SCAF11
chr1:10467189-10469338	68	DFFA
chrX:72488932-72495268	56	HDAC8
chr7:23360585-23361741	9	IGF2BP3
chr7:128591849-128615970	9	nogene
chr4:86772777-86775652	28	PTPN13
chr3:12496517-12505231	25	TSEN2
chr9:20907149-20929596	40	FOCAD
chr1:161851721-161912380	9	ATF6
chr10:12215739-12238485	179	CDC123
chr3:33072543-33077237	5	GLB1
chr7:36335045-36336297	10	KIAA0895
chr12:62549667-62553174	11	MON2
chr8:71973537-72076627	8	nogene
chr5:37291812-37299568	5	NUP155
chr10:6213622-6219693	23	PFKFB3
chr5:145764930-145776430	12	PRELID2
chr8:47933019-47936517	10	PRKDC
chr17:59910561-59926582	7	RPS6KB1
chr4:185361923-185362711	24	SNX25
chr7:23505745-23521840	9	TRA2A
chr1:162566011-162588833	11	UAP1
chr1:151178000-151185950	8	VPS72
chr14:54995602-55000992	11	WDHD1
chr5:133006758-133007889	10	ZCCHC10
chr10:79310923-79311184	9	ZMIZ1

chr10:26765217-26770345	60	ABI1
chr3:195365481-195392147	7	ACAP2
chr2:157760879-157774187	7	ACVR1
chr9:85585462-85596449	19	AGTPBP1
chr1:246860898-246862153	14	AHCTF1
chr2:9376907-9380808	13	ASAP2
chr1:155378280-155380116	8	ASH1L
chr17:30858160-30869646	9	ATAD5
chr3:182836034-182848557	24	ATP11B
chr12:89655678-89656107	20	ATP2B1
chr3:142465096-142468068	36	ATR
chr16:304303-347106	58	AXIN1
chr18:31638643-31658089	11	B4GALT6
chr2:28237201-28241393	9	BRE
chr7:16685904-16689906	6	BZW2
chr22:24035443-24043084	41	CABIN1
chr18:24143090-24143413	15	CABYR
chr12:67292621-67299095	13	CAND1
chr2:207724799-207751119	8	CCNYL1
chr12:7432657-7433694	19	CD163L1
chr22:17503081-17505016	13	CECR2
chr16:81011929-81014181	11	CENPN
chr17:66062962-66096632	9	CEP112
chr1:179990506-179992221	7	CEP350
chr1:150966755-150969091	5	CERS2
chr21:36387597-36399605	8	CHAF1B
chr3:33619602-33663515	15	CLASP2
chr16:10969160-10982991	69	CLEC16A
chr12:122360958-122364107	9	CLIP1
chr7:107362032-107372760	61	COG5
chr12:38670728-38702921	10	CPNE8
chr3:33120343-33134265	12	CRTAP
chr4:1231634-1241519	9	CTBP1
chr2:227893794-227907252	63	DAW1
chr22:38498084-38498573	9	DDX17
chr5:134816429-134819004	11	DDX46
chr1:197595207-197617759	39	DENND1B
chr12:50724774-50728678	5	DIP2B
chr10:68465666-68470163	48	DNA2
chr5:118928536-118946328	9	DTWD2
chr1:20840855-20865262	24	EIF4G3
chr3:55888388-55950560	6	ERC2
chr11:126234994-126241044	13	FAM118B

chr6:70426569-70452571	23	FAM135A
chr18:13681604-13687882	7	FAM210A
chr6:145804371-145806421	6	FBXO30
chr16:53933984-53937833	13	FTO
chr1:151088189-151107222	15	GABPB2
chr1:93894613-93904588	49	GCLM
chr12:109980951-109991760	8	GIT2
chr8:41609415-41612273	16	GPAT4
chr2:144208602-144235737	38	GTDC1
chr10:999002-1007128	7	GTPBP4
chr11:119126934-119127125	30	HINFP
chr1:20757165-20776750	11	HP1BP3
chr6:160009009-160010785	5	IGF2R
chr1:153752278-153757763	6	INTS3
chr13:97992891-98000645	16	IPO5
chr12:30665219-30669282	16	IPO8
chr15:90486029-90487582	8	IQGAP1
chr19:5016256-5047669	10	KDM4B
chr9:7103684-7128236	7	KDM4C
chr4:122272159-122289192	11	KIAA1109
chr12:122530021-122534784	8	KNTC1
chr5:177334283-177338605	9	LMAN2
chr15:70973248-70984257	10	LRRC49
chr8:55966714-55999549	5	LYN
chr3:47918718-47921878	17	MAP4
chr18:54159764-54166166	36	MBD2
chr18:21773623-21804014	8	MIB1
chr10:72407928-72508269	11	MICU1
chr17:42568469-42570183	8	MLX
chr6:44114076-44116629	63	MRPL14
chr2:15415545-15478289	27	NBAS
chrX:135034668-135038178	15	nogene
chr11:29282461-29285063	12	nogene
chr2:183182556-183188763	11	nogene
chr20:49053082-49053453	10	nogene
chr22:38355859-38356295	7	nogene
chr1:8865314-8866394	8	nogene
chr22:28514756-28528728	11	nogene
chr3:112153472-112154325	9	nogene
chr5:177191883-177204292	90	NSD1
chr5:37342548-37358151	12	NUP155
chr11:3731390-3731578	47	NUP98
chr20:62281789-62291793	15	OSBPL2

chr9:75127568-75140932	7	OSTF1
chr14:70968197-70978648	15	PCNX
chr1:233160282-233208689	58	PCNXL2
chr15:43763076-43765566	5	PDIA3
chr9:41025763-41027114	7	PGM5P2
chr18:62830034-62860601	12	PHLPP1
chr10:122412919-122417968	15	PLEKHA1
chr12:19283279-19287556	16	PLEKHA5
chr2:43712224-43741126	5	PLEKHH2
chr10:77980140-77981559	8	POLR3A
chr2:44201185-44209327	23	PPM1B
chr5:40764513-40771863	34	PRKAA1
chr8:140826978-140864399	15	PTK2
chr19:49844315-49844598	14	PTOV1-AS1
chr1:174250474-174305127	9	RABGAP1L
chr9:107300140-107325004	102	RAD23B
chr7:39686630-39690590	27	RALA
chr3:141509567-141529802	25	RASA2
chr7:5047969-5057779	15	RBAK
chr22:35765422-35778078	32	RBFOX2
chr1:8614560-8624380	31	RERE
chr2:99449335-99451501	7	REV1
chr19:33021570-33026632	13	RHPN2
chr8:86474819-86507112	7	RMDN1
chr18:32111753-32124845	213	RNF138
chr2:11249660-11287736	7	ROCK2
chr17:59935192-59937774	8	RPS6KB1
chr7:29936555-29955360	33	SCRN1
chr4:109481676-109494856	18	SEC24B
chr2:241342991-241346249	17	2-Sep
chr16:70532478-70539207	61	SF3B3
chr16:70556178-70557029	13	SF3B3
chr15:75400729-75401970	13	SIN3A
chr19:16863796-16865382	14	SIN3B
chr19:10637643-10638315	17	SLC44A2
chr18:21622724-21623939	64	SNRPD1
chr16:69245601-69284244	8	SNTB2
chr11:130903254-130906721	12	SNX19
chr14:50174453-50204409	5	SOS2
chr14:35000935-35008831	6	SRP54
chr19:55341793-55346687	11	SUV420H2
chr17:35820023-35822833	31	TAF15
chr8:119817983-119819506	9	TAF2

chr9:79704782-79709699	37	TLE4
chr9:71740062-71740232	10	TMEM2
chr7:130185191-130192823	21	TMEM209
chr1:25340473-25351693	74	TMEM50A
chr7:128982247-128993914	13	TNPO3
chr17:78064721-78067923	12	TNRC6C
chr21:44032090-44037927	8	TRAPPC10
chr17:59061031-59070947	7	TRIM37
chr5:139614585-139623461	80	UBE2D2
chr8:102311298-102361626	6	UBR5
chr1:77739264-77741748	11	USP33
chr3:184936245-184983094	32	VPS8
chr16:22131204-22131729	6	VWA3A
chr6:169572418-169602321	6	WDR27
chr19:12672846-12673316	104	WDR83
chr22:40868998-40882177	9	XPNPEP3
chr16:28132333-28135324	10	XPO6
chr12:64409961-64420521	7	XPOT
chr20:63202286-63203807	40	YTHDF1
chr16:31722625-31723353	424	ZNF720
chr4:145902165-145903215	16	ZNF827
chr12:133047954-133048848	27	ZNF84
chr10:124940373-124943307	27	ZRANB1
chr1:155264467-155270977	9	CLK2
chr17:4547957-4549442	16	MYBBP1A
chr1:244845246-244848856	32	nogene
chr5:134379553-134380010	8	nogene
chrX:121736190-121761171	12	nogene
chr13:110651033-110683134	12	CARS2
chr2:63433744-63571626	35	WDPCP
chr9:129638684-129639201	6	ASB6
chr8:140717597-140743330	6	PTK2
chr1:1815755-1879902	8	GNB1
chr3:195320700-195336976	18	ACAP2
chr16:58585337-58588906	19	CNOT1
chr22:46312140-46326654	12	GTSE1
chr5:132726127-132734478	7	KIF3A
chr3:196805351-196807914	32	PAK2
chr1:77206639-77212347	35	PIGK
chr1:1692448-1739704	8	SLC35E2B
chr12:66145840-66153448	15	TMBIM4
chr1:70274719-70315566	6	ANKRD13C
chr7:16665436-16697061	39	BZW2

chr6:99558496-99562948	15	CCNC
chr22:17141058-17149745	10	CECR5
chr19:4408902-4410523	30	CHAF1A
chr16:58528474-58534395	6	CNOT1
chr1:92677157-92704744	41	EVI5
chr7:134137022-134174636	11	LRGUK
chr14:71400793-71418196	31	nogene
chr5:93584329-93584824	9	nogene
chrX:65740042-65740250	14	nogene
chr2:202292776-202295837	8	NOP58
chr1:233198938-233252788	64	PCNXL2
chr2:158603069-158642699	13	PKP4
chr2:112547011-112547567	8	POLR1B
chr1:2395783-2402342	6	RER1
chr9:136898712-136909994	8	TRAF2
chr3:141368620-141404031	153	ZBTB38
chr16:84990303-84996007	19	ZDHHC7
chr5:65273339-65291470	9	ADAMTS6
chr22:17900841-17906886	12	MICAL3
chr17:57114458-57116261	6	AKAP1
chr7:111989014-112004131	7	DOCK4
chr11:68347846-68363943	10	LRP5
chr1:198232552-198253180	9	NEK7
chr11:47797778-47801930	19	NUP160
chr12:108818248-108823361	8	SSH1
chr17:35820023-35820437	27	TAF15
chr6:37929958-37930576	14	ZFAND3
chr20:47262287-47347926	9	ZMYND8
chr20:47287228-47294779	7	ZMYND8
chr9:86033281-86052591	10	GOLM1
chrX:63674037-63724711	8	ARHGEF9
chr10:5648969-5653050	6	ASB13
chr12:122785718-122792121	28	CCDC62
chr7:148828739-148847305	7	EZH2
chr1:37568850-37576556	5	GNL2
chr14:92809299-92816421	24	GOLGA5
chr19:17058548-17062779	8	HAUS8
chr7:86925527-86926916	5	KIAA1324L
chr1:10371140-10375373	7	KIF1B
chr7:130945719-131053465	9	LINC-PINT
chr4:128292933-128303035	7	nogene
chr6:79025518-79026164	10	PHIP
chr22:21939531-21941587	14	PPM1F

chr6:30608778-30609955	13	PPP1R10
chr16:471294-489000	13	RAB11FIP3
chr19:11335446-11335783	65	RAB3D
chr3:141553856-141559995	141	RASA2
chrX:48575167-48577109	39	RBM3
chr2:218580560-218587695	7	RQCD1
chr22:23816769-23825415	7	SMARCB1
chr5:6651841-6662966	37	SRD5A1
chrX:123610917-123614189	7	THOC2
chr15:77046775-77056255	12	TSPAN3
chr15:90999676-90999975	19	VPS33B
chr12:118042840-118043377	17	WSB2
chr17:36952885-36953907	125	AATF
chr1:75745805-75750546	46	ACADM
chr1:147650142-147659775	6	ACP6
chr7:149891190-150295347	9	ACTR3C
chr5:65470777-65473952	10	ADAMTS6
chr1:35901473-35902337	23	AGO1
chrX:130129501-130131799	5	AIFM1
chr15:85655416-85655787	8	AKAP13
chr6:131199460-131219808	20	AKAP7
chr11:111836164-111840809	15	ALG9
chr8:107284681-107303367	6	ANGPT1
chr1:113897839-113901885	7	AP4B1
chr11:108227594-108247127	16	ATM
chr18:79096475-79126375	150	ATP9B
chr6:16585779-16753347	13	ATXN1
chr8:102833055-102843747	16	AZIN1
chr17:60902619-60924500	23	BCAS3
chr2:32439507-32453954	17	BIRC6
chr2:70160624-70181997	13	C2orf42
chr7:82060427-82084900	23	CACNA2D1
chr13:110663450-110705571	14	CARS2
chr9:65690966-65708958	12	CBWD5
chr7:26202970-26208555	40	CBX3
chr10:32543299-32635158	11	CCDC7
chr10:32472480-32544301	10	CCDC7
chr10:35483403-35530243	23	CCNY
chr1:156333546-156335888	30	CCT3
chr3:101731839-101732654	23	CEP97
chr16:69160355-69167185	6	CIRH1A
chr3:33606590-33644903	53	CLASP2
chr1:9743883-9744643	11	CLSTN1

chr19:54142928-54149758	6	CNOT3
chr7:43639448-43648652	6	COA1
chr1:230663140-230664587	27	COG2
chrX:108577951-108578383	5	COL4A5
chr1:160316218-160332557	13	COPA
chr20:49066191-49066510	55	CSE1L
chr15:64203081-64216713	98	CSNK1G1
chr20:488677-489881	6	CSNK2A1
chr1:40995916-40997526	6	CTPS1
chr11:70405264-70422995	9	CTTN
chr11:107308282-107353736	26	CWF19L2
chr4:51863436-51899378	45	DCUN1D4
chr10:21882281-21929141	17	DNAJC1
chr11:103399662-103468705	8	DYNC2H1
chr9:137754170-137762820	51	EHMT1
chr5:66012048-66014725	50	ERBB2IP
chr3:186079816-186081175	202	ETV5
chr5:74796029-74805284	27	FAM169A
chr5:94050730-94057203	7	FAM172A
chr22:45574510-45577108	21	FBLN1
chr9:20758089-20778768	7	FOCAD
chr7:4740837-4742849	14	FOXK1
chr6:108663454-108664889	766	FOXO3
chr6:29623304-29624024	13	GABBR1
chr16:58716013-58716813	17	GOT2
chr5:21491320-21497196	19	GUSBP1
chr8:29045360-29049048	9	HMBOX1
chr2:38585643-38591648	74	HNRNPLL
chr3:122759223-122777906	6	HSPBAP1
chr16:71894720-71921453	14	IST1
chr20:34424479-34462221	6	ITCH
chr19:17973807-17989843	6	KCNN1
chr6:18159882-18161454	9	KDM1B
chr1:155921374-155921918	135	KIAA0907
chr18:6213148-6264038	15	L3MBTL4
chr7:130967388-131052555	8	LINC-PINT
chr7:156724111-156756465	39	LMBR1
chr15:30745177-30750556	7	LOC100288637
chr16:199061-208188	43	LUC7L
chr18:35070194-35102159	24	MAPRE2
chr14:103405075-103448967	8	MARK3
chr19:12851933-12852395	68	MAST1
chr10:101792838-101810314	19	MGEA5

chr7:7594979-7596461	11	MIOS
chr9:126381063-126395697	46	MVB12B
chr2:191329934-191350225	6	MYO1B
chr19:17162349-17163122	12	MYO9B
chr12:76049199-76074239	6	NAP1L1
chr20:34768463-34776448	9	NCOA6
chr2:206149019-206152510	33	NDUFS1
chr6:130287819-130288395	12	nogene
chr1:1786934-1825482	11	nogene
chrX:91414903-91418867	39	nogene
chr5:134799645-134800042	12	nogene
chr15:92910606-92911506	7	nogene
chr12:108813137-108813490	15	nogene
chr4:48833400-48857365	12	OCIAD1
chr1:233236255-233236980	8	PCNXL2
chrX:24494741-24505298	18	PDK3
chr11:601573-603436	6	PHRF1
chr2:131120933-131126786	5	PLEKHB2
chr10:131934457-131947791	19	PPP2R2D
chr3:170270420-170275287	6	PRKCI
chr19:49599271-49601918	17	PRR12
chr9:111574733-111589382	10	PTGR1
chr7:105481051-105508544	6	PUS7
chr3:111144898-111147484	37	PVRL3
chr15:40728710-40731344	6	RAD51
chr20:34072065-34076040	10	RALY
chr6:138907494-138914761	6	REPS1
chr1:8465924-8614686	7	RERE
chr9:5689958-5720750	17	RIC1
chr7:4983670-4989177	284	RNF216P1
chr9:36375933-36376127	6	RNF38
chr6:42880860-42884750	23	RPL7L1
chr6:154773988-154792976	12	SCAF8
chr2:241337381-241348191	7	2-Sep
chr11:17926737-18008076	7	SERGEF
chr10:110964124-110967278	8	SHOC2
chr21:36709167-36731151	15	SIM2
chr4:42022837-42039053	26	SLC30A9
chr9:96343903-96352109	9	SLC35D2
chr19:11007901-11010531	23	SMARCA4
chr7:127694827-127707647	24	SND1
chr4:185247293-185259064	6	SNX25
chr1:179302676-179337896	24	SOAT1

chr1:48395266-48399644	10	SPATA6
chr12:130795994-130808704	23	STX2
chr11:59788872-59795482	11	STX3
chr17:55072899-55081183	19	STXBP4
chr10:102592581-102597293	7	SUFU
chr19:19019108-19031082	7	SUGP2
chr7:27785162-27785489	67	TAX1BP1
chr4:106230362-106236808	7	TBCK
chr2:74073548-74080591	41	TET3
chr2:70988534-70993775	21	TEX261
chr13:113534044-113539507	7	TMCO3
chr12:121743628-121750439	7	TMEM120B
chr9:109033306-109038117	8	TMEM245
chr9:105705596-105748190	25	TMEM38B
chr8:132722039-132757294	7	TMEM71
chr18:68697225-68713900	8	TMX3
chr19:55140882-55141920	36	TNNT1
chr17:18285141-18285520	31	TOP3A
chr18:31873429-31901025	11	TRAPPC8
chr22:46337778-46346544	17	TRMU
chr18:24118124-24125550	8	TTC39C
chr2:178535731-178600667	14	TTN-AS1
chr17:59884660-59891041	7	TUBD1
chr1:162576776-162590511	18	UAP1
chr6:42650283-42652645	8	UBR2
chr17:60294682-60301704	9	USP32
chr6:99464603-99468618	78	USP45
chr4:119245336-119273708	12	USP53
chr3:51419733-51420997	6	VPRBP
chr16:74892483-74904100	9	WDR59
chr7:5199575-5203481	8	WIPI2
chr12:57946994-57953689	39	XRCC6BP1
chr11:57692610-57696043	10	ZDHHC5
chr1:52233170-52239595	8	ZFYVE9
chr3:125313307-125313656	52	ZNF148
chr7:149153944-149154340	147	ZNF398
chr6:57124733-57142095	101	ZNF451
chr19:53404473-53486758	9	ZNF813
chr17:19958013-19958571	29	AKAP10
chr17:19939712-19947505	12	AKAP10
chr5:126582850-126584012	5	ALDH7A1
chr2:127869453-127874272	6	AMMECR1L
chr16:71745143-71750332	12	AP1G1

chr1:10440346-10442441	62	APITD1-CORT
chr4:36187450-36229645	11	ARAP2
chr11:118581245-118593698	5	ARCN1
chr5:143054438-143134105	6	ARHGAP26
chr10:121870005-121924329	39	ATE1
chr17:18021122-18026416	39	ATPAF2
chr7:32543140-32552295	16	AVL9
chr2:202513718-202519052	16	BMPR2
chr7:140776911-140834703	11	BRAF
chr1:169798856-169804240	5	C1orf112
chr5:37120215-37121784	31	C5orf42
chr12:67307396-67310316	10	CAND1
chr11:34086063-34086413	48	CAPRIN1
chr1:93226332-93232593	11	CCDC18
chr15:59107321-59117368	10	CCNB2
chrX:2615815-2626642	5	CD99P1
chr22:46436173-46439411	18	CELSR1
chr4:184710071-184717196	7	CENPU
chr1:243185778-243225321	10	CEP170
chr18:12999420-13013025	14	CEP192
chr3:113399905-113407041	15	CFAP44
chr2:201129728-201136107	16	CFLAR
chr7:132975168-133024627	48	CHCHD3
chr22:28695709-28699937	5	CHEK2
chr12:119822819-119834228	14	CIT
chr12:122332986-122334705	6	CLIP1
chr1:155264220-155270977	5	CLK2
chr12:70319297-70346324	6	CNOT2
chr5:116291516-116303683	36	COMMD10
chr4:83056925-83063246	11	COPS4
chr14:92142163-92143294	6	CPSF2
chr2:36378356-36464655	8	CRIM1
chr5:138886211-138917898	6	CTNNA1
chr4:150193137-150203889	12	DCLK2
chr2:233363076-233390483	76	DGKD
chr19:47355038-47355350	32	DHX34
chr12:50671185-50683248	9	DIP2B
chr12:50671185-50674629	8	DIP2B
chr3:197051576-197066754	7	DLG1
chr10:77853353-77854370	73	DLG5
chr15:51507133-51517167	8	DMXL2
chr19:10163325-10173905	13	DNMT1
chr12:63644402-63661481	18	DPY19L2

chr12:101908185-101920201	6	DRAM1
chr7:107564587-107576592	11	DUS4L
chr11:103311877-103323990	72	DYNC2H1
chr1:21221746-21228041	17	ECE1
chr1:231364326-231374099	12	EGLN1
chr10:119069445-119071085	8	EIF3A
chr17:39709812-39710481	6	ERBB2
chr5:65975986-65994864	5	ERBB2IP
chr1:27978373-28004419	8	EYA3
chr5:154033790-154034946	7	FAM114A2
chr7:22960255-22978448	11	FAM126A
chr2:222613810-222624479	17	FARSB
chr5:171876284-171878129	5	FBXW11
chr4:53414614-53428183	537	FIP1L1
chr1:154987465-154990528	5	FLAD1
chr9:20715321-20770238	89	FOCAD
chr5:151794158-151799313	31	G3BP1
chr9:83799184-83806560	7	GKAP1
chr12:132795851-132822311	11	GOLGA3
chr14:76166662-76178042	86	GPATCH2L
chr12:12908242-12909171	25	GPRC5A
chr15:28292887-28321464	9	HERC2
chr10:68014025-68067217	7	HERC4
chr21:36896859-36897131	19	HLCS
chr9:112403994-112409021	23	HSDL2
chr4:3086938-3103883	7	HTT
chr11:62128150-62130590	10	INCENP
chr8:94842346-94856978	7	INTS8
chr12:26621122-26632059	23	ITPR2
chr4:128831732-128862225	7	JADE1
chr17:46066536-46082542	36	KANSL1
chr3:20071363-20072459	15	KAT2B
chr1:23044426-23059167	18	KDM1A
chr1:32037834-32038619	7	KHDRBS1
chr2:61103852-61106662	49	KIAA1841
chr7:152309965-152330739	15	KMT2C
chr12:122557383-122557689	8	KNTC1
chr22:33761370-33856802	84	LARGE
chr7:22571774-22575762	11	LOC100506178
chr9:110972072-110973558	2714	LPAR1
chr11:68357649-68365702	61	LRP5
chr12:12165078-12187119	10	LRP6
chr14:74514605-74516941	9	LTBP2

chr6:90536336-90571807	7	MAP3K7
chr12:111867571-111871180	12	MAPKAPK5
chr10:119847612-119859884	14	MCMBP
chr17:76738281-76742048	6	MFSD11
chr3:127694974-127695190	45	MGLL
chrX:154789953-154792285	148	MPP1
chr2:55246336-55249534	10	MTIF2
chr6:73480674-73482244	116	MTO1
chr4:139376364-139386230	9	NAA15
chr18:10539761-10540399	8	NAPG
chr8:89970363-89978323	41	NBN
chr14:75109684-75114313	7	NEK9
chr19:1602756-1603327	13	nogene
chr14:34969169-34972064	64	nogene
chr5:134942480-134960698	17	nogene
chr2:61197271-61197791	10	nogene
chr16:81516411-81517984	6	nogene
chr2:177246891-177247408	11	nogene
chr11:69371402-69373513	13	nogene
chr16:67939885-67940831	13	nogene
chr22:17928400-17928882	7	nogene
chr1:58713055-58723169	5	nogene
chr7:157391091-157391847	5	nogene
chr8:53984804-53985133	19	nogene
chr9:91883257-91886770	72	nogene
chr6:108567171-108569742	12	nogene
chr9:22767174-22768403	6	nogene
chr15:63567616-63568079	14	nogene
chr8:125102050-125182256	20	NSMCE2
chr5:37301436-37303414	59	NUP155
chr4:56451871-56453761	23	PAICS
chr6:10702368-10704844	10	PAK1IP1
chr6:10702368-10702639	99	PAK1IP1
chr14:24096891-24102890	6	PCK2
chr3:123106748-123124343	7	PDIA5
chr16:47463898-47499894	11	PHKB
chr16:47497398-47596531	54	PHKB
chr16:71655239-71656681	7	PHLPP2
chr12:19253939-19257537	6	PLEKHA5
chr11:68567013-68583129	19	PPP6R3
chr11:68558565-68576043	8	PPP6R3
chr17:66495571-66496283	17	PRKCA
chr1:2169517-2175313	24	PRKCZ

chr2:53892807-53893799	39	PSME4
chr2:86121478-86125880	10	PTCD3
chr8:140818276-140879637	233	PTK2
chr8:140739017-140752316	616	PTK2
chr18:12814202-12859254	30	PTPN2
chr1:30992389-31007102	267	PUM1
chr10:118009081-118040565	14	RAB11FIP2
chr18:23001585-23006432	28	RBBP8
chr14:22906194-22911403	388	RBM23
chr6:111472012-111474479	9	REV3L
chr4:39326562-39327756	80	RFC1
chr2:110562611-110569724	16	RGPD5
chr15:59091058-59092640	5	RNF111
chr3:196483769-196487578	11	RNF168
chr11:77683709-77691238	16	RSF1
chr3:134207471-134211607	30	RYK
chr2:128014802-128017915	7	SAP130
chr4:109530288-109539667	6	SEC24B
chr3:47046486-47062346	5	SETD2
chr10:70844472-70851210	21	SGPL1
chr3:72841044-72844453	5	SHQ1
chr5:128147621-128152805	8	SLC12A2
chr2:113742691-113746339	9	SLC35F5
chr12:104909654-104928694	45	SLC41A2
chr7:17814833-17845694	11	SNX13
chr1:109340723-109345881	56	SORT1
chr12:49484589-49500205	7	SPATS2
chr15:44606024-44610985	7	SPG11
chr4:26919503-26957726	8	STIM2
chr2:196141248-196146055	10	STK17B
chr13:98474820-98475358	5	STK24
chr11:62824165-62827631	18	STX5
chr14:24974664-24974850	11	STXBP6
chr20:62857394-62860308	8	TCFL5
chr13:60509762-60510755	5	TDRD3
chr8:55802456-55805036	8	TGS1
chr10:49952125-49973142	17	TIMM23B
chr2:171082780-171092227	5	TLK1
chr11:61366044-61366216	5	TMEM138
chr2:102783893-102798000	9	TMEM182
chr18:23352195-23373763	6	TMEM241
chr15:43469857-43481022	12	TP53BP1
chr5:14316512-14330900	15	TRIO

chr2:229858771-229860531	57	TRIP12
chr7:98930632-98931665	9	TRRAP
chr15:42829937-42872758	20	TTBK2
chr18:24064139-24069271	445	TTC39C
chr8:102342472-102346395	6	UBR5
chr1:62441487-62443319	10	USP1
chr17:78827244-78838750	42	USP36
chr7:6139091-6145656	21	USP42
chr17:31884423-31892794	19	UTP6
chr10:28589735-28596041	15	WAC
chr2:31339488-31383847	7	XDH
chr6:43570501-43573601	25	XPO5
chr3:142355400-142359931	6	XRN1
chr12:42199135-42210680	9	YAF2
chr3:141396258-141404031	47	ZBTB38
chr18:62544801-62556297	9	ZCCHC2
chr9:37126311-37190651	9	ZCCHC7
chr10:45639750-45663842	8	ZFAND4
chr17:32360597-32362984	17	ZNF207
chr6:87215902-87218731	1492	ZNF292
chr12:119728501-119730630	7	CIT
chr8:141136508-141138137	20	DENND3
chr1:225828724-225829534	6	EPHX1
chr8:42966472-42974194	11	HOOK3
chr13:41318805-41336756	17	NAA16
chr12:123110384-123141399	13	PITPNM2
chr8:99096311-99103120	14	VPS13B
chr18:76830675-76851939	7	ZNF236
chr1:6820180-6853189	7	CAMTA1
chr5:179708238-179710065	103	CANX
chr4:185188950-185191212	10	CFAP97
chr12:119757370-119767182	6	CIT
chr3:33535232-33551395	7	CLASP2
chr14:99850123-99865646	31	EML1
chr7:158735502-158739121	58	ESYT2
chr1:16294782-16305919	13	FBXO42
chrX:131695843-131794466	6	FIRRE
chr10:130159994-130166980	17	GLRX3
chr13:30240459-30280223	15	KATNAL1
chr5:62347129-62350120	6	KIF2A
chr20:25835983-25848948	7	LOC101926935
chr3:197814907-197832317	6	LRCH3
chr2:43905691-43925961	46	LRPPRC

chr7:1936686-1957719	26	MAD1L1
chrX:57087613-57095538	18	nogene
chrX:27158904-27186657	30	nogene
chr18:9593767-9595153	28	PPP4R1
chr11:68567013-68596218	36	PPP6R3
chr11:64766899-64776626	6	SF1
chr7:17845594-17850964	223	SNX13
chr14:30955619-30956242	36	STRN3
chr1:108793581-108800305	30	STXBP3
chr7:7877280-7892554	5	UMAD1
chr21:15864267-15870147	8	USP25
chr2:61265396-61266167	6	USP34
chr4:147875020-147881932	16	ARHGAP10
chr12:27380246-27385560	13	ARNTL2
chr2:175097443-175121540	13	ATF2
chr9:97647491-97648223	20	NCBP1
chr7:5641153-5641376	28	RNF216
chr1:70228421-70239520	31	SRSF11
chr10:31852516-31854206	21	ARHGAP12
chr12:45836588-45839496	46	ARID2
chr5:179709872-179720560	204	CANX
chr20:39023689-39030775	5	DHX35
chr3:172761609-172783909	7	ECT2
chr6:15374116-15468718	5	JARID2
chr10:132337564-132344650	8	LRRC27
chr19:8539731-8540060	8	nogene
chr13:24469023-24486305	9	PARP4
chr2:65331986-65344896	25	SPRED2
chr8:119756005-119762608	14	TAF2
chr2:169986644-170029448	6	UBR3
chr6:38577599-38613033	14	BTBD9
chr14:20313467-20326793	8	CCNB1IP1
chr1:161851721-161863312	10	ATF6
chr11:129942425-129961082	12	PRDM10
chr19:58294097-58295581	20	ZNF8
chr17:40391505-40392737	34	TOP2A
chr19:43203885-43266695	14	PSG4
chr1:243545509-243552943	7	AKT3
chr2:38318534-38319019	11	ATL2
chr17:67928354-67929487	24	BPTF
chr18:23515855-23519174	35	C18orf8
chr12:31495809-31500670	7	DENND5B
chr10:99894945-99909146	8	DNMBP

chr8:55941854-55947723	45	LYN
chr15:99598429-99645764	5	MEF2A
chr8:128009589-128020719	6	nogene
chr3:196213105-196213911	5	nogene
chr5:171240927-171242820	103	RANBP17
chr3:159886206-159888943	10	SCHIP1
chr16:70567410-70569141	14	SF3B3
chr15:44592330-44598836	5	SPG11
chr12:63983946-64017012	17	SRGAP1
chr10:74070669-74072852	65	VCL
chr16:46668929-46677398	26	VPS35
chr3:119905754-120093593	18	GSK3B
chr16:70258979-70262524	102	AARS
chr2:65246523-65261392	6	ACTR2
chr9:85575314-85633374	7	AGTPBP1
chr16:56385918-56389375	524	AMFR
chr12:110381751-110396452	36	ANAPC7
chr14:34783761-34792921	30	BAZ1A
chr12:111658735-111660675	35	BRAP
chr12:433173-433472	6	CCDC77
chr2:171448680-171473975	35	DCAF17
chr3:50778674-50934077	16	DOCK3
chr7:2366316-2369682	47	EIF3B
chr17:80136003-80138280	28	EIF4A3
chr19:7973724-7991831	8	ELAVL1
chr6:53269086-53295707	32	ELOVL5
chr10:99679260-99679875	10	ENTPD7
chr1:22781420-22785076	34	EPHB2
chr17:76097795-76101863	10	EXOC7
chr6:70426569-70428419	15	FAM135A
chr12:32607956-32611283	65	FGD4
chr15:28167686-28169655	9	HERC2
chr11:9420410-9425262	97	IPO7
chr17:50075598-50076681	21	ITGA3
chr13:48256176-48258236	6	ITM2B
chr2:8798201-8803127	8	KIDINS220
chr1:183114530-183115637	36	LAMC1
chr12:50427761-50429090	149	LARP4
chr19:11106564-11107514	6	LDLR
chr4:103343091-103437046	6	LOC101929448
chr3:185428496-185437630	10	MAP3K13
chr6:136669282-136698682	60	MAP3K5
chr19:17200292-17200829	10	MYO9B

chr12:76067370-76068994	53	NAP1L1
chr14:75117194-75124223	7	NEK9
chr7:66479008-66488038	5	nogene
chr3:28251711-28298350	6	nogene
chr12:73329105-73363397	10	nogene
chr11:78513675-78514252	9	nogene
chr1:50598942-50599194	10	nogene
chr20:3907925-3910830	41	PANK2
chr2:10787280-10790833	6	PDIA6
chr4:39913610-39922748	26	PDS5A
chr5:115230975-115241606	23	PGGT1B
chr1:28407409-28408135	23	PHACTR4
chr7:11040671-11062085	19	PHF14
chr1:145853503-145854880	11	PIAS3
chr6:149540941-149541586	8	PPIL4
chr4:120798266-120821345	20	PRDM5
chr5:145703974-145826200	10	PRELID2
chr1:43619287-43620992	13	PTPRF
chrX:16852045-16853842	20	RBBP7
chr6:154787860-154795139	51	SCAF8
chr1:151658234-151658427	23	SNX27
chrX:124021366-124031125	8	STAG2
chr15:75859877-75873568	189	UBE2Q2
chr7:93252652-93276305	9	VPS50
chr16:74938149-74942826	10	WDR59
chr7:100023418-100024307	278	ZKSCAN1
chr19:52353681-52354379	20	ZNF610
chr1:64582155-64602912	8	CACHD1
chr4:113661712-113679496	6	CAMK2D
chr5:110742046-110748263	5	SLC25A46
chr16:69284047-69284244	70	SNTB2
chr3:142736378-142781029	5	TRPC1
chr1:155709772-155721618	16	DAP3
chr18:12358669-12360051	55	AFG3L2
chr4:80008520-80036032	13	ANTXR2
chr12:28225794-28391411	11	CCDC91
chr12:28255580-28259442	114	CCDC91
chr5:95906522-95913934	17	ELL2
chr17:41379569-41395370	18	KRT34
chr16:72555264-72617553	9	LINC01572
chr1:235793502-235801097	12	LYST
chr17:62029540-62035608	60	MED13
chr21:36362182-36364259	20	MORC3

chr4:169463242-169508331	5	NEK1
chr1:88859132-88859592	11	nogene
chr9:78420279-78423212	50	nogene
chrX:131344059-131364926	37	nogene
chr5:25180728-25193209	13	nogene
chr8:73955910-73964625	7	TCEB1
chr1:21715388-21721764	11	USP48
chr7:93291702-93297243	78	VPS50
chr14:23081747-23090633	35	ACIN1
chr3:11355420-11426926	67	ATG7
chr6:80127546-80203212	37	BCKDHB
chr16:69139824-69154457	6	CIRH1A
chr4:38908591-38915073	6	FAM114A1
chr3:180947864-180951468	7	FXR1
chr22:37623326-37623633	13	GGA1
chr14:105155561-105157763	6	JAG2
chr6:52272300-52273916	9	MCM3
chr18:21791334-21804014	9	MIB1
chr22:29957035-29979052	24	MTMR3
chr11:74222311-74239256	18	PPME1
chr12:110730528-110731901	14	PPP1CC
chr19:34458768-34464131	6	UBA2
chr6:89329782-89343756	16	UBE2J1
chr1:93642303-93674941	483	BCAR3
chr9:15622737-15623413	10	CCDC171
chr1:156320838-156334918	53	CCT3
chr5:138191473-138201448	6	CDC23
chr10:68957530-68960249	6	DDX21
chr2:229585857-229591888	33	DNER
chr3:150575648-150581746	56	EIF2A
chr11:6222441-6224707	12	FAM160A2
chr2:85013935-85028056	12	KCMF1
chr14:55653016-55658614	27	KTN1
chr16:4657245-4673657	8	MGRN1
chr2:15287072-15309247	20	NBAS
chr3:131080422-131133956	7	NEK11
chr3:42617569-42621516	7	NKTR
chr14:76229807-76236003	8	nogene
chr11:75404059-75404835	6	nogene
chr12:122403786-122404352	14	nogene
chr6:17647806-17665385	7	NUP153
chr16:71690518-71714801	78	PHLPP2
chr8:78558342-78572939	23	PKIA

chr1:145839889-145842477	95	POLR3C
chr9:131432616-131439061	19	PRRC2B
chr14:90943077-90978524	11	RPS6KA5
chr12:48083590-48098124	81	SENP1
chr10:67887416-67889123	14	SIRT1
chr6:99531362-99531790	9	TSTD3
chr3:120000551-120002239	14	GSK3B
chr22:38267726-38268122	6	nogene
chr3:39063049-39068859	7	WDR48
chr2:97142639-97154741	61	ANKRD36
chr15:83030135-83041925	5	BTBD1
chr10:73146275-73163950	18	ECD
chr12:1027747-1028572	896	ERC1
chr5:176486354-176494275	6	FAF2
chr16:53810139-53844298	8	FTO
chr1:45727630-45729769	16	IPP
chr1:113585386-113590658	7	MAGI3
chr20:5958527-5986131	5	MCM8
chr12:5270204-5276889	51	nogene
chr1:53302011-53302440	5	nogene
chr2:158631752-158640759	15	PKP4
chr22:42599697-42603160	6	POLDIP3
chr1:100011364-100017815	11	SLC35A3
chr7:17868406-17897446	357	SNX13
chr6:121170404-121256285	10	TBC1D32
chr2:84839148-84853410	9	TRABD2A
chr15:50570103-50575789	5	TRPM7
chr16:2070455-2074389	6	TSC2
chr3:184957373-184971752	39	VPS8
chr21:39209994-39215362	9	BRWD1
chr17:81099927-81108509	6	BAIAP2
chr6:592468-599225	126	EXOC2
chr10:68393137-68404844	11	RUFY2
chr11:108127885-108134316	6	ACAT1
chr2:9509978-9536828	8	ADAM17
chr5:143014079-143057747	6	ARHGAP26
chr6:34646600-34654779	143	C6orf106
chr5:10260911-10261518	14	CCT5
chr10:14945104-14945565	7	DCLRE1C
chr16:23532656-23552304	6	EARS2
chr3:96936013-96987993	8	EPHA6
chr7:50669721-50710990	16	GRB10
chr2:177219036-177222133	12	HNRNPA3

chr6:116692054-116705160	7	KPNA5
chr3:113721794-113724095	8	NAA50
chr1:155732479-155732757	47	nogene
chrX:14244206-14245044	7	nogene
chr3:38183002-38216151	12	OXSR1
chr12:79613574-79621207	17	PAWR
chr21:46334396-46334768	24	PCNT
chr14:71009633-71036157	12	PCNX
chr1:233160282-233227371	7	PCNXL2
chr3:127660377-127662311	13	PODXL2
chr18:9570156-9595102	11	PPP4R1
chr7:579255-607452	7	PRKAR1B
chr3:196471978-196475312	17	RNF168
chr10:68383797-68386128	7	RUFY2
chr11:62881018-62882066	7	SLC3A2
chr10:69483690-69498396	53	TSPAN15
chr4:102799406-102809855	14	UBE2D3
chr9:83679774-83686155	273	UBQLN1
chr6:41803805-41916939	7	USP49
chr17:61776400-61781005	10	BRIP1
chr5:157294782-157296774	18	CYFIP2
chr6:56735227-56900621	7	DST
chr9:111386376-111391824	1842	KIAA0368
chr14:92116101-92117642	12	NDUFB1
chr22:28514756-28550151	38	nogene
chrX:54636234-54636427	189	nogene
chr20:36793248-36794599	8	SOGA1
chr9:36594627-36651877	6	MELK
chr8:102264473-102270183	5	UBR5
chr2:202555251-202556531	11	BMPR2
chr17:41997114-42003557	9	DNAJC7
chr4:105691975-105693486	67	INTS12
chr22:38687306-38688316	9	nogene
chr3:17622647-17625224	20	nogene
chr6:85618817-85624112	64	SYNCRIP
chr8:124503839-124516030	10	TATDN1
chr16:71749893-71764726	6	AP1G1
chr5:180569109-180571432	59	CNOT6
chr1:176116623-176136547	59	RFWD2
chr18:12366964-12371691	183	AFG3L2
chr21:25975069-25982477	94	APP
chr12:22517985-22527892	11	C2CD5
chr12:122797306-122798200	10	CCDC62

chr1:160305432-160314125	9	COPA
chr7:66573414-66576932	9	GS1-124K5.11
chr6:57052685-57055797	28	KIAA1586
chr3:183037217-183041750	5	MCCC1
chr4:102533847-102567135	8	NFKB1
chr4:103536345-103593429	16	nogene
chr1:229495494-229498306	21	NUP133
chr17:16325799-16326905	11	PIGL
chr3:125451304-125460860	8	SNX4
chr17:82797756-82814934	7	TBCD
chr6:126008391-126013101	10	TRMT11
chr2:178535731-178537691	27	TTN-AS1
chr12:133041277-133048848	18	ZNF84
chr18:21773623-21779685	70	MIB1
chr9:130854063-130863035	16	ABL1
chr2:9521202-9527954	11	ADAM17
chr8:130401884-130427822	8	ASAP1
chr13:25769045-25774959	45	ATP8A2
chr11:119273867-119274953	183	CBL
chr12:28362437-28391411	15	CCDC91
chr2:207740654-207751119	6	CCNYL1
chr22:28734402-28734727	13	CHEK2
chr3:33663444-33689932	21	CLASP2
chr14:69089947-69091887	5	DCAF5
chr1:168038370-168045227	21	DCAF6
chr22:38495795-38498573	18	DDX17
chr12:50685832-50697175	8	DIP2B
chr21:37410569-37421866	11	DYRK1A
chr12:53021805-53028188	72	EIF4B
chr9:20717793-20758191	9	FOCAD
chr14:51689690-51712551	6	FRMD6
chr5:151786571-151795575	75	G3BP1
chr12:120175161-120176217	35	GCN1
chr10:5768212-5800705	174	GDI2
chr8:41530183-41541899	17	GINS4
chr18:21395384-21403994	7	GREB1L
chr11:18283794-18286710	13	HPS5
chr15:98891324-98899621	12	IGF1R
chr7:23317638-23351586	9	IGF2BP3
chr10:863742-870083	15	LARP4B
chr19:45280373-45280734	79	MARK4
chr3:152445281-152447773	24	MBNL1
chr15:41696074-41697023	33	MGA

chr10:68174065-68174656	79	MYPN
chr18:12323715-12324544	7	nogene
chr2:155809927-155812794	9	nogene
chr2:10788696-10793202	11	PDIA6
chr6:42966781-42975038	6	PEX6
chr22:20764816-20765693	6	PI4KA
chr1:88740987-88760376	23	PKN2
chr1:167332469-167338258	24	POU2F1
chr12:42351884-42398994	108	PPHLN1
chr2:135661269-135675486	7	R3HDM1
chr2:37929261-38017235	6	RMDN2
chrX:72272639-72276234	6	RPS4X
chr3:9443307-9445740	7	SETD5
chr3:125451304-125453955	13	SNX4
chr4:123028185-123057288	52	SPATA5
chr2:168873584-168877384	13	SPC25
chr6:45105921-45365301	23	SUPT3H
chr1:11016843-11018873	28	TARDBP
chr6:42678538-42679832	10	UBR2
chr6:99507427-99510230	12	USP45
chr7:93291702-93311272	71	VPS50
chr18:56756582-56781656	9	WDR7
chr17:40256390-40260667	12	WIPF2
chr11:57682421-57688665	14	ZDHHC5
chr16:84979188-84981994	9	ZDHHC7
chr1:229525935-229531731	9	ABCB10
chr20:25306832-25309575	19	ABHD12
chr8:39054480-39055772	19	ADAM9
chr3:123347781-123352581	6	ADCY5
chr22:40358863-40360492	7	ADSL
chr1:243664771-243695716	8	AKT3
chr17:4235771-4249248	12	ANKFY1
chr2:96853400-96854441	8	ANKRD39
chr9:97994630-98005153	132	ANP32B
chr15:89888520-89889140	7	AP3S2
chr11:130126699-130133728	53	APLP2
chr15:34927034-34932434	12	AQR
chr15:34890214-34893773	8	AQR
chr14:32090501-32094386	9	ARHGAP5
chr6:157110471-157133207	51	ARID1B
chr5:78955294-78985558	62	ARSB
chr10:121910905-121924329	31	ATE1
chr12:123718622-123724791	17	ATP6V0A2

chr1:179378636-179395202	6	AXDND1
chr6:80342857-80343812	6	BCKDHB
chr17:67853939-67866687	7	BPTF
chr12:111649938-111660675	12	BRAP
chr17:43063332-43074521	25	BRCA1
chr7:25151595-25155192	5	C7orf31
chr1:200760838-200761098	33	CAMSAP2
chr11:34071725-34076642	468	CAPRIN1
chr12:48712261-48714524	10	CCNT1
chr3:138570317-138591956	8	CEP70
chr11:46795593-46809874	8	CKAP5
chr15:65153546-65155081	53	CLPX
chr9:67173859-67201219	15	CNTNAP3P2
chr7:43639448-43645399	7	COA1
chr16:67628369-67629533	156	CTCF
chr11:70405264-70414652	54	CTTN
chr10:14926842-14935564	6	DCLRE1C
chr3:182961225-182965753	20	DCUN1D1
chr1:85350414-85360537	45	DDAH1
chr10:68963290-68974743	8	DDX21
chr1:197617658-197658369	10	DENND1B
chr1:197583151-197772932	18	DENND1B
chr8:123015392-123030716	17	DERL1
chr12:50685832-50686682	6	DIP2B
chr11:112037272-112045230	7	DLAT
chr3:197130526-197149796	72	DLG1
chr7:87163494-87174669	119	DMTF1
chr10:127042624-127061776	32	DOCK1
chr15:65478879-65481615	11	DPP8
chr16:68025414-68054617	57	DUS2
chr2:55870719-55881734	21	EFEMP1
chr22:36523208-36524732	14	EIF3D
chr17:13010612-13013275	11	ELAC2
chr3:9986506-10023747	18	EMC3
chr19:43526200-43526659	11	ETHE1
chr12:11884444-11886026	17	ETV6
chr10:92997473-93058335	19	EXOC6
chr5:138006989-138011945	53	FAM13B
chr2:131055271-131055695	35	FAM168B
chr3:56627036-56628614	99	FAM208A
chr7:102822039-102822440	6	FBXL13
chr5:108186116-108367953	7	FBXL17
chr2:47835871-47839769	7	FBXO11

chrX:131749305-131770551	7	FIRRE
chrX:131711650-131785258	9	FIRRE
chr9:20715321-20874807	8	FOCAD
chr4:75658842-75662049	23	G3BP2
chr4:881906-884086	7	GAK
chr4:911672-913668	21	GAK
chr10:130145210-130166679	8	GLRX3
chr17:30481142-30490217	45	GOSR1
chr2:27638209-27641279	5	GPN1
chr10:1000676-1001013	5	GTPBP4
chr6:104833041-104852371	85	HACE1
chr2:171925536-171976308	19	HAT1
chr2:37037854-37057480	5	HEATR5B
chr10:68032777-68073686	15	HERC4
chr22:35262322-35263226	6	HMGXB4
chr8:42925556-42943445	85	HOOK3
chr7:27163574-27164967	10	HOXA10-HOXA9
chr9:92287790-92288282	13	IARS
chr10:132607914-132650505	13	INPP5A
chr19:7172289-7174731	6	INSR
chr6:18197056-18201657	8	KDM1B
chr5:138372673-138375206	30	KDM3B
chr5:138398177-138415239	10	KDM3B
chr14:35126734-35180777	60	KIAA0391
chr4:128466607-128483487	9	LOC100507487
chr1:84865384-84866138	20	LPAR3
chr2:43960540-43982434	6	LRPPRC
chr17:63634708-63657907	5	MAP3K3
chr18:54159764-54205157	32	MBD2
chr3:183057310-183071357	11	MCCC1
chr5:71592925-71604468	132	MCCC2
chr12:82369386-82438791	11	METTL25
chr2:42695764-42723035	10	MTA3
chr8:65669872-65693759	16	MTFR1
chr6:150905649-150926295	20	MTHFD1L
chr4:74174505-74281550	29	MTHFD2L
chr1:236803427-236806233	14	MTR
chr10:93323077-93329834	10	MYOF
chr1:58681784-58682545	13	MYSM1
chr11:78566131-78571444	15	NARS2
chr2:15511211-15558634	14	NBAS
chr1:148106180-148108654	7	NBPF11
chr12:124419956-124437996	28	NCOR2

chr4:169580841-169590809	97	NEK1
chr4:47884345-47894302	6	NFXL1
chr4:47903192-47905346	17	NFXL1
chr14:50792711-50806818	7	NIN
chr1:25315937-25318271	12	nogene
chr2:111321602-111344237	6	nogene
chr5:126626507-126626672	12	nogene
chr4:139403848-139404490	14	nogene
chr1:246186742-246199767	31	nogene
chrX:131332206-131364926	6	nogene
chr2:38782704-38782963	28	nogene
chr6:77151200-77212053	96	nogene
chr2:75120928-75122967	6	nogene
chr10:13637996-13648230	10	nogene
chr17:47596352-47613725	7	NPEPPS
chr12:68731109-68731719	6	NUP107
chr2:219566829-219568324	21	OBSL1
chr7:24861612-24892621	9	OSBPL3
chr17:2638098-2638320	5	PAFAH1B1
chr10:34470084-34696419	7	PARD3
chr1:233198938-233236980	102	PCNXL2
chr5:115236389-115238009	6	PGGT1B
chr11:17102661-17114465	5	PIK3C2A
chr16:8801798-8806407	15	PMM2
chr2:189791789-189795951	8	PMS1
chr18:54271359-54277855	27	POLI
chr2:44201185-44202045	6	PPM1B
chr14:103773632-103797518	8	PPP1R13B
chr14:23301885-23302159	9	PPP1R3E
chr9:125153532-125153985	11	PPP6C
chr14:73147794-73198129	65	PSEN1
chr1:96751424-96785254	10	PTBP2
chr8:140717597-140752316	11	PTK2
chr6:43138848-43139525	106	PTK7
chr2:119926597-119945240	28	PTPN4
chr4:17501718-17509363	35	QDPR
chr10:98156092-98163397	68	R3HCC1L
chr20:20639784-20653587	6	RALGAPA2
chr7:22219865-22230919	5	RAPGEF5
chr9:133408767-133412921	32	REXO4
chr17:32182756-32194107	8	RHOT1
chr6:45422592-45438051	5	RUNX2
chr11:9989496-10002689	11	SBF2

chr3:195668518-195677848	6	SDHAP2
chr7:3619079-3642105	57	SDK1
chr10:119902798-119904277	24	SEC23IP
chr4:109530288-109531522	25	SEC24B
chr8:66793615-66798625	6	SGK3
chr9:37948634-37974837	5	SHB
chr19:16552805-16555397	5	SLC35E1
chr10:103990674-103993133	10	SLK
chr3:57841298-57858159	29	SLMAP
chr14:77732484-77736006	7	SNW1
chr18:26052623-26078160	61	SS18
chr2:152144887-152150229	12	STAM2
chr15:42665252-42669337	16	STARD9
chr6:10893866-10898115	25	SYCP2L
chr6:10910147-10912927	5	SYCP2L
chr6:121279120-121299505	8	TBC1D32
chr3:17403180-17428519	9	TBC1D5
chr3:196062581-196065600	8	TFRC
chr2:105307613-105308318	6	TGFBRAP1
chr2:70224553-70230854	53	TIA1
chr12:29755708-29758777	5	TMTC1
chr10:91819269-91842391	20	TNKS2
chr1:235113767-235122372	7	TOMM20
chr3:133637875-133640170	6	TOPBP1
chr1:179085627-179088089	11	TOR3A
chr1:3740232-3746186	11	TP73-AS1
chr6:99526063-99531790	7	TSTD3
chr15:43376097-43377067	32	TUBGCP4
chr9:35236468-35259050	49	UNC13B
chr18:196636-203190	40	USP14
chr2:61211771-61214694	5	USP34
chr2:218474629-218474885	6	USP37
chr6:144789193-144803147	5	UTRN
chr4:84849901-84912319	8	WDFY3
chr7:64851798-64858602	7	ZNF138
chr16:2448854-2449915	13	CCNF
chr16:67947668-67950493	6	SLC12A4
chr18:12493071-12496102	23	SPIRE1
chr7:103115059-103128792	29	NAPEPLD
chr2:44490785-44505730	40	nogene
chr5:109713495-109716264	6	MAN2A1
chr10:133398434-133399653	55	MTG1
chr10:11948368-11952249	20	UPF2

chr15:22523346-22539247	23	HERC2P2
chr16:66816580-66821559	7	NAE1
chr1:75732643-75734871	26	ACADM
chr11:120406117-120431911	91	ARHGEF12
chr10:72128081-72213331	6	ASCC1
chr16:87975047-87984259	472	BANP
chr3:15230441-15240853	6	CAPN7
chr20:33573973-33574261	6	CBFA2T2
chr16:12704623-12705049	15	CPPED1
chr7:87163494-87171089	25	DMTF1
chr4:152411302-152469988	28	FBXW7
chr10:87057690-87062835	6	GLUD1
chr7:107189291-107196151	7	HBP1
chr14:61744704-61745817	14	HIF1A
chr19:38845852-38847434	16	HNRNPL
chr2:186633328-186641588	20	ITGAV
chr17:46082440-46172232	6	KANSL1
chr13:30255446-30280223	20	KATNAL1
chr11:67207509-67219403	9	KDM2A
chr18:50918109-50921187	17	ME2
chr11:20054079-20083179	8	NAV2
chr9:137272222-137272481	7	NELFB
chr2:172586277-172596023	6	PDK1
chr4:185523361-185525171	6	PDLIM3
chr13:32629292-32652007	5	PDS5B
chr10:3101364-3109480	12	PFKP
chr1:88740987-88786213	32	PKN2
chrX:338603-347693	171	PPP2R3B
chr6:170537233-170549113	83	PSMB1
chr10:98162882-98186563	9	R3HCC1L
chr2:135090997-135135932	6	RAB3GAP1
chr10:6097047-6115311	8	RBM17
chr6:33272627-33276066	7	RPS18
chr15:76753807-76767088	13	SCAPER
chr10:49943234-49958480	9	TIMM23B
chr20:50143501-50151315	15	TMEM189
chr1:211353238-211354467	69	TRAF5
chr5:14297071-14304592	12	TRIO
chr2:159248371-159282873	7	WDSUB1
chr8:38290474-38299590	6	WHSC1L1
chr1:35005131-35008924	10	ZMYM6
chr6:116647458-116660846	8	ZUFSP
chr10:94592228-94594854	7	HELLS

chr8:127982359-128010444	12	nogene
chr10:31387123-31387798	10	nogene
chr14:88852970-88872452	8	TTC8
chr11:125643791-125644643	10	CHEK1
chr3:32762732-32764809	9	CNOT10
chr7:6040891-6041219	10	EIF2AK1
chr9:19086733-19096769	19	HAUS6
chr16:1557934-1607297	6	IFT140
chr1:233135012-233161363	162	PCNXL2
chr2:152143826-152150229	11	STAM2
chr11:118779631-118786518	43	DDX6
chr8:47440322-47491994	14	SPIDR
chr7:149153944-149166930	5	ZNF398
chr2:158620989-158621421	8	PKP4
chr9:131436619-131459356	9	PRRC2B
chr14:101892999-101909690	10	PPP2R5C
chr16:66608308-66610003	112	CMTM3
chr20:63928334-63931182	21	DNAJC5
chr4:53399729-53444103	47	FIP1L1
chr16:56935234-56936817	7	HERPUD1
chr3:152414940-152432920	58	MBNL1
chr6:43175705-43176667	8	SRF
chr2:43428099-43430302	53	THADA
chr4:39733358-39774933	13	UBE2K
chr15:34256228-34261020	17	SLC12A6
chr15:58640776-58665197	13	ADAM10
chr6:16657775-16753347	30	ATXN1
chr13:31215050-31286819	19	B3GLCT
chrX:130024989-130025379	29	BCORL1
chr2:32478634-32479617	76	BIRC6
chr9:135881632-135883078	4492	CAMSAP1
chr11:66599115-66600549	11	CCS
chr17:64519320-64529427	16	CEP95
chr16:66622061-66636581	13	CMTM4
chr5:138810037-138812302	149	CTNNA1
chr11:103115176-103121496	6	DYNC2H1
chr17:82092454-82092812	8	FASN
chr10:102375358-102377140	7	GBF1
chr3:155893517-155898840	13	GMPS
chr11:9420410-9431003	11	IPO7
chr10:63380317-63450105	11	JMJD1C
chr11:67217730-67228163	9	KDM2A
chr16:25128474-25151615	8	LCMT1

chr22:47704588-47728995	11	LOC284930
chr19:34221506-34221738	42	LSM14A
chr13:35040932-35050395	20	NBEA
chr5:150614557-150626886	9	nogene
chr1:28372167-28372574	8	nogene
chr1:233177801-233227371	21	PCNXL2
chr4:39913610-39920399	8	PDS5A
chr9:131506113-131507514	12	POMT1
chr18:12801969-12862744	6	PTPN2
chr17:5361207-5365238	6	RABEP1
chr9:136446854-136448161	27	SEC16A
chr4:26620612-26640187	20	TBC1D19
chr12:98527885-98531838	10	TMPO
chr11:1288623-1290409	14	TOLLIP
chr3:179681877-179708957	49	USP13
chr13:20799164-20800990	23	XPO4
chr10:74358443-74398579	7	ADK
chr11:119271734-119278713	8	CBL
chr6:33320010-33321567	7	DAXX
chrX:97348115-97462165	7	DIAPH2
chr8:42288633-42293512	12	IKBKB
chr9:131430089-131447206	9	PRRC2B
chr9:6460572-6465846	11	UHRF2
chr5:77463094-77467470	7	WDR41
chr9:86303085-86304947	34	ZCCHC6
chr1:226961376-226965737	37	ADCK3
chrX:110264488-110279274	9	AMMECR1
chr2:200634790-200642801	7	AOX1
chr5:140563593-140563915	6	APBB3
chr13:111283138-111288443	7	ARHGEF7
chr17:61743012-61744591	6	BRIP1
chr6:149578821-149580723	11	GINM1
chr2:190910262-190913364	10	GLS
chr14:21230318-21263336	39	HNRNPC
chr19:48161371-48165623	7	LIG1
chr3:188341655-188406313	7	LPP
chr2:170752526-170753006	55	nogene
chr12:122417218-122417622	8	nogene
chr4:34979647-35005121	14	nogene
chr9:131162990-131164144	12	NUP214
chr14:20352244-20354247	33	PARP2
chr5:160422301-160424330	15	PTTG1
chr12:55796024-55803728	15	SARNP

chr2:32193888-32206933	6	SLC30A6
chr2:152147161-152148300	7	STAM2
chr20:13528432-13537049	6	TASP1
chr2:97888051-97927487	6	TMEM131
chr15:63532646-63537156	63	USP3
chr17:31878249-31880754	31	UTP6
chr11:62839113-62839706	10	WDR74
chr7:139073855-139089759	14	ZC3HAV1
chr5:32414968-32415187	8	ZFR
chr3:195306510-195320813	62	ACAP2
chr5:73911274-73911575	19	ARHGEF28
chr4:83267585-83279114	36	COQ2
chr2:186740227-186754049	7	FAM171B
chr15:92885514-92892072	79	LINC01578
chr1:67395404-67402417	7	nogene
chr1:28202012-28202389	12	nogene
chr17:43204215-43204530	18	nogene
chr11:3773631-3776021	19	NUP98
chr7:5740972-5741644	15	RNF216
chr21:34834409-34887096	8	RUNX1
chr3:42555929-42563686	8	SEC22C
chr16:68875153-68880630	25	TANGO6
chr10:12403101-12406326	7	nogene
chr8:103401515-103407784	28	SLC25A32
chr4:107687038-107701285	12	PAPSS1
chr11:108138897-108140215	11	ACAT1
chr22:25661575-25678915	72	ADRBK2
chr12:49665831-49668554	20	FMNL3
chr1:176027571-176116681	10	RFWD2
chr5:148394713-148399132	6	FBXO38
chr11:57698558-57700397	41	ZDHHC5
chr13:24477700-24498229	12	PARP4
chr16:70329291-70365131	13	DDX19B
chr2:61343814-61350693	13	USP34
chr6:90550467-90571807	24	MAP3K7
chr11:76007533-76016980	8	UVRAG
chr15:72555270-72572165	9	ARIH1
chr17:63764015-63766194	43	CCDC47
chr15:74923647-74926887	219	COX5A
chr19:10173870-10177367	40	DNMT1
chr1:16633094-16635168	7	nogene
chr5:134158454-134174007	6	SKP1
chr8:124495471-124516030	8	TATDN1

chr20:31778812-31782390	9	TPX2
chr18:31864626-31890866	7	TRAPPC8
chr5:43122038-43123310	48	ZNF131
chr16:71738941-71739341	42	AP1G1
chr11:130109428-130130166	12	APLP2
chr8:130358016-130370274	13	ASAP1
chr2:9318523-9327911	138	ASAP2
chr21:33907340-33909211	8	ATP5O
chr13:32319076-32325184	6	BRCA2
chr2:202954004-202955758	12	CARF
chr6:110201557-110213160	5	CDC40
chr7:92117043-92118615	7	CYP51A1
chr6:113953276-113960018	6	HDAC2
chr20:34489265-34504403	30	ITCH
chr11:68347846-68365702	137	LRP5
chr1:39350784-39359264	38	MACF1
chr5:36197540-36227565	5	NADK2
chr2:15504144-15558634	14	NBAS
chr8:89964409-89971290	9	NBN
chr17:28163542-28185265	6	NLK
chr20:44305744-44306001	24	nogene
chr5:170878096-170892553	21	RANBP17
chr6:89368055-89387590	5	RRAGD
chr9:113256113-113258862	27	SLC31A1
chr13:29516137-29524253	7	SLC7A1
chr6:85517755-85536924	15	SNX14
chr9:88426381-88426591	8	SPIN1
chr12:108817037-108823361	69	SSH1
chr3:196073929-196077122	32	TFRC
chr2:229802251-229807864	15	TRIP12
chr6:42673790-42684871	5	UBR2
chr10:28535561-28596041	12	WAC
chr7:6580554-6585260	38	ZDHHC4
chr1:35405022-35405468	11	ZMYM4
chr19:11105219-11107514	12	LDLR
chr3:100283606-100290701	7	TBC1D23
chr7:33146241-33191563	10	BBS9
chr9:120437294-120439972	12	CDK5RAP2
chr8:60816386-60824016	11	CHD7
chr3:167704841-167720273	15	PDCD10
chr4:102722870-102726683	38	MANBA
chr4:128831732-128852268	46	JADE1
chr3:127710575-127722566	19	MGLL

chr2:177461892-177482186	6	AGPS
chr4:15625251-15644708	50	FBXL5
chr18:63344409-63362868	7	KDSR
chr3:170281165-170281968	9	PRKCI
chr10:69359896-69369624	8	HK1
chr7:6430479-6435020	8	DAGLB
chr3:195326884-195392147	12	ACAP2
chr2:177505505-177513908	6	AGPS
chr21:26021839-26053348	6	APP
chr14:96341521-96347341	158	ATG2B
chr15:59671182-59680308	31	BNIP2
chr2:230759958-230798897	24	CAB39
chr11:61323978-61326893	8	DDB1
chr1:244686596-244692020	14	DESI2
chr4:24570653-24576678	21	DHX15
chr19:45368629-45369147	37	ERCC2
chr5:172897001-172914838	6	ERGIC1
chr10:124806830-124826785	10	FAM175B
chr1:240329075-240355908	61	FMN2
chr4:88383220-88398209	6	HERC6
chr8:42957093-42982696	5	HOOK3
chr2:127335869-127343194	9	MAP3K2
chr3:183276871-183389779	6	MCF2L2
chr7:131375423-131414710	13	MKLN1
chr5:150541569-150542971	80	NDST1
chr4:47884345-47890703	10	NFXL1
chr17:81560027-81560455	7	nogene
chr20:63993998-63994526	14	nogene
chr1:246186742-246219976	6	nogene
chr5:97427235-97439618	8	nogene
chr16:70214471-70214904	6	nogene
chr10:73030270-73074915	9	P4HA1
chr10:34131462-34269899	6	PARD3
chr18:54280666-54287411	16	POLI
chr2:68186467-68188690	20	PPP3R1
chr11:20395802-20397721	12	PRMT3
chr9:120831330-120833456	131	PSMD5
chr8:140668268-140674404	17	PTK2
chr4:86672364-86701801	8	PTPN13
chr19:33011678-33026632	17	RHPN2
chr22:31602211-31602785	56	SFI1
chr5:72861807-72865729	73	TNPO1
chr2:32633875-32640410	74	TTC27

chr17:59731420-59765138	12	VMP1
chrX:118442243-118443687	12	WDR44
chr2:32290482-32301495	68	YIPF4
chr5:62517105-62517497	11	nogene
chr15:30922234-30925939	10	FAN1
chr16:53844154-53888951	71	FTO
chr14:102221153-102223957	10	nogene
chr15:65016435-65027040	12	MTFMT
chr18:21039471-21049880	6	ROCK1
chr6:148440183-148487715	7	SASH1
chr1:3781511-3785203	9	LRRC47
chr9:36651745-36665581	10	MELK
chr5:38996818-39021136	69	RICTOR
chr7:26684735-26739964	5	SKAP2
chr6:35619095-35620274	42	FKBP5
chr17:50964510-50964872	15	nogene
chr6:77151200-77214751	9	nogene
chr9:83999708-84001476	7	RMI1
chr14:73892026-73893932	13	ZNF410
chr3:49699475-49700709	16	RNF123
chr4:435769-438632	6	nogene
chr21:43004325-43024947	145	PKNOX1
chr2:210153494-210155404	19	KANSL1L
chr7:143292299-143292698	27	CASP2
chr15:78878212-78886227	34	MORF4L1
chr22:31187046-31196990	5	RNF185
chr12:101753371-101760143	9	GNPTAB
chr7:131429032-131466415	8	MKLN1
chr2:127520438-127521021	14	nogene
chr21:36247516-36253909	6	DOPEY2
chr15:42321784-42330672	15	GANC
chr14:73355625-73366981	10	NUMB
chr16:67744287-67759732	12	RANBP10
chr22:38245934-38248019	119	TMEM184B
chr20:34457389-34471515	9	ITCH
chr11:17959469-18008076	90	SERGEF
chr4:4279446-4281897	33	LYAR
chr1:247433768-247434062	7	nogene
chr1:94201719-94231643	76	ARHGAP29
chr10:7797046-7799194	11	ATP5C1
chrX:77589833-77595785	7	ATRX
chr22:17683213-17702386	33	BCL2L13
chr17:67920014-67924589	6	BPTF

chr21:39224407-39238573	19	BRWD1
chr11:47161190-47161438	26	C11orf49
chr6:107039748-107040524	18	C6orf203
chr11:34090178-34090678	13	CAPRIN1
chr17:40161752-40162153	10	CASC3
chr18:69873143-69896045	9	CD226
chr17:80994580-80998420	19	CHMP6
chr12:122288488-122316855	74	CLIP1
chr20:62909698-62914406	17	DIDO1
chr21:46484756-46498833	14	DIP2A
chr3:132473144-132478140	24	DNAJC13
chr12:63617303-63647365	39	DPY19L2
chr17:44852408-44855004	6	EFTUD2
chr1:20941490-20950108	10	EIF4G3
chr8:28155941-28162078	188	ELP3
chr2:26373366-26375148	92	EPT1
chr5:66012048-66028343	26	ERBB2IP
chr5:138517560-138529678	74	ETF1
chr5:176488950-176500146	5	FAF2
chrX:131743095-131756709	8	FIRRE
chr20:28586298-28591519	12	FRG1CP
chr2:31073062-31125253	11	GALNT14
chr19:19474032-19492712	17	GATAD2A
chr9:130099456-130101223	13	GPR107
chr9:5021962-5055788	17	JAK2
chr1:23044426-23050520	10	KDM1A
chr10:32017142-32019959	43	KIF5B
chr7:152220522-152224616	56	KMT2C
chr3:197832196-197835822	29	LRCH3
chr6:53833066-53842227	11	LRRC1
chr2:159729008-159743253	10	7-Mar
chr14:60808324-60818847	20	MNAT1
chr2:176296859-176328912	304	MTX2
chr12:124347824-124350737	15	NCOR2
chr1:236024069-236048989	9	NID1
chr5:15628257-15634464	11	nogene
chr3:28292735-28298350	7	nogene
chr11:8669671-8670034	25	nogene
chr12:100180552-100196979	13	nogene
chr20:44916755-44924256	12	PABPC1L
chr11:34931403-34992379	18	PDHX
chr7:5982823-6002636	7	PMS2
chr4:105396248-105424322	11	PPA2

chr19:45138513-45146480	9	PPP1R37
chr11:68515027-68519651	159	PPP6R3
chr12:112446275-112456898	50	PTPN11
chr8:127867438-127890998	11	PVT1
chr6:3727550-3738148	8	PXDC1
chr2:1673642-1693134	10	PXDN
chr10:98162882-98209899	6	R3HCC1L
chr3:128797942-128807671	7	RAB7A
chr17:58694930-58696859	7	RAD51C
chr5:170916464-170968377	7	RANBP17
chr2:86751895-86753558	13	RMND5A
chr12:120562944-120566980	7	RNF10
chr3:196483769-196488683	107	RNF168
chr20:37184179-37184373	95	RPN2
chr5:1065282-1073801	14	SLC12A7
chr2:200416378-200419496	10	SPATS2L
chr7:105203627-105203785	8	SRPK2
chr3:136417884-136477412	8	STAG1
chr1:234446802-234472811	20	TARBP1
chr7:5374054-5378024	126	TNRC18
chr1:154253899-154255755	11	UBAP2L
chr4:102809671-102826636	24	UBE2D3
chr2:61370320-61383336	6	USP34
chr10:11518534-11527567	27	USP6NL
chr13:51719197-51727790	14	WDFY2
chr6:43533906-43548460	75	XPO5
chr10:27131858-27136385	77	YME1L1
chr1:52431012-52435465	9	ZCCHC11
chr9:37126311-37247130	9	ZCCHC7
chr20:41213733-41269083	6	ZHX3
chr6:87215902-87222894	17	ZNF292
chr11:125575262-125579068	10	EI24
chr9:131430089-131444328	13	PRRC2B
chr15:50958491-50968377	14	AP4E1
chr1:180397515-180424223	6	ACBD6
chr9:34338712-34343583	7	NUDT2
chr5:32379114-32390437	19	ZFR
chr3:184319688-184324347	13	EIF4G1
chr1:176081151-176135086	10	RFWD2
chr2:222908732-222934687	8	ACSL3
chr16:53254437-53268126	19	CHD9
chr13:25309246-25321093	15	NUPL1
chr4:22461736-22478233	12	ADGRA3

chr11:107390073-107418287	337	CWF19L2
chr5:765842-770117	17	nogene
chr5:116298246-116303683	6	nogene
chr12:2917784-2918208	10	nogene
chr17:58703195-58720812	11	RAD51C
chr4:109521116-109521626	14	SEC24B
chr9:128352637-128353541	71	SLC27A4
chr4:20528948-20539584	15	SLIT2
chr1:43858134-43858636	26	ST3GAL3
chr22:41125863-41130003	13	EP300
chr19:33113766-33119117	7	GPATCH1
chr16:72523331-72570515	18	LINC01572
chr13:100301459-100309908	174	PCCA
chr7:45747214-45751646	9	SEPT7P2
chr1:83904078-83907661	7	TTLL7
chr20:3290128-3294629	13	C20orf194
chr2:201160431-201164084	23	CFLAR
chr8:127932052-128010444	6	nogene
chr3:170259968-170275287	6	PRKCI
chr17:82884144-82893632	34	TBCD
chr2:171117317-171117857	6	TLK1
chr11:108486156-108491167	6	KDELC2
chr1:50535368-50584811	27	FAF1
chr22:17655661-17702386	21	BCL2L13
chr17:44862712-44865065	6	EFTUD2
chr9:85958471-85962180	14	NAA35
chr11:68564302-68576043	12	PPP6R3
chr2:230045744-230046722	8	SLC16A14
chr9:127804284-127804700	10	FPGS
chr15:29766265-29773357	27	TJP1
chr7:75052710-75070769	15	WBSCR16
chr8:123344883-123348273	20	ATAD2
chrX:118542724-118546116	58	DOCK11
chr7:67243494-67279526	535	nogene
chr17:1675151-1676711	26	PRPF8
chr10:73713203-73721504	30	BMS1P4
chr20:28581918-28591519	12	FRG1CP
chr2:53750782-53765585	33	GPR75-ASB3
chr5:142131807-142135817	37	NDFIP1
chr16:69260049-69284244	6	SNTB2
chr5:180529274-180553471	114	CNOT6
chr17:68114421-68125480	42	LINC00674
chr3:185612606-185617611	8	SENP2

chr9:88418849-88426591	25	SPIN1
chr14:73892026-73898262	14	ZNF410
chr3:189127056-189132697	19	nogene
chr18:79096475-79214038	8	ATP9B
chr5:66012048-66023364	18	ERBB2IP
chr22:47671640-47687206	8	LOC284930
chr3:183070998-183092545	16	MCCC1
chr12:62484169-62501698	100	MON2
chr16:28955977-28964039	9	NFATC2IP
chr2:53948420-53949283	14	PSME4
chr18:8244057-8253414	13	PTPRM
chr15:56394164-56394834	34	TEX9
chr3:100372605-100377912	7	TOMM70A
chr12:10713210-10719143	23	YBX3
chr7:64917029-64918292	149	ZNF273
chr3:126470894-126472305	14	ZXDC
chr1:26794112-26795234	7	PIGV
chr13:28256291-28261458	91	PAN3
chr4:153580825-153589007	10	KIAA0922
chr4:10097710-10103986	480	WDR1
chr11:64859923-64860336	25	EHD1
chr15:67231229-67231897	8	AAGAB
chr3:195320700-195345317	42	ACAP2
chr5:5187724-5209246	6	ADAMTS16
chr17:19651546-19671956	24	ALDH3A2
chr17:68250187-68251178	7	AMZ2
chr12:110387738-110388623	13	ANAPC7
chr6:89566833-89606105	7	ANKRD6
chr4:41142986-41177690	82	APBB2
chr16:24964196-24970580	8	ARHGAP17
chr2:9297299-9323250	9	ASAP2
chr10:86890061-86900126	8	BMPR1A
chr12:111681636-111683307	13	BRAP
chr21:39213480-39225197	10	BRWD1
chr21:39247700-39250889	9	BRWD1
chr19:32879067-32882013	6	CEP89
chr2:200856681-200856985	37	CLK1
chr16:58580632-58583182	29	CNOT1
chr5:75379336-75381201	8	COL4A3BP
chr10:68936935-68943257	92	DDX50
chr3:172773902-172784803	14	ECT2
chr10:119107947-119120452	20	FAM45A
chr8:129862228-129879471	7	FAM49B

chr1:59339957-59378837	118	FGGY
chr4:48619273-48634490	19	FRYL
chr3:81670837-81753280	11	GBE1
chr16:74459681-74467848	6	GLG1
chr7:80188950-80203832	21	GNAI1
chr3:37286013-37296219	10	GOLGA4
chr6:134993757-135002842	8	HBS1L
chr12:112243352-112244009	12	HECTD4
chr15:28229673-28233341	9	HERC2
chr8:42964310-42974194	14	HOOK3
chr5:138425410-138428086	9	KDM3B
chr17:50741101-50741731	61	LUC7L3
chr7:2002064-2069338	14	MAD1L1
chr2:127329920-127343194	10	MAP3K2
chr3:179378340-179386629	27	MFN1
chr1:167765582-167788019	38	MPZL1
chr20:34762726-34768586	33	NCOA6
chrX:119930015-119932216	38	NKAP
chrX:1383600-1384388	11	nogene
chr12:122493917-122494273	7	nogene
chr5:65668047-65668676	17	nogene
chr2:111344060-111469987	10	nogene
chr5:177135940-177204292	9	NSD1
chr11:59639047-59639383	20	PATL1
chr11:86000642-86003451	16	PICALM
chr11:68596096-68603492	79	PPP6R3
chr2:135565736-135605016	8	R3HDM1
chr5:131430858-131433658	11	RAPGEF6
chr3:29868857-29897475	15	RBMS3
chr6:166934090-166952548	6	RNASET2
chr8:42870003-42887871	12	RNF170
chr19:49496471-49497168	7	RPS11
chr15:84687624-84691644	185	SEC11A
chr4:42020415-42023384	19	SLC30A9
chr7:140334428-140337349	20	SLC37A3
chr1:204116506-204117707	12	SOX13
chr3:136568764-136630981	11	STAG1
chr3:4362038-4376389	10	SUMF1
chr1:159918841-159920537	25	TAGLN2
chr12:110626361-110634779	9	TCTN1
chr13:113503495-113520733	28	TMCO3
chr15:43134256-43160794	7	TMEM62
chr14:102830333-102903429	37	TRAF3

chr22:28629551-28633620	14	TTC28
chr11:8089609-8090231	7	TUB
chr6:158429735-158452268	42	TULP4
chr17:18797578-18804266	29	TVP23B
chr22:21610856-21611043	14	UBE2L3
chr16:23562624-23567069	6	UBFD1
chr9:83677726-83678599	614	UBQLN1
chr15:43036177-43038232	6	UBR1
chr4:39531968-39534629	7	UGDH-AS1
chr19:18845979-18852296	6	UPF1
chr1:150077668-150110627	7	VPS45
chr16:74908907-74909917	8	WDR59
chr5:37437921-37479987	6	WDR70
chr4:1974862-1976679	17	WHSC1
chrX:68513326-68521497	28	YIPF6
chr1:52515890-52526373	10	ZCCHC11
chr12:42317339-42324104	28	ZCRB1
chr1:35413971-35415714	99	ZMYM4
chr2:95148884-95153259	34	ZNF514
chr19:51878388-51886912	6	ZNF577
chr1:227650955-227654476	7	ZNF678
chr16:19071927-19074035	11	COQ7
chr16:70567410-70570149	6	SF3B3
chr6:84212956-84215922	13	CEP162
chr17:75485427-75486426	9	KIAA0195
chr3:160423424-160423840	7	SMC4
chr16:68273817-68287871	6	SLC7A6
chr1:185214506-185231708	81	SWT1
chr3:15735409-15766312	12	ANKRD28
chr3:11347876-11426926	11	ATG7
chr10:35025131-35033273	10	CUL2
chr14:69075344-69091887	6	DCAF5
chr8:119846989-119853215	8	DSCC1
chr15:66435026-66436892	231	MAP2K1
chr1:30945345-30950261	6	PUM1
chr3:27411641-27452498	91	SLC4A7
chr12:76826907-76842981	6	ZDHHC17
chr21:39293810-39298582	10	BRWD1
chr11:9178131-9181084	10	DENND5A
chr21:37238459-37240639	47	DSCR3
chr7:13931501-13939246	7	ETV1
chr9:129899964-129900547	8	FNBP1
chr16:58718195-58719255	119	GOT2

chr10:97616230-97619683	7	MORN4
chr1:53787157-53797144	7	NDC1
chr12:12832701-12839204	12	nogene
chr2:60786946-60792289	15	PAPOLG
chr8:140864311-140925749	271	PTK2
chr19:40786664-40786978	37	RAB4B-EGLN2
chr3:17508473-17672705	11	TBC1D5
chr1:235437321-235442911	17	TBCE
chr6:158580939-158608750	8	TMEM181
chr2:71365428-71365706	8	ZNF638
chr20:47249286-47347926	9	ZMYND8
chr10:327005-345110	10	DIP2C
chr3:119923372-120093593	9	GSK3B
chr5:168500590-168510686	30	RARS
chr20:35709223-35739017	6	RBM39
chr5:108020924-108224228	37	FBXL17
chr18:79126266-79277196	61	ATP9B
chr1:92088697-92147786	6	BTBD8
chr10:49524032-49528525	6	ERCC6-PGBD3
chr1:212863724-212889257	16	FLVCR1
chr3:172133470-172251541	8	FNDC3B
chr9:97369921-97371706	17	LOC100499484-C9ORF174
chr18:58681169-58700591	12	MALT1
chrX:152468181-152487023	36	nogene
chr3:138681966-138691143	9	PIK3CB
chr9:112250928-112276013	25	PTBP3
chr2:65001447-65010763	6	SLC1A4
chr15:58912562-58921584	6	SLTM
chr10:94441895-94507347	8	TBC1D12
chr21:37108391-37148647	14	TTC3
chr4:84860411-84860622	19	WDFY3
chr1:15529087-15536814	15	DNAJC16
chr2:40160764-40164984	11	SLC8A1
chr11:122776218-122789308	10	UBASH3B
chr12:76821057-76822531	57	ZDHHC17
chr17:2613657-2638320	6	PAFAH1B1
chr20:33366858-33370629	40	CDK5RAP1
chr1:93210801-93217869	7	CCDC18
chr11:103280347-103399872	8	DYNC2H1
chr4:20256671-20268881	7	SLIT2
chr19:34430575-34445121	24	UBA2
chr11:108256214-108267342	10	ATM
chr6:335113-348841	8	DUSP22

chr12:50427764-50429090	12	LARP4
chr9:35231119-35243364	41	UNC13B
chr2:58198578-58232112	86	FANCL
chr6:149572284-149572603	8	GINM1
chr7:131411305-131445903	12	MKLN1
chr10:50519830-50590247	14	SGMS1
chr2:43498832-43527988	123	THADA
chr5:14374228-14381252	13	TRIO
chr20:35016007-35021356	6	TRPC4AP
chr11:122806409-122809907	11	UBASH3B
chr4:102576875-102584820	6	NFKB1
chr1:94458606-94483239	54	ABCD3
chr2:15603191-15607313	42	DDX1
chr5:171876284-171900100	11	FBXW11
chr8:81679125-81680783	7	IMPA1
chr6:116689319-116702150	8	KPNA5
chr22:21788256-21807846	7	MAPK1
chr6:130801816-130803194	175	nogene
chr1:171579353-171587751	12	PRRC2C
chr22:41257947-41261580	7	RANGAP1
chr8:100268784-100288267	16	RNF19A
chr10:50306843-50311415	7	SGMS1
chr22:30646597-30649253	13	SLC35E4
chr7:832504-833229	7	SUN1
chr8:119788337-119797846	197	TAF2
chr7:130184183-130192823	23	TMEM209
chr2:63484603-63487494	7	WDPCP
chr4:342291-344430	6	ZNF141
chr19:36823284-36825692	14	ZNF790
chr11:118754704-118757287	31	DDX6
chr4:73090666-73092300	91	ANKRD17
chr4:150415437-150491035	14	LRBA
chr14:102280647-102283592	20	MOK
chr7:66796617-66805396	21	RABGEF1
chr3:10046579-10064875	35	FANCD2
chr12:49752463-49759321	6	TMBIM6
chr11:102186017-102223752	7	YAP1
chr1:154966318-154967797	17	SHC1
chr13:98482264-98519473	14	STK24
chr20:32309712-32316874	12	KIF3B
chr11:93788000-93801972	8	MED17
chr5:135360496-135389126	8	H2AFY
chr5:180248972-180269409	6	MAPK9

chr17:60623520-60648082	91	PPM1D
chr1:231616643-231618059	11	TSNAX-DISC1
chr8:94738368-94739790	10	DPY19L4
chr1:94464774-94475737	7	ABCD3
chr22:25594298-25604453	38	ADRBK2
chr20:63700601-63706738	5	ARFRP1
chr17:68306942-68307711	50	ARSG
chr10:72152868-72196989	7	ASCC1
chr2:38313150-38319019	7	ATL2
chr11:74028286-74034278	11	C2CD3
chr1:93212100-93216746	52	CCDC18
chr10:32711619-32729457	9	CCDC7
chr1:227069776-227074364	10	CDC42BPA
chr16:3739577-3745354	7	CREBBP
chr17:82251378-82265796	9	CSNK1D
chr2:171448680-171453213	6	DCAF17
chr19:18924232-18924979	19	DDX49
chr10:12106245-12108015	17	DHTKD1
chr1:27993399-28004419	6	EYA3
chr6:170317369-170330550	53	FAM120B
chr8:93709770-93718788	6	FAM92A1
chr2:169538012-169544835	20	FASTKD1
chr5:151786571-151797428	9	G3BP1
chr16:4817597-4832912	6	GLYR1
chr1:155754374-155760641	5	GON4L
chr11:18339563-18341607	8	GTF2H1
chr2:177215538-177216188	9	HNRNPA3
chr18:35478281-35480563	35	INO80C
chr15:90426109-90433795	13	IQGAP1
chr6:18197056-18197661	10	KDM1B
chr10:32047411-32048551	31	KIF5B
chr19:11113534-11113762	6	LDLR
chr4:41603874-41619440	30	LIMCH1
chr5:1488390-1501603	90	LPCAT1
chr19:325634-336173	135	MIER2
chr4:70950699-70975286	13	MOB1B
chr10:30340225-30341640	25	MTPAP
chr16:47128324-47132025	35	NETO2
chr12:6983001-6997342	12	nogene
chr19:4949820-4950260	8	nogene
chr17:75120172-75120539	11	nogene
chr21:22062008-22066645	5	nogene
chr11:73909398-73914504	11	PAAF1

chr1:233227225-233252788	30	PCNXL2
chr11:17145667-17150655	19	PIK3C2A
chr8:140818276-140921132	13	PTK2
chr1:51919926-51937412	10	RAB3B
chr3:8890388-8913720	8	RAD18
chr3:128620256-128622409	7	RPN1
chr14:54702061-54737287	20	SAMD4A
chr3:47434924-47443091	6	SCAP
chr8:73582769-73673242	8	STAU2
chr11:1286001-1290409	16	TOLLIP
chr15:63044026-63062645	6	TPM1
chr2:61229547-61241819	5	USP34
chr6:148953871-148964563	9	UST
chr3:184886109-184900972	8	VPS8
chr1:155674684-155679512	6	YY1AP1
chr10:45663541-45663842	7	ZFAND4
chr1:35358924-35370627	9	ZMYM4
chr4:102580534-102582957	11	NFKB1
chr8:38247712-38253718	8	DDHD2
chr3:197076585-197119530	13	DLG1
chr5:71041521-71060170	21	GTF2H2C_2
chr12:122129629-122129915	21	MLXIP
chr19:13090298-13095729	13	NFIX
chr8:100220278-100231288	64	SPAG1
chr3:136398748-136423044	49	STAG1
chr1:161778243-161784096	89	ATF6
chr7:6046070-6050045	147	EIF2AK1
chr2:143951981-143970762	12	GTDC1
chr13:27567639-27581803	40	LNX2
chr2:149575710-149582271	6	MMADHC
chr1:37864311-37868614	8	INPP5B
chr1:147254869-147259918	12	CHD1L
chr16:22432725-22435812	7	RRN3P3
chr6:100766564-100848707	9	ASCC3
chr8:117804721-117812961	7	EXT1
chr19:32973183-32976678	7	FAAP24
chr10:93359832-93373085	8	MYOF
chr9:128498412-128499126	8	ODF2
chr2:199323802-199433514	7	SATB2
chr3:33408689-33425741	8	UBP1
chr7:44569983-44570877	9	DDX56
chr5:16877608-16917042	9	MYO10
chr1:40413015-40417096	8	SMAP2

chr2:9517900-9536828	6	ADAM17
chrX:131768548-131784039	21	FIRRE
chr4:3103823-3107423	38	HTT
chr20:34457389-34481206	10	ITCH
chr1:229486370-229487613	32	NUP133
chr10:89588651-89612048	12	PANK1
chr4:86693586-86701801	32	PTPN13
chr15:43842414-43844054	19	WDR76
chr2:29131257-29145367	6	CLIP4
chr5:141582311-141583615	128	DIAPH1
chr10:5709529-5714207	138	nogene
chr8:140735250-140739107	10	PTK2
chr9:6477621-6486925	8	UHRF2
chr8:102836255-102843747	10	AZIN1
chr5:69191794-69197109	37	CENPH
chr3:138570317-138572932	371	CEP70
chr11:68794803-68799355	9	CPT1A
chr16:1625972-1641187	330	CRAMP1L
chr10:14919737-14935564	15	DCLRE1C
chrX:54455447-54456567	10	FGD1
chr6:135039572-135042126	325	HBS1L
chr3:197704541-197705175	23	KIAA0226
chr1:113112478-113119523	24	LRIG2
chr2:171325840-171330698	21	METTL8
chr17:36508896-36511455	11	MYO19
chr20:63874340-63874890	7	nogene
chrX:46521966-46523125	9	nogene
chr4:105424195-105434161	10	PPA2
chr21:36712532-36731151	8	SIM2
chr9:33971650-33973237	143	UBAP2
chr5:109774787-109823837	8	MAN2A1
chr7:66560578-66573550	24	GS1-124K5.11
chr18:46855344-46864248	15	PIAS2
chr7:141621731-141641908	7	AGK
chr18:9204475-9211784	10	ANKRD12
chr12:110018344-110030758	8	ANKRD13A
chr2:95899307-95914180	7	ANKRD36C
chr5:78216054-78228239	9	AP3B1
chr2:70169556-70181997	9	C2orf42
chr7:25151595-25158530	27	C7orf31
chr21:36386059-36387730	6	CHAF1B
chr11:9178131-9207632	6	DENND5A
chr11:112043465-112051349	10	DLAT

chr2:25607235-25628384	67	DTNB
chr14:69228815-69230598	18	EXD2
chr4:55893551-55899884	9	EXOC1
chr19:33088133-33101574	28	GPATCH1
chr1:23337753-23338608	7	HNRNPR
chr15:40987090-40987974	18	INO80
chr5:137634036-137639977	12	KLHL3
chr19:2438162-2438531	39	LMNB2
chr17:45289235-45290765	9	MAP3K14
chr2:39258347-39267247	15	MAP4K3
chr2:31891991-31920910	146	MEMO1
chr9:21837907-21859425	13	MTAP
chr1:21470631-21473585	10	NBPF3
chr15:64499292-64504346	6	nogene
chr12:7201750-7202704	9	PEX5
chr6:43587271-43605319	17	POLH
chr19:33002424-33002812	10	RHPN2
chr11:77675035-77678153	48	RSF1
chr15:34256228-34258944	7	SLC12A6
chr18:2666156-2688747	9	SMCHD1
chr1:109347482-109355469	12	SORT1
chr15:29772063-29800702	23	TJP1
chr2:73730559-73732285	7	TPRKB
chr16:2050399-2055519	11	TSC2
chr12:109488571-109507735	19	UBE3B
chr2:128143093-128145967	8	UGGT1
chr3:184849070-184886156	6	VPS8
chr6:142189425-142204065	6	VTA1
chr18:76849525-76868863	100	ZNF236
chr5:177041777-177044533	8	ZNF346
chr19:52655560-52660843	255	ZNF83
chr8:140562452-140585311	16	AGO2
chr6:161146523-161154310	36	AGPAT4
chr7:129368469-129379749	11	AHCYL2
chr18:9208656-9239556	6	ANKRD12
chr1:161791407-161802272	11	ATF6
chr21:29351633-29352663	10	BACH1
chr7:82335134-82349649	7	CACNA2D1
chr10:118707491-118730410	17	CACUL1
chr12:28304625-28391411	11	CCDC91
chr4:185175785-185176051	113	CFAP97
chr7:6424751-6436533	19	DAGLB
chr14:95124195-95141748	20	DICER1

chr9:137743391-137762820	10	EHMT1
chr8:116724908-116726172	294	EIF3H
chr22:31441773-31449531	7	EIF4ENIF1
chr1:20904882-20942330	13	EIF4G3
chr17:7594237-7594757	59	FXR2
chr5:151786602-151804446	8	G3BP1
chr10:102375358-102377128	21	GBF1
chr20:34935575-34937023	10	GSS
chr20:34413741-34449480	7	ITCH
chr1:35466607-35479212	23	KIAA0319L
chr3:44829970-44840456	31	KIF15
chr9:34269256-34271930	53	KIF24
chr7:23123776-23140944	9	KLHL7
chr19:48157013-48162351	14	LIG1
chr5:140167364-140167802	19	LOC101929719
chr15:66435026-66444707	9	MAP2K1
chr3:183318067-183389779	10	MCF2L2
chr14:46855023-46882221	6	MDGA2
chr1:169235130-169287408	7	NME7
chr17:7475257-7475561	12	nogene
chr6:141900322-142006118	15	nogene
chr6:17668974-17669546	9	NUP153
chr2:174123179-174182524	12	OLA1
chr4:39866860-39869462	7	PDS5A
chr1:109281107-109282575	7	PSRC1
chr1:96769702-96777946	9	PTBP2
chr5:170918712-170968377	7	RANBP17
chr22:41264663-41274727	6	RANGAP1
chr20:18510821-18515736	20	SEC23B
chr16:29457631-29458015	19	SLX1A-SULT1A3
chr4:185264437-185288082	6	SNX25
chr2:230436380-230450255	10	SP100
chr2:45488239-45553730	12	SRBD1
chr6:35869009-35872728	12	SRPK1
chr11:3967551-4023987	7	STIM1
chr20:13559007-13600617	10	TASP1
chr9:100509004-100516771	13	TMEFF1
chr11:87067693-87157406	13	TMEM135
chrX:155506897-155545277	17	TMLHE
chr1:193075819-193077050	13	TROVE2
chr21:37108391-37135514	29	TTC3
chr6:42603587-42605859	17	UBR2
chr8:99156548-99193057	17	VPS13B

chr4:183207952-183209025	94	WWC2
chr12:10713210-10715783	11	YBX3
chr9:112111653-112157613	6	SUSD1
chr4:48684672-48687160	46	FRYL
chr10:7220410-7285954	109	SFMBT2
chr11:83273646-83280260	11	CCDC90B
chr9:128262562-128263092	10	GOLGA2
chrX:7077219-7085471	12	HDHD1
chr12:95494055-95496098	9	METAP2
chrX:1726066-1726490	10	nogene
chr2:45413113-45477075	18	SRBD1
chr17:17879626-17907531	25	TOM1L2
chr1:97549559-97573970	36	DPYD
chr13:111205288-111233293	32	ARHGEF7
chr18:79154503-79214038	6	ATP9B
chr7:33146241-33177591	365	BBS9
chr10:68736875-68742569	10	CCAR1
chr2:26373366-26383347	10	EPT1
chr1:230249183-230255344	6	GALNT2
chr2:26230191-26239143	9	HADHA
chr7:18829160-18835997	8	HDAC9
chr9:94429188-94441099	6	HIATL1
chr19:18931324-18939049	11	HOMER3
chr3:151409242-151416422	12	MED12L
chr1:70163379-70164137	6	nogene
chr14:73179582-73180018	10	nogene
chr9:125153532-125158382	6	PPP6C
chr20:20635417-20653587	6	RALGAPA2
chr2:72988257-73058596	13	SFXN5
chr8:91289135-91318380	6	SLC26A7
chr13:60439687-60485948	10	TDRD3
chr16:70258979-70268379	16	AARS
chr11:108131906-108134316	131	ACAT1
chr11:78101092-78109581	28	ALG8
chr3:43549719-43574864	8	ANO10
chr2:9344531-9356345	18	ASAP2
chr12:32327460-32328555	63	BICD1
chr16:8858349-8859335	91	CARHSP1
chr7:107281299-107283732	78	COG5
chr13:39694633-39727548	8	COG6
chr20:495718-508660	18	CSNK2A1
chr5:138824529-138904441	7	CTNNA1
chr7:6425987-6430607	18	DAGLB

chr4:51860542-51886638	7	DCUN1D4
chr6:83090217-83097115	8	DOPEY1
chr6:20479845-20486803	6	E2F3
chr2:152558739-152581049	6	FMNL2
chr10:13663452-13675044	37	FRMD4A
chr10:104259575-104263077	15	GSTO1
chr6:135037810-135042126	15	HBS1L
chr17:50064076-50068305	12	ITGA3
chr17:47676408-47677127	21	KPNB1
chr2:210455106-210476412	9	LANCL1
chr2:101823927-101829594	8	MAP4K4
chr22:20551435-20555148	13	MED15
chr8:117528650-117530827	395	MED30
chr6:167870382-167889326	25	MLLT4
chr10:46010222-46011206	20	NCOA4
chr8:97694810-97695233	6	nogene
chr8:42324479-42325463	8	nogene
chr22:32232605-32232962	20	nogene
chr12:49469795-49470126	12	nogene
chr4:80335743-80420400	7	nogene
chr17:82207730-82208048	7	nogene
chr3:189383743-189385040	6	nogene
chr5:177238236-177239865	35	NSD1
chr11:47797778-47803536	6	NUP160
chr17:2665371-2667198	23	PAFAH1B1
chr5:79656957-79669028	15	PAPD4
chr13:24498115-24501834	26	PARP4
chr20:9217389-9339037	24	PLCB4
chr3:127639244-127662311	7	PODXL2
chr11:20388018-20408032	7	PRMT3
chr10:122983271-122990407	9	PSTK
chr8:140846259-140890769	2477	PTK2
chr8:127855162-127890998	25	PVT1
chr1:10007569-10008274	74	RBP7
chr18:23462963-23464628	6	RIOK3
chr6:89372436-89380367	14	RRAGD
chr15:41466140-41470392	10	RTF1
chr2:109585125-109593119	498	10-Sep
chrX:119640691-119653040	85	6-Sep
chr1:150929966-150941428	51	SETDB1
chr10:67887416-67891554	23	SIRT1
chr7:134299522-134309479	6	SLC35B4
chr19:11003028-11003397	21	SMARCA4

chr7:17801587-17826091	10	SNX13
chrX:71368053-71387461	8	TAF1
chr17:62536169-62565137	70	TLK2
chr22:40277076-40285770	19	TNRC6B
chr3:189207375-189310539	10	TPRG1
chr1:184070615-184073006	6	TSEN15
chr20:44479984-44484530	6	TTPAL
chrX:16828093-16829770	11	TXLNG
chr2:202896064-202897415	24	WDR12
chr16:69783031-69787080	8	WWP2
chr1:31346639-31348974	60	ZCCHC17
chr10:31373017-31461237	259	ZEB1
chr6:38078039-38082457	93	ZFAND3
chr2:113913171-113917008	26	ACTR3
chr2:9509978-9521316	13	ADAM17
chr10:74600378-74602161	7	ADK
chr10:4830674-4909606	13	AKR1E2
chr8:130401884-130406193	11	ASAP1
chr2:175080659-175097593	15	ATF2
chr6:32120770-32126252	33	ATF6B
chr11:108329020-108332037	30	ATM
chr6:57182031-57183911	114	BAG2
chr7:73459535-73463099	11	BAZ1B
chr17:30266884-30274197	11	BLMH
chr16:53141178-53157541	37	CHD9
chr12:70342106-70346324	7	CNOT2
chr17:63792411-63800617	12	DDX42
chr11:112037272-112039397	25	DLAT
chr5:95906522-95919545	33	ELL2
chr8:108449822-108455930	451	EMC2
chr3:9969718-9977446	6	EMC3
chr12:131992172-132006302	10	EP400
chr6:35619095-35642843	503	FKBP5
chr6:2382853-2399037	6	GMDS-AS1
chr1:109579203-109586882	21	GNAI3
chr3:160300882-160319939	17	IFT80
chr15:69429110-69436722	11	KIF23
chr6:108319616-108402054	8	LACE1
chr6:149676259-149676737	7	LATS1
chrX:77870807-77875597	14	MAGT1
chr5:109729341-109774965	15	MAN2A1
chr19:18134578-18135841	14	MAST3
chr22:37911951-37919178	40	MICALL1

chr17:62664547-62664949	40	MRC2
chr5:32268700-32276742	105	MTMR12
chr2:95832599-95834977	37	nogene
chr1:58506059-58534331	21	OMA1
chr5:6737509-6742597	8	PAPD7
chr20:18142207-18145351	8	PET117
chr9:41031370-41053899	10	PGM5P2
chr5:149836262-149842377	16	PPARGC1B
chr1:30980061-30995220	16	PUM1
chr4:98404476-98421394	9	RAP1GDS1
chr5:131548046-131624334	7	RAPGEF6
chr6:70576819-70579566	10	SDHAF4
chr19:17497254-17497464	11	SLC27A1
chr12:64062916-64065219	8	SRGAP1
chr3:196053417-196060247	14	TFRC
chr5:14270824-14304592	32	TRIO
chr3:12505153-12519197	10	TSEN2
chr2:46950362-46957007	11	TTC7A
chr6:158429735-158431663	58	TULP4
chr3:44945167-44959460	52	ZDHHC3
chr3:44629073-44632599	10	ZNF197
chr22:23292540-23295155	9	BCR
chr1:225252300-225259253	13	DNAH14
chr9:100006505-100022226	7	ERP44
chr19:58243964-58246794	8	ZNF544
chr4:124709999-124711274	6	ANKRD50
chr16:4441374-4448848	6	DNAJA3
chr1:54205017-54210131	7	MRPL37
chr6:36158647-36158941	24	nogene
chrX:121048129-121048330	13	nogene
chr2:28935520-28942381	9	WDR43
chr22:41656902-41658352	6	XRCC6
chr16:29904851-29906127	106	ASPHD1
chr3:15217424-15220981	9	CAPN7
chr12:102039867-102046119	41	CCDC53
chr10:87059149-87068157	8	GLUD1
chr10:67988042-68014186	7	HERC4
chr16:4651962-4657363	9	MGRN1
chr4:139342825-139349581	13	NAA15
chr2:105855047-105855289	6	NCK2
chr5:170909660-170924550	45	RANBP17
chr12:99772921-99812311	19	ANKS1B
chr4:147909731-147966839	10	ARHGAP10

chr8:61517527-61567318	8	ASPH
chr8:61637946-61653660	9	ASPH
chr17:61847100-61861569	13	BRIP1
chr9:120467859-120491477	36	CDK5RAP2
chr16:58574608-58599511	16	CNOT1
chr15:69436137-69436722	82	KIF23
chr12:50435487-50441643	46	LARP4
chr8:91200612-91219005	35	LRRC69
chr17:61972726-61983114	11	MED13
chr15:25085189-25087599	30	nogene
chr2:200938301-200938801	9	nogene
chr14:31562067-31599379	9	NUBPL
chr11:68548066-68564432	6	PPP6R3
chr13:114040999-114056662	8	RASA3
chr17:76487711-76488132	6	RHBDF2
chr3:42555929-42569073	7	SEC22C
chr1:168231513-168243093	13	SFT2D2
chr8:103400414-103407784	59	SLC25A32
chr1:151658234-151683445	64	SNX27
chr1:109316849-109317969	20	SORT1
chr21:37087246-37108446	31	TTC3
chr4:75790149-75790797	35	USO1
chr2:61300950-61301454	24	USP34
chr4:84796520-84801864	8	WDFY3
chr6:13652658-13658832	18	RANBP9
chr6:88890494-88904955	8	RNGTT
chr1:19682159-19683444	6	TMCO4
chr1:229530198-229531731	19	ABCB10
chr7:1879377-1879840	6	nogene
chr16:2581606-2586893	6	PDPK1
chr15:68141945-68146700	113	PIAS1
chr2:112647652-112652798	6	SLC20A1
chr3:196072002-196075360	8	TFRC
chr22:46463706-46464345	15	CELSR1
chr19:34431860-34464131	52	UBA2
chr15:82627192-82641682	20	CPEB1
chr16:10977224-10982991	8	CLEC16A
chr5:82058494-82178587	6	ATG10
chr17:61034665-61084564	6	BCAS3
chr10:86838864-86892229	29	BMPR1A
chr7:122615180-122629328	28	CADPS2
chr12:96298868-96300360	56	CDK17
chr15:65450998-65452102	55	DPP8

chr10:119069445-119073937	9	EIF3A
chr3:10036286-10039845	12	FANCD2
chr7:5491230-5505630	12	FBXL18
chr10:67966682-67992682	19	HERC4
chr10:32019857-32021287	95	KIF5B
chr14:67269700-67279537	21	MPP5
chr10:12389870-12403369	10	nogene
chr17:81610209-81613494	8	NPLOC4
chr3:186791724-186792947	47	RFC4
chr14:49586579-49586878	7	RN7SL1
chr14:51243855-51249765	168	TMX1
chr7:98961260-98962427	34	TRRAP
chr5:77449759-77464809	166	WDR41
chr14:74809379-74810420	11	YLPM1
chr1:235484321-235496308	8	B3GALNT2
chr2:32473111-32477583	16	BIRC6
chr8:43002106-43013400	7	HOOK3
chrUn_GL000195v1:46556-49119	6	LOC389831
chr2:177231884-177234271	27	NFE2L2
chr4:47875126-47914205	6	NFXL1
chr6:44121861-44122192	23	nogene
chr4:84780107-84783074	8	WDFY3
chr12:894561-897681	35	WNK1
chr16:28132333-28156527	18	XPO6
chr16:89892192-89900794	14	TCF25
chrX:2392353-2425304	41	DHRSX
chrX:120447840-120449128	9	LAMP2
chr1:100913662-100921841	49	SLC30A7
chr3:172782162-172786574	56	ECT2
chr11:66210361-66211259	9	PACS1
chr14:47035010-47097123	13	MDGA2
chr8:65745406-65782843	12	PDE7A
chr10:127018709-127026424	24	DOCK1
chr11:103280347-103287605	71	DYNC2H1
chr1:240206798-240258032	114	FMN2
chr16:47258631-47313823	8	ITFG1
chr2:151438683-151443709	6	RIF1
chr2:101294013-101295181	19	RNF149
chr15:50634381-50639548	15	TRPM7
chr12:998323-1028572	172	ERC1
chr1:246437147-246441299	10	nogene
chr6:161022009-161049979	8	MAP3K4
chr7:102913085-102931933	33	FBXL13

chr14:21499763-21500682	8	METTL3
chr3:138698906-138712304	26	PIK3CB
chr4:112562370-112584174	9	ZGRF1
chr6:44104376-44116629	10	MRPL14
chr17:29507895-29510992	21	TAOK1
chr4:88038250-88056267	9	PKD2
chr3:56592969-56598300	6	CCDC66
chr8:67103036-67116156	14	CSPP1
chr19:47352753-47353735	9	DHX34
chr2:11455341-11457233	17	E2F6
chr2:54813231-54829477	26	EML6
chr3:33068229-33072713	18	GLB1
chr5:180261683-180287067	58	MAPK9
chr9:125559632-125585727	15	MAPKAP1
chr10:27158548-27165539	29	MASTL
chrX:121163288-121185843	6	nogene
chr10:68060510-68061295	14	nogene
chr20:58271362-58286446	12	PPP4R1L
chr12:118386113-118391282	17	SUDS3
chr1:1256991-1267992	67	UBE2J2
chr1:244422834-244424387	18	ADSS
chr10:31861394-31910563	45	ARHGAP12
chr1:93582300-93584121	56	BCAR3
chr2:96797011-96799648	7	CNNM4
chr1:155709772-155726019	11	DAP3
chr10:127362063-127384909	6	DOCK1
chr9:137728348-137744090	47	EHMT1
chr8:28737518-28743214	9	EXTL3
chr5:138011007-138019146	7	FAM13B
chr4:75648638-75655870	106	G3BP2
chr8:42906172-42950455	27	HOOK3
chr20:34480598-34481206	14	ITCH
chr3:183643479-183665039	8	KLHL24
chr3:197015364-197017290	11	MFI2
chr20:34714589-34715365	7	NCOA6
chr4:34996297-35005121	10	nogene
chr8:127842514-127843102	23	nogene
chr14:20318454-20318844	32	nogene
chr17:75208526-75213119	8	NUP85
chr2:174123179-174157864	15	OLA1
chr15:64817945-64819223	59	PIF1
chr4:83094677-83107950	9	PLAC8
chr2:86121478-86125515	24	PTCD3

chr19:16552805-16553909	6	SLC35E1
chr4:123056386-123090705	18	SPATA5
chr21:44052279-44063785	28	TRAPPC10
chr5:14290715-14316743	87	TRIO
chr12:122235785-122239945	11	VPS33A
chr16:28111816-28125848	31	XPO6
chr7:122129360-122133741	6	AASS
chr11:34197324-34204690	15	ABTB2
chr7:92327782-92371607	6	ANKIB1
chr10:27092405-27093799	17	ANKRD26
chr12:27380246-27390239	53	ARNTL2
chr2:175130137-175151158	6	ATF2
chr20:51656937-51690819	55	ATP9A
chr17:61743012-61780401	21	BRIP1
chr12:28204092-28259442	9	CCDC91
chr6:99561366-99562948	8	CCNC
chr1:156317165-156317547	14	CCT3
chr1:207757013-207761446	9	CD46
chr1:193135390-193138173	28	CDC73
chr16:29860580-29861259	11	CDIPT
chr7:90747680-90790652	112	CDK14
chr4:184716396-184717196	6	CENPU
chr5:98873592-98876833	6	CHD1
chr3:129265548-129267099	51	COPG1
chr10:35178888-35207051	25	CREM
chr8:67095292-67105975	10	CSPP1
chr5:141581914-141583615	54	DIAPH1
chr6:56843054-56900621	6	DST
chr4:55877916-55890386	8	EXOC1
chr3:33358966-33385728	11	FBXL2
chr2:47838858-47839769	46	FBXO11
chr5:108954728-108959347	19	FER
chr4:53414614-53444103	106	FIP1L1
chr19:18537600-18538436	324	FKBP8
chr12:2864319-2866521	21	FOXM1
chr13:32078833-32117473	21	FRY
chr3:180947864-180949343	132	FXR1
chr17:36554819-36557336	11	GGNBP2
chr9:83780381-83788678	8	GKAP1
chr7:66554584-66576929	33	GS1-124K5.11
chr15:63652413-63658718	7	HERC1
chr10:68025545-68073686	24	HERC4
chr1:212006638-212021212	20	INTS7

chr11:9408485-9420698	15	IPO7
chr9:539676-540667	8	KANK1
chr13:30208576-30241086	64	KATNAL1
chr4:165294358-165299656	26	KLHL2
chr12:25225613-25227412	6	KRAS
chr10:836402-885760	30	LARP4B
chr6:79513211-79519085	16	LCA5
chr4:118619978-118628226	11	LOC729218
chr4:150817123-150831976	10	LRBA
chr7:1980452-2014642	9	MAD1L1
chr15:67692478-67703408	32	MAP2K5
chr2:159729008-159752571	11	7-Mar
chr10:27158548-27161182	33	MASTL
chr2:8943186-8982144	13	MBOAT2
chr2:99186176-99195756	62	MRPL30
chr6:24405167-24409573	6	MRS2
chr1:155612420-155747896	9	MSTO2P
chrX:150592604-150619139	80	MTM1
chr9:137215202-137215377	25	NDOR1
chr16:3654960-3658064	10	nogene
chr7:66491939-66492217	37	nogene
chr11:642723-642883	8	nogene
chr2:189854502-189855000	7	nogene
chr17:7245214-7245608	6	nogene
chr5:73447480-73448201	9	nogene
chr4:148120157-148152613	7	NR3C2
chr12:102099105-102118583	15	NUP37
chr1:113738109-113738799	68	PHTF1
chr18:46827958-46829867	7	PIAS2
chr1:151224247-151232703	61	PIP5K1A
chr14:23301884-23302159	26	PPP1R3E
chr17:66687110-66689047	27	PRKCA
chr4:118313200-118316323	9	PRSS12
chr2:73088049-73089315	72	RAB11FIP5
chr8:60558851-60591969	12	RAB2A
chr19:11405146-11417059	6	RGL3
chr6:125045210-125046661	6	RNF217
chr2:218580560-218592707	9	RQCD1
chr2:218582970-218584721	9	RQCD1
chr11:4101992-4109703	6	RRM1
chr1:41105741-41117010	7	SCMH1
chr4:75967276-75974133	12	SDAD1
chr6:107921796-107929514	128	SEC63

chr3:47710682-47729094	9	SMARCC1
chr6:85557975-85565419	19	SNX14
chr1:43920403-43921638	11	ST3GAL3
chr19:1218416-1219413	12	STK11
chr1:172555868-172579267	13	SUCO
chr12:111933885-111938210	6	TMEM116
chr15:43415593-43416416	27	TP53BP1
chrX:101035612-101042312	73	TRMT2B
chr13:37745936-37746455	9	TRPC4
chrX:16818573-16820255	106	TXLNG
chr7:139269393-139279360	6	UBN2
chr1:77713199-77714783	7	USP33
chr6:99439768-99465136	39	USP45
chr17:59731420-59738947	731	VMP1
chr8:99156548-99170163	6	VPS13B
chr2:63949036-63965966	18	VPS54
chr2:58084088-58089723	441	VRK2
chr13:41865735-41868477	284	VWA8
chr2:28917892-28929710	40	WDR43
chr12:32745679-32747363	23	YARS2
chr18:62556202-62565096	37	ZCCHC2
chr8:141160631-141163429	91	DENND3
chr5:108294851-108348530	9	FBXL17
chr12:100591735-100618587	42	GAS2L3
chr12:112216291-112219489	8	HECTD4
chr16:4505483-4507012	14	HMOX2
chr7:2568953-2573488	14	IQCE
chr12:124340005-124342074	7	NCOR2
chr11:127940964-127941306	14	nogene
chr14:23322180-23325291	7	PABPN1
chr11:77353535-77374365	21	PAK1
chr14:70946914-70978648	9	PCNX
chr2:48474682-48486758	7	PPP1R21
chr3:49032182-49033228	13	QRICH1
chr1:245853463-245863886	6	SMYD3
chr6:43478086-43503508	9	TJAP1
chr7:157382245-157385611	1981	DNAJB6
chr11:78119181-78124214	8	ALG8
chrX:110264488-110270854	8	AMMECR1
chr3:48922747-48927813	142	ARIH2
chr16:18794598-18798834	9	ARL6IP1
chr2:215312497-215336124	14	ATIC
chr10:115120184-115215880	12	ATRNL1

chr12:111552279-111552977	151	ATXN2
chr14:34758703-34762223	18	BAZ1A
chr17:43063332-43067695	9	BRCA1
chr21:39218151-39218660	14	BRWD1
chr17:82171700-82193830	10	CCDC57
chr9:4676461-4676749	12	CDC37L1-AS1
chr11:95812931-95818904	87	CEP57
chr2:201129728-201149835	21	CFLAR
chr12:122340752-122341697	6	CLIP1
chr2:207567462-207577655	54	CREB1
chr10:35137781-35148491	11	CREM
chr10:125088190-125111093	64	CTBP2
chr11:108088526-108089623	10	CUL5
chr10:100260217-100262063	20	CWF19L1
chr15:22879912-22883011	7	CYFIP1
chr14:22571878-22575233	11	DAD1
chr12:32710928-32713371	11	DNM1L
chr10:126970701-127008804	23	DOCK1
chr1:212100251-212102952	64	DTL
chr11:103253284-103259977	7	DYNC2H1
chr21:37420298-37496258	10	DYRK1A
chr17:44881686-44894525	6	EFTUD2
chr9:137752330-137758011	52	EHMT1
chr22:41125863-41146816	6	EP300
chr5:138518691-138529678	9	ETF1
chr14:45008912-45025872	7	FAM179B
chr10:15816834-15843272	50	FAM188A
chr5:177537872-177539042	6	FAM193B
chr16:30942115-30942536	17	FBXL19
chr1:240177920-240234450	10	FMN2
chr17:82563353-82587272	40	FOXK2
chr14:30598482-30602131	101	G2E3
chr19:19492305-19494383	10	GATAD2A
chr10:87051582-87068157	7	GLUD1
chr4:88390651-88393582	11	HERC6
chr13:30463206-30463694	7	HMGB1
chr12:54283078-54283967	23	HNRNPA1
chrX:134473358-134498436	70	HPRT1
chr5:119525215-119536550	14	HSD17B4
chr1:154448124-154454581	6	IL6R
chr15:41079700-41092182	7	INO80
chr15:90491332-90492711	9	IQGAP1
chr3:124841382-124848558	15	ITGB5

chr12:26681873-26716242	12	ITPR2
chr5:138420705-138425582	9	KDM3B
chr17:34983001-34983552	12	LIG3
chr15:30647667-30667275	10	LOC100288637
chr4:128292933-128483487	16	LOC100507487
chr4:150277852-150286034	9	LRBA
chr4:150735257-150831976	7	LRBA
chr7:2229983-2230724	40	MAD1L1
chr2:216277671-216283729	45	4-Mar
chr14:103462404-103468186	10	MARK3
chr6:83216497-83227477	6	ME1
chr17:40053297-40053635	10	MED24
chr12:95485878-95496098	12	METAP2
chr14:45217018-45218454	12	MIS18BP1
chr7:24623662-24680520	8	MPP6
chr9:21815432-21838010	7	MTAP
chr14:64431531-64440266	8	MTHFD1
chr13:41252644-41254620	9	MTRF1
chr8:17713213-17723833	17	MTUS1
chr9:126370164-126395697	13	MVB12B
chr22:36348903-36349255	10	MYH9
chr12:6521797-6522037	38	NCAPD2
chr12:124372021-124378384	10	NCOR2
chr18:58315981-58333892	24	NEDD4L
chr9:104750955-104759171	7	NIPSNAP3A
chr10:31373017-31391224	6	nogene
chr14:102281246-102281689	7	nogene
chr7:39949850-39950810	8	nogene
chr4:143808313-143866076	6	nogene
chr6:33307223-33307496	6	nogene
chr13:99071509-99072508	10	nogene
chr17:82100853-82104138	8	nogene
chr1:247199359-247200193	6	nogene
chr7:5613765-5616208	14	nogene
chr2:10644319-10684612	8	NOL10
chr6:17665238-17669028	28	NUP153
chr11:47783072-47786554	20	NUP160
chr11:47783072-47788611	6	NUP160
chr3:13351878-13354107	18	NUP210
chr7:24830767-24852634	6	OSBPL3
chr5:141409267-141410620	29	PCDHGB6
chr4:730629-737521	10	PCGF3
chr21:46349011-46349820	384	PCNT

chr2:61031418-61032113	31	PEX13
chr11:85996825-86014963	14	PICALM
chr14:103757649-103797518	23	PPP1R13B
chr11:68519500-68564432	7	PPP6R3
chr5:145817893-145823134	21	PRELID2
chr5:40764513-40775503	6	PRKAA1
chr15:78544868-78546698	48	PSMA4
chr1:96769702-96770851	38	PTBP2
chr9:94064733-94088263	11	PTPDC1
chr3:128806371-128807671	239	RAB7A
chr5:171158333-171242820	10	RANBP17
chr3:78932470-78938927	6	ROBO1
chr2:218580560-218583086	13	RQCD1
chr3:159812757-159866281	9	SCHIP1
chr3:72812670-72824551	10	SHQ1
chr15:75411491-75413045	45	SIN3A
chr2:17731077-17745951	42	SMC6
chr16:69245601-69260260	17	SNTB2
chr2:54612160-54618193	6	SPTBN1
chr4:56476670-56478649	7	SRP72
chr10:17704430-17705741	118	STAM
chr20:45000391-45001353	17	STK4
chr15:101703858-101714963	7	TARSL2
chr13:113610995-113623286	9	TFDP1
chr15:29759890-29773357	10	TJP1
chr3:129857249-129880559	28	TMCC1
chr16:24776932-24777358	6	TNRC6A
chr5:40728344-40730343	87	TTC33
chr9:121974008-121989770	25	TTLL11
chr1:83904078-83921394	6	TTLL7
chr1:83890320-83906463	9	TTLL7
chr21:43094470-43100519	13	U2AF1
chr7:139237004-139261741	14	UBN2
chr17:60265411-60271481	80	USP32
chr2:61203139-61211929	7	USP34
chr3:49292509-49294598	12	USP4
chrX:155220182-155228482	7	VBP1
chrX:118392631-118398470	6	WDR44
chr8:86398341-86402203	8	WWP1
chr6:43560177-43567354	39	XPO5
chr2:216113015-216132387	8	XRCC5
chr3:183772304-183777700	7	YEATS2
chr6:87215902-87233527	140	ZNF292

chr1:77633114-77641655	100	ZZZ3
chr15:41757787-41760529	11	MGA
chr8:99102952-99121445	12	VPS13B
chr5:73058432-73077493	108	FCHO2
chr15:72020917-72046634	13	MYO9A
chr4:67795747-67802411	6	nogene
chr12:79786373-79788783	6	PPP1R12A
chr4:120777187-120821345	13	PRDM5
chr4:76995780-77020670	16	11-Sep
chr13:45411361-45411938	14	SLC25A30
chr8:38337304-38348215	6	WHSC1L1
chr1:162566011-162581459	12	UAP1
chr17:63764015-63764847	47	CCDC47
chr19:3496540-3496886	8	DOHH
chr10:67988662-68044563	7	HERC4
chr5:70042366-70044143	20	nogene
chr1:206415888-206430222	8	SRGAP2
chr13:102622876-102627924	73	TPP2
chr5:173108623-173110711	7	CREBRF
chr20:3912457-3918796	27	PANK2
chr7:5725323-5741644	10	RNF216
chr9:132288235-132311856	9	SETX
chrX:118441367-118444494	12	WDR44
chr12:6522827-6523346	13	NCAPD2
chr2:174915057-174940894	10	CHN1
chr6:52438303-52440603	8	EFHC1
chr17:18340042-18353817	19	SHMT1
chr1:16630889-16635168	12	CROCCP2
chr16:28711656-28715434	7	EIF3C
chr21:14968324-15043574	7	NRIP1
chr17:18867281-18877870	41	PRPSAP2
chr1:176149005-176176007	43	RFWD2
chr1:86719506-86724405	18	SH3GLB1
chr21:32650183-32653366	15	SYNJ1
chr14:73147794-73149550	8	PSEN1
chr12:19462509-19466850	86	AEBP2
chr2:202518821-202519052	27	BMPR2
chr4:2904763-2908604	10	ADD1
chr17:60656598-60657042	9	PPM1D
chr5:27276140-27281091	13	nogene
chr9:113256113-113257185	15	SLC31A1
chr19:33462994-33501001	11	PEPD
chr6:57193841-57196606	72	RAB23

chr17:80640442-80643810	9	RPTOR
chr1:243378720-243418076	6	SDCCAG8
chr1:168183901-168184878	11	TIPRL
chr15:50458999-50465191	7	USP8
chr2:159275545-159283093	14	WDSUB1
chr3:195295707-195302174	54	ACAP2
chr2:73550266-73559142	6	ALMS1
chr21:39224407-39232498	12	BRWD1
chr15:82552479-82557986	8	CPEB1
chr2:241407536-241431774	11	FARP2
chr9:92256679-92285839	47	IARS
chr15:99255897-99287951	8	LRRC28
chr8:97690951-97723035	9	MTDH
chr15:25393745-25415842	18	nogene
chr14:70946914-70969110	28	PCNX
chr8:47887546-47893387	6	PRKDC
chr15:34250297-34251057	12	SLC12A6
chr1:156199834-156200472	49	SLC25A44
chr19:43744771-43748052	9	SMG9
chr3:30671637-30674246	26	TGFBR2
chr1:162499954-162501104	11	UHMK1
chr8:65693643-65704929	6	MTFR1
chr6:98899267-98905670	12	FBXL4
chr1:155343313-155346469	6	ASH1L
chr15:79356752-79383106	6	nogene
chr13:30637743-30647057	8	USPL1
chrX:100914620-100923061	6	XKRX
chr10:101672914-101673673	21	FBXW4
chr9:34338712-34339166	11	NUDT2
chr18:46827958-46846841	15	PIAS2
chr5:179705678-179706331	45	CANX
chr1:61762857-61775334	15	INADL
chr16:71676446-71681905	9	PHLPP2
chr5:39002534-39021136	238	RICTOR
chr14:102830333-102876525	32	TRAF3
chr4:109491326-109516640	13	SEC24B
chr14:105454192-105460953	8	MTA1
chr11:67217730-67219403	14	KDM2A
chr10:124681606-124696212	48	FAM53B
chr1:173839366-173840973	8	DARS2
chr10:13333816-13336350	16	SEPHS1
chr1:193135390-193152444	6	CDC73
chr5:131180094-131187109	24	LYRM7

chr18:70047970-70075541	6	RTTN
