# Supplementary material for: Accurate identification of circRNA landscape and complexity reveals their pivotal roles in human oligodendroglia differentiation
Source: Genome Biol. 2022 Feb 7;23:48. doi: 10.1186/s13059-022-02621-1 (PMC8819885; doi:10.1186/s13059-022-02621-1)
Supplement: Supplementary file 3 — Additional file 3. CircRNA list of each class detected by A-tailing RNase R method in HOG. [file 13059_2022_2621_MOESM3_ESM.pdf]

	SingleExon (1075)	
chr10	100238021	100238231
chr10	101803734	101804019
chr10	102139099	102142004
chr10	102360183	102360395
chr10	11851996	11852215
chr10	11956319	11956523
chr10	124045666	124046724
chr10	124681606	124682379
chr10	124706635	124706887
chr10	125038996	125039155
chr10	133467197	133467353
chr10	14896845	14897517
chr10	15283877	15284105
chr10	27169943	27171083
chr10	27208245	27208445
chr10	28595732	28596041
chr10	30450257	30450519
chr10	31461036	31461237
chr10	31861394	31861658
chr10	32017142	32017359
chr10	32284697	32285050
chr10	45625950	45627105
chr10	46010222	46011206
chr10	50343491	50344345
chr10	5906036	5906632
chr10	68121437	68122340
chr10	68174065	68174656
chr10	68422696	68422890
chr10	68747373	68747566
chr10	68959805	68960249
chr10	72196810	72196989
chr10	73053350	73053590
chr10	74842599	74843478
chr10	75150513	75151106
chr10	79310923	79311184
chr10	829384	829594
chr10	86517570	86518091
chr10	86875866	86876085
chr10	87151349	87152879
chr10	89611695	89612048
chr10	90748833	90749594
chr10	92537365	92537550
chr10	94030682	94032252
chr10	94245945	94246621

chr10	96949007	96949295
chr10	97567913	97568141
chr11	102114143	102114394
chr11	102335965	102336220
chr11	102368305	102368548
chr11	108172198	108173851
chr11	108267170	108267342
chr11	110136662	110137414
chr11	118786051	118786518
chr11	119126934	119127125
chr11	119232447	119232695
chr11	12162078	12162419
chr11	122903924	122904444
chr11	123059058	123059261
chr11	12764178	12764434
chr11	128480190	128480451
chr11	130260855	130261929
chr11	17356650	17357001
chr11	18291441	18292019
chr11	19933175	19934277
chr11	28058925	28059161
chr11	33286412	33287511
chr11	35725923	35726163
chr11	36227084	36227430
chr11	3702462	3702892
chr11	3731390	3731578
chr11	3768580	3768744
chr11	4074501	4074679
chr11	45979759	45979966
chr11	46816197	46816404
chr11	62589030	62589221
chr11	63749990	63750198
chr11	64859923	64860336
chr11	65079352	65079787
chr11	66639699	66640123
chr11	66643449	66644140
chr11	66668614	66669291
chr11	67231565	67231960
chr11	68175017	68175183
chr11	68189916	68190152
chr11	68347846	68348243
chr11	68537658	68537891
chr11	70272172	70272436
chr11	71147119	71147338
chr11	73844806	73844976

chr11	74078114	74078717
chr11	75404671	75404868
chr11	77672041	77672230
chr11	86306244	86306482
chr11	94799389	94800311
chr12	1027747	1028572
chr12	104927972	104928694
chr12	10709907	10710114
chr12	108701123	108701323
chr12	108792285	108792829
chr12	110342227	110342448
chr12	110377392	110377617
chr12	110551788	110551968
chr12	111418118	111418877
chr12	111883580	111883768
chr12	116237467	116237705
chr12	116985205	116985402
chr12	118095532	118095814
chr12	11869423	11869969
chr12	120224150	120224377
chr12	120546404	120546601
chr12	121509566	121510039
chr12	121819403	121819895
chr12	12203202	12203400
chr12	122340752	122341697
chr12	122377388	122377960
chr12	12244261	12244655
chr12	124372021	124372610
chr12	12635776	12635974
chr12	128814774	128815070
chr12	1289851	1290012
chr12	12908242	12909171
chr12	132729678	132730270
chr12	132754674	132755133
chr12	1754257	1754514
chr12	19283279	19283745
chr12	19493799	19493986
chr12	2873976	2874525
chr12	2994737	2994992
chr12	31451939	31452476
chr12	32305696	32306122
chr12	32327460	32328555
chr12	45421070	45421273
chr12	45836740	45836991
chr12	49460769	49461037

chr12	51067672	51067969
chr12	51073822	51074082
chr12	51662763	51663093
chr12	52073574	52073902
chr12	53018797	53019006
chr12	53381658	53381813
chr12	56210296	56210568
chr12	56643402	56643587
chr12	57250992	57251193
chr12	62552880	62553174
chr12	63802090	63802405
chr12	6521797	6522037
chr12	68302619	68302872
chr12	72286680	72286954
chr12	78006418	78007445
chr12	78137176	78137365
chr12	82857009	82857580
chr12	8930434	8930927
chr12	93678768	93679072
chr12	95282312	95282644
chr12	96246939	96247734
chr13	19772241	19772543
chr13	21045684	21046230
chr13	21167987	21168399
chr13	24222818	24224582
chr13	24297362	24297735
chr13	24455016	24455212
chr13	27581296	27581803
chr13	29517572	29517790
chr13	29535818	29536202
chr13	30283615	30283791
chr13	30630834	30631474
chr13	40559508	40560860
chr13	51251467	51252201
chr13	52024005	52024937
chr13	72778186	72778380
chr13	98213219	98213413
chr13	98519242	98519473
chr14	102085757	102086124
chr14	102325984	102326887
chr14	103405075	103405267
chr14	103474992	103475210
chr14	104166858	104167048
chr14	20407872	20408463
chr14	23033367	23033674

chr14	24974664	24974850
chr14	30605504	30605812
chr14	32433459	32433817
chr14	34862043	34862322
chr14	35053277	35053451
chr14	39095897	39096139
chr14	44945029	44945928
chr14	45175070	45176976
chr14	45246742	45247377
chr14	50150007	50150230
chr14	54702061	54702580
chr14	54737023	54737287
chr14	55612018	55612571
chr14	59726918	59727668
chr14	62950132	62950559
chr14	64065431	64065650
chr14	65561336	65561766
chr14	69658512	69658713
chr14	70742350	70742591
chr14	70800666	70801080
chr14	71101989	71102220
chr14	71105234	71105440
chr14	71587570	71589370
chr14	71685361	71685631
chr14	72993142	72993357
chr14	73287109	73287314
chr14	74692040	74692328
chr14	80784894	80785545
chr14	88550237	88550619
chr14	88754511	88754671
chr14	89411933	89412490
chr14	92021555	92021831
chr14	92651581	92653075
chr14	93263784	93263993
chr14	99257470	99257836
chr14	99257470	99257839
chr15	101235081	101235577
chr15	22534067	22534243
chr15	29053844	29054835
chr15	34003031	34003209
chr15	39617734	39618245
chr15	40571603	40571900
chr15	41668827	41669958
chr15	41696074	41697023
chr15	42736301	42736780

chr15	42751973	42753247
chr15	44458990	44459200
chr15	48644605	48644950
chr15	49292326	49292537
chr15	50643339	50643553
chr15	58851770	58851965
chr15	58899468	58899937
chr15	59030803	59031702
chr15	59080935	59081284
chr15	63774693	63775649
chr15	64300318	64300723
chr15	64376742	64376905
chr15	64823777	64824354
chr15	65106095	65106359
chr15	65500605	65500779
chr15	71660529	71660734
chr15	72045723	72046634
chr15	74655732	74656068
chr15	74671454	74671774
chr15	75422646	75422823
chr15	77133004	77133750
chr15	79889092	79889354
chr15	83041727	83041925
chr15	85533583	85533880
chr15	85655416	85655787
chr15	89601736	89601976
chr15	90067109	90068595
chr15	90981747	90981981
chr15	91915992	91916458
chr15	92967324	92967513
chr15	98707561	98708107
chr16	11003073	11003305
chr16	11123741	11123946
chr16	12051846	12052222
chr16	12704623	12705049
chr16	14644273	14644609
chr16	1602369	1602591
chr16	16044449	16044680
chr16	16056091	16056295
chr16	1659885	1660063
chr16	19871815	19872846
chr16	2173181	2173399
chr16	22225653	22225975
chr16	22471627	22471852
chr16	2319580	2319840

chr16	27545313	27545523
chr16	2756333	2756606
chr16	27749583	27750306
chr16	29809539	29809764
chr16	30704063	30704315
chr16	3308312	3308836
chr16	346147	347106
chr16	3850296	3851009
chr16	46909683	46909927
chr16	48252130	48252365
chr16	50078740	50079018
chr16	50287911	50288350
chr16	53155925	53157541
chr16	53825863	53826491
chr16	58534146	58534395
chr16	58560211	58560362
chr16	58599235	58599511
chr16	67685456	67685802
chr16	68121986	68123121
chr16	68190767	68191775
chr16	68274690	68275249
chr16	68859883	68860524
chr16	69284047	69284244
chr16	69336504	69336712
chr16	696772	697419
chr16	69695135	69695379
chr16	70120460	70120719
chr16	70530744	70530917
chr16	71714511	71714801
chr16	72950468	72950965
chr16	74360632	74360891
chr16	74651919	74652122
chr16	74660931	74661451
chr16	75242469	75243090
chr16	80684537	80685129
chr16	84101620	84102107
chr16	84744632	84745673
chr16	85633913	85634132
chr16	9102983	9103259
chr16	954356	954666
chr17	1364858	1365058
chr17	1436619	1437155
chr17	1436789	1437155
chr17	16061400	16061894
chr17	16101249	16101805

chr17	1799856	1801024
chr17	19958013	19958571
chr17	20204332	20205912
chr17	2638098	2638320
chr17	28628264	28628559
chr17	29285991	29287716
chr17	30803900	30804108
chr17	30904065	30904529
chr17	31325819	31326252
chr17	31988319	31988497
chr17	3518575	3518850
chr17	35362738	35363826
chr17	37161780	37162050
chr17	37330172	37330425
chr17	37553114	37554059
chr17	38716366	38716981
chr17	40126182	40126406
chr17	40330856	40331396
chr17	4064360	4064829
chr17	4242255	4242448
chr17	43200166	43200608
chr17	44117493	44117704
chr17	44147857	44149027
chr17	45475099	45475726
chr17	46170854	46172232
chr17	4940486	4940993
chr17	4946627	4946820
chr17	5331948	5332152
chr17	58494931	58496330
chr17	59353214	59353526
chr17	59698870	59700189
chr17	60623520	60623749
chr17	62010549	62011233
chr17	62664547	62664949
chr17	63354782	63355390
chr17	64212573	64214242
chr17	6447462	6447758
chr17	65053501	65053728
chr17	6628116	6628741
chr17	67853939	67854762
chr17	68306942	68307711
chr17	72849633	72849804
chr17	73235475	73236995
chr17	7448578	7448788
chr17	76063975	76064171

chr17	77402058	77402703
chr17	78031515	78031842
chr17	783082	783327
chr17	78400676	78400855
chr17	78803384	78803978
chr17	7945727	7945986
chr17	80339200	80340356
chr17	9022310	9023391
chr18	13681604	13682105
chr18	13731475	13731934
chr18	21532472	21532660
chr18	26285881	26286499
chr18	2937691	2938037
chr18	31931328	31931523
chr18	37066889	37067545
chr18	46215810	46216595
chr18	46890579	46891054
chr18	47115131	47115348
chr18	47896520	47896809
chr18	50984095	50984563
chr18	51067018	51067187
chr18	57731626	57731829
chr18	756556	756835
chr18	76378854	76380303
chr18	76441244	76443211
chr18	77016831	77017268
chr18	79410402	79411501
chr18	8824698	8826232
chr18	9522159	9522509
chr19	10143765	10143987
chr19	10491576	10492262
chr19	10499394	10500080
chr19	11113534	11113762
chr19	13025020	13025552
chr19	13993524	13993895
chr19	15272814	15273133
chr19	16500791	16501551
chr19	16509498	16509988
chr19	17101659	17102557
chr19	17497254	17497464
chr19	18147442	18147624
chr19	19296988	19297344
chr19	19465339	19465614
chr19	19498442	19498722
chr19	1969507	1969959

chr19	27797753	27798176
chr19	30012284	30012531
chr19	32352828	32354957
chr19	32953614	32953801
chr19	3381711	3382243
chr19	34221506	34221738
chr19	3550983	3551194
chr19	3623687	3624162
chr19	38119305	38119882
chr19	38130497	38130772
chr19	39422114	39422288
chr19	39453355	39453521
chr19	4053970	4055247
chr19	40557022	40557403
chr19	4117418	4117629
chr19	41292244	41292511
chr19	41378280	41378519
chr19	4408902	4409759
chr19	4433069	4433539
chr19	44707860	44708187
chr19	44907759	44907952
chr19	45271471	45271708
chr19	45421177	45421393
chr19	45831383	45831588
chr19	47352753	47353735
chr19	47355038	47355350
chr19	47362475	47362693
chr19	47716557	47717016
chr19	47725811	47726224
chr19	48182454	48182767
chr19	48446680	48446843
chr19	48633443	48633655
chr19	49196439	49196874
chr19	49601490	49601918
chr19	54458313	54458521
chr19	5648988	5649499
chr19	58089864	58090386
chr19	6270578	6270759
chr19	7184315	7184637
chr19	7444203	7444454
chr19	7462151	7462334
chr1	101075394	101075716
chr1	1022200	1022462
chr1	11013715	11013965
chr1	110222963	110223900

chr1	111147641	111148053
chr1	112917177	112918044
chr1	113797362	113798036
chr1	113904604	113904793
chr1	113908008	113908191
chr1	113940381	113941459
chr1	116544310	116544604
chr1	12275824	12278038
chr1	12506852	12507093
chr1	13778417	13782831
chr1	150334934	150335241
chr1	150446225	150446401
chr1	150457287	150457570
chr1	150460059	150460317
chr1	151161735	151162100
chr1	151236557	151236763
chr1	151315572	151316509
chr1	151638887	151639119
chr1	151658234	151658427
chr1	153949114	153949384
chr1	154601040	154602626
chr1	155338088	155338390
chr1	15536474	15536814
chr1	155438326	155439068
chr1	155477885	155482449
chr1	155521099	155521618
chr1	155777621	155777820
chr1	155853275	155853806
chr1	156135900	156136121
chr1	156199834	156200472
chr1	15872815	15873136
chr1	16148377	16149047
chr1	162566011	162566348
chr1	168104476	168104894
chr1	169477154	169477757
chr1	173802962	173803505
chr1	179341027	179341310
chr1	182884612	182884813
chr1	200760838	200761098
chr1	201817087	201817285
chr1	202924967	202925207
chr1	203847183	203847687
chr1	205187417	205187806
chr1	205268949	205269884
chr1	206205428	206206037

chr1	20941490	20942330
chr1	210919639	210920069
chr1	212100251	212101084
chr1	214464629	214464957
chr1	221706094	221706466
chr1	223798214	223798677
chr1	225023614	225023865
chr1	225987895	225988388
chr1	227028656	227029250
chr1	227747691	227748233
chr1	233258027	233259344
chr1	233262957	233263163
chr1	23960928	23961134
chr1	24514313	24514567
chr1	246591511	246591941
chr1	247418052	247419077
chr1	26255170	26255865
chr1	26794112	26795234
chr1	26897300	26897725
chr1	28473553	28474151
chr1	28480450	28480604
chr1	28742402	28743986
chr1	28987430	28987905
chr1	29154695	29154910
chr1	29275447	29275756
chr1	30721287	30721722
chr1	31059203	31059577
chr1	31918969	31919658
chr1	32592930	32593754
chr1	35415465	35415714
chr1	35478965	35479212
chr1	35746765	35746992
chr1	39368147	39368314
chr1	39547578	39548951
chr1	41142877	41143112
chr1	41628748	41628947
chr1	42278347	42278672
chr1	44008352	44008623
chr1	44411980	44412722
chr1	45087880	45088237
chr1	45457563	45457871
chr1	4711899	4712699
chr1	52525562	52526373
chr1	54209945	54210131
chr1	58681784	58682545

chr1	59321535	59321750
chr1	6231901	6232119
chr1	62447340	62447511
chr1	6599046	6599452
chr1	68494429	68494695
chr1	76313804	76313999
chr1	77631849	77633405
chr1	78013050	78013619
chr1	81907016	81907230
chr1	84865384	84866138
chr1	85164525	85164757
chr1	85190056	85190752
chr1	88740987	88741288
chr1	93674613	93674941
chr20	11918333	11918601
chr20	17969800	17970370
chr20	2116665	2117415
chr20	33379460	33379691
chr20	33573973	33574261
chr20	33611093	33611335
chr20	34105367	34105545
chr20	35477870	35478101
chr20	35842572	35842744
chr20	35899369	35899648
chr20	36578740	36578966
chr20	36793248	36794599
chr20	37044085	37044250
chr20	37184179	37184373
chr20	41533049	41533570
chr20	43702489	43702903
chr20	46240962	46241257
chr20	47246007	47246517
chr20	47276313	47276795
chr20	49269941	49271750
chr20	51009564	51010364
chr20	62893665	62894194
chr20	63202286	63203807
chr20	63619266	63619454
chr20	63775677	63775962
chr20	63789820	63790790
chr21	29321220	29321514
chr21	31266009	31266983
chr21	36896859	36897131
chr21	46168307	46168658
chr21	46334396	46334768

chr22	19253958	19254227
chr22	19977414	19977586
chr22	20488984	20489244
chr22	20554935	20555148
chr22	20933778	20934244
chr22	21610856	21611043
chr22	23095246	23096418
chr22	24187958	24188135
chr22	25375812	25376008
chr22	28163091	28163599
chr22	28734402	28734727
chr22	30972854	30973587
chr22	31462931	31463133
chr22	31738386	31738911
chr22	32478980	32479275
chr22	33761370	33761558
chr22	35809779	35810004
chr22	36348903	36349255
chr22	40881769	40882177
chr22	41117186	41117821
chr22	41650722	41650891
chr22	42209650	42215341
chr22	42890946	42891182
chr22	45178237	45178900
chr22	45842990	45843178
chr22	46198341	46198591
chr22	46463706	46464345
chr22	49822950	49824331
chr22	50240203	50240367
chr22	50265320	50265494
chr2	10419733	10420135
chr2	10441508	10441723
chr2	105307613	105308318
chr2	105342847	105343223
chr2	109347673	109347949
chr2	111123732	111124139
chr2	111863674	111863895
chr2	112502911	112503181
chr2	118096418	118096800
chr2	121605700	121606180
chr2	127293512	127293718
chr2	127954985	127955344
chr2	127989563	127989866
chr2	159282671	159283093
chr2	173954872	173956232

chr2	174877839	174878128
chr2	189863742	189864228
chr2	191693202	191693416
chr2	196433131	196433458
chr2	200419249	200419496
chr2	201266486	201266791
chr2	201284815	201285317
chr2	201420416	201420706
chr2	201760880	201761818
chr2	202518821	202519052
chr2	202555251	202556531
chr2	205440369	205440672
chr2	207123773	207124404
chr2	207976650	207977586
chr2	210153494	210154611
chr2	214780559	214781509
chr2	218474629	218474885
chr2	218656149	218656463
chr2	219252007	219252230
chr2	222908732	222909150
chr2	229858771	229859574
chr2	230045744	230046722
chr2	232087486	232087721
chr2	235040887	235043247
chr2	23823257	23823569
chr2	24080818	24081031
chr2	241373083	241373290
chr2	241468139	241468377
chr2	241632916	241634079
chr2	25313912	25314161
chr2	25628170	25628384
chr2	27607844	27608039
chr2	32414768	32416163
chr2	33020908	33021206
chr2	36396613	36396787
chr2	36517326	36517542
chr2	38343267	38343512
chr2	40428472	40430304
chr2	42682400	42682589
chr2	44201185	44202045
chr2	48346200	48346751
chr2	55025085	55027485
chr2	55598415	55599039
chr2	61031418	61032113
chr2	62000700	62000982

chr2	63404047	63404657
chr2	71189844	71190021
chr2	71348752	71350271
chr2	71426459	71427414
chr2	74046277	74048411
chr2	84870224	84870785
chr2	85356966	85357252
chr2	85542564	85542747
chr2	86151296	86151520
chr2	95405552	95405803
chr3	101785401	101785689
chr3	10238747	10239039
chr3	114350273	114351878
chr3	120750522	120751000
chr3	122565858	122566070
chr3	125313307	125313656
chr3	127639244	127639523
chr3	128637798	128638105
chr3	129670329	129671264
chr3	129827802	129828508
chr3	129880308	129880559
chr3	134361511	134361807
chr3	134370699	134371494
chr3	136604308	136604473
chr3	136945582	136946295
chr3	142512259	142512470
chr3	143985542	143985846
chr3	150571957	150572529
chr3	152299404	152300367
chr3	156677656	156678614
chr3	159764442	159765138
chr3	169381186	169381524
chr3	172251259	172251541
chr3	181699597	181699883
chr3	185689354	185689627
chr3	188609160	188609844
chr3	194427074	194427252
chr3	196055079	196055301
chr3	196362293	196362687
chr3	196566876	196567307
chr3	196885150	196886694
chr3	19950805	19951061
chr3	23900807	23901049
chr3	31830039	31830231
chr3	41398078	41398264

chr3	41954621	41954807
chr3	42200817	42201054
chr3	43576691	43577261
chr3	44455120	44455696
chr3	44794216	44794426
chr3	45110472	45110841
chr3	47610065	47610327
chr3	47998637	47998879
chr3	48927461	48927813
chr3	49510418	49510819
chr3	51419733	51420997
chr3	51590366	51590559
chr3	51684412	51684634
chr3	78938600	78938927
chr3	94003658	94003908
chr3	96987329	96987993
chr3	9744223	9744508
chr3	97864216	97864647
chr3	9828959	9829395
chr3	98881539	98881767
chr4	109462900	109463644
chr4	119606708	119607297
chr4	123028185	123028395
chr4	124709999	124711274
chr4	139043073	139043343
chr4	145514437	145515013
chr4	145902165	145903215
chr4	148435103	148436862
chr4	150102477	150102812
chr4	150908659	150908857
chr4	152411302	152411872
chr4	153295312	153296036
chr4	157152717	157153010
chr4	165310552	165310750
chr4	169136317	169136620
chr4	1727707	1728787
chr4	173247979	173248440
chr4	177353307	177353728
chr4	184009157	184011423
chr4	185175785	185176051
chr4	1900625	1901251
chr4	1918140	1918623
chr4	25847293	25847864
chr4	2630934	2631169
chr4	26362445	26362628

chr4	2672120	2672372
chr4	2699679	2700544
chr4	3253206	3253482
chr4	3316069	3318051
chr4	36228581	36229645
chr4	37902002	37902512
chr4	38993737	38993998
chr4	41013582	41014398
chr4	41142986	41143142
chr4	48710518	48710698
chr4	53145332	53145582
chr4	55411613	55411839
chr4	56019352	56019555
chr4	65601640	65602304
chr4	6824184	6824684
chr4	6923372	6924111
chr4	73090666	73092300
chr4	84860411	84860622
chr4	87195323	87195690
chr5	10239066	10239356
chr5	108798123	108798389
chr5	111669418	111669635
chr5	123545416	123545841
chr5	126603569	126604183
chr5	131315975	131316144
chr5	133959493	133960020
chr5	133980728	133980956
chr5	134661118	134661586
chr5	134741062	134741523
chr5	134854897	134855327
chr5	136153591	136154163
chr5	137948954	137949184
chr5	138418952	138419232
chr5	139307238	139308327
chr5	140194445	140194652
chr5	142353225	142353583
chr5	143399655	143400852
chr5	14487463	14488260
chr5	150520867	150521767
chr5	150618102	150618767
chr5	157791702	157791995
chr5	160010499	160010858
chr5	160093100	160094065
chr5	168568645	168568998
chr5	172234121	172234358

chr5	173090314	173091401
chr5	176289653	176290955
chr5	176496485	176496663
chr5	177531281	177531521
chr5	177631365	177632738
chr5	180571229	180571432
chr5	19838758	19839242
chr5	2749381	2749787
chr5	31983154	31983656
chr5	32414968	32415187
chr5	33881118	33881480
chr5	34822955	34824491
chr5	41927012	41927248
chr5	43161248	43161931
chr5	43381800	43382052
chr5	43453657	43454043
chr5	61472680	61473037
chr5	66053405	66054951
chr5	677749	678064
chr5	68226289	68227009
chr5	69419370	69420531
chr5	73840479	73840760
chr5	73911274	73911575
chr5	74634683	74636498
chr5	77118319	77118521
chr5	78509783	78510398
chr5	98779579	98780088
chr6	100848147	100848707
chr6	107633513	107635469
chr6	108663454	108664889
chr6	109481074	109482054
chr6	128322038	128322310
chr6	138255435	138255769
chr6	138291732	138292053
chr6	138333969	138335188
chr6	144577098	144577288
chr6	145804371	145806421
chr6	149378017	149379518
chr6	149623102	149623283
chr6	149683078	149684592
chr6	149701778	149702266
chr6	15496131	15497170
chr6	157084661	157084905
chr6	161048615	161049979
chr6	17625807	17626164

chr6	17688395	17688618
chr6	22020338	22020542
chr6	31663852	31664580
chr6	32971594	32972927
chr6	34132977	34133859
chr6	34606554	34606904
chr6	34646600	34646798
chr6	35017472	35018059
chr6	35410986	35411217
chr6	35423945	35424148
chr6	36211257	36211560
chr6	38061592	38061775
chr6	42268153	42269848
chr6	42573733	42573993
chr6	42828492	42830172
chr6	42966241	42966447
chr6	44250290	44250599
chr6	4891712	4892379
chr6	4935514	4935771
chr6	52452687	52454287
chr6	5368549	5369182
chr6	63646526	63646795
chr6	73440208	73440445
chr6	75702644	75703072
chr6	81751589	81752178
chr6	82214226	82214829
chr6	82240165	82240843
chr6	83125499	83125733
chr6	89725198	89725396
chr6	89781398	89781592
chr6	89803327	89803554
chr7	100591666	100591902
chr7	100856867	100857176
chr7	1009949	1010143
chr7	102178468	102178657
chr7	102227366	102227669
chr7	102438943	102439181
chr7	103128482	103128792
chr7	105203627	105203785
chr7	105508114	105508544
chr7	105665065	105665288
chr7	112286872	112287074
chr7	116699070	116700284
chr7	123496695	123496906
chr7	128479157	128479513

chr7	135438157	135438423
chr7	138871156	138871362
chr7	139715931	139717015
chr7	143381556	143381779
chr7	149153944	149154340
chr7	152176190	152178010
chr7	1569123	1569360
chr7	18666212	18666476
chr7	195553	195732
chr7	21481923	21482123
chr7	23185069	23185298
chr7	23611170	23611553
chr7	2364371	2364529
chr7	2369471	2369682
chr7	2794927	2795143
chr7	30456720	30457042
chr7	36356901	36357590
chr7	39987598	39988258
chr7	42148225	42148468
chr7	43595900	43596113
chr7	47368364	47369621
chr7	4740837	4741023
chr7	4877604	4878203
chr7	5287805	5287985
chr7	5287808	5287985
chr7	5312502	5313863
chr7	5370374	5371364
chr7	5500487	5502031
chr7	5641153	5641376
chr7	5740972	5741644
chr7	5740972	5741815
chr7	6017813	6018045
chr7	6111124	6111374
chr7	6153755	6155195
chr7	6466070	6466323
chr7	6591645	6591867
chr7	66240324	66241270
chr7	66286510	66286709
chr7	66805139	66805396
chr7	66944975	66945261
chr7	66993216	66993417
chr7	67017852	67018143
chr7	6737075	6737936
chr7	69899285	69899498
chr7	7234302	7234539

chr7	72831601	72831798
chr7	74338447	74339004
chr7	77749423	77750064
chr7	83134249	83135656
chr7	90726566	90726812
chr7	91973710	91973968
chr7	92294888	92295166
chr7	92307358	92307656
chr7	99388038	99388261
chr7	99434789	99435268
chr7	99525812	99526418
chr8	100261541	100261755
chr8	100287500	100288267
chr8	103697085	103697296
chr8	103766226	103766537
chr8	103885297	103886223
chr8	11444030	11444808
chr8	116726015	116726172
chr8	118580714	118580893
chr8	119801793	119802025
chr8	120515924	120516191
chr8	123231299	123231757
chr8	130091972	130092143
chr8	140585118	140585311
chr8	140890542	140890769
chr8	141253988	141254629
chr8	142343965	142344472
chr8	143440040	143440363
chr8	143803646	143803940
chr8	144580845	144581277
chr8	1565682	1565894
chr8	19319343	19319728
chr8	27294079	27294310
chr8	28970042	28970519
chr8	30069641	30070059
chr8	33388962	33389798
chr8	37846853	37847175
chr8	37877108	37877551
chr8	38347496	38348215
chr8	38819521	38820635
chr8	41598291	41599304
chr8	42436988	42437577
chr8	47440322	47440542
chr8	47893138	47893387
chr8	55762634	55763028

chr8	60741258	60743097
chr8	63186146	63187745
chr8	67095292	67095732
chr8	73688653	73688813
chr8	74244528	74245175
chr8	80499493	80500382
chr8	86402018	86402203
chr8	88167668	88167973
chr8	94491577	94491909
chr8	9580158	9580383
chr8	96784863	96785330
chr8	98547968	98548161
chr8	99511103	99511512
chr9	100441905	100442356
chr9	104127285	104127480
chr9	110136126	110138539
chr9	110156318	110156497
chr9	110941420	110942168
chr9	112574056	112575251
chr9	113988320	113988580
chr9	116651527	116651793
chr9	120518426	120518645
chr9	120787768	120788278
chr9	121150169	121150483
chr9	121989194	121989770
chr9	124535877	124536132
chr9	125298889	125299106
chr9	127890636	127891043
chr9	127912267	127912680
chr9	128185546	128185776
chr9	128345154	128345549
chr9	128508875	128509097
chr9	128907156	128909321
chr9	129502396	129503139
chr9	129503322	129503672
chr9	131459163	131459356
chr9	131470787	131470983
chr9	135882815	135883078
chr9	136049635	136050580
chr9	137180180	137180384
chr9	137716625	137717182
chr9	137728348	137728529
chr9	19276157	19276479
chr9	19331977	19332184
chr9	2161685	2161903

chr9	22820737	22821598
chr9	23762005	23762249
chr9	26919309	26919529
chr9	27359033	27359236
chr9	27455132	27455748
chr9	33294419	33295427
chr9	33948373	33948587
chr9	34241184	34242108
chr9	35546429	35548535
chr9	36246030	36246482
chr9	37126311	37126942
chr9	4117767	4118881
chr9	4286037	4286523
chr9	6433913	6434173
chr9	6984165	6984404
chr9	710803	713464
chr9	71740062	71740232
chr9	71750042	71750385
chr9	71874613	71875083
chr9	83677726	83677961
chr9	88426381	88426591
chr9	88462495	88462749
chr9	95880868	95881293
chr9	96458378	96458541
chr9	99137858	99138089
chrX	103585858	103586107
chrX	10566887	10567603
chrX	107088435	107088839
chrX	119410189	119410362
chrX	123885630	123886539
chrX	130024989	130025379
chrX	132054630	132054861
chrX	148661907	148662768
chrX	155506897	155507134
chrX	24179182	24179770
chrX	361404	361590
chrX	41123470	41123724
chrX	48925846	48926050
chrX	53411994	53412253
chrX	71378823	71379031
chrX	71567499	71567752
	**MultiExon (27501)**	
chr10	1000676	1001013
chr10	1000676	1007128
chr10	1000676	1009580

chr10	1000947	1007128
chr10	100154859	100176070
chr10	100174207	100179247
chr10	100190868	100205199
chr10	100204505	100218130
chr10	100217994	100222980
chr10	100243697	100246935
chr10	100243697	100250332
chr10	100243697	100253539
chr10	100243697	100256476
chr10	100245798	100246935
chr10	100256261	100260319
chr10	100256261	100262063
chr10	100260217	100262063
chr10	100497666	100498207
chr10	100507424	100509102
chr10	100536410	100546049
chr10	1005817	1007128
chr10	1005817	1009580
chr10	100750693	100781365
chr10	100779497	100781365
chr10	100779497	100787011
chr10	100806429	100809236
chr10	100915998	100918441
chr10	100918383	100926019
chr10	100923974	100926019
chr10	100923974	100931078
chr10	100923974	100938736
chr10	100937401	100944128
chr10	100937401	100959496
chr10	100950675	100959496
chr10	100990435	100991054
chr10	101584601	101585478
chr10	101584601	101586156
chr10	101624744	101676436
chr10	101667885	101673673
chr10	101667885	101676436
chr10	101672914	101673673
chr10	101672914	101676436
chr10	101790895	101798154
chr10	101792838	101807901
chr10	101798841	101800400
chr10	101798841	101804019
chr10	101798841	101807901
chr10	101798841	101810314

chr10	101798841	101813606
chr10	101800241	101807901
chr10	101800241	101810314
chr10	101806044	101813606
chr10	101889411	101957732
chr10	101889411	102025248
chr10	101939862	101957732
chr10	101939862	101975300
chr10	101939862	101992038
chr10	101975211	101995355
chr10	101975211	102014024
chr10	101990550	102002072
chr10	101990550	102009449
chr10	101990550	102029745
chr10	102001970	102014024
chr10	102006539	102040125
chr10	102013967	102040125
chr10	102023476	102025248
chr10	102023476	102029745
chr10	102023476	102033339
chr10	102023476	102040125
chr10	102025155	102033339
chr10	102029637	102033339
chr10	102029637	102040125
chr10	102033025	102040125
chr10	102109028	102111300
chr10	102109028	102111536
chr10	102157018	102158214
chr10	102258928	102260116
chr10	102351255	102353654
chr10	102358038	102359435
chr10	102360183	102370860
chr10	102361021	102363798
chr10	102362474	102363798
chr10	102362474	102365599
chr10	102366382	102367210
chr10	102375358	102377128
chr10	102375358	102377140
chr10	102397526	102398085
chr10	102397980	102398297
chr10	102465981	102469197
chr10	102473569	102474012
chr10	102484159	102485733
chr10	102592581	102597293
chr10	102592581	102599544

chr10	102654652	102656385
chr10	102685815	102705489
chr10	102699372	102705489
chr10	102726611	102727157
chr10	102731722	102733603
chr10	102872447	102874661
chr10	102872447	102878991
chr10	103091563	103095980
chr10	103099925	103174982
chr10	103139405	103174982
chr10	103139405	103181328
chr10	103174857	103181328
chr10	103314185	103333952
chr10	103326533	103348999
chr10	103345023	103347450
chr10	103345023	103348999
chr10	103347237	103348999
chr10	103373357	103380019
chr10	103378234	103379771
chr10	103432128	103434975
chr10	103438014	103438808
chr10	103439745	103440850
chr10	103897547	103910622
chr10	103990674	103993133
chr10	104008176	104018908
chr10	104122964	104124124
chr10	104259575	104263077
chr10	1072115	1078003
chr10	1072115	1080476
chr10	1072115	1086357
chr10	1072115	1096246
chr10	1072115	1105267
chr10	10786644	10792525
chr10	1080010	1080476
chr10	1080010	1086357
chr10	1080010	1096246
chr10	1084402	1086357
chr10	1084402	1096246
chr10	1084402	1105267
chr10	1093451	1105267
chr10	109893011	109915099
chr10	109907690	109915099
chr10	110100624	110119354
chr10	110100624	110122292
chr10	110100624	110124274

chr10	110118586	110124274
chr10	110125825	110130486
chr10	1103601	1105267
chr10	110502720	110507154
chr10	110581922	110582642
chr10	110589604	110591132
chr10	110593072	110598290
chr10	110596397	110598290
chr10	110780800	110784889
chr10	110881232	110883097
chr10	110964124	110985765
chr10	110985627	111004794
chr10	112168852	112173066
chr10	1124217	1125024
chr10	112527086	112538330
chr10	112951206	113146097
chr10	113040024	113089540
chr10	113141183	113146097
chr10	113721031	113726434
chr10	113876520	113884380
chr10	113898109	113905036
chr10	11481769	11485911
chr10	11481769	11501208
chr10	11481769	11518574
chr10	11481769	11527567
chr10	114830851	114836246
chr10	114830851	114847233
chr10	114830851	114848737
chr10	114843740	114848737
chr10	114951093	114974385
chr10	114959725	114974385
chr10	114970367	114974385
chr10	11501100	11527567
chr10	11509594	11527567
chr10	115120184	115129535
chr10	115120184	115165645
chr10	115120184	115171292
chr10	115120184	115241725
chr10	11518534	11527567
chr10	11518534	11597717
chr10	11525385	11597717
chr10	115268325	115286397
chr10	115426249	115469329
chr10	115461940	115469329
chr10	115461940	115519324

chr10	116681151	116681877
chr10	116901764	116915484
chr10	116901764	116921516
chr10	116901764	116927891
chr10	117318871	117341102
chr10	11747348	11755608
chr10	118038971	118040565
chr10	118691264	118701408
chr10	118695140	118730410
chr10	118701305	118730410
chr10	118729294	118730410
chr10	119041993	119044142
chr10	119051198	119059397
chr10	119051198	119059718
chr10	119056739	119059718
chr10	119059211	119061328
chr10	119059211	119065570
chr10	119065398	119073937
chr10	119069445	119071085
chr10	119069445	119073937
chr10	119070885	119073937
chr10	119072889	119073577
chr10	119072889	119073937
chr10	119073440	119073937
chr10	119107947	119117667
chr10	119107947	119120452
chr10	119146235	119156756
chr10	119146235	119164196
chr10	119155061	119160969
chr10	11929864	11943168
chr10	11936544	11952249
chr10	11942664	11952249
chr10	11948368	11952249
chr10	119515508	119527424
chr10	119526031	119527424
chr10	119576610	119580010
chr10	119576610	119588248
chr10	119577079	119580010
chr10	119577079	119582223
chr10	119578725	119588248
chr10	11964008	12014184
chr10	11967340	12014184
chr10	11979056	12004727
chr10	11979056	12014184
chr10	119796714	119811955

chr10	119804172	119811955
chr10	119836895	119849576
chr10	119838534	119842595
chr10	119843253	119849576
chr10	119847612	119853172
chr10	119902798	119904277
chr10	119904083	119909130
chr10	119912043	119915889
chr10	119914729	119926227
chr10	119926035	119932318
chr10	119930328	119933787
chr10	11997671	12004727
chr10	11997671	12014184
chr10	11997671	12035441
chr10	12028744	12035441
chr10	12081471	12084751
chr10	12081471	12087729
chr10	12081471	12091684
chr10	12081471	12094271
chr10	12081471	12101181
chr10	12081471	12108015
chr10	12081471	12113064
chr10	12081471	12120267
chr10	120858642	120871346
chr10	120878352	120904142
chr10	120880825	120890009
chr10	12088985	12094271
chr10	120900028	120904142
chr10	12094072	12097996
chr10	12100177	12101181
chr10	12112899	12120267
chr10	12117672	12120267
chr10	12118748	12120267
chr10	121538591	121565704
chr10	121551289	121565704
chr10	121564501	121565704
chr10	12157907	12161121
chr10	12177792	12186332
chr10	121790168	121870038
chr10	121836717	121870038
chr10	121898840	121924329
chr10	121902390	121911151
chr10	121902390	121922411
chr10	121902390	121924329
chr10	121910905	121913893

chr10	121910905	121924329
chr10	121913789	121924329
chr10	121959323	121961517
chr10	121959323	121963328
chr10	121960357	121974081
chr10	121963237	121965385
chr10	121965285	121974081
chr10	12209966	12217467
chr10	12209966	12238485
chr10	122132608	122143706
chr10	12215739	12238485
chr10	12217360	12238485
chr10	12230947	12238485
chr10	12235047	12238485
chr10	12235047	12246277
chr10	122392302	122406673
chr10	122392302	122413045
chr10	122392302	122416002
chr10	122392302	122417968
chr10	122392302	122424959
chr10	122392302	122427031
chr10	122393180	122417968
chr10	122400342	122416002
chr10	122412919	122417968
chr10	122412919	122424959
chr10	122488901	122489626
chr10	122937704	122953032
chr10	122948648	122953032
chr10	122951953	122953032
chr10	122995993	123007176
chr10	124012332	124046724
chr10	124021255	124046724
chr10	124038514	124044919
chr10	124038514	124046724
chr10	124405435	124412200
chr10	124408541	124408965
chr10	124408541	124412200
chr10	124484138	124488575
chr10	124484138	124498128
chr10	124496960	124517271
chr10	124681606	124696212
chr10	124681606	124706887
chr10	124696157	124706887
chr10	124774674	124790338
chr10	124806830	124826785

chr10	124806830	124831463
chr10	124816575	124831463
chr10	124828755	124831463
chr10	124966593	124974398
chr10	125038996	125111093
chr10	125038996	125133612
chr10	125729002	125741201
chr10	125747535	125749565
chr10	125749411	125753845
chr10	125752798	125753845
chr10	125807412	125816525
chr10	125812213	125816525
chr10	125827557	125831607
chr10	12666735	12769799
chr10	126970701	126981973
chr10	126970701	126987617
chr10	126970701	126990603
chr10	126970701	127000307
chr10	126970701	127008804
chr10	126970701	127012374
chr10	126970701	127018835
chr10	126970701	127023324
chr10	126970701	127037816
chr10	126970701	127127764
chr10	126977947	127000307
chr10	126996747	127000307
chr10	127008731	127012374
chr10	127008731	127018835
chr10	127008731	127023324
chr10	127008731	127127764
chr10	127012294	127061776
chr10	127018709	127026424
chr10	127018709	127032320
chr10	127018709	127037816
chr10	127018709	127061776
chr10	127023199	127026424
chr10	127023199	127032320
chr10	127031649	127037816
chr10	127031649	127043164
chr10	127031649	127052815
chr10	127037718	127127764
chr10	127042624	127061776
chr10	127052680	127061776
chr10	127061667	127110354
chr10	127061667	127127764

chr10	127061667	127257429
chr10	127106230	127127764
chr10	127125473	127127764
chr10	12760947	12769799
chr10	12791157	12816328
chr10	128040874	128056213
chr10	128073336	128079695
chr10	130145210	130160997
chr10	130145210	130166679
chr10	130145210	130166980
chr10	130145210	130175089
chr10	130159994	130166980
chr10	130159994	130175089
chr10	130166506	130169490
chr10	130166506	130171636
chr10	130169432	130175089
chr10	13108137	13109288
chr10	13108961	13109288
chr10	13110273	13122487
chr10	13123994	13133581
chr10	13127744	13133581
chr10	13164127	13171263
chr10	13164127	13175681
chr10	13172375	13172765
chr10	13172375	13175681
chr10	13172375	13183100
chr10	13172375	13186280
chr10	13172375	13189080
chr10	13175509	13183100
chr10	13180441	13192568
chr10	13182932	13192568
chr10	13182932	13195269
chr10	13188880	13192568
chr10	13188880	13201534
chr10	13191298	13192568
chr10	13191298	13195269
chr10	131934457	131947791
chr10	131940030	131945459
chr10	131940030	131947791
chr10	131970637	131973943
chr10	131970863	131973943
chr10	131973033	131973943
chr10	13197622	13198807
chr10	13197622	13201534
chr10	132222640	132228128

chr10	132337564	132344650
chr10	132607914	132650505
chr10	132607914	132697919
chr10	132645867	132650505
chr10	132690391	132697919
chr10	132690391	132710456
chr10	132690391	132726905
chr10	132708312	132726905
chr10	132708312	132749845
chr10	13280975	13283839
chr10	13280975	13288541
chr10	13280975	13295606
chr10	13280975	13298245
chr10	13283689	13298245
chr10	13291830	13295606
chr10	13291830	13298245
chr10	13294427	13295606
chr10	13322834	13345028
chr10	13328350	13345028
chr10	133297951	133302980
chr10	13333816	13336350
chr10	13333816	13345028
chr10	133379998	133384212
chr10	133395712	133402773
chr10	133395712	133419592
chr10	133401528	133402773
chr10	13496765	13500080
chr10	13597457	13616553
chr10	13605630	13611683
chr10	13605630	13616553
chr10	13663452	13666325
chr10	13663452	13675044
chr10	13737843	13747819
chr10	14848608	14855943
chr10	14851218	14867909
chr10	14867082	14867909
chr10	14881499	14897517
chr10	14919737	14935564
chr10	14922980	14935564
chr10	14926842	14935564
chr10	14928015	14935564
chr10	14934379	14934775
chr10	14945104	14945565
chr10	15109701	15119513
chr10	15109701	15128458

chr10	15109701	15130312
chr10	15119342	15141557
chr10	15128349	15130312
chr10	15128349	15135418
chr10	15128349	15141557
chr10	15140986	15141557
chr10	15248638	15254879
chr10	15248638	15284105
chr10	15254720	15284105
chr10	15275854	15284105
chr10	15796099	15847943
chr10	15816834	15834616
chr10	15816834	15838279
chr10	15816834	15843272
chr10	15816834	15847943
chr10	15821655	15834616
chr10	15821655	15843272
chr10	15821655	15847943
chr10	15833629	15843272
chr10	15833629	15847943
chr10	15837203	15847943
chr10	16467131	16505160
chr10	16484336	16486617
chr10	16695022	16754989
chr10	16752538	16754989
chr10	16752538	16764510
chr10	16752538	16782084
chr10	17157440	17168917
chr10	17603559	17604047
chr10	17693221	17696869
chr10	17700190	17705741
chr10	17704430	17705741
chr10	17705587	17708951
chr10	179993	210048
chr10	179993	221356
chr10	179993	240111
chr10	18585948	18596327
chr10	18585948	18616293
chr10	18596207	18616293
chr10	18609844	18616293
chr10	18609844	18648645
chr10	18642475	18648645
chr10	18666574	18668677
chr10	21534644	21538912
chr10	21586293	21617207

chr10	21612347	21651768
chr10	21614830	21670704
chr10	21670448	21682257
chr10	21688490	21727928
chr10	21713771	21727928
chr10	21713771	21735235
chr10	21805979	21882439
chr10	21805979	21929141
chr10	21882281	21920963
chr10	21904521	21920963
chr10	21904521	21929141
chr10	21918778	21920963
chr10	21918778	21929141
chr10	22317883	22319018
chr10	22326430	22328698
chr10	22326430	22329128
chr10	22550658	22573457
chr10	22550658	22591781
chr10	22567850	22591781
chr10	22567850	22609717
chr10	22591628	22608023
chr10	22591628	22609717
chr10	22607926	22609717
chr10	24591886	24595039
chr10	24619472	24630050
chr10	24619472	24722279
chr10	24629995	24635103
chr10	24629995	24670397
chr10	24666984	24670397
chr10	26702161	26709768
chr10	26704676	26705394
chr10	26704676	26709768
chr10	26705285	26709768
chr10	26705285	26720359
chr10	26720217	26724123
chr10	26765217	26770345
chr10	26765217	26771089
chr10	26934412	26935627
chr10	27006916	27017792
chr10	27012881	27014711
chr10	27014493	27017792
chr10	27039964	27043567
chr10	27067156	27086609
chr10	27077337	27086609
chr10	27082802	27093799

chr10	27092405	27093799
chr10	27093348	27093799
chr10	27114520	27117727
chr10	27114520	27121448
chr10	27114520	27136385
chr10	27117575	27121448
chr10	27119293	27121448
chr10	27119293	27126786
chr10	27121385	27123699
chr10	27122840	27123699
chr10	27122840	27126786
chr10	27122840	27131941
chr10	27131858	27136385
chr10	27134038	27136385
chr10	27134830	27136385
chr10	27136275	27145590
chr10	27142386	27145590
chr10	27158548	27159758
chr10	27158548	27161182
chr10	27158548	27165539
chr10	27165063	27165539
chr10	27167101	27171083
chr10	27169943	27173259
chr10	27169943	27181581
chr10	27173117	27181581
chr10	27173120	27181581
chr10	27205197	27215641
chr10	27208245	27211081
chr10	27215534	27219857
chr10	27215534	27231820
chr10	27526827	27533994
chr10	27532506	27533994
chr10	27907662	27944962
chr10	27935009	27936880
chr10	27935009	27944962
chr10	27981465	27983979
chr10	27981465	27985211
chr10	27981465	27987543
chr10	27981465	27995180
chr10	28535561	28583505
chr10	28535561	28589851
chr10	28535561	28590832
chr10	28535561	28596041
chr10	28583398	28590832
chr10	28583398	28596041

chr10	28583398	28608431
chr10	28589735	28596041
chr10	28589735	28608431
chr10	28595732	28608431
chr10	28608185	28614685
chr10	30322390	30326635
chr10	30322390	30341640
chr10	30326423	30341640
chr10	30336802	30341640
chr10	30340225	30341640
chr10	30450257	30451744
chr10	30451637	30459501
chr10	30876997	30910440
chr10	30876997	30941651
chr10	30908932	30984426
chr10	310030	349454
chr10	3101364	3109480
chr10	3112221	3116846
chr10	3116775	3120044
chr10	3116775	3134582
chr10	3118781	3120044
chr10	3119891	3133314
chr10	3132379	3133314
chr10	3132379	3135838
chr10	3144291	3147737
chr10	31461036	31495838
chr10	31461036	31502509
chr10	31461036	31514708
chr10	31495775	31502509
chr10	3158039	3160330
chr10	3158913	3160330
chr10	3160203	3170206
chr10	3163724	3167042
chr10	3163724	3170206
chr10	31817787	31831800
chr10	31820386	31843586
chr10	31820386	31852597
chr10	31820386	31854206
chr10	31820386	31861658
chr10	31826303	31861658
chr10	31831738	31852597
chr10	31839636	31852597
chr10	31843460	31854206
chr10	31843460	31861658
chr10	31852516	31854206

chr10	31852516	31861658
chr10	31861394	31910563
chr10	32017142	32018562
chr10	32017142	32019959
chr10	32019857	32021287
chr10	32021021	32021287
chr10	32022139	32023036
chr10	32028427	32037607
chr10	32034689	32037378
chr10	32034689	32037607
chr10	32037253	32037607
chr10	32037253	32040457
chr10	32038162	32040457
chr10	32038162	32048551
chr10	32273162	32291322
chr10	32284697	32293737
chr10	32451591	32462703
chr10	32451591	32474023
chr10	32451591	32565620
chr10	32471063	32474023
chr10	32472480	32518505
chr10	32472480	32567891
chr10	32517944	32571893
chr10	32543299	32567891
chr10	32583033	32584304
chr10	32583033	32686080
chr10	327005	345110
chr10	327005	349454
chr10	32711619	32729457
chr10	32908367	32910455
chr10	32908367	32935558
chr10	32919884	32935558
chr10	32920244	32930044
chr10	32920244	32935558
chr10	32923584	32926109
chr10	33226133	33270856
chr10	34119612	34131583
chr10	341198	345110
chr10	341198	349454
chr10	34131462	34269899
chr10	34131462	34284245
chr10	34269656	34284245
chr10	34269656	34341816
chr10	34317106	34341816
chr10	34331116	34341816

chr10	34331116	34360259
chr10	34347964	34360259
chr10	34359146	34360259
chr10	34359146	34384254
chr10	34359146	34399413
chr10	34359146	34401917
chr10	34372497	34384254
chr10	34374873	34384254
chr10	34377966	34384254
chr10	34399329	34401917
chr10	34399329	34450448
chr10	34450316	34470263
chr10	34450316	34517159
chr10	34450316	34696419
chr10	34470084	34517159
chr10	34470084	34696419
chr10	34516978	34696419
chr10	35011847	35028887
chr10	35013698	35028887
chr10	35013698	35029640
chr10	35013698	35031619
chr10	35013698	35033273
chr10	35016191	35031619
chr10	35025131	35029640
chr10	35028809	35033273
chr10	35029487	35031386
chr10	35029487	35033273
chr10	35029487	35049765
chr10	35032434	35039082
chr10	35032434	35049765
chr10	35044565	35054539
chr10	35044565	35071339
chr10	35054433	35063062
chr10	35054433	35071339
chr10	35060873	35071339
chr10	35148367	35179276
chr10	35178888	35188388
chr10	35178888	35207051
chr10	35188199	35207051
chr10	35483403	35516623
chr10	35483403	35530243
chr10	35483403	35553185
chr10	35501500	35530243
chr10	35516522	35530243
chr10	35525963	35530243

chr10	35525963	35553185
chr10	35529972	35566185
chr10	366274	384146
chr10	37833024	37856337
chr10	37833024	37856532
chr10	37837616	37838111
chr10	38012297	38017386
chr10	38016870	38017386
chr10	382646	390863
chr10	384026	409017
chr10	408925	415888
chr10	408925	423033
chr10	408925	440996
chr10	408925	472549
chr10	413912	423033
chr10	419064	423033
chr10	419064	472549
chr10	419064	486530
chr10	422823	486530
chr10	42614281	42636972
chr10	42631928	42632439
chr10	42631928	42636972
chr10	42787167	42790511
chr10	42796473	42802218
chr10	42797421	42802218
chr10	42816598	42817494
chr10	42816598	42823784
chr10	43154896	43160595
chr10	43158714	43167098
chr10	43158714	43176032
chr10	440870	486530
chr10	45625950	45639963
chr10	45639815	45653059
chr10	45639815	45663842
chr10	45648293	45663842
chr10	45651965	45663842
chr10	45662579	45663842
chr10	45728861	45746647
chr10	45752587	45753237
chr10	45862985	45900426
chr10	45888164	45910706
chr10	45888164	45916171
chr10	45888164	45939447
chr10	45888164	45946911
chr10	45888164	45962994

chr10	45900346	45916171
chr10	45939360	45962994
chr10	45946836	45962994
chr10	45975453	45988847
chr10	45982522	45988847
chr10	46010222	46013639
chr10	46014443	46016694
chr10	472438	486530
chr10	47542166	47544529
chr10	4830674	4835809
chr10	4830674	4842504
chr10	4830674	4847299
chr10	48401611	48404981
chr10	48401611	48410168
chr10	48420154	48431270
chr10	48555462	48583152
chr10	49482686	49506012
chr10	49493116	49506012
chr10	49500537	49506012
chr10	49832802	49843632
chr10	49832802	49857453
chr10	49832802	49861663
chr10	49832802	49865381
chr10	49832802	49869555
chr10	49832802	49879830
chr10	49841949	49861663
chr10	49841949	49869555
chr10	49857305	49861663
chr10	49879672	49935142
chr10	49915916	49932283
chr10	49915916	49934163
chr10	49915916	49935142
chr10	49922335	49935142
chr10	49943234	49955060
chr10	49943234	49958480
chr10	49952125	49952533
chr10	49952125	49955060
chr10	49952125	49958480
chr10	49989307	49994429
chr10	50001995	50008076
chr10	50069546	50084165
chr10	50090775	50093917
chr10	50104041	50113997
chr10	50327204	50344345
chr10	50433475	50466932

chr10	50433475	50519921
chr10	50433475	50590247
chr10	50460672	50466932
chr10	50460672	50590247
chr10	50466889	50590247
chr10	50519830	50590247
chr10	5452357	5453346
chr10	56359475	56360383
chr10	5717658	5720970
chr10	5720543	5727123
chr10	5726884	5731203
chr10	5726884	5735546
chr10	5730486	5735546
chr10	5730486	5742526
chr10	5766493	5800705
chr10	5768212	5773941
chr10	5768212	5800705
chr10	5773841	5786050
chr10	5773841	5800705
chr10	5785141	5800705
chr10	5785851	5800705
chr10	5794884	5796862
chr10	5794884	5800705
chr10	5796762	5800705
chr10	58216144	58237814
chr10	58361337	58368016
chr10	58388189	58390860
chr10	5880297	5883167
chr10	5883005	5885765
chr10	5903019	5911128
chr10	5903019	5927541
chr10	5956378	5960567
chr10	5959753	5960567
chr10	5959753	5966339
chr10	59804419	59814755
chr10	59812634	59852702
chr10	59832524	59852702
chr10	60780140	60785787
chr10	60784704	60792289
chr10	6097047	6101387
chr10	6097047	6112361
chr10	6101270	6106238
chr10	6213622	6217191
chr10	63176296	63197563
chr10	63185562	63191108

chr10	63190893	63194375
chr10	63192937	63194375
chr10	63193344	63197563
chr10	63217206	63219983
chr10	63610186	63619800
chr10	65571424	65615761
chr10	67887416	67889123
chr10	67887416	67891554
chr10	67887416	67906937
chr10	67888881	67891554
chr10	67891401	67909442
chr10	67932596	67936235
chr10	67932596	67941105
chr10	67932596	67955130
chr10	67932596	67956976
chr10	67932596	67992682
chr10	67932596	68038169
chr10	67939587	67941105
chr10	67954594	67956976
chr10	67954594	67966802
chr10	67954594	67988835
chr10	67954594	68014186
chr10	67954594	68034186
chr10	67954594	68044563
chr10	67956877	68044563
chr10	67966682	67992682
chr10	67966682	68014186
chr10	67966682	68025676
chr10	67966682	68038169
chr10	67988662	67991199
chr10	67988662	68014186
chr10	67988662	68044563
chr10	67988662	68073686
chr10	67990210	68014186
chr10	67990903	68014186
chr10	67990903	68044563
chr10	67992198	68014186
chr10	68014025	68044563
chr10	68014025	68073186
chr10	68014025	68073686
chr10	68025545	68038169
chr10	68025545	68044563
chr10	68025545	68073686
chr10	68032777	68038169
chr10	68032777	68073686

chr10	68033964	68073186
chr10	68033964	68073686
chr10	68038092	68044563
chr10	68038092	68073686
chr10	68044403	68073686
chr10	68072882	68073686
chr10	68109604	68122340
chr10	68114232	68158627
chr10	68121437	68158627
chr10	68142939	68148467
chr10	68142939	68150111
chr10	68142939	68158627
chr10	68142939	68161752
chr10	68142939	68166666
chr10	68142939	68174656
chr10	68142939	68175461
chr10	68142939	68189126
chr10	68142939	68206903
chr10	68145474	68158627
chr10	68145474	68161752
chr10	68150039	68158627
chr10	68150039	68166666
chr10	68165701	68189126
chr10	68165701	68195532
chr10	68174065	68175461
chr10	68175322	68189126
chr10	68175322	68195532
chr10	68197351	68199575
chr10	68288482	68289019
chr10	68292009	68306903
chr10	68296265	68306903
chr10	68337198	68341309
chr10	68337198	68341680
chr10	68338502	68341680
chr10	68341173	68341851
chr10	68355352	68364113
chr10	68355352	68386128
chr10	68363589	68364113
chr10	68381231	68393203
chr10	68383797	68386128
chr10	68383797	68401737
chr10	68393137	68394451
chr10	68393137	68396881
chr10	68393137	68401737
chr10	68393137	68404844

chr10	68394074	68394451
chr10	68394074	68401737
chr10	68394327	68401737
chr10	68394327	68404844
chr10	68422224	68422890
chr10	68422514	68432315
chr10	68430435	68432510
chr10	68430435	68470163
chr10	68431856	68437241
chr10	68437010	68445083
chr10	68437010	68446413
chr10	68437010	68470163
chr10	68442916	68445083
chr10	68442916	68446413
chr10	68450027	68468306
chr10	68450027	68470163
chr10	68459103	68465812
chr10	68459103	68470163
chr10	68459103	68471885
chr10	68465666	68468306
chr10	68465666	68470163
chr10	68468122	68470163
chr10	68487143	68488629
chr10	68487143	68493570
chr10	68487143	68503695
chr10	68487143	68506718
chr10	68487143	68516843
chr10	68488466	68493570
chr10	68493131	68493570
chr10	68722454	68742569
chr10	68722454	68749685
chr10	68722454	68754077
chr10	68722454	68757377
chr10	68736875	68737889
chr10	68736875	68742569
chr10	68736875	68747566
chr10	68737844	68742569
chr10	68737844	68747566
chr10	68740628	68742569
chr10	68740628	68747566
chr10	68742375	68749685
chr10	68747160	68747566
chr10	68747160	68755536
chr10	68747373	68749685
chr10	68753851	68757377

chr10	68753851	68766079
chr10	68754713	68757377
chr10	68757293	68766079
chr10	68786135	68786692
chr10	68786545	68789915
chr10	68787926	68789915
chr10	68881957	68892773
chr10	68906710	68910382
chr10	68906710	68911246
chr10	68906710	68914204
chr10	68906710	68919981
chr10	68910306	68911246
chr10	68910306	68914204
chr10	68911067	68914204
chr10	68911067	68919981
chr10	68913161	68914204
chr10	68913161	68919981
chr10	68913161	68934918
chr10	68934198	68936079
chr10	68936935	68941194
chr10	68936935	68943257
chr10	68941059	68943257
chr10	68959805	68962157
chr10	68959805	68963469
chr10	68959805	68965494
chr10	68959805	68967203
chr10	68963290	68967203
chr10	68963290	68969121
chr10	68963290	68970350
chr10	68963290	68974743
chr10	68965376	68967203
chr10	68965376	68970350
chr10	68967017	68969121
chr10	68968975	68974743
chr10	68970200	68977688
chr10	68970200	68978976
chr10	68973544	68974743
chr10	68973544	68981581
chr10	69000423	69011015
chr10	69005045	69011015
chr10	69155811	69158211
chr10	69185986	69186550
chr10	69185986	69187756
chr10	69185986	69191766
chr10	69189266	69197083

chr10	69195187	69203043
chr10	69196991	69199197
chr10	69198371	69203043
chr10	69359896	69369624
chr10	69364782	69369624
chr10	69364782	69382791
chr10	69376933	69380095
chr10	69483690	69485215
chr10	69483690	69495689
chr10	69483690	69498396
chr10	70153837	70161069
chr10	70153837	70161931
chr10	70204872	70209685
chr10	70206263	70209685
chr10	70209204	70218817
chr10	70217811	70218817
chr10	70844472	70851210
chr10	70844472	70859499
chr10	70844472	70868433
chr10	70868344	70871986
chr10	70885151	70885929
chr10	71510081	71511212
chr10	7188623	7220537
chr10	72128081	72133181
chr10	72128081	72152988
chr10	72128081	72196989
chr10	72128081	72213331
chr10	72161537	72213331
chr10	72196810	72203524
chr10	72196810	72213331
chr10	7220410	7227937
chr10	7220410	7276989
chr10	7220410	7285954
chr10	7227854	7285954
chr10	72340788	72341170
chr10	72407928	72423371
chr10	72407928	72477256
chr10	7243557	7276989
chr10	7243557	7285954
chr10	72475099	72508269
chr10	7248547	7285954
chr10	72508154	72566794
chr10	72562894	72566794
chr10	72715110	72715902
chr10	7276891	7285954

chr10	7283903	7285954
chr10	72859176	72871580
chr10	73010968	73074915
chr10	73014223	73051249
chr10	73030270	73047101
chr10	73030270	73068983
chr10	73030270	73074915
chr10	73043886	73053590
chr10	73068845	73074915
chr10	73068845	73077813
chr10	73072028	73074915
chr10	73138002	73163950
chr10	73146275	73152421
chr10	73146275	73163950
chr10	73152292	73160551
chr10	73156274	73156655
chr10	73156274	73163950
chr10	73160433	73163950
chr10	73174686	73210438
chr10	73192555	73193593
chr10	73192555	73210438
chr10	73378910	73380201
chr10	73396518	73398385
chr10	73396518	73400857
chr10	73398180	73400857
chr10	73454411	73479517
chr10	73470686	73471613
chr10	73474918	73479517
chr10	73478498	73479968
chr10	73504849	73505426
chr10	73516374	73519992
chr10	73534599	73539593
chr10	73536268	73539593
chr10	73539443	73543131
chr10	7367648	7370375
chr10	7367648	7381949
chr10	7370280	7381949
chr10	73704985	73721504
chr10	73713203	73721504
chr10	73713203	73722577
chr10	73718014	73722577
chr10	73719159	73721504
chr10	73725960	73728587
chr10	73727292	73728587
chr10	73746629	73747004

chr10	73800010	73800472
chr10	73802673	73810903
chr10	73802971	73803359
chr10	73817022	73848082
chr10	73819531	73824084
chr10	74043082	74074903
chr10	74070669	74072852
chr10	74070669	74074903
chr10	74070669	74083513
chr10	74070669	74094461
chr10	74070974	74074903
chr10	74074742	74090198
chr10	74089195	74094461
chr10	74089195	74105353
chr10	74097203	74101097
chr10	74097203	74105353
chr10	74107229	74109156
chr10	74200763	74398579
chr10	74314666	74398579
chr10	74394140	74398579
chr10	74394140	74525426
chr10	74394140	74600493
chr10	74525255	74600493
chr10	74589281	74670269
chr10	74600378	74670269
chr10	74838682	74843478
chr10	74838682	74960078
chr10	74838682	74989112
chr10	74969659	74985241
chr10	74969659	74989112
chr10	75150513	75164740
chr10	75150513	75168658
chr10	75150513	75176216
chr10	75168540	75176216
chr10	75219062	75220970
chr10	75219062	75222402
chr10	75882655	75883525
chr10	76036007	76047303
chr10	76036007	76058783
chr10	76909965	76970067
chr10	76944772	76970067
chr10	76949141	76970067
chr10	76969973	77019099
chr10	77073096	77090510
chr10	77084636	77090510

chr10	7759889	7766103
chr10	7759889	7769345
chr10	7778837	7780307
chr10	77794007	77796594
chr10	77794007	77812377
chr10	7780055	7783175
chr10	77824383	77830873
chr10	77833913	77835922
chr10	77853353	77854370
chr10	77853353	77856892
chr10	77853353	77869197
chr10	7796120	7799194
chr10	7796120	7800091
chr10	7796120	7802854
chr10	7797046	7802854
chr10	77980140	77981559
chr10	77982153	77984298
chr10	7798989	7802854
chr10	77991053	78007866
chr10	77993196	78019265
chr10	78002196	78004888
chr10	78002196	78019265
chr10	78002196	78022384
chr10	78004715	78010540
chr10	78007701	78010540
chr10	78009536	78010540
chr10	78035351	78035720
chr10	78035351	78037304
chr10	79118914	79162133
chr10	7963919	7977323
chr10	79804484	79814716
chr10	80280172	80280792
chr10	80280172	80284038
chr10	80425865	80427496
chr10	80507227	80509471
chr10	825064	825870
chr10	829384	845055
chr10	836402	845055
chr10	836402	864270
chr10	836402	885760
chr10	842931	885760
chr10	84371013	84373815
chr10	84371013	84425893
chr10	84371013	84477664
chr10	84417770	84425893

chr10	84417770	84438707
chr10	84417770	84464016
chr10	84417770	84477664
chr10	84425730	84477664
chr10	84438511	84477664
chr10	84463932	84477664
chr10	863742	864270
chr10	863742	885760
chr10	86443274	86446449
chr10	86451966	86453831
chr10	86451966	86461287
chr10	86451966	86473973
chr10	86458988	86473973
chr10	86461175	86473973
chr10	86467278	86473973
chr10	86470991	86473973
chr10	86472207	86473973
chr10	86472207	86500743
chr10	86838864	86876085
chr10	86838864	86900126
chr10	86875866	86892229
chr10	86875866	86900126
chr10	86875866	86917326
chr10	86890061	86892229
chr10	86890061	86900126
chr10	86912239	86917326
chr10	87053341	87062835
chr10	87057690	87062835
chr10	87057690	87068157
chr10	87059149	87068157
chr10	87060687	87062835
chr10	87062655	87076656
chr10	87068062	87076656
chr10	87151349	87158155
chr10	87170477	87170974
chr10	87170477	87187200
chr10	87508335	87521169
chr10	87709195	87727489
chr10	87756788	87776427
chr10	87767672	87776427
chr10	87767672	87784670
chr10	87770951	87776427
chr10	87776320	87814612
chr10	87784469	87790430
chr10	87784469	87814612

chr10	87925512	87933251
chr10	87925512	87952259
chr10	87925512	87961118
chr10	87931045	87952259
chr10	87942610	87952259
chr10	884446	885760
chr10	88901655	88905660
chr10	88913100	88922436
chr10	89714046	89717722
chr10	89714046	89725158
chr10	89714954	89717495
chr10	89714954	89727896
chr10	89716435	89725158
chr10	89718709	89725158
chr10	89718709	89726521
chr10	89758705	89760636
chr10	89758705	89768888
chr10	90902242	90903276
chr10	90917731	90918972
chr10	91240483	91251440
chr10	91240483	91264520
chr10	91425280	91462184
chr10	91812982	91820033
chr10	91812982	91822362
chr10	91827016	91842391
chr10	91830922	91836998
chr10	91830922	91848635
chr10	91836918	91842391
chr10	91840560	91842391
chr10	91844918	91855126
chr10	91849511	91851336
chr10	91939951	91942568
chr10	91942421	91956657
chr10	91951402	91953873
chr10	91951402	91956657
chr10	91953736	91964201
chr10	91980453	91984404
chr10	91992118	91997751
chr10	91992118	92008275
chr10	91994534	91997751
chr10	91994534	92008275
chr10	91996368	91997751
chr10	91996368	92008275
chr10	92008122	92011415
chr10	92008828	92011415

chr10	92016339	92018935
chr10	92081319	92091944
chr10	92111075	92192636
chr10	92143028	92145112
chr10	92143028	92181019
chr10	92143028	92192636
chr10	92483254	92508205
chr10	92483254	92508890
chr10	92483254	92537550
chr10	92504793	92508205
chr10	92504793	92537550
chr10	92510049	92537550
chr10	92531747	92534785
chr10	92531747	92537550
chr10	92534577	92537550
chr10	92606264	92607237
chr10	92606264	92609509
chr10	92616736	92630364
chr10	92628807	92630364
chr10	92632485	92633795
chr10	92637183	92639900
chr10	92637183	92645642
chr10	92639793	92645642
chr10	92639793	92650517
chr10	92648211	92649986
chr10	92648211	92650517
chr10	92649834	92650517
chr10	92893348	92899644
chr10	92893348	92909631
chr10	92893348	92920050
chr10	92909426	92915913
chr10	92909426	92920050
chr10	92934143	92940824
chr10	93310019	93310643
chr10	93313019	93316813
chr10	93347616	93349969
chr10	93355627	93369776
chr10	93359832	93373085
chr10	93359832	93374955
chr10	93363960	93369776
chr10	93366391	93377429
chr10	93381218	93389154
chr10	93396141	93404219
chr10	93408786	93409739
chr10	93408786	93426158

chr10	93426070	93456937
chr10	93500039	93507056
chr10	93515404	93519807
chr10	93676652	93687449
chr10	93684055	93687449
chr10	94258799	94259150
chr10	94349249	94355093
chr10	94350112	94352403
chr10	94350112	94358215
chr10	94352309	94355093
chr10	94356534	94358215
chr10	94441895	94474783
chr10	94441895	94497172
chr10	94441895	94511654
chr10	94441895	94522453
chr10	94474667	94497172
chr10	94474667	94500327
chr10	94474667	94507347
chr10	94474667	94510179
chr10	94474667	94511654
chr10	94474667	94522453
chr10	94493364	94500327
chr10	94493364	94507347
chr10	94493364	94511654
chr10	94497054	94500327
chr10	94497054	94511654
chr10	94500220	94511654
chr10	94500220	94522083
chr10	94500220	94522453
chr10	94507266	94511654
chr10	94507266	94522453
chr10	94510090	94511654
chr10	94510090	94522453
chr10	94510090	94531460
chr10	94511582	94522453
chr10	94554125	94571429
chr10	94574553	94581522
chr10	94588228	94594854
chr10	94594694	94597111
chr10	95625361	95628492
chr10	95632958	95637197
chr10	95632958	95643206
chr10	95637092	95643206
chr10	95680471	95693476
chr10	96044352	96050466

chr10	96132585	96156047
chr10	96544075	96551411
chr10	96544075	96565426
chr10	96544075	96576829
chr10	96552927	96576829
chr10	96559658	96576829
chr10	96907264	96944245
chr10	96907264	96949295
chr10	96907264	96952196
chr10	96944112	96952196
chr10	96949007	96952196
chr10	97229146	97266125
chr10	97235216	97264906
chr10	97243967	97246337
chr10	97243967	97263629
chr10	97246271	97259628
chr10	97259401	97266125
chr10	97263419	97266125
chr10	97385165	97388624
chr10	97388251	97388624
chr10	97390422	97393760
chr10	97460694	97464013
chr10	97476682	97478389
chr10	97656284	97656974
chr10	97656284	97662968
chr10	97656284	97664984
chr10	97743093	97744915
chr10	98156092	98163397
chr10	98156092	98209899
chr10	98162882	98186563
chr10	98186466	98209899
chr10	98231511	98236164
chr10	992488	1001013
chr10	992488	999095
chr10	99402579	99420805
chr10	995928	1005907
chr10	99661445	99696122
chr10	99679260	99679875
chr10	99716347	99727563
chr10	99888824	99896397
chr10	99894945	99898285
chr10	99894945	99898760
chr10	99894945	99900066
chr10	99894945	99909146
chr10	99898085	99900066

chr10	999002	1005907
chr10	999002	1007128
chr10	99907994	99909146
chr10	99969114	99972134
chr11	1000431	1003804
chr11	10028451	10031170
chr11	10028451	10042981
chr11	100941783	100949956
chr11	102114143	102186131
chr11	102162455	102206074
chr11	102186017	102206074
chr11	102186017	102206086
chr11	102186017	102209564
chr11	102186017	102223752
chr11	102186017	102227581
chr11	102205892	102209564
chr11	102205892	102223752
chr11	102350843	102368548
chr11	102362895	102368548
chr11	103064274	103082839
chr11	103115176	103121496
chr11	103116569	103121496
chr11	103158676	103166048
chr11	103188496	103198063
chr11	103215720	103223086
chr11	103219914	103223086
chr11	103220622	103223086
chr11	103222029	103223086
chr11	103222029	103253448
chr11	103243692	103304720
chr11	103253284	103259977
chr11	103255414	103259977
chr11	103255414	103286386
chr11	103255414	103287605
chr11	103259887	103307831
chr11	103280347	103283085
chr11	103280347	103287605
chr11	103280347	103307831
chr11	103280347	103316620
chr11	103280347	103323990
chr11	103280347	103358359
chr11	103280347	103399872
chr11	103280347	103436032
chr11	103282178	103307831
chr11	103303092	103312033

chr11	103303092	103316620
chr11	103303092	103323990
chr11	103303092	103358359
chr11	103303092	103399872
chr11	103311877	103323990
chr11	103311877	103358359
chr11	103316544	103323990
chr11	103321028	103323990
chr11	103358242	103468705
chr11	103399662	103456356
chr11	103399662	103468705
chr11	103435942	103468705
chr11	103455185	103456356
chr11	103455185	103468705
chr11	10478525	10485039
chr11	10519033	10534205
chr11	106079466	106091477
chr11	107329917	107353736
chr11	107348936	107353736
chr11	107348936	107392895
chr11	107390073	107392895
chr11	107390073	107418287
chr11	107390073	107433749
chr11	107416208	107418287
chr11	107428798	107443049
chr11	107433633	107439183
chr11	107433633	107455776
chr11	107510886	107522555
chr11	107510886	107532406
chr11	107522298	107532406
chr11	107618104	107656066
chr11	10761946	10763879
chr11	107625396	107635765
chr11	107630416	107650403
chr11	107630416	107656066
chr11	10763430	10763879
chr11	107803000	107806876
chr11	107803000	107815965
chr11	107806716	107811794
chr11	107806716	107815965
chr11	10800730	10801087
chr11	10800730	10802209
chr11	10803029	10806047
chr11	10803210	10804050
chr11	108046269	108052801

chr11	108070095	108073497
chr11	108070095	108078240
chr11	108078175	108089623
chr11	108088526	108089623
chr11	108088526	108098529
chr11	108094390	108095691
chr11	108094390	108098529
chr11	108094811	108095691
chr11	108131906	108134316
chr11	108131906	108135242
chr11	108131906	108139041
chr11	108133819	108135242
chr11	108138897	108140215
chr11	108140064	108142550
chr11	108140064	108146359
chr11	108169743	108173851
chr11	108169743	108188179
chr11	108176245	108177090
chr11	108185231	108186569
chr11	108185231	108190500
chr11	108185402	108189330
chr11	108186481	108189330
chr11	108186481	108190500
chr11	108192117	108197420
chr11	108227594	108229323
chr11	108227594	108235834
chr11	108227594	108244118
chr11	108227594	108267342
chr11	108227594	108272852
chr11	108227775	108229323
chr11	108227775	108235834
chr11	108235669	108245026
chr11	108235669	108249102
chr11	108235669	108259075
chr11	108248932	108259075
chr11	108256214	108259075
chr11	108256214	108267342
chr11	108258985	108267342
chr11	108267170	108268609
chr11	108267170	108271406
chr11	108267170	108279608
chr11	108271063	108307984
chr11	108279490	108284473
chr11	108282709	108284473
chr11	108293312	108297382

chr11	108297286	108304852
chr11	108297286	108317521
chr11	108297286	108332037
chr11	108301647	108304852
chr11	108302852	108315911
chr11	108307896	108317521
chr11	108312410	108327758
chr11	108312410	108335961
chr11	108315822	108335961
chr11	108315822	108354874
chr11	108317372	108327758
chr11	108317372	108329238
chr11	108317372	108332037
chr11	108319953	108321420
chr11	108327644	108331557
chr11	108327644	108332037
chr11	108329020	108332037
chr11	108330213	108335961
chr11	108332761	108347365
chr11	108477606	108491167
chr11	108486156	108491167
chr11	108673466	108679560
chr11	108673466	108693600
chr11	108673466	108706837
chr11	108673466	108723462
chr11	108677084	108678435
chr11	108688935	108693600
chr11	108688935	108723462
chr11	108715878	108723462
chr11	108719796	108723462
chr11	108838445	108841476
chr11	110136662	110153058
chr11	110136662	110159490
chr11	110136662	110163379
chr11	110233236	110237652
chr11	110236098	110237652
chr11	110247702	110279756
chr11	110255288	110272619
chr11	110263959	110264874
chr11	110272535	110279756
chr11	110435833	110457047
chr11	111616242	111688162
chr11	111700885	111705139
chr11	111752158	111755450
chr11	111754498	111755450

chr11	111754498	111759951
chr11	111836164	111838378
chr11	111836164	111840809
chr11	111836164	111857737
chr11	111844600	111857737
chr11	111844600	111865251
chr11	112028514	112033530
chr11	112033403	112051349
chr11	112037272	112039397
chr11	112037272	112045230
chr11	112037272	112051349
chr11	112043465	112051349
chr11	112045137	112051349
chr11	112045137	112061174
chr11	112078214	112080558
chr11	112199698	112202190
chr11	11327085	11341004
chr11	113338773	113352207
chr11	113741693	113744040
chr11	113758553	113760918
chr11	113760208	113760918
chr11	113808297	113829345
chr11	113817657	113831993
chr11	113829196	113854335
chr11	113833419	113854335
chr11	113840597	113854335
chr11	113852500	113854335
chr11	11432620	11448936
chr11	116757145	116758407
chr11	116759073	116765446
chr11	116760734	116765446
chr11	116784854	116787643
chr11	116863667	116873636
chr11	116927218	116957064
chr11	117152440	117161261
chr11	117152440	117163892
chr11	117159933	117161261
chr11	117159933	117163892
chr11	117181415	117182806
chr11	117234266	117246948
chr11	117279907	117280259
chr11	117335604	117338668
chr11	117335604	117344277
chr11	117335604	117351988
chr11	118374895	118376694

chr11	118468774	118480238
chr11	118480173	118482495
chr11	118481714	118482495
chr11	118481714	118484975
chr11	118497935	118499420
chr11	118507528	118512025
chr11	118509135	118512025
chr11	118554458	118559864
chr11	118556905	118559864
chr11	118581245	118584014
chr11	118581245	118584644
chr11	118583178	118584644
chr11	118583808	118584644
chr11	118754704	118758902
chr11	118754704	118763306
chr11	118756259	118758902
chr11	118758773	118760044
chr11	118758773	118763306
chr11	118758773	118765355
chr11	118759921	118763306
chr11	118759921	118768352
chr11	118765208	118768352
chr11	118765208	118786518
chr11	118768222	118781184
chr11	118768222	118786518
chr11	118779631	118786518
chr11	118781120	118786518
chr11	11880176	11897693
chr11	11880176	11903342
chr11	119020149	119021886
chr11	119070233	119071843
chr11	119123567	119127125
chr11	119131534	119133219
chr11	119179692	119182345
chr11	11920341	11933935
chr11	119271734	119274953
chr11	119271734	119276134
chr11	119271734	119278713
chr11	119271734	119287946
chr11	119273867	119274953
chr11	119273867	119276134
chr11	119273867	119278713
chr11	119273867	119285566
chr11	119275996	119297481
chr11	119284968	119285566

chr11	119296917	119297481
chr11	119311089	119314749
chr11	119675158	119678765
chr11	120406117	120424415
chr11	120406117	120431911
chr11	120406117	120437382
chr11	120406117	120440221
chr11	120420752	120437382
chr11	120431770	120437382
chr11	120440128	120445464
chr11	120440128	120447085
chr11	120447873	120475507
chr11	120448233	120449214
chr11	120457117	120459320
chr11	120457720	120467308
chr11	120457720	120473127
chr11	120457720	120475507
chr11	120465236	120467308
chr11	120465236	120473127
chr11	120473049	120477526
chr11	120474559	120477526
chr11	120475339	120477526
chr11	120476660	120477526
chr11	121303365	121304493
chr11	12138389	12162419
chr11	121488031	121497049
chr11	121520656	121532552
chr11	121532463	121559657
chr11	12162078	12216319
chr11	12204249	12208139
chr11	12226170	12227131
chr11	12236176	12239585
chr11	122776218	122777210
chr11	122776218	122789308
chr11	122798941	122806516
chr11	123083084	123084713
chr11	123594081	123594838
chr11	123594081	123596037
chr11	123594081	123619224
chr11	123608658	123610338
chr11	123728132	123730985
chr11	124624930	124631417
chr11	124647364	124648175
chr11	124654654	124669521
chr11	12473744	12477949

chr11	12473744	12496598
chr11	12477846	12514065
chr11	124975095	124988134
chr11	12504313	12514065
chr11	125572457	125575408
chr11	125575262	125576315
chr11	125575262	125579068
chr11	125577503	125580204
chr11	125595880	125608289
chr11	125597058	125611519
chr11	125602302	125604247
chr11	125602302	125608289
chr11	125605628	125608289
chr11	125629231	125633351
chr11	125643791	125644643
chr11	125983871	125989759
chr11	126021247	126023537
chr11	126272968	126273454
chr11	12675408	12764434
chr11	12764178	12902113
chr11	128480190	128490576
chr11	128484822	128490576
chr11	128489289	128490576
chr11	1286001	1288776
chr11	1286001	1290409
chr11	1286001	1295794
chr11	12862249	12883125
chr11	128758114	128768272
chr11	128758114	128772985
chr11	128758114	128782023
chr11	128768117	128782023
chr11	128781957	128809204
chr11	12879707	12902113
chr11	12881004	12902113
chr11	1288623	1290409
chr11	1288623	1295794
chr11	12901939	12930326
chr11	129040927	129066868
chr11	129040927	129164427
chr11	129062279	129066868
chr11	129062279	129124894
chr11	129063901	129066868
chr11	129093620	129124894
chr11	129093620	129164427
chr11	129123445	129124894

chr11	129123445	129164427
chr11	129124802	129164427
chr11	129923247	129925229
chr11	129937597	129947370
chr11	129942425	129961082
chr11	129955511	129961082
chr11	130109428	130110661
chr11	130109428	130122513
chr11	130120705	130122513
chr11	130126699	130133728
chr11	130126699	130135715
chr11	130126699	130137291
chr11	130903254	130906721
chr11	13403167	13445181
chr11	13413529	13421838
chr11	13413529	13445181
chr11	134159891	134177457
chr11	13417157	13445181
chr11	134176306	134194738
chr11	134184636	134185526
chr11	134194015	134194738
chr11	134206598	134209477
chr11	134256547	134257257
chr11	14294470	14295855
chr11	14474494	14498985
chr11	14486366	14494439
chr11	14488491	14494439
chr11	14513569	14519041
chr11	14771936	14789242
chr11	14771936	14804050
chr11	14789105	14819209
chr11	14818182	14819209
chr11	1557793	1559055
chr11	17091995	17092276
chr11	17094260	17099969
chr11	17097056	17099969
chr11	17097056	17112666
chr11	17099859	17112666
chr11	17102661	17117671
chr11	17131915	17136625
chr11	17134818	17136625
chr11	17145667	17150655
chr11	17145667	17155629
chr11	17878207	18008076
chr11	17959469	18008076

chr11	17988596	17995909
chr11	17988596	18004535
chr11	17988596	18008076
chr11	18000496	18008076
chr11	18083534	18089510
chr11	18083534	18096854
chr11	18086865	18089510
chr11	18281949	18297717
chr11	18283794	18285459
chr11	18283794	18286710
chr11	18291441	18292976
chr11	18295019	18297717
chr11	18333059	18341607
chr11	18339563	18341607
chr11	18347587	18347919
chr11	18399430	18403811
chr11	18399430	18405572
chr11	18400836	18403811
chr11	18402839	18403811
chr11	18412708	18429910
chr11	18434739	18438645
chr11	18502485	18519603
chr11	18506856	18509665
chr11	18514677	18516164
chr11	18514677	18519603
chr11	18544622	18558330
chr11	18544622	18565010
chr11	18546879	18558330
chr11	18546879	18570377
chr11	18564891	18578808
chr11	18611476	18612488
chr11	19142977	19152320
chr11	19155795	19166385
chr11	19225220	19235058
chr11	19832483	19880127
chr11	19832483	19892594
chr11	19868924	19892594
chr11	19892433	19939773
chr11	19939660	19984247
chr11	20043980	20045670
chr11	20043980	20055957
chr11	20054079	20062359
chr11	20054079	20083179
chr11	20062306	20083179
chr11	20367173	20376717

chr11	20388018	20402984
chr11	20388018	20408032
chr11	20389743	20402984
chr11	20389743	20408032
chr11	20392210	20397721
chr11	20392210	20402984
chr11	20395802	20397721
chr11	20395802	20408032
chr11	20395802	20426865
chr11	20402918	20408032
chr11	20402918	20426865
chr11	20402918	20464546
chr11	20402918	20494254
chr11	20407910	20452208
chr11	20407910	20464546
chr11	20407910	20494254
chr11	20452129	20464546
chr11	20461979	20494254
chr11	224077	233215
chr11	22674849	22685789
chr11	22674849	22737768
chr11	230451	233534
chr11	238997	244761
chr11	244040	248855
chr11	244160	247448
chr11	2570627	2588854
chr11	2587569	2588854
chr11	28058925	28084808
chr11	28058925	28091513
chr11	28062394	28091513
chr11	28077006	28091513
chr11	28077006	28094800
chr11	28211061	28296931
chr11	2949226	2959909
chr11	2949226	2979237
chr11	2951258	2959909
chr11	2951258	2979237
chr11	2951779	2958544
chr11	2955743	2979237
chr11	2969802	2972243
chr11	2969802	2979237
chr11	2976023	2979237
chr11	30330885	30332993
chr11	3122347	3129169
chr11	31456259	31473786

chr11	31460625	31463305
chr11	31627109	31650221
chr11	32097143	32098528
chr11	32587011	32589135
chr11	32587011	32589641
chr11	32587011	32601822
chr11	32588593	32589135
chr11	32588593	32589641
chr11	32589546	32595013
chr11	32589546	32596047
chr11	32927156	32928123
chr11	32927156	32958086
chr11	32953856	32973549
chr11	32957868	32973549
chr11	32966299	32973549
chr11	33027685	33028792
chr11	33043749	33054725
chr11	33043749	33058139
chr11	33043749	33059095
chr11	33102176	33106064
chr11	33105566	33106064
chr11	33141666	33141986
chr11	33286412	33328633
chr11	33286412	33341686
chr11	33328509	33341686
chr11	33337074	33341686
chr11	33338756	33341686
chr11	33347292	33349287
chr11	33551039	33552241
chr11	33559748	33598947
chr11	33583337	33598947
chr11	33583337	33618662
chr11	33583337	33661014
chr11	33591236	33618662
chr11	33618532	33646036
chr11	34071725	34076642
chr11	34071725	34090678
chr11	34076559	34082877
chr11	34076559	34083041
chr11	34079627	34083041
chr11	34079627	34092056
chr11	34082824	34089456
chr11	34086063	34086413
chr11	34086063	34090678
chr11	34086063	34092056

chr11	34086304	34090678
chr11	34089394	34090678
chr11	34089394	34092056
chr11	34090178	34090678
chr11	34090178	34092056
chr11	34108210	34127599
chr11	34108210	34132221
chr11	34113715	34118503
chr11	34113715	34127599
chr11	34115822	34118503
chr11	34122458	34124400
chr11	34134318	34135291
chr11	34197324	34204690
chr11	34453089	34456817
chr11	34931403	34960518
chr11	34931403	34966814
chr11	34931403	34970286
chr11	34931403	34992379
chr11	34947505	34970286
chr11	34947505	34978182
chr11	34957383	34960518
chr11	34957383	34970286
chr11	34957383	34978182
chr11	34957383	34984728
chr11	34957383	34992379
chr11	34970138	34984728
chr11	34978123	34984728
chr11	35176574	35186900
chr11	35189834	35219387
chr11	35196745	35211449
chr11	35201081	35201787
chr11	35204511	35206243
chr11	35211245	35221732
chr11	36081652	36098461
chr11	36494975	36501537
chr11	36610080	36636117
chr11	36610080	36648155
chr11	36633283	36648155
chr11	3679553	3686194
chr11	3683199	3691489
chr11	3691346	3693375
chr11	3691346	3695606
chr11	3693231	3695606
chr11	3695448	3700839
chr11	3695448	3702892

chr11	3695448	3720825
chr11	3699081	3702892
chr11	3702462	3706627
chr11	3705199	3706627
chr11	3705199	3753408
chr11	3713817	3720825
chr11	3713817	3725219
chr11	3713817	3744649
chr11	3719411	3720825
chr11	3719411	3725219
chr11	3719411	3753408
chr11	3731390	3735324
chr11	3731390	3744649
chr11	3731390	3753408
chr11	3731390	3763039
chr11	3735190	3753408
chr11	3744508	3753408
chr11	3744508	3760626
chr11	3744508	3763039
chr11	3753315	3768744
chr11	375638	376351
chr11	3762901	3776021
chr11	3762901	3782145
chr11	3768580	3771928
chr11	3768580	3776021
chr11	3768580	3779257
chr11	3768580	3782145
chr11	3773631	3776021
chr11	3778872	3782145
chr11	3967551	4023987
chr11	4055525	4070203
chr11	4055525	4074679
chr11	4070025	4086543
chr11	4101992	4106223
chr11	4101992	4109703
chr11	4101992	4112062
chr11	4106045	4109703
chr11	4106045	4112062
chr11	4107434	4112062
chr11	4107434	4126833
chr11	4121603	4123384
chr11	4127034	4129150
chr11	43318639	43330564
chr11	43326506	43328893
chr11	43326506	43335994

chr11	43327788	43335994
chr11	43379232	43389821
chr11	43389651	43398113
chr11	43389651	43414776
chr11	43391464	43398113
chr11	43403997	43407577
chr11	43414589	43436334
chr11	43448001	43451265
chr11	43750910	43798427
chr11	43798319	43840064
chr11	43815436	43840064
chr11	43830975	43840064
chr11	43882582	43892129
chr11	44107682	44109283
chr11	44107682	44114301
chr11	44107682	44126955
chr11	44107682	44130138
chr11	44107682	44171742
chr11	44114184	44130138
chr11	44114184	44171742
chr11	44124788	44130138
chr11	44935560	44938366
chr11	45181760	45204778
chr11	45858730	45861032
chr11	45862059	45872233
chr11	45903988	45905249
chr11	45913818	45916303
chr11	45965314	45979966
chr11	46076753	46084302
chr11	46076753	46092223
chr11	46084165	46092223
chr11	46408512	46512813
chr11	46433473	46435037
chr11	46433473	46443598
chr11	46433473	46508370
chr11	46493607	46508370
chr11	46493607	46512813
chr11	46508190	46512813
chr11	46541944	46548500
chr11	46547112	46548500
chr11	46688172	46696156
chr11	46750273	46751534
chr11	46752634	46755067
chr11	46754887	46760784
chr11	46754887	46788773

chr11	46758922	46760784
chr11	46760611	46763179
chr11	46769962	46788773
chr11	46776254	46788773
chr11	46776254	46790583
chr11	46778459	46788773
chr11	46778459	46790583
chr11	46780193	46788773
chr11	46783273	46788773
chr11	46783273	46790583
chr11	46788680	46790583
chr11	46790075	46790583
chr11	46795593	46809874
chr11	46797804	46809874
chr11	46801199	46809874
chr11	46808030	46809874
chr11	46808030	46811178
chr11	46808030	46821268
chr11	46809399	46811178
chr11	46809399	46821268
chr11	46809741	46821268
chr11	46811006	46816404
chr11	46811006	46821268
chr11	46816197	46821268
chr11	46987207	47052518
chr11	47166267	47168251
chr11	47420263	47420727
chr11	47422573	47422973
chr11	47424045	47425950
chr11	47472190	47483532
chr11	47472190	47489024
chr11	47482694	47484523
chr11	47482694	47489024
chr11	47483452	47484523
chr11	47484388	47489024
chr11	47486749	47500932
chr11	47499452	47500932
chr11	47625673	47631711
chr11	47627079	47631087
chr11	47627079	47635571
chr11	47627079	47639051
chr11	47628952	47635571
chr11	47638698	47639051
chr11	47722975	47724172
chr11	47724027	47731561

chr11	47731373	47734129
chr11	47731373	47736740
chr11	47734024	47736740
chr11	47750915	47751290
chr11	47750915	47765362
chr11	47752915	47754664
chr11	47783072	47786554
chr11	47783072	47788611
chr11	47783072	47792946
chr11	47797778	47801930
chr11	47803437	47808529
chr11	47815478	47819458
chr11	47835650	47837623
chr11	48142918	48150183
chr11	48144999	48160049
chr11	48153795	48156119
chr11	48159929	48164515
chr11	497970	502181
chr11	57414614	57421373
chr11	57491223	57491862
chr11	57672020	57693915
chr11	57692610	57696043
chr11	57692610	57696873
chr11	57794009	57796992
chr11	57795576	57802196
chr11	581491	582081
chr11	581491	587464
chr11	581491	592674
chr11	58584855	58611738
chr11	58609829	58616815
chr11	58610949	58611738
chr11	592558	598502
chr11	59600495	59610589
chr11	59608483	59610589
chr11	59639047	59639383
chr11	59639047	59643035
chr11	59649461	59651641
chr11	59658865	59659469
chr11	596922	598502
chr11	59773210	59788947
chr11	59787036	59797396
chr11	59788872	59795482
chr11	60897845	60903861
chr11	60919685	60920988
chr11	61303864	61312084

chr11	61311783	61314486
chr11	61311783	61331691
chr11	61321594	61323094
chr11	61323010	61331691
chr11	61323978	61326893
chr11	61326778	61330074
chr11	61329362	61330074
chr11	61334619	61342844
chr11	61334619	61345946
chr11	61356529	61357828
chr11	61364251	61366216
chr11	61366044	61367998
chr11	61410937	61411937
chr11	61410937	61416519
chr11	61410937	61429290
chr11	61411768	61429290
chr11	61415665	61416519
chr11	61415665	61429290
chr11	61420469	61429290
chr11	61437624	61438113
chr11	61527914	61533124
chr11	61806663	61811075
chr11	61810750	61813353
chr11	62128150	62128883
chr11	62128150	62130590
chr11	62128150	62137883
chr11	6222441	6224707
chr11	62535002	62536567
chr11	62535944	62536567
chr11	62566805	62567528
chr11	62581105	62581701
chr11	62595263	62595891
chr11	62605112	62607099
chr11	62606957	62611596
chr11	62628768	62629295
chr11	62633004	62639731
chr11	62638982	62639731
chr11	62663235	62664973
chr11	62694567	62702549
chr11	62720022	62724426
chr11	62723586	62724426
chr11	62781893	62782825
chr11	62801093	62802260
chr11	62827154	62827631
chr11	62839113	62839428

chr11	62839113	62839659
chr11	62852810	62854938
chr11	62853551	62855466
chr11	62853800	62854938
chr11	62853800	62855216
chr11	62870744	62871669
chr11	62881018	62882066
chr11	62881892	62885608
chr11	62881892	62888218
chr11	62882907	62885608
chr11	62882907	62888218
chr11	63590099	63598163
chr11	63631039	63644261
chr11	63643356	63646563
chr11	63644168	63659252
chr11	63651935	63652575
chr11	63658760	63659252
chr11	63895158	63895633
chr11	63952237	63952839
chr11	6400988	6403822
chr11	64151238	64152393
chr11	64193077	64194620
chr11	64193077	64197974
chr11	64193077	64203622
chr11	64194188	64197397
chr11	64194188	64197974
chr11	64197270	64197974
chr11	64264950	64265502
chr11	64685645	64690476
chr11	64757778	64758704
chr11	64766899	64767251
chr11	64766899	64776626
chr11	64769222	64776626
chr11	64927801	64928425
chr11	64930321	64930589
chr11	65034846	65035663
chr11	65187954	65210872
chr11	6519599	6520102
chr11	65266581	65268729
chr11	65266581	65275998
chr11	65266581	65282521
chr11	65275891	65282521
chr11	65287715	65295990
chr11	65294152	65295990
chr11	65340384	65346359

chr11	65389360	65394425
chr11	65393572	65394425
chr11	653961	688460
chr11	65605760	65608448
chr11	65635528	65636307
chr11	65645853	65647658
chr11	65649756	65650051
chr11	65655687	65658499
chr11	65655687	65662205
chr11	65661686	65662205
chr11	65861946	65865894
chr11	65862210	65865145
chr11	65864496	65865145
chr11	65894013	65897006
chr11	65920342	65920662
chr11	66059200	66059595
chr11	66063627	66068333
chr11	66210361	66211259
chr11	66263659	66263952
chr11	66271796	66275935
chr11	66365935	66366564
chr11	66365935	66367869
chr11	66365935	66369532
chr11	66486539	66487342
chr11	66486539	66488007
chr11	66521269	66526807
chr11	66599115	66600549
chr11	66748378	66759096
chr11	66748378	66788269
chr11	66748378	66828364
chr11	66748378	66838445
chr11	66796280	66828364
chr11	66803994	66822674
chr11	66816052	66822674
chr11	66816052	66828364
chr11	66816052	66838445
chr11	66821642	66822674
chr11	66821642	66828364
chr11	66871314	66872159
chr11	67180078	67181892
chr11	67180078	67219403
chr11	67180078	67243092
chr11	67181845	67219403
chr11	67207509	67219403
chr11	67207509	67231960

chr11	67215339	67215949
chr11	67215339	67217884
chr11	67215339	67219403
chr11	67215339	67228163
chr11	67215339	67231960
chr11	67217730	67228163
chr11	67217730	67231960
chr11	67281649	67282365
chr11	67283706	67285188
chr11	67291475	67292180
chr11	67393495	67393790
chr11	674535	688460
chr11	67497916	67498322
chr11	67608395	67609635
chr11	67608395	67611207
chr11	67608551	67609635
chr11	678693	681089
chr11	679687	681089
chr11	68032151	68033283
chr11	68061952	68068937
chr11	68061952	68070857
chr11	68061952	68081457
chr11	68065785	68081457
chr11	68066428	68074830
chr11	68068878	68097130
chr11	68070188	68081457
chr11	68081403	68097130
chr11	68166981	68185928
chr11	68166981	68190152
chr11	68171542	68180200
chr11	68175017	68185928
chr11	68180131	68190152
chr11	68185780	68190152
chr11	68347846	68357847
chr11	68347846	68365702
chr11	68347846	68390052
chr11	68357649	68363943
chr11	68357649	68365702
chr11	68357649	68429700
chr11	68363746	68365702
chr11	68386315	68390052
chr11	68403482	68406813
chr11	68403482	68416527
chr11	68403482	68426187
chr11	68406523	68416527

chr11	68423488	68426187
chr11	68423488	68429700
chr11	68423488	68433838
chr11	68429574	68433838
chr11	684897	688460
chr11	68505144	68519651
chr11	68505144	68537891
chr11	68505144	68548204
chr11	68505144	68603492
chr11	68519500	68537891
chr11	68519500	68548204
chr11	68519500	68551186
chr11	68519500	68554257
chr11	68519500	68558679
chr11	68519500	68583129
chr11	68537658	68551186
chr11	68537658	68554257
chr11	68548066	68564432
chr11	68548066	68571104
chr11	68548066	68574224
chr11	68551120	68564432
chr11	68554144	68583129
chr11	68558565	68596218
chr11	68564302	68576043
chr11	68564302	68603492
chr11	68567013	68571104
chr11	68567013	68576043
chr11	68567013	68583129
chr11	68567013	68596218
chr11	68569747	68576043
chr11	68569747	68596218
chr11	68571039	68603492
chr11	68574108	68583129
chr11	68574108	68596218
chr11	68583042	68596218
chr11	68590659	68603492
chr11	68596096	68603492
chr11	68600340	68603492
chr11	68601862	68603492
chr11	68759568	68761687
chr11	68780639	68785010
chr11	68780639	68793402
chr11	68780639	68794911
chr11	68793314	68796933
chr11	68793314	68807638

chr11	68793314	68812576
chr11	68794803	68799355
chr11	68796855	68812576
chr11	68799217	68807638
chr11	68803999	68807638
chr11	68803999	68812576
chr11	68807466	68812576
chr11	69054032	69057694
chr11	69054032	69062990
chr11	69067502	69079020
chr11	6932093	6943641
chr11	6941570	6943641
chr11	69667894	69673321
chr11	70324401	70326818
chr11	70330172	70335694
chr11	70330172	70339306
chr11	70330172	70343892
chr11	70333469	70343892
chr11	70343668	70348420
chr11	70348188	70356254
chr11	70350993	70356254
chr11	70354300	70356254
chr11	70354300	70362488
chr11	70362094	70376600
chr11	70376531	70378195
chr11	70405264	70407591
chr11	70405264	70409960
chr11	70405264	70414652
chr11	70405264	70417123
chr11	70405264	70419856
chr11	70405264	70420510
chr11	70405264	70422995
chr11	70409830	70414652
chr11	70409830	70420510
chr11	70417012	70419856
chr11	70417012	70420510
chr11	70419745	70420510
chr11	70422939	70431280
chr11	70659827	70661678
chr11	70659827	70698763
chr11	70798442	70820682
chr11	71109940	71119032
chr11	71147119	71224796
chr11	71464052	71473366
chr11	71916073	71919026

chr11	71982760	71987111
chr11	71982760	71990717
chr11	71999926	72001552
chr11	72006034	72007435
chr11	72098288	72099256
chr11	72110087	72111496
chr11	72307198	72380384
chr11	72380280	72408204
chr11	72707174	72714321
chr11	72709869	72711141
chr11	72709869	72711499
chr11	72709869	72714321
chr11	72712195	72714321
chr11	72726619	72732597
chr11	72755700	72759381
chr11	72757766	72759381
chr11	72757766	72781294
chr11	72849754	72889945
chr11	72849754	72902638
chr11	72867864	72889945
chr11	72867864	72902638
chr11	72867864	72921950
chr11	72887469	72889945
chr11	72887469	72902638
chr11	72984087	72989097
chr11	72984087	73001134
chr11	72984087	73015885
chr11	72984087	73083740
chr11	73000989	73015885
chr11	73000989	73083740
chr11	73359833	73361161
chr11	73389111	73391223
chr11	73409486	73411536
chr11	73419873	73430770
chr11	73707419	73716362
chr11	73707419	73718718
chr11	73707419	73718890
chr11	73707419	73720899
chr11	73707419	73730823
chr11	73716250	73720899
chr11	73716250	73730823
chr11	73825707	73844976
chr11	73825707	73860009
chr11	73844806	73860009
chr11	73899145	73900420

chr11	73909398	73919032
chr11	73964877	73968458
chr11	74028286	74034278
chr11	74048204	74057544
chr11	74074252	74078717
chr11	74109033	74118382
chr11	74113779	74139828
chr11	74118227	74133557
chr11	74118227	74161556
chr11	74132843	74133557
chr11	74132843	74139828
chr11	74132843	74161556
chr11	74203727	74204445
chr11	74203727	74222369
chr11	74203727	74239256
chr11	74222311	74239256
chr11	74225204	74239256
chr11	74230244	74239256
chr11	74368491	74374582
chr11	74604691	74613010
chr11	74634582	74636275
chr11	74789625	74810330
chr11	74789625	74817714
chr11	74810183	74817714
chr11	74810183	74834775
chr11	74848114	74852082
chr11	74859157	74863021
chr11	74906238	74907273
chr11	74965033	74969699
chr11	75477618	75490320
chr11	755878	760253
chr11	75797157	75798429
chr11	75851882	75861780
chr11	75851882	75888903
chr11	75851882	75961549
chr11	75861745	75888903
chr11	75879878	75888903
chr11	75879878	75912039
chr11	75879878	75961549
chr11	75911953	75983513
chr11	75961443	75983513
chr11	75961443	76065788
chr11	75983386	76016980
chr11	76007533	76016980
chr11	76007533	76065788

chr11	76451857	76464080
chr11	76458182	76464080
chr11	76458182	76472840
chr11	76463820	76472840
chr11	76496214	76516312
chr11	76496214	76528466
chr11	76513385	76516312
chr11	76513385	76523291
chr11	76513385	76528466
chr11	76523154	76528466
chr11	76958978	76998821
chr11	76959076	76990574
chr11	76959076	76998821
chr11	76976288	76998821
chr11	771332	775142
chr11	77332729	77340763
chr11	77332729	77353599
chr11	77340645	77349287
chr11	77349238	77379994
chr11	77355667	77359017
chr11	77355667	77379994
chr11	77374327	77379994
chr11	77374327	77392541
chr11	77379240	77379994
chr11	77619605	77622673
chr11	77619605	77625818
chr11	77619605	77629899
chr11	77622499	77625818
chr11	77622499	77629899
chr11	77624962	77625818
chr11	77624962	77629899
chr11	77672041	77678153
chr11	77672041	77693611
chr11	77675035	77678153
chr11	77675035	77693611
chr11	77683709	77693611
chr11	77685104	77693611
chr11	77691158	77693611
chr11	77691158	77698693
chr11	77725544	77747128
chr11	77725544	77764689
chr11	77740730	77747128
chr11	77740730	77764689
chr11	77883831	77891840
chr11	77891318	77907810

chr11	77921339	77922471
chr11	77928341	77981576
chr11	77938650	77941251
chr11	77938650	77961138
chr11	77938650	77981576
chr11	77941179	77961138
chr11	77941179	77981576
chr11	77955941	77961138
chr11	77955941	77981576
chr11	77955941	77991299
chr11	77978995	77981576
chr11	77978995	77991299
chr11	78103979	78124214
chr11	78106806	78121174
chr11	78109441	78121174
chr11	78112649	78121174
chr11	78112649	78127436
chr11	78113885	78121174
chr11	78113885	78127436
chr11	78119181	78124214
chr11	78119181	78127436
chr11	78207529	78210744
chr11	78443660	78478683
chr11	78465875	78478683
chr11	78465875	78493195
chr11	78465875	78571444
chr11	78469246	78493195
chr11	78469246	78571444
chr11	78493062	78571444
chr11	78528841	78566272
chr11	78528841	78571444
chr11	78559538	78571444
chr11	78568631	78571444
chr11	8089609	8090231
chr11	821957	824436
chr11	82838386	82860117
chr11	82849048	82860117
chr11	83167124	83171914
chr11	83198478	83211430
chr11	83206057	83211430
chr11	83206057	83227872
chr11	83206057	83236509
chr11	83273646	83278829
chr11	83273646	83280260
chr11	83278725	83280260

chr11	847200	850367
chr11	847200	866001
chr11	85111660	85626753
chr11	85598656	85626753
chr11	85634085	85635778
chr11	85974707	85981228
chr11	85974707	85983973
chr11	85974707	86003451
chr11	85974707	86014963
chr11	85974707	86026367
chr11	85974707	86031611
chr11	85981128	85983973
chr11	85981128	85990399
chr11	85981128	85996929
chr11	85981128	86003451
chr11	85981128	86007583
chr11	85981128	86031611
chr11	85981744	85982003
chr11	85981744	85983973
chr11	85981744	85990399
chr11	85981744	85996929
chr11	85990249	86031611
chr11	85996825	86001158
chr11	85996825	86003451
chr11	85996825	86007583
chr11	85996825	86012392
chr11	85996825	86014963
chr11	85996825	86031611
chr11	86000642	86003451
chr11	86000642	86031611
chr11	86003365	86031611
chr11	86007541	86026367
chr11	86007541	86031611
chr11	86011029	86022469
chr11	86011029	86026367
chr11	86011029	86031611
chr11	86014869	86026367
chr11	86014869	86031611
chr11	86022366	86031611
chr11	86250295	86252240
chr11	86250295	86256512
chr11	86250295	86257596
chr11	86250295	86264263
chr11	86250295	86266216
chr11	86264171	86268561

chr11	86487336	86508867
chr11	86556552	86559823
chr11	87067693	87071615
chr11	87067693	87091395
chr11	87067693	87157406
chr11	8707084	8712735
chr11	87295781	87306005
chr11	8750620	8811335
chr11	88300529	88312554
chr11	88309162	88312554
chr11	8912452	8914930
chr11	8965903	8966656
chr11	8965903	8968041
chr11	90210535	90218184
chr11	90214017	90218184
chr11	9144994	9165967
chr11	9150080	9170777
chr11	9152357	9170777
chr11	9160712	9170777
chr11	9160712	9193681
chr11	9160712	9207632
chr11	9178131	9207632
chr11	9193493	9207632
chr11	9203659	9207632
chr11	9283370	9287806
chr11	9283370	9299701
chr11	9286454	9295387
chr11	9286454	9299701
chr11	9287701	9295387
chr11	9288441	9299701
chr11	93154107	93184520
chr11	93154107	93185814
chr11	93162705	93166016
chr11	93675570	93679552
chr11	93675570	93687865
chr11	93679411	93691775
chr11	93679411	93728821
chr11	93683558	93687865
chr11	93702459	93702919
chr11	93702459	93706897
chr11	93724253	93725831
chr11	93729869	93730148
chr11	93747296	93754037
chr11	93747296	93757465
chr11	93747296	93759858

chr11	93788000	93790793
chr11	93788000	93794035
chr11	93790573	93794035
chr11	93790573	93796540
chr11	93790573	93801972
chr11	93793727	93794035
chr11	93793950	93796540
chr11	93794907	93801972
chr11	93796409	93801972
chr11	93807517	93809876
chr11	9403289	9414411
chr11	9403289	9420698
chr11	9408485	9414411
chr11	9408485	9420698
chr11	9408485	9425262
chr11	9409927	9417148
chr11	9414254	9417148
chr11	9414254	9425262
chr11	9420410	9420698
chr11	9420410	9423876
chr11	9420410	9425262
chr11	9420410	9431003
chr11	9423005	9425262
chr11	9428539	9429196
chr11	9428539	9431003
chr11	9429673	9431003
chr11	9429673	9433846
chr11	9433569	9442197
chr11	9434933	9438285
chr11	9436270	9440661
chr11	9437753	9438285
chr11	9437753	9442197
chr11	9438079	9442197
chr11	9440454	9442197
chr11	94429910	94437235
chr11	94429910	94447438
chr11	94429910	94456338
chr11	94429910	94461036
chr11	94429910	94464239
chr11	94435831	94447438
chr11	94435831	94471759
chr11	94445809	94447438
chr11	94445809	94456338
chr11	94445809	94461036
chr11	94456275	94492906

chr11	94459407	94471759
chr11	94478734	94492906
chr11	94485923	94490965
chr11	94568729	94577492
chr11	94568729	94589232
chr11	94568729	94597915
chr11	94574998	94583569
chr11	94583447	94587247
chr11	94583447	94589232
chr11	94595308	94601979
chr11	9471301	9474633
chr11	94795010	94800311
chr11	94795010	94831541
chr11	94799389	94821821
chr11	94821529	94831541
chr11	94830049	94831541
chr11	94850113	94859715
chr11	94853932	94859715
chr11	9494645	9497800
chr11	9494645	9516362
chr11	95775921	95779926
chr11	95779606	95788563
chr11	95783064	95788563
chr11	95799231	95813111
chr11	95799231	95813589
chr11	95812931	95813589
chr11	95812931	95817903
chr11	95812931	95818904
chr11	95812931	95828027
chr11	95841616	95847899
chr11	95847713	95850749
chr11	95847713	95857635
chr11	95847713	95888261
chr11	959436	972255
chr11	95985530	95991723
chr11	96358992	96383473
chr11	9713465	9729451
chr11	9713465	9732710
chr11	972061	981299
chr11	9724657	9732710
chr11	9738212	9749183
chr11	977094	988689
chr11	9795830	9809002
chr11	9807999	9809002
chr11	9839497	9852749

chr11	9856457	9858396
chr11	992502	1000598
chr11	992502	994245
chr11	9961956	10002689
chr11	9961956	10193987
chr11	9961956	9963882
chr11	9961956	9989595
chr11	9989496	10001022
chr11	9989496	10002689
chr11	9989496	10031170
chr11	9989496	9998379
chr12	100047529	100072813
chr12	100095666	100108548
chr12	100097364	100098541
chr12	100097364	100103951
chr12	100102765	100108548
chr12	100204939	100205744
chr12	100204939	100205757
chr12	100204939	100207786
chr12	100207662	100212528
chr12	100282942	100291660
chr12	100282942	100298175
chr12	100282942	100312653
chr12	100313421	100329319
chr12	100591735	100601753
chr12	100591735	100612141
chr12	100591735	100618587
chr12	100591735	100622382
chr12	100594874	100601753
chr12	100594874	100612141
chr12	100594874	100618587
chr12	100618448	100622382
chr12	101306698	101308343
chr12	101308184	101311798
chr12	101308184	101312276
chr12	101311718	101312276
chr12	101319544	101321629
chr12	101329240	101333444
chr12	101333300	101334504
chr12	101334424	101338957
chr12	101340522	101343093
chr12	101340522	101344750
chr12	101342445	101343093
chr12	101356553	101365625
chr12	101363575	101367976

chr12	101372883	101373767
chr12	101383040	101383669
chr12	101396398	101405981
chr12	101714089	101716812
chr12	101714089	101720254
chr12	101714089	101723847
chr12	101753371	101760143
chr12	101780151	101790057
chr12	101786011	101790057
chr12	101786011	101796762
chr12	101897862	101901433
chr12	101897862	101920201
chr12	101908185	101914232
chr12	102025973	102046119
chr12	102039867	102046119
chr12	102085765	102118583
chr12	102099105	102112232
chr12	102101031	102118583
chr12	102148229	102165883
chr12	102148229	102182627
chr12	1027747	1116033
chr12	1027747	1263165
chr12	1027747	1371977
chr12	1027747	1444750
chr12	103793163	103797031
chr12	103796893	103815121
chr12	103798586	103806929
chr12	103932825	103943319
chr12	103976917	103980962
chr12	103979830	103980962
chr12	103982798	103985728
chr12	104019629	104021491
chr12	104093385	104096433
chr12	104120399	104123423
chr12	104120399	104135532
chr12	104288930	104334332
chr12	104288930	104339273
chr12	104311289	104315896
chr12	104311289	104327671
chr12	104311289	104339273
chr12	104318912	104327671
chr12	104321090	104327671
chr12	104325336	104327671
chr12	104327514	104331641
chr12	104331533	104339273

chr12	104844471	104895345
chr12	104861290	104928694
chr12	104886292	104895345
chr12	104909654	104928694
chr12	105149614	105160148
chr12	105149614	105162845
chr12	105177225	105195501
chr12	105189990	105195501
chr12	106070773	106072843
chr12	106363869	106369683
chr12	106363869	106380139
chr12	106366515	106369683
chr12	106376358	106380139
chr12	106393030	106410960
chr12	106410825	106427358
chr12	106410825	106444590
chr12	106437056	106444590
chr12	106454501	106457296
chr12	106783996	106825820
chr12	106814695	106825820
chr12	106814695	106843947
chr12	106825725	106843947
chr12	106825725	106860412
chr12	106842588	106843947
chr12	106842588	106851594
chr12	106842588	106860412
chr12	106851449	106860412
chr12	106992964	106999862
chr12	106999550	107000082
chr12	10713210	10715783
chr12	10713210	10719143
chr12	107688447	107704747
chr12	107699372	107710510
chr12	107742220	107746424
chr12	107743196	107746424
chr12	107744542	107746424
chr12	1083163	1104824
chr12	1083163	1190052
chr12	1083163	1263165
chr12	1083163	1290012
chr12	108530141	108539089
chr12	108538933	108549214
chr12	108545138	108549214
chr12	108652271	108654410
chr12	108652271	108662158

chr12	108652271	108678394
chr12	108652271	108701323
chr12	108657303	108658919
chr12	108657303	108662158
chr12	108657303	108701323
chr12	108658737	108662158
chr12	108678271	108701323
chr12	108817037	108823361
chr12	108817037	108852678
chr12	108818248	108852678
chr12	10882216	10883096
chr12	10882216	10884275
chr12	10882216	11047188
chr12	109056681	109073532
chr12	109067523	109073532
chr12	109101901	109103611
chr12	109102838	109103611
chr12	109216617	109216920
chr12	109222506	109223904
chr12	109249983	109259108
chr12	109258267	109259108
chr12	109456117	109458078
chr12	109481636	109483981
chr12	109516764	109526416
chr12	109586021	109591357
chr12	109590770	109595181
chr12	10973654	11047188
chr12	109855618	109858741
chr12	109906571	109908937
chr12	109938379	109939247
chr12	109938379	109961685
chr12	109951166	109959958
chr12	109953091	109961375
chr12	109961612	109991760
chr12	109980951	109983694
chr12	109980951	109991760
chr12	110012004	110013249
chr12	110012004	110016433
chr12	110012004	110019328
chr12	110013124	110029635
chr12	110016387	110025823
chr12	110018344	110019328
chr12	110018344	110025823
chr12	110018344	110029635
chr12	110024045	110027766

chr12	110025741	110029635
chr12	110029477	110030758
chr12	110033796	110036328
chr12	110127359	110129130
chr12	110128949	110132636
chr12	110128949	110147048
chr12	110128949	110163065
chr12	110134947	110135437
chr12	110180421	110203950
chr12	110203863	110205680
chr12	110282603	110296737
chr12	110292019	110296737
chr12	110292019	110323072
chr12	110322991	110328017
chr12	110326389	110328017
chr12	110326389	110334143
chr12	110332596	110334143
chr12	110332596	110340994
chr12	110340658	110342448
chr12	11034781	11047188
chr12	110381751	110396452
chr12	110382842	110388623
chr12	110387738	110388623
chr12	110387738	110396452
chr12	110388511	110396452
chr12	110455585	110455930
chr12	110455585	110457634
chr12	110473077	110486717
chr12	1104749	1116033
chr12	1104749	1141787
chr12	110485077	110487943
chr12	110505616	110506693
chr12	110538143	110551968
chr12	110545879	110551968
chr12	110730528	110731901
chr12	1110191	1116033
chr12	1110191	1190052
chr12	111464661	111510582
chr12	111470107	111470742
chr12	111470107	111486824
chr12	111485270	111486824
chr12	111485270	111510582
chr12	111509548	111510582
chr12	111509548	111513539
chr12	111509548	111520076

chr12	111509548	111552977
chr12	111513356	111518427
chr12	111513356	111520076
chr12	111513356	111525316
chr12	111513356	111555919
chr12	111516153	111520076
chr12	111520881	111525316
chr12	111525191	111552977
chr12	111525191	111555919
chr12	111552279	111552977
chr12	111552279	111555919
chr12	1115865	1190052
chr12	1115865	1236904
chr12	111649938	111660675
chr12	111649938	111665787
chr12	111655565	111660675
chr12	111658735	111659345
chr12	111658735	111660675
chr12	111658735	111679340
chr12	111658735	111681835
chr12	111672660	111683307
chr12	111692696	111712657
chr12	111692696	111734068
chr12	111705737	111709684
chr12	111727961	111734068
chr12	111792060	111798242
chr12	111865249	111871180
chr12	111867571	111871180
chr12	111932585	111943369
chr12	111933885	111938210
chr12	111933885	111943369
chr12	111933885	111991889
chr12	111936691	111943369
chr12	112043087	112061388
chr12	112061180	112087801
chr12	112068879	112074764
chr12	112068879	112078741
chr12	112068879	112087801
chr12	112071894	112075789
chr12	112071894	112078741
chr12	112071894	112090864
chr12	112071894	112093136
chr12	112074674	112075789
chr12	112074674	112093136
chr12	112075677	112078741

chr12	112075677	112081134
chr12	112075677	112093136
chr12	112078187	112087801
chr12	112078187	112093136
chr12	112081059	112093136
chr12	112090725	112093136
chr12	112130510	112135066
chr12	112167316	112167917
chr12	112175735	112176702
chr12	112203635	112204623
chr12	112203635	112228811
chr12	112204485	112228811
chr12	112207873	112208630
chr12	112210014	112228811
chr12	112216291	112217195
chr12	112216291	112219489
chr12	112229697	112231715
chr12	112229697	112233085
chr12	112229697	112251134
chr12	112239051	112240027
chr12	112258495	112259265
chr12	112258495	112264212
chr12	112258495	112265983
chr12	112269703	112273795
chr12	112269703	112274960
chr12	112269703	112283302
chr12	112306063	112309669
chr12	112306063	112313147
chr12	112306063	112319742
chr12	112313016	112319742
chr12	112446275	112456063
chr12	112446275	112473040
chr12	112450317	112456063
chr12	112472943	112478015
chr12	112472943	112482205
chr12	112472943	112486629
chr12	112477650	112478015
chr12	112482073	112489175
chr12	112486474	112489175
chr12	112502143	112504796
chr12	112946742	112950975
chr12	113176839	113181058
chr12	113225243	113226964
chr12	113267842	113269845
chr12	113320330	113321348

chr12	113942323	113945924
chr12	116096668	116111512
chr12	116096668	116237705
chr12	116111427	116237705
chr12	116719762	116723223
chr12	116749840	116779348
chr12	116833791	116836282
chr12	116928022	116949706
chr12	116928022	116964854
chr12	116945363	116949706
chr12	116945363	116964854
chr12	116949617	116964854
chr12	117155790	117174341
chr12	117172470	117177641
chr12	11752449	11853561
chr12	118019071	118020985
chr12	118019071	118022359
chr12	118019071	118025828
chr12	118019071	118028030
chr12	118024850	118028030
chr12	118042840	118043377
chr12	1183280	1263165
chr12	118386113	118391282
chr12	11839139	11869969
chr12	11884444	11886026
chr12	1189858	1290012
chr12	119714196	119782637
chr12	119718244	119735357
chr12	119752049	119767182
chr12	119757370	119761055
chr12	119757370	119767182
chr12	119760938	119767182
chr12	119775785	119776842
chr12	119803205	119825368
chr12	119803205	119850275
chr12	119822819	119825368
chr12	119822819	119834228
chr12	119822819	119850275
chr12	119832770	119850275
chr12	119850173	119857698
chr12	119869059	119876181
chr12	120098810	120103949
chr12	120149605	120151391
chr12	120154969	120155719
chr12	120155591	120156604

chr12	120155591	120156992
chr12	120173652	120174169
chr12	120173652	120176217
chr12	120175161	120176217
chr12	120177446	120178950
chr12	120199117	120199485
chr12	120221622	120224377
chr12	120222862	120224377
chr12	120353298	120364755
chr12	120557281	120557682
chr12	120560725	120566980
chr12	120562944	120563943
chr12	120562944	120566980
chr12	120565071	120566980
chr12	120716627	120717042
chr12	120782654	120791557
chr12	121232413	121233092
chr12	12124564	12126921
chr12	12124564	12130893
chr12	121252660	121270945
chr12	121268637	121270945
chr12	121268637	121274585
chr12	12130782	12135300
chr12	12130782	12147556
chr12	121318276	121320459
chr12	121330582	121347005
chr12	121335532	121337392
chr12	121335532	121346031
chr12	121335532	121347005
chr12	121342002	121347881
chr12	121345838	121347881
chr12	121416158	121417911
chr12	121416158	121420334
chr12	121416158	121420778
chr12	121417860	121420778
chr12	12148941	12151038
chr12	121494578	121578946
chr12	121532805	121534590
chr12	121548876	121549638
chr12	12158828	12162419
chr12	12158828	12165295
chr12	121743628	121752223
chr12	121743628	121761738
chr12	121743628	121771549
chr12	121761648	121773513

chr12	121780871	121781192
chr12	12179809	12203400
chr12	121799846	121802784
chr12	121894738	121903107
chr12	121899633	121903107
chr12	121942533	121949061
chr12	122030699	122035427
chr12	122030699	122044053
chr12	122035330	122044053
chr12	122138814	122141083
chr12	122216487	122218397
chr12	122216487	122224748
chr12	122235785	122239945
chr12	122238586	122239945
chr12	122238586	122242508
chr12	122242381	122244762
chr12	122249870	122251099
chr12	122261260	122264199
chr12	122278153	122279145
chr12	122288488	122316855
chr12	122288488	122319348
chr12	122309761	122316855
chr12	122336631	122341697
chr12	122347374	122380558
chr12	122352725	122380558
chr12	122360958	122364107
chr12	122377388	122380558
chr12	122477840	122478292
chr12	122477840	122480311
chr12	122489385	122492789
chr12	122506833	122508447
chr12	122508217	122511188
chr12	122511108	122515227
chr12	122517226	122521428
chr12	122534663	122543634
chr12	122544158	122546674
chr12	122544158	122547969
chr12	122544158	122549864
chr12	122546175	122547969
chr12	122546175	122549864
chr12	122557383	122557689
chr12	122557383	122562699
chr12	122568260	122574380
chr12	122568260	122577029
chr12	122569680	122574380

chr12	122570875	122574380
chr12	122577671	122580670
chr12	122577671	122582985
chr12	122579904	122582985
chr12	122582704	122584990
chr12	122584277	122584990
chr12	122584277	122585774
chr12	122584277	122586757
chr12	122584277	122588816
chr12	122587710	122588816
chr12	122594275	122602887
chr12	122597730	122624688
chr12	122618342	122622607
chr12	122618342	122624688
chr12	122777490	122781330
chr12	122777490	122792121
chr12	122777490	122798200
chr12	122777490	122806295
chr12	122781163	122792121
chr12	122785718	122788929
chr12	122785718	122792121
chr12	122788757	122792121
chr12	122797306	122798200
chr12	122932191	122935431
chr12	122935271	122940995
chr12	122981128	122982538
chr12	123004393	123005548
chr12	123012612	123014042
chr12	123160780	123164090
chr12	123166654	123203375
chr12	123194385	123198334
chr12	123194385	123203375
chr12	123194385	123223127
chr12	123210055	123223127
chr12	123210055	123227616
chr12	123214743	123227616
chr12	123218375	123223127
chr12	123218375	123227616
chr12	123221371	123223127
chr12	123309309	123309856
chr12	123309309	123315660
chr12	123313619	123315660
chr12	123313619	123320866
chr12	123315372	123315660
chr12	123315372	123320866

chr12	123319899	123323831
chr12	123327425	123330509
chr12	123327425	123331375
chr12	123331241	123341088
chr12	123334052	123348133
chr12	123345257	123350441
chr12	123348028	123350441
chr12	123484179	123499536
chr12	123586746	123590449
chr12	123605930	123610128
chr12	123605930	123615316
chr12	123605930	123616603
chr12	123609938	123616603
chr12	12361205	12435785
chr12	123616510	123617872
chr12	123616510	123618837
chr12	123617757	123618837
chr12	123630168	123630533
chr12	123639263	123652735
chr12	123645454	123652735
chr12	123673614	123679289
chr12	123673614	123690674
chr12	123679188	123687035
chr12	123688050	123690674
chr12	123688050	123692723
chr12	123718622	123724791
chr12	123718622	123735624
chr12	123718622	123748785
chr12	123733925	123737271
chr12	124347824	124348314
chr12	124347824	124350737
chr12	124362125	124363799
chr12	124372021	124385887
chr12	124372021	124398181
chr12	124398118	124422555
chr12	124398118	124430787
chr12	124419956	124430787
chr12	124419956	124437996
chr12	124419956	124449867
chr12	124422500	124449867
chr12	124426621	124430787
chr12	124426621	124437996
chr12	124437929	124457162
chr12	124449814	124495368
chr12	124466172	124473131

chr12	124800123	124815114
chr12	124807760	124810289
chr12	124807760	124817707
chr12	124811869	124817707
chr12	124952397	124954211
chr12	124956690	124957135
chr12	124964393	124965812
chr12	124966792	124967218
chr12	124968533	124968968
chr12	124971301	124975511
chr12	12500518	12521463
chr12	125073875	125086443
chr12	125102678	125107268
chr12	125102678	125114557
chr12	125102678	125118765
chr12	12519861	12521463
chr12	1263033	1290012
chr12	1263033	1371977
chr12	12635776	12637714
chr12	12821207	12824677
chr12	128799258	128801009
chr12	128799258	128815070
chr12	128800853	128815070
chr12	128808787	128815070
chr12	1289851	1371977
chr12	1289851	1408247
chr12	1289851	1444750
chr12	130795994	130801488
chr12	130795994	130808704
chr12	130801152	130827267
chr12	130806981	130813031
chr12	130808630	130827267
chr12	131714070	131725630
chr12	131725404	131728428
chr12	131725404	131766308
chr12	131753122	131766308
chr12	131753122	131778330
chr12	131764455	131778330
chr12	131764455	131786588
chr12	131766117	131778330
chr12	131778064	131786588
chr12	131838194	131841800
chr12	131838194	131845449
chr12	131939172	131941983
chr12	131981488	131982478

chr12	131992172	132006302
chr12	132005076	132006302
chr12	132005076	132006877
chr12	132037681	132038096
chr12	132043303	132045900
chr12	13211468	13213821
chr12	132120419	132121980
chr12	132121439	132121980
chr12	132140619	132141420
chr12	132147280	132148106
chr12	132147280	132148895
chr12	132150980	132151820
chr12	132151257	132151820
chr12	132508152	132525823
chr12	132508152	132527988
chr12	132525733	132548032
chr12	132572287	132576931
chr12	132572287	132581516
chr12	132657135	132659509
chr12	132668810	132672322
chr12	132679953	132681279
chr12	132680606	132681279
chr12	132714857	132718120
chr12	132734384	132735512
chr12	132734384	132737065
chr12	132734384	132748331
chr12	132734384	132755133
chr12	132735405	132748331
chr12	132735405	132755133
chr12	132741418	132748020
chr12	132741418	132755133
chr12	132743153	132755133
chr12	132747831	132755133
chr12	132780797	132782493
chr12	132795851	132822311
chr12	132804715	132822311
chr12	132807176	132822311
chr12	132813306	132822311
chr12	132932712	132933155
chr12	132933034	132945797
chr12	132933034	132948270
chr12	132945620	132948270
chr12	133041277	133048848
chr12	133047954	133048848
chr12	133083102	133083561

chr12	133144433	133151904
chr12	133151027	133151904
chr12	133191488	133192003
chr12	1371832	1408247
chr12	1371832	1444750
chr12	14423908	14438267
chr12	14423908	14475968
chr12	14436105	14447053
chr12	14446987	14457295
chr12	14456560	14457295
chr12	14456560	14466590
chr12	14475889	14481185
chr12	15660613	15681302
chr12	15665755	15681302
chr12	15669386	15670923
chr12	15956935	15962947
chr12	15956935	15982436
chr12	16721	17859
chr12	1754257	1772961
chr12	1754257	1778025
chr12	1772841	1778025
chr12	1772841	1781076
chr12	1777853	1784073
chr12	19253939	19255165
chr12	19253939	19261021
chr12	19253939	19265850
chr12	19269769	19287556
chr12	19269769	19322667
chr12	19320561	19322667
chr12	19322309	19322667
chr12	19343322	19348519
chr12	19345841	19359546
chr12	19348398	19359546
chr12	19358227	19366109
chr12	19361581	19366109
chr12	19462509	19473355
chr12	19462509	19493986
chr12	19462509	19500221
chr12	19462509	19514784
chr12	19473247	19493986
chr12	19473247	19500221
chr12	19473247	19514784
chr12	19493799	19500221
chr12	19493799	19518113
chr12	19500096	19514784

chr12	21304426	21318923
chr12	21490198	21499615
chr12	21637070	21647016
chr12	21646898	21654677
chr12	22055148	22062434
chr12	22474750	22484888
chr12	22478312	22493337
chr12	22482556	22506819
chr12	22482556	22513379
chr12	22482556	22518137
chr12	22484696	22490218
chr12	22484696	22506819
chr12	22484696	22513379
chr12	22490122	22506819
chr12	22493222	22506819
chr12	22506710	22518137
chr12	22506710	22524627
chr12	22506710	22527892
chr12	22506710	22544179
chr12	22517985	22527892
chr12	22517985	22544179
chr12	22523425	22527892
chr12	22643762	22673660
chr12	22659013	22673660
chr12	24829822	24836596
chr12	24832722	24836596
chr12	24842081	24849949
chr12	24878529	24894475
chr12	24878529	24901885
chr12	25225613	25227412
chr12	25225613	25245395
chr12	26398875	26419213
chr12	26411319	26443650
chr12	26415302	26443650
chr12	26475295	26483898
chr12	26550246	26602706
chr12	26578712	26658130
chr12	26602369	26655852
chr12	26621122	26632059
chr12	26628032	26655852
chr12	26681873	26686632
chr12	26681873	26695650
chr12	26681873	26716242
chr12	26695605	26716242
chr12	26695605	26725765

chr12	26711172	26716242
chr12	26711172	26725765
chr12	26715298	26725765
chr12	26911177	26917733
chr12	26914407	26922700
chr12	26916001	26917733
chr12	26922615	26925851
chr12	26922615	26928905
chr12	26924354	26928905
chr12	26928204	26928905
chr12	26954144	26957743
chr12	26979783	26982859
chr12	26979783	26990627
chr12	26980565	26990627
chr12	26980565	26999676
chr12	26980565	27003390
chr12	26982772	26990627
chr12	26982772	26999676
chr12	26990449	26999676
chr12	26990449	27003390
chr12	26995236	27003390
chr12	26996741	26999676
chr12	27308338	27309197
chr12	27314503	27315117
chr12	27368261	27370230
chr12	27368261	27380404
chr12	27368261	27385560
chr12	27368261	27387320
chr12	27368261	27390239
chr12	27368261	27403541
chr12	27368261	27418196
chr12	27376345	27385560
chr12	27376345	27390239
chr12	27380246	27390239
chr12	27400533	27403541
chr12	27400533	27418196
chr12	27633361	27635115
chr12	27650009	27656730
chr12	27650009	27667320
chr12	27650009	27671546
chr12	27660883	27671546
chr12	27676427	27687507
chr12	27681545	27691928
chr12	27714779	27724186
chr12	27714779	27735556

chr12	27714779	27737608
chr12	27719807	27724186
chr12	2797728	2800577
chr12	2817222	2818277
chr12	2820085	2821792
chr12	2821691	2824252
chr12	2822793	2824252
chr12	28305648	28307749
chr12	28305648	28362515
chr12	28305648	28391411
chr12	28362437	28391411
chr12	28362437	28452654
chr12	2864319	2866521
chr12	2866392	2868754
chr12	2909528	2922402
chr12	2920762	2922402
chr12	2920762	2934561
chr12	2933417	2938285
chr12	29345442	29361685
chr12	2994737	3012232
chr12	2994737	3022017
chr12	3017397	3022017
chr12	30649136	30666251
chr12	30661140	30663654
chr12	30661140	30665309
chr12	30661140	30666251
chr12	30665219	30666251
chr12	30665219	30669282
chr12	30669182	30690577
chr12	30673989	30684457
chr12	30673989	30690577
chr12	30676497	30684457
chr12	30711565	30716676
chr12	30713784	30724451
chr12	30723258	30726088
chr12	30723258	30751133
chr12	30728647	30741106
chr12	30728647	30751133
chr12	30730238	30741106
chr12	30734967	30741106
chr12	31287633	31298224
chr12	31293804	31298224
chr12	31295233	31305123
chr12	31389323	31402643
chr12	31389323	31409384

chr12	31392266	31392696
chr12	31398174	31409384
chr12	31398174	31415448
chr12	31409262	31415448
chr12	31413435	31426424
chr12	31413435	31433248
chr12	31413435	31447769
chr12	31415366	31426424
chr12	31415366	31433248
chr12	31415366	31442925
chr12	31423596	31424687
chr12	31423596	31426424
chr12	31423596	31433248
chr12	31426292	31447769
chr12	31433154	31447769
chr12	31442774	31447769
chr12	31442774	31452476
chr12	31451939	31460381
chr12	31451939	31499671
chr12	31460193	31499671
chr12	31479588	31499671
chr12	31689006	31702007
chr12	31689006	31709425
chr12	31689006	31719356
chr12	31714881	31719356
chr12	32293993	32306122
chr12	32293993	32337706
chr12	32334515	32338979
chr12	32564136	32611283
chr12	32576265	32611283
chr12	32581959	32611283
chr12	32581959	32625779
chr12	32598496	32601423
chr12	32598496	32602317
chr12	32598496	32611283
chr12	32598496	32619870
chr12	32598496	32625779
chr12	32607956	32611283
chr12	32625653	32633689
chr12	32707366	32713371
chr12	32708152	32713371
chr12	32708152	32718763
chr12	32710928	32713371
chr12	32710928	32718763
chr12	32710928	32722633

chr12	32713208	32718763
chr12	32713208	32722633
chr12	32718642	32722633
chr12	32718642	32733807
chr12	333486	334422
chr12	350620	352324
chr12	350620	354234
chr12	350620	356537
chr12	350620	366104
chr12	352204	354234
chr12	355157	363097
chr12	38675716	38702921
chr12	38693656	38730358
chr12	38730282	38776301
chr12	38760846	38776301
chr12	38760846	38839955
chr12	38760846	38848662
chr12	38829378	38848662
chr12	38829378	38874511
chr12	38839915	38874511
chr12	39301479	39318201
chr12	39366349	39370261
chr12	39864761	39871961
chr12	40028300	40048210
chr12	40346752	40348509
chr12	409367	418636
chr12	42317339	42324104
chr12	42335882	42375074
chr12	42351884	42387535
chr12	42351884	42398994
chr12	42355160	42375074
chr12	42355160	42387535
chr12	42355160	42393689
chr12	42355160	42398994
chr12	42374862	42387535
chr12	42374862	42393689
chr12	42374862	42398994
chr12	42384939	42398994
chr12	42387455	42398994
chr12	42393569	42398994
chr12	428768	438554
chr12	43452273	43454052
chr12	43742456	43748609
chr12	44143524	44211387
chr12	4500137	4518880

chr12	4517060	4518880
chr12	4517060	4525583
chr12	4518773	4534302
chr12	45302013	45331423
chr12	45331294	45348315
chr12	45331294	45357424
chr12	45367687	45390498
chr12	45367687	45409487
chr12	45378052	45390498
chr12	45416698	45423062
chr12	45421070	45423062
chr12	45731216	45821487
chr12	45811417	45860949
chr12	45836588	45839496
chr12	45860800	45893721
chr12	45891779	45893721
chr12	45931505	45934285
chr12	45931505	45934505
chr12	45931505	45951727
chr12	45945248	45951727
chr12	45945248	45964188
chr12	46197719	46198743
chr12	46197719	46229639
chr12	46197918	46204590
chr12	46206079	46229268
chr12	46229152	46229639
chr12	46229152	46239893
chr12	46229152	46243314
chr12	46229152	46254936
chr12	46239678	46243314
chr12	46239678	46254936
chr12	46364643	46367340
chr12	46366863	46370857
chr12	46370775	46371379
chr12	4657747	4685325
chr12	47666979	47670345
chr12	47865046	47882774
chr12	48063709	48074793
chr12	48071666	48074793
chr12	48071666	48096427
chr12	48074323	48074793
chr12	48083590	48098124
chr12	48083590	48101516
chr12	48088800	48098124
chr12	48088800	48101516

chr12	48096342	48098124
chr12	48096342	48101516
chr12	48151200	48153529
chr12	48679035	48681641
chr12	48695998	48705896
chr12	48833276	48834497
chr12	48837280	48837657
chr12	48843939	48845782
chr12	48939654	48941188
chr12	49030600	49031033
chr12	49042740	49048769
chr12	49100387	49107045
chr12	49269464	49269976
chr12	49328926	49329454
chr12	49371227	49490731
chr12	49460769	49489573
chr12	49460769	49490731
chr12	49484589	49490731
chr12	49494740	49500205
chr12	49496832	49500205
chr12	49496832	49514613
chr12	49656403	49657190
chr12	49656403	49658594
chr12	49661965	49668554
chr12	49665831	49668554
chr12	49752463	49755755
chr12	49752463	49759321
chr12	49991997	49992655
chr12	49991997	50002300
chr12	49999140	49999733
chr12	49999140	50002300
chr12	49999140	50005392
chr12	50004234	50006636
chr12	50004234	50016719
chr12	50016630	50018589
chr12	50094436	50096972
chr12	50427761	50429090
chr12	50427761	50430570
chr12	50427761	50435624
chr12	50427761	50437838
chr12	50427761	50441643
chr12	50427761	50454417
chr12	50427761	50461347
chr12	50427764	50429090
chr12	50427764	50430570

chr12	50427764	50454417
chr12	50427764	50461347
chr12	50430494	50437838
chr12	50430494	50441643
chr12	50430494	50454417
chr12	50435487	50441643
chr12	50435487	50454417
chr12	50437734	50441643
chr12	50437734	50454417
chr12	50453459	50454417
chr12	50453459	50461347
chr12	50453459	50467120
chr12	50454313	50461347
chr12	50454313	50467120
chr12	50461134	50467120
chr12	50461134	50474167
chr12	50462581	50474167
chr12	50466958	50474167
chr12	50625975	50660319
chr12	50671185	50674629
chr12	50671185	50686682
chr12	50685832	50686682
chr12	50685832	50697175
chr12	50692948	50697175
chr12	50692948	50699202
chr12	50780139	50814439
chr12	50814009	50814439
chr12	50996816	51010766
chr12	50999173	51010766
chr12	51004787	51010766
chr12	51049033	51056502
chr12	51049033	51058128
chr12	51053777	51056502
chr12	51055834	51056502
chr12	51055834	51058128
chr12	51073822	51076647
chr12	51099654	51104203
chr12	51101934	51104203
chr12	51109120	51116420
chr12	51110876	51117747
chr12	51116314	51117747
chr12	51116314	51118772
chr12	51240342	51241116
chr12	51359131	51365579
chr12	51452123	51453699

chr12	51662763	51687219
chr12	51684173	51687219
chr12	51684173	51688849
chr12	51684173	51702914
chr12	51699569	51721908
chr12	51701143	51702914
chr12	51786541	51794641
chr12	51975264	51991993
chr12	51980968	51986942
chr12	51986817	51991993
chr12	52172197	52185587
chr12	52180512	52185587
chr12	52235154	52237569
chr12	52950327	52952342
chr12	53016472	53019006
chr12	53016472	53020026
chr12	53016472	53022627
chr12	53016472	53028188
chr12	53016472	53034709
chr12	53018797	53020026
chr12	53018797	53022627
chr12	53019909	53022627
chr12	53019909	53028188
chr12	53021805	53028188
chr12	53027781	53028188
chr12	53033805	53037622
chr12	53033805	53038411
chr12	53037408	53038411
chr12	53287971	53291860
chr12	53297849	53298150
chr12	53406584	53409561
chr12	53454725	53471807
chr12	53455884	53461143
chr12	53459271	53471807
chr12	53461014	53471807
chr12	53462492	53471807
chr12	53502014	53504469
chr12	53523275	53552637
chr12	53669147	53669948
chr12	54342205	54348051
chr12	54343224	54348051
chr12	55789074	55796076
chr12	55789074	55800900
chr12	55789074	55803728
chr12	55796024	55803728

chr12	55800561	55803728
chr12	56002211	56002720
chr12	56086530	56088868
chr12	56133416	56134428
chr12	56133587	56134428
chr12	56158683	56160320
chr12	56169528	56170208
chr12	56169775	56174764
chr12	56210296	56214070
chr12	56224833	56226419
chr12	56252014	56254952
chr12	56274776	56282991
chr12	56275995	56282991
chr12	56282419	56282991
chr12	56282419	56286645
chr12	56314850	56315242
chr12	56326312	56328358
chr12	56348528	56349636
chr12	56430880	56432524
chr12	56433025	56434231
chr12	56433380	56434231
chr12	56562416	56571855
chr12	56562416	56586926
chr12	56568974	56571855
chr12	56581183	56582152
chr12	56603358	56605327
chr12	56609735	56612246
chr12	56613952	56617532
chr12	56639105	56640192
chr12	56642457	56643958
chr12	56644780	56645353
chr12	56666203	56670364
chr12	56666203	56673065
chr12	56670274	56673065
chr12	56671748	56673065
chr12	56741434	56744123
chr12	56746044	56751195
chr12	56746780	56751195
chr12	57069233	57072912
chr12	57211926	57212261
chr12	57230802	57234110
chr12	57289008	57296551
chr12	57298089	57310463
chr12	57299351	57310463
chr12	57489895	57498623

chr12	57498156	57498623
chr12	57498156	57500522
chr12	57498156	57504299
chr12	57524028	57529009
chr12	57533952	57534452
chr12	57535055	57535845
chr12	57696274	57715970
chr12	57749181	57749504
chr12	57783109	57787162
chr12	57786162	57787162
chr12	57945627	57953689
chr12	57946994	57953689
chr12	62325871	62355475
chr12	62349220	62355475
chr12	62381489	62384302
chr12	62470700	62501698
chr12	62484169	62495147
chr12	62484169	62499048
chr12	62484169	62501698
chr12	62484169	62508480
chr12	62493914	62508480
chr12	62498918	62501698
chr12	62498918	62526102
chr12	62500782	62508480
chr12	62501572	62508480
chr12	62535524	62543198
chr12	62535524	62556192
chr12	62543096	62553174
chr12	62549667	62553174
chr12	62555993	62571582
chr12	62565236	62585501
chr12	62580296	62588156
chr12	62689698	62720289
chr12	62801815	62832279
chr12	6333068	6334244
chr12	63597808	63663845
chr12	63608615	63621337
chr12	63617303	63647365
chr12	63624039	63663845
chr12	63626468	63647365
chr12	63644402	63647365
chr12	63644402	63661481
chr12	63644402	63663845
chr12	63781018	63805404
chr12	63983946	64017012

chr12	64016949	64043575
chr12	64016949	64065219
chr12	64016949	64080370
chr12	64042789	64043575
chr12	64062916	64065219
chr12	64062916	64087026
chr12	64062916	64097375
chr12	64080285	64115893
chr12	64091275	64097375
chr12	64091275	64115893
chr12	64108931	64115893
chr12	64409961	64419094
chr12	64409961	64420254
chr12	64409961	64420521
chr12	64418045	64419094
chr12	64418045	64420521
chr12	64423004	64431823
chr12	64455839	64467082
chr12	64466900	64474390
chr12	64466900	64490119
chr12	6446960	6448737
chr12	64480011	64486017
chr12	64480011	64490119
chr12	64484302	64486017
chr12	64485454	64486017
chr12	64495482	64496406
chr12	64688215	64691579
chr12	64728955	64737107
chr12	6509716	6511252
chr12	6514264	6514920
chr12	6520985	6522037
chr12	65210925	65241087
chr12	6521797	6523346
chr12	6522827	6523346
chr12	65238501	65238814
chr12	65238501	65241087
chr12	6526447	6527888
chr12	65306862	65308655
chr12	65306862	65328603
chr12	65306862	65369026
chr12	65308528	65328603
chr12	65308528	65369026
chr12	65326825	65369026
chr12	6534809	6537216
chr12	6536683	6537216

chr12	6560097	6566822
chr12	6563314	6563946
chr12	6594458	6595430
chr12	66145840	66153448
chr12	66371693	66377285
chr12	66432547	66596927
chr12	66515618	66529914
chr12	66515618	66541950
chr12	66529830	66596927
chr12	6670759	6688226
chr12	6678945	6688226
chr12	67281909	67299095
chr12	6730401	6730820
chr12	6749847	6750766
chr12	68302619	68325575
chr12	6833591	6834049
chr12	6842969	6845802
chr12	6855754	6856150
chr12	68648698	68654252
chr12	68648698	68657217
chr12	68650399	68654252
chr12	68654111	68657217
chr12	68690630	68696922
chr12	68690630	68700853
chr12	68690630	68702784
chr12	6869257	6869773
chr12	6869257	6870136
chr12	68696818	68702784
chr12	68696818	68715740
chr12	68702735	68710093
chr12	68709237	68715740
chr12	68710004	68715740
chr12	68710004	68725796
chr12	68715626	68721986
chr12	68715626	68722152
chr12	68715626	68725796
chr12	68715626	68727389
chr12	68715626	68731719
chr12	68715626	68732739
chr12	68719340	68722152
chr12	68731109	68731719
chr12	68747929	68752190
chr12	68816811	68824651
chr12	68816811	68828931
chr12	68820324	68824651

chr12	68824362	68828931
chr12	68828770	68836749
chr12	68866895	68871956
chr12	6905584	6906662
chr12	69251128	69253154
chr12	69251128	69260197
chr12	69251128	69262562
chr12	69253050	69262562
chr12	69256696	69262562
chr12	69260043	69262562
chr12	69362787	69370975
chr12	69365632	69370975
chr12	69365789	69370975
chr12	69586752	69588262
chr12	69589484	69592159
chr12	69589484	69593613
chr12	69592058	69593613
chr12	69597155	69598421
chr12	6974338	6974693
chr12	6974338	6975378
chr12	69755383	69756663
chr12	69784719	69795344
chr12	69800208	69801721
chr12	70278131	70311017
chr12	70278131	70319364
chr12	70294070	70311017
chr12	70319297	70330469
chr12	70329422	70332846
chr12	70329422	70346324
chr12	70342106	70346324
chr12	7064266	7065299
chr12	7099685	7102087
chr12	7142868	7143311
chr12	71614534	71615316
chr12	71619314	71620124
chr12	71623359	71624292
chr12	71626259	71629037
chr12	71630822	71634825
chr12	71632885	71633438
chr12	71634154	71642559
chr12	71634703	71636654
chr12	71642435	71644318
chr12	71689590	71699109
chr12	71769799	71774022
chr12	71769799	71782658

chr12	72286680	72378121
chr12	72469757	72499635
chr12	72562164	72575542
chr12	72562164	72621751
chr12	72562852	72575542
chr12	72618890	72621751
chr12	7367235	7379298
chr12	7373319	7433694
chr12	7403534	7433694
chr12	7432415	7433694
chr12	75312847	75326527
chr12	76049755	76074239
chr12	76053090	76053909
chr12	76053090	76056161
chr12	76053769	76056161
chr12	76055018	76056161
chr12	76055018	76060279
chr12	76056032	76074239
chr12	76059797	76060279
chr12	76059797	76068994
chr12	76059797	76074239
chr12	76060137	76068994
chr12	76060137	76074239
chr12	76067370	76068994
chr12	76067370	76074239
chr12	76450850	76487618
chr12	76797433	76822531
chr12	76805316	76809857
chr12	76805316	76822531
chr12	76826907	76842981
chr12	76845708	76849470
chr12	77043064	77056130
chr12	77055854	77064635
chr12	77940318	77941133
chr12	77940318	77968702
chr12	77940318	77998476
chr12	77940318	78007445
chr12	77940318	78021862
chr12	77940318	78059115
chr12	77966228	77968702
chr12	77966228	78007445
chr12	77994802	78021862
chr12	77998336	78021862
chr12	78006418	78021862
chr12	78021746	78059115

chr12	78049992	78059115
chr12	78116771	78118297
chr12	78121939	78140334
chr12	78128705	78137365
chr12	78128705	78159286
chr12	78137176	78168866
chr12	78159202	78168866
chr12	78159202	78181045
chr12	78175305	78181045
chr12	78175305	78190219
chr12	78185600	78190219
chr12	79613574	79621207
chr12	79786373	79788783
chr12	79807225	79810010
chr12	79817393	79822190
chr12	79817393	79828464
chr12	79821077	79828464
chr12	79828319	79845420
chr12	79828319	79872938
chr12	79845301	79872938
chr12	8089935	8090299
chr12	8092675	8095703
chr12	813641	830160
chr12	813641	861343
chr12	82386802	82389922
chr12	82386802	82403130
chr12	82402982	82438791
chr12	82430892	82456820
chr12	827041	830160
chr12	82965023	82966997
chr12	82965023	82986046
chr12	84872601	84884040
chr12	84872601	84892308
chr12	84883858	84892308
chr12	84885434	84886068
chr12	85065261	85066898
chr12	8713810	8754093
chr12	8713810	8780739
chr12	8749861	8754093
chr12	88106674	88107098
chr12	88139144	88141334
chr12	88148287	88174727
chr12	88148287	88176319
chr12	88148287	88190622
chr12	88160113	88172745

chr12	88160113	88176319
chr12	88160113	88188946
chr12	882942	883831
chr12	8920984	8921750
chr12	8943404	8943910
chr12	894561	897681
chr12	89459637	89466922
chr12	89459637	89467685
chr12	89459637	89472275
chr12	89459637	89492115
chr12	89466769	89472275
chr12	89466769	89497342
chr12	89470360	89472275
chr12	89491935	89497342
chr12	89603042	89604346
chr12	89609936	89617039
chr12	916343	927263
chr12	925449	930144
chr12	925449	933076
chr12	92809016	92811434
chr12	92826165	92853025
chr12	92827911	92853025
chr12	92842464	92864987
chr12	92842464	92891721
chr12	92851110	92853025
chr12	92851110	92853954
chr12	929763	930144
chr12	93469188	93487660
chr12	93469191	93487660
chr12	93476953	93487660
chr12	9424527	9427865
chr12	94331699	94367943
chr12	94375885	94403262
chr12	94400849	94403262
chr12	94400849	94412591
chr12	94578393	94582538
chr12	95025149	95040597
chr12	95028386	95051943
chr12	95028386	95057643
chr12	95031348	95057878
chr12	95040475	95049233
chr12	95057552	95057878
chr12	95152810	95172744
chr12	95257149	95274889
chr12	95266356	95287857

chr12	95270038	95282644
chr12	95294271	95296258
chr12	95485878	95504161
chr12	95494055	95496098
chr12	95494055	95504161
chr12	95494055	95511998
chr12	95494956	95504161
chr12	96002959	96017580
chr12	96018762	96019240
chr12	96018762	96024547
chr12	96280807	96300360
chr12	96282508	96297721
chr12	96282508	96334865
chr12	96289166	96313454
chr12	96294998	96298983
chr12	96297626	96298983
chr12	96297626	96334865
chr12	96298868	96300360
chr12	96311051	96324112
chr12	96311051	96334865
chr12	96323947	96334865
chr12	96909751	96917737
chr12	96909751	96920125
chr12	96909751	96936812
chr12	96934975	96945849
chr12	98527885	98531838
chr12	98527885	98537572
chr12	98527885	98546447
chr12	98544229	98544537
chr12	98595726	98598035
chr12	98597855	98598703
chr12	98648318	98649684
chr12	98662455	98662806
chr12	98677424	98699569
chr12	99655066	99782097
chr12	99772921	99782097
chr12	99772921	99806700
chr12	99772921	99812311
chr12	99772921	99825389
chr12	99806403	99825389
chr13	100209331	100368574
chr13	100257594	100273346
chr13	100257594	100309908
chr13	100257594	100340259
chr13	100257594	100449305

chr13	100262728	100309908
chr13	100262728	100368574
chr13	100268688	100273346
chr13	100273195	100309908
chr13	100301459	100309908
chr13	100301459	100340259
chr13	100301459	100368574
chr13	100301459	100449305
chr13	100302923	100330671
chr13	100309832	100368574
chr13	100330560	100340259
chr13	100330560	100368574
chr13	100642210	100670569
chr13	100656380	100670569
chr13	101567698	101583356
chr13	102604792	102618846
chr13	102622876	102627166
chr13	102622876	102627924
chr13	102627011	102640376
chr13	102768259	102770086
chr13	102807145	102822159
chr13	102807145	102834552
chr13	102816427	102822159
chr13	102821036	102831697
chr13	102821036	102834552
chr13	102821742	102831697
chr13	110207402	110211922
chr13	110429884	110501784
chr13	110622215	110624279
chr13	110622215	110627547
chr13	110634544	110635588
chr13	110651033	110683134
chr13	110651033	110705571
chr13	110663450	110705571
chr13	110683050	110701555
chr13	110687720	110705571
chr13	111153904	111244294
chr13	111205288	111217880
chr13	111205288	111233293
chr13	111209871	111233293
chr13	111209871	111244294
chr13	111217678	111233293
chr13	111217678	111244294
chr13	111243871	111267670
chr13	112504031	112527484

chr13	112516439	112527484
chr13	112516439	112545865
chr13	112518974	112527484
chr13	112554056	112559399
chr13	112804956	112816211
chr13	112804956	112819407
chr13	112823344	112851218
chr13	112831374	112842379
chr13	112834588	112842379
chr13	112842275	112854530
chr13	113184345	113190975
chr13	113190871	113200516
chr13	113242967	113245237
chr13	113474123	113484056
chr13	113495501	113503588
chr13	113495501	113510424
chr13	113495501	113520733
chr13	113503495	113520733
chr13	113510237	113520733
chr13	113510237	113534240
chr13	113520615	113539507
chr13	113585773	113636700
chr13	113610995	113623286
chr13	113823374	113828711
chr13	113999584	114000932
chr13	114024278	114029887
chr13	114040999	114073837
chr13	114052051	114073837
chr13	114236644	114259398
chr13	114236798	114250674
chr13	114242120	114247004
chr13	114262878	114272481
chr13	19705661	19709599
chr13	19730238	19782791
chr13	19741564	19759418
chr13	19751270	19759418
chr13	19759322	19772543
chr13	19835476	19862466
chr13	19851354	19852190
chr13	19959957	20036909
chr13	19959957	20067390
chr13	19993062	20006586
chr13	20019546	20036909
chr13	20026611	20036909
chr13	20026611	20052311

chr13	20051432	20059562
chr13	20051432	20067390
chr13	20058574	20059562
chr13	20058574	20064545
chr13	20061052	20067390
chr13	20062845	20067019
chr13	20064450	20083032
chr13	20641289	20644958
chr13	20731840	20737805
chr13	20790461	20855765
chr13	20796075	20800990
chr13	20799164	20800990
chr13	20821703	20822289
chr13	20821703	20843886
chr13	20821703	20868701
chr13	20827066	20868701
chr13	20855626	20868701
chr13	20862716	20868701
chr13	21155692	21158125
chr13	21157921	21172681
chr13	21159901	21172681
chr13	21161789	21168399
chr13	21161789	21172681
chr13	21167987	21172681
chr13	21381433	21387634
chr13	21381433	21391854
chr13	21381433	21401685
chr13	21381433	21402866
chr13	21387507	21425678
chr13	21400372	21413772
chr13	21400372	21425678
chr13	23353784	23365277
chr13	23353784	23368487
chr13	23353784	23371165
chr13	23358334	23368487
chr13	23358334	23371165
chr13	23365165	23368487
chr13	23365165	23371165
chr13	23365165	23375269
chr13	23756544	23806069
chr13	23756544	23809924
chr13	23756544	23862362
chr13	23756544	23886506
chr13	23805949	23809924
chr13	23805949	23839726

chr13	23805949	23841488
chr13	23805949	23870195
chr13	23805949	23874909
chr13	23805949	23886506
chr13	23836239	23841488
chr13	23836239	23864189
chr13	23836239	23886506
chr13	23837551	23886506
chr13	23839648	23886506
chr13	23841334	23879354
chr13	23841334	23886506
chr13	23858859	23870195
chr13	23858859	23874909
chr13	23858859	23881787
chr13	23858859	23886506
chr13	23862301	23870195
chr13	23862301	23881787
chr13	23862301	23886506
chr13	23864140	23881787
chr13	23864140	23886506
chr13	23869291	23881787
chr13	23869291	23886506
chr13	23870012	23886506
chr13	23874845	23886506
chr13	23879267	23886506
chr13	24222818	24251862
chr13	24249476	24251862
chr13	24249476	24284271
chr13	24278613	24290884
chr13	24284134	24286393
chr13	24284134	24290884
chr13	24286213	24290884
chr13	24288998	24290884
chr13	24441845	24443730
chr13	24441845	24447186
chr13	24441845	24449817
chr13	24441845	24452593
chr13	24441845	24455212
chr13	24452405	24456478
chr13	24452405	24460136
chr13	24455016	24460136
chr13	24455016	24500382
chr13	24456340	24493733
chr13	24456340	24499376
chr13	24456340	24501834

chr13	24459043	24469110
chr13	24459043	24475596
chr13	24459043	24478276
chr13	24459043	24493733
chr13	24459263	24475596
chr13	24469023	24475596
chr13	24469023	24478276
chr13	24469023	24486305
chr13	24469893	24499376
chr13	24475471	24478276
chr13	24475471	24498229
chr13	24477700	24478276
chr13	24477700	24484748
chr13	24477700	24486305
chr13	24477700	24493733
chr13	24477700	24501834
chr13	24477700	24503777
chr13	24478092	24493733
chr13	24484652	24486305
chr13	24484652	24493733
chr13	24484652	24498229
chr13	24484652	24499376
chr13	24484652	24501834
chr13	24484652	24503777
chr13	24490667	24493733
chr13	24490667	24498229
chr13	24490667	24501834
chr13	24490667	24503777
chr13	24492420	24498229
chr13	24493595	24498229
chr13	24498115	24501834
chr13	24498115	24503777
chr13	24499300	24501834
chr13	24499300	24503777
chr13	24500315	24501834
chr13	25251648	25274187
chr13	25251852	25274187
chr13	25265818	25267941
chr13	25307805	25313751
chr13	25307805	25315467
chr13	25307805	25321093
chr13	25307805	25325068
chr13	25309246	25321093
chr13	25312882	25321093
chr13	25312882	25327512

chr13	25319325	25321093
chr13	25320529	25338731
chr13	25326915	25327512
chr13	25769045	25774959
chr13	26218510	26221330
chr13	26337566	26353880
chr13	26337566	26385342
chr13	26353739	26400550
chr13	26382813	26401624
chr13	26400452	26401624
chr13	26612960	26642403
chr13	26612960	26671989
chr13	26612960	26676724
chr13	27075190	27090158
chr13	27075190	27105944
chr13	27089882	27105944
chr13	27089882	27116596
chr13	27427091	27430621
chr13	27437115	27445156
chr13	27567639	27569276
chr13	27567639	27581803
chr13	28174271	28177935
chr13	28174271	28197346
chr13	28174271	28220378
chr13	28174271	28256539
chr13	28177864	28220378
chr13	28197184	28220378
chr13	28197184	28272071
chr13	28256291	28261458
chr13	28256291	28266876
chr13	28256291	28270866
chr13	28256291	28281379
chr13	28260446	28280541
chr13	28266714	28270866
chr13	28662409	28668574
chr13	28662409	28672432
chr13	28703942	28710843
chr13	28703942	28713550
chr13	28710759	28713550
chr13	28710759	28718022
chr13	29281703	29324711
chr13	29281703	29359473
chr13	29324612	29359473
chr13	29480149	29492719
chr13	29487899	29492719

chr13	29492645	29501194
chr13	29516137	29519549
chr13	29517143	29517790
chr13	29517143	29532982
chr13	29517143	29536202
chr13	29517143	29553860
chr13	29517572	29536202
chr13	29522316	29524253
chr13	29522316	29532982
chr13	29522316	29536202
chr13	29522316	29553860
chr13	29523265	29536202
chr13	29530537	29532982
chr13	29530537	29536202
chr13	29530537	29553860
chr13	29532823	29536202
chr13	29532823	29553860
chr13	29535818	29553860
chr13	29767617	29777263
chr13	30210315	30280223
chr13	30227411	30231472
chr13	30227411	30241086
chr13	30227411	30255615
chr13	30227411	30283791
chr13	30230467	30280223
chr13	30231313	30241086
chr13	30240459	30255615
chr13	30240459	30280223
chr13	30240459	30283791
chr13	30240958	30255615
chr13	30255446	30280223
chr13	30255446	30283791
chr13	30280062	30283791
chr13	30621763	30642757
chr13	30621763	30653305
chr13	30630834	30647057
chr13	30630834	30653305
chr13	30637743	30647057
chr13	30642627	30647057
chr13	31215050	31229294
chr13	31215050	31247966
chr13	31215050	31261082
chr13	31215050	31286819
chr13	31222951	31286819
chr13	31229184	31269277

chr13	31247022	31261082
chr13	31269213	31286819
chr13	31946008	31956471
chr13	32078833	32117473
chr13	32265449	32267359
chr13	32316421	32326282
chr13	32319076	32325184
chr13	32319076	32326613
chr13	32325075	32326613
chr13	32346826	32355288
chr13	32346826	32357929
chr13	32362522	32363533
chr13	32379316	32380145
chr13	32517856	32527532
chr13	32521372	32527532
chr13	32522181	32527532
chr13	32648753	32652007
chr13	32648753	32658531
chr13	32648753	32678929
chr13	32658238	32659280
chr13	32658238	32667844
chr13	32658238	32678929
chr13	32667763	32710106
chr13	32678834	32696902
chr13	32687133	32696902
chr13	32732100	32735330
chr13	32732100	32746100
chr13	32753331	32760720
chr13	35040932	35045401
chr13	35040932	35048684
chr13	35040932	35050395
chr13	35117413	35123574
chr13	35176995	35232619
chr13	35208699	35232619
chr13	36189945	36202093
chr13	36326574	36335832
chr13	36853418	36879875
chr13	36952513	36985740
chr13	36985626	36995596
chr13	36993603	36995596
chr13	37040404	37051583
chr13	37047534	37051583
chr13	39679537	39727548
chr13	39694633	39727548
chr13	40749137	40759106

chr13	40805117	40808596
chr13	40826505	40836885
chr13	40826505	40855815
chr13	41252644	41260915
chr13	41316845	41320824
chr13	41316845	41323190
chr13	41316845	41331369
chr13	41318805	41336756
chr13	41325697	41331369
chr13	41328723	41331369
chr13	41369089	41372830
chr13	41670945	41685242
chr13	41670945	41703411
chr13	41727193	41811340
chr13	41761127	41778056
chr13	41761127	41833531
chr13	41777984	41811340
chr13	41777984	41819386
chr13	41777984	41833531
chr13	41787436	41819386
chr13	41811224	41819386
chr13	41811224	41833531
chr13	41811224	41868477
chr13	41865735	41868477
chr13	41865735	41887361
chr13	41865735	41891587
chr13	41883386	41912168
chr13	41883386	41950013
chr13	41891419	41912168
chr13	41907585	41912168
chr13	41907585	41950013
chr13	42155290	42155799
chr13	42155290	42160136
chr13	42159265	42160136
chr13	42165330	42168818
chr13	42168439	42187148
chr13	42168439	42190525
chr13	42190402	42195016
chr13	42198477	42209465
chr13	42285985	42295742
chr13	42895008	42900383
chr13	43065685	43085838
chr13	43411915	43412988
chr13	44949721	44959574
chr13	44959380	44982154

chr13	44965843	44982154
chr13	44979189	44982154
chr13	45136732	45207505
chr13	45136732	45252970
chr13	45149769	45207505
chr13	45151686	45207505
chr13	45207423	45267376
chr13	45338659	45340184
chr13	45402274	45404412
chr13	45516142	45519094
chr13	45516142	45525051
chr13	45979812	45989097
chr13	45988786	46010505
chr13	46356431	46363213
chr13	46368041	46368815
chr13	46689132	46728984
chr13	46692535	46695017
chr13	46692535	46728984
chr13	46711790	46733998
chr13	46723220	46728984
chr13	46723220	46733998
chr13	47948939	47954557
chr13	47948939	47997023
chr13	47988540	47997023
chr13	48079843	48090418
chr13	48083370	48090418
chr13	48342598	48349023
chr13	48367493	48377034
chr13	48373404	48381443
chr13	49006151	49138805
chr13	49075288	49136601
chr13	49131136	49145935
chr13	49138746	49145935
chr13	49138746	49167303
chr13	49145777	49172096
chr13	49167243	49175541
chr13	49168612	49178654
chr13	49168612	49188633
chr13	49187121	49188633
chr13	49359713	49401270
chr13	49451552	49461162
chr13	49451552	49467960
chr13	49480218	49483563
chr13	49523174	49524216
chr13	49551325	49552285

chr13	49705620	49706372
chr13	49705620	49711022
chr13	49706097	49711022
chr13	49709571	49722563
chr13	49709571	49725501
chr13	49732370	49746993
chr13	49927918	49931134
chr13	49997073	50044768
chr13	50433412	50492761
chr13	50927406	50934999
chr13	50927406	50943394
chr13	50927406	50949505
chr13	51376047	51389444
chr13	51395299	51430383
chr13	51675169	51727790
chr13	51703595	51739175
chr13	51719197	51739175
chr13	51957515	51965033
chr13	52397231	52402681
chr13	52416016	52427066
chr13	52416016	52442445
chr13	52417991	52426066
chr13	52417991	52427219
chr13	52433661	52434882
chr13	52434792	52442445
chr13	52621988	52637446
chr13	52632421	52637446
chr13	52657531	52659249
chr13	52658398	52665733
chr13	52658398	52680155
chr13	52661474	52666919
chr13	52676229	52680155
chr13	59774188	59810923
chr13	59774188	59839448
chr13	59833106	59839448
chr13	59833106	59861536
chr13	59833106	59916249
chr13	59861406	59916249
chr13	59861406	59924870
chr13	59879228	59916249
chr13	59879228	59924870
chr13	59911734	59916249
chr13	59911734	59924870
chr13	59911734	59971160
chr13	59969943	59974456

chr13	60008543	60010669
chr13	60008543	60016145
chr13	60008543	60042820
chr13	60093627	60112186
chr13	60093627	60132989
chr13	60439687	60460540
chr13	60439687	60467379
chr13	60439687	60485948
chr13	60444682	60483846
chr13	60444682	60485948
chr13	60444682	60494575
chr13	60460379	60467379
chr13	60460379	60483846
chr13	60467237	60494575
chr13	60483774	60510755
chr13	60483774	60535233
chr13	60509762	60510755
chr13	60535107	60567650
chr13	72743536	72747111
chr13	72854056	72931267
chr13	72893783	72908681
chr13	72893783	72931267
chr13	72908530	72931267
chr13	73813151	73846373
chr13	75294853	75306471
chr13	75302242	75306471
chr13	75326196	75327826
chr13	75326196	75349302
chr13	75336920	75359858
chr13	75336920	75362607
chr13	75560752	75569507
chr13	75566694	75569507
chr13	77015408	77021861
chr13	77081426	77087633
chr13	77171491	77180318
chr13	77176496	77180318
chr13	77205255	77217957
chr13	77217839	77233263
chr13	77217839	77243951
chr13	77217839	77264002
chr13	77243058	77251355
chr13	77243058	77262129
chr13	77251150	77264002
chr13	77251150	77296674
chr13	77257670	77264002

chr13	77260427	77264002
chr13	77278757	77296674
chr13	78635109	78644997
chr13	79368729	79371930
chr13	79370944	79371930
chr13	79520809	79539775
chr13	94605390	94619517
chr13	95034604	95053184
chr13	95034604	95062859
chr13	95034604	95083290
chr13	95043681	95053184
chr13	95071661	95083290
chr13	95115921	95170628
chr13	95115921	95188542
chr13	95115921	95194937
chr13	95161188	95166367
chr13	95161188	95170628
chr13	95161188	95178091
chr13	95161188	95188542
chr13	95170531	95178091
chr13	95170531	95188542
chr13	95177706	95188542
chr13	95206531	95210781
chr13	95206531	95234834
chr13	95709226	95725252
chr13	95723241	95725252
chr13	95757643	95760798
chr13	95757643	95763953
chr13	95832926	95837202
chr13	95832926	95859675
chr13	95925679	95940091
chr13	95925679	95949454
chr13	95925679	95972671
chr13	95947036	95996135
chr13	95947995	95996135
chr13	95970111	95996135
chr13	95970111	95999307
chr13	95983803	95990073
chr13	95983803	95996135
chr13	95983803	95999307
chr13	95983803	96013481
chr13	95986332	95996135
chr13	96013306	96031971
chr13	97992891	98000645
chr13	98457167	98466561

chr13	98457167	98475358
chr13	98457167	98519473
chr13	98463690	98475358
chr13	98474820	98475358
chr13	98474820	98482321
chr13	98474820	98519473
chr13	98482264	98519473
chr13	98824397	98837609
chr13	98883812	98885092
chr13	98920953	98923371
chr13	98920953	98930257
chr13	99238426	99244624
chr13	99238426	99318069
chr13	99243831	99244624
chr13	99314096	99340565
chr13	99529466	99537863
chr13	99536607	99559534
chr13	99541558	99549222
chr14	100084686	100097658
chr14	100262303	100274758
chr14	100337061	100343387
chr14	100353686	100361921
chr14	100380909	100468168
chr14	100380909	100484124
chr14	100468020	100484124
chr14	101882160	101890296
chr14	101892999	101909690
chr14	101901718	101906529
chr14	101901718	101917947
chr14	101912400	101917947
chr14	101983018	101987632
chr14	101991526	101995300
chr14	102002536	102002965
chr14	102009842	102012113
chr14	102018450	102020056
chr14	102018450	102022880
chr14	102019892	102022880
chr14	102027173	102027544
chr14	102038459	102039732
chr14	102038459	102042748
chr14	102040235	102040673
chr14	102040235	102044698
chr14	102043874	102044698
chr14	102193462	102195120
chr14	102194937	102197847

chr14	102325984	102331846
chr14	102325984	102335838
chr14	102325984	102339223
chr14	102331621	102339223
chr14	102376649	102408619
chr14	102376649	102414793
chr14	102424978	102428382
chr14	102681894	102707512
chr14	102681894	102711013
chr14	102701277	102708583
chr14	102714422	102721377
chr14	102830333	102876525
chr14	102830333	102897401
chr14	102870184	102871968
chr14	102870184	102876525
chr14	102870184	102891417
chr14	102870184	102897401
chr14	102870184	102903429
chr14	102875623	102891417
chr14	102889559	102903429
chr14	102946467	102950602
chr14	102963060	102964650
chr14	102968252	102970261
chr14	102980772	102986580
chr14	102983555	102986580
chr14	102999564	103008555
chr14	102999564	103012188
chr14	103003927	103012188
chr14	103008471	103012188
chr14	103335652	103339803
chr14	103336676	103339803
chr14	103338326	103339803
chr14	103405075	103428440
chr14	103405075	103448967
chr14	103448918	103457212
chr14	103451917	103462461
chr14	103451917	103466442
chr14	103451917	103468186
chr14	103468032	103492034
chr14	103474992	103480490
chr14	103491776	103500200
chr14	103571622	103574170
chr14	103654563	103657776
chr14	103662115	103662927
chr14	103662115	103670283

chr14	103669510	103673431
chr14	103669510	103677523
chr14	103728907	103729846
chr14	103746372	103749934
chr14	103746372	103757751
chr14	103746372	103797518
chr14	103752999	103754244
chr14	103752999	103757751
chr14	103752999	103797518
chr14	103778744	103797518
chr14	103784794	103797518
chr14	104157754	104167048
chr14	104774937	104792722
chr14	104775651	104792722
chr14	105151282	105155989
chr14	105226068	105226760
chr14	105226068	105228913
chr14	105226068	105241414
chr14	105252506	105272894
chr14	105252506	105286376
chr14	105348492	105355177
chr14	105438671	105454310
chr14	105450057	105460953
chr14	105450057	105466742
chr14	105450260	105454310
chr14	105454192	105460953
chr14	19064582	19066347
chr14	20316226	20329373
chr14	20325538	20329373
chr14	20344931	20345464
chr14	20344931	20346913
chr14	20344931	20351122
chr14	20344931	20352347
chr14	20346862	20354247
chr14	20350525	20352347
chr14	20352244	20354247
chr14	20369343	20369779
chr14	20369343	20401581
chr14	20389609	20403448
chr14	20395449	20395949
chr14	20395449	20396730
chr14	20400983	20403884
chr14	20403376	20406400
chr14	20996208	20996711
chr14	21076359	21088876

chr14	21091864	21093674
chr14	21211405	21213117
chr14	21211405	21234229
chr14	21211809	21234229
chr14	21211809	21263336
chr14	21230318	21234229
chr14	21230318	21263336
chr14	21357196	21361213
chr14	21368268	21373430
chr14	21371873	21373430
chr14	21390946	21393254
chr14	21390946	21395352
chr14	21401956	21403663
chr14	21415573	21429335
chr14	21426127	21429335
chr14	21488589	21489403
chr14	21499763	21500682
chr14	21500494	21501903
chr14	21503172	21503881
chr14	22769790	22771348
chr14	22906194	22909595
chr14	22906194	22911403
chr14	22908332	22911403
chr14	22909482	22911403
chr14	22924269	22928211
chr14	22926132	22926814
chr14	22926132	22927660
chr14	22947170	22948013
chr14	22947170	22950410
chr14	22947170	22952683
chr14	22947170	22955176
chr14	22947600	22955176
chr14	22950313	22952683
chr14	22950313	22955176
chr14	22974046	22976712
chr14	23061083	23061622
chr14	23061083	23064488
chr14	23081747	23090633
chr14	23322180	23324289
chr14	24210347	24210768
chr14	24210347	24211829
chr14	24211414	24211829
chr14	24266429	24268619
chr14	24440782	24442452
chr14	29599026	29626556

chr14	29624151	29636494
chr14	29632868	29638904
chr14	30581075	30593639
chr14	30586717	30593639
chr14	30597419	30602131
chr14	30598482	30602131
chr14	30628208	30634037
chr14	30628208	30638247
chr14	30628208	30639864
chr14	30628208	30643405
chr14	30628208	30653588
chr14	30628208	30670395
chr14	30633946	30638247
chr14	30633946	30639864
chr14	30633946	30643405
chr14	30633946	30653588
chr14	30638124	30653588
chr14	30639776	30650650
chr14	30643315	30670395
chr14	30670255	30673997
chr14	30670255	30675065
chr14	30670255	30700258
chr14	30670255	30708065
chr14	30673256	30694869
chr14	30673256	30702375
chr14	30673256	30705885
chr14	30705822	30722559
chr14	30705822	30734858
chr14	30715923	30734858
chr14	30902535	30911162
chr14	30905417	30913657
chr14	30905417	30956242
chr14	30911040	30913657
chr14	30911776	30956242
chr14	30913523	30919106
chr14	30935162	30950944
chr14	30935162	30956242
chr14	30947089	30950944
chr14	30947089	30956242
chr14	30950862	30956242
chr14	30955619	30956242
chr14	31066125	31069929
chr14	31066125	31072973
chr14	31105382	31112543
chr14	31112415	31114340

chr14	31112415	31119866
chr14	31112415	31133675
chr14	31113103	31114340
chr14	31116235	31121505
chr14	31116235	31133675
chr14	31127784	31133414
chr14	31127784	31133675
chr14	31127784	31144941
chr14	31133237	31133675
chr14	31133237	31136665
chr14	31141857	31172122
chr14	31144110	31172122
chr14	31156839	31157303
chr14	31156839	31169468
chr14	31156839	31172122
chr14	31168297	31169468
chr14	31168297	31172122
chr14	31168297	31178254
chr14	31295908	31308020
chr14	31302294	31308020
chr14	31302294	31309182
chr14	31304904	31306879
chr14	31306731	31308020
chr14	31306731	31309182
chr14	31306731	31315949
chr14	31315769	31321680
chr14	31326162	31337614
chr14	31326162	31347907
chr14	31337475	31347907
chr14	31343895	31345276
chr14	31345086	31347907
chr14	31349788	31350717
chr14	31358636	31387375
chr14	31364188	31387375
chr14	31371809	31387375
chr14	31374815	31387375
chr14	31380466	31383771
chr14	31394051	31403049
chr14	31562067	31599379
chr14	31562067	31673574
chr14	31599288	31673574
chr14	32117139	32152528
chr14	34524696	34533539
chr14	34529357	34533539
chr14	34567685	34568013

chr14	34567853	34597645
chr14	34567853	34608140
chr14	34575755	34586305
chr14	34575755	34609742
chr14	34581560	34609742
chr14	34586229	34597645
chr14	34586229	34609742
chr14	34593044	34597645
chr14	34593044	34609742
chr14	34597549	34609742
chr14	34603347	34609742
chr14	34603347	34629954
chr14	34758703	34762223
chr14	34775918	34780310
chr14	34775918	34783232
chr14	34783761	34786221
chr14	34783761	34792921
chr14	34783761	34795765
chr14	34792774	34795765
chr14	34800223	34807538
chr14	34802853	34807538
chr14	34807450	34862322
chr14	34810934	34862322
chr14	34826012	34862322
chr14	34999557	35001020
chr14	35000935	35008831
chr14	35000935	35014830
chr14	35007282	35008831
chr14	35007282	35011659
chr14	35013345	35019074
chr14	35018691	35019074
chr14	35046504	35053451
chr14	35096557	35108236
chr14	35099251	35108236
chr14	35099251	35110629
chr14	35107303	35110629
chr14	35109818	35110629
chr14	35122951	35180777
chr14	35126734	35127611
chr14	35126734	35180777
chr14	35307993	35310895
chr14	35307993	35314455
chr14	35595633	35635598
chr14	35595633	35659196
chr14	35625360	35627951

chr14	35625360	35635598
chr14	35651804	35659196
chr14	35721687	35725153
chr14	35721687	35742565
chr14	35738512	35762753
chr14	35770941	35775745
chr14	35832977	35862686
chr14	36314511	36316812
chr14	37247102	37247907
chr14	37247102	37270525
chr14	37247102	37285447
chr14	37247828	37422949
chr14	37285317	37308519
chr14	37285317	37369624
chr14	37285317	37422949
chr14	37369516	37500138
chr14	39039030	39076093
chr14	39042785	39085906
chr14	39045162	39048729
chr14	39045162	39063413
chr14	39048651	39067296
chr14	39055142	39063413
chr14	39055142	39067296
chr14	39055142	39076093
chr14	39061764	39087008
chr14	39064912	39085906
chr14	39074414	39085906
chr14	39074414	39087008
chr14	39091476	39092627
chr14	39093186	39096139
chr14	39117998	39128348
chr14	39128279	39132068
chr14	39151745	39159550
chr14	39154210	39159550
chr14	39158284	39159550
chr14	39177411	39177916
chr14	39179090	39179462
chr14	39291018	39295029
chr14	39291018	39299986
chr14	39313339	39327022
chr14	39313339	39348977
chr14	39345903	39348977
chr14	44995745	45054549
chr14	44999362	45012075
chr14	45006007	45022973

chr14	45006007	45025872
chr14	45006007	45032376
chr14	45008912	45025872
chr14	45008912	45027474
chr14	45008912	45054549
chr14	45011974	45028329
chr14	45011974	45054549
chr14	45025782	45028329
chr14	45025782	45032376
chr14	45027298	45054549
chr14	45032222	45046703
chr14	45043685	45046703
chr14	45046544	45054549
chr14	45095220	45097005
chr14	45095220	45107616
chr14	45120886	45130800
chr14	45148836	45155459
chr14	45148836	45159280
chr14	45148836	45170746
chr14	45151396	45154052
chr14	45154696	45159280
chr14	45154696	45170746
chr14	45187780	45189362
chr14	45217018	45218454
chr14	45231140	45237721
chr14	45235813	45237721
chr14	46873432	46882221
chr14	46873432	46920160
chr14	46873432	47035304
chr14	46920011	47035304
chr14	46957373	47035304
chr14	46957373	47097123
chr14	47061248	47097123
chr14	49647292	49655843
chr14	49653989	49655843
chr14	49663314	49665163
chr14	49663314	49666413
chr14	49663314	49669598
chr14	49663314	49674427
chr14	49663314	49683693
chr14	49664625	49666413
chr14	49664625	49669598
chr14	49664625	49674427
chr14	49669523	49674427
chr14	49723873	49725769

chr14	49795790	49800696
chr14	49799474	49800696
chr14	49802452	49806133
chr14	49802452	49814857
chr14	49825866	49831361
chr14	49828290	49832277
chr14	49831298	49834449
chr14	49832050	49834449
chr14	49832050	49838206
chr14	49832050	49840866
chr14	49840717	49846265
chr14	50129960	50145596
chr14	50134122	50150230
chr14	50145169	50145596
chr14	50145169	50158646
chr14	50145476	50150230
chr14	50150007	50153173
chr14	50156998	50174552
chr14	50174453	50182606
chr14	50180571	50188700
chr14	50180571	50201084
chr14	50180571	50204409
chr14	50182462	50199855
chr14	50265357	50269330
chr14	50265357	50284033
chr14	50267752	50303017
chr14	50283870	50303017
chr14	50321482	50322719
chr14	50338946	50341232
chr14	50423120	50435065
chr14	50425906	50435065
chr14	50434393	50440441
chr14	50434393	50486194
chr14	50437475	50440441
chr14	50442731	50445194
chr14	50442731	50448832
chr14	50443938	50448832
chr14	50445040	50448832
chr14	50456515	50464133
chr14	50462664	50475149
chr14	50462664	50482416
chr14	50466582	50476306
chr14	50466582	50482416
chr14	50466582	50486194
chr14	50466582	50504857

chr14	50475076	50486194
chr14	50475076	50504857
chr14	50485577	50504857
chr14	50640749	50645014
chr14	50735515	50744365
chr14	50738139	50752733
chr14	50741581	50744365
chr14	50761789	50766890
chr14	50763825	50773095
chr14	50770387	50822077
chr14	50792711	50806818
chr14	50792711	50822077
chr14	50806736	50822077
chr14	50908270	50917105
chr14	50908270	50921067
chr14	50913028	50917105
chr14	50915335	50917105
chr14	50920540	50937837
chr14	51022834	51025360
chr14	51243855	51247220
chr14	51243855	51249765
chr14	51689690	51712551
chr14	51689690	51715499
chr14	51698141	51702588
chr14	52470036	52470680
chr14	52470036	52491005
chr14	52482229	52491005
chr14	52490838	52542453
chr14	52511239	52544371
chr14	52536718	52544371
chr14	52598271	52600341
chr14	52653068	52658009
chr14	52711100	52711524
chr14	52757318	52759754
chr14	52780678	52784664
chr14	53051843	53093444
chr14	53058476	53061201
chr14	53058476	53091932
chr14	53061125	53091932
chr14	53072596	53091932
chr14	53091784	53103856
chr14	54401523	54411706
chr14	54408744	54415930
chr14	54430260	54436437
chr14	54702061	54751537

chr14	54737023	54751537
chr14	54737023	54764540
chr14	54737023	54776540
chr14	54737023	54784620
chr14	54748814	54764540
chr14	54748814	54776540
chr14	54774933	54776540
chr14	54859680	54865436
chr14	54955560	54957204
chr14	54957033	54957635
chr14	54957033	54981696
chr14	54966474	54967394
chr14	54966474	54981696
chr14	54966474	54987387
chr14	54966474	55002185
chr14	54981539	55000992
chr14	54981539	55002185
chr14	54984722	54987387
chr14	54984722	55000992
chr14	54984722	55002185
chr14	54995602	55000992
chr14	54995602	55002185
chr14	55000502	55000992
chr14	55008315	55013596
chr14	55010308	55013596
chr14	55151694	55154806
chr14	55158521	55163075
chr14	55175345	55176018
chr14	55175893	55177336
chr14	55181212	55183753
chr14	55377818	55382191
chr14	55612018	55619312
chr14	55616516	55619312
chr14	55616516	55628028
chr14	55616516	55630097
chr14	55648024	55648870
chr14	55648024	55651927
chr14	55652849	55659703
chr14	55653016	55658614
chr14	55653016	55661612
chr14	55656041	55658614
chr14	55671565	55671877
chr14	56585000	56636598
chr14	56618593	56636598
chr14	57209566	57229881

chr14	57217981	57222416
chr14	57229733	57234032
chr14	57229733	57247712
chr14	57231505	57233883
chr14	57235710	57244359
chr14	57237337	57244359
chr14	57244164	57247712
chr14	57246710	57247712
chr14	58211291	58223856
chr14	58213630	58223856
chr14	58215204	58223856
chr14	58215204	58230480
chr14	58219693	58223856
chr14	58247749	58263770
chr14	58252118	58257998
chr14	58304943	58318805
chr14	58304943	58330169
chr14	58318541	58330169
chr14	58410842	58411971
chr14	58442705	58444175
chr14	58472198	58487166
chr14	58482512	58487166
chr14	58488620	58512627
chr14	58492143	58512627
chr14	59263440	59291306
chr14	59487673	59498908
chr14	60114699	60116060
chr14	60114699	60125823
chr14	60115718	60125251
chr14	60115718	60125823
chr14	60118328	60121299
chr14	60149352	60153178
chr14	60149352	60156152
chr14	60796216	60798160
chr14	60796216	60818847
chr14	60796216	60879835
chr14	60798086	60808428
chr14	60798086	60812127
chr14	60808324	60818847
chr14	60811986	60879835
chr14	61015903	61046167
chr14	61015903	61052135
chr14	61030444	61046167
chr14	61043146	61046167
chr14	61043146	61052135

chr14	61043449	61078927
chr14	61720378	61721823
chr14	61720381	61721823
chr14	61720381	61727655
chr14	61720381	61734285
chr14	61721508	61721823
chr14	61721508	61732524
chr14	61721508	61734285
chr14	61721508	61738373
chr14	61726705	61727655
chr14	61738086	61741188
chr14	61740504	61744813
chr14	61766875	61768740
chr14	61766875	61778910
chr14	61768635	61778140
chr14	63391816	63396716
chr14	63415139	63453885
chr14	63961524	63967846
chr14	63980985	63991115
chr14	63982629	63986617
chr14	63998913	64000719
chr14	64080455	64081580
chr14	64101931	64121061
chr14	64186423	64190237
chr14	64400792	64412525
chr14	64412471	64418024
chr14	64412471	64419925
chr14	64412471	64424931
chr14	64417887	64419925
chr14	64417887	64424931
chr14	64431531	64439172
chr14	64431531	64440266
chr14	64431531	64441453
chr14	64439095	64440266
chr14	64439095	64441453
chr14	64440125	64441453
chr14	64442053	64444734
chr14	64442053	64448317
chr14	64442053	64449622
chr14	64442053	64453861
chr14	64448216	64449622
chr14	64731713	64732901
chr14	65455620	65561766
chr14	65455620	65629606
chr14	65561336	65629606

chr14	65615977	65629606
chr14	65721774	65724323
chr14	66824473	66924292
chr14	66879938	67023675
chr14	66916002	67058786
chr14	66916002	67100911
chr14	66922665	66924292
chr14	66924193	66965325
chr14	66965190	67023675
chr14	66965190	67058786
chr14	66965190	67089075
chr14	67023632	67122377
chr14	67100855	67143449
chr14	67110139	67113171
chr14	67122255	67159488
chr14	67269700	67279537
chr14	67269700	67302118
chr14	67269700	67303599
chr14	67269700	67312710
chr14	67301388	67303599
chr14	67312526	67321259
chr14	67340439	67347453
chr14	67345767	67347453
chr14	67345767	67350690
chr14	67345767	67353040
chr14	67557268	67559691
chr14	67592634	67593952
chr14	67685014	67690421
chr14	67685014	67692593
chr14	67685014	67693052
chr14	67690211	67693052
chr14	67692437	67693052
chr14	67761365	67762819
chr14	67767703	67769730
chr14	67777558	67781529
chr14	67806544	67809289
chr14	67823541	67825577
chr14	67823541	67835196
chr14	67823541	67865139
chr14	67823541	67887204
chr14	67865002	67887204
chr14	67885868	67887204
chr14	68291883	68411527
chr14	68902476	68921125
chr14	68904654	68912242

chr14	68912155	68925672
chr14	68921005	68925672
chr14	69075344	69091887
chr14	69075344	69122360
chr14	69116365	69119230
chr14	69116365	69122360
chr14	69203916	69209803
chr14	69203916	69210201
chr14	69228815	69230598
chr14	69441831	69442266
chr14	69453240	69455866
chr14	69703394	69704742
chr14	69768137	69768674
chr14	70584816	70591381
chr14	70584816	70597777
chr14	70592879	70597777
chr14	70748828	70750081
chr14	70748828	70761182
chr14	70946914	70962331
chr14	70946914	70969110
chr14	70946914	70978648
chr14	70946914	71076419
chr14	70962225	70969110
chr14	70962225	70978648
chr14	70962225	70995925
chr14	70968197	70978648
chr14	70976941	70995925
chr14	70976941	71011549
chr14	70976941	71013202
chr14	70988566	70995925
chr14	70988566	71009724
chr14	70988566	71011549
chr14	70988566	71013202
chr14	70988566	71023232
chr14	71009633	71019162
chr14	71009633	71023232
chr14	71009633	71036157
chr14	71012999	71036157
chr14	71019008	71036157
chr14	71026116	71036157
chr14	71026771	71036157
chr14	71036064	71052012
chr14	71036064	71055562
chr14	71036064	71073798
chr14	71045132	71047984

chr14	71045132	71057724
chr14	71045132	71076419
chr14	71045132	71089342
chr14	71055503	71057724
chr14	71073544	71076419
chr14	71105234	71109046
chr14	71587570	71624236
chr14	71618756	71624236
chr14	71618756	71672622
chr14	71658332	71672622
chr14	71702380	71709664
chr14	72714422	72731934
chr14	72714422	72771893
chr14	72723632	72731934
chr14	72937970	72940377
chr14	72974079	72975721
chr14	72974079	72978969
chr14	72993142	72998315
chr14	73071626	73077536
chr14	73071626	73088161
chr14	73071626	73097100
chr14	73076318	73077536
chr14	73077368	73088161
chr14	73083493	73088161
chr14	73147794	73148094
chr14	73147794	73148106
chr14	73147794	73173707
chr14	73147794	73186920
chr14	73147794	73198129
chr14	73147794	73206472
chr14	73147794	73211942
chr14	73147794	73217244
chr14	73170796	73173707
chr14	73170796	73217244
chr14	73198030	73211942
chr14	73198030	73217244
chr14	73206385	73211942
chr14	73249983	73255018
chr14	73653169	73671597
chr14	73653169	73687385
chr14	73653169	73689515
chr14	73654846	73671597
chr14	73654846	73689515
chr14	73725271	73729985
chr14	73736997	73740255

chr14	73858815	73874214
chr14	73892026	73893932
chr14	73892026	73896534
chr14	73892026	73905083
chr14	73892026	73909430
chr14	73892026	73923522
chr14	73893796	73904110
chr14	73896315	73905083
chr14	73903959	73905083
chr14	73909340	73921105
chr14	73953434	73955928
chr14	73955450	73955928
chr14	73958146	73959522
chr14	73977023	73983161
chr14	74034027	74040645
chr14	74091231	74101238
chr14	74549862	74555693
chr14	74585853	74603705
chr14	74669648	74671483
chr14	74683295	74692328
chr14	74714871	74716099
chr14	74778446	74782325
chr14	74780404	74782325
chr14	74780404	74802676
chr14	74802555	74824307
chr14	74809379	74810420
chr14	74810224	74816265
chr14	74889094	74894409
chr14	75018828	75033490
chr14	75030542	75038412
chr14	75030542	75042477
chr14	75032067	75038412
chr14	75039910	75042477
chr14	75041614	75042477
chr14	75087017	75088641
chr14	75097099	75103997
chr14	75100991	75103997
chr14	75100991	75107487
chr14	75103841	75124223
chr14	75109684	75110371
chr14	75109684	75118935
chr14	75109684	75124223
chr14	75117194	75124223
chr14	75663054	75734050
chr14	75707017	75720703

chr14	75717860	75720703
chr14	75717860	75735289
chr14	75732337	75735289
chr14	75745094	75752955
chr14	75771733	75793100
chr14	75775483	75776850
chr14	75863666	75902224
chr14	75882684	75902224
chr14	76154353	76176690
chr14	76154353	76195972
chr14	76166662	76176690
chr14	76166662	76178042
chr14	76166662	76180849
chr14	76166662	76195972
chr14	76171842	76176690
chr14	76171842	76178042
chr14	76171842	76180849
chr14	76176622	76195972
chr14	76803370	76809643
chr14	77296163	77304800
chr14	77296163	77306441
chr14	77442559	77454102
chr14	77444248	77454102
chr14	77462159	77465667
chr14	77464597	77465667
chr14	77464597	77468508
chr14	77552095	77562489
chr14	77552095	77576915
chr14	77557040	77576915
chr14	77570383	77576915
chr14	77570383	77579109
chr14	77576766	77579109
chr14	77675655	77679970
chr14	77720710	77723277
chr14	77720710	77737075
chr14	77730987	77736006
chr14	77732484	77736006
chr14	77738777	77739061
chr14	77751318	77755120
chr14	77818967	77822518
chr14	80504911	80530886
chr14	80526868	80580423
chr14	80743074	80761613
chr14	80743074	80778046
chr14	80756891	80778046

chr14	80831142	80838278
chr14	80831142	80840768
chr14	80862756	80900029
chr14	80862756	80906081
chr14	80895717	80906081
chr14	81185530	81216514
chr14	81196107	81216514
chr14	81485671	81490465
chr14	81495080	81499662
chr14	81495080	81502883
chr14	81497891	81502883
chr14	81502720	81506241
chr14	87963383	87988523
chr14	88496412	88508020
chr14	88496412	88517261
chr14	88496412	88550619
chr14	88500782	88501368
chr14	88500782	88508020
chr14	88563650	88578140
chr14	88574692	88578140
chr14	88574692	88607363
chr14	88575839	88578140
chr14	88657375	88658388
chr14	88657375	88665489
chr14	88657375	88696952
chr14	88712270	88715195
chr14	88726540	88746283
chr14	88726540	88754671
chr14	88736363	88746283
chr14	88736363	88754671
chr14	88744022	88754671
chr14	88746184	88754671
chr14	88841424	88872452
chr14	88852970	88861332
chr14	88870058	88872452
chr14	89931540	89932627
chr14	89963107	89971271
chr14	89963107	89993483
chr14	89966146	89971271
chr14	89966146	89993483
chr14	89966146	90019418
chr14	89971174	89985210
chr14	89975780	89993483
chr14	89980539	89985210
chr14	89980539	89993483

chr14	89984515	89993483
chr14	89984515	90019418
chr14	90019315	90033214
chr14	90286353	90304382
chr14	90312393	90318113
chr14	90400064	90403968
chr14	90403861	90404514
chr14	90578105	90618045
chr14	90593485	90618045
chr14	90644047	90647023
chr14	90644047	90655110
chr14	90644047	90658387
chr14	90644047	90680535
chr14	90644047	90786328
chr14	90676522	90689712
chr14	90676522	90695578
chr14	90676522	90730196
chr14	90730074	90744922
chr14	90730074	90786328
chr14	90780737	90786328
chr14	90875200	90894583
chr14	90920205	90947550
chr14	90943077	90947550
chr14	90943077	90978524
chr14	90943077	91001159
chr14	90947434	91001159
chr14	90978305	91001159
chr14	91289104	91300070
chr14	91473032	91476986
chr14	91481575	91485730
chr14	91481575	91490802
chr14	92007639	92011072
chr14	92070934	92082466
chr14	92088729	92096838
chr14	92093251	92096838
chr14	92096092	92096838
chr14	92126121	92143294
chr14	92142163	92143294
chr14	92555750	92577477
chr14	92651581	92685150
chr14	92716135	92717461
chr14	92806735	92809519
chr14	92809299	92816421
chr14	92809299	92837449
chr14	92958200	92962849

chr14	92993879	93076619
chr14	93212031	93220411
chr14	93214928	93215281
chr14	93219211	93220411
chr14	93220248	93222374
chr14	93245824	93263993
chr14	93257194	93263993
chr14	93261601	93296157
chr14	93293857	93296157
chr14	94057821	94079747
chr14	94241787	94242408
chr14	95124195	95141748
chr14	95132514	95141748
chr14	95140658	95141748
chr14	96289655	96291682
chr14	96328346	96329634
chr14	96341521	96343281
chr14	96341521	96347341
chr14	96520054	96521072
chr14	96520054	96525391
chr14	96520054	96528006
chr14	96525309	96532649
chr14	96527429	96532649
chr14	96527429	96547918
chr14	96542242	96552622
chr14	96833466	96837817
chr14	96833466	96847344
chr14	96833466	96853166
chr14	96833466	96856586
chr14	96833466	96860735
chr14	96833466	96876120
chr14	96846094	96856586
chr14	96846094	96860735
chr14	96846094	96876120
chr14	96847256	96860735
chr14	96852830	96856586
chr14	96852830	96860735
chr14	96853073	96860735
chr14	99412950	99465813
chr14	99458278	99463578
chr14	99458278	99465813
chr14	99461191	99463578
chr14	99492625	99503644
chr14	99850852	99865646
chr14	99914178	99914697

chr15	100132006	100155320
chr15	100132006	100199423
chr15	100132006	100281401
chr15	100152611	100155320
chr15	100152611	100199423
chr15	100199317	100331054
chr15	100254135	100281401
chr15	100281228	100331054
chr15	100609706	100612427
chr15	100609706	100630042
chr15	100885266	100887712
chr15	100885266	100898185
chr15	100885266	100905687
chr15	100892509	100894082
chr15	100900574	100905687
chr15	100983527	100989398
chr15	100983527	101029232
chr15	101008836	101015402
chr15	101054945	101058141
chr15	101284966	101293172
chr15	101684074	101686062
chr15	101703858	101714963
chr15	20461191	20466370
chr15	22516875	22539247
chr15	22572928	22574073
chr15	22839654	22860789
chr15	22843101	22845267
chr15	22845145	22853268
chr15	22872824	22873729
chr15	22879912	22883011
chr15	22909193	22910813
chr15	22909193	22912275
chr15	22914725	22932340
chr15	22925981	22932340
chr15	22925981	22933893
chr15	22927905	22933893
chr15	22937103	22947291
chr15	22943172	22947291
chr15	22944557	22947291
chr15	23008698	23010133
chr15	23008698	23019334
chr15	23008698	23022161
chr15	23021958	23024830
chr15	23023946	23037152
chr15	23026115	23037152

chr15	23030884	23037152
chr15	24962113	24968082
chr15	24975357	24977029
chr15	24977777	24978318
chr15	25340084	25360527
chr15	25354352	25360527
chr15	25354352	25375763
chr15	25375464	25405502
chr15	25405460	25411971
chr15	28141431	28141846
chr15	28141431	28142393
chr15	28141431	28152830
chr15	28143872	28146344
chr15	28167686	28169655
chr15	28190964	28192151
chr15	28190964	28196569
chr15	28229194	28230500
chr15	28229673	28233341
chr15	28245880	28246897
chr15	28245880	28248736
chr15	28245880	28257261
chr15	28245880	28263169
chr15	28248551	28254518
chr15	28254339	28256317
chr15	28254339	28257261
chr15	28260776	28263169
chr15	28260776	28265974
chr15	28265617	28280287
chr15	28270694	28280287
chr15	28280067	28293022
chr15	28280067	28321464
chr15	28292887	28299516
chr15	29098489	29114016
chr15	29708558	29711000
chr15	29737263	29742781
chr15	29761138	29762438
chr15	29761138	29773357
chr15	29761138	29800702
chr15	29766265	29772166
chr15	29766265	29773357
chr15	29766265	29800702
chr15	29772063	29773357
chr15	29772063	29800702
chr15	30908117	30914091
chr15	30908117	30918295

chr15	30910613	30914091
chr15	30913857	30920653
chr15	30958862	30976955
chr15	30967919	30976955
chr15	30974313	30976955
chr15	32526812	32528701
chr15	32620107	32628802
chr15	34145397	34148750
chr15	34145397	34154684
chr15	34148631	34153069
chr15	34148631	34163690
chr15	34152789	34163690
chr15	34235180	34250355
chr15	34250297	34251057
chr15	34398633	34407735
chr15	34860041	34867609
chr15	34882501	34886661
chr15	34890214	34893773
chr15	34890214	34897705
chr15	34896896	34897705
chr15	34900621	34932434
chr15	34915037	34927138
chr15	34915037	34930371
chr15	34920331	34938813
chr15	34927034	34932434
chr15	34927034	34934635
chr15	34927034	34948384
chr15	34930257	34948384
chr15	34932317	34942080
chr15	34940898	34944428
chr15	34948263	34964290
chr15	35410834	35454820
chr15	35450684	35454820
chr15	35538273	35542507
chr15	36691684	36709961
chr15	38299372	38324809
chr15	38299372	38339895
chr15	38465268	38474298
chr15	38470226	38474298
chr15	39713443	39741900
chr15	39763820	39776531
chr15	39939504	39955768
chr15	39996963	40008195
chr15	39996963	40011346
chr15	39998730	40001224

chr15	40009613	40019200
chr15	40170061	40176673
chr15	40170061	40183883
chr15	40170061	40185642
chr15	40176476	40196774
chr15	40183713	40185642
chr15	40183713	40200980
chr15	40183713	40210210
chr15	40185164	40185642
chr15	40208636	40218562
chr15	40383227	40387206
chr15	40383227	40392023
chr15	40386361	40389929
chr15	40386361	40392023
chr15	40387158	40392023
chr15	40602914	40610297
chr15	40602914	40611511
chr15	40602914	40615378
chr15	40605109	40611511
chr15	40608846	40619011
chr15	40650300	40652105
chr15	40698756	40701201
chr15	40698756	40706294
chr15	40698756	40709116
chr15	40706176	40709116
chr15	40706176	40728824
chr15	40709024	40728824
chr15	40709024	40729974
chr15	40737123	40738042
chr15	40737123	40745259
chr15	40744046	40745259
chr15	40744046	40752178
chr15	40773726	40779997
chr15	40982861	40987974
chr15	40985337	40987974
chr15	40987090	40987974
chr15	40997528	41021125
chr15	41005592	41027736
chr15	41016087	41027736
chr15	41016087	41096353
chr15	41020899	41027736
chr15	41047407	41050102
chr15	41047407	41054014
chr15	41055246	41056706
chr15	41069569	41070547

chr15	41069569	41072058
chr15	41069569	41074565
chr15	41069569	41079904
chr15	41071848	41074565
chr15	41073427	41079904
chr15	41073427	41081073
chr15	41079700	41081073
chr15	41081019	41085583
chr15	41081019	41087682
chr15	41085368	41087682
chr15	41085368	41092182
chr15	41085368	41096353
chr15	41095600	41096353
chr15	41243666	41270618
chr15	41356038	41358258
chr15	41356038	41365589
chr15	41356038	41371684
chr15	41356038	41375828
chr15	41356038	41377304
chr15	41371526	41377304
chr15	41375711	41377304
chr15	41394858	41397140
chr15	41438320	41453048
chr15	41452900	41457876
chr15	41464770	41471349
chr15	41466140	41471349
chr15	41471171	41477515
chr15	41474619	41477515
chr15	41523770	41527068
chr15	41529469	41529979
chr15	41529469	41531202
chr15	41562547	41565141
chr15	41668827	41707859
chr15	41696074	41698941
chr15	41696074	41699159
chr15	41696074	41708208
chr15	41696074	41713496
chr15	41707727	41713496
chr15	41713150	41729349
chr15	41727179	41729349
chr15	41734521	41736698
chr15	41748636	41750615
chr15	41754436	41760529
chr15	41754436	41762362
chr15	41799822	41813101

chr15	41810882	41812127
chr15	41810882	41813101
chr15	42161681	42165113
chr15	42233212	42268032
chr15	42236319	42268032
chr15	42244049	42268032
chr15	42260957	42268032
chr15	42274167	42308318
chr15	42287690	42329449
chr15	42321784	42329449
chr15	42321784	42330672
chr15	42338388	42340754
chr15	42444201	42444981
chr15	42547260	42548456
chr15	42559338	42563857
chr15	42583345	42585637
chr15	42634855	42638812
chr15	42651015	42663917
chr15	42661157	42669337
chr15	42662793	42669337
chr15	42665252	42669337
chr15	42726309	42735178
chr15	42775134	42817097
chr15	42775134	42840433
chr15	42783418	42817097
chr15	42783418	42840433
chr15	42794643	42817097
chr15	42794643	42840433
chr15	42794643	42872758
chr15	42810613	42817097
chr15	42810613	42830078
chr15	42810613	42840433
chr15	42810613	42872758
chr15	42817031	42830078
chr15	42817031	42840433
chr15	42827927	42830078
chr15	42827927	42840433
chr15	42827927	42878684
chr15	42829937	42840433
chr15	42872610	42878684
chr15	42950261	42960701
chr15	42950261	42966286
chr15	42950261	42970607
chr15	42958012	42970607
chr15	42966152	42970607

chr15	42983896	43025429
chr15	42988818	43007284
chr15	43002554	43022801
chr15	43003836	43025429
chr15	43007078	43025429
chr15	43015687	43017181
chr15	43015687	43022801
chr15	43015687	43025429
chr15	43017094	43025429
chr15	43021274	43025429
chr15	43021274	43048491
chr15	43024828	43025429
chr15	43047160	43048491
chr15	43054741	43060114
chr15	43054741	43086240
chr15	43134256	43138619
chr15	43134256	43160794
chr15	43135511	43160794
chr15	43146492	43169677
chr15	43146492	43181299
chr15	43148754	43160794
chr15	43333310	43335826
chr15	43335695	43343075
chr15	43335695	43349045
chr15	43376097	43377067
chr15	43376097	43377903
chr15	43377846	43383504
chr15	43380083	43383504
chr15	43380083	43386330
chr15	43385790	43404552
chr15	43398037	43401850
chr15	43398040	43401850
chr15	43400043	43401850
chr15	43407942	43420735
chr15	43415593	43416416
chr15	43432193	43438416
chr15	43438323	43447485
chr15	43441525	43447485
chr15	43446386	43447485
chr15	43469857	43477759
chr15	43469857	43481022
chr15	43479396	43481022
chr15	43753823	43756766
chr15	43753823	43761531
chr15	43756648	43763206

chr15	43761423	43763206
chr15	43765449	43766910
chr15	43768488	43769646
chr15	43768488	43770329
chr15	43768488	43770580
chr15	43814500	43815074
chr15	43827964	43835150
chr15	43835060	43844054
chr15	43836160	43839728
chr15	43836160	43844054
chr15	43842414	43844054
chr15	43843900	43857163
chr15	44322964	44332078
chr15	44322964	44338317
chr15	44328684	44332078
chr15	44328684	44338317
chr15	44328684	44380976
chr15	44331987	44338317
chr15	44331987	44380976
chr15	44337762	44338317
chr15	44379689	44380976
chr15	44458990	44490999
chr15	44484223	44486700
chr15	44484223	44490999
chr15	44484223	44499813
chr15	44496379	44497138
chr15	44514597	44519295
chr15	44569397	44570658
chr15	44572682	44575041
chr15	44592330	44596943
chr15	44595258	44596943
chr15	44606024	44608605
chr15	44606024	44610985
chr15	44615362	44620403
chr15	44626330	44629388
chr15	44626330	44659303
chr15	44633504	44660616
chr15	44648865	44652266
chr15	44648865	44660616
chr15	45040407	45043421
chr15	45068180	45069052
chr15	45175218	45178306
chr15	45405319	45410530
chr15	45405319	45418682
chr15	45417268	45418682

chr15	45488840	45490881
chr15	48596282	48613092
chr15	48610727	48644950
chr15	48767059	48769081
chr15	48772486	48791376
chr15	48797947	48805656
chr15	48996366	49019552
chr15	49011730	49017628
chr15	49012647	49028682
chr15	49027364	49037769
chr15	49137149	49139653
chr15	49201161	49239367
chr15	49235850	49239367
chr15	49235850	49283718
chr15	49235850	49292537
chr15	49625112	49634794
chr15	49632158	49634794
chr15	50300788	50301368
chr15	50300788	50304133
chr15	50300788	50309798
chr15	50303965	50309798
chr15	50439008	50449485
chr15	50441348	50465191
chr15	50449399	50459162
chr15	50449399	50462322
chr15	50458999	50462322
chr15	50458999	50465191
chr15	50458999	50471795
chr15	50462279	50471795
chr15	50465046	50471795
chr15	50465046	50477499
chr15	50476848	50498728
chr15	50570103	50575789
chr15	50574273	50575789
chr15	50574636	50575789
chr15	50574851	50575789
chr15	50583088	50592626
chr15	50586391	50592626
chr15	50607199	50619798
chr15	50613706	50614263
chr15	50613706	50628249
chr15	50613706	50632992
chr15	50619744	50632992
chr15	50631416	50632992
chr15	50631416	50634556

chr15	50631416	50648885
chr15	50631416	50663046
chr15	50634381	50639548
chr15	50637421	50639548
chr15	50643339	50648885
chr15	50643339	50663046
chr15	50648686	50657819
chr15	50648686	50663046
chr15	50726320	50739828
chr15	50732602	50749746
chr15	50736100	50739828
chr15	50739679	50748870
chr15	50747494	50748870
chr15	50747494	50749746
chr15	50912077	50930971
chr15	50941441	50950169
chr15	50948019	50950169
chr15	50948019	50958794
chr15	50948019	50968377
chr15	50958491	50968377
chr15	51394834	51401743
chr15	51451644	51455228
chr15	51456065	51463496
chr15	51463378	51464876
chr15	51507133	51517167
chr15	51535662	51538452
chr15	51535662	51547408
chr15	51542332	51576181
chr15	51563380	51568558
chr15	51563380	51576181
chr15	51773711	51777018
chr15	51781043	51782828
chr15	51862810	51869373
chr15	51869216	51889145
chr15	51887588	51893945
chr15	51887588	51896526
chr15	51896418	51902036
chr15	51900154	51902036
chr15	51959898	51960733
chr15	52045829	52050137
chr15	52049992	52061500
chr15	52133377	52141272
chr15	52405286	52410476
chr15	52405286	52433285
chr15	52425829	52433285

chr15	52584744	52593736
chr15	55223888	55234956
chr15	55223888	55238491
chr15	55376948	55378376
chr15	55432496	55450367
chr15	55439471	55450367
chr15	55624341	55627128
chr15	55624341	55673676
chr15	55627007	55629004
chr15	55637169	55638676
chr15	55679285	55740684
chr15	55680053	55680628
chr15	55834037	55847034
chr15	55850541	55856196
chr15	55860406	55874008
chr15	55862912	55874008
chr15	55869578	55874008
chr15	55924645	55966546
chr15	55951375	55966546
chr15	56142777	56179303
chr15	56383951	56412436
chr15	56388471	56412436
chr15	56391242	56394834
chr15	56394164	56394834
chr15	56689040	56707306
chr15	56700932	56707306
chr15	56919891	56921098
chr15	56919891	57063823
chr15	57063749	57091891
chr15	57091788	57197825
chr15	57166401	57197825
chr15	57197772	57282598
chr15	57231151	57234107
chr15	58610296	58611991
chr15	58610296	58633359
chr15	58611807	58717727
chr15	58621470	58627883
chr15	58621470	58643978
chr15	58621470	58646204
chr15	58621470	58665197
chr15	58621470	58679282
chr15	58621470	58682314
chr15	58627699	58682314
chr15	58633195	58717727
chr15	58640776	58682314

chr15	58665096	58717727
chr15	58802312	58847470
chr15	58886974	58887540
chr15	58912562	58913696
chr15	58912562	58916999
chr15	59030803	59058550
chr15	59030803	59076215
chr15	59030803	59085785
chr15	59052304	59058550
chr15	59052304	59067083
chr15	59080935	59089759
chr15	59084128	59085785
chr15	59084128	59089759
chr15	59084128	59092640
chr15	59107321	59107670
chr15	59153589	59214720
chr15	59173745	59174240
chr15	59174125	59214720
chr15	59195460	59214720
chr15	59195460	59218087
chr15	59202325	59214720
chr15	59202325	59218087
chr15	59205399	59218087
chr15	59217890	59256378
chr15	59256283	59272449
chr15	59458549	59460206
chr15	59507212	59516840
chr15	59671182	59672739
chr15	59672636	59682514
chr15	59677910	59682514
chr15	60355918	60364523
chr15	60355918	60382441
chr15	60357145	60382441
chr15	60360940	60382441
chr15	60360940	60386086
chr15	60431933	60448145
chr15	60453602	60466713
chr15	60455002	60468322
chr15	61910176	61912004
chr15	61913310	61927320
chr15	61927090	61936750
chr15	61929500	61931259
chr15	61949442	61954554
chr15	61972624	61981478
chr15	61990999	61991802

chr15	61990999	62035052
chr15	62007307	62013992
chr15	62012106	62044255
chr15	62020478	62044255
chr15	62033440	62044255
chr15	62675216	62692941
chr15	63249644	63259692
chr15	63255506	63259692
chr15	63529013	63537156
chr15	63529013	63563008
chr15	63529013	63574403
chr15	63529013	63589011
chr15	63532646	63537156
chr15	63532646	63556748
chr15	63532646	63558188
chr15	63532646	63559970
chr15	63532646	63563008
chr15	63532646	63574403
chr15	63537024	63558188
chr15	63537024	63574403
chr15	63553714	63558188
chr15	63553714	63559970
chr15	63553714	63563008
chr15	63553714	63570579
chr15	63553714	63574403
chr15	63558105	63559970
chr15	63559856	63563008
chr15	63570432	63574403
chr15	63622814	63624327
chr15	63628676	63630635
chr15	63648068	63659936
chr15	63651252	63659936
chr15	63652413	63658718
chr15	63655741	63658718
chr15	63659735	63666472
chr15	63664469	63666472
chr15	63693963	63716473
chr15	63696123	63716473
chr15	63696123	63749791
chr15	63698727	63716473
chr15	63706779	63716473
chr15	63712774	63716473
chr15	63718573	63734849
chr15	63727646	63749791
chr15	63732923	63755325

chr15	63732923	63756748
chr15	63746917	63747858
chr15	63747723	63756748
chr15	63758174	63775649
chr15	63764095	63775649
chr15	64112572	64118854
chr15	64112572	64123546
chr15	64118116	64118854
chr15	64118116	64123546
chr15	64127173	64131892
chr15	64180347	64216713
chr15	64203081	64207594
chr15	64203081	64216713
chr15	64204440	64204949
chr15	64213889	64216713
chr15	64251511	64300723
chr15	64406329	64409828
chr15	64418540	64425631
chr15	64741172	64751638
chr15	64748418	64750381
chr15	64748418	64751638
chr15	64817945	64819223
chr15	64818256	64819223
chr15	64822265	64824354
chr15	64926065	64931585
chr15	64965319	64981025
chr15	64969254	64974747
chr15	64974601	64976555
chr15	64974601	64981025
chr15	64974601	64983593
chr15	64976474	64983593
chr15	64980863	64983593
chr15	65016435	65021616
chr15	65023671	65027040
chr15	65058426	65059418
chr15	65152429	65180204
chr15	65153546	65155081
chr15	65178933	65180204
chr15	65260884	65271072
chr15	65450998	65452102
chr15	65450998	65456371
chr15	65450998	65467223
chr15	65454262	65456371
chr15	65454262	65474288
chr15	65463760	65474288

chr15	65463760	65480399
chr15	65463760	65481615
chr15	65463760	65490299
chr15	65463760	65512564
chr15	65466677	65467223
chr15	65466677	65481615
chr15	65474208	65480399
chr15	65474208	65490299
chr15	65478879	65480399
chr15	65478879	65481615
chr15	65480221	65481615
chr15	65481514	65512564
chr15	65485098	65487818
chr15	65485098	65490299
chr15	65487689	65500779
chr15	65487689	65512564
chr15	65490188	65500779
chr15	65497863	65500779
chr15	65500605	65507355
chr15	65507242	65512564
chr15	65512294	65515791
chr15	65551675	65558731
chr15	65551675	65564342
chr15	65554886	65558731
chr15	65554886	65564342
chr15	65570090	65572366
chr15	65591597	65593572
chr15	65591597	65595825
chr15	65598871	65607442
chr15	65667448	65676634
chr15	65669778	65670188
chr15	65696365	65697383
chr15	65702304	65706224
chr15	65715477	65732818
chr15	65715477	65756472
chr15	65722847	65756472
chr15	65729071	65732818
chr15	65729071	65738875
chr15	65729071	65741784
chr15	65729071	65756472
chr15	65737706	65738875
chr15	65741714	65756472
chr15	65752378	65756472
chr15	65752378	65761438
chr15	65756139	65761438

chr15	65877331	65877955
chr15	66119085	66128411
chr15	66294987	66314117
chr15	66294987	66315215
chr15	66306823	66308844
chr15	66311723	66318618
chr15	66318448	66320732
chr15	66349059	66349437
chr15	66349059	66351600
chr15	66435026	66436892
chr15	66435026	66444707
chr15	66436745	66444707
chr15	66481754	66485191
chr15	66481754	66487292
chr15	66508840	66537263
chr15	66513987	66546705
chr15	66518878	66521205
chr15	66520589	66523748
chr15	66528851	66533013
chr15	66528851	66540210
chr15	66532246	66537263
chr15	66535932	66537263
chr15	66535932	66540210
chr15	67164894	67165384
chr15	67164894	67166853
chr15	67164894	67170604
chr15	67231813	67236820
chr15	67235978	67236820
chr15	67550033	67586913
chr15	67550033	67600749
chr15	67580753	67600749
chr15	67580753	67630927
chr15	67580753	67664645
chr15	67600684	67646469
chr15	67600684	67664645
chr15	67600684	67748601
chr15	67600684	67772752
chr15	67630887	67664645
chr15	67646230	67664645
chr15	67646230	67703408
chr15	67658552	67703408
chr15	67664596	67703408
chr15	67664596	67748601
chr15	67692478	67703408
chr15	67692478	67727945

chr15	67703336	67772752
chr15	67830190	67833257
chr15	68141945	68146700
chr15	68141945	68153695
chr15	68141945	68164804
chr15	68141945	68173892
chr15	68145815	68164804
chr15	68145815	68173892
chr15	68153589	68183667
chr15	68164730	68176654
chr15	68173731	68176654
chr15	68173731	68181354
chr15	68173731	68183667
chr15	68175636	68176654
chr15	68209636	68218650
chr15	68211262	68218650
chr15	68214289	68218650
chr15	69210315	69261329
chr15	69417382	69422435
chr15	69421646	69422435
chr15	69421646	69423329
chr15	69423158	69426457
chr15	69426069	69438405
chr15	69426335	69429213
chr15	69426335	69436261
chr15	69436137	69436722
chr15	69440307	69441079
chr15	70682757	70699660
chr15	70687539	70699660
chr15	70900921	70919193
chr15	70900921	70936822
chr15	70911531	70936822
chr15	70911531	70980184
chr15	71242648	71256715
chr15	71411686	71660734
chr15	71875790	71888116
chr15	71878039	71893778
chr15	71916369	71938927
chr15	71978170	72010447
chr15	71991102	71994585
chr15	71991102	72007952
chr15	71991102	72010447
chr15	71991102	72027793
chr15	71994468	72010447
chr15	71999850	72010447

chr15	71999850	72027793
chr15	72007825	72027793
chr15	72007825	72032588
chr15	72007825	72046634
chr15	72019038	72046634
chr15	72027730	72046634
chr15	72032493	72046634
chr15	72206727	72208891
chr15	72217408	72219110
chr15	72217408	72221284
chr15	72257347	72259661
chr15	72261557	72271287
chr15	72350517	72356617
chr15	72518066	72544964
chr15	72518066	72555907
chr15	72518066	72561549
chr15	72518066	72572165
chr15	72544819	72555907
chr15	72544819	72572165
chr15	72544819	72582187
chr15	72555270	72572165
chr15	72561482	72570307
chr15	72561482	72572165
chr15	72563393	72570307
chr15	72563393	72572165
chr15	72566562	72572165
chr15	72570176	72572165
chr15	72695176	72716850
chr15	72695176	72724655
chr15	72709699	72716850
chr15	72731304	72735966
chr15	72760406	72775097
chr15	72771782	72775097
chr15	73116539	73136027
chr15	73122524	73136027
chr15	73176402	73178427
chr15	73570149	73573795
chr15	73699585	73704472
chr15	73702254	73703086
chr15	73702771	73703997
chr15	73708338	73711170
chr15	74635408	74640619
chr15	74635408	74675142
chr15	74640465	74656068
chr15	74655732	74675142

chr15	74671454	74675142
chr15	74923647	74926887
chr15	74923647	74929232
chr15	74926765	74929232
chr15	75043440	75048721
chr15	75394679	75400156
chr15	75400729	75401970
chr15	75400729	75422823
chr15	75407054	75413045
chr15	75407054	75422823
chr15	75409835	75413045
chr15	75409835	75422823
chr15	75411491	75413045
chr15	75412762	75414311
chr15	75412762	75422823
chr15	75414204	75422823
chr15	75422646	75430408
chr15	75473688	75490301
chr15	75479847	75527261
chr15	75490207	75509027
chr15	75490207	75527261
chr15	75505674	75517364
chr15	75505674	75527261
chr15	75517258	75524298
chr15	75517258	75527261
chr15	75523120	75524298
chr15	75523120	75527261
chr15	75601137	75621056
chr15	75609557	75609994
chr15	75609557	75617552
chr15	75609557	75621056
chr15	75617407	75621056
chr15	75854385	75873568
chr15	75854385	75879188
chr15	75854385	75883424
chr15	75859877	75869010
chr15	75859877	75873568
chr15	75859877	75883424
chr15	75873427	75876271
chr15	75876186	75883424
chr15	75883365	75897061
chr15	75913202	75930049
chr15	76225848	76286481
chr15	76231332	76295737
chr15	76274411	76286481

chr15	76274411	76292700
chr15	76274411	76295737
chr15	76283756	76287945
chr15	76283756	76292700
chr15	76283756	76295737
chr15	76285636	76292700
chr15	76287845	76292700
chr15	76287845	76295737
chr15	76292430	76295737
chr15	76574157	76665789
chr15	76621763	76665789
chr15	76753807	76767088
chr15	76753807	76775117
chr15	76764960	76767088
chr15	76766917	76795440
chr15	76771741	76775117
chr15	76795279	76862533
chr15	76841733	76862533
chr15	76841733	76883876
chr15	76857808	76862533
chr15	76857808	76883876
chr15	77052384	77056255
chr15	77252366	77285078
chr15	77252366	77286504
chr15	77284957	77286504
chr15	77467094	77471814
chr15	77477554	77479321
chr15	77478294	77479321
chr15	78001618	78017957
chr15	78024155	78030170
chr15	78044899	78054187
chr15	78157547	78161768
chr15	78157547	78163780
chr15	78160091	78162367
chr15	78289618	78295725
chr15	78462921	78466489
chr15	78462921	78473381
chr15	78462921	78478397
chr15	78462921	78497311
chr15	78465250	78466489
chr15	78470531	78471924
chr15	78470531	78478397
chr15	78471740	78478397
chr15	78490421	78490761
chr15	78542176	78546698

chr15	78544189	78545764
chr15	78544189	78546698
chr15	78544868	78545764
chr15	78544868	78546698
chr15	78580810	78588423
chr15	78878212	78886227
chr15	78886140	78887349
chr15	78887268	78894230
chr15	80075163	80122800
chr15	80098415	80121820
chr15	80098415	80122800
chr15	80120327	80122800
chr15	80978930	80982182
chr15	82138657	82152423
chr15	82214716	82230971
chr15	82214716	82241403
chr15	82219651	82229110
chr15	82229033	82241403
chr15	82241269	82252775
chr15	82400659	82408367
chr15	82538305	82540132
chr15	82552479	82557986
chr15	82852141	82854800
chr15	83030135	83041925
chr15	83030135	83056545
chr15	83041727	83050178
chr15	83151214	83158041
chr15	83151214	83164075
chr15	84538531	84546277
chr15	84542144	84555070
chr15	84647889	84653699
chr15	84680712	84687774
chr15	84680712	84691644
chr15	84687624	84691644
chr15	84797868	84799977
chr15	85064369	85067204
chr15	85064369	85083644
chr15	85067013	85083644
chr15	85075861	85098036
chr15	85097947	85109130
chr15	85097947	85117839
chr15	85109052	85116119
chr15	85113872	85115487
chr15	85113872	85121014
chr15	85123060	85126374

chr15	85655416	85669830
chr15	85662387	85682212
chr15	85664562	85669830
chr15	85664562	85682212
chr15	85664562	85693451
chr15	85669721	85710645
chr15	85710578	85719326
chr15	85715787	85719326
chr15	88527598	88536381
chr15	89113724	89116521
chr15	89247628	89260843
chr15	89247628	89264607
chr15	89268398	89274304
chr15	89268398	89285218
chr15	89273376	89285218
chr15	89292941	89301442
chr15	89299799	89303915
chr15	89307475	89314707
chr15	89323814	89333913
chr15	89584285	89585942
chr15	89600584	89616495
chr15	89642202	89649941
chr15	89790459	89793126
chr15	89837614	89889140
chr15	89877305	89889140
chr15	89888520	89889140
chr15	89908279	89910819
chr15	90067109	90073262
chr15	90217438	90219891
chr15	90217438	90221765
chr15	90230933	90231507
chr15	90251553	90253367
chr15	90426109	90429666
chr15	90426109	90433795
chr15	90426109	90441684
chr15	90439331	90441684
chr15	90439331	90443478
chr15	90439331	90467592
chr15	90441505	90443478
chr15	90443393	90456315
chr15	90466000	90466436
chr15	90466000	90467592
chr15	90486953	90492711
chr15	90491332	90492711
chr15	90492544	90494835

chr15	90540038	90604447
chr15	90638445	90641199
chr15	90747388	90754938
chr15	90765295	90769586
chr15	90766909	90769586
chr15	90766909	90785081
chr15	90769132	90769586
chr15	90803520	90804359
chr15	90947795	90948794
chr15	90974135	90980389
chr15	90976675	90981981
chr15	90979157	90980389
chr15	90979157	90981981
chr15	90980241	90984140
chr15	90999676	90999975
chr15	91000489	91003131
chr15	91000489	91014433
chr15	92885514	92892072
chr15	92924320	92927330
chr15	92924320	92929091
chr15	92927243	92939718
chr15	92945820	92967513
chr15	92946037	92967513
chr15	92948951	92967513
chr15	92953356	92956649
chr15	92953356	92967513
chr15	92967324	92974950
chr15	92991475	92997095
chr15	92996956	92998621
chr15	93004616	93014909
chr15	96269847	96290783
chr15	96282831	96290783
chr15	96285325	96290783
chr15	98891324	98896905
chr15	98891324	98899621
chr15	99199918	99245510
chr15	99218587	99221864
chr15	99218587	99228732
chr15	99276575	99287951
chr15	99333922	99363265
chr15	99598429	99633173
chr15	99598429	99645764
chr15	99645560	99675458
chr15	99671322	99675458
chr15	99671322	99690428

chr15	99993047	99997589
chr16	100371	119255
chr16	10969160	10982991
chr16	10977224	10982991
chr16	11020192	11024921
chr16	11020192	11044072
chr16	11020192	11051641
chr16	11020192	11061022
chr16	11020192	11126146
chr16	11051512	11126146
chr16	110524	130591
chr16	11120614	11126146
chr16	11553532	11556735
chr16	11698124	11700564
chr16	11698124	11721670
chr16	11721576	11734079
chr16	11721576	11736233
chr16	11730644	11736233
chr16	11733981	11736233
chr16	11752668	11758539
chr16	11763477	11765685
chr16	11774235	11779363
chr16	11774235	11782388
chr16	11779165	11782388
chr16	11841694	11847806
chr16	11846500	11846843
chr16	11846500	11847806
chr16	11846500	11850418
chr16	11875760	11887750
chr16	11875760	11896785
chr16	11883014	11887750
chr16	11883014	11891139
chr16	11883014	11898035
chr16	11885180	11887750
chr16	11886470	11887750
chr16	11886470	11898035
chr16	11894953	11896785
chr16	11894953	11897881
chr16	11894953	11898035
chr16	11896557	11898035
chr16	11999296	12027444
chr16	11999296	12078915
chr16	12027319	12052222
chr16	12061527	12078915
chr16	1314019	1320517

chr16	1315653	1320517
chr16	13934191	13935743
chr16	14079289	14140760
chr16	14482637	14629674
chr16	14552020	14555709
chr16	14552020	14584791
chr16	14552020	14586361
chr16	14552020	14593378
chr16	14552020	14604226
chr16	14580873	14584791
chr16	14580873	14604226
chr16	14586317	14604226
chr16	14586317	14617650
chr16	14586317	14627336
chr16	1459106	1465338
chr16	14593300	14604226
chr16	14593300	14610809
chr16	14599903	14609123
chr16	14599903	14617650
chr16	14606483	14617650
chr16	14606483	14627336
chr16	14617589	14629674
chr16	14644273	14648592
chr16	14644273	14662065
chr16	14661891	14665071
chr16	14853466	14866691
chr16	1494245	1503002
chr16	14997752	15004333
chr16	15004186	15030056
chr16	15038573	15048099
chr16	15065218	15074861
chr16	15068168	15071251
chr16	15068168	15073080
chr16	15072949	15076650
chr16	15072949	15092614
chr16	15083512	15092614
chr16	1557934	1571534
chr16	1557934	1607297
chr16	15581216	15583238
chr16	15615829	15617535
chr16	15620450	15623123
chr16	15664735	15667439
chr16	15677800	15687511
chr16	15691143	15696860
chr16	15694164	15696860

chr16	1580758	1607297
chr16	1586129	1588024
chr16	15873490	15884205
chr16	15879803	15884205
chr16	16007815	16016621
chr16	16009775	16016621
chr16	16014490	16016621
chr16	16033108	16048303
chr16	16033108	16052816
chr16	16033108	16056295
chr16	16033108	16068302
chr16	16044449	16071729
chr16	16044449	16102717
chr16	16052723	16056295
chr16	16052723	16071729
chr16	16071641	16079478
chr16	16076325	16086991
chr16	16079351	16086991
chr16	16086823	16090588
chr16	16086823	16102717
chr16	16106737	16115076
chr16	16121974	16125911
chr16	16124788	16125911
chr16	1625972	1632365
chr16	1625972	1637907
chr16	1625972	1641187
chr16	16277369	16278789
chr16	1632211	1641187
chr16	1729137	1729578
chr16	1743334	1748720
chr16	1747028	1748720
chr16	1809753	1823037
chr16	1816892	1820014
chr16	1816892	1823037
chr16	1822299	1823037
chr16	18543616	18549627
chr16	18591103	18592716
chr16	18794598	18798834
chr16	18797924	18798834
chr16	18815174	18819654
chr16	18819501	18828168
chr16	18819501	18830369
chr16	18828030	18834438
chr16	18829285	18830115
chr16	18834891	18845651

chr16	18836359	18845651
chr16	18841564	18845651
chr16	18841564	18850467
chr16	18845428	18850467
chr16	18849216	18850467
chr16	18858169	18859703
chr16	18870800	18885162
chr16	18876123	18885666
chr16	19037873	19040446
chr16	19073920	19075860
chr16	192926	199238
chr16	19532415	19537587
chr16	19710508	19715037
chr16	19730429	19735450
chr16	19733697	19735450
chr16	199061	208188
chr16	199061	220747
chr16	199061	227336
chr16	2048586	2050486
chr16	2050399	2055519
chr16	2054295	2058873
chr16	2056195	2057178
chr16	2056195	2058873
chr16	2056643	2057178
chr16	206003	208188
chr16	2065518	2080377
chr16	2070455	2074389
chr16	2075798	2080377
chr16	2079031	2081798
chr16	20802795	20803670
chr16	20813189	20816212
chr16	20821761	20828537
chr16	20825832	20833123
chr16	20827057	20828537
chr16	20911182	20915714
chr16	21463702	21473249
chr16	21612644	21625115
chr16	2175300	2175953
chr16	21962459	21971620
chr16	21962459	21972122
chr16	21962459	21976243
chr16	21962459	21980700
chr16	21968627	21976243
chr16	21971524	21976243
chr16	22248754	22251322

chr16	22313619	22315208
chr16	22349278	22349849
chr16	22429795	22435812
chr16	22432725	22435812
chr16	22481030	22482643
chr16	2262277	2263287
chr16	2262277	2264331
chr16	23079945	23082557
chr16	23102318	23108183
chr16	2317282	2317764
chr16	2317647	2319840
chr16	2317647	2329854
chr16	2328452	2329854
chr16	23392379	23417121
chr16	23392379	23418827
chr16	23406075	23417121
chr16	23413447	23417121
chr16	23413447	23418827
chr16	23413447	23445961
chr16	23416966	23418827
chr16	23442476	23445961
chr16	23525243	23552304
chr16	23529501	23529897
chr16	23544513	23552304
chr16	23559467	23562271
chr16	23559467	23567069
chr16	23562624	23567069
chr16	23621361	23623130
chr16	23629203	23630469
chr16	23642954	23661281
chr16	23642954	23665728
chr16	2431129	2439826
chr16	2439352	2439826
chr16	2439352	2443800
chr16	2439352	2445622
chr16	2448854	2449915
chr16	24553512	24559677
chr16	24561611	24564865
chr16	24750725	24777358
chr16	24758338	24777358
chr16	24776932	24797966
chr16	24804176	24809481
chr16	24935469	24949484
chr16	24949403	24959979
chr16	24952288	24970580

chr16	24959670	24970580
chr16	24964196	24970580
chr16	24964196	24979005
chr16	24968350	24970580
chr16	25161101	25170805
chr16	25164597	25170805
chr16	25164597	25175034
chr16	25170713	25175034
chr16	25252945	25255392
chr16	2557702	2561522
chr16	2557702	2566453
chr16	2566355	2583585
chr16	2566355	2586893
chr16	2577424	2581703
chr16	2577424	2583585
chr16	2577424	2586893
chr16	2581606	2586893
chr16	27213584	27215008
chr16	27233000	27235299
chr16	27340185	27346618
chr16	27346466	27352696
chr16	27483043	27489178
chr16	27489042	27492713
chr16	27494762	27495492
chr16	27528597	27545523
chr16	27533290	27545523
chr16	2756333	2757945
chr16	2757471	2757945
chr16	2757471	2758547
chr16	2757471	2759402
chr16	2757471	2760499
chr16	27701588	27708920
chr16	27703895	27708920
chr16	27749583	27751924
chr16	27754172	27761590
chr16	28101457	28101945
chr16	28101457	28107677
chr16	28101457	28125848
chr16	28111816	28125848
chr16	28121669	28132403
chr16	28125688	28132403
chr16	28132333	28156527
chr16	28146093	28156527
chr16	28146093	28169909
chr16	28152658	28156527

chr16	28152658	28169909
chr16	28166507	28169909
chr16	28166507	28181031
chr16	28169749	28177332
chr16	28169749	28181031
chr16	28175897	28181031
chr16	28486346	28488662
chr16	28487455	28489386
chr16	28488590	28489386
chr16	28723163	28727245
chr16	28829400	28831072
chr16	28880914	28882589
chr16	28897999	28898451
chr16	289439	293718
chr16	28955977	28956337
chr16	28958716	28959100
chr16	28979154	28983290
chr16	293487	298251
chr16	29385649	29394076
chr16	29457631	29458015
chr16	29553085	29554992
chr16	29554415	29560215
chr16	297055	298251
chr16	29799627	29800048
chr16	29799627	29802937
chr16	29830514	29835798
chr16	29833732	29834066
chr16	29833732	29835798
chr16	29835703	29836958
chr16	29876755	29878425
chr16	29879863	29888725
chr16	29895258	29895860
chr16	29910977	29912049
chr16	30067233	30070029
chr16	30200724	30201293
chr16	30298376	30301358
chr16	30364899	30365594
chr16	304303	314683
chr16	304303	347106
chr16	30652913	30654339
chr16	30664214	30666541
chr16	30664214	30667622
chr16	30707182	30723229
chr16	30716065	30716479
chr16	30720161	30721476

chr16	30728965	30729572
chr16	30728965	30734008
chr16	30733279	30734008
chr16	30733279	30734615
chr16	30764937	30765327
chr16	30766162	30766558
chr16	30927307	30942536
chr16	30942115	30942536
chr16	309972	314683
chr16	309972	347106
chr16	31111299	31112027
chr16	31120185	31128139
chr16	31182512	31184002
chr16	31188324	31189794
chr16	31189122	31190842
chr16	31297513	31297954
chr16	31461921	31465982
chr16	31473681	31473977
chr16	31722625	31723353
chr16	31884497	31885256
chr16	31884497	31890250
chr16	3476221	3479600
chr16	3504719	3508468
chr16	3504719	3512478
chr16	3504719	3523299
chr16	3506330	3512478
chr16	3656101	3656753
chr16	3674338	3679790
chr16	3675323	3689137
chr16	3677497	3679790
chr16	3679718	3689137
chr16	3679718	3690985
chr16	3738558	3740549
chr16	3738558	3745354
chr16	3739577	3745354
chr16	3740398	3751806
chr16	3749626	3770986
chr16	375132	376812
chr16	3767719	3770986
chr16	3777612	3778817
chr16	3791980	3793626
chr16	3791980	3810779
chr16	3810602	3851009
chr16	3977481	3983440
chr16	3979115	3983440

chr16	3988993	3993510
chr16	3992145	3993510
chr16	4261778	4262701
chr16	4405490	4413404
chr16	4441374	4443164
chr16	4441374	4448848
chr16	4448732	4450497
chr16	4466152	4469465
chr16	4650364	4652824
chr16	4650364	4657363
chr16	4651962	4657363
chr16	4651962	4665151
chr16	4657245	4668312
chr16	46603974	46604461
chr16	46608296	46618372
chr16	46615945	46617749
chr16	46615945	46618372
chr16	46617633	46618372
chr16	4664708	4673657
chr16	46661717	46663162
chr16	46661717	46674660
chr16	46668929	46677398
chr16	46674313	46677398
chr16	46677314	46679156
chr16	46680670	46683606
chr16	4671390	4673657
chr16	4677462	4683932
chr16	4681549	4683932
chr16	46897647	46900790
chr16	46897647	46906975
chr16	46906841	46916707
chr16	46959002	46967646
chr16	46964610	46967646
chr16	4700969	4724831
chr16	47086225	47132025
chr16	47109482	47122912
chr16	47109482	47129364
chr16	47109482	47132025
chr16	47122656	47132025
chr16	47128324	47132025
chr16	471294	489000
chr16	47218867	47365869
chr16	4724749	4727177
chr16	47258631	47313823
chr16	47311239	47313823

chr16	47311239	47365869
chr16	47311239	47375940
chr16	47428803	47459175
chr16	47451395	47459175
chr16	47463881	47503090
chr16	47463881	47515601
chr16	47463881	47547548
chr16	47463881	47589102
chr16	47463881	47610920
chr16	47463881	47669417
chr16	47497398	47503090
chr16	47497398	47515601
chr16	47497398	47547548
chr16	47497398	47580358
chr16	47497398	47594214
chr16	47497398	47596531
chr16	47497398	47610920
chr16	47497398	47669417
chr16	47580294	47589102
chr16	47587667	47610920
chr16	4760491	4766076
chr16	47641034	47698588
chr16	47660505	47669417
chr16	4811169	4812248
chr16	48252130	48258740
chr16	48252130	48262872
chr16	48252130	48270274
chr16	48252130	48277479
chr16	482524	489000
chr16	482524	503097
chr16	48256609	48262872
chr16	48256609	48277479
chr16	48256609	48303305
chr16	48256609	48334358
chr16	48258617	48262872
chr16	48258617	48277479
chr16	48277337	48303305
chr16	48296014	48334358
chr16	4831978	4832912
chr16	48347506	48348290
chr16	48546146	48553669
chr16	48553538	48562444
chr16	4857989	4861102
chr16	4871154	4872934
chr16	50068779	50079018

chr16	50068779	50086351
chr16	50075570	50094793
chr16	50078740	50086351
chr16	50086214	50100373
chr16	50086214	50102435
chr16	50094704	50102435
chr16	50211322	50217663
chr16	50214220	50217663
chr16	50216074	50225285
chr16	5027134	5028185
chr16	503003	519024
chr16	50333573	50334895
chr16	50333573	50340086
chr16	50333573	50354922
chr16	50350022	50354922
chr16	50548716	50621708
chr16	50749575	50754424
chr16	50754318	50786946
chr16	50786854	50793664
chr16	5079747	5081056
chr16	53222643	53227603
chr16	53228982	53243016
chr16	53228982	53250066
chr16	53235184	53243016
chr16	53235184	53247503
chr16	53235184	53250066
chr16	53235184	53254605
chr16	53254437	53255779
chr16	53254437	53268126
chr16	53254437	53274302
chr16	53255599	53268126
chr16	53255599	53274302
chr16	53255599	53293052
chr16	53267293	53268126
chr16	53273625	53274302
chr16	53285595	53293052
chr16	53285595	53307905
chr16	53291724	53308854
chr16	53303719	53306397
chr16	53303719	53307953
chr16	53307680	53308854
chr16	53307680	53318340
chr16	53314376	53315044
chr16	533945	536142
chr16	53439015	53447106

chr16	53439015	53451831
chr16	53439015	53453769
chr16	53442657	53447106
chr16	53442657	53454842
chr16	53454655	53470922
chr16	53479153	53480769
chr16	53498390	53500329
chr16	53610966	53622356
chr16	53619024	53622356
chr16	53636438	53641116
chr16	53636438	53649115
chr16	53686432	53692364
chr16	53810139	53826491
chr16	53810139	53844298
chr16	53810139	53888951
chr16	53810139	53934109
chr16	53825863	53844298
chr16	53844154	53879987
chr16	53844154	53888951
chr16	53873785	53879987
chr16	53873785	53888951
chr16	53873785	53934109
chr16	53879843	53888951
chr16	53888831	53934109
chr16	55525507	55551102
chr16	55532823	55549402
chr16	55534442	55551102
chr16	55537577	55579244
chr16	55545734	55551102
chr16	56367443	56369327
chr16	56385918	56389375
chr16	56385918	56414344
chr16	56389184	56405041
chr16	56401732	56414344
chr16	56407963	56409588
chr16	56458547	56467293
chr16	56466151	56475565
chr16	56470002	56470791
chr16	56501352	56506224
chr16	56509956	56514680
chr16	56748233	56758655
chr16	56758537	56805632
chr16	56818663	56823846
chr16	56836600	56839604
chr16	56837607	56839604

chr16	56837607	56841833
chr16	56838951	56839604
chr16	56935234	56936817
chr16	56939236	56940245
chr16	57110707	57137282
chr16	57119194	57119650
chr16	57123413	57127903
chr16	57164000	57172874
chr16	57164000	57173869
chr16	57167092	57173869
chr16	57172775	57173869
chr16	57204503	57217035
chr16	57204503	57231319
chr16	57216903	57221415
chr16	57216903	57227453
chr16	57216903	57231319
chr16	57216903	57235228
chr16	57227341	57231319
chr16	57246079	57249852
chr16	574065	576254
chr16	57451039	57452936
chr16	57521067	57526127
chr16	57762139	57764247
chr16	58002478	58010112
chr16	58009928	58017439
chr16	58164053	58196844
chr16	58174450	58186856
chr16	58174450	58196844
chr16	58186754	58196844
chr16	58523369	58530347
chr16	58525988	58530347
chr16	58536988	58538914
chr16	58543606	58546749
chr16	58549718	58560362
chr16	58555250	58560362
chr16	58574608	58575129
chr16	58574608	58576582
chr16	58574608	58583182
chr16	58574608	58599511
chr16	58576462	58583182
chr16	58578698	58585506
chr16	58580632	58581515
chr16	58580632	58583182
chr16	58582792	58583182
chr16	58585337	58588906

chr16	58585337	58599511
chr16	58586544	58587413
chr16	58586544	58599511
chr16	58709416	58716813
chr16	58716013	58716813
chr16	58716013	58719255
chr16	58716013	58723902
chr16	58718195	58719255
chr16	58722149	58723902
chr16	625431	626121
chr16	66517135	66531469
chr16	66517135	66549009
chr16	66517788	66549009
chr16	66528993	66541953
chr16	66528993	66549009
chr16	66608308	66610003
chr16	66622061	66636581
chr16	66725827	66729099
chr16	66727687	66729099
chr16	66730111	66736244
chr16	66732338	66749313
chr16	66805776	66806026
chr16	66805776	66810772
chr16	66805776	66823600
chr16	66805911	66809075
chr16	66810373	66816672
chr16	66813563	66823600
chr16	66816580	66823600
chr16	66938036	66939358
chr16	66963178	66964727
chr16	67036638	67066798
chr16	67036638	67082308
chr16	67036638	67082339
chr16	67066681	67082308
chr16	67066681	67082339
chr16	67120111	67132525
chr16	67139983	67140594
chr16	671696	672384
chr16	67229792	67238128
chr16	67236556	67237382
chr16	67438792	67443178
chr16	67540578	67541578
chr16	67571147	67612121
chr16	67571147	67621591
chr16	67571147	67626715

chr16	67610823	67612121
chr16	67610823	67621591
chr16	67610823	67628552
chr16	67611950	67620817
chr16	67620696	67629533
chr16	67626554	67629533
chr16	67628369	67629533
chr16	67727326	67738035
chr16	67727326	67744455
chr16	67865101	67868599
chr16	67947668	67950493
chr16	68025414	68038149
chr16	68025414	68049550
chr16	68025414	68054617
chr16	68025414	68056424
chr16	68038005	68054617
chr16	68038005	68056424
chr16	68053563	68056424
chr16	68056363	68066636
chr16	68056363	68071108
chr16	68121986	68126610
chr16	68121986	68167015
chr16	68121986	68174514
chr16	68121986	68183366
chr16	68121986	68191775
chr16	68126447	68167015
chr16	68157868	68167015
chr16	68157868	68174514
chr16	68157868	68183366
chr16	68157868	68191775
chr16	68166842	68183366
chr16	68166842	68191775
chr16	68174373	68183366
chr16	68174373	68191775
chr16	68181474	68183366
chr16	68181474	68191775
chr16	68190767	68214432
chr16	68266592	68273904
chr16	68266592	68275249
chr16	68266592	68287871
chr16	68274690	68287871
chr16	68287745	68291661
chr16	68321425	68329174
chr16	68859883	68863061
chr16	68859883	68867220

chr16	68859883	68880630
chr16	68859883	68909402
chr16	68867078	68878280
chr16	68867078	68880630
chr16	68875153	68880630
chr16	68875153	68902504
chr16	68907442	68909402
chr16	69136695	69143389
chr16	69136695	69150904
chr16	69139824	69143389
chr16	69139824	69154457
chr16	69143177	69154457
chr16	69150536	69150904
chr16	69150536	69154457
chr16	69155870	69163178
chr16	69155870	69167185
chr16	69160355	69167185
chr16	69165340	69167185
chr16	69245601	69260260
chr16	69245601	69270285
chr16	69245601	69284244
chr16	69260049	69284244
chr16	69361403	69367199
chr16	69361403	69368482
chr16	69366806	69368482
chr16	69366806	69370629
chr16	69366806	69372355
chr16	69368375	69372355
chr16	69370482	69372355
chr16	69372268	69385486
chr16	69384579	69385486
chr16	69626402	69655799
chr16	69626402	69695379
chr16	69647027	69659899
chr16	69653235	69655799
chr16	69653235	69659899
chr16	69669976	69677335
chr16	69669976	69684970
chr16	69718122	69718534
chr16	69748231	69752371
chr16	69776188	69799295
chr16	69776188	69842120
chr16	69786995	69799295
chr16	69798681	69799295
chr16	69871803	69888249

chr16	69908760	69917883
chr16	69931801	69934129
chr16	69982349	69982757
chr16	69982349	69986509
chr16	69982349	70014167
chr16	70010750	70014167
chr16	70010750	70018097
chr16	70021823	70031968
chr16	70036431	70043003
chr16	70120460	70127393
chr16	70120460	70130544
chr16	70130422	70132300
chr16	70130422	70136386
chr16	70143509	70148553
chr16	70144420	70153573
chr16	70219911	70222480
chr16	70255727	70261157
chr16	70255727	70268379
chr16	70258032	70259186
chr16	70258979	70262524
chr16	70258979	70268379
chr16	70261043	70262524
chr16	70261043	70268379
chr16	70261043	70270340
chr16	70261043	70271972
chr16	70264957	70265662
chr16	70267658	70268379
chr16	70269617	70282784
chr16	70276485	70277154
chr16	70276485	70282784
chr16	70314901	70316104
chr16	70314901	70325688
chr16	70366084	70366861
chr16	70463168	70463774
chr16	70463168	70465176
chr16	70468848	70469323
chr16	70508405	70519731
chr16	70514334	70517740
chr16	70526586	70529199
chr16	70526586	70530917
chr16	70526586	70532620
chr16	70526586	70544533
chr16	70526586	70548442
chr16	70530744	70539207
chr16	70532478	70539207

chr16	70532478	70541834
chr16	70532478	70544533
chr16	70532478	70570149
chr16	70535307	70541834
chr16	70538322	70544533
chr16	70541668	70544533
chr16	70541668	70548442
chr16	70541668	70557029
chr16	70548369	70555206
chr16	70556178	70557029
chr16	70556178	70561784
chr16	70563875	70565524
chr16	70565064	70567536
chr16	70565064	70569141
chr16	70567410	70568495
chr16	70567410	70569141
chr16	70567410	70570149
chr16	70568282	70570149
chr16	70731494	70744579
chr16	70762539	70763025
chr16	70772108	70786365
chr16	71652789	71681905
chr16	71655239	71656681
chr16	71655239	71658815
chr16	71656570	71664099
chr16	71656570	71679535
chr16	71658232	71679535
chr16	71658232	71684601
chr16	71658232	71714801
chr16	71663898	71667333
chr16	71663898	71676649
chr16	71667177	71672322
chr16	71669274	71676649
chr16	71672261	71676649
chr16	71676446	71679535
chr16	71676446	71681905
chr16	71676446	71690709
chr16	71676446	71714801
chr16	71678754	71681905
chr16	71679388	71690709
chr16	71681750	71684601
chr16	71684475	71690709
chr16	71684475	71714801
chr16	71690518	71702731
chr16	71690518	71714801

chr16	71702597	71714801
chr16	71738941	71739341
chr16	71738941	71745614
chr16	71745143	71745614
chr16	71745143	71746692
chr16	71745472	71750332
chr16	71746587	71750332
chr16	71746587	71753887
chr16	71756018	71764726
chr16	71761511	71769699
chr16	71765488	71774592
chr16	71915625	71917134
chr16	71915625	71922680
chr16	71915625	71923380
chr16	71916461	71921453
chr16	71920738	71921453
chr16	71920738	71923380
chr16	72088986	72090786
chr16	72096821	72098792
chr16	72284571	72349206
chr16	72284571	72368722
chr16	72522552	72555326
chr16	72522552	72570515
chr16	72523331	72570515
chr16	72811576	72812038
chr16	72950468	72960194
chr16	74300114	74302292
chr16	74336593	74360891
chr16	74338444	74338894
chr16	74348866	74350738
chr16	74353776	74360891
chr16	74456648	74457994
chr16	74456648	74459789
chr16	74457873	74459789
chr16	74459681	74463479
chr16	74472348	74477533
chr16	74474545	74477533
chr16	74474545	74494831
chr16	74480240	74483124
chr16	74485795	74491215
chr16	74485795	74493140
chr16	74485795	74494831
chr16	74491000	74493140
chr16	74494759	74496644
chr16	74496440	74508925

chr16	74503530	74508925
chr16	74503530	74532153
chr16	74626342	74632673
chr16	74626342	74636577
chr16	74626342	74637970
chr16	74626342	74644735
chr16	74632522	74636577
chr16	74632522	74637970
chr16	74636345	74637970
chr16	74644361	74644735
chr16	74644361	74652122
chr16	74644361	74661451
chr16	74649131	74652122
chr16	74649131	74661451
chr16	74651919	74661451
chr16	74674959	74675764
chr16	74682650	74691463
chr16	74885652	74886396
chr16	74885652	74909657
chr16	74885652	74909917
chr16	74892483	74904100
chr16	74908907	74909917
chr16	74915869	74916259
chr16	74917928	74922103
chr16	74923925	74942826
chr16	74923925	74951543
chr16	74938149	74942826
chr16	74938149	74949798
chr16	74938149	74951543
chr16	74949717	74956610
chr16	74951457	74956610
chr16	75603084	75622464
chr16	75608223	75608988
chr16	75612242	75622464
chr16	75620265	75620820
chr16	75634172	75640349
chr16	75640183	75641723
chr16	79424	83256
chr16	81011929	81014181
chr16	81351582	81357002
chr16	81351582	81365478
chr16	81356784	81357931
chr16	81607566	81664349
chr16	81652202	81664349
chr16	81670141	81678628

chr16	81699684	81705604
chr16	81854443	81859163
chr16	81891471	81895927
chr16	83906986	83912367
chr16	84099052	84102107
chr16	84129547	84135605
chr16	84733434	84745673
chr16	84740308	84745673
chr16	84758715	84768358
chr16	84758715	84775225
chr16	84764085	84768358
chr16	84979188	84981994
chr16	84990303	84996007
chr16	87342876	87347250
chr16	87415275	87418901
chr16	87419782	87460131
chr16	87726764	87762040
chr16	87730551	87755292
chr16	87748672	87756477
chr16	87748672	87762040
chr16	87836497	87851849
chr16	879569	911079
chr16	879569	954666
chr16	87975047	87981127
chr16	87975047	87984259
chr16	87975047	88018667
chr16	87981035	87984259
chr16	87984059	88006265
chr16	88004294	88006265
chr16	88004294	88018667
chr16	88004294	88027650
chr16	88004294	88038011
chr16	88006089	88038011
chr16	8801798	8804843
chr16	8801798	8806407
chr16	88018427	88027650
chr16	88018427	88038011
chr16	88027482	88038011
chr16	88027482	88065332
chr16	88033108	88038011
chr16	8804766	8813106
chr16	8811078	8813106
chr16	8811637	8813106
chr16	88485938	88489153
chr16	8858349	8859335

chr16	88598177	88598712
chr16	88598177	88611536
chr16	88599790	88611536
chr16	88608933	88611536
chr16	88622196	88624062
chr16	88625201	88628119
chr16	88627621	88628854
chr16	88627919	88628854
chr16	88698501	88701418
chr16	88804761	88807203
chr16	88831997	88836267
chr16	88835212	88841971
chr16	8903267	8904565
chr16	8906425	8910827
chr16	89098590	89101347
chr16	89098590	89102759
chr16	89098590	89114487
chr16	89100661	89114487
chr16	89100661	89133262
chr16	89133135	89146049
chr16	8919030	8921295
chr16	8919030	8930397
chr16	8920358	8930397
chr16	89220996	89222183
chr16	89288527	89317078
chr16	8929368	8930397
chr16	893006	954666
chr16	89305205	89313589
chr16	89305205	89317075
chr16	89305205	89317078
chr16	89418283	89431326
chr16	89510489	89513037
chr16	89510489	89532636
chr16	89524005	89526468
chr16	89524005	89532636
chr16	89531903	89532636
chr16	89544647	89548113
chr16	89544647	89550609
chr16	89560939	89561368
chr16	89589896	89591260
chr16	89689251	89692516
chr16	89733481	89737905
chr16	89739993	89742938
chr16	89744958	89748767
chr16	89748658	89762022

chr16	89752137	89762022
chr16	89758576	89762022
chr16	89758576	89767237
chr16	89761948	89792547
chr16	89769836	89770259
chr16	89791402	89792547
chr16	89791402	89803341
chr16	89795905	89805392
chr16	89883350	89885966
chr16	89892192	89896083
chr16	89892192	89900794
chr16	89895037	89900794
chr16	89895037	89907322
chr16	89895989	89900794
chr16	89978809	89981923
chr16	89988851	89992828
chr16	90027635	90040509
chr16	910930	954666
chr16	98144	100509
chr17	1050049	1070090
chr17	1050049	1091850
chr17	1050049	1100735
chr17	1067076	1070090
chr17	1067076	1083627
chr17	1067076	1091850
chr17	1069968	1083627
chr17	1072613	1100735
chr17	1079329	1100735
chr17	1083519	1091850
chr17	1083519	1100735
chr17	1091664	1100735
chr17	11990461	11992911
chr17	12054888	12081530
chr17	12054888	12095694
chr17	12054888	12113360
chr17	12054888	12129287
chr17	12081355	12095694
chr17	12081355	12113360
chr17	12095574	12113360
chr17	12908896	12919831
chr17	12908896	12929046
chr17	12908896	12952581
chr17	12943587	12944196
chr17	12949139	12952581
chr17	13000155	13002579

chr17	13010612	13013275
chr17	13010612	13017121
chr17	1354210	1362008
chr17	1354210	1365058
chr17	1354210	1369741
chr17	1361091	1362008
chr17	1361091	1365058
chr17	1361091	1369741
chr17	1364858	1369741
chr17	14074322	14077056
chr17	1467241	1475032
chr17	1470174	1478735
chr17	1479430	1479705
chr17	1480526	1480885
chr17	1521578	1558559
chr17	15327991	15331614
chr17	1534098	1538952
chr17	1534098	1541640
chr17	1534098	1553149
chr17	1534098	1558559
chr17	1538868	1553149
chr17	1548295	1553149
chr17	1548295	1558559
chr17	15545784	15547148
chr17	15545784	15553829
chr17	15545784	15555364
chr17	15605222	15615809
chr17	15612700	15615809
chr17	15677175	15680953
chr17	16001914	16006568
chr17	16034764	16039654
chr17	16034764	16040494
chr17	16034764	16048988
chr17	16057513	16058599
chr17	16057906	16058599
chr17	16061400	16064187
chr17	16071408	16073569
chr17	16079963	16101805
chr17	16098366	16101805
chr17	16098366	16118027
chr17	16137310	16139186
chr17	16137310	16146548
chr17	16139007	16143696
chr17	16143605	16171995
chr17	16149450	16171995

chr17	16158759	16165161
chr17	16158759	16171995
chr17	16164978	16171995
chr17	16186553	16194639
chr17	1655349	1656761
chr17	1675151	1676711
chr17	1676975	1681054
chr17	1678517	1681054
chr17	1679290	1679799
chr17	17171717	17180088
chr17	17177249	17180088
chr17	17214984	17217182
chr17	17214984	17219209
chr17	17254858	17260474
chr17	17254858	17264981
chr17	17261965	17264981
chr17	17866295	17869473
chr17	1786809	1787604
chr17	17869339	17907531
chr17	17879626	17893810
chr17	17879626	17907531
chr17	17884633	17893810
chr17	1799856	1804715
chr17	18021122	18028659
chr17	18024623	18028659
chr17	18229940	18238214
chr17	18282697	18291027
chr17	18285141	18285520
chr17	18285141	18291027
chr17	18285141	18294785
chr17	18290556	18291027
chr17	18290556	18302723
chr17	18292644	18294785
chr17	18292644	18299633
chr17	18292644	18302434
chr17	18292644	18306966
chr17	18294702	18306966
chr17	18299558	18306966
chr17	18305111	18306966
chr17	18306890	18308941
chr17	18329277	18353817
chr17	18330554	18340813
chr17	18330554	18348440
chr17	18330554	18353817
chr17	18340042	18340813

chr17	18340042	18348440
chr17	18340042	18353817
chr17	18347495	18348440
chr17	18347495	18353817
chr17	18348324	18353817
chr17	1842802	1844686
chr17	1842802	1853189
chr17	1842802	1872526
chr17	1842802	1875793
chr17	1842802	1877314
chr17	1842802	1895095
chr17	18514588	18517473
chr17	1875660	1877314
chr17	1875660	1883944
chr17	18789352	18798943
chr17	18797578	18804266
chr17	18865468	18865952
chr17	18865468	18867334
chr17	18865468	18872649
chr17	18865468	18882683
chr17	18865468	18889877
chr17	18865468	18911251
chr17	18865468	18923984
chr17	18867281	18877870
chr17	18872582	18877870
chr17	18882567	18911251
chr17	18911102	18928957
chr17	18923913	18928957
chr17	19281954	19283714
chr17	19309884	19329647
chr17	19542392	19548133
chr17	19542392	19549677
chr17	19542392	19551468
chr17	19546434	19549677
chr17	19547984	19560493
chr17	19555211	19560296
chr17	19560187	19560493
chr17	19560187	19567228
chr17	19651546	19657862
chr17	19651546	19671956
chr17	19671720	19673275
chr17	19795621	19804830
chr17	19816748	19846910
chr17	19841488	19865828
chr17	19909180	19924517

chr17	19909180	19963022
chr17	19920035	19936430
chr17	19920035	19939849
chr17	19924407	19931978
chr17	19931804	19963022
chr17	19936285	19958571
chr17	19939712	19947505
chr17	19939712	19958571
chr17	20227412	20232405
chr17	20227412	20232414
chr17	20227412	20247319
chr17	20227412	20306082
chr17	20231757	20232405
chr17	20231757	20232414
chr17	20245925	20260294
chr17	20245925	20306082
chr17	2040504	2041181
chr17	21004201	21004990
chr17	21006895	21021226
chr17	21006895	21028674
chr17	21017941	21021226
chr17	21017941	21028674
chr17	21300544	21302259
chr17	2172657	2188515
chr17	2186662	2188515
chr17	2282646	2283735
chr17	2292551	2292977
chr17	2315556	2323345
chr17	2375491	2377953
chr17	2394042	2395454
chr17	2438108	2467876
chr17	2438108	2473664
chr17	2464207	2473664
chr17	2464207	2477885
chr17	2464207	2502331
chr17	2638098	2672757
chr17	2665371	2667198
chr17	2665371	2670331
chr17	2665371	2680320
chr17	2666015	2672757
chr17	2691608	2692097
chr17	2691760	2704564
chr17	27301787	27304911
chr17	27301787	27309272
chr17	27303366	27309272

chr17	27303366	27310174
chr17	27303366	27311616
chr17	27304779	27309272
chr17	27304779	27311616
chr17	28122602	28172618
chr17	28163542	28172618
chr17	28163542	28185265
chr17	281767	321415
chr17	281767	327579
chr17	28349082	28355876
chr17	28350437	28351800
chr17	28351663	28353791
chr17	28352911	28355876
chr17	28358915	28360847
chr17	28558475	28561629
chr17	28591697	28593066
chr17	28618790	28624390
chr17	28642868	28644187
chr17	28678544	28678963
chr17	28682726	28683816
chr17	28688090	28690229
chr17	28689353	28690229
chr17	28748016	28748666
chr17	28917284	28919275
chr17	28917284	28921808
chr17	28917284	28927063
chr17	28921687	28924302
chr17	28921687	28927063
chr17	29103598	29107189
chr17	29103598	29114097
chr17	29267497	29287716
chr17	29451454	29467216
chr17	29451454	29475771
chr17	29451454	29482288
chr17	29451454	29502723
chr17	29451454	29510992
chr17	29451454	29530619
chr17	29451454	29534300
chr17	29467144	29491865
chr17	29475669	29482288
chr17	29475669	29489757
chr17	29475669	29517656
chr17	29480367	29491865
chr17	29482196	29491865
chr17	29482196	29498521

chr17	29489663	29491865
chr17	29507895	29510992
chr17	29510863	29522519
chr17	29517452	29522519
chr17	29522279	29534300
chr17	29530406	29534300
chr17	29571590	29572057
chr17	29581337	29582144
chr17	2962669	2964068
chr17	29648143	29650800
chr17	29648143	29655607
chr17	29648143	29672129
chr17	29655560	29672129
chr17	29666866	29684684
chr17	29666866	29703062
chr17	29671934	29677741
chr17	29676819	29684684
chr17	29676819	29703062
chr17	29684562	29695523
chr17	29684562	29703062
chr17	2991296	3008145
chr17	2991296	3020595
chr17	2995336	3008145
chr17	3005368	3008145
chr17	3020476	3027070
chr17	3026007	3027070
chr17	30266884	30286902
chr17	30272560	30289482
chr17	30384988	30431881
chr17	30443801	30451846
chr17	30445690	30456351
chr17	30451710	30456526
chr17	30456461	30461311
chr17	30481142	30484770
chr17	30481142	30490217
chr17	30481142	30492753
chr17	30481142	30510909
chr17	30484213	30492753
chr17	30607551	30610942
chr17	30785918	30792572
chr17	30785918	30796337
chr17	30785918	30804108
chr17	30792439	30793672
chr17	30792439	30804108
chr17	30797310	30804108

chr17	30843912	30844916
chr17	30843912	30860612
chr17	30855142	30860612
chr17	30858160	30865800
chr17	30858160	30869646
chr17	30860432	30869646
chr17	30865703	30869646
chr17	30868332	30879487
chr17	30984616	30988106
chr17	31155982	31163376
chr17	31155982	31169997
chr17	31155982	31181785
chr17	31155982	31182665
chr17	31155982	31206371
chr17	31155982	31219118
chr17	31163185	31182665
chr17	31169890	31182665
chr17	31169890	31206371
chr17	31169890	31219118
chr17	31200421	31206371
chr17	31200421	31214585
chr17	31200421	31227606
chr17	31223443	31227606
chr17	31248983	31260515
chr17	31248983	31265339
chr17	31252937	31259129
chr17	31252937	31265339
chr17	31252937	31326252
chr17	31258343	31260515
chr17	31258343	31265339
chr17	31260368	31265339
chr17	31349119	31360703
chr17	31352256	31360703
chr17	31358968	31360703
chr17	31865365	31875413
chr17	31865365	31878327
chr17	31865365	31878781
chr17	31878249	31899730
chr17	31884423	31887313
chr17	31884423	31892794
chr17	31940285	31966196
chr17	31940285	31973231
chr17	31940285	31976614
chr17	31940285	31988497
chr17	31940421	31976614

chr17	319419	321415
chr17	319419	327579
chr17	31947616	31976614
chr17	31966146	31976614
chr17	31966146	31988497
chr17	31973145	31988497
chr17	31975481	31988497
chr17	31982998	31988497
chr17	31982998	31994721
chr17	31993241	31995762
chr17	31993864	31994721
chr17	31993864	31995762
chr17	31993864	31996877
chr17	32027768	32031458
chr17	32171042	32176213
chr17	32171042	32194107
chr17	32173830	32183272
chr17	32173830	32192299
chr17	32173830	32194107
chr17	32173830	32201056
chr17	32182756	32194107
chr17	32193135	32199550
chr17	32200955	32202900
chr17	32333416	32335442
chr17	32360597	32362984
chr17	32365329	32368014
chr17	32446944	32464578
chr17	34983001	34986131
chr17	35014739	35017606
chr17	35016369	35017606
chr17	35103253	35106481
chr17	35103253	35107447
chr17	35137000	35137890
chr17	35139234	35140066
chr17	35258650	35261096
chr17	35360242	35363826
chr17	35594007	35608387
chr17	35598229	35608387
chr17	35605704	35608387
chr17	35624396	35627726
chr17	35624396	35650789
chr17	35627384	35627726
chr17	35650529	35717349
chr17	35657598	35682824
chr17	35671753	35674321

chr17	35671753	35717349
chr17	3580456	3591116
chr17	35817715	35822833
chr17	35817715	35824198
chr17	35820023	35820437
chr17	35820023	35822833
chr17	35820331	35822833
chr17	35834565	35838553
chr17	35834565	35845038
chr17	35836131	35838553
chr17	3585767	3588367
chr17	35920186	35930255
chr17	35922576	35930255
chr17	3621236	3624231
chr17	36503096	36505404
chr17	36509061	36511455
chr17	36513428	36515990
chr17	36513628	36515990
chr17	36525227	36527699
chr17	36525227	36528202
chr17	36525227	36534104
chr17	36554819	36557336
chr17	36554819	36567776
chr17	36557082	36560871
chr17	36577982	36587245
chr17	36585299	36586198
chr17	36585299	36587245
chr17	3664194	3664798
chr17	36952885	36953907
chr17	36986616	37021014
chr17	36986616	37031685
chr17	36988518	36990857
chr17	37019004	37031685
chr17	37111530	37113265
chr17	37111530	37151421
chr17	37129364	37155780
chr17	37149863	37155780
chr17	37149863	37181356
chr17	37149863	37193415
chr17	37149863	37210490
chr17	37151300	37155780
chr17	37179259	37181356
chr17	37179259	37193415
chr17	37179259	37207800
chr17	37221723	37226452

chr17	37221723	37289528
chr17	37243370	37246976
chr17	37244587	37246976
chr17	37246825	37253036
chr17	37248010	37253036
chr17	37270750	37289528
chr17	37275950	37283405
chr17	37283266	37284970
chr17	37283266	37289528
chr17	37284837	37289528
chr17	37330172	37339850
chr17	37411268	37465541
chr17	37426949	37440662
chr17	37437737	37442652
chr17	37437737	37444768
chr17	37440504	37442652
chr17	37440504	37444768
chr17	37440504	37462121
chr17	37440504	37465541
chr17	37442563	37462121
chr17	37458523	37465541
chr17	37535978	37538420
chr17	37535978	37539245
chr17	37538323	37540543
chr17	37568790	37571987
chr17	37570636	37585430
chr17	37571790	37585430
chr17	37576340	37585430
chr17	37619767	37621520
chr17	37620872	37632298
chr17	37625894	37628672
chr17	3814321	3816270
chr17	3814321	3818572
chr17	3816115	3822052
chr17	3821952	3826215
chr17	38361710	38366417
chr17	38361710	38368050
chr17	38361710	38377842
chr17	38395308	38395998
chr17	38462845	38463382
chr17	38466166	38500628
chr17	38736089	38740442
chr17	38777686	38786920
chr17	38784242	38786920
chr17	38806764	38815097

chr17	39202316	39203109
chr17	39268826	39281463
chr17	39281388	39285573
chr17	39281388	39297195
chr17	39297126	39301075
chr17	39297126	39303639
chr17	39297126	39343241
chr17	39415025	39419918
chr17	39419716	39424738
chr17	39423326	39424738
chr17	39423326	39427790
chr17	39424626	39432016
chr17	39424626	39439193
chr17	39427700	39432016
chr17	39427700	39439193
chr17	39427700	39440677
chr17	39427700	39447904
chr17	39431114	39434320
chr17	39439164	39443628
chr17	3947390	3951685
chr17	39490556	39492890
chr17	39490556	39494694
chr17	39490556	39511630
chr17	39490556	39526316
chr17	39515730	39526316
chr17	39684596	39686019
chr17	39708320	39710481
chr17	39723318	39723660
chr17	39723911	39725204
chr17	39908957	39909924
chr17	39986574	39995295
chr17	39989738	39990197
chr17	39994953	39995295
chr17	39994953	39995527
chr17	40035116	40036154
chr17	40053297	40053635
chr17	40096442	40097403
chr17	4013448	4019769
chr17	40161752	40162901
chr17	40169323	40169662
chr17	4021128	4022828
chr17	40256390	40265146
chr17	40256390	40277184
chr17	40260534	40262641
chr17	40273789	40277184

chr17	40289407	40291668
chr17	40291057	40291668
chr17	40293455	40294503
chr17	40295355	40296767
chr17	4032125	4033002
chr17	4032125	4034292
chr17	40391505	40392737
chr17	40391505	40395539
chr17	40395448	40396465
chr17	40404790	40411455
chr17	40406383	40406942
chr17	40406383	40408630
chr17	40406383	40413625
chr17	40411108	40413625
chr17	40411644	40413292
chr17	40412758	40413625
chr17	40413194	40416068
chr17	4049707	4051043
chr17	4051970	4064829
chr17	4051970	4067242
chr17	4064360	4067242
chr17	4066446	4067242
chr17	4066446	4072756
chr17	4081375	4086655
chr17	4085669	4086655
chr17	4102316	4117166
chr17	4109652	4124051
chr17	4114298	4124051
chr17	41378955	41379731
chr17	4169197	4172680
chr17	4172555	4179877
chr17	4173353	4174056
chr17	4173353	4182349
chr17	4173908	4179057
chr17	41803286	41811284
chr17	41828789	41832477
chr17	4183397	4185046
chr17	41871688	41884274
chr17	41883121	41886308
chr17	4189381	4209947
chr17	41898630	41905658
chr17	41898630	41910284
chr17	41909509	41910284
chr17	4197372	4209947
chr17	41981854	41982401

chr17	41987818	41990382
chr17	41996310	41997239
chr17	4206320	4209947
chr17	4206320	4217118
chr17	4209823	4235890
chr17	4216982	4242448
chr17	42207557	42217464
chr17	42217159	42217464
chr17	42217159	42232137
chr17	42223381	42227685
chr17	42227528	42232137
chr17	42324710	42325061
chr17	42329409	42329776
chr17	42337434	42338812
chr17	42345558	42348517
chr17	42345558	42348539
chr17	42346568	42348539
chr17	4235771	4242448
chr17	42468009	42470218
chr17	42480666	42487367
chr17	42480666	42494473
chr17	42498923	42501304
chr17	42500706	42501304
chr17	42538338	42541206
chr17	42568836	42569303
chr17	42568836	42570183
chr17	42612074	42613073
chr17	42703739	42708083
chr17	42703739	42722915
chr17	42703739	42724424
chr17	42706006	42708083
chr17	42706006	42719207
chr17	42712288	42713389
chr17	42727634	42728952
chr17	42727634	42730918
chr17	42787277	42788797
chr17	42813947	42820841
chr17	42818220	42820841
chr17	4282797	4289408
chr17	4282797	4296814
chr17	4282797	4307123
chr17	42834343	42839380
chr17	42838092	42839012
chr17	42838730	42839380
chr17	4289229	4296814

chr17	4296716	4307123
chr17	43047642	43057135
chr17	43049120	43057135
chr17	43049120	43074521
chr17	43057051	43063951
chr17	43063332	43074521
chr17	43067607	43074521
chr17	43095845	43104956
chr17	43097243	43115779
chr17	43097243	43124115
chr17	43099774	43104956
chr17	43099774	43115779
chr17	43099774	43124115
chr17	43104121	43115779
chr17	43104121	43124115
chr17	43177935	43186444
chr17	43377670	43381660
chr17	43489448	43493040
chr17	43498861	43500103
chr17	43498861	43504825
chr17	43504643	43508520
chr17	43508338	43520267
chr17	43521368	43522226
chr17	43808955	43811205
chr17	43808955	43818110
chr17	43816037	43824005
chr17	44110728	44117704
chr17	44145202	44152777
chr17	44150960	44152777
chr17	44154555	44155411
chr17	44154555	44157941
chr17	44195096	44196079
chr17	44210123	44218296
chr17	44215653	44216704
chr17	44405920	44436545
chr17	44405920	44464544
chr17	44424348	44436545
chr17	44424348	44464544
chr17	44424348	44474903
chr17	44435064	44464544
chr17	44435064	44474903
chr17	44729904	44734163
chr17	44852408	44855004
chr17	44857074	44860543
chr17	44857074	44865065

chr17	44862712	44865065
chr17	44875933	44880644
chr17	44879555	44883724
chr17	44933922	44936358
chr17	45081643	45086652
chr17	45081643	45096285
chr17	45120324	45121372
chr17	45237280	45239065
chr17	45273502	45274593
chr17	4542463	4543165
chr17	45450617	45454272
chr17	4547957	4549442
chr17	45991459	45996664
chr17	46038537	46094701
chr17	46050532	46067667
chr17	46050532	46082542
chr17	46050532	46094701
chr17	46066536	46082542
chr17	46066536	46094701
chr17	46066536	46172232
chr17	46082440	46094701
chr17	46094559	46172232
chr17	46674413	46728934
chr17	46692902	46704854
chr17	46710962	46713986
chr17	4682501	4691442
chr17	46935028	46938704
chr17	47129392	47143982
chr17	47132256	47138891
chr17	47132256	47142436
chr17	47137151	47138891
chr17	47137151	47143982
chr17	47137151	47151918
chr17	47137151	47172064
chr17	47138738	47151918
chr17	47141852	47151918
chr17	47143882	47151918
chr17	47143882	47154786
chr17	47143882	47172064
chr17	47151805	47170042
chr17	47156912	47170042
chr17	47157229	47158303
chr17	47169916	47172064
chr17	47326225	47345098
chr17	47328268	47344292

chr17	47328268	47345098
chr17	47335195	47345098
chr17	47370436	47377903
chr17	47370436	47379253
chr17	4738407	4738920
chr17	47391436	47414919
chr17	47394024	47404633
chr17	47402131	47409691
chr17	47402131	47414919
chr17	47585499	47590881
chr17	47587229	47599739
chr17	47587229	47601747
chr17	47590716	47601747
chr17	47590716	47605552
chr17	47590716	47613725
chr17	47596352	47599739
chr17	47599675	47605552
chr17	47601607	47605552
chr17	47601607	47619164
chr17	47603914	47613725
chr17	47613668	47619164
chr17	47618349	47619164
chr17	47618349	47619784
chr17	47656859	47658660
chr17	47663088	47665158
chr17	47663088	47668410
chr17	47663088	47670832
chr17	47663088	47674782
chr17	47664158	47668410
chr17	47664158	47670832
chr17	47664158	47674782
chr17	47664158	47678413
chr17	47664158	47680669
chr17	47669677	47674782
chr17	47669677	47677127
chr17	47676408	47677127
chr17	47678045	47678413
chr17	47698594	47699697
chr17	47826636	47828670
chr17	48050606	48056598
chr17	48112030	48113401
chr17	48118703	48119186
chr17	4880983	4881257
chr17	48848063	48860449
chr17	48910807	48922937

chr17	48916075	48921272
chr17	48916075	48922937
chr17	4895697	4896813
chr17	49038167	49040091
chr17	49041377	49042377
chr17	49311311	49312042
chr17	49311311	49312845
chr17	4938338	4938975
chr17	4952794	4953845
chr17	49611279	49619385
chr17	49618980	49622876
chr17	49621945	49622876
chr17	4969629	4986286
chr17	49719759	49720436
chr17	49805362	49809208
chr17	49805362	49811574
chr17	4982756	4986286
chr17	5003997	5007342
chr17	5006914	5014837
chr17	50073915	50081408
chr17	50075598	50076681
chr17	50075598	50077447
chr17	50075598	50078926
chr17	5013652	5014837
chr17	50354407	50356773
chr17	50696939	50700716
chr17	50736959	50741731
chr17	50736959	50743810
chr17	50741101	50741731
chr17	50970706	50987237
chr17	50970706	50989872
chr17	50979745	50987237
chr17	50979745	50989872
chr17	50979745	50995533
chr17	50982523	50985778
chr17	50982523	50987237
chr17	50982523	50990668
chr17	50984922	50987237
chr17	50990449	50993935
chr17	50993763	50995533
chr17	50993763	51001845
chr17	50998443	50999717
chr17	51014231	51021365
chr17	51014231	51056482
chr17	51020158	51021365

chr17	51020158	51031722
chr17	51020158	51041651
chr17	51031680	51056482
chr17	51041500	51056482
chr17	51041500	51079704
chr17	51047374	51056482
chr17	51056411	51079704
chr17	51201591	51203228
chr17	51206887	51217416
chr17	51217333	51220463
chr17	51217333	51225209
chr17	51218929	51225209
chr17	51263273	51268904
chr17	51263273	51273450
chr17	51263273	51288203
chr17	51268836	51280479
chr17	51273361	51280479
chr17	51275865	51280479
chr17	5214049	5215293
chr17	5214049	5223484
chr17	5308693	5332152
chr17	5308693	5346925
chr17	532841	537176
chr17	5335183	5338138
chr17	5335183	5346925
chr17	5338018	5361675
chr17	5338018	5363016
chr17	5346789	5350629
chr17	5350450	5365238
chr17	5354358	5363016
chr17	5354358	5368468
chr17	5361207	5363016
chr17	5361207	5365238
chr17	5361207	5368468
chr17	5361207	5378232
chr17	5365121	5368468
chr17	5388801	5399650
chr17	5388801	5404246
chr17	5388801	5414134
chr17	5394890	5414134
chr17	5399551	5404246
chr17	5410702	5416682
chr17	5448808	5462546
chr17	5450202	5450934
chr17	5460938	5462546

chr17	5460938	5463689
chr17	54905488	54915862
chr17	54905488	54939020
chr17	54905488	54950126
chr17	54912665	54915862
chr17	54912665	54947312
chr17	54913747	54930206
chr17	54930072	54939020
chr17	54938923	54947312
chr17	54938923	54950126
chr17	54985613	54990957
chr17	54985613	55000883
chr17	54999344	55007597
chr17	55031167	55047154
chr17	55031167	55073076
chr17	55031167	55081183
chr17	551871	586364
chr17	562502	655953
chr17	56844018	56845809
chr17	56844018	56848065
chr17	56844018	56849232
chr17	56844018	56862251
chr17	56847921	56856625
chr17	56847921	56858665
chr17	56847921	56862251
chr17	56848695	56856625
chr17	56848695	56858665
chr17	56848695	56862251
chr17	56895342	56899180
chr17	56895342	56904488
chr17	56901418	56904488
chr17	57105440	57119044
chr17	57114458	57116261
chr17	58005800	58006527
chr17	58216087	58216736
chr17	58613420	58616305
chr17	586269	628231
chr17	586269	631628
chr17	586269	661895
chr17	58694930	58696859
chr17	58694930	58709990
chr17	58696692	58709990
chr17	58696692	58720812
chr17	58696692	58724100
chr17	58703195	58720812

chr17	58703195	58724100
chr17	58955648	58972925
chr17	58969538	58972925
chr17	58999380	59001714
chr17	58999380	59017424
chr17	58999380	59070947
chr17	59012327	59017424
chr17	59012327	59028723
chr17	59012327	59041898
chr17	59015609	59017424
chr17	59017295	59041898
chr17	59031895	59047819
chr17	59031895	59057054
chr17	59041812	59057054
chr17	59047682	59049393
chr17	59047682	59070947
chr17	59051213	59057054
chr17	59064354	59070947
chr17	59064354	59084089
chr17	59070822	59084089
chr17	59075646	59081219
chr17	59075646	59084089
chr17	59079753	59084089
chr17	59081096	59088407
chr17	59169747	59195443
chr17	59169747	59197789
chr17	59185053	59197789
chr17	59193491	59197789
chr17	59197543	59197789
chr17	59257121	59267174
chr17	59257121	59270995
chr17	59570517	59577365
chr17	59573739	59575471
chr17	59586152	59588053
chr17	59647397	59651316
chr17	59648239	59660588
chr17	59651202	59656027
chr17	59651202	59664909
chr17	59655853	59661643
chr17	59664786	59679519
chr17	59682906	59683756
chr17	59731420	59738947
chr17	59731420	59765138
chr17	59731420	59773885
chr17	59737452	59808876

chr17	59737452	59817773
chr17	59738836	59817773
chr17	59764970	59773885
chr17	59764970	59817773
chr17	59880893	59891041
chr17	59910561	59914703
chr17	59910561	59936541
chr17	59912683	59914703
chr17	59912683	59931722
chr17	59926434	59930174
chr17	59934433	59936541
chr17	59935192	59940943
chr17	60070705	60073886
chr17	60180544	60183453
chr17	60181323	60185651
chr17	60207020	60208828
chr17	60207020	60214774
chr17	60236137	60271481
chr17	60252380	60269557
chr17	60252380	60294801
chr17	60265411	60271481
chr17	60265411	60294801
chr17	60269449	60271481
chr17	60288522	60301704
chr17	60288522	60345608
chr17	60294682	60301704
chr17	60294682	60345608
chr17	60451879	60479271
chr17	60460662	60462061
chr17	60460662	60479271
chr17	60460662	60500487
chr17	60464020	60494617
chr17	60623520	60633977
chr17	60623520	60648082
chr17	60623520	60656841
chr17	60633852	60648082
chr17	60679452	60689761
chr17	60679452	60709325
chr17	60679452	60747279
chr17	60679452	60808076
chr17	60679452	60910702
chr17	6080370	6095223
chr17	6087989	6095223
chr17	60902619	60924500
chr17	60989970	61040892

chr17	61037888	61075020
chr17	61074919	61084564
chr17	6109606	6110935
chr17	61715950	61744591
chr17	61715950	61776562
chr17	61743012	61776562
chr17	61743012	61780401
chr17	61743012	61784424
chr17	61743012	61793729
chr17	61743012	61808757
chr17	61744431	61780401
chr17	61776400	61780401
chr17	61776400	61781005
chr17	61776400	61799299
chr17	61776400	61801474
chr17	61780260	61808757
chr17	61780839	61801474
chr17	61793596	61861569
chr17	61847100	61861569
chr17	61857057	61859907
chr17	61877886	61881171
chr17	61881006	61904585
chr17	61889785	61891689
chr17	61889785	61897739
chr17	61904459	61919513
chr17	61904459	61925099
chr17	61919399	61925099
chr17	61921724	61925099
chr17	61950824	61956481
chr17	61952964	61961779
chr17	61960866	61962971
chr17	61965005	61968258
chr17	61972726	61983114
chr17	61982197	61985090
chr17	61984170	61985090
chr17	61984170	61987128
chr17	61987006	61995365
chr17	61992539	62011233
chr17	62010549	62063301
chr17	62029540	62035608
chr17	62029540	62052705
chr17	62029540	62063301
chr17	62033786	62035608
chr17	623532	627316
chr17	623532	628231

chr17	623532	631628
chr17	623532	653410
chr17	623532	661895
chr17	62520772	62524331
chr17	62520772	62536337
chr17	62524235	62536337
chr17	62524235	62553755
chr17	62536169	62565137
chr17	62552301	62553755
chr17	62552301	62565137
chr17	62560015	62565137
chr17	62578476	62580192
chr17	62586134	62606241
chr17	62664547	62667533
chr17	62666093	62667533
chr17	63099174	63200957
chr17	63193990	63200957
chr17	63237813	63267873
chr17	63267747	63355390
chr17	63314387	63319090
chr17	63340100	63355390
chr17	63340100	63389544
chr17	63340100	63398914
chr17	63351249	63355390
chr17	63379717	63395928
chr17	63578469	63579448
chr17	63578469	63579943
chr17	63578469	63583711
chr17	63578469	63589237
chr17	63585210	63589237
chr17	63604533	63606440
chr17	63632680	63652656
chr17	63632680	63657907
chr17	63634708	63657907
chr17	63704340	63714108
chr17	63751939	63752799
chr17	63751939	63754518
chr17	63756239	63766194
chr17	63760913	63764190
chr17	63760913	63764847
chr17	63760913	63766194
chr17	63764015	63764847
chr17	63764015	63766194
chr17	63787033	63792562
chr17	63787033	63800617

chr17	63792411	63800617
chr17	63792411	63806654
chr17	63792411	63809659
chr17	63798037	63800617
chr17	63800467	63805175
chr17	63800467	63807900
chr17	63805070	63809659
chr17	63835411	63837044
chr17	64171099	64188415
chr17	64171099	64188436
chr17	64480288	64482999
chr17	64485727	64493021
chr17	64519320	64522895
chr17	64519320	64529427
chr17	64521401	64522895
chr17	64522701	64526200
chr17	64551583	64562966
chr17	64551583	64586170
chr17	64555819	64571956
chr17	64557607	64578576
chr17	64561499	64562966
chr17	64561499	64571956
chr17	64561499	64580991
chr17	64561499	64586170
chr17	64562770	64571956
chr17	64562770	64583544
chr17	64571797	64583544
chr17	64571797	64586170
chr17	64571797	64591149
chr17	64571797	64598490
chr17	64571797	64606640
chr17	64578491	64580991
chr17	64578491	64606640
chr17	64580788	64598490
chr17	64583460	64586170
chr17	64583460	64606640
chr17	64586085	64598490
chr17	64591083	64593573
chr17	64591083	64598490
chr17	64591083	64606640
chr17	64751806	64764548
chr17	64754004	64762710
chr17	64786566	64797409
chr17	64821766	64822393
chr17	653290	655953

chr17	653290	661895
chr17	65689128	65750724
chr17	65743067	65750724
chr17	6589778	6590630
chr17	6589778	6600458
chr17	6594971	6600458
chr17	6594971	6612148
chr17	66027500	66030023
chr17	66053735	66183307
chr17	66062962	66070001
chr17	66062962	66096632
chr17	6606872	6610160
chr17	66096250	66183307
chr17	66129745	66183307
chr17	66175043	66183307
chr17	66176829	66183307
chr17	6620787	6635171
chr17	6628116	6635171
chr17	66687110	66689047
chr17	66687110	66742760
chr17	66732687	66742760
chr17	66732687	66774067
chr17	67114323	67124014
chr17	67137930	67178926
chr17	67145742	67151224
chr17	67166477	67167796
chr17	67193966	67203443
chr17	67215898	67218822
chr17	67342185	67344780
chr17	67344605	67350336
chr17	67347115	67350336
chr17	67347335	67348654
chr17	67724048	67736023
chr17	67853939	67866687
chr17	67866463	67886316
chr17	67886127	67904840
chr17	67891843	67893725
chr17	67891843	67894165
chr17	67891843	67904840
chr17	67920014	67922990
chr17	67928354	67929487
chr17	67931910	67944372
chr17	67945408	67948306
chr17	67945408	67964404
chr17	67959540	67975958

chr17	68037109	68045921
chr17	68114421	68125480
chr17	68250187	68251178
chr17	68306942	68385172
chr17	68368547	68370524
chr17	68444492	68450897
chr17	68444492	68452992
chr17	68522755	68524958
chr17	69273958	69284072
chr17	697417	710613
chr17	7224145	7224552
chr17	7224320	7224884
chr17	72648802	72649268
chr17	7313028	7314597
chr17	73196899	73203793
chr17	73202999	73203793
chr17	7321864	7322866
chr17	7321864	7323123
chr17	74243373	74250357
chr17	7465710	7467327
chr17	7499038	7499491
chr17	7499038	7503791
chr17	7499038	7508475
chr17	7503120	7503490
chr17	75040091	75042659
chr17	75042180	75042659
chr17	75147555	75148671
chr17	75148528	75149185
chr17	75174751	75181188
chr17	75208526	75209985
chr17	75208526	75213119
chr17	75208526	75215823
chr17	75208526	75232968
chr17	75238651	75240412
chr17	75238651	75241696
chr17	75242335	75243570
chr17	75242335	75246796
chr17	75277352	75278335
chr17	75277352	75283593
chr17	75321658	75326020
chr17	75485427	75486426
chr17	75660954	75661708
chr17	75812111	75813878
chr17	75899157	75902154
chr17	75899157	75904719

chr17	75917731	75918269
chr17	7594237	7594757
chr17	75950773	75955947
chr17	75955565	75955947
chr17	75957458	75973785
chr17	7595823	7601525
chr17	75986186	75991623
chr17	7601408	7606149
chr17	7602908	7606149
chr17	76043828	76046194
chr17	76057402	76061219
chr17	76067216	76070444
chr17	76101270	76101863
chr17	76287191	76290077
chr17	76287191	76304941
chr17	76312869	76313891
chr17	76312869	76330098
chr17	76738281	76742048
chr17	76738281	76744466
chr17	76740956	76769871
chr17	76740956	76776541
chr17	76767385	76776541
chr17	769868	776988
chr17	769868	778744
chr17	78064721	78067923
chr17	78064721	78075278
chr17	78073036	78087093
chr17	78077184	78086586
chr17	78079394	78087093
chr17	78086502	78087093
chr17	78086502	78093763
chr17	78091439	78093763
chr17	78092932	78093763
chr17	78117268	78117935
chr17	78170816	78171649
chr17	78392475	78398351
chr17	78392475	78399537
chr17	78392475	78404089
chr17	78398251	78404089
chr17	78414878	78419675
chr17	78414878	78419725
chr17	7850071	7851394
chr17	78680189	78701751
chr17	78680189	78709732
chr17	78698268	78709732

chr17	78698819	78709732
chr17	78802323	78803978
chr17	78803384	78807636
chr17	78812859	78820012
chr17	78812859	78821061
chr17	78813772	78814552
chr17	78818666	78821061
chr17	78827244	78838750
chr17	78835279	78838750
chr17	7890570	7891064
chr17	79077429	79077864
chr17	79077429	79081073
chr17	79077429	79084686
chr17	79079488	79081073
chr17	79085233	79085734
chr17	7939512	7940242
chr17	79942059	79952818
chr17	80048582	80050283
chr17	80048582	80058980
chr17	80049905	80050283
chr17	80058493	80058980
chr17	80136003	80138280
chr17	80212070	80213885
chr17	80263573	80273404
chr17	80294719	80317277
chr17	80307127	80309171
chr17	803681	805247
chr17	80707840	80754185
chr17	80791449	80857900
chr17	80883418	80893865
chr17	80922723	80923673
chr17	80922723	80925480
chr17	80922723	80940601
chr17	80940495	80949547
chr17	80945666	80949547
chr17	80994580	80995758
chr17	80994580	80998420
chr17	81053667	81057967
chr17	81053667	81086580
chr17	81053667	81108509
chr17	81084831	81086580
chr17	81099927	81108509
chr17	81206743	81207239
chr17	81260237	81277133
chr17	81260237	81284895

chr17	81270917	81276151
chr17	81270917	81277133
chr17	81270917	81284895
chr17	81275968	81277133
chr17	81275968	81283502
chr17	81275968	81284895
chr17	81280608	81284895
chr17	81569015	81589104
chr17	81588943	81597316
chr17	81588943	81600427
chr17	81588943	81608822
chr17	81596115	81600427
chr17	81596115	81604727
chr17	81596115	81608822
chr17	81600340	81608822
chr17	81604547	81608822
chr17	81604547	81629805
chr17	81606690	81608822
chr17	81610209	81613494
chr17	81610209	81622278
chr17	81610209	81629805
chr17	81613317	81629805
chr17	8161472	8162369
chr17	81667728	81670453
chr17	81667728	81672155
chr17	81693653	81694853
chr17	81693653	81695067
chr17	81700466	81700814
chr17	81855141	81855586
chr17	81908567	81909602
chr17	81913773	81915681
chr17	81979183	81985607
chr17	82036383	82036643
chr17	8206490	8207840
chr17	82084512	82084953
chr17	82092454	82093419
chr17	82095319	82096452
chr17	82127691	82128597
chr17	82163199	82172860
chr17	82171700	82172860
chr17	82171700	82178605
chr17	82171700	82179189
chr17	82171700	82183932
chr17	82178473	82179189
chr17	82183773	82188419

chr17	82183773	82193830
chr17	82183773	82198422
chr17	82248874	82249602
chr17	82248874	82265796
chr17	82249430	82265796
chr17	82252433	82253244
chr17	82252433	82265796
chr17	8234454	8237519
chr17	8238030	8238629
chr17	8238030	8243148
chr17	82403936	82406482
chr17	82403936	82409410
chr17	82403936	82411530
chr17	82444446	82446696
chr17	82459991	82472698
chr17	8253858	8254301
chr17	8253858	8258199
chr17	82563353	82568201
chr17	82563353	82571870
chr17	82563353	82587272
chr17	82568053	82571870
chr17	82571723	82584188
chr17	8257806	8258199
chr17	82582740	82587272
chr17	82625388	82631022
chr17	8262919	8263636
chr17	82756164	82768566
chr17	82756164	82772507
chr17	82756164	82781721
chr17	82756164	82797802
chr17	82756164	82809782
chr17	82756164	82814934
chr17	82756164	82870380
chr17	82756164	82893632
chr17	82763964	82768566
chr17	82763964	82772507
chr17	82763964	82781721
chr17	82763964	82800996
chr17	82763964	82814934
chr17	82763964	82870380
chr17	82763964	82893632
chr17	82763964	82900731
chr17	82766266	82768566
chr17	82797756	82800996
chr17	82805874	82809782

chr17	82805874	82814934
chr17	82814839	82889697
chr17	82814839	82911789
chr17	82870223	82893632
chr17	82870223	82900731
chr17	82870223	82903478
chr17	82870223	82907821
chr17	82870223	82911789
chr17	82884144	82893632
chr17	82884144	82900731
chr17	82884144	82903478
chr17	82884144	82906053
chr17	82884144	82909307
chr17	82884144	82911789
chr17	82889667	82900731
chr17	82889667	82903478
chr17	82889667	82909307
chr17	82889667	82911789
chr17	82893546	82900731
chr17	82893546	82903478
chr17	82893546	82911789
chr17	82900650	82906053
chr17	82900650	82909307
chr17	82900650	82911789
chr17	82903404	82909307
chr17	82903404	82911789
chr17	82905935	82911789
chr17	82905935	82930643
chr17	82923651	82932735
chr17	82956694	82961181
chr17	82956694	82965746
chr17	83005114	83048785
chr17	83014453	83048570
chr17	83014453	83048785
chr17	83035034	83048785
chr17	83048470	83048785
chr17	8379795	8382315
chr17	8444259	8450953
chr17	8444259	8454887
chr17	8444259	8460160
chr17	8444259	8463354
chr17	8484137	8492509
chr17	8499269	8504906
chr17	8499269	8509949
chr17	8506317	8509949

chr17	8542106	8554018
chr17	8552045	8577338
chr17	9162812	9182969
chr17	9378551	9557528
chr17	9491828	9557528
chr17	9505037	9557528
chr17	9680230	9693230
chr17	998778	999584
chr18	10539761	10540399
chr18	12348272	12353158
chr18	12351084	12351413
chr18	12351084	12353158
chr18	12351084	12356831
chr18	12351084	12371691
chr18	12353004	12356831
chr18	12353004	12371691
chr18	12356693	12360051
chr18	12356693	12371691
chr18	12358669	12360051
chr18	12358669	12371691
chr18	12363781	12371691
chr18	12366964	12367382
chr18	12366964	12371691
chr18	12370848	12371691
chr18	12420324	12421629
chr18	12452254	12635096
chr18	12454345	12464958
chr18	12463350	12464958
chr18	12463350	12479871
chr18	12493071	12535601
chr18	12493071	12546904
chr18	12506476	12512531
chr18	12506476	12535601
chr18	12506476	12546904
chr18	12506476	12635096
chr18	12535475	12635096
chr18	12695253	12697408
chr18	12698978	12701113
chr18	12718516	12720683
chr18	12794383	12859254
chr18	12801969	12814355
chr18	12814202	12831041
chr18	12814202	12836891
chr18	12814202	12840962
chr18	12825809	12831041

chr18	12825809	12859254
chr18	12951854	12963371
chr18	12951854	12984190
chr18	12999420	13001582
chr18	12999420	13008631
chr18	12999420	13013025
chr18	12999420	13019206
chr18	12999420	13030608
chr18	12999420	13040956
chr18	12999420	13042334
chr18	12999420	13073185
chr18	13001456	13019206
chr18	13001456	13030608
chr18	13008455	13019206
chr18	13008455	13030608
chr18	13012972	13019206
chr18	13012972	13030608
chr18	13029662	13042334
chr18	13037236	13040956
chr18	13048858	13059312
chr18	13087016	13092527
chr18	13087016	13096307
chr18	13089455	13103588
chr18	13099475	13103588
chr18	13104983	13116503
chr18	13104983	13117643
chr18	13671861	13682105
chr18	13681604	13687882
chr18	13731475	13754147
chr18	13734463	13737135
chr18	13740166	13752427
chr18	14183651	14189313
chr18	14183928	14191917
chr18	163307	180339
chr18	163307	192900
chr18	178932	192900
chr18	178932	199316
chr18	192841	199316
chr18	192841	204692
chr18	196636	199316
chr18	196636	203190
chr18	196636	204692
chr18	197615	204692
chr18	20953577	20955245
chr18	20986949	20992937

chr18	21006350	21008194
chr18	21039471	21042709
chr18	21039471	21044186
chr18	21039471	21049880
chr18	21366026	21384403
chr18	21366026	21395561
chr18	21366026	21403994
chr18	21383509	21384403
chr18	21383509	21403994
chr18	21384205	21403994
chr18	21439520	21440388
chr18	21439520	21441537
chr18	21439520	21477356
chr18	21536041	21568094
chr18	21539919	21560990
chr18	21539919	21566206
chr18	21539919	21568094
chr18	21560858	21566206
chr18	21560858	21568094
chr18	21564202	21566206
chr18	21564202	21568094
chr18	21622724	21623939
chr18	21656860	21659343
chr18	21656860	21664230
chr18	21765771	21768752
chr18	21765771	21773728
chr18	21765771	21779685
chr18	21765771	21791557
chr18	21765771	21798228
chr18	21765771	21799974
chr18	21765771	21804014
chr18	21765771	21819646
chr18	21765771	21849388
chr18	21768622	21773728
chr18	21773623	21779685
chr18	21773623	21804014
chr18	21778102	21779685
chr18	21778102	21804014
chr18	21791373	21799974
chr18	21791373	21804014
chr18	21791373	21819646
chr18	21791373	21844253
chr18	21815615	21838497
chr18	21815615	21847125
chr18	21815615	21858646

chr18	21838364	21844253
chr18	22171107	22183043
chr18	22936753	22949713
chr18	22968805	22982393
chr18	22968805	22984990
chr18	22975152	22984990
chr18	22984885	22991049
chr18	22990936	22996462
chr18	22992747	22993847
chr18	22992747	22996462
chr18	23001585	23006432
chr18	23006362	23022270
chr18	23213976	23237245
chr18	23234607	23237245
chr18	23309679	23373763
chr18	23352195	23373763
chr18	23466132	23467526
chr18	23467398	23479424
chr18	23506969	23509279
chr18	23515855	23519174
chr18	23520201	23524482
chr18	23561359	23572180
chr18	23568822	23573574
chr18	23777556	23784157
chr18	23833827	23842750
chr18	23898737	23899455
chr18	24064139	24069271
chr18	24064139	24083081
chr18	24080584	24083081
chr18	24118124	24125550
chr18	24123833	24125550
chr18	24123833	24130417
chr18	24155700	24160069
chr18	24166578	24171498
chr18	24166578	24172483
chr18	24178012	24179835
chr18	24196124	24239382
chr18	24225041	24239382
chr18	24280841	24314347
chr18	24280841	24341658
chr18	24280841	24377535
chr18	24303636	24312106
chr18	24303636	24341658
chr18	24332941	24341658
chr18	24366891	24368372

chr18	24366891	24377535
chr18	24427918	24428921
chr18	24427918	24440618
chr18	246323	252612
chr18	2544194	2555038
chr18	2577745	2579029
chr18	26032398	26057742
chr18	26038554	26078160
chr18	26052623	26078160
chr18	26052623	26087577
chr18	26265169	26293531
chr18	26274662	26293531
chr18	26285881	26357794
chr18	26292245	26327140
chr18	26315228	26335231
chr18	26315228	26357794
chr18	26321069	26335231
chr18	26476514	26501250
chr18	2666156	2673363
chr18	2666156	2674145
chr18	2666156	2688747
chr18	2673280	2674145
chr18	2688393	2688747
chr18	2694526	2698041
chr18	2703691	2707645
chr18	2706363	2729409
chr18	2718314	2726524
chr18	2718314	2728596
chr18	2732264	2740821
chr18	2732264	2762236
chr18	2738396	2739520
chr18	2762104	2771618
chr18	2769693	2770108
chr18	2771532	2777915
chr18	2775733	2784621
chr18	2777805	2784621
chr18	2784449	2796521
chr18	27963356	28013909
chr18	28002996	28013909
chr18	2920777	2940712
chr18	2951054	2960849
chr18	31530986	31535412
chr18	31626282	31645479
chr18	31630958	31658089
chr18	31630958	31666372

chr18	31638643	31658089
chr18	31638643	31666372
chr18	31645354	31666372
chr18	31657975	31666372
chr18	31839311	31855807
chr18	31846715	31849739
chr18	31846715	31852663
chr18	31864626	31867476
chr18	31864626	31871120
chr18	31870371	31871120
chr18	31873429	31901025
chr18	31873429	31917667
chr18	31890734	31909760
chr18	31890734	31913522
chr18	31897785	31909760
chr18	31897785	31917667
chr18	31897785	31931523
chr18	31907459	31931523
chr18	32042178	32045732
chr18	32111753	32113860
chr18	32111753	32124845
chr18	32111753	32126800
chr18	32270216	32288203
chr18	32270216	32310323
chr18	3249360	3254050
chr18	3253232	3254050
chr18	35478281	35480563
chr18	35478281	35489385
chr18	35683390	35692320
chr18	36026899	36031097
chr18	36026899	36033837
chr18	36030807	36031097
chr18	36112500	36127016
chr18	36133237	36138872
chr18	36141136	36160015
chr18	36142280	36159830
chr18	36142280	36160015
chr18	36144938	36146381
chr18	36160931	36171160
chr18	36195256	36203189
chr18	36195256	36205276
chr18	36195256	36220217
chr18	36198689	36200324
chr18	36198689	36205276
chr18	36213365	36257073

chr18	36220054	36251283
chr18	36220054	36260175
chr18	36256967	36260175
chr18	36501931	36512543
chr18	36576450	36658188
chr18	36658074	36681570
chr18	36658074	36693423
chr18	36681435	36693423
chr18	36681435	36709391
chr18	36755118	36769426
chr18	36834319	36959435
chr18	37160199	37173081
chr18	41993269	42033957
chr18	41996637	42020705
chr18	42015475	42029441
chr18	42027442	42040741
chr18	42027442	42043817
chr18	42027442	42049605
chr18	42037691	42049605
chr18	42038780	42049605
chr18	42043732	42049605
chr18	45865614	45867748
chr18	45866797	45870742
chr18	45880074	45882487
chr18	45880074	45912456
chr18	45889797	45910742
chr18	45899403	45904117
chr18	45899403	45910742
chr18	45899403	45912456
chr18	45910520	45912456
chr18	45913705	45917819
chr18	45922340	45923387
chr18	45922340	45946768
chr18	45928868	45934966
chr18	45928868	45944119
chr18	45928868	45952643
chr18	45939599	45952643
chr18	45943160	45951238
chr18	45948502	45952643
chr18	46084503	46095131
chr18	46088108	46095131
chr18	46118180	46120060
chr18	46122466	46123364
chr18	46122466	46124893
chr18	46815311	46821072

chr18	46815311	46828130
chr18	46815311	46836517
chr18	46820932	46844839
chr18	46827958	46846841
chr18	46836356	46864248
chr18	46855344	46864248
chr18	46864163	46891054
chr18	47112976	47136449
chr18	47130243	47134704
chr18	47130243	47136449
chr18	47845339	47896809
chr18	47865058	47870564
chr18	47865058	47896809
chr18	47868322	47870564
chr18	47868322	47896809
chr18	48619537	48670744
chr18	48636613	48670744
chr18	48663751	48670744
chr18	48663751	48711695
chr18	48757918	48761689
chr18	49097401	49257104
chr18	49118743	49163787
chr18	49163684	49379758
chr18	49257009	49258493
chr18	49257009	49282175
chr18	49257009	49286616
chr18	49257009	49379758
chr18	49272177	49282175
chr18	49272177	49286616
chr18	49272177	49379758
chr18	49281996	49286616
chr18	49281996	49379758
chr18	49331863	49333853
chr18	49331863	49379758
chr18	49331863	49391645
chr18	49363160	49379758
chr18	49363160	49391645
chr18	49363160	49430447
chr18	50375819	50385353
chr18	50895808	50908196
chr18	50895808	50921187
chr18	50895808	50932360
chr18	50895808	50940386
chr18	50918109	50921187
chr18	50918109	50924212

chr18	50918109	50925898
chr18	50918109	50932360
chr18	51046919	51049324
chr18	51046919	51067187
chr18	51054780	51059916
chr18	51054780	51067187
chr18	54159764	54166166
chr18	54159764	54205157
chr18	54188873	54205157
chr18	54271359	54274090
chr18	54271359	54277855
chr18	54271359	54280903
chr18	54271359	54284013
chr18	54271359	54287411
chr18	54271359	54292038
chr18	54277702	54287411
chr18	54358840	54365821
chr18	54362770	54372597
chr18	56614423	56618126
chr18	56616244	56626457
chr18	56717963	56731597
chr18	56717963	56781656
chr18	56717963	56816144
chr18	56731382	56758953
chr18	56731382	56781656
chr18	56756582	56758953
chr18	56756582	56781656
chr18	56758864	56781656
chr18	56758864	56816144
chr18	56779430	56816144
chr18	56781532	56962529
chr18	56816030	56880165
chr18	56879943	56962529
chr18	56923921	56939393
chr18	56935787	56962529
chr18	57554259	57554952
chr18	57605856	57609443
chr18	57609356	57615730
chr18	57611636	57615975
chr18	57613601	57615975
chr18	57653991	57662615
chr18	57653991	57695532
chr18	57671467	57691997
chr18	57685071	57695532
chr18	57688298	57695532

chr18	58165787	58252054
chr18	58227829	58252054
chr18	58315981	58325162
chr18	58315981	58333892
chr18	58322424	58351045
chr18	58328994	58333892
chr18	58341037	58351045
chr18	58341037	58357252
chr18	58342905	58357252
chr18	58349536	58357252
chr18	58364267	58367867
chr18	58503930	58529238
chr18	58514992	58529238
chr18	58681169	58700591
chr18	58696365	58700591
chr18	58709377	58715967
chr18	58709377	58723251
chr18	58709377	58735329
chr18	58714082	58734381
chr18	59338556	59338953
chr18	59338556	59347571
chr18	59347512	59349236
chr18	59438110	59439816
chr18	59466738	59469607
chr18	59466738	59480238
chr18	6080880	6093528
chr18	6093354	6171942
chr18	6093354	6215835
chr18	6171827	6312056
chr18	62072672	62085264
chr18	62072672	62095950
chr18	62082672	62090578
chr18	62082672	62095950
chr18	62082672	62101183
chr18	62106788	62154651
chr18	62114560	62148338
chr18	6213148	6239872
chr18	6213148	6312056
chr18	62138242	62148338
chr18	62138242	62163656
chr18	6215749	6312056
chr18	62221036	62232427
chr18	62244763	62261658
chr18	62244763	62268948
chr18	62252663	62298860

chr18	62280645	62298860
chr18	62348167	62349937
chr18	6237963	6244588
chr18	6237963	6264038
chr18	62539680	62550460
chr18	62539680	62556297
chr18	62539680	62565096
chr18	62539680	62575550
chr18	62539680	62576652
chr18	62542497	62565096
chr18	62556202	62565096
chr18	6263946	6312056
chr18	62830034	62838909
chr18	62830034	62920114
chr18	62838783	62860601
chr18	62895010	62903166
chr18	62895010	62920114
chr18	62958628	62963472
chr18	6301902	6312056
chr18	63338799	63362868
chr18	63344409	63362868
chr18	63350887	63362868
chr18	63355203	63362868
chr18	63399241	63400703
chr18	63399241	63411578
chr18	63979800	63981838
chr18	659640	662320
chr18	662145	669173
chr18	662145	671451
chr18	670691	671451
chr18	683245	706578
chr18	68697225	68701790
chr18	68697225	68711403
chr18	68836748	68874785
chr18	6961585	6965432
chr18	6961944	6965432
chr18	6973056	6980637
chr18	6974902	6980637
chr18	69873143	69896045
chr18	69946733	69947433
chr18	70024721	70030981
chr18	70047970	70060042
chr18	70047970	70065922
chr18	70047970	70075541
chr18	70051410	70065922

chr18	70051410	70128546
chr18	70065828	70092804
chr18	70086612	70092804
chr18	70086612	70128546
chr18	70086612	70135280
chr18	70092109	70109717
chr18	70121555	70128546
chr18	70127501	70128546
chr18	70145611	70150733
chr18	70166061	70176845
chr18	70166061	70190719
chr18	7032065	7046367
chr18	7034478	7037751
chr18	7043226	7044839
chr18	7043226	7046367
chr18	7046277	7050936
chr18	7079974	7080457
chr18	742917	743415
chr18	742917	756835
chr18	74641007	74890038
chr18	74781427	74890038
chr18	747919	756835
chr18	74877196	74890038
chr18	74877196	74920692
chr18	76370527	76371571
chr18	76370527	76443211
chr18	76371466	76380303
chr18	76378854	76463141
chr18	76849525	76851939
chr18	76849525	76868863
chr18	76849525	76871825
chr18	76849525	76875664
chr18	76849525	76880316
chr18	76849525	76881512
chr18	76868684	76881512
chr18	76927036	76960855
chr18	76937155	76960855
chr18	76947520	76960855
chr18	7774148	7888377
chr18	7774148	7955414
chr18	7888105	7906583
chr18	7888105	7926683
chr18	79096475	79126375
chr18	79096475	79154555
chr18	79096475	79176907

chr18	79096475	79207012
chr18	79096475	79214038
chr18	79096475	79253541
chr18	79096475	79277196
chr18	79110354	79126375
chr18	79110354	79154555
chr18	79113240	79154555
chr18	79126266	79154555
chr18	79126266	79176907
chr18	79126266	79193263
chr18	79126266	79207012
chr18	79126266	79214038
chr18	79126266	79253541
chr18	79126266	79277196
chr18	79126266	79307234
chr18	79143801	79176907
chr18	79143801	79253541
chr18	79154503	79193263
chr18	79154503	79214038
chr18	79193182	79214038
chr18	79206936	79277196
chr18	7926567	7955414
chr18	79303603	79307234
chr18	79410402	79433738
chr18	79433578	79467582
chr18	79448781	79451816
chr18	79695224	79697988
chr18	79695224	79704917
chr18	79710345	79729069
chr18	79710345	79736521
chr18	79728906	79736521
chr18	8069685	8076564
chr18	8076454	8088851
chr18	8076454	8114827
chr18	8076454	8143779
chr18	8076454	8247919
chr18	8085670	8088851
chr18	8113485	8114827
chr18	8244057	8253414
chr18	8624937	8633327
chr18	8635532	8636357
chr18	8718423	8720496
chr18	8777832	8798291
chr18	8777832	8807060
chr18	8783529	8786091

chr18	8796231	8798291
chr18	9117837	9124983
chr18	9117837	9126907
chr18	9182381	9208803
chr18	9182381	9211784
chr18	9182381	9221999
chr18	9195550	9204544
chr18	9195550	9211784
chr18	9204475	9211784
chr18	9208656	9221999
chr18	9211583	9221999
chr18	9524593	9525851
chr18	9524593	9530941
chr18	9570156	9577191
chr18	9570156	9595102
chr18	9577063	9584828
chr18	9577063	9595102
chr18	9577063	9595153
chr18	9583116	9584828
chr18	9583116	9593874
chr18	9583116	9595102
chr18	9583116	9595153
chr18	9584514	9588853
chr18	9584514	9595102
chr18	9588088	9588853
chr18	9588088	9595102
chr18	9588088	9595153
chr18	9593767	9595102
chr18	9593767	9595153
chr18	9775277	9815222
chr18	9931809	9937066
chr18	9931809	9950568
chr18	9936109	9945058
chr18	9936109	9950568
chr18	9944923	9950568
chr19	1003129	1005553
chr19	1011740	1012188
chr19	10143765	10149968
chr19	10151397	10155056
chr19	10151749	10160063
chr19	10154292	10155056
chr19	10154292	10155945
chr19	10154292	10166685
chr19	10154904	10160063
chr19	10159657	10160418

chr19	10162666	10166685
chr19	10163325	10173905
chr19	10163325	10175618
chr19	10163325	10177367
chr19	10168329	10177367
chr19	10168329	10182077
chr19	10173089	10177367
chr19	10173089	10182077
chr19	10173870	10177367
chr19	10180349	10182077
chr19	10258221	10258685
chr19	1031070	1032696
chr19	1031070	1036562
chr19	1032391	1032696
chr19	10328641	10330526
chr19	1036129	1036562
chr19	10368054	10368418
chr19	10446217	10449150
chr19	10446217	10450941
chr19	10448916	10463975
chr19	10461003	10461999
chr19	10489191	10489853
chr19	10546838	10547253
chr19	10551896	10552324
chr19	10557551	10558239
chr19	10574416	10575029
chr19	10677162	10681329
chr19	10683635	10683970
chr19	10759737	10777216
chr19	10759737	10786706
chr19	10772478	10777216
chr19	10772478	10786706
chr19	10805915	10830378
chr19	10812263	10830378
chr19	10904950	10909207
chr19	10913876	10916777
chr19	10923271	10923999
chr19	10984120	10987924
chr19	11003028	11003397
chr19	11007901	11010531
chr19	11030729	11041560
chr19	11033289	11041560
chr19	11059752	11060187
chr19	11106564	11116212
chr19	11110651	11111639

chr19	11111513	11116212
chr19	11113277	11113762
chr19	11116093	11116998
chr19	11116093	11129670
chr19	11120091	11120522
chr19	11120091	11123344
chr19	11120091	11128085
chr19	1116020	1127765
chr19	11178344	11178720
chr19	1119515	1120023
chr19	11214274	11217391
chr19	11214274	11252550
chr19	11216913	11217391
chr19	11245562	11245878
chr19	11335446	11335783
chr19	11397243	11397597
chr19	11405146	11417059
chr19	11415936	11417059
chr19	11448913	11449428
chr19	1147308	1154402
chr19	1149368	1154402
chr19	11495949	11498339
chr19	11507456	11519193
chr19	11513055	11514221
chr19	11724193	11725327
chr19	12017875	12018301
chr19	12046367	12046760
chr19	1218416	1219413
chr19	1218416	1223172
chr19	1254166	1255694
chr19	12623699	12624710
chr19	12649135	12650222
chr19	12658062	12658510
chr19	12663316	12663835
chr19	12672846	12673316
chr19	12679456	12680325
chr19	12702822	12703801
chr19	1270927	1272051
chr19	12710620	12711613
chr19	1271328	1272051
chr19	12714820	12715739
chr19	12745376	12745759
chr19	12764813	12766240
chr19	12769880	12771477
chr19	12810070	12810404

chr19	12851933	12852395
chr19	12851933	12858739
chr19	12878381	12878834
chr19	12928341	12928847
chr19	12928341	12930749
chr19	12939133	12940147
chr19	12939427	12940147
chr19	12947847	12948358
chr19	12947847	12949408
chr19	13073046	13075671
chr19	13073046	13081855
chr19	13078612	13081855
chr19	13135199	13136405
chr19	1357008	1361032
chr19	13817215	13819492
chr19	13909822	13912593
chr19	13912322	13912593
chr19	13912322	13913638
chr19	13913167	13913638
chr19	13923332	13923811
chr19	13927892	13928188
chr19	13955911	13956522
chr19	1398915	1399587
chr19	14039507	14039910
chr19	14042452	14042789
chr19	14046153	14046618
chr19	14097360	14100908
chr19	1417499	1422396
chr19	1417499	1428995
chr19	1418665	1422396
chr19	14390923	14398139
chr19	14401385	14404562
chr19	14402596	14404562
chr19	14458030	14458375
chr19	14479411	14480778
chr19	14479411	14492912
chr19	14563981	14564858
chr19	1510149	1511160
chr19	15268904	15273133
chr19	1584539	1585214
chr19	16081912	16086540
chr19	16081912	16089120
chr19	16081912	16093753
chr19	1625575	1632405
chr19	1627358	1632405

chr19	1632037	1632405
chr19	16361778	16395467
chr19	16425082	16428761
chr19	16503064	16509988
chr19	16679983	16680581
chr19	16679983	16682508
chr19	16841767	16854261
chr19	16862351	16866556
chr19	16863679	16866556
chr19	17058548	17062779
chr19	17152643	17154415
chr19	17153966	17157038
chr19	17153966	17168064
chr19	17156908	17159484
chr19	17156908	17163122
chr19	17159394	17168064
chr19	17172335	17172963
chr19	17200292	17200829
chr19	17209585	17211774
chr19	17276494	17276909
chr19	17559310	17560465
chr19	17559310	17572602
chr19	17720935	17725189
chr19	17973807	17989843
chr19	18134578	18134982
chr19	1816224	1816569
chr19	18162966	18163388
chr19	1816697	1817329
chr19	18175039	18175697
chr19	18537600	18538433
chr19	18537600	18538436
chr19	18539370	18539720
chr19	18573292	18573748
chr19	18742909	18749875
chr19	18759550	18760228
chr19	18845979	18850242
chr19	18845979	18852296
chr19	18855924	18864251
chr19	18863437	18864251
chr19	18878929	18893575
chr19	18910970	18913046
chr19	18924232	18924979
chr19	18931324	18933045
chr19	18931324	18939049
chr19	18934302	18939049

chr19	18938352	18939049
chr19	19001612	19008428
chr19	19001612	19031082
chr19	19008316	19010342
chr19	19024618	19031082
chr19	19104912	19106385
chr19	19120764	19121166
chr19	19132351	19133210
chr19	19196965	19199234
chr19	19198106	19199234
chr19	19296988	19306076
chr19	19335717	19349436
chr19	19337169	19343940
chr19	19340845	19341407
chr19	19492305	19492712
chr19	19492305	19494383
chr19	19535336	19535679
chr19	19564932	19569584
chr19	19564932	19570833
chr19	19640084	19640571
chr19	19645873	19646347
chr19	19645873	19647528
chr19	19878480	19880146
chr19	21033455	21034184
chr19	21309245	21310627
chr19	2180712	2194577
chr19	2185854	2193783
chr19	2185854	2194577
chr19	2189731	2193783
chr19	2189731	2194577
chr19	2213538	2214596
chr19	23373741	23374764
chr19	23662113	23663158
chr19	24105939	24106643
chr19	2438162	2438531
chr19	2762505	2769091
chr19	2936523	2939407
chr19	29971192	29986417
chr19	29971192	30005708
chr19	29971192	30012531
chr19	29985222	29986417
chr19	30007469	30012531
chr19	30009004	30012531
chr19	3008868	3015760
chr19	3011021	3015760

chr19	3110148	3115072
chr19	311844	313643
chr19	3192469	3196277
chr19	3192469	3198897
chr19	3192469	3201626
chr19	3193283	3201626
chr19	32356615	32360618
chr19	32408216	32411372
chr19	32432715	32437339
chr19	32432715	32439910
chr19	32436445	32455040
chr19	325634	327989
chr19	325634	336173
chr19	32605834	32607832
chr19	32615657	32622619
chr19	32615657	32625966
chr19	32615657	32626827
chr19	32615657	32643631
chr19	32617588	32622619
chr19	32617588	32625966
chr19	32617588	32643631
chr19	32622421	32625966
chr19	32622421	32626827
chr19	32625873	32626827
chr19	32625873	32628165
chr19	32625873	32628849
chr19	32625873	32643631
chr19	32631401	32640385
chr19	32631401	32642145
chr19	32631401	32643352
chr19	32631401	32644479
chr19	32639355	32642145
chr19	32639355	32643631
chr19	32639355	32649792
chr19	32643122	32643631
chr19	32643122	32646615
chr19	32644324	32659045
chr19	32881843	32887841
chr19	32899856	32901412
chr19	32899856	32915517
chr19	32899856	32918339
chr19	32901244	32915517
chr19	32901244	32931571
chr19	32915336	32918339
chr19	32915336	32926273

chr19	32915336	32933669
chr19	32915336	32948368
chr19	32931428	32933669
chr19	32931428	32939885
chr19	32931428	32960058
chr19	32948265	32953801
chr19	32973183	32973562
chr19	32990513	32991969
chr19	32996025	32999705
chr19	32996025	33003000
chr19	33002246	33003000
chr19	33008013	33012724
chr19	33008013	33021646
chr19	33011678	33012724
chr19	33011678	33026632
chr19	33088133	33101574
chr19	33093358	33094269
chr19	33109716	33111902
chr19	33112485	33119117
chr19	33113766	33114419
chr19	33125104	33126733
chr19	334399	336173
chr19	33462994	33501001
chr19	33511027	33512776
chr19	33667766	33689391
chr19	33798667	33801342
chr19	33798667	33807007
chr19	33798667	33811552
chr19	33798670	33801342
chr19	33798670	33807007
chr19	33798670	33811552
chr19	34194477	34196763
chr19	34194477	34209051
chr19	34194477	34215661
chr19	34196633	34215661
chr19	34208928	34219877
chr19	34215123	34215661
chr19	3425105	3435207
chr19	3425105	3449139
chr19	3425105	3452666
chr19	34327420	34330347
chr19	3433517	3449139
chr19	34377502	34377881
chr19	34396300	34396657
chr19	34409160	34421667

chr19	34430575	34433412
chr19	34430575	34434968
chr19	34430575	34438766
chr19	34430575	34445121
chr19	34430575	34464131
chr19	34430575	34467014
chr19	34431860	34434968
chr19	34431860	34438766
chr19	34431860	34443911
chr19	34431860	34464131
chr19	34431860	34467014
chr19	34433347	34438766
chr19	34433347	34445121
chr19	34433347	34464131
chr19	34433347	34467014
chr19	34434867	34443911
chr19	34438644	34445121
chr19	34443843	34445121
chr19	34443843	34467014
chr19	34444999	34464131
chr19	34450264	34460566
chr19	34450264	34464131
chr19	34450264	34467014
chr19	34451980	34464131
chr19	34454259	34464131
chr19	34458768	34467014
chr19	34464025	34467014
chr19	3449013	3452666
chr19	34490375	34495771
chr19	34493036	34494637
chr19	34678736	34683238
chr19	34929883	34933723
chr19	35009118	35013691
chr19	35258944	35262692
chr19	35498873	35502886
chr19	35499957	35510252
chr19	35615041	35615386
chr19	35631139	35634373
chr19	35729158	35730462
chr19	3573291	3578113
chr19	35754016	35754319
chr19	35762018	35762356
chr19	35764853	35765176
chr19	36065957	36068010
chr19	36065957	36071716

chr19	36067305	36090520
chr19	36089037	36089306
chr19	36092688	36094164
chr19	36094030	36099617
chr19	36120709	36125523
chr19	36142651	36149636
chr19	36145805	36146312
chr19	36194232	36202050
chr19	36472910	36473458
chr19	3651825	3656557
chr19	3651825	3667353
chr19	3656404	3662001
chr19	3660965	3662001
chr19	3660965	3667353
chr19	36712385	36712867
chr19	36762304	36762675
chr19	36823284	36825692
chr19	36917578	36937242
chr19	37080344	37091121
chr19	37089038	37091121
chr19	37155864	37156355
chr19	37165594	37170054
chr19	37347189	37347872
chr19	37425867	37426378
chr19	37425867	37444964
chr19	37644609	37647899
chr19	37707913	37711692
chr19	37770004	37773751
chr19	38081255	38130772
chr19	38099961	38119882
chr19	38106536	38119882
chr19	38110226	38119882
chr19	38119305	38130772
chr19	38130497	38142710
chr19	38141183	38142710
chr19	38141183	38152967
chr19	38152839	38164906
chr19	38162252	38164906
chr19	38192144	38193780
chr19	38216139	38217876
chr19	38307596	38309328
chr19	38404907	38407077
chr19	38700599	38701121
chr19	38905014	38907659
chr19	38917723	38918813

chr19	39354005	39357089
chr19	39417554	39418105
chr19	39417554	39419003
chr19	3981338	3982424
chr19	3981946	3983291
chr19	39840232	39840515
chr19	39840232	39840787
chr19	39879554	39899697
chr19	39883304	39896143
chr19	39889478	39902837
chr19	39979816	39980717
chr19	40047374	40050377
chr19	40081818	40084798
chr19	40237968	40240110
chr19	40255157	40257054
chr19	40255157	40265351
chr19	40333850	40336407
chr19	40458154	40458545
chr19	40582350	40583717
chr19	40583397	40583717
chr19	40616888	40619493
chr19	40617099	40622667
chr19	40623603	40624082
chr19	40703540	40714635
chr19	40705095	40705447
chr19	40710058	40714635
chr19	40725339	40733364
chr19	40736985	40737769
chr19	40757331	40759610
chr19	40783920	40786978
chr19	40786664	40786978
chr19	4110508	4117629
chr19	41220635	41253708
chr19	41230966	41231298
chr19	41243615	41248820
chr19	41243615	41256611
chr19	41248513	41248820
chr19	41252350	41256611
chr19	41268222	41276298
chr19	41268222	41279176
chr19	41268222	41281275
chr19	41276158	41281275
chr19	41332127	41348455
chr19	41341882	41342247
chr19	41378280	41382921

chr19	41410636	41419296
chr19	41422163	41423169
chr19	41869030	41869753
chr19	418922	425231
chr19	42117249	42122576
chr19	42232056	42236941
chr19	42232056	42240142
chr19	42236605	42240142
chr19	42286770	42294735
chr19	42335034	42335385
chr19	430683	440932
chr19	43497117	43497876
chr19	4354948	4355173
chr19	43652224	43655573
chr19	43652224	43656640
chr19	4365481	4366994
chr19	43655438	43656640
chr19	43656478	43665459
chr19	43732857	43733733
chr19	43737596	43740218
chr19	43744771	43748052
chr19	43747441	43750747
chr19	43831317	43835592
chr19	43918869	43925458
chr19	43922170	43934694
chr19	43924237	43925458
chr19	43925324	43934694
chr19	43988458	43991934
chr19	4405911	4409759
chr19	4405911	4418076
chr19	44076321	44082105
chr19	44144556	44150505
chr19	44165756	44172952
chr19	4422565	4433539
chr19	44228445	44229816
chr19	4430548	4432207
chr19	4452363	4454093
chr19	4475274	4475583
chr19	4475274	4491682
chr19	4475274	4501317
chr19	44756231	44758841
chr19	44865270	44874478
chr19	44900729	44901310
chr19	45022052	45025737
chr19	45022052	45028992

chr19	45025328	45025737
chr19	45025328	45028992
chr19	45025328	45034528
chr19	45138513	45146049
chr19	45138513	45146480
chr19	45258988	45263367
chr19	45258988	45271708
chr19	45258988	45278615
chr19	45258988	45287664
chr19	45263112	45266281
chr19	45277922	45278615
chr19	45277922	45280734
chr19	45280373	45280734
chr19	45287446	45294452
chr19	45355664	45357699
chr19	45357269	45361642
chr19	45363742	45370232
chr19	45364423	45364954
chr19	45368629	45369147
chr19	45398004	45398339
chr19	45413676	45416897
chr19	45413676	45423381
chr19	45416820	45421393
chr19	45419097	45421393
chr19	45517662	45518094
chr19	45521321	45522818
chr19	45522167	45522818
chr19	45613540	45614700
chr19	45626704	45630046
chr19	45821383	45823875
chr19	45831383	45835228
chr19	45842249	45844200
chr19	45852311	45854507
chr19	45854174	45854507
chr19	46375603	46376574
chr19	46605826	46608588
chr19	46652659	46653075
chr19	46693874	46714001
chr19	46724806	46725648
chr19	46727451	46733236
chr19	46733038	46738888
chr19	46748026	46748717
chr19	46776974	46784559
chr19	47040443	47042904
chr19	47081512	47082295

chr19	47081512	47094608
chr19	47143493	47155213
chr19	47143493	47169923
chr19	47150201	47155213
chr19	47150201	47169923
chr19	47152897	47155213
chr19	47264602	47264946
chr19	47352753	47355350
chr19	47352753	47362693
chr19	47357865	47362693
chr19	47406459	47409547
chr19	47416651	47417350
chr19	47489710	47495596
chr19	47500632	47503502
chr19	47670442	47675916
chr19	47718508	47726224
chr19	47725811	47736533
chr19	47746966	47752607
chr19	48127276	48128020
chr19	48149762	48150210
chr19	48149762	48157140
chr19	48218870	48221855
chr19	48221729	48241063
chr19	48230437	48241063
chr19	48231659	48234543
chr19	48389552	48390524
chr19	48446384	48446843
chr19	4859589	4860024
chr19	48627974	48628277
chr19	48853681	48854264
chr19	48913010	48913564
chr19	48970545	48971023
chr19	48981529	48982837
chr19	49004276	49007368
chr19	49004276	49011310
chr19	49007017	49007368
chr19	49090290	49098704
chr19	49127858	49130599
chr19	49342437	49359499
chr19	49342437	49360081
chr19	49355147	49359499
chr19	4941527	4941931
chr19	4944131	4944450
chr19	4944131	4947211
chr19	49449474	49450848

chr19	49458485	49459848
chr19	4947104	4950996
chr19	49478908	49480620
chr19	4950610	4950996
chr19	49614532	49616219
chr19	49621524	49625200
chr19	49645020	49657889
chr19	49679871	49680588
chr19	49781756	49782724
chr19	49854405	49860336
chr19	5016256	5033031
chr19	5016256	5041251
chr19	5016256	5047669
chr19	50217614	50224177
chr19	50247003	50250688
chr19	5032865	5041251
chr19	5039835	5041251
chr19	5039835	5047669
chr19	50398847	50399484
chr19	50398850	50399484
chr19	50408784	50409666
chr19	5047475	5082504
chr19	5047475	5138070
chr19	50686236	50702660
chr19	5077366	5082504
chr19	5131075	5144902
chr19	51860903	51877377
chr19	51860903	51880877
chr19	51860903	51886912
chr19	51877281	51880877
chr19	51877281	51886912
chr19	52001812	52002281
chr19	52040487	52047060
chr19	52076043	52077084
chr19	52076043	52089002
chr19	52160464	52169781
chr19	52215778	52216663
chr19	52220188	52225808
chr19	52282110	52283413
chr19	52314152	52315962
chr19	52347706	52354379
chr19	52353681	52354379
chr19	52373110	52374427
chr19	52532907	52547638
chr19	52574076	52576021

chr19	52618935	52635153
chr19	52655560	52660843
chr19	52728729	52730005
chr19	53074139	53075183
chr19	53115703	53116433
chr19	53148680	53153793
chr19	535836	536340
chr19	54142928	54144332
chr19	54142928	54146657
chr19	54142928	54149758
chr19	54142928	54153840
chr19	54143441	54144134
chr19	55047386	55048763
chr19	55098783	55099095
chr19	55098783	55103568
chr19	55098783	55112795
chr19	55140882	55141302
chr19	55140882	55141920
chr19	55196521	55197416
chr19	55245119	55245678
chr19	55265277	55266286
chr19	55294016	55294397
chr19	55341793	55343997
chr19	55341793	55346687
chr19	55386349	55388048
chr19	55659209	55663744
chr19	55792300	55801739
chr19	5592746	5600260
chr19	5604582	5604936
chr19	5604582	5616486
chr19	56146246	56147208
chr19	5616131	5616486
chr19	56407025	56414440
chr19	56413958	56414440
chr19	5641593	5641946
chr19	5641739	5651072
chr19	5648988	5654456
chr19	5650977	5657347
chr19	5653114	5653420
chr19	5653114	5654456
chr19	5653114	5657347
chr19	56547507	56550605
chr19	5700788	5705992
chr19	5711770	5713253
chr19	5711770	5714271

chr19	5727267	5737205
chr19	5727267	5739439
chr19	5733855	5739439
chr19	5751646	5768242
chr19	57546456	57552722
chr19	57925622	57933902
chr19	57961390	57966632
chr19	57988206	57988721
chr19	57988206	57999896
chr19	58061579	58063541
chr19	58088687	58090386
chr19	58392975	58393487
chr19	5921200	5925737
chr19	5927967	5941707
chr19	5941620	5957973
chr19	6004200	6008224
chr19	6010135	6016271
chr19	6026162	6040241
chr19	613247	614016
chr19	6217953	6227102
chr19	6230569	6270759
chr19	6262227	6270759
chr19	6362445	6366363
chr19	6364451	6366363
chr19	6387388	6389637
chr19	651610	652884
chr19	6742966	6743836
chr19	6749933	6750633
chr19	6934986	6937648
chr19	7122613	7132317
chr19	7172289	7174731
chr19	726137	727694
chr19	726137	734194
chr19	726137	736078
chr19	7440343	7447168
chr19	7444203	7447168
chr19	7462151	7464690
chr19	7462151	7470315
chr19	7627242	7628298
chr19	7938026	7941197
chr19	7973724	7991831
chr19	7981082	7991831
chr19	803560	805187
chr19	804291	804939
chr19	805012	806556

chr19	8371055	8371522
chr19	8455404	8463686
chr19	8455404	8467584
chr19	8455404	8471427
chr19	8462528	8467584
chr19	8462528	8471427
chr19	8462528	8474244
chr19	8589241	8589991
chr19	879936	886201
chr19	879936	890244
chr19	879936	891149
chr19	9337795	9339319
chr19	9355656	9364948
chr19	9364861	9371752
chr19	9417827	9419299
chr19	9530984	9533966
chr19	9532844	9535279
chr19	9535187	9536356
chr19	9614020	9618179
chr19	9656546	9659467
chr19	9656546	9660821
chr19	9656546	9660874
chr19	966571	968503
chr19	9828591	9828874
chr19	991080	991351
chr1	100007033	100015420
chr1	100007033	100017815
chr1	100011364	100015420
chr1	100049908	100061949
chr1	100049908	100068586
chr1	100049908	100069685
chr1	100058665	100068586
chr1	10007569	10008274
chr1	100102954	100110483
chr1	100140181	100148326
chr1	100377545	100390824
chr1	100424221	100442996
chr1	100424221	100462881
chr1	100424221	100468094
chr1	100439931	100462881
chr1	100439931	100468094
chr1	100455404	100484451
chr1	100494817	100499262
chr1	100906851	100913806
chr1	100906851	100918127

chr1	100906851	100921841
chr1	100911062	100921841
chr1	100912111	100921841
chr1	100913662	100921841
chr1	100913662	100974902
chr1	10095460	10105744
chr1	10095460	10122076
chr1	100995109	101021765
chr1	101001466	101025466
chr1	10102947	10145039
chr1	10119512	10130813
chr1	10132368	10137205
chr1	10134987	10137205
chr1	10146962	10161286
chr1	10149183	10151561
chr1	10232249	10256323
chr1	10232249	10261970
chr1	10256246	10261970
chr1	10256246	10268263
chr1	10258492	10268263
chr1	10261904	10291161
chr1	10267379	10268263
chr1	10271501	10275503
chr1	10271501	10276399
chr1	10275427	10278128
chr1	10275427	10282533
chr1	10276320	10291161
chr1	10291081	10297246
chr1	10320042	10343287
chr1	10342049	10345953
chr1	103527694	103551073
chr1	10363282	10365648
chr1	10371140	10375373
chr1	10403070	10404279
chr1	10403070	10413251
chr1	10404160	10408140
chr1	10408070	10417509
chr1	10411417	10413251
chr1	10433841	10442441
chr1	10440346	10442441
chr1	10467189	10469338
chr1	108143542	108161293
chr1	108148278	108161293
chr1	108154982	108185954
chr1	108181940	108185954

chr1	108616381	108628804
chr1	108624644	108629657
chr1	108650210	108657579
chr1	108650210	108659962
chr1	108654715	108657579
chr1	108654715	108659962
chr1	108657450	108659962
chr1	108782421	108794907
chr1	108782421	108796726
chr1	108793581	108796726
chr1	108793581	108800305
chr1	108796233	108798237
chr1	108885274	108918789
chr1	108896863	108904254
chr1	108901789	108918789
chr1	108939635	108944057
chr1	108939635	108950448
chr1	108943474	108943907
chr1	108943474	108944057
chr1	108943474	108950448
chr1	108974535	109004718
chr1	108982608	108986680
chr1	108986522	109011718
chr1	108991253	108995837
chr1	108995579	109004715
chr1	109223977	109231786
chr1	109230877	109231786
chr1	109262805	109265009
chr1	109281107	109282575
chr1	109336239	109342158
chr1	109336239	109345878
chr1	109336239	109369589
chr1	109340723	109345881
chr1	109340723	109347532
chr1	109342013	109345878
chr1	109342013	109345881
chr1	109345750	109355469
chr1	109347482	109355469
chr1	109350928	109355469
chr1	109354366	109355469
chr1	109409927	109415363
chr1	109411876	109415363
chr1	109573736	109582565
chr1	109573736	109586882
chr1	109573895	109579361

chr1	109579203	109582565
chr1	109579203	109586882
chr1	109582436	109586882
chr1	110009033	110014857
chr1	11016843	11018873
chr1	11018732	11020599
chr1	110222963	110226178
chr1	11030175	11030882
chr1	11068007	11076948
chr1	11070899	11076948
chr1	11073933	11076948
chr1	11077364	11081238
chr1	11121245	11122126
chr1	11124497	11133197
chr1	111456085	111459636
chr1	11157151	11167517
chr1	111627469	111697497
chr1	111627469	111704486
chr1	111627469	111709264
chr1	111691333	111704486
chr1	111691333	111709264
chr1	111759399	111761284
chr1	11199257	11199703
chr1	11228667	11234265
chr1	112412193	112416264
chr1	11247624	11248094
chr1	112555867	112616895
chr1	112576985	112582472
chr1	112576985	112584126
chr1	112576985	112591603
chr1	112576985	112598086
chr1	112576985	112600848
chr1	112576985	112616844
chr1	112576985	112616895
chr1	112581991	112584126
chr1	112581991	112598086
chr1	112581991	112600848
chr1	112581991	112616895
chr1	112591524	112598086
chr1	112591524	112611003
chr1	112591524	112616844
chr1	112591524	112616895
chr1	112597970	112616895
chr1	112600793	112611003
chr1	112610840	112616895

chr1	112647209	112659779
chr1	112653597	112654671
chr1	112653597	112659779
chr1	112653597	112667145
chr1	112654464	112659779
chr1	112654464	112669605
chr1	112691664	112694172
chr1	112692760	112694172
chr1	112917177	112929352
chr1	113091317	113094482
chr1	113091317	113096365
chr1	113091317	113100488
chr1	113093205	113094755
chr1	113549514	113585596
chr1	113549514	113590658
chr1	113549514	113622994
chr1	113585386	113590658
chr1	113671733	113673465
chr1	113704080	113706722
chr1	113704080	113724893
chr1	113710253	113712113
chr1	113710253	113724893
chr1	113724758	113726574
chr1	113724758	113738799
chr1	113726417	113738799
chr1	113738109	113738799
chr1	113777209	113777808
chr1	113829591	113834439
chr1	113897839	113902862
chr1	113901235	113901885
chr1	113906995	113908191
chr1	113952765	113954770
chr1	114397939	114401463
chr1	114399456	114407100
chr1	114463103	114463556
chr1	114463103	114464388
chr1	114587386	114595873
chr1	114608685	114622065
chr1	114708530	114716177
chr1	114709568	114713978
chr1	114719578	114720717
chr1	114719578	114724002
chr1	114719578	114738072
chr1	114723882	114732816
chr1	114725220	114738072

chr1	114733731	114738072
chr1	114734441	114750207
chr1	114739691	114741673
chr1	115659640	115664268
chr1	115992030	116037388
chr1	116019581	116020885
chr1	11671265	11672023
chr1	11676847	11680613
chr1	117084017	117091410
chr1	117090054	117096299
chr1	117402185	117405645
chr1	117402185	117414831
chr1	117402185	117420649
chr1	117402185	117442325
chr1	117402185	117466427
chr1	117414712	117420649
chr1	117414712	117442325
chr1	117414712	117466427
chr1	117442230	117466427
chr1	117442230	117502970
chr1	117460488	117466427
chr1	117460488	117493262
chr1	117460488	117502970
chr1	117493146	117502970
chr1	117878008	117899216
chr1	117881822	117886653
chr1	117881822	117920424
chr1	117896832	117912683
chr1	117896832	117920424
chr1	117906505	117918736
chr1	117911993	117918736
chr1	117933287	117942544
chr1	117951975	117953541
chr1	119146952	119150746
chr1	11947975	11972997
chr1	119721169	119727102
chr1	119734633	119737266
chr1	11989164	11992690
chr1	119948413	119955232
chr1	119950723	119987082
chr1	119953542	119963807
chr1	119963573	119969744
chr1	119963573	119997332
chr1	119996996	120029987
chr1	12001400	12002103

chr1	12005710	12009726
chr1	120163209	120168949
chr1	1223243	1223968
chr1	1223243	1228946
chr1	12234190	12253826
chr1	12253721	12257087
chr1	12253721	12258103
chr1	12268705	12271124
chr1	12293523	12308641
chr1	12299201	12308641
chr1	12299201	12311925
chr1	12299201	12333366
chr1	12299201	12335827
chr1	12329828	12341885
chr1	12353973	12363247
chr1	12400180	12403973
chr1	12400180	12415221
chr1	12400180	12416827
chr1	1256044	1257310
chr1	1256044	1267992
chr1	1256991	1257310
chr1	1256991	1267992
chr1	12578717	12579412
chr1	1319295	1321093
chr1	1340246	1342758
chr1	1390229	1395514
chr1	144439828	144456805
chr1	144456536	144459464
chr1	145643868	145663424
chr1	145747994	145784596
chr1	145771710	145788966
chr1	145825756	145827005
chr1	145833259	145833582
chr1	145839889	145841071
chr1	145851019	145853664
chr1	145853503	145854880
chr1	146943290	146946607
chr1	147252622	147256562
chr1	147252622	147259918
chr1	147252622	147264584
chr1	147252622	147268878
chr1	147252622	147276257
chr1	147254869	147280191
chr1	147255812	147268878
chr1	147259836	147267518

chr1	147275353	147285487
chr1	147286297	147287733
chr1	147648245	147659775
chr1	1482137	1486668
chr1	1485015	1487914
chr1	1485015	1490671
chr1	1489203	1490671
chr1	148961836	148962640
chr1	148977936	148978415
chr1	148977936	148979849
chr1	148977936	148981397
chr1	149001590	149005437
chr1	149655698	149661089
chr1	149655698	149669310
chr1	149947250	149950221
chr1	149959683	149964349
chr1	149959683	149965878
chr1	149964221	149977576
chr1	149965776	149971251
chr1	150068609	150110627
chr1	150081341	150110627
chr1	150230570	150231926
chr1	150324894	150325881
chr1	150334934	150338326
chr1	150334934	150340477
chr1	150343308	150344547
chr1	150417595	150446401
chr1	150440922	150446401
chr1	150444250	150446401
chr1	150492410	150496908
chr1	150492410	150497747
chr1	150492410	150499293
chr1	150497529	150499293
chr1	150625641	150627592
chr1	150661813	150663763
chr1	150817919	150826617
chr1	150823193	150826617
chr1	150831817	150836493
chr1	150834537	150836493
chr1	150927703	150930118
chr1	150927703	150941428
chr1	150929966	150941428
chr1	150941328	150945108
chr1	150942562	150947012
chr1	150949121	150949525

chr1	150951364	150962719
chr1	150960562	150962158
chr1	150966475	150969091
chr1	151017811	151027327
chr1	151024610	151025673
chr1	151024610	151027327
chr1	151024610	151028944
chr1	151025514	151027327
chr1	151027232	151028944
chr1	151088189	151098002
chr1	151139424	151140090
chr1	151166933	151167414
chr1	151178000	151184493
chr1	151185505	151185950
chr1	151224244	151232365
chr1	151224244	151234496
chr1	151224244	151239178
chr1	151224247	151232703
chr1	151227319	151232703
chr1	151231670	151232703
chr1	151232247	151232703
chr1	151239954	151242567
chr1	151242122	151242567
chr1	151262160	151264028
chr1	151412295	151442205
chr1	151423396	151442205
chr1	151427822	151430841
chr1	151427822	151442205
chr1	151430665	151442205
chr1	151440927	151442205
chr1	151562090	151578826
chr1	151562584	151566228
chr1	151638887	151658427
chr1	151638887	151662270
chr1	151638887	151666011
chr1	151638887	151668635
chr1	151638887	151683445
chr1	151638887	151693483
chr1	151658234	151662270
chr1	151658234	151666011
chr1	151658234	151668635
chr1	151658234	151683445
chr1	151660797	151666011
chr1	151762104	151763146
chr1	1522743	1527862

chr1	153626319	153632240
chr1	153663214	153664474
chr1	153663214	153665336
chr1	153664042	153668557
chr1	153665219	153668557
chr1	153667554	153668557
chr1	153682497	153685084
chr1	153751094	153757763
chr1	153752278	153759613
chr1	153759525	153761676
chr1	153759525	153765063
chr1	153760310	153765063
chr1	153761569	153765063
chr1	153762727	153765063
chr1	153811730	153813452
chr1	153812021	153819735
chr1	153816272	153819735
chr1	153817371	153818171
chr1	153818790	153819735
chr1	153932203	153933030
chr1	153932860	153933319
chr1	153937487	153937863
chr1	153937487	153944397
chr1	153941230	153942113
chr1	153951259	153952446
chr1	154170399	154173201
chr1	154170399	154176248
chr1	154172907	154173201
chr1	154172907	154176248
chr1	154212319	154214574
chr1	154225083	154228725
chr1	154225083	154236611
chr1	154225083	154237136
chr1	154225083	154241565
chr1	154227281	154237136
chr1	154234590	154235291
chr1	154234590	154237136
chr1	154234590	154246375
chr1	154235195	154246375
chr1	154243216	154246375
chr1	154246203	154251653
chr1	154249238	154251653
chr1	154249238	154257434
chr1	154251040	154251653
chr1	154251040	154257434

chr1	154253899	154255755
chr1	154257062	154257434
chr1	15425870	15427284
chr1	154258976	154266568
chr1	154260891	154261697
chr1	154260891	154266568
chr1	154266500	154268954
chr1	154273335	154273961
chr1	154346230	154346758
chr1	154434518	154449980
chr1	15493891	15495452
chr1	154965542	154966517
chr1	154966161	154967797
chr1	154966318	154967797
chr1	154967670	154969448
chr1	155239615	155240077
chr1	155264220	155270977
chr1	155264467	155270977
chr1	155265859	155266895
chr1	155265859	155270977
chr1	15529087	15534303
chr1	15529087	15536814
chr1	15529087	15546871
chr1	15529087	15548428
chr1	155310042	155312395
chr1	155341935	155346469
chr1	15534236	15536814
chr1	15534236	15548428
chr1	155343313	155346469
chr1	155346382	155347904
chr1	155347655	155349596
chr1	155349326	155354630
chr1	155352705	155354630
chr1	155352705	155357749
chr1	155352705	155360409
chr1	155357315	155357749
chr1	155360300	155395553
chr1	15536474	15548428
chr1	155370503	155370983
chr1	155370503	155459898
chr1	155378280	155380116
chr1	155378280	155395553
chr1	155378280	155415923
chr1	155395458	155439068
chr1	155395458	155459898

chr1	155415743	155439068
chr1	155415743	155459898
chr1	155438326	155459898
chr1	15544398	15548428
chr1	15562141	15564359
chr1	15563928	15564359
chr1	155661306	155672731
chr1	155668626	155675096
chr1	155668626	155679512
chr1	155668626	155680456
chr1	155670319	155679512
chr1	155670319	155680456
chr1	155675009	155679512
chr1	155676547	155679512
chr1	155676547	155680456
chr1	155709032	155726019
chr1	155709772	155717128
chr1	155709772	155721618
chr1	155717005	155721618
chr1	155717005	155726019
chr1	155721516	155726019
chr1	155725381	155726019
chr1	155725381	155727738
chr1	155729041	155729366
chr1	155754374	155757323
chr1	155756957	155760641
chr1	155773065	155776481
chr1	155776394	155784089
chr1	155777621	155784089
chr1	155783985	155814449
chr1	155785333	155815900
chr1	155795049	155814449
chr1	155795049	155827028
chr1	155804948	155814449
chr1	155804948	155816262
chr1	155804948	155822476
chr1	155813633	155814449
chr1	155813633	155827028
chr1	155820605	155827028
chr1	155822285	155853806
chr1	155904310	155904804
chr1	155915872	155917672
chr1	155915872	155921918
chr1	155921374	155921918
chr1	155921374	155923687

chr1	155921374	155925843
chr1	155923626	155929840
chr1	155925631	155927156
chr1	155926675	155927156
chr1	155926675	155929840
chr1	155954901	155958396
chr1	155966415	155969300
chr1	156014960	156018368
chr1	156225556	156236483
chr1	156265780	156266678
chr1	156265780	156274686
chr1	15626668	15638434
chr1	15626668	15643650
chr1	156268289	156279034
chr1	156272319	156274686
chr1	156272319	156278048
chr1	15630324	15638434
chr1	15630324	15643650
chr1	15630324	15651895
chr1	156317165	156317547
chr1	156317165	156325089
chr1	156320838	156334918
chr1	156324971	156334918
chr1	156333546	156334918
chr1	156333546	156335888
chr1	15638306	15643650
chr1	15638306	15649823
chr1	15638306	15651895
chr1	15643521	15649823
chr1	15643521	15651895
chr1	156475107	156477202
chr1	15649719	15651895
chr1	156530104	156531247
chr1	156743651	156745373
chr1	156782281	156787174
chr1	156782281	156797341
chr1	156951572	156955799
chr1	156963202	156977054
chr1	156963202	156979286
chr1	156971696	156986173
chr1	156976982	156980486
chr1	156976982	156986173
chr1	15717892	15721388
chr1	15719733	15721388
chr1	15764495	15765233

chr1	15767375	15768630
chr1	15767375	15770578
chr1	15767375	15774796
chr1	15770408	15774796
chr1	158084421	158088454
chr1	15872815	15876678
chr1	15909320	15916279
chr1	15911100	15916279
chr1	15911100	15922349
chr1	15937162	15938006
chr1	160231296	160240370
chr1	160237134	160244034
chr1	160279800	160283639
chr1	160294483	160297745
chr1	160305432	160305773
chr1	160305432	160314125
chr1	160311867	160313167
chr1	160313084	160325652
chr1	160323430	160332557
chr1	160325542	160335322
chr1	161102047	161102375
chr1	161123838	161124526
chr1	161162918	161164250
chr1	161213379	161213921
chr1	161323613	161328497
chr1	161323613	161340655
chr1	161323613	161356840
chr1	16137852	16138430
chr1	161751667	161752464
chr1	161751865	161753261
chr1	161752367	161753261
chr1	161778243	161784096
chr1	161778243	161792327
chr1	161778243	161802272
chr1	161778243	161863312
chr1	161791407	161792327
chr1	161791407	161802272
chr1	161791407	161821161
chr1	161791407	161863312
chr1	161792123	161821161
chr1	161802051	161821161
chr1	161819632	161863312
chr1	161846448	161853323
chr1	161846448	161863312
chr1	161851721	161912380

chr1	16201781	16202574
chr1	162333016	162357136
chr1	162355186	162357136
chr1	162499954	162501104
chr1	162499954	162503848
chr1	162503753	162512823
chr1	162512499	162518190
chr1	162566011	162576981
chr1	162566011	162581459
chr1	162566011	162588833
chr1	162566011	162590511
chr1	162566011	162597858
chr1	162576776	162579603
chr1	162576776	162581459
chr1	162576776	162590511
chr1	162579427	162588833
chr1	162581286	162597858
chr1	162587474	162588833
chr1	162588692	162590511
chr1	162655210	162719145
chr1	162799742	162804323
chr1	16305802	16315435
chr1	163326031	163328907
chr1	16391374	16393482
chr1	16443631	16452015
chr1	16447869	16452015
chr1	16451837	16455893
chr1	165665017	165683110
chr1	165668925	165680683
chr1	165679441	165683110
chr1	165679441	165695397
chr1	165682106	165683110
chr1	165682106	165695397
chr1	165890203	165891322
chr1	165890203	165896332
chr1	165890203	165903279
chr1	166862202	166863921
chr1	167765582	167773368
chr1	167765582	167776166
chr1	167772274	167773368
chr1	167772274	167776166
chr1	167919978	167924537
chr1	167951799	167975015
chr1	167966628	167975015
chr1	167966628	167987608

chr1	167966628	167993440
chr1	167966628	168004793
chr1	167987494	168004793
chr1	168002481	168004793
chr1	168002481	168015951
chr1	168002481	168045227
chr1	168015780	168023047
chr1	168022987	168045227
chr1	168022987	168068463
chr1	168038370	168045227
chr1	168038370	168068463
chr1	168043024	168045227
chr1	168063620	168068463
chr1	168065589	168068463
chr1	168096507	168114549
chr1	168104476	168114549
chr1	168183901	168184878
chr1	168183901	168191500
chr1	168231513	168231919
chr1	168231513	168239160
chr1	168291159	168305196
chr1	168300421	168305196
chr1	169230717	169237687
chr1	169230717	169287408
chr1	169230717	169298763
chr1	169230717	169303195
chr1	169230717	169310080
chr1	169287302	169298763
chr1	169287302	169303195
chr1	169287302	169310080
chr1	169298555	169310080
chr1	169298555	169324500
chr1	16930285	16931397
chr1	169303144	169324500
chr1	169309969	169324500
chr1	169323116	169324500
chr1	169376539	169380609
chr1	169798856	169804240
chr1	169798856	169806088
chr1	169798856	169821759
chr1	169798856	169823472
chr1	169798856	169827199
chr1	169798856	169832506
chr1	169806003	169827199
chr1	169830695	169843769

chr1	169836926	169850345
chr1	169859040	169873752
chr1	169866895	169878819
chr1	169875977	169878819
chr1	169954010	169984690
chr1	169972512	169992255
chr1	170016461	170039288
chr1	170016461	170055436
chr1	170024417	170055436
chr1	17022607	17024074
chr1	170539209	170544845
chr1	171512031	171513172
chr1	171512031	171527844
chr1	171522176	171523522
chr1	171523220	171524965
chr1	171523220	171527844
chr1	171535427	171537473
chr1	171568246	171571421
chr1	171568246	171575128
chr1	171568246	171587751
chr1	171579353	171584526
chr1	171584418	171588505
chr1	171783739	171786078
chr1	171783739	171790616
chr1	171783739	171794527
chr1	172042008	172048750
chr1	172387132	172388809
chr1	172551511	172557794
chr1	172551511	172570730
chr1	172551511	172579267
chr1	172555868	172557794
chr1	172555868	172570730
chr1	172555868	172575623
chr1	172555868	172579267
chr1	172600063	172602787
chr1	173481325	173486401
chr1	173751763	173753307
chr1	173751763	173757159
chr1	173751763	173766289
chr1	173755922	173757159
chr1	173774304	173775842
chr1	173807266	173811306
chr1	173826686	173834519
chr1	173830659	173833499
chr1	173830659	173839546

chr1	173830659	173840973
chr1	173838189	173845291
chr1	173839366	173840973
chr1	173941264	173943588
chr1	173941264	173943615
chr1	173946475	173947582
chr1	173961076	173970617
chr1	173961076	173984619
chr1	173964838	173972627
chr1	17412121	17413717
chr1	17416479	17420828
chr1	174219124	174221164
chr1	174219124	174231355
chr1	174219124	174241657
chr1	174220971	174252590
chr1	174220971	174305127
chr1	174231144	174305127
chr1	174241482	174305127
chr1	174250474	174305127
chr1	174250474	174371072
chr1	174250474	174394145
chr1	174252479	174275935
chr1	174272413	174305127
chr1	174272413	174371072
chr1	174272413	174394145
chr1	174275832	174371072
chr1	174275832	174394145
chr1	174304985	174394145
chr1	174370978	174394145
chr1	174637374	174702256
chr1	174637374	174811960
chr1	174699524	174702256
chr1	175004613	175008706
chr1	17580552	17588479
chr1	175986942	175989479
chr1	175986942	176046324
chr1	176027571	176046324
chr1	176027571	176116681
chr1	176027571	176136547
chr1	176027571	176176007
chr1	176043185	176046324
chr1	176043185	176085890
chr1	176043185	176136547
chr1	176081151	176116681
chr1	176081151	176136547

chr1	176081151	176149074
chr1	176116623	176136547
chr1	176116623	176149074
chr1	176116623	176176007
chr1	176116623	176184692
chr1	176135009	176149074
chr1	176135009	176176007
chr1	176149005	176163891
chr1	176149005	176176007
chr1	176149005	176184692
chr1	176162868	176176007
chr1	176162868	176184692
chr1	17627329	17640302
chr1	17648553	17664595
chr1	178283563	178300118
chr1	178283563	178390206
chr1	178299991	178390206
chr1	178464277	178467421
chr1	178776681	178785607
chr1	178776681	178811404
chr1	178776681	178889696
chr1	178808044	178833550
chr1	178877497	178886120
chr1	178877497	178894024
chr1	178877497	178906867
chr1	178885968	178906867
chr1	1789052	1790596
chr1	1790394	1817875
chr1	179043714	179050365
chr1	179043714	179054638
chr1	179043714	179064496
chr1	179085627	179088089
chr1	179110281	179121867
chr1	179112308	179117516
chr1	179112308	179118764
chr1	179117331	179118764
chr1	179117331	179133374
chr1	179118586	179120274
chr1	179118586	179121867
chr1	179120189	179121867
chr1	179121594	179133374
chr1	179131310	179133374
chr1	179302676	179323495
chr1	179302676	179343635
chr1	179323436	179337896

chr1	1793244	1806538
chr1	1793244	1817875
chr1	179335505	179337896
chr1	179335505	179341310
chr1	179335505	179343635
chr1	179337836	179341310
chr1	179337836	179342943
chr1	179337836	179343635
chr1	179337836	179350431
chr1	179341027	179342943
chr1	179341027	179348942
chr1	179342113	179343635
chr1	179347599	179348942
chr1	179347599	179350431
chr1	179378636	179385359
chr1	179378636	179395202
chr1	179393902	179395202
chr1	179491537	179492951
chr1	179900125	179908673
chr1	179901301	179908673
chr1	179986168	179992221
chr1	179986168	179997175
chr1	179986168	180006567
chr1	179990506	179992221
chr1	179990506	180015970
chr1	180003173	180006567
chr1	180022697	180024582
chr1	180062219	180065272
chr1	180062219	180075221
chr1	180075021	180084178
chr1	180075021	180090796
chr1	180175320	180178884
chr1	180183915	180194392
chr1	180314691	180413471
chr1	180314691	180495525
chr1	180397515	180413471
chr1	180397515	180430262
chr1	180397515	180495525
chr1	1804418	1806538
chr1	1804418	1817875
chr1	1804418	1825499
chr1	1804418	1839238
chr1	1806474	1817875
chr1	1806474	1825499
chr1	1806474	1839238

chr1	180787752	180811488
chr1	180803387	180811488
chr1	180824752	180825344
chr1	180824752	180863874
chr1	180834873	180863874
chr1	180984676	180988345
chr1	180984676	180993425
chr1	180984676	181005463
chr1	180988238	180993425
chr1	181002605	181005463
chr1	1815755	1825499
chr1	1815755	1839238
chr1	1817836	1839238
chr1	1825396	1839238
chr1	182842544	182843434
chr1	182842544	182860184
chr1	182852232	182858894
chr1	182874853	182881647
chr1	183114530	183115637
chr1	183114530	183118146
chr1	183131298	183132537
chr1	183512836	183517820
chr1	183528891	183533326
chr1	183533163	183544497
chr1	183742125	183866073
chr1	183880615	183897897
chr1	184702810	184749592
chr1	184706642	184711877
chr1	184726254	184749592
chr1	184732836	184749592
chr1	184803584	184831962
chr1	184823166	184831962
chr1	184884632	184894206
chr1	185087551	185093276
chr1	185147181	185151935
chr1	185204699	185206763
chr1	185214506	185231708
chr1	185299998	185305643
chr1	185300436	185305643
chr1	186318446	186318828
chr1	186318446	186327660
chr1	186343325	186344090
chr1	186379766	186389624
chr1	186379766	186394015
chr1	186390710	186391791

chr1	186390710	186394015
chr1	186398312	186406268
chr1	186977592	186979472
chr1	18916902	18919773
chr1	19126445	19129074
chr1	19170761	19175033
chr1	19172863	19185286
chr1	19176591	19187540
chr1	19183810	19185286
chr1	19186539	19187540
chr1	19192187	19192540
chr1	19193432	19197265
chr1	19193432	19201815
chr1	19197140	19201815
chr1	19232623	19235252
chr1	19242344	19245030
chr1	19255947	19257984
chr1	19284685	19286372
chr1	193028084	193029657
chr1	193069033	193077050
chr1	193069033	193082708
chr1	193135390	193138173
chr1	193135390	193152444
chr1	193138084	193142066
chr1	193138084	193152444
chr1	193141849	193152444
chr1	193141849	193212100
chr1	193203794	193212477
chr1	193203794	193236356
chr1	193232992	193236356
chr1	193232992	193249871
chr1	19344357	19357563
chr1	19356634	19357563
chr1	19378539	19419750
chr1	197117788	197139871
chr1	197124109	197125191
chr1	197129186	197139871
chr1	197172159	197178512
chr1	197172159	197191899
chr1	19739823	19787117
chr1	197553021	197658369
chr1	197553021	197772932
chr1	19755633	19787117
chr1	197583151	197611976
chr1	197583151	197617759

chr1	197583151	197645743
chr1	197583151	197658369
chr1	197583151	197715074
chr1	197583151	197772932
chr1	197595207	197617759
chr1	197595207	197715074
chr1	197607072	197658369
chr1	197617658	197658369
chr1	197617658	197715074
chr1	197617658	197772932
chr1	197642710	197647114
chr1	197642710	197652315
chr1	197642710	197658369
chr1	197642710	197674169
chr1	197642710	197715074
chr1	197642710	197772932
chr1	197652234	197674169
chr1	197652234	197715074
chr1	197672036	197674169
chr1	197672036	197715074
chr1	197672036	197735796
chr1	19770541	19798215
chr1	197715030	197735796
chr1	198232552	198264235
chr1	198232552	198279061
chr1	198232552	198297240
chr1	198253039	198264235
chr1	198253039	198279061
chr1	198277960	198297240
chr1	198292944	198297240
chr1	19904266	19904987
chr1	200554467	200559452
chr1	200565068	200575691
chr1	200580253	200592240
chr1	200581200	200592240
chr1	200586100	200592240
chr1	200593666	200615609
chr1	200600355	200602068
chr1	200603225	200608928
chr1	200608829	200615609
chr1	200614317	200615609
chr1	200659026	200666751
chr1	200760838	200815644
chr1	200807375	200815644
chr1	200807375	200832845

chr1	200815560	200832845
chr1	201485282	201496304
chr1	201780420	201781309
chr1	201847278	201848594
chr1	201852103	201857194
chr1	201852103	201858994
chr1	201854594	201857194
chr1	201854594	201858994
chr1	201857095	201858994
chr1	201988903	202004496
chr1	201996146	202000015
chr1	202001375	202001767
chr1	202001375	202003461
chr1	202135149	202135800
chr1	202333009	202335818
chr1	202416786	202428929
chr1	202422619	202449171
chr1	202427039	202449171
chr1	202428854	202434768
chr1	202431479	202449171
chr1	202440705	202449171
chr1	202755270	202764145
chr1	202773117	202777094
chr1	202893238	202895717
chr1	203067694	203071760
chr1	203707008	203708104
chr1	203816925	203818689
chr1	203816925	203840374
chr1	203828298	203831771
chr1	203828298	203840374
chr1	204112914	204114605
chr1	204112914	204117707
chr1	204116506	204117707
chr1	204525483	204532246
chr1	205103856	205105168
chr1	205150679	205157386
chr1	205157272	205187806
chr1	2055440	2059591
chr1	205618956	205623891
chr1	205720500	205729621
chr1	205723925	205729621
chr1	206415888	206430222
chr1	206430161	206440081
chr1	206446074	206455024
chr1	20652620	20656187

chr1	207061952	207065160
chr1	20713453	20715639
chr1	20747543	20776750
chr1	20749722	20773610
chr1	20757165	20767664
chr1	20757165	20771073
chr1	20757165	20773610
chr1	20757165	20779911
chr1	20757165	20780540
chr1	20765376	20773610
chr1	20765376	20776750
chr1	20767583	20773610
chr1	20770929	20773610
chr1	20770929	20780540
chr1	207757013	207759724
chr1	207757013	207770362
chr1	20776596	20779911
chr1	20840855	20865262
chr1	20849414	20851478
chr1	20851257	20886371
chr1	20857402	20862332
chr1	20857402	20886371
chr1	20879322	20892729
chr1	20893516	20895501
chr1	20893516	20942330
chr1	20899696	20904971
chr1	20904882	20942330
chr1	20941490	20950111
chr1	20969473	20980448
chr1	20969473	20981227
chr1	20980333	20981227
chr1	21050865	21089213
chr1	21050865	21111383
chr1	210587897	210623670
chr1	211260107	211289396
chr1	211353238	211354467
chr1	211353238	211361162
chr1	211778917	211793190
chr1	211787631	211793190
chr1	211944783	211952701
chr1	212006638	212011421
chr1	212006638	212021212
chr1	212016885	212021212
chr1	212020121	212021212
chr1	212042992	212047417

chr1	212042992	212080750
chr1	212068223	212080750
chr1	21221746	21228041
chr1	212329134	212342280
chr1	212329134	212347406
chr1	212342187	212347406
chr1	21235849	21238244
chr1	21244988	21260392
chr1	21244988	21279332
chr1	21260270	21272911
chr1	21272698	21290156
chr1	212863724	212872818
chr1	212863724	212885396
chr1	212863724	212888001
chr1	212863724	212888594
chr1	212872677	212889257
chr1	212997154	213008466
chr1	212997154	213013418
chr1	213071005	213117410
chr1	213071005	213176492
chr1	213072869	213104569
chr1	213077695	213117410
chr1	213077695	213129889
chr1	213104453	213129889
chr1	213117316	213129889
chr1	213129526	213176492
chr1	213167857	213176492
chr1	213240701	213242658
chr1	2135261	2150978
chr1	214314761	214318983
chr1	214369456	214372839
chr1	214383310	214398001
chr1	2144209	2150978
chr1	2144209	2156092
chr1	214451804	214464957
chr1	214629045	214638001
chr1	214644556	214648827
chr1	21470631	21473585
chr1	2148871	2156092
chr1	215573785	215579999
chr1	215573785	215586685
chr1	215575900	215579137
chr1	215586494	215595471
chr1	21680807	21695221
chr1	21680807	21706187

chr1	21680807	21724095
chr1	21701497	21703618
chr1	21701497	21706868
chr1	21715388	21721764
chr1	21721035	21751615
chr1	21723897	21736625
chr1	21747066	21751615
chr1	21747066	21753119
chr1	21747066	21757783
chr1	21748137	21751615
chr1	21748137	21753119
chr1	21751506	21757783
chr1	217610320	217614202
chr1	21842769	21849031
chr1	21847353	21849031
chr1	21861756	21862115
chr1	219972068	219978719
chr1	219996982	220007338
chr1	220001137	220007338
chr1	220005247	220007338
chr1	220005247	220019079
chr1	220006105	220007338
chr1	220102128	220102777
chr1	220102128	220103562
chr1	220136808	220139139
chr1	220141802	220145653
chr1	220167292	220195377
chr1	220182256	220182931
chr1	220191067	220210876
chr1	220212886	220213979
chr1	22074093	22086866
chr1	22078428	22086866
chr1	222569509	222570665
chr1	222577444	222579872
chr1	222648828	222654793
chr1	222654388	222659657
chr1	222658721	222660314
chr1	222686929	222703217
chr1	222715683	222724164
chr1	222724091	222725555
chr1	222725449	222730177
chr1	223744099	223745439
chr1	223746996	223749122
chr1	223759269	223762251
chr1	223759269	223772239

chr1	223799898	223821367
chr1	223804173	223806947
chr1	223804173	223810513
chr1	223806845	223821367
chr1	223810430	223814353
chr1	223810430	223818337
chr1	224130471	224134212
chr1	224231225	224268133
chr1	224233200	224268133
chr1	224236505	224268133
chr1	224250211	224268133
chr1	224250211	224281185
chr1	224268033	224289733
chr1	224281122	224300663
chr1	224281122	224303857
chr1	224286025	224289733
chr1	224294266	224303857
chr1	224296500	224303857
chr1	224296500	224308263
chr1	224304735	224305166
chr1	224365878	224371423
chr1	224393827	224399034
chr1	224398096	224399034
chr1	224398096	224401069
chr1	224400949	224404570
chr1	224411426	224419615
chr1	224418259	224424654
chr1	22489879	22491533
chr1	224952669	224964609
chr1	224952669	224968874
chr1	224952669	224974153
chr1	224952669	225007544
chr1	224952669	225043983
chr1	225002782	225007544
chr1	225002782	225023865
chr1	225002782	225097239
chr1	225007412	225023865
chr1	225007412	225097239
chr1	22501491	22502441
chr1	225038693	225043114
chr1	225038693	225043789
chr1	225038693	225043983
chr1	225038693	225085789
chr1	225038693	225097239
chr1	225038693	225152883

chr1	225079206	225097239
chr1	225080378	225097239
chr1	225082548	225152883
chr1	225097117	225100884
chr1	225117683	225147249
chr1	225117683	225152883
chr1	225152004	225153826
chr1	225152004	225159485
chr1	225167938	225185425
chr1	225167938	225206179
chr1	225167938	225231151
chr1	225167938	225266769
chr1	225185290	225192911
chr1	225185290	225206179
chr1	225185290	225207220
chr1	225185290	225240822
chr1	225204182	225231151
chr1	225231072	225290082
chr1	225252300	225259253
chr1	225264196	225290082
chr1	225270734	225273125
chr1	225412453	225415332
chr1	225412453	225419798
chr1	225412453	225424089
chr1	225417983	225424089
chr1	225512870	225517306
chr1	225554905	225567414
chr1	225871075	225879410
chr1	225921207	225921644
chr1	226152334	226159358
chr1	226159183	226166000
chr1	226233095	226239096
chr1	226265532	226301205
chr1	226266212	226277932
chr1	226277774	226287797
chr1	226286332	226287797
chr1	226295841	226301205
chr1	226388655	226392314
chr1	226871261	226875550
chr1	226885537	226889049
chr1	226961376	226965737
chr1	226994822	227005111
chr1	227004993	227017050
chr1	227016079	227035607
chr1	227026054	227035607

chr1	227026054	227048010
chr1	227035470	227069853
chr1	227069776	227074364
chr1	227112311	227112913
chr1	227119803	227147559
chr1	227119803	227160636
chr1	227139575	227147559
chr1	227139575	227213219
chr1	227142944	227147559
chr1	227160542	227213219
chr1	227193785	227199652
chr1	227193785	227213219
chr1	227193785	227254155
chr1	227747691	227759485
chr1	227747691	227767083
chr1	227758994	227767083
chr1	22781420	22785076
chr1	228213440	228215098
chr1	228213440	228264391
chr1	228291897	228304566
chr1	22863036	22882483
chr1	229444913	229465519
chr1	229458160	229460769
chr1	229458160	229463676
chr1	229460610	229465519
chr1	229463542	229465519
chr1	229463542	229475732
chr1	229466633	229487613
chr1	229470579	229502102
chr1	229486370	229487613
chr1	229486370	229498306
chr1	229489954	229496047
chr1	229489954	229498306
chr1	229495494	229496047
chr1	229525935	229531731
chr1	229525935	229540752
chr1	229525935	229542371
chr1	229527228	229542371
chr1	229530198	229531731
chr1	229530198	229542371
chr1	229539455	229542371
chr1	229539455	229549434
chr1	229542236	229547701
chr1	229632268	229647752
chr1	229632268	229654388

chr1	229643532	229654388
chr1	230178217	230203290
chr1	230236013	230243427
chr1	230236013	230249271
chr1	230236013	230250560
chr1	230243305	230246150
chr1	230243305	230250560
chr1	230243305	230255344
chr1	230243305	230265367
chr1	230246062	230250560
chr1	230249183	230255344
chr1	230262572	230265367
chr1	2302977	2304585
chr1	23030468	23044486
chr1	23030468	23050520
chr1	23030468	23053839
chr1	23030468	23059167
chr1	23044426	23057565
chr1	23044426	23059167
chr1	23050386	23057565
chr1	23050386	23059167
chr1	23053760	23059167
chr1	23055068	23057565
chr1	23055068	23069151
chr1	23055068	23079667
chr1	230659463	230664587
chr1	230659463	230679052
chr1	230663140	230664587
chr1	230663140	230671640
chr1	230663140	230675124
chr1	230663140	230679052
chr1	230664483	230683635
chr1	230669355	230671640
chr1	23071224	23072197
chr1	23071224	23073403
chr1	230933778	230961303
chr1	230943805	230954403
chr1	230943805	230958299
chr1	230943805	230961303
chr1	230945388	230958299
chr1	230945388	230961303
chr1	230954332	230961303
chr1	230995759	230997267
chr1	23109021	23109650
chr1	231270757	231276196

chr1	231370561	231374099
chr1	231529254	231542611
chr1	231537212	231542611
chr1	231616643	231618059
chr1	231795241	231818517
chr1	232415493	232432471
chr1	232490873	232493660
chr1	232955556	232956443
chr1	232998250	233001681
chr1	233014664	233025399
chr1	233014664	233057290
chr1	233025145	233057290
chr1	233025145	233208689
chr1	233054267	233057290
chr1	233054267	233095863
chr1	233054267	233179175
chr1	233090060	233095863
chr1	233090060	233161363
chr1	233090060	233208689
chr1	233135012	233139855
chr1	233135012	233161363
chr1	233135012	233179175
chr1	233135012	233208689
chr1	233160282	233179175
chr1	233160282	233208689
chr1	233160282	233227371
chr1	233177801	233200264
chr1	233177801	233208689
chr1	233177801	233227371
chr1	233177801	233252788
chr1	233198938	233208689
chr1	233198938	233218184
chr1	233198938	233227371
chr1	233198938	233236980
chr1	233198938	233252788
chr1	233198938	233259344
chr1	233200153	233259344
chr1	233208517	233218184
chr1	233208517	233236980
chr1	233208517	233252788
chr1	23321527	23323732
chr1	233217898	233227371
chr1	233217898	233236980
chr1	233217898	233252788
chr1	233227225	233236980

chr1	233227225	233252788
chr1	233227225	233259344
chr1	233227225	233263163
chr1	23323555	23333631
chr1	233236844	233263163
chr1	233250738	233263163
chr1	233252353	233259344
chr1	233258027	233261321
chr1	233346441	233355011
chr1	233346441	233362293
chr1	233346441	233376527
chr1	233353806	233355011
chr1	233353806	233362293
chr1	23337753	23338608
chr1	234418083	234427766
chr1	234418083	234448579
chr1	234418083	234472811
chr1	234425672	234427766
chr1	234446802	234467650
chr1	234448479	234459326
chr1	234450427	234472811
chr1	234457666	234472811
chr1	23451478	23456194
chr1	235113767	235122372
chr1	235135651	235138174
chr1	235135651	235152765
chr1	235137876	235155112
chr1	235148403	235155112
chr1	235152705	235155112
chr1	235172617	235177913
chr1	235229230	235246511
chr1	235229230	235257225
chr1	235234412	235255750
chr1	235240312	235260752
chr1	235246419	235260752
chr1	235252729	235260752
chr1	235255659	235260752
chr1	235419416	235434280
chr1	235419472	235430804
chr1	235419472	235434280
chr1	235427139	235434280
chr1	235427139	235437474
chr1	235434203	235437474
chr1	235437321	235441882
chr1	235438768	235441882

chr1	235438768	235442911
chr1	235453089	235494828
chr1	235454155	235455684
chr1	235455558	235484515
chr1	235458602	235470960
chr1	235465635	235470960
chr1	235465635	235484515
chr1	235465635	235494828
chr1	235470849	235484515
chr1	235470849	235494828
chr1	235470849	235496308
chr1	235480053	235489268
chr1	235480053	235494828
chr1	235480053	235496308
chr1	235484321	235494828
chr1	235484321	235496308
chr1	235494680	235496308
chr1	235677090	235697272
chr1	235686948	235693486
chr1	235686948	235697272
chr1	235693349	235716778
chr1	235755477	235762851
chr1	235762719	235775086
chr1	235766078	235770297
chr1	235773841	235777308
chr1	235780864	235782087
chr1	235791698	235793612
chr1	235793502	235801097
chr1	235800319	235801097
chr1	235830225	235833667
chr1	235985378	235991058
chr1	236011920	236017273
chr1	236017147	236038253
chr1	236024069	236038253
chr1	236029549	236048989
chr1	236032400	236038253
chr1	236038103	236048989
chr1	236041909	236048989
chr1	236168705	236178636
chr1	236168705	236180028
chr1	236168705	236183906
chr1	236168705	236205250
chr1	236178413	236180028
chr1	236178413	236183906
chr1	236220831	236222010

chr1	236249884	236253505
chr1	236581221	236588043
chr1	236582735	236588043
chr1	236594011	236596976
chr1	236803427	236806233
chr1	236803427	236816543
chr1	236803427	236838599
chr1	236803427	236853088
chr1	236806143	236816543
chr1	236824118	236826896
chr1	236824118	236838599
chr1	236824118	236853088
chr1	236824118	236863554
chr1	236826828	236835687
chr1	236835546	236838599
chr1	236850343	236853088
chr1	236859832	236863554
chr1	2372480	2389996
chr1	23750844	23752518
chr1	23772110	23772426
chr1	23785674	23786423
chr1	23808134	23820593
chr1	23814189	23820593
chr1	23816674	23820593
chr1	2395783	2402342
chr1	23971572	23972012
chr1	23971849	23978817
chr1	240123178	240178068
chr1	240123178	240188262
chr1	240123178	240258032
chr1	240123178	240294883
chr1	240177920	240188262
chr1	240177920	240211235
chr1	240177920	240294883
chr1	240188206	240211235
chr1	240188206	240294883
chr1	240206798	240211235
chr1	240206798	240258032
chr1	240206798	240294883
chr1	240206798	240334229
chr1	240206798	240392562
chr1	240211090	240334229
chr1	240257944	240294883
chr1	240257944	240334229
chr1	240257944	240355908

chr1	240257944	240392562
chr1	240294821	240334229
chr1	240294821	240355908
chr1	240294821	240392562
chr1	240329075	240330749
chr1	240329075	240334229
chr1	240329075	240355908
chr1	240333886	240392562
chr1	240355815	240438210
chr1	240392510	240438210
chr1	241506002	241508785
chr1	241511966	241517316
chr1	24169416	24180854
chr1	241850408	241861502
chr1	241866829	241882017
chr1	241866829	241885507
chr1	243136142	243156455
chr1	243164283	243225321
chr1	243169627	243186422
chr1	243169627	243225321
chr1	243185778	243186422
chr1	243185778	243191494
chr1	243185778	243211964
chr1	243186258	243200680
chr1	243191017	243225321
chr1	243199059	243211964
chr1	243199059	243221813
chr1	243199059	243225321
chr1	243221723	243225321
chr1	243270104	243286397
chr1	243270104	243293219
chr1	243270104	243304777
chr1	243270104	243308177
chr1	243270104	243316893
chr1	243270104	243330692
chr1	243270104	243344331
chr1	243274542	243304777
chr1	243274542	243378863
chr1	243286271	243344331
chr1	243286271	243418076
chr1	243293090	243308177
chr1	243293090	243330692
chr1	243293090	243344331
chr1	243304712	243316893
chr1	243304712	243330692

chr1	243304712	243341173
chr1	243304712	243344331
chr1	243307988	243344331
chr1	243316754	243378863
chr1	243330539	243344331
chr1	243341038	243378863
chr1	243378720	243418076
chr1	243415701	243418076
chr1	243415701	243426558
chr1	243512323	243573048
chr1	243512323	243615161
chr1	243512323	243843282
chr1	243545509	243563848
chr1	243545509	243573048
chr1	243545509	243637742
chr1	243545509	243843282
chr1	243552728	243573048
chr1	243563719	243695716
chr1	243563719	243843282
chr1	243572925	243695716
chr1	243613670	243637742
chr1	243613670	243664883
chr1	243613670	243695716
chr1	243613670	243843282
chr1	243637610	243664883
chr1	243637610	243695716
chr1	243645892	243843282
chr1	243664771	243695716
chr1	243664771	243843282
chr1	243695590	243843282
chr1	244411286	244417752
chr1	244411286	244424387
chr1	244415980	244424387
chr1	244418759	244420296
chr1	244422834	244424387
chr1	244422834	244432595
chr1	244686596	244692020
chr1	244858728	244859374
chr1	245002120	245017326
chr1	245002120	245059458
chr1	245002120	245083688
chr1	245017241	245059458
chr1	245017241	245083688
chr1	245059385	245083688
chr1	245764040	245929937

chr1	245858495	245863886
chr1	245858495	245915640
chr1	245858495	245929937
chr1	245915529	245929937
chr1	245927930	245929937
chr1	246327200	246335474
chr1	246327200	246355094
chr1	24646666	24649029
chr1	24649970	24651612
chr1	246544520	246556721
chr1	246544520	246557534
chr1	246548544	246557534
chr1	246551212	246557534
chr1	246556572	246557534
chr1	246621428	246634587
chr1	246631893	246648037
chr1	24666814	24670315
chr1	246726891	246740301
chr1	246726891	246744125
chr1	246726891	246764357
chr1	246735974	246740301
chr1	246735974	246744125
chr1	246740171	246758356
chr1	246744075	246758356
chr1	246758225	246766139
chr1	246860898	246862153
chr1	246863923	246867811
chr1	246876036	246877302
chr1	246876036	246888517
chr1	246913231	246918377
chr1	246988120	247000074
chr1	246999347	247000074
chr1	247159005	247159813
chr1	247308474	247310456
chr1	247328682	247330081
chr1	247423229	247425599
chr1	247423229	247436140
chr1	247435969	247444821
chr1	247443971	247444821
chr1	247860616	247864935
chr1	247860616	247868063
chr1	247864704	247868063
chr1	24797741	24827116
chr1	24814093	24827116
chr1	2484447	2489867

chr1	248844482	248845662
chr1	24919239	24929852
chr1	2511337	2514089
chr1	25340473	25343073
chr1	25340473	25351693
chr1	25340473	25352974
chr1	25351625	25352974
chr1	25351625	25356853
chr1	25446761	25454382
chr1	25448807	25454382
chr1	25553921	25557267
chr1	25554859	25557267
chr1	25901490	25903813
chr1	26239761	26244318
chr1	26239761	26255865
chr1	26239761	26259802
chr1	26255170	26259802
chr1	26257596	26259802
chr1	26268482	26269614
chr1	26268482	26272071
chr1	26294204	26298062
chr1	26432890	26438284
chr1	26432890	26460144
chr1	26438167	26447660
chr1	26446315	26447660
chr1	26446315	26460144
chr1	26447558	26460144
chr1	26457790	26460144
chr1	26729650	26732792
chr1	26760855	26763285
chr1	26884159	26886133
chr1	26924088	26941818
chr1	26941456	26941818
chr1	26941456	26945686
chr1	26942659	26943065
chr1	27105884	27107865
chr1	27105884	27109777
chr1	27107647	27114286
chr1	27260955	27287861
chr1	27260955	27298111
chr1	27330719	27334281
chr1	27414832	27428933
chr1	27558304	27558883
chr1	27668225	27669346
chr1	27755194	27759627

chr1	27789561	27793632
chr1	27789561	27812268
chr1	27832755	27843330
chr1	27838698	27843330
chr1	27841013	27843330
chr1	27897004	27914169
chr1	27906927	27907282
chr1	27978373	28004419
chr1	27988534	28004419
chr1	27997319	28000049
chr1	28004335	28017239
chr1	28004335	28035680
chr1	28010946	28017239
chr1	28027788	28042650
chr1	28027788	28058094
chr1	28035543	28042650
chr1	28035543	28048426
chr1	28035543	28058094
chr1	28038838	28058094
chr1	28203746	28205349
chr1	28269182	28279241
chr1	28271673	28279241
chr1	28274824	28279241
chr1	28459084	28460292
chr1	28459084	28466768
chr1	28465684	28480604
chr1	28473553	28476291
chr1	28473553	28480604
chr1	28476106	28480604
chr1	28490950	28493091
chr1	28491649	28493091
chr1	28508127	28516867
chr1	28529857	28531990
chr1	28529857	28532350
chr1	28560632	28564834
chr1	28567293	28571900
chr1	28571175	28571900
chr1	28579532	28581229
chr1	28580559	28581229
chr1	28605371	28622165
chr1	28617952	28622165
chr1	28683582	28690186
chr1	28683582	28697084
chr1	28683585	28691709
chr1	28690103	28691709

chr1	28690103	28693045
chr1	28690103	28704329
chr1	28702437	28704329
chr1	28704191	28710642
chr1	28993329	28997319
chr1	28993329	29097935
chr1	29015691	29033245
chr1	29015691	29039426
chr1	29018223	29039426
chr1	29035825	29039426
chr1	29035825	29053312
chr1	29058588	29097935
chr1	29060421	29065158
chr1	29060421	29097935
chr1	29109335	29115803
chr1	29150102	29154910
chr1	29195940	29202045
chr1	29216004	29216685
chr1	29216004	29216812
chr1	29258504	29260903
chr1	30936642	30967310
chr1	30945345	30950261
chr1	30950132	30953981
chr1	30953713	30967310
chr1	30964673	30974802
chr1	30964673	30981405
chr1	30965981	30974802
chr1	30974650	30981405
chr1	30980061	30995220
chr1	30980061	31007102
chr1	30981311	31007102
chr1	30992389	30995220
chr1	30992389	31007102
chr1	31005852	31007102
chr1	31005852	31028864
chr1	31028795	31059577
chr1	31267870	31292006
chr1	31269157	31292006
chr1	31271378	31292006
chr1	31281373	31292006
chr1	31310043	31339048
chr1	31319108	31348974
chr1	31337174	31339048
chr1	31337174	31348974
chr1	31346639	31348974

chr1	31423692	31425913
chr1	31423692	31426823
chr1	31737428	31740541
chr1	31910037	31911826
chr1	31910037	31919658
chr1	31915894	31919658
chr1	32030297	32031640
chr1	32030297	32033334
chr1	32030297	32037043
chr1	32030297	32038619
chr1	32030297	32045390
chr1	32036909	32038036
chr1	32036909	32038119
chr1	32036909	32038619
chr1	32036909	32039573
chr1	32037834	32038619
chr1	32091674	32100562
chr1	32094783	32100562
chr1	32154587	32158361
chr1	32154587	32159531
chr1	32160614	32167296
chr1	32163234	32167296
chr1	32184524	32188124
chr1	32187953	32190249
chr1	32192310	32193300
chr1	32224033	32224475
chr1	32224033	32226530
chr1	32226170	32226530
chr1	32324478	32327677
chr1	32326938	32327677
chr1	32383829	32386895
chr1	32592930	32595223
chr1	32627507	32634110
chr1	32627507	32634792
chr1	32631826	32634072
chr1	32631826	32634110
chr1	32631826	32634792
chr1	32633644	32634110
chr1	32633644	32634792
chr1	32651913	32672526
chr1	32657426	32672901
chr1	32668224	32672901
chr1	32779381	32781145
chr1	32779381	32787075
chr1	32782403	32787075

chr1	32797762	32811057
chr1	32810590	32811057
chr1	32825312	32830067
chr1	32825312	32853166
chr1	32942251	32944159
chr1	32942251	32945628
chr1	32942251	32949774
chr1	32948221	32949774
chr1	33013206	33014594
chr1	33276099	33281811
chr1	34992233	35015162
chr1	35010446	35012581
chr1	35093913	35097566
chr1	35093913	35110447
chr1	35097316	35110447
chr1	35104294	35104769
chr1	35104294	35110447
chr1	35358924	35361789
chr1	35358924	35370627
chr1	35370028	35370627
chr1	35370028	35399576
chr1	35381258	35381758
chr1	35381258	35389082
chr1	35381258	35390098
chr1	35385441	35390098
chr1	35388909	35390098
chr1	35388909	35392352
chr1	35398863	35399576
chr1	35405022	35405468
chr1	35405022	35408159
chr1	35413971	35415714
chr1	35460304	35466695
chr1	35460304	35470960
chr1	35466607	35479212
chr1	35470862	35479212
chr1	35474804	35479212
chr1	35743140	35746992
chr1	35745450	35746992
chr1	35747906	35748604
chr1	35747906	35753936
chr1	35760341	35763321
chr1	35816881	35822982
chr1	35816881	35826835
chr1	35816881	35841750
chr1	35825312	35835993

chr1	35831426	35835993
chr1	35901473	35902337
chr1	35901947	35902337
chr1	35902203	35918423
chr1	35913841	35915542
chr1	35945691	35967075
chr1	35945691	35973511
chr1	35966954	35973511
chr1	35972023	35973511
chr1	36004340	36014048
chr1	36004340	36027298
chr1	36004340	36043548
chr1	36008689	36014048
chr1	36013629	36014048
chr1	36034173	36040441
chr1	36036176	36040441
chr1	36039789	36040441
chr1	36170970	36173478
chr1	36172463	36176842
chr1	36289059	36301084
chr1	36307769	36309590
chr1	36348699	36361340
chr1	36358238	36358822
chr1	36358238	36361340
chr1	3682332	3683180
chr1	3682332	3707791
chr1	37574342	37576556
chr1	37582222	37587495
chr1	37695909	37703347
chr1	37700435	37703347
chr1	3781511	3784111
chr1	3781511	3785203
chr1	37835078	37835744
chr1	37835078	37838756
chr1	37864311	37868614
chr1	3833861	3852421
chr1	3865811	3872572
chr1	38851614	38856980
chr1	38998170	39001106
chr1	39231181	39258028
chr1	39295786	39300362
chr1	39349477	39388658
chr1	39350784	39359264
chr1	39350784	39372596
chr1	39360792	39361677

chr1	39360792	39368314
chr1	39385433	39388658
chr1	39422373	39422900
chr1	39429241	39432654
chr1	39434413	39439500
chr1	39447431	39448152
chr1	39447431	39448763
chr1	39451051	39460794
chr1	39451051	39463686
chr1	39451051	39468732
chr1	39459085	39462037
chr1	39740164	39753052
chr1	39763091	39764817
chr1	39847219	39857417
chr1	39853969	39857417
chr1	39957086	39958825
chr1	40059336	40061812
chr1	40064226	40064559
chr1	40078559	40091399
chr1	40091328	40092507
chr1	40189054	40190853
chr1	40189054	40195764
chr1	40189054	40202614
chr1	40189054	40222710
chr1	40190771	40202614
chr1	40260838	40268535
chr1	40267785	40272035
chr1	40406735	40417096
chr1	40413015	40417096
chr1	40456944	40457439
chr1	40540585	40541710
chr1	40641708	40647842
chr1	40738835	40753246
chr1	40738835	40758294
chr1	40747533	40753246
chr1	40749572	40758294
chr1	40758120	40769415
chr1	40983277	40984991
chr1	40983277	40991845
chr1	40983277	40997526
chr1	40983277	41002254
chr1	40995916	41003176
chr1	41001028	41003176
chr1	41001028	41006094
chr1	41008793	41010254

chr1	41046406	41075451
chr1	41070594	41075451
chr1	41070594	41161432
chr1	41113282	41117010
chr1	41113282	41143112
chr1	41113282	41151684
chr1	41113282	41161432
chr1	41113282	41186250
chr1	41116921	41143112
chr1	41142877	41151684
chr1	41575543	41628947
chr1	42181916	42191719
chr1	42188736	42205863
chr1	42188736	42278672
chr1	42194889	42323827
chr1	42227882	42278672
chr1	42227882	42311110
chr1	42227882	42323827
chr1	42265114	42278672
chr1	42265114	42311110
chr1	42265114	42323827
chr1	42278347	42311110
chr1	42278347	42323827
chr1	42311049	42323827
chr1	42665979	42667440
chr1	42754868	42755217
chr1	42927604	42931206
chr1	43359166	43359638
chr1	43425473	43426151
chr1	43451427	43452319
chr1	43538197	43545166
chr1	43538197	43553941
chr1	43538197	43569778
chr1	43538197	43578920
chr1	43538197	43609498
chr1	43545030	43553941
chr1	43553491	43553941
chr1	43553491	43569778
chr1	43553491	43578920
chr1	43569589	43578920
chr1	43578809	43589000
chr1	43588730	43592601
chr1	43603415	43609498
chr1	43613617	43617568
chr1	43618629	43619202

chr1	43792101	43838311
chr1	43814890	43858308
chr1	43824730	43838311
chr1	43838218	43920928
chr1	43894382	43899727
chr1	43920403	43920928
chr1	43958241	43964321
chr1	44247938	44319744
chr1	44247938	44339322
chr1	44247938	44352925
chr1	44284834	44319744
chr1	44284834	44339322
chr1	44284834	44352925
chr1	44308309	44319744
chr1	44308309	44339322
chr1	44319627	44339322
chr1	44319627	44352925
chr1	44753131	44753833
chr1	44753732	44756237
chr1	44753732	44762658
chr1	44757555	44758140
chr1	44757555	44759348
chr1	44757555	44760702
chr1	44776033	44776774
chr1	44776033	44778129
chr1	44874677	44880008
chr1	45330515	45334511
chr1	45552435	45561878
chr1	45566568	45567020
chr1	45613168	45617591
chr1	45614095	45616393
chr1	45615012	45616393
chr1	45630481	45640403
chr1	45633488	45642499
chr1	45633488	45655319
chr1	45634095	45659142
chr1	45640209	45642499
chr1	45640209	45661225
chr1	45642426	45655319
chr1	45642426	45659142
chr1	45654542	45655319
chr1	45654542	45661225
chr1	45716894	45719340
chr1	45824432	45882395
chr1	45824432	45959477

chr1	46055794	46077613
chr1	46055794	46080750
chr1	46061928	46077613
chr1	46077514	46080750
chr1	46258685	46261015
chr1	46258685	46261385
chr1	46267458	46270785
chr1	46279181	46281385
chr1	46309100	46310316
chr1	46309896	46310316
chr1	46562648	46572167
chr1	46580529	46594280
chr1	46583227	46594280
chr1	46652580	46658737
chr1	46683299	46691937
chr1	47260288	47263113
chr1	47269634	47272241
chr1	47269634	47300152
chr1	47280240	47282459
chr1	47280240	47301748
chr1	47280240	47304996
chr1	47287550	47293544
chr1	47287550	47295848
chr1	47287550	47300152
chr1	47287550	47301748
chr1	47287550	47302346
chr1	47293457	47304996
chr1	47295764	47301748
chr1	47295764	47304996
chr1	47299904	47301748
chr1	47301560	47302346
chr1	47301560	47304996
chr1	47301560	47310362
chr1	47302233	47304996
chr1	47368468	47373107
chr1	47368468	47375293
chr1	47374908	47375293
chr1	47408319	47409901
chr1	48305786	48359770
chr1	48305786	48413151
chr1	48355669	48359770
chr1	48355669	48399644
chr1	48385308	48399644
chr1	48385308	48403882
chr1	48385308	48413151

chr1	48395266	48399644
chr1	48395266	48403882
chr1	48395266	48413151
chr1	48411463	48453131
chr1	48451551	48453131
chr1	50490587	50535457
chr1	50490587	50596216
chr1	50535368	50567231
chr1	50535368	50584811
chr1	50535368	50596216
chr1	50535368	50655528
chr1	50539591	50596216
chr1	50567076	50584811
chr1	50567076	50596216
chr1	50582617	50596216
chr1	50583651	50744775
chr1	50596120	50744775
chr1	50655441	50705891
chr1	50655441	50744775
chr1	50705785	50738954
chr1	50705785	50788205
chr1	50705785	50857997
chr1	50738862	50788205
chr1	50738862	50801677
chr1	50787999	50801677
chr1	50787999	50857997
chr1	51361170	51366029
chr1	51363865	51394447
chr1	51365952	51400953
chr1	51394380	51400953
chr1	51394380	51403532
chr1	51399031	51402525
chr1	51399031	51448135
chr1	51402434	51408332
chr1	51402434	51465326
chr1	51405904	51408332
chr1	51440346	51448135
chr1	51461090	51465326
chr1	51461090	51481314
chr1	51463672	51465326
chr1	51714002	51750195
chr1	51745535	51756358
chr1	51745535	51761971
chr1	51745535	51765981
chr1	51746709	51765981

chr1	51748368	51765981
chr1	51750144	51765981
chr1	51756319	51765981
chr1	51760689	51782643
chr1	51772604	51782643
chr1	51798248	51814089
chr1	51800555	51814089
chr1	51810280	51816389
chr1	51814034	51821555
chr1	51814034	51834170
chr1	51814550	51819873
chr1	51818065	51825382
chr1	51818065	51840514
chr1	51819799	51823786
chr1	51819799	51834170
chr1	51821497	51834170
chr1	51823663	51827869
chr1	51823663	51834170
chr1	51823663	51837620
chr1	51827795	51834170
chr1	51933317	51977117
chr1	51937293	51977117
chr1	52023490	52028630
chr1	52024509	52028630
chr1	52216368	52266831
chr1	52233170	52239595
chr1	52281660	52303925
chr1	52293452	52295977
chr1	52332767	52337934
chr1	52374809	52383569
chr1	52383419	52385949
chr1	52396045	52397863
chr1	52431012	52465173
chr1	52445786	52458449
chr1	52445786	52461769
chr1	52458335	52465173
chr1	52465069	52472102
chr1	52477707	52481923
chr1	52488908	52526373
chr1	52490731	52509712
chr1	52493610	52497183
chr1	52493610	52509712
chr1	52495426	52509712
chr1	52515890	52526373
chr1	52802091	52816629

chr1	52854464	52857749
chr1	52854464	52860871
chr1	52854464	52867777
chr1	52856997	52864157
chr1	52856997	52867777
chr1	52860730	52864157
chr1	52860730	52867777
chr1	52877681	52881665
chr1	52974768	52980543
chr1	52978216	52988136
chr1	53014889	53039046
chr1	53233541	53235635
chr1	53255116	53258471
chr1	53266472	53271346
chr1	53594107	53600278
chr1	53772328	53789196
chr1	53772328	53793279
chr1	53787157	53797144
chr1	53787157	53800848
chr1	53787157	53806517
chr1	53789132	53797144
chr1	53800692	53804009
chr1	53800692	53832591
chr1	53800692	53835620
chr1	53803927	53806517
chr1	53803927	53809746
chr1	53803927	53832591
chr1	53818970	53832591
chr1	53825797	53832591
chr1	53832489	53835620
chr1	53923903	53940097
chr1	54036609	54047372
chr1	54040755	54043525
chr1	54040755	54047372
chr1	54195418	54196491
chr1	54728345	54731700
chr1	54865302	54876047
chr1	54871349	54876047
chr1	54875092	54876047
chr1	55100838	55107430
chr1	55123446	55134413
chr1	58506059	58534331
chr1	58506059	58536741
chr1	58527260	58536741
chr1	58530600	58536741

chr1	5863889	5880239
chr1	5865101	5866458
chr1	58667037	58685251
chr1	58667846	58675580
chr1	58671869	58677056
chr1	58673572	58677056
chr1	58673572	58685251
chr1	58681784	58685251
chr1	58690339	58695207
chr1	5887285	5909213
chr1	5887285	5986327
chr1	5890867	5933329
chr1	5904616	5909213
chr1	5904616	5933329
chr1	5927648	5933329
chr1	5927648	5952836
chr1	5927648	5961949
chr1	5927648	5986327
chr1	59321535	59340069
chr1	59321535	59346398
chr1	59321535	59378837
chr1	59321535	59457076
chr1	59339957	59346398
chr1	59339957	59378837
chr1	59346246	59378837
chr1	5947103	5952836
chr1	5947103	5961949
chr1	5947103	5986327
chr1	5952699	5986327
chr1	59554123	59607910
chr1	5961793	5969259
chr1	5961793	5986327
chr1	5967298	5986327
chr1	5978269	5986327
chr1	61277519	61359274
chr1	61332511	61359274
chr1	61352449	61359274
chr1	61383236	61406727
chr1	61683416	61694770
chr1	61762857	61763179
chr1	61762857	61775334
chr1	61791347	61808530
chr1	61827421	61884408
chr1	61827421	61927829
chr1	61833653	61884408

chr1	61856029	61901459
chr1	61899582	61901459
chr1	6192929	6197756
chr1	61990167	62038049
chr1	61990167	62051058
chr1	61990167	62084648
chr1	62017855	62084648
chr1	62114052	62123058
chr1	62123020	62128945
chr1	6231901	6233643
chr1	6231901	6234974
chr1	62439798	62443319
chr1	62441487	62442299
chr1	62444737	62448666
chr1	62473981	62504782
chr1	62488933	62492847
chr1	62504629	62513898
chr1	62537890	62539892
chr1	62537890	62552901
chr1	62555824	62578966
chr1	62577261	62578966
chr1	62577261	62586624
chr1	62577261	62625401
chr1	62586506	62663130
chr1	62618705	62625401
chr1	62618705	62663130
chr1	62647690	62663130
chr1	62653724	62663130
chr1	62803718	62816808
chr1	62803718	62821209
chr1	62803718	62834852
chr1	62803718	62841547
chr1	6281101	6294980
chr1	62816574	62821209
chr1	62829039	62841547
chr1	6318491	6333568
chr1	6318491	6349866
chr1	6327298	6349866
chr1	6333476	6349866
chr1	63411925	63428801
chr1	63453917	63454968
chr1	63453917	63490218
chr1	63478763	63490218
chr1	63478763	63508570
chr1	63525571	63534216

chr1	63532669	63534216
chr1	63545915	63555515
chr1	63629424	63654466
chr1	63651668	63654466
chr1	64009304	64050716
chr1	64049690	64050716
chr1	64550593	64602912
chr1	64602805	64641969
chr1	64629354	64634260
chr1	64629354	64641969
chr1	64663694	64664679
chr1	64666056	64675983
chr1	64673347	64675561
chr1	6469041	6469676
chr1	64777622	64781571
chr1	64777622	64789514
chr1	64777622	64814840
chr1	64812737	64814840
chr1	64873369	64886341
chr1	6528993	6532760
chr1	6531967	6533441
chr1	6531967	6541927
chr1	6533279	6549698
chr1	65364634	65366196
chr1	65364634	65384326
chr1	65364634	65395032
chr1	6541829	6549698
chr1	6549570	6550615
chr1	6595484	6599452
chr1	6616756	6620337
chr1	6667708	6681037
chr1	66891153	66905375
chr1	66940027	66958220
chr1	66940027	66959743
chr1	66940027	66963160
chr1	66946149	66958220
chr1	66970807	66976722
chr1	67020368	67047367
chr1	67420008	67425223
chr1	67424199	67426285
chr1	68137779	68148660
chr1	68137779	68155260
chr1	6820180	6825210
chr1	70148486	70159438
chr1	70173464	70189273

chr1	70184784	70189273
chr1	70187264	70189273
chr1	70221614	70228555
chr1	70221614	70237552
chr1	70228421	70239520
chr1	70239438	70244815
chr1	70270855	70276844
chr1	70270855	70306290
chr1	70274719	70292549
chr1	70274719	70306290
chr1	70276764	70315566
chr1	70292387	70300908
chr1	70292387	70306290
chr1	70292387	70315566
chr1	70292387	70324957
chr1	70296129	70306290
chr1	70296129	70315566
chr1	70300763	70313790
chr1	70300763	70315566
chr1	70306223	70324957
chr1	74710097	74719372
chr1	75728400	75734871
chr1	75732643	75733628
chr1	75732643	75734871
chr1	75732643	75750546
chr1	75739979	75750546
chr1	75745805	75750546
chr1	75749418	75750546
chr1	75787496	75789356
chr1	75787496	75791571
chr1	75790151	75790517
chr1	75803730	75816546
chr1	77122274	77169395
chr1	77122274	77210489
chr1	77154448	77169395
chr1	77154448	77210489
chr1	77576067	77579628
chr1	77580997	77582126
chr1	77631849	77641655
chr1	77639448	77641655
chr1	77641383	77641655
chr1	77701368	77713281
chr1	77711746	77714783
chr1	77711746	77715868
chr1	77725621	77739417

chr1	77728294	77736158
chr1	77734346	77736158
chr1	77734346	77739417
chr1	77734346	77741748
chr1	77736055	77741748
chr1	77739264	77741748
chr1	77740876	77741748
chr1	7777159	7778169
chr1	77783237	77807101
chr1	77783237	77843407
chr1	77801330	77807101
chr1	77801330	77815231
chr1	77801330	77843407
chr1	77801330	77859056
chr1	77801330	77861322
chr1	77801330	77864028
chr1	77801330	77866391
chr1	77801330	77873120
chr1	77803269	77807101
chr1	77803269	77813867
chr1	77803269	77815231
chr1	77813733	77866391
chr1	77843306	77864028
chr1	77843306	77866391
chr1	77858937	77861322
chr1	77858937	77864028
chr1	77858937	77866391
chr1	77949154	77962930
chr1	77955254	77956700
chr1	77956571	77963715
chr1	77964645	77968203
chr1	7962762	7970963
chr1	83890320	83906463
chr1	83904078	83933766
chr1	83917603	83921394
chr1	84179176	84185182
chr1	84179176	84214317
chr1	84196615	84197824
chr1	8422726	8541318
chr1	84563256	84570720
chr1	84655831	84671308
chr1	8465924	8541318
chr1	8465924	8557523
chr1	8465924	8614686
chr1	84669680	84671308

chr1	84937754	84952530
chr1	84937754	84965708
chr1	84947032	84965708
chr1	8495062	8497529
chr1	8495062	8508675
chr1	8495062	8541318
chr1	8495062	8557523
chr1	8495062	8614686
chr1	8495062	8624380
chr1	84952242	84965708
chr1	84958528	84965708
chr1	8508626	8557523
chr1	8508626	8614686
chr1	8508626	8624380
chr1	85164525	85178272
chr1	85169043	85178272
chr1	85182097	85190752
chr1	85350414	85358847
chr1	8541213	8557523
chr1	8541213	8614686
chr1	8541213	8624380
chr1	8556474	8624380
chr1	8614560	8624380
chr1	86352858	86376418
chr1	86371017	86385590
chr1	86372430	86383196
chr1	86372430	86384801
chr1	8655972	8792588
chr1	86715723	86719635
chr1	86715723	86722673
chr1	86715723	86735179
chr1	86719506	86724405
chr1	86722539	86735179
chr1	87072933	87084279
chr1	87072933	87092669
chr1	87097837	87103589
chr1	87339535	87340202
chr1	8862886	8864092
chr1	8862886	8867250
chr1	8866278	8867250
chr1	88740987	88760376
chr1	88740987	88771879
chr1	88740987	88805671
chr1	88760221	88771879
chr1	88760221	88786213

chr1	88770351	88786213
chr1	88771420	88786213
chr1	88771420	88805671
chr1	88784638	88786213
chr1	88786103	88806017
chr1	88786103	88822003
chr1	88804390	88813733
chr1	88807312	88822003
chr1	88857205	88860286
chr1	89010903	89015414
chr1	89058840	89060324
chr1	90005132	90007750
chr1	91734775	91758750
chr1	92088697	92102669
chr1	92088697	92129776
chr1	92247061	92264638
chr1	92262862	92271652
chr1	92286489	92291537
chr1	92297403	92298029
chr1	92307187	92345914
chr1	92320598	92324375
chr1	92323444	92336427
chr1	92323444	92345914
chr1	92333390	92345914
chr1	92333390	92380873
chr1	9244924	9247083
chr1	9244924	9262328
chr1	92563641	92605402
chr1	92563641	92607727
chr1	92563641	92636336
chr1	92563641	92704744
chr1	925921	931089
chr1	92605306	92607727
chr1	92605306	92624334
chr1	92605306	92625934
chr1	92605306	92636336
chr1	92605306	92704744
chr1	92624175	92636336
chr1	92663419	92704744
chr1	92665938	92693899
chr1	92665938	92695453
chr1	92665938	92704744
chr1	92677157	92704744
chr1	92693801	92697985
chr1	92693801	92704744

chr1	92693801	92736627
chr1	92697859	92704744
chr1	92702140	92704744
chr1	92702140	92736627
chr1	92833388	92834913
chr1	92833388	92837633
chr1	92834778	92837633
chr1	930154	931089
chr1	93114687	93115618
chr1	93120548	93134195
chr1	93127231	93129448
chr1	93156299	93160226
chr1	93183359	93192106
chr1	93183359	93193744
chr1	93183359	93201988
chr1	93186344	93193744
chr1	93210801	93214966
chr1	93210801	93216746
chr1	93210801	93217869
chr1	93212100	93216746
chr1	93212100	93217869
chr1	93212100	93226449
chr1	93221608	93221936
chr1	93226332	93232593
chr1	93256334	93258885
chr1	93256334	93264901
chr1	93324634	93325895
chr1	93324634	93339536
chr1	93324634	93340510
chr1	93499467	93524323
chr1	93499467	93530883
chr1	93567278	93567851
chr1	93567278	93576129
chr1	93571669	93576129
chr1	93571669	93584121
chr1	935771	939460
chr1	93582300	93584121
chr1	93582300	93592393
chr1	93642303	93674941
chr1	93894613	93897898
chr1	93894613	93901669
chr1	93894613	93904588
chr1	93896617	93904588
chr1	94177611	94184288
chr1	94177611	94190083

chr1	94201719	94220392
chr1	94201719	94231643
chr1	94203929	94205683
chr1	94220257	94231643
chr1	94458606	94468007
chr1	94458606	94480606
chr1	94458606	94491247
chr1	94464774	94475737
chr1	94464774	94480606
chr1	94480463	94491247
chr1	94483169	94491247
chr1	94487541	94491247
chr1	94498601	94499614
chr1	94498782	94506642
chr1	95143890	95151419
chr1	95143890	95173889
chr1	9553625	9573412
chr1	9553625	9580234
chr1	955922	957025
chr1	955922	957273
chr1	9595889	9598625
chr1	96751424	96777946
chr1	96751424	96785254
chr1	96769702	96770851
chr1	96769702	96777946
chr1	9743883	9744643
chr1	97450058	97515941
chr1	97515725	97549744
chr1	97515725	97595166
chr1	97515725	97699547
chr1	97549559	97573970
chr1	9755113	9773394
chr1	97573759	97595166
chr1	97679094	97699547
chr1	97679094	97740479
chr1	97691716	97740479
chr1	98684884	98698905
chr1	98684884	98738389
chr1	98691074	98701903
chr1	98691074	98738389
chr1	9931890	9932898
chr1	9931890	9934860
chr1	9931890	9935627
chr1	9931890	9936627
chr1	9972017	9975775

chr1	9972017	9981170
chr1	99728492	99738268
chr1	99740792	99749001
chr1	99747293	99749001
chr1	99874686	99902794
chr1	99910711	99916731
chr1	99993536	99999415
chr1	99999260	100017815
chr20	10556230	10560820
chr20	1125497	1135306
chr20	13270503	13279898
chr20	13417447	13534141
chr20	13417447	13587370
chr20	13483226	13569586
chr20	13483226	13587370
chr20	13483226	13630152
chr20	13528432	13534141
chr20	13528432	13559114
chr20	13528432	13569586
chr20	13528432	13580981
chr20	13559007	13569586
chr20	13559007	13580981
chr20	13559007	13587370
chr20	13559007	13630152
chr20	13569506	13587370
chr20	13569506	13630152
chr20	13580896	13630152
chr20	13587249	13630152
chr20	13623445	13630152
chr20	1372075	1375603
chr20	1445665	1453140
chr20	1452492	1453140
chr20	1452492	1455132
chr20	1452492	1458274
chr20	16427103	16429982
chr20	16494290	16515664
chr20	16507957	16528440
chr20	16526091	16528440
chr20	17943109	17950207
chr20	17943109	17954117
chr20	17947485	17951595
chr20	17947485	17957037
chr20	17949063	17957037
chr20	17950131	17957037
chr20	17951499	17957037

chr20	17952586	17954117
chr20	17952586	17955475
chr20	17952586	17957037
chr20	17955364	17957037
chr20	18297984	18306393
chr20	18298288	18306393
chr20	18305007	18306393
chr20	18389700	18398626
chr20	18412645	18415449
chr20	18412645	18427159
chr20	18433651	18449069
chr20	18480037	18481810
chr20	18510821	18512282
chr20	18510821	18515736
chr20	18510821	18532744
chr20	18510821	18535742
chr20	18524432	18530803
chr20	18527495	18535742
chr20	18527495	18542402
chr20	18532663	18542402
chr20	18543018	18548770
chr20	18543018	18551175
chr20	18543018	18554390
chr20	18551088	18554390
chr20	18593730	18596241
chr20	1895438	1915455
chr20	19935098	19960811
chr20	19935098	19975787
chr20	19956614	19960811
chr20	19970837	19975787
chr20	19970837	19992299
chr20	19990005	19992299
chr20	19990005	19996842
chr20	19992167	19996842
chr20	20520916	20536784
chr20	20531686	20546832
chr20	20616042	20620630
chr20	20635417	20680801
chr20	20639784	20653587
chr20	20653529	20680801
chr20	21132096	21163159
chr20	21136389	21163159
chr20	21145564	21215648
chr20	21161870	21163159
chr20	21214534	21229115

chr20	21214534	21232830
chr20	21214534	21244288
chr20	21326278	21328670
chr20	21326278	21334185
chr20	21326278	21349461
chr20	21326278	21357792
chr20	21326489	21330539
chr20	21326489	21333994
chr20	21332282	21333837
chr20	21339043	21344208
chr20	21339043	21346550
chr20	21340720	21346550
chr20	21346414	21356177
chr20	21348145	21349461
chr20	21348340	21356177
chr20	21354788	21368590
chr20	21365420	21368590
chr20	21365420	21382057
chr20	23389945	23403072
chr20	2492698	2494044
chr20	2492698	2500742
chr20	24968891	24973737
chr20	24968891	24978882
chr20	24969525	24973737
chr20	24969525	24984019
chr20	24978766	24984019
chr20	25021388	25023641
chr20	25030758	25048181
chr20	25209848	25213406
chr20	25209848	25218614
chr20	25302218	25309575
chr20	25306832	25317078
chr20	25307965	25309575
chr20	25314924	25320318
chr20	25323324	25339351
chr20	25413789	25418195
chr20	25413789	25425327
chr20	25413789	25441776
chr20	25417103	25425327
chr20	25418104	25425327
chr20	25418104	25441776
chr20	25455672	25462541
chr20	25461521	25470095
chr20	25489243	25491525
chr20	25489243	25496803

chr20	25489243	25526598
chr20	25517752	25526598
chr20	25632157	25634170
chr20	25632157	25634827
chr20	25634048	25634827
chr20	25685566	25686466
chr20	26018831	26019202
chr20	26018831	26019924
chr20	2655324	2656214
chr20	2663445	2664005
chr20	28583018	28591519
chr20	28590556	28591519
chr20	2923206	2965202
chr20	2923206	2988474
chr20	2947981	2965202
chr20	2964271	2965202
chr20	2964271	2988474
chr20	2986764	2988474
chr20	2986764	3007420
chr20	30388452	30393655
chr20	30388452	30398045
chr20	31544946	31550121
chr20	31550063	31561736
chr20	31742540	31766682
chr20	31757406	31760179
chr20	31757406	31766682
chr20	31757406	31771682
chr20	31770342	31778984
chr20	31778812	31782390
chr20	31782248	31797515
chr20	31792734	31794548
chr20	31792734	31798552
chr20	3191764	3203416
chr20	3200000	3203416
chr20	32133012	32143105
chr20	32133012	32146855
chr20	32141496	32143105
chr20	32155102	32158514
chr20	32215268	32216721
chr20	32316217	32316874
chr20	32366383	32369123
chr20	32366383	32428422
chr20	32453303	32453761
chr20	32704022	32728142
chr20	3270426	3287790

chr20	32780317	32789012
chr20	32784757	32792770
chr20	32788853	32792770
chr20	3278911	3290217
chr20	32791600	32792770
chr20	32795408	32797299
chr20	32795408	32799328
chr20	32825924	32833862
chr20	32825924	32836841
chr20	32825924	32839856
chr20	32825924	32846770
chr20	3290128	3294629
chr20	3315032	3318400
chr20	3322216	3343731
chr20	3330645	3348623
chr20	33366858	33370629
chr20	33366858	33379691
chr20	33372641	33379691
chr20	33379460	33385770
chr20	33379460	33397084
chr20	33385649	33392242
chr20	33392141	33397084
chr20	33394031	33397084
chr20	33606955	33625017
chr20	33619516	33623296
chr20	33619516	33628435
chr20	33619516	33629914
chr20	33619516	33640531
chr20	33628349	33629914
chr20	33628349	33636708
chr20	33636639	33640531
chr20	33740901	33762055
chr20	33740901	33767041
chr20	33766850	33770292
chr20	33766850	33783864
chr20	33783731	33789588
chr20	34031521	34072330
chr20	34072065	34073866
chr20	34072065	34076815
chr20	34072065	34077245
chr20	34072065	34078553
chr20	34093674	34098633
chr20	34096656	34098633
chr20	34096656	34103565
chr20	34096656	34105545

chr20	34098497	34103565
chr20	34290331	34294156
chr20	34290331	34295585
chr20	34369393	34413879
chr20	34369393	34424525
chr20	34408650	34424525
chr20	34412514	34413879
chr20	34413741	34449480
chr20	34413741	34489926
chr20	34424479	34438631
chr20	34424479	34440344
chr20	34424479	34462221
chr20	34424479	34471515
chr20	34438473	34440344
chr20	34438473	34442303
chr20	34438473	34449480
chr20	34438473	34462221
chr20	34438473	34471515
chr20	34440154	34449480
chr20	34440154	34457474
chr20	34442207	34449480
chr20	34457389	34471515
chr20	34462092	34479789
chr20	34462092	34489926
chr20	34462092	34492597
chr20	34470047	34479789
chr20	34470047	34481206
chr20	34470047	34492597
chr20	34471443	34481206
chr20	34471443	34492597
chr20	34477771	34479789
chr20	34477771	34480732
chr20	34480598	34481206
chr20	34489265	34489926
chr20	34489265	34492597
chr20	34489265	34504403
chr20	34519419	34534795
chr20	34575103	34588607
chr20	34634614	34644226
chr20	34634614	34645334
chr20	34727258	34736758
chr20	34768463	34782404
chr20	34782120	34792563
chr20	34854530	34854923
chr20	34913095	34921866

chr20	34931335	34937023
chr20	34935575	34937023
chr20	34941712	34951860
chr20	35013007	35021356
chr20	35016007	35021356
chr20	35035122	35055031
chr20	35044504	35057571
chr20	35069295	35078174
chr20	35118597	35142478
chr20	35123889	35126375
chr20	35131641	35142478
chr20	35246839	35254754
chr20	35246839	35269898
chr20	35314687	35384133
chr20	35347163	35384133
chr20	3535252	3540335
chr20	3535252	3549338
chr20	35366556	35382025
chr20	35366556	35384133
chr20	35366556	35394196
chr20	35374183	35384133
chr20	3540221	3549338
chr20	3545761	3549338
chr20	35479230	35480145
chr20	35501844	35505005
chr20	35507737	35510054
chr20	35508942	35510054
chr20	35547411	35548865
chr20	35548508	35548865
chr20	3559392	3560905
chr20	3562275	3565432
chr20	35631113	35658982
chr20	35632310	35632696
chr20	3565347	3572951
chr20	35672754	35681981
chr20	35680736	35681981
chr20	35680736	35690565
chr20	35709223	35732135
chr20	35713018	35714389
chr20	35713018	35725155
chr20	35714184	35725155
chr20	35714184	35732135
chr20	35716739	35725155
chr20	35716739	35732135
chr20	35716739	35739017

chr20	35721739	35725155
chr20	35721739	35732135
chr20	35721739	35734257
chr20	35724569	35725155
chr20	35724569	35729527
chr20	35724569	35732135
chr20	35729311	35732135
chr20	35731940	35739017
chr20	35801490	35842744
chr20	35801490	35847434
chr20	35801490	35858381
chr20	35801490	35863400
chr20	35801490	35931444
chr20	35842572	35847434
chr20	35842572	35858381
chr20	35847349	35863400
chr20	35847349	35871829
chr20	35858301	35863400
chr20	35858301	35871829
chr20	35863012	35871829
chr20	35863012	35899648
chr20	35863012	35931444
chr20	35869437	35871829
chr20	35871649	35917662
chr20	35913248	35914197
chr20	36212271	36222394
chr20	36496704	36500611
chr20	36607714	36609730
chr20	36656359	36665301
chr20	36656359	36666392
chr20	36656359	36682578
chr20	36660336	36666392
chr20	36660336	36671397
chr20	36665045	36671397
chr20	36665045	36682578
chr20	36665045	36688784
chr20	36680815	36688784
chr20	36684412	36688784
chr20	36687491	36688784
chr20	36758086	36768042
chr20	36793248	36797573
chr20	36802855	36805957
chr20	36802855	36808743
chr20	36804734	36805957
chr20	36829053	36839441

chr20	36904156	36905503
chr20	36911217	36912552
chr20	36911217	36919519
chr20	36916721	36919519
chr20	37007410	37020730
chr20	37007410	37032876
chr20	37007410	37035508
chr20	37007410	37040285
chr20	37022649	37068186
chr20	37032664	37068186
chr20	37040152	37047190
chr20	37040152	37055656
chr20	37040152	37068186
chr20	37044085	37047190
chr20	37044085	37065473
chr20	37047052	37068186
chr20	37056145	37062270
chr20	37056145	37065473
chr20	37056145	37068186
chr20	37061102	37062270
chr20	37061102	37065473
chr20	37065423	37068186
chr20	37066723	37068186
chr20	37198396	37213865
chr20	37203884	37213865
chr20	37207272	37210165
chr20	37232295	37234095
chr20	37382618	37386174
chr20	37386074	37393993
chr20	37393894	37396311
chr20	3758535	3760186
chr20	37732878	37842419
chr20	37746467	37768044
chr20	37765196	37768044
chr20	37765196	37779335
chr20	37765196	37803048
chr20	37777344	37779335
chr20	37777344	37803048
chr20	38006196	38013857
chr20	38040434	38059520
chr20	38040434	38066256
chr20	38048347	38066256
chr20	38057531	38059520
chr20	38057531	38066256
chr20	38448661	38450447

chr20	38488402	38493132
chr20	38488402	38499633
chr20	38488402	38509208
chr20	38488402	38518000
chr20	38497352	38509208
chr20	38499446	38509208
chr20	38524777	38532859
chr20	38531166	38532859
chr20	38754956	38756039
chr20	38765401	38767596
chr20	38941958	38948000
chr20	38983698	38994880
chr20	38988812	38994880
chr20	39001729	39006356
chr20	39023689	39030775
chr20	3907925	3910830
chr20	3907925	3912634
chr20	3907925	3917050
chr20	3907925	3918796
chr20	3910576	3912634
chr20	3910576	3918796
chr20	3912457	3918796
chr20	3945176	3974400
chr20	41092471	41098337
chr20	41092471	41100243
chr20	41092471	41101353
chr20	41112781	41114155
chr20	41133486	41135811
chr20	41168766	41169175
chr20	41423491	41426153
chr20	41437273	41440129
chr20	41437273	41447971
chr20	41439999	41447971
chr20	41447881	41457428
chr20	41512845	41514952
chr20	41512845	41551360
chr20	41533049	41551360
chr20	417525	417930
chr20	417525	422238
chr20	43459770	43460598
chr20	43594692	43613976
chr20	43594692	43620925
chr20	43596434	43613976
chr20	43613849	43620925
chr20	43613849	43624045

chr20	43620856	43642624
chr20	43673805	43687072
chr20	43673805	43700044
chr20	43686851	43700044
chr20	43702489	43705358
chr20	43711487	43715283
chr20	43973366	44006792
chr20	44006546	44064857
chr20	44051305	44054526
chr20	441587	442043
chr20	441587	448074
chr20	44202960	44206998
chr20	44479984	44484530
chr20	44479984	44486706
chr20	44484336	44486706
chr20	44500257	44506996
chr20	44503814	44504891
chr20	44503814	44506996
chr20	44506826	44514040
chr20	44509890	44511368
chr20	44509890	44512994
chr20	44509890	44514040
chr20	44511288	44514040
chr20	44582584	44589866
chr20	44912659	44916871
chr20	44916755	44919277
chr20	44916755	44924256
chr20	44918905	44919277
chr20	44921593	44924256
chr20	44972077	44981943
chr20	44972077	44987296
chr20	44972077	44995257
chr20	44972077	45001353
chr20	44978442	44987296
chr20	44978442	44997306
chr20	44978442	45001353
chr20	44981828	44987296
chr20	44995089	45001353
chr20	44997168	45001353
chr20	45000391	45001353
chr20	45000391	45025130
chr20	45330365	45335920
chr20	45795344	45801458
chr20	45950452	45952521
chr20	46063359	46079453

chr20	46068501	46079453
chr20	46070209	46079453
chr20	46368890	46394532
chr20	46374331	46393143
chr20	46380252	46394503
chr20	46393092	46394503
chr20	46393092	46398771
chr20	46588058	46613725
chr20	46610445	46613725
chr20	47227202	47236516
chr20	47246007	47249439
chr20	47249286	47262428
chr20	47262287	47276795
chr20	47262287	47283648
chr20	47262287	47347926
chr20	47276313	47283648
chr20	47276313	47287284
chr20	47276313	47290274
chr20	47276313	47347926
chr20	47282101	47287284
chr20	47282101	47347926
chr20	47294665	47298872
chr20	47294665	47298947
chr20	47298728	47310204
chr20	47623910	47633636
chr20	47625380	47628023
chr20	47634047	47640051
chr20	47635321	47637783
chr20	47642212	47651276
chr20	486375	492364
chr20	48690946	48708421
chr20	48726289	48747880
chr20	4883641	4884823
chr20	4883641	4899712
chr20	48941198	48963898
chr20	48951322	48952884
chr20	48951322	48953790
chr20	48951322	48963898
chr20	48952704	48963898
chr20	48953555	48963898
chr20	48953555	48969277
chr20	48971119	48985613
chr20	48972325	48973284
chr20	48974765	48976199
chr20	48976015	48985613

chr20	48988303	48989684
chr20	48998192	48998505
chr20	48998192	49005221
chr20	4902441	4932716
chr20	49058452	49067280
chr20	49058452	49068822
chr20	49058452	49070297
chr20	49063201	49067280
chr20	49063201	49070297
chr20	49063201	49078622
chr20	49066191	49066510
chr20	49066191	49067280
chr20	49066191	49070297
chr20	49066191	49072697
chr20	49066191	49075520
chr20	49066191	49085386
chr20	49067189	49070297
chr20	49067189	49072697
chr20	49072285	49072697
chr20	49072285	49074850
chr20	49072285	49075520
chr20	49072285	49089746
chr20	49072285	49092127
chr20	49074784	49077064
chr20	49074784	49078622
chr20	49074784	49089746
chr20	49074784	49091022
chr20	49074784	49092127
chr20	49076979	49091022
chr20	49084025	49089397
chr20	49085282	49089397
chr20	49088008	49089746
chr20	49088008	49091022
chr20	49090741	49091022
chr20	49090741	49094963
chr20	49092045	49094963
chr20	49094139	49094963
chr20	49115781	49124605
chr20	49117125	49124587
chr20	49119981	49135931
chr20	49135832	49154071
chr20	49135832	49166285
chr20	49135832	49174269
chr20	49151581	49154071
chr20	49151581	49166285

chr20	49151581	49174269
chr20	49165996	49174269
chr20	49226429	49230349
chr20	49233305	49233709
chr20	49233305	49236510
chr20	49233305	49239338
chr20	49241892	49242681
chr20	49251526	49253811
chr20	49260462	49263483
chr20	49263333	49266266
chr20	49264715	49266266
chr20	49279103	49280964
chr20	4932454	4970919
chr20	49637340	49643650
chr20	49637340	49647078
chr20	49637340	49656702
chr20	49639677	49640665
chr20	49839540	49855581
chr20	49839540	49864844
chr20	49849578	49855581
chr20	49855437	49864844
chr20	49855437	49874821
chr20	49941560	49949355
chr20	49945381	49949355
chr20	50127974	50130947
chr20	50561362	50574654
chr20	50574516	50579329
chr20	50902016	50904001
chr20	50902016	50904849
chr20	50940864	50955285
chr20	50942030	50955285
chr20	50945846	50955285
chr20	5106187	5109445
chr20	51473979	51475660
chr20	51516783	51523596
chr20	51604816	51610165
chr20	51613676	51619043
chr20	51617489	51619043
chr20	51639342	51690819
chr20	51639342	51697482
chr20	51656937	51671257
chr20	51656937	51690819
chr20	51669996	51690819
chr20	51669996	51697482
chr20	51674152	51676208

chr20	51674152	51689139
chr20	51674152	51690819
chr20	51674152	51697482
chr20	51690738	51697482
chr20	51694007	51697482
chr20	51712965	51729978
chr20	51725818	51729978
chr20	5173522	5178956
chr20	5173522	5183143
chr20	5175182	5178956
chr20	52088391	52098587
chr20	52097372	52098587
chr20	52160122	52187065
chr20	54157168	54169688
chr20	54157168	54171670
chr20	5575815	5580131
chr20	56370484	56383176
chr20	56370484	56388202
chr20	56373407	56386533
chr20	56373407	56388202
chr20	56381432	56384324
chr20	56381432	56386533
chr20	56381432	56388202
chr20	56381432	56390665
chr20	56382984	56386533
chr20	56382984	56388202
chr20	56386256	56388202
chr20	56388686	56390665
chr20	56395520	56397841
chr20	56473300	56477124
chr20	56473300	56484189
chr20	56474677	56477124
chr20	56631204	56634268
chr20	58418210	58434705
chr20	58418210	58439025
chr20	58418210	58441083
chr20	58434601	58441083
chr20	58438944	58441083
chr20	58667489	58671297
chr20	58667489	58673711
chr20	58667986	58673711
chr20	58895611	58905480
chr20	58895611	58909579
chr20	58988913	58989640
chr20	5941950	5943683

chr20	5952010	5952528
chr20	5952010	5957229
chr20	5958527	5963359
chr20	5958527	5968025
chr20	5958527	5972037
chr20	5958527	5973196
chr20	5958527	5978017
chr20	5958527	5983165
chr20	5958527	5985000
chr20	5967435	5968025
chr20	5973055	5983165
chr20	5984780	5986131
chr20	6085065	6094988
chr20	6094938	6097631
chr20	6096901	6097631
chr20	6112476	6116044
chr20	61844667	61852898
chr20	61997549	62000721
chr20	61997549	62014707
chr20	62007546	62014707
chr20	62009051	62014707
chr20	62124616	62131506
chr20	62137363	62140944
chr20	62138170	62140944
chr20	62139074	62140944
chr20	62139780	62140944
chr20	62162751	62163622
chr20	62162751	62164246
chr20	62195801	62199257
chr20	62256056	62260125
chr20	62256056	62263691
chr20	62256056	62264718
chr20	62256056	62272259
chr20	62273308	62284169
chr20	62286582	62289330
chr20	62336339	62362552
chr20	62337803	62338369
chr20	62345817	62347028
chr20	62346912	62353251
chr20	62656358	62665088
chr20	62656358	62669078
chr20	62660411	62661175
chr20	62667744	62669078
chr20	62854015	62857635
chr20	62854015	62857638

chr20	62854015	62859526
chr20	62854015	62860308
chr20	62857394	62859526
chr20	62857394	62860308
chr20	62890959	62914406
chr20	62896232	62896996
chr20	62905886	62914406
chr20	62909698	62914406
chr20	62941490	62943692
chr20	62942986	62943692
chr20	63278116	63278995
chr20	63278116	63284922
chr20	63424176	63439708
chr20	63424176	63446837
chr20	63442405	63446837
chr20	63521283	63521589
chr20	63659232	63667553
chr20	63672555	63674093
chr20	63672555	63680719
chr20	63741369	63742056
chr20	63775677	63790790
chr20	63928334	63931022
chr20	64264428	64265429
chr20	6446722	6473655
chr20	7996025	8010315
chr20	9217389	9339037
chr20	9217389	9365514
chr20	9307799	9339037
chr20	9337125	9339037
chr20	9338007	9380162
chr20	9371213	9393678
chr20	9389878	9395618
chr20	9407916	9459810
chr20	9407916	9473365
chr20	9419806	9423952
chr20	9435559	9457489
chr20	9437001	9444243
chr20	9437001	9457489
chr20	9457413	9473365
chr20	9468570	9473365
chr21	15014343	15043574
chr21	15762890	15766141
chr21	15762890	15778027
chr21	15762890	15805258
chr21	15762890	15811210

chr21	15777903	15791664
chr21	15777903	15805258
chr21	15777903	15811210
chr21	15791501	15842540
chr21	15799756	15811210
chr21	15799756	15842540
chr21	15805120	15842540
chr21	15808808	15831629
chr21	15811136	15827203
chr21	15833347	15842540
chr21	15864267	15874526
chr21	21280577	21286412
chr21	21280577	21292241
chr21	21280577	21338534
chr21	21324382	21338534
chr21	22041219	22096586
chr21	25588834	25593958
chr21	25588834	25601467
chr21	25592811	25601467
chr21	25597301	25599866
chr21	25597301	25603935
chr21	25683882	25698879
chr21	25741572	25752234
chr21	25741572	25758204
chr21	25741572	25762365
chr21	25891721	25911962
chr21	25897572	25911962
chr21	25954589	25955755
chr21	25954589	25982477
chr21	25954589	25997416
chr21	25954589	26000182
chr21	25975069	25982477
chr21	25975069	25997416
chr21	25975069	26000182
chr21	25975953	25982477
chr21	26021839	26053348
chr21	26050999	26053348
chr21	28941219	28947606
chr21	28945806	28957476
chr21	28982315	28986934
chr21	28986138	28986934
chr21	29046666	29050178
chr21	29060540	29065105
chr21	29060540	29071988
chr21	29061495	29062415

chr21	29067555	29071988
chr21	29305203	29321514
chr21	29305203	29329693
chr21	29321220	29329693
chr21	29326058	29329693
chr21	29326058	29351813
chr21	29351633	29352663
chr21	31135932	31147003
chr21	31152635	31154426
chr21	31164961	31195305
chr21	31164961	31203012
chr21	31182420	31195305
chr21	31182420	31203012
chr21	31213397	31217699
chr21	31245487	31252189
chr21	31266009	31276908
chr21	31685048	31701918
chr21	31690796	31694289
chr21	31700994	31705467
chr21	31700994	31706357
chr21	32368402	32375483
chr21	32368402	32384464
chr21	32372506	32378541
chr21	32378444	32384464
chr21	32383421	32385684
chr21	32453311	32467848
chr21	32453311	32495170
chr21	32457596	32467848
chr21	32656686	32666573
chr21	32694227	32695282
chr21	32694227	32702047
chr21	32738172	32755353
chr21	32743677	32751218
chr21	32748498	32755353
chr21	33241839	33245074
chr21	33241839	33246890
chr21	33241885	33252830
chr21	33246717	33248854
chr21	33340998	33343676
chr21	33345245	33349543
chr21	33414887	33432871
chr21	33421479	33432871
chr21	33426883	33432871
chr21	33432176	33432871
chr21	33455726	33467122

chr21	33460412	33467122
chr21	33460412	33468926
chr21	33465341	33468926
chr21	33520905	33539356
chr21	33524768	33539356
chr21	33530758	33535320
chr21	33530758	33539356
chr21	33534578	33535320
chr21	33534578	33539356
chr21	33579349	33583666
chr21	33579349	33584768
chr21	33581301	33583666
chr21	33581301	33586181
chr21	33583487	33586181
chr21	33718796	33722651
chr21	33718796	33735204
chr21	33722587	33735204
chr21	33735043	33761986
chr21	33750142	33755397
chr21	33750142	33761986
chr21	33750142	33766012
chr21	33765874	33767828
chr21	33781460	33782133
chr21	33810974	33829745
chr21	33813912	33834424
chr21	33823486	33826863
chr21	33903934	33914500
chr21	33907340	33909211
chr21	34521498	34523710
chr21	34834409	34859578
chr21	34834409	34887096
chr21	34859473	34887096
chr21	36045581	36058973
chr21	36199069	36208904
chr21	36208714	36212097
chr21	36223230	36227877
chr21	36237261	36239955
chr21	36245047	36248568
chr21	36247516	36248568
chr21	36247516	36251284
chr21	36247516	36260732
chr21	36263545	36270157
chr21	36281482	36292233
chr21	36288755	36292233
chr21	36333645	36341546

chr21	36333645	36345031
chr21	36336873	36338921
chr21	36338773	36344707
chr21	36344578	36349408
chr21	36344578	36364259
chr21	36362182	36364259
chr21	36362182	36369876
chr21	36364092	36372531
chr21	36386059	36387730
chr21	36387597	36399605
chr21	36387597	36402851
chr21	36391550	36394650
chr21	36391550	36397511
chr21	36391550	36402851
chr21	36394546	36399605
chr21	36402757	36411604
chr21	36709167	36731151
chr21	36712532	36731151
chr21	36719820	36723130
chr21	36719820	36731151
chr21	36741716	36745136
chr21	36759726	36765172
chr21	37087246	37088886
chr21	37087246	37096643
chr21	37087246	37108446
chr21	37088195	37094090
chr21	37108391	37123028
chr21	37108391	37126143
chr21	37108391	37132766
chr21	37108391	37135514
chr21	37108391	37138714
chr21	37108391	37140673
chr21	37108391	37147603
chr21	37108391	37150170
chr21	37132681	37140673
chr21	37144524	37147603
chr21	37144524	37148647
chr21	37187048	37196036
chr21	37232376	37240639
chr21	37238459	37240639
chr21	37420298	37472880
chr21	37420298	37480826
chr21	37472683	37480826
chr21	37472683	37496258
chr21	37478180	37486614

chr21	39178448	39179986
chr21	39198762	39200386
chr21	39200218	39202545
chr21	39202324	39213553
chr21	39206107	39212707
chr21	39206107	39213553
chr21	39206107	39229436
chr21	39213480	39218660
chr21	39213480	39225197
chr21	39215236	39218660
chr21	39215236	39225197
chr21	39218151	39225197
chr21	39218151	39229436
chr21	39224407	39225197
chr21	39224407	39229436
chr21	39224407	39232498
chr21	39224407	39238573
chr21	39232176	39232498
chr21	39232176	39238573
chr21	39232176	39250889
chr21	39236594	39238573
chr21	39247700	39258672
chr21	39258486	39274472
chr21	39264459	39298582
chr21	39264890	39295903
chr21	39269898	39270433
chr21	39269898	39274472
chr21	39270282	39280248
chr21	39274373	39277351
chr21	39274373	39278813
chr21	39276172	39278813
chr21	39278742	39298582
chr21	39293810	39298582
chr21	39390697	39393280
chr21	41226265	41246063
chr21	41226265	41257326
chr21	41237512	41246063
chr21	41237512	41257326
chr21	41243375	41250901
chr21	41815704	41823449
chr21	41854565	41857329
chr21	41854565	41860372
chr21	41899052	41922085
chr21	41901621	41909523
chr21	41912331	41922085

chr21	41914597	41922085
chr21	42751115	42772520
chr21	42753989	42760432
chr21	42850040	42859722
chr21	42850040	42863596
chr21	42850040	42876767
chr21	42852254	42863596
chr21	42852254	42876767
chr21	42859661	42863596
chr21	42859661	42876767
chr21	42862281	42863596
chr21	42862281	42876767
chr21	42873550	42876767
chr21	42896926	42904276
chr21	43004325	43010224
chr21	43004325	43018230
chr21	43004325	43021431
chr21	43004325	43024947
chr21	43004325	43028874
chr21	43592224	43613947
chr21	43592224	43644392
chr21	43613829	43633421
chr21	43613829	43656737
chr21	43630321	43633421
chr21	43630321	43644392
chr21	43630321	43656737
chr21	43644288	43656737
chr21	43669883	43673955
chr21	43676732	43691502
chr21	43686803	43688240
chr21	43734068	43743807
chr21	43797906	43800614
chr21	43967945	43971490
chr21	43967945	43978121
chr21	44032090	44037927
chr21	44032090	44052476
chr21	44052279	44055893
chr21	44052279	44059214
chr21	44052279	44063785
chr21	44052279	44077784
chr21	44055697	44094233
chr21	44063537	44075153
chr21	44076551	44080127
chr21	44077692	44080127
chr21	44079563	44080127

chr21	44094062	44098534
chr21	44855209	44861271
chr21	45128401	45134832
chr21	45171609	45176664
chr21	45171609	45185091
chr21	45175729	45204736
chr21	45180329	45185091
chr21	45269838	45278169
chr21	45486860	45488444
chr21	45525816	45532148
chr21	45525816	45538008
chr21	45530769	45538008
chr21	46270400	46285394
chr21	46273387	46277717
chr21	46279992	46280575
chr21	46314715	46315547
chr21	46326376	46334768
chr21	46346127	46347512
chr21	46349011	46349820
chr21	46363479	46367139
chr21	46381693	46385983
chr21	46381693	46391376
chr21	46388741	46391376
chr21	46484756	46497107
chr21	46484756	46509376
chr21	46484756	46511614
chr21	46496987	46511614
chr21	46496987	46539991
chr21	46504360	46529183
chr21	46509256	46539991
chr21	46529091	46534687
chr21	46537223	46541895
chr21	46643534	46644488
chr21	46643534	46649739
chr21	46644305	46648619
chr22	16611657	16614178
chr22	16637039	16638740
chr22	17044872	17047426
chr22	17097061	17102302
chr22	17141058	17149745
chr22	17143097	17149745
chr22	17145023	17149745
chr22	17499409	17500735
chr22	17500630	17505016
chr22	17503081	17505016

chr22	17594528	17601181
chr22	17655661	17702386
chr22	17683213	17689142
chr22	17683213	17702386
chr22	17688985	17696210
chr22	17808843	17823060
chr22	17810702	17823060
chr22	17864898	17872023
chr22	17900841	17906886
chr22	19063201	19068225
chr22	19063201	19089490
chr22	19139611	19142894
chr22	19187557	19199841
chr22	19221950	19232598
chr22	19355759	19356288
chr22	19375630	19378066
chr22	19375630	19410778
chr22	19383619	19410778
chr22	19405785	19408593
chr22	19820597	19821375
chr22	19878365	19883461
chr22	19911376	19931098
chr22	20141216	20141530
chr22	20400631	20401372
chr22	20446442	20489244
chr22	20457807	20489244
chr22	20551435	20555148
chr22	20551435	20568631
chr22	20554935	20566817
chr22	20764816	20765693
chr22	20810966	20813506
chr22	20824325	20838731
chr22	20870336	20881134
chr22	21483019	21484134
chr22	21486799	21488381
chr22	21681298	21684913
chr22	21681298	21688849
chr22	21769194	21799128
chr22	21799011	21807846
chr22	21965284	21975807
chr22	23260949	23268515
chr22	23273080	23273774
chr22	23285032	23292638
chr22	23292540	23295155
chr22	23309423	23314714

chr22	23659864	23683867
chr22	23683724	23695517
chr22	23683724	23709867
chr22	23705428	23709867
chr22	23714186	23715194
chr22	23791755	23793688
chr22	24035443	24038461
chr22	24035443	24043084
chr22	24035443	24076284
chr22	24072353	24076284
chr22	24087451	24113748
chr22	24091582	24134415
chr22	24113565	24134415
chr22	24302194	24313466
chr22	24302194	24338477
chr22	24302194	24347176
chr22	24328845	24330431
chr22	24347085	24369320
chr22	24365475	24369320
chr22	25170819	25185119
chr22	25455711	25459515
chr22	25457258	25459515
chr22	25604376	25678915
chr22	25604376	25690283
chr22	25661575	25674528
chr22	25661575	25678915
chr22	25661575	25690283
chr22	25663629	25674528
chr22	25663629	25687667
chr22	25667738	25674528
chr22	25667738	25678915
chr22	25672295	25690283
chr22	25672295	25695214
chr22	25678815	25695214
chr22	26457858	26458577
chr22	26496072	26496889
chr22	27873737	27897892
chr22	27873737	27911109
chr22	27873737	27914347
chr22	27894554	27897892
chr22	27894554	27914347
chr22	27910963	27914347
chr22	27985248	27992663
chr22	28014247	28030366
chr22	28094079	28096408

chr22	28105278	28108403
chr22	28296197	28297852
chr22	28296197	28306643
chr22	28694031	28695873
chr22	28694031	28699937
chr22	28694031	28719485
chr22	28695709	28699937
chr22	28699837	28712017
chr22	28699837	28719485
chr22	28699837	28725367
chr22	28703504	28725367
chr22	28703504	28734727
chr22	28710005	28719485
chr22	28710005	28725367
chr22	28710005	28734727
chr22	28719394	28725367
chr22	28719394	28734727
chr22	28724976	28725367
chr22	28724976	28734727
chr22	28744614	28746008
chr22	28987075	29043430
chr22	29042494	29044890
chr22	29094257	29098953
chr22	29121356	29125416
chr22	29288605	29292606
chr22	29338989	29342383
chr22	29349217	29354871
chr22	29350034	29354871
chr22	29511105	29519120
chr22	29539329	29542956
chr22	29549051	29550843
chr22	29558869	29570532
chr22	29636750	29639212
chr22	29636750	29642285
chr22	29636750	29681601
chr22	29642201	29661339
chr22	29654656	29665064
chr22	29813429	29814767
chr22	29813429	29822464
chr22	29825086	29832342
chr22	29957035	29979052
chr22	29970975	29979052
chr22	29970975	29991670
chr22	29970975	29998857
chr22	29978441	29991670

chr22	29978441	29998857
chr22	29988479	29991670
chr22	30012367	30018072
chr22	30285831	30287560
chr22	30407094	30409486
chr22	30407094	30410679
chr22	30415758	30416403
chr22	30694207	30741369
chr22	30946340	30958694
chr22	31089698	31090180
chr22	31095302	31097338
chr22	31095302	31101049
chr22	31258290	31260077
chr22	31258290	31262791
chr22	31265945	31273507
chr22	31403028	31411081
chr22	31411011	31423410
chr22	31411011	31427182
chr22	31420255	31423410
chr22	31420255	31427182
chr22	31441773	31443094
chr22	31447425	31448232
chr22	31447425	31449531
chr22	31447425	31450360
chr22	31454143	31463133
chr22	31455851	31463133
chr22	31462931	31463967
chr22	31463680	31468302
chr22	31468174	31471917
chr22	31508254	31546971
chr22	31508254	31550348
chr22	31528689	31550348
chr22	31528689	31557059
chr22	31556941	31561392
chr22	31556941	31573214
chr22	31580271	31589577
chr22	31583874	31589577
chr22	31583874	31602785
chr22	31602211	31602785
chr22	31620552	31621885
chr22	31623683	31626016
chr22	31688155	31704726
chr22	31688155	31725610
chr22	31701655	31704726
chr22	31725537	31738911

chr22	31758545	31765060
chr22	31758545	31766668
chr22	31758545	31768863
chr22	31758545	31778168
chr22	31764974	31768863
chr22	31766584	31784875
chr22	31778098	31792817
chr22	31792744	31798656
chr22	31792744	31802838
chr22	31798581	31804223
chr22	31810520	31815212
chr22	31845017	31846967
chr22	31870589	31879752
chr22	32478980	32485209
chr22	32478980	32487828
chr22	32478980	32493281
chr22	32479132	32485209
chr22	32479132	32487828
chr22	32487744	32495530
chr22	33304228	33316248
chr22	33316084	33337801
chr22	33564847	33604558
chr22	33604434	33626326
chr22	33761370	33856802
chr22	35262322	35263226
chr22	35283961	35288407
chr22	35756104	35761468
chr22	35756104	35781746
chr22	35765422	35778078
chr22	35781599	35810004
chr22	35809779	35938897
chr22	36285657	36285953
chr22	36298918	36301665
chr22	36316516	36318325
chr22	36341369	36349255
chr22	36501240	36504766
chr22	36523208	36524732
chr22	36523208	36526131
chr22	37053099	37059438
chr22	37495516	37497178
chr22	37495759	37497178
chr22	37496448	37497178
chr22	37496448	37510421
chr22	37510211	37518108
chr22	37618447	37621696

chr22	37620812	37623467
chr22	37623326	37623633
chr22	37687909	37688992
chr22	37812886	37813609
chr22	37815125	37816725
chr22	37850014	37851490
chr22	37858678	37870347
chr22	37863271	37878171
chr22	37870175	37878171
chr22	37874369	37876011
chr22	37874369	37878171
chr22	37875840	37878171
chr22	37911951	37919178
chr22	38224784	38231334
chr22	38245934	38248019
chr22	38293254	38314169
chr22	38298785	38300065
chr22	38298785	38314169
chr22	38299894	38314169
chr22	38302860	38303248
chr22	38361398	38380990
chr22	38492055	38498150
chr22	38492055	38501280
chr22	38493709	38494802
chr22	38493709	38495046
chr22	38493709	38499499
chr22	38493709	38501280
chr22	38494020	38501280
chr22	38494629	38495046
chr22	38494629	38501280
chr22	38495795	38498573
chr22	38495795	38499499
chr22	38495795	38501280
chr22	38498084	38498573
chr22	38498084	38501280
chr22	38521607	38568289
chr22	38537591	38568289
chr22	38552665	38568289
chr22	38562286	38568289
chr22	38620211	38623279
chr22	38715906	38717000
chr22	39374315	39376197
chr22	39416790	39421971
chr22	39682495	39684498
chr22	40117054	40156182

chr22	40261831	40270280
chr22	40261831	40273600
chr22	40270121	40273600
chr22	40273424	40280143
chr22	40273424	40285770
chr22	40277076	40285770
chr22	40312504	40316012
chr22	40353072	40359306
chr22	40358863	40359306
chr22	40358863	40360492
chr22	40358863	40363071
chr22	40400695	40402205
chr22	40429605	40431480
chr22	40429605	40435554
chr22	40463220	40594735
chr22	40552105	40594735
chr22	40777039	40792676
chr22	40777039	40799083
chr22	40779008	40792676
chr22	40832568	40848369
chr22	40835559	40850880
chr22	40840625	40850880
chr22	40844838	40850880
chr22	40868998	40886515
chr22	40881769	40886515
chr22	40886312	40914324
chr22	40907586	40914324
chr22	40907586	40924482
chr22	40914238	40924482
chr22	40953554	40967884
chr22	41117186	41140257
chr22	41125863	41127748
chr22	41125863	41140257
chr22	41135812	41137790
chr22	41135812	41140257
chr22	41137652	41141222
chr22	41140139	41141222
chr22	41146738	41150198
chr22	41158411	41162779
chr22	41172498	41173784
chr22	41219418	41221287
chr22	41256020	41258106
chr22	41257947	41261580
chr22	41264663	41274727
chr22	41264663	41281082

chr22	41268096	41281082
chr22	41274599	41281082
chr22	41325563	41325918
chr22	41330022	41343576
chr22	41338312	41339191
chr22	41342528	41343576
chr22	41530686	41540795
chr22	41540698	41544121
chr22	41583958	41584603
chr22	41636112	41637791
chr22	41637607	41647082
chr22	41637607	41650891
chr22	41646895	41650891
chr22	41646895	41657032
chr22	41650722	41653690
chr22	41656902	41658352
chr22	41658251	41661444
chr22	41680243	41682587
chr22	41808874	41810000
chr22	41808874	41810291
chr22	41808874	41813822
chr22	41866830	41868792
chr22	41866830	41871035
chr22	41943609	41945304
chr22	42179608	42215341
chr22	42599697	42603160
chr22	42799038	42817857
chr22	42808766	42817857
chr22	42808766	42822409
chr22	42810812	42826999
chr22	42817141	42817857
chr22	42823655	42835493
chr22	42826939	42835493
chr22	42876136	42882304
chr22	43059383	43064324
chr22	43989110	43990406
chr22	44151482	44163930
chr22	44285427	44300977
chr22	44885425	44888121
chr22	44885425	44896083
chr22	44887962	44896083
chr22	44913821	44920490
chr22	45171599	45178900
chr22	45178237	45181367
chr22	45330602	45332710

chr22	45425947	45431066
chr22	45518681	45550615
chr22	45525542	45528009
chr22	45574510	45577108
chr22	45689711	45693078
chr22	45689711	45702847
chr22	45689711	45718493
chr22	45689711	45729590
chr22	45689711	45740538
chr22	45692995	45702847
chr22	45692995	45718493
chr22	45692995	45740538
chr22	45700281	45718493
chr22	45700281	45729590
chr22	45700281	45740538
chr22	45718412	45740538
chr22	45729424	45740538
chr22	46176752	46198591
chr22	46218262	46220014
chr22	46308149	46308943
chr22	46308149	46323262
chr22	46308149	46326654
chr22	46312140	46326654
chr22	46316031	46326654
chr22	46337778	46346544
chr22	46343261	46346544
chr22	46384542	46386585
chr22	46384542	46389499
chr22	46386401	46389499
chr22	46390391	46391816
chr22	46433392	46436289
chr22	46433392	46439411
chr22	46436173	46439411
chr22	46668688	46675552
chr22	46720085	46721015
chr22	46792519	46797620
chr22	46878652	46894846
chr22	46878652	46912188
chr22	46912073	46997709
chr22	46912073	47037198
chr22	46974289	46997709
chr22	46974289	47037198
chr22	46997633	47037198
chr22	47686199	47686938
chr22	47686199	47687206

chr22	47686199	47704747
chr22	49775590	49777813
chr22	49775590	49794294
chr22	49775590	49804360
chr22	49787389	49799119
chr22	49787389	49804360
chr22	49787389	49824331
chr22	49794033	49804360
chr22	49797804	49799119
chr22	49797804	49804360
chr22	50206316	50210911
chr22	50224140	50226378
chr22	50372019	50394135
chr22	50422253	50423614
chr22	50459254	50464917
chr22	50703859	50715753
chr22	50776669	50782351
chr2	10046149	10048595
chr2	100566502	100569721
chr2	101002701	101006071
chr2	101036017	101050641
chr2	101050400	101059539
chr2	101054107	101059539
chr2	10124716	10129154
chr2	101257790	101262691
chr2	101257790	101265047
chr2	101262538	101266879
chr2	10126874	10127220
chr2	101281858	101286177
chr2	101281858	101295181
chr2	101288972	101295181
chr2	101294013	101295181
chr2	10141853	10142375
chr2	102657563	102665350
chr2	102683260	102695113
chr2	102694413	102704675
chr2	105049170	105080062
chr2	105284315	105298705
chr2	105296355	105298705
chr2	105816429	105855289
chr2	10600852	10603157
chr2	10600852	10607311
chr2	10602775	10607311
chr2	106112645	106125679
chr2	106112645	106129778

chr2	106122969	106129778
chr2	106122969	106145370
chr2	106122969	106166083
chr2	106129673	106166083
chr2	106145189	106166083
chr2	10644319	10657891
chr2	10644319	10663044
chr2	10644319	10668723
chr2	10644319	10671690
chr2	10644319	10684612
chr2	10654480	10668723
chr2	10654480	10671690
chr2	10657741	10684612
chr2	10659171	10668723
chr2	10662958	10668723
chr2	10662958	10671690
chr2	10667217	10671690
chr2	10671553	10684612
chr2	10675793	10684612
chr2	10787280	10789889
chr2	10787280	10802640
chr2	10788696	10789889
chr2	10788696	10790833
chr2	10788696	10793202
chr2	10790718	10793202
chr2	10791794	10793202
chr2	10791794	10797757
chr2	10797080	10802640
chr2	108659604	108673029
chr2	108729131	108740681
chr2	108740488	108753563
chr2	108752997	108753563
chr2	108752997	108773046
chr2	108771700	108773046
chr2	108788031	108816105
chr2	108788851	108794721
chr2	108788851	108812927
chr2	108788851	108816105
chr2	108791674	108805008
chr2	108791674	108816105
chr2	108791674	108846930
chr2	108791674	108856970
chr2	108812630	108812927
chr2	108839184	108856970
chr2	108856793	108873589

chr2	109347673	109398943
chr2	109553086	109593119
chr2	109564365	109565859
chr2	109564365	109567976
chr2	109564365	109585838
chr2	109564365	109593119
chr2	109567814	109585838
chr2	109574580	109585838
chr2	109574580	109593119
chr2	109585125	109585838
chr2	109585125	109593119
chr2	109821904	109824953
chr2	110178422	110179684
chr2	110562611	110569724
chr2	110649233	110661841
chr2	110666262	110669553
chr2	110666262	110674224
chr2	110672660	110674365
chr2	111792361	111803839
chr2	111800796	111803839
chr2	111802427	111803839
chr2	111802427	111805893
chr2	111815369	111825167
chr2	111822421	111825855
chr2	111831285	111838512
chr2	111831285	111850910
chr2	111833219	111858401
chr2	111838437	111850910
chr2	111843411	111850910
chr2	111843411	111856886
chr2	111847137	111850910
chr2	111850775	111858401
chr2	111856613	111878971
chr2	111858305	111873664
chr2	111863674	111868096
chr2	111864805	111878971
chr2	111872629	111878971
chr2	111872629	111880849
chr2	111873307	111880849
chr2	11192147	11195025
chr2	111944959	111947567
chr2	111965190	111982993
chr2	112001200	112003984
chr2	112059976	112067067
chr2	11207725	11208447

chr2	11207725	11235962
chr2	112098594	112112929
chr2	11214356	11217169
chr2	11215320	11217169
chr2	11221197	11224460
chr2	112316469	112325197
chr2	11235701	11249798
chr2	112485916	112494375
chr2	112485916	112501341
chr2	112485916	112520425
chr2	112494142	112503181
chr2	11249660	11287736
chr2	112501205	112503181
chr2	112547011	112547567
chr2	112547011	112552816
chr2	112547011	112559574
chr2	112547011	112564499
chr2	112547082	112568137
chr2	112550865	112552816
chr2	112557909	112559574
chr2	112557909	112573815
chr2	112746419	112760764
chr2	112755976	112760764
chr2	11286538	11287736
chr2	113460672	113482228
chr2	113632914	113635130
chr2	113717752	113755544
chr2	113719153	113735858
chr2	113723103	113735858
chr2	113729400	113735858
chr2	113729400	113743794
chr2	113731583	113750568
chr2	113734585	113750568
chr2	113742691	113750568
chr2	113746276	113755544
chr2	113913171	113917008
chr2	113913171	113942359
chr2	113916883	113927455
chr2	113916883	113931396
chr2	113916883	113940102
chr2	113916883	113942359
chr2	113927344	113931396
chr2	113927344	113934386
chr2	113939958	113942359
chr2	113951478	113955706

chr2	11765532	11767858
chr2	11765532	11773745
chr2	11782200	11783922
chr2	117824568	117826382
chr2	11784885	11792006
chr2	117935494	117941297
chr2	117948104	117952435
chr2	117985968	118008658
chr2	11802906	11805156
chr2	11815087	11820514
chr2	119311621	119339971
chr2	119317465	119340361
chr2	119809836	119862643
chr2	119809836	119920241
chr2	119862535	119900806
chr2	119877322	119885882
chr2	119877322	119915242
chr2	119882096	119915242
chr2	119900717	119934958
chr2	119920068	119926666
chr2	119926597	119934958
chr2	119926597	119945240
chr2	119926597	119952129
chr2	119932423	119934958
chr2	119932423	119946574
chr2	119932423	119965645
chr2	119951972	119962744
chr2	119951972	119967972
chr2	119960806	119967972
chr2	119962615	119967972
chr2	120019076	120042110
chr2	120073177	120078581
chr2	120091554	120100814
chr2	120127687	120146289
chr2	120278617	120289757
chr2	120285873	120289757
chr2	121347037	121348718
chr2	121363171	121367831
chr2	121363171	121398400
chr2	121410865	121457757
chr2	121425138	121469960
chr2	121430072	121457757
chr2	121448952	121457757
chr2	121450912	121458975
chr2	121461100	121527890

chr2	121503166	121525920
chr2	121503166	121527890
chr2	121503166	121530325
chr2	121515664	121530325
chr2	121525844	121530325
chr2	121527798	121530325
chr2	121730892	121732204
chr2	121757239	121758806
chr2	121757239	121761524
chr2	121757239	121763084
chr2	127057472	127059155
chr2	127279172	127293718
chr2	127286702	127293718
chr2	127322045	127323994
chr2	127335869	127339050
chr2	127724332	127726777
chr2	127763061	127771004
chr2	127768188	127771004
chr2	127850589	127853105
chr2	127869453	127871359
chr2	127949868	127955344
chr2	127989563	127993308
chr2	127989563	127996491
chr2	127993186	127996491
chr2	128014802	128017915
chr2	128097428	128104014
chr2	128097428	128109746
chr2	128107937	128116343
chr2	128107937	128129179
chr2	128107937	128145967
chr2	128123185	128129179
chr2	128127360	128134961
chr2	128127360	128145967
chr2	128138716	128145967
chr2	128143093	128145967
chr2	128164729	128171284
chr2	128164729	128173939
chr2	128170287	128171284
chr2	128172572	128174858
chr2	128183674	128187614
chr2	128186682	128187614
chr2	130172348	130176637
chr2	130358866	130359646
chr2	130917396	130946635
chr2	130930951	130946635

chr2	131038852	131043583
chr2	131052890	131055695
chr2	131055271	131055695
chr2	131055271	131082657
chr2	131071854	131082657
chr2	131125752	131130760
chr2	131125752	131132991
chr2	131125752	131140275
chr2	134270385	134318739
chr2	134317528	134318739
chr2	134317528	134349938
chr2	134317528	134362408
chr2	134936840	134947899
chr2	134942611	134947899
chr2	134946100	134947899
chr2	135090997	135093693
chr2	135090997	135113270
chr2	135090997	135115381
chr2	135120818	135126656
chr2	135120818	135135932
chr2	135129994	135130721
chr2	135129994	135135932
chr2	135150368	135153876
chr2	135224425	135228015
chr2	135313488	135315530
chr2	135313488	135345635
chr2	135349983	135353628
chr2	135602499	135605016
chr2	135602499	135616757
chr2	135602499	135675486
chr2	135602499	135680324
chr2	135645378	135680324
chr2	135651729	135675486
chr2	135651729	135680324
chr2	135661269	135680324
chr2	135675331	135680324
chr2	135709432	135710231
chr2	135748266	135755691
chr2	135748266	135761911
chr2	135748266	135780385
chr2	135848052	135852915
chr2	135851401	135870361
chr2	135857896	135859442
chr2	135862606	135865163
chr2	135865012	135872843

chr2	135916225	135924498
chr2	135920452	135924498
chr2	135920452	135933990
chr2	135932782	135943480
chr2	135932782	135961498
chr2	135943377	135961498
chr2	135961395	135983454
chr2	138559021	138564993
chr2	143951981	143970762
chr2	144141881	144211579
chr2	144208602	144211579
chr2	144208602	144235737
chr2	144424795	144430026
chr2	144424795	144517419
chr2	147896300	147899567
chr2	147896300	147899898
chr2	147896363	147899898
chr2	147948050	147975975
chr2	147955346	147975975
chr2	147972738	147975975
chr2	147972738	147982081
chr2	148021647	148233395
chr2	148021647	148342336
chr2	148178699	148233395
chr2	148178699	148342336
chr2	148458202	148470461
chr2	148761781	148771387
chr2	148922136	148937388
chr2	148922136	148942760
chr2	149575710	149582271
chr2	150486693	150487455
chr2	151275483	151278990
chr2	151411259	151420379
chr2	151416560	151420379
chr2	151416560	151428922
chr2	151416806	151420379
chr2	151420189	151435580
chr2	151420189	151437351
chr2	151428783	151446575
chr2	151436826	151443709
chr2	151436826	151446575
chr2	151438683	151443709
chr2	151438683	151446575
chr2	151441904	151443709
chr2	15178987	15330765

chr2	15186741	15309247
chr2	151870530	151883370
chr2	151872416	151883370
chr2	151876425	151883370
chr2	152123765	152150229
chr2	152143826	152148300
chr2	152144887	152148300
chr2	152144887	152150229
chr2	152147161	152150229
chr2	152542738	152549097
chr2	152542738	152561035
chr2	152542738	152578964
chr2	152558739	152561035
chr2	152558739	152581049
chr2	152560882	152578964
chr2	152560882	152581049
chr2	152575135	152581049
chr2	152662602	152669270
chr2	152662602	152693068
chr2	152664137	152679109
chr2	152670230	152671381
chr2	152670230	152679472
chr2	152672458	152693068
chr2	152673597	152693068
chr2	15275483	15277101
chr2	15287072	15309247
chr2	15327749	15330765
chr2	15351991	15356416
chr2	15351991	15383317
chr2	15351991	15396475
chr2	15351991	15478289
chr2	15366579	15402301
chr2	15374607	15402301
chr2	15374607	15417712
chr2	153900935	153944639
chr2	153900935	154140505
chr2	15394226	15402301
chr2	15394226	15417712
chr2	15394226	15427794
chr2	15394226	15478289
chr2	15402167	15427794
chr2	15402167	15478289
chr2	15415545	15417712
chr2	15415545	15427794
chr2	15461200	15478289

chr2	15461200	15489022
chr2	15461200	15558634
chr2	15467328	15474324
chr2	15467328	15478289
chr2	15478225	15511350
chr2	15478225	15534641
chr2	15488893	15511350
chr2	15488893	15534641
chr2	15488893	15539356
chr2	15488893	15558634
chr2	15504144	15511350
chr2	15504144	15534641
chr2	15504144	15539356
chr2	15504144	15558634
chr2	15511211	15558634
chr2	15534542	15539356
chr2	15534542	15558634
chr2	15551492	15558634
chr2	15553425	15558634
chr2	15595144	15602631
chr2	15595144	15604509
chr2	15595489	15599716
chr2	15603191	15607313
chr2	15618180	15629697
chr2	15623435	15629697
chr2	156476097	156513496
chr2	156496043	156513496
chr2	156510795	156513496
chr2	156549607	156557582
chr2	157760879	157770514
chr2	157799426	157818559
chr2	158309432	158345318
chr2	158338725	158340680
chr2	158533179	158577383
chr2	158533179	158603104
chr2	158533179	158621421
chr2	158533179	158642699
chr2	158533179	158663445
chr2	158577270	158642699
chr2	158603069	158631941
chr2	158603069	158634289
chr2	158603069	158642699
chr2	158620989	158631941
chr2	158620989	158634289
chr2	158631752	158640759

chr2	159001079	159136298
chr2	159065895	159097834
chr2	159136193	159150556
chr2	159149141	159150556
chr2	159169249	159170805
chr2	159169249	159172272
chr2	159185790	159199053
chr2	159194256	159199053
chr2	159224231	159225779
chr2	159248371	159256375
chr2	159248371	159259843
chr2	159248371	159279945
chr2	159271701	159283093
chr2	159275545	159283093
chr2	159373044	159374753
chr2	159446781	159478721
chr2	159715680	159729175
chr2	159729008	159743253
chr2	159729008	159752571
chr2	159729008	159762993
chr2	159743060	159745937
chr2	159743060	159748903
chr2	160300650	160318227
chr2	160313155	160367391
chr2	161179613	161204793
chr2	161179613	161224746
chr2	161203486	161224746
chr2	161367477	161371322
chr2	161367477	161395203
chr2	165898685	165901910
chr2	165907677	165912624
chr2	165912513	165930364
chr2	165924548	165945690
chr2	1673642	1684151
chr2	1673642	1693134
chr2	1676926	1693134
chr2	168012633	168065381
chr2	168012633	168075231
chr2	168012633	168129758
chr2	168012633	168161842
chr2	168012633	168182090
chr2	1680192	1693134
chr2	168063499	168075231
chr2	168063499	168129758
chr2	168074981	168129758

chr2	168074981	168182090
chr2	168129540	168140758
chr2	168129540	168167407
chr2	168138087	168140758
chr2	168138087	168167407
chr2	168138087	168182090
chr2	168161786	168167407
chr2	168161786	168182090
chr2	168873584	168877384
chr2	169482250	169488114
chr2	169482250	169493836
chr2	169482250	169499620
chr2	169530586	169544835
chr2	169530586	169572171
chr2	169537226	169557297
chr2	169538012	169544835
chr2	169540050	169557297
chr2	169544720	169557297
chr2	169546217	169557297
chr2	169555123	169557297
chr2	169555123	169563350
chr2	169557186	169563350
chr2	169560386	169569252
chr2	169560386	169572171
chr2	169603641	169606146
chr2	169603641	169607148
chr2	169603641	169608758
chr2	169603641	169614724
chr2	169606038	169608758
chr2	169606038	169614724
chr2	169631765	169633247
chr2	169800951	169805839
chr2	169872235	169878574
chr2	169872235	169906164
chr2	169872235	169942634
chr2	169891164	169896735
chr2	169891164	169906164
chr2	169896506	169906164
chr2	169905113	169933008
chr2	169925618	169933008
chr2	169946287	170015366
chr2	169958437	170029448
chr2	169958437	170040985
chr2	169986644	170040985
chr2	170015280	170055584

chr2	170040881	170061443
chr2	170055459	170061443
chr2	170073427	170080684
chr2	170849285	170854022
chr2	170951327	170956559
chr2	170954649	170956559
chr2	171006146	171014948
chr2	171006146	171028405
chr2	171011372	171028405
chr2	171014850	171028405
chr2	171028338	171046362
chr2	171028338	171050174
chr2	171028338	171061156
chr2	171046173	171050174
chr2	171046173	171082852
chr2	171053760	171061156
chr2	171053760	171082852
chr2	171325840	171330698
chr2	171325840	171331867
chr2	171325840	171339554
chr2	171337452	171339554
chr2	171435082	171453213
chr2	171435082	171476950
chr2	171443522	171453213
chr2	171448680	171453213
chr2	171448680	171458075
chr2	171448680	171458477
chr2	171448680	171469030
chr2	171448680	171473975
chr2	171690146	171715443
chr2	171787570	171809686
chr2	171809605	171815202
chr2	171809605	171855949
chr2	171826797	171855949
chr2	171833962	171855949
chr2	171844368	171868823
chr2	171855833	171868823
chr2	171925536	171953001
chr2	171925536	171976308
chr2	171925536	171979363
chr2	171946707	171953001
chr2	171965337	171966949
chr2	172474054	172480051
chr2	172479640	172498100
chr2	172484781	172485264

chr2	172487263	172498100
chr2	172558707	172562291
chr2	172558707	172562800
chr2	172558707	172570824
chr2	172562219	172565073
chr2	172562219	172566933
chr2	172564502	172565073
chr2	172564502	172570824
chr2	172566855	172596023
chr2	172568740	172593048
chr2	172568740	172596023
chr2	172570725	172596023
chr2	172586277	172596023
chr2	173169804	173191177
chr2	173238372	173258815
chr2	173358711	173359491
chr2	173358711	173363462
chr2	173358711	173365592
chr2	173363225	173365592
chr2	174078967	174082064
chr2	174123179	174142000
chr2	174123594	174142000
chr2	174141824	174229451
chr2	174223032	174229451
chr2	174223032	174246815
chr2	174398274	174404315
chr2	174453736	174460088
chr2	174581309	174585611
chr2	174846879	174878128
chr2	174846879	174944943
chr2	174846879	174952202
chr2	174915057	174944943
chr2	174915057	174952202
chr2	175080659	175097593
chr2	175080659	175111654
chr2	175080659	175114868
chr2	175080659	175118369
chr2	175080659	175121540
chr2	175092542	175121540
chr2	175093060	175118369
chr2	175093060	175121540
chr2	175111567	175114868
chr2	175111567	175121540
chr2	175117989	175136486
chr2	175130137	175151158

chr2	175937343	175939657
chr2	175937343	175947692
chr2	175937343	175964589
chr2	175937343	175993223
chr2	175947479	175979868
chr2	175970763	175994280
chr2	175970763	175995646
chr2	175979809	175994280
chr2	175979809	175995646
chr2	176296859	176326901
chr2	176296859	176328912
chr2	176296859	176329426
chr2	176296859	176330660
chr2	176323391	176328912
chr2	176328873	176330660
chr2	17708638	17715065
chr2	17708638	17745951
chr2	17714860	17745951
chr2	177232391	177234271
chr2	17725256	17745951
chr2	17731077	17745951
chr2	17738220	17745951
chr2	177420268	177441036
chr2	177420268	177462018
chr2	177434326	177445626
chr2	177436763	177442486
chr2	177436763	177462018
chr2	177440964	177462018
chr2	177440964	177468524
chr2	177442406	177462018
chr2	177442406	177468524
chr2	177461892	177468524
chr2	177461892	177482186
chr2	177461892	177493199
chr2	177482058	177513908
chr2	177505505	177513908
chr2	177505505	177523805
chr2	177513818	177523805
chr2	17759928	17768811
chr2	17761395	17768811
chr2	178284926	178328378
chr2	178361681	178374027
chr2	178383023	178385521
chr2	178485640	178490814
chr2	178535731	178537691

chr2	178535731	178542361
chr2	178540106	178542361
chr2	178583523	178600667
chr2	178591138	178600667
chr2	179172119	179191866
chr2	179964546	179971076
chr2	179970500	179971076
chr2	180982017	180984093
chr2	181921947	181928278
chr2	182736248	182743712
chr2	182751657	182762801
chr2	182934751	182942163
chr2	182934751	183023916
chr2	182952404	182953331
chr2	182952404	182957596
chr2	182962158	182967361
chr2	182967215	182989186
chr2	182967215	182995838
chr2	182976892	183023916
chr2	182981243	182982927
chr2	182994838	183003325
chr2	182994838	183023916
chr2	183001952	183003325
chr2	183128286	183130545
chr2	183128286	183131117
chr2	183128286	183133623
chr2	183133565	183159652
chr2	186501272	186502587
chr2	186602020	186622430
chr2	186602020	186625587
chr2	186633328	186641588
chr2	186638276	186652089
chr2	186646685	186649885
chr2	186675603	186675927
chr2	186740227	186754049
chr2	187484123	187503770
chr2	189450902	189455444
chr2	189450902	189469439
chr2	189459335	189466582
chr2	189719571	189720787
chr2	189728659	189744788
chr2	189753915	189754345
chr2	189785345	189818180
chr2	189791789	189795951
chr2	189791789	189805754

chr2	189791789	189818180
chr2	189791789	189852777
chr2	189791789	189855128
chr2	189795768	189818180
chr2	189805651	189818180
chr2	189852654	189855128
chr2	190212955	190252307
chr2	190244886	190252307
chr2	190287585	190296953
chr2	190360038	190362687
chr2	190510360	190514678
chr2	190510360	190525378
chr2	190655976	190673152
chr2	190659157	190670459
chr2	190659157	190673152
chr2	190895151	190905167
chr2	190895151	190910321
chr2	190895151	190921203
chr2	190895151	190924593
chr2	190900563	190905167
chr2	190900563	190910321
chr2	190900563	190921203
chr2	190900563	190923983
chr2	190900563	190924593
chr2	190900563	190927482
chr2	190900563	190931637
chr2	190910262	190927482
chr2	190979771	190983740
chr2	191276886	191296226
chr2	191276886	191330029
chr2	191276886	191346282
chr2	191276886	191350225
chr2	191329934	191364276
chr2	191360630	191364276
chr2	196271192	196308085
chr2	196656632	196677361
chr2	196665566	196666848
chr2	196672344	196677361
chr2	196766564	196779067
chr2	196772915	196779067
chr2	196789203	196791460
chr2	196870940	196873758
chr2	196912881	196920150
chr2	197007805	197009031
chr2	197078702	197090032

chr2	197407997	197410007
chr2	197416740	197423974
chr2	197459931	197462731
chr2	197487857	197490296
chr2	197493323	197495376
chr2	197493323	197498850
chr2	197494156	197495376
chr2	197494156	197497392
chr2	197494156	197498850
chr2	197494656	197495376
chr2	197523623	197540437
chr2	197523623	197548415
chr2	197535529	197540437
chr2	197535529	197550386
chr2	19930395	19982534
chr2	199308759	199328910
chr2	199308759	199368707
chr2	199308759	199381820
chr2	199323802	199328910
chr2	199323802	199368707
chr2	199323802	199381820
chr2	199328697	199381820
chr2	199348700	199381820
chr2	199348700	199433514
chr2	19935470	19951484
chr2	199368604	199381820
chr2	199368604	199433514
chr2	199380363	199381820
chr2	199380363	199433514
chr2	200329430	200419496
chr2	200412310	200419496
chr2	200416378	200419496
chr2	200439121	200440784
chr2	200497752	200507091
chr2	200498890	200507091
chr2	200569662	200575461
chr2	200612608	200627449
chr2	200813207	200817241
chr2	200854615	200856985
chr2	200856681	200856985
chr2	200856681	200858089
chr2	200876918	200887685
chr2	200920221	200937966
chr2	200925835	200933974
chr2	200925835	200937966

chr2	200925835	200959440
chr2	200931338	200933974
chr2	200931338	200942777
chr2	200941247	200958133
chr2	200942684	200958133
chr2	200992886	201009088
chr2	201016990	201024039
chr2	201016990	201045593
chr2	201129728	201133134
chr2	201129728	201136107
chr2	201129728	201140439
chr2	201129728	201149835
chr2	201133028	201140439
chr2	201133028	201149835
chr2	201266460	201274953
chr2	201399376	201407597
chr2	201407402	201420706
chr2	201705138	201715839
chr2	201707868	201710038
chr2	201723042	201733438
chr2	201723042	201738735
chr2	201738669	201744429
chr2	201741673	201757759
chr2	201749711	201757759
chr2	201753145	201761818
chr2	201760880	201768945
chr2	202275112	202282472
chr2	202275112	202284481
chr2	202277949	202282472
chr2	202282350	202284481
chr2	202282350	202287724
chr2	202284344	202287724
chr2	202287659	202291270
chr2	202287659	202295837
chr2	202290322	202291270
chr2	202290322	202292903
chr2	202290322	202295837
chr2	202290322	202297906
chr2	202295673	202297906
chr2	202297378	202297906
chr2	202464808	202467689
chr2	202464808	202520201
chr2	202464808	202552888
chr2	202513718	202519052
chr2	202513718	202532732

chr2	202518821	202532732
chr2	20253821	20256170
chr2	20254862	20256170
chr2	20255215	20263460
chr2	202755537	202759353
chr2	20278582	20283486
chr2	20278582	20327378
chr2	202785917	202819899
chr2	202785917	202829016
chr2	202796889	202829016
chr2	202811745	202819899
chr2	202811745	202821481
chr2	20282946	20327378
chr2	202882710	202883741
chr2	202883608	202896219
chr2	202896064	202897415
chr2	20297552	20318645
chr2	203041764	203057453
chr2	203049813	203057453
chr2	203056426	203084569
chr2	203056426	203099712
chr2	20307977	20318645
chr2	20307977	20327378
chr2	203083218	203084569
chr2	203099628	203127937
chr2	203110149	203126926
chr2	203122253	203126926
chr2	203135676	203138748
chr2	203171927	203180512
chr2	203172728	203188589
chr2	203193794	203197391
chr2	203245845	203246921
chr2	203245845	203266681
chr2	203246754	203252109
chr2	203278572	203289876
chr2	203366876	203395780
chr2	203380207	203391143
chr2	203380207	203402734
chr2	203380207	203417081
chr2	203391045	203402734
chr2	203394699	203411371
chr2	203411284	203417081
chr2	203444867	203455580
chr2	203444867	203461408
chr2	203444867	203461925

chr2	203444867	203477156
chr2	203447958	203448836
chr2	203454429	203457595
chr2	203454429	203461925
chr2	203489583	203495353
chr2	205158721	205193320
chr2	205172210	205185863
chr2	205172210	205193320
chr2	206028110	206046612
chr2	206046503	206056943
chr2	206046503	206063244
chr2	206126538	206133105
chr2	206127796	206130242
chr2	206127796	206142069
chr2	206130087	206142831
chr2	206132944	206142069
chr2	206132944	206142831
chr2	206138484	206142831
chr2	206138484	206152510
chr2	206141940	206145026
chr2	206141940	206149096
chr2	206141940	206149925
chr2	206149019	206152510
chr2	206279539	206281909
chr2	206279539	206297373
chr2	206445306	206481308
chr2	206481231	206550160
chr2	206542051	206550160
chr2	206770090	206774397
chr2	206770090	206790686
chr2	207088457	207124404
chr2	207088457	207165706
chr2	20739970	20774979
chr2	20739970	20801465
chr2	207555627	207570321
chr2	207555627	207577655
chr2	207560225	207577655
chr2	207567462	207577655
chr2	207575271	207577655
chr2	207724799	207742342
chr2	207724799	207751119
chr2	20774809	20790398
chr2	20774809	20801465
chr2	208271510	208277708
chr2	208276711	208285933

chr2	208276711	208288818
chr2	208285725	208288818
chr2	208288728	208302353
chr2	208298640	208302353
chr2	208298640	208304318
chr2	208298640	208305013
chr2	208302241	208330694
chr2	208304170	208305013
chr2	208312235	208317941
chr2	208312235	208330694
chr2	208312235	208348023
chr2	208333314	208348023
chr2	208342553	208348023
chr2	208345110	208348023
chr2	210104103	210154611
chr2	210129030	210154611
chr2	210455106	210472076
chr2	210455106	210476412
chr2	210556718	210582709
chr2	211619176	211630594
chr2	211701966	211704194
chr2	213297261	213350645
chr2	213310058	213317356
chr2	213310058	213350645
chr2	213310058	213364145
chr2	213310058	213375119
chr2	213340162	213364145
chr2	214745066	214752555
chr2	214745066	214769312
chr2	214745066	214781509
chr2	214745066	214797117
chr2	214752446	214769312
chr2	214752446	214781509
chr2	214752446	214797117
chr2	214767481	214781509
chr2	214767481	214792445
chr2	214767481	214797117
chr2	214780559	214792445
chr2	214780559	214797117
chr2	214792296	214797117
chr2	215312497	215319731
chr2	215312497	215336124
chr2	215319664	215326138
chr2	215319664	215346941
chr2	215325240	215333457

chr2	215325240	215338907
chr2	215325986	215338907
chr2	215332381	215336124
chr2	215332381	215338907
chr2	215336034	215346941
chr2	215344778	215349249
chr2	216113015	216117794
chr2	216113015	216132387
chr2	216113015	216148276
chr2	216113015	216162048
chr2	216125916	216137225
chr2	216141185	216148276
chr2	216190224	216194986
chr2	216277671	216283729
chr2	216420298	216435496
chr2	216464596	216478299
chr2	216467943	216475451
chr2	216499269	216500031
chr2	218217462	218228850
chr2	218217462	218239484
chr2	218228737	218239484
chr2	218234351	218239484
chr2	218238663	218239484
chr2	218245419	218249921
chr2	218339782	218342144
chr2	218457091	218463366
chr2	218463305	218466176
chr2	218466009	218474885
chr2	218466009	218479715
chr2	218474629	218485743
chr2	218474629	218562813
chr2	218476839	218482234
chr2	218488303	218495950
chr2	218495759	218498157
chr2	218498025	218510140
chr2	218509978	218530040
chr2	218509978	218534706
chr2	218509978	218562813
chr2	218529955	218546298
chr2	218546220	218547091
chr2	218546220	218562813
chr2	218546918	218560883
chr2	218546918	218562813
chr2	218549808	218560883
chr2	218553552	218562813

chr2	218558497	218562813
chr2	218580560	218583086
chr2	218580560	218584721
chr2	218582970	218584721
chr2	218582970	218587695
chr2	218656149	218658801
chr2	218660938	218663000
chr2	218661758	218662260
chr2	218727268	218740584
chr2	218737578	218740584
chr2	219444652	219451279
chr2	219447973	219464608
chr2	219451135	219464608
chr2	219473503	219473903
chr2	219566829	219568324
chr2	221563730	221568785
chr2	222599927	222619737
chr2	222599927	222624479
chr2	222599927	222624775
chr2	222599927	222643005
chr2	222613810	222624479
chr2	222613810	222624775
chr2	222613810	222634541
chr2	222613810	222643005
chr2	222619644	222624775
chr2	222619644	222634541
chr2	222623649	222624479
chr2	222623649	222643005
chr2	222628836	222643005
chr2	222639579	222643005
chr2	222639579	222648795
chr2	222640861	222643005
chr2	222900673	222909150
chr2	222908732	222919202
chr2	222916318	222921430
chr2	222918045	222924595
chr2	222921279	222934687
chr2	222927016	222934687
chr2	222930620	222934687
chr2	223765212	223777869
chr2	223775900	223777869
chr2	223880123	223882072
chr2	223880123	223884749
chr2	223881941	223884749
chr2	223884647	223895630

chr2	223894233	223895630
chr2	223998114	224001922
chr2	224495831	224503072
chr2	224495831	224503822
chr2	224495831	224514772
chr2	224495831	224535641
chr2	224505955	224557856
chr2	224535527	224557856
chr2	226838072	226914351
chr2	226839408	226914351
chr2	226864603	226867318
chr2	226864603	226908878
chr2	226864603	226914351
chr2	226906792	226914351
chr2	226908821	226914351
chr2	227328677	227329783
chr2	227328677	227340380
chr2	227330625	227332588
chr2	227491546	227524915
chr2	227519947	227524915
chr2	227519947	227536993
chr2	227523762	227524915
chr2	227531090	227535025
chr2	227531090	227536993
chr2	227531090	227552117
chr2	227885350	227893917
chr2	227893794	227898281
chr2	227893794	227907252
chr2	227898181	227907252
chr2	227903001	227905035
chr2	227918779	227921561
chr2	229155817	229262893
chr2	229447315	229477253
chr2	229447315	229591888
chr2	229477139	229512936
chr2	229477139	229547092
chr2	229512782	229547092
chr2	229585857	229591888
chr2	229588393	229591888
chr2	229768615	229771632
chr2	229774096	229777479
chr2	229802251	229804227
chr2	229802251	229807864
chr2	229802251	229811045
chr2	229805729	229818512

chr2	229805729	229830839
chr2	229807707	229811045
chr2	229813869	229818512
chr2	229829192	229836984
chr2	229836847	229840927
chr2	229836847	229860531
chr2	229840821	229860531
chr2	229858771	229860531
chr2	229858771	229880128
chr2	229965	253115
chr2	229976240	229996923
chr2	230357804	230361697
chr2	230357804	230383575
chr2	230440475	230450255
chr2	230442936	230449710
chr2	230442936	230450255
chr2	230461261	230474447
chr2	230466300	230474447
chr2	230759958	230793331
chr2	230759958	230814114
chr2	230790871	230793331
chr2	230790871	230798897
chr2	230798728	230817897
chr2	231066905	231075571
chr2	231066905	231087181
chr2	231075510	231087181
chr2	231138735	231153666
chr2	231146239	231166017
chr2	231206197	231216793
chr2	231206197	231235381
chr2	231206197	231240041
chr2	231214830	231216793
chr2	231235223	231240041
chr2	231272954	231291443
chr2	231272954	231296253
chr2	232014834	232015671
chr2	232014834	232024330
chr2	232014834	232030080
chr2	232014834	232087721
chr2	232130618	232163632
chr2	232136471	232163632
chr2	232136471	232210405
chr2	232210325	232263440
chr2	232329812	232334735
chr2	232329885	232335874

chr2	232556415	232567214
chr2	232564246	232567214
chr2	232703423	232724691
chr2	232703423	232761436
chr2	232735154	232756334
chr2	232735154	232761436
chr2	232747614	232756334
chr2	232747614	232761436
chr2	232748986	232761436
chr2	232787149	232794944
chr2	232787149	232796221
chr2	232790697	232791170
chr2	232811243	232812491
chr2	232832856	232839971
chr2	233256101	233265143
chr2	233256101	233270067
chr2	233256101	233282753
chr2	233388256	233390483
chr2	233388256	233437479
chr2	233434379	233436441
chr2	233434379	233437479
chr2	233434379	233438379
chr2	233435817	233441995
chr2	233438216	233441995
chr2	233449981	233452060
chr2	233450921	233452060
chr2	233485530	233488304
chr2	233549100	233551519
chr2	23475559	23562481
chr2	235034870	235043247
chr2	235744697	235750488
chr2	235744697	235807331
chr2	235750353	235799522
chr2	235750353	235807331
chr2	235750353	235883449
chr2	235797758	235807331
chr2	235797758	235908906
chr2	235883344	235908906
chr2	235883344	235930923
chr2	235883344	235968623
chr2	235908737	235930923
chr2	235930764	235968623
chr2	235968461	236049281
chr2	236036560	236049281
chr2	23762208	23788647

chr2	237708543	237720822
chr2	237748363	237760205
chr2	23782868	23823569
chr2	23788511	23834078
chr2	237973091	237973747
chr2	237973091	237994809
chr2	237973091	238035940
chr2	23798137	23823569
chr2	23798137	23834078
chr2	238025341	238032254
chr2	238025341	238035940
chr2	238083247	238096876
chr2	23810315	23823569
chr2	23816636	23819882
chr2	23816636	23834078
chr2	238173533	238185287
chr2	238173533	238188257
chr2	238173533	238194870
chr2	238182064	238185287
chr2	238182064	238194870
chr2	238185180	238189972
chr2	238185180	238194870
chr2	238188130	238189972
chr2	238188130	238194870
chr2	23819746	23823569
chr2	23819746	23863555
chr2	23823257	23828939
chr2	238325305	238329342
chr2	238332823	238338457
chr2	238332823	238356080
chr2	238347454	238356080
chr2	238433553	238444727
chr2	238435710	238444727
chr2	23863380	23875904
chr2	23867834	23875904
chr2	23880638	23885829
chr2	23880638	23888399
chr2	23887831	23895970
chr2	239081094	239090116
chr2	239126455	239156773
chr2	239126455	239176563
chr2	239134244	239134643
chr2	239134244	239163923
chr2	239139683	239163923
chr2	239144582	239236664

chr2	239156651	239163923
chr2	239156651	239176563
chr2	239156651	239352918
chr2	239176412	239236664
chr2	239176412	239352918
chr2	239189832	239352918
chr2	239236592	239352918
chr2	23958300	23977075
chr2	23958300	23983033
chr2	23958300	23984831
chr2	23971275	23984831
chr2	23982895	23984831
chr2	239990073	240007370
chr2	239990073	240011696
chr2	239990073	240014860
chr2	239990073	240021412
chr2	239990073	240022340
chr2	240005209	240014860
chr2	240005209	240022340
chr2	240011616	240022340
chr2	240499957	240525849
chr2	240499957	240555055
chr2	240523884	240525849
chr2	240552913	240555055
chr2	24068320	24074194
chr2	241122731	241143074
chr2	241132873	241143074
chr2	241140520	241143074
chr2	241160331	241169867
chr2	241236614	241238787
chr2	241238648	241249980
chr2	241246751	241247142
chr2	241246751	241268541
chr2	241262710	241268541
chr2	241324215	241337790
chr2	241324215	241348191
chr2	241325992	241343093
chr2	241335125	241346249
chr2	241342991	241343897
chr2	241342991	241346249
chr2	241342991	241348191
chr2	241343751	241348191
chr2	24135118	24139450
chr2	24135118	24147086
chr2	24137908	24147086

chr2	241403827	241413421
chr2	241403827	241418109
chr2	241403827	241434321
chr2	241407536	241413421
chr2	241407536	241418109
chr2	241407536	241431774
chr2	241407536	241434321
chr2	241407536	241436538
chr2	241411032	241418109
chr2	241411032	241431774
chr2	241456746	241475987
chr2	241462522	241475987
chr2	241601895	241603079
chr2	241601895	241606473
chr2	241651009	241659187
chr2	241654545	241655343
chr2	241654545	241668685
chr2	241668142	241668685
chr2	242114638	242122455
chr2	242114638	242138888
chr2	24293687	24295804
chr2	24308616	24315224
chr2	24312211	24315224
chr2	24643965	24665915
chr2	24673365	24697798
chr2	24682950	24697798
chr2	24691480	24697798
chr2	24726588	24752156
chr2	247537	253115
chr2	24793851	24799844
chr2	24799674	24814493
chr2	25235706	25237005
chr2	25274940	25275543
chr2	25274940	25314161
chr2	25427534	25433995
chr2	25531472	25607321
chr2	25531472	25628384
chr2	25576837	25596240
chr2	25576837	25607321
chr2	25576837	25628384
chr2	25607235	25628384
chr2	25607235	25652661
chr2	25628170	25639094
chr2	25628170	25652661
chr2	25749695	25756114

chr2	25749695	25771540
chr2	25767582	25771540
chr2	25799384	25845563
chr2	26098661	26127951
chr2	26127143	26127951
chr2	26209779	26215175
chr2	26209779	26234355
chr2	26230191	26234355
chr2	26230191	26236988
chr2	26230191	26239143
chr2	26232159	26239143
chr2	26263379	26277160
chr2	26263379	26279315
chr2	26269952	26277160
chr2	26278613	26280115
chr2	26278613	26284957
chr2	26279134	26283051
chr2	26279993	26284204
chr2	26364301	26367220
chr2	26364301	26373629
chr2	26364301	26375148
chr2	26364301	26383347
chr2	26373366	26375148
chr2	26373366	26383347
chr2	26792130	26792833
chr2	26924886	26928323
chr2	26924886	26931684
chr2	26940030	26944824
chr2	27057302	27059404
chr2	27058399	27059404
chr2	27204460	27211533
chr2	27206876	27211533
chr2	27213176	27216620
chr2	27221217	27226962
chr2	27236283	27236830
chr2	27237378	27239798
chr2	27337243	27343578
chr2	27366758	27367201
chr2	27382125	27387158
chr2	27462700	27465882
chr2	27470927	27472362
chr2	27483288	27484266
chr2	27485017	27485503
chr2	27598066	27608039
chr2	27599582	27601442

chr2	27599582	27603307
chr2	27599582	27608039
chr2	27615167	27617571
chr2	27638209	27641279
chr2	27660983	27662281
chr2	27668842	27675692
chr2	27668842	27677924
chr2	27675531	27677924
chr2	27677294	27677924
chr2	27677294	27682359
chr2	27987992	28025420
chr2	27987992	28129380
chr2	28025225	28129380
chr2	28237201	28241393
chr2	28237201	28244862
chr2	28776850	28788809
chr2	28783906	28788809
chr2	28783906	28793997
chr2	28815996	28822410
chr2	28819047	28829319
chr2	28829147	28840469
chr2	28850327	28861308
chr2	28850327	28865119
chr2	28861117	28865119
chr2	28901986	28906581
chr2	28901986	28914208
chr2	28906459	28914208
chr2	28906459	28917995
chr2	28906459	28937994
chr2	28906459	28941574
chr2	28912589	28914208
chr2	28912589	28917995
chr2	28917892	28929710
chr2	28922918	28927700
chr2	28926467	28929710
chr2	28926467	28935607
chr2	28935520	28942381
chr2	28936921	28942381
chr2	28937930	28942381
chr2	29121373	29133816
chr2	29121373	29135666
chr2	29121373	29145367
chr2	29121373	29167540
chr2	29121373	29174445
chr2	29131257	29135666

chr2	29131257	29143945
chr2	29133654	29143945
chr2	29133654	29145367
chr2	29133654	29157347
chr2	29133654	29157402
chr2	29133654	29160467
chr2	29133654	29167540
chr2	30459551	30476635
chr2	30459551	30533314
chr2	30459551	30568176
chr2	30525586	30533314
chr2	30525586	30568176
chr2	30552492	30568176
chr2	30562145	30568176
chr2	30942200	30945870
chr2	30942200	30955977
chr2	30955617	30966302
chr2	30955617	30993007
chr2	30992837	31125253
chr2	31383000	31383847
chr2	31883385	31920910
chr2	31883385	31932135
chr2	31883385	31943383
chr2	31891991	31920910
chr2	31891991	31932135
chr2	31891991	31943383
chr2	31917925	31920910
chr2	31917925	31932135
chr2	31917925	31943383
chr2	32098795	32116212
chr2	32098795	32128479
chr2	32114637	32116212
chr2	32115701	32116212
chr2	32128407	32141946
chr2	3214148	3257455
chr2	32171286	32184338
chr2	32174062	32184338
chr2	32174062	32192953
chr2	32192335	32197826
chr2	32192335	32206933
chr2	32193888	32206933
chr2	32377587	32380290
chr2	32377587	32392150
chr2	32377587	32395593
chr2	32377587	32401623

chr2	32377587	32406557
chr2	32377587	32416163
chr2	32388749	32392150
chr2	32388749	32395593
chr2	32388749	32416163
chr2	32433643	32436184
chr2	32436052	32453942
chr2	32439507	32443588
chr2	32439507	32453942
chr2	32439507	32468111
chr2	32445520	32453942
chr2	32464508	32485759
chr2	32473111	32477583
chr2	32476212	32477583
chr2	32478634	32479617
chr2	32478634	32482582
chr2	32478634	32493667
chr2	32482428	32500109
chr2	32488587	32493667
chr2	32488587	32500109
chr2	32491424	32493667
chr2	32491424	32500109
chr2	32491424	32503236
chr2	32491424	32508259
chr2	32499546	32513154
chr2	32501712	32505205
chr2	32501712	32513154
chr2	32509737	32518946
chr2	32510525	32513154
chr2	32514989	32518946
chr2	32518253	32518946
chr2	32524887	32549481
chr2	32524887	32575366
chr2	32529650	32575366
chr2	32543240	32549481
chr2	32543240	32575366
chr2	32575155	32594060
chr2	32593914	32607643
chr2	32595033	32597968
chr2	32597750	32603083
chr2	32597750	32611582
chr2	32607454	32611582
chr2	32630522	32634005
chr2	32630522	32678922
chr2	32633875	32640410

chr2	32633875	32666768
chr2	32640269	32678922
chr2	32664302	32666768
chr2	32702806	32758519
chr2	32736693	32758519
chr2	32758291	32787149
chr2	32777881	32787149
chr2	3387619	3401893
chr2	3387619	3424663
chr2	3401776	3424663
chr2	3421880	3424663
chr2	3421880	3443891
chr2	3547930	3552308
chr2	36396613	36442735
chr2	36396613	36464655
chr2	36396613	36479694
chr2	36396613	36510139
chr2	36396613	36522313
chr2	36396613	36544498
chr2	36441257	36442735
chr2	36441257	36464655
chr2	36441257	36479694
chr2	36464533	36479694
chr2	36464533	36513765
chr2	36476888	36479694
chr2	36476888	36522313
chr2	36476888	36537546
chr2	36499218	36517542
chr2	36499218	36522313
chr2	36509982	36522313
chr2	36517326	36522313
chr2	36555682	36591011
chr2	36578596	36583469
chr2	36578596	36591011
chr2	36581289	36591011
chr2	36877890	36916151
chr2	36883931	36886826
chr2	36988645	36990799
chr2	36988645	37020836
chr2	37003541	37007304
chr2	37008610	37020836
chr2	37013840	37020836
chr2	37013840	37028920
chr2	37019808	37020836
chr2	37032628	37041292

chr2	37037854	37041292
chr2	37049652	37060733
chr2	37053501	37058560
chr2	37056439	37057480
chr2	37068680	37077019
chr2	37119958	37122664
chr2	37139630	37141701
chr2	37199703	37201726
chr2	37213863	37217037
chr2	37222379	37229036
chr2	37236095	37237867
chr2	37259582	37290999
chr2	37267429	37293271
chr2	37282541	37290999
chr2	37282541	37293271
chr2	37289355	37290999
chr2	37289355	37293271
chr2	37929261	37975314
chr2	37974039	38004216
chr2	37989540	37997514
chr2	38309378	38319019
chr2	38315283	38319019
chr2	38568198	38591648
chr2	38585643	38591648
chr2	38746143	38750194
chr2	38802714	38806693
chr2	38837830	38843210
chr2	38843004	38855252
chr2	38880178	38881578
chr2	39006411	39010703
chr2	39051143	39058804
chr2	39056701	39058804
chr2	39258347	39267247
chr2	39272282	39288280
chr2	39272282	39315388
chr2	39278406	39286964
chr2	39278406	39288280
chr2	39278406	39290334
chr2	39282512	39308005
chr2	39286851	39288280
chr2	39286851	39290334
chr2	39286851	39292826
chr2	39290291	39308005
chr2	39292772	39309519
chr2	39299742	39333574

chr2	39307942	39315388
chr2	39331916	39337581
chr2	39331916	39356339
chr2	39331916	39378123
chr2	39336919	39356339
chr2	39336919	39378123
chr2	39343387	39378123
chr2	40139400	40164984
chr2	40139400	40177855
chr2	40160764	40164984
chr2	40160764	40177855
chr2	40160764	40178493
chr2	42256500	42264731
chr2	42261120	42264731
chr2	42280849	42304551
chr2	42284633	42295259
chr2	42286268	42304551
chr2	42295124	42326252
chr2	42301240	42304551
chr2	42570436	42609584
chr2	42570436	42644244
chr2	42609457	42644244
chr2	42640172	42656302
chr2	42640172	42659862
chr2	42644126	42659862
chr2	42682400	42695839
chr2	42682400	42697834
chr2	42695764	42723035
chr2	42718987	42723035
chr2	43291695	43293213
chr2	43320445	43344237
chr2	43320445	43398139
chr2	43320445	43430302
chr2	43397970	43428231
chr2	43397970	43498955
chr2	43397970	43527988
chr2	43428099	43430302
chr2	43428099	43485325
chr2	43428099	43527988
chr2	43485233	43508780
chr2	43485233	43527988
chr2	43498832	43508780
chr2	43498832	43527988
chr2	43498832	43541316
chr2	43527878	43549368

chr2	43527878	43592416
chr2	43549209	43570510
chr2	43552203	43570510
chr2	43578512	43590954
chr2	43581740	43592416
chr2	43586400	43592416
chr2	43590823	43592416
chr2	43712224	43726451
chr2	43720668	43731602
chr2	43742740	43745963
chr2	43776781	43789721
chr2	43789632	43794643
chr2	43794456	43801709
chr2	43794456	43805246
chr2	43894544	43905780
chr2	43894544	43925961
chr2	43899218	43918133
chr2	43899218	43918398
chr2	43905691	43918398
chr2	43905691	43925961
chr2	43905691	43934878
chr2	43912431	43918398
chr2	43912431	43925961
chr2	43912431	43934296
chr2	43912431	43934878
chr2	43918024	43918398
chr2	43918024	43925961
chr2	43918024	43948518
chr2	43918255	43945417
chr2	43918255	43948518
chr2	43925066	43934878
chr2	43925066	43948518
chr2	43925892	43947370
chr2	43934189	43973900
chr2	43934189	43982434
chr2	43943686	43948199
chr2	43943686	43957451
chr2	43943686	43982434
chr2	43960540	43963706
chr2	43960540	43975217
chr2	43960540	43982434
chr2	43963587	43975217
chr2	43963587	43982434
chr2	43973606	43975217
chr2	43973606	43982434

chr2	43975090	43982434
chr2	43977154	43982434
chr2	44201185	44218537
chr2	44209209	44218537
chr2	44338350	44346390
chr2	44704282	44715353
chr2	44704282	44743696
chr2	45413113	45488331
chr2	45476992	45488331
chr2	45476992	45553730
chr2	45476992	45562756
chr2	45488239	45553730
chr2	45488239	45562756
chr2	45488239	45585774
chr2	45546731	45551282
chr2	45546731	45553730
chr2	45546731	45562756
chr2	45546731	45574723
chr2	45546731	45605441
chr2	45573206	45585774
chr2	45599448	45605441
chr2	45601902	45605441
chr2	45976428	45980381
chr2	45976428	45984680
chr2	46360637	46361090
chr2	46576086	46576656
chr2	46592474	46615185
chr2	46612227	46615185
chr2	46907809	46909177
chr2	46950362	46957007
chr2	46950362	46978907
chr2	46956838	46978907
chr2	46974972	46978907
chr2	46974972	47006724
chr2	46974972	47011435
chr2	47005921	47011435
chr2	47151257	47153016
chr2	47161722	47172459
chr2	47162285	47170764
chr2	47408400	47445657
chr2	47463030	47471062
chr2	47790926	47796063
chr2	47818778	47819078
chr2	47822217	47823360
chr2	47834578	47834871

chr2	47834578	47839769
chr2	47838858	47839769
chr2	48328560	48346751
chr2	48328560	48359147
chr2	48328560	48373360
chr2	48346200	48362707
chr2	48451007	48465642
chr2	48461137	48465642
chr2	48474682	48480016
chr2	48474682	48486758
chr2	48474682	48491170
chr2	48479923	48491170
chr2	48495678	48511468
chr2	48498492	48510113
chr2	48498492	48511468
chr2	48507268	48511468
chr2	53693883	53716743
chr2	53693883	53729570
chr2	53693883	53750941
chr2	53700270	53714581
chr2	53714383	53716743
chr2	53714383	53729570
chr2	53728711	53750941
chr2	53728711	53765585
chr2	53750782	53765585
chr2	53797514	53801842
chr2	53808298	53814935
chr2	53866084	53887467
chr2	53874338	53885775
chr2	53875626	53888812
chr2	53885689	53887989
chr2	53887258	53893799
chr2	53887857	53906868
chr2	53892807	53893799
chr2	53895006	53901559
chr2	53899880	53901559
chr2	53919150	53921104
chr2	53919150	53926023
chr2	53919150	53927483
chr2	53919150	53934727
chr2	53922516	53927483
chr2	53936086	53937540
chr2	53948420	53949283
chr2	54612160	54618193
chr2	54813231	54820462

chr2	54813231	54829477
chr2	54813231	54853855
chr2	54816786	54829477
chr2	54820393	54829477
chr2	54827565	54871605
chr2	54928312	54928751
chr2	54928312	54950779
chr2	54982514	54987698
chr2	55240010	55244233
chr2	55246336	55249534
chr2	55246336	55263865
chr2	55249394	55262427
chr2	55262315	55268739
chr2	55263639	55267635
chr2	55308808	55309254
chr2	55317205	55319004
chr2	55322527	55328432
chr2	55355578	55364033
chr2	55362307	55374883
chr2	55528877	55535763
chr2	55533872	55537585
chr2	55558774	55573777
chr2	55558774	55588956
chr2	55564312	55581698
chr2	55573618	55588956
chr2	55577314	55588956
chr2	55581566	55588956
chr2	55585050	55588956
chr2	55598415	55604076
chr2	55598415	55615506
chr2	55640626	55643425
chr2	55640626	55644720
chr2	55640626	55645432
chr2	55643157	55644720
chr2	55643157	55645432
chr2	55644636	55646486
chr2	55644636	55667958
chr2	55647346	55656371
chr2	55647346	55671376
chr2	55647346	55685048
chr2	55654899	55662026
chr2	55660156	55667093
chr2	55661955	55680918
chr2	55661955	55685048
chr2	55666990	55667958

chr2	55666990	55672046
chr2	55667861	55673079
chr2	55671318	55680918
chr2	55679681	55685048
chr2	55679681	55686444
chr2	55683784	55687705
chr2	55870719	55881734
chr2	55874945	55918267
chr2	55917664	55922447
chr2	58048826	58089723
chr2	58058351	58089723
chr2	58084088	58089723
chr2	58198578	58222042
chr2	58198578	58232112
chr2	58198593	58229874
chr2	58198593	58232112
chr2	58221941	58232112
chr2	60781884	60783209
chr2	60782685	60787620
chr2	60786946	60787620
chr2	60786946	60792289
chr2	60786946	60793715
chr2	60891682	60901083
chr2	61068848	61070500
chr2	61068848	61071792
chr2	61076919	61088468
chr2	61081451	61088468
chr2	61092463	61106662
chr2	61092463	61134276
chr2	61103852	61106662
chr2	61108316	61118116
chr2	61115978	61118116
chr2	61162841	61163232
chr2	61203139	61211929
chr2	61203139	61214694
chr2	61204496	61214694
chr2	61206016	61211929
chr2	61211771	61214694
chr2	61220309	61222663
chr2	61220309	61223296
chr2	61223059	61227218
chr2	61228644	61232532
chr2	61229547	61232532
chr2	61232451	61283449
chr2	61235844	61246477

chr2	61241559	61293550
chr2	61245209	61266167
chr2	61245209	61278443
chr2	61248510	61266167
chr2	61256383	61257350
chr2	61259710	61266167
chr2	61259710	61281242
chr2	61259710	61293550
chr2	61265396	61266167
chr2	61278164	61281242
chr2	61278164	61283449
chr2	61278164	61293550
chr2	61278164	61295290
chr2	61280243	61281242
chr2	61280243	61284957
chr2	61280243	61296925
chr2	61284874	61293550
chr2	61284874	61295290
chr2	61284874	61296925
chr2	61296799	61333971
chr2	61300950	61301454
chr2	61300950	61333971
chr2	61311539	61370579
chr2	61325374	61333971
chr2	61325374	61339681
chr2	61325374	61350693
chr2	61343814	61350693
chr2	61343814	61370579
chr2	61347869	61350693
chr2	61350259	61350693
chr2	61378362	61395233
chr2	61394852	61406128
chr2	61481184	61484105
chr2	61481184	61485962
chr2	61482379	61490776
chr2	61482956	61490776
chr2	61483936	61490776
chr2	61485767	61490776
chr2	61485767	61494091
chr2	61490641	61493053
chr2	61490641	61494091
chr2	61490641	61498913
chr2	61490641	61502310
chr2	61492034	61497007
chr2	61493893	61495613

chr2	61493893	61497007
chr2	61493893	61498913
chr2	61493893	61502310
chr2	61493893	61526521
chr2	61495454	61498913
chr2	61495454	61499894
chr2	61496878	61526521
chr2	61498672	61499894
chr2	61498672	61502310
chr2	61498672	61526521
chr2	61498864	61533903
chr2	61499712	61502310
chr2	61522610	61526521
chr2	61522610	61533903
chr2	61872081	61872588
chr2	61872081	61876234
chr2	61872081	61879011
chr2	61872081	61885072
chr2	61872457	61883548
chr2	61873001	61880394
chr2	61873001	61883548
chr2	61876094	61879011
chr2	61876094	61880394
chr2	61876094	61883548
chr2	62747394	62771392
chr2	62831018	62859291
chr2	62943801	62949162
chr2	62955516	62990840
chr2	62979187	62996766
chr2	62987930	62996766
chr2	63152913	63174832
chr2	63259306	63439871
chr2	63313247	63382094
chr2	63313247	63439871
chr2	63404047	63439871
chr2	63433744	63439871
chr2	63484603	63487494
chr2	63484603	63492940
chr2	63597398	63599292
chr2	63604695	63606028
chr2	63856305	63857936
chr2	63856305	63884093
chr2	63856305	63886538
chr2	63882465	63884093
chr2	63882465	63887644

chr2	63912344	63916963
chr2	63913222	63916963
chr2	63933672	63944655
chr2	63933672	63984019
chr2	63949036	63965966
chr2	63962057	63965966
chr2	63962057	63984019
chr2	63968956	63981887
chr2	63972165	63984019
chr2	63981645	63984019
chr2	64551442	64573068
chr2	64551442	64585545
chr2	64572945	64585545
chr2	65001447	65010763
chr2	65003952	65010763
chr2	65090982	65098066
chr2	65090982	65104806
chr2	65239851	65255694
chr2	65239851	65265175
chr2	65246523	65251099
chr2	65246523	65255694
chr2	65246523	65265175
chr2	65251026	65265175
chr2	65261246	65265175
chr2	65331986	65334773
chr2	66464108	66512294
chr2	68134701	68147567
chr2	68138741	68147567
chr2	68141693	68147567
chr2	68161682	68162663
chr2	68186467	68188690
chr2	68490189	68502903
chr2	68490189	68513680
chr2	68490189	68526242
chr2	68490189	68545312
chr2	68490189	68567387
chr2	68502730	68526242
chr2	68513079	68545312
chr2	68526060	68545312
chr2	69070646	69077488
chr2	69328270	69329798
chr2	69328270	69345999
chr2	69328270	69374113
chr2	69337897	69338565
chr2	69337897	69342249

chr2	69337897	69345999
chr2	69338444	69356557
chr2	69345903	69374113
chr2	69348170	69363670
chr2	69350077	69374113
chr2	69354488	69363670
chr2	69354488	69374113
chr2	69356495	69363670
chr2	69358328	69363670
chr2	69363544	69374113
chr2	6940947	7014772
chr2	69415184	69432005
chr2	69505568	69507578
chr2	69505568	69509460
chr2	69505568	69643274
chr2	69530007	69532162
chr2	69806384	69810673
chr2	69806384	69812709
chr2	69816100	69819338
chr2	69837546	69871832
chr2	69844130	69864975
chr2	69847542	69861347
chr2	69847542	69864975
chr2	69864899	69871832
chr2	69988741	69996888
chr2	70032132	70085624
chr2	70051202	70085624
chr2	70083537	70085624
chr2	7014453	7020680
chr2	7014453	7030215
chr2	70160624	70181997
chr2	70165091	70181997
chr2	70175672	70181997
chr2	70179531	70181997
chr2	70214348	70230854
chr2	70215370	70236175
chr2	70224553	70230854
chr2	70224553	70236175
chr2	70227734	70236175
chr2	70229263	70236175
chr2	70259359	70261386
chr2	71348752	71363191
chr2	71355718	71370005
chr2	71355718	71380565
chr2	71363152	71368528

chr2	71363152	71370005
chr2	71363152	71380565
chr2	71365428	71370005
chr2	71365428	71380565
chr2	71368381	71370005
chr2	71368381	71396191
chr2	71396140	71400180
chr2	71396140	71400518
chr2	71396140	71406262
chr2	71396140	71418639
chr2	71401955	71418639
chr2	72134760	72135419
chr2	72334946	72379870
chr2	72465159	72480750
chr2	72559452	72575668
chr2	72559452	72733118
chr2	72718102	72731245
chr2	72718102	72733118
chr2	72718102	72741469
chr2	72731006	72733118
chr2	72731006	72741469
chr2	72731154	72741469
chr2	72988257	73001578
chr2	73020238	73040931
chr2	73088049	73089315
chr2	73239642	73244715
chr2	73408621	73432291
chr2	73419122	73426553
chr2	73419122	73432291
chr2	73432197	73455295
chr2	73534823	73573424
chr2	73550266	73559142
chr2	73550266	73573424
chr2	73557219	73559142
chr2	73730559	73732285
chr2	73938909	73946906
chr2	73946718	73950732
chr2	73950584	73957240
chr2	74003109	74048411
chr2	74046277	74080591
chr2	74073548	74080591
chr2	74159090	74172752
chr2	74165217	74172752
chr2	74167013	74172752
chr2	74205704	74208721

chr2	74207703	74208721
chr2	74207703	74211291
chr2	74207703	74211866
chr2	74304488	74315025
chr2	74377431	74378245
chr2	74481222	74481538
chr2	74506731	74507147
chr2	74527470	74528006
chr2	75518055	75522487
chr2	75665928	75672016
chr2	75670137	75672016
chr2	75694240	75706651
chr2	84425414	84449752
chr2	84431507	84449752
chr2	84441046	84449752
chr2	84832054	84842007
chr2	84839148	84842007
chr2	85027888	85035155
chr2	85027888	85046278
chr2	85027888	85049648
chr2	85043563	85046278
chr2	85043563	85049648
chr2	85345974	85350241
chr2	85349383	85351021
chr2	85368685	85371209
chr2	85368685	85371562
chr2	85541082	85542373
chr2	85541082	85543034
chr2	85541632	85543034
chr2	85541828	85543034
chr2	85542564	85543034
chr2	85555483	85556260
chr2	85555483	85559075
chr2	85619219	85625691
chr2	85619219	85636130
chr2	85623645	85625691
chr2	85637368	85641118
chr2	85637368	85645083
chr2	85644947	85648853
chr2	85647929	85648853
chr2	85861180	85863485
chr2	86052816	86070272
chr2	86065273	86081706
chr2	86075029	86081706
chr2	86098610	86100172

chr2	86108349	86119044
chr2	86116529	86119044
chr2	86121478	86125515
chr2	86121478	86125880
chr2	86121478	86127991
chr2	86123700	86125880
chr2	86127160	86131106
chr2	86132317	86134987
chr2	86133345	86134987
chr2	86151296	86166644
chr2	86158591	86173761
chr2	86159535	86162079
chr2	86159535	86173761
chr2	86166507	86173761
chr2	86206105	86207327
chr2	86251956	86264041
chr2	86251956	86282242
chr2	86442017	86457071
chr2	86442017	86470408
chr2	86474775	86480362
chr2	86507478	86510479
chr2	86740926	86753558
chr2	86740926	86766024
chr2	86751895	86753558
chr2	86765026	86766024
chr2	86770022	86771712
chr2	8733443	8779814
chr2	8747886	8751644
chr2	8770669	8776892
chr2	8798201	8803127
chr2	8864169	8958642
chr2	88729277	88738076
chr2	88735668	88738076
chr2	8877029	8908716
chr2	8882510	8958642
chr2	8888017	8958642
chr2	8908620	8958642
chr2	9279316	9297445
chr2	9279316	9323250
chr2	9279316	9327911
chr2	9279316	9335179
chr2	9279316	9350895
chr2	9279316	9358889
chr2	9297299	9335179
chr2	9318523	9323250

chr2	9318523	9327911
chr2	9318523	9334813
chr2	9318523	9335179
chr2	9320287	9368519
chr2	9344531	9344800
chr2	9344531	9350895
chr2	9344531	9356345
chr2	9344531	9358889
chr2	9344531	9368519
chr2	9344531	9374944
chr2	9350807	9358889
chr2	9350807	9368519
chr2	9350807	9374944
chr2	9356046	9358889
chr2	9356046	9368519
chr2	9358755	9368519
chr2	9358755	9393647
chr2	9368424	9385358
chr2	9376907	9380808
chr2	9376907	9388546
chr2	9385244	9393647
chr2	9408112	9418732
chr2	9412268	9418732
chr2	9428764	9436361
chr2	9428764	9441976
chr2	9430751	9440666
chr2	9432510	9433960
chr2	9441817	9453021
chr2	9443514	9459618
chr2	9448197	9459618
chr2	9448197	9471439
chr2	9475986	9484550
chr2	9509978	9527954
chr2	9509978	9536828
chr2	9509978	9543285
chr2	95100473	95101723
chr2	95100473	95104730
chr2	95100473	95117945
chr2	95152673	95157439
chr2	9517900	9536828
chr2	9517900	9543285
chr2	9521202	9523338
chr2	9521202	9527954
chr2	9526110	9543285
chr2	9527785	9536828

chr2	9535833	9536828
chr2	9535833	9543285
chr2	95374295	95375208
chr2	9587413	9591515
chr2	95978121	95982362
chr2	96192368	96195549
chr2	96194957	96195549
chr2	96267320	96269355
chr2	96289798	96290815
chr2	96360142	96360710
chr2	96360142	96367373
chr2	96601642	96602862
chr2	96610725	96612934
chr2	96707713	96738067
chr2	96733518	96738067
chr2	96797011	96797647
chr2	96797011	96799648
chr2	96853400	96854441
chr2	96861578	96864148
chr2	96861578	96867923
chr2	97142639	97154741
chr2	97146485	97154741
chr2	97158106	97167767
chr2	97167576	97187401
chr2	97179737	97194917
chr2	97658878	97661946
chr2	97766113	97766602
chr2	97805550	97812555
chr2	97813970	97818721
chr2	97813970	97833426
chr2	97833364	97844261
chr2	97888051	97927487
chr2	9845219	9854421
chr2	9845219	9876018
chr2	9845219	9882805
chr2	9849372	9868429
chr2	9849372	9882805
chr2	9851540	9905006
chr2	98609755	98616533
chr2	98655444	98658264
chr2	98655444	98678471
chr2	9882705	9919820
chr2	9910735	9919820
chr2	99169549	99171429
chr2	99186176	99195756

chr2	99194750	99195756
chr2	99369391	99371730
chr2	99412730	99435941
chr2	99418827	99435941
chr2	99421498	99435941
chr2	99424151	99464985
chr2	99429839	99435941
chr2	99442316	99464985
chr2	99449335	99464985
chr2	99462495	99464985
chr2	9954914	9964042
chr2	9961045	9986282
chr2	9961045	9993244
chr2	9995878	9999029
chr3	100160429	100162432
chr3	100279648	100302237
chr3	100283606	100290701
chr3	100295086	100298045
chr3	100295301	100299331
chr3	100296171	100319204
chr3	100319068	100320971
chr3	10032831	10035233
chr3	10032831	10036339
chr3	100339086	100341161
chr3	100339086	100348881
chr3	10034468	10035233
chr3	10036286	10039845
chr3	100372605	100373645
chr3	100372605	100386978
chr3	100381614	100386978
chr3	10046579	10049505
chr3	10046579	10052497
chr3	100555208	100558589
chr3	100555208	100568967
chr3	10065393	10065979
chr3	10065393	10072981
chr3	10065393	10078197
chr3	10065393	10088950
chr3	100713642	100720058
chr3	100719974	100732672
chr3	100732507	100736716
chr3	10078080	10081464
chr3	10081099	10081464
chr3	10088448	10088950
chr3	101398860	101459052

chr3	101417592	101459052
chr3	101579789	101585511
chr3	101652495	101652938
chr3	101652495	101654821
chr3	101664537	101665808
chr3	101664537	101671361
chr3	101664537	101672213
chr3	101671129	101672213
chr3	101731839	101732654
chr3	101801234	101807126
chr3	101857072	101857459
chr3	10209588	10213548
chr3	10213206	10213548
chr3	10226370	10239039
chr3	10234458	10239039
chr3	105552467	105571964
chr3	105670232	105685466
chr3	105681723	105704173
chr3	107645835	107716849
chr3	107710451	107716849
chr3	107710451	107728960
chr3	107710451	107733023
chr3	107710451	107744710
chr3	107710451	107755678
chr3	107728764	107733023
chr3	107732955	107755678
chr3	108057476	108071182
chr3	108554375	108560028
chr3	108576270	108585212
chr3	108605334	108616657
chr3	108661876	108662257
chr3	108669680	108677598
chr3	111112029	111118952
chr3	111112029	111122238
chr3	111112029	111126335
chr3	111112029	111147484
chr3	111144898	111147484
chr3	111913318	111920419
chr3	111913318	111932397
chr3	111913318	111945357
chr3	111913318	111952712
chr3	111919071	111939630
chr3	111945267	111962312
chr3	112537734	112550262
chr3	112541802	112558417

chr3	112548532	112558417
chr3	112607175	112616858
chr3	11298685	11299416
chr3	112993025	112999564
chr3	113010642	113017602
chr3	113011044	113014032
chr3	11306942	11315493
chr3	11313303	11333093
chr3	11315343	11342279
chr3	11331339	11342279
chr3	11331339	11348035
chr3	11331339	11364734
chr3	11331339	11380052
chr3	11331339	11426926
chr3	113341743	113344712
chr3	113358744	113366309
chr3	113379305	113416627
chr3	113395749	113416627
chr3	113395749	113427339
chr3	113399905	113407041
chr3	113399905	113416627
chr3	113399905	113427339
chr3	11340644	11348035
chr3	11340644	11426926
chr3	113416524	113433669
chr3	113427186	113433669
chr3	11347876	11380052
chr3	113488944	113499582
chr3	113488944	113506605
chr3	113493205	113506605
chr3	113499438	113506605
chr3	11358417	11362928
chr3	11358417	11364734
chr3	11358417	11380052
chr3	11358417	11426926
chr3	113722905	113724095
chr3	113723421	113724095
chr3	113778740	113786383
chr3	113778740	113788875
chr3	113786231	113788875
chr3	113788712	113789840
chr3	11379971	11426926
chr3	114065236	114070838
chr3	11601832	11703047
chr3	11807832	11817337

chr3	11807832	11839314
chr3	11809516	11829864
chr3	11829713	11839314
chr3	11829713	11844211
chr3	119216239	119218772
chr3	119380903	119383226
chr3	119500694	119504021
chr3	119500694	119513719
chr3	119500694	119517315
chr3	119503531	119513719
chr3	119503531	119517315
chr3	119503953	119517315
chr3	119513640	119517315
chr3	119843254	119876508
chr3	119843254	119905852
chr3	119863418	119876508
chr3	119863418	119905852
chr3	119876412	120002239
chr3	119905754	119916174
chr3	120334861	120335953
chr3	120770727	120776664
chr3	121439991	121473487
chr3	121449314	121467640
chr3	121449314	121468431
chr3	121467518	121498670
chr3	121468304	121473487
chr3	121485040	121498670
chr3	121493477	121498670
chr3	121496807	121498670
chr3	121510038	121544906
chr3	121519870	121537208
chr3	121519870	121544906
chr3	121529644	121533209
chr3	121532989	121541479
chr3	121532989	121544906
chr3	121714860	121730973
chr3	121772556	121781874
chr3	121781742	121809009
chr3	121790072	121809009
chr3	121844452	121857156
chr3	121854686	121872788
chr3	122362967	122371800
chr3	122433660	122437295
chr3	122437169	122442116
chr3	122437169	122467429

chr3	122442037	122452064
chr3	122442037	122454001
chr3	122442037	122464041
chr3	122449573	122454001
chr3	122449573	122467429
chr3	122453869	122464041
chr3	122461223	122467429
chr3	122550583	122552639
chr3	122687079	122695662
chr3	122703741	122708268
chr3	122752590	122755431
chr3	122752590	122777906
chr3	122759223	122768882
chr3	122768700	122777906
chr3	122833316	122860202
chr3	123089167	123092442
chr3	123089167	123111004
chr3	123089167	123124343
chr3	123106748	123124343
chr3	123110943	123124343
chr3	123116230	123124343
chr3	123124065	123124343
chr3	123124065	123130616
chr3	123209198	123225297
chr3	123209198	123259589
chr3	123223558	123259589
chr3	123347781	123352581
chr3	123638070	123640504
chr3	123707753	123708895
chr3	12379703	12406081
chr3	123931101	123944990
chr3	123933672	123947319
chr3	123933672	123949132
chr3	123944848	123949132
chr3	12405881	12417154
chr3	124735092	124738239
chr3	124740023	124742266
chr3	124773689	124859446
chr3	124796387	124809156
chr3	124796387	124817710
chr3	124817620	124848558
chr3	124819738	124821474
chr3	124841382	124848558
chr3	12489783	12496554
chr3	12489783	12505231

chr3	12496517	12503784
chr3	12496517	12505231
chr3	125027204	125029488
chr3	12503261	12519197
chr3	12505153	12519197
chr3	12516610	12519197
chr3	125277725	125288228
chr3	125277725	125313656
chr3	125277725	125331238
chr3	125288102	125331238
chr3	12528887	12531659
chr3	125313307	125323444
chr3	125313307	125331238
chr3	125323308	125331238
chr3	125451304	125453955
chr3	125451304	125460860
chr3	125469453	125504744
chr3	125489407	125504744
chr3	125497340	125504744
chr3	125560378	125567595
chr3	125560378	125582978
chr3	125576188	125582978
chr3	125578959	125582978
chr3	12570070	12572373
chr3	12574791	12576741
chr3	12599690	12600415
chr3	12599690	12604289
chr3	12599690	12606299
chr3	12604135	12606299
chr3	126050942	126068246
chr3	12608765	12618747
chr3	126459652	126472305
chr3	126470894	126472305
chr3	126621741	126622540
chr3	126629378	126647296
chr3	126642031	126647296
chr3	126727077	126730630
chr3	126727077	126733222
chr3	127003329	127006178
chr3	127003329	127007913
chr3	127032386	127032836
chr3	127676966	127677563
chr3	127710575	127722566
chr3	127710575	127781895
chr3	128060101	128060661

chr3	128087705	128119414
chr3	128097299	128104924
chr3	128246835	128264781
chr3	128262127	128264781
chr3	12839348	12840242
chr3	128622163	128626832
chr3	128629950	128632157
chr3	128637798	128644983
chr3	128795359	128798069
chr3	128795359	128807671
chr3	128806371	128807671
chr3	128893554	128901349
chr3	128902552	128904505
chr3	129263903	129265792
chr3	129265548	129267099
chr3	12935447	12941865
chr3	129458598	129464781
chr3	129463559	129469417
chr3	129583566	129589527
chr3	129827802	129832826
chr3	129827802	129880559
chr3	129832773	129880559
chr3	130684249	130708492
chr3	130703722	130708492
chr3	130716395	130730442
chr3	130930415	130941699
chr3	130930415	130956179
chr3	130930415	130980679
chr3	130953820	130956179
chr3	130953820	130980679
chr3	130967144	130969396
chr3	130967144	130980679
chr3	130967329	130975488
chr3	130992950	131001344
chr3	131109802	131133956
chr3	131132744	131133956
chr3	131152387	131170887
chr3	131152387	131273574
chr3	131162407	131170887
chr3	131228527	131243496
chr3	131468090	131471279
chr3	131489980	131501715
chr3	132446474	132450847
chr3	132473144	132478140
chr3	132483374	132484672

chr3	132488297	132491051
chr3	132499125	132500913
chr3	132499125	132502468
chr3	132525609	132531097
chr3	132575771	132605205
chr3	132576943	132605205
chr3	132603228	132631479
chr3	132618633	132631479
chr3	132626690	132644896
chr3	132639491	132644896
chr3	132641971	132644896
chr3	132704197	132705814
chr3	132704197	132708257
chr3	132713125	132716909
chr3	13351878	13354107
chr3	13351878	13388453
chr3	133608534	133611141
chr3	133608534	133612552
chr3	133612388	133624175
chr3	133616813	133649943
chr3	133623090	133624175
chr3	133628361	133628733
chr3	133637875	133640170
chr3	134175608	134183071
chr3	134175608	134188923
chr3	134175608	134191974
chr3	134175608	134195182
chr3	134175608	134202874
chr3	134183001	134191974
chr3	134183001	134195182
chr3	134183001	134222539
chr3	134188836	134191974
chr3	134188836	134195182
chr3	134188836	134202874
chr3	134202729	134211607
chr3	134202729	134222539
chr3	134207471	134211607
chr3	134207471	134222539
chr3	134531844	134537268
chr3	13500712	13502980
chr3	136026831	136049361
chr3	136082264	136106322
chr3	136255855	136262065
chr3	136260478	136262065
chr3	136260478	136317064

chr3	136283836	136317064
chr3	136316940	136328857
chr3	136326802	136328857
chr3	136349157	136359296
chr3	136357719	136377752
chr3	136363365	136398829
chr3	136369107	136377752
chr3	136377659	136417972
chr3	136377659	136423044
chr3	136398748	136422613
chr3	136398748	136422857
chr3	136398748	136423044
chr3	136398748	136477412
chr3	136417884	136423044
chr3	136417884	136477412
chr3	136417884	136502779
chr3	136421092	136433659
chr3	136433555	136477412
chr3	136433555	136502779
chr3	136443286	136464988
chr3	136443286	136473637
chr3	136443286	136477412
chr3	136443286	136502779
chr3	136464880	136477412
chr3	136464880	136502779
chr3	136472412	136477412
chr3	136542118	136630981
chr3	136980465	136995556
chr3	136989440	136995556
chr3	138209816	138223733
chr3	138209816	138245183
chr3	138221925	138223733
chr3	138221925	138229010
chr3	138221925	138235114
chr3	138263738	138273116
chr3	138263738	138274544
chr3	138272966	138284526
chr3	138274448	138290639
chr3	138525489	138532570
chr3	138525489	138537347
chr3	138570317	138571356
chr3	138570317	138572914
chr3	138570317	138572932
chr3	138570317	138591956
chr3	138621406	138622416

chr3	138621406	138629156
chr3	138657689	138665203
chr3	138657689	138684803
chr3	138663905	138684803
chr3	138663905	138694907
chr3	138663905	138699095
chr3	138681966	138684803
chr3	138681966	138688974
chr3	138681966	138691143
chr3	138681966	138694907
chr3	138681966	138699095
chr3	138683677	138688974
chr3	138683677	138691143
chr3	138683677	138694907
chr3	138688874	138691143
chr3	138688874	138694907
chr3	138688874	138699095
chr3	138690999	138699095
chr3	138694785	138699095
chr3	138698906	138707289
chr3	138698906	138712304
chr3	138698906	138714719
chr3	138707158	138733438
chr3	138733360	138737886
chr3	138733360	138755979
chr3	138733360	138759359
chr3	138755753	138759359
chr3	141260122	141287453
chr3	141278341	141279504
chr3	141278341	141287453
chr3	141368620	141369946
chr3	141368620	141381487
chr3	141368620	141386937
chr3	141368620	141404031
chr3	141396258	141404031
chr3	141512162	141540609
chr3	141553856	141559995
chr3	141555839	141559995
chr3	141903619	141916020
chr3	141907166	141922063
chr3	141913651	141922063
chr3	141959673	141978682
chr3	141959673	141995141
chr3	141974047	142093127
chr3	141978519	141995141

chr3	142054114	142101841
chr3	142093060	142101841
chr3	142170324	142177576
chr3	142170324	142183049
chr3	142170324	142198933
chr3	142184710	142198933
chr3	142185928	142198933
chr3	142186451	142215692
chr3	142347233	142359931
chr3	142355400	142365366
chr3	142355400	142370620
chr3	142365046	142370620
chr3	142365046	142403772
chr3	142376478	142384685
chr3	142397328	142400547
chr3	142397328	142405076
chr3	142417139	142423642
chr3	142425221	142426841
chr3	142465096	142468068
chr3	142485139	142497192
chr3	142493131	142498774
chr3	142493131	142499718
chr3	142512259	142536201
chr3	142519668	142522841
chr3	142558623	142560454
chr3	142566120	142568154
chr3	142664201	142671122
chr3	142664201	142678113
chr3	142669389	142671122
chr3	142669389	142678113
chr3	142736378	142743586
chr3	142736378	142748460
chr3	142736378	142781029
chr3	142736378	142785040
chr3	142736378	142791158
chr3	142736378	142792967
chr3	142736378	142804630
chr3	142748257	142781029
chr3	142748257	142785040
chr3	142748257	142791158
chr3	142748257	142792967
chr3	142748257	142802344
chr3	142777631	142785040
chr3	142777631	142792967
chr3	142802168	142804630

chr3	143012221	143016975
chr3	143021349	143028646
chr3	143021472	143022662
chr3	143038107	143043276
chr3	14443623	14447816
chr3	14443623	14466650
chr3	14466515	14468212
chr3	14466515	14481841
chr3	146071041	146086908
chr3	146076781	146091901
chr3	146079115	146085273
chr3	146081737	146085273
chr3	146081737	146121248
chr3	146086786	146091901
chr3	146088585	146121248
chr3	146102754	146110448
chr3	146102754	146124229
chr3	146110284	146124229
chr3	146121111	146124229
chr3	14654425	14661707
chr3	148994141	148996904
chr3	148994141	149009402
chr3	148994141	149024272
chr3	149059717	149060858
chr3	149059717	149073322
chr3	149063430	149064866
chr3	149063430	149071657
chr3	149063430	149076047
chr3	149074214	149076047
chr3	149145353	149155215
chr3	149150598	149158846
chr3	149160045	149163949
chr3	149813924	149872154
chr3	149846010	149872154
chr3	149846010	149895560
chr3	149846010	149902162
chr3	149846010	149912083
chr3	149846010	149921227
chr3	149852515	149921227
chr3	149852515	149960136
chr3	149872028	149921227
chr3	149895472	149912083
chr3	149895472	149921227
chr3	149902071	149921227
chr3	15003875	15004613

chr3	15003875	15013789
chr3	15013588	15016254
chr3	15016151	15030452
chr3	15028585	15030452
chr3	15032378	15034809
chr3	150558387	150568292
chr3	150562541	150568292
chr3	150564298	150575762
chr3	150575648	150581746
chr3	150622384	150624899
chr3	150706926	150708009
chr3	15080731	15082608
chr3	15080731	15085961
chr3	15082366	15085961
chr3	151116337	151122974
chr3	151116337	151127984
chr3	151127824	151193666
chr3	151163892	151193666
chr3	151185329	151193666
chr3	151188353	151190931
chr3	15212103	15218540
chr3	15212103	15230535
chr3	15217424	15220981
chr3	15230441	15233973
chr3	15232518	15247457
chr3	15240472	15245671
chr3	152414940	152432920
chr3	152445281	152447773
chr3	154129573	154194718
chr3	15415497	15426635
chr3	154187684	154194718
chr3	154187684	154217958
chr3	154194643	154240579
chr3	154217868	154240579
chr3	154284582	154295339
chr3	154288865	154292694
chr3	155114993	155118811
chr3	15573158	15596424
chr3	155767508	155802591
chr3	155828181	155834041
chr3	155828181	155842619
chr3	155829687	155842619
chr3	155833467	155834041
chr3	155833467	155842619
chr3	155893517	155898041

chr3	155893517	155906263
chr3	155893517	155911279
chr3	155893517	155919338
chr3	155893517	155931880
chr3	155893517	155935046
chr3	155897926	155906263
chr3	155897926	155911279
chr3	155903862	155911279
chr3	155906159	155911279
chr3	155906159	155916192
chr3	155910691	155914570
chr3	155910691	155925366
chr3	155910691	155935046
chr3	155914418	155919338
chr3	155914418	155922302
chr3	155919232	155925366
chr3	155919232	155935046
chr3	155922186	155925366
chr3	156677656	156696025
chr3	156694019	156696025
chr3	15675897	15679563
chr3	15675897	15690220
chr3	15685225	15690220
chr3	15707923	15711274
chr3	157149286	157153156
chr3	157149484	157152241
chr3	15720914	15751820
chr3	15735409	15737233
chr3	15735409	15751820
chr3	15735409	15766312
chr3	158122102	158123991
chr3	158122102	158203245
chr3	158646164	158646947
chr3	158646164	158653467
chr3	158652095	158659059
chr3	158652095	158684668
chr3	158658921	158666386
chr3	158658921	158682157
chr3	158681994	158684668
chr3	159866162	159888943
chr3	159886206	159892190
chr3	160277305	160280814
chr3	160277305	160282613
chr3	160277305	160319939
chr3	160285803	160303989

chr3	160300882	160319939
chr3	160356012	160366152
chr3	160356012	160377540
chr3	160356012	160381718
chr3	160356012	160384646
chr3	160366042	160384646
chr3	160375811	160381718
chr3	160375811	160384646
chr3	160423424	160423840
chr3	160423424	160428942
chr3	160424866	160428942
chr3	160504957	160531557
chr3	160514076	160535565
chr3	160521778	160528039
chr3	160521778	160531557
chr3	161224929	161235212
chr3	161233398	161242653
chr3	161234726	161235212
chr3	161234726	161238826
chr3	161241045	161247330
chr3	16269125	16271722
chr3	16301625	16302748
chr3	16377717	16434037
chr3	167695595	167704895
chr3	167695595	167720273
chr3	167704841	167720273
chr3	167704841	167725473
chr3	167720061	167725473
chr3	168036835	168041474
chr3	168043378	168048365
chr3	168309304	168314176
chr3	170113359	170117476
chr3	170113359	170122744
chr3	170117225	170122744
chr3	170122590	170129552
chr3	170122590	170136665
chr3	170122590	170149244
chr3	170136418	170145521
chr3	170136418	170178938
chr3	170145422	170149244
chr3	170145422	170172712
chr3	170145422	170178938
chr3	170149085	170178938
chr3	170171372	170178938
chr3	170235229	170280403

chr3	170259968	170275287
chr3	170259968	170280403
chr3	170270420	170275287
chr3	170270420	170280403
chr3	170270420	170284596
chr3	170280226	170284596
chr3	170284460	170293508
chr3	170284460	170299110
chr3	170291853	170293508
chr3	170291853	170297393
chr3	170291853	170299110
chr3	170293382	170297393
chr3	170293382	170299110
chr3	170359698	170381341
chr3	170359698	170384765
chr3	170359698	170390464
chr3	170381243	170391260
chr3	170384532	170390464
chr3	170384532	170391260
chr3	170390222	170391260
chr3	171123595	171126151
chr3	171188701	171194635
chr3	171642839	171677694
chr3	171674499	171692442
chr3	17185108	17214370
chr3	17185108	17258591
chr3	172112451	172133546
chr3	172112451	172226947
chr3	172112451	172251541
chr3	172133470	172247776
chr3	172133470	172251541
chr3	172133470	172333175
chr3	172226870	172247776
chr3	172226870	172251541
chr3	172247532	172251541
chr3	172247532	172285984
chr3	172247532	172295514
chr3	172247532	172298787
chr3	172247532	172310881
chr3	172251259	172310881
chr3	172285925	172298787
chr3	172285925	172310881
chr3	172295362	172310881
chr3	172295362	172353083
chr3	172334943	172347361

chr3	172362632	172381093
chr3	172635915	172648114
chr3	172645622	172648114
chr3	172760155	172783909
chr3	172761609	172783909
chr3	172769006	172786574
chr3	172773902	172783909
chr3	172773902	172784803
chr3	172773902	172786574
chr3	172782162	172786574
chr3	172786492	172807924
chr3	17372074	17384015
chr3	17383912	17428519
chr3	17508473	17623913
chr3	177046128	177065022
chr3	177049996	177053918
chr3	177049996	177065022
chr3	179203543	179204588
chr3	179378340	179378814
chr3	179378584	179385721
chr3	179385568	179386629
chr3	179405189	179426242
chr3	179413411	179419505
chr3	179414884	179419505
chr3	179416492	179426242
chr3	179569823	179576913
chr3	179592643	179598771
chr3	179592643	179601790
chr3	179614970	179618521
chr3	179681877	179707076
chr3	179681877	179708957
chr3	179690240	179707076
chr3	179690240	179708957
chr3	179690240	179730709
chr3	179690240	179761255
chr3	179701007	179708957
chr3	179706933	179721589
chr3	179708772	179730709
chr3	179719939	179721589
chr3	179719939	179745217
chr3	179730188	179730709
chr3	179740246	179745217
chr3	179740246	179765848
chr3	179761111	179765848
chr3	179761111	179781823

chr3	179764001	179781823
chr3	180602890	180606603
chr3	180933333	180949343
chr3	180933333	180953840
chr3	180947864	180949343
chr3	180951297	180953840
chr3	180968050	180970358
chr3	182828119	182837174
chr3	182836034	182837174
chr3	182836034	182848557
chr3	182859161	182869151
chr3	182884752	182887713
chr3	182884752	182913994
chr3	182889409	182913994
chr3	182947237	182961356
chr3	182947237	182965753
chr3	182961225	182965753
chr3	183037217	183041750
chr3	183041566	183071357
chr3	183057310	183071357
chr3	183070998	183071357
chr3	183086692	183092545
chr3	183086692	183094605
chr3	183205875	183207823
chr3	183276871	183389779
chr3	183309715	183318217
chr3	183318067	183389779
chr3	183323234	183389779
chr3	183338799	183389779
chr3	183379296	183389779
chr3	183643479	183665039
chr3	183643479	183672484
chr3	183650295	183672484
chr3	183663457	183672484
chr3	183715143	183718592
chr3	183715143	183722136
chr3	183715143	183728851
chr3	183717650	183728851
chr3	183724418	183728851
chr3	183724418	183747716
chr3	183736717	183752253
chr3	183736717	183754365
chr3	183736717	183762279
chr3	183747671	183762279
chr3	183747671	183777700

chr3	183747671	183786301
chr3	183752072	183762279
chr3	183754125	183756689
chr3	183756527	183762279
chr3	183761506	183762279
chr3	183772304	183776123
chr3	183772304	183777700
chr3	183772304	183786301
chr3	183775914	183790980
chr3	183777541	183790980
chr3	183797922	183801528
chr3	183797922	183807092
chr3	183833491	183833825
chr3	183833491	183868060
chr3	183840569	183844326
chr3	183840569	183868060
chr3	183844230	183868060
chr3	183862752	183868060
chr3	183927329	183938060
chr3	183937900	183947510
chr3	183937900	183961654
chr3	183942726	183947510
chr3	183963384	183967766
chr3	183967694	183977624
chr3	183977516	183978651
chr3	184140079	184140730
chr3	184142501	184142886
chr3	184163656	184168065
chr3	184180848	184182248
chr3	184181695	184182868
chr3	184181911	184182868
chr3	184244600	184245812
chr3	184305767	184306834
chr3	184315762	184317497
chr3	184317320	184317816
chr3	184324877	184328990
chr3	184327829	184328756
chr3	184358681	184359131
chr3	184824544	184826231
chr3	184824544	184850022
chr3	184824544	184863067
chr3	184824544	184894925
chr3	184824544	184898654
chr3	184849070	184855818
chr3	184849070	184869528

chr3	184852499	184869528
chr3	184886109	184900972
chr3	184886109	184999861
chr3	184900920	184920198
chr3	184913518	184920198
chr3	184913518	184930568
chr3	184924861	184971752
chr3	184924861	184983094
chr3	184924861	184999861
chr3	184936245	184957521
chr3	184936245	184966713
chr3	184936245	184971752
chr3	184936245	184983094
chr3	184936245	184999861
chr3	184936245	185024389
chr3	184957373	184966713
chr3	184957373	184971752
chr3	184957373	184983094
chr3	184964467	184983094
chr3	184964467	184999861
chr3	184982565	184999861
chr3	185437446	185443636
chr3	185437446	185451395
chr3	185494622	185496981
chr3	185590113	185600855
chr3	185590113	185609350
chr3	185598411	185613408
chr3	185598411	185614740
chr3	185598411	185626393
chr3	185600764	185613408
chr3	185614563	185621905
chr3	185617479	185624082
chr3	185619298	185621905
chr3	185619298	185626393
chr3	185672540	185675913
chr3	185672540	185689627
chr3	185687056	185689627
chr3	185687056	185692762
chr3	185687056	185698347
chr3	185692698	185698347
chr3	185919436	185923984
chr3	185921103	185922126
chr3	185921103	185923984
chr3	186057074	186066072
chr3	186079816	186081175

chr3	186079816	186105942
chr3	186105304	186105942
chr3	186551128	186558572
chr3	186784563	186785101
chr3	186784563	186786051
chr3	186786501	186787264
chr3	186786501	186787584
chr3	186787126	186787584
chr3	186787126	186787882
chr3	186791724	186792947
chr3	186791724	186794777
chr3	186791724	186804724
chr3	186797534	186804724
chr3	186801116	186804724
chr3	186963784	187043310
chr3	186963784	187051346
chr3	187038741	187051346
chr3	188406111	188524787
chr3	189207375	189238909
chr3	189215291	189238909
chr3	189238732	189310539
chr3	189983045	189989038
chr3	189986787	189995442
chr3	190550341	190564353
chr3	190604127	190609181
chr3	190608994	190629498
chr3	191357087	191361159
chr3	191357087	191370036
chr3	191357087	191380932
chr3	192335360	192360538
chr3	193643372	193664996
chr3	194425341	194431892
chr3	194427074	194437573
chr3	194429677	194431892
chr3	194430942	194438972
chr3	194433771	194437573
chr3	194433771	194441461
chr3	194433771	194450276
chr3	194433771	194454392
chr3	194437094	194448009
chr3	194444724	194448009
chr3	194446926	194457174
chr3	194448456	194454392
chr3	194455892	194462236
chr3	194459470	194462236

chr3	194604286	194611020
chr3	194610915	194615697
chr3	194610915	194617269
chr3	194623223	194628509
chr3	194644572	194646243
chr3	194658956	194660133
chr3	194665556	194666572
chr3	195156448	195226856
chr3	195289120	195320813
chr3	195291705	195336976
chr3	195294718	195297281
chr3	195294718	195320813
chr3	195295707	195302174
chr3	195295707	195308837
chr3	195295707	195345317
chr3	195301574	195302174
chr3	195306510	195308837
chr3	195306510	195320813
chr3	195306510	195333123
chr3	195306510	195345317
chr3	195320700	195345317
chr3	195326884	195345317
chr3	195342470	195345317
chr3	195342470	195392147
chr3	195381008	195392147
chr3	195523691	195524896
chr3	195668518	195673987
chr3	195668518	195677848
chr3	195671131	195673987
chr3	195724679	195729739
chr3	195866888	195888606
chr3	195878252	195888606
chr3	195963299	195982310
chr3	195974396	195979866
chr3	195979743	195982310
chr3	196053417	196058632
chr3	196053417	196060247
chr3	196055079	196060247
chr3	196058283	196060247
chr3	196058283	196062939
chr3	196058283	196065600
chr3	196058283	196077122
chr3	196062581	196065600
chr3	196064308	196065600
chr3	196064308	196077122

chr3	196065442	196077122
chr3	196067517	196077122
chr3	196069454	196071498
chr3	196069454	196074125
chr3	196069454	196077122
chr3	196071395	196077122
chr3	196072002	196075360
chr3	196072002	196077122
chr3	196073929	196075360
chr3	196073929	196077122
chr3	196075158	196077122
chr3	196241947	196248323
chr3	196247366	196248323
chr3	196257787	196270541
chr3	196257787	196283538
chr3	196270414	196283538
chr3	196323743	196324440
chr3	196323743	196327591
chr3	196361843	196362687
chr3	196361843	196368155
chr3	196361843	196372042
chr3	196361843	196403019
chr3	196362293	196369511
chr3	196362293	196393619
chr3	196368027	196369511
chr3	196368027	196372042
chr3	196368027	196403019
chr3	196369420	196372042
chr3	196369420	196403019
chr3	196371895	196403019
chr3	196391812	196393619
chr3	196391812	196403019
chr3	196391812	196407393
chr3	196402951	196407393
chr3	196475230	196488683
chr3	196483769	196487578
chr3	196483769	196488683
chr3	196487398	196488683
chr3	196722414	196731092
chr3	196782625	196803164
chr3	196782625	196805383
chr3	196801926	196803164
chr3	196801926	196805383
chr3	196805351	196807914
chr3	196806578	196807914

chr3	196806578	196818156
chr3	196810589	196814568
chr3	196810589	196818156
chr3	196812218	196814568
chr3	196812218	196820567
chr3	196814450	196818156
chr3	196814450	196820567
chr3	196818056	196820567
chr3	196899665	196900412
chr3	196899665	196903610
chr3	197003900	197006736
chr3	197003900	197009812
chr3	197015364	197016369
chr3	197015364	197017290
chr3	197051576	197059998
chr3	197051576	197066754
chr3	197051576	197069260
chr3	197065275	197081117
chr3	197076585	197091026
chr3	197081050	197085756
chr3	197081050	197119530
chr3	197085579	197105005
chr3	197085579	197116083
chr3	197085579	197119530
chr3	197090911	197119530
chr3	197090911	197130671
chr3	197104902	197116083
chr3	197104902	197119530
chr3	197104902	197130671
chr3	197104902	197149796
chr3	197115926	197119530
chr3	197115926	197142768
chr3	197115926	197149796
chr3	197119409	197142768
chr3	197130526	197149796
chr3	197136541	197142768
chr3	197136541	197149796
chr3	197136541	197161738
chr3	197149742	197194589
chr3	197282678	197297235
chr3	197532411	197546486
chr3	197693714	197694585
chr3	197694374	197705175
chr3	197704541	197705175
chr3	197756425	197768587

chr3	197756425	197770244
chr3	197756425	197778464
chr3	197773402	197778464
chr3	197814907	197820430
chr3	197814907	197829673
chr3	197814907	197832317
chr3	197817175	197820430
chr3	197826877	197832317
chr3	197830769	197839397
chr3	197830769	197866219
chr3	197832196	197839397
chr3	197832196	197866219
chr3	197835673	197865471
chr3	197847408	197848021
chr3	197847408	197852620
chr3	197858833	197866219
chr3	197990558	197996282
chr3	19975600	19976169
chr3	19975642	19978403
chr3	20119597	20148487
chr3	20125904	20140364
chr3	20176600	20184034
chr3	20183607	20184034
chr3	23208691	23217312
chr3	23499607	23532701
chr3	23900807	23911450
chr3	23910810	23911450
chr3	23954536	23956125
chr3	23954536	23959815
chr3	23954536	23968023
chr3	23956036	23959815
chr3	25629034	25630939
chr3	25638164	25642385
chr3	25643693	25645455
chr3	25751097	25778688
chr3	25764065	25778688
chr3	27201509	27284961
chr3	27344271	27352919
chr3	27379248	27391808
chr3	27379248	27403384
chr3	27383152	27391808
chr3	27389930	27398353
chr3	27394517	27395115
chr3	27394517	27403384
chr3	27397683	27403384

chr3	27404829	27421779
chr3	27411641	27424152
chr3	27411641	27434104
chr3	27418485	27424152
chr3	27418485	27434104
chr3	27420699	27434104
chr3	27421621	27424152
chr3	27424036	27452498
chr3	27433915	27452498
chr3	27437387	27448797
chr3	27437387	27452498
chr3	27448650	27452498
chr3	29868857	29897475
chr3	29868857	29899755
chr3	29868857	29936196
chr3	29988142	29991209
chr3	30644746	30650460
chr3	30644746	30674246
chr3	30671637	30674246
chr3	3137259	3140648
chr3	3137259	3144710
chr3	3140509	3144710
chr3	3156218	3174261
chr3	31576395	31580096
chr3	31576395	31600459
chr3	31596797	31633147
chr3	31615104	31619830
chr3	31615104	31633147
chr3	3167633	3174261
chr3	3167633	3175269
chr3	31683633	31702508
chr3	31876432	31879830
chr3	32128075	32146734
chr3	32138586	32140366
chr3	32138586	32146734
chr3	32357372	32367988
chr3	32487937	32491886
chr3	32528445	32533097
chr3	32532985	32537104
chr3	32532985	32541206
chr3	32534510	32541206
chr3	32541036	32545965
chr3	32703867	32704972
chr3	32703867	32708820
chr3	32716224	32717237

chr3	32716224	32737490
chr3	32733422	32734976
chr3	32733422	32737490
chr3	32762732	32764809
chr3	33014055	33018561
chr3	33014055	33046232
chr3	33051757	33068970
chr3	33053490	33072713
chr3	33065462	33072713
chr3	33068229	33068970
chr3	33068229	33072713
chr3	33120343	33134265
chr3	33124407	33134265
chr3	33358966	33364719
chr3	33392562	33425741
chr3	33400188	33425741
chr3	33408689	33425741
chr3	33409235	33425741
chr3	33411580	33425741
chr3	33535232	33584920
chr3	33543432	33559385
chr3	33551251	33604209
chr3	33573109	33584920
chr3	33576168	33584920
chr3	33581820	33584920
chr3	33581820	33592496
chr3	33581820	33604209
chr3	33584749	33604209
chr3	33592394	33604209
chr3	33592394	33608626
chr3	33592394	33644903
chr3	33592394	33663515
chr3	33602951	33632374
chr3	33602951	33644903
chr3	33602951	33663515
chr3	33602951	33696933
chr3	33604153	33608626
chr3	33606590	33608626
chr3	33606590	33612071
chr3	33606590	33644903
chr3	33619602	33632371
chr3	33619602	33632374
chr3	33619602	33644903
chr3	33619602	33663515
chr3	33619602	33689932

chr3	33626987	33644903
chr3	33626987	33663515
chr3	33632291	33663515
chr3	33644756	33689932
chr3	33644756	33696933
chr3	33663444	33689932
chr3	33663444	33696933
chr3	33684358	33689932
chr3	33684358	33696933
chr3	33687059	33689932
chr3	33813558	33826580
chr3	33855165	33865430
chr3	36996618	37007063
chr3	36996618	37017599
chr3	36996623	37026007
chr3	37006990	37012099
chr3	37020309	37026007
chr3	37047518	37049017
chr3	37075023	37096660
chr3	37083635	37096660
chr3	37121491	37149038
chr3	37129062	37149038
chr3	37251394	37282272
chr3	37251394	37289291
chr3	37273535	37282272
chr3	37273535	37289291
chr3	37273535	37296219
chr3	37281957	37302332
chr3	37286013	37299371
chr3	37286013	37302332
chr3	37289234	37296219
chr3	37294978	37296219
chr3	37302184	37315598
chr3	37335052	37340199
chr3	37335052	37347296
chr3	37335052	37355187
chr3	37337142	37355187
chr3	37411137	37417492
chr3	37947056	37975894
chr3	37964570	37975894
chr3	38008234	38009811
chr3	38183002	38198863
chr3	38183002	38236961
chr3	38190730	38216151
chr3	38221577	38230430

chr3	38223811	38230430
chr3	38372654	38400985
chr3	38379245	38400985
chr3	38501354	38506968
chr3	39063049	39068859
chr3	39069642	39084741
chr3	39408605	39410999
chr3	39411648	39412061
chr3	40310892	40311571
chr3	41224020	41224753
chr3	41224020	41227352
chr3	41224020	41239354
chr3	41398078	41455595
chr3	41566024	41717861
chr3	41681507	41681804
chr3	41681507	41717861
chr3	41681507	41754488
chr3	41705056	41717861
chr3	41705056	41754488
chr3	41705253	41754488
chr3	41715236	41717861
chr3	41789660	41835971
chr3	41789660	41954807
chr3	41800131	41835971
chr3	41835863	41935940
chr3	41883873	41919818
chr3	41895517	41932006
chr3	41895517	41935940
chr3	41896821	41900829
chr3	41931843	41938197
chr3	41931843	41954807
chr3	42184684	42189124
chr3	42184684	42191636
chr3	42555929	42563686
chr3	42617569	42621516
chr3	42619019	42632823
chr3	42621428	42632823
chr3	43299753	43303792
chr3	43299753	43332310
chr3	43299753	43343478
chr3	43332168	43343478
chr3	43549719	43561402
chr3	43549719	43565727
chr3	43549719	43574864
chr3	43549719	43577261

chr3	43561219	43574864
chr3	43561219	43600581
chr3	43565652	43577261
chr3	43565652	43580472
chr3	43574808	43577261
chr3	43574808	43580472
chr3	43576691	43580472
chr3	43598531	43605863
chr3	4410864	4420146
chr3	4410864	4453049
chr3	44354738	44401334
chr3	44367455	44401334
chr3	44455120	44458364
chr3	4449265	4453049
chr3	44629064	44631221
chr3	44629064	44632599
chr3	44629073	44631221
chr3	44629073	44632599
chr3	44775253	44800437
chr3	44775253	44805986
chr3	44778114	44828300
chr3	44780884	44786574
chr3	44780884	44797956
chr3	44780884	44800437
chr3	44784844	44794426
chr3	44784844	44801526
chr3	44794216	44797956
chr3	44801449	44812289
chr3	44813074	44840456
chr3	44814910	44828300
chr3	44826038	44828300
chr3	44826374	44840456
chr3	44828213	44838421
chr3	44829970	44831018
chr3	44829970	44840456
chr3	44929305	44933984
chr3	44929305	44959460
chr3	44945167	44959460
chr3	45089053	45091538
chr3	45089053	45093657
chr3	45093276	45095568
chr3	45110472	45112445
chr3	45110472	45118621
chr3	4516475	4521094
chr3	45394432	45419729

chr3	45400244	45419729
chr3	45400244	45446980
chr3	45400244	45458886
chr3	45474242	45500579
chr3	45496274	45513235
chr3	45703437	45709647
chr3	45703437	45723543
chr3	45703437	45732151
chr3	45721999	45723543
chr3	4674201	4675248
chr3	46999629	47001179
chr3	47037665	47042700
chr3	47037665	47046621
chr3	47037665	47057490
chr3	47037665	47062346
chr3	47037665	47067118
chr3	47042560	47057490
chr3	47046486	47057490
chr3	47046486	47067118
chr3	47056820	47067118
chr3	47062162	47067118
chr3	47067069	47098081
chr3	47083719	47098081
chr3	47086194	47098081
chr3	47086194	47103423
chr3	47086194	47106120
chr3	47097954	47103423
chr3	47097954	47106120
chr3	47105996	47116754
chr3	47113875	47116754
chr3	47113875	47126663
chr3	47423932	47428670
chr3	47425484	47428670
chr3	47425484	47435137
chr3	47425996	47428670
chr3	47427156	47428670
chr3	47435007	47443091
chr3	47498285	47501851
chr3	47498285	47511226
chr3	47610065	47622341
chr3	47610065	47638780
chr3	47610065	47678311
chr3	47638724	47678311
chr3	47661293	47676782
chr3	47661293	47678311

chr3	47661293	47686170
chr3	47661293	47693300
chr3	47662333	47675588
chr3	47662333	47678311
chr3	47670657	47676782
chr3	47670657	47678311
chr3	47675474	47676782
chr3	47675474	47678311
chr3	47675474	47686170
chr3	47678197	47686170
chr3	47678197	47701402
chr3	47686048	47693300
chr3	47686048	47701402
chr3	47689386	47729094
chr3	47693240	47701402
chr3	47693240	47714490
chr3	47701277	47714490
chr3	47701277	47729094
chr3	47706408	47714490
chr3	47706408	47720735
chr3	47706408	47729094
chr3	47706408	47736126
chr3	47706408	47772936
chr3	47710682	47714490
chr3	47710682	47720735
chr3	47710682	47745993
chr3	47710682	47772936
chr3	47714414	47745993
chr3	47714414	47772936
chr3	47720665	47745993
chr3	47720665	47772936
chr3	47729024	47745993
chr3	47729024	47772936
chr3	47736033	47745993
chr3	47736033	47772936
chr3	47738028	47745993
chr3	47738028	47772936
chr3	47745907	47772936
chr3	47818021	47829134
chr3	47840876	47843255
chr3	47853162	47977933
chr3	47867245	47869327
chr3	47867245	47872100
chr3	47867245	47877523
chr3	47870812	47872100

chr3	47870812	47877523
chr3	47918718	47921878
chr3	47977864	47998879
chr3	48167845	48177988
chr3	48174283	48177988
chr3	48174283	48183030
chr3	48174283	48183836
chr3	48180720	48184695
chr3	48450036	48451899
chr3	48450036	48454418
chr3	48450036	48459916
chr3	48535511	48536463
chr3	48535511	48550234
chr3	48543579	48550234
chr3	48549863	48550234
chr3	48599634	48605856
chr3	48689537	48691482
chr3	48756378	48794049
chr3	48756378	48807684
chr3	48765003	48783092
chr3	48765003	48794049
chr3	48765003	48807684
chr3	48765247	48794049
chr3	48772954	48794049
chr3	48772954	48807684
chr3	48782985	48794049
chr3	48782985	48807684
chr3	48922747	48927813
chr3	48927461	48980496
chr3	48979481	48980496
chr3	49016773	49017356
chr3	49024895	49025269
chr3	49024895	49028806
chr3	49032182	49033228
chr3	49032182	49092456
chr3	49044389	49057890
chr3	49044389	49077038
chr3	49056861	49077038
chr3	49098191	49103409
chr3	49113991	49118120
chr3	49283986	49284919
chr3	49286097	49298635
chr3	49292509	49294598
chr3	49300466	49302542
chr3	49302383	49311654

chr3	49305714	49311654
chr3	49305714	49325845
chr3	49305714	49327816
chr3	49324701	49325845
chr3	49324701	49327816
chr3	49368427	49375591
chr3	49839771	49844580
chr3	49967469	49975392
chr3	49967469	49999513
chr3	49967469	50048319
chr3	49972058	49999513
chr3	50048244	50059746
chr3	50059648	50062108
chr3	50060955	50062108
chr3	50065030	50066502
chr3	50068689	50070552
chr3	50092042	50093875
chr3	50093719	50105709
chr3	50099981	50105709
chr3	50105076	50110770
chr3	50106766	50108304
chr3	50106766	50109688
chr3	50106766	50114251
chr3	50113382	50114251
chr3	50117073	50117379
chr3	50173792	50175188
chr3	50173792	50176861
chr3	50252099	50253184
chr3	50252099	50257714
chr3	50256191	50257714
chr3	50288408	50290679
chr3	50288799	50289628
chr3	50640365	50642332
chr3	50640365	50647203
chr3	50778674	50934077
chr3	50890025	51075440
chr3	51146548	51160702
chr3	51159243	51160702
chr3	51208773	51229609
chr3	51208773	51246807
chr3	51260155	51271007
chr3	51270814	51280204
chr3	51280105	51333253
chr3	51310231	51374587
chr3	51416786	51418876

chr3	51418115	51420997
chr3	51427371	51433264
chr3	51429260	51430212
chr3	51429260	51433264
chr3	51433105	51441897
chr3	51440969	51441897
chr3	51440969	51443903
chr3	51443765	51466876
chr3	51463113	51483836
chr3	51470928	51483836
chr3	51470928	51496780
chr3	51627552	51630931
chr3	51629333	51635789
chr3	51630271	51630931
chr3	51633576	51634035
chr3	51674270	51684634
chr3	5170502	5172740
chr3	5170502	5174414
chr3	5172149	5172740
chr3	5195208	5207273
chr3	5195208	5208263
chr3	5195208	5211216
chr3	52122378	52125181
chr3	52122378	52138302
chr3	52122378	52151100
chr3	52202506	52208247
chr3	52204692	52206751
chr3	52204692	52212420
chr3	52412810	52414587
chr3	52576540	52579199
chr3	52576540	52589255
chr3	52634601	52642045
chr3	52634601	52643343
chr3	52634601	52648442
chr3	52641953	52648442
chr3	52658198	52679723
chr3	52678499	52679723
chr3	52686768	52687615
chr3	52686768	52689206
chr3	52687245	52690704
chr3	52687501	52690704
chr3	52690591	52691071
chr3	52692871	52693807
chr3	52693407	52694103
chr3	52695927	52700496

chr3	52737585	52739634
chr3	52737585	52741499
chr3	52737585	52752336
chr3	52743351	52746904
chr3	52743351	52752336
chr3	52744238	52766375
chr3	52749691	52752336
chr3	52760794	52766375
chr3	52763469	52766375
chr3	52911002	52916214
chr3	52912537	52913617
chr3	52912537	52916214
chr3	52912537	52931035
chr3	52918483	52928341
chr3	52920536	52928341
chr3	52926030	52928341
chr3	52930328	52932308
chr3	52943352	52954411
chr3	52943352	52969258
chr3	52954316	52969258
chr3	53178403	53179776
chr3	53178403	53183581
chr3	53225750	53242242
chr3	53228275	53242242
chr3	53240250	53242242
chr3	53290790	53292827
chr3	53290790	53304290
chr3	53874210	53876080
chr3	53874210	53878467
chr3	55888388	55992250
chr3	56080816	56173520
chr3	56139508	56149132
chr3	56558845	56564125
chr3	56563683	56567053
chr3	56563683	56571302
chr3	56566593	56567053
chr3	56566949	56571302
chr3	56592969	56594028
chr3	56624478	56641752
chr3	56627036	56628614
chr3	56638705	56641752
chr3	56638705	56649057
chr3	56648821	56673725
chr3	56660730	56671692
chr3	56660730	56673725

chr3	56662384	56670145
chr3	56663540	56673725
chr3	56666259	56673725
chr3	56668396	56673725
chr3	56669699	56671692
chr3	56669699	56673725
chr3	57235565	57242914
chr3	57242855	57248351
chr3	57242855	57253738
chr3	57242855	57267792
chr3	57246075	57248351
chr3	57246075	57253738
chr3	57247394	57253738
chr3	57575547	57577387
chr3	57575547	57584464
chr3	57583897	57584464
chr3	57630420	57633354
chr3	57641652	57646438
chr3	57657679	57663716
chr3	57657679	57672438
chr3	57659117	57663716
chr3	57663635	57672438
chr3	57831382	57849816
chr3	57831382	57864716
chr3	57841298	57858159
chr3	57847196	57858159
chr3	57847196	57862086
chr3	57847196	57864716
chr3	57871635	57896932
chr3	57890040	57923023
chr3	57907883	57923023
chr3	57916905	57923023
chr3	58077045	58081776
chr3	58077045	58098908
chr3	58094835	58096218
chr3	58094835	58098908
chr3	58106679	58121503
chr3	58118871	58121503
chr3	58132807	58163330
chr3	58145920	58150227
chr3	58256561	58267345
chr3	58256561	58274815
chr3	58365873	58382700
chr3	58369430	58391847
chr3	58429707	58430942

chr3	58431592	58431984
chr3	58806394	58831889
chr3	58913707	58937762
chr3	61989624	62003497
chr3	62078162	62168163
chr3	62157066	62168163
chr3	62191468	62195170
chr3	62191468	62203950
chr3	62218850	62262894
chr3	63835153	63839773
chr3	63898398	63913225
chr3	63898398	63952483
chr3	63912587	63913225
chr3	63912587	63952483
chr3	64018598	64022523
chr3	65361198	65364946
chr3	65361198	65383623
chr3	65429519	65442849
chr3	66236543	66243312
chr3	66236543	66263379
chr3	66243202	66263379
chr3	66405197	66417266
chr3	66405197	66462509
chr3	66412870	66417266
chr3	67495797	67498295
chr3	67495797	67508903
chr3	67495797	67520634
chr3	67495797	67529186
chr3	67495797	67609596
chr3	67508806	67529186
chr3	67518246	67529186
chr3	67518246	67609596
chr3	67528131	67529186
chr3	68998010	69004482
chr3	69001607	69004482
chr3	69029814	69035115
chr3	69033547	69044595
chr3	69039550	69044595
chr3	69042806	69044595
chr3	69043749	69048562
chr3	69057255	69077918
chr3	69063438	69077918
chr3	69064067	69075510
chr3	69071534	69077918
chr3	70976940	70978029

chr3	70987993	71015653
chr3	70987993	71053773
chr3	71015548	71047095
chr3	71015548	71053773
chr3	71041327	71053773
chr3	71041327	71359244
chr3	71690009	71699708
chr3	72792915	72817384
chr3	72792915	72824551
chr3	72792915	72842402
chr3	72792915	72844423
chr3	72812670	72817384
chr3	72812670	72824551
chr3	72812670	72844423
chr3	72815349	72844423
chr3	72817229	72842402
chr3	72817229	72844423
chr3	72824423	72844423
chr3	72832368	72844423
chr3	72841044	72842402
chr3	72841044	72844423
chr3	72955097	72957352
chr3	75741275	75741736
chr3	78646147	78717883
chr3	78667882	78714524
chr3	78670095	78685917
chr3	78685745	78717883
chr3	78714396	78717883
chr3	81577924	81581275
chr3	81577924	81586190
chr3	81577924	81591164
chr3	81577924	81594023
chr3	81577924	81642990
chr3	81577924	81649921
chr3	81577924	81670953
chr3	81577924	81705613
chr3	81591036	81648991
chr3	81591036	81705613
chr3	81642780	81705613
chr3	81670837	81705613
chr3	8532736	8537440
chr3	87240698	87245908
chr3	8767265	8768591
chr3	8890388	8913720
chr3	8898893	8902520

chr3	8898893	8913720
chr3	8912311	8941804
chr3	8935870	8941804
chr3	8935870	8959001
chr3	9365015	9366985
chr3	9365015	9371536
chr3	9366907	9371536
chr3	93995873	94003908
chr3	94049405	94053300
chr3	9424466	9434485
chr3	9424466	9435906
chr3	9424466	9442245
chr3	9424466	9448630
chr3	9424466	9464672
chr3	9428822	9434882
chr3	9434333	9475156
chr3	9443307	9445740
chr3	9453738	9464672
chr3	9662266	9671170
chr3	9662266	9672758
chr3	9669431	9671170
chr3	96866824	96987993
chr3	9734130	9739958
chr3	9750944	9751949
chr3	9750944	9754885
chr3	97843194	97864647
chr3	97886630	97889390
chr3	97895958	97900485
chr3	97895958	97915736
chr3	97947360	97959179
chr3	97949843	97959179
chr3	9797658	9801756
chr3	9816073	9817759
chr3	9840183	9840616
chr3	9840183	9843601
chr3	98756368	98772916
chr3	98756368	98773983
chr3	98768429	98773983
chr3	98817768	98881767
chr3	98849460	98881767
chr3	9948066	9948469
chr3	9973627	9977446
chr4	10083021	10084530
chr4	10097710	10099139
chr4	10097710	10103986

chr4	10097710	10116234
chr4	101080531	101099722
chr4	101080531	101109078
chr4	101093775	101109078
chr4	102304316	102315830
chr4	102525511	102537956
chr4	102525511	102567135
chr4	102533844	102567135
chr4	102576875	102584820
chr4	102580534	102582957
chr4	102664684	102674070
chr4	102664684	102714561
chr4	102668962	102726683
chr4	102689573	102726683
chr4	102714437	102726683
chr4	102722870	102726683
chr4	102799406	102802638
chr4	102799406	102809855
chr4	102799406	102826636
chr4	103028746	103047226
chr4	103048892	103067592
chr4	103066326	103067592
chr4	103194228	103196252
chr4	105241338	105243778
chr4	105370836	105399164
chr4	105370836	105438036
chr4	105386566	105399164
chr4	105386566	105438036
chr4	105396248	105424322
chr4	105396248	105438036
chr4	105424195	105446502
chr4	105424195	105449403
chr4	105424195	105453642
chr4	105424195	105456745
chr4	105437949	105449403
chr4	105437949	105456745
chr4	105446382	105453642
chr4	105446382	105456745
chr4	105691975	105693486
chr4	105691975	105695668
chr4	105693298	105695668
chr4	105693298	105700014
chr4	105719059	105729499
chr4	105719059	105842134
chr4	105822953	105825800

chr4	106171094	106212835
chr4	106171094	106236808
chr4	106171094	106242569
chr4	106171094	106308989
chr4	106193608	106212835
chr4	106193608	106242569
chr4	106212749	106236808
chr4	106230362	106236808
chr4	106242469	106262212
chr4	106260436	106308989
chr4	106295093	106308989
chr4	106324984	106331883
chr4	106324984	106337037
chr4	107631630	107645070
chr4	107631630	107701285
chr4	107644801	107660072
chr4	107644801	107701285
chr4	107682014	107687177
chr4	107682014	107694006
chr4	107682014	107701285
chr4	107687038	107701285
chr4	107693770	107701285
chr4	107944969	107947537
chr4	108009758	108014588
chr4	108009758	108027760
chr4	108655563	108657647
chr4	109449416	109463644
chr4	109462900	109494856
chr4	109473003	109481781
chr4	109481676	109494856
chr4	109491326	109494856
chr4	109491326	109510111
chr4	109491326	109512083
chr4	109491326	109516640
chr4	109506327	109513856
chr4	109506327	109516640
chr4	109513746	109520484
chr4	109513746	109530464
chr4	109521116	109521626
chr4	109527321	109533685
chr4	109530288	109531522
chr4	109659010	109664394
chr4	109943841	109945275
chr4	110488540	110491164
chr4	112253207	112260862

chr4	112558149	112563274
chr4	112560732	112585725
chr4	112562370	112585725
chr4	112562370	112589874
chr4	112603523	112633242
chr4	112606007	112620190
chr4	113174415	113199109
chr4	113292415	113360897
chr4	113293439	113346022
chr4	113302766	113318620
chr4	113457290	113465604
chr4	113500462	113517657
chr4	113509637	113531299
chr4	113509637	113552096
chr4	113531215	113548729
chr4	118282830	118298938
chr4	118308435	118318556
chr4	118313200	118316323
chr4	118313200	118318556
chr4	118313200	118338314
chr4	118331715	118338314
chr4	118605583	118608722
chr4	118728560	118732912
chr4	118815027	118815726
chr4	119245336	119248882
chr4	119267319	119269837
chr4	119460182	119462979
chr4	119460730	119462979
chr4	119493332	119497903
chr4	119596522	119607297
chr4	120710308	120811449
chr4	120754552	120799745
chr4	120754552	120811449
chr4	120754552	120816574
chr4	120777187	120821345
chr4	120811369	120821345
chr4	1212912	1241519
chr4	1215990	1241519
chr4	121677893	121681761
chr4	121677893	121686372
chr4	121801826	121810099
chr4	121804621	121810099
chr4	121807539	121811671
chr4	122189999	122201126
chr4	122189999	122207265

chr4	122192228	122207638
chr4	122219339	122220470
chr4	122262763	122266958
chr4	122272159	122289192
chr4	122272159	122301397
chr4	122279774	122281774
chr4	1225359	1228343
chr4	1225359	1238337
chr4	1225359	1241519
chr4	1228198	1241519
chr4	122912567	122917704
chr4	122927633	122938249
chr4	122927633	122979386
chr4	122927633	123028395
chr4	122933438	122938249
chr4	122933438	122979386
chr4	122935713	122938249
chr4	122935713	122979386
chr4	122935713	123028395
chr4	122979231	123028395
chr4	122979231	123090705
chr4	123028185	123057288
chr4	123028185	123090705
chr4	123056386	123090705
chr4	1238182	1241519
chr4	124668983	124678905
chr4	125463562	125481738
chr4	127663380	127687867
chr4	127669035	127687867
chr4	127794076	127798709
chr4	127805072	127811636
chr4	127811436	127822894
chr4	127889865	127893881
chr4	127889865	127895093
chr4	127921523	127939997
chr4	127930682	127933093
chr4	127930682	127938838
chr4	127930682	127942158
chr4	127982938	128028853
chr4	128017362	128028853
chr4	128074459	128077962
chr4	128074459	128082305
chr4	128074459	128091144
chr4	128074459	128091512
chr4	128074459	128098330

chr4	128074459	128107313
chr4	128074933	128082305
chr4	128077787	128091512
chr4	128091000	128091512
chr4	128091000	128098330
chr4	128114569	128178642
chr4	128121825	128162317
chr4	128162193	128179512
chr4	128176871	128179512
chr4	128179405	128207383
chr4	128466607	128470549
chr4	128831732	128852268
chr4	128831732	128862225
chr4	128842952	128852268
chr4	128888678	128946152
chr4	128936654	128943188
chr4	128936654	128946152
chr4	128936654	128948570
chr4	128959599	128999794
chr4	128992166	128999794
chr4	128992166	129003876
chr4	128992166	129039096
chr4	128997873	129003876
chr4	129039040	129044051
chr4	129039040	129082373
chr4	129043394	129082373
chr4	130787	131505
chr4	1311978	1315600
chr4	1311978	1322503
chr4	1311978	1327703
chr4	13376544	13460828
chr4	13460698	13479526
chr4	1348089	1351835
chr4	1348089	1366431
chr4	1349523	1366431
chr4	13586395	13597168
chr4	138214584	138236451
chr4	139060323	139073567
chr4	139061864	139073567
chr4	139071865	139073567
chr4	139125163	139137867
chr4	139334173	139341069
chr4	139334173	139344339
chr4	139336847	139341069
chr4	139336847	139344339

chr4	139342825	139344339
chr4	139342825	139349581
chr4	139342825	139370404
chr4	139349461	139354098
chr4	139351190	139351611
chr4	139351190	139354098
chr4	139354025	139357555
chr4	139376364	139386230
chr4	139378755	139386230
chr4	139517884	139520394
chr4	139517884	139523435
chr4	139517884	139533366
chr4	139529030	139533366
chr4	139695196	139704196
chr4	139703454	139704196
chr4	140525419	140540301
chr4	140525419	140543586
chr4	140676893	140679843
chr4	141226766	141230270
chr4	142082030	142086256
chr4	142082030	142112682
chr4	142082030	142124760
chr4	142108092	142124760
chr4	142108092	142145996
chr4	142145839	142173809
chr4	142173631	142270774
chr4	142193086	142209026
chr4	142208424	142314762
chr4	142237863	142260564
chr4	142270662	142305537
chr4	142305457	142405324
chr4	142402937	142431385
chr4	143197822	143203566
chr4	143203407	143212424
chr4	143415476	143440382
chr4	143433490	143440382
chr4	143524366	143528714
chr4	143525450	143530526
chr4	143527867	143528714
chr4	143527867	143530526
chr4	143534854	143536678
chr4	143543508	143543972
chr4	145104385	145105690
chr4	145110967	145121391
chr4	145123020	145125101

chr4	145139950	145155686
chr4	145155410	145171720
chr4	145159502	145171720
chr4	145162639	145171720
chr4	145539803	145546924
chr4	145823421	145849561
chr4	145845955	145849561
chr4	145845955	145892415
chr4	145870244	145903215
chr4	145885677	145892415
chr4	146442782	146510049
chr4	146503848	146510049
chr4	147653851	147661038
chr4	147670640	147680471
chr4	147822726	147822957
chr4	147847150	147881932
chr4	147847150	147906719
chr4	147857552	147866816
chr4	147857552	147875150
chr4	147857552	147881932
chr4	147857552	147913139
chr4	147864845	147881932
chr4	147864845	147913139
chr4	147864845	147966839
chr4	147875020	147881932
chr4	147879231	147881932
chr4	147906637	147913139
chr4	147906637	147939899
chr4	147906637	147955374
chr4	147909731	147946704
chr4	147909731	147955374
chr4	147909731	147966839
chr4	147939824	147946704
chr4	147939824	147955374
chr4	147939824	147966839
chr4	147939824	148047051
chr4	147946616	147966839
chr4	147946616	148023413
chr4	147955315	147966839
chr4	147965023	147966839
chr4	147965023	148023413
chr4	148023262	148047051
chr4	148063147	148064507
chr4	148152468	148154901
chr4	15007304	15033211

chr4	150193137	150203889
chr4	150277852	150310384
chr4	150282449	150286034
chr4	150282449	150310384
chr4	150302624	150325898
chr4	150302624	150491035
chr4	150415437	150491035
chr4	150467672	150491035
chr4	150467672	150599131
chr4	15052413	15062260
chr4	150588047	150599131
chr4	150588047	150683717
chr4	150599006	150761847
chr4	150683550	150808398
chr4	150683550	150849575
chr4	150735257	150761847
chr4	150735257	150808398
chr4	150735257	150817257
chr4	150735257	150872755
chr4	150735257	150896456
chr4	150735257	150897818
chr4	150761782	150808398
chr4	150761782	150817257
chr4	150798080	150808398
chr4	150798080	150817257
chr4	150798080	150831976
chr4	150798080	150848998
chr4	150798080	150849575
chr4	150806270	150808398
chr4	150806270	150817257
chr4	150806270	150831976
chr4	150806270	150849575
chr4	150817123	150831976
chr4	150817123	150848998
chr4	150844099	150872755
chr4	150844657	150850902
chr4	150850723	150868305
chr4	150870524	150872755
chr4	150870524	150893149
chr4	150870524	150897818
chr4	150893051	150921293
chr4	150896393	150916738
chr4	150914194	150916738
chr4	150928515	150929065
chr4	151127617	151128356

chr4	151132330	151135132
chr4	151132330	151137862
chr4	151143909	151149561
chr4	151147921	151149561
chr4	151174669	151179397
chr4	151701328	151708101
chr4	152329671	152337936
chr4	152330731	152337936
chr4	152332595	152337936
chr4	152332595	152350124
chr4	152337801	152350124
chr4	152829624	152872564
chr4	152829624	152888307
chr4	152863605	152888307
chr4	152872451	152888307
chr4	152880962	152888307
chr4	153270334	153276130
chr4	153270337	153276130
chr4	153350063	153358622
chr4	153350063	153409015
chr4	153394261	153397333
chr4	153550072	153558368
chr4	153626146	153632838
chr4	15536919	15563522
chr4	15550823	15563522
chr4	15574149	15589679
chr4	155774967	155794086
chr4	15625251	15636676
chr4	15625251	15644708
chr4	15630665	15644708
chr4	15636493	15644708
chr4	156850220	156861489
chr4	157120555	157143959
chr4	157120555	157153010
chr4	157136468	157153010
chr4	158215190	158217420
chr4	158215190	158219390
chr4	158684591	158690425
chr4	158690347	158697699
chr4	158825915	158835476
chr4	158851320	158861776
chr4	158861341	158870469
chr4	159304341	159314768
chr4	159304341	159323617
chr4	16174203	16176228

chr4	16174203	16213898
chr4	163137198	163148434
chr4	165085585	165100892
chr4	165085585	165103096
chr4	165100627	165103096
chr4	165219933	165228913
chr4	165219933	165238899
chr4	165219933	165263359
chr4	165219933	165299656
chr4	165228806	165263359
chr4	165238777	165263359
chr4	165263196	165297725
chr4	165263196	165299656
chr4	165294358	165299656
chr4	165305607	165310750
chr4	1654977	1657468
chr4	1654977	1668905
chr4	168220654	168237795
chr4	168415407	168416797
chr4	168471746	168472809
chr4	168890921	168894677
chr4	168890921	168916027
chr4	168894578	168898714
chr4	168898492	168903906
chr4	169002070	169007756
chr4	169006754	169007756
chr4	169106846	169122266
chr4	169106846	169136620
chr4	169116268	169136620
chr4	169130045	169136620
chr4	169155479	169156679
chr4	169424552	169433665
chr4	169424552	169438259
chr4	169463242	169508331
chr4	169463242	169556095
chr4	169477123	169477497
chr4	169477123	169479534
chr4	1694773	1700070
chr4	1694773	1703700
chr4	169507036	169508331
chr4	169537808	169556095
chr4	169555719	169556095
chr4	169576927	169599197
chr4	169576927	169602678
chr4	169580841	169590809

chr4	169580841	169599197
chr4	169580841	169602678
chr4	169585348	169590809
chr4	169585348	169599197
chr4	169590725	169602678
chr4	169602007	169602678
chr4	1696201	1700070
chr4	169687657	169690652
chr4	169741956	169753835
chr4	170088395	170089736
chr4	1723420	1728787
chr4	1727707	1731301
chr4	1730886	1731301
chr4	1730886	1735834
chr4	173292107	173304118
chr4	174259698	174263100
chr4	174297965	174316290
chr4	174299071	174310056
chr4	174308688	174310056
chr4	17501718	17504475
chr4	17501718	17509363
chr4	176111574	176115979
chr4	176111574	176120097
chr4	176148132	176151967
chr4	176149806	176151967
chr4	176711498	176729746
chr4	176727777	176729746
chr4	177339782	177351549
chr4	177339782	177353728
chr4	177339782	177360677
chr4	177351379	177353728
chr4	177353307	177360677
chr4	17812220	17815358
chr4	17812916	17825561
chr4	17814852	17815358
chr4	17814852	17818088
chr4	17814852	17825057
chr4	17814852	17831116
chr4	17822982	17831116
chr4	17823671	17828388
chr4	17909093	17963049
chr4	17909093	17972885
chr4	17961902	17963049
chr4	17961902	17972885
chr4	182894488	182915575

chr4	182914922	182915575
chr4	183193598	183209025
chr4	183207952	183209025
chr4	183240182	183249993
chr4	183240182	183253999
chr4	183249919	183253999
chr4	183268970	183289635
chr4	183285966	183289635
chr4	1836428	1841797
chr4	183663846	183668117
chr4	183674712	183686748
chr4	183683974	183685403
chr4	183683974	183686748
chr4	183683974	183693147
chr4	184001324	184011423
chr4	184418166	184419568
chr4	184631764	184635418
chr4	184631764	184638468
chr4	184652022	184659437
chr4	184659339	184666064
chr4	184659339	184678394
chr4	184659339	184685684
chr4	184661773	184678394
chr4	184661773	184691712
chr4	184665916	184678394
chr4	184672172	184682336
chr4	184685408	184685684
chr4	184710071	184725062
chr4	184716396	184717196
chr4	184780331	184803546
chr4	185247293	185259064
chr4	185247293	185264610
chr4	185247293	185267155
chr4	185247293	185288082
chr4	185247293	185310816
chr4	185247293	185323800
chr4	185258847	185267155
chr4	185264437	185267155
chr4	185288011	185310816
chr4	185310634	185346650
chr4	185320732	185332759
chr4	185339378	185342116
chr4	185339378	185346650
chr4	185357670	185362711
chr4	185361923	185362711

chr4	185523361	185525171
chr4	186628153	186628763
chr4	186633683	186639783
chr4	186636584	186709845
chr4	188099034	188105587
chr4	188101055	188105587
chr4	188104836	188105587
chr4	189952161	189960839
chr4	189955036	189960839
chr4	1900625	1904378
chr4	1900625	1918623
chr4	1900625	1930770
chr4	1900625	1935262
chr4	1900625	1939778
chr4	1900625	1961151
chr4	1904215	1918623
chr4	1904215	1935262
chr4	1916870	1918623
chr4	1916870	1935262
chr4	1918140	1930770
chr4	1918140	1935262
chr4	1957932	1961151
chr4	20467751	20491899
chr4	20467751	20519453
chr4	20491760	20539584
chr4	20510494	20524177
chr4	20528948	20533715
chr4	20528948	20539584
chr4	20549056	20598395
chr4	20704465	20704814
chr4	2156787	2179465
chr4	2170678	2179465
chr4	22389087	22392690
chr4	22389087	22402799
chr4	22389087	22413814
chr4	22401430	22402799
chr4	22413181	22421089
chr4	22435310	22473843
chr4	24537050	24556410
chr4	24541872	24556410
chr4	24554724	24556410
chr4	24554724	24570847
chr4	24570653	24576678
chr4	2470986	2500738
chr4	2490336	2497121

chr4	2490336	2500738
chr4	25127263	25159107
chr4	25144773	25159107
chr4	25151959	25159107
chr4	25154997	25159107
chr4	25252320	25263849
chr4	25255064	25260591
chr4	25255064	25263849
chr4	25260523	25269203
chr4	25314045	25351689
chr4	25333182	25333988
chr4	25345547	25351689
chr4	25383260	25391015
chr4	25765325	25767830
chr4	25802282	25835323
chr4	25818137	25847864
chr4	25832994	25835323
chr4	2630934	2639859
chr4	2630934	2646832
chr4	26362445	26415640
chr4	26386352	26424743
chr4	26406174	26415640
chr4	2646684	2659670
chr4	2655057	2689704
chr4	2657802	2660054
chr4	2657802	2663288
chr4	2657802	2672372
chr4	2657802	2690970
chr4	26613168	26640187
chr4	26613168	26666405
chr4	26620612	26640187
chr4	26620612	26666405
chr4	26620612	26673888
chr4	26620612	26748526
chr4	2662837	2663288
chr4	2662837	2672372
chr4	26659596	26673888
chr4	26666332	26688407
chr4	26666332	26748526
chr4	26683674	26720125
chr4	26717932	26748526
chr4	26717932	26753890
chr4	26919503	26957726
chr4	2867971	2884666
chr4	2875895	2884666

chr4	2894012	2894731
chr4	2904763	2908604
chr4	2949933	2951245
chr4	2949933	2953645
chr4	2949933	2954563
chr4	2952274	2953645
chr4	2952274	2956811
chr4	2953510	2956811
chr4	2953510	2957740
chr4	2954423	2956811
chr4	2956669	2957740
chr4	3007735	3013828
chr4	3019640	3022451
chr4	3086938	3099394
chr4	3086938	3107423
chr4	3086938	3116263
chr4	3099273	3107423
chr4	3103823	3107423
chr4	3103823	3116263
chr4	3125548	3131775
chr4	3127263	3131775
chr4	3127263	3140656
chr4	3130304	3131775
chr4	3140509	3148207
chr4	3142765	3148207
chr4	3154292	3160392
chr4	3186596	3189093
chr4	3186596	3199939
chr4	3235283	3238609
chr4	3316069	3343053
chr4	3422904	3428169
chr4	343781	344430
chr4	36107564	36121326
chr4	36107564	36124967
chr4	36107564	36151044
chr4	36124861	36151044
chr4	36177826	36193647
chr4	36187450	36229645
chr4	36210389	36214480
chr4	36210389	36229645
chr4	37631384	37635123
chr4	37631384	37638504
chr4	37631384	37649500
chr4	37834617	37840259
chr4	38014508	38027879

chr4	38014508	38035698
chr4	38020590	38027879
chr4	38027787	38035698
chr4	38027787	38052060
chr4	38035587	38045903
chr4	38035587	38049898
chr4	38035587	38054338
chr4	38054198	38096090
chr4	38054198	38103157
chr4	38089931	38103157
chr4	38089931	38118192
chr4	38095928	38103157
chr4	38905521	38905861
chr4	38931450	38935790
chr4	38932234	38935790
chr4	38986335	38993998
chr4	39075964	39081239
chr4	39075964	39086727
chr4	39075964	39096878
chr4	39081102	39086727
chr4	39081102	39115330
chr4	39081962	39086727
chr4	39113019	39115330
chr4	39215840	39218105
chr4	39224883	39232272
chr4	39224883	39240334
chr4	39228209	39228690
chr4	39253905	39257554
chr4	39267994	39270100
chr4	39300020	39303151
chr4	39302277	39303151
chr4	39302277	39304928
chr4	39304813	39309032
chr4	39320382	39323417
chr4	39326562	39327756
chr4	39467517	39473211
chr4	39508565	39514184
chr4	39737419	39755739
chr4	39737419	39774933
chr4	39737419	39777810
chr4	39745751	39774933
chr4	39745751	39777810
chr4	39755656	39777810
chr4	39774833	39777810
chr4	39837855	39842056

chr4	39837855	39848970
chr4	39841947	39844801
chr4	39841947	39845880
chr4	39841947	39849652
chr4	39845817	39849652
chr4	39848850	39849652
chr4	39862218	39904191
chr4	39866860	39869462
chr4	39866860	39873144
chr4	39869393	39890364
chr4	39872985	39877153
chr4	39872985	39890364
chr4	39872985	39925933
chr4	39874288	39877153
chr4	39874288	39890364
chr4	39874288	39902460
chr4	39879727	39890364
chr4	39890248	39902460
chr4	39890248	39925933
chr4	39898388	39902460
chr4	39898388	39920399
chr4	39898388	39925933
chr4	39902346	39917188
chr4	39902346	39922748
chr4	39902346	39925933
chr4	39904039	39922748
chr4	39904039	39925933
chr4	39904039	39976617
chr4	39910243	39920399
chr4	39910243	39925933
chr4	39913610	39920399
chr4	39913610	39922748
chr4	39913610	39925933
chr4	39913610	39928164
chr4	39917047	39925933
chr4	39922621	39928164
chr4	39922621	39976617
chr4	40106899	40118024
chr4	40126133	40137082
chr4	40821870	40945073
chr4	40890363	40893411
chr4	40890363	40935139
chr4	40890363	40945070
chr4	40890363	40945073
chr4	40890363	41143142

chr4	40934455	40945070
chr4	40934455	40945073
chr4	41013582	41033304
chr4	41013582	41100750
chr4	41100638	41143142
chr4	41598919	41650608
chr4	41603874	41619440
chr4	41605893	41619440
chr4	41605893	41650608
chr4	41613465	41619440
chr4	41661419	41671594
chr4	41661419	41692384
chr4	41666560	41676462
chr4	41666560	41692384
chr4	42001615	42022930
chr4	42001615	42023384
chr4	42001615	42039053
chr4	42001615	42078325
chr4	42018110	42023384
chr4	42018110	42035333
chr4	42018110	42039053
chr4	42018110	42049479
chr4	42020415	42023384
chr4	42020415	42039053
chr4	42022837	42039053
chr4	42062985	42078325
chr4	4237826	4246343
chr4	42522159	42556040
chr4	42616032	42627109
chr4	4279446	4281897
chr4	4299926	4315970
chr4	4306137	4315970
chr4	4313040	4315970
chr4	4315635	4315970
chr4	4434769	4471706
chr4	4457190	4471706
chr4	44680440	44686023
chr4	44710952	44722242
chr4	44717112	44722242
chr4	4648976	4649542
chr4	47535508	47536938
chr4	47535508	47546862
chr4	47744401	47786925
chr4	47744401	47807047
chr4	47855058	47875293

chr4	47855058	47910994
chr4	47862845	47885999
chr4	47875126	47879117
chr4	47875126	47885999
chr4	47875126	47894302
chr4	47875126	47914205
chr4	47879095	47898857
chr4	47884345	47890703
chr4	47884345	47894302
chr4	47884345	47896647
chr4	47884345	47914205
chr4	47894179	47905346
chr4	48171367	48176186
chr4	48369848	48379848
chr4	48369848	48383784
chr4	48377895	48383784
chr4	48383646	48420443
chr4	48505546	48515275
chr4	48556977	48562988
chr4	48562888	48567420
chr4	48590658	48605833
chr4	48590658	48609067
chr4	48593929	48609067
chr4	48595896	48609823
chr4	48602019	48609823
chr4	48606437	48609823
chr4	48619273	48634490
chr4	48684672	48710698
chr4	48832619	48848446
chr4	48833400	48857365
chr4	48851805	48857365
chr4	505717	507593
chr4	505717	508970
chr4	51848204	51899378
chr4	51863436	51877854
chr4	51863436	51886638
chr4	51863436	51891851
chr4	51874270	51877854
chr4	51874270	51891851
chr4	51874270	51899378
chr4	51886567	51913392
chr4	52626017	52631144
chr4	52627949	52631144
chr4	52907456	52920870
chr4	53379072	53391498

chr4	53379072	53399839
chr4	53379072	53414722
chr4	53379224	53391139
chr4	53382277	53383876
chr4	53383772	53391139
chr4	53383772	53391498
chr4	53383772	53399839
chr4	53383772	53428183
chr4	53383772	53444103
chr4	53383772	53453133
chr4	53399729	53428183
chr4	53399729	53444103
chr4	53414614	53428183
chr4	53414614	53444103
chr4	53414614	53453133
chr4	53425871	53428183
chr4	53425871	53444103
chr4	53425871	53453133
chr4	55403235	55411839
chr4	55411613	55417985
chr4	55417071	55417985
chr4	55448778	55450232
chr4	55453053	55456300
chr4	55458891	55459261
chr4	55470716	55482828
chr4	55470716	55489465
chr4	55470716	55510065
chr4	55475962	55482828
chr4	55475962	55489465
chr4	55475962	55510065
chr4	55478814	55510065
chr4	55486392	55510065
chr4	55877916	55890386
chr4	55888887	55892711
chr4	55891314	55893780
chr4	55952085	55954383
chr4	55959681	55965859
chr4	55959681	55985358
chr4	55964273	55971408
chr4	55999490	56024614
chr4	56011411	56011985
chr4	56017647	56019555
chr4	56017647	56024614
chr4	56298213	56316689
chr4	56353403	56355423

chr4	56378147	56384341
chr4	56399178	56406701
chr4	56403039	56406701
chr4	56451871	56453761
chr4	56474053	56474391
chr4	56478378	56484864
chr4	5663106	5665649
chr4	56910629	56919870
chr4	56973147	56974022
chr4	56990747	56999781
chr4	57010360	57030399
chr4	57015501	57025537
chr4	57023661	57030399
chr4	57024885	57025537
chr4	5729306	5741814
chr4	5729306	5756362
chr4	5835914	5843161
chr4	5843092	5851469
chr4	5861025	5866756
chr4	61934839	61998265
chr4	61934839	62044549
chr4	61946913	61998265
chr4	6347845	6381094
chr4	6609106	6611278
chr4	6610879	6611278
chr4	67518155	67519502
chr4	67530814	67544195
chr4	67663138	67665292
chr4	6804522	6824684
chr4	6804522	6842204
chr4	6824184	6842204
chr4	6842066	6858506
chr4	6923372	6967424
chr4	6994183	6999202
chr4	6994183	7001251
chr4	6994183	7014557
chr4	6996324	7004924
chr4	6999084	7004924
chr4	6999084	7014557
chr4	7004843	7014557
chr4	7004843	7025262
chr4	7006631	7014557
chr4	70762518	70764576
chr4	70762518	70768661
chr4	70762518	70778438

chr4	70762518	70784879
chr4	70762518	70789592
chr4	70764474	70778438
chr4	70783090	70784879
chr4	70824293	70826245
chr4	70824293	70828036
chr4	70827851	70833273
chr4	70831538	70832450
chr4	70831538	70833273
chr4	70832306	70836314
chr4	70950699	70959040
chr4	70950699	70975286
chr4	70958873	70970024
chr4	70958873	70979291
chr4	71236575	71255399
chr4	71339369	71357187
chr4	71532061	71534388
chr4	73064777	73065413
chr4	73076939	73092300
chr4	73078641	73092300
chr4	73090666	73102547
chr4	73097116	73102547
chr4	73102375	73113908
chr4	73118687	73125312
chr4	73120161	73125312
chr4	73120880	73151524
chr4	73124912	73125312
chr4	73141740	73177533
chr4	73151429	73177533
chr4	734930	744688
chr4	74174505	74175403
chr4	74174505	74201370
chr4	74174505	74225394
chr4	74174505	74281550
chr4	74199793	74201370
chr4	74199793	74225394
chr4	75515432	75516979
chr4	75646337	75648741
chr4	75646337	75657730
chr4	75648638	75655870
chr4	75655065	75662049
chr4	75656923	75662049
chr4	75658842	75662049
chr4	75757496	75782858
chr4	75770438	75771137

chr4	75790149	75790797
chr4	75804133	75805303
chr4	75806485	75812375
chr4	75925734	75936235
chr4	75957846	75961308
chr4	75960065	75965822
chr4	75964134	75982037
chr4	75967276	75971458
chr4	75967276	75974133
chr4	75969295	75971458
chr4	75981371	75982037
chr4	76124648	76144473
chr4	76130655	76136412
chr4	76130655	76144473
chr4	76131229	76132719
chr4	76134174	76144473
chr4	76144148	76144473
chr4	76730803	76741926
chr4	76995780	76996539
chr4	76995780	77020670
chr4	76995780	77030970
chr4	76996424	77030970
chr4	77005600	77020670
chr4	77019164	77020670
chr4	77055961	77056323
chr4	77055961	77066405
chr4	77056248	77066405
chr4	77058506	77066405
chr4	77158532	77164479
chr4	7772819	7778876
chr4	77742140	77776392
chr4	77756861	77776392
chr4	77773080	77776392
chr4	7786193	7800653
chr4	7786193	7843350
chr4	77883241	77909372
chr4	7793680	7800653
chr4	7793680	7809763
chr4	7800441	7809763
chr4	7809613	7819171
chr4	7809613	7843350
chr4	7816017	7838703
chr4	7838523	7843350
chr4	7838523	7855574
chr4	7855465	7872080

chr4	7868621	7872080
chr4	78826036	78833687
chr4	78826036	78851056
chr4	78826036	78878891
chr4	78842384	78851056
chr4	78870782	78878891
chr4	78871849	78878891
chr4	78878733	78887284
chr4	79977620	79984863
chr4	80008520	80036032
chr4	80008520	80069507
chr4	82355303	82356895
chr4	82355303	82359639
chr4	82356536	82356895
chr4	82358658	82359639
chr4	82358658	82371584
chr4	82426036	82428446
chr4	82428017	82428446
chr4	82447919	82451245
chr4	82447919	82454854
chr4	82454721	82457038
chr4	82680706	82705413
chr4	82844385	82848977
chr4	82848803	82861708
chr4	82848803	82881940
chr4	82851430	82867316
chr4	82854902	82861708
chr4	82866807	82867316
chr4	82870324	82881940
chr4	82871943	82875822
chr4	82874610	82875822
chr4	82874610	82881940
chr4	82878729	82880922
chr4	82878729	82881937
chr4	82878729	82881940
chr4	82930942	82979006
chr4	82946257	82970469
chr4	82946257	82979006
chr4	82970326	82979006
chr4	82978882	82984876
chr4	83049165	83057408
chr4	83056925	83063246
chr4	83267585	83279114
chr4	83272086	83279114
chr4	83302149	83313287

chr4	83416730	83418206
chr4	83427562	83432267
chr4	83439862	83446086
chr4	83463493	83467538
chr4	83463493	83469151
chr4	83469031	83472288
chr4	83470202	83482244
chr4	83581561	83590292
chr4	8451959	8452989
chr4	84678167	84679242
chr4	84683942	84696823
chr4	84695969	84705511
chr4	84695969	84709347
chr4	84702352	84709347
chr4	84702352	84715383
chr4	84702352	84717016
chr4	84702352	84721572
chr4	84704337	84709347
chr4	84708908	84717016
chr4	84718421	84740416
chr4	84739009	84740416
chr4	84766251	84774981
chr4	84774819	84794979
chr4	84780107	84783074
chr4	84796520	84801864
chr4	84829003	84841263
chr4	84829003	84897010
chr4	84849901	84860622
chr4	84860411	84897010
chr4	84860411	84932363
chr4	84896910	84932363
chr4	865121	884086
chr4	86672364	86693674
chr4	86672364	86701801
chr4	86686709	86689190
chr4	86686709	86693674
chr4	86686709	86701801
chr4	86693586	86701801
chr4	86693586	86717117
chr4	86764592	86767976
chr4	86772777	86775652
chr4	86780401	86785936
chr4	87046165	87047594
chr4	870710	883463
chr4	870710	884086

chr4	87084119	87091829
chr4	87084119	87094969
chr4	87170078	87176868
chr4	87170078	87195690
chr4	87183202	87195690
chr4	87434980	87454455
chr4	87438276	87454455
chr4	87441828	87454455
chr4	88019457	88043457
chr4	88036219	88065879
chr4	88038250	88052158
chr4	88038250	88056267
chr4	88051990	88065879
chr4	88051990	88068061
chr4	88061905	88065879
chr4	88131060	88140014
chr4	88383220	88398209
chr4	88390651	88393582
chr4	88423859	88431305
chr4	88472408	88476030
chr4	88475840	88479507
chr4	88475840	88494331
chr4	88649839	88654133
chr4	88767552	88851183
chr4	88781164	88805052
chr4	888846	904779
chr4	88906378	88938241
chr4	88906378	89029649
chr4	890531	904779
chr4	893376	904779
chr4	904636	913668
chr4	911672	913668
chr4	94226118	94237018
chr4	94226118	94241006
chr4	94226118	94253007
chr4	94236951	94249755
chr4	94276338	94283303
chr4	94523723	94576034
chr4	94573350	94586444
chr4	94618003	94640450
chr4	94640275	94657547
chr4	947708	953354
chr4	97981158	98134459
chr4	98105952	98109305
chr4	98105952	98134459

chr4	98109192	98134459
chr4	98128427	98134459
chr4	98352475	98379163
chr4	98404476	98421394
chr4	98416744	98418791
chr4	98416744	98420144
chr4	98416744	98437068
chr4	98887078	98891332
chr4	99028866	99048876
chr4	99041042	99045310
chr5	10258110	10263314
chr5	102865822	102867393
chr5	102913933	102961229
chr5	102946836	102974436
chr5	102948377	102950820
chr5	102990271	103009866
chr5	103003032	103009866
chr5	103096540	103097781
chr5	103136731	103146681
chr5	103136731	103158573
chr5	103138383	103149313
chr5	1032151	1036384
chr5	10377797	10394785
chr5	10381799	10391731
chr5	10381799	10394785
chr5	10386993	10391731
chr5	10386993	10394785
chr5	10402383	10407202
chr5	10405557	10417404
chr5	10414432	10417404
chr5	10415487	10417404
chr5	1057470	1065478
chr5	1063843	1065478
chr5	108020924	108186247
chr5	108020924	108224228
chr5	108020924	108348530
chr5	108186116	108348530
chr5	108186116	108367953
chr5	108224120	108348530
chr5	108348398	108367953
chr5	108768092	108798389
chr5	108768092	108835807
chr5	108768092	108959347
chr5	108782662	108798389
chr5	108798123	108835807

chr5	108798123	108883518
chr5	108798123	108946222
chr5	108798123	109100519
chr5	108832769	108835807
chr5	108867766	108872212
chr5	108867766	108883518
chr5	108867766	108897848
chr5	108883395	108897848
chr5	108883395	108946222
chr5	108883395	108959347
chr5	108883395	109100519
chr5	108897658	108959347
chr5	108897658	109047198
chr5	108946129	108959347
chr5	108946129	109100519
chr5	108954728	108959347
chr5	108959224	109100519
chr5	109037421	109047198
chr5	109037421	109100519
chr5	109344189	109356026
chr5	109362839	109383520
chr5	109713519	109716264
chr5	109713519	109729513
chr5	109713519	109755456
chr5	109713519	109774965
chr5	109713519	109789527
chr5	109729341	109755456
chr5	109729341	109774965
chr5	109755328	109774965
chr5	109767534	109774965
chr5	109767534	109789527
chr5	109781395	109789527
chr5	109781395	109823837
chr5	109784743	109823837
chr5	109817272	109823837
chr5	109817272	109847790
chr5	109842327	109847790
chr5	109842327	109855334
chr5	110618428	110627880
chr5	110742046	110748263
chr5	110742046	110756759
chr5	111097078	111102399
chr5	111098721	111111278
chr5	111110188	111113153
chr5	111344023	111394782

chr5	111374849	111394782
chr5	112194567	112205504
chr5	112234633	112240810
chr5	112234633	112266330
chr5	112259228	112266330
chr5	112259228	112280323
chr5	112259228	112307490
chr5	112259890	112266330
chr5	112262493	112280323
chr5	112264895	112280323
chr5	112275325	112307490
chr5	112754872	112767390
chr5	112754872	112775737
chr5	112766325	112767390
chr5	112766325	112780903
chr5	112766325	112792529
chr5	112766325	112801383
chr5	112767188	112792529
chr5	112775628	112780903
chr5	112775628	112792529
chr5	112780789	112801383
chr5	112792445	112828006
chr5	112792445	112828972
chr5	112818965	112828972
chr5	112821895	112828006
chr5	112985834	112992248
chr5	112985834	113001674
chr5	112985834	113004077
chr5	113001083	113001674
chr5	113001083	113004077
chr5	113001083	113008042
chr5	113003941	113010807
chr5	113063983	113085310
chr5	113082859	113122826
chr5	113524980	113526785
chr5	113524980	113539181
chr5	113524980	113549020
chr5	113532878	113535798
chr5	113532878	113542503
chr5	113534304	113539181
chr5	113534304	113542503
chr5	113539073	113542503
chr5	113553766	113563492
chr5	113553766	113579695
chr5	113556051	113563492

chr5	113556051	113579695
chr5	113579585	113592178
chr5	115130591	115134147
chr5	115130591	115137616
chr5	115130591	115141374
chr5	115137362	115147394
chr5	115141278	115163752
chr5	115216864	115238009
chr5	115216864	115241606
chr5	115221823	115241606
chr5	115230975	115238009
chr5	115230975	115253255
chr5	115236389	115238009
chr5	115236389	115253255
chr5	115866669	115870128
chr5	115866669	115895158
chr5	116087496	116092700
chr5	116087496	116134178
chr5	116091078	116092700
chr5	116091078	116134178
chr5	118928536	118944649
chr5	119097978	119102006
chr5	119097978	119116336
chr5	119097978	119119004
chr5	119101934	119110283
chr5	119101934	119114541
chr5	119101934	119116336
chr5	119105179	119114541
chr5	119105179	119121139
chr5	119110150	119114541
chr5	119110150	119116336
chr5	119110150	119119004
chr5	119164506	119216987
chr5	119175260	119178244
chr5	119189707	119203436
chr5	119220471	119239080
chr5	119240418	119244576
chr5	119473907	119496646
chr5	119477416	119496646
chr5	119506817	119515046
chr5	119525215	119536550
chr5	121974195	121975387
chr5	121974195	121994670
chr5	121994598	122022407
chr5	122019259	122022407

chr5	122795265	122803613
chr5	122795265	122808355
chr5	122795265	122819023
chr5	122799691	122801935
chr5	122799691	122808355
chr5	122801868	122803613
chr5	122801868	122819023
chr5	122936733	122946159
chr5	122936733	123002004
chr5	123364495	123399284
chr5	123399135	123423030
chr5	123412398	123418515
chr5	123416009	123418515
chr5	123545416	123553147
chr5	123545416	123557564
chr5	123557494	123604830
chr5	123575728	123588153
chr5	123588067	123591414
chr5	123590412	123591414
chr5	1255286	1260600
chr5	1258597	1268633
chr5	126465425	126473368
chr5	126469676	126477787
chr5	126477691	126484524
chr5	126546323	126552137
chr5	126552020	126570859
chr5	126554286	126556015
chr5	126559239	126570859
chr5	126561082	126577211
chr5	126568258	126570859
chr5	126568258	126577211
chr5	126568258	126584012
chr5	126570781	126577211
chr5	126575419	126584012
chr5	126575419	126593404
chr5	126577078	126593404
chr5	126582850	126584012
chr5	126582850	126593404
chr5	1266463	1282624
chr5	126804775	126811898
chr5	126804775	126826107
chr5	126804775	126832801
chr5	126811772	126826107
chr5	126818921	126826107
chr5	126818921	126832801

chr5	126820909	126826107
chr5	126878184	126918227
chr5	126914929	126918227
chr5	127523459	127524920
chr5	127523459	127526778
chr5	127526617	127539143
chr5	127526617	127547921
chr5	1278640	1282624
chr5	128112813	128114681
chr5	128112813	128141981
chr5	128112813	128161800
chr5	128114585	128131206
chr5	128131066	128135808
chr5	128131066	128141981
chr5	128131066	128152805
chr5	128131066	128161800
chr5	128138596	128152805
chr5	128141829	128167867
chr5	128148753	128158164
chr5	128174540	128182941
chr5	128302972	128311953
chr5	128446480	128530693
chr5	128464723	128519368
chr5	128464723	128527967
chr5	128519272	128530693
chr5	128985132	128990464
chr5	129104955	129107062
chr5	131180094	131187109
chr5	131385542	131391108
chr5	131430858	131433658
chr5	131439580	131472744
chr5	131453053	131456012
chr5	131504625	131508207
chr5	131504625	131548190
chr5	131521389	131548190
chr5	131521389	131562047
chr5	131548046	131592466
chr5	131548046	131604693
chr5	131647089	131651999
chr5	131670462	131679175
chr5	131677702	131699002
chr5	131677702	131709272
chr5	131677702	131719060
chr5	131677702	131744690
chr5	131698916	131709272

chr5	131698916	131719060
chr5	131704064	131731038
chr5	131710577	131719060
chr5	132217196	132218644
chr5	132559283	132580066
chr5	132591223	132595810
chr5	132699170	132711057
chr5	132710958	132716984
chr5	132715756	132734478
chr5	132720608	132734478
chr5	132726127	132734478
chr5	132760801	132762645
chr5	132883339	132885119
chr5	132892163	132893118
chr5	132892163	132897240
chr5	132899103	132904404
chr5	132927120	132934941
chr5	132927120	132937193
chr5	133006758	133022906
chr5	133064979	133070496
chr5	133064979	133074126
chr5	133064979	133076898
chr5	133067416	133076898
chr5	133070373	133074126
chr5	133073229	133074126
chr5	133073229	133076898
chr5	133086781	133089695
chr5	133091192	133092789
chr5	133091192	133099652
chr5	133990854	133991154
chr5	133990854	133993015
chr5	133990854	133993018
chr5	134158454	134174022
chr5	134199085	134202021
chr5	134199085	134206131
chr5	134319357	134321903
chr5	134350248	134360091
chr5	134374382	134388413
chr5	134535857	134538083
chr5	134535857	134552209
chr5	134535857	134564610
chr5	134608371	134609674
chr5	134666822	134671886
chr5	134674614	134676125
chr5	134674614	134682482

chr5	134674614	134697246
chr5	134679601	134682482
chr5	134679601	134688299
chr5	134682372	134688299
chr5	134682372	134698057
chr5	134692601	134693933
chr5	134692601	134708888
chr5	134697125	134698057
chr5	134697125	134703932
chr5	134697125	134705437
chr5	134697898	134703932
chr5	134697898	134705437
chr5	134697898	134708888
chr5	134703758	134718173
chr5	134703758	134721090
chr5	134708712	134718173
chr5	134708712	134723670
chr5	134741062	134744052
chr5	134763903	134767060
chr5	134763903	134770999
chr5	134766916	134770999
chr5	134777573	134783065
chr5	134781132	134782086
chr5	134781132	134785586
chr5	134788512	134796150
chr5	134790105	134796150
chr5	134790105	134807941
chr5	134790469	134796150
chr5	134790469	134811358
chr5	134790469	134819004
chr5	134811220	134817714
chr5	134816429	134817714
chr5	134816429	134819004
chr5	134817495	134819004
chr5	134893491	134896424
chr5	135343259	135353045
chr5	135343259	135370142
chr5	135360496	135370142
chr5	135360496	135389126
chr5	135369405	135370142
chr5	135369405	135389126
chr5	136147831	136154163
chr5	136147831	136163391
chr5	136147831	136174632
chr5	136153591	136163391

chr5	136160855	136174632
chr5	137658130	137698408
chr5	137692284	137720584
chr5	137941943	137943216
chr5	137942874	137943216
chr5	137942874	137949184
chr5	137948954	137962469
chr5	137952627	137954376
chr5	137959615	137962469
chr5	137959615	138019146
chr5	137985256	137988315
chr5	137985256	138011149
chr5	137985256	138018514
chr5	137985256	138019146
chr5	137985256	138021197
chr5	138006989	138011149
chr5	138006989	138011945
chr5	138006989	138018514
chr5	138006989	138019146
chr5	138006989	138021197
chr5	138011007	138011945
chr5	138011007	138021197
chr5	138018301	138021197
chr5	138159554	138166727
chr5	138161795	138164133
chr5	138169221	138177667
chr5	138189632	138202155
chr5	138191473	138192657
chr5	138191473	138201448
chr5	138191473	138202155
chr5	138198198	138202155
chr5	138286021	138326054
chr5	138289500	138292116
chr5	138319218	138328529
chr5	138319218	138329647
chr5	138325814	138329647
chr5	138372673	138375206
chr5	138372673	138377825
chr5	138372673	138379708
chr5	138372673	138381590
chr5	138372673	138386621
chr5	138372673	138400012
chr5	138375092	138381590
chr5	138377719	138381590
chr5	138381515	138386621

chr5	138386021	138400012
chr5	138386021	138420962
chr5	138398177	138400012
chr5	138420705	138425582
chr5	138424074	138425582
chr5	138424074	138430425
chr5	138425410	138428086
chr5	138429825	138431559
chr5	138508668	138513706
chr5	138510564	138512954
chr5	138512763	138513706
chr5	138513567	138517700
chr5	138513567	138518867
chr5	138513567	138529678
chr5	138517560	138518867
chr5	138517560	138529678
chr5	138518691	138529678
chr5	138557401	138561789
chr5	138557401	138567722
chr5	138557401	138574126
chr5	138558552	138560091
chr5	138559863	138561789
chr5	138559863	138567722
chr5	138561579	138567561
chr5	138561579	138567722
chr5	138566625	138567722
chr5	138566625	138569049
chr5	138567000	138573850
chr5	138568924	138571141
chr5	138568924	138573850
chr5	138568924	138574126
chr5	138781922	138783372
chr5	138781922	138812302
chr5	138781922	138827718
chr5	138783176	138827718
chr5	138810037	138812302
chr5	138810037	138827718
chr5	138810037	138887642
chr5	138824529	138827718
chr5	138824529	138904441
chr5	138824529	138917898
chr5	138886211	138904441
chr5	138886211	138932712
chr5	138951170	138951884
chr5	139021170	139026992

chr5	139026800	139051046
chr5	13911385	13931244
chr5	139121034	139127853
chr5	13914519	13931244
chr5	139276120	139279129
chr5	139278326	139279129
chr5	139314674	139319033
chr5	139317052	139319033
chr5	139317052	139319501
chr5	139317052	139322967
chr5	139318907	139319501
chr5	139321897	139326284
chr5	139363758	139364743
chr5	139600371	139614966
chr5	139600371	139623461
chr5	139614585	139614966
chr5	139614585	139623461
chr5	139614696	139614966
chr5	139614696	139623461
chr5	139865548	139887511
chr5	139871720	139880974
chr5	139880855	139887511
chr5	140281448	140300582
chr5	140436103	140459355
chr5	140438460	140445975
chr5	140438460	140449305
chr5	140438460	140487060
chr5	140440118	140445975
chr5	140440118	140449305
chr5	140445741	140459355
chr5	140449210	140464776
chr5	140458624	140464776
chr5	140482579	140487060
chr5	140485120	140510181
chr5	140485588	140506977
chr5	140504820	140505869
chr5	140504820	140506977
chr5	140504820	140509812
chr5	140506834	140513479
chr5	140507784	140513479
chr5	140509636	140510181
chr5	140563593	140563915
chr5	141534334	141579196
chr5	141576229	141583615
chr5	141580743	141583615

chr5	141582311	141583615
chr5	141638794	141640445
chr5	141928150	141930274
chr5	141933258	141934586
chr5	141973582	141983552
chr5	142131807	142135817
chr5	142131807	142140629
chr5	142131807	142144676
chr5	142135729	142140629
chr5	142135729	142144676
chr5	142345415	142353583
chr5	14270824	14280436
chr5	14270824	14287063
chr5	14270824	14297263
chr5	14270824	14304592
chr5	14270824	14316743
chr5	14270824	14330900
chr5	14270824	14336727
chr5	14270824	14401064
chr5	14280321	14304592
chr5	14280321	14336727
chr5	14286870	14304592
chr5	14286870	14316743
chr5	14286870	14336727
chr5	142873399	142879445
chr5	142873399	142885399
chr5	142879373	142907804
chr5	142879373	142913293
chr5	142885297	142913293
chr5	142885297	142932125
chr5	142885297	143014116
chr5	142885297	143057747
chr5	142901934	142907804
chr5	142901934	142932125
chr5	14290715	14297263
chr5	14290715	14304592
chr5	14290715	14316743
chr5	14290715	14330900
chr5	14290715	14336727
chr5	14290715	14359531
chr5	14290715	14374343
chr5	14293011	14304592
chr5	14293011	14316743
chr5	14293011	14336727
chr5	14297071	14304592

chr5	14297071	14316743
chr5	14297071	14336727
chr5	143014079	143057747
chr5	143037195	143057747
chr5	14304460	14316743
chr5	14304460	14330900
chr5	14304460	14336727
chr5	143054438	143057747
chr5	14316512	14330900
chr5	14316512	14336727
chr5	14316512	14381252
chr5	1432463	1443242
chr5	143295459	143300763
chr5	143295459	143314168
chr5	14330777	14336727
chr5	143310096	143314168
chr5	143310096	143400852
chr5	14358177	14359531
chr5	14358177	14374343
chr5	14358177	14381252
chr5	14358177	14401064
chr5	14363731	14378127
chr5	14363731	14381252
chr5	14374228	14381252
chr5	14389288	14472658
chr5	14398879	14406672
chr5	14405847	14406672
chr5	14405847	14420021
chr5	14476893	14502657
chr5	14496878	14498640
chr5	14498088	14502657
chr5	145764930	145796547
chr5	145764930	145820018
chr5	145764930	145823134
chr5	145796441	145823134
chr5	145796441	145826200
chr5	145817893	145823134
chr5	14600964	14602332
chr5	146120370	146124086
chr5	146120370	146126545
chr5	146128671	146143084
chr5	146131018	146144349
chr5	146132897	146143084
chr5	146132897	146144349
chr5	146140203	146144709

chr5	146142871	146144709
chr5	146144266	146144709
chr5	146149621	146164471
chr5	146160373	146164471
chr5	146160373	146171990
chr5	146160373	146172774
chr5	146164309	146168265
chr5	146164309	146172774
chr5	146164309	146177665
chr5	146168127	146171990
chr5	146218984	146223527
chr5	146218984	146229910
chr5	146223402	146233743
chr5	146223402	146237432
chr5	146233449	146258593
chr5	146233449	146271058
chr5	146237297	146251875
chr5	146251710	146255092
chr5	146254942	146258593
chr5	146254942	146260898
chr5	146260744	146263631
chr5	146263490	146271058
chr5	146483539	146493038
chr5	146483539	146503539
chr5	146503374	146504006
chr5	14673641	14681607
chr5	14678680	14681607
chr5	1470825	1489858
chr5	14741826	14751239
chr5	14741826	14758598
chr5	14745869	14758598
chr5	1477403	1489858
chr5	1480941	1501603
chr5	148394713	148402513
chr5	148394713	148404822
chr5	148394713	148410765
chr5	148394713	148417204
chr5	148394713	148425701
chr5	148401981	148416070
chr5	148406256	148410765
chr5	148433423	148440527
chr5	148548667	148550262
chr5	1488390	1501603
chr5	149210725	149233300
chr5	1494699	1501603

chr5	149505446	149513209
chr5	149505446	149550181
chr5	149505482	149550181
chr5	149507026	149513209
chr5	149509878	149513209
chr5	149511793	149520388
chr5	149517793	149525171
chr5	149820432	149826885
chr5	149836262	149842377
chr5	149836262	149845914
chr5	149899374	149915007
chr5	150006472	150018697
chr5	150361155	150367917
chr5	150367843	150369602
chr5	150367843	150372236
chr5	150379228	150390023
chr5	150387901	150390023
chr5	150445608	150447735
chr5	150532944	150535885
chr5	150539227	150540264
chr5	150541569	150542971
chr5	150541569	150545486
chr5	150541569	150549787
chr5	150695505	150699271
chr5	150730630	150733475
chr5	151042539	151052259
chr5	151049823	151063747
chr5	151060317	151063747
chr5	151060317	151065131
chr5	151198996	151205475
chr5	151458787	151467925
chr5	151458787	151479489
chr5	151786571	151791062
chr5	151786571	151795575
chr5	151786571	151797428
chr5	151786571	151800869
chr5	151786602	151791062
chr5	151786602	151795575
chr5	151786602	151800869
chr5	151790322	151791062
chr5	151790322	151794249
chr5	151790322	151795575
chr5	151790322	151799313
chr5	151794158	151799313
chr5	151797226	151799313

chr5	151799211	151800869
chr5	151799888	151800869
chr5	153997802	154002969
chr5	153997802	154034967
chr5	154002846	154027334
chr5	154011240	154034967
chr5	154026398	154034967
chr5	154029488	154034967
chr5	154033790	154034967
chr5	154294815	154298079
chr5	154294815	154329738
chr5	154294815	154386430
chr5	154329571	154380631
chr5	154790324	154792796
chr5	154790324	154795319
chr5	154790324	154799759
chr5	154790324	154800042
chr5	154790324	154811640
chr5	154790644	154793723
chr5	154790644	154811640
chr5	154794099	154811640
chr5	154865191	154872651
chr5	154871729	154872651
chr5	154901338	154913038
chr5	154911726	154913038
chr5	154911726	154928659
chr5	154916997	154926074
chr5	154919966	154937185
chr5	154924468	154928659
chr5	154924468	154936022
chr5	154927384	154932250
chr5	154950820	154960049
chr5	157285338	157287108
chr5	157285338	157296774
chr5	157294782	157296774
chr5	157294782	157304366
chr5	157294782	157311781
chr5	157359004	157361598
chr5	157488264	157489186
chr5	157492972	157494795
chr5	157492972	157499662
chr5	157509300	157513505
chr5	157518822	157520031
chr5	157518822	157537991
chr5	157791702	157794972

chr5	157791702	157803719
chr5	157794897	157806112
chr5	157803649	157817547
chr5	157805865	157817547
chr5	159181959	159203656
chr5	159194715	159203656
chr5	159268869	159275246
chr5	159268869	159278354
chr5	159272022	159275246
chr5	160010499	160036803
chr5	160010499	160043169
chr5	160010499	160049662
chr5	160035139	160036803
chr5	160035139	160043169
chr5	160035139	160049662
chr5	160035139	160051183
chr5	160080650	160094065
chr5	160276268	160280738
chr5	160422301	160422893
chr5	160422301	160424330
chr5	160422301	160427873
chr5	162093827	162104026
chr5	163469640	163471463
chr5	163471184	163471463
chr5	163513554	163517674
chr5	16670525	16675150
chr5	16673681	16680104
chr5	16676030	16689919
chr5	16700962	16702588
chr5	16754827	16761546
chr5	16758117	16763755
chr5	16761463	16762637
chr5	16762044	16762637
chr5	16763480	16764396
chr5	16763480	16766198
chr5	16779544	16780608
chr5	16779544	16781829
chr5	16779544	16783469
chr5	16779544	16794833
chr5	16794645	16818167
chr5	168371423	168399567
chr5	168397723	168409995
chr5	168399487	168406327
chr5	168422007	168431444
chr5	168428032	168431444

chr5	168441681	168460742
chr5	168453967	168460742
chr5	168488601	168492847
chr5	168488601	168494650
chr5	168488601	168495436
chr5	168488601	168497348
chr5	168488601	168500720
chr5	168493893	168494650
chr5	168493893	168495436
chr5	168493893	168497348
chr5	168493893	168502105
chr5	168493893	168518062
chr5	168495314	168518062
chr5	168500590	168502105
chr5	168500590	168510686
chr5	168500590	168516950
chr5	168500590	168518062
chr5	168506020	168510686
chr5	168506020	168516950
chr5	168510580	168516950
chr5	168559031	168564065
chr5	168559031	168566266
chr5	168559031	168568998
chr5	168561392	168566266
chr5	168566012	168568998
chr5	169591047	169594681
chr5	170878096	170881896
chr5	170878096	170892553
chr5	170878096	170896115
chr5	170878096	170924550
chr5	170881805	170896115
chr5	170892386	170896115
chr5	170892386	170924550
chr5	170896049	170924550
chr5	170909660	170924550
chr5	170909660	170968377
chr5	170916464	170953702
chr5	170918712	170953702
chr5	170918712	170968377
chr5	170919440	170968377
chr5	171170129	171213738
chr5	171183166	171205612
chr5	171183321	171213738
chr5	171183321	171221840
chr5	171183321	171242820

chr5	171199669	171221840
chr5	171205523	171221840
chr5	171221757	171242820
chr5	171240927	171242820
chr5	171390050	171391799
chr5	171390050	171392978
chr5	171391304	171392978
chr5	171436092	171449253
chr5	171868609	171900100
chr5	171868609	171914405
chr5	171876284	171878129
chr5	171876284	171900100
chr5	171891466	171900100
chr5	171891466	171910797
chr5	171891466	171914405
chr5	171910571	171914405
chr5	172055587	172057473
chr5	172117480	172156788
chr5	172346135	172347332
chr5	172350362	172373815
chr5	172358772	172362869
chr5	172358772	172382127
chr5	172362734	172382127
chr5	172382035	172406352
chr5	172897001	172914838
chr5	172909666	172914838
chr5	172924004	172935310
chr5	172932435	172935310
chr5	172959864	172968599
chr5	173080584	173086626
chr5	173080584	173091401
chr5	173080584	173112379
chr5	173108623	173110711
chr5	173108623	173112379
chr5	173944966	173953272
chr5	175492094	175512196
chr5	175509031	175512196
chr5	175964950	175967267
chr5	176289653	176296320
chr5	176322272	176324757
chr5	176336719	176337146
chr5	176350612	176355749
chr5	176352621	176355749
chr5	176355570	176359567
chr5	176479187	176489027

chr5	176486354	176494275
chr5	176498913	176500146
chr5	176908522	176958154
chr5	176943334	176951577
chr5	176943334	176958154
chr5	176943334	176969706
chr5	176943334	176970866
chr5	176943334	176982623
chr5	176951473	176975480
chr5	176951473	176982623
chr5	176955958	176970866
chr5	176955958	176982623
chr5	176968554	176970866
chr5	176970741	176982623
chr5	176975395	176982623
chr5	177041125	177044533
chr5	177041125	177050936
chr5	177135086	177192019
chr5	177135086	177204292
chr5	177135940	177192019
chr5	177191883	177204292
chr5	177191883	177212195
chr5	177191883	177239865
chr5	177235820	177239865
chr5	177235820	177244270
chr5	177238236	177239865
chr5	177244194	177246796
chr5	177244194	177248324
chr5	177248180	177260168
chr5	177251729	177257151
chr5	177256950	177288925
chr5	177269601	177283928
chr5	177280564	177283928
chr5	177280564	177288925
chr5	177288818	177292158
chr5	177334283	177338605
chr5	177626435	177632738
chr5	178147532	178147883
chr5	178211889	178231523
chr5	178222354	178222934
chr5	178222354	178224729
chr5	178222354	178225429
chr5	178860996	178867071
chr5	178912583	178913071
chr5	179024969	179028127

chr5	179152141	179158879
chr5	179560024	179562664
chr5	179560024	179569425
chr5	179560024	179577136
chr5	179562546	179569425
chr5	179562546	179577136
chr5	179562546	179585865
chr5	179567460	179569425
chr5	179567460	179577136
chr5	179580946	179589647
chr5	179580946	179593645
chr5	179580946	179594963
chr5	179585795	179596681
chr5	179591624	179593645
chr5	179591624	179594963
chr5	179591624	179601986
chr5	179594865	179596681
chr5	179615545	179616958
chr5	179616125	179616958
chr5	179617513	179621397
chr5	179617798	179621397
chr5	179617988	179618323
chr5	179620891	179623164
chr5	179705678	179706331
chr5	179705678	179707190
chr5	179705678	179708380
chr5	179705678	179709059
chr5	179705678	179710065
chr5	179705678	179716294
chr5	179705678	179723019
chr5	179705678	179724783
chr5	179705678	179726759
chr5	179706257	179716294
chr5	179707131	179709059
chr5	179707131	179710065
chr5	179707131	179720560
chr5	179708238	179709059
chr5	179708238	179710065
chr5	179709872	179716294
chr5	179709872	179719781
chr5	179709872	179720560
chr5	179709872	179724783
chr5	179719667	179720560
chr5	179719667	179724783
chr5	179765325	179769089

chr5	179765325	179771243
chr5	179768849	179771243
chr5	179798512	179799121
chr5	179822957	179824323
chr5	179822957	179825226
chr5	179822957	179833782
chr5	179823857	179825226
chr5	179824181	179833782
chr5	179833031	179833782
chr5	179840871	179853437
chr5	179841906	179844227
chr5	179841906	179853437
chr5	179869768	179878523
chr5	179872891	179904812
chr5	179888102	179893467
chr5	179888102	179894614
chr5	179888102	179899307
chr5	179891378	179894614
chr5	179966805	179970506
chr5	179966805	179980200
chr5	179978202	180040647
chr5	180238331	180241155
chr5	180248972	180269409
chr5	180248972	180280608
chr5	180261683	180269409
chr5	180261683	180280608
chr5	180264780	180280608
chr5	180269279	180280608
chr5	180302422	180312544
chr5	180316340	180317058
chr5	180316340	180335953
chr5	180316340	180336577
chr5	180495420	180529388
chr5	180529274	180550117
chr5	180529274	180553471
chr5	180549930	180553471
chr5	180553385	180571432
chr5	180569109	180571432
chr5	181237608	181239582
chr5	181238098	181241639
chr5	181238098	181242345
chr5	181239066	181242345
chr5	181239486	181241639
chr5	181241491	181242345
chr5	21491320	21495182

chr5	223481	228333
chr5	223481	240476
chr5	230875	251468
chr5	250991	251468
chr5	31406852	31410887
chr5	31421271	31424471
chr5	31421271	31451640
chr5	31429474	31451640
chr5	31435764	31451640
chr5	31437238	31468063
chr5	31448546	31451640
chr5	31448546	31472232
chr5	31448546	31486562
chr5	31448546	31495372
chr5	31483553	31486562
chr5	31483553	31495372
chr5	31504554	31521215
chr5	31535743	31545712
chr5	32135571	32143880
chr5	32263112	32268794
chr5	32268700	32276742
chr5	32379114	32390437
chr5	32379114	32420103
chr5	32390274	32397338
chr5	32397218	32404097
chr5	32414968	32417792
chr5	32588511	32599069
chr5	33448540	33453412
chr5	33454944	33457403
chr5	33460901	33463825
chr5	33576053	33596060
chr5	33648821	33662040
chr5	33998640	34005899
chr5	34795938	34814669
chr5	34808583	34811945
chr5	34813573	34821850
chr5	34918363	34923234
chr5	34919839	34923234
chr5	34922216	34923234
chr5	34938857	34941183
chr5	353729	376716
chr5	353729	413433
chr5	36136308	36143406
chr5	36207169	36227565
chr5	36211843	36227565

chr5	36225541	36227565
chr5	36953617	36958231
chr5	36953617	36961583
chr5	36953617	36962274
chr5	36953617	36972041
chr5	36953617	36976402
chr5	36970875	36976402
chr5	36984675	36995804
chr5	37010086	37022149
chr5	37044346	37052566
chr5	37114959	37120340
chr5	37114959	37121784
chr5	37114959	37122488
chr5	37114959	37125409
chr5	37120215	37121784
chr5	37120215	37122488
chr5	37120215	37125409
chr5	37120215	37162566
chr5	37153739	37162566
chr5	37153739	37165671
chr5	37164272	37167213
chr5	37165538	37169561
chr5	37170040	37175986
chr5	37173754	37179443
chr5	37179360	37181005
chr5	37187413	37187842
chr5	37187413	37201808
chr5	37195857	37201808
chr5	37198701	37201808
chr5	37213558	37224740
chr5	37213558	37239869
chr5	37221323	37224740
chr5	37238856	37239869
chr5	37238856	37245845
chr5	37238856	37247745
chr5	37243012	37244607
chr5	37243012	37247745
chr5	37244374	37247745
chr5	37292878	37299568
chr5	37292878	37307432
chr5	37292878	37310743
chr5	37301436	37303414
chr5	37301436	37305210
chr5	37301436	37309267
chr5	37301436	37318085

chr5	37301436	37324107
chr5	37302778	37303414
chr5	37302778	37305210
chr5	37302778	37307432
chr5	37302778	37324107
chr5	37309128	37314328
chr5	37325900	37328420
chr5	37325900	37358151
chr5	37329189	37331795
chr5	37331684	37333633
chr5	37331684	37341242
chr5	37333462	37341242
chr5	37341089	37348596
chr5	37342548	37350265
chr5	37342548	37351356
chr5	37342548	37352829
chr5	37348504	37358151
chr5	37349171	37358151
chr5	37351189	37352829
chr5	37351189	37358151
chr5	37358080	37364384
chr5	37391999	37443372
chr5	37437921	37605238
chr5	37479833	37605238
chr5	37605063	37697754
chr5	37605063	37703087
chr5	37697654	37703087
chr5	37701057	37727045
chr5	37721114	37727045
chr5	38510463	38530666
chr5	38523418	38530666
chr5	38527154	38530666
chr5	38869031	38885484
chr5	38876200	38885484
chr5	38903881	38921794
chr5	38903881	38924595
chr5	38967160	38968030
chr5	38971876	38978650
chr5	38971876	38982036
chr5	38981866	39021136
chr5	38990948	38996882
chr5	38990948	39003622
chr5	38990948	39021136
chr5	38996818	39003622
chr5	38996818	39021136

chr5	39002534	39021136
chr5	40728344	40730343
chr5	40728344	40747019
chr5	40764513	40767690
chr5	40764513	40771863
chr5	40764513	40775503
chr5	40764513	40777586
chr5	40767465	40775503
chr5	40771718	40777586
chr5	41739389	41762200
chr5	41739389	41794078
chr5	41739389	41807438
chr5	41749526	41805681
chr5	41762110	41807438
chr5	41794002	41801070
chr5	41794002	41807438
chr5	41840450	41853554
chr5	41840450	41862750
chr5	41850029	41862750
chr5	41927012	41929917
chr5	41927012	41934308
chr5	42688889	42700002
chr5	42688889	42718121
chr5	43122038	43123310
chr5	43122038	43139309
chr5	43292473	43297166
chr5	43292473	43298975
chr5	43292473	43307824
chr5	43292847	43297166
chr5	43294055	43297166
chr5	43294055	43298975
chr5	43294690	43298975
chr5	43295751	43297166
chr5	43298008	43298975
chr5	43609142	43659350
chr5	43609142	43677806
chr5	43612907	43628387
chr5	43628199	43659350
chr5	43628199	43677806
chr5	43644191	43659350
chr5	50399858	50411383
chr5	50402285	50405852
chr5	50402285	50411383
chr5	50668070	50788589
chr5	50750150	50763242

chr5	50750150	50778650
chr5	50750150	50788589
chr5	50759642	50763242
chr5	50759642	50797233
chr5	50788522	50797032
chr5	50788522	50797233
chr5	50821212	50828384
chr5	5187724	5209246
chr5	5209092	5232516
chr5	5222788	5242191
chr5	53045000	53051559
chr5	53071937	53073259
chr5	53646232	53658624
chr5	5441111	5444326
chr5	5441111	5447897
chr5	5447426	5447897
chr5	5466333	5476079
chr5	55267137	55272175
chr5	55274613	55281515
chr5	55322326	55324198
chr5	55322326	55328811
chr5	55327715	55341771
chr5	55340009	55358698
chr5	55340009	55379195
chr5	55340009	55400421
chr5	55343330	55351029
chr5	55343330	55358698
chr5	55343330	55366875
chr5	55349572	55366875
chr5	55353167	55379195
chr5	55397415	55416132
chr5	55400232	55405588
chr5	55405424	55422982
chr5	55649206	55652723
chr5	55649206	55664863
chr5	55649206	55672695
chr5	55652528	55664863
chr5	55947492	55968396
chr5	55952249	55969855
chr5	55960401	55968396
chr5	55968275	55969855
chr5	56856599	56865977
chr5	56864733	56865977
chr5	57230845	57231321
chr5	57230845	57247215

chr5	57235965	57249576
chr5	57246299	57247215
chr5	57246299	57249576
chr5	58988492	58993465
chr5	58988492	59038971
chr5	60638749	60647533
chr5	60642811	60647533
chr5	60644744	60647533
chr5	60887439	60902508
chr5	60918264	60928959
chr5	61472680	61494410
chr5	61490785	61494410
chr5	618989	620261
chr5	618989	624579
chr5	618989	644425
chr5	62347129	62350120
chr5	62347129	62357745
chr5	62361241	62363320
chr5	62362449	62363899
chr5	62362449	62373837
chr5	62362449	62381253
chr5	62363177	62366481
chr5	62390875	62394607
chr5	62437273	62451933
chr5	62437273	62474464
chr5	62437273	62561257
chr5	62449926	62451933
chr5	62470249	62494124
chr5	62476682	62494124
chr5	62476682	62515501
chr5	62504666	62515501
chr5	62526141	62561257
chr5	62530708	62551336
chr5	62530708	62561257
chr5	62530708	62591672
chr5	62536701	62601848
chr5	62550366	62561257
chr5	62550366	62591672
chr5	633768	644425
chr5	64325185	64330406
chr5	64785480	64804386
chr5	64788950	64804386
chr5	65170616	65173008
chr5	65170616	65197151
chr5	65170616	65215487

chr5	65188015	65197151
chr5	65188015	65215487
chr5	65214793	65471142
chr5	65224319	65226219
chr5	65224319	65242206
chr5	65224319	65291470
chr5	65242103	65273447
chr5	65242103	65291470
chr5	65242103	65300131
chr5	65242103	65334085
chr5	65242103	65473952
chr5	65260599	65273447
chr5	65260599	65291470
chr5	65260599	65300131
chr5	65260599	65334085
chr5	65260599	65452918
chr5	65273339	65291470
chr5	65273339	65300131
chr5	65291328	65300131
chr5	65291328	65334085
chr5	65291328	65471142
chr5	65299984	65334085
chr5	65329377	65473952
chr5	65451474	65452918
chr5	65451474	65460338
chr5	65451474	65471142
chr5	65451474	65473952
chr5	65452132	65473952
chr5	65470777	65473952
chr5	65521474	65529199
chr5	65521474	65542848
chr5	65521474	65554946
chr5	65528451	65529199
chr5	65528451	65542848
chr5	65528451	65552549
chr5	65567512	65572286
chr5	65604910	65618255
chr5	65635300	65637780
chr5	65635300	65650882
chr5	65650809	65658501
chr5	65671914	65704378
chr5	65680493	65685472
chr5	65704278	65708558
chr5	65704278	65713064
chr5	65758566	65763108

chr5	65777426	65781421
chr5	65780178	65788484
chr5	65780178	65792655
chr5	65988634	65994864
chr5	65988634	66014725
chr5	65988634	66023364
chr5	65988634	66038482
chr5	65992709	65994864
chr5	65992709	66014725
chr5	65994746	66014725
chr5	65994746	66028343
chr5	66012048	66014725
chr5	66012048	66023364
chr5	66012048	66025977
chr5	66012048	66028343
chr5	66021321	66028343
chr5	66021321	66038482
chr5	66024305	66044310
chr5	6604137	6605408
chr5	66043076	66044310
chr5	6610954	6611798
chr5	66153462	66159334
chr5	66158808	66164897
chr5	6616726	6625669
chr5	6622015	6625669
chr5	6623213	6625669
chr5	6651841	6662966
chr5	6737509	6739852
chr5	6737509	6742597
chr5	6737509	6746427
chr5	6737509	6748590
chr5	6737509	6750503
chr5	6737509	6753037
chr5	6739731	6746427
chr5	69100806	69108436
chr5	69104630	69108436
chr5	69104630	69114496
chr5	69123198	69128132
chr5	69171269	69174409
chr5	69174876	69175537
chr5	69191794	69197109
chr5	69191794	69202970
chr5	69194646	69202970
chr5	69226337	69228387
chr5	69252417	69262304

chr5	69258042	69259936
chr5	69258042	69262304
chr5	69258042	69269293
chr5	69262204	69269293
chr5	69285336	69300127
chr5	69294792	69311204
chr5	69300011	69311204
chr5	69355648	69355953
chr5	69371034	69374711
chr5	69373829	69374711
chr5	69373829	69384933
chr5	69381900	69389145
chr5	69419370	69424636
chr5	69513947	69534839
chr5	69513947	69548101
chr5	69513947	69551585
chr5	69534693	69548101
chr5	69566966	69572550
chr5	69579020	69579864
chr5	69585593	69586356
chr5	70063414	70070751
chr5	71055351	71062796
chr5	71461816	71470489
chr5	71495249	71502791
chr5	71501561	71504751
chr5	71517321	71522949
chr5	71522288	71544507
chr5	71545038	71549606
chr5	71548681	71549606
chr5	71548681	71560237
chr5	71553115	71560237
chr5	71559981	71562520
chr5	71592925	71596364
chr5	71592925	71599760
chr5	71592925	71604468
chr5	71596279	71604468
chr5	71604355	71646277
chr5	71632120	71650183
chr5	72223682	72238128
chr5	72232442	72238128
chr5	72331257	72335885
chr5	72848384	72865729
chr5	72855773	72865729
chr5	72855773	72875737
chr5	72861807	72865729

chr5	72861807	72875737
chr5	72861807	72877346
chr5	72861807	72887222
chr5	72861807	72893703
chr5	72872638	72883232
chr5	72877227	72883232
chr5	72887069	72888303
chr5	72887069	72889957
chr5	72888077	72889957
chr5	72889785	72905445
chr5	72968497	73006549
chr5	72989426	72990864
chr5	72989426	73017308
chr5	73006444	73017308
chr5	73006444	73041315
chr5	73037142	73052507
chr5	73051348	73054549
chr5	73051348	73068779
chr5	73051348	73082825
chr5	73058432	73077493
chr5	73074741	73077493
chr5	73074741	73078312
chr5	73074741	73082825
chr5	73078179	73082825
chr5	73749836	73753202
chr5	73749836	73776696
chr5	73749836	73780745
chr5	73749836	73794454
chr5	73749836	73795391
chr5	73773854	73776696
chr5	73773854	73780745
chr5	73773854	73795391
chr5	73794401	73832459
chr5	73832337	73852692
chr5	73832337	73858219
chr5	73832337	73873246
chr5	73840479	73852692
chr5	73840479	73858219
chr5	73852649	73898093
chr5	73857655	73858219
chr5	73868099	73870209
chr5	73892051	73901284
chr5	73904221	73911575
chr5	74715509	74716673
chr5	74722378	74741609

chr5	74722378	74745857
chr5	74725639	74726126
chr5	74745677	74750667
chr5	74745677	74751493
chr5	74747691	74763766
chr5	74750578	74751493
chr5	74758848	74763766
chr5	74796029	74805284
chr5	74796029	74814019
chr5	74796029	74839050
chr5	74796029	74841679
chr5	74804492	74841679
chr5	74813839	74834597
chr5	74813839	74841679
chr5	74834425	74841679
chr5	74838964	74841679
chr5	74840073	74841679
chr5	75343852	75345658
chr5	75384641	75400297
chr5	75399309	75411103
chr5	75400204	75411103
chr5	75411010	75417033
chr5	75411010	75426478
chr5	75416875	75426478
chr5	75416875	75459181
chr5	75547009	75552591
chr5	75547009	75569492
chr5	75569339	75590440
chr5	75685206	75692500
chr5	75685206	75702810
chr5	75694654	75707875
chr5	77034417	77036720
chr5	77034417	77039719
chr5	77046346	77048272
chr5	77046346	77048987
chr5	77048935	77055596
chr5	77053964	77055596
chr5	77449759	77453928
chr5	77449759	77463226
chr5	77449759	77464809
chr5	77449759	77489572
chr5	77451290	77464809
chr5	77459061	77464809
chr5	78015409	78165672
chr5	78020691	78039274

chr5	78020691	78101025
chr5	78089392	78128160
chr5	78089392	78129307
chr5	78089392	78165672
chr5	78100952	78129307
chr5	78100952	78165672
chr5	78110206	78165672
chr5	78113751	78165672
chr5	78128029	78165672
chr5	78156257	78165672
chr5	78156257	78216237
chr5	78156257	78267595
chr5	78162818	78181662
chr5	78165609	78216237
chr5	78175625	78181662
chr5	78216054	78228239
chr5	78216054	78267595
chr5	78388836	78421960
chr5	78388836	78459362
chr5	78415519	78421960
chr5	7870769	7885854
chr5	7875257	7885854
chr5	7875257	7897247
chr5	78839355	78955502
chr5	7889094	7897247
chr5	78955294	78969192
chr5	78955294	78985558
chr5	7895733	7897247
chr5	78964415	78985558
chr5	79314256	79316310
chr5	79396822	79457018
chr5	79439009	79451121
chr5	79439009	79457018
chr5	79447052	79457018
chr5	79613010	79648693
chr5	79619611	79620083
chr5	79619611	79623489
chr5	79640850	79642910
chr5	79645122	79649190
chr5	79645122	79669028
chr5	79656957	79669028
chr5	79668891	79682061
chr5	79986949	79990236
chr5	801099	821895
chr5	80174953	80178064

chr5	80177314	80178064
chr5	80434108	80440032
chr5	80434108	80445405
chr5	80434108	80459494
chr5	80443122	80445405
chr5	80449590	80474830
chr5	80455691	80474830
chr5	80519692	80522138
chr5	80656410	80679093
chr5	80665142	80679093
chr5	80678926	80728965
chr5	80678926	80744615
chr5	80678926	80769003
chr5	80725452	80741548
chr5	80725452	80744615
chr5	80725452	80761678
chr5	80728850	80744615
chr5	80767932	80778836
chr5	80767932	80792844
chr5	80775693	80778836
chr5	80775693	80787672
chr5	80775693	80792844
chr5	80775693	80813741
chr5	80778719	80792844
chr5	80787564	80792844
chr5	81086834	81092961
chr5	81489249	81650339
chr5	81976192	81987678
chr5	81987558	82178587
chr5	82058494	82178587
chr5	82164398	82178587
chr5	82276397	82277852
chr5	83104909	83111203
chr5	83104909	83204921
chr5	83104909	83258677
chr5	83195769	83204921
chr5	83203551	83258677
chr5	84060299	84066606
chr5	87331347	87338091
chr5	87331347	87341321
chr5	87331347	87346724
chr5	87331347	87349364
chr5	87331347	87353235
chr5	87331347	87362671
chr5	87332506	87346724

chr5	87332506	87349364
chr5	87341289	87353235
chr5	87372117	87374916
chr5	881106	889766
chr5	88196375	88203075
chr5	88202961	88240916
chr5	88225768	88228528
chr5	886591	891854
chr5	887360	891854
chr5	90405684	90407834
chr5	90485524	90506674
chr5	90487999	90501988
chr5	907129	911996
chr5	91373683	91378775
chr5	91376620	91378775
chr5	93544794	93563463
chr5	93553760	93563463
chr5	93824147	93881688
chr5	93958776	94053227
chr5	93958776	94074769
chr5	94050730	94053227
chr5	94050730	94074769
chr5	94628810	94630743
chr5	94628810	94643435
chr5	94643272	94651845
chr5	94654629	94663908
chr5	94662297	94671008
chr5	94670150	94678955
chr5	94868332	94924021
chr5	94888878	94912976
chr5	94909250	94912976
chr5	94909250	94924021
chr5	95490907	95495017
chr5	95494681	95498591
chr5	95506933	95517060
chr5	95736888	95763620
chr5	95736888	95768166
chr5	95736888	95783963
chr5	95748332	95763620
chr5	95752238	95768166
chr5	95755395	95763620
chr5	95755395	95783963
chr5	95763507	95780425
chr5	95763507	95783963
chr5	95763507	95788858

chr5	95768045	95783963
chr5	95768045	95788858
chr5	95898239	95906782
chr5	95906522	95913934
chr5	95906522	95919545
chr5	95913770	95943049
chr5	96722638	96729725
chr5	96722638	96730860
chr5	96729152	96737947
chr5	96736171	96741580
chr5	96750586	96754741
chr5	96912636	96913457
chr5	96912636	96915769
chr5	96979137	96986670
chr5	96985079	96986670
chr5	97024520	97026757
chr5	98868494	98872555
chr5	98879551	98881375
chr5	98885577	98889238
chr5	98892524	98894686
chr6	100589633	100607088
chr6	100589633	100631213
chr6	100589633	100638821
chr6	100589633	100767345
chr6	100625191	100631213
chr6	100625191	100662536
chr6	100650537	100662536
chr6	100652724	100662536
chr6	100679617	100805880
chr6	100715461	100725703
chr6	100715461	100798838
chr6	100715461	100805880
chr6	100766564	100767345
chr6	100766564	100805880
chr6	100798712	100800504
chr6	100798712	100805880
chr6	10400447	10402610
chr6	10404507	10410335
chr6	104744159	104750472
chr6	104744159	104772074
chr6	104744159	104777317
chr6	104744159	104785319
chr6	104771192	104772074
chr6	104771192	104796756
chr6	104784085	104849246

chr6	104791503	104797025
chr6	104795578	104852371
chr6	104796654	104811393
chr6	104796654	104833173
chr6	104811310	104843298
chr6	104811310	104849246
chr6	104833041	104843298
chr6	104833041	104849246
chr6	104833041	104850996
chr6	104833041	104852371
chr6	104843222	104849246
chr6	105115685	105116868
chr6	105115685	105133588
chr6	105124546	105133588
chr6	10527473	10529836
chr6	105323664	105333505
chr6	106201971	106248244
chr6	106201971	106293106
chr6	106248149	106279823
chr6	106248149	106293106
chr6	106248149	106308491
chr6	106248149	106316266
chr6	106279660	106308491
chr6	106544570	106544933
chr6	106583327	106602922
chr6	106583327	106622969
chr6	10697323	10702639
chr6	10702368	10702639
chr6	10702368	10704844
chr6	10703404	10704844
chr6	107039748	107044332
chr6	10724569	10726008
chr6	10749201	10755232
chr6	107503656	107506427
chr6	107503656	107533610
chr6	107876563	107883146
chr6	107881148	107883146
chr6	107893481	107924932
chr6	107901369	107902998
chr6	107901369	107924932
chr6	107901369	107929514
chr6	107904628	107906777
chr6	107904628	107924932
chr6	107911345	107924932
chr6	107912715	107924932

chr6	107921796	107924932
chr6	107921796	107929514
chr6	108049252	108054587
chr6	108214497	108223045
chr6	108323824	108402054
chr6	108355653	108366332
chr6	108355653	108447296
chr6	108356689	108402054
chr6	108476864	108477292
chr6	108759417	108760403
chr6	108854224	108876350
chr6	108928087	108936999
chr6	10893866	10898115
chr6	10902676	10907684
chr6	10907541	10912927
chr6	109145218	109146937
chr6	10931439	10956242
chr6	10935057	10942746
chr6	10935057	10956242
chr6	109467652	109476930
chr6	109475398	109476930
chr6	10961304	10963843
chr6	109656755	109672167
chr6	109715077	109716568
chr6	109715077	109735298
chr6	109738324	109741544
chr6	110101587	110127629
chr6	110127468	110160732
chr6	110207505	110226243
chr6	110210706	110213160
chr6	110217701	110219869
chr6	110667433	110670541
chr6	110667433	110746201
chr6	110887504	110888983
chr6	110887504	110890356
chr6	110887504	110893065
chr6	110960413	110962505
chr6	110997182	111015856
chr6	111172694	111177665
chr6	111172694	111219042
chr6	111177211	111219042
chr6	111262256	111263946
chr6	111343924	111368028
chr6	111387764	111405630
chr6	111387764	111416472

chr6	111390085	111405630
chr6	111405469	111472140
chr6	111416282	111472140
chr6	113945361	113953418
chr6	113948978	113956151
chr6	113948978	113956693
chr6	113953276	113956151
chr6	113956618	113960018
chr6	116106243	116117945
chr6	116645713	116660846
chr6	116647458	116660846
chr6	116651571	116658924
chr6	116689319	116692156
chr6	116689319	116692392
chr6	116689319	116705160
chr6	116689319	116716318
chr6	116692054	116705160
chr6	116692054	116726622
chr6	116698703	116705160
chr6	116698703	116716318
chr6	116716218	116724375
chr6	116716218	116732368
chr6	117503766	117525434
chr6	117503766	117539379
chr6	117519815	117525434
chr6	117532259	117539379
chr6	117540667	117543211
chr6	117566853	117569736
chr6	117693041	117694180
chr6	118479870	118483858
chr6	118511297	118566316
chr6	118523801	118566316
chr6	118826146	118829250
chr6	118826781	118856545
chr6	121106022	121170527
chr6	121126377	121170527
chr6	121160009	121170527
chr6	121205074	121242339
chr6	121205074	121255410
chr6	121205074	121256285
chr6	121223235	121242339
chr6	121223235	121255410
chr6	121255327	121317672
chr6	121279120	121294660
chr6	121279120	121310847

chr6	121279120	121317672
chr6	121279120	121321794
chr6	121292052	121299505
chr6	12129758	12130942
chr6	121303616	121310847
chr6	121307975	121310847
chr6	122413524	122423686
chr6	123224092	123267751
chr6	123252411	123267751
chr6	123316456	123382147
chr6	123331878	123382147
chr6	123366134	123382147
chr6	125009173	125058106
chr6	125045210	125058106
chr6	125045210	125076858
chr6	125815290	125855240
chr6	125874888	125878370
chr6	125920942	125922834
chr6	125993756	126021280
chr6	126008391	126021280
chr6	126011252	126013101
chr6	126011252	126021280
chr6	127285207	127286177
chr6	127314815	127331030
chr6	127327001	127331030
chr6	127976922	127996988
chr6	128005083	128067792
chr6	128005083	128089992
chr6	128064757	128067792
chr6	128064757	128089992
chr6	128078812	128089992
chr6	128184431	128322310
chr6	128218921	128242602
chr6	128322038	128397688
chr6	129491900	129492483
chr6	129583987	129618852
chr6	129611532	129618852
chr6	129611532	129629522
chr6	129611532	129638629
chr6	129629352	129634105
chr6	130022226	130042801
chr6	130022226	130094367
chr6	130078557	130104575
chr6	130154824	130176008
chr6	130154824	130184623

chr6	130175840	130184623
chr6	130175840	130222813
chr6	130184102	130184623
chr6	130863637	130867581
chr6	130885095	130908863
chr6	130890293	130908863
chr6	130894343	130904540
chr6	130899490	130908863
chr6	130955104	130956499
chr6	131145284	131169273
chr6	131160058	131169273
chr6	131160058	131199573
chr6	131598286	131598761
chr6	131598286	131608071
chr6	131606478	131610246
chr6	131618406	131621979
chr6	131854925	131864571
chr6	131884930	131886724
chr6	132372607	132374777
chr6	132469950	132472375
chr6	132783932	132797121
chr6	13316570	13321095
chr6	13316570	13326906
chr6	133982567	133984671
chr6	134942408	134943966
chr6	134986065	134993875
chr6	134993757	135002842
chr6	135002733	135042126
chr6	135039572	135042126
chr6	135039572	135050647
chr6	135290422	135323324
chr6	135300499	135323324
chr6	135300499	135394896
chr6	135318518	135358187
chr6	135318518	135394896
chr6	135318518	135428759
chr6	135358131	135394896
chr6	135358131	135411544
chr6	135358131	135428759
chr6	135358131	135442714
chr6	135433026	135455926
chr6	135433026	135457713
chr6	135438374	135457713
chr6	135442581	135457713
chr6	135447007	135448475

chr6	135453340	135455926
chr6	135453340	135457713
chr6	13579450	13584225
chr6	13625652	13644729
chr6	136267028	136282687
chr6	136277864	136282687
chr6	13632369	13644729
chr6	13632369	13658832
chr6	13637807	13658832
chr6	136388392	136389517
chr6	13639562	13644729
chr6	13639562	13657276
chr6	13639562	13658832
chr6	13641198	13644729
chr6	13652658	13658832
chr6	13652658	13696896
chr6	136561532	136567874
chr6	13657108	13658832
chr6	136592172	136592614
chr6	136601021	136622981
chr6	136637324	136642569
chr6	136637324	136698682
chr6	136669282	136698682
chr6	136694139	136697387
chr6	136694139	136698682
chr6	136694139	136705133
chr6	136694139	136720589
chr6	136825213	136870003
chr6	136866626	136872253
chr6	137875687	137877256
chr6	13793893	13807044
chr6	13798832	13807044
chr6	138207041	138210041
chr6	138207041	138229852
chr6	138229783	138245591
chr6	138441982	138447193
chr6	138473305	138496371
chr6	138907494	138914761
chr6	138907494	138917627
chr6	138907494	138941489
chr6	138911275	138930098
chr6	138926400	138941489
chr6	138926400	138947913
chr6	138941334	138945697
chr6	138941334	138947913

chr6	138943512	138944622
chr6	138943512	138947913
chr6	138944497	138947913
chr6	142166227	142170421
chr6	142166227	142189534
chr6	142189425	142204065
chr6	142198438	142204065
chr6	142390257	142420104
chr6	142759771	142837047
chr6	143060946	143284225
chr6	143165081	143284225
chr6	143470960	143474856
chr6	143748984	143753912
chr6	143748984	143777383
chr6	143765260	143777383
chr6	143765260	143788910
chr6	143765260	143807133
chr6	143772257	143774215
chr6	143772257	143777383
chr6	143844485	143846224
chr6	143844485	143850218
chr6	143850130	143858007
chr6	143960468	143985174
chr6	144421877	144429741
chr6	144426286	144429741
chr6	144451369	144459354
chr6	144451369	144474759
chr6	144458769	144462866
chr6	144473719	144474759
chr6	144482208	144488834
chr6	144487547	144493456
chr6	144490070	144493456
chr6	144513908	144514820
chr6	144521979	144523188
chr6	144531051	144539443
chr6	144531051	144557311
chr6	144531051	144577288
chr6	144537581	144539443
chr6	144537581	144542870
chr6	144537581	144557311
chr6	144577098	144757989
chr6	144678405	144757989
chr6	144700086	144730486
chr6	144730356	144757989
chr6	144748245	144774364

chr6	144748245	144782123
chr6	144781921	144793991
chr6	144781921	144803147
chr6	144793833	144821018
chr6	144820881	144836541
chr6	145888019	145894977
chr6	145893214	145894977
chr6	145919347	145933178
chr6	147205970	147239270
chr6	147205970	147278204
chr6	147205970	147291172
chr6	147205970	147334222
chr6	147260614	147291172
chr6	147260614	147316407
chr6	147310083	147339238
chr6	147314263	147316407
chr6	147314263	147334222
chr6	147315514	147334222
chr6	148440183	148487715
chr6	148468544	148487715
chr6	148886985	148964563
chr6	148941278	148964563
chr6	149019138	149021481
chr6	149342684	149370099
chr6	149342684	149379518
chr6	149512154	149517450
chr6	149512154	149526776
chr6	149512154	149541586
chr6	149521059	149541586
chr6	149525142	149534774
chr6	149533457	149535738
chr6	149533457	149541586
chr6	149540941	149541586
chr6	149572284	149582603
chr6	149579833	149582603
chr6	149597062	149598350
chr6	149601593	149604782
chr6	149603267	149604782
chr6	149623102	149632916
chr6	149623102	149638560
chr6	149632758	149638560
chr6	149676259	149676737
chr6	149676259	149680457
chr6	149676259	149684592
chr6	149683078	149695221

chr6	149695073	149702266
chr6	149731612	149736622
chr6	149736470	149738778
chr6	149738642	149742570
chr6	149738642	149743715
chr6	149742389	149743715
chr6	149771161	149793669
chr6	149771161	149796500
chr6	149771161	149802416
chr6	149789953	149796500
chr6	149826263	149853160
chr6	149836084	149853160
chr6	150876089	150882886
chr6	150876089	150887981
chr6	150876089	151013820
chr6	150877633	150885734
chr6	150877633	150887981
chr6	150882761	150887981
chr6	150905649	150926295
chr6	150905649	150965037
chr6	150918576	150972058
chr6	150926121	150938745
chr6	150926121	150956071
chr6	150936803	150972058
chr6	150944485	150965037
chr6	150949030	150972058
chr6	150955994	150965037
chr6	150955994	150972058
chr6	151009818	151015693
chr6	151009818	151037117
chr6	151014879	151015693
chr6	151034492	151037117
chr6	151417278	151427582
chr6	15374116	15410365
chr6	15374116	15452175
chr6	15374116	15468718
chr6	15410223	15468718
chr6	154410342	154450258
chr6	154422505	154422983
chr6	154422505	154442290
chr6	154428127	154450258
chr6	154441291	154442290
chr6	154441291	154450258
chr6	154773988	154792976
chr6	154773988	154795139

chr6	154778000	154805486
chr6	154787860	154792976
chr6	154787860	154795139
chr6	154787860	154805486
chr6	154787860	154810208
chr6	154805368	154810208
chr6	154808069	154810208
chr6	154808069	154818592
chr6	154815715	154820333
chr6	154818478	154827240
chr6	15507135	15508454
chr6	155296952	155311339
chr6	15533239	15615399
chr6	15533239	15637804
chr6	15593058	15637804
chr6	15615266	15627475
chr6	15615266	15637804
chr6	15627342	15637804
chr6	156829226	156901525
chr6	156829226	156935576
chr6	157084661	157110561
chr6	157084661	157133207
chr6	157110471	157133207
chr6	157110471	157167185
chr6	157167039	157190210
chr6	157628348	157653627
chr6	157647258	157653627
chr6	157867546	157875176
chr6	157896826	157906212
chr6	157896826	157910025
chr6	157921530	157932272
chr6	158017203	158033680
chr6	158074579	158078281
chr6	158081108	158081510
chr6	158114788	158120575
chr6	158119028	158128270
chr6	158143055	158155351
chr6	158146781	158158364
chr6	158413064	158461729
chr6	158429735	158452268
chr6	158429735	158461729
chr6	158448995	158452268
chr6	158448995	158461729
chr6	158448995	158481289
chr6	158481054	158489732

chr6	158573419	158589782
chr6	158573419	158605347
chr6	158573419	158607343
chr6	158573419	158608750
chr6	158573419	158625754
chr6	158573419	158631389
chr6	158580939	158589782
chr6	158583953	158585425
chr6	158583953	158589782
chr6	158583953	158608750
chr6	158589671	158608750
chr6	158605266	158608750
chr6	158637127	158641360
chr6	158776407	158789371
chr6	158783519	158787203
chr6	158783519	158789371
chr6	158980773	158983807
chr6	158980773	158986421
chr6	159682473	159688242
chr6	159736257	159743792
chr6	159736257	159748369
chr6	159775169	159777456
chr6	159780442	159781110
chr6	159783940	159785997
chr6	159783940	159788143
chr6	159784665	159785496
chr6	159784665	159785997
chr6	159991183	160010785
chr6	159991183	160027314
chr6	160040559	160050652
chr6	160046497	160048543
chr6	160058905	160063630
chr6	161034258	161049979
chr6	161102698	161111958
chr6	16485971	16753347
chr6	16522626	16657901
chr6	16585779	16657901
chr6	16585779	16753347
chr6	166328275	166330247
chr6	16657775	16753347
chr6	16657775	16754091
chr6	166934090	166952548
chr6	167010812	167033971
chr6	167022408	167024894
chr6	167022408	167026615

chr6	167022408	167033971
chr6	167864550	167880517
chr6	167864550	167898467
chr6	167864550	167907289
chr6	167870382	167880517
chr6	167870382	167889326
chr6	167870382	167891029
chr6	167870382	167898467
chr6	167870385	167880517
chr6	167870385	167902386
chr6	167890861	167902386
chr6	167898204	167962567
chr6	169582835	169602321
chr6	169582835	169613656
chr6	169582835	169649275
chr6	169582835	169662424
chr6	169602218	169652008
chr6	169613558	169634525
chr6	169632946	169638660
chr6	169632946	169652008
chr6	169658275	169662424
chr6	169705132	169721111
chr6	169710235	169721111
chr6	169756718	169776673
chr6	169758967	169762531
chr6	169759837	169768171
chr6	169762428	169768171
chr6	170323078	170358318
chr6	170348150	170358318
chr6	170388286	170395579
chr6	170388286	170404601
chr6	170537233	170549113
chr6	170543600	170549113
chr6	1726415	1742586
chr6	17421554	17426690
chr6	17421554	17513954
chr6	17462995	17507726
chr6	17462995	17513954
chr6	17507168	17507726
chr6	17507168	17513954
chr6	17539268	17543143
chr6	17540972	17543143
chr6	17624560	17649300
chr6	17632649	17640064
chr6	17637152	17646154

chr6	17639938	17649300
chr6	17639938	17675033
chr6	17646066	17649300
chr6	17647806	17649300
chr6	17649162	17665385
chr6	17661652	17665385
chr6	17661652	17669028
chr6	17661652	17669546
chr6	17661652	17675033
chr6	17661652	17675770
chr6	17665238	17669028
chr6	17665238	17669546
chr6	17665238	17675770
chr6	17669292	17675033
chr6	17674904	17675770
chr6	17674904	17688618
chr6	17675228	17675770
chr6	17771907	17779099
chr6	17775014	17779099
chr6	17783645	17796820
chr6	17794248	17794704
chr6	17825767	17834071
chr6	17833960	17837583
chr6	17836877	17856122
chr6	17855436	17873437
chr6	18147822	18149171
chr6	18159882	18162924
chr6	18159882	18166378
chr6	18161326	18162924
chr6	18185771	18217885
chr6	18207397	18217885
chr6	18212487	18213781
chr6	18212487	18217885
chr6	18213655	18217885
chr6	18215006	18217885
chr6	18236451	18237516
chr6	18236451	18249839
chr6	18255730	18258405
chr6	18255730	18263996
chr6	18256360	18258405
chr6	1959866	1960966
chr6	1959866	2117556
chr6	20112875	20144315
chr6	20144219	20152769
chr6	20535350	20548705

chr6	20546345	20548705
chr6	20649292	20781265
chr6	20781144	20846178
chr6	20781144	20955585
chr6	21108400	21201274
chr6	2115770	2117556
chr6	2115770	2124731
chr6	24405167	24409573
chr6	24405167	24412395
chr6	24405167	24418578
chr6	24408407	24415163
chr6	24412221	24416513
chr6	24416396	24418578
chr6	24445545	24445831
chr6	24456497	24472636
chr6	24475120	24479959
chr6	24502522	24504985
chr6	24502522	24515310
chr6	24502522	24522925
chr6	24578109	24583702
chr6	24658560	24666611
chr6	24777221	24781621
chr6	25449897	25472521
chr6	25465872	25495215
chr6	25482256	25500235
chr6	25509655	25520337
chr6	25515674	25520337
chr6	25515674	25528893
chr6	25550909	25556850
chr6	25580923	25581439
chr6	25966334	25969420
chr6	26385014	26388294
chr6	26426241	26428785
chr6	2768690	2779492
chr6	2768690	2783561
chr6	2779262	2783561
chr6	2779262	2784403
chr6	28271853	28272787
chr6	2836107	2840594
chr6	2954591	2959342
chr6	29604493	29605696
chr6	29621100	29622205
chr6	29621100	29624024
chr6	29889327	29890485
chr6	30034907	30035983

chr6	30061514	30062333
chr6	3006467	3012674
chr6	3006467	3016985
chr6	30196515	30204765
chr6	30328888	30330876
chr6	30585878	30589720
chr6	30608778	30609955
chr6	30619492	30619989
chr6	30627459	30628430
chr6	30627459	30642247
chr6	30627459	30643011
chr6	30627850	30628430
chr6	30649557	30651466
chr6	30650993	30651466
chr6	30670409	30671274
chr6	30730021	30731100
chr6	30888687	30889430
chr6	31148404	31148728
chr6	31410542	31412460
chr6	31507021	31507531
chr6	31623731	31626162
chr6	31647590	31649627
chr6	31648676	31649627
chr6	31741162	31747432
chr6	31742876	31744581
chr6	31742876	31753629
chr6	31743903	31744581
chr6	31743903	31753629
chr6	31772953	31773437
chr6	31784213	31785733
chr6	31835410	31837435
chr6	31892406	31892910
chr6	31961040	31961386
chr6	31968327	31968796
chr6	32118955	32120902
chr6	32120770	32121348
chr6	32120770	32126252
chr6	32126116	32127750
chr6	32127102	32127750
chr6	32154586	32162622
chr6	32170424	32171070
chr6	32835153	32835773
chr6	3283851	3298218
chr6	32974461	32975112
chr6	32974461	32980464

chr6	32975383	32976169
chr6	33266556	33270283
chr6	33267681	33269557
chr6	33272627	33276066
chr6	33294687	33295045
chr6	33303954	33304638
chr6	33320010	33321567
chr6	33398028	33398387
chr6	33403313	33406665
chr6	33403728	33406875
chr6	33406195	33406875
chr6	33432160	33432806
chr6	335113	348841
chr6	33688655	33690198
chr6	33690916	33691719
chr6	33690916	33691928
chr6	33776953	33786774
chr6	3410187	3415855
chr6	34237203	34243518
chr6	34240736	34242795
chr6	34240736	34243518
chr6	345853	348841
chr6	34606554	34646798
chr6	34606554	34654779
chr6	34646600	34654779
chr6	34762594	34770395
chr6	34821652	34821955
chr6	34821652	34823348
chr6	34821652	34834861
chr6	34823256	34835464
chr6	34834216	34834861
chr6	34834216	34836348
chr6	34867224	34867616
chr6	34967238	34985278
chr6	34981689	34983425
chr6	34981689	34994422
chr6	34994301	35018059
chr6	35312301	35312772
chr6	35410986	35421958
chr6	35420126	35421958
chr6	35421819	35424779
chr6	35587033	35620274
chr6	35587033	35637158
chr6	35597247	35620274
chr6	35597247	35637158

chr6	35597247	35642843
chr6	35619095	35620274
chr6	35619095	35637158
chr6	35619095	35642843
chr6	35637013	35642843
chr6	35857260	35869901
chr6	35857260	35870959
chr6	35857260	35872728
chr6	35869009	35869901
chr6	35874232	35891013
chr6	35888023	35891041
chr6	36052698	36059347
chr6	36072872	36102649
chr6	36213879	36218010
chr6	36222167	36229056
chr6	36474788	36481759
chr6	36496710	36499990
chr6	36496710	36507602
chr6	36496710	36525642
chr6	36506582	36525642
chr6	36515337	36517840
chr6	36515337	36540207
chr6	36524340	36540204
chr6	36524340	36540207
chr6	36894503	36902434
chr6	36894503	36914693
chr6	36899425	36902434
chr6	36899425	36916574
chr6	36899425	36919701
chr6	37279303	37284464
chr6	37279303	37287072
chr6	37279303	37291357
chr6	37312917	37317206
chr6	3737078	3738148
chr6	37371511	37381354
chr6	37374619	37381354
chr6	37443998	37446449
chr6	37443998	37479255
chr6	37458611	37463008
chr6	37929958	38082457
chr6	37929958	38116739
chr6	38061592	38082457
chr6	38082391	38116739
chr6	38256408	38288461
chr6	38577599	38580437

chr6	38577599	38598121
chr6	38577599	38613033
chr6	38592575	38598121
chr6	4042486	4044023
chr6	41079028	41084192
chr6	41090203	41093085
chr6	4117307	4119275
chr6	4117307	4133701
chr6	4125249	4126237
chr6	4125249	4127831
chr6	41565744	41578081
chr6	41771815	41778176
chr6	41798723	41871637
chr6	41871563	41891875
chr6	42051356	42068607
chr6	42055530	42057513
chr6	42055530	42068607
chr6	42066311	42068607
chr6	42259338	42263568
chr6	42573733	42592229
chr6	42573733	42594304
chr6	42592150	42594304
chr6	42592150	42606651
chr6	42592150	42612291
chr6	42603587	42605859
chr6	42603587	42606651
chr6	42603587	42612291
chr6	42603587	42615178
chr6	42603587	42637194
chr6	42603587	42642481
chr6	42606588	42635546
chr6	42612170	42617507
chr6	42612170	42632904
chr6	42615070	42617507
chr6	42615070	42632904
chr6	42616001	42632904
chr6	42616001	42644336
chr6	42632551	42632904
chr6	42632551	42637194
chr6	42637010	42642481
chr6	42637010	42666245
chr6	42644213	42648170
chr6	42650283	42652645
chr6	42650283	42658824
chr6	42658023	42666245

chr6	42659655	42670715
chr6	42662183	42666245
chr6	42663257	42666245
chr6	42665408	42670240
chr6	42673790	42679832
chr6	42676055	42679832
chr6	42676055	42684871
chr6	42678538	42679832
chr6	42678538	42684871
chr6	42822795	42830172
chr6	42822795	42855917
chr6	42855855	42862612
chr6	42934474	42935670
chr6	42934474	42938207
chr6	42966043	42966447
chr6	42967367	42969987
chr6	42968289	42969987
chr6	42974002	42975038
chr6	43012925	43013389
chr6	43024984	43026141
chr6	43024984	43027477
chr6	43026036	43027477
chr6	43055544	43056445
chr6	43055896	43056445
chr6	43056080	43056445
chr6	43138848	43139525
chr6	43141930	43144606
chr6	43175705	43176667
chr6	43196068	43196862
chr6	43206006	43206510
chr6	43226032	43226395
chr6	43432141	43438795
chr6	43436137	43438795
chr6	43478086	43503508
chr6	43478086	43503706
chr6	43497880	43503706
chr6	43498922	43503706
chr6	43502282	43503706
chr6	43520065	43521048
chr6	43524470	43525214
chr6	43524470	43526747
chr6	43525838	43528205
chr6	43527633	43531575
chr6	43527633	43534007
chr6	43527633	43555964

chr6	43527633	43567354
chr6	43531478	43549578
chr6	43533906	43548460
chr6	43533906	43551453
chr6	43533906	43553503
chr6	43546570	43548460
chr6	43546570	43553503
chr6	43546570	43555964
chr6	43546570	43573601
chr6	43549488	43567354
chr6	43549488	43572578
chr6	43553372	43567354
chr6	43555835	43558591
chr6	43555835	43567354
chr6	43555835	43568737
chr6	43555835	43570994
chr6	43555835	43572578
chr6	43555835	43573601
chr6	43560177	43567354
chr6	43560177	43573601
chr6	43562246	43567354
chr6	43562246	43573601
chr6	43565659	43567354
chr6	43565659	43573601
chr6	43568710	43573601
chr6	43570501	43570994
chr6	43570501	43573601
chr6	43570856	43573601
chr6	43572505	43573601
chr6	43582315	43583141
chr6	43582315	43587489
chr6	43582315	43610723
chr6	43587271	43597865
chr6	43587271	43601091
chr6	43587271	43605319
chr6	43587271	43610723
chr6	43597695	43601091
chr6	43604614	43605319
chr6	43774340	43778918
chr6	44134560	44141098
chr6	44138479	44141098
chr6	44139466	44139759
chr6	44227262	44232428
chr6	44227262	44233006
chr6	44250020	44250599

chr6	44251047	44252267
chr6	44390267	44393573
chr6	44403808	44406467
chr6	44403808	44408632
chr6	44419448	44429910
chr6	44932652	44954607
chr6	44932652	45014891
chr6	44932652	45020632
chr6	44932652	45365301
chr6	45003652	45020632
chr6	45003652	45106006
chr6	45003652	45365301
chr6	45105921	45365301
chr6	45422592	45438051
chr6	45422592	45492114
chr6	45422592	45512407
chr6	45431862	45438051
chr6	45431862	45512407
chr6	45491940	45512407
chr6	46655842	46671228
chr6	47283937	47286595
chr6	47503279	47533755
chr6	47503279	47554766
chr6	47533601	47544706
chr6	47533601	47554766
chr6	47544605	47554766
chr6	47554645	47596026
chr6	47574063	47582065
chr6	47576523	47599443
chr6	47576523	47609304
chr6	47580863	47612536
chr6	47595860	47606277
chr6	488978	499700
chr6	4935514	4937737
chr6	4999808	5002245
chr6	52267864	52269226
chr6	52267864	52273916
chr6	52267864	52277688
chr6	52267864	52283406
chr6	52272300	52273916
chr6	52276267	52277198
chr6	52278741	52283406
chr6	52279360	52282861
chr6	52282652	52283406
chr6	52423686	52454287

chr6	52423686	52465115
chr6	52423686	52469473
chr6	52423686	52479787
chr6	52423945	52454287
chr6	52423945	52465115
chr6	52469332	52479787
chr6	52504590	52509586
chr6	52504590	52516737
chr6	52507552	52516737
chr6	52509527	52516737
chr6	52516005	52516737
chr6	53071056	53076543
chr6	53071056	53082618
chr6	53073480	53092547
chr6	53076485	53081098
chr6	53076485	53092824
chr6	532468	576882
chr6	53270592	53291963
chr6	53273219	53276256
chr6	53273219	53287942
chr6	53273219	53291963
chr6	53273219	53295707
chr6	53275089	53291963
chr6	53275089	53295707
chr6	53514203	53522527
chr6	53520777	53522527
chr6	5368549	5431172
chr6	5368549	5545340
chr6	53842109	53882976
chr6	53878992	53897359
chr6	53882886	53899891
chr6	53896497	53904462
chr6	53896497	53913969
chr6	5404541	5431172
chr6	56463564	56466195
chr6	56466077	56472222
chr6	56471105	56482877
chr6	56492933	56504098
chr6	56508528	56517620
chr6	56515449	56517620
chr6	56562137	56600221
chr6	56572099	56600221
chr6	56592181	56598006
chr6	56624529	56640057
chr6	56629249	56634258

chr6	56631210	56636652
chr6	56670640	56704369
chr6	56670640	56735289
chr6	56699652	56704369
chr6	56699652	56735289
chr6	56851396	56900621
chr6	56981710	56992555
chr6	56992377	57015353
chr6	57124733	57128840
chr6	57124733	57133192
chr6	57124733	57134870
chr6	57124733	57142095
chr6	57124733	57150862
chr6	57124733	57161152
chr6	57128728	57134870
chr6	57133041	57148693
chr6	57141301	57142095
chr6	57150718	57161152
chr6	57152220	57161152
chr6	57193841	57196606
chr6	57193841	57210445
chr6	57207627	57210445
chr6	572519	576882
chr6	572519	599225
chr6	57318436	57326045
chr6	57324200	57326045
chr6	57379900	57382168
chr6	57379900	57507454
chr6	57379900	57537625
chr6	57507386	57537625
chr6	57507386	57606457
chr6	57532410	57537625
chr6	57532410	57606457
chr6	57601092	57606457
chr6	576756	599225
chr6	576756	610178
chr6	57924416	57930291
chr6	57926425	57930291
chr6	592468	599225
chr6	592468	610178
chr6	592468	619543
chr6	598020	617835
chr6	610097	619543
chr6	617710	633117
chr6	617710	637861

chr6	629834	637861
chr6	632940	637861
chr6	63646526	63680161
chr6	63678827	63680161
chr6	63694580	63700466
chr6	69737941	69752356
chr6	69741788	69752356
chr6	69749340	69790472
chr6	70415290	70502791
chr6	70426438	70452571
chr6	70426438	70491083
chr6	70428303	70482154
chr6	70428303	70491083
chr6	70452491	70502791
chr6	70486166	70502791
chr6	70533159	70536411
chr6	70732377	70773425
chr6	70732377	70798737
chr6	70754979	70798737
chr6	71293292	71296817
chr6	7176654	7182082
chr6	7176654	7189322
chr6	7181123	7189322
chr6	7181123	7211709
chr6	7187433	7189322
chr6	7187433	7211709
chr6	7187433	7226656
chr6	72640733	72677214
chr6	73003907	73042062
chr6	73105256	73120577
chr6	73111307	73169854
chr6	73133420	73169854
chr6	73428708	73440445
chr6	73428708	73445747
chr6	73440208	73445747
chr6	73466208	73466606
chr6	73479731	73482244
chr6	73480674	73482244
chr6	73480674	73492352
chr6	73610399	73636707
chr6	73635381	73636707
chr6	73723250	73736508
chr6	73723250	73771581
chr6	73730343	73759028
chr6	7398697	7404555

chr6	7398697	7405355
chr6	7410385	7411451
chr6	7410385	7414390
chr6	75142031	75145455
chr6	75152332	75155854
chr6	75177662	75194947
chr6	75188361	75192355
chr6	75258779	75265338
chr6	75260823	75267748
chr6	75621531	75623960
chr6	75621531	75633726
chr6	75621531	75634811
chr6	75621531	75647801
chr6	75621531	75659407
chr6	75634706	75670720
chr6	75640683	75663518
chr6	75647730	75659407
chr6	75647730	75666941
chr6	75659261	75670720
chr6	75702644	75709630
chr6	75702644	75711416
chr6	75817500	75832947
chr6	75817500	75841378
chr6	75822781	75832947
chr6	75822781	75841378
chr6	75835900	75841378
chr6	75835900	75844977
chr6	75840584	75841378
chr6	75840584	75844977
chr6	75844896	75858993
chr6	75844896	75866621
chr6	75844896	75873300
chr6	75848350	75858993
chr6	75861022	75873300
chr6	75866525	75881818
chr6	75879819	75880120
chr6	75886003	75886994
chr6	7601335	7606195
chr6	7602622	7606195
chr6	7845139	7862498
chr6	78954813	78961810
chr6	78954813	78966056
chr6	78988208	78990985
chr6	78997413	79026164
chr6	79015081	79025619

chr6	79015081	79026164
chr6	79015081	79043003
chr6	79017482	79025619
chr6	79017482	79026164
chr6	79019088	79026164
chr6	79025518	79026164
chr6	79042842	79060818
chr6	79042842	79077913
chr6	79060477	79060818
chr6	79513211	79519085
chr6	79924951	79926381
chr6	80005841	80014586
chr6	80011728	80040703
chr6	80013278	80022472
chr6	80027884	80035417
chr6	80127546	80171390
chr6	80127546	80273221
chr6	80129160	80203212
chr6	80167677	80203212
chr6	80200933	80203212
chr6	82181878	82194642
chr6	82191072	82196446
chr6	82194478	82211572
chr6	82202527	82212793
chr6	82210813	82211572
chr6	82210813	82212793
chr6	82214226	82223620
chr6	82223439	82231842
chr6	82223439	82234255
chr6	82224067	82225647
chr6	82224067	82231842
chr6	82224067	82234255
chr6	82225476	82231842
chr6	82225476	82234255
chr6	82957311	83024038
chr6	82957311	83038485
chr6	82957311	83044659
chr6	83018972	83038485
chr6	83018972	83044659
chr6	83018972	83054238
chr6	83022452	83054238
chr6	83044427	83058022
chr6	83096730	83100886
chr6	83096730	83113421
chr6	83096924	83100886

chr6	83096924	83113421
chr6	83108909	83110314
chr6	83148758	83152367
chr6	83152287	83154043
chr6	83170304	83172059
chr6	83171936	83187075
chr6	83174373	83178756
chr6	83216497	83227477
chr6	83227334	83239636
chr6	83227334	83352139
chr6	83315309	83352139
chr6	83315309	83407901
chr6	83346172	83352139
chr6	83346172	83398516
chr6	83346172	83407901
chr6	83398366	83407901
chr6	83641095	83665581
chr6	83909008	83924592
chr6	83918004	83924592
chr6	83924469	83936376
chr6	83934594	83936376
chr6	84185186	84186623
chr6	84185186	84204096
chr6	84194883	84200905
chr6	84201736	84221171
chr6	8420720	8434430
chr6	84212956	84221171
chr6	84444386	84470904
chr6	84738496	84748087
chr6	84738496	84756869
chr6	84738496	84762748
chr6	85467059	85492177
chr6	85471236	85487489
chr6	85507232	85526237
chr6	85507232	85558060
chr6	85513799	85558060
chr6	85517755	85528362
chr6	85517755	85530275
chr6	85517755	85533800
chr6	85517755	85536924
chr6	85517755	85567577
chr6	85526125	85542043
chr6	85530191	85542043
chr6	85533598	85542043
chr6	85536791	85542043

chr6	85541984	85548376
chr6	85543181	85567577
chr6	85543604	85547226
chr6	85543604	85558060
chr6	85557975	85565419
chr6	85557975	85567577
chr6	85557975	85572374
chr6	85557975	85574378
chr6	85565331	85572374
chr6	85565331	85574378
chr6	85567533	85574378
chr6	85618817	85619417
chr6	85618817	85622687
chr6	85618817	85624112
chr6	85636966	85637356
chr6	85636966	85641451
chr6	85640220	85641451
chr6	87215902	87218731
chr6	87215902	87233527
chr6	87215902	87245644
chr6	87518629	87530942
chr6	87541917	87569590
chr6	87555407	87569590
chr6	87601783	87606021
chr6	87601783	87612248
chr6	87605916	87609229
chr6	87621353	87622013
chr6	87621353	87636486
chr6	87621353	87653249
chr6	87656902	87658018
chr6	87675859	87681763
chr6	87677817	87681763
chr6	88678352	88844521
chr6	88678352	88904955
chr6	88769773	88844521
chr6	88769773	88853764
chr6	88769773	88904955
chr6	88801563	88853764
chr6	88801563	88904955
chr6	88844356	88853764
chr6	88844356	88891915
chr6	88844356	88904955
chr6	88844356	88941180
chr6	88890494	88891915
chr6	88890494	88941180

chr6	88904714	88906440
chr6	88904714	88941180
chr6	88906364	88941180
chr6	88928984	88941180
chr6	89333085	89343756
chr6	89335301	89343756
chr6	89338458	89342455
chr6	89342323	89343756
chr6	89372436	89380367
chr6	89372436	89387590
chr6	89377670	89387590
chr6	89595915	89606105
chr6	89670918	89673462
chr6	89677569	89678745
chr6	89677569	89685973
chr6	89677569	89688808
chr6	89677569	89690143
chr6	89677569	89725396
chr6	89683131	89688808
chr6	89690672	89694183
chr6	89690672	89702061
chr6	89714542	89725396
chr6	89722954	89723619
chr6	89722954	89725396
chr6	89732556	89743714
chr6	89738325	89743714
chr6	89738325	89762530
chr6	89740233	89754230
chr6	89743149	89747470
chr6	89743149	89750532
chr6	89745272	89750532
chr6	89747328	89750532
chr6	89749222	89750532
chr6	89751430	89753622
chr6	89751430	89756390
chr6	89751430	89762530
chr6	89753511	89762530
chr6	89771560	89774733
chr6	89771560	89780293
chr6	89781398	89785126
chr6	89781398	89790401
chr6	89781398	89794207
chr6	89781398	89803554
chr6	89785011	89803554
chr6	89789779	89794801

chr6	89846561	89855544
chr6	89846561	89857199
chr6	89853165	89855544
chr6	89853165	89857199
chr6	89854816	89857199
chr6	90536336	90571807
chr6	90547257	90553586
chr6	90547257	90568623
chr6	90550467	90571807
chr6	90556499	90560214
chr6	90556499	90571807
chr6	90560075	90571807
chr6	90561621	90571807
chr6	96523145	96526435
chr6	96523145	96528632
chr6	96523145	96540655
chr6	97165245	97168240
chr6	97228893	97263448
chr6	97254556	97263448
chr6	98875414	98927822
chr6	98880552	98927822
chr6	98880552	98934879
chr6	98899267	98905670
chr6	98899267	98927822
chr6	98899267	98934879
chr6	98926476	98927822
chr6	99375939	99380341
chr6	99375939	99383824
chr6	99377385	99380341
chr6	99377385	99383824
chr6	99406030	99408271
chr6	99412550	99416428
chr6	99437245	99443662
chr6	99439768	99465136
chr6	99464603	99468618
chr6	99468536	99510230
chr6	99476142	99488820
chr6	99476142	99510230
chr6	99482752	99510230
chr6	99488199	99510230
chr6	99503764	99510230
chr6	99507427	99510230
chr6	99545111	99551895
chr6	99549507	99561681
chr6	99558496	99561681

chr6	99558496	99562948
chr6	99561366	99562948
chr7	100023418	100024307
chr7	100075134	100077433
chr7	100075134	100079656
chr7	100077302	100079656
chr7	100102674	100102960
chr7	100103408	100104154
chr7	100222596	100223698
chr7	100325690	100329019
chr7	100330462	100332581
chr7	100355142	100359537
chr7	100548821	100555735
chr7	100553346	100554258
chr7	100683549	100683918
chr7	100687297	100687616
chr7	100812746	100813208
chr7	100819556	100820296
chr7	100856867	100859161
chr7	100884922	100885370
chr7	101211590	101213204
chr7	101916114	102028145
chr7	101916114	102070417
chr7	101916114	102115273
chr7	102028097	102115273
chr7	102070338	102104459
chr7	102070338	102115273
chr7	102097363	102115273
chr7	102158559	102178657
chr7	102158559	102189871
chr7	102198801	102200172
chr7	102314335	102317395
chr7	102375822	102376322
chr7	102375822	102399648
chr7	102397619	102404683
chr7	102397619	102407498
chr7	102399547	102407498
chr7	102465816	102466270
chr7	102539605	102569924
chr7	102566996	102569924
chr7	102757853	102761411
chr7	102757853	102787469
chr7	102772408	102787469
chr7	102822039	102822440
chr7	102822039	102854860

chr7	102822039	102883685
chr7	102822039	102884312
chr7	102832839	102883467
chr7	102832839	102884312
chr7	102854776	102883467
chr7	102854776	102884312
chr7	102877466	102884312
chr7	102878330	102884312
chr7	102913085	102931933
chr7	102963532	103029418
chr7	103025062	103029418
chr7	103115059	103120223
chr7	103115059	103128792
chr7	103298567	103310475
chr7	103300177	103304490
chr7	103303841	103304490
chr7	103303841	103310475
chr7	103308951	103310475
chr7	103316829	103319678
chr7	103361956	103362758
chr7	104136780	104165282
chr7	104136780	104168525
chr7	104161071	104188381
chr7	104161071	104204234
chr7	104166771	104204234
chr7	104188250	104204234
chr7	105038125	105041023
chr7	105038125	105066807
chr7	105040838	105066807
chr7	105040838	105081797
chr7	105062163	105066807
chr7	105062163	105078963
chr7	105062163	105081797
chr7	105073618	105074815
chr7	105073618	105077433
chr7	105073618	105081797
chr7	105076042	105077193
chr7	105078845	105091314
chr7	105090008	105091314
chr7	105160506	105169265
chr7	105160506	105203785
chr7	105160506	105268869
chr7	105168007	105169265
chr7	105203627	105268869
chr7	105203627	105297533

chr7	105268805	105297533
chr7	105459167	105465414
chr7	105459167	105495253
chr7	105459167	105508544
chr7	105462620	105465414
chr7	105462620	105468463
chr7	105462620	105495253
chr7	105462620	105506273
chr7	105465312	105470848
chr7	105468336	105470848
chr7	105468336	105472193
chr7	105470687	105495253
chr7	105472131	105482440
chr7	105472131	105502564
chr7	105472131	105506273
chr7	105481051	105482440
chr7	105481051	105495253
chr7	105481051	105502564
chr7	105481051	105508544
chr7	105491539	105502564
chr7	105491539	105506056
chr7	105491539	105506273
chr7	105495141	105506273
chr7	105505954	105506273
chr7	105542407	105565648
chr7	105546909	105548710
chr7	105546909	105551707
chr7	105551569	105555227
chr7	105551569	105565648
chr7	105563732	105565648
chr7	106092948	106099282
chr7	106097689	106099282
chr7	107140846	107151023
chr7	107230614	107258383
chr7	107248395	107258383
chr7	107248395	107283732
chr7	107281299	107283732
chr7	107281299	107298346
chr7	107324439	107372760
chr7	107362032	107372760
chr7	107362032	107412632
chr7	107527236	107548332
chr7	107527236	107558115
chr7	107564587	107576592
chr7	107567049	107571266

chr7	107959248	107960649
chr7	107959690	107960649
chr7	107974985	107980811
chr7	10990702	11022979
chr7	10990702	11028818
chr7	10990702	11042814
chr7	11013746	11022979
chr7	11013746	11028818
chr7	11013746	11038855
chr7	11013746	11051780
chr7	11013746	11062085
chr7	11022867	11051780
chr7	11028680	11037091
chr7	11028680	11051780
chr7	11028680	11062085
chr7	11036984	11051780
chr7	11038759	11111467
chr7	11040671	11051780
chr7	11040671	11062085
chr7	11042682	11062085
chr7	11051611	11062085
chr7	11051611	11111467
chr7	11061790	11062085
chr7	11061790	11111467
chr7	110886592	111561923
chr7	111487237	111521449
chr7	111487237	111561923
chr7	111808820	111834686
chr7	111935539	111945798
chr7	111977131	112004131
chr7	111989014	112004131
chr7	112295868	112318273
chr7	112455762	112456086
chr7	112455762	112468115
chr7	112456001	112462378
chr7	112456001	112472861
chr7	112467980	112472861
chr7	112895568	112915447
chr7	114942274	114979781
chr7	116249019	116250496
chr7	116739949	116741025
chr7	116740851	116769791
chr7	116758458	116771654
chr7	117099761	117131960
chr7	117099761	117138532

chr7	117136080	117170976
chr7	120838776	120840109
chr7	120951163	120955624
chr7	122092842	122133741
chr7	122113117	122133741
chr7	122115073	122116957
chr7	122129360	122133741
chr7	12214808	12218521
chr7	12214808	12224385
chr7	12214808	12230438
chr7	12224225	12229819
chr7	122379367	122388738
chr7	122379367	122393582
chr7	122379367	122416164
chr7	122554549	122581290
chr7	122554549	122621717
chr7	122581178	122621717
chr7	122615180	122629328
chr7	122615180	122663569
chr7	123689041	123692867
chr7	124840972	124846998
chr7	124842806	124859112
chr7	124846941	124892380
chr7	127694827	127707647
chr7	127698874	127707647
chr7	127698874	127721400
chr7	127701162	127707647
chr7	127701162	127721400
chr7	127704838	127721400
chr7	127704838	127807573
chr7	127707556	127721400
chr7	127807483	127844424
chr7	127807483	127888012
chr7	127807483	127904819
chr7	127807483	127929329
chr7	127844323	127888012
chr7	127887901	127929329
chr7	128321265	128337202
chr7	128330818	128336042
chr7	128335542	128336042
chr7	128400094	128401116
chr7	128758870	128759852
chr7	128835325	128855314
chr7	128967279	128972582
chr7	128967279	128992090

chr7	128972425	128980031
chr7	128978982	128980031
chr7	128982247	128986920
chr7	128986728	128993914
chr7	128989960	128993914
chr7	128997388	129001234
chr7	128997388	129015135
chr7	128997388	129018157
chr7	129000428	129005159
chr7	129005015	129018157
chr7	129014978	129018157
chr7	129014978	129041717
chr7	129203383	129206587
chr7	129205202	129206587
chr7	129205609	129206369
chr7	129379637	129413688
chr7	129379640	129389734
chr7	129400289	129406466
chr7	129630077	129671543
chr7	129630077	129690546
chr7	129630077	129727365
chr7	129657342	129671543
chr7	129657342	129690546
chr7	129657345	129671543
chr7	129657345	129690546
chr7	129657345	129711576
chr7	129657345	129727365
chr7	129671428	129690546
chr7	129671428	129727365
chr7	129690405	129711576
chr7	129709074	129727365
chr7	129710373	129711576
chr7	129710373	129727365
chr7	129839206	129880971
chr7	129857510	129880971
chr7	130022318	130029029
chr7	130026157	130029029
chr7	130026157	130039547
chr7	130026157	130049144
chr7	130039463	130049144
chr7	130040950	130049144
chr7	130096920	130127451
chr7	130116444	130122202
chr7	130116444	130125931
chr7	130116444	130127451

chr7	130116444	130129576
chr7	130120748	130122202
chr7	130170399	130181719
chr7	130173631	130175609
chr7	130173631	130178527
chr7	130181622	130192823
chr7	130184183	130192823
chr7	130185191	130192823
chr7	130201849	130202663
chr7	130201849	130203846
chr7	130201849	130204110
chr7	130203787	130204110
chr7	130547673	130552430
chr7	130610952	130617065
chr7	130610952	130652948
chr7	130610952	130667534
chr7	130945719	131052555
chr7	130967388	131052555
chr7	130984052	131052555
chr7	131375423	131388972
chr7	131375423	131399433
chr7	131375423	131411383
chr7	131375423	131414710
chr7	131375423	131429145
chr7	131375423	131445903
chr7	131387119	131399433
chr7	131387119	131411383
chr7	131387119	131414710
chr7	131387119	131429145
chr7	131387119	131445903
chr7	131387119	131470944
chr7	131397266	131414710
chr7	131411305	131429145
chr7	131411305	131445903
chr7	131429032	131443702
chr7	131429032	131445903
chr7	131429032	131478677
chr7	131437784	131445903
chr7	131443480	131478677
chr7	131463216	131478677
chr7	132796441	132885745
chr7	132838398	132885745
chr7	132975168	133024627
chr7	132975168	133070229
chr7	133274981	133289116

chr7	133288921	133317390
chr7	133305876	133317390
chr7	133317283	133375002
chr7	133630044	133938069
chr7	133817324	133917738
chr7	134137022	134163540
chr7	134137022	134174636
chr7	134137022	134178609
chr7	134137022	134183853
chr7	134137022	134191751
chr7	134137022	134201576
chr7	134139435	134221918
chr7	134148237	134174636
chr7	134174555	134183853
chr7	134304804	134309479
chr7	135197992	135209755
chr7	135410523	135422353
chr7	135410523	135438423
chr7	135413487	135422353
chr7	135571104	135591600
chr7	135577795	135593192
chr7	135584831	135587992
chr7	135587574	135607371
chr7	135592986	135594729
chr7	135600869	135604460
chr7	135619422	135626361
chr7	135622776	135630470
chr7	135625163	135630470
chr7	135635580	135638683
chr7	135643191	135646231
chr7	137619823	137690002
chr7	137678556	137690002
chr7	137901353	137916012
chr7	138504289	138515359
chr7	138519188	138551180
chr7	138525240	138554966
chr7	138525240	138573642
chr7	138576372	138579532
chr7	138861138	138869761
chr7	138861138	138881584
chr7	138867974	138869761
chr7	138894341	138911323
chr7	138894341	138912460
chr7	138908990	138912460
chr7	138908990	138919438

chr7	138911145	138912460
chr7	139026707	139034678
chr7	139053450	139061138
chr7	139055204	139061138
chr7	139061035	139074030
chr7	13906429	13911307
chr7	139073855	139089759
chr7	139083779	139089759
chr7	139142255	139148377
chr7	139166839	139178598
chr7	139237004	139252057
chr7	139237004	139259370
chr7	139237004	139266403
chr7	139237004	139273410
chr7	139251955	139266403
chr7	139266332	139272440
chr7	139269393	139276147
chr7	139269393	139279360
chr7	13931501	13977480
chr7	139376061	139417729
chr7	139398598	139407350
chr7	139402136	139407350
chr7	139405643	139412580
chr7	139407173	139409654
chr7	139407173	139412580
chr7	139620400	139626785
chr7	139620400	139629039
chr7	139780739	139911321
chr7	140034234	140047007
chr7	140034234	140058034
chr7	140041643	140047007
chr7	140041643	140058034
chr7	140054661	140058034
chr7	140096554	140111184
chr7	140111094	140120529
chr7	140119112	140120529
chr7	140127441	140129653
chr7	140355667	140358785
chr7	140355667	140369682
chr7	140459006	140472011
chr7	140702865	140704963
chr7	140739811	140754233
chr7	140739811	140787584
chr7	140749286	140754233
chr7	140749286	140794467

chr7	140776911	140781693
chr7	140776911	140783157
chr7	140776911	140787584
chr7	140776911	140794467
chr7	140776911	140808995
chr7	140781575	140783157
chr7	140781575	140794467
chr7	140794307	140808995
chr7	140794307	140834872
chr7	140794307	140850212
chr7	140800361	140808995
chr7	140807959	140808995
chr7	141596561	141611287
chr7	141601204	141621801
chr7	141601204	141633980
chr7	141601204	141641908
chr7	141611194	141621801
chr7	141633900	141641908
chr7	141633900	141649333
chr7	141636959	141641908
chr7	141636959	141649333
chr7	141685477	141686658
chr7	143292299	143292698
chr7	1438096	1450288
chr7	144623165	144648879
chr7	1473071	1473693
chr7	1486624	1489682
chr7	148756982	148759638
chr7	148766560	148767749
chr7	148809309	148847305
chr7	148817876	148819687
chr7	148826468	148847305
chr7	148832633	148847305
chr7	148846469	148847305
chr7	148846496	148847305
chr7	149011845	149021147
chr7	149014903	149021147
chr7	149018991	149021147
chr7	149074385	149080717
chr7	149153944	149166930
chr7	149166157	149166930
chr7	149198332	149213814
chr7	149206695	149207470
chr7	149206695	149213814
chr7	149266334	149278527

chr7	149476921	149494503
chr7	151117094	151118632
chr7	151123793	151128684
chr7	151470570	151491014
chr7	151477332	151491014
chr7	151560523	151574949
chr7	151564077	151570225
chr7	151564077	151572709
chr7	151781151	151786541
chr7	152094189	152110645
chr7	152103111	152113398
chr7	152108037	152113398
chr7	152185557	152199459
chr7	152194008	152199459
chr7	152194008	152238826
chr7	152199278	152207428
chr7	152205105	152207428
chr7	152220522	152222066
chr7	152220522	152222682
chr7	152220522	152224616
chr7	152220522	152238826
chr7	152229922	152238826
chr7	152230219	152238826
chr7	152235816	152238826
chr7	152238706	152250966
chr7	152247901	152252715
chr7	152263015	152265209
chr7	152263015	152315338
chr7	152263015	152358675
chr7	152273704	152315338
chr7	152309965	152315338
chr7	152309965	152330739
chr7	152309965	152358675
chr7	152330600	152358675
chr7	152783186	152801731
chr7	152783186	152825122
chr7	152800530	152801731
chr7	152800530	152825122
chr7	154954254	154955631
chr7	154960892	154962458
chr7	154975695	154998784
chr7	154983218	154998784
chr7	155301565	155302417
chr7	155665174	155678684
chr7	155665174	155680908

chr7	155672866	155680908
chr7	155672866	155707068
chr7	155678607	155680908
chr7	155700772	155707068
chr7	155700772	155718443
chr7	155706859	155711455
chr7	155706859	155718443
chr7	155763811	155766655
chr7	156724111	156728720
chr7	156724111	156734257
chr7	156724111	156756465
chr7	156724111	156763795
chr7	156724111	156826744
chr7	156734176	156826744
chr7	156756392	156763795
chr7	156756392	156826744
chr7	156762133	156796492
chr7	156762133	156826744
chr7	156796388	156826744
chr7	156796388	156836885
chr7	156826604	156836885
chr7	156959850	156964026
chr7	156966269	156967092
chr7	157163809	157170450
chr7	157163809	157175034
chr7	157163809	157231327
chr7	157169047	157187021
chr7	157169047	157216971
chr7	157181517	157187021
chr7	157183877	157201807
chr7	157201720	157207935
chr7	157201720	157220776
chr7	157201720	157225539
chr7	157201720	157231327
chr7	157201720	157248580
chr7	157207397	157216971
chr7	157207702	157225539
chr7	157216866	157225539
chr7	157216866	157231327
chr7	157220688	157223351
chr7	157220688	157231327
chr7	157220688	157248580
chr7	157220688	157254310
chr7	157220688	157257044
chr7	157223253	157225539

chr7	157223253	157231327
chr7	157223253	157248580
chr7	157231079	157248580
chr7	157358546	157385611
chr7	157382245	157385611
chr7	1574714	1575888
chr7	158644288	158646563
chr7	158644288	158664750
chr7	158664183	158687442
chr7	158671513	158675616
chr7	158671513	158675656
chr7	158671513	158687442
chr7	158675476	158689953
chr7	158679959	158689953
chr7	158679959	158690722
chr7	158686171	158689953
chr7	158686171	158690722
chr7	158692841	158693497
chr7	158735502	158739121
chr7	158735502	158748280
chr7	158735502	158788417
chr7	158739022	158748280
chr7	158739022	158767774
chr7	158748193	158767774
chr7	158748193	158793726
chr7	158748193	158799072
chr7	158759485	158764853
chr7	158759485	158767774
chr7	158759485	158773396
chr7	158759485	158793726
chr7	158759485	158799072
chr7	158761495	158799072
chr7	158773340	158799072
chr7	158788003	158799072
chr7	158788344	158799072
chr7	158793649	158798076
chr7	158879683	158906091
chr7	158901738	158906091
chr7	158911549	158918869
chr7	16624982	16636455
chr7	16665436	16674588
chr7	16665436	16682845
chr7	16665436	16689906
chr7	16665436	16697061
chr7	16674411	16681404

chr7	16674411	16682845
chr7	16681300	16689906
chr7	16681300	16697061
chr7	16682779	16697061
chr7	16685904	16689906
chr7	16685904	16697061
chr7	16689796	16697061
chr7	16694833	16697061
chr7	16698047	16704669
chr7	16776210	16779116
chr7	17309935	17322607
chr7	17309935	17335786
chr7	17796826	17826091
chr7	17799008	17830047
chr7	17814833	17816289
chr7	17814833	17830047
chr7	17814833	17850964
chr7	17814833	17868490
chr7	17814833	17875790
chr7	17821508	17850435
chr7	17830009	17845694
chr7	17830009	17873616
chr7	17834051	17840000
chr7	17834051	17850964
chr7	17834051	17873616
chr7	17845594	17850964
chr7	17845594	17868490
chr7	17845594	17875790
chr7	17845594	17897446
chr7	17850346	17875790
chr7	17850825	17875790
chr7	17868406	17875790
chr7	17868406	17890484
chr7	17868406	17893434
chr7	17868406	17897446
chr7	17873527	17875790
chr7	17875479	17875790
chr7	17875479	17893434
chr7	17890362	17893434
chr7	17890362	17897446
chr7	18585280	18594029
chr7	18585280	18666476
chr7	18644670	18648683
chr7	18644670	18666476
chr7	18647784	18666476

chr7	18727579	18749138
chr7	1898199	1936897
chr7	1898199	1957719
chr7	1898199	1980541
chr7	1898199	2014642
chr7	1898199	2069338
chr7	1936686	1957719
chr7	1936686	1980541
chr7	1936686	2002121
chr7	1957628	1980541
chr7	1957628	2002121
chr7	1957628	2014642
chr7	1980452	2014642
chr7	1980452	2069338
chr7	1980452	2149238
chr7	2002064	2014642
chr7	2002064	2069338
chr7	2014501	2069338
chr7	2014501	2219456
chr7	2069193	2219456
chr7	2069193	2222754
chr7	208897	248430
chr7	21477078	21482123
chr7	2149151	2219456
chr7	2149151	2222754
chr7	2213211	2218043
chr7	2213211	2219456
chr7	2213211	2222754
chr7	2213211	2225550
chr7	22150406	22160615
chr7	22219865	22267012
chr7	22219865	22291241
chr7	2222574	2230727
chr7	22266963	22318037
chr7	22291174	22315476
chr7	22291174	22318037
chr7	2229983	2230724
chr7	2229983	2230727
chr7	2229983	2230764
chr7	22308338	22318037
chr7	2244562	2250002
chr7	22492564	22494886
chr7	2256873	2278305
chr7	2263229	2269639
chr7	2264297	2278305

chr7	2269558	2275229
chr7	2269558	2278305
chr7	2271849	2278305
chr7	2275111	2278305
chr7	22960255	22964531
chr7	22960255	22977421
chr7	22960255	22978448
chr7	22960255	22984045
chr7	22960255	22991139
chr7	22961234	22991139
chr7	22964414	22977421
chr7	22964414	22991139
chr7	22976209	22977421
chr7	22976209	22984045
chr7	22976209	22991139
chr7	22977340	22991139
chr7	22983943	22991139
chr7	23117833	23125172
chr7	23123776	23125172
chr7	23123776	23140944
chr7	23123776	23144025
chr7	23123776	23152209
chr7	23140768	23144025
chr7	23140768	23152209
chr7	23185069	23187146
chr7	23185069	23196766
chr7	23317638	23346062
chr7	23317638	23351586
chr7	23342063	23346062
chr7	23342063	23351586
chr7	23342063	23361741
chr7	23345939	23351586
chr7	23345939	23361741
chr7	23351304	23468542
chr7	23505745	23507535
chr7	23505745	23513082
chr7	23505745	23521840
chr7	23505745	23522432
chr7	23507419	23513082
chr7	23507419	23516528
chr7	23521706	23522432
chr7	2360709	2364529
chr7	2362644	2363760
chr7	2362644	2364529
chr7	2363631	2364529

chr7	2363631	2367045
chr7	2364371	2366448
chr7	2364371	2367045
chr7	2364371	2369682
chr7	2364371	2371849
chr7	2366316	2369682
chr7	2366316	2371849
chr7	2366524	2379511
chr7	2369471	2371849
chr7	2371776	2372795
chr7	2374527	2377075
chr7	2375388	2379242
chr7	2375388	2379511
chr7	2376949	2379511
chr7	24620051	24641868
chr7	24620051	24650712
chr7	24620051	24663721
chr7	24620051	24666089
chr7	24620051	24668660
chr7	24620051	24680520
chr7	24623665	24641868
chr7	24623665	24650712
chr7	24623665	24663721
chr7	24623665	24668660
chr7	24623665	24680520
chr7	246414	248430
chr7	24641715	24650712
chr7	24641715	24663721
chr7	24641715	24666089
chr7	24641715	24668660
chr7	24641715	24680520
chr7	24649611	24650712
chr7	24649611	24668660
chr7	24663589	24666089
chr7	24679130	24680520
chr7	24744561	24749793
chr7	24804314	24806902
chr7	24804314	24809951
chr7	24804314	24834736
chr7	24815058	24816688
chr7	24815058	24820238
chr7	24815058	24834736
chr7	24816609	24849176
chr7	24830767	24842413
chr7	24830767	24852634

chr7	24830767	24863599
chr7	24834485	24849176
chr7	24840689	24849176
chr7	24849068	24861769
chr7	24861612	24872069
chr7	24861612	24892621
chr7	24863199	24870845
chr7	24863495	24872069
chr7	24870731	24872069
chr7	25142240	25142814
chr7	25151595	25155192
chr7	25151595	25158530
chr7	25151595	25161260
chr7	25151595	25168465
chr7	2524694	2526921
chr7	2567115	2572326
chr7	2567115	2573488
chr7	2567115	2601464
chr7	2568953	2573488
chr7	2571525	2572326
chr7	26196400	26197732
chr7	26196556	26197866
chr7	26196806	26197732
chr7	26202970	26208555
chr7	2646852	2649988
chr7	26670090	26727006
chr7	26684735	26725986
chr7	26684735	26727006
chr7	26725427	26739964
chr7	26844029	26854890
chr7	27531191	27649633
chr7	27629370	27632445
chr7	27629370	27649633
chr7	27769675	27796219
chr7	27785162	27785489
chr7	27787417	27800090
chr7	27794322	27800090
chr7	27796115	27800090
chr7	27796115	27827820
chr7	2794927	2814389
chr7	29926451	29936721
chr7	29926451	29969068
chr7	29936555	29955360
chr7	29936555	29969068
chr7	29940681	29944179

chr7	29940681	29955360
chr7	29943976	29969068
chr7	30018995	30022816
chr7	30020212	30022816
chr7	30045084	30049382
chr7	30045084	30055342
chr7	30045084	30062742
chr7	30323641	30362456
chr7	30455136	30457042
chr7	30598795	30603572
chr7	30598795	30632437
chr7	30603033	30622462
chr7	30778431	30782212
chr7	30836657	30840848
chr7	32543140	32552295
chr7	32543140	32554596
chr7	32543140	32558628
chr7	32543140	32559464
chr7	32544693	32554596
chr7	32548846	32552295
chr7	32548846	32554596
chr7	32558558	32559464
chr7	32570019	32576072
chr7	32620403	32635351
chr7	32620403	32639365
chr7	32620403	32679130
chr7	32632542	32639365
chr7	32632542	32679130
chr7	32655986	32679130
chr7	32675931	32679130
chr7	32974616	32980553
chr7	32974616	32988652
chr7	33015669	33026915
chr7	33015669	33035988
chr7	33017438	33022099
chr7	33026816	33035988
chr7	33146241	33155702
chr7	33146241	33177591
chr7	33344580	33388144
chr7	33349067	33388144
chr7	33351218	33367862
chr7	33351218	33388144
chr7	33357854	33383838
chr7	33357854	33388144
chr7	33357854	33534176

chr7	33367766	33388144
chr7	33383665	33388144
chr7	33505462	33604975
chr7	34078856	34086092
chr7	34966893	34973605
chr7	34966893	34989941
chr7	34966893	35018596
chr7	34969432	35018596
chr7	34973513	35018596
chr7	34989883	35018596
chr7	35141032	35150274
chr7	35638349	35673278
chr7	35667433	35673278
chr7	35670214	35673278
chr7	35781208	35794022
chr7	35863551	35873775
chr7	35879822	35883987
chr7	35879822	35885879
chr7	35879822	35890793
chr7	3619079	3642105
chr7	36281114	36287777
chr7	36281114	36297187
chr7	36399078	36415884
chr7	36406180	36411166
chr7	36410513	36411166
chr7	36410513	36420314
chr7	36420596	36427028
chr7	36423816	36424742
chr7	38207446	38219660
chr7	38214356	38219660
chr7	38789800	38796864
chr7	38789800	38830328
chr7	38817816	38830328
chr7	38862544	38869253
chr7	39686630	39690590
chr7	39686630	39696859
chr7	39690381	39696859
chr7	39987598	39997664
chr7	39987598	39999500
chr7	39987598	40002031
chr7	39987598	40047877
chr7	39997493	40002031
chr7	39997493	40047877
chr7	40045835	40063100
chr7	40045835	40088331

chr7	40062825	40088331
chr7	40180946	40189594
chr7	40180946	40237726
chr7	4049347	4051830
chr7	4049347	4077189
chr7	4049347	4079584
chr7	4067837	4079584
chr7	4076997	4079584
chr7	4174221	4178586
chr7	41972336	41978748
chr7	42023467	42026412
chr7	42023467	42048696
chr7	42040037	42048696
chr7	42148225	42223295
chr7	42926535	42927459
chr7	43595900	43608400
chr7	43595900	43619723
chr7	43639448	43640649
chr7	43639448	43645399
chr7	43639448	43647634
chr7	43881658	43887816
chr7	43965866	43973323
chr7	44013592	44014783
chr7	44013592	44016523
chr7	44014605	44016523
chr7	44392296	44404583
chr7	44392296	44427703
chr7	44404439	44427703
chr7	44404439	44485284
chr7	44624316	44647534
chr7	44624316	44647759
chr7	44645326	44647759
chr7	44666735	44681848
chr7	44673786	44675268
chr7	44674410	44675268
chr7	44674410	44676149
chr7	44674410	44681848
chr7	44693824	44694576
chr7	44700140	44701615
chr7	44835528	44843354
chr7	45736786	45759174
chr7	45744996	45759174
chr7	45747214	45751646
chr7	45747214	45759174
chr7	45751528	45759174

chr7	45914804	45916667
chr7	47291954	47297213
chr7	47297081	47305003
chr7	47344754	47346356
chr7	47368364	47400914
chr7	47396799	47400914
chr7	47396799	47415206
chr7	4740837	4755383
chr7	4740837	4757187
chr7	47435281	47442055
chr7	47435281	47529147
chr7	4754458	4757187
chr7	4754458	4761288
chr7	4756993	4759595
chr7	47881908	47888150
chr7	47975612	47978816
chr7	47994314	47996146
chr7	4815277	4817351
chr7	4976300	4989177
chr7	4983670	4989177
chr7	5048032	5057779
chr7	5057294	5057779
chr7	50669721	50674658
chr7	50669721	50703908
chr7	50669721	50756056
chr7	50674435	50703908
chr7	510808	512455
chr7	510808	517490
chr7	51184101	51219944
chr7	5199575	5214704
chr7	5199575	5217187
chr7	5214534	5218014
chr7	5216562	5217187
chr7	5287805	5291821
chr7	5287808	5291821
chr7	5298987	5300662
chr7	5324213	5325248
chr7	5324213	5333049
chr7	5324213	5394595
chr7	5345561	5352094
chr7	5359397	5362825
chr7	5374054	5378024
chr7	5376846	5377576
chr7	5491230	5505630
chr7	55157662	55165437

chr7	55163732	55165437
chr7	55171174	55174820
chr7	55172982	55191874
chr7	55172982	55192841
chr7	5528003	5529663
chr7	55391792	55401320
chr7	55391792	55425430
chr7	55492281	55497690
chr7	55492281	55521130
chr7	55923161	55923699
chr7	55978125	55981567
chr7	55978125	55984878
chr7	55982209	55983868
chr7	55982209	55984878
chr7	55982209	55994988
chr7	55982209	55997449
chr7	55984846	55997449
chr7	5603256	5604030
chr7	5603495	5604030
chr7	56052421	56056414
chr7	56054368	56056414
chr7	56057992	56058702
chr7	56057992	56060416
chr7	56068481	56073111
chr7	56083277	56083715
chr7	5641153	5652510
chr7	5641153	5739352
chr7	5711760	5716766
chr7	5711760	5730817
chr7	5711760	5739352
chr7	5712714	5721172
chr7	5715052	5716766
chr7	5715052	5725438
chr7	5715052	5730817
chr7	5721032	5741644
chr7	5725323	5730817
chr7	5725323	5741644
chr7	5729431	5741644
chr7	5729431	5741815
chr7	5739275	5741644
chr7	5739275	5741815
chr7	579255	596304
chr7	579255	607452
chr7	579255	711527
chr7	5901656	5912954

chr7	596145	607452
chr7	596145	711527
chr7	5978595	5995633
chr7	5982823	6002636
chr7	5986758	5992057
chr7	5991972	5999275
chr7	5997325	6002636
chr7	5997325	6006031
chr7	6015145	6018045
chr7	6026727	6029032
chr7	6028614	6029032
chr7	6028614	6050045
chr7	6037423	6050045
chr7	6040891	6050045
chr7	6042932	6050045
chr7	6044561	6050045
chr7	6046070	6047091
chr7	6046070	6050045
chr7	6111124	6115523
chr7	6111124	6135951
chr7	6111124	6145656
chr7	6111124	6146248
chr7	6135840	6145656
chr7	6135840	6147892
chr7	6139091	6144196
chr7	6139091	6145656
chr7	6139091	6146248
chr7	6142931	6145656
chr7	6149582	6150506
chr7	6170534	6171314
chr7	6170534	6177941
chr7	6177822	6187721
chr7	6187049	6190531
chr7	6387211	6392041
chr7	6391923	6400188
chr7	6412810	6416921
chr7	6412810	6426114
chr7	6416626	6421804
chr7	6421726	6426114
chr7	6421726	6430607
chr7	6421726	6432959
chr7	6421726	6436533
chr7	6424751	6426114
chr7	6424751	6436533
chr7	6425987	6432959

chr7	6425987	6435020
chr7	6425987	6436533
chr7	6430479	6435020
chr7	6430479	6436533
chr7	6432836	6435020
chr7	6432836	6436533
chr7	6432836	6446104
chr7	6434761	6436533
chr7	64543706	64544432
chr7	6466070	6469754
chr7	64691247	64691960
chr7	6469595	6474284
chr7	64917029	64918292
chr7	65066252	65067050
chr7	65726770	65727108
chr7	65754526	65756574
chr7	65755779	65756574
chr7	65755779	65759998
chr7	65757982	65759271
chr7	65757982	65760556
chr7	6578500	6585260
chr7	6578565	6585260
chr7	6580554	6583431
chr7	6580554	6585260
chr7	6581606	6582251
chr7	6581606	6583431
chr7	6591702	6602140
chr7	6594434	6602140
chr7	65964322	65967907
chr7	65964322	65970366
chr7	65964322	65974704
chr7	65974294	65974704
chr7	65974294	65975071
chr7	65979398	65980409
chr7	6599828	6602140
chr7	66081802	66083174
chr7	66086584	66087791
chr7	66087728	66092086
chr7	66088806	66089695
chr7	66127703	66130842
chr7	66127703	66134374
chr7	66127703	66145500
chr7	66130126	66145500
chr7	66130743	66134374
chr7	66130743	66145500

chr7	66240324	66286709
chr7	66286510	66356871
chr7	6642963	6644593
chr7	66541241	66545686
chr7	66560578	66573550
chr7	66573384	66576929
chr7	66573384	66576932
chr7	66768830	66775393
chr7	66768830	66799414
chr7	66771882	66775393
chr7	66771882	66783841
chr7	66771882	66795592
chr7	66771882	66797506
chr7	66771882	66799414
chr7	66771882	66805396
chr7	66775226	66783841
chr7	66775226	66799414
chr7	66783674	66799414
chr7	66795510	66797506
chr7	66795510	66799414
chr7	66795510	66805396
chr7	66797373	66799414
chr7	66797373	66805396
chr7	66799322	66805396
chr7	66941847	66945261
chr7	66941847	66948694
chr7	66941847	66951135
chr7	66944975	66948694
chr7	66950951	66953369
chr7	6697338	6697811
chr7	6697338	6705223
chr7	66991136	66994341
chr7	66993216	66994341
chr7	66998064	67018143
chr7	67009582	67018143
chr7	67009582	67025022
chr7	67014366	67025022
chr7	67017852	67055887
chr7	67024899	67067403
chr7	67024899	67083539
chr7	67049948	67067403
chr7	67055834	67067403
chr7	67055834	67083539
chr7	67055834	67098718
chr7	67055834	67117618

chr7	67067284	67083539
chr7	67067284	67098718
chr7	67067284	67117618
chr7	67098540	67117618
chr7	67183125	67195337
chr7	67295617	67297303
chr7	67295617	67299424
chr7	67305986	67309715
chr7	6735304	6737936
chr7	6735304	6741852
chr7	6737075	6741852
chr7	6737075	6746188
chr7	6741684	6746188
chr7	6741684	6747205
chr7	677228	680726
chr7	677228	711527
chr7	6804937	6824737
chr7	72694686	72713798
chr7	72713620	72728931
chr7	72728821	72744601
chr7	72830691	72831798
chr7	72890626	72891110
chr7	729662	775162
chr7	73182076	73183298
chr7	73459535	73463099
chr7	73459535	73470483
chr7	73462921	73470483
chr7	73465438	73466401
chr7	73469516	73470483
chr7	73539881	73543644
chr7	73686635	73686900
chr7	73686635	73687095
chr7	73708588	73709122
chr7	740818	741465
chr7	740818	763974
chr7	74219743	74220734
chr7	74235531	74249220
chr7	74239937	74246763
chr7	74239937	74249118
chr7	74239937	74252498
chr7	74243145	74249118
chr7	74356409	74357477
chr7	74397073	74401567
chr7	74539878	74545809
chr7	74539878	74547286

chr7	74589850	74591017
chr7	74689123	74716950
chr7	74698960	74706433
chr7	74705163	74711109
chr7	74705163	74718941
chr7	74705163	74728896
chr7	74733922	74738103
chr7	74884143	74884524
chr7	75017597	75020149
chr7	75052710	75057616
chr7	75061206	75070769
chr7	75066663	75070769
chr7	75197947	75209559
chr7	75437514	75441647
chr7	754588	763974
chr7	754588	775162
chr7	75474703	75475188
chr7	75555415	75558255
chr7	75568198	75586833
chr7	7583117	7595132
chr7	7588497	7596461
chr7	7594979	7596461
chr7	75972412	75983636
chr7	75987359	75988516
chr7	761752	763974
chr7	761752	770618
chr7	76480420	76492253
chr7	770470	775162
chr7	770470	780144
chr7	77274481	77280653
chr7	77278751	77280653
chr7	77376847	77404615
chr7	77376847	77406105
chr7	77396981	77406105
chr7	774047	775162
chr7	77765494	77773314
chr7	77765494	77778446
chr7	77920278	77922778
chr7	78343777	78346043
chr7	79454885	79458999
chr7	8003907	8055549
chr7	8003907	8060248
chr7	8003907	8071131
chr7	80188950	80203832
chr7	80188950	80212869

chr7	80199224	80203832
chr7	80199224	80212869
chr7	8022482	8071131
chr7	80748897	80818418
chr7	80758330	80810701
chr7	80761615	80800826
chr7	80761615	80828745
chr7	80765154	80805758
chr7	80765154	80828745
chr7	80789305	80805758
chr7	80789305	80810701
chr7	80789305	80828745
chr7	80798091	80805758
chr7	80798091	80810701
chr7	80798091	80818418
chr7	80810610	80828745
chr7	80818298	80828745
chr7	81959274	81965665
chr7	82032796	82084900
chr7	82060427	82084900
chr7	82060427	82117173
chr7	82064303	82117173
chr7	82117043	82170609
chr7	8218304	8235940
chr7	8218304	8236005
chr7	82822494	82841509
chr7	832504	842130
chr7	832504	843232
chr7	832504	843520
chr7	83466481	83490274
chr7	838797	843232
chr7	838797	843520
chr7	85006801	85022613
chr7	85012781	85022613
chr7	85018251	85037033
chr7	85036888	85042285
chr7	85036888	85055859
chr7	85036888	85068284
chr7	85055716	85068284
chr7	85097804	85121931
chr7	851383	861464
chr7	851383	866067
chr7	851949	860382
chr7	86914722	86919310
chr7	86925527	86942104

chr7	86925527	86945046
chr7	86925527	86947922
chr7	86938125	86945046
chr7	86938125	86947922
chr7	86938125	86965024
chr7	87163494	87165050
chr7	87163494	87174669
chr7	87164933	87179702
chr7	87182227	87185980
chr7	87361389	87377450
chr7	87391588	87393067
chr7	87739790	87741579
chr7	87846948	87858751
chr7	87846948	87860640
chr7	87854203	87860640
chr7	87878052	87888059
chr7	87884978	87888059
chr7	87884978	87897339
chr7	87884978	87900878
chr7	886055	887519
chr7	886055	893971
chr7	890927	893971
chr7	90225049	90232484
chr7	90352815	90355230
chr7	90372154	90378211
chr7	90372154	90383079
chr7	90374301	90378211
chr7	904125	926644
chr7	90604217	90726812
chr7	90726566	90747775
chr7	90726566	90790652
chr7	90726566	90863269
chr7	90747680	90790652
chr7	90747680	90863269
chr7	90747680	90899353
chr7	90747680	90917724
chr7	90955696	90984241
chr7	91992157	91993055
chr7	919967	926644
chr7	919967	935505
chr7	92012428	92023009
chr7	92012428	92031604
chr7	92022237	92023009
chr7	92040673	92052958
chr7	92042667	92052958

chr7	92117043	92118615
chr7	92117043	92131872
chr7	92123119	92131872
chr7	92123733	92129056
chr7	92126252	92127631
chr7	92126252	92131872
chr7	92201306	92222978
chr7	92213194	92214777
chr7	92213194	92226682
chr7	92213194	92236542
chr7	92225719	92234923
chr7	92234448	92242137
chr7	92294888	92319512
chr7	92294888	92327900
chr7	92294888	92343232
chr7	92294888	92352642
chr7	92294888	92362273
chr7	92294888	92371607
chr7	92307358	92362273
chr7	92319329	92327900
chr7	92319329	92352642
chr7	92319329	92371607
chr7	92343023	92352642
chr7	92343023	92362273
chr7	92343023	92371607
chr7	92344977	92362273
chr7	92350949	92362273
chr7	92448751	92454685
chr7	92489292	92489911
chr7	92494486	92501673
chr7	92511579	92513967
chr7	92517275	92518255
chr7	92517275	92519078
chr7	92575770	92578393
chr7	926552	935505
chr7	92774695	92833690
chr7	93239865	93253931
chr7	93239865	93259632
chr7	93252652	93272733
chr7	93252652	93276305
chr7	93252652	93311272
chr7	93252652	93323732
chr7	93253859	93271262
chr7	93256508	93259632
chr7	93257393	93259632

chr7	93258356	93272733
chr7	93271219	93297243
chr7	93271219	93311272
chr7	93276164	93323732
chr7	93291702	93297243
chr7	93291702	93311272
chr7	93291702	93334197
chr7	93303459	93334197
chr7	93308823	93334197
chr7	93334116	93341575
chr7	94537471	94554575
chr7	94549532	94554575
chr7	94598774	94603452
chr7	94598774	94628359
chr7	94598774	94629841
chr7	94618757	94629841
chr7	96076056	96080488
chr7	96170044	96193183
chr7	96184276	96208977
chr7	96277195	96296951
chr7	97855352	97859398
chr7	97856689	97869179
chr7	98137314	98154876
chr7	98137314	98159425
chr7	98137314	98171674
chr7	98141396	98159425
chr7	98141396	98171674
chr7	98185050	98190877
chr7	98186876	98190877
chr7	98190727	98194572
chr7	98228013	98229166
chr7	98230960	98233911
chr7	98240850	98241244
chr7	98304195	98320298
chr7	98307688	98310592
chr7	98881974	98893881
chr7	98890334	98893881
chr7	98890334	98897866
chr7	98910055	98911271
chr7	98912021	98917679
chr7	98915722	98917679
chr7	98925111	98927366
chr7	98933240	98948685
chr7	98937155	98937820
chr7	98948220	98948685

chr7	98961260	98962427
chr7	98962301	98971945
chr7	98964628	98971945
chr7	98967040	98967698
chr7	98967040	98971945
chr7	98976148	98978904
chr7	99030594	99033039
chr7	99047683	99052446
chr7	99051356	99057551
chr7	99333324	99344515
chr7	99333324	99348959
chr7	99338180	99348959
chr7	99385701	99394119
chr7	99386684	99388261
chr7	99386684	99390012
chr7	99388038	99390012
chr7	99390892	99391253
chr7	99392670	99394119
chr7	99403397	99404953
chr7	99433277	99435268
chr7	99448120	99450795
chr7	99506004	99512591
chr7	99512452	99520304
chr7	99618524	99621574
chr7	99903217	99908812
chr7	99908581	99909660
chr8	100184627	100194268
chr8	100213089	100233537
chr8	100220278	100231288
chr8	100220278	100233537
chr8	100220278	100239404
chr8	100225172	100231288
chr8	100225172	100233537
chr8	100261541	100264195
chr8	100264033	100288267
chr8	100268784	100288267
chr8	100274952	100288267
chr8	100704296	100705673
chr8	100704925	100718280
chr8	100705588	100709223
chr8	100706650	100706997
chr8	100709132	100712789
chr8	100709132	100713181
chr8	100709132	100717888
chr8	100709132	100718280

chr8	100712361	100713181
chr8	100712361	100718280
chr8	100715461	100718280
chr8	100923954	100925039
chr8	10207832	10319989
chr8	102212775	102232304
chr8	102261145	102267007
chr8	102261914	102264558
chr8	102261914	102267007
chr8	102261914	102270183
chr8	102265099	102288266
chr8	102271133	102272772
chr8	102285126	102289576
chr8	102311298	102346395
chr8	102313803	102347092
chr8	102327741	102329399
chr8	102327741	102346395
chr8	102329089	102345545
chr8	102329089	102346395
chr8	102342472	102345545
chr8	102342472	102347092
chr8	102342472	102361626
chr8	102345395	102347092
chr8	102346235	102361626
chr8	102360070	102361206
chr8	102360070	102361626
chr8	102833055	102834747
chr8	102833055	102836390
chr8	102833055	102838916
chr8	102833055	102843747
chr8	102834188	102836390
chr8	102834188	102838916
chr8	102834188	102839823
chr8	102836255	102838916
chr8	102838743	102839823
chr8	102838743	102843747
chr8	102839649	102843747
chr8	102843550	102850144
chr8	103040797	103042407
chr8	103040797	103055936
chr8	103040797	103064811
chr8	103048869	103055936
chr8	103401515	103404861
chr8	103401515	103407784
chr8	103404775	103407784

chr8	103420263	103421082
chr8	103430611	103435790
chr8	103697085	103766537
chr8	103915494	103918487
chr8	103916413	103942926
chr8	103921671	103942926
chr8	103961064	104014615
chr8	104244915	104248813
chr8	104499316	104509074
chr8	106679209	106684359
chr8	106679209	106692877
chr8	10820192	10834775
chr8	108203400	108229195
chr8	108214606	108229195
chr8	108214606	108241913
chr8	108228266	108241913
chr8	108229069	108241913
chr8	108234997	108240075
chr8	108234997	108241913
chr8	108239957	108241913
chr8	10826151	10834775
chr8	10831664	10834775
chr8	108449822	108450492
chr8	108449822	108453147
chr8	108449822	108455930
chr8	109271004	109322463
chr8	109280967	109289933
chr8	11304840	11319838
chr8	115619131	115623758
chr8	116646470	116658980
chr8	116655855	116658980
chr8	116657214	116658980
chr8	116657214	116726172
chr8	116850617	116866761
chr8	116854244	116856771
chr8	116854244	116866761
chr8	11748915	11750236
chr8	11748918	11757083
chr8	117528650	117530827
chr8	117804721	117812961
chr8	117818434	117835551
chr8	117818434	117837201
chr8	117830229	117837201
chr8	117835443	117837201
chr8	11808793	11809850

chr8	11808793	11826215
chr8	11808793	11831670
chr8	11852609	11861479
chr8	118621534	118726067
chr8	119742533	119746934
chr8	119742533	119762608
chr8	119756005	119791459
chr8	119758072	119791459
chr8	119783380	119791459
chr8	119783380	119806401
chr8	119788337	119797846
chr8	119788789	119797846
chr8	119791323	119802025
chr8	119793365	119802025
chr8	119795531	119797846
chr8	119795531	119819506
chr8	119801793	119806401
chr8	119803877	119806401
chr8	119838258	119843747
chr8	119838258	119850516
chr8	119841793	119850516
chr8	119846989	119853215
chr8	119850381	119853215
chr8	120413990	120432123
chr8	120413990	120443308
chr8	120451002	120490570
chr8	120453846	120461255
chr8	120453846	120470937
chr8	120455434	120470937
chr8	120459214	120470937
chr8	120459214	120488332
chr8	120459214	120510029
chr8	120488158	120522719
chr8	123019194	123025050
chr8	123019194	123030716
chr8	123021446	123030716
chr8	123022683	123030716
chr8	123023712	123030716
chr8	123077110	123084340
chr8	123077110	123093742
chr8	123077110	123097441
chr8	123077110	123101007
chr8	123077110	123105464
chr8	123077110	123109620
chr8	123077110	123120188

chr8	123077110	123128513
chr8	123084161	123097441
chr8	123084161	123128513
chr8	123093590	123097441
chr8	123093590	123105464
chr8	123105287	123109620
chr8	123109316	123120188
chr8	123120054	123134206
chr8	123126055	123126687
chr8	123129065	123130333
chr8	123134113	123144855
chr8	123226499	123231757
chr8	123226499	123239144
chr8	123231299	123239144
chr8	123253321	123267386
chr8	123322937	123328579
chr8	123322937	123339446
chr8	123322937	123348273
chr8	123325892	123336532
chr8	123325892	123337821
chr8	123328189	123336532
chr8	123328189	123337821
chr8	123333877	123336532
chr8	123333877	123337821
chr8	123333877	123339446
chr8	123344883	123348273
chr8	123344883	123349444
chr8	123344883	123361646
chr8	123356388	123359685
chr8	123356388	123361646
chr8	123356388	123370024
chr8	123359220	123361646
chr8	123359576	123371338
chr8	123370902	123371835
chr8	123778024	123806229
chr8	123786494	123789745
chr8	123798089	123810351
chr8	124320085	124327421
chr8	124320085	124330792
chr8	124493832	124516030
chr8	124495471	124516030
chr8	124504270	124516030
chr8	124504270	124523002
chr8	124508473	124516030
chr8	124508473	124522200

chr8	124515745	124522200
chr8	125003175	125007487
chr8	125007390	125009343
chr8	125008970	125009343
chr8	125044535	125063651
chr8	125049005	125050665
chr8	125049005	125057666
chr8	125078737	125082467
chr8	125102050	125182256
chr8	125151170	125182256
chr8	125357218	125357818
chr8	127939507	127989291
chr8	128070159	128096654
chr8	129846803	129862334
chr8	129846803	129871496
chr8	129852161	129862334
chr8	129855610	129862334
chr8	129855610	129879471
chr8	129862228	129871496
chr8	129871374	129903350
chr8	129871374	129904585
chr8	130057953	130061069
chr8	130057953	130092143
chr8	130060578	130076406
chr8	130076347	130092143
chr8	130076347	130126089
chr8	130079901	130092143
chr8	130091972	130112322
chr8	130091972	130126089
chr8	130112093	130118675
chr8	130112093	130180880
chr8	130116879	130128090
chr8	130118160	130126089
chr8	130134295	130169067
chr8	130134295	130180880
chr8	130136950	130169067
chr8	130152735	130169067
chr8	130152735	130180880
chr8	130152735	130187285
chr8	130159863	130169067
chr8	130159863	130180880
chr8	130159863	130187285
chr8	130159863	130358143
chr8	130167535	130187285
chr8	130179263	130180880

chr8	130179263	130187285
chr8	130179263	130188183
chr8	130179263	130358143
chr8	130180750	130214701
chr8	130187235	130358143
chr8	130214555	130236994
chr8	130214555	130358143
chr8	130214555	130361771
chr8	130214555	130401970
chr8	130236921	130358143
chr8	130236921	130361771
chr8	130358016	130361771
chr8	130358016	130401970
chr8	13088486	13092825
chr8	13091317	13092825
chr8	13094758	13100770
chr8	131940498	131946633
chr8	131940498	131950090
chr8	131940498	131955905
chr8	131940498	131959663
chr8	131940498	131970643
chr8	131940498	131979019
chr8	131949968	131955905
chr8	131949968	131959663
chr8	131949968	131970643
chr8	132583693	132638107
chr8	132622610	132661627
chr8	132656829	132661627
chr8	132722039	132757294
chr8	132777791	132794817
chr8	132777791	132804740
chr8	132777791	132814889
chr8	132777795	132794581
chr8	132777795	132798860
chr8	132794409	132798860
chr8	132836539	132842875
chr8	132836539	132844318
chr8	134509618	134521001
chr8	134509618	134590355
chr8	134565332	134590355
chr8	134583831	134590355
chr8	134583831	134610655
chr8	134600435	134610655
chr8	134608728	134610655
chr8	135521236	135548900

chr8	135521236	135557587
chr8	135521236	135582073
chr8	135542653	135548900
chr8	135542653	135557587
chr8	135542653	135582073
chr8	135542653	135607037
chr8	139885878	139910300
chr8	139885878	140024079
chr8	139988725	140024079
chr8	139988725	140252929
chr8	140023936	140252929
chr8	140221458	140252929
chr8	140252776	140291078
chr8	140275657	140287734
chr8	140275657	140291078
chr8	140275657	140300614
chr8	140275657	140311374
chr8	140283888	140287734
chr8	140290992	140360193
chr8	140290992	140426641
chr8	140300468	140311374
chr8	140300468	140360193
chr8	140300468	140426641
chr8	140360049	140405698
chr8	140397619	140405698
chr8	140397619	140426641
chr8	140397619	140439197
chr8	140539319	140541358
chr8	140549113	140551436
chr8	140551302	140585311
chr8	140555895	140585311
chr8	140562452	140585311
chr8	140572811	140585311
chr8	140664916	140674404
chr8	140668268	140674404
chr8	140668268	140706205
chr8	140674297	140686694
chr8	140674297	140706205
chr8	140674297	140752316
chr8	140675429	140706205
chr8	140686631	140706205
chr8	140686631	140752316
chr8	140700890	140702707
chr8	140700890	140706205
chr8	140700890	140717709

chr8	140700890	140743330
chr8	140700890	140746860
chr8	140700890	140752316
chr8	140717597	140752316
chr8	140735250	140739107
chr8	140735250	140744767
chr8	140735250	140746860
chr8	140735250	140752316
chr8	140735250	140761262
chr8	140735250	140764290
chr8	140739017	140752316
chr8	140743229	140746860
chr8	140746759	140761262
chr8	140746759	140764290
chr8	140752231	140846678
chr8	140752231	140890769
chr8	140761164	140789526
chr8	140761164	140793384
chr8	140761164	140800576
chr8	140761164	140803650
chr8	140761164	140819020
chr8	140761164	140864399
chr8	140764233	140803650
chr8	140789473	140803650
chr8	140789473	140819020
chr8	140789473	140830526
chr8	140789473	140846678
chr8	140789473	140864399
chr8	140789473	140879637
chr8	140789473	140890769
chr8	140793353	140830526
chr8	140793353	140846678
chr8	140793353	140864399
chr8	140793353	140879637
chr8	140793353	140890769
chr8	140800458	140803650
chr8	140800458	140819020
chr8	140800458	140830526
chr8	140800458	140864399
chr8	140800458	140879637
chr8	140800458	140890769
chr8	140800458	140925749
chr8	140818276	140819020
chr8	140818276	140830526
chr8	140818276	140846678

chr8	140818276	140864399
chr8	140818276	140879637
chr8	140818276	140890769
chr8	140818276	140925749
chr8	140830471	140864399
chr8	140830471	140879637
chr8	140830471	140890769
chr8	140830471	140925749
chr8	140846259	140864399
chr8	140846259	140879637
chr8	140846259	140890769
chr8	140846259	140925749
chr8	140846598	140879637
chr8	140846598	140890769
chr8	140864311	140879637
chr8	140864311	140890769
chr8	140864311	140925749
chr8	140879470	140890769
chr8	140879470	140925749
chr8	140890542	140925749
chr8	141136557	141138137
chr8	141150833	141155970
chr8	141155848	141163429
chr8	141155848	141168525
chr8	141155848	141185278
chr8	141160631	141163429
chr8	141175199	141178196
chr8	141175199	141180854
chr8	141176590	141185278
chr8	141185138	141190417
chr8	141215758	141221765
chr8	141421387	141425140
chr8	142300485	142315089
chr8	142300485	142318634
chr8	142318543	142331831
chr8	142330900	142345892
chr8	142343965	142345892
chr8	143465716	143475585
chr8	143580011	143586849
chr8	143688661	143690123
chr8	143689203	143690123
chr8	143792484	143795530
chr8	143935197	143938692
chr8	143935847	143938692
chr8	143937164	143938692

chr8	143991199	143991804
chr8	144096123	144097134
chr8	144098865	144103225
chr8	144105639	144106963
chr8	144190783	144200541
chr8	144262395	144265071
chr8	144263477	144263911
chr8	144263830	144264142
chr8	144309454	144314054
chr8	144356599	144356915
chr8	144510597	144510903
chr8	144512391	144512771
chr8	144533125	144534202
chr8	144555559	144581277
chr8	144791772	144792140
chr8	144802869	144804238
chr8	144994478	144999769
chr8	15623079	15662296
chr8	15623079	15673836
chr8	15623079	15743612
chr8	15650696	15673836
chr8	1565682	1626887
chr8	1565682	1633046
chr8	15659506	15673836
chr8	15730665	15748465
chr8	17064083	17081740
chr8	17064083	17090584
chr8	17077782	17090584
chr8	17237211	17245247
chr8	17242991	17245247
chr8	17265906	17268956
chr8	17265906	17276467
chr8	17265906	17284616
chr8	17265906	17286427
chr8	17280374	17286427
chr8	17284472	17286427
chr8	17331149	17349081
chr8	17341362	17349081
chr8	17558294	17562110
chr8	17743603	17755961
chr8	17950614	17953186
chr8	17963100	17967170
chr8	17963100	17969748
chr8	17980590	18011827
chr8	17989858	17993619

chr8	17989858	18014840
chr8	18009546	18014840
chr8	18011236	18011827
chr8	1843352	1860184
chr8	1843352	1869250
chr8	1843352	1885707
chr8	1843352	1909470
chr8	18572527	18632806
chr8	1857959	1869250
chr8	1866525	1894572
chr8	18765448	18804898
chr8	1876570	1885707
chr8	18867673	18872733
chr8	1893568	1905716
chr8	1903280	1909470
chr8	19364071	19373660
chr8	19819572	19837160
chr8	19823300	19826559
chr8	19824802	19830559
chr8	20209432	20212905
chr8	20210345	20211316
chr8	22072325	22073835
chr8	22248480	22249309
chr8	22404695	22408496
chr8	22474953	22475624
chr8	22474953	22498112
chr8	22474953	22513432
chr8	22528505	22533018
chr8	22605735	22607325
chr8	22606084	22608065
chr8	23027132	23029721
chr8	23199265	23202761
chr8	23302026	23303397
chr8	23302026	23309911
chr8	23309667	23320052
chr8	23566107	23568378
chr8	25275385	25366970
chr8	25298943	25300657
chr8	25298943	25310532
chr8	25308782	25317131
chr8	25332250	25334196
chr8	25340876	25345611
chr8	25366869	25369641
chr8	25366869	25372718
chr8	25407982	25408940

chr8	25460383	25462208
chr8	25460383	25469980
chr8	25462053	25488689
chr8	25483398	25484210
chr8	26360168	26363890
chr8	26581968	26583983
chr8	27775498	27784057
chr8	27822318	27833133
chr8	28106712	28113173
chr8	28129501	28137891
chr8	28137697	28162078
chr8	28155941	28162078
chr8	28520637	28528164
chr8	28713456	28718207
chr8	28731222	28743214
chr8	28835291	28837776
chr8	28835291	28859563
chr8	28846746	28859563
chr8	28963810	28970519
chr8	28963810	28980156
chr8	28970042	28980156
chr8	28970042	29009182
chr8	28980070	29018913
chr8	29148576	29155856
chr8	30101897	30104486
chr8	30607056	30614715
chr8	30680903	30684199
chr8	30684087	30693055
chr8	30684087	30700135
chr8	30689160	30693055
chr8	30696379	30703240
chr8	30700080	30703240
chr8	30700080	30708141
chr8	30700080	30712088
chr8	30791196	30799755
chr8	31087775	31091898
chr8	31141429	31150455
chr8	31147052	31150455
chr8	32595827	32616885
chr8	33453215	33461725
chr8	33488377	33489139
chr8	33495541	33497297
chr8	37765525	37766355
chr8	37835173	37837939
chr8	37870419	37877551

chr8	38114191	38119363
chr8	38114191	38121149
chr8	38114191	38128951
chr8	38114191	38135766
chr8	38128290	38128951
chr8	38242249	38253718
chr8	38247712	38253718
chr8	38290474	38299590
chr8	38314646	38316042
chr8	38337304	38338607
chr8	38337304	38348215
chr8	38424508	38426245
chr8	38788703	38820635
chr8	38825307	38831177
chr8	38825307	38836287
chr8	38836161	38840267
chr8	38917906	38946221
chr8	38946123	38957386
chr8	39007885	39018918
chr8	39007885	39026810
chr8	39021642	39023325
chr8	39054480	39055772
chr8	39054480	39083073
chr8	39077227	39083073
chr8	39077227	39091346
chr8	39090046	39091346
chr8	39136656	39169997
chr8	39136656	39187045
chr8	39147129	39165196
chr8	39221609	39257343
chr8	41530183	41537293
chr8	41530183	41541899
chr8	41609415	41610810
chr8	41609415	41611992
chr8	41609415	41612273
chr8	41609415	41612960
chr8	41611902	41612273
chr8	41611902	41615048
chr8	41955295	41987563
chr8	41974703	41978777
chr8	41974703	41987563
chr8	41980845	41987563
chr8	42185080	42189071
chr8	42185080	42191414
chr8	42325969	42329214

chr8	42344952	42350065
chr8	42344952	42352568
chr8	42344952	42357392
chr8	42350006	42361365
chr8	42350006	42362698
chr8	42352518	42362698
chr8	42355515	42361365
chr8	42394209	42403461
chr8	42398711	42403461
chr8	42401787	42403461
chr8	42428757	42430249
chr8	42436988	42444762
chr8	42439449	42465917
chr8	42439449	42472654
chr8	42459895	42465917
chr8	42459895	42472654
chr8	42463004	42472654
chr8	42861744	42870112
chr8	42861744	42874006
chr8	42861744	42887871
chr8	42870003	42874006
chr8	42870003	42887871
chr8	42906172	42930172
chr8	42906172	42943445
chr8	42906172	42950455
chr8	42906172	42959314
chr8	42906172	42964474
chr8	42906172	42982696
chr8	42906172	42986795
chr8	42906172	42997637
chr8	42925556	42943445
chr8	42925556	42950455
chr8	42925556	42959314
chr8	42925556	42964474
chr8	42925556	42982696
chr8	42943312	42966613
chr8	42943312	42982696
chr8	42950387	42959314
chr8	42950387	42964474
chr8	42950387	42968214
chr8	42950387	42974194
chr8	42950387	42997637
chr8	42957093	42964474
chr8	42957093	42974194
chr8	42964310	42966613

chr8	42964310	42968214
chr8	42966472	42968214
chr8	42968012	42982696
chr8	42973288	42974194
chr8	42973288	42986795
chr8	42982626	43010405
chr8	43002106	43013400
chr8	43007846	43013400
chr8	43010304	43013400
chr8	43059091	43064215
chr8	43059091	43069659
chr8	43059091	43072307
chr8	43059091	43077364
chr8	43064100	43072307
chr8	43064100	43077364
chr8	43069554	43072307
chr8	43069554	43084881
chr8	43097523	43103830
chr8	431340	435707
chr8	43146947	43159044
chr8	43146947	43161507
chr8	43158574	43169242
chr8	43158574	43172386
chr8	43158574	43173743
chr8	47279861	47291137
chr8	47279861	47294030
chr8	47279861	47407961
chr8	47279861	47440542
chr8	47279861	47599196
chr8	47284027	47294030
chr8	47291032	47294030
chr8	47293866	47407961
chr8	47396375	47407961
chr8	47396375	47440542
chr8	47407860	47440542
chr8	47595810	47599196
chr8	47595810	47713641
chr8	47700402	47701864
chr8	47700402	47702015
chr8	47712661	47713641
chr8	47776843	47783809
chr8	47782161	47794501
chr8	47783741	47785317
chr8	47783741	47798397
chr8	47785112	47794501

chr8	47785112	47798397
chr8	47794289	47798397
chr8	47794289	47807326
chr8	47798236	47807326
chr8	47798236	47904976
chr8	47799209	47807326
chr8	47800792	47819510
chr8	47817449	47831926
chr8	47840015	47854214
chr8	47849153	47852784
chr8	47852672	47854214
chr8	47852672	47855373
chr8	47852672	47858635
chr8	47859610	47864763
chr8	47877723	47879658
chr8	47877723	47886147
chr8	47885943	47897294
chr8	47887546	47893387
chr8	47888517	47893387
chr8	47897160	47904976
chr8	47900372	47904976
chr8	47900372	47930012
chr8	47902568	47912562
chr8	47912409	47915418
chr8	47912409	47954446
chr8	47918276	47927890
chr8	47918276	47930012
chr8	47927193	47927890
chr8	47927193	47930012
chr8	47927193	47930787
chr8	47927193	47936517
chr8	47927193	47939697
chr8	47930671	47934090
chr8	47930671	47935058
chr8	47933019	47935058
chr8	47933019	47936517
chr8	47933019	47939697
chr8	47933019	47954446
chr8	47935008	47936517
chr8	47943208	47944029
chr8	47953619	47953919
chr8	47953619	47954446
chr8	47953619	47957431
chr8	47954337	47957431
chr8	47957170	47957431

chr8	47969797	47971468
chr8	47970510	47971468
chr8	48043032	48049978
chr8	492458	494597
chr8	51845660	51861246
chr8	52661534	52668191
chr8	52683548	52686967
chr8	53743576	53772167
chr8	53743576	53829533
chr8	53756554	53772167
chr8	53769617	53772167
chr8	53795646	53801896
chr8	53795646	53817530
chr8	53795646	53829533
chr8	53811163	53829533
chr8	53946664	53954310
chr8	53978522	53979171
chr8	53979024	54010492
chr8	53984362	54000050
chr8	53984362	54003092
chr8	53993667	54000050
chr8	53993667	54010492
chr8	55750963	55756411
chr8	55750963	55763980
chr8	55782747	55790299
chr8	55782747	55805036
chr8	55795977	55805036
chr8	55802456	55805036
chr8	55941854	55947723
chr8	55941854	55953984
chr8	55946447	55966897
chr8	55950458	55952115
chr8	55950458	55953984
chr8	55950458	55969793
chr8	55966714	55969793
chr8	56978049	56980197
chr8	58416849	58439770
chr8	58430518	58434504
chr8	58585651	58590934
chr8	58589451	58590934
chr8	58601284	58603386
chr8	58623219	58635546
chr8	58623219	58643073
chr8	60558851	60591969
chr8	60572045	60591969

chr8	60584207	60591969
chr8	60780999	60808272
chr8	60794985	60800525
chr8	60794985	60801593
chr8	60794985	60808272
chr8	60800387	60808272
chr8	60816386	60822145
chr8	60816386	60824016
chr8	60820006	60822145
chr8	60820006	60824016
chr8	61517527	61526112
chr8	61525976	61556022
chr8	61525976	61576858
chr8	61548070	61556022
chr8	61567167	61576858
chr8	61567167	61584029
chr8	61567167	61619019
chr8	61633682	61644632
chr8	61633682	61653660
chr8	61637946	61646878
chr8	61637946	61653660
chr8	61637946	61684188
chr8	61642887	61684188
chr8	61643944	61681036
chr8	61644599	61681036
chr8	61644599	61684188
chr8	61651049	61684188
chr8	61680967	61684188
chr8	61682438	61684188
chr8	63017492	63030217
chr8	63175330	63187745
chr8	6455142	6499929
chr8	6477593	6499929
chr8	65613243	65627433
chr8	65669872	65682451
chr8	65669872	65693759
chr8	65669872	65704929
chr8	65669872	65708011
chr8	65693643	65704929
chr8	65723540	65745470
chr8	65734793	65745470
chr8	65734793	65782843
chr8	66566683	66573506
chr8	66572481	66573506
chr8	66572481	66576375

chr8	66572481	66580366
chr8	66592439	66602523
chr8	66595582	66602523
chr8	66599049	66602523
chr8	66601697	66602523
chr8	66793615	66798625
chr8	66793615	66804447
chr8	66793615	66813928
chr8	66804374	66822459
chr8	66804374	66836074
chr8	66813852	66831311
chr8	66828653	66840247
chr8	66835762	66836074
chr8	66835762	66840247
chr8	67045811	67059445
chr8	67074242	67076581
chr8	67074242	67086110
chr8	67076481	67095732
chr8	67086006	67095732
chr8	67093542	67095732
chr8	67093542	67112065
chr8	67095292	67113862
chr8	67103036	67116122
chr8	67103036	67118821
chr8	67103036	67132080
chr8	67103036	67137603
chr8	67105904	67112065
chr8	67105904	67116122
chr8	67105904	67118821
chr8	67105904	67137603
chr8	67111971	67118821
chr8	67111971	67137603
chr8	67113804	67132080
chr8	67115913	67118821
chr8	67115913	67132080
chr8	67118742	67137603
chr8	67131950	67137603
chr8	67131950	67154136
chr8	67149782	67164508
chr8	67154023	67159137
chr8	67161810	67164508
chr8	67164390	67179926
chr8	67172415	67177726
chr8	67175295	67190759
chr8	67238342	67238893

chr8	6724869	6732650
chr8	6724869	6747828
chr8	67257731	67288065
chr8	67259814	67277457
chr8	67266005	67277457
chr8	67275975	67288065
chr8	67291846	67301380
chr8	67299208	67301380
chr8	67299208	67302466
chr8	6730710	6732650
chr8	6730710	6741751
chr8	69755423	69761875
chr8	70121301	70144848
chr8	70123883	70124865
chr8	70131836	70141399
chr8	70159504	70162854
chr8	70170201	70174859
chr8	70213902	70216764
chr8	70213902	70296800
chr8	70583163	70583793
chr8	70583163	70587176
chr8	70583163	70596321
chr8	70583163	70598255
chr8	70594505	70598255
chr8	70657755	70661897
chr8	70674817	70683630
chr8	73013894	73027052
chr8	73020683	73024971
chr8	73291042	73292797
chr8	73292238	73292797
chr8	73552011	73673242
chr8	73582769	73603863
chr8	73582769	73673242
chr8	73582769	73688813
chr8	73582769	73709162
chr8	73595165	73617451
chr8	73595165	73709162
chr8	73603725	73673242
chr8	73603725	73688813
chr8	73603725	73739868
chr8	73613743	73615782
chr8	73613743	73738349
chr8	73673106	73688813
chr8	73673106	73709162
chr8	73673106	73738349

chr8	73673106	73739868
chr8	73688653	73739868
chr8	73738283	73739868
chr8	73805650	73830472
chr8	73955910	73959818
chr8	73955910	73964625
chr8	76704042	76708048
chr8	78675786	78689373
chr8	78675786	78698513
chr8	78689221	78698513
chr8	80042619	80053430
chr8	80051526	80053430
chr8	80051526	80064593
chr8	80499493	80518954
chr8	81670938	81679230
chr8	81670938	81680783
chr8	81670938	81681584
chr8	81673840	81681584
chr8	81679125	81680783
chr8	81713917	81718224
chr8	81815324	81829516
chr8	81829429	81840082
chr8	85202146	85207489
chr8	85202146	85209409
chr8	86368938	86381629
chr8	86374029	86381629
chr8	86374029	86411874
chr8	86380725	86398638
chr8	86380725	86402203
chr8	86398341	86402203
chr8	86402018	86411874
chr8	86461223	86461846
chr8	86474819	86488639
chr8	86478922	86488639
chr8	86484871	86488639
chr8	86486483	86488639
chr8	86515461	86531229
chr8	86515461	86546681
chr8	86528535	86532580
chr8	86528535	86537646
chr8	86528535	86540334
chr8	86554850	86556338
chr8	88167668	88197306
chr8	89722014	89725641
chr8	89724628	89725641

chr8	89909566	89921171
chr8	89937025	89946295
chr8	89937025	89947892
chr8	89958724	89971290
chr8	89964409	89971290
chr8	89964409	89978323
chr8	89970363	89978323
chr8	89970363	89980893
chr8	89971172	89978323
chr8	90017123	90036940
chr8	90017123	90044995
chr8	90042727	90044995
chr8	9007969	9020464
chr8	91008742	91021404
chr8	91018389	91021404
chr8	91124492	91135739
chr8	91189521	91200792
chr8	91289135	91295703
chr8	92916928	92921660
chr8	92916928	92954564
chr8	93704908	93709875
chr8	93755019	93803684
chr8	93786222	93791319
chr8	93786222	93799758
chr8	93786222	93803684
chr8	93786222	93816515
chr8	94378179	94407720
chr8	94399413	94411315
chr8	94458267	94467555
chr8	94495730	94499506
chr8	94499373	94506717
chr8	94506499	94512089
chr8	94530962	94538346
chr8	94534838	94538346
chr8	94534838	94543942
chr8	94537102	94543942
chr8	94734629	94739790
chr8	94738368	94739790
chr8	94756035	94766685
chr8	94766611	94770571
chr8	94777665	94783802
chr8	94824892	94827403
chr8	94824892	94827793
chr8	94824892	94829026
chr8	94824892	94836631

chr8	94824892	94842488
chr8	94824892	94849532
chr8	94824892	94859632
chr8	94827262	94829026
chr8	94827262	94832174
chr8	94827262	94842488
chr8	94827262	94849532
chr8	94827721	94832174
chr8	94828974	94841591
chr8	94838462	94859632
chr8	94842346	94856978
chr8	94851552	94866191
chr8	94859510	94867337
chr8	94865505	94866191
chr8	94867139	94876489
chr8	94867275	94876147
chr8	94871883	94874602
chr8	94885066	94885558
chr8	94887926	94890550
chr8	94939859	94941091
chr8	95031994	95035576
chr8	95031994	95048558
chr8	95031994	95052230
chr8	95035453	95047127
chr8	95035453	95052230
chr8	95045544	95047127
chr8	95252187	95263774
chr8	9580158	9615677
chr8	96243918	96258700
chr8	96284108	96299845
chr8	96295097	96320345
chr8	96304039	96309622
chr8	96306443	96331095
chr8	9679950	9706253
chr8	96834972	96880005
chr8	97029402	97066210
chr8	9704662	9710220
chr8	9763146	9766425
chr8	97661071	97691188
chr8	97686667	97687605
chr8	97686667	97689103
chr8	97686667	97691188
chr8	97686667	97713769
chr8	97687428	97689103
chr8	97687428	97713769

chr8	97690951	97706750
chr8	97699753	97713769
chr8	97699753	97723035
chr8	97713661	97719189
chr8	97713661	97723035
chr8	97805352	97819238
chr8	97805352	97825153
chr8	97815327	97819238
chr8	97815327	97825153
chr8	98104488	98108751
chr8	98123335	98128540
chr8	98123335	98130226
chr8	98123335	98134659
chr8	98129977	98136954
chr8	98133948	98134659
chr8	98133948	98136738
chr8	98136481	98136954
chr8	98526741	98548161
chr8	98526741	98596169
chr8	98706466	98707311
chr8	98706466	98749390
chr8	98749275	98774819
chr8	98767242	98774819
chr8	99013759	99038566
chr8	99013759	99156743
chr8	99038422	99121445
chr8	99096311	99103120
chr8	99096311	99111279
chr8	99096311	99121445
chr8	99096311	99148010
chr8	99096311	99193057
chr8	99096311	99275254
chr8	99102952	99121445
chr8	99111097	99121445
chr8	99121176	99136752
chr8	99134631	99136752
chr8	99134631	99143165
chr8	99134631	99156743
chr8	99134631	99193057
chr8	99142973	99156743
chr8	99156548	99170163
chr8	99156548	99193057
chr8	99156548	99275254
chr8	99170038	99193057
chr8	99170038	99275254

chr8	99192875	99275254
chr8	99192875	99391704
chr8	99274197	99275254
chr8	99274197	99391704
chr8	99274197	99442635
chr8	99384207	99442635
chr8	99391556	99521010
chr8	99431536	99467634
chr8	99431536	99481802
chr8	99431536	99521010
chr8	99442400	99467634
chr8	99442400	99481802
chr8	99442400	99521010
chr8	99467413	99481802
chr8	99467413	99577633
chr8	99481598	99521010
chr8	99502835	99511512
chr8	99511103	99521010
chr8	99520898	99556653
chr8	99520898	99577633
chr8	99520898	99661491
chr8	99556449	99577633
chr8	99575657	99577633
chr8	99641810	99661491
chr8	99661353	99721047
chr8	99717170	99721047
chr8	99809374	99823978
chr8	99809374	99835738
chr9	100006505	100022226
chr9	100052416	100060172
chr9	100104497	100126549
chr9	100226061	100229827
chr9	100229659	100253136
chr9	100240059	100253136
chr9	100246615	100253136
chr9	100252282	100273076
chr9	100303631	100310379
chr9	100308499	100310379
chr9	100308499	100321771
chr9	100308499	100349372
chr9	100310298	100321771
chr9	100310298	100349372
chr9	100320264	100321771
chr9	100320264	100330169
chr9	100320264	100349369

chr9	100320264	100349372
chr9	100340256	100349372
chr9	100498764	100509134
chr9	100498764	100516771
chr9	100498764	100550160
chr9	100509004	100516771
chr9	100513306	100561520
chr9	101540489	101544885
chr9	104129644	104132125
chr9	104750955	104759171
chr9	105299219	105335699
chr9	105348357	105358433
chr9	105348357	105361330
chr9	105362820	105374735
chr9	105464235	105472005
chr9	105464235	105484502
chr9	105471903	105484502
chr9	105534492	105539351
chr9	105601144	105618092
chr9	105615277	105620061
chr9	105705596	105722621
chr9	105705596	105748190
chr9	105721536	105722621
chr9	106923353	106935621
chr9	106923353	106939107
chr9	106972004	106974273
chr9	106972004	106984409
chr9	106972004	107003426
chr9	107300140	107306647
chr9	107300140	107311737
chr9	107300140	107318879
chr9	107300140	107325004
chr9	107302034	107306647
chr9	107302034	107311737
chr9	107302034	107325004
chr9	107321982	107325004
chr9	108874894	108882187
chr9	108899821	108919349
chr9	108922841	108929921
chr9	108952208	108952494
chr9	108965377	108999256
chr9	108990725	108999256
chr9	109033306	109038117
chr9	109033306	109050692
chr9	109033306	109057322

chr9	109033306	109060452
chr9	109033306	109108570
chr9	109036205	109060452
chr9	109050282	109050692
chr9	109050282	109057322
chr9	109050282	109060452
chr9	109050282	109064567
chr9	109050282	109073438
chr9	109057190	109060452
chr9	109057190	109064567
chr9	109057190	109073438
chr9	109057190	109093591
chr9	109057190	109108570
chr9	109073355	109087342
chr9	109073355	109108570
chr9	109080838	109087342
chr9	109080838	109108570
chr9	109087172	109091155
chr9	109087172	109108570
chr9	109090921	109108570
chr9	109093474	109108570
chr9	109106507	109108570
chr9	109404447	109408377
chr9	109420423	109438234
chr9	109780487	109925082
chr9	109867490	109880681
chr9	110941420	110973558
chr9	110972072	110973558
chr9	111373169	111385442
chr9	111378579	111385442
chr9	111383210	111391824
chr9	111386376	111391824
chr9	111391755	111394305
chr9	111392767	111394305
chr9	111392767	111410213
chr9	111408570	111410213
chr9	111408570	111451555
chr9	111410979	111416352
chr9	111425417	111444494
chr9	111428041	111442424
chr9	111433232	111442424
chr9	111433232	111444494
chr9	111436939	111451555
chr9	111440371	111444494
chr9	111440371	111451555

chr9	111527267	111530963
chr9	111527267	111534353
chr9	111530874	111534353
chr9	111533738	111534353
chr9	111570090	111586165
chr9	111570090	111597432
chr9	111574733	111586165
chr9	111574733	111597432
chr9	111578795	111586165
chr9	111578795	111592982
chr9	111578795	111597432
chr9	111583471	111586165
chr9	111775790	111786035
chr9	111914604	111922951
chr9	111914604	111926496
chr9	111914604	111929678
chr9	111914604	111931357
chr9	111922848	111926496
chr9	111922848	111929678
chr9	111922848	111931357
chr9	111924776	111931357
chr9	111929499	111931357
chr9	112052398	112063033
chr9	112062936	112078724
chr9	112078537	112080165
chr9	112078537	112098662
chr9	112078537	112102285
chr9	112098469	112112868
chr9	112098469	112124436
chr9	112111653	112112868
chr9	112124256	112143623
chr9	112142319	112157613
chr9	112149243	112157613
chr9	112232098	112252788
chr9	112250928	112252788
chr9	112250928	112262599
chr9	112250928	112268195
chr9	112250928	112276013
chr9	112250928	112297916
chr9	112250928	112330474
chr9	112252677	112262599
chr9	112252677	112297916
chr9	112262434	112276013
chr9	112262434	112297916
chr9	112262434	112330474

chr9	112268048	112276013
chr9	112268048	112297916
chr9	112275843	112297916
chr9	112275843	112330474
chr9	112403994	112405722
chr9	112403994	112409021
chr9	112403994	112459577
chr9	112405623	112409021
chr9	112405623	112418958
chr9	112438430	112441770
chr9	113171580	113176673
chr9	113198854	113203035
chr9	113256113	113257185
chr9	113256113	113258862
chr9	113276547	113291016
chr9	113278944	113283482
chr9	113282645	113283482
chr9	113286231	113288264
chr9	113328880	113332050
chr9	113328880	113336440
chr9	113969116	113988580
chr9	113969116	114016784
chr9	114162714	114169463
chr9	114162714	114183075
chr9	114209674	114243560
chr9	114341532	114343803
chr9	114466266	114478771
chr9	114624349	114628250
chr9	114633767	114637070
chr9	114636989	114638726
chr9	116618323	116620443
chr9	120415039	120439972
chr9	120419787	120422741
chr9	120422692	120471878
chr9	120437294	120439972
chr9	120437294	120448126
chr9	120437294	120460667
chr9	120467859	120491477
chr9	120477349	120487437
chr9	120477349	120491477
chr9	120477349	120518645
chr9	120487293	120491477
chr9	120487293	120518645
chr9	120487293	120545790
chr9	120518426	120539164

chr9	120529977	120568388
chr9	120536371	120539164
chr9	120536371	120568388
chr9	120539040	120568388
chr9	120545713	120568388
chr9	120771347	120772840
chr9	120820838	120826907
chr9	120831330	120833456
chr9	120923704	120926688
chr9	120980082	120981939
chr9	121161855	121162271
chr9	121161855	121166180
chr9	121182929	121193419
chr9	121190553	121193419
chr9	121699324	121760439
chr9	121699324	121782401
chr9	121960	172172
chr9	121974008	121974979
chr9	121974008	121989770
chr9	122148111	122152408
chr9	122251846	122261508
chr9	122270863	122280598
chr9	122270863	122291840
chr9	122280442	122291840
chr9	122280442	122313386
chr9	122853951	122859102
chr9	122853951	122865657
chr9	122865348	122877583
chr9	122865348	122883379
chr9	122877470	122880794
chr9	122877470	122883379
chr9	122877470	122897576
chr9	122892908	122897576
chr9	122957010	122986419
chr9	122984484	122986419
chr9	122989296	122990213
chr9	122996538	123010528
chr9	122998596	123020459
chr9	123070349	123076762
chr9	123097740	123099549
chr9	123128213	123147870
chr9	123128213	123170046
chr9	123128213	123184298
chr9	123132844	123139687
chr9	123136040	123184298

chr9	123139528	123147870
chr9	123146854	123147870
chr9	123146854	123160462
chr9	123158331	123184298
chr9	123160976	123184298
chr9	123168188	123184298
chr9	123169901	123173842
chr9	123169901	123179227
chr9	123169901	123184298
chr9	123173676	123184298
chr9	123179006	123184298
chr9	123440359	123452347
chr9	123440359	123457897
chr9	123452275	123457897
chr9	123557569	123583270
chr9	123557569	123652123
chr9	123557569	123879021
chr9	123583168	123652123
chr9	123583168	123757822
chr9	123583168	123879021
chr9	123609435	123652123
chr9	123609435	123879021
chr9	123630375	123676789
chr9	123630375	123879021
chr9	123652012	123671371
chr9	123652012	123879021
chr9	123667025	123757822
chr9	123676719	123879021
chr9	123757702	123792630
chr9	123757702	123879021
chr9	124301935	124313985
chr9	124301935	124327445
chr9	124301935	124339665
chr9	124312508	124327445
chr9	124321458	124327445
chr9	124384597	124414935
chr9	124412351	124414935
chr9	124538091	124540187
chr9	124898548	124912026
chr9	124899328	124900547
chr9	124908376	124912026
chr9	124921128	124923223
chr9	124923094	124938866
chr9	124938576	124940155
chr9	124971369	124976186

chr9	124971369	124999990
chr9	124971369	125003570
chr9	124971369	125056007
chr9	124994933	125003570
chr9	124999890	125003570
chr9	124999890	125026910
chr9	125018798	125056007
chr9	125055875	125066058
chr9	125153532	125153985
chr9	125153532	125158382
chr9	125158240	125160906
chr9	125207563	125220700
chr9	125213369	125220700
chr9	125227909	125232745
chr9	125295457	125299106
chr9	125301982	125323923
chr9	125307412	125307880
chr9	125326415	125330218
chr9	125337017	125337591
chr9	125341176	125346941
chr9	125341176	125350872
chr9	125467971	125559809
chr9	125484442	125506417
chr9	125484442	125672643
chr9	125506309	125559809
chr9	125543058	125559809
chr9	125559632	125585727
chr9	125657650	125672643
chr9	125669817	125672643
chr9	125915685	125935607
chr9	125929654	125935607
chr9	126340507	126386658
chr9	126340507	126392195
chr9	126340507	126395697
chr9	126340507	126421948
chr9	126381063	126392195
chr9	126381063	126395697
chr9	126386561	126395697
chr9	126392065	126395697
chr9	126392065	126421948
chr9	126962224	126977745
chr9	127034430	127052939
chr9	127034430	127069356
chr9	127050042	127069356
chr9	127195090	127199066

chr9	127264951	127325095
chr9	127291167	127325095
chr9	127311635	127320778
chr9	127311635	127325095
chr9	127332273	127339751
chr9	127339644	127342334
chr9	127344234	127345477
chr9	127353845	127354410
chr9	127353845	127365366
chr9	127435089	127436003
chr9	127447876	127449708
chr9	127461172	127462373
chr9	127461172	127473931
chr9	127467739	127473931
chr9	127487675	127492897
chr9	127507866	127508538
chr9	127517825	127531778
chr9	127525057	127531778
chr9	127658374	127661205
chr9	127658374	127668187
chr9	127675803	127676753
chr9	127804284	127804700
chr9	128101095	128107179
chr9	128122361	128123134
chr9	128169051	128180799
chr9	128179072	128180799
chr9	128180414	128190862
chr9	128185546	128187934
chr9	128262562	128263092
chr9	128325142	128325881
chr9	128352637	128353541
chr9	128422867	128424614
chr9	128459566	128461010
chr9	128459566	128461067
chr9	128472912	128473741
chr9	128487889	128492805
chr9	128492425	128492805
chr9	128508875	128515639
chr9	128508875	128523846
chr9	128508875	128533951
chr9	128522667	128523846
chr9	128523279	128523846
chr9	128525191	128533951
chr9	128533512	128533951
chr9	128566737	128568897

chr9	128579636	128588943
chr9	128598399	128604417
chr9	128600079	128604417
chr9	128600079	128605477
chr9	128607603	128613485
chr9	128607603	128619003
chr9	128608129	128617760
chr9	128608129	128626687
chr9	128630320	128632955
chr9	128740771	128742745
chr9	128790734	128804119
chr9	128803249	128804119
chr9	128842653	128847643
chr9	128952772	128957032
chr9	128968505	128985014
chr9	128970757	128981390
chr9	128982548	128983550
chr9	128998150	128999805
chr9	129097249	129098650
chr9	129120512	129123138
chr9	129120512	129137692
chr9	129128984	129131639
chr9	129128984	129134894
chr9	129807187	129809032
chr9	129849812	129861612
chr9	129895837	129903001
chr9	129899964	129900547
chr9	129957359	129958553
chr9	129978464	129979374
chr9	130075635	130083602
chr9	130076411	130083602
chr9	130079629	130083602
chr9	130079629	130107539
chr9	130099456	130101223
chr9	130099456	130107539
chr9	130124914	130128761
chr9	130217831	130226504
chr9	130219724	130223159
chr9	130222649	130223159
chr9	130222649	130226504
chr9	130595482	130620458
chr9	130698161	130700935
chr9	130854063	130863035
chr9	130854063	130875052
chr9	130854063	130880664

chr9	131079148	131085723
chr9	131130765	131147584
chr9	131135939	131147584
chr9	131139280	131147584
chr9	131139280	131151894
chr9	131150323	131151894
chr9	131159382	131164144
chr9	131159382	131195294
chr9	131174054	131178410
chr9	131192207	131201717
chr9	131195232	131201717
chr9	131197215	131201717
chr9	13136091	13138153
chr9	131430089	131432794
chr9	131430089	131439061
chr9	131430089	131444328
chr9	131430089	131447206
chr9	131430089	131447804
chr9	131430089	131455166
chr9	131430089	131459356
chr9	131430089	131467753
chr9	131432616	131436722
chr9	131432616	131439061
chr9	131436619	131439061
chr9	131436619	131447804
chr9	131436619	131455166
chr9	131444184	131455166
chr9	131447084	131459356
chr9	131455075	131459356
chr9	131455075	131470983
chr9	131467562	131470983
chr9	131506113	131506453
chr9	131506113	131507514
chr9	131621795	131650949
chr9	131638634	131650949
chr9	131643238	131650949
chr9	131884057	132014467
chr9	131893884	131939474
chr9	131893884	132014467
chr9	13205035	13206099
chr9	13205035	13247801
chr9	13205035	13250372
chr9	13216773	13223710
chr9	13216773	13224583
chr9	132271709	132278257

chr9	132275255	132311856
chr9	132277059	132311856
chr9	132278069	132281574
chr9	132278069	132286494
chr9	132296886	132300803
chr9	132296886	132311856
chr9	132300629	132311856
chr9	132311756	132349435
chr9	132334607	132349435
chr9	132342689	132349435
chr9	132342689	132353755
chr9	132346260	132349435
chr9	132346260	132353755
chr9	132386555	132396511
chr9	132386555	132402828
chr9	13247634	13250372
chr9	132612086	132625745
chr9	132612086	132632091
chr9	132612086	132642063
chr9	132612086	132652492
chr9	132618329	132625745
chr9	132625663	132632091
chr9	132645894	132648295
chr9	132658670	132662695
chr9	132927200	132928952
chr9	133042086	133043926
chr9	133105931	133112152
chr9	133408767	133412921
chr9	133412298	133412921
chr9	133437748	133438366
chr9	133806080	133812216
chr9	133806080	133834340
chr9	133834271	133939219
chr9	134139819	134140811
chr9	134139819	134148343
chr9	134141509	134142719
chr9	134401631	134409119
chr9	134690911	134701333
chr9	134699908	134701333
chr9	134727265	134732127
chr9	134727265	134738808
chr9	134795081	134802995
chr9	135482682	135484082
chr9	135850136	135850461
chr9	135850136	135862608

chr9	135850136	135866536
chr9	135850136	135881794
chr9	135850136	135883078
chr9	135862466	135883078
chr9	135866455	135883078
chr9	135881632	135883078
chr9	135945027	135947905
chr9	136013198	136016429
chr9	136223762	136226874
chr9	136224006	136226874
chr9	136334446	136340993
chr9	136348696	136349763
chr9	136380739	136382073
chr9	136387484	136387848
chr9	136394248	136395770
chr9	136404630	136407938
chr9	136405904	136409067
chr9	136407144	136407938
chr9	136446854	136448161
chr9	136451255	136455793
chr9	136459133	136460123
chr9	136466956	136468512
chr9	136515980	136517385
chr9	136517751	136519565
chr9	136522849	136523979
chr9	136823524	136832370
chr9	136862905	136863871
chr9	136898712	136900520
chr9	136898712	136909994
chr9	136898712	136916615
chr9	136899593	136909994
chr9	136908069	136916615
chr9	136920233	136921215
chr9	136920233	136924000
chr9	137080001	137082064
chr9	137088074	137089005
chr9	137265876	137267346
chr9	137564433	137565154
chr9	137574207	137577603
chr9	137586966	137588718
chr9	137710966	137744090
chr9	137716625	137744090
chr9	137716625	137752408
chr9	137716625	137758011
chr9	137728348	137744090

chr9	137728348	137754291
chr9	137728348	137758011
chr9	137728348	137762820
chr9	137743370	137754291
chr9	137743370	137758011
chr9	137743370	137762820
chr9	137743370	137818138
chr9	137752330	137754291
chr9	137752330	137758011
chr9	137754170	137758011
chr9	137754170	137762820
chr9	137754170	137782397
chr9	137754170	137818138
chr9	137757879	137762820
chr9	137775108	137782397
chr9	137776617	137782397
chr9	137790847	137800984
chr9	137790847	137818138
chr9	137811460	137818138
chr9	137813385	137817525
chr9	137815946	137818138
chr9	14102421	14125766
chr9	14120439	14179780
chr9	14125631	14146807
chr9	14146688	14155893
chr9	14146688	14179780
chr9	146101	164037
chr9	146101	172172
chr9	14639895	14662326
chr9	14639895	14674385
chr9	14639895	14680162
chr9	14672829	14680162
chr9	15177696	15187035
chr9	15199860	15211397
chr9	152033	172172
chr9	15210087	15214249
chr9	15210087	15226012
chr9	15433551	15447244
chr9	15474008	15490124
chr9	15478476	15490124
chr9	15486005	15490124
chr9	15846701	15874663
chr9	162431	172172
chr9	17298189	17309252
chr9	17298189	17342444

chr9	17309057	17342444
chr9	19057960	19080672
chr9	19060087	19096769
chr9	19063007	19063580
chr9	19063007	19076704
chr9	19063007	19096769
chr9	19070218	19080672
chr9	19070218	19096769
chr9	19076601	19096769
chr9	19082872	19096769
chr9	19086733	19096769
chr9	19093170	19096769
chr9	19276157	19288665
chr9	19276157	19290876
chr9	19276157	19332184
chr9	19286768	19288665
chr9	19286768	19300331
chr9	19286768	19305527
chr9	19288595	19290876
chr9	19288595	19300331
chr9	19288595	19305527
chr9	19290703	19300331
chr9	19316416	19316839
chr9	19316416	19328162
chr9	19352072	19360489
chr9	19356971	19358160
chr9	19356971	19360489
chr9	19360243	19361963
chr9	19361845	19371820
chr9	19423861	19435084
chr9	2028986	2039900
chr9	20353524	20365744
chr9	20360741	20365744
chr9	20363475	20365744
chr9	20413720	20456786
chr9	20448122	20456786
chr9	20694519	20720534
chr9	20694519	20740340
chr9	20715321	20720534
chr9	20715321	20740340
chr9	20715321	20758191
chr9	20715321	20765073
chr9	20715321	20770238
chr9	20715321	20781929
chr9	20715321	20789608

chr9	20715321	20823115
chr9	20715321	20867012
chr9	20758089	20770238
chr9	20758089	20778768
chr9	20778680	20789608
chr9	20819795	20823115
chr9	20819795	20874807
chr9	20819795	20885230
chr9	20819795	20926417
chr9	20874680	20885230
chr9	20874680	20907242
chr9	20881870	20916937
chr9	20881870	20923768
chr9	20881870	20929596
chr9	20881870	20933103
chr9	20885108	20916937
chr9	20907149	20916937
chr9	20907149	20926417
chr9	20907149	20929596
chr9	20907149	20933103
chr9	20923659	20933103
chr9	20944626	20953065
chr9	20948270	20953065
chr9	21476898	21477292
chr9	21815432	21818202
chr9	2181570	2186228
chr9	21837907	21859425
chr9	21854630	21859425
chr9	22723308	22821598
chr9	23602684	23605101
chr9	2536358	2539552
chr9	26861065	26887513
chr9	26884809	26887513
chr9	26886103	26887513
chr9	26907833	26913947
chr9	26910337	26913947
chr9	26910337	26928408
chr9	26919309	26928408
chr9	26923177	26928220
chr9	26923177	26928408
chr9	26923177	26935206
chr9	26926392	26935206
chr9	26928099	26935206
chr9	26984256	26990195
chr9	27044741	27048274

chr9	2811360	2820098
chr9	32418127	32436397
chr9	32429418	32431843
chr9	32433727	32436397
chr9	32440464	32449081
chr9	32480218	32481497
chr9	3275499	3293258
chr9	3288130	3293258
chr9	3301545	3395596
chr9	33026474	33026990
chr9	33029884	33034330
chr9	33036573	33037115
chr9	33048105	33060584
chr9	33060464	33071892
chr9	33068823	33071892
chr9	33068823	33073806
chr9	33270622	33278225
chr9	33271151	33278225
chr9	33276455	33278225
chr9	3330258	3346764
chr9	3330258	3395596
chr9	33303190	33319127
chr9	33311105	33319127
chr9	33313653	33328678
chr9	33318730	33328678
chr9	33318730	33332502
chr9	33318730	33352719
chr9	33328580	33338589
chr9	33328580	33347117
chr9	33338509	33344188
chr9	33347037	33352719
chr9	33347037	33354892
chr9	33351559	33352719
chr9	33351559	33354892
chr9	33504537	33509336
chr9	33508362	33510711
chr9	33528726	33529173
chr9	33528726	33536945
chr9	33886880	33900271
chr9	33886880	33912098
chr9	33900173	33912098
chr9	33932561	33933628
chr9	33941648	33953474
chr9	33941648	33963791
chr9	33941648	33989126

chr9	33943419	33948587
chr9	33943419	33956146
chr9	33944364	33948587
chr9	33944364	33953474
chr9	33944364	33956146
chr9	33948373	33953474
chr9	33948373	33956146
chr9	33948373	33963791
chr9	33948373	33973237
chr9	33948373	33996333
chr9	33953284	33956146
chr9	33953284	33960878
chr9	33953284	33963791
chr9	33953284	33973237
chr9	33953284	34017189
chr9	33956078	34017189
chr9	33960825	33973237
chr9	33971650	33973237
chr9	33971650	34017189
chr9	33986759	34017189
chr9	33996222	34017189
chr9	34096715	34098517
chr9	34096715	34125277
chr9	34106433	34107565
chr9	34220907	34242108
chr9	34234215	34242108
chr9	34263100	34271930
chr9	34269256	34271930
chr9	34269256	34286704
chr9	34269256	34306441
chr9	34271808	34290389
chr9	34286616	34290389
chr9	34286616	34297114
chr9	35067890	35068362
chr9	35100597	35101962
chr9	35228014	35237826
chr9	35228014	35243364
chr9	35228014	35259050
chr9	35231119	35243364
chr9	35231119	35259050
chr9	35236468	35243364
chr9	35236468	35259050
chr9	35295695	35313989
chr9	35295695	35366993
chr9	35376027	35382507

chr9	35555059	35556448
chr9	35558196	35559272
chr9	35703774	35705670
chr9	36052264	36065624
chr9	36198978	36204179
chr9	36222776	36249397
chr9	36227247	36246482
chr9	36233919	36246482
chr9	36246030	36249397
chr9	36344831	36353331
chr9	36351114	36353331
chr9	36351114	36357942
chr9	36351114	36376127
chr9	36352741	36390616
chr9	36369718	36390616
chr9	36581643	36589652
chr9	36581643	36607673
chr9	36583626	36589652
chr9	36594627	36607673
chr9	36594627	36633200
chr9	36594627	36643083
chr9	36594627	36651877
chr9	36597221	36607673
chr9	36597221	36643083
chr9	36607574	36643083
chr9	36607578	36643083
chr9	36630298	36651877
chr9	36642996	36665581
chr9	36651745	36665581
chr9	36657240	36674937
chr9	36669309	36674937
chr9	37126311	37305714
chr9	37126311	37327834
chr9	37126311	37349452
chr9	37302187	37305714
chr9	37302187	37349452
chr9	37305543	37354824
chr9	37327798	37354824
chr9	37424844	37426654
chr9	37424844	37432138
chr9	37486702	37498224
chr9	37489314	37493703
chr9	37495168	37498224
chr9	37515903	37532058
chr9	37521568	37541774

chr9	37819287	37842286
chr9	37948634	37956054
chr9	38575475	38578148
chr9	3879426	3898835
chr9	3879426	3932470
chr9	3932359	4125941
chr9	40993252	40994699
chr9	40993252	41011730
chr9	41008880	41011730
chr9	41025763	41031512
chr9	41031370	41053899
chr9	41953182	42013525
chr9	4718418	4722625
chr9	4823547	4827033
chr9	4823547	4849550
chr9	5021962	5050831
chr9	5021962	5055788
chr9	5064882	5081861
chr9	539515	540667
chr9	5656582	5690038
chr9	5689958	5720750
chr9	5732387	5738538
chr9	5732387	5757451
chr9	5770086	5773080
chr9	5801175	5805785
chr9	5810010	5824001
chr9	6420911	6434173
chr9	6420911	6460791
chr9	6460572	6475500
chr9	6475390	6477808
chr9	6475390	6482099
chr9	6477621	6482099
chr9	6477621	6486925
chr9	6493825	6499931
chr9	65682765	65693473
chr9	65682765	65700337
chr9	65682765	65720092
chr9	65690966	65708958
chr9	6587140	6620319
chr9	6602108	6606669
chr9	67173859	67201219
chr9	67188784	67201219
chr9	676889	731266
chr9	6814630	6893232
chr9	6849506	6893232

chr9	6880011	6893232
chr9	69046384	69053260
chr9	69046384	69065035
chr9	69220886	69240147
chr9	69220886	69252900
chr9	69234438	69252900
chr9	69236027	69237136
chr9	69246689	69252900
chr9	69718649	69741858
chr9	69723325	69750950
chr9	69732138	69734254
chr9	69732138	69741858
chr9	69732138	69750950
chr9	6984165	7011879
chr9	7011697	7015929
chr9	7011697	7049200
chr9	7011697	7128236
chr9	70264303	70267975
chr9	70267922	70278625
chr9	70277309	70280899
chr9	70277309	70286271
chr9	70280758	70282583
chr9	70347612	70348038
chr9	7103684	7128236
chr9	71740062	71750385
chr9	71866798	71875083
chr9	71870082	71875083
chr9	72360627	72363606
chr9	734747	740934
chr9	74996446	74999870
chr9	74996446	75017448
chr9	75016267	75017448
chr9	76067954	76107351
chr9	77214328	77227485
chr9	77225925	77227485
chr9	77225925	77228264
chr9	77228121	77273364
chr9	77238001	77238386
chr9	77238001	77260224
chr9	77247258	77252352
chr9	77247258	77260224
chr9	77247258	77283650
chr9	77275497	77293508
chr9	77339515	77351446
chr9	77381975	77407607

chr9	77403235	77407607
chr9	77794462	77797648
chr9	77794462	77815770
chr9	78304734	78317804
chr9	78306313	78308583
chr9	79612655	79654075
chr9	79627373	79654075
chr9	79652589	79654075
chr9	79652592	79654075
chr9	79704782	79709699
chr9	79708117	79720293
chr9	79709622	79721888
chr9	81590804	81593274
chr9	81610219	81620557
chr9	83290602	83313747
chr9	83309535	83313747
chr9	83343189	83389708
chr9	83663874	83678599
chr9	83665029	83677961
chr9	83665029	83678599
chr9	83677726	83678599
chr9	83677726	83680037
chr9	83677726	83686155
chr9	83678440	83683066
chr9	83678440	83686155
chr9	83679774	83683066
chr9	83679774	83686155
chr9	83682950	83686155
chr9	83753257	83768970
chr9	83768817	83788678
chr9	83780381	83788678
chr9	83799184	83806560
chr9	83908451	83915678
chr9	83970736	83972189
chr9	85575314	85579096
chr9	85575314	85633374
chr9	85585462	85596449
chr9	85596361	85633374
chr9	85596361	85646418
chr9	85618982	85621285
chr9	85618982	85633374
chr9	85618982	85657643
chr9	85618982	85660973
chr9	85618982	85712566
chr9	85632661	85657643

chr9	85642826	85660973
chr9	85642826	85712566
chr9	85655142	85692813
chr9	85669484	85681335
chr9	85669484	85692813
chr9	85669484	85712566
chr9	85672549	85681335
chr9	85677435	85692813
chr9	85677435	85712566
chr9	85681267	85712566
chr9	85815878	85823080
chr9	85822879	85829606
chr9	85822879	85830576
chr9	85829462	85840849
chr9	85956359	85962180
chr9	85958471	85962180
chr9	85959792	85996577
chr9	85962012	85978381
chr9	85962012	86009931
chr9	85974966	85978381
chr9	86013045	86018821
chr9	86016538	86018821
chr9	86033281	86040868
chr9	86033281	86046572
chr9	86040738	86052591
chr9	86077411	86079341
chr9	86303085	86304947
chr9	86305191	86310017
chr9	86308428	86310017
chr9	86308428	86322475
chr9	86310705	86319670
chr9	86317218	86322475
chr9	86343074	86346480
chr9	86346298	86353230
chr9	88426381	88448989
chr9	88426381	88462749
chr9	88426381	88468605
chr9	89328659	89332986
chr9	89396736	89418118
chr9	91296020	91325404
chr9	91296020	91356155
chr9	92034809	92038365
chr9	92055404	92068098
chr9	92067965	92080963
chr9	92222519	92229126

chr9	92222519	92253453
chr9	92240855	92269983
chr9	92242153	92269983
chr9	92247376	92251885
chr9	92256679	92260234
chr9	92256679	92269983
chr9	92256679	92285839
chr9	92258853	92265553
chr9	92262968	92265553
chr9	92264928	92265553
chr9	92265479	92269983
chr9	92268173	92269983
chr9	92268173	92285839
chr9	92268173	92288282
chr9	92277862	92289426
chr9	92285721	92288282
chr9	92285721	92289426
chr9	92287790	92288282
chr9	92299889	92305830
chr9	92310170	92311259
chr9	92640709	92652639
chr9	92648058	92658681
chr9	92813595	92822277
chr9	92855810	92856318
chr9	93471140	93476338
chr9	93471140	93497599
chr9	93471140	93498886
chr9	93471140	93516269
chr9	93471140	93532329
chr9	93476255	93497599
chr9	93476255	93498886
chr9	93476255	93516269
chr9	93476255	93532329
chr9	93497470	93498886
chr9	93497470	93529580
chr9	93515669	93516269
chr9	93527154	93550691
chr9	93556381	93561250
chr9	93561108	93562304
chr9	93629969	93636525
chr9	93674926	93676963
chr9	94064733	94088263
chr9	94064733	94095454
chr9	94085250	94088263
chr9	94278757	94293069

chr9	94415157	94441099
chr9	94438400	94441099
chr9	94438400	94446936
chr9	94440984	94451527
chr9	94759648	94792918
chr9	94773001	94801002
chr9	94792764	94801002
chr9	94923985	94928531
chr9	94923985	94956015
chr9	94923985	94967801
chr9	94955176	95005616
chr9	95107065	95111637
chr9	95111462	95172147
chr9	95125085	95150087
chr9	95135345	95172147
chr9	95240648	95249369
chr9	95247431	95249369
chr9	95476033	95485874
chr9	95880868	95907271
chr9	95897848	95921315
chr9	95897848	95928864
chr9	95897848	95941549
chr9	95897848	95956013
chr9	95907077	95916434
chr9	95907077	95921315
chr9	95907077	95928864
chr9	95921174	95941549
chr9	95928078	95941549
chr9	95928078	95970656
chr9	95978060	96004701
chr9	96320997	96324169
chr9	96336716	96352109
chr9	96343903	96352109
chr9	96360153	96368305
chr9	96392397	96398314
chr9	96458378	96465498
chr9	96458378	96465778
chr9	96458378	96471094
chr9	96458378	96484633
chr9	96465336	96465778
chr9	96465336	96471094
chr9	96465336	96484633
chr9	96509672	96565483
chr9	96522505	96534542
chr9	96522505	96565483

chr9	96533926	96534542
chr9	96533926	96541892
chr9	96533926	96565483
chr9	96539077	96565483
chr9	96541825	96565483
chr9	96551795	96565483
chr9	96562692	96565483
chr9	96774787	96776245
chr9	96827079	96845016
chr9	96827079	96852005
chr9	96827079	96853069
chr9	96949554	96972948
chr9	97034725	97035693
chr9	97276568	97281669
chr9	97347313	97350555
chr9	97354568	97357725
chr9	97369921	97371706
chr9	97439244	97441875
chr9	97610345	97611699
chr9	97647491	97648223
chr9	97647491	97654944
chr9	97653797	97656085
chr9	97994630	98005153
chr9	97994630	98012472
chr9	98076917	98078347
chr9	98091908	98095029
chr9	98091908	98100164
chr9	98128606	98157660
chr9	98201478	98221228
chr9	98213107	98221228
chr9	98745558	98771046
chr9	98751028	98771046
chr9	98777404	98782573
chr9	98780188	98782573
chr9	99832688	99833454
chr9	99844648	99847615
chr9	99915177	99928843
chrX	100689855	100691209
chrX	100823885	100832909
chrX	10090770	10098579
chrX	101018970	101019403
chrX	101018970	101020588
chrX	101018970	101023616
chrX	101020486	101038051
chrX	101021967	101023616

chrX	101035612	101038051
chrX	101035612	101041371
chrX	101035612	101042312
chrX	101037916	101042312
chrX	101041316	101042312
chrX	101101057	101102411
chrX	101101057	101109998
chrX	101101057	101120784
chrX	101101057	101127665
chrX	101101057	101132456
chrX	101120401	101148161
chrX	101126708	101146277
chrX	101126708	101148161
chrX	101127137	101130073
chrX	101127137	101148161
chrX	101129981	101145199
chrX	10482479	10495691
chrX	106853520	106854296
chrX	106978079	106986200
chrX	106985095	106986200
chrX	107067555	107069402
chrX	107147742	107153503
chrX	107526581	107530511
chrX	107639294	107641000
chrX	107639294	107642490
chrX	107639294	107647765
chrX	107840669	107854704
chrX	107840669	107905626
chrX	107903957	107917649
chrX	108170608	108188677
chrX	108595508	108597568
chrX	108595508	108603061
chrX	108598701	108603061
chrX	108601392	108655457
chrX	108620258	108626349
chrX	109659353	109683666
chrX	109668100	109669173
chrX	109674401	109678415
chrX	109674401	109682896
chrX	111730524	111736879
chrX	112804946	112815877
chrX	115122526	115130199
chrX	115122526	115150070
chrX	115629197	115640503
chrX	115629197	115643508

chrX	115646401	115648017
chrX	11760402	11762252
chrX	11762129	11762997
chrX	118378418	118393271
chrX	118387339	118398470
chrX	118387339	118410959
chrX	118392631	118395344
chrX	118392631	118398470
chrX	118394054	118404444
chrX	118394054	118407026
chrX	118394054	118444494
chrX	118396969	118398470
chrX	118396969	118407026
chrX	118396969	118441559
chrX	118398386	118404444
chrX	118404337	118407026
chrX	118404337	118410959
chrX	118441367	118442345
chrX	118441367	118443687
chrX	118441367	118444494
chrX	118442243	118444494
chrX	118542724	118543593
chrX	118542724	118546116
chrX	118654601	118654961
chrX	118741016	118761289
chrX	118741016	118766976
chrX	118741016	118776511
chrX	118746953	118766976
chrX	118749657	118776511
chrX	118758054	118766976
chrX	118766529	118776511
chrX	119406459	119410362
chrX	119542504	119545504
chrX	119625334	119633492
chrX	119640691	119653040
chrX	119640691	119663677
chrX	119843190	119851601
chrX	119931935	119936682
chrX	119936296	119938810
chrX	120447840	120449128
chrX	120447840	120455570
chrX	120532421	120534586
chrX	120534480	120535943
chrX	123610917	123613708
chrX	123613638	123614189

chrX	123622757	123624669
chrX	123625911	123627968
chrX	123631687	123639027
chrX	123633952	123639027
chrX	123665641	123671761
chrX	123665641	123686714
chrX	123665641	123703505
chrX	123671668	123686714
chrX	123671668	123697751
chrX	123686547	123697751
chrX	123686547	123703505
chrX	123686547	123706949
chrX	123686547	123712908
chrX	123696020	123712908
chrX	123885630	123892773
chrX	123885630	123900693
chrX	123888618	123892773
chrX	123888618	123900693
chrX	124021366	124031125
chrX	124021366	124037623
chrX	124021366	124042645
chrX	124022530	124042645
chrX	124068563	124078058
chrX	124068563	124081528
chrX	124071148	124095449
chrX	124081379	124086770
chrX	12799206	12810146
chrX	1282677	1290509
chrX	1282677	1300626
chrX	1285777	1290509
chrX	1288518	1288888
chrX	1288518	1290509
chrX	129465519	129471326
chrX	129465519	129481185
chrX	129480700	129516497
chrX	129490059	129511983
chrX	129515686	129518447
chrX	129548562	129559001
chrX	129557324	129559001
chrX	129557324	129569399
chrX	129558632	129559001
chrX	129558839	129562786
chrX	129558839	129569399
chrX	129558839	129575250
chrX	129565771	129569399

chrX	129810904	129811509
chrX	129823678	129829141
chrX	129908059	129911921
chrX	129919174	129921587
chrX	130005187	130016213
chrX	130069299	130072417
chrX	130071039	130072417
chrX	130074048	130074752
chrX	130139794	130156603
chrX	130226758	130260465
chrX	130236213	130247005
chrX	130656580	130679748
chrX	131709497	131794466
chrX	131749305	131756709
chrX	131749305	131768696
chrX	131749305	131784039
chrX	131749305	131794466
chrX	131755597	131784039
chrX	131768548	131784039
chrX	131768548	131794466
chrX	131783886	131794466
chrX	134377571	134378106
chrX	134377571	134393634
chrX	134377571	134425330
chrX	134413490	134417302
chrX	134473358	134475364
chrX	134473358	134493590
chrX	134473358	134498436
chrX	134473358	134498684
chrX	134781820	134789266
chrX	134804100	134807708
chrX	135545422	135549805
chrX	135545422	135552146
chrX	135545422	135556300
chrX	136022585	136033493
chrX	136219397	136222486
chrX	136236243	136241277
chrX	136241159	136244795
chrX	136872267	136875585
chrX	13735247	13749533
chrX	13736477	13744519
chrX	139737915	139757867
chrX	139757807	139774953
chrX	139768259	139785299
chrX	139782546	139787244

chrX	139802235	139826823
chrX	139814877	139826823
chrX	1412731	1439144
chrX	1421657	1425687
chrX	1421657	1428121
chrX	1425524	1439144
chrX	1435021	1439144
chrX	147921932	147932763
chrX	147925539	147932763
chrX	147928321	147945616
chrX	147940575	147945616
chrX	14850504	14859334
chrX	148651998	148662768
chrX	149490313	149501037
chrX	150445453	150471490
chrX	150462771	150473802
chrX	150462771	150510046
chrX	150592604	150619139
chrX	150596497	150619139
chrX	150614588	150619139
chrX	150614588	150641418
chrX	150619037	150658027
chrX	150638942	150649901
chrX	150657820	150660484
chrX	150699194	150718700
chrX	150699194	150732730
chrX	150699194	150750843
chrX	150718624	150744453
chrX	150718624	150755865
chrX	150727214	150732730
chrX	150727214	150737448
chrX	150727214	150750843
chrX	150736594	150737448
chrX	150793690	150795486
chrX	150793690	150816078
chrX	152189729	152208144
chrX	152284667	152364596
chrX	152345580	152364596
chrX	152846281	152850423
chrX	152846281	152858916
chrX	152963838	152970541
chrX	153594546	153596187
chrX	153702006	153704094
chrX	153787822	153789824
chrX	153789711	153790851

chrX	153796433	153798136
chrX	153963224	153964726
chrX	154016031	154017067
chrX	15425905	15459740
chrX	154351580	154368090
chrX	154354824	154357623
chrX	154361307	154361787
chrX	154361669	154362784
chrX	154361978	154362332
chrX	154364820	154368090
chrX	154366307	154367544
chrX	154399337	154399941
chrX	154399486	154399941
chrX	154399486	154400626
chrX	154413206	154420735
chrX	154413481	154419623
chrX	154446414	154449297
chrX	154507405	154515812
chrX	154512313	154512936
chrX	154556164	154560559
chrX	15455847	15459740
chrX	154695916	154700059
chrX	154712228	154716329
chrX	154764898	154767382
chrX	154764898	154770879
chrX	154765906	154768432
chrX	154768301	154770879
chrX	154789953	154792285
chrX	155116056	155120168
chrX	155506897	155514265
chrX	155506897	155524632
chrX	155506897	155545277
chrX	155511672	155545277
chrX	155889457	155900587
chrX	155898111	155919880
chrX	15764577	15774397
chrX	1593443	1595532
chrX	16667513	16702941
chrX	16678361	16683226
chrX	16693140	16693487
chrX	16693140	16702941
chrX	16698235	16702941
chrX	16818573	16820255
chrX	16852045	16853842
chrX	16857593	16869220

chrX	17687741	17692468
chrX	18247768	18257030
chrX	18256847	18260291
chrX	18256847	18265802
chrX	18265584	18305215
chrX	18304971	18334095
chrX	18442374	18510854
chrX	18506934	18510854
chrX	18575353	18588143
chrX	18608810	18619966
chrX	18905757	18910960
chrX	18948743	18954412
chrX	19349311	19350110
chrX	19349311	19353173
chrX	19392001	19400663
chrX	19398225	19400663
chrX	19407187	19415257
chrX	19407187	19457019
chrX	19425530	19464406
chrX	19456912	19464406
chrX	19541924	19546050
chrX	19588642	19595000
chrX	19588642	19645475
chrX	19607937	19684028
chrX	19683822	19695741
chrX	19695611	19836282
chrX	19929784	19935898
chrX	19950752	19955566
chrX	20162963	20167747
chrX	20172745	20177084
chrX	20186295	20195145
chrX	20186295	20204103
chrX	20186295	20209404
chrX	20193486	20204103
chrX	20204021	20234814
chrX	21516484	21532067
chrX	21516484	21563452
chrX	21526866	21532067
chrX	21851508	21853503
chrX	21868466	21869678
chrX	21878041	21878692
chrX	21971896	21972571
chrX	2243022	2291603
chrX	2266739	2291603
chrX	2266739	2425304

chrX	23679164	23683705
chrX	23704693	23706739
chrX	23707876	23722753
chrX	23713134	23722753
chrX	23713134	23730564
chrX	23721880	23736016
chrX	23722669	23733217
chrX	23730526	23733217
chrX	23856301	23868688
chrX	24057420	24057754
chrX	24057420	24060182
chrX	24064200	24068108
chrX	24064200	24071727
chrX	24065997	24071727
chrX	2408744	2425304
chrX	24172714	24179770
chrX	24494741	24505298
chrX	24518932	24534068
chrX	24723154	24748992
chrX	24732369	24743329
chrX	24745417	24843677
chrX	24809897	24826601
chrX	24809897	24843677
chrX	24814978	24821583
chrX	24814978	24843677
chrX	24821451	24843677
chrX	24821451	24888122
chrX	24841651	24843677
chrX	2610995	2626642
chrX	2612149	2626642
chrX	2615815	2626642
chrX	2618739	2626642
chrX	2719660	2726373
chrX	2722626	2726373
chrX	2915555	2918227
chrX	30665510	30677452
chrX	30859486	30871801
chrX	338603	341931
chrX	338603	347693
chrX	338603	361590
chrX	345515	347693
chrX	345515	361590
chrX	3621258	3641971
chrX	3626418	3641971
chrX	37386598	37426000

chrX	37406208	37426000
chrX	40062138	40064599
chrX	40588985	40599741
chrX	40659226	40659607
chrX	40679863	40688530
chrX	40712913	40729345
chrX	41128999	41134837
chrX	41144521	41151057
chrX	41196250	41205493
chrX	41198527	41210682
chrX	41205302	41219231
chrX	41210508	41219231
chrX	41223216	41225137
chrX	41542690	41671530
chrX	41626603	41671530
chrX	41660438	41671530
chrX	44961283	45020730
chrX	45010960	45020730
chrX	45010960	45037689
chrX	45034930	45053955
chrX	45051708	45063817
chrX	46620976	46648797
chrX	46669606	46682535
chrX	46984883	46998277
chrX	46984883	47033788
chrX	47024723	47028103
chrX	47846104	47895940
chrX	48575167	48577109
chrX	48576313	48576604
chrX	48934463	48935018
chrX	48976017	48997357
chrX	49115725	49117810
chrX	49115725	49119814
chrX	49115725	49122776
chrX	49242015	49243462
chrX	51895052	51897271
chrX	52955153	52956755
chrX	53217142	53218398
chrX	53394777	53396617
chrX	53403565	53403893
chrX	53405011	53405391
chrX	53409061	53409503
chrX	53414757	53415169
chrX	53535383	53539080
chrX	53539656	53542539

chrX	53539656	53549505
chrX	53557381	53558809
chrX	53557381	53559060
chrX	53557381	53560416
chrX	53561755	53562244
chrX	53561755	53562929
chrX	53568691	53569827
chrX	53580830	53583916
chrX	53595186	53607699
chrX	53600117	53604834
chrX	53614533	53615835
chrX	53615743	53617446
chrX	53616969	53617446
chrX	53624594	53631034
chrX	53632486	53680186
chrX	53645310	53654131
chrX	53987077	53987944
chrX	54022258	54022843
chrX	54253958	54259338
chrX	54465450	54465852
chrX	54467783	54471487
chrX	55729293	55731586
chrX	55729293	55757331
chrX	63655493	63678572
chrX	63665885	63678572
chrX	63665885	63706449
chrX	63665885	63724711
chrX	63674037	63724711
chrX	63697124	63706449
chrX	63697124	63724711
chrX	64328771	64337393
chrX	64331556	64337393
chrX	64345044	64359527
chrX	64348659	64350070
chrX	64348659	64359527
chrX	64349941	64359527
chrX	64354776	64356338
chrX	65523559	65532630
chrX	68048415	68073299
chrX	68052539	68053810
chrX	68052539	68073299
chrX	68063853	68073299
chrX	68063853	68113239
chrX	68096869	68113239
chrX	68096869	68194498

chrX	68096869	68206673
chrX	68096869	68299096
chrX	68111853	68119332
chrX	68111853	68194498
chrX	68192918	68201710
chrX	68192918	68206673
chrX	68192918	68210282
chrX	68192918	68212212
chrX	68192918	68299096
chrX	68234486	68299096
chrX	68274737	68299096
chrX	68511848	68513405
chrX	68511848	68521497
chrX	68511848	68522917
chrX	68513326	68521497
chrX	68513326	68522917
chrX	70146632	70150322
chrX	70148760	70150322
chrX	70290437	70290805
chrX	70290437	70302398
chrX	70296997	70302398
chrX	70296997	70333689
chrX	70329404	70333689
chrX	70341798	70353807
chrX	70341798	70376210
chrX	70341798	70387297
chrX	70386617	70404034
chrX	70404714	70407075
chrX	70406258	70407075
chrX	71137557	71137943
chrX	71290628	71294528
chrX	71368053	71375286
chrX	71368053	71387461
chrX	71381742	71387461
chrX	71381742	71389665
chrX	71383961	71389665
chrX	71397252	71398737
chrX	71397252	71401739
chrX	71398571	71401739
chrX	71398571	71408151
chrX	71421308	71424238
chrX	71423116	71424238
chrX	71536177	71538072
chrX	71536177	71544635
chrX	71555953	71557672

chrX	71556679	71564753
chrX	72273800	72276234
chrX	72275543	72276234
chrX	72351732	72464731
chrX	72462003	72464731
chrX	72462003	72489041
chrX	72462003	72491006
chrX	72462003	72495268
chrX	72488932	72495268
chrX	72490928	72495268
chrX	72567888	72568884
chrX	72602157	72605619
chrX	72605270	72619305
chrX	72635154	72636386
chrX	72656119	72676150
chrX	72666150	72667473
chrX	73577406	73584572
chrX	75060222	75070522
chrX	75065069	75069136
chrX	75098941	75114834
chrX	75112885	75114834
chrX	75379119	75431520
chrX	75417086	75431520
chrX	75417086	75505847
chrX	75421863	75431520
chrX	75450801	75505847
chrX	77520787	77593849
chrX	77557450	77558846
chrX	77557450	77574358
chrX	77557450	77593849
chrX	77557450	77600564
chrX	77574249	77593849
chrX	77599410	77616730
chrX	77616612	77620532
chrX	77654097	77664778
chrX	77663381	77664778
chrX	77663381	77676298
chrX	77688817	77696704
chrX	77688817	77698629
chrX	77688817	77717243
chrX	77830804	77841320
chrX	77856732	77857497
chrX	77870807	77875597
chrX	77988241	78003236
chrX	78012878	78020398

chrX	78014661	78020398
chrX	78109866	78114160
chrX	78113743	78114160
chrX	78122834	78123374
chrX	78122834	78125425
chrX	78133337	78138098
chrX	80288905	80291024
chrX	80288905	80310233
chrX	80289663	80310233
chrX	80695907	80736088
chrX	80699956	80704846
chrX	80707426	80719656
chrX	80716156	80736088
chrX	80719488	80730020
chrX	80728751	80793772
chrX	80735987	80793772
chrX	80744031	80745729
chrX	80744031	80793772
chrX	80791853	80793772
chrX	84145477	84164387
chrX	84333758	84361612
chrX	84344249	84361612
chrX	84468471	84475397
chrX	85046445	85055925
chrX	85067126	85074391
chrX	85255258	85270146
chrX	85873051	85911338
chrX	85873051	85964052
chrX	85956152	85964052
chrX	85978766	86027557
chrX	96735757	96738762
chrX	9684042	9697429
chrX	9688016	9692254
chrX	9688016	9697429
chrX	96881578	96918617
chrX	96912327	96918617
chrX	96912327	96930843
chrX	9691578	9692254
chrX	96930732	96945591
chrX	96937232	96945591
chrX	96937232	96965207
chrX	96937232	97075261
chrX	96939265	97247839
chrX	96948934	96965207
chrX	97072940	97075261

chrX	97099693	97114965
chrX	97114725	97348280
chrX	97247714	97348280
	**Intron (3321)**	
chr10	100221357	100221747
chr10	100917035	100924289
chr10	102109424	102109674
chr10	1022562	1026820
chr10	102482010	102483098
chr10	102487935	102489553
chr10	103178796	103179036
chr10	103389417	103390429
chr10	103390030	103390521
chr10	103410745	103410935
chr10	103602610	103603852
chr10	1056347	1069552
chr10	11475531	11476966
chr10	118744925	118745319
chr10	119160438	119160705
chr10	119213418	119214069
chr10	119575690	119580538
chr10	12077519	12078146
chr10	122488714	122492038
chr10	12372540	12373719
chr10	12397268	12403369
chr10	12400015	12403369
chr10	12400015	12403411
chr10	12400720	12403369
chr10	12400720	12403411
chr10	12403101	12406177
chr10	12403101	12406326
chr10	12406177	12432033
chr10	124462502	124462962
chr10	124759999	124765578
chr10	124777061	124777777
chr10	124807560	124815997
chr10	124940373	124962408
chr10	124940373	124994706
chr10	124942456	124962408
chr10	125069386	125070892
chr10	125074742	125111882
chr10	125074742	125122868
chr10	125074742	125138986
chr10	127000174	127012312
chr10	127051841	127052273

chr10	127924270	127967074
chr10	133491221	133556677
chr10	13849756	13849937
chr10	18513405	18551939
chr10	18553509	18562797
chr10	18556308	18558216
chr10	18601787	18603702
chr10	18603780	18603923
chr10	21085109	21088333
chr10	21826160	21826400
chr10	21875847	21876220
chr10	245900	263658
chr10	245900	267102
chr10	27143009	27143446
chr10	27175450	27175826
chr10	27197902	27198872
chr10	30313890	30349277
chr10	30970906	30974652
chr10	30972169	30974652
chr10	31355143	31358333
chr10	31355143	31387266
chr10	31355146	31387266
chr10	31373017	31387266
chr10	31373017	31387798
chr10	31373017	31389695
chr10	31373017	31391224
chr10	31373121	31387266
chr10	31387123	31387798
chr10	31901744	31902084
chr10	319207	320648
chr10	33280904	33281159
chr10	34356762	34357261
chr10	34493285	34493725
chr10	34799340	34799743
chr10	35030505	35030791
chr10	35080585	35080982
chr10	35375404	35375885
chr10	35434112	35435247
chr10	37853230	37853628
chr10	37952962	37972021
chr10	42802079	42810193
chr10	49960135	49969883
chr10	50885237	50943674
chr10	545404	546112
chr10	5689285	5714207

chr10	5699524	5714207
chr10	5709529	5714207
chr10	5718649	5719456
chr10	5730915	5742364
chr10	5783323	5812601
chr10	5801206	5801463
chr10	584823	585703
chr10	5884654	5885290
chr10	63271995	63272361
chr10	63453395	63508047
chr10	65585484	65585802
chr10	65585484	65670844
chr10	65637869	65699112
chr10	68127282	68131112
chr10	68391865	68392158
chr10	68416664	68430660
chr10	68454728	68455048
chr10	68504279	68505542
chr10	68522737	68523199
chr10	68577716	68586272
chr10	68761575	68762041
chr10	68922534	68923054
chr10	68966203	68966537
chr10	68974915	68975935
chr10	69916483	70007536
chr10	71977570	71978126
chr10	72339946	72340163
chr10	72708282	72741845
chr10	72708282	72742273
chr10	72708282	72790331
chr10	72736484	72742273
chr10	72741721	72742273
chr10	73129422	73129759
chr10	73176061	73186478
chr10	73182959	73186478
chr10	73484975	73486415
chr10	73831824	73832388
chr10	74022453	74022731
chr10	74057200	74057677
chr10	74240927	74243033
chr10	74358443	74528093
chr10	74369558	74435748
chr10	74435574	74454331
chr10	75169700	75170975
chr10	75685991	75814212

chr10	75796206	75799657
chr10	76396916	76403366
chr10	77493797	77495103
chr10	7770208	7771665
chr10	79160595	79161119
chr10	79349938	79350522
chr10	807848	817884
chr10	87330891	87334310
chr10	87875404	87880438
chr10	900269	900729
chr10	90882877	90888357
chr10	91807141	91807445
chr10	91958262	91958488
chr10	92012328	92012686
chr10	92196893	92227975
chr10	92346743	92347234
chr10	92925680	93058335
chr10	93414474	93414838
chr10	94429758	94443423
chr10	94520458	94520754
chr10	94559713	94560418
chr10	94559730	94560410
chr10	94565244	94567393
chr10	95173067	95228430
chr10	95691451	95692243
chr10	96056895	96058294
chr10	97283073	97283361
chr10	97288321	97291261
chr10	97604966	97606236
chr10	989997	990473
chr10	993535	1014355
chr10	99907300	99907490
chr10	99919709	99920185
chr11	102177094	102177343
chr11	102349877	102379913
chr11	102349877	102390613
chr11	102350345	102390613
chr11	107796051	107796788
chr11	108246697	108246898
chr11	108261352	108276790
chr11	108929488	108940335
chr11	110125595	110127618
chr11	110241595	110242205
chr11	110430681	110443085
chr11	110441153	110442999

chr11	113809970	113810769
chr11	114400016	114540760
chr11	114495572	114540760
chr11	114501659	114540760
chr11	117023972	117024639
chr11	117240595	117243241
chr11	117254225	117254640
chr11	118582360	118582804
chr11	119004375	119005329
chr11	119009380	119009891
chr11	119211307	119211542
chr11	120575668	120577074
chr11	122116132	122128092
chr11	122128024	122132832
chr11	122661930	122664820
chr11	122670857	122671886
chr11	122712171	122714877
chr11	122712171	122716499
chr11	123081862	123082661
chr11	123126819	123127231
chr11	123132999	123134618
chr11	123187620	123189422
chr11	124767343	124784719
chr11	14296054	14356769
chr11	14311116	14311629
chr11	14358758	14359050
chr11	14461247	14468728
chr11	17845270	18011762
chr11	17863565	18010139
chr11	17873506	18010139
chr11	17874219	17874477
chr11	17926737	18010139
chr11	18336376	18337175
chr11	19762228	19762631
chr11	19823618	19825016
chr11	20390985	20391281
chr11	2585940	2586213
chr11	28227994	28230985
chr11	2986189	2986859
chr11	2986296	2986727
chr11	31452717	31488241
chr11	31452845	31454387
chr11	3361975	3369502
chr11	3395712	3397406
chr11	35139302	35176734

chr11	36001085	36038184
chr11	36022493	36038184
chr11	36180660	36197771
chr11	378069	378221
chr11	45805995	45812567
chr11	46355549	46355841
chr11	46359050	46359698
chr11	46604294	46605835
chr11	46757459	46757669
chr11	46811769	46812071
chr11	47060039	47060300
chr11	47142612	47143561
chr11	47158649	47160186
chr11	47502966	47506897
chr11	47738487	47738790
chr11	47800144	47800523
chr11	48069699	48070069
chr11	57326074	57326858
chr11	59620414	59639521
chr11	61414419	61414998
chr11	61807352	61808104
chr11	62561475	62562711
chr11	62561499	62562711
chr11	62569396	62570171
chr11	62578467	62582092
chr11	62585093	62585593
chr11	62585890	62588938
chr11	62604439	62607569
chr11	62809596	62815117
chr11	63684395	63686185
chr11	63686794	63692052
chr11	63687517	63688318
chr11	63696326	63696590
chr11	63813704	63817959
chr11	63819341	63820256
chr11	63819441	63820161
chr11	63850874	63851367
chr11	64004812	64005210
chr11	64139068	64139693
chr11	64228264	64228894
chr11	64274907	64281098
chr11	6497449	6498939
chr11	65631545	65632871
chr11	65897995	65898726
chr11	65898455	65898727

chr11	66232198	66243410
chr11	66516415	66516915
chr11	66516670	66517078
chr11	66635523	66637163
chr11	66652276	66657259
chr11	66756756	66762450
chr11	66795041	66795396
chr11	67125061	67125553
chr11	67125121	67125522
chr11	67130423	67131044
chr11	67138066	67138505
chr11	67142626	67143103
chr11	67146590	67147464
chr11	67190317	67193084
chr11	67225677	67235399
chr11	67271704	67271920
chr11	67336577	67337142
chr11	68515027	68522689
chr11	68824170	68824908
chr11	68896584	68897983
chr11	6955614	6997020
chr11	72224779	72230538
chr11	73496858	73497219
chr11	73536139	73537300
chr11	73677720	73752045
chr11	73795058	73795209
chr11	73862534	73867487
chr11	73916518	73931616
chr11	74767960	74768323
chr11	74843801	74851018
chr11	74850929	74862280
chr11	74850929	74874895
chr11	74850929	74911425
chr11	74850943	74876977
chr11	75413519	75414320
chr11	75961452	76141414
chr11	76447069	76448680
chr11	76491466	76492184
chr11	77626561	77626801
chr11	77825902	77826149
chr11	77825922	77826168
chr11	77991659	77992462
chr11	78122670	78123060
chr11	78269052	78273808
chr11	78511913	78512299

chr11	840591	840986
chr11	857693	861423
chr11	86318935	86326676
chr11	8929436	8929694
chr11	90096818	90168505
chr11	9245550	9245715
chr11	93365346	93386041
chr11	93790830	93791135
chr11	9404394	9404621
chr11	9408517	9411470
chr11	9431882	9432296
chr11	94485215	94485688
chr11	96040790	96041915
chr11	96130348	96136347
chr11	96327703	96327882
chr11	9779462	9808144
chr11	9779887	9780575
chr12	100167069	100196979
chr12	100591671	100592449
chr12	1010388	1010605
chr12	102019284	102050291
chr12	10216278	10216634
chr12	102277673	102423180
chr12	104290487	104291000
chr12	104557295	104558269
chr12	104834090	104900301
chr12	104865442	104900301
chr12	105361064	105362102
chr12	106245903	106246469
chr12	106245906	106246466
chr12	108532505	108532896
chr12	108541329	108541648
chr12	108813137	108813490
chr12	109090817	109091289
chr12	109943422	109946069
chr12	11021544	11028515
chr12	11028400	11121180
chr12	110286191	110286970
chr12	110298625	110300449
chr12	110396786	110398007
chr12	11053032	11121180
chr12	11121009	11124187
chr12	11121009	11124323
chr12	11121009	11129363
chr12	11121009	11133367

chr12	11121009	11142155
chr12	111507184	111510444
chr12	111572323	111586331
chr12	111649179	111649771
chr12	111661979	111662290
chr12	111661983	111662844
chr12	111750368	111751477
chr12	112069742	112070856
chr12	112137248	112137758
chr12	112320667	112320928
chr12	112360906	112361376
chr12	113197540	113200813
chr12	113264614	113264870
chr12	115986511	115987113
chr12	116202955	116205153
chr12	116230432	116237632
chr12	116230432	116240462
chr12	116230532	116240462
chr12	11710496	11711395
chr12	117181928	117187189
chr12	117641747	117642386
chr12	1181061	1181538
chr12	120154842	120155716
chr12	120523932	120524370
chr12	121291123	121291811
chr12	121293199	121294042
chr12	121293258	121294014
chr12	121502582	121503065
chr12	121666426	121667042
chr12	121742845	121743275
chr12	121900892	121905318
chr12	121905658	121906833
chr12	122049797	122050025
chr12	122338639	122340190
chr12	122378385	122378865
chr12	122403786	122404352
chr12	122575226	122576090
chr12	122877801	122879505
chr12	123322528	123322692
chr12	123623298	123623554
chr12	123636824	123637597
chr12	124088263	124090937
chr12	125009567	125010817
chr12	125115342	125117062
chr12	126730762	126748077

chr12	128794032	128814803
chr12	12935258	12947238
chr12	131937794	131938326
chr12	131939193	131941746
chr12	131961198	132105304
chr12	132562050	132562924
chr12	132570173	132571158
chr12	132755805	132756151
chr12	132796603	132827713
chr12	132822019	132822649
chr12	1386249	1386434
chr12	15209268	15223969
chr12	21233699	21234299
chr12	26370522	26371140
chr12	27426131	27426317
chr12	28198942	28328191
chr12	28255580	28315308
chr12	2880260	2880674
chr12	2917784	2918208
chr12	29659879	29660885
chr12	3087421	3102532
chr12	31455790	31457976
chr12	31465597	31466499
chr12	31469250	31470224
chr12	31469256	31470224
chr12	31523356	31524392
chr12	32141739	32142134
chr12	32729737	32730134
chr12	32745679	32755172
chr12	32747075	32750839
chr12	38817894	38819529
chr12	39911985	40018935
chr12	40018845	40085700
chr12	42352608	42352870
chr12	46194915	46195176
chr12	46261295	46261851
chr12	47103692	47108164
chr12	48149326	48153525
chr12	48346221	48346734
chr12	48784897	48786593
chr12	48823350	48828121
chr12	49406472	49408042
chr12	49468370	49469184
chr12	49469795	49470126
chr12	49501883	49503071

chr12	50153876	50163308
chr12	50179828	50180440
chr12	50183733	50190127
chr12	50216459	50216642
chr12	50219407	50220170
chr12	50450778	50451421
chr12	50457688	50458046
chr12	50701619	50702005
chr12	51376095	51376440
chr12	51683741	51689096
chr12	5198501	5276889
chr12	54109820	54110220
chr12	54185810	54186391
chr12	54270411	54270961
chr12	54275925	54277127
chr12	55780376	55786559
chr12	55814841	55815536
chr12	56139360	56141838
chr12	56680268	56680572
chr12	56681065	56682627
chr12	57706454	57706843
chr12	57748983	57749722
chr12	57932009	57932421
chr12	62879115	62880653
chr12	63934632	63937998
chr12	63937844	64028707
chr12	6515395	6515978
chr12	6518751	6524421
chr12	65278734	65463323
chr12	6600551	6605995
chr12	66362029	66372018
chr12	68692486	68692844
chr12	69262615	69263154
chr12	69539352	69539977
chr12	6983001	6983206
chr12	6983001	6988369
chr12	6983001	6997342
chr12	70380196	70381594
chr12	71657525	71660427
chr12	71657548	71660427
chr12	71657974	71658347
chr12	71659346	71660427
chr12	71774742	71775553
chr12	7316960	7326019
chr12	7396287	7436602

chr12	76776324	76777002
chr12	79668274	79668843
chr12	85833336	85840709
chr12	8926371	8928156
chr12	92872315	92872709
chr12	9606352	9622436
chr12	96203848	96206411
chr12	98523893	98524353
chr13	102873242	102875914
chr13	107368491	107468528
chr13	107466014	107467250
chr13	107523563	107693167
chr13	107598840	107635379
chr13	107617216	107638963
chr13	107617216	107693167
chr13	107650237	107693167
chr13	107664846	107681862
chr13	107664846	107693167
chr13	107684166	107693167
chr13	110686524	110687442
chr13	112793463	112794620
chr13	113348354	113348604
chr13	113769720	113769935
chr13	114235719	114272665
chr13	114287526	114287842
chr13	19824919	19851957
chr13	20891829	20892174
chr13	20982586	20982773
chr13	23361047	23361515
chr13	23361051	23361520
chr13	23745852	23746121
chr13	23745852	23746124
chr13	23826330	23869387
chr13	23855033	23858922
chr13	24164371	24164513
chr13	24292024	24292705
chr13	24436447	24439230
chr13	24493688	24494717
chr13	24951594	24967453
chr13	26349116	26404155
chr13	26573714	26593722
chr13	27236444	27236858
chr13	28669590	28669897
chr13	28701789	28717655
chr13	29445578	29487935

chr13	29453067	29487935
chr13	29453067	29492665
chr13	29453067	29492707
chr13	29453067	29493753
chr13	29476766	29498462
chr13	29485085	29489765
chr13	29514352	29527365
chr13	29550203	29554673
chr13	29550203	29560345
chr13	29550203	29560349
chr13	30207641	30283687
chr13	30650056	30650358
chr13	31936448	31985734
chr13	31941307	31985734
chr13	32355106	32398359
chr13	32436712	32436990
chr13	32444544	32445017
chr13	32477886	32480637
chr13	32502343	32502929
chr13	32593020	32629771
chr13	41230052	41230334
chr13	44510314	44510702
chr13	49665564	49666450
chr13	49723836	49724678
chr13	49991273	50045232
chr13	50023901	50024385
chr13	52457143	52457568
chr13	60512356	60513540
chr13	63732971	63748397
chr13	77317250	77317678
chr13	87600955	87621158
chr13	95049613	95051776
chr13	95163141	95166264
chr13	98145769	98146323
chr14	100264095	100264858
chr14	101917925	101925341
chr14	102146429	102147125
chr14	102159235	102159536
chr14	102235608	102236294
chr14	102240309	102242175
chr14	102281246	102281689
chr14	102340541	102340795
chr14	102478043	102478297
chr14	102657125	102663938
chr14	102788132	102788523

chr14	102961827	102962618
chr14	102965232	102965696
chr14	103390239	103390731
chr14	103398950	103411765
chr14	103398950	103438333
chr14	103464530	103484981
chr14	103494854	103495269
chr14	103564146	103564571
chr14	103701856	103704429
chr14	103913286	103919908
chr14	104782781	104782997
chr14	105320553	105320939
chr14	20261612	20306134
chr14	20318454	20318844
chr14	21246054	21246464
chr14	21250127	21263829
chr14	21429170	21431003
chr14	22833885	22834553
chr14	22917704	22918131
chr14	22917712	22918132
chr14	24209889	24211852
chr14	26735241	26782208
chr14	29724469	29743343
chr14	29871748	29915357
chr14	31154569	31155107
chr14	31703116	31704138
chr14	32149679	32154245
chr14	34756730	34757120
chr14	35002261	35002527
chr14	35009132	35010187
chr14	35027240	35027542
chr14	35746406	35749164
chr14	45005099	45005428
chr14	45132726	45133222
chr14	45148875	45160830
chr14	45182215	45182504
chr14	49603575	49604240
chr14	50237676	50303034
chr14	50516022	50519626
chr14	50635213	50649389
chr14	54604371	54609625
chr14	54668791	54669024
chr14	55060436	55060888
chr14	58322129	58322701
chr14	58322180	58322748

chr14	58399240	58408414
chr14	58423104	58423257
chr14	61713935	61715603
chr14	63463024	63463249
chr14	64441452	64442032
chr14	64751411	64751792
chr14	64916822	64917171
chr14	65537599	65538688
chr14	67008385	67008600
chr14	67110464	67180986
chr14	67164175	67164586
chr14	67312057	67336738
chr14	68089208	68118479
chr14	68115818	68116112
chr14	68115818	68118479
chr14	68157668	68158239
chr14	69769510	69770042
chr14	70969743	70976621
chr14	71035816	71053412
chr14	73089048	73089455
chr14	73101598	73101789
chr14	73179582	73180018
chr14	73182746	73183212
chr14	73420249	73420559
chr14	73494152	73505073
chr14	73866414	73866812
chr14	73870674	73874676
chr14	74092583	74093689
chr14	74119855	74120164
chr14	74298569	74299794
chr14	75772937	75773613
chr14	77673559	77675794
chr14	77734660	77735405
chr14	77748885	77749519
chr14	77801679	77802690
chr14	80306851	80313579
chr14	80497468	80535062
chr14	85596238	85597407
chr14	86028368	86041833
chr14	89471273	89486671
chr14	89977062	89977392
chr14	90285081	90285532
chr14	91135557	91136029
chr14	91169009	91169491
chr14	92076906	92077727

chr14	93288500	93288865
chr14	94084190	94085264
chr14	96520126	96565214
chr14	96931330	96945382
chr14	96943516	96945382
chr14	96943516	96945386
chr14	96943516	96980767
chr14	96943516	96998134
chr14	99490105	99491973
chr14	99533075	99534095
GL000194v1_	92152	92446
chr15	100889866	100890010
chr15	25393745	25415842
chr15	28960370	28960854
chr15	32633934	32636408
chr15	34143889	34144680
chr15	38499490	38516226
chr15	40641378	40650819
chr15	40645691	40650819
chr15	40779083	40792856
chr15	40804263	40805117
chr15	40896579	40898497
chr15	41102592	41103499
chr15	41109854	41113376
chr15	41111425	41111953
chr15	41289571	41290214
chr15	41378680	41379048
chr15	41401352	41403355
chr15	41500207	41500747
chr15	41764045	41764348
chr15	42638707	42669351
chr15	43017732	43018112
chr15	43435185	43436122
chr15	43811039	43811183
chr15	43852324	43852624
chr15	43876959	43890703
chr15	44094068	44096203
chr15	44166844	44172981
chr15	44169196	44172981
chr15	44392127	44451387
chr15	44410792	44411135
chr15	44449541	44449777
chr15	44597930	44620697
chr15	45044780	45045449
chr15	49225204	49308777

chr15	49225204	49328130
chr15	49235624	49328130
chr15	49308607	49328130
chr15	49318971	49328130
chr15	51065428	51066305
chr15	51244629	51245314
chr15	51723364	51724946
chr15	51895475	51895854
chr15	51900164	51934354
chr15	56019395	56162240
chr15	56156515	56162240
chr15	56229655	56230879
chr15	56960660	56960903
chr15	57072652	57095163
chr15	58899730	58900531
chr15	58999566	59000465
chr15	63213988	63214386
chr15	63567616	63568079
chr15	63567627	63568061
chr15	64086334	64086723
chr15	64296414	64296612
chr15	64421189	64421431
chr15	64689235	64689998
chr15	64817467	64820506
chr15	64983180	64983893
chr15	65002885	65005718
chr15	65467476	65469070
chr15	65503895	65504440
chr15	65534348	65538937
chr15	67231229	67236805
chr15	67544612	67546400
chr15	67600644	67661494
chr15	67604824	67605108
chr15	67623874	67624045
chr15	67645801	67647921
chr15	68068340	68068497
chr15	69194435	69196745
chr15	72241388	72271226
chr15	74795957	74797185
chr15	75000317	75003635
chr15	75477502	75477935
chr15	75529283	75529662
chr15	77283882	77365225
chr15	77346541	77388802
chr15	77361314	77371899

chr15	77361314	77388802
chr15	77361314	77412774
chr15	77364073	77365225
chr15	77365162	77388802
chr15	78480916	78481450
chr15	78884286	78888008
chr15	79046048	79047082
chr15	79808076	79846856
chr15	79845157	79846856
chr15	79845248	79846856
chr15	82380397	82382879
chr15	83054303	83055345
chr15	83110000	83115741
chr15	84526780	84570643
chr15	84700936	84701407
chr15	85039028	85043798
chr15	89290457	89305224
chr15	89477531	89480580
chr15	90598449	90600441
chr15	90713142	90714086
chr15	90713142	90715042
chr15	90759501	90759701
chr15	90759504	90759701
chr15	90971444	90971910
chr15	92910606	92911506
chr15	96306250	96319013
chr15	98819861	98824242
chr16	10444075	10447451
chr16	11904500	11905587
chr16	11994028	11995160
chr16	11995355	11995773
chr16	12226862	12227609
chr16	1360561	1360984
chr16	14579166	14579850
chr16	14984049	14986841
chr16	15513283	15533135
chr16	15556667	15557966
chr16	1721240	1723129
chr16	1721578	1722610
chr16	18874220	18874831
chr16	19705998	19712772
chr16	19706591	19712772
chr16	20896307	20897874
chr16	21315310	21317126
chr16	216255	217825

chr16	21993299	22007696
chr16	22146396	22147642
chr16	223343	223705
chr16	22402691	22437737
chr16	236530	238113
chr16	2445940	2447369
chr16	25054818	25134290
chr16	27525112	27527263
chr16	27671625	27672456
chr16	2768504	2768941
chr16	2790876	2799535
chr16	2892099	2893732
chr16	28979664	28980058
chr16	29793402	29794613
chr16	29806676	29807422
chr16	29816669	29819936
chr16	29895101	29896389
chr16	29969479	29969965
chr16	30079458	30080069
chr16	30112975	30113219
chr16	30362278	30362777
chr16	30712315	30713534
chr16	30930321	30942405
chr16	30977214	30978000
chr16	31122021	31122604
chr16	31700661	31706999
chr16	31700685	31706999
chr16	328287	330203
chr16	3545883	3565121
chr16	3654960	3658064
chr16	3710384	3715088
chr16	3778948	3779422
chr16	402882	403093
chr16	4036780	4037044
chr16	4103006	4103598
chr16	4107473	4108483
chr16	4259010	4259243
chr16	46923743	46924206
chr16	50103051	50103742
chr16	53109954	53110316
chr16	53759597	53760342
chr16	54108774	54111889
chr16	56840302	56840713
chr16	56840304	56840711
chr16	57182845	57183427

chr16	57435786	57436253
chr16	57793732	57794352
chr16	58708182	58716111
chr16	58717142	58717949
chr16	66580066	66598840
chr16	67072701	67073518
chr16	67122939	67123363
chr16	67320774	67325140
chr16	67410974	67411845
chr16	67744144	67759732
chr16	67792484	67793421
chr16	67901413	67902182
chr16	67912775	67914549
chr16	68036650	68037370
chr16	68042943	68043181
chr16	68271883	68272351
chr16	68281872	68284194
chr16	69314617	69314906
chr16	69314617	69315064
chr16	69336379	69336843
chr16	69628981	69630031
chr16	69651902	69652215
chr16	69656188	69656546
chr16	69669981	69693003
chr16	70280364	70282049
chr16	70287445	70288592
chr16	70326384	70326631
chr16	70366070	70368168
chr16	70464598	70469609
chr16	70530033	70530414
chr16	70539455	70539871
chr16	71637876	71641040
chr16	71702600	71714587
chr16	71744210	71744657
chr16	71744225	71744657
chr16	71792066	71792592
chr16	71898606	71902325
chr16	72089535	72090121
chr16	72236768	72391215
chr16	72284576	72308188
chr16	72284576	72391215
chr16	72308055	72391215
chr16	72349045	72391215
chr16	72377728	72391215
chr16	72617182	72617553

chr16	74365060	74366037
chr16	74573957	74575133
chr16	74599388	74599908
chr16	75154131	75154892
chr16	75232426	75233128
chr16	85264948	85357643
chr16	87394812	87394993
chr16	8745591	8746488
chr16	89361494	89422305
chr16	89392379	89392679
chr16	89393613	89394390
chr16	89513272	89514746
chr16	89515789	89516473
chr16	89578667	89592103
chr16	89587716	89588100
chr16	89654606	89655856
chr16	89786610	89790141
chr16	89801810	89802213
chr17	1372136	1373418
chr17	1393708	1395296
chr17	14071807	14072315
chr17	1429884	1430126
chr17	1439378	1440072
chr17	1527051	1528877
chr17	15503097	15506986
chr17	15962177	15962638
chr17	15988981	15990433
chr17	15999387	16028337
chr17	16172278	16173040
chr17	16452711	16454114
chr17	17329255	17330024
chr17	17685411	17685657
chr17	17929994	17931541
chr17	17987089	18016260
chr17	18016060	18039050
chr17	1816712	1817119
chr17	18275674	18276914
chr17	18278900	18279366
chr17	18284312	18285024
chr17	19003307	19003567
chr17	19295467	19296201
chr17	19349670	19350285
chr17	19922822	19923187
chr17	20161118	20161889
chr17	2034288	2034427

chr17	21290997	21292613
chr17	2314675	2322871
chr17	2410554	2431055
chr17	2413763	2415954
chr17	2603507	2603835
chr17	2603518	2603835
chr17	28168470	28194708
chr17	28590791	28591383
chr17	28604275	28605215
chr17	28672658	28676968
chr17	28720772	28722882
chr17	29280088	29281063
chr17	29579514	29581041
chr17	2961450	2961938
chr17	29784320	29784532
chr17	2987445	2988132
chr17	2992430	2992935
chr17	30201154	30226115
chr17	30445871	30449680
chr17	30709658	30786537
chr17	30743300	30745851
chr17	30743300	30745946
chr17	30743300	30776420
chr17	30745713	30784892
chr17	30753760	30754213
chr17	30780591	30784892
chr17	31868882	31869627
chr17	32351528	32377980
chr17	34037509	34038505
chr17	3467473	3557800
chr17	35110070	35111495
chr17	35670026	35723814
chr17	35696659	35697687
chr17	36512962	36513164
chr17	36587773	36588352
chr17	37048653	37049308
chr17	3720876	3722221
chr17	3726583	3726839
chr17	37295916	37296371
chr17	37346624	37347418
chr17	37412705	37413009
chr17	37522942	37525572
chr17	38397500	38397936
chr17	38505285	38505652
chr17	38735491	38748308

chr17	38753197	38755929
chr17	39317998	39318473
chr17	39466521	39467073
chr17	39495702	39496006
chr17	40289548	40291323
chr17	40394625	40395031
chr17	41819942	41820584
chr17	41902489	41903901
chr17	41951952	41954235
chr17	42149973	42150479
chr17	42241262	42241552
chr17	42332799	42334362
chr17	42380685	42381464
chr17	42575053	42575744
chr17	42594773	42595075
chr17	42725133	42726599
chr17	43155800	43156009
chr17	43204215	43204530
chr17	43377912	43380107
chr17	43528234	43532914
chr17	44038577	44039161
chr17	44158613	44161068
chr17	44187616	44191284
chr17	44209651	44209954
chr17	44798390	44807502
chr17	45275390	45275871
chr17	46083699	46084545
chr17	46112476	46112956
chr17	47543044	47543989
chr17	48082834	48083564
chr17	4888123	4888792
chr17	4945920	4948373
chr17	50434593	50434965
chr17	50695853	50700651
chr17	50723856	50724305
chr17	50742047	50743243
chr17	51054729	51055076
chr17	5287521	5288477
chr17	5388861	5397536
chr17	5462232	5462826
chr17	57377197	57377789
chr17	57847254	57848462
chr17	57913107	57913404
chr17	59063449	59063915
chr17	59164786	59165115

chr17	59717258	59717638
chr17	59878226	59888329
chr17	59883665	59884399
chr17	59943777	59944893
chr17	60192213	60192544
chr17	60343956	60344238
chr17	60347657	60347842
chr17	60377093	60380399
chr17	61354361	61392121
chr17	61712449	61712722
chr17	61844159	61844391
chr17	62429535	62433816
chr17	62484575	62484767
chr17	63111244	63126463
chr17	63116268	63116575
chr17	63660873	63663216
chr17	64560045	64560700
chr17	64561802	64562018
chr17	64644163	64644470
chr17	6532028	6533072
chr17	6580108	6580424
chr17	66363157	66364589
chr17	66367966	66369943
chr17	67884336	67885281
chr17	68039239	68039966
chr17	68432215	68432835
chr17	7245214	7245608
chr17	72721374	72721742
chr17	72924414	72925830
chr17	735300	735675
chr17	7408011	7415671
chr17	7475257	7475561
chr17	74989909	74991127
chr17	75054223	75057981
chr17	75120172	75120539
chr17	75175864	75176411
chr17	75178122	75178641
chr17	75383011	75383588
chr17	7596460	7599590
chr17	76393027	76393495
chr17	76443581	76443967
chr17	76557969	76558600
chr17	76558373	76558600
chr17	76584217	76594118
chr17	76584504	76614005

chr17	7690197	7690949
chr17	78004169	78009697
chr17	78004169	78031781
chr17	78182946	78183668
chr17	78192596	78193089
chr17	78834208	78834534
chr17	79078328	79079154
chr17	80266712	80266985
chr17	80390607	80390831
chr17	81086474	81086817
chr17	81510953	81512022
chr17	81560027	81560455
chr17	81705150	81709685
chr17	81849295	81850718
chr17	81914932	81915406
chr17	81998057	81998944
chr17	8208347	8209130
chr17	8208513	8209257
chr17	82207730	82208048
chr17	82207846	82208048
chr17	82248967	82252527
chr17	82265478	82266795
chr17	82405050	82405587
chr17	82583454	82584637
chr17	82791363	82792044
chr17	83029492	83030902
chr17	8438240	8440237
chr17	9486402	9486687
chr18	12323715	12324544
chr18	12324277	12324544
chr18	12375056	12375577
chr18	1269669	1278633
chr18	1269669	1361221
chr18	13666478	13672222
chr18	20990616	20990870
chr18	21017483	21017712
chr18	21049775	21111762
chr18	21621075	21621672
chr18	21661607	21662027
chr18	23017284	23017802
chr18	24070750	24071023
chr18	26018018	26080423
chr18	26078887	26079612
chr18	26170674	26171200
chr18	3003370	3004964

chr18	36756158	36781922
chr18	36946248	36965076
chr18	36959315	37084232
chr18	45937660	45938247
chr18	46216179	46263101
chr18	47091173	47097404
chr18	49232862	49233148
chr18	5240186	5241889
chr18	54360756	54378326
chr18	56042285	56115547
chr18	56096074	56097195
chr18	57695944	57780956
chr18	57731413	57732023
chr18	57731413	57735592
chr18	57734025	57735592
chr18	57763778	57780956
chr18	59346302	59347286
chr18	62891779	62892183
chr18	63280886	63281214
chr18	68701615	68708817
chr18	68891552	68897682
chr18	692769	693175
chr18	69935731	69947532
chr18	708831	709155
chr18	74980327	74982049
chr18	7755322	7755920
chr18	79321102	79322555
chr18	79503572	79504059
chr18	8618628	8620020
chr18	8731496	8731754
chr18	9367487	9388140
chr18	9388039	9388420
chr18	9924995	9927254
chr19	1005754	1006098
chr19	10146059	10147417
chr19	10164159	10164348
chr19	10283614	10284971
chr19	1033051	1035626
chr19	10371558	10372100
chr19	10373617	10374015
chr19	10490545	10491025
chr19	10657741	10657950
chr19	10731589	10734123
chr19	10884248	10893132
chr19	11109448	11110210

chr19	11114013	11114607
chr19	11673588	11680972
chr19	12106469	12111372
chr19	12106469	12135927
chr19	12112164	12157058
chr19	12135774	12147834
chr19	12183879	12184284
chr19	12276147	12291250
chr19	1241660	1242686
chr19	12707295	12709114
chr19	12732046	12732699
chr19	12811078	12812252
chr19	12927944	12931373
chr19	12950294	12952618
chr19	13124167	13124433
chr19	13761348	13761756
chr19	13814514	13814865
chr19	13992375	13992750
chr19	13994637	13996833
chr19	1410441	1410608
chr19	14127943	14129416
chr19	14134106	14134962
chr19	14415590	14415811
chr19	14481195	14481527
chr19	16079143	16101409
chr19	16079987	16080498
chr19	16080914	16101409
chr19	16081019	16101409
chr19	16214622	16215156
chr19	16432694	16438639
chr19	16647119	16655368
chr19	16858125	16858697
chr19	17080640	17081122
chr19	17216885	17217230
chr19	17277479	17277900
chr19	17492867	17494383
chr19	1771325	1771616
chr19	17934761	17935924
chr19	17986707	17987788
chr19	18316636	18319024
chr19	18838616	18839120
chr19	18991173	18993648
chr19	19133738	19134358
chr19	19183332	19183992
chr19	19191121	19191833

chr19	19341619	19342442
chr19	19482367	19483651
chr19	19482813	19483551
chr19	1950432	1951097
chr19	21024687	21025681
chr19	2182134	2183265
chr19	2672920	2673382
chr19	2771957	2772180
chr19	2937407	2937866
chr19	29818998	29820323
chr19	3003977	3004406
chr19	3190779	3190992
chr19	3206608	3207034
chr19	32371381	32371734
chr19	32654204	32655606
chr19	32655116	32655634
chr19	32709160	32711905
chr19	32983398	32983721
chr19	32985912	32986558
chr19	33004880	33006128
chr19	33013491	33015164
chr19	33061715	33062256
chr19	33174138	33174979
chr19	33194286	33205759
chr19	33374451	33379073
chr19	33378781	33379073
chr19	33464129	33464807
chr19	3425690	3428275
chr19	34298859	34308444
chr19	34371762	34372868
chr19	35002666	35003946
chr19	3540801	3541923
chr19	3541091	3541432
chr19	3541135	3541890
chr19	3574929	3575737
chr19	35762967	35763252
chr19	35763790	35764373
chr19	36078358	36078607
chr19	36216580	36217144
chr19	36232816	36233179
chr19	36657403	36662366
chr19	3758524	3758977
chr19	3758527	3758989
chr19	37992405	37994150
chr19	38084888	38085220

chr19	38136511	38137283
chr19	38155988	38160496
chr19	38312790	38313114
chr19	38377782	38378366
chr19	38691810	38694354
chr19	38818790	38819685
chr19	38942510	38951516
chr19	39378692	39379785
chr19	3964861	3969714
chr19	3980919	3982379
chr19	3983343	3983843
chr19	40363701	40364080
chr19	40758994	40759264
chr19	40847821	40851592
chr19	4091952	4092811
chr19	41245144	41245508
chr19	41245191	41245447
chr19	41253637	41256575
chr19	41390227	41391104
chr19	41390232	41391104
chr19	41390232	41391117
chr19	41866111	41867454
chr19	42397136	42522098
chr19	42994749	43029275
chr19	43137516	43182918
chr19	43181361	43259688
chr19	43492381	43493018
chr19	43516577	43520121
chr19	43925141	43926500
chr19	44166408	44167413
chr19	44227530	44227946
chr19	44876450	44876948
chr19	44895824	44896773
chr19	45021082	45021669
chr19	45035476	45036709
chr19	45035733	45036167
chr19	45050606	45050794
chr19	45065514	45066153
chr19	45278835	45279280
chr19	45369542	45369834
chr19	4544864	4545570
chr19	45639978	45640475
chr19	45727520	45728024
chr19	45859516	45860121
chr19	45995468	46003114

chr19	4642088	4647403
chr19	46603321	46603532
chr19	46607865	46608140
chr19	46776052	46776288
chr19	46971296	46971613
chr19	46971313	46971594
chr19	47033191	47033703
chr19	47136725	47137118
chr19	47156386	47156613
chr19	47173285	47178914
chr19	47187801	47189260
chr19	47356206	47357442
chr19	47358893	47359505
chr19	48173557	48176803
chr19	4854068	4854883
chr19	48693234	48707411
chr19	48802714	48803275
chr19	48959867	48960303
chr19	4942659	4943024
chr19	49487768	49488057
chr19	49488836	49489355
chr19	4949820	4950260
chr19	4953953	4956029
chr19	49748141	49757236
chr19	49774826	49775409
chr19	49866527	49867021
chr19	50394562	50394930
chr19	50797584	50801585
chr19	50800677	50801585
chr19	50800677	50801750
chr19	51102657	51102999
chr19	51573209	51588078
chr19	51689771	51690145
chr19	52216981	52217410
chr19	52550731	52551138
chr19	52588531	52599170
chr19	52771708	52772409
chr19	53112237	53112826
chr19	53344311	53344874
chr19	53521855	53576819
chr19	53521889	53576853
chr19	5710355	5710782
chr19	57778749	57790398
chr19	57790273	57810077
chr19	57843848	57844240

chr19	57870721	57900514
chr19	57922464	57930557
chr19	6388257	6388496
chr19	653711	654297
chr19	660071	662058
chr19	7164019	7173428
chr19	7406373	7406775
chr19	7908369	7909048
chr19	8221819	8235597
chr19	8241428	8243083
chr19	8313529	8314646
chr19	8402092	8402631
chr19	8479290	8480451
chr19	8539731	8540060
chr19	8539731	8546207
chr19	9366279	9367053
chr19	9662528	9664150
chr1	10000163	10000771
chr1	100050992	100052526
chr1	100054184	100079741
chr1	10008706	10009118
chr1	100196040	100206255
chr1	10417997	10418484
chr1	10617458	10617615
chr1	108188838	108195480
chr1	108877530	108929975
chr1	109657781	109661592
chr1	11028129	11028808
chr1	110381576	110382205
chr1	110404970	110405270
chr1	112612232	112612886
chr1	112935337	112946663
chr1	112996840	113023554
chr1	113017947	113023554
chr1	113024967	113025415
chr1	113074816	113078735
chr1	113481747	113482278
chr1	113550023	113564944
chr1	113749505	113758733
chr1	114742548	114743273
chr1	115983539	116078992
chr1	117478090	117483400
chr1	119015835	119124683
chr1	119151163	119161791
chr1	1373761	1374361

chr1	1403036	1403338
chr1	145787494	145788191
chr1	145837081	145837283
chr1	1478520	1517187
chr1	149638670	149638970
chr1	150079033	150079482
chr1	150299138	150299536
chr1	150301405	150301700
chr1	150485044	150546857
chr1	150848809	150848972
chr1	150948929	150953311
chr1	151004171	151005233
chr1	151202699	151203275
chr1	151202704	151203301
chr1	151202999	151203275
chr1	151211259	151212669
chr1	151237065	151237402
chr1	151896257	151899261
chr1	152205859	152251758
chr1	152223626	152249012
chr1	153618555	153620055
chr1	153620209	153621266
chr1	153893305	153897343
chr1	153898855	153900412
chr1	153916271	153916836
chr1	153975758	153975979
chr1	154025524	154025862
chr1	154175307	154175914
chr1	154231585	154232047
chr1	154964697	154966898
chr1	15497091	15498323
chr1	154986885	154987659
chr1	155132434	155132596
chr1	155249368	155249516
chr1	155314724	155315284
chr1	155358610	155359386
chr1	155374323	155374847
chr1	155519903	155520644
chr1	155665054	155665681
chr1	155675616	155675970
chr1	155681368	155683388
chr1	155719506	155720758
chr1	155725771	155726184
chr1	155732479	155732757
chr1	155732500	155732748

chr1	155733695	155735766
chr1	155901103	155901897
chr1	155906094	155906516
chr1	155925695	155929361
chr1	156014818	156018476
chr1	156132377	156132934
chr1	156136092	156136237
chr1	1561614	1561749
chr1	156207456	156208192
chr1	156215089	156217512
chr1	156263459	156265868
chr1	156266011	156267657
chr1	156730699	156731159
chr1	156785464	156785857
chr1	156790603	156791069
chr1	15780455	15781114
chr1	158072178	158072661
chr1	15887935	15888418
chr1	161175877	161176475
chr1	161211085	161211435
chr1	16130765	16131348
chr1	16261000	16316657
chr1	16268745	16269333
chr1	16331971	16332650
chr1	16335789	16336047
chr1	165635319	165637009
chr1	165847956	165848377
chr1	165884631	165885379
chr1	165885141	165907892
chr1	166103723	166147959
chr1	166130243	166141022
chr1	166130243	166147959
chr1	1678546	1678902
chr1	168055998	168056194
chr1	169377137	169378480
chr1	17002691	17004201
chr1	171540680	171575047
chr1	171849176	171850840
chr1	173440462	173442048
chr1	174711208	174711387
chr1	174774363	174775408
chr1	1752548	1754341
chr1	175869847	175922177
chr1	176111068	176111403
chr1	178737107	178741609

chr1	179030336	179049391
chr1	179030336	179050440
chr1	179111563	179111816
chr1	179113081	179113668
chr1	179555340	179573505
chr1	179893172	179893496
chr1	180640702	180641517
chr1	1819474	1820143
chr1	183900530	183901196
chr1	184477464	184509452
chr1	186435224	186470395
chr1	1868741	1869944
chr1	1868780	1869894
chr1	187254375	187255868
chr1	187254375	187269508
chr1	187326920	187443726
chr1	1877069	1877600
chr1	18942362	18943433
chr1	19158747	19158910
chr1	192925611	192957422
chr1	193049016	193049467
chr1	193299207	193315390
chr1	19473786	19474115
chr1	197138758	197139101
chr1	19898183	19898457
chr1	200370913	200374246
chr1	200560538	200560987
chr1	201995669	201995945
chr1	202598469	202604699
chr1	202825175	202835197
chr1	202886489	202886860
chr1	206410008	206410955
chr1	206591721	206612357
chr1	20744800	20776732
chr1	20774585	20776005
chr1	20775723	20776005
chr1	20965671	21059794
chr1	21081874	21082380
chr1	21105352	21107285
chr1	21241728	21250736
chr1	214284648	214286916
chr1	21919441	21919931
chr1	21924381	21924983
chr1	219970120	219970531
chr1	220761655	220784071

chr1	222688829	222689244
chr1	222823883	222835215
chr1	222827427	222835215
chr1	222830928	222837325
chr1	224229076	224229696
chr1	224424597	224433837
chr1	225851667	225852237
chr1	226253245	226253981
chr1	229295019	229304000
chr1	229550828	229553192
chr1	230266101	230269280
chr1	230269467	230269918
chr1	233933369	233938564
chr1	233933369	233945881
chr1	234139092	234141171
chr1	23520446	23520741
chr1	235437036	235442102
chr1	235606348	235607020
chr1	235612123	235612503
chr1	235612123	235612506
chr1	235621906	235622674
chr1	236400668	236401067
chr1	236600425	236602654
chr1	23766365	23767413
chr1	2389034	2390858
chr1	2393072	2403589
chr1	2393269	2403589
chr1	243348338	243348674
chr1	24370789	24371340
chr1	244659478	244672502
chr1	244845246	244848856
chr1	245853463	245885755
chr1	245959914	245960630
chr1	246143821	246193963
chr1	246186742	246186942
chr1	246186742	246193963
chr1	246186742	246195464
chr1	246186742	246199767
chr1	246186742	246207459
chr1	246186742	246219976
chr1	246199688	246207505
chr1	246881760	246882107
chr1	246929974	246930654
chr1	247155565	247159867
chr1	247156405	247159867

chr1	247156999	247159795
chr1	248840171	248841840
chr1	25306895	25318271
chr1	25315937	25318271
chr1	25867026	25868866
chr1	26440047	26441019
chr1	26711371	26711815
chr1	26735580	26736030
chr1	26737772	26738118
chr1	26763187	26779779
chr1	26884124	26886986
chr1	27109543	27109798
chr1	27486846	27487210
chr1	27578555	27578778
chr1	28011305	28011711
chr1	28202012	28202389
chr1	28329576	28329955
chr1	28372167	28372574
chr1	28372984	28394505
chr1	28397994	28398535
chr1	28430288	28430896
chr1	28487273	28487831
chr1	28519983	28520959
chr1	28568086	28570329
chr1	28572842	28575961
chr1	28639313	28639972
chr1	28752952	28753801
chr1	28890322	28891801
chr1	29148079	29168638
chr1	29149008	29168638
chr1	29166728	29168638
chr1	29173727	29173957
chr1	30948145	30948506
chr1	31045096	31046139
chr1	31045409	31045843
chr1	32017165	32017702
chr1	32040345	32041640
chr1	32056181	32056670
chr1	32164979	32165920
chr1	32581790	32582667
chr1	32674937	32679078
chr1	32798170	32798589
chr1	32814620	32815155
chr1	32821224	32821536
chr1	34856353	34856943

chr1	35087508	35087982
chr1	35090171	35090666
chr1	35110338	35113181
chr1	36170963	36173480
chr1	36268087	36268820
chr1	36275197	36277854
chr1	36287476	36287871
chr1	37537671	37542270
chr1	37693629	37693919
chr1	37805167	37806295
chr1	37933642	37934043
chr1	38868813	38869183
chr1	38993711	38994925
chr1	39030191	39030491
chr1	39119904	39122396
chr1	39161476	39166162
chr1	39198732	39199080
chr1	39214079	39215429
chr1	39340501	39340953
chr1	40088444	40089021
chr1	40450655	40462597
chr1	40695174	40695470
chr1	41142873	41162951
chr1	41846382	41849035
chr1	41864577	41869630
chr1	43588795	43591453
chr1	44701178	44707389
chr1	44703437	44707389
chr1	44749324	44751309
chr1	44763940	44764604
chr1	44859580	44859866
chr1	44866320	44866664
chr1	45689163	45689588
chr1	45810718	45810947
chr1	46312455	46313277
chr1	46587108	46587570
chr1	47291283	47291685
chr1	47335544	47335858
chr1	51236733	51271473
chr1	51251224	51251558
chr1	51919926	51941834
chr1	51922927	51923522
chr1	52325394	52346917
chr1	52338927	52346917
chr1	52376437	52377132

chr1	52378586	52382689
chr1	52404680	52416743
chr1	52404680	52418104
chr1	52913375	52914095
chr1	52913894	52914095
chr1	53302011	53302440
chr1	53834076	53834847
chr1	58576217	58718748
chr1	58591300	58718748
chr1	58637031	58715641
chr1	61151067	61152356
chr1	6120224	6120543
chr1	61917987	61918363
chr1	6193728	6194791
chr1	6227856	6228374
chr1	62455181	62492825
chr1	63478764	63494287
chr1	63499449	63500130
chr1	67387626	67388455
chr1	67395404	67402417
chr1	70163378	70164145
chr1	70163379	70164137
chr1	70338021	70338519
chr1	77633114	77675689
chr1	7777158	7778165
chr1	7978308	7979910
chr1	85019152	85025779
chr1	8592903	8593175
chr1	8865314	8866394
chr1	8872654	8874334
chr1	88859132	88859592
chr1	88859159	88859642
chr1	92479166	92479587
chr1	93154655	93162569
chr1	93184022	93202048
chr1	93620467	93621445
chr1	93622203	93624958
chr1	93633000	93661569
chr1	93648040	93650141
chr1	98014923	98019979
chr1	9893008	9894243
chr1	99429459	99492969
chr1	99668622	99671404
chr1	99872325	99872514
chr20	13528445	13537049

chr20	13551886	13600617
chr20	13806444	13807091
chr20	14019557	14020182
chr20	1772413	1776829
chr20	17975464	17976218
chr20	17985330	17986163
chr20	19997666	19998073
chr20	19998109	19998465
chr20	20018495	20019023
chr20	21207970	21208449
chr20	24959156	24962857
chr20	25558057	25558317
chr20	25850572	25851795
chr20	2663530	2665504
chr20	2927917	2928230
chr20	31556926	31557802
chr20	31743121	31744257
chr20	33667116	33668885
chr20	34002990	34003560
chr20	34023799	34024010
chr20	34286027	34287500
chr20	34471397	34507908
chr20	34867202	34867481
chr20	34920614	34927361
chr20	35272522	35273161
chr20	35409451	35409887
chr20	35713560	35713800
chr20	35718735	35719892
chr20	35719599	35719892
chr20	35727252	35727606
chr20	35728035	35737602
chr20	35800093	35812719
chr20	35815346	35815566
chr20	3626139	3626881
chr20	36473241	36474009
chr20	36517569	36517993
chr20	36583804	36584641
chr20	36587973	36590056
chr20	36657454	36658213
chr20	36781458	36781947
chr20	37057907	37058654
chr20	37064045	37064550
chr20	37307182	37307537
chr20	3761641	3763062
chr20	37696673	37696945

chr20	37751485	37751666
chr20	38070224	38070511
chr20	38155028	38155448
chr20	38420532	38422368
chr20	39035349	39036439
chr20	3933863	3939770
chr20	43512888	43518703
chr20	43675543	43676149
chr20	45838054	45838477
chr20	45885843	45888127
chr20	45895795	45895981
chr20	47214757	47216262
chr20	48998250	48998532
chr20	49041529	49041890
chr20	49041529	49042685
chr20	49042248	49042713
chr20	49250213	49251215
chr20	49518824	49519352
chr20	50091496	50093923
chr20	50938901	50939723
chr20	51019132	51021018
chr20	51349940	51396876
chr20	5164026	5168187
chr20	56378437	56379098
chr20	58393980	58399040
chr20	58999342	58999809
chr20	59000359	59000911
chr20	62276203	62276962
chr20	63496081	63497371
chr20	63498282	63498716
chr20	6355479	6448711
chr20	63594023	63594830
chr20	63672082	63672549
chr20	63736411	63736816
chr20	63767398	63768118
chr20	63804814	63805030
chr20	63804814	63806718
chr20	63817638	63820545
chr20	63874340	63874890
chr20	63898776	63899637
chr20	63905258	63906959
chr20	63910450	63910767
chr20	63993998	63994526
chr20	6436014	6477363
chr21	22032384	22043087

chr21	22032384	22051350
chr21	25605762	25606173
chr21	25991608	25992189
chr21	29552522	29559910
chr21	29577029	29577891
chr21	31461223	31477344
chr21	32339304	32340342
chr21	32481762	32482225
chr21	33421533	33432796
chr21	34102834	34142707
chr21	34102834	34165036
chr21	34129118	34129462
chr21	36220969	36221318
chr21	36373558	36374145
chr21	36478388	36480375
chr21	36969857	36973777
chr21	37375050	37375837
chr21	37415489	37421866
chr21	37415489	37425781
chr21	37415489	37430400
chr21	38893960	38991024
chr21	39395641	39396253
chr21	42901349	42904885
chr21	42978282	42980096
chr21	42978289	42980091
chr21	42979124	42979442
chr21	42979370	43031617
chr21	43529757	43595254
chr21	45080997	45144701
chr21	45116932	45144701
chr22	17595276	17595631
chr22	17656175	17656401
chr22	17705180	17705392
chr22	19328397	19331682
chr22	19418554	19421781
chr22	20485649	20487466
chr22	20533780	20534261
chr22	20710214	20710540
chr22	20744307	20744573
chr22	20758326	20759653
chr22	21008778	21013184
chr22	21600603	21608989
chr22	21619922	21620100
chr22	21770502	21770906
chr22	21815333	21815771

chr22	23574214	23639569
chr22	23814100	23814695
chr22	24567653	24568436
chr22	24568953	24571486
chr22	24570590	24570913
chr22	25180078	25181016
chr22	26711829	26726794
chr22	26726643	26738240
chr22	27873681	27892330
chr22	27930693	27966547
chr22	28383019	28386469
chr22	28499431	28519320
chr22	28514565	28517279
chr22	28514565	28519320
chr22	28514756	28515716
chr22	28514756	28517279
chr22	28514756	28519320
chr22	28514756	28528728
chr22	28514756	28545577
chr22	28514756	28550151
chr22	28514756	28583669
chr22	28515626	28517279
chr22	28515626	28519320
chr22	28547673	28550151
chr22	28996203	28997730
chr22	29341157	29341450
chr22	29520789	29526272
chr22	29606237	29606764
chr22	30243739	30246797
chr22	30926724	30968296
chr22	31334946	31335249
chr22	31786569	31786869
chr22	31880922	31889635
chr22	31888794	31890464
chr22	32232605	32232962
chr22	33847656	33880733
chr22	36520407	36520981
chr22	37612696	37613446
chr22	37615671	37615935
chr22	37678057	37678854
chr22	37881672	37882024
chr22	37939033	37939499
chr22	38212519	38212838
chr22	38252332	38252904
chr22	38256162	38256461

chr22	38267726	38268122
chr22	38267738	38268131
chr22	38687299	38688344
chr22	38791089	38791463
chr22	39016494	39016952
chr22	39314109	39316807
chr22	39403827	39404132
chr22	39403830	39404132
chr22	39482235	39483234
chr22	40278444	40278834
chr22	40353908	40354588
chr22	40788053	40788451
chr22	40954241	40954812
chr22	40956942	40957369
chr22	40963117	40963359
chr22	41276620	41278118
chr22	41474879	41475610
chr22	41530913	41531754
chr22	41536903	41539122
chr22	41849462	41849865
chr22	42001099	42030823
chr22	42935754	42935942
chr22	44921971	44922363
chr22	44977487	44978469
chr22	46269906	46270270
chr22	46310827	46311172
chr22	46850465	46929635
chr22	46974291	46978934
chr22	47667764	47670405
chr22	47747153	47747591
chr22	49805973	49806804
chr22	50409074	50409471
chr22	50410874	50411377
chr22	50728363	50728647
chr2	101009391	101009838
chr2	101703386	101703547
chr2	101727954	101728528
chr2	1055941	1056173
chr2	108500471	108501077
chr2	109191905	109193075
chr2	109263130	109263685
chr2	111344060	111480061
chr2	112545664	112546015
chr2	120019104	120175004
chr2	120127717	120175004

chr2	120278614	120280907
chr2	120840579	120841536
chr2	121582156	121582823
chr2	121601567	121601922
chr2	121759515	121760964
chr2	127263992	127264232
chr2	127520438	127521021
chr2	127794800	127795182
chr2	130156908	130157114
chr2	131062980	131089336
chr2	131067090	131089336
chr2	131483963	131486181
chr2	131684708	131733952
chr2	135529637	135530132
chr2	135565736	135568809
chr2	148051909	148054449
chr2	151465853	151475219
chr2	151465853	151483335
chr2	151506209	151507845
chr2	151970280	151971704
chr2	152075254	152087428
chr2	152085567	152086011
chr2	152414578	152450409
chr2	152633351	152634178
chr2	157857464	157860179
chr2	158546297	158588987
chr2	158994901	159011440
chr2	159240183	159241115
chr2	168046288	168048322
chr2	168123791	168124519
chr2	170752526	170753006
chr2	171016000	171016251
chr2	171052163	171053489
chr2	173096623	173097101
chr2	174139904	174140958
chr2	174452927	174453384
chr2	177214730	177215110
chr2	177231888	177234237
chr2	177245908	177247401
chr2	178210734	178212889
chr2	178216770	178230513
chr2	178434394	178434839
chr2	178539949	178644501
chr2	178636573	178695451
chr2	179826474	179941285

chr2	185757579	185788943
chr2	190886126	190886661
chr2	196856323	196859055
chr2	197301233	197303448
chr2	197559625	197590606
chr2	199309461	199312663
chr2	200460658	200460907
chr2	200573807	200583559
chr2	200938301	200938801
chr2	201277978	201278809
chr2	202292224	202292404
chr2	202382857	202386960
chr2	202403038	202403792
chr2	20267296	20268380
chr2	202914686	202915111
chr2	203351554	203383321
chr2	203416913	203427269
chr2	207719578	207719911
chr2	207746883	207747643
chr2	210019143	210019623
chr2	210041686	210043132
chr2	210150763	210151097
chr2	211908742	211968374
chr2	216120966	216123569
chr2	216302512	216302666
chr2	218239675	218243698
chr2	218635682	218636642
chr2	218635704	218636642
chr2	218715900	218716514
chr2	218735460	218736101
chr2	223878528	223899021
chr2	224556294	224558241
chr2	232701710	232702153
chr2	232723641	232730391
chr2	232922245	232922597
chr2	233267256	233271373
chr2	233359516	233372183
chr2	233372044	233376936
chr2	233403490	233403695
chr2	234974936	234975195
chr2	235524414	235524713
chr2	235585724	235590023
chr2	235585724	235595045
chr2	236259506	236268343
chr2	237676867	237684755

chr2	237682624	237683495
chr2	237769625	237779457
chr2	23792349	23792716
chr2	238061758	238073847
chr2	238179105	238179916
chr2	238433312	238446655
chr2	238433548	238446655
chr2	239377050	239379053
chr2	23989391	23989819
chr2	241502734	241504942
chr2	24564294	24593721
chr2	24595670	24596113
chr2	24823955	24824639
chr2	26069573	26069800
chr2	26257385	26257748
chr2	27034872	27035042
chr2	27052332	27070212
chr2	27229313	27229547
chr2	27404925	27405155
chr2	27656082	27656762
chr2	28757097	28758305
chr2	29120029	29174525
chr2	31073062	31085023
chr2	31073062	31132712
chr2	32002665	32002933
chr2	32295813	32296258
chr2	32302292	32303078
chr2	3243995	3244297
chr2	3243995	3261827
chr2	32494503	32494770
chr2	3456946	3485844
chr2	36358903	36360192
chr2	36369107	36370407
chr2	36378356	36413019
chr2	36387971	36413019
chr2	36463062	36463203
chr2	36517331	36522274
chr2	37091434	37104132
chr2	37306495	37317179
chr2	38121984	38147998
chr2	38339197	38339772
chr2	39736821	39769758
chr2	40322550	40430301
chr2	40347906	40348903
chr2	42278843	42279552

chr2	42413698	42456361
chr2	42571869	42574216
chr2	44174761	44180397
chr2	44490785	44505730
chr2	44490785	44549728
chr2	44502688	44505730
chr2	44505632	44537721
chr2	44505632	44549728
chr2	44549554	44558013
chr2	45677831	45701491
chr2	45697940	45701491
chr2	45697940	45701524
chr2	45840379	45860198
chr2	46971029	46976387
chr2	47792224	47793093
chr2	48350091	48350640
chr2	53966969	53967411
chr2	54785196	54788051
chr2	55261275	55261678
chr2	55843992	55873968
chr2	61157861	61164540
chr2	61178980	61186754
chr2	61178980	61186781
chr2	61178980	61187029
chr2	61197271	61197791
chr2	61294268	61294528
chr2	61479374	61479675
chr2	61516341	61516712
chr2	63180166	63181021
chr2	64555496	64556038
chr2	65093925	65095885
chr2	65237347	65238042
chr2	70246234	70246609
chr2	7068215	7075684
chr2	72680613	72721164
chr2	73985979	74010653
chr2	74156417	74172749
chr2	77939830	77951533
chr2	77939830	78002142
chr2	77987569	78002142
chr2	78076535	78076954
chr2	78199604	78203781
chr2	78199604	78203818
chr2	79799311	79800671
chr2	84850010	84853410

chr2	86111495	86112151
chr2	9237832	9239953
chr2	9395382	9395702
chr2	9410142	9411459
chr2	9410807	9411533
chr2	95148884	95159373
chr2	9581670	9616809
chr2	96015108	96023245
chr2	96236683	96242141
chr2	96349637	96351414
chr2	98462383	98462899
chr2	99466465	99476362
chr3	10046285	10065020
chr3	10240386	10240618
chr3	111112284	111134205
chr3	112567133	112567388
chr3	11399283	11399625
chr3	11426879	11574118
chr3	11553105	11553270
chr3	11573921	11574118
chr3	121430388	121433036
chr3	121525263	121533986
chr3	121533791	121535484
chr3	123243811	123264771
chr3	125313326	125323387
chr3	125467932	125479777
chr3	125514811	125515568
chr3	125848229	125848447
chr3	125848229	125857101
chr3	126741359	126741905
chr3	127013397	127013756
chr3	128044086	128091582
chr3	128303310	128305030
chr3	128906426	128906728
chr3	129756695	129757573
chr3	129838897	129880562
chr3	129857249	129880562
chr3	129857249	129889104
chr3	129860939	129880562
chr3	130679326	130716498
chr3	130918899	130919319
chr3	135997500	136018737
chr3	136315603	136316551
chr3	136709391	136715592
chr3	136709391	136720173

chr3	136713457	136720173
chr3	136832291	136832792
chr3	138069010	138070596
chr3	138498652	138499662
chr3	138542690	138572984
chr3	142486884	142487399
chr3	151795738	151803925
chr3	151803705	151803925
chr3	155846753	155847480
chr3	155890667	155937881
chr3	15721765	15722310
chr3	158218689	158219715
chr3	165503224	165519587
chr3	168044036	168044407
chr3	168057080	168057613
chr3	168075572	168076442
chr3	169821890	169837653
chr3	17004027	17025126
chr3	170062432	170062992
chr3	170077320	170080646
chr3	170097171	170113456
chr3	170269865	170274226
chr3	171787426	171789535
chr3	172079353	172081715
chr3	17562018	17586557
chr3	17562018	17733460
chr3	17586352	17625224
chr3	17586352	17647056
chr3	17622647	17625224
chr3	17622647	17672705
chr3	17622647	17681616
chr3	17622647	17733460
chr3	177152010	177152367
chr3	178841544	178982465
chr3	179253007	179253574
chr3	179566889	179567147
chr3	182894792	182895982
chr3	183015418	183022557
chr3	184303706	184305926
chr3	184305396	184307045
chr3	184318543	184318794
chr3	184363973	184364275
chr3	185473191	185490201
chr3	185590477	185597403
chr3	186942347	186996673

chr3	189207109	189220027
chr3	192583187	192728554
chr3	192664074	192745356
chr3	192880501	192880750
chr3	195245088	195245270
chr3	195247999	195248401
chr3	195248036	195248401
chr3	195357690	195412916
chr3	195365481	195412916
chr3	195410706	195412916
chr3	195437731	195438135
chr3	195438188	195438622
chr3	195890673	195891205
chr3	195959182	195960423
chr3	196061801	196062273
chr3	196078623	196078856
chr3	196213715	196218759
chr3	196245384	196245731
chr3	196321303	196322714
chr3	196330102	196332075
chr3	196356357	196397216
chr3	196714672	196714862
chr3	196916234	196916622
chr3	197613224	197624720
chr3	23259618	23310727
chr3	23309839	23310727
chr3	23310160	23310727
chr3	23898003	23898399
chr3	27381867	27382359
chr3	28251711	28253765
chr3	28251711	28267654
chr3	29288282	29334644
chr3	29467130	29467606
chr3	32334790	32391383
chr3	32565861	32566527
chr3	32770557	32772083
chr3	33298603	33299136
chr3	33388853	33412798
chr3	33699800	33701200
chr3	37010833	37011224
chr3	38216672	38220507
chr3	39076170	39084727
chr3	40309870	40312169
chr3	42184752	42223184
chr3	43531811	43535385

chr3	44578289	44578740
chr3	45130412	45131981
chr3	45519398	45519742
chr3	47052738	47052999
chr3	47174039	47175828
chr3	47179427	47179683
chr3	47414222	47414925
chr3	47506992	47507535
chr3	47507283	47508874
chr3	47696794	47697769
chr3	47897376	47897743
chr3	48441097	48443456
chr3	48698564	48699345
chr3	49040410	49041553
chr3	49395125	49404987
chr3	49781349	49785256
chr3	50043965	50044498
chr3	50051563	50052146
chr3	50073353	50073518
chr3	51490154	51492513
chr3	51541497	51552063
chr3	51541497	51573326
chr3	51541588	51552063
chr3	5192269	5216410
chr3	52255246	52260059
chr3	52467231	52467856
chr3	52579017	52579255
chr3	52581482	52581719
chr3	52637868	52638468
chr3	53018085	53043254
chr3	53252062	53252949
chr3	56570090	56570499
chr3	57909424	57909694
chr3	60215076	60233983
chr3	61584737	61623555
chr3	61617659	61623140
chr3	61617659	61623555
chr3	61621969	61623140
chr3	61621969	61623555
chr3	61622462	61623140
chr3	61622462	61623555
chr3	61981707	62015932
chr3	65609743	65609966
chr3	65609743	65659051
chr3	65717301	65718614

chr3	65717301	65725748
chr3	69794386	69805435
chr3	71287702	71287990
chr3	71697323	71699907
chr3	75711186	75784885
chr3	78932470	78965428
chr3	8703025	8840297
chr3	88084603	88086402
chr3	9418732	9418882
chr3	9421322	9421592
chr3	97877896	97943570
chr3	97956793	97957238
chr3	9987814	9998899
chr4	10085992	10087508
chr4	102304337	102315742
chr4	102779587	102787190
chr4	103343091	103437842
chr4	103343091	103453812
chr4	103536345	103593429
chr4	103547853	103593429
chr4	103548742	103593429
chr4	103569300	103593429
chr4	105195137	105261320
chr4	108594669	108598695
chr4	110190913	110191766
chr4	112180673	112186836
chr4	112808902	112819813
chr4	113607973	113679496
chr4	118056661	118136735
chr4	118251568	118259075
chr4	118299577	118300140
chr4	118724590	118728053
chr4	119488293	119488717
chr4	121825847	121847094
chr4	122838973	122892296
chr4	123071387	123075393
chr4	124457180	124642014
chr4	124641877	124669791
chr4	128017357	128021238
chr4	129724509	129782025
chr4	13425109	13426354
chr4	13425109	13428299
chr4	139021405	139021964
chr4	142654586	142729497
chr4	143859015	143939920

chr4	143865970	143982026
chr4	143974446	144076453
chr4	145125948	145132087
chr4	147963647	147992009
chr4	148384591	148384916
chr4	151586760	151662628
chr4	15684849	15687148
chr4	169212854	169217154
chr4	169240477	169240999
chr4	169694746	169700352
chr4	1710184	1710809
chr4	1718511	1719215
chr4	173039859	173056657
chr4	173224113	173226809
chr4	173224309	173230033
chr4	173384650	173403950
chr4	17617814	17618611
chr4	17815804	17816855
chr4	182874569	182916539
chr4	183966370	183966640
chr4	185181636	185182828
chr4	185314632	185315529
chr4	186592000	186592905
chr4	20280268	20280877
chr4	20617025	20617590
chr4	20700670	20727397
chr4	22727390	22727772
chr4	24583325	24583644
chr4	2474285	2488905
chr4	2488370	2488905
chr4	253099	254837
chr4	25318635	25319106
chr4	2625261	2648225
chr4	2642211	2642827
chr4	351030	352104
chr4	38680957	38682153
chr4	39185990	39186337
chr4	39528942	39529145
chr4	39530446	39532291
chr4	39750994	39751412
chr4	39750994	39751420
chr4	39759544	39759926
chr4	39829874	39830541
chr4	39864101	39865613
chr4	40971047	41002112

chr4	40978403	40986415
chr4	41004759	41005200
chr4	42039693	42040569
chr4	4275926	4276663
chr4	430343	437511
chr4	4308658	4308861
chr4	435769	437511
chr4	435769	438632
chr4	44293403	44311727
chr4	467776	468821
chr4	468754	497286
chr4	4760545	4763048
chr4	48151785	48152178
chr4	48645947	48710637
chr4	48672989	48687160
chr4	51860542	51914078
chr4	51863444	51887244
chr4	53015464	53018527
chr4	56496896	56497314
chr4	56504266	56507370
chr4	5865242	5870712
chr4	67619029	67624165
chr4	67728539	67745973
chr4	67728539	67768696
chr4	67745826	67790511
chr4	70829043	70829395
chr4	70933978	70934296
chr4	73217536	73235090
chr4	74330197	74374839
chr4	75516782	75527598
chr4	7932157	7933310
chr4	80287336	80376917
chr4	80295558	80336902
chr4	80301282	80420400
chr4	80575886	80648987
chr4	82871761	82872299
chr4	84879405	84898597
chr4	88419301	88420428
chr4	88419573	88437762
chr4	920280	920625
chr4	924107	928167
chr4	98583518	98586087
chr4	98915786	98915997
chr4	98922517	98922935
chr5	100536548	100586336

chr5	10213490	10227663
chr5	10681378	10682305
chr5	108341926	108378726
chr5	108980624	108985487
chr5	109789000	109866994
chr5	11118430	11121808
chr5	115282543	115283022
chr5	115539664	115542901
chr5	115852978	115913513
chr5	116317516	116399622
chr5	116317516	116412760
chr5	116379424	116552127
chr5	116446163	116471255
chr5	120639290	120676919
chr5	122829052	122829249
chr5	126585861	126586844
chr5	126793647	126794160
chr5	126803121	126803296
chr5	126808730	126836300
chr5	128064736	128077093
chr5	131653520	131653823
chr5	132365026	132365890
chr5	132920343	132920876
chr5	132961037	132961442
chr5	133085025	133085432
chr5	133096171	133098602
chr5	134166497	134167632
chr5	134317389	134317988
chr5	134379553	134380010
chr5	134668661	134669527
chr5	134685896	134686329
chr5	134767551	134770598
chr5	134772166	134772837
chr5	134799645	134800042
chr5	134942480	134960698
chr5	137757866	137859214
chr5	137757866	137861931
chr5	137999241	138000302
chr5	138030544	138030773
chr5	138296505	138299431
chr5	139061636	139104698
chr5	139281430	139285530
chr5	139288794	139289268
chr5	139290286	139290590
chr5	139417112	139417683

chr5	139598840	139600075
chr5	140210805	140211646
chr5	140245929	140300557
chr5	140251546	140263281
chr5	140302283	140303033
chr5	140665581	140666114
chr5	140925895	140928738
chr5	140925895	140931106
chr5	141410978	141411458
chr5	141593748	141594268
chr5	141594126	141594773
chr5	141936713	141937491
chr5	14219408	14222930
chr5	14219408	14242944
chr5	142390920	142467831
chr5	14269938	14283798
chr5	142789429	142792773
chr5	14286145	14286398
chr5	146237918	146238273
chr5	14809713	14810077
chr5	148634865	148677235
chr5	149371499	149393912
chr5	149541534	149545831
chr5	150364956	150365889
chr5	150367868	150368846
chr5	150521305	150553328
chr5	150594047	150626886
chr5	150614557	150626886
chr5	150622474	150633928
chr5	150623034	150626886
chr5	150626662	150633928
chr5	151033048	151058030
chr5	151795236	151796312
chr5	151853271	151889465
chr5	151857705	151906708
chr5	151946638	152055452
chr5	152094539	152095575
chr5	154779768	154780812
chr5	154823198	154824092
chr5	15545854	15547424
chr5	15628257	15634464
chr5	157505716	157507116
chr5	157731514	157734618
chr5	157733541	157733765
chr5	157745724	157758701

chr5	157830237	157830626
chr5	157841488	157841730
chr5	160018888	160065766
chr5	160023163	160025122
chr5	160063541	160066612
chr5	160063541	160066871
chr5	160077977	160089610
chr5	160089505	160108614
chr5	160096760	160097227
chr5	160257397	160257956
chr5	1611668	1612072
chr5	16668431	16670752
chr5	16916054	16917042
chr5	171028871	171034980
chr5	171394451	171394902
chr5	171396216	171396668
chr5	171987706	171988631
chr5	171995806	171997051
chr5	172123968	172124173
chr5	172184954	172186013
chr5	172185566	172186008
chr5	172254401	172255817
chr5	172259407	172259810
chr5	172270650	172271169
chr5	172274432	172274795
chr5	172864148	172864303
chr5	172960365	172963110
chr5	175765155	175940995
chr5	176308202	176308611
chr5	176494795	176496233
chr5	176972697	176972997
chr5	177164850	177165266
chr5	177167023	177167260
chr5	177460390	177467003
chr5	178885613	178931326
chr5	179582376	179582674
chr5	179600946	179608675
chr5	179621782	179622010
chr5	179831569	179831878
chr5	179839173	179840054
chr5	179875815	179876452
chr5	179957412	179957909
chr5	179969484	179970326
chr5	179999265	179999503
chr5	180199823	180201334

chr5	180494694	180574269
chr5	180495651	180529414
chr5	180513649	180514105
chr5	180550023	180576684
chr5	180851380	180856249
chr5	181237641	181239566
chr5	27472382	27525163
chr5	31474661	31475070
chr5	32438567	32440387
chr5	34048649	34049185
chr5	34051881	34064715
chr5	34064262	34064715
chr5	34658995	34757614
chr5	34733115	34750581
chr5	34788183	34788688
chr5	37061586	37061936
chr5	37533137	37534043
chr5	43171459	43171805
chr5	50399106	50411312
chr5	60834300	60834554
chr5	60962740	60962980
chr5	61417077	61418325
chr5	614469	614681
chr5	614469	655584
chr5	62517105	62517497
chr5	65197192	65198360
chr5	65317997	65319953
chr5	65435903	65436604
chr5	69282889	69286086
chr5	69561033	69562042
chr5	71461100	71461494
chr5	72283504	72320526
chr5	77098654	77100391
chr5	78370619	78374339
chr5	78459265	78475706
chr5	78625024	78626891
chr5	80186447	80186884
chr5	80410832	80413190
chr5	80448221	80451607
chr5	80638812	80640027
chr5	80657338	80658153
chr5	81981308	82129769
chr5	84009343	84012230
chr5	88220589	88231786
chr5	88220589	88250324

chr5	88305931	88313930
chr5	93682949	93683210
chr5	95782717	95783216
chr5	97581813	97670684
chr5	98884345	98885153
chr6	107324555	107359828
chr6	10757898	10759013
chr6	107946201	107946760
chr6	107946434	107946810
chr6	107955464	107955692
chr6	108235884	108236411
chr6	108298496	108299024
chr6	108319616	108332999
chr6	108567171	108569742
chr6	109050172	109051618
chr6	109451469	109452925
chr6	11144493	11207574
chr6	111469195	111474479
chr6	111486299	111497979
chr6	118894026	118895339
chr6	12077492	12078049
chr6	126008656	126009334
chr6	126013043	126038925
chr6	126014803	126021123
chr6	126324663	126435320
chr6	128409293	128411334
chr6	129463399	129502791
chr6	129494067	129558166
chr6	129494181	129558166
chr6	129502604	129528738
chr6	129502604	129558166
chr6	130287819	130288395
chr6	130288226	130334845
chr6	130288226	130341122
chr6	130311290	130334845
chr6	130311290	130341122
chr6	131030966	131031817
chr6	131031692	131033905
chr6	131869441	131877142
chr6	136047308	136094247
chr6	142820032	142823253
chr6	14284194	14292214
chr6	142901663	142926022
chr6	142917195	142918075
chr6	142949512	142983812

chr6	143555164	143558644
chr6	144578125	144578416
chr6	149643989	149644349
chr6	149670107	149672304
chr6	149734100	149734547
chr6	149759765	149760573
chr6	149803988	149804608
chr6	149826029	149828631
chr6	151331792	151332131
chr6	151412672	151418603
chr6	15306593	15306987
chr6	156799659	156806004
chr6	156979804	156980222
chr6	157584519	157608402
chr6	158570673	158571706
chr6	16391084	16392605
chr6	1675210	1675608
chr6	17398772	17400596
chr6	17655684	17656011
chr6	17671056	17671794
chr6	17671097	17674508
chr6	2283498	2283763
chr6	2283498	2306526
chr6	24705725	24714354
chr6	24714872	24715633
chr6	26467192	26467434
chr6	29001182	29007522
chr6	29889112	29927834
chr6	30629325	30630236
chr6	30637605	30638402
chr6	30733280	30733547
chr6	31759200	31759349
chr6	33266919	33267307
chr6	33272898	33275406
chr6	33302620	33303200
chr6	33302906	33303205
chr6	33307223	33307496
chr6	33309819	33310754
chr6	34240770	34245641
chr6	34638194	34638453
chr6	35659040	35659329
chr6	35672591	35679074
chr6	35672591	35680422
chr6	35855224	35855844
chr6	35867733	35868082

chr6	36682407	36682687
chr6	36740802	36764250
chr6	36970786	36971068
chr6	36974060	36974899
chr6	36977369	36977534
chr6	37862977	37939219
chr6	41134556	41140955
chr6	41840892	41874239
chr6	4189045	4190672
chr6	42051873	42052416
chr6	42863332	42863757
chr6	42934128	42934881
chr6	42964293	42977826
chr6	42964532	42964780
chr6	42977691	42977953
chr6	42994219	42999986
chr6	42997794	42998637
chr6	42998970	42999991
chr6	42998970	43002214
chr6	43002451	43003099
chr6	43028259	43029681
chr6	43038747	43040312
chr6	43084424	43086557
chr6	43194596	43194779
chr6	43479877	43480609
chr6	43536663	43537093
chr6	43588001	43588486
chr6	43598542	43599089
chr6	44121861	44122192
chr6	44406842	44407382
chr6	52264512	52266117
chr6	52274452	52274591
chr6	52274650	52275012
chr6	52429968	52430374
chr6	52560166	52561392
chr6	5263984	5264642
chr6	52665273	52666878
chr6	52665273	52667493
chr6	53285909	53286315
chr6	53293679	53293978
chr6	53538677	53539357
chr6	5475389	5475561
chr6	56509665	56511373
chr6	56540590	56540782
chr6	56833032	56834338

chr6	57050571	57050975
chr6	63740741	63752076
chr6	63990764	63992171
chr6	70435382	70435907
chr6	72647931	72648904
chr6	72647931	72738372
chr6	72807744	72808701
chr6	72880876	72897142
chr6	72880876	72898206
chr6	73223698	73225421
chr6	73462636	73463118
chr6	75239068	75239888
chr6	75272463	75272955
chr6	75272463	75279189
chr6	75621926	75622858
chr6	75695098	75695523
chr6	80017432	80018009
chr6	83039872	83040189
chr6	83194530	83195739
chr6	85519835	85520439
chr6	8607007	8654784
chr6	87240259	87240517
chr6	87375479	87399583
chr6	87526439	87526780
chr6	88861609	88862514
chr6	89197176	89198222
chr6	89368055	89387396
chr6	89765610	89766033
chr7	100356259	100356585
chr7	100469149	100470401
chr7	100469149	100470405
chr7	100554845	100555368
chr7	101311275	101311725
chr7	101930110	101930819
chr7	102052128	102052902
chr7	102210345	102211185
chr7	102813276	102968100
chr7	103075160	103098526
chr7	103194976	103199965
chr7	103194976	103200403
chr7	103336089	103336453
chr7	105092970	105093124
chr7	105334698	105340635
chr7	105466074	105466367
chr7	105519475	105520264

chr7	105520491	105521419
chr7	105864348	105864764
chr7	11075203	11075850
chr7	1109327	1137404
chr7	111453209	111453414
chr7	122853900	122854363
chr7	122864563	122865625
chr7	123582138	123635772
chr7	127328581	127373479
chr7	128641202	128648986
chr7	128669921	128683255
chr7	129398577	129398923
chr7	129921621	129922093
chr7	129939881	129941064
chr7	130046627	130046988
chr7	131049537	131053465
chr7	134295611	134310894
chr7	135504930	135505293
chr7	135596188	135596636
chr7	135646557	135647535
chr7	138875618	138875883
chr7	138927560	138928030
chr7	139740041	139742038
chr7	139755018	139762689
chr7	140366407	140366874
chr7	143395077	143396255
chr7	143880415	143882099
chr7	148819736	148826198
chr7	149217471	149222742
chr7	149860008	149860247
chr7	1500395	1500891
chr7	151091877	151092251
chr7	151216819	151217547
chr7	152652489	152652750
chr7	155307295	155308172
chr7	155711346	155745186
chr7	158783449	158798036
chr7	1715785	1716162
chr7	17794250	17799131
chr7	2053383	2053538
chr7	22240132	22240481
chr7	2238014	2239676
chr7	22455179	22455879
chr7	22474601	22495480
chr7	22475944	22476413

chr7	2261433	2262069
chr7	2284688	2284981
chr7	2286314	2286869
chr7	2296911	2298773
chr7	2309088	2309471
chr7	23330208	23330907
chr7	23376722	23405110
chr7	23394341	23396198
chr7	23447933	23448474
chr7	23509726	23510355
chr7	2360799	2363124
chr7	2437803	2438545
chr7	2437803	2439330
chr7	2437803	2443746
chr7	2442691	2443746
chr7	24620154	24649713
chr7	24748429	24749263
chr7	26207795	26207934
chr7	27108085	27110300
chr7	27526120	27541755
chr7	27608263	27632441
chr7	29923955	29969013
chr7	29983130	29983945
chr7	32562674	32563280
chr7	33014534	33020519
chr7	33146268	33183405
chr7	3434176	3440256
chr7	39970274	39971620
chr7	40079326	40080082
chr7	42227650	42228908
chr7	43586271	43626586
chr7	43780120	43780886
chr7	43963935	43965029
chr7	44007187	44007557
chr7	44413190	44414027
chr7	44468081	44468338
chr7	44481291	44485622
chr7	44615108	44615762
chr7	44841637	44843042
chr7	4704253	4704760
chr7	50695591	50710990
chr7	5313822	5345757
chr7	5366627	5369083
chr7	5389754	5390115
chr7	5391577	5392220

chr7	5414195	5415888
chr7	5480864	5485820
chr7	5487482	5487817
chr7	5506651	5509128
chr7	5506660	5510238
chr7	55555946	55556534
chr7	56028067	56028250
chr7	5604364	5605662
chr7	5647522	5648298
chr7	5661372	5661531
chr7	5674260	5674802
chr7	5716822	5720130
chr7	5914343	5921046
chr7	6343299	6344851
chr7	6418963	6419876
chr7	6428268	6428596
chr7	6428935	6429517
chr7	6466221	6469718
chr7	64906004	64937630
chr7	65002626	65004104
chr7	65724925	65725430
chr7	65875821	65899620
chr7	65891227	65899620
chr7	65893448	65894049
chr7	65903442	65932789
chr7	65916480	65916721
chr7	65976407	65977750
chr7	6601934	6602973
chr7	66210876	66211229
chr7	66337467	66339831
chr7	66551519	66578727
chr7	66551519	66580042
chr7	66945037	66950971
chr7	67010267	67011596
chr7	67023295	67023549
chr7	67032556	67032947
chr7	67120313	67121299
chr7	67314379	67315984
chr7	67316137	67316946
chr7	67320264	67356102
chr7	696551	698177
chr7	73545516	73545868
chr7	73553239	73553413
chr7	73714383	73714906
chr7	74222336	74222619

chr7	74551843	74584896
chr7	74552157	74553224
chr7	74563821	74564062
chr7	74674094	74674345
chr7	75472861	75473093
chr7	75732753	75733800
chr7	7582629	7607136
chr7	7640738	7788864
chr7	76603422	76619304
chr7	76615493	76618390
chr7	76617965	76618390
chr7	76623120	76623531
chr7	768858	785897
chr7	78828294	78902612
chr7	78950545	78963455
chr7	8040475	8041611
chr7	80888330	80918410
chr7	846239	846436
chr7	846319	846644
chr7	848417	849603
chr7	87893508	87894173
chr7	92249098	92249505
chr7	93889061	93890240
chr7	96156839	96157550
chr7	96275092	96309691
chr7	98961306	98964720
chr7	99039115	99039455
chr7	99047654	99048023
chr7	99074561	99075729
chr7	99113580	99113835
chr7	99375714	99377209
chr7	99377561	99378420
chr7	99396599	99408573
chr7	99462046	99462326
chr7	99597935	99610601
chr8	100271098	100271851
chr8	102400046	102402394
chr8	103522004	103525991
chr8	104039269	104148848
chr8	104068511	104148848
chr8	104093470	104115709
chr8	104115531	104148848
chr8	104148630	104233710
chr8	105780460	105798799
chr8	105843127	105846409

chr8	108229028	108240098
chr8	117440959	117614274
chr8	118016806	118072777
chr8	118068161	118072777
chr8	118070787	118072777
chr8	118239714	118241270
chr8	120467597	120469068
chr8	122851601	122873594
chr8	122880172	122880556
chr8	123378624	123378898
chr8	123378624	123378901
chr8	123803580	123804196
chr8	124490432	124490892
chr8	124492200	124492560
chr8	124492205	124492560
chr8	124512347	124513024
chr8	124516610	124517185
chr8	124541997	124542164
chr8	125130212	125195272
chr8	125139413	125195272
chr8	125147080	125195272
chr8	125351390	125358573
chr8	125359174	125359428
chr8	127932052	128010444
chr8	127969415	128010444
chr8	127982359	128010444
chr8	127996560	128010444
chr8	128009567	128010444
chr8	128009589	128010444
chr8	128009589	128020719
chr8	128009589	128048609
chr8	128009589	128070361
chr8	128016634	128020719
chr8	128020315	128020719
chr8	128020338	128022705
chr8	128060163	128061004
chr8	130210089	130319668
chr8	130288250	130290138
chr8	13191428	13192051
chr8	133513899	133515882
chr8	140176490	140185770
chr8	140347756	140365686
chr8	140377393	140377895
chr8	140429322	140429612
chr8	140455739	140456137

chr8	140532056	140605914
chr8	140585090	140605914
chr8	140605523	140605914
chr8	140605531	140605914
chr8	140617537	140618050
chr8	140672308	140674953
chr8	140710652	140711019
chr8	140745922	140747000
chr8	140864316	140890774
chr8	140920813	140931063
chr8	140944983	140948723
chr8	141174922	141176216
chr8	143254993	143257020
chr8	143802643	143804456
chr8	144196197	144196451
chr8	144384201	144385673
chr8	144403996	144404618
chr8	144654359	144655890
chr8	144738492	144738706
chr8	144748168	144748309
chr8	1564004	1638584
chr8	22412924	22413793
chr8	23223363	23224059
chr8	23288515	23289362
chr8	23297337	23303628
chr8	23534201	23536950
chr8	38340631	38341279
chr8	41998673	42049707
chr8	42158526	42159072
chr8	42324479	42325463
chr8	42352981	42353203
chr8	42354424	42371662
chr8	42508281	42508621
chr8	42517197	42523925
chr8	42859628	42860454
chr8	42926547	42928793
chr8	42944401	42945326
chr8	43080277	43085396
chr8	47482512	47491994
chr8	47483334	47492046
chr8	47660345	47662962
chr8	47729996	47730981
chr8	48058375	48059503
chr8	55966054	55966426
chr8	55986055	55986852

chr8	61661805	61684186
chr8	6522703	6523048
chr8	65608831	65609212
chr8	65609780	65611043
chr8	65689243	65689599
chr8	66568538	66568843
chr8	70295190	70360000
chr8	71866530	71973659
chr8	73542901	73597488
chr8	73947289	73947932
chr8	73947710	73948095
chr8	76858015	76873493
chr8	79909543	79958435
chr8	79958256	79979470
chr8	79958256	80127629
chr8	86369008	86467010
chr8	86398574	86467010
chr8	88290710	88291177
chr8	89714423	89728755
chr8	94743307	94743727
chr8	94763224	94764301
chr8	94763809	94764294
chr8	94934036	94934467
chr8	96680442	96682076
chr8	96863675	96902096
chr8	97668194	97668649
chr8	97694810	97695233
chr8	98663929	98722969
chr9	100239998	100272982
chr9	100457757	100471231
chr9	104750954	104759440
chr9	105612894	105614939
chr9	106925968	107011037
chr9	110762468	110784980
chr9	110764671	110785728
chr9	110959492	111011690
chr9	111005464	111011690
chr9	111005464	111036201
chr9	111009147	111011690
chr9	111472896	111473939
chr9	111591219	111591479
chr9	111692375	111809084
chr9	111911025	111911611
chr9	113239896	113240836
chr9	113277090	113277715

chr9	116318603	116325747
chr9	121917166	121928144
chr9	124684600	124684986
chr9	125129409	125129980
chr9	125205770	125206807
chr9	125206789	125207288
chr9	125229340	125230059
chr9	125514062	125514454
chr9	125785455	125797465
chr9	125797394	125800988
chr9	125797394	125818978
chr9	126695286	126695584
chr9	126822720	126822919
chr9	126863591	126864235
chr9	126863591	126865803
chr9	127400435	127400778
chr9	128228291	128228717
chr9	128308877	128320870
chr9	128357425	128358460
chr9	128464436	128465460
chr9	128795729	128796659
chr9	128914992	128915276
chr9	128951215	128951887
chr9	128961419	128961716
chr9	129506176	129506420
chr9	130059423	130059774
chr9	130065344	130070398
chr9	130593297	130593887
chr9	130842272	130884485
chr9	131126760	131127145
chr9	13172658	13173464
chr9	13265663	13276771
chr9	132676602	132677111
chr9	133925842	133926327
chr9	133925842	133927892
chr9	133947680	133947871
chr9	133976429	133978130
chr9	133976432	133978130
chr9	134696465	134696786
chr9	135625331	135658151
chr9	135933056	135940245
chr9	136220549	136221526
chr9	136243020	136243353
chr9	136680936	136681563
chr9	136891396	136891610

chr9	136892787	136893844
chr9	137651702	137655093
chr9	15435459	15435938
chr9	15497596	15498177
chr9	19098944	19099276
chr9	19353535	19354646
chr9	21939159	22092465
chr9	22767174	22768403
chr9	2690950	2783594
chr9	2720343	2782807
chr9	2720343	2782846
chr9	32414502	32415806
chr9	33781875	33807480
chr9	33834807	33835241
chr9	34280206	34281135
chr9	35176316	35177025
chr9	35689603	35689831
chr9	36254754	36256157
chr9	36265420	36294559
chr9	37147361	37217122
chr9	37160064	37165935
chr9	37165869	37206494
chr9	37186711	37197879
chr9	37345655	37346038
chr9	37832062	37879131
chr9	37964899	37965766
chr9	38015758	38025547
chr9	38544753	38545193
chr9	41007439	41033022
chr9	41007439	41037838
chr9	4823530	4860901
chr9	4841204	4860901
chr9	4841224	4860901
chr9	4853609	4854754
chr9	5665677	5666072
chr9	5765468	5776856
chr9	5872157	5914062
chr9	5931543	5933881
chr9	593608	594080
chr9	593608	611565
chr9	6476805	6476999
chr9	71730738	71732475
chr9	71862550	71906650
chr9	71907807	71908177
chr9	71907820	71908130

chr9	77972297	77999328
chr9	78308862	78309457
chr9	79675500	79676265
chr9	83661836	83679921
chr9	88418777	88475513
chr9	88418777	88482355
chr9	88418849	88462631
chr9	89334638	89358853
chr9	91251107	91251592
chr9	91327499	91348504
chr9	91852320	91883383
chr9	91852320	91886770
chr9	91883257	91886770
chr9	91883257	91887113
chr9	91883257	91913127
chr9	93470328	93501120
chr9	93472195	93473073
chr9	94424859	94425175
chr9	95101614	95113611
chr9	95882273	95902088
chr9	96895895	96942752
chr9	96897934	96933915
chr9	96918642	96942752
chr9	97658689	97672781
chr9	97690823	97692028
chr9	98007633	98015517
chr9	98115692	98115934
chr9	98189384	98189641
chr9	99853203	99881985
Un_GL00021	74510	75077
Un_GL00021	90229	90629
chrX	100914620	100949326
chrX	101010373	101019721
chrX	102637995	102639533
chrX	102825992	102839972
chrX	102825992	102905737
chrX	103570567	103612987
chrX	104136815	104137983
chrX	108238400	108239982
chrX	108440005	108440504
chrX	108607274	108607578
chrX	109527169	109581793
chrX	110020351	110046484
chrX	110046356	110173453
chrX	115946446	115946730

chrX	118422626	118423204
chrX	123265487	123268356
chrX	123304877	123313950
chrX	123863358	123871037
chrX	123973773	123974288
chrX	129921093	129929895
chrX	130007733	130007959
chrX	130010933	130011282
chrX	130042880	130043126
chrX	130071101	130071727
chrX	131743095	131785258
chrX	131777622	131785258
chrX	131792749	131793043
chrX	134783845	134784242
chrX	134807536	134833128
chrX	1352619	1356140
chrX	1365102	1368090
chrX	1378040	1378812
chrX	14710945	14797298
chrX	14843659	14864809
chrX	14843895	14864703
chrX	152320808	152457574
chrX	152320808	152458262
chrX	152562850	152639794
chrX	152635881	152639794
chrX	152635881	152641295
chrX	152639603	152641295
chrX	152834650	152835121
chrX	153701056	153701877
chrX	153722963	153723258
chrX	154011312	154016215
chrX	154069171	154069569
chrX	154089936	154090225
chrX	154352582	154352883
chrX	154354862	154357547
chrX	154510447	154510715
chrX	154532949	154534494
chrX	154768143	154768699
chrX	16607023	16607972
chrX	17432851	17477320
chrX	17473060	17477320
chrX	17477145	17512549
chrX	18933036	18935712
chrX	19368864	19369479
chrX	21855513	21855764

chrX	2238869	2240061
chrX	2345070	2345915
chrX	3692816	3693676
chrX	3819098	3819478
chrX	3822072	3826729
chrX	40715117	40726595
chrX	46625262	46625978
chrX	48003633	48003810
chrX	48986164	48986501
chrX	53097040	53097447
chrX	53406967	53408091
chrX	53534750	53535362
chrX	53550721	53551405
chrX	63722761	63743446
chrX	68081700	68154229
chrX	68517357	68517740
chrX	68517416	68517691
chrX	70350620	70351808
chrX	71291830	71294340
chrX	71292453	71293326
chrX	72272620	72273353
chrX	73563226	73681116
chrX	74128014	74161374
chrX	74285914	74290430
chrX	75379171	75446032
chrX	77603647	77605188
chrX	77705642	77706246
chrX	77868643	77869049
chrX	80753575	80754735
chrX	84318851	84351815
chrX	84321841	84351815
chrX	85211855	85243726
chrX	86174147	86206904
chrX	86174692	86175903
chrX	91414903	91418867
chrX	91414903	91453186
chrX	91414903	91570957
chrX	9484252	9484836
chrX	96707250	96707570
chrX	97348117	97353891
	**ExonIntron (8124)**	
chr10	100536410	100546636
chr10	100540633	100550219
chr10	100923977	100926019
chr10	100923977	100931078

chr10	100923977	100938736
chr10	101781573	101782597
chr10	101812247	101813606
chr10	101956596	102014842
chr10	102351374	102351816
chr10	102735861	102810054
chr10	103111744	103174982
chr10	103136248	103174982
chr10	103967709	103993133
chr10	1056347	1072293
chr10	1076710	1078003
chr10	1079555	1080476
chr10	110295227	110304875
chr10	1103598	1105267
chr10	1103598	1125024
chr10	110502720	110510596
chr10	110951607	110965061
chr10	110951607	110985765
chr10	110964124	110967278
chr10	112964555	113089540
chr10	113040024	113097471
chr10	113086046	113089540
chr10	113898109	113908523
chr10	115461974	115469329
chr10	11594630	11597717
chr10	11597630	11601980
chr10	117318871	117345449
chr10	117340979	117345449
chr10	11745491	11749592
chr10	118009081	118040565
chr10	118685982	118695230
chr10	118685982	118701408
chr10	118685982	118730410
chr10	11921199	11952249
chr10	119326515	119327569
chr10	119326515	119342254
chr10	119576610	119593550
chr10	119577079	119580538
chr10	119579934	119596960
chr10	119581921	119591404
chr10	119581921	119593550
chr10	119588151	119588704
chr10	119588151	119594227
chr10	12014023	12021514
chr10	12072756	12084751

chr10	121790168	121926527
chr10	121898840	121926527
chr10	122226339	122228008
chr10	122389281	122406673
chr10	122485447	122489626
chr10	12249580	12250469
chr10	122880670	122888723
chr10	122928310	122938159
chr10	122983271	122990407
chr10	124760317	124765608
chr10	124760317	124774782
chr10	124760317	124790338
chr10	124767223	124790338
chr10	124940373	124943307
chr10	124940373	124966781
chr10	124942456	124943307
chr10	124942456	124966781
chr10	124942456	124974398
chr10	125038996	125040877
chr10	125038996	125074843
chr10	125038996	125088319
chr10	125038996	125122868
chr10	125038996	125138986
chr10	125038996	125139869
chr10	125074742	125111093
chr10	125074742	125133612
chr10	125079906	125111093
chr10	125088190	125111093
chr10	125110989	125111882
chr10	125110989	125122868
chr10	12529821	12553356
chr10	125745771	125763524
chr10	12649653	12666810
chr10	12662153	12666810
chr10	126926276	126990603
chr10	127052680	127067137
chr10	127409036	127451613
chr10	127982273	127982977
chr10	12814194	12828051
chr10	12814194	12828899
chr10	130159994	130180321
chr10	13123994	13136897
chr10	13127744	13136897
chr10	13172375	13172767
chr10	131955683	131956211

chr10	132244878	132245955
chr10	132540725	132650505
chr10	132705192	132710456
chr10	132940995	132942144
chr10	13333816	13336345
chr10	133398434	133399653
chr10	13480898	13526221
chr10	14848895	14849692
chr10	15102217	15104553
chr10	15128194	15128458
chr10	15128194	15130312
chr10	15212471	15214601
chr10	15248638	15309344
chr10	15275854	15313771
chr10	16695022	16734064
chr10	17390530	17405361
chr10	17590345	17604047
chr10	17599273	17604047
chr10	17660463	17673078
chr10	17725115	17776170
chr10	17733834	17752552
chr10	17733834	17776170
chr10	17733834	17907698
chr10	18533036	18548237
chr10	18585948	18622737
chr10	18596207	18643640
chr10	18609844	18643640
chr10	21571974	21595440
chr10	21571974	21617207
chr10	21649261	21651768
chr10	21668916	21673919
chr10	21668916	21681376
chr10	21879743	21882439
chr10	21879743	21920963
chr10	245900	387809
chr10	24851387	24864220
chr10	26720134	26724123
chr10	26720217	26724127
chr10	26742496	26743320
chr10	27077337	27086606
chr10	27093348	27098326
chr10	27111985	27116345
chr10	27526827	27538028
chr10	27526827	27542875
chr10	28583398	28584418

chr10	29420490	29458520
chr10	29457650	29458520
chr10	30313890	30322617
chr10	30322390	30337031
chr10	30941574	31017082
chr10	31336798	31461237
chr10	31355143	31461237
chr10	31373017	31461237
chr10	31373017	31502509
chr10	3138363	3138903
chr10	31839192	31839711
chr10	32009438	32023036
chr10	32048463	32050523
chr10	32451591	32456642
chr10	32451591	32481782
chr10	32451591	32485144
chr10	32469384	32474023
chr10	32469542	32474023
chr10	32471063	32481782
chr10	32471063	32584304
chr10	32511625	32518505
chr10	32543299	32584304
chr10	32543299	32595194
chr10	32543299	32623817
chr10	32543299	32635158
chr10	32565557	32584304
chr10	32565557	32635158
chr10	32634253	32729457
chr10	34243527	34269899
chr10	34251601	34269899
chr10	34284134	34302429
chr10	34450316	34493725
chr10	34450316	34545288
chr10	34470084	34493363
chr10	34516978	34545288
chr10	34694268	34696419
chr10	34696317	34700204
chr10	34696317	34728116
chr10	34696317	34739896
chr10	34696317	34746781
chr10	34696317	34800102
chr10	35137781	35152380
chr10	35188199	35193155
chr10	35374525	35501535
chr10	35422461	35516623

chr10	35434112	35516623
chr10	35516522	35530510
chr10	37775343	37776575
chr10	3779346	3782214
chr10	37803832	37838111
chr10	37803832	37856337
chr10	37803832	37856532
chr10	37831231	37838111
chr10	37953694	37972021
chr10	38159635	38177975
chr10	38177900	38178924
chr10	422823	522859
chr10	42451608	42472265
chr10	43557031	43558171
chr10	44885207	44923226
chr10	45639750	45663842
chr10	45663541	45665513
chr10	45862985	45964871
chr10	45888164	45964871
chr10	45939360	45964871
chr10	45939360	45965855
chr10	46006446	46016694
chr10	4830674	4847660
chr10	4830674	4860971
chr10	4830674	4889923
chr10	4830674	4908420
chr10	4830674	4909606
chr10	48308094	48401782
chr10	48325896	48404981
chr10	48325896	48410168
chr10	48325896	48420320
chr10	484780	486530
chr10	49827401	49832908
chr10	49932099	49934163
chr10	49945031	49973142
chr10	49952125	49973142
chr10	50243201	50251511
chr10	50340386	50344345
chr10	50433475	50439579
chr10	50433475	50555499
chr10	50584239	50590247
chr10	5412588	5451937
chr10	5709529	5712918
chr10	5709529	5717750
chr10	5709529	5720970

chr10	5783323	5800705
chr10	5785141	5811956
chr10	5956378	5960755
chr10	5959753	5960755
chr10	59832524	59906223
chr10	63183267	63197563
chr10	63264650	63414903
chr10	63380317	63384661
chr10	63380317	63404965
chr10	63380317	63409988
chr10	63380317	63414899
chr10	63380317	63414903
chr10	63380317	63429333
chr10	63380317	63447370
chr10	63380317	63450105
chr10	63380317	63455089
chr10	63380317	63497050
chr10	65571424	65585802
chr10	65571424	65670871
chr10	65572934	65585802
chr10	65585664	65615761
chr10	65585664	65689611
chr10	65684968	65689611
chr10	67966682	68073004
chr10	67988042	68014186
chr10	67990903	68014154
chr10	68014025	68067217
chr10	68025545	68044558
chr10	68025545	68067217
chr10	68038092	68067217
chr10	68109673	68122340
chr10	6812249	6838008
chr10	68206769	68210547
chr10	68206769	68215903
chr10	68398930	68401737
chr10	68430435	68430660
chr10	68437321	68470163
chr10	68442916	68483588
chr10	68457480	68465812
chr10	68458738	68470163
chr10	68483419	68488629
chr10	68483419	68516843
chr10	68493131	68517293
chr10	68672894	68673487
chr10	68747160	68901471

chr10	68754678	68766079
chr10	68906225	68910382
chr10	68906225	68913279
chr10	68907910	68919981
chr10	68936935	68948168
chr10	68955757	68967203
chr10	68957530	68960249
chr10	68959946	68967203
chr10	68963469	68965342
chr10	68999714	69011015
chr10	70152262	70161069
chr10	70152262	70161931
chr10	70339995	70340702
chr10	70419913	70421966
chr10	72097291	72102967
chr10	72336266	72343511
chr10	7240302	7285954
chr10	7243557	7283959
chr10	7243557	7290833
chr10	72562894	72584263
chr10	72708282	72715902
chr10	72715110	72719445
chr10	72715110	72721282
chr10	72715110	72736571
chr10	72715110	72742273
chr10	72790175	72871580
chr10	73072001	73074915
chr10	73109905	73163950
chr10	73134602	73148404
chr10	73141145	73146361
chr10	73177917	73235318
chr10	73471069	73479992
chr10	73541374	73576361
chr10	73545537	73606468
chr10	73571420	73576361
chr10	73575511	73576361
chr10	7365223	7381949
chr10	73692042	73713332
chr10	73704985	73724838
chr10	73911524	73911843
chr10	74063685	74083513
chr10	74242737	74314745
chr10	74242737	74398579
chr10	74267564	74398579
chr10	74340620	74398579

chr10	74358443	74398579
chr10	74380928	74398579
chr10	74394140	74533656
chr10	75150513	75168693
chr10	75168540	75182011
chr10	76034263	76058783
chr10	76046878	76058783
chr10	76324400	76346553
chr10	76324400	76347110
chr10	76324400	76369315
chr10	76557208	76561504
chr10	77249564	77251256
chr10	77376009	77404023
chr10	77403861	77418934
chr10	77403861	77420179
chr10	77403861	77445879
chr10	77403861	77447869
chr10	77403861	77459337
chr10	7797053	7802854
chr10	78035351	78040747
chr10	79110437	79139777
chr10	79310923	79313987
chr10	79691621	79692056
chr10	80280172	80284032
chr10	80468546	80489314
chr10	80492535	80507374
chr10	80504778	80507204
chr10	812920	817889
chr10	836402	890829
chr10	84380097	84438707
chr10	84380097	84477664
chr10	84415391	84477664
chr10	84417770	84456626
chr10	84438281	84477664
chr10	863742	903516
chr10	86457527	86460496
chr10	86470991	86488504
chr10	86470991	86490210
chr10	87049400	87062835
chr10	87051582	87068157
chr10	87051712	87068157
chr10	87506150	87521169
chr10	87508335	87552541
chr10	87864453	87933251
chr10	87864453	87952259

chr10	87864453	87958019
chr10	87875404	87933251
chr10	87875404	87952259
chr10	87878925	87933251
chr10	87880299	87933251
chr10	87894024	87895777
chr10	87922462	87933251
chr10	88309386	88314641
chr10	884446	889057
chr10	884446	890829
chr10	88893870	88905660
chr10	89717795	89718872
chr10	91959217	91966757
chr10	92081319	92243395
chr10	92151232	92192636
chr10	92333469	92340803
chr10	92508727	92559215
chr10	92531747	92559215
chr10	92893329	92909631
chr10	92948273	92961458
chr10	92997473	93058335
chr10	93381266	93389154
chr10	93452049	93460351
chr10	94169774	94171496
chr10	94333389	94334305
chr10	94350112	94350541
chr10	94354962	94361441
chr10	94429758	94474783
chr10	94441895	94444539
chr10	94441895	94451609
chr10	94468874	94474783
chr10	94474667	94477829
chr10	94588228	94608026
chr10	94683268	94683987
chr10	94733296	94775539
chr10	95225547	95238685
chr10	95228337	95247366
chr10	95876517	95904938
chr10	96131024	96156047
chr10	96155325	96160402
chr10	96544075	96567804
chr10	96544075	96578320
chr10	96858291	96907747
chr10	96884402	96944245
chr10	96944140	96952196

chr10	97358643	97358942
chr10	97388251	97388715
chr10	97388251	97388795
chr10	97436416	97437750
chr10	97436416	97438703
chr10	97469127	97469645
chr10	97616230	97619683
chr10	98208100	98244298
chr10	99405755	99421079
chr10	99739709	99755778
chr10	99750368	99756910
chr10	99884010	99909525
chr10	99899918	99931445
chr10	99969114	100006342
chr10	99969114	99973229
chr10	99971979	100003023
chr11	10188348	10193987
chr11	101986831	101997733
chr11	102114143	102164693
chr11	102174317	102209564
chr11	102178820	102206074
chr11	102197857	102223752
chr11	102350345	102368548
chr11	102350843	102378157
chr11	102401915	102452721
chr11	102950109	102955973
chr11	10305679	10307081
chr11	10305948	10306615
chr11	103062274	103066567
chr11	103158676	103163350
chr11	103316544	103317004
chr11	106009582	106011067
chr11	106090556	106096893
chr11	107308282	107336713
chr11	107308282	107349053
chr11	107308282	107353736
chr11	107326005	107330019
chr11	107326908	107336713
chr11	107326923	107336713
chr11	107353523	107418284
chr11	107410086	107418287
chr11	108061039	108072462
chr11	108061039	108073497
chr11	108127885	108131954
chr11	108135141	108148858

chr11	108158919	108170043
chr11	108197301	108204810
chr11	108223952	108235834
chr11	108223952	108256340
chr11	108227594	108236172
chr11	108713380	108723462
chr11	108715878	108717615
chr11	108715878	108768001
chr11	108908862	108918018
chr11	110263331	110264874
chr11	110435833	110442885
chr11	111683926	111688162
chr11	111785788	111786520
chr11	112230230	112230595
chr11	113799145	113799415
chr11	113799145	113805046
chr11	114443855	114450166
chr11	11448743	11476452
chr11	116756227	116758407
chr11	116949713	116957064
chr11	117391215	117392203
chr11	11873799	11880380
chr11	11873799	11922891
chr11	119015962	119017361
chr11	119088254	119090335
chr11	119126934	119128448
chr11	119228140	119232695
chr11	119232447	119232729
chr11	119255177	119271881
chr11	119277784	119278713
chr11	119284968	119299587
chr11	119296917	119299587
chr11	119296917	119307828
chr11	12109700	12162419
chr11	12153223	12162419
chr11	122116132	122155632
chr11	122116132	122180398
chr11	122155550	122202909
chr11	122202763	122203580
chr11	122808176	122809716
chr11	122946497	122959530
chr11	123097794	123113646
chr11	12318059	12327266
chr11	123480815	123577577
chr11	124801983	124809098

chr11	12495060	12496598
chr11	125629231	125656335
chr11	125643791	125656335
chr11	125653745	125656335
chr11	126203344	126205781
chr11	126289664	126294639
chr11	126338285	126345499
chr11	126380829	126409411
chr11	129040927	129113625
chr11	129093620	129127305
chr11	129123445	129127305
chr11	129164318	129170627
chr11	129164318	129267361
chr11	130260855	130288893
chr11	134248370	134251308
chr11	14294470	14356769
chr11	14457783	14458687
chr11	14745009	14789242
chr11	14852673	14885917
chr11	14867505	14869630
chr11	16751713	16752894
chr11	17145667	17169806
chr11	17168676	17169806
chr11	17168676	17184120
chr11	17356650	17379665
chr11	17873506	18008076
chr11	17918732	18008076
chr11	17959469	17962291
chr11	17959469	18010139
chr11	17988596	18010139
chr11	18403811	18405376
chr11	18480484	18484072
chr11	19819174	19832601
chr11	19976746	19984247
chr11	20364236	20383258
chr11	20388018	20413034
chr11	20404877	20426865
chr11	20407910	20435344
chr11	24729168	24771932
chr11	27491426	27501920
chr11	28211061	28212331
chr11	2951258	2982211
chr11	2969802	2972279
chr11	2969802	2989271
chr11	30330885	30336826

chr11	31452717	31463305
chr11	31456259	31488241
chr11	32097147	32098528
chr11	32388819	32400044
chr11	32388819	32428619
chr11	33061529	33072653
chr11	33141666	33161369
chr11	33276730	33287511
chr11	33286412	33286952
chr11	3362545	3369502
chr11	34142274	34147666
chr11	34882675	34895110
chr11	34957383	34998196
chr11	3500465	3508396
chr11	35198071	35208206
chr11	35200645	35204640
chr11	35201195	35201643
chr11	35208104	35210181
chr11	35663684	35726163
chr11	36036102	36038184
chr11	36036102	36038224
chr11	3705199	3710325
chr11	3941215	4023987
chr11	3967551	3970235
chr11	4132285	4138445
chr11	43326506	43337596
chr11	43335857	43342687
chr11	43372270	43389821
chr11	43373509	43379322
chr11	43743293	43754121
chr11	43750910	43760583
chr11	45181765	45183123
chr11	45861462	45862148
chr11	458860	460288
chr11	46512564	46512813
chr11	47161190	47161436
chr11	47161190	47161438
chr11	47216335	47238955
chr11	47422573	47422989
chr11	47615962	47625741
chr11	47747505	47765362
chr11	47806152	47808793
chr11	497970	505001
chr11	562776	563165
chr11	5645684	5678434

chr11	57696760	57700397
chr11	57698558	57700397
chr11	57704301	57704815
chr11	59658038	59659469
chr11	601573	603436
chr11	61132353	61138835
chr11	61132419	61138835
chr11	61322295	61333008
chr11	61366117	61367998
chr11	61411768	61416189
chr11	61436760	61438113
chr11	61437624	61438080
chr11	61437624	61438120
chr11	61437624	61438362
chr11	61437624	61446113
chr11	61437624	61446231
chr11	61795340	61798153
chr11	61879532	61880040
chr11	62381796	62391632
chr11	62533660	62536567
chr11	62632972	62639731
chr11	62735381	62738490
chr11	62839113	62839706
chr11	62839113	62839867
chr11	64320660	64321874
chr11	64802683	64802884
chr11	65032203	65032409
chr11	65546941	65547438
chr11	65551125	65552083
chr11	65862529	65862976
chr11	65863901	65864463
chr11	65870290	65870535
chr11	65894013	65894497
chr11	65894013	65897111
chr11	65998441	65999675
chr11	66000052	66001143
chr11	66003234	66003753
chr11	6610905	6611818
chr11	66269562	66275935
chr11	66365300	66367548
chr11	66365381	66366564
chr11	66435972	66438259
chr11	66639677	66640123
chr11	66646026	66657259
chr11	66756352	66762450

chr11	66759042	66762450
chr11	66813845	66828364
chr11	66815015	66822674
chr11	66828265	66828943
chr11	66844364	66844842
chr11	67304048	67310339
chr11	67490457	67490768
chr11	67608399	67609635
chr11	68261751	68262730
chr11	68347846	68365698
chr11	68357649	68365698
chr11	68515027	68519651
chr11	68515027	68537891
chr11	68533793	68537891
chr11	68775119	68775432
chr11	6932093	6955782
chr11	6932093	6988315
chr11	6932093	6988320
chr11	6941570	6955782
chr11	69666189	69671819
chr11	70337364	70339311
chr11	70349922	70356254
chr11	70402838	70405361
chr11	70429050	70433142
chr11	71215342	71224796
chr11	71916073	71926510
chr11	71935304	71987111
chr11	71954621	71960891
chr11	71982760	71983770
chr11	71982806	71990717
chr11	71999926	72019009
chr11	72231189	72231474
chr11	72354307	72359008
chr11	72402965	72410372
chr11	72758656	72759233
chr11	73409486	73418470
chr11	73419873	73582899
chr11	73676706	73679720
chr11	73677720	73679720
chr11	73706388	73716362
chr11	73707419	73716345
chr11	73716250	73718885
chr11	73716726	73718718
chr11	73787901	73825796
chr11	73825707	73864433

chr11	73843246	73844976
chr11	74012577	74123135
chr11	74085521	74085886
chr11	74132843	74165062
chr11	7461238	7469766
chr11	74625407	74640841
chr11	74629216	74640841
chr11	74634582	74640841
chr11	74775145	74817714
chr11	74784432	74789699
chr11	74784432	74810330
chr11	74963594	74969699
chr11	752263	756002
chr11	75477618	75479488
chr11	75566971	75568704
chr11	75979777	75983513
chr11	76463801	76472840
chr11	76544258	76549017
chr11	7665323	7672891
chr11	76888497	76926667
chr11	77616205	77629899
chr11	77624941	77625818
chr11	77722054	77725699
chr11	77724347	77725699
chr11	77841018	77842628
chr11	77918875	77941251
chr11	77938650	77991293
chr11	78069891	78073141
chr11	78101092	78109581
chr11	78436694	78443758
chr11	78436694	78493195
chr11	78489912	78493195
chr11	78536825	78571444
chr11	82901007	82901822
chr11	82913286	82931849
chr11	82914722	82931849
chr11	83073302	83081800
chr11	836062	838367
chr11	85046477	85154651
chr11	85280533	85285365
chr11	85974707	86042885
chr11	86031468	86042885
chr11	8621343	8621644
chr11	86249470	86252240
chr11	86249470	86257596

chr11	86256427	86266216
chr11	8638653	8654578
chr11	8682816	8683179
chr11	87067693	87075786
chr11	87067693	87158771
chr11	87157340	87158771
chr11	8750620	8871088
chr11	8796720	8811335
chr11	8802829	8811335
chr11	88309162	88337683
chr11	88334936	88337683
chr11	8912452	8919520
chr11	8912452	8922115
chr11	899425	900003
chr11	9283370	9299629
chr11	9287701	9300116
chr11	93162705	93170221
chr11	93673977	93679552
chr11	93683558	93695673
chr11	93736140	93736751
chr11	93747296	93761725
chr11	93788000	93814611
chr11	9414235	9420698
chr11	9423140	9423743
chr11	9437728	9442197
chr11	94485927	94490965
chr11	94607555	94608686
chr11	94869197	94871030
chr11	9575887	9588537
chr11	95799231	95832805
chr11	95827785	95832805
chr11	96091891	96093135
chr11	9779462	9785318
chr11	9961956	10006856
chr11	9961956	10020284
chr12	100095666	100128725
chr12	100591697	100622382
chr12	101768036	101770507
chr12	102039867	102050291
chr12	102074368	102077503
chr12	102145976	102148463
chr12	102148229	102150371
chr12	102164437	102196087
chr12	10307977	10315473
chr12	104309758	104327671

chr12	104348352	104387984
chr12	104565119	104601991
chr12	104844471	104915088
chr12	104886292	104900301
chr12	104895273	104915088
chr12	104909654	104931791
chr12	104991711	104994723
chr12	104991711	104996026
chr12	104994477	104996026
chr12	105174095	105197953
chr12	105229124	105233216
chr12	106842588	106870942
chr12	10709907	10710788
chr12	10712929	10713333
chr12	10831067	10848407
chr12	108652271	108657699
chr12	108701123	108717762
chr12	108701123	108718954
chr12	108701123	108729170
chr12	108817037	108818558
chr12	108818248	108818558
chr12	108849034	108852678
chr12	109451590	109460805
chr12	10973654	10993219
chr12	10973654	11121180
chr12	110011990	110013249
chr12	110180421	110218187
chr12	11028400	11047188
chr12	11034781	11059410
chr12	11047019	11121180
chr12	11047019	11124187
chr12	11047019	11129363
chr12	11047019	11133367
chr12	11047019	11147135
chr12	110473077	110486700
chr12	110473077	110490171
chr12	1104749	1168046
chr12	110485077	110490171
chr12	110536923	110546078
chr12	110536923	110551968
chr12	110730528	110736035
chr12	111418118	111420488
chr12	111485264	111513539
chr12	111522694	111525316
chr12	111552279	111565419

chr12	111552279	111571182
chr12	111692696	111749103
chr12	112039228	112049611
chr12	112047664	112049611
chr12	112250143	112252528
chr12	112256319	112259174
chr12	112445582	112456063
chr12	112446275	112456898
chr12	112446275	112457379
chr12	113304399	113306494
chr12	115996475	115997227
chr12	116066801	116111512
chr12	116096668	116237542
chr12	116180035	116237705
chr12	116205052	116237705
chr12	116227809	116237705
chr12	116230432	116237705
chr12	116230532	116237705
chr12	116236820	116237705
chr12	116237156	116237705
chr12	116237467	116240462
chr12	116237467	116246305
chr12	116918530	116949706
chr12	117039029	117046838
chr12	11732362	11752579
chr12	11738626	11752579
chr12	11750526	11752579
chr12	11750953	11752579
chr12	118024850	118032618
chr12	118033646	118035324
chr12	1183280	1255946
chr12	118400658	118414470
chr12	118403411	118414470
chr12	11884444	11891569
chr12	119690150	119690451
chr12	120200909	120201082
chr12	120539367	120546601
chr12	120557339	120557682
chr12	120713273	120727189
chr12	121232413	121233740
chr12	121296637	121494665
chr12	121420241	121423517
chr12	121452392	121453344
chr12	121453119	121455536
chr12	121548876	121581134

chr12	121934044	121934356
chr12	122035330	122035740
chr12	122054804	122055408
chr12	122129629	122129915
chr12	122152892	122186300
chr12	122232325	122232968
chr12	12244261	12259895
chr12	122483459	122498863
chr12	122522142	122522906
chr12	122530021	122539754
chr12	122530021	122542127
chr12	122530021	122543634
chr12	122568321	122577029
chr12	122579904	122582848
chr12	122805369	122806295
chr12	122868479	122871396
chr12	122935271	122946220
chr12	123110384	123141399
chr12	123334052	123357852
chr12	123498543	123518494
chr12	123603847	123606159
chr12	12361205	12367409
chr12	123647962	123651156
chr12	123673617	123677081
chr12	123688779	123697198
chr12	123751109	123758163
chr12	124347936	124348173
chr12	124362125	124476764
chr12	12616160	12637714
chr12	12635776	12644428
chr12	1282798	1290012
chr12	128814774	128815652
chr12	1289851	1319288
chr12	1289851	1326260
chr12	130785809	130801488
chr12	130791673	130808704
chr12	130808602	130827267
chr12	130827192	130827481
chr12	130872835	130875997
chr12	131932174	131944015
chr12	132086374	132090943
chr12	13211468	13214673
chr12	132507251	132508350
chr12	132507251	132525823
chr12	132720677	132721217

chr12	132734384	132737305
chr12	132747831	132748257
chr12	132774342	132775140
chr12	132816659	132822311
chr12	133038874	133048848
chr12	1371832	1412538
chr12	14794970	14796950
chr12	15217428	15223901
chr12	15217428	15229533
chr12	15217428	15337470
chr12	15955896	15982436
chr12	1616795	1617143
chr12	1631297	1646297
chr12	16354230	16383604
chr12	1690038	1772961
chr12	1702885	1754514
chr12	1702885	1772961
chr12	1744521	1754514
chr12	1754257	1757459
chr12	19443118	19473355
chr12	19462509	19466850
chr12	19462509	19484053
chr12	19462509	19518841
chr12	19466747	19500221
chr12	19471715	19500221
chr12	21304426	21334704
chr12	21506884	21515931
chr12	21512294	21515931
chr12	22642416	22644022
chr12	22642416	22661205
chr12	22659013	22685186
chr12	25209798	25227412
chr12	25213113	25227412
chr12	25995036	25999113
chr12	26041973	26055446
chr12	26769348	26790227
chr12	26922615	26930342
chr12	27368261	27371447
chr12	27368261	27376416
chr12	27368261	27376428
chr12	27368261	27377564
chr12	27368261	27426317
chr12	27368261	27524365
chr12	27426131	27474913
chr12	27426131	27475731

chr12	2797728	2804363
chr12	28197122	28391411
chr12	28198942	28307749
chr12	28225794	28259442
chr12	28225794	28307749
chr12	28225794	28391411
chr12	2822793	2826793
chr12	2822793	2830885
chr12	28235569	28391411
chr12	28255580	28259442
chr12	28255580	28307749
chr12	28255580	28391411
chr12	28296855	28307749
chr12	28303191	28307749
chr12	28304625	28307749
chr12	28304625	28391411
chr12	28305648	28328191
chr12	2830833	2840995
chr12	2866392	2877145
chr12	30741019	30753609
chr12	31281860	31298224
chr12	31282715	31298224
chr12	31396318	31398362
chr12	31495809	31541031
chr12	31495809	31542949
chr12	31495809	31571180
chr12	3201176	3206418
chr12	32107275	32306122
chr12	32305663	32306122
chr12	32559618	32701562
chr12	32698220	32701562
chr12	32706689	32740240
chr12	32742588	32743508
chr12	32745679	32747363
chr12	32747075	32750874
chr12	362962	376724
chr12	38670728	38679714
chr12	39822628	39830228
chr12	39822628	39871961
chr12	39951256	39991421
chr12	40028300	40085700
chr12	40028300	40092933
chr12	40028300	40100089
chr12	42199135	42210680
chr12	42210354	42210619

chr12	42373392	42398994
chr12	42374637	42375074
chr12	42374637	42398994
chr12	45176240	45190961
chr12	45293271	45357424
chr12	45860800	45872962
chr12	45891248	45893721
chr12	45922462	45926291
chr12	46229152	46256769
chr12	46239678	46256769
chr12	47104112	47108164
chr12	47212596	47216689
chr12	48142781	48145746
chr12	48151200	48153525
chr12	48659904	48660619
chr12	48662559	48679833
chr12	48712261	48714524
chr12	48923781	48926011
chr12	48923867	48924222
chr12	48936151	48941188
chr12	48939034	48941188
chr12	49097565	49098105
chr12	49097565	49100454
chr12	49460997	49497009
chr12	49483735	49500205
chr12	49486249	49490731
chr12	49524681	49527713
chr12	49749617	49759321
chr12	50427744	50441643
chr12	50770028	50796009
chr12	50775581	50796009
chr12	51056349	51063553
chr12	51683741	51688849
chr12	51684173	51689096
chr12	51769335	51769864
chr12	51980988	51986942
chr12	52171133	52185587
chr12	53024841	53027919
chr12	53065992	53066313
chr12	53227361	53232192
chr12	53454749	53471807
chr12	53455884	53456747
chr12	54053159	54055198
chr12	54241743	54257692
chr12	54391234	54391940

chr12	55967856	55971712
chr12	56019317	56023764
chr12	56106587	56112889
chr12	56159586	56162331
chr12	56224833	56229675
chr12	56248892	56252014
chr12	56254374	56254577
chr12	56285915	56298691
chr12	56731095	56734245
chr12	57256097	57256413
chr12	57292571	57295507
chr12	57783283	57783586
chr12	62549667	62560657
chr12	62720170	62879233
chr12	63844795	64042972
chr12	63983946	63997128
chr12	64051919	64065219
chr12	64752697	64756762
chr12	6492201	6492972
chr12	6494115	6522037
chr12	6521857	6523002
chr12	65238501	65246505
chr12	65326825	65454663
chr12	6556949	6557642
chr12	65828000	65881890
chr12	6602177	6602375
chr12	66145840	66161172
chr12	66305031	66306514
chr12	6655637	6656798
chr12	6724613	6731210
chr12	68321346	68325247
chr12	6837086	6837372
chr12	6837086	6837399
chr12	6837086	6837401
chr12	68686996	68692112
chr12	68690602	68715740
chr12	68748075	68752190
chr12	68757147	68759239
chr12	69251128	69269668
chr12	69252988	69262562
chr12	69258034	69269668
chr12	69258940	69262562
chr12	69258998	69262562
chr12	6934578	6935408
chr12	69597275	69597616

chr12	70278131	70285586
chr12	71618331	71620315
chr12	72286314	72286954
chr12	72370763	72499635
chr12	7316960	7433694
chr12	7326897	7374756
chr12	7347027	7357486
chr12	7368057	7374756
chr12	7379236	7433694
chr12	7403534	7436602
chr12	7428811	7433694
chr12	76063108	76068994
chr12	76821057	76822531
chr12	76841962	76846679
chr12	76842024	76846679
chr12	76842024	76849470
chr12	794268	830160
chr12	79574865	79596658
chr12	79574865	79621207
chr12	79592610	79596658
chr12	79592610	79621207
chr12	79593902	79594433
chr12	79612492	79621207
chr12	79613574	79666631
chr12	81676790	81711865
chr12	820496	827262
chr12	82369386	82403130
chr12	82369386	82456820
chr12	82896541	82930545
chr12	84883854	84892308
chr12	86838665	86838880
chr12	871264	873178
chr12	88049291	88055717
chr12	89420279	89425379
chr12	89448912	89472275
chr12	89642157	89677816
chr12	89655678	89656107
chr12	89655678	89663091
chr12	8971077	8995288
chr12	91986561	91993529
chr12	93409908	93411299
chr12	93565612	93567159
chr12	94308782	94331829
chr12	94366713	94376017
chr12	95476073	95496098

chr12	9606352	9617351
chr12	96194539	96259853
chr12	96198025	96223773
chr12	96223564	96225261
chr12	96223564	96247283
chr12	96311051	96395917
chr12	9680922	9694969
chr12	96934975	96952162
chr12	98537474	98547671
chr12	98544229	98547671
chr12	98595432	98595661
chr12	99375390	99399811
chr12	99386261	99399811
chr12	99709902	99782097
chr12	99709902	99806700
chr12	99744404	99779972
chr12	99759333	99806700
chr12	99774602	99825389
chr12	998323	1028572
chr13	100386768	100425731
chr13	100515426	100539370
chr13	101688637	101692701
chr13	102807557	102822159
chr13	102807611	102822159
chr13	108303252	108335572
chr13	110520749	110524197
chr13	110627438	110638492
chr13	111280350	111280505
chr13	113013264	113024758
chr13	113302211	113309862
chr13	113835512	113838096
chr13	114040999	114057441
chr13	114040999	114057474
chr13	114052051	114057441
chr13	114052051	114057474
chr13	19751270	19752316
chr13	20615792	20641510
chr13	20728279	20757617
chr13	20821703	20856002
chr13	20842894	20868697
chr13	20855626	20863123
chr13	21045684	21046446
chr13	23360313	23365277
chr13	23798723	23809924
chr13	23798723	23837756

chr13	23798723	23841488
chr13	23798723	23870195
chr13	23798723	23886506
chr13	23836239	23841407
chr13	24211274	24224582
chr13	24245775	24251862
chr13	24249476	24253220
chr13	24249476	24255219
chr13	24249476	24257409
chr13	24300400	24304846
chr13	24474531	24478276
chr13	24475620	24477700
chr13	24482146	24493733
chr13	24484652	24488284
chr13	26262549	26353880
chr13	26299566	26337642
chr13	26299566	26353880
chr13	26324771	26353880
chr13	26573714	26613058
chr13	26577354	26642403
chr13	26593607	26613058
chr13	26593607	26642403
chr13	27069089	27075388
chr13	27069089	27090158
chr13	27069110	27075388
chr13	27069110	27090158
chr13	27578718	27581803
chr13	28239578	28261458
chr13	28266717	28272071
chr13	28701500	28713075
chr13	29024456	29034125
chr13	29222511	29359473
chr13	29242296	29281865
chr13	29277319	29281865
chr13	29445578	29536202
chr13	29453067	29485306
chr13	29453067	29536202
chr13	29484868	29492665
chr13	29497237	29503492
chr13	29514352	29524253
chr13	29514352	29536202
chr13	29514352	29553860
chr13	29517015	29530712
chr13	29517015	29532982
chr13	29517143	29527365

chr13	29517330	29517572
chr13	29522316	29536485
chr13	29523506	29524131
chr13	29523531	29524131
chr13	29530537	29540603
chr13	29530624	29532982
chr13	29530743	29532823
chr13	29550203	29553860
chr13	29553760	29554673
chr13	29553760	29560349
chr13	29553760	29576701
chr13	29553760	29588846
chr13	29767098	29777263
chr13	30207641	30210442
chr13	30208514	30283791
chr13	30208576	30241086
chr13	30208576	30283791
chr13	30227411	30283687
chr13	30227446	30283791
chr13	30227499	30283791
chr13	30230510	30241086
chr13	30230510	30283791
chr13	30240459	30241681
chr13	30240459	30284383
chr13	30240958	30284383
chr13	30251930	30255615
chr13	30251930	30283791
chr13	30254104	30255615
chr13	30254104	30283791
chr13	30255446	30257867
chr13	30255446	30284882
chr13	30274693	30283791
chr13	30280062	30284383
chr13	30280062	30284882
chr13	31269213	31270355
chr13	31941307	31946181
chr13	32432788	32444126
chr13	32629687	32710106
chr13	35040977	35070852
chr13	35171271	35196302
chr13	36865536	36884763
chr13	39687502	39771363
chr13	39694633	39751144
chr13	39719208	39727548
chr13	40343956	40350302

chr13	40836596	40864243
chr13	40933469	40941370
chr13	41252644	41260875
chr13	41811224	41837250
chr13	41881958	41912168
chr13	42119094	42129632
chr13	45151686	45195515
chr13	45184413	45207505
chr13	45516142	45534816
chr13	46003138	46045513
chr13	46020448	46045513
chr13	46020448	46052633
chr13	46042163	46045513
chr13	48077152	48090418
chr13	48253807	48261321
chr13	48994471	49006289
chr13	49097547	49145935
chr13	49701418	49746993
chr13	49997073	50045232
chr13	50390197	50415202
chr13	50492650	50493534
chr13	50492650	50505684
chr13	50912994	50934999
chr13	50927406	50950318
chr13	51365702	51395483
chr13	59861406	59888367
chr13	59911734	59932815
chr13	72893783	72939934
chr13	73813151	73813677
chr13	75560752	75590129
chr13	75560752	75621868
chr13	75621762	75800882
chr13	75621762	75804541
chr13	75621762	75819535
chr13	77118339	77126542
chr13	77126317	77129230
chr13	87445010	87511470
chr13	87511365	87560417
chr13	94586963	94592309
chr13	94603260	94621235
chr13	95157160	95170628
chr13	95194835	95216371
chr13	95209247	95210781
chr13	95234609	95266264
chr13	95815808	95837202

chr13	95985238	95996135
chr13	96023039	96030751
chr13	98148592	98213413
chr13	98213219	98271346
chr13	98403871	98412034
chr13	98500060	98519473
chr13	98504914	98519473
chr13	98519242	98551973
chr13	98955434	98976089
chr13	99237346	99238554
chr13	99238426	99254954
chr13	99340434	99367906
chr13	99501018	99501777
chr14	100084686	100102379
chr14	102193462	102197847
chr14	102265822	102283897
chr14	102348278	102350048
chr14	102662322	102681978
chr14	102663790	102714617
chr14	102714422	102743710
chr14	102816738	102830472
chr14	102861524	102876525
chr14	103398950	103405267
chr14	103398950	103451983
chr14	103405075	103408139
chr14	103405075	103411765
chr14	103405075	103438333
chr14	103405075	103441563
chr14	103468085	103492034
chr14	103587273	103590429
chr14	103587273	103590977
chr14	103773612	103797518
chr14	103773632	103797518
chr14	103778744	103798868
chr14	103778744	103807869
chr14	104713444	104714395
chr14	104780087	104793304
chr14	105272720	105279033
chr14	105379829	105380056
chr14	20395449	20395946
chr14	21082478	21082827
chr14	21230318	21248866
chr14	21230957	21236460
chr14	21426127	21429038
chr14	21495493	21499137

chr14	21503172	21508235
chr14	22571878	22575233
chr14	22906194	22913845
chr14	22920987	22923160
chr14	22987519	22998741
chr14	23033367	23033608
chr14	23075495	23093544
chr14	23293530	23299541
chr14	23301878	23302159
chr14	23301880	23302159
chr14	23301884	23302159
chr14	23301885	23302159
chr14	23966282	23995133
chr14	24202382	24211829
chr14	24206535	24211829
chr14	24209889	24211829
chr14	24211410	24211829
chr14	24235736	24240283
chr14	24290721	24296881
chr14	24430931	24431439
chr14	26472319	26483402
chr14	29712937	29725674
chr14	29724469	29725674
chr14	30580863	30581116
chr14	30580863	30593639
chr14	30622427	30628279
chr14	30625116	30675065
chr14	30935162	30959498
chr14	31127784	31129307
chr14	31156839	31173311
chr14	31162592	31169468
chr14	31169353	31173311
chr14	31561405	31562215
chr14	31771815	31787873
chr14	33922418	33931215
chr14	33925617	33931215
chr14	34783761	34792835
chr14	34862043	34862289
chr14	35085392	35091207
chr14	35091231	35095047
chr14	35125440	35180777
chr14	35127478	35135244
chr14	37240432	37247907
chr14	37240432	37268793
chr14	37240432	37308519

chr14	37240446	37308519
chr14	37324763	37422949
chr14	39180502	39183830
chr14	39399635	39402511
chr14	45046544	45047024
chr14	45118027	45130790
chr14	45149730	45155459
chr14	46920011	46994727
chr14	47035010	47097039
chr14	47301410	47440220
chr14	49653989	49655811
chr14	49683592	49686503
chr14	50114284	50140233
chr14	50180571	50212676
chr14	50199690	50223670
chr14	50237676	50303017
chr14	50247040	50294246
chr14	50359027	50396017
chr14	50626381	50645014
chr14	50635213	50645014
chr14	51243855	51288063
chr14	51333392	51489420
chr14	51554589	51570410
chr14	52455323	52472987
chr14	52457089	52476347
chr14	52596916	52600341
chr14	52730458	52771183
chr14	52778303	52784664
chr14	54436078	54436437
chr14	54567672	54702580
chr14	54604371	54702580
chr14	54668791	54702580
chr14	54681934	54702580
chr14	54702061	54705753
chr14	54702061	54789092
chr14	54702061	54790908
chr14	54736675	54737287
chr14	54736796	54737287
chr14	54737023	54790908
chr14	54962067	55000992
chr14	55350380	55355222
chr14	55671788	55678444
chr14	58286639	58291707
chr14	58290698	58291707
chr14	58291634	58293926

chr14	58304943	58321512
chr14	58324466	58346505
chr14	60114699	60134048
chr14	60115376	60116060
chr14	60115376	60125823
chr14	60115718	60124310
chr14	60723211	60724047
chr14	60796216	60819051
chr14	60811485	60879835
chr14	61995875	61996542
chr14	62778664	62779924
chr14	63598755	63599973
chr14	63598755	63625033
chr14	64055921	64070910
chr14	64223188	64225498
chr14	64390336	64400877
chr14	65432036	65455718
chr14	65455620	65458207
chr14	65455620	65464473
chr14	66668905	66776521
chr14	66681106	66688386
chr14	66709356	66776521
chr14	66813218	66824566
chr14	67182864	67198320
chr14	67254203	67279537
chr14	67569216	67569582
chr14	67592314	67592718
chr14	67653436	67663190
chr14	67656415	67663190
chr14	67821277	67887204
chr14	67864787	67887204
chr14	68387112	68468250
chr14	69089947	69122360
chr14	69115667	69122360
chr14	69424093	69425578
chr14	70976941	70995843
chr14	70988566	70995843
chr14	70988569	70995925
chr14	71050651	71060642
chr14	71418129	71589370
chr14	71467046	71529370
chr14	71508379	71529370
chr14	72771732	72845651
chr14	72937970	72959028
chr14	72958611	73058705

chr14	73064971	73077536
chr14	73272494	73274459
chr14	73355625	73357395
chr14	73650008	73671597
chr14	73729639	73730179
chr14	73892026	73910417
chr14	73892151	73896534
chr14	73931502	73932112
chr14	73931502	73932943
chr14	74004604	74015930
chr14	74514605	74516941
chr14	74683295	74701774
chr14	74829212	74835908
chr14	75018828	75048632
chr14	75030542	75046589
chr14	75100228	75103997
chr14	75622078	75646627
chr14	75663085	75683656
chr14	75771733	75803253
chr14	75782486	75803253
chr14	75792912	75820161
chr14	75792915	75823937
chr14	76154743	76172019
chr14	76154743	76195972
chr14	77427371	77454102
chr14	77512156	77518167
chr14	77673559	77694900
chr14	77899099	77902748
chr14	80421424	80530886
chr14	80497468	80526982
chr14	80497468	80530886
chr14	80726098	80743267
chr14	80743074	80745001
chr14	80830151	80840768
chr14	80836391	80840768
chr14	80862756	80907650
chr14	80878852	80906081
chr14	80904812	80924346
chr14	86028368	86070967
chr14	88416710	88438146
chr14	88626837	88644511
chr14	88685014	88688359
chr14	89908711	89932627
chr14	89924512	89932627
chr14	89931540	89934707

chr14	89983113	89985210
chr14	90019315	90023111
chr14	90541197	90578308
chr14	90570592	90578308
chr14	90687941	90689712
chr14	90718500	90786328
chr14	90729629	90730196
chr14	90943077	91041893
chr14	90978305	91047039
chr14	92116101	92117642
chr14	92124290	92138347
chr14	93274073	93296157
chr14	94060645	94060912
chr14	96341577	96347341
chr14	96799636	96860735
chr14	96814212	96860735
chr14	96828172	96860735
chr14	96833466	96945382
chr14	99231344	99238511
chr14	99457555	99458535
chr14	99458278	99466055
chr14	99493464	99503644
chr14	99850123	99851035
chr14	99850123	99865646
chr15	100132006	100134621
chr15	100327375	100331054
chr15	100330888	100334180
chr15	100914700	100924729
chr15	101151719	101178980
chr15	22523346	22539247
chr15	22995098	23003153
chr15	23008644	23010133
chr15	23008698	23019380
chr15	24967028	24968082
chr15	24967931	24968529
chr15	24976304	24982437
chr15	25393745	25411971
chr15	25405460	25415842
chr15	25405460	25429680
chr15	28259694	28265974
chr15	28292887	28312874
chr15	28751487	28754897
chr15	28995752	29028364
chr15	28995756	29101784
chr15	29704161	29705737

chr15	29759890	29773357
chr15	30635079	30641617
chr15	30635486	30641617
chr15	30638745	30716342
chr15	30658765	30716342
chr15	30658765	30750556
chr15	30716219	30721271
chr15	30716219	30769064
chr15	30745021	30750556
chr15	30745177	30750556
chr15	30748575	30769064
chr15	31685344	31689481
chr15	32523028	32524600
chr15	32625486	32643818
chr15	32633932	32636408
chr15	32643585	32644359
chr15	32857014	33018931
chr15	32926173	33018927
chr15	34003031	34005851
chr15	34003031	34023497
chr15	34084208	34088133
chr15	34163559	34177999
chr15	34882501	34883282
chr15	34963659	34964290
chr15	35450684	35484507
chr15	35450684	35490197
chr15	38283496	38299547
chr15	39588396	39741900
chr15	40628069	40650819
chr15	40628208	40628588
chr15	40629272	40642972
chr15	40645655	40650819
chr15	40676071	40706294
chr15	40706176	40731344
chr15	40709024	40731344
chr15	40709024	40734704
chr15	40729504	40734704
chr15	40731054	40731344
chr15	40736498	40737726
chr15	40854689	40855027
chr15	40902615	40903831
chr15	41517515	41518182
chr15	41526897	41533463
chr15	41668787	41669958
chr15	41668827	41675721

chr15	41740056	41754567
chr15	41750380	41761850
chr15	42198464	42199961
chr15	42338388	42354453
chr15	42716676	42719566
chr15	42794643	42908467
chr15	42829937	42855200
chr15	42855117	42878684
chr15	42871444	42878684
chr15	42878548	42893468
chr15	43333270	43351041
chr15	43361161	43361941
chr15	43828216	43842516
chr15	44337762	44339481
chr15	44413335	44414615
chr15	44458990	44464848
chr15	44497026	44503850
chr15	44560248	44563225
chr15	44604802	44613536
chr15	44648865	44657263
chr15	44736482	44756463
chr15	48331479	48332196
chr15	48596282	48717169
chr15	49225204	49239367
chr15	49225204	49292537
chr15	49225204	49319805
chr15	49235850	49308777
chr15	49235850	49328130
chr15	49292326	49308777
chr15	49489168	49508348
chr15	50432337	50439177
chr15	50432337	50459162
chr15	50634381	50725323
chr15	50643339	50675566
chr15	50662966	50675566
chr15	50949878	50999262
chr15	51533655	51538452
chr15	52023259	52047015
chr15	52024582	52047015
chr15	55223888	55389433
chr15	55915283	55916911
chr15	56142777	56181554
chr15	56142777	56243287
chr15	56631437	56635250
chr15	56887805	56890167

chr15	57091788	57095163
chr15	58698279	58717727
chr15	58771958	58831916
chr15	58879979	58883786
chr15	58886974	58887620
chr15	58899468	58899709
chr15	58916934	58933582
chr15	59030803	59095030
chr15	59099993	59123627
chr15	59272305	59275196
chr15	60436142	60436713
chr15	62007307	62014013
chr15	63526919	63537156
chr15	63528628	63537156
chr15	63530497	63537156
chr15	63532646	63543165
chr15	63542054	63563008
chr15	63588828	63594786
chr15	63745726	63747858
chr15	64156161	64156724
chr15	64156184	64156724
chr15	64203081	64353806
chr15	64495839	64500166
chr15	64499292	64500166
chr15	64622826	64626632
chr15	64678115	64681900
chr15	64818369	64819116
chr15	64859893	64865228
chr15	65118671	65119954
chr15	65579354	65591731
chr15	66119085	66135090
chr15	66308708	66487292
chr15	66331409	66352955
chr15	66435026	66435259
chr15	66499555	66503529
chr15	66499559	66502750
chr15	66708498	66716498
chr15	67226900	67236820
chr15	67231229	67231897
chr15	67231229	67236820
chr15	67830190	67833942
chr15	68645159	68652019
chr15	70656929	70664814
chr15	70656963	70660216
chr15	70656963	70664814

chr15	71133253	71154932
chr15	71411686	71542465
chr15	71601958	71660734
chr15	71660529	71682137
chr15	71991102	72010450
chr15	72199780	72200473
chr15	72209672	72223524
chr15	72241900	72271104
chr15	72488782	72518134
chr15	72496790	72544964
chr15	72524905	72561549
chr15	72760406	72762598
chr15	72760406	72780322
chr15	72774871	72780322
chr15	73558305	73573795
chr15	74640465	74645004
chr15	74918756	74926887
chr15	75364487	75364862
chr15	75400729	75436443
chr15	75422646	75437580
chr15	75597339	75598681
chr15	75598278	75598681
chr15	75598278	75621056
chr15	75600636	75601218
chr15	75601137	75621002
chr15	76214524	76231398
chr15	76274411	76297348
chr15	76434077	76567405
chr15	76504858	76541725
chr15	76567269	76574284
chr15	76728408	76728737
chr15	76728408	76733384
chr15	76816424	76841931
chr15	76850734	76862533
chr15	77046775	77056255
chr15	77046788	77056255
chr15	77239781	77286504
chr15	77252366	77284030
chr15	77252366	77357099
chr15	77252366	77365225
chr15	77252366	77412774
chr15	77283882	77286504
chr15	77284957	77346611
chr15	77284957	77412774
chr15	77477554	77517356

chr15	78008679	78009114
chr15	78155212	78169081
chr15	78157547	78169077
chr15	78289618	78290556
chr15	78289618	78290575
chr15	78289618	78290583
chr15	78593091	78593628
chr15	78593091	78598537
chr15	78593091	78601387
chr15	79456097	79469145
chr15	79843858	79845442
chr15	79884857	79889354
chr15	79889092	79899125
chr15	80158059	80197219
chr15	80970461	80982182
chr15	80978765	80982182
chr15	81079884	81143820
chr15	81127589	81259880
chr15	82151464	82215668
chr15	82400632	82408367
chr15	82400659	82414804
chr15	82404611	82414804
chr15	82404611	82415675
chr15	82414600	82420075
chr15	82627192	82628550
chr15	82627192	82641682
chr15	82849688	82854800
chr15	82852141	82892801
chr15	83151214	83192308
chr15	83151214	83194460
chr15	84523199	84555070
chr15	84526780	84555070
chr15	84542144	84544649
chr15	84637346	84641262
chr15	84642194	84643723
chr15	84755661	84764815
chr15	85474755	85521575
chr15	85653270	85710645
chr15	88464912	88465586
chr15	89245606	89260843
chr15	89245755	89260843
chr15	89835461	89837722
chr15	89852360	89877424
chr15	89860922	89889140
chr15	89865494	89889140

chr15	90593635	90593939
chr15	90614509	90641257
chr15	90713166	90715042
chr15	90948283	90948627
chr15	90959899	90961774
chr15	92950320	92956649
chr15	96118842	96132062
chr15	96118842	96191394
chr15	96285296	96290783
chr15	96334075	96337535
chr15	99215620	99228732
chr15	99256031	99287951
chr15	99256031	99352471
chr15	99598429	99602109
chr15	99645560	99651688
chr16	10387143	10472183
chr16	10515062	10547677
chr16	11020192	11033943
chr16	11841694	11849672
chr16	11846500	11849385
chr16	11886470	11894148
chr16	11894953	11905831
chr16	12042846	12129758
chr16	13235029	13237321
chr16	1455816	1456112
chr16	14590489	14604226
chr16	1499192	1500488
chr16	15034553	15035426
chr16	15047417	15048099
chr16	15483936	15515428
chr16	15498065	15515428
chr16	15513283	15515428
chr16	15532986	15583238
chr16	15695907	15696860
chr16	16016495	16016917
chr16	16141172	16142942
chr16	1614638	1632365
chr16	1614638	1652581
chr16	1743331	1748720
chr16	1809237	1809833
chr16	18543616	18558854
chr16	18547227	18558854
chr16	18782780	18783102
chr16	18782780	18784823
chr16	18782780	18788142

chr16	18782780	18789118
chr16	18889371	18900044
chr16	19071927	19074097
chr16	192225	193015
chr16	1962930	1963148
chr16	1962932	1963148
chr16	19706591	19711440
chr16	19732828	19733827
chr16	2002753	2002990
chr16	2020312	2020506
chr16	2055979	2058873
chr16	20860047	20862643
chr16	2097147	2098025
chr16	2111926	2112339
chr16	21315883	21317126
chr16	21338920	21352138
chr16	21611672	21625115
chr16	21961327	21976243
chr16	220648	229498
chr16	2246094	2246986
chr16	2253303	2254063
chr16	23102318	23120246
chr16	2317439	2317647
chr16	23388985	23417121
chr16	23523053	23532765
chr16	23544513	23554635
chr16	23581053	23587319
chr16	23603586	23608012
chr16	23621361	23629959
chr16	2448978	2449251
chr16	24540611	24553557
chr16	24750725	24758599
chr16	24776932	24778468
chr16	24910305	24911494
chr16	24927683	24939597
chr16	25151553	25158958
chr16	25252945	25255384
chr16	2613959	2697997
chr16	27211145	27215008
chr16	27334315	27340273
chr16	2756333	2895320
chr16	27627845	27628830
chr16	28169456	28169909
chr16	288275	289439
chr16	28871779	28873579

chr16	2896351	2899231
chr16	28963704	28964039
chr16	2937384	2938162
chr16	2948687	2951125
chr16	296176	298251
chr16	29798747	29798950
chr16	29831073	29833699
chr16	29871589	29873729
chr16	29904851	29905959
chr16	29904851	29906127
chr16	29905787	29906127
chr16	29963106	29972140
chr16	29983242	29985578
chr16	30365288	30365494
chr16	3057032	3058272
chr16	30666990	30667296
chr16	31061609	31062280
chr16	31061609	31064481
chr16	31090774	31091342
chr16	31473440	31473838
chr16	31718912	31723353
chr16	346147	347101
chr16	3674514	3675323
chr16	3850296	3854850
chr16	425650	489000
chr16	4332214	4333519
chr16	4332214	4335393
chr16	4446885	4455780
chr16	4450399	4455780
chr16	4466152	4469698
chr16	4634722	4657363
chr16	46674313	46681520
chr16	46897647	46900883
chr16	47109482	47134410
chr16	47122656	47134410
chr16	47128324	47134410
chr16	47129223	47134410
chr16	47365787	47429957
chr16	47439842	47459175
chr16	47463898	47499894
chr16	47463898	47515601
chr16	47474358	47547548
chr16	47497398	47550205
chr16	47497398	47562495
chr16	4751298	4766076

chr16	48252224	48262872
chr16	48252224	48270274
chr16	48252224	48303305
chr16	48252224	48334358
chr16	482524	491268
chr16	48256643	48303305
chr16	48275086	48303305
chr16	48334215	48336498
chr16	48343801	48348290
chr16	48355337	48362430
chr16	48553538	48570292
chr16	48560753	48570292
chr16	50786838	50793664
chr16	53154446	53157541
chr16	53245421	53263097
chr16	53245766	53285699
chr16	53307680	53319951
chr16	53442657	53490301
chr16	53764161	53844298
chr16	53810139	53826456
chr16	53825863	53838205
chr16	53844154	53845086
chr16	53873785	53919764
chr16	53933984	53937294
chr16	53933984	53937441
chr16	53933984	53937833
chr16	53933984	53954508
chr16	56385918	56415884
chr16	56454773	56467293
chr16	56484760	56501025
chr16	56497098	56497742
chr16	56837607	56839035
chr16	57172223	57172874
chr16	57431306	57432486
chr16	58403097	58405110
chr16	58504009	58541620
chr16	58559803	58560362
chr16	58708078	58709567
chr16	66604818	66609530
chr16	66730111	66732505
chr16	66939226	66942737
chr16	67046151	67066798
chr16	67063627	67066798
chr16	67066681	67073822
chr16	67066681	67079782

chr16	67066681	67099799
chr16	67144833	67147558
chr16	67174161	67175238
chr16	67200968	67201336
chr16	67255838	67256689
chr16	67685456	67699341
chr16	67685456	67705094
chr16	67744287	67759732
chr16	67939885	67940478
chr16	68093355	68126610
chr16	68157958	68183366
chr16	68175999	68191775
chr16	68249289	68252653
chr16	68271333	68275249
chr16	68287322	68287871
chr16	68287322	68290540
chr16	68287745	68297476
chr16	68287745	68316074
chr16	68557997	68579938
chr16	69105684	69110031
chr16	69133511	69139914
chr16	69148993	69150904
chr16	69155870	69168998
chr16	69235747	69245815
chr16	69235747	69260260
chr16	69235747	69284244
chr16	69260170	69284244
chr16	69288292	69299774
chr16	69311405	69318711
chr16	69328637	69332882
chr16	69586209	69626528
chr16	69936452	69937076
chr16	70036431	70039100
chr16	70213656	70222480
chr16	70213660	70222480
chr16	70219870	70222480
chr16	70269783	70270195
chr16	70317495	70356247
chr16	70356111	70361636
chr16	70356111	70372777
chr16	70379170	70381862
chr16	70677791	70678368
chr16	70780789	70789052
chr16	71662697	71664099
chr16	71676446	71677404

chr16	71702245	71702731
chr16	71748407	71749893
chr16	71789278	71798970
chr16	71894720	71895589
chr16	72173922	72349206
chr16	72193276	72349206
chr16	72284571	72308188
chr16	72308055	72368722
chr16	72368652	72391215
chr16	72391070	72523415
chr16	72523331	72617553
chr16	72554313	72555326
chr16	72555264	72555946
chr16	72555264	72617553
chr16	72570434	72617553
chr16	736421	737185
chr16	74348402	74350738
chr16	74451054	74459789
chr16	74451054	74463479
chr16	74459819	74462093
chr16	74660931	74664745
chr16	74923925	74949947
chr16	75293909	75305182
chr16	75362874	75395209
chr16	75385649	75395209
chr16	75600219	75622464
chr16	75627835	75634292
chr16	81041355	81045141
chr16	81319368	81365478
chr16	81351582	81377702
chr16	81377218	81377702
chr16	84124658	84130171
chr16	84135543	84140262
chr16	84661577	84663165
chr16	84740308	84745826
chr16	86485631	86491004
chr16	869962	879737
chr16	87308532	87311296
chr16	87748672	87762036
chr16	87755193	87762150
chr16	87975047	87987904
chr16	88004294	88008187
chr16	88610441	88611536
chr16	88722420	88722582
chr16	89098484	89133262

chr16	89098576	89102759
chr16	89102603	89117086
chr16	89305205	89427069
chr16	89361494	89418368
chr16	89418283	89422305
chr16	89418283	89427069
chr16	89426766	89431326
chr16	89560712	89560916
chr16	89978809	89979448
chr16	9102983	9117064
chr16	910930	913353
chr16	934189	954666
chr17	1050049	1102027
chr17	1112118	1125367
chr17	12066156	12095694
chr17	12820485	12896511
chr17	14033551	14034416
chr17	15503097	15547148
chr17	15677175	15677721
chr17	15677175	15683157
chr17	15977962	15987368
chr17	16034764	16036719
chr17	16194461	16198719
chr17	16228872	16234070
chr17	16325799	16326905
chr17	17070879	17078076
chr17	17078010	17098193
chr17	17261599	17264981
chr17	17683545	17724159
chr17	1781214	1787604
chr17	18206814	18208375
chr17	18273243	18278357
chr17	1842802	1846641
chr17	18519481	18521145
chr17	18680854	18681614
chr17	19274050	19274318
chr17	19274050	19274430
chr17	19276396	19283714
chr17	19279861	19283714
chr17	19306092	19329647
chr17	19773167	19776407
chr17	19919154	19924517
chr17	20017986	20110562
chr17	20204332	20205936
chr17	20204332	20213240

chr17	20296960	20314223
chr17	21002444	21028674
chr17	2163759	2172859
chr17	2328975	2329475
chr17	2362988	2364376
chr17	2372488	2372926
chr17	2379035	2381162
chr17	238380	247285
chr17	2414394	2415954
chr17	2414394	2420596
chr17	2414394	2420904
chr17	2438108	2470220
chr17	247110	258418
chr17	2617196	2638320
chr17	2672757	2674031
chr17	2700845	2701139
chr17	2700854	2701139
chr17	27301805	27303635
chr17	27303512	27311616
chr17	28122602	28132809
chr17	28122602	28196288
chr17	28172516	28205124
chr17	28348018	28355876
chr17	28591697	28592477
chr17	28598509	28599830
chr17	28612796	28613077
chr17	28649188	28655483
chr17	28921687	28944569
chr17	2905283	2909070
chr17	29451454	29542672
chr17	29451486	29482288
chr17	30384988	30390159
chr17	30495425	30520021
chr17	30607551	30648456
chr17	30769000	30780739
chr17	31349119	31374731
chr17	31988111	31988497
chr17	31993887	31995762
chr17	32020696	32031458
chr17	32362913	32369745
chr17	32367771	32377993
chr17	34951949	35017606
chr17	34983231	34986131
chr17	35026373	35036518
chr17	35063575	35066466

chr17	35846905	35850048
chr17	3605210	3608565
chr17	36492731	36496406
chr17	36507162	36507398
chr17	36508896	36511455
chr17	37568790	37579434
chr17	37576340	37579434
chr17	38359962	38377842
chr17	38361710	38400855
chr17	38806764	38810400
chr17	39708329	39710481
chr17	40026355	40026646
chr17	40100063	40100300
chr17	40308951	40309286
chr17	4064360	4065008
chr17	41689069	41689401
chr17	41874002	41878102
chr17	41907598	41908988
chr17	4197372	4249248
chr17	41976792	41977260
chr17	41987594	41990382
chr17	41994869	42003557
chr17	41996310	42003557
chr17	42000481	42003557
chr17	4206320	4249248
chr17	4216982	4249248
chr17	42322281	42348520
chr17	42613646	42663503
chr17	42727661	42730918
chr17	4282797	4296809
chr17	4289229	4296809
chr17	4289229	4303745
chr17	4296716	4303745
chr17	43045661	43063951
chr17	43045661	43067695
chr17	44075087	44075287
chr17	44154555	44155441
chr17	44194516	44198130
chr17	44464471	44493720
chr17	44681948	44683959
chr17	44797562	44807469
chr17	44804587	44807469
chr17	44805253	44807469
chr17	45024372	45034999
chr17	45033934	45034390

chr17	4541913	4542463
chr17	4541916	4542463
chr17	45437823	45450763
chr17	46048021	46094701
chr17	46050532	46169605
chr17	46066536	46120049
chr17	46066536	46167564
chr17	46082440	46120049
chr17	46082440	46140444
chr17	46082440	46162525
chr17	46094559	46130753
chr17	46094559	46162525
chr17	46094559	46169605
chr17	46156773	46172232
chr17	46167479	46172232
chr17	46169530	46172232
chr17	46170854	46172106
chr17	46170854	46177272
chr17	47169916	47172013
chr17	47453326	47491192
chr17	47696078	47699697
chr17	48112030	48121565
chr17	48848063	48866301
chr17	4894633	4895048
chr17	49041377	49042388
chr17	49298105	49317983
chr17	49311311	49322508
chr17	49312697	49317499
chr17	49316825	49322508
chr17	50139604	50140092
chr17	50551679	50551945
chr17	50749218	50750694
chr17	51041500	51052891
chr17	51041500	51070258
chr17	51047374	51068012
chr17	5297344	5308822
chr17	5297344	5346925
chr17	5297355	5308822
chr17	5344843	5346925
chr17	54912665	54942491
chr17	55031191	55081183
chr17	55315226	55320851
chr17	56847921	56856665
chr17	56848695	56856665
chr17	57978077	57979313

chr17	58003650	58006527
chr17	58087081	58089421
chr17	58705877	58709990
chr17	58720745	58730307
chr17	58810882	58972925
chr17	58999380	59049257
chr17	59070822	59084037
chr17	59104003	59104394
chr17	59169587	59169880
chr17	59257121	59275970
chr17	59696118	59697978
chr17	59731420	59773944
chr17	59764970	59773944
chr17	59764970	59792686
chr17	59838294	59840088
chr17	59935192	59937774
chr17	60226038	60226879
chr17	60345480	60377292
chr17	60345480	60380399
chr17	60500398	60509375
chr17	60500398	60517923
chr17	60630354	60648082
chr17	60656598	60657042
chr17	60679452	60709552
chr17	60980557	60990235
chr17	61456487	61457631
chr17	61758673	61793729
chr17	61776400	61808548
chr17	62029540	62063222
chr17	62029850	62063222
chr17	62052536	62063222
chr17	623532	632841
chr17	62680453	62680777
chr17	63009536	63030089
chr17	63028003	63074014
chr17	63151269	63200957
chr17	63232514	63267873
chr17	63576963	63579943
chr17	63799588	63806151
chr17	63805043	63809659
chr17	64542943	64578576
chr17	64591083	64594104
chr17	64821766	64824872
chr17	6525355	6590630
chr17	655837	657549

chr17	65851803	65888152
chr17	66096250	66139600
chr17	66468590	66496283
chr17	66472504	66496283
chr17	66495571	66496283
chr17	66496200	66514466
chr17	67151045	67185145
chr17	67166477	67173083
chr17	67891846	67894165
chr17	68306942	68359753
chr17	68401359	68420506
chr17	69273942	69284072
chr17	697417	701857
chr17	72736649	72738902
chr17	72841757	72849804
chr17	72849104	72849804
chr17	73203036	73203793
chr17	744435	748032
chr17	75262867	75263321
chr17	75651185	75666620
chr17	75671815	75691535
chr17	7572864	7574227
chr17	75791759	75797397
chr17	76001541	76001846
chr17	76385450	76387437
chr17	76385456	76385654
chr17	76487711	76488132
chr17	76558373	76559043
chr17	76738395	76776541
chr17	78004169	78031842
chr17	78004169	78051448
chr17	78031515	78036410
chr17	78190969	78192785
chr17	78400676	78401106
chr17	78400734	78401057
chr17	78414878	78424181
chr17	78414878	78424192
chr17	78414951	78419725
chr17	787964	792509
chr17	7936817	7937127
chr17	80135312	80140140
chr17	80470526	80473444
chr17	80619541	80643810
chr17	80619626	80643810
chr17	80619626	80707999

chr17	80640442	80643810
chr17	80707444	80707999
chr17	80707840	80709114
chr17	80707840	80710103
chr17	80707840	80755040
chr17	80730559	80732125
chr17	81194995	81195834
chr17	81270524	81271024
chr17	81588932	81596242
chr17	81588932	81597316
chr17	81667728	81672768
chr17	81667728	81672802
chr17	81696356	81701717
chr17	81696823	81701717
chr17	81700466	81701717
chr17	81889993	81890688
chr17	81909055	81909513
chr17	8204849	8206888
chr17	8206490	8207901
chr17	82082696	82082913
chr17	82127691	82134194
chr17	82166389	82172860
chr17	82183773	82185303
chr17	82265685	82266894
chr17	82265685	82268760
chr17	82394330	82407771
chr17	82403936	82409730
chr17	82449310	82450699
chr17	8253879	8254301
chr17	82655528	82659657
chr17	82718905	82720841
chr17	82720271	82720841
chr17	82756164	82783714
chr17	82763964	82783714
chr17	82781588	82783714
chr17	82876780	82889697
chr17	8553954	8576831
chr17	9162812	9164772
chr17	9376576	9378653
chr17	9378551	9391284
chr18	12028042	12031098
chr18	12506476	12563727
chr18	12785735	12817365
chr18	12794383	12862744
chr18	12801969	12819091

chr18	12814202	12840950
chr18	12999420	13002681
chr18	12999424	13008631
chr18	1358725	1361221
chr18	1358725	1368597
chr18	13666478	13682105
chr18	14077328	14085076
chr18	163307	167794
chr18	21536041	21540700
chr18	21539919	21540700
chr18	21622724	21631378
chr18	21761441	21779685
chr18	21765771	21782763
chr18	21791334	21804014
chr18	23775753	23784157
chr18	24019859	24083081
chr18	24277789	24377535
chr18	26018018	26078160
chr18	26018018	26087577
chr18	26389844	26394481
chr18	26476514	26501245
chr18	31637441	31666372
chr18	31830870	31839457
chr18	31870356	31890866
chr18	31931328	31936456
chr18	31931328	31942893
chr18	32108866	32113860
chr18	32111753	32118352
chr18	32111753	32129327
chr18	32111753	32135975
chr18	3253232	3255962
chr18	35654559	35692320
chr18	35654559	35703643
chr18	36142843	36159830
chr18	36334396	36355645
chr18	36353568	36355645
chr18	36353568	36372744
chr18	36355538	36412014
chr18	36411898	36512543
chr18	36834202	36844302
chr18	36834202	36959435
chr18	36954430	36959435
chr18	36959315	37067545
chr18	37066889	37084232
chr18	37084124	37173081

chr18	37160176	37173081
chr18	41351660	41501276
chr18	41437370	41501276
chr18	41501119	41525526
chr18	41516419	41525526
chr18	41955256	41962632
chr18	46118180	46128837
chr18	46118180	46129213
chr18	46730205	46821072
chr18	46775489	46821072
chr18	46890579	46903635
chr18	46946056	46946923
chr18	47154798	47157537
chr18	47829473	47870564
chr18	47841517	47896809
chr18	47841705	47896809
chr18	47841791	47896809
chr18	47865058	47930446
chr18	47868051	47870564
chr18	47889020	47896809
chr18	48857587	48860429
chr18	49249180	49258493
chr18	49257009	49286613
chr18	49272177	49286613
chr18	49329572	49333853
chr18	49329572	49379758
chr18	49417842	49430447
chr18	49491621	49491833
chr18	49491625	49491833
chr18	50382128	50392208
chr18	51175894	51177576
chr18	54166075	54187837
chr18	54187713	54189011
chr18	54187713	54205157
chr18	54271359	54277853
chr18	54277771	54287411
chr18	54287280	54293915
chr18	56672496	56673353
chr18	56778439	56816144
chr18	56918071	56939393
chr18	57731626	57731832
chr18	57731626	57761939
chr18	57731626	57765361
chr18	59466738	59471769
chr18	6058537	6080951

chr18	61814606	61816598
chr18	61816033	61816598
chr18	6213148	6356783
chr18	62282261	62287467
chr18	62348112	62349937
chr18	63350887	63386623
chr18	68823365	68874785
chr18	68836748	68897682
chr18	68897218	68897682
chr18	69010933	69054133
chr18	705256	706578
chr18	724312	756835
chr18	74155144	74159912
chr18	756556	787986
chr18	76830675	76851939
chr18	76849525	76855201
chr18	76849621	76875664
chr18	76862185	76875664
chr18	7754372	7774271
chr18	79933968	79935598
chr18	8624937	8638416
chr18	8714138	8720496
chr18	8718423	8741134
chr18	9158346	9221999
chr18	9182026	9221999
chr18	9208656	9239556
chr18	9583116	9595238
chr18	9924259	9931962
chr18	9927095	9937066
chr18	9927095	9950568
chr18	9931809	9954160
chr18	9936109	9938341
chr19	10135438	10136287
chr19	10155079	10155852
chr19	10159657	10179982
chr19	10224011	10224947
chr19	1083290	1084346
chr19	10869217	10869663
chr19	1089570	1089883
chr19	10984123	10987924
chr19	10984373	10985242
chr19	11056216	11060187
chr19	11116858	11131795
chr19	11123173	11131795
chr19	11147817	11148113

chr19	1116947	1117322
chr19	11415946	11417059
chr19	11682545	11685260
chr19	11722953	11725327
chr19	11913408	11915672
chr19	12043639	12045209
chr19	12075311	12077637
chr19	12110304	12111719
chr19	12110304	12147834
chr19	12111245	12111719
chr19	12145257	12146224
chr19	12162661	12163206
chr19	1216267	1219413
chr19	12185784	12188058
chr19	12194705	12195143
chr19	12349612	12352070
chr19	12349612	12353114
chr19	12391802	12466850
chr19	12431576	12433197
chr19	12517744	12528696
chr19	12526919	12528696
chr19	12703713	12705797
chr19	12714820	12715710
chr19	12813082	12813565
chr19	12876179	12878579
chr19	12928337	12928847
chr19	12928341	12928932
chr19	12928341	12928936
chr19	12954139	12954526
chr19	13090298	13095729
chr19	13094634	13095729
chr19	13104891	13105606
chr19	13817169	13819492
chr19	13993524	13998317
chr19	14177530	14189689
chr19	1510149	1510917
chr19	15272814	15275842
chr19	15272814	15297942
chr19	16080914	16082046
chr19	16081912	16101409
chr19	16403732	16404714
chr19	16509149	16509988
chr19	16528079	16529753
chr19	16647119	16654125
chr19	16652604	16654125

chr19	17058106	17062779
chr19	17276494	17277479
chr19	17557322	17559421
chr19	17733192	17734416
chr19	18564868	18565190
chr19	18567131	18567584
chr19	18574869	18575141
chr19	19007346	19008428
chr19	19070342	19106385
chr19	1918888	1919442
chr19	19316617	19320462
chr19	19436130	19465614
chr19	19456921	19465614
chr19	19457006	19465614
chr19	19465339	19474118
chr19	19474032	19492712
chr19	19585199	19599365
chr19	19588085	19599365
chr19	19592505	19599365
chr19	19677863	19680007
chr19	19677863	19680187
chr19	19915279	19933302
chr19	19916559	19933302
chr19	20934948	20937361
chr19	21062605	21098910
chr19	2109228	2109872
chr19	2109253	2109872
chr19	23932342	23943589
chr19	24105939	24108043
chr19	29980158	29986417
chr19	29980158	30012531
chr19	31276331	31279752
chr19	3206261	3207747
chr19	32382466	32386721
chr19	32432680	32439910
chr19	32709184	32711905
chr19	32878127	32882013
chr19	32878139	32879378
chr19	32878143	32882013
chr19	32879067	32882013
chr19	32891954	32899998
chr19	32973183	32976678
chr19	33002424	33002812
chr19	33798263	33798766
chr19	33798263	33811552

chr19	34178221	34209051
chr19	34178221	34219877
chr19	34446043	34467014
chr19	35270758	35279074
chr19	35729296	35729945
chr19	35737263	35737599
chr19	3620294	3620706
chr19	3620296	3620706
chr19	36449230	36450001
chr19	36449398	36450001
chr19	36449398	36476616
chr19	36823730	36827849
chr19	37089038	37096115
chr19	37347189	37359546
chr19	37425867	37429808
chr19	37580728	37586745
chr19	37738147	37740194
chr19	37738902	37740194
chr19	37738902	37759139
chr19	37760189	37771696
chr19	38002458	38029156
chr19	38008095	38029156
chr19	38028269	38029156
chr19	38697027	38701121
chr19	38697027	38709476
chr19	38845616	38846090
chr19	38845628	38847434
chr19	39169757	39172916
chr19	39354005	39357409
chr19	39453355	39454325
chr19	3979454	3979807
chr19	3983313	3984135
chr19	40048669	40049056
chr19	40068672	40081914
chr19	40197876	40198555
chr19	41220858	41221107
chr19	41221615	41256611
chr19	41243274	41243707
chr19	41248513	41248839
chr19	41248513	41248948
chr19	41276158	41276525
chr19	43186341	43203973
chr19	43506722	43508060
chr19	43560909	43561544
chr19	43655418	43656640

chr19	43656478	43658597
chr19	4365657	4366500
chr19	43880009	43884051
chr19	43924237	43931365
chr19	43925324	43930000
chr19	44007010	44010397
chr19	44076321	44085803
chr19	4408902	4410523
chr19	44264313	44289704
chr19	44429057	44432366
chr19	44657761	44662398
chr19	45011935	45012276
chr19	45686533	45687727
chr19	45686533	45688566
chr19	45702903	45703294
chr19	45791121	45792315
chr19	45821531	45822755
chr19	46375612	46376574
chr19	46605826	46608140
chr19	46838053	46846142
chr19	46845992	46846824
chr19	46918487	46989675
chr19	46937263	46938154
chr19	47270406	47273406
chr19	47492145	47492960
chr19	47670442	47757148
chr19	47746966	47757148
chr19	48211504	48215384
chr19	48231659	48242644
chr19	48831427	48831759
chr19	48918784	48919004
chr19	48999318	48999833
chr19	49135778	49136122
chr19	49341020	49360081
chr19	49341335	49360081
chr19	49496471	49497168
chr19	49678065	49686726
chr19	49844315	49844598
chr19	49864438	49865126
chr19	50061799	50067800
chr19	50216184	50217771
chr19	5047475	5050463
chr19	51573209	51581882
chr19	51811472	51886912
chr19	51839892	51880877

chr19	51839892	51886912
chr19	51948204	51949235
chr19	51948592	51949235
chr19	51965512	51966214
chr19	52021543	52025961
chr19	52123922	52125781
chr19	52282110	52290167
chr19	52408225	52435209
chr19	52574076	52584197
chr19	52603422	52635153
chr19	52652357	52660843
chr19	52655560	52670951
chr19	52728729	52735276
chr19	53404473	53486758
chr19	53407697	53409438
chr19	53446226	53460952
chr19	53521855	53522684
chr19	53521855	53555908
chr19	54153840	54155286
chr19	54207397	54207872
chr19	55404060	55404478
chr19	55613592	55617470
chr19	55663605	55675435
chr19	56190836	56222326
chr19	56449556	56462429
chr19	56494162	56500247
chr19	5711420	5712002
chr19	5715971	5729944
chr19	57543264	57543650
chr19	57619411	57621493
chr19	57776542	57780146
chr19	57778750	57780146
chr19	57868457	57877042
chr19	5787378	5787588
chr19	5787380	5787588
chr19	57876915	57889008
chr19	57876915	57922500
chr19	5790364	5791043
chr19	57923191	57930557
chr19	57925622	57930557
chr19	58142213	58142661
chr19	58260850	58263271
chr19	58294097	58295581
chr19	58556403	58556686
chr19	5950716	5951596

chr19	6230379	6270759
chr19	6230594	6270759
chr19	6258327	6262310
chr19	7082877	7084277
chr19	7440188	7447168
chr19	7627229	7628298
chr19	8337794	8339167
chr19	8368043	8369332
chr19	8455404	8466448
chr19	852875	861956
chr19	897508	902130
chr19	9156748	9157241
chr19	9337795	9343784
chr19	9338493	9343784
chr19	9341101	9343784
chr19	9350803	9364948
chr19	9566088	9568175
chr19	9566335	9568175
chr19	9566335	9569082
chr19	9632227	9645189
chr19	9652952	9653881
chr19	9652952	9656653
chr19	9652952	9660874
chr19	9658224	9693820
chr1	100007033	100043229
chr1	100007033	100050004
chr1	100043072	100061949
chr1	10007569	10008379
chr1	100121377	100123977
chr1	100140844	100144143
chr1	100283628	100285322
chr1	100888752	100895528
chr1	10232249	10235016
chr1	10320042	10376676
chr1	10360928	10381641
chr1	10433843	10442441
chr1	1053977	1054420
chr1	10597591	10599366
chr1	108718788	108798237
chr1	108882475	108885578
chr1	108914337	108929975
chr1	108947337	108950448
chr1	108947610	108962469
chr1	109236804	109237373
chr1	109281288	109281618

chr1	109344165	109345878
chr1	11013715	11025208
chr1	11107404	11130777
chr1	111147641	111153371
chr1	111147641	111184280
chr1	11121094	11122126
chr1	111454210	111468259
chr1	1116059	1267992
chr1	111727366	111727804
chr1	112411703	112416264
chr1	112596835	112600848
chr1	112616812	112618369
chr1	112690098	112691622
chr1	112912178	112960575
chr1	112913873	112929352
chr1	112921989	112939491
chr1	113093205	113103785
chr1	113544664	113622994
chr1	113622920	113646642
chr1	113705889	113758733
chr1	113710253	113747014
chr1	113724758	113758733
chr1	114456212	114463556
chr1	114461053	114464388
chr1	114463103	114477087
chr1	114726210	114749904
chr1	117086416	117086781
chr1	117396951	117405645
chr1	117402185	117442335
chr1	117402185	117467772
chr1	117456084	117466427
chr1	117460488	117522937
chr1	117493146	117522937
chr1	117870395	117920424
chr1	119026814	119045662
chr1	119178643	119181461
chr1	121280749	121284995
chr1	12299201	12308604
chr1	12572727	12617303
chr1	1267861	1271637
chr1	143974543	144068326
chr1	144955438	144960398
chr1	145405097	145420756
chr1	145771710	145794196
chr1	145839889	145842477

chr1	1459160	1525291
chr1	145936483	145937699
chr1	1468383	1529331
chr1	147272381	147294517
chr1	1477273	1477939
chr1	148135885	148137809
chr1	1482137	1518990
chr1	1482544	1520306
chr1	148433523	148436564
chr1	1485015	1520306
chr1	150408905	150417725
chr1	150648667	150663763
chr1	150929397	150941428
chr1	150966161	150969091
chr1	151117391	151118139
chr1	151239954	151240259
chr1	151262160	151267339
chr1	151265165	151267339
chr1	151440927	151451050
chr1	151638887	151699485
chr1	151982665	151986251
chr1	152314905	152341210
chr1	152327271	152335439
chr1	153665167	153668557
chr1	153982354	153982534
chr1	153983218	153986294
chr1	153991223	153991510
chr1	1541813	1542167
chr1	154225083	154241797
chr1	154254089	154254812
chr1	154987465	154990528
chr1	155215053	155235311
chr1	155216383	155236469
chr1	155251501	155254722
chr1	155337588	155415923
chr1	155343313	155404350
chr1	155370503	155462599
chr1	155408141	155415923
chr1	155415743	155424672
chr1	155415743	155440593
chr1	155415743	155477958
chr1	155438326	155462599
chr1	155438326	155477958
chr1	155612420	155747896
chr1	155674684	155675096

chr1	155674684	155679512
chr1	155676551	155679512
chr1	155900315	155910804
chr1	156018131	156018368
chr1	1561331	1564916
chr1	156207885	156210427
chr1	15638306	15646984
chr1	15643521	15646984
chr1	15643521	15660190
chr1	1564486	1564916
chr1	1564604	1564916
chr1	156743651	156746433
chr1	156951572	157044499
chr1	15776946	15777287
chr1	158073806	158076262
chr1	158077990	158078507
chr1	159918762	159920537
chr1	159918841	159920537
chr1	160222598	160261358
chr1	160290136	160299264
chr1	160316218	160332557
chr1	160317199	160332557
chr1	161021009	161100859
chr1	161791407	161871061
chr1	162365403	162367204
chr1	16255710	16316571
chr1	16255710	16328849
chr1	162566011	162568386
chr1	162566011	162655374
chr1	162576776	162599355
chr1	162576776	162600903
chr1	162576776	162601514
chr1	162576776	162655374
chr1	162579527	162597858
chr1	162587474	162600903
chr1	162590322	162601514
chr1	162597791	162601514
chr1	162597791	162655374
chr1	162599270	162601514
chr1	16294782	16316568
chr1	16294782	16316571
chr1	16305802	16316571
chr1	16305802	16316727
chr1	1637761	1704655
chr1	16484822	16631109

chr1	16625774	16633346
chr1	16625774	16635168
chr1	16630889	16633346
chr1	16630889	16635168
chr1	166848937	166852772
chr1	1669663	1734835
chr1	167332469	167338258
chr1	167744902	167776166
chr1	167765582	167769135
chr1	167765582	167788019
chr1	167765582	167788137
chr1	167772274	167788019
chr1	167772274	167788137
chr1	167987494	168019667
chr1	168019544	168023047
chr1	168028425	168038488
chr1	168231513	168242987
chr1	168231513	168243069
chr1	168231513	168243093
chr1	168235100	168243069
chr1	168235100	168243093
chr1	168239130	168293278
chr1	1692448	1739704
chr1	169319158	169324500
chr1	169377137	169378529
chr1	169451132	169477757
chr1	169476414	169477757
chr1	169836926	169850327
chr1	169862612	169869128
chr1	169978084	170031975
chr1	171485685	171517814
chr1	171579353	171584541
chr1	172038318	172055926
chr1	172442848	172443987
chr1	173460377	173462454
chr1	173785162	173787962
chr1	173800248	173803505
chr1	173830254	173830761
chr1	173864745	173865228
chr1	173866230	173866527
chr1	173941264	173943708
chr1	173946475	173993135
chr1	173970504	173993135
chr1	173978487	173993135
chr1	173992754	173993135

chr1	17408979	17422836
chr1	174250569	174278779
chr1	174800327	174811960
chr1	175014672	175018576
chr1	176081151	176144100
chr1	176144004	176163891
chr1	176461053	176463418
chr1	1779904	1825482
chr1	178123047	178300118
chr1	178386993	178390206
chr1	178737107	178833550
chr1	179101711	179133374
chr1	179846106	179852811
chr1	179856057	179865854
chr1	180033950	180041802
chr1	180273337	180284248
chr1	180397515	180424223
chr1	1804418	1879902
chr1	180976117	180993425
chr1	1815755	1825482
chr1	1815755	1853297
chr1	1815755	1879902
chr1	1817836	1853297
chr1	1825396	1848036
chr1	1825396	1879902
chr1	183697402	183757273
chr1	1839189	1853297
chr1	1839189	1879902
chr1	184477464	184507682
chr1	186406082	186411893
chr1	186406082	186419077
chr1	186451178	186470395
chr1	187161748	187329060
chr1	187167865	187329060
chr1	187254972	187329060
chr1	187269395	187329060
chr1	187294159	187329060
chr1	187303465	187443726
chr1	187324092	187329060
chr1	18908569	18921121
chr1	19201840	19210072
chr1	193069033	193070662
chr1	193096647	193101204
chr1	19385140	19385626
chr1	197117788	197146594

chr1	19770541	19790048
chr1	197732378	197735796
chr1	198220998	198264235
chr1	19890384	19907876
chr1	19904266	19907660
chr1	200368664	200374246
chr1	200368664	200379537
chr1	202411617	202428929
chr1	202448988	202502005
chr1	204112914	204126015
chr1	205098988	205101690
chr1	205161257	205170924
chr1	205616477	205619073
chr1	205616477	205620838
chr1	205616477	205623891
chr1	20648314	20649353
chr1	20744743	20749882
chr1	20744743	20757256
chr1	20744743	20773610
chr1	20744743	20780540
chr1	20749722	20749975
chr1	20853559	20857442
chr1	20879281	20895501
chr1	20879281	20942330
chr1	20892618	20950111
chr1	20941258	20942330
chr1	21001198	21002808
chr1	21001198	21050994
chr1	21001198	21089213
chr1	21001198	21111383
chr1	21046958	21050994
chr1	21046958	21111383
chr1	21050865	21134034
chr1	210919639	210920967
chr1	21098214	21111383
chr1	21105116	21111383
chr1	211266864	211274262
chr1	211422538	211426283
chr1	212100251	212102952
chr1	212325982	212345933
chr1	21272698	21320960
chr1	212738447	212739601
chr1	212782371	212791475
chr1	212872677	212895354
chr1	213061381	213077816

chr1	213077695	213088030
chr1	213077695	213142801
chr1	213205238	213230544
chr1	2144209	2145511
chr1	214451804	214551278
chr1	214655240	214663780
chr1	2150789	2157316
chr1	21695065	21721163
chr1	21715388	21721737
chr1	219179146	219211752
chr1	219193081	219211752
chr1	219193081	219218712
chr1	219193081	219241308
chr1	219193081	219316005
chr1	219193129	219218712
chr1	219193129	219241308
chr1	220195082	220196398
chr1	220761657	220770556
chr1	22078428	22091501
chr1	22081721	22091501
chr1	223954476	223962273
chr1	223954476	223962708
chr1	224267003	224268133
chr1	224365878	224377177
chr1	224411426	224413335
chr1	22490525	22491533
chr1	224952669	224967514
chr1	224952669	224980270
chr1	224952669	224999440
chr1	224952673	224974153
chr1	224952684	225007544
chr1	224980143	225097239
chr1	225002782	225123614
chr1	225023614	225058172
chr1	225072754	225097239
chr1	225080561	225097239
chr1	225117683	225123614
chr1	225123526	225185425
chr1	225144521	225185425
chr1	225159313	225185425
chr1	22517464	22520002
chr1	225270734	225276081
chr1	225921207	225923388
chr1	227040130	227051985
chr1	227139575	227317246

chr1	227160542	227317246
chr1	227193785	227317196
chr1	227193785	227317246
chr1	227213135	227317246
chr1	227254063	227317246
chr1	228097077	228098184
chr1	228292522	228304468
chr1	228394736	228395252
chr1	229286485	229304000
chr1	229286485	229313760
chr1	229497683	229498306
chr1	229518840	229556947
chr1	229525935	229549584
chr1	229538813	229542371
chr1	229538813	229549434
chr1	230226828	230236725
chr1	23030468	23040434
chr1	230839855	230843896
chr1	231247306	231251143
chr1	231364326	231367636
chr1	231364326	231374099
chr1	231749925	231774817
chr1	232513856	232515608
chr1	232513856	232533583
chr1	233013216	233057290
chr1	233090060	233112966
chr1	233095754	233155812
chr1	233112798	233161363
chr1	233112798	233179175
chr1	233112844	233161363
chr1	233125458	233139855
chr1	233125458	233208689
chr1	233135012	233208610
chr1	233177801	233208610
chr1	233198938	233208610
chr1	233236255	233236980
chr1	23333517	23341020
chr1	23333517	23341038
chr1	234229678	234231741
chr1	234391073	234391745
chr1	234418083	234452875
chr1	234606957	234607852
chr1	235341707	235342883
chr1	235341707	235427239
chr1	235465635	235496308

chr1	236168705	236185478
chr1	236183777	236194796
chr1	236803404	236806233
chr1	236803430	236816543
chr1	236806143	236807726
chr1	23694791	23695774
chr1	2393207	2399514
chr1	2393269	2397220
chr1	2393269	2402342
chr1	2395783	2403589
chr1	23967501	23972012
chr1	239811120	239827378
chr1	239826852	239827378
chr1	240121572	240123345
chr1	240123178	240137802
chr1	240123178	240140496
chr1	240140358	240188262
chr1	240150187	240188262
chr1	240158139	240211235
chr1	240158139	240294883
chr1	240163121	240178068
chr1	240165688	240178068
chr1	240165688	240211235
chr1	240171269	240188262
chr1	240177920	240234450
chr1	240177920	240258596
chr1	240186914	240211235
chr1	240206798	240215851
chr1	240355815	240414448
chr1	240414332	240438210
chr1	241850408	241869209
chr1	241885313	241897032
chr1	243270091	243286397
chr1	243270091	243308177
chr1	243660074	243843282
chr1	243693666	243843282
chr1	244408711	244416078
chr1	244409484	244437768
chr1	244476156	244479784
chr1	244845246	244856158
chr1	244845246	244862730
chr1	245002120	245022296
chr1	245017241	245028034
chr1	245915319	245915640
chr1	246274304	246327337

chr1	246327200	246390206
chr1	246327200	246440124
chr1	246540937	246551302
chr1	246618257	246634587
chr1	246627355	246631924
chr1	246726891	246780583
chr1	246735974	246736550
chr1	246748796	246758356
chr1	247138715	247159813
chr1	247155565	247159813
chr1	247156405	247159813
chr1	247156999	247159813
chr1	247157083	247159813
chr1	247860616	247896195
chr1	247864704	247876037
chr1	25458390	25468563
chr1	26170417	26171365
chr1	26237831	26244318
chr1	26442707	26447660
chr1	26446315	26447682
chr1	26474135	26474547
chr1	26848607	26853825
chr1	27113790	27114286
chr1	27303620	27306272
chr1	28032348	28035680
chr1	28214939	28234492
chr1	28272317	28272793
chr1	28407409	28408135
chr1	28407409	28409039
chr1	28459084	28466535
chr1	28476111	28480604
chr1	28553639	28564985
chr1	28580673	28581110
chr1	28683582	28683976
chr1	29031787	29039426
chr1	29044194	29053312
chr1	29148079	29154910
chr1	29148079	29159486
chr1	29148566	29154910
chr1	29154443	29154910
chr1	30988308	30992660
chr1	30992389	31007099
chr1	31005852	31007099
chr1	31005852	31015973
chr1	31005852	31019509

chr1	31055312	31059577
chr1	31059298	31059577
chr1	31907660	31911826
chr1	31907660	31919658
chr1	31911695	31931142
chr1	31915890	31919658
chr1	31915894	31931142
chr1	32030297	32033399
chr1	32184524	32188148
chr1	32187581	32188124
chr1	32296389	32302733
chr1	32553458	32557965
chr1	32595053	32600415
chr1	32657426	32660694
chr1	32657426	32662337
chr1	32668224	32680614
chr1	33330070	33335359
chr1	33491702	33493159
chr1	35093913	35113181
chr1	35358924	35362266
chr1	35381260	35408159
chr1	35451523	35460437
chr1	35901473	35902329
chr1	35938437	35973511
chr1	35972023	35984548
chr1	36258665	36259484
chr1	36259381	36272894
chr1	36358238	36362656
chr1	36361220	36362656
chr1	37452239	37455912
chr1	37537671	37538012
chr1	37537671	37540755
chr1	37540156	37554242
chr1	37587310	37589082
chr1	3780637	3785203
chr1	3781150	3781601
chr1	3783769	3785203
chr1	37866458	37874088
chr1	37930457	37932053
chr1	38997881	39001106
chr1	39361001	39361338
chr1	39741365	39748767
chr1	39844100	39854155
chr1	39850118	39852951
chr1	39850122	39857417

chr1	39853969	39862935
chr1	39957090	39958825
chr1	40070158	40071079
chr1	40227412	40231658
chr1	40259349	40260985
chr1	40260838	40262983
chr1	41070594	41071973
chr1	41105741	41117010
chr1	41113282	41162979
chr1	41142873	41186250
chr1	41186120	41192900
chr1	41628748	41646243
chr1	42227882	42278784
chr1	42265114	42278784
chr1	42773562	42775479
chr1	42773562	42775522
chr1	42773562	42782736
chr1	42789795	42794613
chr1	43447861	43452013
chr1	43604189	43604880
chr1	43792101	43792925
chr1	43831654	43838311
chr1	43858134	43858636
chr1	43920403	43921638
chr1	44241914	44248038
chr1	44410583	44412722
chr1	44753254	44753699
chr1	44777613	44778717
chr1	45334390	45334967
chr1	45457563	45460492
chr1	45600384	45602365
chr1	45634095	45660377
chr1	45824432	45834766
chr1	45911039	46010939
chr1	45917338	45959477
chr1	45959385	45960625
chr1	46022971	46023213
chr1	46033801	46033987
chr1	46632758	46645256
chr1	46678468	46691937
chr1	47287550	47300072
chr1	47289434	47300072
chr1	47348986	47373107
chr1	47368468	47376882
chr1	50529662	50596216

chr1	50563305	50567231
chr1	50567076	50606942
chr1	50567076	50752523
chr1	50655441	50781044
chr1	50705785	50781044
chr1	50738862	50752523
chr1	50738862	50781044
chr1	51269955	51271473
chr1	51271150	51271473
chr1	51437105	51448135
chr1	52083339	52086853
chr1	52515890	52529873
chr1	52860730	52892975
chr1	52860730	52899713
chr1	52877681	52892975
chr1	52877772	52881665
chr1	52886955	52892975
chr1	52954739	52968083
chr1	52978227	52980543
chr1	54190737	54195715
chr1	56496554	56537112
chr1	56655003	56674522
chr1	58492782	58536741
chr1	58506059	58536595
chr1	58506059	58537466
chr1	58506059	58544724
chr1	58515743	58527335
chr1	58522826	58536741
chr1	5922059	5933329
chr1	5922059	5986327
chr1	5927648	5986232
chr1	59321535	59332032
chr1	59321535	59474202
chr1	5947103	5986232
chr1	5952699	5990971
chr1	61088148	61159155
chr1	61672625	61709437
chr1	61972925	62051058
chr1	62577261	62626904
chr1	62803718	62810586
chr1	63411925	63436915
chr1	63478763	63502426
chr1	63628204	63654466
chr1	6667708	6691831
chr1	6792012	6825210

chr1	6796814	6825210
chr1	6806982	6825210
chr1	6871756	6903630
chr1	70221632	70228555
chr1	70228421	70230618
chr1	75790135	75790517
chr1	77154448	77169372
chr1	77154448	77212347
chr1	77206639	77212347
chr1	77565441	77584655
chr1	77621691	77633405
chr1	77631843	77641655
chr1	77631849	77675689
chr1	77633114	77641655
chr1	77639448	77675689
chr1	77858937	77875111
chr1	77873003	77875111
chr1	77944182	77960495
chr1	77968164	77979103
chr1	78268849	78342640
chr1	7977651	7985135
chr1	84111534	84144548
chr1	84144219	84144548
chr1	84144219	84214317
chr1	84179176	84235364
chr1	84647497	84671308
chr1	847653	853522
chr1	84865384	84865802
chr1	8495062	8551356
chr1	85321412	85324883
chr1	85350414	85360537
chr1	85350414	85361500
chr1	85350414	85361854
chr1	85350414	85367779
chr1	85350414	85409711
chr1	8541213	8557537
chr1	8541213	8621825
chr1	86715723	86743235
chr1	8736882	8792588
chr1	88691723	88741288
chr1	88784638	88790053
chr1	88936098	88955225
chr1	90916639	90918154
chr1	90916639	90941309
chr1	90916759	90918154

chr1	91513182	91515876
chr1	92087208	92088895
chr1	92088697	92147786
chr1	92107883	92147786
chr1	92177810	92180416
chr1	92333390	92363770
chr1	92333415	92380873
chr1	925921	939412
chr1	92605306	92639237
chr1	92624175	92648985
chr1	92662718	92665992
chr1	92662718	92704744
chr1	92725597	92736627
chr1	92732509	92736627
chr1	92736397	92738040
chr1	92736397	92745802
chr1	92736397	92774023
chr1	93154655	93160226
chr1	93156299	93162785
chr1	93305387	93311608
chr1	93305387	93325895
chr1	93305387	93340510
chr1	93324634	93331812
chr1	93360492	93362958
chr1	93490743	93499583
chr1	93490743	93534908
chr1	935771	939412
chr1	93588976	93605200
chr1	93667095	93674941
chr1	94455732	94468007
chr1	94455732	94475737
chr1	94901807	94902120
chr1	94961316	94963170
chr1	95138274	95151419
chr1	95138274	95173889
chr1	95143890	95218458
chr1	9726908	9727772
chr1	9730404	9730705
chr1	9740657	9741256
chr1	97679094	97757863
chr1	9773271	9809407
chr1	9922185	9934860
chr1	9922185	9935627
chr1	9931890	9942711
chr1	9970752	9981170

chr1	99710805	99729748
chr20	10449355	10458231
chr20	1164476	1165006
chr20	13483226	13600617
chr20	13483226	13610919
chr20	13483226	13617123
chr20	13528432	13537049
chr20	13528432	13610919
chr20	13528432	13617123
chr20	13528445	13559114
chr20	13528445	13569586
chr20	13551886	13587370
chr20	13559007	13600617
chr20	13559007	13610919
chr20	13559007	13611015
chr20	13569506	13617123
chr20	13580896	13610919
chr20	13616856	13630152
chr20	14002287	14009973
chr20	17658604	17658799
chr20	17943109	17954231
chr20	17952586	17954231
chr20	17955364	17961326
chr20	17956932	17961326
chr20	17969800	17970130
chr20	18142207	18142919
chr20	18142207	18145351
chr20	18142207	18150942
chr20	18142207	18154244
chr20	18305178	18305666
chr20	18543026	18554390
chr20	18742152	18744271
chr20	19935098	19935607
chr20	20022455	20022988
chr20	20025668	20026919
chr20	20472755	20526362
chr20	20629362	20644083
chr20	2116665	2143892
chr20	21331818	21332274
chr20	24962695	24973737
chr20	25306919	25307965
chr20	25319051	25320318
chr20	25323324	25342096
chr20	25338884	25339351
chr20	25413789	25426100

chr20	25632157	25645648
chr20	25632157	25674007
chr20	25632157	25683223
chr20	25634048	25645648
chr20	25639728	25645648
chr20	25835983	25848948
chr20	2886289	2965202
chr20	29091593	29100460
chr20	31527483	31528050
chr20	31766576	31794024
chr20	320504	322565
chr20	321738	322565
chr20	3218516	3218994
chr20	32316217	32331551
chr20	32326770	32331546
chr20	32366383	32369883
chr20	32366383	32371874
chr20	32366383	32372239
chr20	32366383	32376182
chr20	32366383	32389728
chr20	32366383	32391167
chr20	32366383	32391336
chr20	32366383	32393973
chr20	32366383	32410418
chr20	32824705	32826048
chr20	33379460	33382179
chr20	33392134	33397084
chr20	33589611	33623296
chr20	33782288	33783864
chr20	34017409	34031604
chr20	34029768	34031604
chr20	34031521	34033318
chr20	34059192	34078553
chr20	34065043	34072330
chr20	34065043	34078553
chr20	34072065	34080072
chr20	34072065	34084809
chr20	34089515	34090602
chr20	34280521	34281165
chr20	34280521	34285634
chr20	34281026	34295585
chr20	34424479	34426381
chr20	34481065	34507908
chr20	34714589	34715365
chr20	34762726	34768586

chr20	34882793	34883615
chr20	35035122	35044606
chr20	35126250	35127016
chr20	35366556	35375945
chr20	35384037	35386389
chr20	35477867	35478101
chr20	35501966	35502328
chr20	35511362	35512066
chr20	35631267	35648376
chr20	35653527	35658982
chr20	35653527	35659014
chr20	35653797	35659014
chr20	35655156	35659014
chr20	35655201	35659014
chr20	35714184	35726424
chr20	35721739	35742113
chr20	35738967	35742113
chr20	35792999	35801605
chr20	35800093	35801605
chr20	35801490	35812719
chr20	36173883	36198041
chr20	36609600	36614350
chr20	36687417	36688784
chr20	36751877	36752897
chr20	36904156	36917096
chr20	36911217	36917096
chr20	37003701	37032751
chr20	37301207	37302425
chr20	38035215	38057644
chr20	38057537	38066256
chr20	38065802	38066256
chr20	38130335	38132500
chr20	38429953	38434626
chr20	38479806	38493132
chr20	38479806	38499633
chr20	3854674	3864788
chr20	3896863	3910830
chr20	39028417	39038618
chr20	3912457	3913612
chr20	41201285	41205066
chr20	41213733	41269083
chr20	41238687	41269083
chr20	41267462	41269083
chr20	41512845	41564151
chr20	417219	417930

chr20	43594692	43605216
chr20	44500156	44501300
chr20	44719896	44722446
chr20	44975323	44987296
chr20	45024972	45053180
chr20	45366874	45410255
chr20	45888890	45890249
chr20	45952597	45957702
chr20	46063336	46079453
chr20	46122358	46122582
chr20	47276230	47276795
chr20	47309658	47310204
chr20	47339204	47347926
chr20	48939916	48963898
chr20	4899554	4970915
chr20	49084025	49091066
chr20	49114768	49124605
chr20	49135832	49159146
chr20	49153932	49156671
chr20	49153932	49159146
chr20	49642616	49643525
chr20	49646964	49647892
chr20	49952075	49960298
chr20	50172582	50173017
chr20	50645697	50651404
chr20	50902016	50918781
chr20	50935029	50942126
chr20	51009564	51012416
chr20	5144792	5173659
chr20	5189066	5190757
chr20	52084491	52088643
chr20	52084491	52097435
chr20	52084491	52098587
chr20	5598724	5609766
chr20	56382984	56390100
chr20	5700744	5773043
chr20	58247321	58271505
chr20	58271362	58272875
chr20	58271362	58286446
chr20	58343717	58359735
chr20	58991446	58991857
chr20	62179181	62181995
chr20	62195801	62199283
chr20	62286582	62295792
chr20	62658676	62670120

chr20	62660533	62660891
chr20	62852722	62854157
chr20	62852722	62857638
chr20	62852722	62860308
chr20	62854015	62860256
chr20	6355479	6473655
chr20	63733739	63734673
chr20	63775677	63805464
chr20	63917576	63931022
chr20	63926541	63931022
chr20	63928334	63931182
chr20	63928334	63931734
chr20	63944396	63945988
chr20	6446722	6530103
chr20	6473538	6484225
chr20	8603566	8658704
chr20	9217389	9218382
chr20	9326279	9339037
chr20	9457413	9460596
chr20	9468672	9472761
chr21	14853273	15014466
chr21	14857541	15043574
chr21	14964469	15014466
chr21	14964621	15043574
chr21	14964712	15014466
chr21	14968296	15043574
chr21	14979200	15043574
chr21	14991200	15014466
chr21	14994694	15043574
chr21	15011169	15014466
chr21	15011169	15043574
chr21	15014343	15015225
chr21	15014343	15043570
chr21	15014343	15045323
chr21	15014343	15045613
chr21	15042716	15043574
chr21	15740415	15778027
chr21	15761561	15766141
chr21	17594053	17609152
chr21	18589526	18612293
chr21	18612052	18738025
chr21	21196109	21374013
chr21	22096528	22190634
chr21	28162191	28163717
chr21	28163601	28197822

chr21	28163601	28228478
chr21	28212239	28228478
chr21	28212239	28402154
chr21	29321220	29361713
chr21	29327280	29329693
chr21	29351633	29411624
chr21	29370920	29372563
chr21	29582311	29597694
chr21	29597569	29637721
chr21	31338634	31339422
chr21	31690796	31697474
chr21	32274829	32284155
chr21	32368402	32375461
chr21	32691284	32695282
chr21	32693171	32695282
chr21	33407174	33415020
chr21	34094862	34125480
chr21	34102834	34125480
chr21	34125340	34164706
chr21	34834142	34834601
chr21	36007318	36007834
chr21	36232803	36293575
chr21	36280284	36293575
chr21	36338771	36344707
chr21	36338774	36344707
chr21	36338778	36344707
chr21	36341194	36344707
chr21	36377409	36386262
chr21	36754179	36765172
chr21	36754179	36767285
chr21	36892579	36897131
chr21	36896859	36910538
chr21	37065467	37072537
chr21	37084008	37096643
chr21	37087246	37096906
chr21	37087246	37098010
chr21	37087246	37098160
chr21	37087246	37099748
chr21	37420298	37421866
chr21	37420298	37425781
chr21	37420298	37427401
chr21	37420298	37430400
chr21	37420298	37456308
chr21	39177488	39179986
chr21	39246952	39258672

chr21	41237512	41241939
chr21	41942146	41943752
chr21	42685821	42686262
chr21	43004325	43029981
chr21	43058279	43058833
chr21	43529757	43613947
chr21	43592224	43620693
chr21	43741666	43756099
chr21	43903956	43904452
chr21	43903956	43906907
chr21	43980988	43982619
chr21	44956714	44967181
chr21	45116932	45134832
chr21	45118563	45128573
chr21	45118563	45134832
chr21	45120793	45128573
chr21	45128401	45144701
chr21	45788297	45850095
chr21	46161382	46168658
chr21	46314715	46318961
chr21	46479551	46498833
chr21	46548428	46549885
chr22	16602063	16614178
chr22	16602063	16638740
chr22	16630980	16638740
chr22	17702242	17704228
chr22	17826453	17832107
chr22	18083436	18089333
chr22	18987756	18988195
chr22	19139854	19140021
chr22	19351071	19410778
chr22	19433264	19435913
chr22	19447377	19450744
chr22	19852539	19854442
chr22	20093679	20094795
chr22	20119012	20127450
chr22	20140217	20140598
chr22	20985840	20986174
chr22	21700975	21710908
chr22	21700975	21807846
chr22	21769194	21805945
chr22	21788256	21805945
chr22	21805824	21807846
chr22	21939531	21941587
chr22	23659864	23686995

chr22	23683724	23686995
chr22	23683724	23695512
chr22	23816624	23825415
chr22	24567983	24572061
chr22	25170819	25192668
chr22	25181698	25192668
chr22	25351611	25354812
chr22	25354595	25381578
chr22	25375812	25381578
chr22	25587962	25604453
chr22	25594298	25604453
chr22	25594298	25644665
chr22	25594298	25667800
chr22	25594298	25678915
chr22	26526028	26544675
chr22	26717252	26718231
chr22	27853512	27860241
chr22	27853512	27873815
chr22	27853524	27860241
chr22	27853536	27860241
chr22	27853536	27873815
chr22	27935150	27940171
chr22	28163091	28163595
chr22	28296197	28299669
chr22	28296197	28360201
chr22	28515626	28629830
chr22	28583546	28629830
chr22	28629551	28633620
chr22	28629551	28633974
chr22	28629551	28646269
chr22	28629551	28656539
chr22	28629551	28661900
chr22	28695709	28697129
chr22	28699837	28724923
chr22	29502378	29519120
chr22	29560852	29561170
chr22	29560855	29561170
chr22	29978424	29991670
chr22	30016542	30018072
chr22	30079138	30098993
chr22	30243739	30244061
chr22	30342909	30344932
chr22	30646597	30649253
chr22	30731496	30741369
chr22	30741160	30743835

chr22	30741160	30773245
chr22	30870428	30919211
chr22	31095609	31096628
chr22	31433871	31435265
chr22	31455821	31463133
chr22	31672210	31704726
chr22	31814564	31815212
chr22	33549931	33565019
chr22	33562855	33565019
chr22	33564847	33577964
chr22	33713547	33761558
chr22	33737479	33856802
chr22	33761370	33761723
chr22	33761370	33765953
chr22	33761370	33767719
chr22	33761370	33769743
chr22	33761370	33847741
chr22	33761370	33880733
chr22	33761370	33900554
chr22	33856739	33900554
chr22	35192184	35195019
chr22	35262322	35293743
chr22	35287346	35293743
chr22	35292991	35293743
chr22	35421460	35423192
chr22	35423341	35424128
chr22	35656378	35659449
chr22	35809779	35992754
chr22	35936290	35938897
chr22	36285347	36285657
chr22	36285356	36285657
chr22	36493632	36493972
chr22	37496448	37588689
chr22	37629526	37629975
chr22	37886764	37888577
chr22	37937079	37937819
chr22	38302860	38303420
chr22	38474232	38474623
chr22	38521607	38541673
chr22	38617738	38623279
chr22	38636450	38650154
chr22	38826499	38828509
chr22	39414879	39421971
chr22	40409018	40409232
chr22	40519463	40552367

chr22	40519463	40594735
chr22	40548718	40552367
chr22	40552105	40586112
chr22	40868998	40870139
chr22	40868998	40926463
chr22	40886312	40888521
chr22	41528540	41540795
chr22	41540698	41541090
chr22	41600697	41604153
chr22	41658352	41661298
chr22	42019310	42030823
chr22	42516010	42577098
chr22	42518213	42519713
chr22	42583940	42585968
chr22	42627412	42627604
chr22	42797467	42799160
chr22	42817141	42822607
chr22	42840943	42858522
chr22	42909426	42912157
chr22	42912020	42999640
chr22	43039779	43046573
chr22	43168092	43174615
chr22	45178590	45178900
chr22	45330602	45340561
chr22	45417601	45419359
chr22	45729404	45740538
chr22	45736967	45807022
chr22	45793544	45807022
chr22	46097925	46109908
chr22	46098010	46098469
chr22	46175988	46198591
chr22	46623412	46626961
chr22	46626750	46637960
chr22	46788979	46797620
chr22	46789311	46797620
chr22	46793509	46912188
chr22	46974289	46977520
chr22	46974289	46978934
chr22	46990014	47037198
chr22	46990694	47037198
chr22	47034455	47037198
chr22	47662848	47687206
chr22	47684516	47687206
chr22	47686199	47707170
chr22	47704588	47710747

chr22	47704588	47728995
chr22	49883333	49895233
chr22	50204918	50206520
chr22	50294202	50294778
chr22	50294718	50296392
chr22	50372019	50389111
chr22	50372019	50394079
chr22	50372019	50394124
chr22	50443865	50444278
chr22	50459969	50467448
chr22	50768759	50782351
chr2	101260489	101262691
chr2	102783893	102798000
chr2	105049170	105056604
chr2	105339582	105345389
chr2	10600852	10608283
chr2	10636524	10659250
chr2	10643320	10644372
chr2	108472041	108475850
chr2	108621391	108659764
chr2	108769259	108773046
chr2	108839184	108850755
chr2	108846737	108882791
chr2	108873450	108876442
chr2	108873450	108877146
chr2	109329223	109347949
chr2	110666269	110669553
chr2	111233627	111367575
chr2	111365560	111367575
chr2	111772340	111792551
chr2	112485916	112494410
chr2	112485916	112496028
chr2	11249660	11288449
chr2	11286538	11288449
chr2	113444963	113457204
chr2	113830851	113843353
chr2	118096418	118108377
chr2	118096418	118109900
chr2	118103196	118108377
chr2	119789751	119809991
chr2	119916622	119934958
chr2	120027589	120042110
chr2	120127687	120175000
chr2	120127687	120175004
chr2	120278617	120293309

chr2	121515664	121530513
chr2	121605700	121621213
chr2	127329920	127343194
chr2	127335869	127343194
chr2	127731782	127771004
chr2	127763061	127770934
chr2	127768188	127770934
chr2	131052282	131055695
chr2	131071854	131072295
chr2	131082576	131089336
chr2	131500203	131502692
chr2	134250049	134270550
chr2	134253094	134254644
chr2	134253094	134270550
chr2	134254261	134270550
chr2	134254264	134270550
chr2	134866541	134869041
chr2	134867519	134869041
chr2	135089650	135093693
chr2	135120818	135127662
chr2	135162554	135176443
chr2	135335399	135345635
chr2	135565736	135605016
chr2	135602499	135606226
chr2	135602499	135633828
chr2	135645378	135671512
chr2	135645378	135695906
chr2	135649901	135652032
chr2	135745899	135755691
chr2	135840528	135852915
chr2	135852286	135859442
chr2	135924386	135930599
chr2	143910138	143970762
chr2	144141881	144275218
chr2	144177134	144182476
chr2	144208602	144319868
chr2	144294364	144319868
chr2	147870450	147899567
chr2	147871445	147899898
chr2	148178699	148186180
chr2	148310129	148342336
chr2	148690213	148703808
chr2	149449048	149470797
chr2	149468644	149486345
chr2	150469923	150487455

chr2	151422949	151425565
chr2	151841667	151842088
chr2	15461200	15478321
chr2	156476097	156483466
chr2	158457152	158642699
chr2	158477243	158533316
chr2	158533179	158578263
chr2	158994901	159001189
chr2	158998477	159001189
chr2	159001079	159011440
chr2	159248371	159282873
chr2	160300650	160329883
chr2	161359715	161367903
chr2	1632505	1644752
chr2	1691927	1734453
chr2	169560386	169571191
chr2	169592650	169614724
chr2	169631598	169631933
chr2	170013840	170040985
chr2	170013840	170061443
chr2	170053818	170061443
chr2	170817048	170818510
chr2	170842568	170854022
chr2	171011372	171023129
chr2	171045061	171046362
chr2	171082780	171092227
chr2	171117302	171117857
chr2	171117317	171117857
chr2	171117738	171132092
chr2	171271218	171339554
chr2	171301011	171330698
chr2	171324043	171331867
chr2	171324116	171339554
chr2	171447686	171473975
chr2	171468887	171469452
chr2	171468887	171469456
chr2	171536888	171541793
chr2	171540807	171541793
chr2	173090997	173137920
chr2	173358711	173367831
chr2	173363225	173367831
chr2	173365451	173367831
chr2	174123179	174157864
chr2	174123179	174182524
chr2	174141824	174182524

chr2	174141824	174203683
chr2	174182416	174229451
chr2	174210880	174229451
chr2	174875787	174878128
chr2	174915057	174940894
chr2	175935707	175964589
chr2	175939551	175964533
chr2	175970198	175995646
chr2	176296859	176337577
chr2	177219036	177222133
chr2	177231884	177234271
chr2	177231888	177234271
chr2	17759931	17768811
chr2	178332640	178382530
chr2	178527195	178542361
chr2	178531313	178542361
chr2	178535731	178542995
chr2	178535731	178548554
chr2	178571299	178575326
chr2	178576946	178583921
chr2	178582571	178583648
chr2	178591117	178600667
chr2	178591138	178608266
chr2	179172119	179237433
chr2	180982017	181063041
chr2	181057692	181063041
chr2	181892031	181899112
chr2	183133565	183161195
chr2	183151507	183161195
chr2	183151507	183182680
chr2	183151507	183188763
chr2	183157443	183161195
chr2	183161053	183182680
chr2	185729328	185788943
chr2	186081329	186086370
chr2	186081329	186099830
chr2	186636009	186641588
chr2	188334340	188383889
chr2	189791789	189818829
chr2	189791789	189820591
chr2	189794139	189795951
chr2	189794848	189795951
chr2	190488697	190510537
chr2	190490708	190510537
chr2	190510360	190519022

chr2	190899116	190924593
chr2	190910262	190913364
chr2	191693202	191693554
chr2	196047351	196075205
chr2	196912881	196923125
chr2	197069829	197090032
chr2	197298791	197301413
chr2	197503038	197503246
chr2	197503038	197503330
chr2	197523623	197559757
chr2	197535529	197536041
chr2	199297362	199349173
chr2	199368604	199419896
chr2	199933026	199948472
chr2	200987364	201011472
chr2	201140356	201164084
chr2	201145377	201164084
chr2	201160431	201164084
chr2	201744257	201761324
chr2	202206736	202220106
chr2	202282350	202304606
chr2	202456535	202467689
chr2	20260336	20333063
chr2	202779389	202819899
chr2	202779389	202829016
chr2	20278194	20297678
chr2	20278582	20333063
chr2	20278582	20346093
chr2	20290651	20333063
chr2	20297552	20333063
chr2	203045635	203049975
chr2	203056410	203057453
chr2	20307977	20333063
chr2	203127780	203128229
chr2	20318536	20331937
chr2	20318536	20333063
chr2	203226122	203252109
chr2	20326260	20333063
chr2	203346364	203367044
chr2	203355153	203367044
chr2	203355153	203402734
chr2	203366876	203427269
chr2	203375931	203427269
chr2	203376056	203427269
chr2	203380207	203383321

chr2	203380207	203427269
chr2	203383246	203417081
chr2	203402575	203427269
chr2	203411284	203427269
chr2	203489583	203497395
chr2	204657141	204686282
chr2	204661808	204686282
chr2	205276261	205301701
chr2	206085900	206095791
chr2	206105964	206126844
chr2	206159684	206159935
chr2	206925921	206939667
chr2	207123770	207124404
chr2	207581793	207582160
chr2	207596913	207597483
chr2	207620640	207621917
chr2	207714341	207742342
chr2	207828222	207861262
chr2	207828222	207908577
chr2	207901127	207908577
chr2	210015972	210021064
chr2	210104103	210107839
chr2	210104103	210155408
chr2	210153494	210155404
chr2	213310058	213356393
chr2	214727659	214792445
chr2	214728636	214745854
chr2	214728917	214769312
chr2	214728917	214792445
chr2	215312497	215326161
chr2	215325750	215326138
chr2	215976561	215996465
chr2	216277671	216295078
chr2	218265705	218266063
chr2	218495759	218565385
chr2	218688390	218690555
chr2	219250642	219252230
chr2	219285986	219286314
chr2	219601715	219601966
chr2	222630112	222647325
chr2	223878528	223880232
chr2	223884647	223899117
chr2	224528946	224535641
chr2	224528946	224557856
chr2	224535527	224558645

chr2	224556294	224557856
chr2	224557658	224558241
chr2	226837547	226914351
chr2	226864603	226867688
chr2	227305459	227309293
chr2	227328677	227357119
chr2	229155817	229202012
chr2	229388264	229394497
chr2	229512782	229574850
chr2	229836847	229842346
chr2	230370907	230372136
chr2	230436380	230450255
chr2	230720455	230760115
chr2	230720455	230793331
chr2	230737806	230760115
chr2	230737806	230793331
chr2	230737806	230798897
chr2	231063849	231087181
chr2	231455088	231457800
chr2	231962239	232030080
chr2	232006244	232015671
chr2	232087486	232088040
chr2	232111196	232136719
chr2	232192273	232210405
chr2	232198526	232210405
chr2	232722553	232724691
chr2	232735154	232765979
chr2	233363076	233390483
chr2	233372044	233390483
chr2	233382520	233390483
chr2	233388256	233398449
chr2	233449958	233452060
chr2	233523390	233525562
chr2	235566487	235744839
chr2	235585724	235750488
chr2	235777621	235807331
chr2	235882295	235968623
chr2	235904987	235908906
chr2	235968461	235968957
chr2	235968461	235969687
chr2	235968461	235975448
chr2	236120191	236124199
chr2	23754178	23801497
chr2	237973091	237973776
chr2	238045681	238068165

chr2	238073782	238083354
chr2	238185009	238185287
chr2	238433312	238433695
chr2	239175448	239352918
chr2	239236592	239354414
chr2	23952469	23984831
chr2	239987515	239990182
chr2	239987515	240011696
chr2	240003008	240005295
chr2	240598397	240598623
chr2	24060813	24063782
chr2	241089187	241094432
chr2	241255602	241256183
chr2	241329610	241336098
chr2	241373083	241395657
chr2	241403827	241434381
chr2	241498383	241498638
chr2	241581011	241584725
chr2	241583542	241584725
chr2	24496319	24584560
chr2	24510968	24584560
chr2	24536714	24584560
chr2	24554388	24584560
chr2	24563903	24584560
chr2	24564294	24584560
chr2	24820876	24821516
chr2	25623517	25628384
chr2	25767582	25806337
chr2	25799384	25806337
chr2	26254429	26260441
chr2	26364301	26390816
chr2	26792130	26793897
chr2	27333654	27350632
chr2	27350313	27350632
chr2	27368143	27368371
chr2	27465410	27471396
chr2	28025225	28051648
chr2	28233142	28241393
chr2	28752037	28799433
chr2	28776850	28799433
chr2	28926467	28930149
chr2	28929578	28947161
chr2	28941460	28947161
chr2	29121373	29181697
chr2	29121373	29181726

chr2	29121373	29191683
chr2	29121373	29192257
chr2	29152684	29181697
chr2	29167475	29181697
chr2	29181571	29182167
chr2	30459551	30477448
chr2	30459551	30479911
chr2	30525586	30552649
chr2	30910947	30932194
chr2	30924118	30938829
chr2	30986834	30993007
chr2	31073062	31125253
chr2	31078933	31085023
chr2	31078933	31125253
chr2	31084899	31125253
chr2	31125153	31132712
chr2	3186022	3197017
chr2	31864905	31869952
chr2	31902459	31920910
chr2	31917925	31932778
chr2	31917925	31950717
chr2	31917925	31956598
chr2	31917925	31972301
chr2	31943004	31943383
chr2	3213541	3214248
chr2	32171286	32184809
chr2	32290482	32298350
chr2	32298233	32307190
chr2	32429145	32453954
chr2	32435495	32471151
chr2	32439507	32453954
chr2	32464954	32467739
chr2	32470270	32479617
chr2	32621968	32650233
chr2	32621968	32672384
chr2	32649096	32678922
chr2	32677960	32678922
chr2	32678452	32678922
chr2	33128431	33134960
chr2	3318856	3338149
chr2	3421880	3449477
chr2	3497670	3500993
chr2	3545700	3552308
chr2	3545700	3556904
chr2	3575888	3576460

chr2	36358903	36396787
chr2	36358903	36442735
chr2	36358903	36464655
chr2	36358903	36479694
chr2	36358903	36522313
chr2	36369107	36396787
chr2	36369107	36442735
chr2	36378356	36396787
chr2	36378356	36442735
chr2	36378356	36464655
chr2	36387971	36396787
chr2	36387971	36442735
chr2	36387971	36464655
chr2	36387971	36479694
chr2	36396613	36413019
chr2	36396613	36415769
chr2	36396613	36431221
chr2	37107257	37107527
chr2	37122505	37148939
chr2	37199703	37206248
chr2	37236095	37248426
chr2	37243862	37248426
chr2	37252974	37290999
chr2	37282541	37317179
chr2	37316236	37317179
chr2	37316236	37317899
chr2	38069177	38071310
chr2	38318534	38377260
chr2	38591529	38597354
chr2	39213399	39229588
chr2	40428472	40430301
chr2	40428472	40446648
chr2	40428474	40430304
chr2	42350722	42353343
chr2	42704196	42747398
chr2	43225796	43226264
chr2	43590823	43592431
chr2	43960540	43989690
chr2	43973606	43989690
chr2	44194197	44209327
chr2	44372715	44505730
chr2	44372715	44558013
chr2	44704282	44707487
chr2	45443836	45553730
chr2	45518354	45562756

chr2	45546316	45553730
chr2	45546316	45562756
chr2	45546731	45554607
chr2	45936718	46086362
chr2	46001403	46010814
chr2	46360884	46361595
chr2	46375689	46378172
chr2	46576086	46581167
chr2	47155170	47156425
chr2	47790926	47798204
chr2	48328516	48346751
chr2	48328516	48362707
chr2	48459716	48491170
chr2	48498492	48514967
chr2	53809213	53809737
chr2	54895272	54908352
chr2	55205604	55206376
chr2	55267568	55268247
chr2	55548855	55558968
chr2	55558774	55591695
chr2	55581566	55591575
chr2	58159649	58165874
chr2	61010655	61011905
chr2	61112521	61118116
chr2	61113769	61118116
chr2	61162841	61164540
chr2	61162841	61185618
chr2	61163051	61164540
chr2	61178980	61184800
chr2	61178980	61185618
chr2	61178980	61186497
chr2	61186616	61187029
chr2	61370320	61444912
chr2	61516341	61533903
chr2	61520390	61526521
chr2	61522610	61529159
chr2	61968968	62000982
chr2	61972852	62000982
chr2	61996822	62000982
chr2	62135830	62141360
chr2	62222211	62225414
chr2	62707040	62831158
chr2	62707040	62874532
chr2	62707163	62831158
chr2	62715300	62771392

chr2	63378385	63404088
chr2	63378385	63439855
chr2	63378385	63571626
chr2	63433744	63439855
chr2	63433744	63571626
chr2	63484603	63571626
chr2	63856305	63859391
chr2	63856305	63862928
chr2	63962057	64006514
chr2	63981645	64012676
chr2	63983863	64001383
chr2	65001447	65011545
chr2	65104733	65108803
chr2	65246184	65265175
chr2	68131276	68137433
chr2	68131276	68147567
chr2	69043731	69077488
chr2	69303548	69374113
chr2	69326170	69374113
chr2	69431901	69439508
chr2	69804532	69808189
chr2	69861277	69867801
chr2	69916120	69938339
chr2	70046502	70051305
chr2	70051202	70085903
chr2	70212734	70230854
chr2	70224553	70242008
chr2	70259359	70277874
chr2	70988534	70993775
chr2	71124205	71126142
chr2	71329539	71370005
chr2	71424649	71426774
chr2	72674531	72733118
chr2	72680613	72733118
chr2	72718102	72721164
chr2	73397512	73432291
chr2	73604915	73674905
chr2	73938909	73946888
chr2	73985979	74080591
chr2	73985979	74090047
chr2	73985979	74099612
chr2	74046281	74080591
chr2	74156417	74172752
chr2	74675524	74711243
chr2	75517516	75518208

chr2	75518055	75520268
chr2	75654735	75655278
chr2	77874979	77951533
chr2	84839148	84853410
chr2	84853313	84870785
chr2	85013935	85028056
chr2	85027888	85035847
chr2	85035015	85035847
chr2	85356966	85364951
chr2	85356966	85387225
chr2	85377343	85378023
chr2	85644947	85649203
chr2	85647929	85649203
chr2	85861180	85870258
chr2	86125880	86127140
chr2	86541116	86542312
chr2	86688231	86701949
chr2	86740926	86773435
chr2	8770709	8776892
chr2	8939820	8958642
chr2	8943186	8974435
chr2	8943186	8987761
chr2	9237832	9297445
chr2	9287003	9297445
chr2	9408112	9420100
chr2	9475986	9488392
chr2	9509978	9543226
chr2	95148884	95153259
chr2	95150196	95157439
chr2	9558951	9588328
chr2	9585183	9591515
chr2	96797011	96817502
chr2	97844144	97862039
chr2	98600582	98604191
chr2	99418827	99424899
chr2	99449335	99451501
chr2	9961045	9974900
chr3	10002716	10043864
chr3	100082485	100147061
chr3	101398860	101403047
chr3	10146513	10150818
chr3	101579841	101585511
chr3	101816794	101821750
chr3	101852084	101859465
chr3	107285319	107291930

chr3	111903965	111920419
chr3	111903965	111940645
chr3	113008170	113011165
chr3	113013271	113017484
chr3	11346646	11426926
chr3	11355420	11358612
chr3	11355420	11380052
chr3	11355420	11426926
chr3	11599891	11602022
chr3	11768554	11829864
chr3	119447017	119463472
chr3	119490718	119491492
chr3	119863418	120093593
chr3	119905754	120093593
chr3	119923372	120093593
chr3	119947267	120093593
chr3	120000551	120002239
chr3	120330618	120335953
chr3	120770757	120776664
chr3	121430388	121436275
chr3	121430388	121472164
chr3	121430388	121476733
chr3	121449314	121487404
chr3	121449314	121487509
chr3	121632589	121633066
chr3	121872536	121874121
chr3	122362967	122365466
chr3	122565858	122572310
chr3	122740257	122741113
chr3	122768700	122786490
chr3	12297868	12312453
chr3	123123335	123124343
chr3	123243811	123259589
chr3	123526895	123528474
chr3	124735092	124744203
chr3	124737567	124744203
chr3	124737914	124738239
chr3	124841382	124878767
chr3	124857231	124859446
chr3	12489783	12506835
chr3	12489783	12532773
chr3	124977858	125013117
chr3	12499480	12503784
chr3	12503261	12532773
chr3	12505153	12506835

chr3	12516610	12532773
chr3	12519058	12532773
chr3	125272455	125279247
chr3	125277725	125315053
chr3	125279123	125323349
chr3	12528887	12532773
chr3	125313307	125315053
chr3	125331157	125338911
chr3	126727077	126741487
chr3	126727077	126756858
chr3	126733077	126741487
chr3	126775794	126914750
chr3	126836555	126914750
chr3	128035378	128087808
chr3	128081213	128082574
chr3	128081213	128087808
chr3	128081253	128087808
chr3	128087705	128091582
chr3	128194558	128264781
chr3	128262127	128303432
chr3	128319519	128341889
chr3	128358216	128369276
chr3	128620256	128622409
chr3	128766987	128798069
chr3	128795359	128813471
chr3	128806371	128813471
chr3	129030702	129039986
chr3	129260400	129260597
chr3	129389888	129397140
chr3	129812216	129828508
chr3	129827802	129857405
chr3	129827802	129880562
chr3	129827802	129880594
chr3	129827802	129889104
chr3	129857249	129880559
chr3	129860939	129880559
chr3	129867009	129880559
chr3	129880308	129880562
chr3	129880308	129880594
chr3	130914349	130959341
chr3	130917311	130941699
chr3	131078483	131133956
chr3	131080426	131133956
chr3	131462748	131471279
chr3	132575771	132607025

chr3	132704197	132705666
chr3	13358025	13399861
chr3	134155135	134195182
chr3	134158120	134191974
chr3	134158120	134195182
chr3	134173115	134195182
chr3	136004254	136152738
chr3	136023026	136049361
chr3	136297951	136370334
chr3	136316940	136329984
chr3	136326802	136329984
chr3	136326802	136333650
chr3	136464880	136504913
chr3	136568764	136591549
chr3	136945582	136958201
chr3	136980310	136995556
chr3	136980465	137017166
chr3	138355193	138373076
chr3	138503746	138532570
chr3	138525489	138572984
chr3	138542690	138572932
chr3	138570317	138571353
chr3	138570317	138572984
chr3	139398860	139423114
chr3	139419556	139444510
chr3	141278341	141308407
chr3	141368620	141405887
chr3	141368620	141427821
chr3	141368620	141438365
chr3	141368644	141369946
chr3	141368644	141386937
chr3	141368644	141404031
chr3	141396258	141413639
chr3	141403926	141405887
chr3	141500887	141529802
chr3	141509567	141529802
chr3	141509567	141540609
chr3	141509567	141559995
chr3	14181559	14198294
chr3	141893811	141922063
chr3	141902135	141907274
chr3	141913651	141925803
chr3	142093060	142116373
chr3	142204216	142204788
chr3	142311702	142318903

chr3	142736378	142762313
chr3	142743484	142762313
chr3	142748371	142804630
chr3	143985542	143989832
chr3	143985542	143989837
chr3	143985542	143989847
chr3	143985542	144048812
chr3	148992542	148996904
chr3	148994146	149009402
chr3	149140003	149172224
chr3	149865820	149921227
chr3	15013588	15014174
chr3	15016151	15017272
chr3	150408179	150438354
chr3	151086501	151193666
chr3	151112301	151127984
chr3	151795738	151814300
chr3	15210801	15233973
chr3	15411244	15414162
chr3	15411244	15415942
chr3	154273721	154277718
chr3	154274461	154277718
chr3	155763496	155767669
chr3	155775700	155804173
chr3	155829855	155834041
chr3	155890667	155903960
chr3	155893517	155937881
chr3	156542164	156549003
chr3	156677656	156680555
chr3	156694019	156696424
chr3	156694021	156696025
chr3	15670109	15709736
chr3	156852767	156888397
chr3	15707923	15797144
chr3	157101934	157122975
chr3	15734690	15737233
chr3	15766233	15814309
chr3	15795222	15814309
chr3	158122102	158128292
chr3	158122102	158132263
chr3	158122102	158146775
chr3	158122102	158154049
chr3	158122102	158225733
chr3	158652095	158691648
chr3	158681994	158691648

chr3	158690162	158691648
chr3	159812757	159866281
chr3	160276583	160282613
chr3	160507692	160508269
chr3	16271658	16271995
chr3	16271658	16272321
chr3	16286341	16303592
chr3	16294855	16303592
chr3	167554682	167566894
chr3	167575917	167658326
chr3	167653260	167658326
chr3	169381186	169435769
chr3	170097171	170122744
chr3	170097171	170149244
chr3	170097171	170178938
chr3	170258754	170263429
chr3	170359698	170364466
chr3	170381018	170381341
chr3	170418387	170419292
chr3	170865317	170868390
chr3	172060709	172133546
chr3	172079353	172112590
chr3	172112451	172153841
chr3	172133470	172153841
chr3	172247532	172265368
chr3	172251259	172262083
chr3	172755482	172756503
chr3	172760160	172774022
chr3	172760160	172783909
chr3	172797219	172816837
chr3	17508473	17543465
chr3	17508473	17562142
chr3	17508473	17576549
chr3	17508473	17586466
chr3	17508473	17586557
chr3	17508473	17669756
chr3	17508473	17672705
chr3	17562018	17623913
chr3	17586352	17623913
chr3	179250742	179251577
chr3	179761111	179785873
chr3	180956328	180970358
chr3	182940358	182965753
chr3	183205875	183208665
chr3	183338799	183354223

chr3	183364254	183389779
chr3	183710153	183728851
chr3	183710168	183728851
chr3	183716346	183752253
chr3	183806865	183830692
chr3	183833491	183868024
chr3	183840569	183856551
chr3	183856431	183868060
chr3	183862696	183868060
chr3	184165109	184166265
chr3	184816773	184834742
chr3	184824146	184849195
chr3	185645341	185652168
chr3	186784696	186784935
chr3	186804276	186804724
chr3	186963784	186975791
chr3	186963784	186996673
chr3	187038394	187051346
chr3	188704369	188708393
chr3	188755057	188760282
chr3	188758631	188760282
chr3	190520489	190564353
chr3	191380158	191396033
chr3	192333701	192335464
chr3	192360427	192402696
chr3	192727180	192728554
chr3	192727180	192738983
chr3	192727180	192745356
chr3	193603499	193662962
chr3	194412198	194427252
chr3	194459470	194484410
chr3	195320700	195357784
chr3	195333027	195367736
chr3	195516841	195524896
chr3	195683751	195689438
chr3	195685817	195689438
chr3	195688532	195689438
chr3	195688532	195699207
chr3	195696360	195708841
chr3	195729055	195729739
chr3	195984111	195987127
chr3	196237446	196242640
chr3	196237791	196239735
chr3	196238918	196239546
chr3	196242121	196242561

chr3	196306942	196327591
chr3	196368027	196483891
chr3	196471639	196475312
chr3	196471639	196483891
chr3	196471978	196475312
chr3	196475230	196486628
chr3	196486532	196487578
chr3	196722414	196733815
chr3	196724042	196731092
chr3	196765920	196782833
chr3	196779419	196782833
chr3	196782625	196783566
chr3	196927795	196932023
chr3	196927795	196932535
chr3	196929683	196930796
chr3	196930812	196932023
chr3	197078437	197119530
chr3	197090911	197099723
chr3	197115926	197130978
chr3	197531939	197532522
chr3	197619739	197624720
chr3	197704541	197706680
chr3	197756425	197763638
chr3	197763465	197768587
chr3	197763465	197770244
chr3	197814907	197875775
chr3	197829563	197871462
chr3	197831959	197839397
chr3	197858833	197875775
chr3	197865422	197871462
chr3	197866111	197871462
chr3	197866111	197875775
chr3	197870159	197871462
chr3	197870159	197875775
chr3	198043231	198077288
chr3	19970439	19978403
chr3	19970547	19976169
chr3	19970547	19978403
chr3	20071363	20072459
chr3	20071583	20072459
chr3	20161108	20178347
chr3	23589733	23595250
chr3	23918576	23919069
chr3	24228937	24238081
chr3	25628846	25632520

chr3	25637214	25645470
chr3	25643693	25645470
chr3	27376715	27424152
chr3	27379248	27403301
chr3	27397683	27483627
chr3	28251711	28263380
chr3	28263290	28294529
chr3	28263290	28298350
chr3	28263290	28319625
chr3	29372874	29434915
chr3	29739719	29768350
chr3	29924296	29936196
chr3	3137259	3148187
chr3	31683779	31702508
chr3	3172505	3174261
chr3	31876432	31916416
chr3	32357372	32370026
chr3	32437954	32442013
chr3	32441839	32454542
chr3	32541092	32545965
chr3	32715991	32717237
chr3	32737409	32751025
chr3	32996795	32997344
chr3	33046119	33077237
chr3	33120362	33124579
chr3	33132554	33136150
chr3	33358966	33385728
chr3	33423530	33425741
chr3	33619602	33640143
chr3	33670642	33696933
chr3	33683265	33696933
chr3	37047518	37051710
chr3	37075023	37096766
chr3	37129062	37154168
chr3	37281984	37302332
chr3	37379041	37416724
chr3	37417042	37417492
chr3	38252392	38252789
chr3	38395563	38397045
chr3	38500089	38506968
chr3	39412061	39412244
chr3	40309870	40311571
chr3	40461406	40462248
chr3	40516547	40530316
chr3	41705056	41747865

chr3	41875054	41883952
chr3	42087103	42125614
chr3	4361975	4376389
chr3	4362038	4376389
chr3	44448560	44458364
chr3	44641899	44646657
chr3	44827094	44831018
chr3	44829948	44831018
chr3	44943293	44945292
chr3	45110472	45118618
chr3	4670757	4685206
chr3	47169168	47174263
chr3	47395086	47396217
chr3	47408490	47408754
chr3	47434924	47443091
chr3	47435007	47466447
chr3	47442871	47466447
chr3	47650690	47670717
chr3	47658557	47678311
chr3	47714414	47718430
chr3	47772467	47772936
chr3	47824766	47827477
chr3	47994423	47998879
chr3	47998637	48065951
chr3	48290272	48298523
chr3	48501136	48511302
chr3	48548138	48550234
chr3	48548531	48550234
chr3	49032182	49033212
chr3	49126190	49126364
chr3	49295545	49300691
chr3	49335019	49335596
chr3	49368427	49373342
chr3	49373218	49375591
chr3	49375433	49376178
chr3	49699475	49700709
chr3	49967197	50058562
chr3	49967332	49975392
chr3	50046715	50054395
chr3	50115980	50117053
chr3	50175095	50175416
chr3	50347575	50350022
chr3	51137442	51146630
chr3	51146548	51174756
chr3	51470928	51492488

chr3	51470928	51492513
chr3	51540363	51590559
chr3	51541497	51590559
chr3	51541588	51590559
chr3	51572821	51590559
chr3	51681341	51684634
chr3	51684412	51684884
chr3	5192269	5208263
chr3	5195208	5215903
chr3	51960666	51960865
chr3	52411028	52414587
chr3	52490231	52490678
chr3	52563282	52564233
chr3	52563282	52579199
chr3	52678499	52679726
chr3	52730324	52741499
chr3	52743351	52758919
chr3	52943352	53043254
chr3	52954316	52971649
chr3	52969100	53043254
chr3	53342143	53347532
chr3	55888388	55950560
chr3	56560957	56571302
chr3	56565637	56567053
chr3	56569312	56571302
chr3	56572336	56594028
chr3	56592969	56598300
chr3	56623643	56628614
chr3	56633027	56633966
chr3	57007181	57035176
chr3	57249359	57269751
chr3	57256956	57276459
chr3	57569686	57584007
chr3	57825760	57831530
chr3	57864806	57865292
chr3	58498712	58501709
chr3	58937647	58966822
chr3	61617661	61748982
chr3	61748877	61769416
chr3	61989624	62015932
chr3	62000751	62078258
chr3	62003348	62026463
chr3	62009467	62078258
chr3	62218850	62220439
chr3	63826823	63839773

chr3	63834062	63838499
chr3	63834062	63839773
chr3	63864897	63898497
chr3	63979914	63999651
chr3	63982938	63999651
chr3	64004325	64006068
chr3	65609743	65622088
chr3	67400730	67409031
chr3	67400730	67409034
chr3	67559500	67609596
chr3	67609454	67637839
chr3	67667257	67671004
chr3	69089811	69102056
chr3	69099557	69102056
chr3	71453115	71493555
chr3	71468695	71493555
chr3	71493425	71501988
chr3	72708843	72750836
chr3	72781304	72793036
chr3	72781304	72842402
chr3	72832368	72842425
chr3	72832368	72844453
chr3	72841044	72844453
chr3	73046356	73047356
chr3	73059036	73065575
chr3	81642780	81753280
chr3	81670837	81753280
chr3	81699972	81705613
chr3	8742458	8753224
chr3	8752883	8768591
chr3	9383198	9384825
chr3	9390289	9391826
chr3	93995877	94003908
chr3	9421322	9435906
chr3	9421478	9453868
chr3	9689963	9702064
chr3	96952114	96987993
chr3	96972606	96987993
chr3	96987329	96990508
chr3	97207775	97244287
chr3	97877578	97886767
chr3	97933693	97943570
chr3	98521132	98522920
chr3	98876666	98881767
chr3	98881539	98889424

chr3	98881539	98892606
chr3	9969718	9981747
chr4	10097710	10108395
chr4	101193558	101196116
chr4	101195915	101250373
chr4	102304316	102315739
chr4	102304316	102315742
chr4	102304316	102324563
chr4	102510879	102529914
chr4	102714437	102730736
chr4	102714437	102734571
chr4	102714437	102736435
chr4	102722870	102730736
chr4	103195265	103195919
chr4	103436934	103453812
chr4	105177591	105261320
chr4	105261147	105399164
chr4	105424195	105434161
chr4	105424195	105441148
chr4	105686691	105689280
chr4	106095481	106197014
chr4	107895309	107910609
chr4	108018445	108019666
chr4	109494614	109500054
chr4	109499903	109533685
chr4	109516567	109521626
chr4	109531384	109539667
chr4	109648483	109664394
chr4	109708454	109718759
chr4	110425960	110520366
chr4	1108342	1111821
chr4	112808902	112818264
chr4	113609151	113679496
chr4	118620413	118628226
chr4	119457970	119462979
chr4	119488644	119497903
chr4	119493332	119512970
chr4	120903260	120907557
chr4	1212786	1241519
chr4	121820541	121835283
chr4	1228198	1241515
chr4	122876320	122892296
chr4	122927633	122984615
chr4	122927636	122947488
chr4	122927636	122979386

chr4	122933438	122949594
chr4	122935713	122952270
chr4	123019161	123028395
chr4	124500895	124522204
chr4	124500895	124556510
chr4	124565	131505
chr4	124663284	124678905
chr4	127669038	127687867
chr4	127982938	128030672
chr4	128017362	128021238
chr4	128021	212781
chr4	128162193	128282740
chr4	128292933	128483487
chr4	128460326	128470549
chr4	128816366	128852268
chr4	129039040	129093148
chr4	129724509	129727033
chr4	130787	132602
chr4	131409	218778
chr4	13266911	13381594
chr4	133075343	133096883
chr4	13332384	13381594
chr4	13371794	13426354
chr4	13376544	13387694
chr4	13376544	13425227
chr4	13376544	13428299
chr4	13425109	13460828
chr4	139043073	139045389
chr4	139116648	139125329
chr4	139125163	139139497
chr4	139125163	139151980
chr4	139125163	139168042
chr4	139137629	139139497
chr4	139137629	139151980
chr4	139533164	139548119
chr4	140525419	140549964
chr4	142383961	142405324
chr4	142402937	142433830
chr4	143543469	143543972
chr4	143858961	143912638
chr4	143861141	143979952
chr4	143912539	144004680
chr4	145041316	145096111
chr4	145886032	145892415
chr4	146488065	146510049

chr4	146509171	146510049
chr4	147628360	147634581
chr4	147633764	147634558
chr4	147633764	147634581
chr4	147742039	147822957
chr4	147854675	147866816
chr4	147854675	147875150
chr4	147854675	147881932
chr4	147854675	147966839
chr4	147857552	147891595
chr4	147906637	147913178
chr4	148063147	148072179
chr4	148064415	148072179
chr4	150415437	150439097
chr4	151415723	151482648
chr4	151424094	151482648
chr4	151454700	151458433
chr4	152382188	152412529
chr4	152411302	152451776
chr4	152411302	152469988
chr4	152809672	152888307
chr4	153315484	153358622
chr4	15550823	15601421
chr4	15567786	15569264
chr4	15627884	15659922
chr4	156850220	156854606
chr4	158680466	158708552
chr4	158851320	158868349
chr4	159238808	159304473
chr4	159304392	159330010
chr4	16238770	16323036
chr4	163158224	163164391
chr4	165079716	165103096
chr4	165218024	165238899
chr4	165219643	165299656
chr4	165219933	165277543
chr4	165238777	165240464
chr4	168889579	168925078
chr4	169002070	169019854
chr4	169149240	169156679
chr4	169477123	169484514
chr4	173195594	173248440
chr4	173224309	173248440
chr4	173232451	173248440
chr4	176148132	176179632

chr4	17623600	17625845
chr4	176699162	176729746
chr4	176708968	176729746
chr4	176711498	176731524
chr4	176711498	176740765
chr4	176727777	176731524
chr4	176727777	176740765
chr4	177353307	177358258
chr4	17794930	17845901
chr4	17804403	17805225
chr4	17961902	18009978
chr4	182769280	182915575
chr4	182837570	182915575
chr4	182874569	182915575
chr4	182877510	182894605
chr4	182877510	182915575
chr4	182891288	182915575
chr4	182891348	182894605
chr4	182891348	182915575
chr4	182891402	182894605
chr4	182891402	182915575
chr4	183142932	183193708
chr4	183173557	183193708
chr4	183183402	183193708
chr4	183188263	183209025
chr4	184427520	184429070
chr4	184775369	184776983
chr4	185188946	185191212
chr4	185188950	185191212
chr4	185213360	185267155
chr4	185213360	185346650
chr4	185247293	185264498
chr4	188101055	188105429
chr4	188101055	188105590
chr4	1892165	1893909
chr4	1892165	1901251
chr4	1892165	1904378
chr4	1892165	1918623
chr4	1892165	1935262
chr4	1900625	1942565
chr4	1901094	1904378
chr4	1912090	1918623
chr4	1974862	1979189
chr4	1974862	1982551
chr4	201080	211885

chr4	201080	212781
chr4	20393349	20394605
chr4	2063759	2064664
chr4	20704465	20727397
chr4	20716106	20727397
chr4	211658	212781
chr4	2186788	2213126
chr4	22327459	22335167
chr4	22335040	22392690
chr4	22341019	22392690
chr4	22375729	22392690
chr4	22389087	22424348
chr4	22397990	22402799
chr4	22401391	22402799
chr4	22447439	22478233
chr4	22461736	22478233
chr4	2494563	2497121
chr4	2596083	2639859
chr4	2596154	2646832
chr4	2625261	2626577
chr4	2625261	2631169
chr4	2625261	2639859
chr4	2625261	2646832
chr4	2638816	2639859
chr4	2638816	2646832
chr4	2655027	2663288
chr4	2655027	2672372
chr4	2657802	2683115
chr4	26748410	26769852
chr4	2937751	2938792
chr4	3080922	3107423
chr4	3086938	3087498
chr4	30920152	30950215
chr4	3314790	3318051
chr4	3314790	3343053
chr4	3342936	3343394
chr4	36145345	36151044
chr4	37902002	37929982
chr4	38035587	38073387
chr4	38611323	38614708
chr4	38986335	38994424
chr4	39605070	39612110
chr4	39731010	39745810
chr4	39837477	39842056
chr4	39926774	39931629

chr4	39926774	39933433
chr4	40152779	40154267
chr4	40881283	40893411
chr4	41013582	41018441
chr4	41093023	41100750
chr4	41142212	41143142
chr4	41142986	41145230
chr4	41142986	41162074
chr4	41142986	41177690
chr4	41257009	41257587
chr4	41257009	41257590
chr4	41374584	41524478
chr4	41438162	41524478
chr4	41453564	41524478
chr4	41494535	41551395
chr4	41524408	41633808
chr4	41978133	42001780
chr4	4236875	4246343
chr4	4298615	4300097
chr4	435769	472701
chr4	4542527	4649542
chr4	4611493	4649542
chr4	4648976	4653757
chr4	4648976	4690036
chr4	47545402	47563765
chr4	48377895	48394653
chr4	48684672	48687160
chr4	48684672	48780173
chr4	505717	506866
chr4	51860542	51877854
chr4	51860542	51891851
chr4	51863436	51887244
chr4	51863436	51893227
chr4	51863436	51914078
chr4	52873815	52885866
chr4	53145332	53186486
chr4	55475171	55489465
chr4	55877563	55890386
chr4	56196343	56272492
chr4	56403039	56406618
chr4	56459371	56459618
chr4	56478378	56480977
chr4	56500535	56505509
chr4	56596499	56604222
chr4	56963455	56964526

chr4	56978908	56990898
chr4	6333561	6348746
chr4	64926591	65008912
chr4	673302	674317
chr4	67720421	67730170
chr4	6996324	6997194
chr4	7004924	7006602
chr4	70911921	70959040
chr4	70942822	70959040
chr4	73053491	73068133
chr4	73168928	73177533
chr4	73177379	73191074
chr4	73177379	73192339
chr4	73177379	73235080
chr4	74173408	74281550
chr4	74174505	74261209
chr4	75482478	75494179
chr4	77040620	77066405
chr4	77048448	77066405
chr4	79085226	79086262
chr4	80286324	80287481
chr4	80286324	80295627
chr4	80287336	80362884
chr4	80295558	80335879
chr4	80295558	80362884
chr4	80301282	80362884
chr4	80321340	80362884
chr4	80335692	80362884
chr4	80335743	80362884
chr4	80362753	80376917
chr4	80566341	80583183
chr4	80790374	80870135
chr4	82354048	82359639
chr4	82427189	82429412
chr4	82427591	82428017
chr4	82961389	82979006
chr4	82970326	82981475
chr4	83262588	83273617
chr4	84713158	84716992
chr4	84829003	84879494
chr4	84836928	84879494
chr4	84849901	84879494
chr4	84879405	84932363
chr4	84894824	84897010
chr4	84896910	84898597

chr4	84896910	84912319
chr4	87020505	87047594
chr4	87159457	87185799
chr4	87371411	87374830
chr4	88265000	88271258
chr4	882839	883314
chr4	88781164	88855308
chr4	88990972	89009078
chr4	89008950	89020669
chr4	89008950	89029649
chr4	97703858	97712814
chr4	98105952	98134425
chr4	99908700	99923185
chr4	99928541	99930621
chr5	10213490	10227647
chr5	10223958	10227647
chr5	10250297	10260911
chr5	10260911	10261518
chr5	102990271	103029089
chr5	103190841	103212007
chr5	103265907	103276397
chr5	103275896	103276397
chr5	10618332	10619378
chr5	108157107	108348530
chr5	108184941	108186247
chr5	108186116	108227601
chr5	108186116	108294904
chr5	108224120	108344026
chr5	108301975	108348530
chr5	108341926	108348530
chr5	108341926	108367953
chr5	108768092	108770709
chr5	108768092	108815404
chr5	108770499	108798389
chr5	108798123	108799985
chr5	108798123	108820524
chr5	108832769	108878440
chr5	108897658	108898132
chr5	109698435	109755456
chr5	109706482	109716264
chr5	109713495	109755456
chr5	109713519	109796127
chr5	109729310	109774965
chr5	109767534	109804277
chr5	109781395	109795046

chr5	109781395	109796803
chr5	109781395	109804277
chr5	109781395	109830980
chr5	109817272	109830980
chr5	111416505	111449203
chr5	112900725	112902518
chr5	112981041	113001674
chr5	112985834	113013536
chr5	112992153	113004077
chr5	115200163	115222008
chr5	115204561	115222008
chr5	115831693	115837764
chr5	115831693	115840612
chr5	115852978	115870128
chr5	115866669	115913513
chr5	115866669	115968136
chr5	116131183	116134178
chr5	116134067	116279151
chr5	116291516	116298392
chr5	116291516	116303683
chr5	116292450	116318300
chr5	116303529	116449817
chr5	116446163	116449817
chr5	118928536	118946328
chr5	120605176	120618565
chr5	120617094	120642455
chr5	122931860	122946159
chr5	122995478	123002004
chr5	122998207	123002004
chr5	123346604	123399284
chr5	123532568	123557564
chr5	126899185	126918227
chr5	1278635	1282624
chr5	128138823	128139824
chr5	129104955	129142006
chr5	131255094	131316144
chr5	131313848	131316144
chr5	131359222	131359547
chr5	131508070	131532194
chr5	131548046	131624334
chr5	131592382	131624334
chr5	131695404	131699002
chr5	131744563	131747515
chr5	132927120	132937740
chr5	134074764	134174022

chr5	134157706	134174022
chr5	134158454	134174007
chr5	134158454	134174514
chr5	134197324	134206131
chr5	134197775	134206131
chr5	134205921	134221412
chr5	134380718	134390363
chr5	134388324	134390363
chr5	134390224	134393891
chr5	134777573	134785515
chr5	134887694	134922115
chr5	134908829	134970092
chr5	134910334	134975324
chr5	134969938	134975324
chr5	135052453	135075388
chr5	135074416	135174172
chr5	135147082	135174172
chr5	135172499	135174172
chr5	136147835	136163391
chr5	137677544	137720863
chr5	137952627	137963176
chr5	138017994	138018514
chr5	138168112	138168469
chr5	138319218	138331686
chr5	138372683	138381590
chr5	138556893	138557401
chr5	138858311	138904441
chr5	138947404	138951884
chr5	138951360	138951787
chr5	139042600	139051046
chr5	139121034	139128052
chr5	139121034	139133471
chr5	139278326	139280058
chr5	139325295	139326284
chr5	139585412	139600435
chr5	139600371	139627059
chr5	139620442	139623461
chr5	139623367	139627059
chr5	139880855	139900326
chr5	140167364	140167863
chr5	140189063	140194652
chr5	140438502	140449305
chr5	140438502	140464776
chr5	140472238	140487060
chr5	140472238	140491290

chr5	140696201	140696499
chr5	141354470	141357621
chr5	141354470	141362556
chr5	141369870	141372809
chr5	141409267	141410620
chr5	14147952	14280436
chr5	141571436	141571925
chr5	141581914	141583615
chr5	141978302	141987975
chr5	14219408	14270899
chr5	14219408	14316743
chr5	14222781	14280436
chr5	14242763	14280436
chr5	14242763	14287063
chr5	14278309	14304592
chr5	142885297	142932739
chr5	14293011	14343806
chr5	14311294	14336727
chr5	143120987	143173155
chr5	143147230	143173155
chr5	143352724	143400852
chr5	145665253	145765000
chr5	145703974	145796547
chr5	145703974	145823134
chr5	145760248	145765000
chr5	145764930	145776430
chr5	145817893	145831047
chr5	146098155	146105113
chr5	146160373	146161564
chr5	146168127	146175370
chr5	146223402	146243879
chr5	14673641	14681624
chr5	14673641	14693094
chr5	14678680	14681624
chr5	14678680	14693396
chr5	14687520	14693094
chr5	148394713	148411238
chr5	148541001	148550262
chr5	148548667	148677235
chr5	148636988	148677235
chr5	149501014	149520388
chr5	149525044	149529719
chr5	149525044	149543898
chr5	149817612	149820606
chr5	150387942	150390023

chr5	150532944	150551894
chr5	151452342	151479489
chr5	151458787	151472722
chr5	151786571	151792201
chr5	151786571	151803996
chr5	151786571	151804446
chr5	151786571	151812977
chr5	151786602	151803996
chr5	154026398	154034946
chr5	154029488	154034946
chr5	154030531	154034967
chr5	154033790	154034946
chr5	154755643	154811640
chr5	154865441	154867792
chr5	154950820	154967237
chr5	15599061	15616072
chr5	15615982	15634464
chr5	157751389	157755192
chr5	157754003	157755192
chr5	160010499	160023692
chr5	160028505	160043169
chr5	160064931	160065766
chr5	160074988	160080770
chr5	160089505	160094065
chr5	160252882	160255708
chr5	160252882	160259771
chr5	160280524	160305126
chr5	163456928	163457633
chr5	16676030	16677113
chr5	168395181	168397800
chr5	168412004	168422097
chr5	168488601	168498046
chr5	168557422	168568998
chr5	170878139	170892553
chr5	170892386	171028989
chr5	170916464	171028989
chr5	170918712	170983245
chr5	170919440	171028989
chr5	170924356	170933053
chr5	171132233	171242820
chr5	171158333	171242820
chr5	171171232	171205612
chr5	171196728	171213738
chr5	171213618	171242820
chr5	171213618	171265847

chr5	171845505	171872990
chr5	171910571	171997051
chr5	171914342	172003310
chr5	172211966	172234358
chr5	172230897	172234358
chr5	172768748	172770306
chr5	172770346	172770585
chr5	172770585	172771189
chr5	172914713	172915690
chr5	173163724	173164296
chr5	174524061	174543516
chr5	176347520	176348356
chr5	176919169	176943488
chr5	176943334	176954740
chr5	176951473	176963843
chr5	177079381	177086266
chr5	177169393	177204292
chr5	177536357	177539042
chr5	177536357	177539194
chr5	177619077	177627614
chr5	178204836	178205046
chr5	178208735	178231523
chr5	178860127	178860974
chr5	179569301	179575648
chr5	179615545	179617911
chr5	179617020	179618323
chr5	179617988	179618259
chr5	179704154	179709059
chr5	179704154	179710065
chr5	179704154	179720560
chr5	179708380	179708946
chr5	179766741	179768826
chr5	179811573	179816065
chr5	179841906	179844252
chr5	179841906	179851424
chr5	179847977	179851424
chr5	179852019	179853437
chr5	179853196	179853437
chr5	179879218	179879627
chr5	179970199	179970506
chr5	180013269	180040647
chr5	180040452	180047062
chr5	180261683	180287067
chr5	180495651	180564593
chr5	180541159	180553471

chr5	180573987	180576676
chr5	180573987	180576684
chr5	180849153	180852060
chr5	180850581	180851493
chr5	1814338	1816054
chr5	21491320	21501826
chr5	225908	240476
chr5	254392	256455
chr5	272710	314596
chr5	27407042	27477801
chr5	27472382	27475695
chr5	27475534	27489386
chr5	311292	314596
chr5	31421271	31424530
chr5	31483429	31495372
chr5	31538480	31541402
chr5	32121494	32126636
chr5	32121494	32143880
chr5	32134563	32143880
chr5	32139551	32143880
chr5	32143748	32170768
chr5	32143748	32171945
chr5	33440975	33448731
chr5	33812464	33881480
chr5	33881118	33891841
chr5	34020586	34052110
chr5	34023908	34031804
chr5	34733115	34757598
chr5	34762196	34796027
chr5	34762196	34814669
chr5	34770627	34796027
chr5	34792905	34796027
chr5	34915686	34924975
chr5	35641584	35654726
chr5	37095431	37125409
chr5	37243007	37247745
chr5	37291812	37299568
chr5	37579029	37605238
chr5	38481298	38530666
chr5	38527123	38530666
chr5	39002534	39026340
chr5	39002534	39030572
chr5	40728344	40771863
chr5	40728344	40775503
chr5	40746797	40771863

chr5	40746797	40775503
chr5	40746797	40777586
chr5	40764513	40780338
chr5	40827699	40832573
chr5	40827699	40834606
chr5	41740464	41803163
chr5	41784569	41807438
chr5	41850029	41862724
chr5	41929824	41934308
chr5	42687514	42700002
chr5	43122043	43139309
chr5	43157108	43161931
chr5	43453657	43456789
chr5	43675510	43704342
chr5	43675510	43704400
chr5	43700118	43704400
chr5	50274987	50411383
chr5	50382570	50411383
chr5	50399106	50403454
chr5	50399106	50405852
chr5	50399106	50411383
chr5	50399106	50428227
chr5	50399231	50411383
chr5	53603451	53604895
chr5	54137646	54171928
chr5	5447840	5457773
chr5	55322326	55324753
chr5	55691071	55691292
chr5	55697845	55704434
chr5	55910531	55954984
chr5	57200199	57231321
chr5	57241433	57247215
chr5	60599074	60628363
chr5	60686961	60687838
chr5	61360123	61473037
chr5	614469	624579
chr5	647804	655584
chr5	65170616	65198360
chr5	65242103	65276268
chr5	65260599	65334458
chr5	65291328	65334458
chr5	65329377	65334458
chr5	65409518	65473952
chr5	65451474	65452221
chr5	65451474	65473655

chr5	65451474	65473715
chr5	65521474	65554898
chr5	65542553	65542848
chr5	65542801	65554933
chr5	65624193	65624500
chr5	65926581	65994864
chr5	65926581	66038482
chr5	65926656	65994864
chr5	65931179	65994864
chr5	65975986	65994864
chr5	65988634	65995353
chr5	6651841	6665639
chr5	6653446	6662966
chr5	66788669	66822898
chr5	6739731	6756188
chr5	69113227	69114403
chr5	69282889	69311204
chr5	69419370	69441643
chr5	69495278	69509819
chr5	69504176	69520101
chr5	69513947	69553773
chr5	70946065	70953279
chr5	71580957	71604468
chr5	72283504	72297702
chr5	72283504	72314158
chr5	72290949	72297702
chr5	72358202	72359697
chr5	72861791	72865729
chr5	73747065	73749984
chr5	73921729	73923164
chr5	74721602	74736985
chr5	748403	837480
chr5	75534827	75552591
chr5	77098654	77118521
chr5	77116855	77118521
chr5	77463094	77467470
chr5	77691430	77708347
chr5	78134922	78165672
chr5	78388836	78475654
chr5	78415519	78429468
chr5	78475503	78483051
chr5	78592144	78632368
chr5	79439009	79513094
chr5	79619611	79623355
chr5	79632420	79669028

chr5	79980263	79990663
chr5	79983592	79990236
chr5	80202885	80205360
chr5	80501204	80522138
chr5	80622407	80638009
chr5	80672243	80673126
chr5	80675002	80728965
chr5	80787564	80843546
chr5	80848447	80854316
chr5	80873115	80875955
chr5	81615472	81657076
chr5	81973947	81987678
chr5	81973947	82058602
chr5	81981308	82178587
chr5	81987558	82129769
chr5	82035308	82058602
chr5	82252561	82254325
chr5	82252561	82254389
chr5	82252561	82261055
chr5	83104909	83124886
chr5	87268829	87333337
chr5	87331347	87338088
chr5	87362550	87390929
chr5	87389392	87390929
chr5	88202971	88207180
chr5	88217749	88228528
chr5	88220562	88245792
chr5	88220589	88228528
chr5	88220589	88240916
chr5	88282041	88292883
chr5	883473	883937
chr5	883937	886704
chr5	93819612	93881688
chr5	93958776	93994504
chr5	93958776	94057203
chr5	94050730	94060348
chr5	94050730	94111301
chr5	95580891	95586733
chr5	95888908	95919545
chr5	95906522	95932713
chr5	95913770	95923217
chr5	95913770	95932713
chr5	96318472	96319355
chr5	97668550	97713497
chr6	100589633	100770473

chr6	100679617	100767236
chr6	100798712	100806070
chr6	100798712	100848333
chr6	100848147	100849096
chr6	101382723	101399392
chr6	104765363	104772074
chr6	10528630	10528841
chr6	105373368	105376232
chr6	106201971	106217601
chr6	107039748	107040524
chr6	107044345	107099296
chr6	107087611	107099296
chr6	107098550	107104512
chr6	107212108	107225064
chr6	10728639	10730849
chr6	107324555	107334332
chr6	107503656	107518388
chr6	107904628	108042748
chr6	107921796	107956106
chr6	107929414	107931314
chr6	108210677	108214622
chr6	108319616	108324048
chr6	108323093	108402054
chr6	108443136	108447296
chr6	108851643	108854485
chr6	10898014	10902963
chr6	109140420	109146937
chr6	109737092	109738453
chr6	110100525	110108681
chr6	110667433	110686021
chr6	110736032	110773937
chr6	110746125	110779547
chr6	110879986	110887591
chr6	110879986	110888983
chr6	110879986	110890356
chr6	110886876	110890356
chr6	110887504	110893662
chr6	111005987	111015856
chr6	111025402	111026547
chr6	111472012	111474476
chr6	111472012	111474479
chr6	111570084	111576470
chr6	113940894	113960018
chr6	11565665	11567535
chr6	116652003	116660778

chr6	11717308	11723500
chr6	11766273	11770089
chr6	119188404	119196657
chr6	12015525	12020418
chr6	125045210	125046661
chr6	125045210	125082923
chr6	126008391	126174911
chr6	127280230	127287007
chr6	127314815	127330948
chr6	128184431	128266607
chr6	128240034	128293355
chr6	128240034	128304828
chr6	128304667	128322310
chr6	130287819	130313093
chr6	130288226	130313093
chr6	130311290	130313093
chr6	130312977	130334845
chr6	130312977	130341122
chr6	130385172	130387592
chr6	131607927	131628108
chr6	131949601	131949948
chr6	132503445	132508345
chr6	134982372	135002842
chr6	134993757	135010759
chr6	135037810	135042126
chr6	135039572	135040657
chr6	135039572	135042118
chr6	135290422	135346796
chr6	135318518	135346796
chr6	136694139	136698751
chr6	136720449	136784719
chr6	138404360	138404597
chr6	138441982	138572061
chr6	138473305	138481490
chr6	138496218	138505638
chr6	138496218	138571813
chr6	138496218	138572061
chr6	138496218	138574844
chr6	138496218	138685683
chr6	138571709	138572061
chr6	138785226	138788439
chr6	138941334	138951185
chr6	14131519	14135909
chr6	142820032	142837047
chr6	142836934	142870339

chr6	142836934	142918628
chr6	142836934	142923868
chr6	142836934	142926022
chr6	143485151	143505457
chr6	143687678	143712183
chr6	143687678	143749065
chr6	143707981	143712183
chr6	144291736	144308570
chr6	144291736	144357729
chr6	144437564	144437862
chr6	144490038	144493456
chr6	145671238	145686296
chr6	145864387	145894977
chr6	145881627	145894977
chr6	149005302	149021481
chr6	149369908	149380143
chr6	149378017	149380143
chr6	149379410	149399184
chr6	149676254	149695221
chr6	149676259	149678745
chr6	149727069	149738778
chr6	149770276	149802416
chr6	149772047	149802369
chr6	151009818	151057364
chr6	151034492	151057364
chr6	15246380	15374252
chr6	15246380	15410365
chr6	154406260	154433507
chr6	154406392	154442290
chr6	154810014	154833409
chr6	154818478	154860998
chr6	154830921	154833409
chr6	155257771	155311339
chr6	156799659	156829421
chr6	156799664	156901525
chr6	156815684	156829421
chr6	156826376	156829421
chr6	156826684	156829421
chr6	156829226	156829705
chr6	156829226	156834824
chr6	156829226	156835950
chr6	156955585	157110561
chr6	157026295	157084905
chr6	157036834	157084905
chr6	157036834	157110561

chr6	157079793	157084905
chr6	157148623	157150853
chr6	157592987	157594446
chr6	157592987	157595723
chr6	157592987	157595824
chr6	157628348	157633148
chr6	157862133	157875176
chr6	158143055	158162152
chr6	158429735	158431663
chr6	158442710	158461729
chr6	158457127	158461729
chr6	158573419	158610003
chr6	159784665	159786939
chr6	161022009	161034449
chr6	161022009	161049979
chr6	161165576	161166417
chr6	1624005	1624540
chr6	163429806	163455421
chr6	163455278	163462300
chr6	163478779	163490557
chr6	16480026	16657901
chr6	16585779	16753402
chr6	16657775	16754746
chr6	167022408	167040339
chr6	169570072	169602321
chr6	169582452	169664286
chr6	169602218	169689308
chr6	169634427	169660753
chr6	169664165	169689308
chr6	169667135	169689308
chr6	169672254	169689308
chr6	170375630	170395579
chr6	170488396	170549113
chr6	17462995	17508900
chr6	17465546	17513954
chr6	17465575	17513954
chr6	17507168	17519326
chr6	17624560	17637289
chr6	17869045	17873437
chr6	18185771	18188002
chr6	18185771	18191381
chr6	18197056	18201657
chr6	18200438	18201657
chr6	1917939	1930230
chr6	1930102	1952129

chr6	20091553	20102412
chr6	20479845	20482949
chr6	20781144	20805719
chr6	20781144	20874430
chr6	2115770	2127833
chr6	2263600	2272777
chr6	2263600	2273417
chr6	2382853	2399037
chr6	2397220	2399037
chr6	24408407	24423728
chr6	24416109	24418578
chr6	24705363	24716324
chr6	26158134	26158559
chr6	26462796	26463525
chr6	28162608	28164901
chr6	2833873	2840594
chr6	2895390	2898048
chr6	29888466	29943026
chr6	29889327	29943543
chr6	29890263	29944616
chr6	30021666	30035983
chr6	30032988	30035983
chr6	30289361	30296237
chr6	303729	311962
chr6	30546695	30548902
chr6	30558149	30561508
chr6	30608778	30614427
chr6	3076763	3077935
chr6	3076763	3081116
chr6	3076763	3083313
chr6	3076763	3085408
chr6	3076763	3089657
chr6	31271598	31356442
chr6	31412324	31507137
chr6	31534783	31535366
chr6	3157406	3226669
chr6	31784737	31785245
chr6	31795272	31795545
chr6	31810098	31811985
chr6	31837235	31839618
chr6	31966013	31966690
chr6	32978388	32979806
chr6	33670815	33671140
chr6	33774804	33786774
chr6	3410187	3438555

chr6	34288739	34341972
chr6	34341861	34343032
chr6	34341861	34370492
chr6	34421829	34424668
chr6	34646600	34649830
chr6	34646600	34679402
chr6	34654624	34688933
chr6	34967238	34972643
chr6	35383576	35411217
chr6	35410213	35420281
chr6	35468439	35468768
chr6	35468439	35468777
chr6	35597247	35646850
chr6	35619095	35646850
chr6	35619095	35670563
chr6	35619095	35672756
chr6	35619095	35672759
chr6	35619095	35680422
chr6	35637013	35646366
chr6	35637013	35672759
chr6	35637013	35680422
chr6	35642719	35672759
chr6	35869943	35870280
chr6	35886723	35891000
chr6	36072872	36078791
chr6	36072872	36080086
chr6	36083030	36096066
chr6	36095794	36107628
chr6	36130618	36132679
chr6	36210384	36222265
chr6	36213859	36218010
chr6	36465600	36470257
chr6	36465600	36475062
chr6	36601707	36605189
chr6	3727550	3739161
chr6	37312879	37317206
chr6	37359182	37369218
chr6	37368483	37391454
chr6	3737078	3739161
chr6	3737078	3742297
chr6	38061592	38062525
chr6	38077133	38082457
chr6	38078039	38082457
chr6	38116571	38116843
chr6	38277564	38288461

chr6	38277564	38345093
chr6	38577599	38600008
chr6	41103942	41124820
chr6	41124484	41140955
chr6	41590347	41591189
chr6	41787708	41789336
chr6	41796112	41916939
chr6	41803805	41856729
chr6	41803805	41916939
chr6	41856625	41871637
chr6	41870252	41871637
chr6	41871297	41871637
chr6	41871563	41874239
chr6	41871563	41907287
chr6	41871563	41916939
chr6	41891793	41907287
chr6	41891793	41909522
chr6	41930768	41931362
chr6	42564199	42594304
chr6	42580293	42606651
chr6	42592152	42594304
chr6	42617408	42632387
chr6	42663277	42666245
chr6	42663277	42679832
chr6	42781838	42823003
chr6	42781838	42830172
chr6	42810305	42830172
chr6	42880860	42886470
chr6	42883450	42886470
chr6	42885973	42886470
chr6	42964293	42975038
chr6	42972882	42975038
chr6	43026036	43029190
chr6	43178882	43179066
chr6	43225646	43226395
chr6	43527633	43528886
chr6	43527633	43528928
chr6	43587271	43587652
chr6	43599427	43601091
chr6	43636380	43640630
chr6	43671839	43675617
chr6	44099734	44116629
chr6	44113872	44116629
chr6	44113886	44116629
chr6	44114076	44116629

chr6	44300739	44301155
chr6	44829763	45106006
chr6	44950042	45020632
chr6	45422592	45513653
chr6	45422592	45546970
chr6	45491940	45494076
chr6	45491940	45513379
chr6	47503279	47504054
chr6	47503279	47504758
chr6	47503279	47515962
chr6	47526627	47554766
chr6	47533601	47559401
chr6	4854524	4892379
chr6	4867355	4892379
chr6	4881364	4892379
chr6	4888736	4892379
chr6	4890254	4892379
chr6	4891712	4894040
chr6	4891712	4896462
chr6	4935580	4937737
chr6	49457185	49458058
chr6	5216617	5243416
chr6	52267864	52281508
chr6	52267864	52281674
chr6	52422888	52452837
chr6	52422917	52469473
chr6	52422917	52490350
chr6	52437561	52438591
chr6	52438303	52440603
chr6	52516005	52517276
chr6	52578311	52579609
chr6	52665273	52666518
chr6	53009438	53010240
chr6	53145557	53158370
chr6	5325135	5431172
chr6	53253048	53276256
chr6	53269086	53276256
chr6	53269086	53291963
chr6	53269086	53295707
chr6	53291775	53297555
chr6	53498606	53522527
chr6	53833066	53842227
chr6	5404541	5433056
chr6	5404541	5445504
chr6	54308596	54326916

chr6	5431040	5433056
chr6	5431040	5445504
chr6	56843054	56900621
chr6	56900420	56954650
chr6	57014374	57015353
chr6	57014461	57015353
chr6	57050773	57051048
chr6	57050773	57055797
chr6	57052685	57055797
chr6	57052685	57059377
chr6	57052685	57059409
chr6	57124733	57136475
chr6	57181190	57190600
chr6	57181190	57210445
chr6	57182031	57183911
chr6	57182031	57184225
chr6	57182031	57190666
chr6	57190392	57207713
chr6	57190392	57210445
chr6	57371169	57382168
chr6	57379900	57402268
chr6	57398004	57537625
chr6	57481876	57537625
chr6	57525745	57537625
chr6	57526954	57537625
chr6	5771290	5775316
chr6	57919783	57924591
chr6	57919783	57926510
chr6	57919783	57930291
chr6	629834	647666
chr6	70426569	70452571
chr6	70699274	70732511
chr6	70727128	70773425
chr6	70747976	70755065
chr6	7140647	7189322
chr6	71416409	71418754
chr6	7174637	7176773
chr6	7174637	7189322
chr6	7176307	7176773
chr6	72640733	72648904
chr6	72647931	72677214
chr6	73425463	73445747
chr6	73482448	73486761
chr6	75240300	75244006
chr6	75621515	75634811

chr6	75622750	75647801
chr6	75702622	75703072
chr6	78897828	78903110
chr6	79015254	79015629
chr6	79654494	79673799
chr6	80123418	80171390
chr6	80342857	80343812
chr6	80343663	80353897
chr6	80343663	80354354
chr6	82181878	82194595
chr6	82214230	82214829
chr6	82224067	82231833
chr6	83018972	83044506
chr6	83018972	83044655
chr6	83090217	83097115
chr6	83707797	83709761
chr6	84444386	84541724
chr6	84444386	84556154
chr6	85547115	85574378
chr6	85565331	85588950
chr6	85617390	85619417
chr6	85622481	85642869
chr6	87203477	87218731
chr6	87210450	87218731
chr6	87210450	87245644
chr6	87214997	87218731
chr6	87215902	87222894
chr6	87337008	87341328
chr6	87337008	87348906
chr6	87337008	87356503
chr6	87345166	87398377
chr6	87356300	87383760
chr6	87477361	87491739
chr6	87598926	87601881
chr6	87657938	87665833
chr6	87663002	87667173
chr6	87675079	87681763
chr6	88803697	88853764
chr6	88887788	88904955
chr6	88904714	88941176
chr6	88928984	88941176
chr6	89595915	89606142
chr6	89627582	89628341
chr6	89661633	89662086
chr6	89718365	89719067

chr7	100029860	100031100
chr7	1000469	1010305
chr7	100056910	100058298
chr7	100089640	100090487
chr7	100098749	100099022
chr7	100355142	100355804
chr7	100367348	100367617
chr7	100706820	100707333
chr7	1009949	1010300
chr7	101127107	101128664
chr7	101127244	101128368
chr7	101134388	101135594
chr7	101223476	101224141
chr7	101893192	101916225
chr7	101916114	101916507
chr7	101916114	101918760
chr7	101916114	101951784
chr7	102173499	102178657
chr7	102455249	102457981
chr7	102457281	102457981
chr7	102703314	102854860
chr7	102813276	102822203
chr7	102813276	102832974
chr7	102813276	102854860
chr7	102854776	102901180
chr7	102874240	102884312
chr7	103025062	103047020
chr7	105200912	105203785
chr7	105203627	105206529
chr7	105203627	105335953
chr7	105284714	105297533
chr7	105285008	105297533
chr7	105285693	105297533
chr7	105285790	105297533
chr7	105293533	105297533
chr7	105293570	105297533
chr7	105297420	105318945
chr7	105457761	105468463
chr7	105470760	105495253
chr7	105508114	105508520
chr7	105508155	105508544
chr7	106251069	106254504
chr7	107274479	107283732
chr7	107298141	107314169
chr7	107362032	107459788

chr7	107753511	107753890
chr7	107916793	107926761
chr7	10986634	11037091
chr7	10986681	11062085
chr7	111487237	111492514
chr7	111487237	111532585
chr7	116695749	116700284
chr7	116758458	116796042
chr7	116954460	117099844
chr7	118185646	118188405
chr7	12212991	12215027
chr7	122530131	122581290
chr7	123596351	123635772
chr7	123684485	123709223
chr7	127698874	127701394
chr7	127717291	127807573
chr7	127804612	127807573
chr7	127928059	127991056
chr7	127976651	127991056
chr7	127976996	127991056
chr7	127990946	128008144
chr7	128310779	128315020
chr7	128641202	128648982
chr7	128641237	128648982
chr7	128715464	128716997
chr7	128758870	128771788
chr7	128792269	128794768
chr7	128881936	128882254
chr7	129274279	129379749
chr7	129327982	129379749
chr7	129368469	129379749
chr7	129480784	129485863
chr7	130039463	130041473
chr7	130040950	130041473
chr7	130116577	130129576
chr7	130173631	130175638
chr7	130912109	130936155
chr7	130945719	131053465
chr7	130967388	130988888
chr7	130984052	130988888
chr7	130984052	131076587
chr7	131052424	131101187
chr7	131338918	131445903
chr7	131373709	131429145
chr7	131429034	131470944

chr7	132975168	133055157
chr7	133305166	133480138
chr7	133308372	133317390
chr7	135636330	135645018
chr7	139146983	139148377
chr7	139237007	139273410
chr7	139282055	139282986
chr7	139365208	139374471
chr7	139620400	139675586
chr7	139715931	139726108
chr7	139715931	139742038
chr7	139715931	139755259
chr7	13986115	13989139
chr7	140053911	140058034
chr7	140334428	140337349
chr7	140334633	140337349
chr7	140355667	140357212
chr7	140355667	140386921
chr7	140776911	140834703
chr7	140781575	140834703
chr7	140794307	140834703
chr7	140800361	140834703
chr7	141674066	141682537
chr7	143264085	143268943
chr7	1444651	1450400
chr7	1459183	1489682
chr7	148724562	148730262
chr7	148729961	148730283
chr7	148729961	148737743
chr7	148740815	148757150
chr7	148832633	148837000
chr7	148832633	148838340
chr7	148832633	148850525
chr7	148836821	148847305
chr7	148842805	148847305
chr7	148846469	148850525
chr7	149166157	149178855
chr7	149166157	149179676
chr7	149206822	149207329
chr7	149264382	149278527
chr7	149292308	149293350
chr7	149492858	149558839
chr7	149547724	149558839
chr7	149547954	149549192
chr7	149549192	149551749

chr7	149549192	149558839
chr7	151119986	151120745
chr7	151218897	151219063
chr7	151467144	151491014
chr7	151470570	151498180
chr7	151477332	151518891
chr7	151484736	151498180
chr7	151675419	151676412
chr7	151675419	151767055
chr7	151781125	151786541
chr7	152263015	152435787
chr7	152286299	152315338
chr7	152309965	152435787
chr7	152343878	152358675
chr7	152358586	152435787
chr7	152854457	152855722
chr7	154998649	155000155
chr7	155672866	155685052
chr7	155672866	155685648
chr7	155706859	155707128
chr7	155763808	155766655
chr7	155763808	155774647
chr7	1566591	1567850
chr7	156779654	156826744
chr7	156796388	156851214
chr7	156826604	156834575
chr7	156826604	156863254
chr7	156958605	156967092
chr7	157339720	157367483
chr7	157339971	157367483
chr7	157382245	157385642
chr7	157382245	157390394
chr7	157382245	157416255
chr7	157382248	157385611
chr7	158630496	158646563
chr7	158631643	158646563
chr7	158637637	158646563
chr7	158733877	158734471
chr7	158788003	158798036
chr7	158789916	158799072
chr7	16685923	16697061
chr7	1688057	1709252
chr7	17868406	17881033
chr7	17868406	17907340
chr7	17888559	17897446

chr7	17890362	17907340
chr7	18561838	18594029
chr7	18585420	18594029
chr7	18585420	18666476
chr7	18627901	18666476
chr7	18722951	18793452
chr7	18722951	18835997
chr7	1898199	1940191
chr7	2002064	2004848
chr7	2013083	2014642
chr7	2068666	2069338
chr7	2069193	2200557
chr7	208897	209384
chr7	208897	259976
chr7	21020867	21023309
chr7	21020867	21033558
chr7	21440957	21482123
chr7	21443590	21482123
chr7	2200261	2218043
chr7	2200261	2219456
chr7	2209946	2219456
chr7	22160517	22189663
chr7	22183143	22194033
chr7	22308338	22359392
chr7	2239417	2239676
chr7	22417896	22419836
chr7	2244562	2245277
chr7	2244562	2250934
chr7	22492564	22495480
chr7	22571774	22575762
chr7	22940971	22991139
chr7	22976209	22998161
chr7	22983943	22998161
chr7	23116639	23144025
chr7	23351304	23470081
chr7	23360585	23361741
chr7	23415247	23418824
chr7	23604085	23611553
chr7	23698191	23702221
chr7	24593301	24650712
chr7	24623662	24641868
chr7	24623662	24650712
chr7	24623662	24668660
chr7	24661840	24668660
chr7	24679130	24693848

chr7	24687437	24693848
chr7	24979885	24982404
chr7	2576585	2578526
chr7	26177942	26186094
chr7	26190992	26197732
chr7	27163574	27164967
chr7	2729255	2731750
chr7	2731130	2733501
chr7	27603137	27649633
chr7	27629370	27632441
chr7	29923955	29926632
chr7	29923955	29955360
chr7	29923955	29969068
chr7	30074070	30075405
chr7	30074070	30078778
chr7	30146176	30159095
chr7	30323641	30324302
chr7	30323641	30366295
chr7	30355727	30366295
chr7	30366034	30391035
chr7	30550635	30562128
chr7	30595823	30603572
chr7	30821231	30836764
chr7	32489248	32490766
chr7	32716717	32726470
chr7	32933399	32933896
chr7	33014699	33035988
chr7	33146241	33183405
chr7	33146268	33273956
chr7	33604864	33606553
chr7	34966893	34989447
chr7	36212535	36239036
chr7	37948839	37950392
chr7	37948839	37966214
chr7	38789800	38806366
chr7	39781769	39789544
chr7	39987598	40003106
chr7	39987598	40018708
chr7	40047820	40094396
chr7	40180946	40217563
chr7	42145437	42148468
chr7	42148225	42172686
chr7	42917513	42917835
chr7	43595900	43625035
chr7	43631474	43647634

chr7	43633278	43647634
chr7	43639448	43644204
chr7	44117265	44117618
chr7	44392296	44413283
chr7	44425899	44427703
chr7	44481291	44485284
chr7	44569983	44570877
chr7	44570149	44570757
chr7	44618823	44624565
chr7	44645326	44654771
chr7	4740837	4742849
chr7	4783798	4784175
chr7	4983670	4996798
chr7	4989062	4996798
chr7	4989062	5005228
chr7	5020526	5057779
chr7	5047969	5057779
chr7	50669721	50705323
chr7	50669721	50710990
chr7	50703820	50705323
chr7	50703820	50710990
chr7	50726012	50756056
chr7	50755886	50780736
chr7	510808	519068
chr7	518938	551470
chr7	5199575	5203481
chr7	5214232	5214704
chr7	5359089	5359569
chr7	5394041	5394595
chr7	5641153	5662565
chr7	5902744	5905058
chr7	596145	601555
chr7	5999107	6002811
chr7	6022786	6029032
chr7	6037423	6039014
chr7	6037423	6039035
chr7	6111124	6116939
chr7	6115322	6115726
chr7	6115651	6135951
chr7	6432788	6436533
chr7	64535818	64544432
chr7	64541729	64544432
chr7	6474183	6482589
chr7	64814917	64817335
chr7	64851798	64858602

chr7	65066230	65067050
chr7	65726770	65734350
chr7	65727054	65734350
chr7	65752163	65756574
chr7	6577720	6578534
chr7	65960951	65974704
chr7	65979385	65980409
chr7	66130743	66152474
chr7	66152207	66153099
chr7	66240324	66248646
chr7	66248528	66286709
chr7	66271670	66286709
chr7	66278952	66286709
chr7	66284577	66286709
chr7	66286510	66307395
chr7	66356824	66360109
chr7	66382236	66390250
chr7	66395878	66402545
chr7	66399691	66401394
chr7	66545600	66576932
chr7	66551519	66576932
chr7	66553988	66576929
chr7	66554584	66576929
chr7	66554584	66576932
chr7	66573384	66578727
chr7	66576754	66578727
chr7	66633274	66633806
chr7	66638252	66643611
chr7	66681423	66682258
chr7	66710830	66712224
chr7	66712166	66712613
chr7	66712166	66712957
chr7	66712166	66713468
chr7	66761125	66775393
chr7	66761125	66783841
chr7	66770322	66775393
chr7	66770322	66783841
chr7	66783674	66810847
chr7	66795510	66805400
chr7	66796617	66805396
chr7	66797373	66827578
chr7	66808885	66810847
chr7	6692601	6698366
chr7	66927641	66948694
chr7	66933695	66945261

chr7	66948543	66955851
chr7	66950951	66951268
chr7	66950951	66955851
chr7	66950951	66955953
chr7	66991136	66994282
chr7	66993216	66994282
chr7	67024899	67151193
chr7	67049948	67059915
chr7	67083429	67084914
chr7	67096465	67183236
chr7	67183125	67203899
chr7	67308934	67320336
chr7	677228	711523
chr7	72694686	72715869
chr7	72713620	72715869
chr7	72830750	72831798
chr7	72830755	72831798
chr7	72881449	72891110
chr7	72890626	72901128
chr7	73489193	73517330
chr7	73498496	73507547
chr7	74194740	74203294
chr7	74375886	74377635
chr7	74559044	74559598
chr7	75312892	75511114
chr7	754588	757709
chr7	75474703	75486247
chr7	75507366	75511114
chr7	7588497	7607136
chr7	75996444	76013887
chr7	761752	789523
chr7	770470	785897
chr7	77292951	77294938
chr7	77421766	77425399
chr7	77765494	77778814
chr7	77778337	77778814
chr7	77778337	77780774
chr7	7786787	7801743
chr7	7877280	7884346
chr7	7877280	7889229
chr7	7877280	7892554
chr7	79455317	79458999
chr7	8003907	8024814
chr7	8003907	8048945
chr7	80788845	80810701

chr7	80789305	80883765
chr7	80818298	80883765
chr7	80915616	80916819
chr7	815643	816673
chr7	82170549	82184692
chr7	8228600	8252021
chr7	82335134	82364813
chr7	82335134	82366668
chr7	82335134	82403855
chr7	832504	833194
chr7	832504	833229
chr7	848417	866067
chr7	85072993	85121931
chr7	85121740	85153739
chr7	85121740	85224441
chr7	87163238	87185980
chr7	87836192	87860640
chr7	87971293	87978412
chr7	886055	895480
chr7	90227286	90232484
chr7	90354457	90385168
chr7	90354457	90385520
chr7	90377506	90385238
chr7	90608529	90726812
chr7	90716339	90726812
chr7	90908317	90955817
chr7	90917600	90921955
chr7	92240748	92245171
chr7	92294888	92333720
chr7	92671425	92731744
chr7	94618757	94644667
chr7	96270584	96277338
chr7	96270584	96296951
chr7	96277195	96309691
chr7	99013596	99014955
chr7	99032771	99033121
chr7	99491076	99494631
chr7	99493039	99494631
chr7	99604385	99610601
chr7	99903217	99917885
chr8	100259106	100259997
chr8	100259106	100264785
chr8	100259106	100275161
chr8	100704296	100721889
chr8	100709132	100721886

chr8	100712478	100712651
chr8	100717772	100721886
chr8	101198263	101201743
chr8	101199194	101201743
chr8	102302270	102312245
chr8	102313943	102314743
chr8	102360070	102391161
chr8	102834188	102836541
chr8	102879894	102887178
chr8	103285837	103407784
chr8	103400414	103403324
chr8	103400414	103404861
chr8	103400414	103407784
chr8	103430611	103437307
chr8	103430611	103462118
chr8	103514017	103697296
chr8	103644043	103697296
chr8	103652204	103697296
chr8	104013441	104018277
chr8	104013441	104148848
chr8	104444247	104485069
chr8	104501193	104509074
chr8	109164767	109245481
chr8	109238634	109281172
chr8	109243150	109245481
chr8	11295193	11300241
chr8	11300086	11319838
chr8	116644485	116658980
chr8	116719272	116726172
chr8	116724908	116726172
chr8	116726015	116726522
chr8	116726015	116734354
chr8	116726015	116737287
chr8	116726015	116755797
chr8	11808793	11838573
chr8	11808793	11845235
chr8	11826078	11838573
chr8	118375781	118379700
chr8	118379559	118403227
chr8	118577679	118580893
chr8	118580714	118585992
chr8	118724518	118764384
chr8	119730135	119732186
chr8	119731882	119778129
chr8	119738807	119742656

chr8	119791323	119831721
chr8	119801793	119831721
chr8	119803917	119819506
chr8	119806282	119831721
chr8	119817983	119819506
chr8	123015392	123030716
chr8	123015519	123030716
chr8	123019194	123036954
chr8	123380528	123381493
chr8	123427923	123441497
chr8	123436452	123441497
chr8	123436452	123448224
chr8	124208115	124226214
chr8	125102013	125102487
chr8	125102013	125182256
chr8	125102050	125127067
chr8	125102050	125195272
chr8	125130212	125151277
chr8	125130212	125182256
chr8	125182102	125195272
chr8	12723099	12743282
chr8	127738247	127740964
chr8	127795893	127890998
chr8	127816827	127890998
chr8	127851933	127890998
chr8	127855154	127890998
chr8	127855162	127890998
chr8	127859890	127890998
chr8	127867438	127890998
chr8	127882061	127890998
chr8	127890588	127890998
chr8	127890588	127939676
chr8	127939507	128010444
chr8	127969415	127989291
chr8	127982359	127989291
chr8	127983903	128010444
chr8	127989161	127992939
chr8	127989161	128010444
chr8	127989161	128032272
chr8	128008528	128070272
chr8	128009589	128070272
chr8	128070159	128073491
chr8	129871374	129973793
chr8	130089766	130092143
chr8	130188108	130290138

chr8	130214555	130290138
chr8	130290000	130358143
chr8	130290000	130361771
chr8	130290000	130401970
chr8	130340408	130361771
chr8	130358016	130362366
chr8	130358016	130402893
chr8	130358016	130406193
chr8	130358016	130427822
chr8	130401884	130406193
chr8	130401884	130427822
chr8	132610165	132661627
chr8	132777795	132817545
chr8	132795965	132817545
chr8	132807684	132825371
chr8	134599729	134610655
chr8	140154963	140221583
chr8	140190874	140252929
chr8	140275657	140409617
chr8	140300468	140347946
chr8	140347756	140360193
chr8	140347756	140405698
chr8	140360049	140365686
chr8	140368017	140405698
chr8	140397619	140409617
chr8	140532056	140585311
chr8	140557258	140558484
chr8	140572811	140597670
chr8	140572811	140605914
chr8	140580904	140585311
chr8	140585090	140585311
chr8	140585118	140605914
chr8	140585118	140616346
chr8	140686631	140687129
chr8	140788163	140890769
chr8	140800458	140921132
chr8	140818276	140921132
chr8	140818276	140993094
chr8	140826978	140864399
chr8	140826978	140890769
chr8	140843946	140864399
chr8	140843946	140890769
chr8	140846259	140893303
chr8	140846259	140921132
chr8	140861223	140890769

chr8	140864311	140912467
chr8	140874462	140890769
chr8	140879503	140890769
chr8	141136508	141138137
chr8	141136508	141168525
chr8	141150833	141155989
chr8	141151618	141155989
chr8	141237748	141254629
chr8	143580059	143586852
chr8	143597004	143597347
chr8	143809047	143809550
chr8	144357139	144357438
chr8	144383080	144394573
chr8	144515484	144515763
chr8	15613379	15673836
chr8	15613470	15673836
chr8	1564004	1565894
chr8	1565682	1569751
chr8	1565682	1583112
chr8	1565682	1590363
chr8	1626739	1655057
chr8	17064083	17120401
chr8	17077782	17120401
chr8	17098457	17120401
chr8	17104390	17120401
chr8	18632612	18674840
chr8	19842816	19844238
chr8	22139082	22139415
chr8	22498000	22505769
chr8	23042040	23043243
chr8	23068750	23069011
chr8	25242194	25243757
chr8	28737518	28750763
chr8	30101897	30106843
chr8	30578365	30614715
chr8	30786210	30799755
chr8	33372643	33389798
chr8	38105984	38115000
chr8	38266140	38269517
chr8	38314646	38348143
chr8	38456316	38457534
chr8	38457355	38457955
chr8	38819521	38827288
chr8	38917541	38918070
chr8	39076146	39091346

chr8	39101862	39103952
chr8	41974685	41987563
chr8	42048377	42049707
chr8	42325969	42329226
chr8	42925556	42946238
chr8	42949319	42966613
chr8	42986654	42988641
chr8	43059091	43085396
chr8	43076363	43077364
chr8	47273336	47440542
chr8	47279861	47300097
chr8	47279861	47326985
chr8	47279861	47330895
chr8	47284027	47300097
chr8	47388340	47440542
chr8	47394977	47440542
chr8	47407860	47461951
chr8	47440322	47491994
chr8	47781811	47782254
chr8	47834428	47836337
chr8	47969797	47976866
chr8	48034865	48043181
chr8	48043032	48043626
chr8	48043032	48062187
chr8	51845660	51870390
chr8	51857459	51861246
chr8	51857492	51861246
chr8	51860844	51870963
chr8	52685398	52686156
chr8	53993667	53995866
chr8	54136510	54147544
chr8	54147381	54148266
chr8	55750963	55770258
chr8	55785891	55786214
chr8	55798247	55805036
chr8	55941854	55947841
chr8	56073694	56074493
chr8	56963834	56980197
chr8	56963962	56966352
chr8	56963962	56980197
chr8	61492382	61584029
chr8	61546845	61567318
chr8	61546874	61548208
chr8	61661805	61684188
chr8	61676139	61684188

chr8	61679276	61684188
chr8	6480713	6503387
chr8	65609920	65613433
chr8	65719224	65727301
chr8	67103036	67116156
chr8	67128225	67132080
chr8	67299208	67320512
chr8	67302435	67308232
chr8	677500	692612
chr8	690897	692612
chr8	690897	716007
chr8	70256437	70296800
chr8	70637814	70641050
chr8	70637814	70644243
chr8	70669849	70707153
chr8	70680780	70713576
chr8	71954137	71963048
chr8	71962922	71973659
chr8	72052538	72062365
chr8	72052538	72076627
chr8	73527690	73561539
chr8	73527690	73688813
chr8	73527690	73709162
chr8	73668980	73739868
chr8	73673106	73697442
chr8	73673106	73744889
chr8	73688172	73688813
chr8	73805650	73850594
chr8	73946752	73956054
chr8	74658411	74752533
chr8	78558342	78572939
chr8	78697406	78702116
chr8	79917977	79919148
chr8	79993976	80003180
chr8	80002998	80009611
chr8	80499493	80577167
chr8	81829429	81840356
chr8	85281229	85281639
chr8	86494828	86507112
chr8	88167668	88327186
chr8	89724628	89728755
chr8	89765340	89790412
chr8	90015594	90044995
chr8	90978578	90985841
chr8	91018389	91024366

chr8	91078355	91102844
chr8	91097038	91102844
chr8	91097038	91219005
chr8	91102218	91102844
chr8	91102218	91133305
chr8	91200612	91219005
chr8	91218049	91219005
chr8	92916928	92929574
chr8	92916928	92930707
chr8	93706044	93718788
chr8	93922015	94020460
chr8	94411120	94418783
chr8	94418655	94467555
chr8	94454146	94458436
chr8	94506499	94511423
chr8	94766611	94767052
chr8	94827318	94859632
chr8	95035453	95036494
chr8	96256961	96260247
chr8	9649318	9710220
chr8	96680442	96785330
chr8	96834972	96850238
chr8	96879797	96884493
chr8	96879797	96902096
chr8	97691255	97699852
chr8	97802620	97819238
chr8	98117317	98123479
chr8	98212404	98217262
chr8	98526741	98529652
chr8	98693136	98707311
chr8	98706466	98722818
chr8	98706466	98722969
chr8	98706466	98728621
chr8	98746709	98749390
chr8	98746709	98774819
chr8	99013759	99064287
chr8	99038422	99041418
chr8	99134234	99156743
chr8	99236270	99275254
chr8	99274197	99289762
chr8	99274197	99328914
chr8	99384207	99400135
chr8	99419915	99577633
chr8	99520898	99585517
chr8	99575657	99585517

chr8	99878139	99887618
chr9	100006505	100022204
chr9	100072546	100126549
chr9	100101695	100126549
chr9	100102119	100126549
chr9	100126382	100131954
chr9	100450556	100471231
chr9	100450556	100516771
chr9	100456742	100509134
chr9	100457757	100572676
chr9	105273496	105309866
chr9	105460720	105472005
chr9	105573654	105603816
chr9	105613301	105620061
chr9	105705596	105738880
chr9	105714365	105748190
chr9	105721536	105774026
chr9	106938915	106939915
chr9	109050282	109078943
chr9	109060353	109091152
chr9	109073247	109093591
chr9	109087172	109091152
chr9	109780487	109828032
chr9	109931926	109943381
chr9	110015953	110016354
chr9	110156318	110198574
chr9	110874905	110875722
chr9	110897494	110973558
chr9	110941420	111005539
chr9	110941420	111009201
chr9	110941420	111011690
chr9	110941420	111036201
chr9	110972072	111005539
chr9	110972072	111011690
chr9	110972072	111036201
chr9	111564156	111586165
chr9	111574733	111589382
chr9	111914604	111915836
chr9	111914604	111917806
chr9	111932359	111932825
chr9	112098469	112130844
chr9	112142319	112160980
chr9	112275843	112288682
chr9	113408780	113410140
chr9	113594429	113597167

chr9	120821354	120823076
chr9	121699324	121699661
chr9	121770724	121772557
chr9	121822106	121822879
chr9	122280442	122286166
chr9	122280442	122322745
chr9	122285168	122286166
chr9	122291748	122294362
chr9	122313226	122322745
chr9	122313226	122322812
chr9	122957010	122979040
chr9	122957148	122990213
chr9	122996684	123020459
chr9	123169901	123236969
chr9	123401704	123440491
chr9	123440359	123452908
chr9	123457347	123462024
chr9	123548003	123557695
chr9	123557569	123562941
chr9	123557569	123565648
chr9	123736293	123879021
chr9	123739789	123757822
chr9	123757702	123917176
chr9	123757702	123923415
chr9	124353541	124384656
chr9	124554327	124684986
chr9	124733307	124738427
chr9	124885359	124888396
chr9	124922626	124923223
chr9	124994666	124999990
chr9	125207563	125208812
chr9	125220326	125220700
chr9	125297428	125299106
chr9	125337017	125337568
chr9	125478079	125484583
chr9	125478079	125506417
chr9	125543058	125550697
chr9	125557889	125559809
chr9	125583405	125585727
chr9	125656179	125672643
chr9	125657650	125672540
chr9	126338926	126386658
chr9	126370164	126395697
chr9	126380500	126421948
chr9	126380526	126395697

chr9	126976570	127069356
chr9	127243078	127265021
chr9	127243078	127365366
chr9	127428831	127445249
chr9	127449382	127449609
chr9	127449389	127449609
chr9	128309678	128311601
chr9	128474686	128482887
chr9	128507215	128509097
chr9	128598399	128599104
chr9	128612246	128613346
chr9	128705543	128705701
chr9	129908889	129909499
chr9	129978464	129999059
chr9	130593642	130595588
chr9	130851393	130875052
chr9	130854063	130887414
chr9	131077898	131079298
chr9	131174086	131178410
chr9	131233453	131233915
chr9	131430093	131432794
chr9	131430093	131439061
chr9	131430093	131447804
chr9	131459163	131459563
chr9	131477949	131478446
chr9	131630235	131647750
chr9	131939380	131947831
chr9	132014336	132044012
chr9	132014336	132060373
chr9	13216773	13258733
chr9	13247634	13259059
chr9	13247634	13276771
chr9	132677976	132689065
chr9	132896788	132897183
chr9	133333717	133367445
chr9	133351835	133356320
chr9	133407905	133408767
chr9	133925842	133939219
chr9	135850136	135857673
chr9	135866455	135888273
chr9	135881632	135888273
chr9	135933056	135933515
chr9	136226773	136232441
chr9	136416950	136423677
chr9	136432639	136432955

chr9	136544023	136677556
chr9	136726582	136727045
chr9	137175499	137175707
chr9	137175501	137175707
chr9	137188778	137189046
chr9	137272222	137272481
chr9	137727166	137728529
chr9	137727166	137744090
chr9	137727166	137762820
chr9	137728348	137731583
chr9	137743391	137758011
chr9	137743391	137762820
chr9	137743394	137754291
chr9	137743394	137762820
chr9	137815946	137835088
chr9	137834348	137835088
chr9	14592493	14640012
chr9	14602717	14640012
chr9	14602717	14680162
chr9	14609849	14674385
chr9	15465484	15469993
chr9	15563977	15597787
chr9	15597629	15623413
chr9	15622737	15623413
chr9	17330234	17342444
chr9	19267012	19290876
chr9	19305415	19316839
chr9	19350701	19352693
chr9	20410560	20414425
chr9	20711753	20885230
chr9	20715321	20740303
chr9	20819795	20825852
chr9	20923659	20933132
chr9	2181570	2192802
chr9	21859302	21862135
chr9	21859302	21862142
chr9	21859302	21864433
chr9	21859302	21866842
chr9	21859302	21872928
chr9	21859302	21890838
chr9	2191265	2192802
chr9	21939159	22097364
chr9	22723308	22757076
chr9	22820737	22821707
chr9	23602654	23659677

chr9	23762005	23765099
chr9	2493074	2539552
chr9	2536358	2552338
chr9	26798510	26884885
chr9	26842274	26887513
chr9	2720343	2820098
chr9	27455132	27459872
chr9	2782755	2812362
chr9	2804238	2812362
chr9	2804256	2812362
chr9	2804321	2812362
chr9	32924896	32974561
chr9	32987543	32987846
chr9	33293679	33295427
chr9	33295284	33313793
chr9	3330258	3363488
chr9	3330258	3416712
chr9	3330258	3420870
chr9	33351559	33370014
chr9	33366628	33370014
chr9	33948373	33948568
chr9	33956857	33963791
chr9	34249778	34255986
chr9	34338712	34343583
chr9	34338712	34346837
chr9	35050063	35059801
chr9	35661712	35664850
chr9	35684573	35685063
chr9	35707920	35708340
chr9	36217256	36220020
chr9	36223034	36223372
chr9	36369718	36372593
chr9	36580072	36583712
chr9	36580072	36589652
chr9	37121498	37305714
chr9	37126311	37127263
chr9	37126311	37147445
chr9	37126311	37165935
chr9	37126311	37190651
chr9	37126311	37206494
chr9	37126311	37219098
chr9	37126311	37247130
chr9	37126311	37249594
chr9	37126311	37260565
chr9	37436660	37436960

chr9	37780657	37784063
chr9	37842088	37861235
chr9	37842088	37861385
chr9	41008880	41033022
chr9	41025763	41027092
chr9	41025763	41027114
chr9	41923892	41929444
chr9	4286037	4286435
chr9	4541032	4622510
chr9	4676461	4676749
chr9	4799158	4827033
chr9	4823547	4828688
chr9	4823547	4860901
chr9	4849446	4860901
chr9	4860124	4860901
chr9	4860124	4928266
chr9	4860124	4928312
chr9	4966106	5044520
chr9	537024	540667
chr9	539676	540667
chr9	5656582	5666072
chr9	5787060	5787593
chr9	5933441	5944960
chr9	6420911	6465846
chr9	6433913	6445540
chr9	64465758	64466310
chr9	6460572	6465846
chr9	6719070	6720997
chr9	6830673	6893232
chr9	6843548	6893232
chr9	69064937	69072830
chr9	69718435	69750950
chr9	7103684	7105500
chr9	71749756	71750385
chr9	71750042	71805209
chr9	71862550	71875083
chr9	71874613	71886414
chr9	71918424	71947112
chr9	71947005	71952132
chr9	74996446	75027151
chr9	75077158	75080941
chr9	75932378	75934724
chr9	77794462	77824880
chr9	77919265	77922345
chr9	77920647	77922345

chr9	77920661	77922345
chr9	77922160	77931856
chr9	77922160	77952431
chr9	81590804	81593248
chr9	83585552	83686155
chr9	83598742	83739744
chr9	83659779	83669327
chr9	83659779	83677961
chr9	83659779	83686155
chr9	83661776	83669327
chr9	83677726	83686076
chr9	83739700	83768970
chr9	85822879	85830437
chr9	88415606	88426591
chr9	88416643	88426591
chr9	88416643	88448989
chr9	88418849	88448989
chr9	88421331	88462749
chr9	88426381	88433828
chr9	88426381	88434829
chr9	88426381	88475457
chr9	88426381	88475513
chr9	89314719	89315297
chr9	91264916	91356155
chr9	91325317	91327651
chr9	91775740	91913127
chr9	92262135	92269983
chr9	92337540	92347506
chr9	92637190	92638280
chr9	93470328	93476338
chr9	93470328	93498886
chr9	93471262	93527242
chr9	93628415	93636525
chr9	93629969	93637695
chr9	94057117	94088263
chr9	94274205	94293069
chr9	94773001	94802519
chr9	94790348	94956015
chr9	95101614	95111637
chr9	95135345	95186645
chr9	95149922	95186645
chr9	95240648	95265754
chr9	95880868	95882340
chr9	95978060	95979866
chr9	95978060	96012357

chr9	96004245	96004701
chr9	96004519	96012357
chr9	96465336	96484659
chr9	96509672	96611559
chr9	96515648	96565483
chr9	96522505	96611559
chr9	96774787	96845016
chr9	96949554	96962963
chr9	97675488	97693759
chr9	98015363	98018489
chr9	98069458	98078347
chr9	98075569	98078347
chr9	99128854	99149301
chr9	99913989	99928843
Un_GL00019	48954	50014
chrX	100914620	100915083
chrX	100914620	100923061
chrX	101010522	101019403
chrX	101010609	101019403
chrX	101109501	101148161
chrX	102814979	102817082
chrX	102816991	102839972
chrX	102825992	102865152
chrX	102826968	102834976
chrX	102826968	102905737
chrX	102829887	102865152
chrX	102834627	102865152
chrX	102839800	102897764
chrX	102839800	102905737
chrX	102865041	102905737
chrX	102884142	102905737
chrX	103517373	103531322
chrX	103517373	103585660
chrX	104175622	104189804
chrX	106980890	106985250
chrX	107150855	107153503
chrX	107154095	107206431
chrX	107639294	107650157
chrX	108440005	108539805
chrX	109659353	109683375
chrX	110020351	110067396
chrX	110020351	110109146
chrX	110046356	110109146
chrX	110067346	110173453
chrX	110109079	110173453

chrX	110171419	110173453
chrX	110216517	110220749
chrX	110264488	110279274
chrX	111742200	111744904
chrX	111757587	111764887
chrX	112822254	112825220
chrX	115629197	115649626
chrX	115946385	115951383
chrX	118396969	118425042
chrX	118737326	118749778
chrX	118989407	119017689
chrX	119406459	119426354
chrX	119406459	119433744
chrX	119410189	119417640
chrX	119410189	119419344
chrX	119410189	119426354
chrX	119410189	119443897
chrX	119616416	119640788
chrX	120229403	120240355
chrX	123590584	123611516
chrX	123610977	123611439
chrX	1282441	1290509
chrX	129576353	129588263
chrX	129806313	129829141
chrX	129994374	130005317
chrX	130783546	130809261
chrX	131692437	131794466
chrX	131695843	131794466
chrX	131711650	131785258
chrX	131731128	131794466
chrX	131736126	131794466
chrX	131743095	131749458
chrX	131743095	131756709
chrX	131743095	131784039
chrX	131743095	131794466
chrX	131749305	131770551
chrX	131749305	131772563
chrX	131749305	131785258
chrX	131749305	131794464
chrX	131755597	131777744
chrX	131755597	131785258
chrX	131768548	131785258
chrX	131777622	131784039
chrX	131777622	131805037
chrX	131783886	131785258

chrX	131783886	131794115
chrX	131793993	131805037
chrX	135276074	135296039
chrX	135287252	135296039
chrX	135347018	135361601
chrX	135906023	135920392
chrX	136870193	136875585
chrX	1386681	1389727
chrX	1396371	1412888
chrX	139814877	139886581
chrX	14007727	14026221
chrX	147924918	147945616
chrX	14843659	14859334
chrX	148543951	148662768
chrX	150744360	150751240
chrX	152320808	152364596
chrX	152345580	152487023
chrX	152364430	152458262
chrX	152862595	152869596
chrX	153454540	153454876
chrX	154012528	154012894
chrX	154030297	154032557
chrX	154349648	154352423
chrX	154359245	154359535
chrX	154490379	154490593
chrX	154506648	154507329
chrX	155046508	155047391
chrX	155116056	155132869
chrX	155334370	155335249
chrX	155351859	155419562
chrX	155506897	155537118
chrX	155545095	155548224
chrX	155889495	155900587
chrX	15688660	15750165
chrX	19456912	19466698
chrX	19456912	19466743
chrX	19456912	19466759
chrX	19588902	19592066
chrX	19803009	19836282
chrX	19836124	19857714
chrX	19929784	19936626
chrX	19950752	19959157
chrX	21513541	21516631
chrX	21978874	21992712
chrX	2211488	2221229

chrX	2240553	2243230
chrX	23721880	23736246
chrX	2392353	2425304
chrX	2424505	2425304
chrX	24493744	24494883
chrX	24821451	24855481
chrX	24840322	24888122
chrX	338603	361519
chrX	338603	362332
chrX	361404	362332
chrX	37354812	37426000
chrX	3817544	3818500
chrX	3817544	3818703
chrX	40052112	40071159
chrX	40697023	40715220
chrX	40712913	40715220
chrX	40713777	40715220
chrX	41165871	41168132
chrX	41184535	41189475
chrX	44524001	44527365
chrX	44524001	44538542
chrX	44956424	45020730
chrX	45070119	45083608
chrX	46620976	46653705
chrX	46620976	46653713
chrX	46651109	46653705
chrX	46679677	46685066
chrX	46984883	47000391
chrX	47222260	47228644
chrX	47842649	47895940
chrX	48046202	48067061
chrX	48575560	48576083
chrX	49113550	49113938
chrX	50042315	50048103
chrX	50204562	50288887
chrX	53561755	53562271
chrX	53575810	53576899
chrX	54558435	54560647
chrX	56977072	56994949
chrX	57262123	57381029
chrX	63399780	63432491
chrX	63509943	63517082
chrX	63637994	63678572
chrX	63638049	63666017
chrX	63638108	63697304

chrX	63722761	63724711
chrX	63724531	63743446
chrX	64193138	64193384
chrX	65366637	65499989
chrX	65445905	65499989
chrX	68046993	68053810
chrX	68047127	68053810
chrX	68053644	68073322
chrX	68081700	68097029
chrX	68081700	68206673
chrX	68299000	68307448
chrX	68838616	68840570
chrX	69528992	69530532
chrX	70290753	70341931
chrX	71061027	71062949
chrX	71121720	71122346
chrX	71141882	71142946
chrX	72272639	72273972
chrX	72272639	72275724
chrX	72462003	72474729
chrX	72462003	72573724
chrX	73563226	73584572
chrX	73577406	73591135
chrX	74060077	74062148
chrX	74274341	74286170
chrX	74592342	74596000
chrX	74611034	74612581
chrX	77557450	77595785
chrX	77574249	77595785
chrX	77829030	77830895
chrX	77870807	77879613
chrX	78129678	78131773
chrX	81276983	81298357
chrX	84321841	84361612
chrX	84321930	84344457
chrX	85067126	85074400
chrX	91414903	91418871
chrX	9640272	9725670
chrX	96942057	97141794
chr10	110228188	110280085
chr10	115215696	115334419
chr10	12160929	12167893
chr10	121836717	121924329
chr10	121841081	121924329
chr10	121870005	121924329

chr10	131934457	131935006
chr10	16947234	16950111
chr10	27046352	27061242
chr10	27048800	27061242
chr10	27060344	27061242
chr10	27060344	27079161
chr10	27066486	27067286
chr10	27066486	27086609
chr10	27907662	27971313
chr10	27935009	27971313
chr10	28056479	28059743
chr10	28069771	28089841
chr10	31808648	31814361
chr10	32462682	32474023
chr10	32685969	32729457
chr10	32805014	32824604
chr10	45618139	45639963
chr10	45624582	45627105
chr10	68121437	68136731
chr10	68135458	68161752
chr10	68651845	68667256
chr10	73310157	73341581
chr10	73335429	73341581
chr10	91222688	91251440
chr10	94341672	94349378
chr10	94687769	94724533
chr10	99955213	99972134
chr11	100959882	100965776
chr11	117239111	117239800
chr11	123577366	123578430
chr11	125978303	126023537
chr11	14789105	14844026
chr11	15177110	15190814
chr11	26678711	26686544
chr11	26678711	26703947
chr11	46727995	46728837
chr11	47663025	47699553
chr11	47699445	47704728
chr11	58549785	58554940
chr11	61264486	61269018
chr11	61280623	61282905
chr11	62702467	62717189
chr11	64626462	64650638
chr11	64751324	64752071
chr11	6538382	6540083

chr11	72605137	72631139
chr11	76360955	76365972
chr11	86386050	86392797
chr11	86386050	86400801
chr11	9047942	9053215
chr11	9065890	9069495
chr12	101729108	101737948
chr12	110503805	110522411
chr12	116964696	116988869
chr12	116964696	117010450
chr12	116985205	116988869
chr12	116985205	117010450
chr12	121244572	121248734
chr12	121923621	121931886
chr12	124340005	124342074
chr12	132869618	132877654
chr12	19348398	19369798
chr12	22506710	22519239
chr12	405510	418636
chr12	43425513	43430471
chr12	57046551	57048343
chr12	57799794	57801212
chr12	6110373	6110968
chr12	66306344	66325126
chr12	66331153	66338212
chr12	6643538	6645337
chr12	68727350	68735344
chr12	68733451	68735344
chr12	7110297	7119553
chr12	71907021	71918548
chr12	75282250	75321576
chr12	79285786	79299551
chr12	79285786	79353619
chr12	85152279	85154094
chr12	878214	882073
chr12	878214	897681
chr12	89621548	89635251
chr12	94378790	94435327
chr12	95665809	95682822
chr13	19835476	19837821
chr13	32194142	32202527
chr13	32326100	32357929
chr13	35040932	35110978
chr13	35048562	35098405
chr13	36312144	36336358

chr13	37637483	37674367
chr13	42926335	42964139
chr13	42953947	42970670
chr13	46356431	46372597
chr13	46356431	46387879
chr13	46368041	46378005
chr13	46368041	46387879
chr13	48511769	48524757
chr13	49480218	49488630
chr13	51771820	51791257
chr13	52143915	52154173
chr13	52627437	52629359
chr13	52629218	52632494
chr13	72731280	72743602
chr13	91693186	91728665
chr13	95863628	95867423
chr14	102194937	102209862
chr14	35008626	35023080
chr14	37212229	37247907
chr14	37212229	37270525
chr14	37622049	37701526
chr14	49709158	49723952
chr14	49709158	49725769
chr14	49732703	49743805
chr14	51333392	51350372
chr14	57274243	57280422
chr14	57274243	57283230
chr14	59324127	59326991
chr14	60455000	60471739
chr14	61443110	61453353
chr14	64268794	64283075
chr14	75622078	75641898
chr14	75633628	75641898
chr14	75965587	75971718
chr14	80831142	80900029
chr14	88391380	88416844
chr14	88705481	88754671
chr14	88839451	88857277
chr14	88839451	88861332
chr14	90890486	90923196
chr14	91988283	92000108
chr14	92007639	92011795
chr14	96471477	96483219
chr15	101701085	101723164
chr15	20669467	20671268

chr15	22667897	22674586
chr15	22667897	22678383
chr15	32857014	32902040
chr15	32908489	33008075
chr15	34256228	34258944
chr15	34256228	34261020
chr15	36640604	36657905
chr15	44672014	44672456
chr15	48851187	48867869
chr15	49568244	49590007
chr15	49588015	49611268
chr15	52223543	52229313
chr15	52584744	52601165
chr15	55327530	55341802
chr15	56373440	56412436
chr15	64915796	64922019
chr15	67421334	67466711
chr15	67825935	67833257
chr15	70895848	70936822
chr15	70919049	70984257
chr15	70936760	70984257
chr15	70980100	70984257
chr15	89871474	89889140
chr15	94356136	94402019
chr15	94440175	94458246
chr15	99799631	99801179
chr16	16255719	16270828
chr16	19616113	19647882
chr16	21019623	21042203
chr16	29320927	29328624
chr16	30993129	30997059
chr16	4571560	4580125
chr16	4785806	4786105
chr16	50716586	50729901
chr16	57041484	57058148
chr16	57041484	57059532
chr16	61713840	61727215
chr16	61789345	61825179
chr16	67820838	67821225
chr16	70646547	70657121
chr16	70656601	70657121
chr16	71894720	71921453
chr16	71894720	71923380
chr16	81017325	81026638
chr16	83670789	83678461

chr16	88586599	88599948
chr17	19780471	19781104
chr17	29052919	29055888
chr17	32653842	32654621
chr17	3446471	3449149
chr17	45987039	45996664
chr17	49141246	49166254
chr17	62573214	62574410
chr17	65902151	66063081
chr17	69100796	69125035
chr17	77307140	77402703
chr17	9608118	9612479
chr18	14828277	14837676
chr18	36501931	36709391
chr18	42212363	42226107
chr18	47052879	47059654
chr18	54907785	54928882
chr19	10782959	10805967
chr19	10795371	10798572
chr19	10795371	10812377
chr19	1270927	1274440
chr19	15170085	15170825
chr19	19630470	19642619
chr19	32731724	32742900
chr19	32859840	32860650
chr19	33137676	33144932
chr19	37155864	37170054
chr19	37156228	37170054
chr19	37344609	37347872
chr19	37520183	37533403
chr19	37583970	37586745
chr19	37758475	37773751
chr19	38343579	38344368
chr19	44336622	44343302
chr19	49696481	49697465
chr19	56511516	56516401
chr19	56511516	56518577
chr19	57232032	57235058
chr19	5724807	5737205
chr19	5724807	5739439
chr1	100152384	100168594
chr1	100156171	100168594
chr1	108727807	108733532
chr1	113856381	113859460
chr1	148863249	148889894

chr1	150747776	150757980
chr1	152332582	152341210
chr1	15308612	15313187
chr1	154117724	154143577
chr1	154331678	154331953
chr1	167875130	167905198
chr1	16929845	16940093
chr1	170041676	170055436
chr1	179368799	179370078
chr1	185180390	185190642
chr1	197539963	197583253
chr1	197539963	197772932
chr1	20879322	20895501
chr1	20879322	20904971
chr1	20886200	20899943
chr1	20886200	20904971
chr1	20892618	21050994
chr1	20969473	21002808
chr1	20969473	21050994
chr1	20969473	21089213
chr1	20969473	21111383
chr1	20980333	21002808
chr1	20980333	21050994
chr1	20980333	21089213
chr1	222538647	222542155
chr1	222651976	222652332
chr1	222990624	222992110
chr1	223132474	223134864
chr1	225152004	225168028
chr1	225152004	225185425
chr1	225152004	225240822
chr1	225152004	225259253
chr1	225304907	225324832
chr1	228352950	228362755
chr1	244477540	244499079
chr1	35831426	35834174
chr1	43197552	43199503
chr1	53266472	53280715
chr1	63419369	63429126
chr1	63478763	63510181
chr1	66862146	66905375
chr1	7732447	7738482
chr1	7820466	7827815
chr1	7937689	7940933
chr1	85816787	85849406

chr1	91394092	91396405
chr1	95143890	95230994
chr20	15933275	15987158
chr20	15967552	15987158
chr20	16356329	16381747
chr20	23379866	23403072
chr20	25634048	25674007
chr20	35595476	35595754
chr20	43528656	43540815
chr20	43534827	43536458
chr20	50611180	50630856
chr20	51788860	51792352
chr20	58691723	58701158
chr21	28163601	28225991
chr21	33856735	33867331
chr22	17497402	17500735
chr22	26774155	26778585
chr22	38605253	38623279
chr22	38665545	38668132
chr22	43755765	43773103
chr22	50406688	50441026
chr2	110143541	110161697
chr2	112074911	112089718
chr2	112074911	112107840
chr2	112081056	112107840
chr2	11455341	11457233
chr2	151548307	151553502
chr2	151841902	151860820
chr2	159714556	159729175
chr2	159714556	159743253
chr2	164692220	164705105
chr2	189689526	189696369
chr2	189752848	189754064
chr2	190900563	190954818
chr2	190927305	190954818
chr2	196047351	196051249
chr2	196646340	196658860
chr2	196656632	196701180
chr2	196666718	196732152
chr2	196676142	196695159
chr2	196890827	196898369
chr2	196890827	196902742
chr2	197000418	197005910
chr2	200412310	200440784
chr2	201307518	201309250

chr2	201565728	201582217
chr2	201565728	201604697
chr2	201854873	201880167
chr2	201854873	201890927
chr2	202785917	202796964
chr2	202785917	202811789
chr2	202917876	202941980
chr2	202917876	202961426
chr2	202952558	202961426
chr2	202977268	202982441
chr2	203110149	203136774
chr2	203122253	203136774
chr2	203133057	203138748
chr2	205763673	205766803
chr2	219461881	219469379
chr2	222887829	222909150
chr2	222887829	222919202
chr2	233540661	233557019
chr2	237717761	237727935
chr2	238332823	238344598
chr2	241094276	241097427
chr2	32087491	32089605
chr2	33257283	33280158
chr2	33257442	33280158
chr2	33280038	33309556
chr2	39056701	39067753
chr2	54891053	54916935
chr2	55206269	55209831
chr2	60760133	60775123
chr2	62858405	62874532
chr2	67399168	67399626
chr2	75687826	75706651
chr2	84604338	84611854
chr2	85847860	85863485
chr2	97167576	97209872
chr2	97200452	97219246
chr2	97578905	97580568
chr2	99064938	99081397
chr3	101361714	101417790
chr3	101364833	101366769
chr3	101458954	101501119
chr3	113249721	113250833
chr3	113904139	113915838
chr3	12742453	12748792
chr3	132446474	132447939

chr3	132446474	132456832
chr3	132733323	132828006
chr3	149140003	149163949
chr3	149141016	149153648
chr3	150423048	150438354
chr3	150669295	150698882
chr3	151394655	151416422
chr3	151411277	151416422
chr3	168024525	168032852
chr3	169107925	169131531
chr3	17042873	17068065
chr3	171123595	171167270
chr3	179198749	179230376
chr3	183276871	183295477
chr3	185428496	185466963
chr3	187243483	187250329
chr3	19342586	19347965
chr3	197075836	197091026
chr3	197075836	197119530
chr3	197119409	197194589
chr3	198063717	198077288
chr3	198076795	198077288
chr3	27256295	27322261
chr3	27291261	27352919
chr3	37094791	37149038
chr3	37503177	37542585
chr3	42757065	42758351
chr3	45833049	45838051
chr3	52327881	52386345
chr3	52394464	52395678
chr3	55950424	56007321
chr3	57501320	57504204
chr3	58390581	58412963
chr3	69937821	69951886
chr3	93995873	94039988
chr3	94035330	94043240
chr4	112637643	112644488
chr4	119245336	119273708
chr4	119538959	119607297
chr4	128082164	128179512
chr4	145642362	145646156
chr4	168302299	168311365
chr4	169463242	169479534
chr4	169555719	169602678
chr4	1893729	1904378

chr4	1893729	1935262
chr4	2072967	2229243
chr4	2128112	2229243
chr4	2170678	2213126
chr4	2179307	2229243
chr4	2984512	3029409
chr4	40097226	40113508
chr4	40131800	40152903
chr4	42503449	42543986
chr4	47568836	47576973
chr4	48163701	48176186
chr4	48831226	48833481
chr4	5565257	5584850
chr4	55999301	55999645
chr4	6078938	6085629
chr4	6923372	7001251
chr4	6923372	7014557
chr4	6967303	7001251
chr4	6967303	7004924
chr4	70632650	70637843
chr4	80869992	80944939
chr4	82904735	82907935
chr4	83416730	83421736
chr4	83416730	83432267
chr4	84631818	84635351
chr4	84633856	84640990
chr4	88107183	88140014
chr4	88383220	88417579
chr4	88404875	88405613
chr4	88649839	88658491
chr4	88652011	88676423
chr4	88662430	88687299
chr4	94584985	94640450
chr5	110605108	110627880
chr5	110605108	110642371
chr5	110638197	110642371
chr5	112194567	112234755
chr5	112204374	112209982
chr5	112204374	112239737
chr5	112209891	112266330
chr5	113340518	113385212
chr5	118510887	118522846
chr5	134302589	134350427
chr5	137634036	137639977
chr5	13817547	13841904

chr5	150942217	150943872
chr5	159332781	159354121
chr5	171293882	171296014
chr5	180136377	180138058
chr5	32774707	32784883
chr5	34020586	34035758
chr5	34023908	34035758
chr5	34686871	34688248
chr5	35691036	35709121
chr5	42514079	42565944
chr5	5303267	5306728
chr5	5303267	5318281
chr5	55441850	55475450
chr5	58993371	59038971
chr5	69355648	69366595
chr5	73554321	73561288
chr5	77311993	77331459
chr5	79277909	79306461
chr5	79300152	79306461
chr5	82280959	82304850
chr5	82280959	82305481
chr6	104730302	104750472
chr6	104824813	104843298
chr6	104824813	104849246
chr6	104824813	104852371
chr6	10907541	10928450
chr6	10935057	10958875
chr6	109419186	109436199
chr6	109426846	109431345
chr6	10955115	10963843
chr6	109641517	109662663
chr6	109644613	109662663
chr6	116877795	116895207
chr6	118470536	118483858
chr6	119002898	119020159
chr6	119188404	119201347
chr6	121160009	121294660
chr6	121170404	121242339
chr6	12129758	12135892
chr6	125786136	125815404
chr6	131851141	131869489
chr6	131882344	131886724
chr6	138812837	138846677
chr6	152233780	152249262
chr6	158661268	158665613

chr6	169582835	169636504
chr6	29621100	29631599
chr6	31656471	31657723
chr6	3410187	3432338
chr6	41108304	41123249
chr6	41117297	41118184
chr6	41117297	41123249
chr6	44360445	44370113
chr6	56735227	56900621
chr6	71429938	71435356
chr6	83145523	83152367
chr6	83147235	83156103
chr6	84160811	84163270
chr6	84444386	84474929
chr6	87409834	87428824
chr6	89265612	89268096
chr7	100591666	100594541
chr7	122436359	122441575
chr7	123496695	123503363
chr7	151116792	151123886
chr7	154946359	154948090
chr7	156642976	156644757
chr7	18829160	18874596
chr7	2700233	2709816
chr7	30872242	30882993
chr7	35117747	35132369
chr7	36335045	36336297
chr7	40237634	40316855
chr7	44624316	44666851
chr7	44645326	44666851
chr7	47792626	47821186
chr7	5623179	5624055
chr7	64336258	64336982
chr7	64814917	64820323
chr7	6521428	6521957
chr7	65964322	65976202
chr7	65970281	65976202
chr7	65974294	65976202
chr7	65974294	65980409
chr7	76005258	76030524
chr7	77330238	77404615
chr7	77349350	77355647
chr7	77349350	77382643
chr7	78343777	78369213
chr7	87359205	87361852

chr7	90288233	90304820
chr7	93348710	93353761
chr8	100165813	100177941
chr8	103404775	103413531
chr8	103885297	103916537
chr8	103910133	103961133
chr8	124503839	124523002
chr8	124555741	124557875
chr8	132727797	132757294
chr8	17713213	17755961
chr8	27322401	27325681
chr8	37473077	37476012
chr8	61576771	61653660
chr8	61618977	61638363
chr8	61618977	61644632
chr8	61618977	61646878
chr8	61618977	61653660
chr8	61618977	61681036
chr8	61618977	61684188
chr8	66874052	66883994
chr8	75543810	75559037
chr8	98222478	98254087
chr8	98236154	98254087
chr9	104858521	104889195
chr9	109258176	109268433
chr9	113423325	113428963
chr9	120989045	120997774
chr9	121146107	121160302
chr9	128485364	128494668
chr9	137505613	137506083
chr9	15744272	15784694
chr9	2028986	2123937
chr9	2028986	2161903
chr9	2028986	2186228
chr9	2039465	2058464
chr9	32987543	32989895
chr9	33503826	33510711
chr9	36087693	36102230
chr9	37881517	37899950
chr9	62866966	62869304
chr9	68378198	68392473
chr9	70305246	70324143
chr9	72223119	72231181
chr9	83880296	83908652
chr9	97246095	97251557

chrX	107526581	107533550
chrX	111192126	111196640
chrX	111708026	111736879
chrX	111708964	111736879
chrX	111711674	111728305
chrX	118566573	118630490
chrX	118605237	118630490
chrX	139585065	139588438
chrX	14895307	14911413
chrX	14900574	14914599
chrX	17006220	17029623
chrX	17052381	17062532
chrX	22038468	22090497
chrX	22090428	22133624
chrX	22219035	22227611
chrX	38273385	38276772
chrX	47982948	47983411
chrX	77616612	77664778
chrX	78014661	78033821
chrX	84116228	84135210
	**Intergenic (1826)**	
chr10	112888734	112906213
chr10	112890965	112906213
chr10	112905976	112906213
chr10	12269937	12270626
chr10	124750213	124750730
chr10	130208053	130252065
chr10	133491221	133498005
chr10	133493286	133498005
chr10	15186725	15189954
chr10	17744966	17746329
chr10	21316071	21321131
chr10	21697452	21698891
chr10	22448724	22448989
chr10	258744	263658
chr10	263203	263658
chr10	26968934	26982813
chr10	27542752	27578713
chr10	27566831	27569933
chr10	27566831	27578713
chr10	27578370	27578713
chr10	27629308	27635429
chr10	31959434	31963390
chr10	32653008	32653655
chr10	32985384	33092437

chr10	33164525	33170630
chr10	34251601	34302429
chr10	34306571	34306996
chr10	34993185	34993395
chr10	35135928	35136591
chr10	35296455	35321112
chr10	35320855	35321670
chr10	37729820	37745548
chr10	37729820	37749733
chr10	37729820	37749758
chr10	37735131	37739714
chr10	37738517	37744286
chr10	37739714	37742449
chr10	37742745	37746074
chr10	37744214	37749758
chr10	37747195	37749758
chr10	37775343	37776570
chr10	46003933	46004526
chr10	47501279	47501514
chr10	55893682	55895668
chr10	57790427	57853636
chr10	57991695	58005413
chr10	57991695	58015971
chr10	58005316	58023651
chr10	6157523	6157996
chr10	65824049	65861593
chr10	719956	720644
chr10	72561792	72562235
chr10	72987962	72988368
chr10	73739926	73740266
chr10	73776943	73778857
chr10	75461385	75461842
chr10	75547030	75547662
chr10	77787669	77789362
chr10	88016242	88069642
chr10	89017650	89035982
chr10	89076695	89138518
chr10	89083245	89085393
chr10	89792838	89794672
chr10	99857938	99859574
chr11	102269129	102274835
chr11	107177045	107180386
chr11	107308282	107311232
chr11	107309795	107310583
chr11	108109931	108110620

chr11	12222663	12226334
chr11	122291846	122301423
chr11	122486787	122487301
chr11	125530343	125530815
chr11	125692887	125693581
chr11	126179187	126180907
chr11	126181494	126183087
chr11	126182836	126183087
chr11	126364530	126381942
chr11	129266866	129267361
chr11	129266866	129269715
chr11	17020472	17023656
chr11	17225495	17226199
chr11	18592715	18593459
chr11	23730310	23731658
chr11	28535827	28564328
chr11	28562227	28623996
chr11	28702686	28741908
chr11	28741824	28811623
chr11	28846789	28922625
chr11	29037741	29044660
chr11	29037741	29044740
chr11	29037741	29063536
chr11	29037741	29211765
chr11	29044444	29063536
chr11	29097063	29106561
chr11	29160191	29211765
chr11	29211654	29282610
chr11	29230935	29384112
chr11	29282461	29285063
chr11	29282461	29384112
chr11	29381072	29384112
chr11	29381090	29384112
chr11	29383255	29384112
chr11	2941491	2942888
chr11	29455710	29518362
chr11	29475513	29481582
chr11	29476144	29484062
chr11	29476144	29487481
chr11	29586604	29596851
chr11	34147542	34149553
chr11	3701460	3704812
chr11	46709152	46709456
chr11	46740479	46740780
chr11	47536527	47537526

chr11	527208	527626
chr11	59547654	59548685
chr11	61374864	61375417
chr11	61467889	61468714
chr11	62768351	62768619
chr11	62872506	62873008
chr11	645591	646016
chr11	645595	646021
chr11	65326387	65326786
chr11	65824522	65826609
chr11	65826275	65826609
chr11	65900777	65901089
chr11	66956079	66956338
chr11	67861939	67873341
chr11	67873205	67873549
chr11	67873205	67874489
chr11	6893000	6898228
chr11	69316833	69341678
chr11	69371402	69373513
chr11	73929003	73929775
chr11	74731729	74732126
chr11	76820299	76843732
chr11	77529947	77560875
chr11	813246	814265
chr11	83075431	83076555
chr11	83081534	83081800
chr11	85771443	85773593
chr11	8669671	8670034
chr11	88399681	88400072
chr11	9102401	9122894
chr12	100177042	100180716
chr12	102205023	102269646
chr12	102257214	102282298
chr12	102257214	102315490
chr12	102257214	102324877
chr12	102267186	102275759
chr12	102269599	102315490
chr12	102277673	102315490
chr12	102279546	102282298
chr12	102281265	102293277
chr12	104381785	104387984
chr12	104381785	104430659
chr12	104940287	104940699
chr12	106051276	106059414
chr12	106899056	106902878

chr12	108433129	108443314
chr12	108832488	108832639
chr12	117982089	117991833
chr12	118116509	118117882
chr12	121868096	121868614
chr12	125027560	125027801
chr12	12832701	12839222
chr12	12833045	12839204
chr12	12839073	12846599
chr12	12846334	12846599
chr12	12855170	12859577
chr12	12855170	12870651
chr12	133014823	133015061
chr12	133028818	133028992
chr12	14734569	14769009
chr12	16736241	16749876
chr12	19280792	19281492
chr12	19552852	19553392
chr12	19552852	19590799
chr12	19595996	19603313
chr12	27034386	27034782
chr12	27046157	27066226
chr12	27144991	27148604
chr12	28204092	28226199
chr12	31278181	31279153
chr12	38432081	38591924
chr12	42172139	42210680
chr12	42210354	42210680
chr12	42210354	42212484
chr12	4567954	4581004
chr12	46272901	46275375
chr12	49534542	49537096
chr12	49775578	49776114
chr12	5159273	5171889
chr12	5160306	5171889
chr12	5160306	5198646
chr12	5169153	5171889
chr12	52266545	52269822
chr12	5270204	5276889
chr12	53530897	53531153
chr12	5358269	5367934
chr12	5358269	5371598
chr12	5388513	5405456
chr12	56728537	56728719
chr12	56980073	56988634

chr12	57019166	57019467
chr12	57395748	57398198
chr12	57395748	57398202
chr12	57398781	57399150
chr12	6393795	6394999
chr12	66796540	66892938
chr12	66891404	66892371
chr12	66891900	66907142
chr12	66891900	66907196
chr12	66891900	66938347
chr12	66891900	66992939
chr12	66925636	66925967
chr12	67318683	67329041
chr12	67318683	67349398
chr12	67419260	67421287
chr12	7042960	7044036
chr12	73329105	73363397
chr12	73363072	73364301
chr12	73459368	73477374
chr12	76717906	76730079
chr12	79570785	79571396
chr12	79715550	79720342
chr12	85342471	85355059
chr12	85342471	85424204
chr12	85353204	85424204
chr12	85399492	85424204
chr12	85414065	85424204
chr12	85594466	85702968
chr12	85685057	85713416
chr12	87377107	87415076
chr12	87527757	87528707
chr12	87548774	87651983
chr12	87648516	87651983
chr12	87648516	87653185
chr12	87648516	87659196
chr12	87648516	87684654
chr12	87648516	87715112
chr12	87648520	87715092
chr12	87659114	87690206
chr12	87684493	87707705
chr12	87690236	87707705
chr12	87707646	87715112
chr12	87707646	87721292
chr12	87707646	87727823
chr12	87721171	87727823

chr12	93977664	93977875
chr12	979969	980403
chr12	98539135	98539517
chr12	98548946	98549335
chr13	112633024	112641755
chr13	24517746	24518043
chr13	28628543	28636986
chr13	28690341	28690687
chr13	28839777	28893763
chr13	28890974	28893821
chr13	28890974	28894197
chr13	28936696	28948737
chr13	29153906	29154159
chr13	29158104	29164725
chr13	29222511	29224162
chr13	29372732	29379127
chr13	29403330	29403810
chr13	41299502	41303695
chr13	46324552	46325246
chr13	49976332	49978189
chr13	50425577	50505684
chr13	80217804	80264374
chr13	87687854	87715494
chr13	99071509	99072508
chr14	102051420	102051739
chr14	102743477	102743710
chr14	102752743	102753028
chr14	103356914	103357310
chr14	103360364	103360885
chr14	21163022	21163290
chr14	22695988	22717220
chr14	24170833	24171379
chr14	24196994	24200642
chr14	26093845	26145822
chr14	26724747	26735372
chr14	26729558	26731156
chr14	27311022	27322141
chr14	27999519	28006399
chr14	28072153	28072568
chr14	29502918	29556457
chr14	34551713	34554912
chr14	34647791	34648139
chr14	34647791	34648300
chr14	34751310	34751468
chr14	34962228	34962438

chr14	34969169	34972064
chr14	39470317	39497592
chr14	39655503	39655808
chr14	39761663	39763587
chr14	40216281	40264929
chr14	45260629	45261944
chr14	45516585	45605277
chr14	53510724	53516145
chr14	53510724	53516374
chr14	55354500	55355222
chr14	57548573	57550572
chr14	59389542	59461173
chr14	64336507	64338070
chr14	64353952	64357503
chr14	71400793	71418196
chr14	71413947	71418196
chr14	71413947	71421869
chr14	71413947	71467247
chr14	72582011	72582159
chr14	83646543	83666209
chr15	100855230	100860035
chr15	101162295	101175622
chr15	22976427	22976961
chr15	24998325	25000606
chr15	24998969	25020761
chr15	25019026	25020761
chr15	25019026	25022964
chr15	25019026	25024630
chr15	25019026	25028668
chr15	25067408	25070259
chr15	25067411	25070259
chr15	30798203	30798460
chr15	34488962	34490796
chr15	39860819	39862419
chr15	39913968	39914423
chr15	40395170	40395686
chr15	40541597	40552237
chr15	40962612	40964740
chr15	41308182	41308817
chr15	48680668	48717169
chr15	48717017	48724029
chr15	48717017	48729540
chr15	49106200	49121829
chr15	50706387	50707157
chr15	56054841	56057637

chr15	56483702	56497073
chr15	56827523	56865889
chr15	56847323	56848600
chr15	56847323	56850186
chr15	60964363	61016035
chr15	61014451	61016035
chr15	61015797	61016035
chr15	61015797	61034926
chr15	61015797	61035277
chr15	61015797	61041134
chr15	61015797	61047560
chr15	61015797	61072325
chr15	61015797	61094611
chr15	61015797	61112119
chr15	61015797	61221110
chr15	61094355	61104228
chr15	61094355	61112119
chr15	61220726	61223307
chr15	62428902	62429322
chr15	62579504	62589762
chr15	62589686	62618475
chr15	63141000	63141533
chr15	65149782	65150169
chr15	69462011	69462315
chr15	72605060	72609197
chr15	72608759	72612326
chr15	74483854	74484215
chr15	74489588	74491582
chr15	75933741	75934479
chr15	79356752	79383106
chr15	79411399	79413557
chr15	80959151	80970659
chr15	82627164	82640865
chr15	82640656	82641682
chr15	82958974	82959308
chr15	87406362	87422036
chr15	89656386	89656979
chr15	94602337	94628223
chr15	96342880	96361289
chr15	99808146	99877148
chr16	11515928	11522112
chr16	11933516	11937668
chr16	11970175	11970424
chr16	11973082	11973677
chr16	11974324	11976159

chr16	13048271	13065154
chr16	13060050	13060527
chr16	13060267	13063698
chr16	13242389	13244460
chr16	13471569	13474341
chr16	15653966	15654368
chr16	15973971	15974814
chr16	1886804	1889295
chr16	19703041	19704172
chr16	22170832	22172331
chr16	22412979	22416463
chr16	23453613	23454205
chr16	29597457	29597880
chr16	30035350	30046387
chr16	30059405	30059657
chr16	30800204	30802470
chr16	30830693	30831067
chr16	34265957	34266371
chr16	3640714	3643036
chr16	436216	436576
chr16	57209599	57210431
chr16	66598177	66598840
chr16	67102412	67102635
chr16	67504731	67505766
chr16	69734290	69734540
chr16	69737328	69738098
chr16	70226769	70233525
chr16	70226769	70246610
chr16	71810145	71810338
chr16	72713332	72747182
chr16	742249	742543
chr16	74613179	74614099
chr16	78994239	78996356
chr16	81312727	81313120
chr16	85264477	85265058
chr16	86527367	86528939
chr16	8883209	8884465
chr16	89266254	89267315
chr16	9222684	9222832
chr17	1045081	1045650
chr17	10678037	10679055
chr17	1258559	1259559
chr17	13865078	13900211
chr17	13892724	13898732
chr17	13892724	13933185

chr17	14209508	14215937
chr17	15265455	15267311
chr17	15429466	15429636
chr17	1671773	1672389
chr17	18704618	18705164
chr17	20056264	20057817
chr17	30645358	30648456
chr17	30771171	30771607
chr17	30775868	30779018
chr17	30776296	30780739
chr17	31001737	31002113
chr17	31021909	31024426
chr17	31071911	31073677
chr17	31327648	31358632
chr17	32344539	32347388
chr17	34966933	34967891
chr17	35468136	35468822
chr17	3705080	3705645
chr17	3804532	3805018
chr17	38319328	38319816
chr17	39537817	39538644
chr17	39556285	39564914
chr17	39564759	39566892
chr17	40119208	40119722
chr17	43569432	43609933
chr17	44457152	44457834
chr17	44492223	44493162
chr17	45538321	45538589
chr17	46963696	46965039
chr17	46963834	46965039
chr17	46966533	46970309
chr17	47054117	47072559
chr17	516109	521993
chr17	517939	518459
chr17	55175858	55201712
chr17	58713743	58714029
chr17	62474354	62476831
chr17	66906254	66906574
chr17	68209653	68210465
chr17	76097131	76097472
chr17	76260836	76261264
chr17	76689364	76689768
chr17	77176197	77176681
chr17	77293431	77293827
chr17	77340222	77341697

chr17	80335153	80335647
chr17	81883765	81884986
chr17	8202952	8203621
chr17	8203504	8203901
chr17	82731375	82732875
chr17	83167543	83169611
chr17	8364134	8364805
chr18	14335463	14335900
chr18	14404798	14408096
chr18	21636026	21636418
chr18	35322387	35327063
chr18	41351660	41437441
chr18	55786585	55791898
chr18	55788612	55814488
chr18	55834162	55839660
chr18	56050079	56050896
chr18	56166569	56168315
chr18	59392543	59392845
chr18	61171847	61203186
chr18	70526064	70531787
chr19	11647323	11652053
chr19	11648357	11650417
chr19	11648357	11652053
chr19	11648357	11654411
chr19	11671295	11672193
chr19	1190028	1190991
chr19	12079094	12079397
chr19	12089873	12090324
chr19	12121605	12121804
chr19	12230459	12234908
chr19	12244580	12250368
chr19	12249977	12250368
chr19	12817008	12818442
chr19	13837562	13838018
chr19	15336491	15344366
chr19	16237487	16238833
chr19	16635806	16636479
chr19	16640919	16641651
chr19	16642326	16643064
chr19	18952720	18952909
chr19	19366690	19367546
chr19	19383482	19383697
chr19	19786608	19789821
chr19	21062605	21077216
chr19	265183	265972

chr19	27403133	27523546
chr19	27472041	27500555
chr19	27472041	27523546
chr19	27472554	27494126
chr19	27472554	27521041
chr19	27493583	27494240
chr19	27773121	27773463
chr19	27918682	27919858
chr19	29645261	29657943
chr19	31123852	31126079
chr19	31228004	31232280
chr19	32339478	32339840
chr19	32714249	32714692
chr19	3568768	3569352
chr19	36534860	36535380
chr19	36678044	36680479
chr19	37001330	37001683
chr19	37246075	37246491
chr19	37322232	37323521
chr19	37729903	37730337
chr19	38963518	38964574
chr19	39820476	39821292
chr19	4076774	4078125
chr19	41369591	41370282
chr19	4269309	4270435
chr19	44181857	44196151
chr19	44669354	44670212
chr19	45187330	45187740
chr19	46891726	46891999
chr19	46895449	46895817
chr19	48949827	48950325
chr19	49052208	49052733
chr19	49255217	49256275
chr19	49331616	49332320
chr19	49719456	49719935
chr19	50795740	50797396
chr19	52242756	52262743
chr19	52244024	52250436
chr19	52587790	52592228
chr19	52588531	52592228
chr19	52670433	52699238
chr19	53423061	53427219
chr19	53536089	53536617
chr19	54398831	54407890
chr19	55040478	55040855

chr19	56363234	56365057
chr19	5670412	5672579
chr19	57180711	57181259
chr19	57784189	57784640
chr19	57790273	57799458
chr19	57790972	57791802
chr19	57819567	57820549
chr19	58265572	58265928
chr19	58269675	58270548
chr19	58462262	58462504
chr19	58492736	58495666
chr19	5911102	5912257
chr19	7089065	7089451
chr19	7095599	7095958
chr19	7945435	7946046
chr19	9292012	9292882
chr19	9563960	9564252
chr1	1008193	1009370
chr1	10395888	10396330
chr1	1063078	1066936
chr1	109063981	109064239
chr1	109210795	109211119
chr1	110347572	110356031
chr1	110351190	110356031
chr1	111565307	111566042
chr1	112858186	112858479
chr1	112877729	112885053
chr1	112996840	112999109
chr1	112998929	113008360
chr1	113127852	113129039
chr1	113932623	113933609
chr1	12015360	12017007
chr1	120419849	120427847
chr1	120489810	120493301
chr1	120530295	120557127
chr1	120533815	120535809
chr1	120741807	120763709
chr1	120741807	120793496
chr1	120763627	120785233
chr1	120811885	120816827
chr1	13843031	13864675
chr1	143635508	143635780
chr1	145422795	145423431
chr1	148657092	148657384
chr1	149100740	149101384

chr1	149557068	149557886
chr1	149849296	149849530
chr1	150620889	150621072
chr1	151195658	151195989
chr1	152226425	152227051
chr1	152226425	152251758
chr1	152248894	152251758
chr1	155243867	155244335
chr1	155243879	155244335
chr1	155634769	155648534
chr1	15792836	15796943
chr1	160895644	160895878
chr1	16164470	16168253
chr1	16633094	16635168
chr1	172797452	172835377
chr1	172797452	172933640
chr1	172797452	172933643
chr1	172817213	172851482
chr1	172817552	172851482
chr1	172835250	172933643
chr1	172886226	172933643
chr1	172924570	172926734
chr1	172924570	172933643
chr1	172924598	172933643
chr1	173073952	173154199
chr1	173798499	173799294
chr1	173859259	173859471
chr1	173862639	173863183
chr1	178123047	178125501
chr1	178123055	178125501
chr1	178123055	178134720
chr1	178123055	178138808
chr1	178123718	178134720
chr1	180284746	180284905
chr1	184093624	184100110
chr1	185370667	185374556
chr1	201516623	201516895
chr1	201622852	201623606
chr1	204558640	204579513
chr1	204565209	204580409
chr1	207815198	207817414
chr1	207815928	207817414
chr1	22154902	22155903
chr1	22154902	22157202
chr1	22172054	22188935

chr1	222594235	222603276
chr1	224496496	224515876
chr1	224546828	224566264
chr1	226140556	226140977
chr1	226896974	226897829
chr1	226897719	226908683
chr1	226909701	226917660
chr1	23240008	23249938
chr1	23287363	23294197
chr1	23733089	23733508
chr1	241892721	241893150
chr1	244772338	244772564
chr1	246435944	246441299
chr1	246435944	246445087
chr1	246437147	246440124
chr1	246437401	246481309
chr1	247145051	247152902
chr1	247199359	247200193
chr1	247333134	247334426
chr1	26261039	26261205
chr1	27002850	27003938
chr1	28243521	28246753
chr1	28513150	28514090
chr1	29126503	29129488
chr1	31394555	31402685
chr1	31920365	31920647
chr1	32905661	32905868
chr1	35117570	35117855
chr1	40430371	40434103
chr1	42480460	42498916
chr1	42490157	42498916
chr1	42728466	42729146
chr1	44050949	44061227
chr1	44773884	44774644
chr1	45248573	45264498
chr1	47368486	47373075
chr1	51507535	51508771
chr1	54420598	54433576
chr1	58713055	58718748
chr1	58713055	58723169
chr1	58714831	58718748
chr1	58715493	58718748
chr1	60254477	60258687
chr1	67398604	67405169
chr1	68920816	68923584

chr1	72341546	72364283
chr1	72363773	72410059
chr1	72380057	72410059
chr1	72391404	72410059
chr1	72749165	72766687
chr1	72749165	72874338
chr1	72917102	72940591
chr1	7703110	7703553
chr1	78025017	78026688
chr1	805798	810170
chr1	84104855	84105388
chr1	84903244	84916794
chr1	93412091	93437654
chr1	97974309	97975100
chr1	98393054	98395390
chr1	9991623	9996873
chr20	11899588	11900436
chr20	32860781	32867747
chr20	32929527	32931336
chr20	32930397	32931336
chr20	33550640	33556738
chr20	35216747	35218061
chr20	37873078	37873498
chr20	44305744	44306001
chr20	44305788	44305973
chr20	44305803	44305973
chr20	44469176	44472247
chr20	45451997	45456703
chr20	45931068	45931482
chr20	51349940	51368853
chr20	51366448	51368853
chr20	51900121	51913814
chr20	51976048	51976472
chr20	52043757	52045258
chr20	5279399	5282311
chr20	5727154	5728030
chr20	61285736	61286114
chr20	61963440	61964030
chr20	62297323	62297672
chr20	6355479	6356702
chr20	63834767	63834973
chr20	64151696	64157313
chr20	8962195	8967355
chr20	9096286	9110029
chr21	10325780	10340478

chr21	14084350	14084549
chr21	14094432	14094759
chr21	14825522	14857708
chr21	14853273	14857708
chr21	14853273	14870273
chr21	18760025	18814661
chr21	21656938	21665196
chr21	27354996	27398565
chr21	28577218	28626676
chr21	29384146	29423167
chr21	29410254	29411624
chr21	29411521	29423167
chr21	29458241	29459812
chr21	36016948	36018807
chr21	37570169	37580835
chr21	43882503	43887405
chr21	45310455	45311429
chr21	45668873	45673735
chr21	45732674	45749475
chr21	45749380	45755452
chr21	45788297	45788870
chr21	46570629	46571064
chr22	16745350	16748600
chr22	17957194	17958362
chr22	22294840	22297745
chr22	25370869	25371865
chr22	25509182	25510862
chr22	25509182	25510908
chr22	28790719	28791819
chr22	29320057	29321054
chr22	29497680	29498520
chr22	30650239	30651295
chr22	30692230	30692752
chr22	31109978	31112209
chr22	31648100	31650778
chr22	32265430	32268579
chr22	32265430	32273507
chr22	32268287	32273507
chr22	35762776	35763331
chr22	35982035	35992774
chr22	35992675	35996030
chr22	36002061	36002651
chr22	37587762	37588446
chr22	38286326	38286793
chr22	39526662	39527175

chr22	40886383	40933336
chr22	40927396	40929273
chr22	42149927	42153200
chr22	42299996	42301247
chr22	42307553	42307760
chr22	44862987	44865175
chr22	45189916	45190545
chr22	45239461	45239774
chr22	47860464	47862122
chr22	47861991	47928944
KI270733v1_r	167967	168876
chr2	10138415	10138822
chr2	10272428	10272718
chr2	111208487	111208936
chr2	111233627	111239996
chr2	111233627	111248241
chr2	111239801	111248241
chr2	111700409	111700812
chr2	113673581	113674253
chr2	117839974	117842201
chr2	118142795	118278471
chr2	119685657	119686874
chr2	127431666	127436297
chr2	127461486	127470385
chr2	130066454	130067902
chr2	131684708	131688303
chr2	134171740	134190248
chr2	138101132	138212897
chr2	138290450	138306284
chr2	138299460	138305527
chr2	138348690	138367611
chr2	146915212	146954229
chr2	14986663	14998224
chr2	151789751	151791908
chr2	152830316	152859498
chr2	155698966	155704210
chr2	155759630	155814067
chr2	155809927	155811890
chr2	155809927	155811964
chr2	155809927	155812794
chr2	155906359	155914227
chr2	173630587	173693837
chr2	173657935	173693837
chr2	173692683	173761730
chr2	173739388	173743522

chr2	173761578	173811253
chr2	173800481	173806580
chr2	182672080	182674518
chr2	183178806	183182680
chr2	183178806	183203227
chr2	183182556	183188763
chr2	183203058	183203227
chr2	183242785	183334209
chr2	183316754	183334209
chr2	186215018	186249732
chr2	186364725	186365129
chr2	186416754	186456851
chr2	187117035	187126675
chr2	18719798	18919377
chr2	190593188	190593723
chr2	190606955	190628715
chr2	19065980	19125387
chr2	191613707	191636219
chr2	191779218	191785489
chr2	192019655	192048848
chr2	199002705	199026655
chr2	201743070	201743516
chr2	202879986	202880329
chr2	206121759	206122352
chr2	206121759	206122357
chr2	206841106	206879440
chr2	206841106	206881891
chr2	206866696	206879440
chr2	206866696	206881891
chr2	207148981	207149200
chr2	207416060	207417117
chr2	219714256	219794170
chr2	219787564	219794170
chr2	219792376	219794170
chr2	219792376	219803218
chr2	219792376	219840845
chr2	219917762	219940777
chr2	220021475	220024789
chr2	220102311	220137863
chr2	220630378	220634128
chr2	223506277	223514236
chr2	224709887	224717763
chr2	229755717	229755925
chr2	231504134	231507255
chr2	236264772	236268343

chr2	237022685	237023199
chr2	240686437	240688825
chr2	241687940	241690653
chr2	24510968	24564430
chr2	24554388	24556055
chr2	24554388	24564430
chr2	24556014	24564430
chr2	24562705	24564430
chr2	24563903	24564430
chr2	25271005	25273056
chr2	25709302	25722165
chr2	25717335	25722165
chr2	26147143	26147326
chr2	30859572	30867375
chr2	31662857	31710268
chr2	32825389	32826130
chr2	33612653	33616692
chr2	3481534	3484771
chr2	37101922	37104132
chr2	38782704	38782963
chr2	46527940	46541453
chr2	46535812	46537576
chr2	53603390	53606080
chr2	53603390	53606776
chr2	54050957	54057360
chr2	54466952	54469986
chr2	54667090	54667447
chr2	55759195	55772912
chr2	63571374	63571626
chr2	63650658	63813707
chr2	70641336	70648030
chr2	74470991	74471298
chr2	74471001	74471298
chr2	74701769	74711243
chr2	75346612	75354979
chr2	78411079	78413176
chr2	86142340	86143695
chr2	87704332	87716790
chr2	94196054	94203287
chr2	95832385	95834565
chr2	95832478	95835422
chr2	95832599	95834977
chr2	95832805	95833829
chr2	95832805	95834565
chr2	95833435	95833750

chr2	95833435	95834172
chr2	95833616	95834565
chr2	95833905	95834224
chr2	95833932	95834148
chr2	95833932	95834565
chr2	95834069	95834898
chr2	95834350	95835127
chr2	95834358	95834565
chr2	95834358	95835113
chr2	95834358	95835127
chr2	95834969	95835319
chr2	95834977	95835127
chr2	95834977	95835643
chr2	97265915	97268415
chr2	97266224	97268003
chr2	97267377	97267900
chr2	97267654	97267971
chr2	97490434	97491145
chr2	97490456	97491153
chr3	100002937	100025558
chr3	100023273	100025558
chr3	104520555	104556044
chr3	104522668	104556044
chr3	104630284	104630579
chr3	104630288	104630579
chr3	104764378	104817613
chr3	104764378	104855451
chr3	104817472	104818695
chr3	104817472	104829713
chr3	104817472	104842744
chr3	104817472	104855451
chr3	104817472	104859959
chr3	106741560	106750905
chr3	106741560	106771161
chr3	106741560	106860748
chr3	110447146	110502152
chr3	11137517	11137804
chr3	113702116	113702863
chr3	115334016	115370176
chr3	115547207	115547488
chr3	125901432	125902054
chr3	129965700	129972108
chr3	129965700	129973298
chr3	136331500	136331826
chr3	137013938	137017166

chr3	138041072	138041482
chr3	139180289	139188610
chr3	142099681	142100454
chr3	144175682	144177055
chr3	144237757	144239267
chr3	144237757	144270349
chr3	144237757	144304804
chr3	145650517	145770312
chr3	145905807	146034688
chr3	145983607	145990927
chr3	145983607	145999967
chr3	145983607	146034688
chr3	145996235	146021522
chr3	146021432	146035473
chr3	146034531	146035473
chr3	149724079	149725135
chr3	150388470	150392667
chr3	153172835	153173746
chr3	153172835	153178789
chr3	155822066	155822327
chr3	155941307	155942177
chr3	158740388	158742481
chr3	158740388	158760383
chr3	158760157	158769482
chr3	15961765	15965796
chr3	161284716	161285241
chr3	161284716	161297701
chr3	169599485	169600053
chr3	170966935	170995025
chr3	170981932	170995025
chr3	174523412	174553757
chr3	178906401	178909706
chr3	178908521	178909706
chr3	178909598	179004184
chr3	184703405	184709731
chr3	185501901	185502509
chr3	188903796	188913462
chr3	189099952	189100204
chr3	189127056	189132697
chr3	189127056	189147647
chr3	189132398	189147647
chr3	194041371	194042073
chr3	194494049	194499043
chr3	195897236	195897424
chr3	195899562	195905431

chr3	195901415	195901805
chr3	195949241	195951037
chr3	196019879	196027260
chr3	196213105	196213911
chr3	196234243	196236776
chr3	196352539	196352733
chr3	197884861	197885357
chr3	197884867	197885322
chr3	24030726	24088061
chr3	27367058	27368099
chr3	27477943	27478257
chr3	31639987	31647091
chr3	37378201	37380440
chr3	42086498	42087400
chr3	42087103	42087400
chr3	42087103	42089932
chr3	44496470	44496763
chr3	44496947	44497510
chr3	45088447	45091881
chr3	48286245	48286930
chr3	48338109	48339212
chr3	48749712	48750170
chr3	53219987	53220547
chr3	56839843	56841523
chr3	56906758	56907091
chr3	67035116	67058596
chr3	67041864	67042057
chr3	67092518	67096603
chr3	67094831	67096603
chr3	67237400	67241819
chr3	67268452	67268994
chr3	72708843	72716617
chr3	81288078	81325698
chr3	8658924	8659801
chr3	8840195	8841539
chr3	8840195	8852948
chr3	8852878	8854774
chr3	99122563	99128056
chr3	99967340	100025558
chr4	103328124	103343235
chr4	103328124	103377175
chr4	103467291	103552602
chr4	103536345	103552602
chr4	103536345	103569402
chr4	103547853	103552602

chr4	103548742	103569402
chr4	10373786	10375292
chr4	110418220	110419105
chr4	110418240	110419105
chr4	110464974	110465858
chr4	114173379	114181726
chr4	1184266	1184627
chr4	119412351	119420155
chr4	119434739	119444433
chr4	120618642	120619403
chr4	1265339	1265907
chr4	1265339	1270295
chr4	127836257	127837054
chr4	128037203	128037435
chr4	128291799	128303035
chr4	128291799	128371745
chr4	128292933	128400298
chr4	129512441	129542703
chr4	129524104	129542703
chr4	129542572	129543727
chr4	13179195	13181146
chr4	13336892	13342312
chr4	13336892	13361633
chr4	139102050	139168042
chr4	139116648	139139497
chr4	139391882	139393722
chr4	139393590	139404490
chr4	139403848	139404168
chr4	139403848	139404343
chr4	139403848	139404490
chr4	141994573	141995619
chr4	143808313	143866076
chr4	143859020	143866076
chr4	148477372	148544584
chr4	151105475	151105738
chr4	151295513	151296481
chr4	151352899	151353391
chr4	156324094	156334361
chr4	159186641	159203232
chr4	159186641	159210583
chr4	159186641	159241368
chr4	159238808	159241368
chr4	168492665	168537786
chr4	173145210	173165132
chr4	173696609	173856466

chr4	173833922	173878280
chr4	176443896	176450824
chr4	183810105	183829990
chr4	183828177	183829990
chr4	187704963	187711957
chr4	189855885	189864786
chr4	189855885	189895643
chr4	189855885	189937333
chr4	189895531	189937333
chr4	22341019	22364337
chr4	23078613	23083788
chr4	23105501	23122436
chr4	23105501	23199724
chr4	23105501	23221222
chr4	23120319	23122436
chr4	23120319	23186098
chr4	23297260	23298605
chr4	23592788	23634627
chr4	2561205	2596329
chr4	2581036	2581348
chr4	2596083	2596329
chr4	26764302	26764737
chr4	34979647	35005121
chr4	34979647	35014118
chr4	34980486	35005121
chr4	34996297	35005121
chr4	34996297	35020067
chr4	36457605	36584796
chr4	36498426	36584796
chr4	36582656	36584796
chr4	378072	378363
chr4	378100	378319
chr4	4073032	4087393
chr4	4085120	4087393
chr4	4086657	4087339
chr4	4086657	4087393
chr4	4086657	4088409
chr4	41406138	41462597
chr4	41422005	41422269
chr4	41462421	41473195
chr4	53853884	53897869
chr4	53853884	53944997
chr4	6314982	6316181
chr4	66025498	66085941
chr4	67728539	67730209

chr4	67795747	67802411
chr4	67798638	67802419
chr4	68038898	68039608
chr4	72934073	72948239
chr4	73234360	73235080
chr4	73250566	73250788
chr4	73343954	73353573
chr4	75448466	75455855
chr4	80295558	80301716
chr4	844256	846876
chr4	845079	845385
chr4	84653407	84659039
chr4	9565858	9567271
chr4	9637640	9638489
chr4	9641070	9641431
chr4	9687543	9688030
chr5	10213490	10224061
chr5	1031595	1038722
chr5	10315971	10322598
chr5	1037854	1038024
chr5	104196313	104197092
chr5	104405310	104440133
chr5	104697858	104707721
chr5	108718740	108723972
chr5	116298246	116303683
chr5	116298246	116318300
chr5	116303529	116318300
chr5	116317516	116318300
chr5	116366385	116366904
chr5	123618017	123653221
chr5	127956803	127966928
chr5	12852739	12867888
chr5	131395562	131408207
chr5	131395562	131423631
chr5	131401480	131401776
chr5	131415070	131417177
chr5	131415070	131417615
chr5	131415070	131423631
chr5	132869215	132869717
chr5	137757866	137759020
chr5	141217689	141222023
chr5	142529994	142531132
chr5	145310575	145381718
chr5	145431457	145440159
chr5	145431457	145441072

chr5	145431457	145489738
chr5	145438071	145440159
chr5	145438071	145473315
chr5	145439990	145489738
chr5	145439990	145490324
chr5	145473199	145671554
chr5	145586380	145671554
chr5	145587324	145591128
chr5	145587324	145670827
chr5	145587324	145704295
chr5	145665253	145704295
chr5	145703974	145704295
chr5	148662606	148677235
chr5	150493474	150493800
chr5	150494087	150494858
chr5	150581883	150592358
chr5	151924933	151947795
chr5	151927173	151947795
chr5	151946638	151947795
chr5	151946638	151951036
chr5	171845505	171847749
chr5	172776482	172777671
chr5	177951716	177957662
chr5	178091088	178093389
chr5	178885613	178887787
chr5	179538973	179540832
chr5	179669084	179669391
chr5	179677042	179677858
chr5	179932940	179934259
chr5	181097606	181100673
chr5	181099848	181100673
chr5	25177590	25180826
chr5	25180728	25191033
chr5	25542353	25542790
chr5	25567671	25576170
chr5	25647011	25674652
chr5	27276140	27281091
chr5	33023074	33027826
chr5	34157534	34182867
chr5	34163117	34182867
chr5	34169026	34182867
chr5	34175444	34179097
chr5	34175444	34182867
chr5	34175821	34182867
chr5	34179012	34182867

chr5	34182679	34182867
chr5	37287979	37288845
chr5	43110830	43111091
chr5	4481691	4530076
chr5	4489481	4525809
chr5	53783966	53785144
chr5	66085446	66093732
chr5	66191155	66203030
chr5	66202824	66204326
chr5	66202824	66208992
chr5	6761219	6764872
chr5	6775558	6777509
chr5	6775558	6791981
chr5	70042366	70044143
chr5	73358843	73406818
chr5	73406444	73406818
chr5	7924232	7928686
chr5	79347816	79349750
chr5	83817769	83818893
chr5	85805306	85821466
chr5	85821331	85827796
chr5	85863807	85907736
chr5	85872428	85888067
chr5	90455714	90455985
chr5	93107575	93112679
chr5	97220955	97229457
chr5	97427235	97439618
chr5	98419359	98433380
chr5	98809501	98841931
chr6	104648142	104688287
chr6	10537030	10537425
chr6	106175468	106175882
chr6	107027015	107027429
chr6	108202402	108202595
chr6	110915033	110915493
chr6	111269448	111270581
chr6	111654023	111655556
chr6	112880591	112937686
chr6	112900344	112937686
chr6	112912876	112921171
chr6	116633185	116634497
chr6	117662905	117664862
chr6	120978891	121010355
chr6	120978891	121016795
chr6	121004169	121016795

chr6	125395038	125444235
chr6	126111218	126174911
chr6	126111218	126177335
chr6	126111218	126204432
chr6	126112865	126115855
chr6	126112865	126174911
chr6	126174824	126177335
chr6	126521536	126571600
chr6	126522079	126637735
chr6	129535931	129540542
chr6	129556551	129558166
chr6	134875094	134879469
chr6	13783422	13784613
chr6	13783429	13784613
chr6	138645754	138675044
chr6	140651477	140664253
chr6	140651477	140664610
chr6	140804234	140816254
chr6	140804234	140840786
chr6	141581999	141656558
chr6	141704581	141705067
chr6	141704581	141708290
chr6	141752379	141910011
chr6	141772872	141850160
chr6	141772872	141910011
chr6	141798082	141910011
chr6	141824165	141900410
chr6	141826594	141831645
chr6	141827440	141910011
chr6	141829735	141910011
chr6	141900322	141910011
chr6	141900322	141927052
chr6	141900322	141927055
chr6	141900322	141981627
chr6	141900322	142006118
chr6	150978152	150978936
chr6	151246353	151268693
chr6	151361485	151363195
chr6	151362022	151363195
chr6	151639052	151656763
chr6	154858319	154860998
chr6	154860737	154860998
chr6	154860737	154863898
chr6	154890687	154941705
chr6	154944115	154961377

chr6	1558958	1562413
chr6	158245179	158245997
chr6	158262644	158282378
chr6	158975110	158975944
chr6	159594102	159595204
chr6	159679915	159680682
chr6	166972738	166980839
chr6	23016724	23049941
chr6	26317123	26317476
chr6	27535500	27539873
chr6	29927383	29928546
chr6	30723619	30723775
chr6	30723658	30724414
chr6	31204641	31205364
chr6	31536592	31539016
chr6	31645533	31646479
chr6	32978168	32979950
chr6	34274244	34275306
chr6	35257316	35258068
chr6	36111568	36116421
chr6	36158647	36158941
chr6	36158647	36160784
chr6	36158647	36172969
chr6	36158647	36196170
chr6	37801615	37804140
chr6	3971257	4011600
chr6	4011505	4018879
chr6	41933549	41933881
chr6	42812951	42813607
chr6	42941509	42943970
chr6	49234530	49245714
chr6	49244965	49245740
chr6	49244965	49250609
chr6	49316602	49331897
chr6	57326579	57327046
chr6	57481876	57488778
chr6	70589007	70589260
chr6	73209955	73213760
chr6	73272683	73272953
chr6	73871929	73872831
chr6	75593818	75600328
chr6	77088445	77151974
chr6	77151096	77228352
chr6	77151200	77151974
chr6	77151200	77212053

chr6	77151200	77214751
chr6	77211980	77273307
chr6	77243120	77273307
chr6	77259666	77273307
chr6	77260161	77273307
chr6	77271339	77273307
chr6	77271339	77291700
chr6	77271339	77316704
chr6	80350090	80353897
chr6	80564421	80665949
chr6	81491490	81495113
chr6	82057938	82059679
chr6	83730887	83769008
chr6	83982528	84024983
chr6	84556018	84567813
chr6	84556018	84602487
chr6	85837956	85840301
chr6	85945691	85950092
chr6	88581610	88582036
chr6	90486986	90498237
chr6	90489838	90498237
chr7	100140171	100140439
chr7	100695753	100696073
chr7	100832135	100832826
chr7	101300768	101301049
chr7	104973523	104978489
chr7	105007939	105008580
chr7	105908407	105910572
chr7	105911016	105912638
chr7	106605178	106610463
chr7	116482812	116484046
chr7	116655580	116664041
chr7	116940363	116947790
chr7	12751406	12758316
chr7	128533644	128580094
chr7	128539731	128540021
chr7	128562314	128592017
chr7	128574719	128580094
chr7	128577952	128580094
chr7	128577952	128639156
chr7	128579998	128592017
chr7	128579998	128609579
chr7	128579998	128615970
chr7	128591849	128609579
chr7	128591849	128615970

chr7	128591849	128617977
chr7	128625796	128626530
chr7	128629393	128631869
chr7	128629393	128638897
chr7	128631679	128638897
chr7	129258580	129338536
chr7	130866492	130868472
chr7	134100187	134101166
chr7	140295984	140297523
chr7	149256869	149257247
chr7	151740424	151772530
chr7	156986371	156986609
chr7	157391091	157391847
chr7	157425779	157430952
chr7	1686701	1688150
chr7	1687915	1688150
chr7	17135407	17150206
chr7	17256397	17286516
chr7	17718053	17723952
chr7	18975301	18976468
chr7	19020901	19056165
chr7	19055252	19056165
chr7	19055252	19060484
chr7	22889358	22891109
chr7	2324042	2324400
chr7	2346100	2346487
chr7	2347284	2347874
chr7	2721272	2722138
chr7	32099293	32111181
chr7	32722722	32726470
chr7	32751393	32753956
chr7	32812756	32817307
chr7	32812756	32823221
chr7	32812756	32838666
chr7	32833007	32834919
chr7	32833007	32838613
chr7	32833007	32838666
chr7	33635176	33642918
chr7	363647	364005
chr7	38010920	38013581
chr7	45797477	45805289
chr7	4644598	4644873
chr7	4644685	4675325
chr7	4797055	4797677
chr7	5006429	5006885

chr7	5434778	5438651
chr7	5615950	5620039
chr7	6081104	6085262
chr7	64722547	64734625
chr7	64937341	64937630
chr7	65088804	65098832
chr7	65091879	65098832
chr7	65313569	65317566
chr7	65313569	65338616
chr7	65336192	65336394
chr7	65347826	65350103
chr7	65403175	65403644
chr7	65799602	65802070
chr7	65827161	65846059
chr7	66155767	66156065
chr7	66156214	66156391
chr7	66156620	66156879
chr7	6634970	6636235
chr7	66400922	66402545
chr7	66405568	66491239
chr7	66407289	66430017
chr7	66407289	66439304
chr7	66423378	66423725
chr7	66423378	66423796
chr7	66427948	66430017
chr7	66427948	66439304
chr7	66439134	66485153
chr7	66450070	66467719
chr7	66450070	66485153
chr7	66476258	66490107
chr7	66479008	66488038
chr7	66479008	66490107
chr7	66491939	66492217
chr7	66518161	66519312
chr7	66528672	66529097
chr7	66533323	66534217
chr7	66534432	66537986
chr7	66535305	66551349
chr7	66540484	66540742
chr7	66542612	66543379
chr7	66543567	66544590
chr7	66544834	66546136
chr7	66681005	66681505
chr7	66684351	66724454
chr7	66696341	66696483

chr7	66739584	66740075
chr7	66742130	66743522
chr7	66742720	66743196
chr7	66742726	66743178
chr7	66846360	66900897
chr7	66848488	66880877
chr7	66855671	66880877
chr7	66880286	66880826
chr7	66880286	66886455
chr7	66882892	66886455
chr7	66882892	66890411
chr7	66889134	66889521
chr7	66890250	66894018
chr7	66890250	66898294
chr7	66898178	66900897
chr7	6708300	6709039
chr7	67243494	67279526
chr7	6731172	6731562
chr7	6731207	6731528
chr7	67413632	67435727
chr7	6751866	6752780
chr7	6794202	6794535
chr7	69040490	69040699
chr7	722753	724864
chr7	74255596	74255853
chr7	74875952	74876373
chr7	84793668	84889860
chr7	84793809	84889860
chr7	85153607	85224441
chr7	85191646	85193238
chr7	88196544	88197239
chr7	91626135	91626656
chr7	93786566	93797279
chr7	94061455	94061864
chr7	94061455	94064477
chr7	96665941	96673859
chr7	96672721	96673859
chr7	9688501	9719071
chr7	9708000	9716909
chr7	9708000	9719071
chr7	97396868	97400188
chr7	978011	979061
chr8	103246293	103246684
chr8	116637954	116641259
chr8	117580726	117678348

chr8	117797934	117799315
chr8	12014830	12015529
chr8	123003992	123004504
chr8	123452562	123453459
chr8	127798211	127804635
chr8	127806368	127807862
chr8	127842514	127843102
chr8	127853688	127856436
chr8	127890211	127890493
chr8	128152676	128155419
chr8	129941568	129942077
chr8	129957132	129959543
chr8	133408173	133408387
chr8	134812609	134813169
chr8	141250923	141254211
chr8	141296205	141300145
chr8	142455078	142455231
chr8	143632289	143634770
chr8	143638645	143638987
chr8	143639180	143640655
chr8	143640578	143641432
chr8	143640848	143644115
chr8	144135635	144136000
chr8	16495792	16497989
chr8	16495792	16514838
chr8	36458581	36507411
chr8	38101049	38101406
chr8	48424352	48429056
chr8	54023228	54023897
chr8	55826706	55827423
chr8	55827138	55827423
chr8	56050156	56050949
chr8	61628999	61629368
chr8	63558679	63560116
chr8	66616187	66617276
chr8	74407087	74407621
chr8	77006817	77007102
chr8	77560573	77582405
chr8	77578034	77609934
chr8	77582169	77587459
chr8	77582169	77639385
chr8	78933742	78943071
chr8	81549711	81596464
chr8	81593228	81613828
chr8	81650820	81652327

chr8	8922288	8922750
chr8	89587890	89596481
chr8	89587909	89596512
chr8	90554350	90554666
chr8	93955054	93955893
chr8	97569024	97601212
chr9	105950113	105977778
chr9	110194874	110198574
chr9	111946477	111946969
chr9	112013211	112014047
chr9	114318193	114319492
chr9	120126446	120132905
chr9	120359196	120363376
chr9	121057893	121067564
chr9	125263550	125273649
chr9	125264949	125279325
chr9	12610570	12631607
chr9	12610904	12631607
chr9	127901530	127902268
chr9	129208868	129209236
chr9	130815115	130816266
chr9	131538592	131538921
chr9	135908651	135909486
chr9	21324075	21330913
chr9	22269012	22284962
chr9	22270998	22284962
chr9	22270998	22301707
chr9	22301599	22303231
chr9	22301599	22308883
chr9	22466115	22468710
chr9	22466341	22526934
chr9	23030864	23076101
chr9	25527947	25622103
chr9	2667150	2668061
chr9	2777872	2783594
chr9	31942990	31973112
chr9	32020881	32021092
chr9	32020881	32032270
chr9	32112169	32121665
chr9	32140008	32157552
chr9	32946387	32947618
chr9	34680905	34682982
chr9	35594874	35597388
chr9	35772162	35786775
chr9	35780266	35782174

chr9	37407284	37409356
chr9	37873238	37873801
chr9	37874947	37885334
chr9	37886217	37887520
chr9	41736441	41736723
chr9	42856951	42864465
chr9	42861683	42877817
chr9	42978850	42986862
chr9	5573511	5573935
chr9	61917467	61920435
chr9	78275430	78275747
chr9	78348315	78450197
chr9	78420279	78422192
chr9	78420279	78422263
chr9	78420279	78423212
chr9	78598224	78624433
chr9	83574088	83575055
chr9	83574909	83586368
chr9	91717306	91722580
chr9	91718638	91719168
chr9	93435500	93441373
chr9	93439402	93441373
chr9	96146620	96147084
chr9	96719508	96721130
chr9	96895895	96898127
chr9	96895895	96903108
chr9	96913237	96913633
chr9	97122170	97151237
chr9	97135545	97139954
chr9	97138642	97139954
chr9	97138781	97139954
chr9	97139852	97144740
chr9	97139852	97144760
chr9	97139852	97146151
chr9	97139852	97148100
chr9	97139852	97148191
chr9	97139852	97151237
chr9	97139852	97161966
chr9	97143857	97144740
chr9	97178263	97191798
chr9	97185441	97194840
chr9	97185441	97196594
chr9	97188464	97200565
chr9	97188464	97211487
chr9	98018288	98026451

chrM	13332	13587
chrM	8657	9189
Un_GL00019	18321	19683
Un_KI27074	1325	34982
chrX	100949107	100962973
chrX	10252888	10287891
chrX	102897573	102905737
chrX	102897573	102926537
chrX	103428627	103450660
chrX	103428627	103461354
chrX	103438261	103438892
chrX	103449273	103450660
chrX	103449273	103486459
chrX	103464128	103466155
chrX	103612238	103612987
chrX	107029126	107030315
chrX	10899315	11056036
chrX	10924729	10925298
chrX	11038602	11110981
chrX	11055935	11110981
chrX	115566067	115568006
chrX	121078057	121093477
chrX	121090398	121093477
chrX	121090398	121164157
chrX	121090398	121185843
chrX	121151610	121185843
chrX	121163288	121185843
chrX	121164058	121185843
chrX	121290859	121291842
chrX	121529566	121560542
chrX	121540922	121646582
chrX	121646461	121736294
chrX	121659247	121761171
chrX	121736190	121761171
chrX	121736190	121867909
chrX	121736190	121906789
chrX	121863502	121906789
chrX	126137960	126139299
chrX	126243345	126250294
chrX	126243345	126287274
chrX	129116609	129122193
chrX	129116609	129169451
chrX	129319081	129326008
chrX	129993458	129994022
chrX	131332206	131364926

chrX	131332206	131369052
chrX	131344059	131364926
chrX	131344812	131364926
chrX	131364748	131369052
chrX	131364748	131399704
chrX	131389016	131399704
chrX	131491212	131495163
chrX	131538342	131540937
chrX	134444877	134453899
chrX	135008542	135019149
chrX	135034668	135038178
chrX	135034668	135049428
chrX	135942878	135947139
chrX	135942878	135960073
chrX	135946564	135947139
chrX	13666316	13680598
chrX	136820680	136847899
chrX	136820680	136848582
chrX	136847804	136848582
chrX	136857061	136857499
chrX	1383565	1384425
chrX	1383600	1384388
chrX	139842395	139877277
chrX	141715007	141721898
chrX	141715007	141737585
chrX	144233227	144297079
chrX	144233227	144341556
chrX	144341463	144390125
chrX	144706102	144715226
chrX	152451699	152458262
chrX	152457249	152458262
chrX	152457249	152459530
chrX	152457249	152487023
chrX	152468181	152487023
chrX	152554376	152568217
chrX	152554376	152574980
chrX	153438907	153441548
chrX	154395361	154396024
chrX	155139218	155172714
chrX	155168139	155175179
chrX	155399000	155419562
chrX	155415574	155419562
chrX	16462842	16463063
chrX	1726066	1726488
chrX	1732512	1734211

chrX	1759980	1760310
chrX	2211488	2212828
chrX	27102783	27186657
chrX	27158904	27186657
chrX	27162420	27186657
chrX	27301038	27336493
chrX	27336409	27376091
chrX	45769785	45784155
chrX	487169	487610
chrX	49265842	49266298
chrX	510260	510945
chrX	54636234	54636427
chrX	55985998	56014961
chrX	56002824	56014961
chrX	57087613	57095538
chrX	57091049	57095538
chrX	57842245	57850693
chrX	63259667	63315853
chrX	63259667	63343289
chrX	63307505	63345571
chrX	63313751	63345571
chrX	63314996	63345571
chrX	63911327	63934393
chrX	65051461	65113813
chrX	65060697	65075912
chrX	65075473	65075912
chrX	65075473	65107023
chrX	65075473	65113813
chrX	65075605	65107023
chrX	65075605	65113813
chrX	65106918	65113813
chrX	65113663	65118284
chrX	65175160	65175753
chrX	65366637	65368998
chrX	65366637	65398704
chrX	65366637	65446000
chrX	67761872	67791892
chrX	76147252	76148456
chrX	76224487	76242405
chrX	77896308	77896804
chrX	81324575	81328708
chrX	91562992	91661744
chrX	91616342	91638418
chrX	91616342	91662446
chrX	97626153	97639812

chrX	97635274	97639812
chrX	97637436	97639812
**IncompleteExon (289)**		
chr1	109252789	109252982
chr1	110341550	110341832
chr1	150575338	150575695
chr1	154156515	154157213
chr1	155562555	155562821
chr1	156743123	156743420
chr1	156743256	156743444
chr1	15931188	15931413
chr1	162522517	162522962
chr1	19216778	19217489
chr1	19259017	19260012
chr1	200849332	200849701
chr1	202962119	202962382
chr1	204552180	204552423
chr1	205774938	205775164
chr1	240092041	240092400
chr1	26696426	26696950
chr1	28497464	28499171
chr1	30869737	30870042
chr1	43972427	43972744
chr1	43972775	43973346
chr1	45509624	45509866
chr1	58782149	58782786
chr1	6222395	6223195
chr1	63659765	63660161
chr1	6522806	6524849
chr1	9267030	9268037
chr10	101579445	101579615
chr10	102147082	102147359
chr10	104025835	104026576
chr10	17229460	17229850
chr10	79354333	79354659
chr10	91908226	91908693
chr11	2403440	2403617
chr11	61786740	61786908
chr11	61796713	61797143
chr11	62129957	62130312
chr11	64371521	64371829
chr11	65320787	65321131
chr11	65424450	65444063
chr11	65434478	65435145
chr11	65434478	65437657

chr11	65434478	65439001
chr11	65434478	65444063
chr11	65435052	65436666
chr11	65435052	65437657
chr11	65435052	65444059
chr11	65435052	65444063
chr11	65435066	65444059
chr11	65435066	65444063
chr11	65435343	65444063
chr11	65436522	65444059
chr11	65436537	65444059
chr11	65436537	65444063
chr11	65499911	65500426
chr11	65500911	65505019
chr11	65502289	65504595
chr11	65502320	65504206
chr11	65502320	65504595
chr11	65502320	65505019
chr11	65503116	65504595
chr11	65503116	65505019
chr11	65503120	65504595
chr11	65503120	65504744
chr11	65503728	65503931
chr11	65503728	65504358
chr11	65503728	65504595
chr11	65503728	65505019
chr11	65503853	65504595
chr11	65503853	65505019
chr11	65504325	65505019
chr11	65504664	65505301
chr11	65504819	65505019
chr11	65505301	65505606
chr11	67285312	67285959
chr11	69651354	69651675
chr11	69651774	69652233
chr11	69654031	69654355
chr11	73662218	73662658
chr11	75406202	75406492
chr11	76383673	76384417
chr11	768729	769573
chr11	769036	769415
chr11	769087	769661
chr11	95128538	95128749
chr12	105020454	105022254
chr12	112506422	112507953

chr12	120698058	120699112
chr12	122143443	122143620
chr12	124911698	124912760
chr12	132722070	132722239
chr12	1492816	1493170
chr12	57096211	57096640
chr12	98548946	98549335
chr13	109782961	109783488
chr13	21374527	21374880
chr13	23330939	23331307
chr13	23333467	23334769
chr13	23340204	23340593
chr13	23354899	23355080
chr13	23355505	23355841
chr14	104946924	104948904
chr14	21460507	21460723
chr14	23058674	23059073
chr14	31093701	31094760
chr14	92059769	92059977
chr15	100915603	100916259
chr15	40220696	40221044
chr15	50557937	50558499
chr15	63264649	63264956
chr15	64156404	64156665
chr15	65888236	65888571
chr15	75933741	75934479
chr15	85580533	85580742
chr16	11835033	11835602
chr16	22287252	22288003
chr16	2763640	2763883
chr16	2973051	2973245
chr16	29807000	29807505
chr16	29810329	29810779
chr16	29810541	29810788
chr16	30580732	30580974
chr16	30979468	30979806
chr16	547606	548161
chr16	87330641	87330864
chr17	1344858	1345238
chr17	3033554	3033944
chr17	39256293	39257066
chr17	44801060	44801884
chr17	44805253	44805577
chr17	4968050	4968501
chr17	5025805	5027145

chr17	50750746	50751362
chr17	50964510	50964872
chr17	50964549	50964840
chr17	517939	518459
chr17	58001488	58005041
chr17	58003650	58005041
chr17	59203435	59203645
chr17	75502414	75502822
chr17	78171933	78172189
chr17	81510291	81510612
chr17	82614875	82615196
chr18	13762870	13763271
chr19	13154281	13154420
chr19	13772154	13773506
chr19	1555052	1556075
chr19	16101602	16102900
chr19	2429764	2430187
chr19	33380103	33380381
chr19	3464426	3464923
chr19	39538878	39539476
chr19	41303979	41304182
chr19	44903046	44903470
chr19	45550128	45552285
chr19	45553262	45553581
chr19	50045620	50045807
chr19	55674122	55674621
chr19	55674123	55674604
chr19	55674124	55674602
chr19	57455652	57456182
chr19	57861375	57862570
chr19	57861793	57862570
chr19	57863364	57863759
chr19	746873	747472
chr19	810767	811135
chr19	811666	812181
chr2	121765509	121765913
chr2	189854502	189855000
chr2	203303215	203303468
chr2	206307046	206308755
chr2	20684815	20686956
chr2	223755755	223756143
chr2	227686100	227686445
chr2	25739880	25740085
chr2	40428474	40430301
chr2	42350692	42351224

chr2	64552228	64552705
chr2	69461890	69462413
chr20	1165673	1166455
chr20	25297189	25297444
chr20	3047806	3047970
chr20	326749	327166
chr20	32848975	32849744
chr20	33705527	33706035
chr20	36046082	36046353
chr20	3784471	3784807
chr20	3784967	3785142
chr20	3867456	3869139
chr20	3867568	3869042
chr20	41123291	41123867
chr20	50889306	50889832
chr21	14961813	14962555
chr21	26836602	26836831
chr21	33550219	33551289
chr21	34102834	34103519
chr22	18088683	18089796
chr22	23894774	23895188
chr22	36488075	36488630
chr22	37568428	37568774
chr22	37568849	37569109
chr22	37805948	37806372
chr22	40927396	40929273
chr3	134356631	134356829
chr3	141945430	141946776
chr3	14944673	14944903
chr3	185486430	185489016
chr3	188882976	188883465
chr3	196470237	196470728
chr3	33152371	33152686
chr3	38142235	38142445
chr3	52075315	52075560
chr3	9429830	9429989
chr4	139388509	139389132
chr4	165077031	165077551
chr4	165077085	165077493
chr4	2938442	2938625
chr4	70689572	70690027
chr4	75645135	75645625
chr5	1037854	1038024
chr5	126625943	126626478
chr5	134900598	134900833

chr5	138466844	138467320
chr5	14615209	14615944
chr5	14706775	14709036
chr5	150400006	150400164
chr5	154814075	154815294
chr5	155014042	155014637
chr5	172768394	172768780
chr5	34831844	34832429
chr5	453689	453995
chr5	65668047	65668676
chr5	65668054	65668676
chr5	73447242	73448206
chr5	73447480	73448201
chr5	93584329	93584824
chr6	105098712	105099121
chr6	157206248	157206842
chr6	30723619	30723775
chr6	30723658	30724414
chr6	30743562	30743769
chr6	31632742	31632907
chr6	33304466	33304611
chr6	43615477	43615754
chr6	43617842	43618265
chr6	85494748	85495026
chr7	107201683	107202138
chr7	128685178	128685938
chr7	135927081	135927388
chr7	135929470	135929668
chr7	138917367	138917692
chr7	149283427	149283567
chr7	151064572	151064741
chr7	152646774	152648175
chr7	22946006	22946145
chr7	25119663	25121096
chr7	25120363	25120897
chr7	29923109	29923538
chr7	5524824	5525118
chr7	65954689	65955203
chr7	66153996	66154226
chr7	88196544	88197239
chr8	100258473	100258925
chr8	102412470	102412624
chr8	127738349	127738838
chr8	142344079	142344469
chr8	43021954	43022920

chr8	43023113	43023547
chr8	96334111	96334477
chr9	105520521	105520916
chr9	105520622	105520916
chr9	120857143	120857384
chr9	134033073	134033279
chr9	136114757	136115073
chr9	137063010	137063161
chr9	137063607	137063775
chr9	137063791	137064102
chr9	33676487	33676667
chrX	119538737	119539285
chrX	119538738	119539270
chrX	119539037	119539709
chrX	121048129	121048330
chrX	154011011	154011172
chrX	48560238	48560631
chrX	54561038	54562059
chrX	54562127	54562587
chrX	65740042	65740250
chrX	65740347	65740883
chrX	85091377	85091770
