# Supplementary material for: Accurate identification of circRNA landscape and complexity reveals their pivotal roles in human oligodendroglia differentiation
Source: Genome Biol. 2022 Feb 7;23:48. doi: 10.1186/s13059-022-02621-1 (PMC8819885; doi:10.1186/s13059-022-02621-1)
Supplement: Supplementary file 6 — Additional file 6. Review history. [file 13059_2022_2621_MOESM6_ESM.docx]

**Review History**

**First round of review**

**Reviewer 1**

**Are you able to assess all statistics in the manuscript, including the appropriateness of statistical tests used?**

No, I do not feel adequately qualified to assess the statistics

**Comments to author:**

The authors have developed a new comprehensive computational framework for accurate circRNA identification and qualification. They use this approach to study circRNA profile changes during human oligodendroglia differentiation and they provide a very nice set of thorough analyses that describe the "big, genome wide picture" while also highlighting very interesting example genes throughout the analyses. The manuscript was very easy to read and very well done.

(1) The circRNA acting as a microRNA sponge data presented by the authors are much more than typically seen in manuscripts, so I applaud them for that but I do have a few specific comments. It would be ideal if the authors knocked down circSPATA13 and showed that some miR-760 target genes were affected. This would directly test the proposed sponging model. When discussing circPEX6, the authors do not state how many miR-17-5p/106a-5p sites are present or the circRNA copy number. Please provide these data.

(2) I may have missed it in the manuscript, but the authors should provide the code for CARP so the tool can be used by the community.

(3) The authors have overall done a really nice job comparing the results of their approach to other published algorithms, which gives the reader confidence in the data analysis. That said, it would be really nice if the authors could add a validation experiment or two where they directly validate an interesting result using a non-high throughput sequencing approach (e.g. like the knockdown experiment proposed above).

Minor points:

(1) Background should state a downstream 5' splice donor is joined to an upstream 3' splice acceptor. The 5' and 3' terms are reversed in the current manuscript.

(2) Fig 1e: Please confirm in the main text that the authors mean that the circRNAs must be identified by ALL 4 algorithms. It is currently ambiguous if being detected by a single algorithm is sufficient to be included in the pseudo-reference set.

(3) Fig S4A: In the main text, "200 bp" should be "200 nt" as circRNAs are single stranded molecules.

(4) When discussing the use of SAILOR to find 71 significant A-I editing changes during HOG differentiation, please clarify the RNA-seq library that was examined. I assume it was a library that was not treated with RNase R.

**Reviewer 2**

**Are you able to assess all statistics in the manuscript, including the appropriateness of statistical tests used?**

No, I do not feel adequately qualified to assess the statistics

**Comments to author:**

In this manuscript entitled "Accurate identification of circRNA landscape and complexity reveals their pivotal roles in human oligodendroglia differentiation", the authors developed a new approach called CARP to identify and quantify circRNA, which was more sensitive for low abundant circRNAs and effective for full-length circRNAs with rare effects on circRNA quantification. Using this method, the authors identified hundreds of circRNA isoforms and circRNA clusters in human oligodendroglia cells during early differentiation. The manuscript presents some technical advance in terms of identifying low abundance circRNAs, but lacks biological insight in circRNA functions. It is also not obvious why they only focus on just one cell type in this study. In addition, a careful comparison between their method and previous methods is missing.

Major concerns

1. The authors showed that only a subset of circRNAs was shared by the outputs of the four published circRNA identification tools. They need to compare the outputs between this pseudo-reference alignment method and other published methods, and give a clear explanation on the advantages of using pseudo-reference alignment method to identify the circRNAs compared to other alignment tools.

2. The CARP method is useful to identify different circRNA isoforms. However there are at least two recent studies using long read sequencing for identifying full length circRNAs, and the authors should give a detailed analysis for the overlap of circRNA isoforms identified from CARP and the other studies using long read sequencing. Speccifically, what's the difference of expression levels of circRNA isoforms judged by CARP and long-read sequencing? Because the limitation of long-read sequencing, the estimation of expression levels of circRNA isoforms might be an advantage of CARP method.

3. They should give more information on the sequence features of the 204 circRNAs isoforms. Are the alternative exons in circRNAs novel unannotated exons (or annotated exons)? For annotated exons, are they annotated to alternative exons or constitutive exons? do they contain longer sequences between two back-spliced exons than other circRNAs without isoform?

4. In Additional file 1: Figure S3I, the authors stated: "Most exon-derived circRNAs bear multiple exons, while a few are from single exon and intron lariats in HOG cells". However, in Fig. 3I pie chart, there is no information about multiple exons and single exons. Additionally, it is not clear what is the meaning of "other" in Fig. S3I?

Minor comments:

1. Please add "a" in the legend of Figure 3a.

2. Please add the information in figure legends that what "expression" mean in Fig. 2c and Fig. 3d.

3. Please add the information in figure legend that how to calculate "inclusion difference" in Fig. 3a.

4. The corresponding figure of this sentence "The circCFAP299 full-length constructed by CARP, particularly the unique circRNA specific exon, was consistent with the long-read sequencing data (Fig. 3d, lower box) " should be "...(Fig. 3c, lower box)".

**Authors Response**

**Point-by-point responses to the reviewers’ comments:**

We would like to sincerely thank the editor for handling our manuscript GBIO-D-21-01261, and the reviewers for providing thoughtful and constructive critiques. We are excited to see the editor and reviewers found our manuscript “very interesting”, with our data being described as “very nice set of thorough analyses that describe the "big, genome wide picture" while also highlighting very interesting example genes throughout the analyses”. They also acknowledged the “technical advance” of developing a new approach and the manuscript was “very easy to read and very well done”. Per reviewer’s suggestions, we have collected new experimental data, updated and improved our analyses to fully address all the concerns/criticisms raised by the reviewers whenever possible, which significantly improved the revised manuscript.

Major changes we made include:

1. To expand on the biological function of circRNAs in human oligodendroglia (OL) per suggested by the reviewers and editor, we knocked down circSPATA13, a novel human OL-specific circRNA identified by CARP, in HOG cells using siRNA targeting the back splicing junction sequence without affecting linear SPATA13 mRNA. As predicted by the reviewer, several computational predicted and experimentally validated miR-760 targets were significantly downregulated upon circSPATA13 knockdown, strongly supporting the function of circSPATA13 in sponging miR-760 to regulate its activities in suppressing mRNA targets in human oligodendroglia. Importantly, circSPATA13 exon sequence is poorly conserved in mouse, suggesting a preferential function of circSPATA13 to modulate the conserved miR-760 pathway to advance human OL differentiation. Our new results revealed and validated the first circRNA-miRNA-mRNA axis that may enhance human OL differentiation, providing an example that supports the evolutionary advantage of circRNAs in human brain.

2. Per reviewer’s suggestion, we carefully compared the output between CARP with embedded pseudo-reference alignment approach and several other published junction reads-based approaches. CARP clearly outperforms these algorithms to achieve better sensitivity in circRNA detection and accurate circRNA quantification. In addition, CARP is designed for processing RNA-seq data from poly-A tails in vitro followed by RNase R treatment in Li+ buffer (A-tailing), the best experimental setting to-date for completely removing linear RNAs. CARP is also adjustable to achieve the desired FDR in different datasets. Our study in combination with A-tailing and CARP, identified accurate and reliable circRNA landscapes from Illumina sequencing data.

3. Per reviewer’s advice, we have conducted detailed analyses for the overlap of circRNA isoforms identified by CARP and a recently long reads sequencing-based study in human brain (Xin et al, 2021, isoCirc). Despite the key circRNA landscape differences between human OLs and bulk brain tissue, we found highly consistent circRNA full-length internal structures identified by CARP and isoCirc using the Oxford Nanopore Technologies (ONT) platform. Among 12,242 common circRNAs detected by both CARP in HOG cells and isoCirc in human brains, 10,472 (85.54%) displayed exact internal structures, suggesting high sensitivity and accuracy for full-length circRNA construction by CARP. Importantly, our A-tailing RNA-seq libraries provide substantial higher sequencing depth than long-reads platform, which offer more accurate quantification especially for circRNAs expressed at low abundance.

A point-by-point response for each reviewer is provided below

Reviewer #1: The authors have developed a new comprehensive computational framework for accurate circRNA identification and qualification. They use this approach to study circRNA profile changes during human oligodendroglia differentiation and they provide a very nice set of thorough analyses that describe the "big, genome wide picture" while also highlighting very interesting example genes throughout the analyses. The manuscript was very easy to read and very well done.

(1) The circRNA acting as a microRNA sponge data presented by the authors are much more than typically seen in manuscripts, so I applaud them for that, but I do have a few specific comments. It would be ideal if the authors knocked down circSPATA13 and showed that some miR-760 target genes were affected. This would directly test the proposed sponging model. When discussing circPEX6, the authors do not state how many miR-17-5p/106a-5p sites are present or the circRNA copy number. Please provide these data.

*We thank the reviewer for bringing up this important point. In our initial submission, we found that predicted targets, especially top targets (top 10% mRNA targets based on ranking of weighted context++ score calculated by TargetScan), of miR-760 were significantly downregulated during HOG differentiation (t-test, p-value = 4.35×10-10) (Fig. 5a and 5b), suggesting miR-760-mediated mRNA decay (Fig. 5c). We also found several published miR-760 targets that function as repressor of OL differentiation and show significant down regulation upon HOG differentiation based on our RNA-seq data. Interestingly, miR-760 levels did not change significantly during HOG differentiation (Fig. 5d), raising a question whether a circRNA may sponge and regulate miR-760 activity without altering miR-760 expression. Using A-tailing and CARP, we identified circSPATA13 (hsa_circ_0004865), which harbors seven predicted miR-760 binding sites, is abundantly expressed in HOG (2k copies per cell) and markedly downregulated upon HOG cell differentiation. This led us to hypothesize that circSPATA13 sponges miR-760 and down-regulation of circSPATA13 during HOG differentiation releases miR-760 to suppress its mRNA targets. To test this hypothesis, we followed the reviewer’s suggestion and conducted circSPATA13 knockdown (KD) in HOG cells using an siRNA that specifically targets the back splicing junction sequence. The level of circSPATA13 was significantly reduced, without affecting the linear SPATA13 mRNA (new Fig. 5g). Importantly, several previously reported and predicted miR-760 targets, including MYC, HIST1H2BM, HIST1H3D and HIST3H2A, were significantly reduced upon circSPATA13 KD (new Fig. 5g; Additional file 1: Figure S7E). These new data provided critical biological insights and validated the functional connection of circSPATA13-miR-760-mRNA pathway in human OL differentiation. We have included these data in the aforementioned figures and corresponding text in the revised manuscript. Regarding circPEX6, we predicted it harbors one miR-17-5p/106a-5p binding site. By comparing the reads per million (RPM) of circSPATA13 and circPEX6 in HOG cell from our CARP analysis, we estimated 396 copies of circPEX6 in each HOG cell. Such abundance of circPEX6 suggests that this circRNA is sufficient to act as a miRNA sponge in HOG cells. This information is now included in the Result section of the revised manuscript.*

(2) I may have missed it in the manuscript, but the authors should provide the code for CARP so the tool can be used by the community.

*We appreciate the reviewer’s comment and do plan to make CARP a public tool for the community after our manuscript is published. At this point, a project called “CARP” in GitHub has been created and could be accessed from this url: https://github.com/YaoLabEmory/CARP. The source code for CARP is readily available upon request, and the full version of CARP will be released upon publication of our manuscript. The information is provided in code availability section in revised manuscript.*

(3) The authors have overall done a really nice job comparing the results of their approach to other published algorithms, which gives the reader confidence in the data analysis. That said, it would be really nice if the authors could add a validation experiment or two where they directly validate an interesting result using a non-high throughput sequencing approach (e.g., like the knockdown experiment proposed above).

*We appreciate the reviewer for bringing this to our attention. As discussed above, we have carefully assessed expression of several miR-760 targets upon circSPATA13 depletion, and our data strongly support a regulatory function of circSPATA13 on the mRNA targets of miR-760.*

Minor points:

(1) Background should state a downstream 5' splice donor is joined to an upstream 3' splice acceptor. The 5' and 3' terms are reversed in the current manuscript.

*We apologize for the confusion and have corrected this in the revised manuscript.*

(2) Fig 1e: Please confirm in the main text that the authors mean that the circRNAs must be identified by ALL 4 algorithms. It is currently ambiguous if being detected by a single algorithm is sufficient to be included in the pseudo-reference set.

*We thank the reviewer for pointing this out and apologize for the confusion. To achieve the maximum index coverage of any possible OL circRNAs, we included all the circRNAs that can be detected by any one of the four published algorithms for pseudo-reference construction to include as much circRNAs as we could. To minimize the false discovery rate, CARP set a series of stringent cutoff to remove potential genomic and transcriptomic reads from linear RNAs, as well as RNase R-sensitive circRNAs, as shown in Fig. 1e, f. Our approach rendered a robust and genuine circRNA identification in HOG cells (CARP-38,143 circRNA identified, in comparison to CIRCexplorer2 (14,159), CIRIquant (35,338), findcirc (23,294) and MapSplice (11,366) at FDR<0.05).*

(3) Fig S4A: In the main text, "200 bp" should be "200 nt" as circRNAs are single stranded molecules. We thank the reviewer for raising this point.

*This has been corrected in the revised manuscript.*

(4) When discussing the use of SAILOR to find 71 significant A-I editing changes during HOG differentiation, please clarify the RNA-seq library that was examined. I assume it was a library that was not treated with RNase R.

*We apologize for not being clear on this point. Since linear RNAs in A-tailing libraries were efficiently degraded, we indeed used the untreated control libraries to detect genome wide A-to-I editing events by SAILOR, as predicted by the reviewer. This has been clarified in Result section of the revised manuscript.*

Reviewer #2: In this manuscript entitled "Accurate identification of circRNA landscape and complexity reveals their pivotal roles in human oligodendroglia differentiation", the authors developed a new approach called CARP to identify and quantify circRNA, which was more sensitive for low abundant circRNAs and effective for full-length circRNAs with rare effects on circRNA quantification. Using this method, the authors identified hundreds of circRNA isoforms and circRNA clusters in human oligodendroglia cells during early differentiation. The manuscript presents some technical advance in terms of identifying low abundance circRNAs, but lacks biological insight in circRNA functions. It is also not obvious why they only focus on just one cell type in this study. In addition, a careful comparison between their method and previous methods is missing.

*We thank the reviewer for pointing out these important points. The goal for us to create CARP, a comprehensive computational algorithm optimized for A-tailing RNase R approach, in this research article is to explore the previously unknown roles of circRNAs in human OL development. In the introduction of the original submission, we pointed out the fact that a comprehensive and precise understanding of the circRNA landscape and its downstream biological pathways in human OL development is lacking. Given the essential roles of OLs in brain function and the numerous brain diseases involving OL malfunction, identification of genome-wide circRNA landscape in human OLs and its dynamic regulation during OL development may reveal novel insights of OL biology in normal and diseased brains. Due to the difficulty in obtaining pure human OLs in culture, we employed the human oligodendroglioma cell line HOG, which can be induced for differentiation in culture as a model of human OL progenitor cells and obtained the first genome-wide circRNA landscape specific in human OLs. The neuroblastoma cell line BE(2)-M17 was used in parallel experiments for cell-specificity comparison of differentiation regulated circRNA landscapes. Regarding biological insights in circRNA function, we now provide mechanistic evidence of circRNA functions in human OL development through modulating critical miRNA-mRNA pathways. In our original submission, we identified potential interaction between circSPATA13 and miR-760. As suggested by Reviewer 1, we knocked down (KD) circSPATA13 in HOG cells using siRNA targeting the back splicing junction sequence without affecting the linear SPATA13 mRNA (Fig. 5g). We demonstrated that several previously reported miR-760 targets, including MYC, HIST1H2BM, HIST1H3D and HIST3H2A, were significantly reduced upon KD of circSPATA13, strongly support the roles of circSPATA13 in regulating the miR-760 pathway. Importantly, MYC suppresses OL progenitor cell differentiation by inducing a global increase of repressive histone methylation and premature peripheral nuclear chromatin compaction (Magri et al, 2014; Neumann et al, 2021). Thus, the decline of circSPATA13 upon OL differentiation releases the sponged miR-760, which in turn suppresses the downstream targets represented by MYC to promote OL differentiation. It is important to point out that circSPATA13 exon sequence is poorly conserved in mouse (blastn identity = 66%), suggesting a preferential function of circSPATA13 in human OL differentiation. Our new results revealed the first circRNA-miRNA-mRNA axis that may enhance human OL differentiation, providing an example that supports the evolutionary advantage of circRNAs in the human brain. In addition to the circSPATA13-miR-760-MYC pathway, we also identified the circPEX6-miR-17-5p/106- 5p-STAT3 pathway, in which up-regulation of circPEX6 during human OL differentiation sequesters miR17-5p/106-5p that suppresses STAT3, a known player that enhances OL differentiation (Steelman et al, 2016). The reciprocal regulation of the circSPATA13-miR-760-MYC pathway and the circPEX6-miR-17- 5p/106-5p-STAT3 pathway during early differentiation of human OLs (Fig. 5h) provided the first example that developmental regulation of circRNAs may facilitate functional integration of differentiation repressors and enhancers to advance neural development. Taken together, our studies not only identified novel circRNA-miRNA-mRNA pathways but also revealed reciprocal regulation and potential functional co-operation of distinct circRNA-miRNA-mRNA pathways to advance human OL differentiation, which provide novel insights of circRNA biology. The above revisions are included in the Result and Discussion sections, respectively. In response to the criticism “a careful comparison between their method and previous methods is missing”, we compared our method with previously published methods, and discussed in detail point to point as indicated by Reviewer 2.*

Major concerns 1. The authors showed that only a subset of circRNAs was shared by the outputs of the four published circRNA identification tools. They need to compare the outputs between this pseudo-reference alignment method and other published methods, and give a clear explanation on the advantages of using pseudoreference alignment method to identify the circRNAs compared to other alignment tools.

*We thank the reviewer for this constructive comment. To minimize the false discovery rate, CARP set a series of stringent cutoff to remove potential genomic and transcriptomic reads from linear RNAs, as well as RNase R-sensitive false positive, as shown in Fig. 1e, f. Our approach rendered a robust and genuine circRNA identification in HOG cells (CARP-38,143 circRNA identified, in comparison to CIRCexplorer2 (14,159), CIRIquant (35,338), findcirc (23,294) and MapSplice (11,366) at FDR<0.05). As for circRNA quantification, we found circRNA quantified by pseudo reference is highly correlated with junction reads-based algorithms, such as CIRCexplorer2 and CIRIquant (Fig. S3F, G). The accurate quantification of circRNAs by CARP was further validated by qPCR (Fig. 2e). Nonetheless, since quantification of circRNA is highly dependent on each individual algorithm, often resulting in difficulties to cross-compare their output results. This issue, however, can be overcome by pseudo-reference-based approach such as CARP by universally merge potential circRNA junctions identified by different approaches. Therefore, CARP offers a streamlined computational approach that maximizes the sensitivity of circRNA detection and standardize the quantified output for better cross-reference comparison. The advantages of using pseudo-reference alignment have also been confirmed by recent publications (Zhang et al, 2020; Rabin et al, 2021). Although PacBio and Oxford Nanopore Technologies (ONT) platforms allow for long-reads sequencing for easy reconstruction of full-length circRNAs, they often suffer from relatively lower coverage, high cost and more sophisticated analysis with higher false positives. We now have provided additional analyses and comparison of full-length circRNA construction and alternative isoforms between our A-tailing data analyzed by CARP and circRNA profiling obtained by ONT below. Among 12,242 common circRNAs identified by CARP in HOG cell and isoCirc in human brain, 10,472 (85.54%) displayed identical internal structure, suggesting accurate circRNA full-length information identified by CARP. CARP is also able to detect circRNAs formed by novel unannotated exon which could also be validated by long reads sequencing data (Fig. 3c). These new data strongly support the numerous advantages of CARP that cannot be fully achieved by any types of current junction-reads based algorithms, and long-reads method given their specific limitations. It is important to note that, unlike any other current pseudo reference-based algorithms, CARP provides the users a full-range of customized and flexible cutoff for number of bases that match to back-splice junction flanking sites for pseudo-reference alignment to achieve the best balance between FDR and sensitivity from different dataset for circRNA identification (Fig. S3b). CARP is also designed to perform reads count comparisons between untreated control libraries and A-tailing RNase R-treated libraries to further exclude false positive. Thus, our data from A-tailing coupled with CARP in this manuscript provided the most accurate circRNA identification and quantification in OLs to date. CARP can be also used to analyze regular RNase R-treated RNA-seq data. Optimizing the best FDR from these datasets by adjusting the number of base pairs in each RNA-seq reads matching to junction sites provided by CARP could achieve the best balance between sensitivity and accuracy, making CARP a broader application to the society. Those information are included in the Discussion section in revised manuscript.*

2. The CARP method is useful to identify different circRNA isoforms. However, there are at least two recent studies using long read sequencing for identifying full length circRNAs, and the authors should give a detailed analysis for the overlap of circRNA isoforms identified from CARP and the other studies using long read sequencing. Specifically, what's the difference of expression levels of circRNA isoforms judged by CARP and long-read sequencing? Because the limitation of long-read sequencing, the estimation of expression levels of circRNA isoforms might be an advantage of CARP method.

*We appreciate the reviewer’s suggestions and thought this is an excellent point to discuss. Per reviewer’s request, we have compared and discussed the advantage of CARP over long-reads sequencing above. As indicated by the reviewer, two recent studies using long reads sequencing for identifying full length circRNAs (Zhang et al and Xin et al). Zhang et al 2021 used ONT data to detect circRNA full-length in mouse brains, which is not comparable with our datasets. Therefore, we focused on comparing our genomewide circRNA landscape in HOG cells with human brain ONT data identified by isoCirc (Xin et al, 2021). Despite cell-type specificity, we were still able to identify 12,242 common circRNAs between the two datasets from 44,705 circRNAs identified in HOG cells by CARP. Importantly, 10,472 (85.54%) of 12,242 circRNAs displayed identical internal structure between the two datasets, suggesting a high level of consistency. The circRNA isoform switch showed strong cell type- and differentiation-specificity. From 91 isoform switch events identified by CARP in HOG differentiation, we found 41 of them also have multiisoforms in human brains identified by ONT. As for circRNA quantification analysis, we compared circRNA reads count in CARP and ONT-isoCirc and found substantial deeper coverage in our libraries (Figure below, P-value < 2.2e-16). The higher reads coverage generated from Illumina platform in our study could result in more sensitivity in circRNA identification and more accuracy in circRNA quantification especially low expressing circRNAs.*

*Furthermore, as discussed above, CARP has much broader applications to vast majority of exiting RNAseq datasets compared to current long-reads approaches. Those statement are included in the Discussion section in the revised manuscript.*

3. They should give more information on the sequence features of the 204 circRNAs isoforms. Are the alternative exons in circRNAs novel unannotated exons (or annotated exons)? For annotated exons, are they annotated to alternative exons or constitutive exons? do they contain longer sequences between two back-spliced exons than other circRNAs without isoform?

*We appreciate the reviewer bringing this to our attention. To annotate sequence features of the 204 circRNAs isoforms, we compared those alternative circular exons detected in HOG differentiation with gene annotation downloaded from UCSC Table Browser and cassette exon information downloaded from HEXEvent. We found 17.89% of alternative circular exons in HOG differentiation are previously annotated cassette exons, 45.83% are previously annotated constitutive exons while 36.27% are novo unannotated exon. Those information are described in the Result section of the revised manuscript. As for length distribution of circRNAs, we didn’t find significant difference in the length between circRNAs with isoform and those without isoforms as shown in the figure below (P-value= 0.159).*

4. In Additional file 1: Figure S3I, the authors stated: "Most exon-derived circRNAs bear multiple exons, while a few are from single exon and intron lariats in HOG cells". However, in Fig. 3I pie chart, there is no information about multiple exons and single exons. Additionally, it is not clear what is the meaning of "other" in Fig. S3I?

*We apologize for the confusion. In our initialsubmission, the circRNAs were classified as “Exon”, “Intron”, “Exon/Intron” and “Other”. For the 28,576 circRNAs that derived from exon sequence in HOG, 27,501 (96.24%) were from multi-exon while 1075 (3.76%) were from single exon. The “Other” include circRNAs that comes from intergenic region and circRNA comprised by incomplete annotated exon. In order to avoid this confusion, we’ve updated the Fig. S3I and attached the list of circRNAs for each class as Additional file 4.*

Minor comments:

1. Please add "a" in the legend of Figure 3a.

*We thank the reviewer for this point. The legend has been revised in the updated manuscript.*

2. Please add the information in figure legends that what "expression" mean in Fig. 2c and Fig. 3d.

*We apologize for the confusion. The “expression” in Fig. 2c and Fig. 3d refer to counts per million (CPM) for each circRNA according to RNA-seq data. We have revised the label and figure legend per reviewer’s suggestion in Fig. 2c, 3d and figure legend.*

3. Please add the information in figure legend that how to calculate "inclusion difference" in Fig. 3a

*We apologize for the confusion. Since most of linear RNA were degraded in A-tailing RNase R treated RNA-seq library, CARP could take advantage of reads mapped to the circRNA body to reconstruct their full length. CircRNAs carry different sequence composition but share the same BSJ sites were defined as circRNA isoforms and percentage of their expression represent the “inclusion level” of alternative spliced exons. The “inclusion level” was calculated by reads count of isoform specific junction reads. By comparing HOG cells with and without differentiation, the “inclusion level” could be different for specific circRNA alternative spliced exons. The “inclusion difference” is the difference of “inclusion level” between conditions. This information has been added to figure legend per reviewer suggested. Detailed methods are described in Method section.*

4. The corresponding figure of this sentence "The circCFAP299 full-length constructed by CARP, particularly the unique circRNA specific exon, was consistent with the long-read sequencing data (Fig. 3d, lower box) " should be "(Fig. 3c, lower box)".

*We thank the reviewer for this point. This has been corrected in the revised manuscript*

**Second round of review**

**Reviewer 1**

The authors have satisfactorily addressed my concerns.

**Reviewer 2**

In the revised manuscript, the authors conducted additional experiments to validate the function of the circSPATA13 as sponges of miR-760, which provide some biological relevance of this work. They also conducted several analyses to compare their methods to previous procedures in identification and quantification of circRNA. While the additional analyses make the paper more clear, there are some new problems and the new experiments did not make the paper more convincing.

The biggest concern I have is the popular thoughts of circRNAs function as miRNA sponge. There have been some debates on how general this function is for circRNAs, and many recent reports in this function are unreliable. Therefore I admit that I tend to be a little critical on such experiments, especially after a recent retraction of paper on another circRNA (circ_0007031) as sponge of the miR-760 to control cancer growth (PMC7500843). It is good that authors of this manuscript measured the abundance of circSPATA13 and found that circSPATA13 are pretty abundant among all circRNAs, but they should compare the level to miR-760 to see if the change of circSPATA13 during OL differentiation are indeed within the dynamic range of meaningful regulation. Since miR-760 level is relatively low (Fig. 5a), the small reduction of circSPATA13 (fig 5e) may not be enough to affect miR-760. Many of the figures are presented in relative expression values of miRNA and circRNAs, it is impossible for me to make an evaluation on this. Also they need to look at the base-pairing of Myc-miR760, as this target may only supported by prediction.

In addition, the level of miR-760 target, Myc, only showed a small change (Fig. 5) upon circRNA KD. The author cited a recent paper in response letter, “(Myc) was recently shown in controlling OL progenitor cells proliferation and differentiation homeostasis”, however it is unclear if such a small decrease of Myc level is enough to affect “OL progenitor cells proliferation and differentiation”. For the circSPATA13, the authors also need to show that the change of miRNAs targets are consistent and have significant overlap between their two analyses (i.e. KD of circSPATA13 vs the OL differentiation).

The same set of problems also exist for circPEX6-miR17-5p/106a-5p, where the seed matches for circRNA-miRNA were not even shown. Similarly, the change of circPEX6 and PEX6 in OL differentiation should be examined before they propose a new model of regulatory switch. My concern is that there are many miRNAs being affected in OL differentiation and their targets should have consistent changes, it is not hard to find a few examples that sounds reasonable. However the validation of this regulation should be carefully done.

Fig. S3C，it is very curious and a little strange that the distribution of circRNA fold have several sharp spikes. One would expect this distribution should be relatively smooth like mRNAs, can the author give an explanation? Is this due to some cutoff setting or some bugs in their program?

The number is still confusing in some places. For example, line 24 in page 8, “A-tailing allowed the identification of an added 37,950 de novo circRNAs by CARP...”. The total number should be 37950+6846 circRNA in HOG cells here, which is different from the “38561 circRNA in HOG cells” in page 9. In addition, these two numbers are not consistent with what they said in the rebuttal letter “(CARP-38,143 circRNA identified, in comparison to CIRCexplorer2 (14,159), CIRIquant (35,338), findcirc (23,294) and MapSplice (11,366) at FDR<0.05). ”. Maybe they used different parameters or cutoffs (for unclear reason), but such conflict of numbers decreased the confidence of their data.

In addition, the additional number of circRNAs identified by adding A-tailing step is very significant, and they may need to use another method on the same datasets (with or without A-tailing) to confirms this ~5 fold increase is real. A related concern is that if this increasement also increased the false positive rate, which is a tricky situation due to the compromise between sensitivity vs. specificity, but the author should give some assessment/discussion.

Minor point, the writing should be polished a little more to be concise and clear. There are many places does not read well and are unnecessarily long. For example, line 32 in page 11, two long subsentences (both starting with which) used in the same long sentence. Line 19 of page 9, “The exons in various circRNAs ranged from 1 to over 20”, it should be “numbers of circRNA exons” because the exon itself do not range from 1. Minor places like this make it hard to read.

**Authors Response**

**Point-by-point responses to the reviewers’ comments:**

*We would like to sincerely thank the Editors for handling our manuscript GBIO-D-21-01261 and providing the Referee comments promptly. In particular, we highly appreciate that the Referees and Editors recognize the significance of our studies that combine the computational and experimental advancements and identify the very first accurate circRNA landscape dynamically regulated during human OL differentiation. In fact, Referee 1 clearly stated that “they use this approach to study circRNA profile changes during human oligodendroglia differentiation and they provide a very nice set of thorough analyses that describe the "big, genome wide picture" while also highlighting very interesting example genes throughout the analyses.” Referee 1 also noted “The circRNA acting as a microRNA sponge data presented by the authors are much more than typically seen in manuscripts, so I applaud them for that…”. Referee 2 primarily suggested to compare our tools and downstream results with published algorithms and long-reads sequencing dataset. During revision, we have thoroughly addressed all the concerns and questions raised in the first round of review by both reviewers. We are pleased and excited to see that Referee 1 found our response satisfactory, as we provided more evidence of the sponge model by circRNA and validated the key targets in our exemplar pathways which are the central focus instructed by the Editor. Although delayed in sending the report, Referee 2 found “the additional analyses make the paper clearer”. No further concerns were raised by Referee 2 regarding their initial set of comments.*

*However, in the second round of review, Referee 2 raised a completely new set of questions that were not in the initial review comments, and most of them were focused on specific circRNA, miRNA and mRNA species that we validated. We would like to draw your attention to the fact that these specific RNAs are only examples we validated that support the developmental regulation of the circRNA landscape in human oligodendroglia differentiation. The specific function and full-scale regulation of these individual genes, as questioned by Referee 2 in the second round of review, are beyond the main scope of this manuscript. Nonetheless, we included explanations and clarifications to address Referee 2’s new concerns. We also revised the text according to Referee 2’s suggestion. A point-by-point response is provided below:*

Referee 2 raised concerns regarding the small magnitude of changes of the miR-760 target.

*The power of miRNAs is that they have multiple mRNA targets to fine-tune the expression of a molecular landscape at post-transcription level. It is expected some of their targets might have subtle changes during precisely controlled biological events. In our OL differentiation model, we have separated top mRNA targets of miR-760 from the total targets (Fig. 5b) and found substantial and significant changes from the “top targets” group (Fig. 5b, blue line), less prominent from “normal targets” (Fig. 5b, orange line) comparing to random control genes (Fig. 5b, green line). Those changes were not at transcription level but rather post-transcriptional (Fig. 5c). We have included 606 “normal” miR-760 targets and 191 top targets in Fig. 5b and 5c. These data clearly indicated preferential regulation of miR-760 top targets during OL differentiation, which is not “a few examples that sounds reasonable” commented by Referee 2. Similar analyses were done in other papers with same magnitude, such as a recent paper from miRNA pioneer David Bartel’s lab (*[*https://pubmed.ncbi.nlm.nih.gov/29887379/*](https://pubmed.ncbi.nlm.nih.gov/29887379/)*). We also validated several key miR-760 target changes by knocking down circSPATA13. Notably, this circRNA knockdown could free its sponged miR-760 under physiological conditions, rather than overexpressing miR-760 that one would expect to see stronger target changes. Importantly, all validated target changes as well as genome-wide targets were all statistically significant.*

It is good that authors of this manuscript measured the abundance of circSPATA13 and found that circSPATA13 are pretty abundant among all circRNAs, but they should compare the level to miR-760 to see if the change of circSPATA13 during OL differentiation are indeed within the dynamic range of meaningful regulation.

*One of the most interesting findings of our manuscript is that the level of miR-760 did not significantly change during OL differentiation, but its top mRNA targets did (Fig 5a-5c). This observation strongly supports a sponging model that circRNA may temporally sequester miR-760 from silencing its target mRNAs without altering the expression levels of miR-760.*

Since miR-760 level is relatively low (Fig. 5a), the small reduction of circSPATA13 (fig 5e) may not be enough to affect miR-760.

*The intention of figure 5a is to include all the 2743 miRNAs detected in our miRNA-seq. In fact, the expression of miR-760 ranked 288 out of total 2743 miRNA, with average CPM 16 in both control and differentiated HOG cells. This is a very abundant miRNA with proved biological functions.*

Many of the figures are presented in relative expression values of miRNA and circRNAs, it is impossible for me to make an evaluation on this.

*We are comparing the trend and fold changes of circRNA, miRNA and mRNA expression between conditions. All the raw data has been deposited in GEO. We calculated the copy number for each detected circRNA in HOG cells and provided as additional file 3.*

Also they need to look at the base-pairing of Myc-miR760, as this target may only supported by prediction.

*There is a recent publication experimentally validated the function and binding sites of miR-760 in regulating myc by both in vivo and in vitro studies (PMID: 31709636). Our data is in agreement with the publication and the novelty is the function of this pathway in human OL system.*

In addition, the level of miR-760 target, Myc, only showed a small change (Fig. 5) upon circRNA KD. The author cited a recent paper in response letter, "(Myc) was recently shown in controlling OL progenitor cells proliferation and differentiation homeostasis", however it is unclear if such a small decrease of Myc level is enough to affect "OL progenitor cells proliferation and differentiation".

*Myc is significantly down regulated in HOG differentiation with log_2_FC=-0.85 and FDR=0.00014, which is one of the most significant down regulated gene in the genome. We conducted circSPATA13 knockdown experiment as requested and have validated Myc is also down regulated in circSPATA13 KD as shown in Fig 5g (log_2_FC= -0.37, P-value=0.035). It is important to note that unlike many studies that overexpress exogenous miRNAs, we knocked down endogenous circSPATA13 at physiological levels, which should only release miRNAs they sequestered. Thus, we do not expect to see a huge change of Myc repression, yet the Myc downregulation is statistically significant. However, we hope the Referee would appreciate that, as a potent transcription factor, Myc does not necessarily have to change “big” to fulfil its biological roles during OL differentiation (PMID: 24502923), and the fact that Myc could only be one of the targets of circSPATA13-miR-760 pathway.*

For the circSPATA13, the authors also need to show that the change of miRNAs targets are consistent and have significant overlap between their two analyses (i.e. KD of circSPATA13 vs the OL differentiation).

*The miR-760 targets we validated by circSPATA13 knockdown indeed showed a consistent change during OL differentiation. However, since numerous factors co-operatively regulate OL differentiation, one would expect multiple direct and indirect mechanisms that underlie each gene’s regulation. Thus, at global levels, one should not oversimplify the rather sophisticated biological event with the assumption that circSPATA13 knockdown could regulate the majority of differentiation regulated miR-760 targets.*

The same set of problems also exist for circPEX6-miR17-5p/106a-5p, where the seed matches for circRNA-miRNA were not even shown. Similarly, the change of circPEX6 and PEX6 in OL differentiation should be examined before they propose a new model of regulatory switch.

*Using full length of circPEX6 that determined by A-tailing RNase R RNA-seq data and CARP, we predicted one 7mer-m8 seed matches of miR17-5p/106a-5p in the body of circPEX6 by TargetScan, suggesting potential sponging function of circPEX6 for miR17-5p/106a-5p. The PEX6 mRNA was up regulated in differentiated HOG cell (log_2_FC = 0.35) while the circPEX6 was much more up regulated (log_2_FC = 0.80). In addition, the expression level of circPEX6 is estimated to be 369 copies per HOG cell (Additional file 3), which is sufficient to serve as miRNA sponge. Importantly, predicted total targets and top targets of miR17-5p/106a-5p both showed global up regulation compared with random genes post-transcriptionally (P-value=1.1x10-6) (Fig S7B, C). This is another example of potential many circRNAs that coordinately contribute to OL differentiation. It will be very interesting to further investigate the detailed downstream pathways, but it has way beyond the scope of this manuscript.*

My concern is that there are many miRNAs being affected in OL differentiation and their targets should have consistent changes, it is not hard to find a few examples that sounds reasonable.

*We did not “find a few examples” but rather provided a complete set of genome-wide miR-760 targets in Fig. 5b and 5c that displayed coordinated changes with the developmental regulation of circSPATA13 (Fig. 5e). During the revision, we were successfully validated several key targets that are regulated by circSPATA13 knockdown using qPCR as requested by the reviewers and editors. This existing data is what Referee 2 asked for in the second round of review “miRNAs targets are consistent and have significant overlap between their two analyses (i.e. KD of circSPATA13 vs the OL differentiation)”.*

Fig. S3C，it is very curious and a little strange that the distribution of circRNA fold have several sharp spikes. One would expect this distribution should be relatively smooth like mRNAs, can the author give an explanation? Is this due to some cutoff setting or some bugs in their program?

*To avoid divided by 0 in calculating the ratio of BSJ reads count in A-tailing RNase R library and untreated library in Fig. 1f and Fig. S3C, we used Ratio = (BSJ reads count in A-Tailing library +1) / (BSJ reads count in untreated library +1). As a result, the low expressed circRNA with 1, 2 or 3 BSJ reads in A-tailing library often have 0 reads in untreated library, leading to lots of the ratio exactly equals to 1, 2, or 3 and the corresponding sharp spikes in Fig. 1f and Fig. S3C. Nonetheless, we were careful to focus on the abundant circRNA with experimentally validated copy number calculation.*

The number is still confusing in some places. For example, line 24 in page 8, "A-tailing allowed the identification of an added 37,950 de novo circRNAs by CARP...". The total number should be 37950+6846 circRNA in HOG cells here, which is different from the "38561 circRNA in HOG cells" in page 9. In addition, these two numbers are not consistent with what they said in the rebuttal letter "(CARP-38,143 circRNA identified, in comparison to CIRCexplorer2 (14,159), CIRIquant (35,338), findcirc (23,294) and MapSplice (11,366) at FDR<0.05). ". Maybe they used different parameters or cutoffs (for unclear reason), but such conflict of numbers decreased the confidence of their data.

*As described in the method section, the candidate circRNAs were first identified independently by several main algorithms, including CIRCexplorer2, CIRIquant, find_circ and MapSplice. Pseudo-reference was built by the combination of all circRNAs identified by any of the algorithms. Total RNA-seq reads were then mapped to pseudo-reference by CARP. For one HOG cell replicate, we identified 44,796 circRNA from A-tailing library, with 6846 circRNAs also found in untreated control (no A-tailing and RNase R treatment). The additional 37,950 were uniquely found in A-tailing library, indicating that A-tailing is a much more sensitive approach for circRNA detection due to the complete removal of linear transcripts. As expected, majority of these A-tailing specific circRNAs display low expression (Fig. 2c). Among all 44,796 circRNAs in this particular HOG replicate, 42,213 of them withheld A-tailing plus RNase R treatment that we consider bona fide circRNAs. By doing so, we were able to identify 42,213, 32,887 and 40,583 bona fide circRNA from triplicated HOG samples with the average number 38,561. Importantly, circRNA DE analysis identified 370 circRNAs that are significantly altered during HOG differentiation (Fig. 3D, FDR<0.05). We have supplied additional file 5 to describe circRNA number and bona fide circRNA number for each replicated M17 and HOG cells.*

In addition, the additional number of circRNAs identified by adding A-tailing step is very significant, and they may need to use another method on the same datasets (with or without A-tailing) to confirms this ~5-fold increase is real.

*In figure 1a-1d, we clearly included four different conditions to compare our results, untreated, RNase R in potassium buffer (no A-tailing), RNase R in lithium buffer (no A-tailing) and RNase R plus A-tailing. We also used four independent algorithms for circRNA identification prior to CARP. The circRNA number identified from A-tailing and untreated library is shown in Additional file 5. The identified number of circRNA all show ~5-fold increase in A-tailing RNase R library compared with untreated library. During the first round of revision, Referee 2 requested us to compare our data with recent published long-reads sequencing data. Among 12,242 common circRNAs detected by both CARP in HOG cells and isoCirc in human brains (Xin et al, 2021), 10,472 (85.54%) displayed exact internal structures, suggesting high sensitivity and accuracy for full-length circRNA construction by CARP. The new comments by the same Referee “they may need to use another method on the same datasets (with or without A-tailing) to confirms this ~5 fold increase is real.” has already been fully addressed in our first round of revision.*

A related concern is that if this increasement also increased the false positive rate, which is a tricky situation due to the compromise between sensitivity vs. specificity, but the author should give some assessment/discussion.

*The false positive reads of circRNA identification were carefully controlled by comparing BSJ reads count in A-tailing library and control library (Fig. 1f and Fig S3C). The sensitivity vs. specificity were extensively investigated by customizing the stringency of seed sequence match after remapped to pseudo-reference (Fig S3B). CARP provided excellent flexibility for users to customize the stringency.*

Minor point, the writing should be polished a little more to be concise and clear. There are many places does not read well and are unnecessarily long. For example, line 32 in page 11, two long subsentences (both starting with which) used in the same long sentence. Line 19 of page 9, "The exons in various circRNAs ranged from 1 to over 20", it should be "numbers of circRNA exons" because the exon itself do not range from 1. Minor places like this make it hard to read.

*Thanks for pointing this out. We carefully went through the manuscript and revised the text accordingly.*

*We hope these responses and revision will address the new set of concerns that were not included in the first round of review by Referee 2.*
